# Origin and Isoform Specific Functions of Exchange Proteins Directly Activated by cAMP: A Phylogenetic Analysis

**DOI:** 10.3390/cells10102750

**Published:** 2021-10-14

**Authors:** Zhuofu Ni, Xiaodong Cheng

**Affiliations:** 1 Department of Integrative Biology & Pharmacology, McGovern Medical School, University of Texas Health Science Center at Houston, Houston, TX 77030, USA; Joseph.Ni@u.northwestern.edu; 2 Texas Therapeutics Institute, Institute of Molecular Medicine, McGovern Medical School, University of Texas Health Science Center at Houston, Houston, TX 77030, USA

**Keywords:** EPAC1, EPAC2, phylogenetics, cyclic nucleotide, guanine nucleotide exchange factor

## Abstract

Exchange proteins directly activated by cAMP (EPAC1 and EPAC2) are one of the several families of cellular effectors of the prototypical second messenger cAMP. To understand the origin and molecular evolution of EPAC proteins, we performed a comprehensive phylogenetic analysis of EPAC1 and EPAC2. Our study demonstrates that unlike its cousin PKA, EPAC proteins are only present in multicellular Metazoa. Within the EPAC family, EPAC1 is only associated with chordates, while EPAC2 spans the entire animal kingdom. Despite a much more contemporary origin, EPAC1 proteins show much more sequence diversity among species, suggesting that EPAC1 has undergone more selection and evolved faster than EPAC2. Phylogenetic analyses of the individual cAMP binding domain (CBD) and guanine nucleotide exchange (GEF) domain of EPACs, two most conserved regions between the two isoforms, further reveal that EPAC1 and EPAC2 are closely clustered together within both the larger cyclic nucleotide receptor and RAPGEF families. These results support the notion that EPAC1 and EPAC2 share a common ancestor resulting from a fusion between the CBD of PKA and the GEF from RAPGEF1. On the other hand, the two terminal extremities and the RAS-association (RA) domains show the most sequence diversity between the two isoforms. Sequence diversities within these regions contribute significantly to the isoform-specific functions of EPACs. Importantly, unique isoform-specific sequence motifs within the RA domain have been identified.

## 1. Introduction

**Figure 1:**
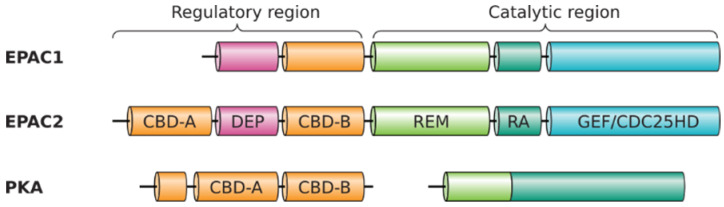
Domain structure of the EPACs and PKA. Individual domains indicated: CBD, cAMP-binding domain; DEP, disheveled, EGL-10 and pleckstrin homology domain; REM, RAS exchange motif; RA, RAS association domain; GEF/CDC25HD, guanine nucleotide exchange factor domain/CDC25 homology domain.

The pleiotropic second messenger cAMP is an ancient stress-response signal that is conserved throughout all domains of life, spanning from the most primitive bacteria to humans, and critical for the optimal fitness of life [1]. In bacteria, the effect of cAMP is mediated by the well-studied cAMP receptor protein (CRP), also known as the catabolite activator protein (CAP). In response to environmental changes in nutrient sources, increases in intracellular cAMP leads to the activation of CRP, a global transcriptional regulator, and results in the expression of a network of catabolite sensitive genes [2]. In humans, the intracellular functions of cAMP are transduced mainly through cAMP-dependent protein kinases (PKA) and the exchange proteins directly activated by cAMP (EPACs) [3], as well as the cyclic nucleotide-gated (CNG) and the hyperpolarization-activated, cyclic nucleotide-gated (HCN) channels [4], the Popeye domain containing (POPDC) proteins [5], and the cyclic nucleotide receptor involved in sperm function (CRIS) [6]. These cAMP receptors share a homologous cAMP binding domain (CBD) that is revolutionary conserved in CRP [7]. Mammalian EPACs exist as two major isoforms, EPAC1 and EPAC2, with major sequence homology [8,9]. EPAC1 and EPAC2 have similar structural architectures with an N-terminal regulatory region and a C-terminal catalytic region. The regulatory regions of EPAC1 and EPAC2 share a Dishevelled/Egl-10/pleckstrin (DEP) domain and a CBD, whereas the catalytic regions of EPAC encompass a RAS exchange motif (REM), a RAS-association (RA) domain and a successive CDC25 homology domain, also known as the guanine nucleotide exchange factor domain (GEF). Full-length EPAC2 also contains an extra CNB domain N-terminal to the DEP domain (Figure 1). This extra CBD-A in EPAC2 binds cAMP with weak affinity and is not required for the autoinhibition of EPAC2 as the CBD-B [10]. While the CBD-A of EPAC2 may not contribute directly to cAMP-mediated regulation under physiological conditions, it appears to play important roles in EPAC2’s subcellular targeting [11,12,13].

In vitro, recombinant EPAC1 and EPAC2 proteins have similar biochemical properties, in terms of their abilities to activate down-stream effectors Rap1 and Rap2 in response to cAMP stimulation. However, the physiological functions of EPAC1 and EPAC2 are diverse due to their distinct tissue/cellular distributions and abilities to form discrete signalosomes through interaction with specific cellular partners [14]. Extensive studies, particularly recent in vivo analyses of EPAC1 and EPAC2 functions using genetic knockout mouse models and pharmacological probes, reveal that EPAC proteins regulate a wide range of processes by interacting with a plethora of intracellular signaling molecules in a precise spatiotemporal fashion [14,15,16,17]. For example, EPAC2 signaling is mainly involved in regulating intracellular calcium mobilization and vesicle trafficking associated with insulin secretion [18,19,20], synapse remodeling [21,22], learning, and social interactions [23,24,25]. EPAC1 signaling is known to crosstalk with signaling pathways such as PI3K/Akt [26,27], phospholipase C (PLC) [28,29,30], TGFβ/SMAD [31,32], leptin/STAT3 [33,34], VEGF and Notch [35,36,37], and contributes to the regulation of cardiovascular functions [38,39,40,41,42,43,44] and energy homeostasis [45]. In addition, dysregulations of EPAC1 signaling have been implicated in the development of numerous pathophysiological conditions in animals and humans, including cancer [46,47,48,49], chronic pain [50,51,52,53], infections [54,55], and vascular proliferative diseases [37,56,57,58,59]. In this study, we performed a comprehensive phylogenetic analysis of EPAC1 and EPAC2 to provide further evolutionary insights into understanding the structural and functional diversity of EPAC proteins.

## 2. Materials and Methods

### 2.1. Protein Sequence Mining and Alignment

Protein sequences were mined through a protein BLAST [60] in NCBI using human EPAC1 and EPAC2 sequence, giving multiple sequence alignments (MSA) with a maximum sequence difference of 0.85. Anomalous and repetitive sequences were filtered out, yielding 368 non-repetitive full-length EPAC1 (154) and EPAC2 (214) sequences across eukaryotic species. The CBD-B of human EPAC1 and EPAC2 were used as query sequences to yield MSA of 1034 RAPGEFs and PKA/G CBD-based sequences. The guanine exchange factor (GEF) domain of human EPAC1 and EPAC2 were used as query sequences to yield MSA of 897 RAPGEF GEF-based sequences.

### 2.2. Phylogenetic Tree Construction

Phylogenetic trees were constructed using the constraint-based multiple alignment tool (COBALT) [61] and the fast minimum evolution algorithm [62], and plotted using Dendroscope [63]. Three major phylogenetic trees were constructed for full-length EPAC sequences, N-terminal CBD alignment, and C-terminal GEF alignment. Without direct evidence of the evolutionary roots of these proteins, these trees were drawn as unrooted cladograms. Additionally, EPAC family trees were isolated from CBD- and GEF-based trees, and drawn as rooted phylograms, where PKA/G and RAPGEFs served as out-groups to indicate a possible root of EPAC origin.

### 2.3. Ancestral Sequence Reconstruction

Ancestral sequences were reconstructed using the maximum-likelihood reconstruction method on the FASTML server. The server created maximum-likelihood phylogenetic trees, which were cross-checked with the COBALT trees. Ancestral sequences for nodes on the phylogenetic trees were compiled for EPAC1 and EPAC2 sequences in the whole sequence tree and domain trees.

### 2.4. Amino Acid Composition of EPAC Isoform Specific Sequence Motifs

Position-specific EPAC isoform specific sequence motifs with sequence weighting, and two-sided representations of amino acid enrichment and depletion were constructed and visualized using Seq2Logo [64].

## 3. Results

### 3.1. EPAC2 Is More Ancient and Conserved Than EPAC1

**Figure 2:**
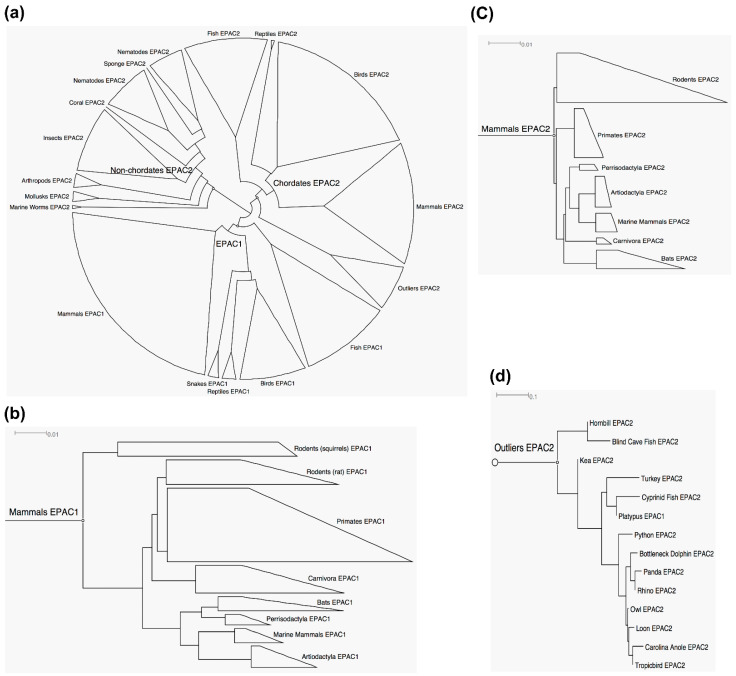
Phylogenetic analyses of EPAC1 and EPAC2. (**a**) Unrooted cladogram of EPAC1 and EPAC2. (**b**) Rooted phylogram of mammalian EPAC1. (**c**) Rooted phylogram of mammalian EPAC2. (**d**) Rooted phylogram of EPAC2 outliers. Scale bars: 0.01 or 0.1 represents 1 aa substitution per 100 or 10 aa, respectively.

To study the evolution of EPAC proteins, we generated phylogenetic trees of EPACs through MSA of 154 EPAC1 and 214 EPAC2 non-repetitive sequences derived from a comprehensive sequence search on BLAST (Supplementary data 1). As a result, we generated an unrooted cladogram of EPAC1 and EPAC2 (Figure 2a). We found EPAC2 sequences spanning across different phyla in the Animalia kingdom, ranging from the most basic phylum Porifera (corals), to phylum Nematoda (*C. elegans*), to all major classes in the phylum Chordata. On the contrary, while species with EPAC1 unanimously contained EPAC2, EPAC1 was not present in any invertebrates. We found EPAC1 sequences limited to the phylum Chordata, spanning from the most primitive fish to all members of the mammal class. The closest ancestral branching point for EPAC1 from EPAC2 is marine worms. Rooted phylograms of mammalian EPAC1 and EPAC2 were constructed for a better understanding their evolutional relationship (Figure 2b,c). While both trees, which were drawn to the same scale of relative rate of amino acid substitution, follow the similar trend of evolutionary path in terms of animal taxonomy, the degree of sequence diversity for EPAC1 evolution is much higher than that of EPAC2. For example, by comparing the EPAC isoform sequences for *Homo sapiens* and *Danio rerio*, we found that the sequence percentage identity for humans and zebrafish EPAC2 is 77.4%, while the identity for EPAC1 between the two species is 57.9%. These results reveal that EPAC1 is more evolutionary advanced and less ancient than EPAC2, while EPAC2 sequences are generally more conserved than EPAC1.

In addition to well-organized EPAC1 and EPAC2 branches, we also noticed a group of outliers, mostly EPAC2 sequences from 14 distinct species containing fishes, reptiles, birds and mammals, as well as platypus, a primitive and egg-laying mammal with evolutionary links with reptiles and birds [65] (Figure 2d). These anomalous sequences were much less conserved than typical mammal EPAC sequences (Figure 2b,c) and lacked clear organization that fits with vertebrate phylogeny trends. However, a manual inspection of these outliers reveal that these sequences are partial and/or predicted sequences which were automatically annotated without verification.

### 3.2. Common Ancestor and Co-Evolution of EPAC1 and EPAC2 CBD

Unlike PKA proteins, which consist of separate regulatory and catalytic subunits, EPAC proteins are single polypeptide molecules with two functional halves: a CBD containing N-terminal regulatory region, and a C-terminal catalytic region with GEF activity (Figure 1). This dual functionality feature of EPAC suggests that the first EPAC gene likely originated from a recombination event, resulting in the fusion of two DNA fragments with coding sequences for a CNB-containing regulatory module and a GEF-containing catalytic module, respectively. Therefore, we hypothesized that performing separate phylogenetic analyses of the CBD and GEF regions would provide a better understanding of the origin of EPAC isoforms and allow a direct comparison of the coevolution process between the two functional entities.

**Figure 3:**
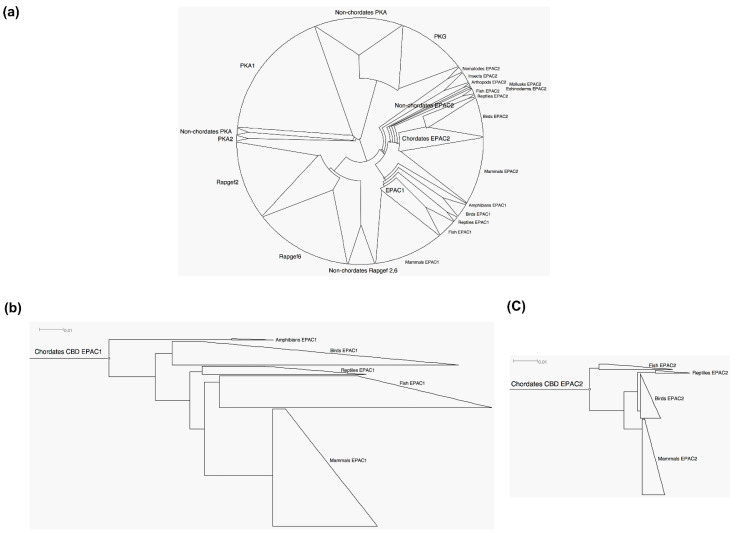
Phylogenetic analyses of the CBD of PKA, PKG and EPAC1, EPAC2, RAPGEF 2 and 6. (**a**) Unrooted cladogram of CBD of PKA, PKG and EPAC1, EPAC2, RAPGEF 2 and 6. (**b**) Rooted phylogram of chordate CBD of EPAC1. (**c**) Rooted phylogram of chordate EPAC2. Scale bars: 0.01 represents 1 aa substitution per 100.

A BLAST search using the CBD-B sequences of EPAC resulted in 1034 CBD sequences from non-repetitive species. These sequences cover several closely related families: PKA/PKG, EPAC1/EPAC2 (RAPGEF3/RAPGEF4), and RAPGEF2/RAPGEF6 (PDZ-GEF1/PDZ-GEF2) (Supplementary data 2). Using these sequences, we generated an unrooted cladogram of CBD with MSA (Figure 3a). Overall, the EPAC CBD phylogenetic branches still followed the similar feature and trend of evolutionary relationship in terms of taxonomy groups under the constraints of the larger CBD families, as compared to the phylogenetic tree based on full-length EPAC sequences (Figure 2a). EPAC1 and EPAC2 CBDs were more closely related to each other among all members of the cyclic nucleotide binding domain family. Using PKA and PKG as out-groups, the root/origin of the CBD in EPAC1/EPAC2 or RAPGEF2/RAPGEF6 could be clearly located on the cladogram. While EPAC CBDs shared a common ancestor closely related to nematode EPAC CBD, EPAC1 CBD originated at a much later root parallel to chordate EPAC2 CBD. Rooted phylograms of chordate EPAC1 and EPAC2 drawn to the same scale (Figure 3b,c) revealed that the sequence diversity among EPAC1 was much higher than that of EPAC2. Similar to the comparison above of EPAC full-length sequence identity between humans and zebrafish, we found that the sequence identity of CBD domains between two species is 96.7% for EPAC2, and 89.1% for EPAC1. It is important to note that RAPGEF2/RAPGEF6 contain putative CBDs and are most closely related to EPAC1/EPAC2 among RAP-specific GEFs [66]. However, the CBD of RAPGEF2/RAPGEF6 does not contain conserved residues important for cyclic nucleotide binding [67] and is not responsive to cAMP or other nucleotides [68].

**Figure 4:**
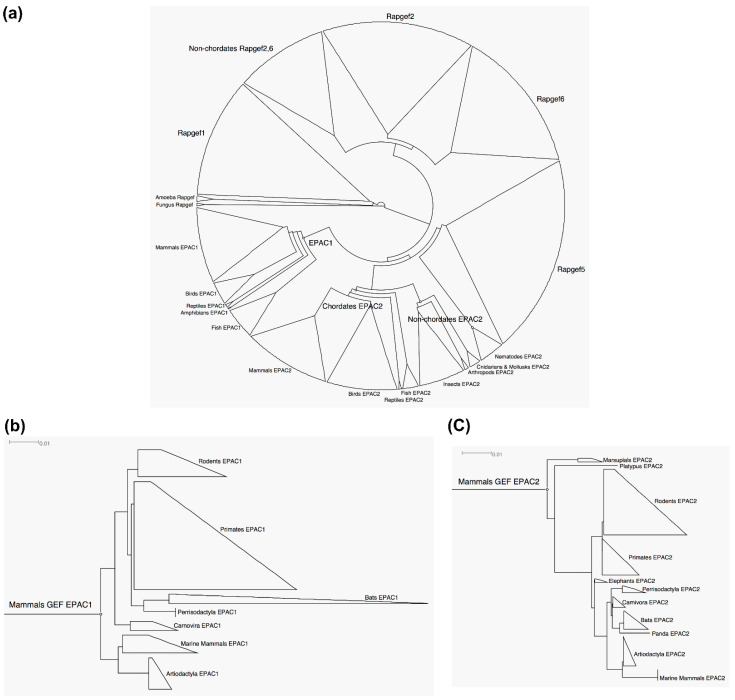
Phylogenetic analyses of the GEF of RAPGEF1-6. (**a**) Unrooted cladogram of the GEF of RAPGEF1-6. (**b**) Rooted phylogram of the mammalian GEF of EPAC1. (**c**) Rooted phylogram of the mammalian GEF of EPAC2. Scale bars: 0.01 represents 1 aa substitution per 100.

A BLAST search using the GEF domain of EPAC1 and EPAC2 led to the identification of 897 sequences across the RAPGEF family from non-repetitive species (Supplementary data 3). An unrooted cladogram of GEF domain of RAPGEF was generated with MSA (Figure 4a). EPAC GEF phylogeny still followed the general trend of animal taxonomy as shown in the full-length EPAC tree (Figure 2a) with the constraints of the larger RAPGEF families. EPAC1 and EPAC2 GEFs were more closely clustered with each other among all RAPGEF members of the family. It appeared that the GEF domain of RAPGEFs is originated from RAPGEF1, which contained species that are more primitive. GEF domain of RAPGEF2 and RAPGEF6 form a separate group, leaving EPAC1, EPAC2 and RAPGEF5 clustered in a relatively closely related group.

We could clearly observe that EPAC1 GEF originates at a later root than the origins of EPAC2 GEF in primitive species, parallel to chordate EPAC2 GEF sequences. Rooted phylograms of mammalian EPAC1 and EPAC2 GEF, drawn to the same scale, showed that EPAC1 GEF are more divergent than EPAC2 counterparts (Figure 4b,c). We compared the sequence identity of GEFs again between humans and zebrafish, and we found that EPAC2 GEFs have a sequence identity of 83.6%, while EPAC1 GEFs have an identity of 66.3%. As expected, the mammalian EPAC1 GEF tree featured the same taxonomy groups (Figure 4b), as compared to the tree derived from the full-length EPAC1 sequence (Figure 2b). On the other hand, the mammalian EPAC2 GEF tree (Figure 4c) contained the marsupial taxa, a group evolutionarily distinct from other placental mammals, as well as pandas and platypus.

### 3.3. Identification of Isoform-Specific Sequence Motifs

**Figure 5:**
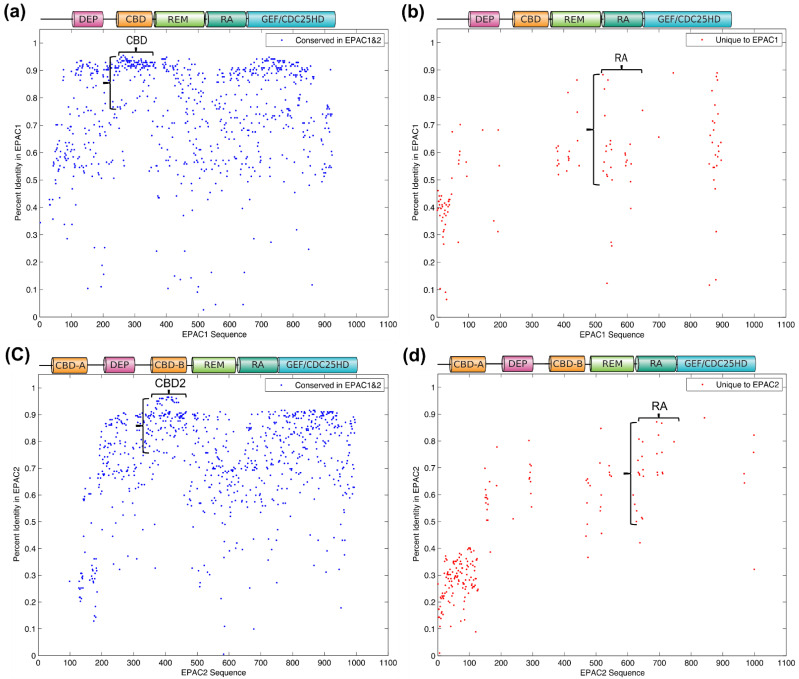
Sequence identity and diversity at individual residue between aligned human EPAC1 and EPAC2. (**a**) The percent identity of residues in EPAC1 over its entire range for the two EPACs. (**b**) Unique residues in aligned human EPACs in EPAC1. (**c**) The percent identity of residues in EPAC2 over its entire range for the two EPACs. (**d**) Unique residues in aligned human EPACs in EPAC2. The x-axes show amino acid residue numbers while the y-axes show percent identity of species in its own isoform.

One of our goals is to search for unique sequence signatures that can differentiate the two EPAC isoforms. Ideally, such a sequence motif would be highly conserved within its own isoform among all species, but absent from the other isoform. To achieve this goal, we aligned sequences for both EPAC isoforms in all species, and at each amino acid position determined (1) whether the aligned human residue for EPAC1 and EPAC2 was the same, and (2) the percent identity of EPAC1/EPAC2 residue against the respective isoform in other species. For EPAC1, blue dots show that the residue on the human EPAC1 isoform is the same on the aligned counterpart of the EPAC2 isoform (Figure 5a) while red dots show that the residue is different. (Figure 5b). A similar calculation was performed for EPAC2 to generate the corresponding plots (Figure 5c,d). It was apparent that the CBDs in EPACs are highly conserved among all species between and within each EPAC isoform. EPAC1 CBD had a percent identity range from 75% to 95%, while EPAC2 CBD-B had a similar percent identity range from 75% to 97%. On the other hand, EPAC2 lacked any conserved sequences from 0–100 residue, because CBD-A was lost in EPAC1. The C-terminal catalytic region was mostly conserved for human EPAC1 and EPAC2, but ranges of the percent identity of individual residues in each isoform were much broader than those of the CBD-B, indicating a lower degree of conservation that CBD among all species within this region (Figure 5a,c).

A congregate of unique residues exist in the N-terminus of EPAC1 and EPAC2, yet none of these residues exhibit high percent identity, ranging from 10% to 45%, within each EPAC isoform (Figure 5b,d), indicating active evolutional drift in this region for both EPACs. Consequently, these sequences are not suitable candidates for isoform-specific sequence motifs as they are not representational for all species.

**Figure 6:**
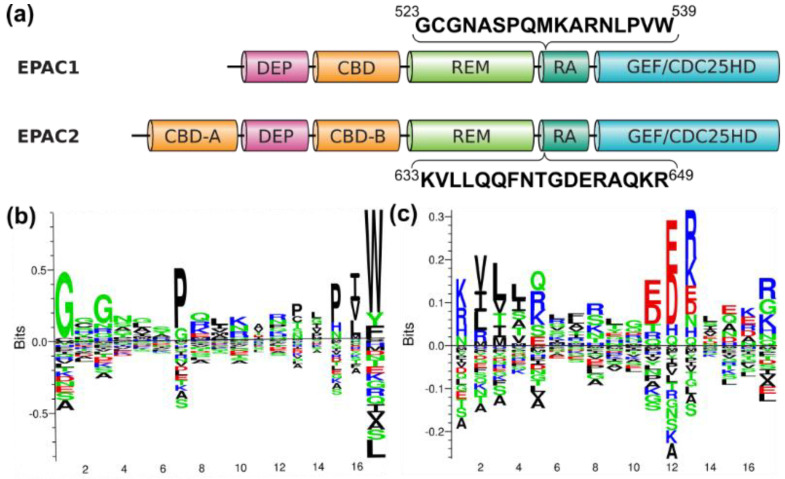
Isoform-specific sequence motifs of EPACs. (**a**) Alignment of human EPAC1 and EPAC2 with sequences spanning the isoform-specific motifs highlighted. Position-specific and isoform specific sequence motifs, with sequence weighting, and two-sided representation of amino acid enrichment and depletion, in EPAC1 (**b**) and EPAC2 (**c**) RA domain.

Other sequentially diverse areas between EPAC1 and EPAC2 included the RA domain and the C-Terminal extremity. In particular, residues within the RA domain contained unique sequences between EPAC1 and EPAC2, and also maintained high levels of sequence identity (50–90%) within each isoform, making this region a suitable target for finding isoform-specific sequence signatures (Supplemental Figure S1). Indeed, further sequence analyses led to the identification of two isoform-specific sequence motifs in human EPAC1 spanning residues from 523 to 539, and in human EPAC2 spanning residues from 633 to 649, respectively (Figure 6).

## 4. Discussion

Our current study, the first comprehensive phylogenetic analysis of EPAC1 and EPAC2, reveals that evolutionally, EPACs have a more modern origin than their cousin PKA. EPAC proteins are only present in multicellular Metazoa, while PKA can be found in unicellular eukaryotes. Within the EPAC family, while EPAC2 spans the entire animal kingdom, EPAC1 is only associated with chordates and above. Based on our analysis, the possible ancestral branching point of EPAC1 away from EPAC2 occurred in organisms related to marine worms. With the development of bilateral symmetry, a critical step in the evolution of animal life, marine worms represent the first ancestor on the family tree that contains most animals today, including humans [69]. Most likely, an EPAC2 gene duplication event during evolution led to the creation of EPAC1, which lacks the N-terminal CNB-A domain. While EPAC2 retains the CNB-A site, its cAMP binding affinity is much weaker than that of CNB-B, and significantly above the physiological concentrations of cAMP [10], suggesting that the functional degeneration of CBD-A occurred before the divergence of EPAC2 and EPAC1. The loss of the cAMP-binding functionality and accompanying conservation pressure likely contributed to the increased sequence diversity observed within this region; the N-terminal extremity sequences of EPAC are the least conserved between EPAC1 and EPAC2, while CBD-B has the highest sequence conservation. Not surprisingly, the N-terminal sequence variation between EPAC1 and EPAC2 plays an important role in their functional diversities. For example, N-terminal sequences to the DEP domain in EPAC1 contain a mitochondrial targeting motif and are important for mitochondrial targeting [70]. The same region has also been reported to interact with the ezrin-radixin-moesin (ERM) family of scaffolding proteins [71]. In contrast, the CDB-A of EPAC2, while very poor at binding cAMP, is required for EPAC2′s proper cellular targeting to the proximity of plasma membrane [11] and critical for directing EPAC2 to the granule sites in β-cells [12]. In addition, CDB-A in EPAC2 shields a conserved nuclear pore localization signal located within the GEF domain, and contributes to the distinct subcellular distributions of EPAC1 and EPAC2 [13]. The interface formed between CDB-A and CDB-B in EPAC2 also provides an allosteric binding site for the development of isoform-specific EPAC2 modulators [72].

As an ancient stress-response signal, cAMP evolves its functionalities to match the increased biological complexity during evolution by expanding its repertoire of intracellular receptors from one single transcriptional factor in bacteria, to multi-families of effectors with diverse functional activities of GEF [8,9], ion channel [4], kinase [73], etc. Nature accomplishes such a remarkable feat elegantly through the assembly of the CBD domain with other functional modalities to create new molecular entities. Indeed, phylogenetic analyses of the individual CBD and GEF domains of EPACs show that within both the larger cyclic nucleotide receptor and RAPGEF family trees, EPAC1 and EPAC2 remain clustered together. These results provide strong evidence that EPAC1 and EPAC2 share a common ancestor, likely resulted from a fusion between the CBD of PKA and the GEF from RAPGEF1. Moreover, the CBD and GEF domains in EPACs exhibit similar evolutionary trajectories and co-evolve together. These findings are consistent with the fact that CBD and GEF are the most conserved regions within the EPAC family.

Besides the N-terminal extremity, the RA domain and the C-terminal end of EPAC1 and EPAC2 also display significant sequence diversity between the two isoforms. However, within individual EPAC isoforms, the RA domain has significant sequence conservation, which allows the identification of unique isoform-specific sequence motifs within this region (Figure 6). RA domain (SM00314) is about 100 residues in size and folds into a ubiquitin alpha/beta roll superfold [74]. It has been found in a wide variety of proteins with diverse functions, and believed to function mainly as protein interaction scaffolds [75]. When mapped to the EPAC2 crystal structures, the isoform-specific sequence motif in EPAC2 is located in a disordered region with no visible electron density in both the inactive and active conformations [76,77]. Similarly, the isoform-specific sequence motif in EPAC1 is located in an extended, disordered surface loop in a recent structural model predicted by AlphaFold2 [78]. These observations suggest that these isoform-specific sequence motifs are likely involved in complex formation, as such, they are unstructured in isolation and only assume folded structure when in complex with other binding partners. Previous studies have demonstrated that RA domain contributes to isoform-specific functions of EPACs. For example, RA domain is responsible for RAS-mediated EPAC2, but not EPAC1, translocation to plasma membrane [12,79] and activation [80]. The expression of an EPAC2 rare coding mutation in the RA domain found in several autistic patients impairs EPAC2’s interaction with RAS and selectively reduces basal dendrite complexity in cortical pyramidal neurons [24]. On the other hand, the RA domain of EPAC1 interacts with β-arrestin2 and differentially regulates cardiac hypertrophic signaling mediated by β-adrenergic receptor subtypes [81]. EPAC1 RA has also been shown to mediate the interaction with Ran-GTP and RanBP2 proteins, and for targeting EPAC1 to the nuclear membrane [82]. It will be interesting to test if EPAC isoform-specific sequence motifs identified in this study are involved in these reported isoform-specific EPAC functions.

## 5. Conclusions

Our study provides valuable information about the origin and evolutionary history of EPAC family proteins. These findings offer major insights into our understanding of isoform-specific EPAC structure and function. In addition, we have identified specific sequence signatures that are unique between the two EPAC isoforms but conserved among all species within individual EPAC isoforms. These isoform-selective sequence motifs likely function as docking sites for interaction with discrete cellular partners and can serve as target sites for developing isoform-specific small molecule probes and/or antibodies as valuable research tools or leads for potential therapeutic uses.

## Data Availability

The data presented in this study are available on request from the corresponding author.

## Supplementary Materials

The following are available online at https://www.mdpi.com/article/10.3390/cells10102750/s1, Supplemental Figure S1. Sequence alignment of EPAC1 and EPAC2 RA domain. Supplementary data 1: Sequence alignment of EPACs. Supplementary data 2: Sequence alignment of CBD of PKA/PKG, RAPGEF2/RAPGEF6 and EPACs. Supplementary data 3: Sequence alignment of GEF of RAPGEF1-6.
