# Supplementary material for: Origin and Isoform Specific Functions of Exchange Proteins Directly Activated by cAMP: A Phylogenetic Analysis
Source: Cells. 2021 Oct 14;10(10):2750. doi: 10.3390/cells10102750 (PMC8534922; doi:10.3390/cells10102750)
Supplement: Supplementary file 1 [file cells-10-02750-s001.zip › cells-1414892-supplementary.pdf]

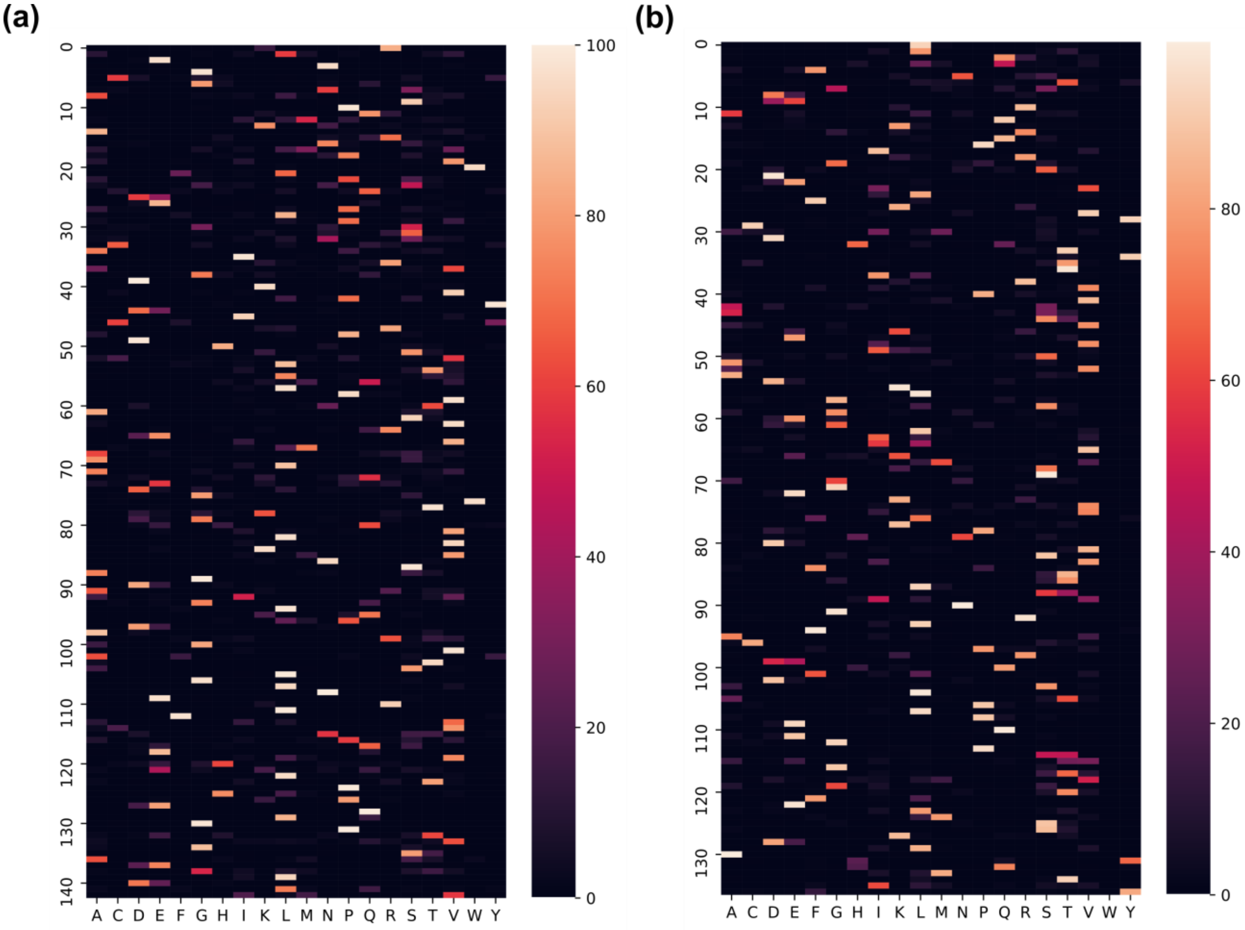

Supplemental Figure S1. Sequence alignment of EPAC1 and EPAC2 RA domain. Distribution of amino acid residues of aligned RA domain of EPAC1 (a) and EPAC2 (b) across species.

>Supplementary data 1: Sequence alignment of EPAC1 and 2

```
>Rapgef0_[Acanthamoeba_castellanii_str._Neff]_470530403
------------------------------------------------------------
------------------------------------------------------------
------------------------------------------------------------
------------------------------------------------------------
------------------------------------------------------------
------------------------------------------------------------
------------------------------------------------------------
------------------------------------------------------------
------------------------------------------------------------
------------------------------------------------------------
------------------------------------------------------------
------------------------------------------------------------
------------------------------------------------------------
------------------------------------------------------------
------------------------------------------------------------
------------------------------------------------------------
------------MEEGLSPRLGSAHS-AAVGSPRSHGAKDYREEIRNKRQLTVIPE----
LHAQESLDALPIRMSEACTLRRTRPNSNNPAPYTITIVGP-----NDGSEAELVSAFVNS
DGAPQLDASPRSHR--GAKESGGDGASQEAMDLYLSSWKKKFNFAGKEYLLELHAGLHTQ
AETALDKGANVHIHDQFESSSGFMFLYDVTSRETFAEVMRVHSLIVRSKN----VEKGKA
KGLIPMIV-LANTRNSFQHQQRQPAQPPPGGDSPSSHHPMVSVT-APDGSDAIASMSGVS
GVAVSASGDSATATTTQSPEREVTTEEGLEFAYACNCPFYEISSTTNYADVLNAFQQLLG
EIIKGQRTRKAAADPASKLTQLGVKGNWRRSQLASLVSSASSPSSYGVSPPLSPPLSPLS
SPPPQPRIEPVLPGSARGVDDDSAAARALAAKAQGGGGLLAPTTT---TSS-----SSEA
KE-ELHESLKDRQKAFRYTRVDAADIKYLPPLDAEPNS-------KPR---IRAATLPKL
IEKLTYPKY------------PDT---DLV------------------------A---T-
FLLTYRS--FT---------------TPEELLHFVM-----RRY----------------
--------ETTA--------------P--ADLDKE--GLRLWEASEMLPIRLRVINVLKS
WISDHFYDFYDS-KTLLDTLK----DFVDK-QLSVPTN------LKGPLQQ---------
--L----------------------KKSIE------------------------------
--------------------TNE-------MER---------------------------
------------------------------------------------------------
------------------------------------------------------------
------------------------------------------------------------
------------------------------------------------------------
------------------------------------------------------------
------------------------------------------------------------
-------RARKE--YM-----------------------------FS-------------
---------------SKPPKSVISK-----------------------------------
-KVAKA------------------------------------------------------
------------K---PQEI----------------------------------------
---DLRDIDPIEMARQLTLIDYDLLSRITAKEWLAHNEWATNKAKKRDDE-SKPAAPNLV
AMINH---------FNCVSKWV-----ATEIVKCAKIKHR------VLLTKKFIEVAQHC
---------------LKLNNFNMLMAIVTGGLGNVSVYRLKYSWK---------------
-------SLPKKTLIAYEE-LSRIL-------------------------CDSLSNF---
-NSLRT----AIRT-------SVPPTI-PYLG----------------------------
LYLSDLMYIEDGNAD---EVE-----------------------------GGLINFE-KR
-------RL---KAETIKDLQHFQT--SP------YCL----------------------
-----------------------TEIP--V--------------------LQY-----YF
RSLDHL-SEAEL-----------------------------------------YRMSCLL
-----------EPHSRQRGASIVNAPSRGMADQL--SASGALDLLESSDGEIQGVSSDED
```

LLGRDGGAHAL----SSSPPPSSPFAQMTRAGGFTPSGLMLSHFEVPLRAKAATGERDTE
RTPRVGAVLRAQAKLLEAVAATFS-------------FADVARATAAVDVLR-----QWE
AQVASRKLFIVKDHSD--------------FAGLDGDDDSGGGGAELKGD---NYSVDGQ
QRKQNGSKRKERARGEKGEKGEKGEKPSSSKLGQHQGTEEAQQHHRTFGFEEVLMESRAV
ELAVERFLQTATVEGILEATRADPIGGGWLAAFEAFLHKRLLGGQRPAFVTELLFALVKL
LDKGTACFDAAAVAGMVGLYKKQPVRLAAHFVTDEAEAELADRHDRLVELEDREGVRTIQ
SYTKEVRLAFQILNLEKRILQAVKTSRPDGSMLYEALCVDREVWHRTQEVLLHTHSPKSP
TAPNPSEPSGGRTSETTHKIIGELIQLVDKQLCEVSREDMQRFNESFEDALEEYFGWMGV
YLRKLWDKLVLIHKRTEKIVQMMKESSGNSHSAKDSNAISKAANAWREKDLVHLHALIAA
VDQAETTVNKFLESCPASSADGFDVTEDELKELLSQNNVFEQIIKMRRLQKTLEEQHARI
ADV
>Rapgef0_[Ancylostoma_ceylanicum]_510856406

--------------------------MLTVSNEAI-------------------------

-------AAYFQ-----SFEARILEV---------------------GSHLK-HAMYTQA
PH-L----------------IRERIIH--------KV---------PYA------DC-MI
GS-----------------EMVDWLLDLS-------------------------------
---VST--GAHSPALSRFQISGMW---QTLLEHNVIC-----------------------
--------------HGMAY---------SFAVGVVARITITG-ELQFVDKL-VCYRWVDG
--GRTDRRMAN----GV-P---
---GRFPLGD
PRLKEEVPSREDI------ASAVFFLSTVGPDAL-------------FRMILKKLPQDRT
PEELE-----LVYEELLHVKALSHLS-TMVKRELA-TVIGYEHHTHAGTVL---------
--FHQGDPG--KNWYIIL
RGSVDVSIHGK-----GV-------------------------------VCTLQEG-DDF
GKLAL-----------VNDSPRA---ATITLREDHAQFLTVDKHDFNR------ILRD--
---V--------------

------------------------EANTVRLKEHGQDVLVLEK-----INL---------
---------P----------------RGAAIEG--GLTNTGKGHCC---YSVMAGLAEKM
LEYVLET-------------RVDA---------------QEDG----AELDVFLE---D-
LVLTHII--YL---------------PTNTLCNYLK-----H------------------
--------YYSR-A--------AEPHS-----DP---LVVLDDLEHRLSARRRVVTFLWL
WVNALGIHYFLD-PAANAFVE----ELYCH--VLEDH--------RTL-----PGMGPI-

--------------LARISALRD-LREEARRTL---------------------------
----AR--------HPAV--------------------------------VLECGVLSTM
A--PSPNP--

------VLPSDI--CN--QI
IHLSDTTSFALPIRMDKTATEICE--------LVRSR-----------LR-SSHGEELAL
VEVKSS----------------------GEKVV------FYDGDVSIATMLSLNSKLYVV
SKDE---I-DSLV-PQLDQ---------------------------NGP------VESVH
AS-VMEYVGSHELAQQLLVLHTQLFEATDEIELVT-------QVIGRDQF-PGRVPSNLD

LLMR--S-
--------------AKENRDLMTLFAITL-GLSNIAVSRLTHLWE---------------
-------RLPAKLRRQFAE-FESLL--------------------------DPSRNH---
-RPYRA----LVAK-------MSPPLI-PFVP----------------------------
LLLKDLTFIHEGNK--TY-YN-----------------------------G-LVNFE-KM
-------HM---IANILRSFRQCKSRYS------VTQM-------------------E--
----------------------QKKI------F----------------ETQN-----FI
RNFRVVDNQRRL---MELSYQI
-----------EPRR----------RRN--------------------------------

>Rapgef0_[Ancylostoma_duodenale]_748380306

---------------------------MWIKDYGK-KKLIFHC-----------------
--SRSVIV
NGLVEVVKPNGER----V---------------------------------EYKLG-DSF
GAEPN-----------PAVQYHV---GEMRTMVDDCEFVLVEHKDFCS------IMST--
---I--------------

------------------------GEHIEKDRDGLTGEIVSETE------R---RT----
-------VGSQ----V-----------------GL---------------VLIKGKPDKL
IQHLVDDRD-------SN---VDP---H------------------------YVD---D-
FLLTYRV--FI--------------HNPTIIFEKLM-----MWF------ADSAL-----
--RDKIARIVLL
WVNNHYNDFETN-DEMTKLLE----RFEGA--LERDGMHSQQSLLNIACSV-KARSRTVV
YAR-------------------ANKDDALHFTVQGGKECGCGIFISEVQVDSPAELCGMK
RGDEVLDVNGQPMKYLTLAKAQEIMKGSISLTITLKSNVLGFKELLGKTERDGSKVSSKN
RALAGVLAQARGSMPNVIPVKSAKAVKIGGKTSMMDKLMTILKSSKEGEDVPDEARTTTN
PLRPSRSNPDITSISQYYGPVKSECPEHVLKIYRSDQTFKYLAVYKETTAQNVVQLALQD

```
DALHFSVQGGKECGCGIFISE----------------------VQ---------------
---V-----DSPAELCGMKRGDEVLDVNGQPMKYLTLAKAQEIMKGSISLTITLKSNVLG
FKELLGKTERDGSKVSSKNRALAGVLAQARGSMPNVIPVKSA-----KAVKIGDVVK---
------------------------TSMMDKLMTILKSSK-DGEDIPD----EART----T
TNPLRSSRSNPD--IT------------SISQYYGPVKSECPEHVLK-------------
IYRSDQTFKYLAVYKETTAQNVVQ--------LALQ---------EFNMTAE-GSLEWSL
CECTVT----------------------EDGVIKQR--RLPPQMENLAERIALNSRYYLK
NNNR------SDP-LVPDDL---------------------------AP------EIMRD
AQAQLLSLNAQVVAAQLTLQDFAVFASIEPTEYI-------DNLFQLESR-YGS--PRLE
EFERI---------FNREMWWV-----ATEVCSERNVQKR------AKLIKKFIKVARH-
--------------CRELRNFNSMFAIMS-GLDKPAVRRLHSTWE---------------
-------RISGKYIRMLED-VQQLV--------------------------DPSRNM---
-SKYRQHLAE-VSQ-------E-PPVV-PIYP----------------------------
VIKKDLTFSHEGNP--TY-CD-----------------------------K-LVNFE-KL
-------RL---IAKSVRAVSKLSSAPYEISSMAERSGGT-ISDALLHMN---TFEGG--
NV------------------ATMRK---G--GRVIQPRKKVYEQALMVRKVKA-----YL
DGLKVVDTESEL-----------------------------------------DRLSYDI
-----------EPQ----------QAGPRV-----------RRAPSPSPSSVSSQSAESN
S------------TDQRR------------FRGGVMFGVDSPQAVQKMLGLVQTSKSK--
-DSSAKRPFPTYTKP-----PSVS-----VTVVQPSRFAQYAHNRSPY-------AMERS
TRSASLTSTMLSDSMTPYFSLKGSRTLQNYISPSSAMIRKENVGGTIKDG----------
------------------------------------------------------------
------------------------------------------------------------
------------------------------------------------------------
------------------------------------------------------------
------------------------------------------------------------
------------------------------------------------------------
------------------------------------------------------------
---
>Rapgef0_[Brugia_malayi]_170587953
------MTSKCIISTHMKYVTITDWFQYGKFGYGSSLLGNKNDSKFQSDGDLLNDSCGSM
EHYKEVFIASIAKE-----------PST-----RTDEDLKIIYSNLRRLDVFHNLNDAPL
RAICCTAR--FERHP--ANFILFRQGQVATCWYILLSGCVFMDEQI---------YLPCG
W----------------FGKRNGLDY-RRKNDCI-VLQPAEMIVIDYPDVKHIKMNKGH-
----------------------MCSTENSGRLRN--------------------------
-----------------------------------SLC----------------------
-------ALSAAAIPSP----------------------SGTV-----------------
--------------TVSI----RSGADS--------------------------ATSN--
------------------PSLPPP-----------------------SRLYKTGKASPLS
ATESPISRSERKKHRSFRQERDDICSSN-----ASGSD----------------------
------------------------------------------------------------
------------------------------------------------------------
------------------------------------------------------------
------------------------------------------------------------
--YASYKSQIYLNGLNVDTDTRLKVKHKARKSNSISSPNSSEFYTHLS----STTSSSTN
EDDLHGLPEAAV----DSEEDDESCPSY------DS----FHELKDSVRECLEKEPFERT
ADDIC-----TLLDFMQHMSALATLP-LSIKRQLC-LKMVFAVVSEANTVV---------
--------------------------------------------MQHNERI--DAWSVIV
NGQVEVVLPDGER----I---------------------------------EYKLG-DSF
GVQPD-----------LVPQYHV---GIMRTLVDDCEFVLVEHRDYCS------IMST--
---------------------------------------------I--------------
------------------------------------------------------------
------------------------------------------------------------
------------------------SEHIEQHSDGITGEIVSEIE------R---RS----
```

-------VGNQ----M-----------------GQ---------------VLIKANREKL
IEHLVEERD-------TA---VDA---H------------------------YIE---D-
FLLMYRV--FI--------------NDPTMIFEKLM-----HWF------AESNL-----
--RDKIARIVLL
WVNNHFNDFECN-GEMMQLLD----RFEQA--LERDQMHSQQSLLNIACSV-KSRTRNIV
YSR---

-------------------SSRD---------------------QPLSLSILGGKENGSS
GIFVSDVQRTSRAEKIGLKRGDQIIEVNGQSFKKISLIRALEVLRSNTHLSITVKSNLLG
FKEMIARSERAVDVEDQCNAYRKGP--EIRYTKHRAVSKQSENVQRGSMSCMVG-MNPRT
GASSFSYLPPSGMTSSIG----NKTSMMDKLLAMLKGLP---STLPDAPVAESKTLNPTR
SNSLRASRSNPD--IAGHSVGASRISTMNIAQYYQPAS-PCPEHVLK-------------
VYRADQSFKYLTIYKETTAQNVVQ--------LALQ---------EFGMSSDSGSLEWAL
CETTVT----------------------LDGVIKQK--RLPDQMNNLAERIQLNSRYYLK
NNSR------SEP-MVPDEL---------------------------AP------EVLKE
AETHLLQLNAQILAAQLTLQDFDVFSSIEPTEYV-------DNLFNLESK-YGW--PKLA
DFESL---------FNKEMWWV-----ATEICREKGLQKR------MKLIKKFIKVARY-
--------------CRDFRNFNSMFAIMS-GLEKPAVRRLHHTWE---------------
-------RVPSKYTKMFED-VQHLV--------------------------DPSRNM---
-SKYRQHLAV-VSQ-------E-PPVV-PIYP----------------------------
VLKKDLTFSHEGNP--TY-CE-----------------------------K-LINFE-KL
-------RM---IAKTIRSVTKLCSSPYEIGVMAQQSGGNDVSDALVNMN---SFDGGAG
TV------------------ATMRKVGPNAMKTATQPRRKLYEQALMVRKVKS-----YL
SNLNVVECETDL---DQLSYEC
-----------EPSY--------GSASVIR-----------RRAPSPSPSSLSSQS----
S------------TSDQHRI------------SAPKFGVESPHAVQKMLSLAQNSRLKQG
---CSGNLIPIS--PLQSPLLSSKGVRLRNTPLPVRRVPSTSQHHSTVTTTSDVQPVDLN
AESSSVT-SLYAPRINELKESVSSTIPYTLVVPRDRGSGKGGKDPIIQNHFKRPYP----

>Rapgef0_[Caenorhabditis_elegans]_71994090
------MDPR------------------------------------KPRQDPVNDAR---
------FYESLIKP-----------PHL-----RTPDDIRNVYEQLRQLDTFSNLFIGPL
KALCKTAR--YERHP--AQYILFRDGDVARSWYILLSGSVFIENQI---------YMPYG
C----------------FGKRTGQNH-RRTHNCL-LLQESEMIVIDYPTEPQ--------
--------------------------SNGMSPRT--------------------------
-----------------------------------PPR----------------------
-------GIHHSGEPVH----------------------QKTP-----------------
--------------RKSA----PNMSVD--------------------------SIAM--
------------------PPPPVP-----------------------PRPLRLPQTAAKG
PAPLPPRGLPRTYPLDFPVDIPTTSSSS-----SNTSY----------------------

--NDQHRSQVYLNGLSADEDTLVRVKHRREKSNSVGGQAQNGISTARRLRGRSTASSTTT

EGETASNE-GAD-----SDEDEGSMPSQ------ESSSGGFMDLRDSVRECLEKEPSERN
SEDLA-----VLLDFMQHMSAFAALP-MSIKRQLC-LKMVFAVVNDAGTVV---------
--LAHNEKL--DSWSVIV
NGCVEVVKPSGER----V---------------------------------EYKLG-DSF
GAEPT-----------PATQIHI---GEMRTMVDDCEFVLVEHRDFCS------IMST--
---I--------------

------------------------GDHIEKDRDGLTGEVVSEVE------R---RT----
-------VGTH----C-----------------GQ---------------VLIKGKPDKL
IHHLVDERD-------HN---VDP---H------------------------YVD---D-
FLLTYRV--FI--------------RDPTTIFEKLM-----LWF------ADSIY-----
--RDKVARLVLL
WVNNHFNDFETN-DEMWNLLE----RFEGA--LERDGMHSQLSLLNIACSV-KAKPRQVI
LTR-RKDDKMMMRLV--GGQESG-------------------------------------

-------------------NS----------------------VY---------------
---VAEVFPDTSAAREGVKRADEMLEVNQQSAKYLSAKKAEDLLTGSLSLTLMLKNNVLG
YKETIGKIEHNKPK-NGTSRSGAG--------IPMVIPVHKTSITGKKSSTTSS--K---
------------------------SGMMEKLMTILKSSKEDSMDFTD----EAKI----S
SADLRPSRSNPD--IT------------SISQYYGPVRSECPEHVLK-------------
IYRNDQTFKYLPVYKETSAQNVVQ--------LALQ---------EFNMTAE-GSPEWSL
CECTVT----------------------IDGVIKQR--RLPPQMENLAERIALNSRYYLK
NNSR------SEP-LVPDEL---------------------------AP------ELLKE
AQTQLLSLNAQVVAAQLTLQDFSVFSAIEPTEFL-------DNLFKLDSK-YGS--PKLE
EFEQL---------FNREMWWV-----ATEICTERHVQKR------AKLIKKFIKVARY-
--------------CRDLRNFNSMFAIMS-GLDKPAVRRLHSSWE---------------
-------RVSSKYIRMLDE-IHQLV--------------------------DPSRNM---
-SKYRQHLAE-VAQ-------E-PPVV-PIYP----------------------------
VIKKDLTFAHDGNA--TY-SE-----------------------------K-LINFE-KL
-------RL---IAKSIRGVMKLSSAPYEIASMAERSGGV-VMDALLHMN---SFENS--
NV------------------ATMRK---GMSGKQNQPRKKVYEQALMVRKVKS-----YL
EGLHVVDNEMEL---DSMSYDI
-----------EPQVQTAHRGANSSSTANI-----------RRVPSPTPSSLSSQSAGSA
D------------QSSRHRLLFNGTGSISSAGGGSKFGVESPQAVQKMLSLVQNSKVKGA
-PPQITSPSTSARSSLQRNMPRVTGRQA----------TSSAQG-----------PVQLN
EETSTVTTYYQSDVLI--

>Rapgef0_[Calliphora_vicina]_158935664


```
------------------------------------------------------------
------------------------------------------------------------
------------------------------------------------------------
------------------------------------------------------------
------------------------------------------------------------
------------------------------------------------------------
------------------------------------------------------------
------------------------------------------------------------
------------------------------------------------------------
------------------------------------------------------------
------------------------------------------------------------
------------------------------------------------------------
-----------------T-------------------------------VATLKSG-DDF
GKLAL-----------INDAPRA---ATIVLKENNCHLLRVDKEHFNR------ILRD--
---------------------------------------------V--------------
------------------------------------------------------------
------------------------------------------------------------
------------------------EANTLRLQEHGKDVLVLER-----VAK---------
------QRGQ----HS---------------AFK---------------YTVMSGTPSKM
LEHLLET-------------RL---G---T-------QVSG--------VDPFLD---D-
FLLTHIV--FM---------------PVVQLVDELA-----N------------------
--------YF---HCDT---------N-----DG---AQTPDDRGYIVNFKKRVIQFMQK
WVMAVRHAAFDE-PSVCDFIE----ELAAE--VEAD-----------------PELQEET
SII---------------------------------------------------------
---HNI--------LTQMARYQD-DRNQNAGQK---------------------------
----WKLPP---NGQPIC--------------------------------LFSGNLT---
---PSKT---V-------------------------------------------------
------------------------------------------------------------
------------------------------------------------------------
------------------------------------------------------------
------------------------------------------------------------
------IRPDDD--II-----------------------------FR-------------
VYCADHTYCTLRFPLHTTAEIIKA--------CAADK-----------LQLNRGPEDLCL
VEVESN----------------------GERSV------FKDNDVSIPTGLSLNGRLFVS
VKDH---L-DALT-PLQEQ---------------------------EGP------TEAIE
I--DLELLSTKELAYYITTFDWDLFWAVHEYELLY-------HTFGRHHF--GKITANLD
VFLRR---------FNELQYWI-----VTEIVSAASMSKR------VGLLRKFIKLAAY-
--------------CKEYQNLNAFFAIVM-GLSNMAVSRLQQTWE---------------
-------KIPSKFRKLYQE-FEALI--------------------------DPSRNH---
-RAYRV----FVGK-------LQPPLI-PFMP----------------------------
LLLKDMTFAHEGNK--TS-LD-----------------------------G-LVNFE-KM
-------HM---MAQTMRTLRFCRS--R------SLGL-----------------EPP--
----------------------SPKSE-----G----------------EVRS-----FI
SCLRVI------------------------------------------------------
------------------------------------------------------------
------------------------------------------------------------
------------------------------------------------------------
------------------------------------------------------------
------------------------------------------------------------
------------------------------------------------------------
------------------------------------------------------------
------------------------------------------------------------
------------------------------------------------------------
------------------------------------------------------------
```


>Rapgef0_[Dictyocaulus_viviparus]_768188889

--MAGRQLVI
PEKR-ITF--SFQSPQRDQN---------------------------GPLE-------SV
HASVMEYVGSYELAQQLYYLHTQLFEATDEIELVT-------QVIGRDQF-PGRIPSNLD
LLMRR---------FNEVQYWA-----TTEVLLAS-PQKR------VHTLRKFIKIALYA
---------------KENRDLMTLFAITL-GLSNVAVSRLTNLWD---------------
-------RLPNKLRRQFAE-FESLL--------------------------NPSRNH---
-RPYRA----LVSK-------MSPPMI-PFVP----------------------------
LLLKDLTFIHEGNK--TY--------------------------Y-----NGLVNFE-KM
-------HM---IANILRSFRQCKSRCTG-------------------------------
-------------------SQVESKKI-----S----------------ETQN-----FI
RNFCVVDNQRRFVYVWSFVTNRIDNTNKNVKQKILYRFDRYAIEVDGVIVSNRTASKKEL
TSDEVVEFGLPEESRQRKHVNTLNTTTR--------------------------------

>Rapgef0_[Ixodes_scapularis]_241789371

---MCSKWSEP---
--PR--RRLSAT------------------------SARD-----G--------------
STRRRESRAQANGTLRTIPPPATGKPASEPGPPPS-------------------------

---------------------------------------RLPPSPTEGFHSNRLLL----
---AQPTAQVY-------------RA---------------------GWVLR-TLLLYQA
PQ-L----------------VRDRKYH------T-RHF----------------RRC-MV
GT-----------------EMVDWLLQVP-------------------------------
PTHMRLN--------SRAQAAGMW---QVLLEEGVIK-----------------------
--------------H------------------------VTH-EHQFKDKY-LFYRFRED
-------EEEP----TK-L---
--PT
SDDR--LHAEEEL------QDVLLLLMQLTPDAV-------------LRMILRKPPLERT
LDDLD-----LIYEELLHIKALSHLS-NSIKRELY-GVMIFESHATSGTIL---------
--FNQGDEG--KSWYIIL
RGSVNVVIYGK-----GI-------------------------------VCSLHEG-DDF
GKLAL-----------VNDAPRS---ATIVTREDNCHFLRVDKEDFNR------ILRD--
---V--------------

------------------------EANTVRLKEHGQDVLLLQK-----IPA-----DARN
SN--GAHS-H--

---YK-------------
VYCADHTYTTLKTAVSATAECIKR--------NAAEK-----------LSLK-DD--LVL
VEVKSS----------------------GERIS------FKDSEVSVPTGLSINGRIFIS
PVDH---L-DALT-PLSEQ---------------------------EGP------LEGTG
AL--LETLSSHDIAYHMSLYDWYLFSCIHEYELIY-------QVFGRHQF-RK-IMSNLD

```
VFQRR---------FNEVQFWV-----VTEMCLATSLSRR------VQLLRKFIKIAAH-
--------------CREYQNLNAFFAIVM-GLSNVAVSRLSQTWE---------------
-------RLPGKLKRTFAE-FETLI--------------------------DPSRNH---
-RRYRV----AVSK-------ISPPLV-PFMP----------------------------
LLLKDMTFCHEGNK--TY-ID-----------------------------G-LVNFE-KM
-------HM---IGQTLRSLRHSRS--Q-------------------------RINLEP-
-P------------------PQ-GKVQ-----Q----------------DVRE-----YI
RTLKVIDNQRRL-----------------------------------------TQLSHAL
-----------EPRR----------P----------------------------------
------------------------------------------------------------
------------------------------------------------------------
------------------------------------------------------------
------------------------------------------------------------
------------------------------------------------------------
------------------------------------------------------------
------------------------------------------------------------
------------------------------------------------------------
------------------------------------------------------------
------------------------------------------------------------
---
>Rapgef0_[Necator_americanus]_915250685
------------------------------------------------------------
------------------------------------------------------------
------------------------------------------------------------
-----------------------------------MLNIQEFVQIPYRV-----------
------------------------------------------------------------
------------------------------------------------------------
------------------------------------------------------------
------------------------------------------------------------
------------------------------------------------------------
------------------------------------------------------------
------------------------------------------------------------
------------------------------------------------------------
------------------------------------------------------------
------------------------------------------------------------
----------------------------------------------------STASSTTT
EGDDASGSAGADPVDSEEEDDDGSCPSH------ES----FVDLKDSVRECLEKEPTERN
NEDLA-----VLLDFMQHMSSFASLP-MSIKRQLC-LKMVFAVVNDAGTVV---------
--------------------------------------------MHHNEKL--DAWSVIV
NGLVEVVKPNGER----V---------------------------------EYKLG-DSF
GAEPN-----------PAVQYHV---GEMRTMVDDCEFVLVEHKDFCS------IMST--
---------------------------------------------I--------------
------------------------------------------------------------
------------------------------------------------------------
------------------------GEHIEKDRDGLTGEIVSETE------R---RT----
-------VGSQ----V-----------------GL---------------VLIKGKPDKL
IQHLVDDRD-------SN---VDP---H------------------------YVD---D-
FLLTYRV--FI--------------HNPVIIFEKLM-----MWF------ADGAL-----
--------------------------------------------------RDKIARIVLL
WVNNHYNDFETN-DEMTKLLE----RFEGA--LERDGMHSQQSLLNIACSV-KARSRTVV
YSR-------------------A-------------------------------------
------------------------------------------------------------
------------------------------------------------------------
------------------------------------------------------------
```

-------------------NK----------------------VQ---------------
---V-----DSPAERCGMKRGDEILDVNGQPMKYLTLMKAQEIMKGSISLTITLKSNILG
FKELLGRTERDGSKISSKNRALAGALTQARGSMPNVIPVKST-----KAVKIGG--K---
------------------------TSMMDKLMTILKSSK-DGEDLVD----EART----T
TSPLRPSRSNPD--IT------------SISQYYGPVKSECPEHVLK-------------
IYRSDQTFKYLAVYKETTAQNVVQ--------LALQVSKAFMGCLEFNMTAE-GSLEWSL
CECTVT----------------------EDGVIKQR--RLPAQMENLAERIALNSRYYLK
NNNR------SEP-LVPDDL---------------------------AP------EIMRD
AQAQLLSLNAQVVAAQLTLQDFAVFASIEPTEYI-------DNLFQLESR-YGS--PRLE
EFERI---------FNREMWWV-----ATEVCSERVIQKR------AKLIKKFIKVARY-
--------------CRELRNFNSMFAIMS-GLDKPAVRRLHSTWE---------------
-------RISGKYIRMLED-IQQLV--------------------------DPSRNM---
-SKYRQHLAE-VSQ-------E-PPVV-PIYP----------------------------
VIKKDLTFSHEGNP--TY-CD-----------------------------K-LVNFE-KL
-------RL---IAKSVRAVSKLSSAPYEISSMAERSGGT-ISDALLHMN---TFEGG--
NV------------------ATMRK---G--GRVIQPRKKVYEQALMVRKVKA-----YL
DGLKVVDTESEL---DRLSYDI
-----------EPQ----------QAGPRI-----------RRAPSPSPSSVSSQSAESN
S------------TDQRR------------FRGGIMFGVDSPQAVQKMLGLVQTSKSK--
-DPSKKRSFPISTKP-----PTVP-----VTVVQPSRYAQ--------------------

>Rapgef0_[Oesophagostomum_dentatum]_732892745

--MIARIVLL
WVNNHYNDFETN-DEMTKLLE----RFEGA--LERDGMHSQQSLLNIACSV-KARARTVI
YTR-------------------A-------------------------------------

-------------------NKDDPLHFAIQGGKESGCGIFVNEVQ---------------
---V-----DSPAERCGIKRGDEVLDVNGQPMKYMALAKAQEMLKGSISLTLTLKSNVLG
FKELLGRSERDGSKVAAKNRALAGALCQSRGSIPNVIPVKTA-----KAVKIGG--KQSL
AFMAIGYITNSFFIAFVEIVKVGKTSMMDKLMTILKASK-DGEDLPD----EART----T
MSPLRPSRSNPD--IT------------SISQYYGPVKSECPEHVLK-------------
IYRSDQTFKYLAVYKETTAQNVVQ--------LALQ---------EFNMTAE-GSLEWSL
CECTVT----------------------EDGVIKQR--RLPPQMENLAERIALNSRYYLK
NNNR------SEP-LVPDDL---------------------------AP------EIMRE
AQAQLLTLNAQVVAAQLTLQDFAVFASIEPTEYI-------DNLFQLESR-YGS--PRLE
EFERI---------FNREMWWV-----ATEVCSERNVQKR------AKLIKKFIKIARH-
--------------CRELRNFNSMFAIMS-GLDKPAVRRLHSTWE---------------
-------RISGKYIRMLED-VQQLV--------------------------DPSRNM---
-SKYRQHLAE-VSQ-------E-PPVV-PIYP----------------------------
VIKKDLTFSHEGNP--TY-CE-----------------------------K-LVNFE-KL
-------RL---IAKSVRAVSKLSSAPYEISSMAERSGGT-ISDALLHMN---TFEGG--
NV------------------ATMRK---G--GRVIQPRKKVYEQALMVRKVKS-----YL
DGLKVVDTESEL---DRLSYDI
-----------EPQ----------QSGPRV-----------RRAPSPSPSSVSSQSAESN
S------------TDQRR------------FRGGGMFGVDSPQAVQKMLGLVQTSKSKGV
PSPSQSPAFSSASRPLQRNVPRVSSRQQSVPTLQLQRTPSFKQE-----------PVDLN
KESSSVTTFYGADVLI--

>Rapgef0_[Pediculus_humanus_corporis]_242021899

-------------------------M-STSNLSV-FNKENFFFDFSFFIFFLFFQ-----
KNKD--SMSEFI--------------G-NSKHKNGYGGKP-GAMTY-----TMTSTG---
-NKPQ----------------------PSSPDIPDSVS----------------------

--PILE--------------
-----------------SPSAPVSHI---------------------GWVLR-SVLLCKT
IL------------------LKDRKIK------N-K-I---------------LWKC-GS
GI-----------------ELVDWLCD---------------------------------
---QMDT--------SRQQATIMW---QVLIEEGVLI-----------------------
--------------H------------------------VNR-EHIFKDKN-LLYQFWED
E---------D----GG---
---GKLPN

QKEL--NEAVECL------SECLAILAKRAPDAA-------------LRMMLRKPSHERT
PEELE-----IIYEELIHITALSHLS-NTVKRELT-SVIVFEAHTKAGTIL---------
--FEQGEEG--KSWYIIL
RGSVDVVIYGK-----GT-------------------------------VTVLQEG-DDF
GKLAL-----------VNDTPRA---ATIILRENNCHFLRVDKENFNR------ILKD--
---V--------------

------------------------EANTVRLQEHGNDVLVLEK-----TIQ---------
------NQHHK----S---------------QFN---------------YVVKAGTPEKM
LEHLLET-------------RMKIRG-----------SVGGKESLQVPHSDYFVD---D-
FLLTYVA--FM---------------TTTDL----------R------------------
--------FFLN-GLNT---------Y-----RA---FLPNQNRDYALACKKKVIRFIHR
WVVTIRYPVFSS-SGFRSFLQ----EISKE--IETES--------ST-----YRSLEEEA
SLM---
---HHI--------LTEMVRHDD-EVKKHSGKK---------------------------
----WKLPP---YGQPIS---------------------------------LFSGGTG---
---PQRM---

------MKPDDD--II-----------------------------FR-------------
VYCADHTYCTLRMPLNSTAEAIKL--------SAADR-----------LKL-RNFDDLAL
AEVKSS----------------------GERII------IKDQDVNVPTALSLNGRLFVS
PRDH---L-DALT-IVPEQ---------------------------EQP------TECEE
N--DIESFSTKELAYHMTHFDWELFCCINEYEMLY-------HVIGKHHF--GELTANLE
MFLRR---------FNEIQYWV-----ATEILHTQSLSKR------VTVLRKLIKLASY-
--------------CKEYQNFNAFLAIIT-GLNNQAVSRLSLTWE---------------
-------KLPNKFRKMFTE-FEILI--------------------------DSSRNH---
-RAYRA----AVGK-------LQPPVL-PFMP----------------------------
LLIKDMNCAQENHK--TF-TD-----------------------------G-LVNFE-KM
-------HL---FAQTIRTMRYSRS--R------HLTL-----------------EPP--
----------------------SPKSE-----G----------------NIRS-----YI
SCLRVIDNQKQL---MSLSQKL
-----------EPRR----------S----------------------------------

>Rapgef0_[Wuchereria_bancrofti]_402590062


```
------------------------------------------------------------
------------------------------------------------------------
------------------------------------------------------------
------------------------------------------------------------
------------------------------------------------------------
------------------------------------------------------------
------------------------------------------------------------
------------------------------------------------------------
------------------------------------------------------------
------------------------------------------------------------
------------------------------------------------------------
------------------------------------------------------------
------------------------------------------------------------
----------------------------MRTLVDDCEFVLVEHRDYCN------IMST--
---------------------------------------------I--------------
------------------------------------------------------------
------------------------------------------------------------
------------------------SEHIEQHSDGITGEIVSEIE------R---RS----
-------VGNQ----M-----------------GQ---------------VLIKANRDKL
IEHLVEERD-------TA---VDA---H------------------------YIE---D-
FLLMYRV--FI--------------NDPTMIFEKLM-----HWF------AESNL-----
--------------------------------------------------RDKIARIVLL
WVNNHFNDFECN-GEMMQLLD----RFEQA--LERDQMHSQQSLLNIACSV-KSRTRNIV
YSR---------------------------------------------------------
------------------------------------------------------------
------------------------------------------------------------
------------------------------------------------------------
-------------------SSRD---------------------QPLSLSILGGKENGSS
GIFVSDVQRGSRAEKIGLKRGDQIIEVNGQSFKKISLIRALEVLRSNTHLSITVKSNLLG
FKEMIARSERAVDVEDQCNAYRKGA--EIRYTKHRAVSKQSESVQRGSVSCMIG-MNPRT
GASSFSYLPPSGMISSVG----NKTSMMDKLLTMLKGLP---STLPDGPIAESKTLNPTR
SNSLRASRSNPD--IAGHSVGTSRISTMNIAQYYQPASSPCPEHVLK-------------
VYRADQSFKYLTIYKETTAQNVVQ--------LALQ---------EFGMSSDSGSLEWAL
CETTVT----------------------LDGVIKQK--RLPDQMNNLAERIQLNSRYYLK
NNSR------SEP-MVPDEL---------------------------AP------EVLKE
AETHLLQLNAQILAAQLTLQDFDVFSSIEPTEYV-------DNLFNLESK-YGW--PKLA
DFESL---------FNKEMWWV-----ATEICREKGLQKR------MKLIKKFIKVARY-
--------------CRDFRNFNSMFAIMS-GLEKPAVRRLHHTWE---------------
-------RVPSKYTKMFED-VQHLV--------------------------DPSRNM---
-SKYRQHLAI-VSQ-------E-PPVV-PIYP----------------------------
ILKKDLTFSHEGNP--TY-CE-----------------------------K-LINFE-KL
-------RM---IAKTIRSVTKLCSSPYEIGVMAQQSGGNDVSDALVNMN---SFDGGAG
TV------------------STMRKVGPNAMKTATQPRRKLYEQALMVRKVKS-----YL
SNLNVVECETDL-----------------------------------------DQLSYEC
-----------EPSY--------GSTNVTR-----------RRAPSPSPSSLSSQS----
S------------TSDQHRI------------SAPKFGVESPHAVQKMLSLAQNSRLKQG
---CSGNLILVS--PLQSPLLPSKGVRLRNTPLPVRRVPSTSQHHSTVTTTSDVQPVDLN
AESSSVTRGL--------------------------------------------------
------------------------------------------------------------
------------------------------------------------------------
------------------------------------------------------------
------------------------------------------------------------
------------------------------------------------------------
------------------------------------------------------------
```


>Rapgef3_[Acinonyx_jubatus]_961722103

---------------------M--------------------------------------

---------------K---------------------------------VGWPGES----
-----RWQVGLAVED-SPALGASQVRG----LPDVVPEGTLLNMVLKRMHRPRSCSYQ-L
LLE------------------------HQRP-S---------CIQ---------------
------------------GLR-------------------WT------------------
-----PLTDSEESLDFSV-SLEQA-------------------S--------T-------
-------ERVL-------------RA---------------------GKQLH-RHLLATC
PT-L----------------IRDRKYH------L-RLY----------------RQC-CS
GR-----------------ELVDGILALS------------------------------L
----GVH--------SRNQAVGIC---QVLLDEGALC-----------------------
--------------H------------------------VKH-DWTFQDRDTQFYRFPGP
-------EPEP--VGVHEL---

---------EEEL------VEAMALLSQRGPDAL-------------LTVALRKPPGQRT
DEELD-----LIFEELLHIKAVAHLS-NSVKRELA-AVLLFEPHSRAGTVL---------
--FSQGDKG--TSWYIIW
KGSVNVVTHGK-----GL-------------------------------VTTLHEG-DDF
GQLAL-----------VNDAPRA---ATIILREDNCHFLRVDKQDFNR------IIKD--
---V--------------

------------------------EAKTMRLEEHGKVVLVLER-------A------SQG
AG-----PSRP------------------PTPGRN------------R-YTVMSGTPEKI
LELLLEA------------MRPDSSAHDPT-ETF-------------------LS---D-
FLLTHSV--FM---------------PSAQLCAALL-----HHF------H---------
-----------------------------AEPS---GGSEQEHSTYVCNKRQQVLRLVSQ
WVALYGPMLYAD-PVATSFLQ----KLSDL--VSRDA--RLCILLR---EQ-WPERRK-H
HRM----ENGCGNA--SP------QMKAR----------------------NMP------
-----VW-----------LPGQD--------QP---------------------------
-----------------L--
---PSSN---

----CAIRVGDK--VP-----------------------------YD-------------
IYRPDHSVLTLRLPVTASVREVMA--------ALAQEDG-----WT-------KGQ--EL
VKVNSA----------------------G------DAIGLPPDARGVATSLGLNERLFVV
NPQE---V-RELT-PLPEQL---------------------------GP------SVGSA
EG--LDLVSAKDLAGQLTDHDWNLFNSIHQVELIH-------YVLGPQHL-RDVTTANLE
RFMRR---------FNELQYWV-----ATELCLCPVPGLR------AQLLRKFIKLAAHL
---------------KEQKNLNSFFAVMF-GLSNSAISRLAHTWE---------------
-------RLPHKVRKLYSA-LERLL--------------------------DPSWNH---
-RVYRL----ALTK-------LSPPII-PFMP----------------------------
LLLKDMTFIHEGNH--TL-VE-----------------------------N-LINFE-KM
-------RM---MARAVRMLHHC-------------------------------------

>Rapgef3_[Ailuropoda_melanoleuca]_752406281

---------------------M--------------------------------------

---------------K---------------------------------VGWPGES----
-----RWQVGLAVED-SPALGVSQVGG----LPAVVPEGTLLNMVLKRMHRPRSCSYQ-L
LLE------------------------HQRP-S---------RIQ---------------
------------------GLR-------------------WT------------------
-----PLTDSEESLDFSV-SLEQA-------------------S--------A-------
-------ERVL-------------RA---------------------GKQLH-RHLLATC
PT-L----------------IRDRKYH------L-RLY----------------RQC-CS
GR-----------------ELVDGILALG------------------------------L
----GVH--------SRSQAVGIC---QVLLDEGALC-----------------------
--------------H------------------------VKH-DWTFQDRDTQFYRFPGS
-------EPEP--VGIHEL---

---------EDEL------VEAVALLSQRGPDAL-------------LTVALRKPPGQRT
DEELD-----LIFEELLHIKAVAHLS-NSVKRELA-AVLLFEPHSKAGTVL---------
--FSQGDKG--TSWYIIW
KGSVNVVTHGK-----GL-------------------------------VTTLHEG-DDF
GQLAL-----------VNDAPRA---ATIIILREDNCHFLRVDKQDFNR-----IIKD--
---V--------------

------------------------EAKTMRLEEHGKVVLVLER-------A------SQG
AG-----PSRP------------------PTPGRN------------R-YTVMSGTPEKI
LELLLEA------------MRPDSSAHDPT-ETF-------------------LS---D-
FLLTYSV--FM---------------PSAQLCAALL-----HHF------H---------
-----------------------------AEPS---GGSEQEQSTYICNRRQQILRLVGQ
WVALYGPMLHTD-PVATSFLQ----KLSDL--VNRDA--RLCILLR---EQ-WPERRR-H
HRL----ENGCGNA--SP------QMKAR----------------------NMA------
-----VW-----------LPGQD--------QP---------------------------
-----------------L--
---PSSN---

----CAIRVGDK--VS-----------------------------YD-------------
ICRPDHSVLTLKLPVTASVREVMA--------ALAQEDG-----WT-------KGQ--VL
VKVNSA----------------------G------DAIGLQPEARGVATSLGLNERLFVV
NPQE---V-HELT-PHPEQL---------------------------GP------TVGSA
EG--LDLVSAKDLAGQLTDHDWNLFNSIHQVELIH-------YVLGPQHL-RDVTTANLE

```
RLMRR---------FNELQYWV-----ATELCLCPVPGLR------AQLLRKFIKLAAHL
---------------KEQKNLNSFFAVMF-GLSNSAVSRLAHTWE---------------
-------RLPHKVRKLYAA-LERLL--------------------------DPSWNH---
-RVYRL----ALTK-------LSPPII-PFMP----------------------------
LLLKDMTFIHEGNH--TL-VE-----------------------------N-LINFE-KM
-------PLPHSLVCGEDSLHYVTWQAEPFRVSSALSPPRSLFFLPPPDAPPVPLSPLRS
RVSHLHEDSQAARTSTCSEQSLSTR-------S--------------PASPWA-----YV
QQLKVIDNQREL-----------------------------------------SRLSREL
-----------EP-----------------------------------------------
------------------------------------------------------------
------------------------------------------------------------
------------------------------------------------------------
------------------------------------------------------------
------------------------------------------------------------
------------------------------------------------------------
------------------------------------------------------------
------------------------------------------------------------
------------------------------------------------------------
------------------------------------------------------------
---
>Rapgef3_[Alligator_mississippiensis]_950944900
------------------------------------------------------------
------------------------------------------------------------
------------------------------------------------------------
------------------------------------------------------------
------------------------------------------------------------
------------------M--------HLFRSYSYHI-------------QLRGE-AGGL
AGVRG-------IRW--------------------------T------------------
-----PLPE-------SEASLDYGH------------------SLTQAS---S-------
-------DRVR-------------RAGRLLY---------------S----C---LLSTC
PN-L----------------IRDHKHH------L-RHH----------------RQC-CS
GK-----------------ELVDWLLRPG------------------------------F
----SMQ--------MRSQAIGVC---QVLVDGGVLT-----------------------
--------------H------------------------VKQ-EWHFQDKDTQFYRFAEL
D-----LSPEP-SAGVRDP-----------------------------------------
------------------------------------------------------------
----------EEL------LEALAFLAQLGPDAL-------------LTMALRKPPAQRT
EDELE-----LIFEELLHIKAVAYLS-SSVKRELA-SVVLFESHARAGTV----------
-------------------------------------------LFSQGDKG--TSWYILW
KGSVNVVTHGK-----GL-------------------------------VTTLHEG-DDF
GQLAL-----------VNDAPRA---ATIIILREDSCHFLRVDKHDFNR-----ILKD--
---------------------------------------------V--------------
------------------------------------------------------------
------------------------------------------------------------
------------------------EANTIRLKEHGKVVLVLEKN--------------SG
L------GGHV------------------AVAGSS-R------------YSVMAGTPEKI
LEHLLEA------------MRLDATFYDPM-DT------------------LLGD---F-
LLT---YSLFM---------------PMAQLCRALL-----HHF------Q---------
-----------------------------AEPSE---GSEQEKATYALSKRQKILRLVGQ
WGLLYGRLLQAE-PSARALLQ----SLSE--AVSQDP--RLCGLLW---DQ-LQDRRR--
HRA---LENGSGNM--SP------QIKVRS---------------------PGH------
------W-----------FASHE--------EA---------------------------
----------------TL------------------------------------------
----GCSS-AI-----------------MAGDR---------------------------
```


--------------VP-----------------------------YD-------------
IYRPDHSCLTVMLPVNASARDVLV--------SLAPRLS-------------QDGD-LVL
VKVNSA----------------------GERVG------LPMDAIGVYTSLGLNERLFVV
NVQE---L-GSLT-PHPEQL---------------------------GP------TAGSW
ET--LELLSSKDLASQLTDHDWTLFSSVHQVELLY-------HVLGPQKF-PGATTANLE
RIMRR---------FNELQYWV-----STELCLCGDVARR------AQLLRKFIKLAAHL
---------------KEQKNLNSFFAVMF-GLSNTAVSRLAKTWE---------------
-------RLPHKVRKLHAV-LERML--------------------------DPSWNH---
-RVYRL----AVAK-------LSPPIF-PFLPL-LLK-----------------------
----DLTFIHEGNR--TLS------------------------------EN-LINFE-KM
-------RM---MAKAVRILHRCRS--HF----------------------HTPVSPLRS
RSPHRLEDPKALRAST-------------------------------------SDF----
-----------C---S------
------------------------KLKRGAQGT---------------------------

>Rapgef3_[Alligator_sinensis]_944358354

---GGL
AGVRG-------IRW--------------------------T------------------
-----PLPE-------SEASLDYGH------------------SLTQAS---S-------
-------DRVR-------------RAGRLLY---------------G----Y---LLSTC
PN-L----------------IRDHKHH------L-RHH----------------RQC-CS
GK-----------------ELVDWLLLPG------------------------------F
----SMQ--------TRSQAIGVC---QVLVDGGVLT-----------------------
--------------H------------------------VKQ-EWHFQDKDTQFYRFAEL
D-----LIPEP-GAGVRDP---

----------EEL------LEALAFLAQLGPDAL-------------LTMALRKPPAQRT
EDELE-----LIFEELLHIKAVAYLS-SSVKRELA-SVVLFESHARAGTV----------
---LFSQGDKG--TSWYILW
KGSVNVVTHGK-----GL-------------------------------VTTLHEG-DDF
GQLAL-----------VNDAPRA---ATIILREDSSHFLRVDKHDFNR------ILKD--
---V--------------

------------------------EANTIRLKEHGKVVLVLEKN--------------SG

```
L------GGHV------------------AVAGSS-R------------YSVMAGTPEKI
LEHLLEA------------MRLDATFYDPM-DPGPP---------------RDGG---S-
PLT---YSLFM---------------PMAQLCRALL-----HHF------Q---------
-----------------------------AEPSE---GSEQEKATYALSKRQKILRLVGQ
WGLLYGRLLQAE-PSARALLQ----SLSE--AVSQDP--RLCGLLW---DQ-LQDRRR--
HRA---LENGSGNV--SP------QIKVRS---------------------PGH------
------W-----------FASHE--------EA---------------------------
----------------TL------------------------------------------
----GCSS-AI-----------------MAGDR---------------------------
------------------------------------------------------------
------------------------------------------------------------
------------------------------------------------------------
------------------------------------------------------------
--------------VP-----------------------------YD-------------
IYRPDHSCLTVMLPVNASARDVLV--------ALAPRLS-------------QDGD-PVL
VKVNSA----------------------GERVG------LPMDAIGVYTSLGLNERLFVV
NVQE---L-GSLT-PHPEQL---------------------------GP------TTGSW
ET--LELLSSKDLASQLTEHDWTLFSSVHQVELLY-------HVLGPQKF-PGATTANLE
RIMRR---------FNELQYWV-----STELCLCGDVARR------AQLLRKFIKLAAHL
---------------KEQKNLNSFFAVMF-GLSNTAVSRLAKTWE---------------
-------KLPHKVRKLHAV-LERML--------------------------DPSWNH---
-RVYRL----AVAK-------LSPPIF-PFLPL-LLK-----------------------
----DLTFIHEGNR--TMS------------------------------EN-LINFE-KM
-------RM---MAKAVRILHRCRS--HV----------------------HTPVSPLRS
RSPHRLEDPKALRAST--------------------------------------------
-----------C-----------------------------------------SEQSLSI
RSPTSASVY-LQHLRVIDNQKELLRLSRDLES----------------------------
------------------------------------------------------------
------------------------------------------------------------
------------------------------------------------------------
------------------------------------------------------------
------------------------------------------------------------
------------------------------------------------------------
------------------------------------------------------------
------------------------------------------------------------
------------------------------------------------------------
------------------------------------------------------------
---
>Rapgef3_[Anolis_carolinensis]_327279590
------------------------------------------------------------
------------------------------------------------------------
------------------------------------------------------------
-------------------MKVVRSR-QGGWNVGLPLGESSDLPQLSEGLPEDPISD---
--NN----LLDVIWR--------------------------------KMHLFRSCSYR-I
Q------------------------LEGQVGSAPRIQ-----------------------
------------------GIR-------------------WT------------------
-----PLPDRESALVPSH-RVKQA------------------------S---S-------
-------NKIQ-------------KA---------------------GRLLG-AHLVTTY
PD-L----------------IRDHKYH------L-RLY----------------RQC-CS
GK-----------------ELVDALLRAG------------------------------L
----SVQ--------TRSQALGLC---QVLMDEGVLS-----------------------
--------------H------------------------VRQ-ESYFQDRDAQFFRFVAL
-------EV------NAE------------------------------------------
-----------------DRDS---------------------------------------
```

----------EEL------LEALALLTQLGPTAL-------------LTTILRKPPGQRT
DEELA-----LIFEELLHIKAVAHLS-SSVKRELA-AVLLFESHTKAGTVL---------
--FSQGDKA--TSWYIIW
KGSVNVVTHDK-----GL-------------------------------VSTLHEG-DEF
GQLAL-----------VNDAPRA---ATIILREDNCHFLRVDKRDFNR------ILKD--
---V--------------

------------------------EANTMRLKEHGKVVLVLEK-------N---------
SQ-GSN---LP------------------GTPGGS------------R-YSVMAGTPEKI
LDYFLEV------------MRPDSMLSDPV-DTG-------------------LG---D-
FLLTHDV--FM---------------PTPQLCRTLF-----QHF------H---------
-----------------------------SEPSE---GTEQEKAAYILSKRQKILRLVNQ
WVSLYGRLLQAE-PSAVSFLQ----NLSER--VAQDF--RLGSLLK---EQ-LQDRRR--
NRT---LENGSNNA--SPT----LQVR-NVVD----------------------------
------W-----------FARQE--------EP---------------------------
-----------------Y--
---LNSS---

----YTIRAKDK--VM-----------------------------YD-------------
IYRPDHSCLTTLLQVNAAVQEVLA--------SLSDCPGWS------------KDW--IL
IKVNSS----------------------G------DKEVLPMETFGVFTSLGLNERLFAV
TAQE---L-HALT-PHPEQQ---------------------------CP------TVGSL
ET--LEFISSKDLANQLTEHDWNLFKSIHQVELVY-------YIFGPQKF-PNATTANLE
RFLRR---------FNELQFWV-----TTELCLCPDVIKR------AQLLRKFIKMAAHL
---------------KDQKNLNTFFAVMF-GLSHSAVSRLSKTWE---------------
-------KLPHKTRKLFNT-MERML--------------------------DPSWNH---
-RIYRL----AMAK-------LTPPMI-PFMP----------------------------
LLLKDMTFIHEGNR--TS-VE-----------------------------N-LVNFE-KL
-------HM---MAKAVHLVHHCRSLPN------------------------LPLSPLRS
RPQHLLEEARAMRASTCSEQSLSTR-------S--------------PAATWV-----YL
QHLKVSDNQKEL---LRLSRDL
-----------ES---

>Rapgef3_[Anser_cygnoides_domesticus]_902933497

---MAEP---------
---P--------------------------------------PRPPTPFFL---------
----------------------------VFQ-----------------------------

```
-----------------------------------------T------------------
-----PLLD-------TESSLEYGH------------------SLTQAS---P-------
-------EKIW-------------RAGKLLF---------------T----H---LTSTC
PG-L----------------IRDHKHH------L-RHH----------------RQC-CS
GK-----------------ELVDWLLNAG------------------------------L
----GVQ--------MRSQAVGVG---QVLVDGGVLT-----------------------
--------------H------------------------VKQ-EWHFQDKDTQFYRFAEL
E-----LSPEP-GAGLRDA-----------------------------------------
------------------------------------------------------------
----------EEL------LEAVAFLAQLGPDAL-------------LTMALRKPPAQRT
EDELE-----LIFEELLHIKAVAHLS-NSVKRELA-AVLMFESHQRAGTV----------
-------------------------------------------LFSQGDKG--TSWYIVW
KGSVNVVTHGK-----GL-------------------------------VATLHEG-DDF
GQLAL-----------VNDAPRA---ASIILREDNCHFLRVDKQDFNH------ILKD--
---------------------------------------------V--------------
------------------------------------------------------------
------------------------------------------------------------
------------------------EANTMRLKEHGKVVLVLQKNL-------------QG
G------SSQP------------------AAARSS-R------------YLVMAGTPEKI
LEHLLEF------------MRLDATLYDPV-D-------------------TLLG---D-
FL----------------------------LCHPILVPLTGPSF------R---------
-----------------------------AEPLE---GSEQEKATYSLHKRRKILRLVSQ
WVLLYGRLLQGD-RSPTALLQ----NLAD--LASRDP--RLGGMVQ---EQ---ERRR--
PRA---LENGDGSA--SP------QPKARS---------------------SVS------
------W-----------LA-----------EA---------------------------
----------------LT------------------------------------------
----PA------------------------------------------------------
------------------------------------------------------------
------------------------------------------------------------
------------------------------------------------------------
------------------------------------------------------------
--------------VP-----------------------------YE-------------
IYRPDHSCLVTVLPINASVRDVLR--------SLAPRLG-------------RDRE-HLL
VKVNSA----------------------GDKVG------LQLDAVGVFTSLGLNERLFAV
SVEE---L-GGLT-PHPEQL---------------------------GP------HVGSS
ET--LDLISSKDLASHLTDYDWNLFKSIHQVEMIH-------YIVGPQKF-HEVTTANLE
RVMRR---------FNELQYWV-----ATELCLCPEVGRR------AQLLRKFIKLAAHL
---------------KEQKNLNSFFAVMF-GVSNTAVSRLAKTWE---------------
-------RLPHKIRKLHAA-LERML--------------------------DPSWNH---
-RVYRL----AVAK-------LSPPII-PFVPL-LLKGGCRRFG-------GAGSPLHA-
----SSLSQRPGNQ--AQGSLWTPLLLGWGVTGQPLLKPQGACVFPAGEQN-GVGWQ-QV
TFVHCPQHM---MAKTVRVLQRCRG--QA----------------------HAPLSPLRS
RSPHRPEDPKAVRMST--------------------------------------------
-----------C-----------------------------------------SEQSLSV
RSPVSTWAY-LQHLKAIDSQKELLRLSRDLES----------------------------
------------------------------------------------------------
------------------------------------------------------------
------------------------------------------------------------
------------------------------------------------------------
------------------------------------------------------------
------------------------------------------------------------
------------------------------------------------------------
------------------------------------------------------------
------------------------------------------------------------
```


>Rapgef3_[Aotus_nancymaae]_817288343

---------------------M--------------------------------------

---------------K---------------------------------VGWPGES----
-----CWQVGLAVED-SSALGAPQVGA----LPDVVPEGTLLNMVLRRMHRPRSCSYQ-L
LLE------------------------HRRP-S---------RIQ---------------
------------------GLR-------------------WT------------------
-----PLTNSEESLDFSE-SLEQA-------------------S--------T-------
-------ERVL-------------RA---------------------GKQLH-RHLLATC
PN-L----------------IRDRKYH------L-RLY----------------RQC-CS
GR-----------------ELVDGILALG------------------------------L
----GVH--------SRSQAVGIC---QVLLDEGALC-----------------------
--------------H------------------------VKH-DWAFQDRDAQFYRFPGP
-------EPEA--VGAHEM--

---------EEEL------AEAVALLSQRGPDAL-------------LTVALRKPPGQRT
DEELD-----LIFEELLHIKAVAHLS-NSVKRELA-AVLLFEPHSKAGTVL---------
--FSQGDKG--TSWYIIW
KGSVNVVTHGK-----GL-------------------------------VTTLHEG-DDF
GQLAL-----------VNDAPRA---ATIILREDNCHFLRVDKQDFNR------IIKD--
---V--------------

------------------------EAKTMRLEEHGKVVLVLER-------A------SQG
AG-----SSRP------------------PTPGRN------------R-YTVMSGTPEKI
LELLLEA------------MGPDSTAHDPT-ETF-------------------LS---D-
FLLTHRV--FM---------------PSAQLCAALL-----HHF------H---------
-----------------------------VEPA---GGSEQERSTYVCNKRQQILRLVSQ
WVALYGSMLHTD-LVATSFLQ----KLSDL--VSRDA--RLSNLLR---EQ-WPERRR-H
YRL----ENGCGNA--SP------QMKAR-----------------------NLS------
-----IW-----------LPNQD--------EP---------------------------
-----------------L--
---PGSS---

----CAIRVGDK--VP-----------------------------YD-------------
ICRPDHSVLTLQLPVTASVREVMALRGHMHPCILSTHPGLRPRCWE-------KQP--PS
LPNSTSTLVSSWSLASLPSPTQATSAVFA------DAIGLQPDARGVATSLGLNERLFVV
NPQE---V-HELT-PHPEQL---------------------------GP------TVGSA
EG--LDLVSAKDLAGQLTDHDWSLFNSIHQVELIH-------YVLGPQHL-RDITTANLE
RFMRR---------FNELQYWV-----ATELCLCAVPGPR------AQLLRKFIKLAAHL
---------------KEQKNLNSFFAIMF-GLSNSAISRLAHTWE---------------
-------RLPHKVRKLYSA-LERLLVSLSPAGPGSA---------------GISWAQPCL
PDTLIL----GFPE-------APLPVLWPVCPSGAPRGLWKRRGKSWGQRAGVWGPMNET
RLFADMTFIHEGNH--TL-VE-----------------------------N-LINFE-KM
-------RM---MARAARMLHHCRSHNP------------------------VPLSPLRS
RVSHLHEDSQVSRISTCSEQSL-TR-------S--------------PASTWA-----YV
QQLKVIDNQREL---SRLSREL
-----------EP---

>Rapgef3_[Aptenodytes_forsteri]_686593488

---MLVA---------
---I--GTQ-EALTS--P
S---
---T------------------
-----PLLD-------TESTLDYGH------------------SLTQAS---S-------
-------EKIW-------------RAGKLLF---------------I----H---LTSTR
PG-L----------------IRDHKHH------L-RHH----------------RQC-CS
GK-----------------ELVDWLLSAG------------------------------L
----AVQ--------TRSQAIGVC---QVLVDGGVLT-----------------------
--------------H------------------------VKQ-EWHFQDKDTQFYRFAEL
E-----LSPEP-STGLRDA---

----------EEL------LEALGFLAQLGPDAL-------------LTMALRKPPAQRT
EDELE-----LIFEELLHIKAVAHLS-NSVKRELA-SVLMFESHQRAGTV----------
---LFSQGDKG--TSWYIVW
KGSVNVVTHGK-----GL-------------------------------VATLHEG-DDF
GQLAL-----------VNDAPRA---ATIIILREDNCHFLRVDKQDFNH------ILKD--
---V--------------

------------------------EANTMRLKEHGKVVLVLQKNL-------------QG
G------SSQP------------------ATARST-R------------YLVMAGTPEKI
LEHLLEF------------MRLDATLYDPV-D-------------------TLLG---D-
FLLTYTV--FM---------------PTSQLCRALL-----HHF------R---------
-----------------------------AELLE---GSEQEKATYSLHKRRKILRLVSQ
WVLLYGRLLQGD-RSTTALLQ----NLAD--LASQDP--RLGGLVQ---EQ-AQDRRR--
PRA---LENGDGSV--SP------QPKARS---------------------SVN------
------W-----------LASQE--------EA---------------------------
----------------IL--
----NSSC-AL-----------------RAQDK---------------------------

--------------VP-----------------------------YE-------------
IYRPDHSCLITVLPVNASVRDILR--------SLAPRLG-------------RDGE-HVL
VKVNSA----------------------GDKVG------LQLDAVGVFTALGLNERLFAV
SVEE---L-GSLT-PHPEQL---------------------------GP------HIGSS
ET--LDLISSKDLASHLTDYDWNLFKSIHQVEMIH-------YIVGPQKF-HDVTTANLE

RVMRR---------FNELQYWV-----ATELCLCPEVGRR------AQLLRKFIKLAAHL
---------------KEQKNLNSFFAVMF-GVSNTAVSRLAKTWE---------------
-------RLPHKIRKLHSA-LERML--------------------------DPSWNH---
-RVYRL----AVAK-------LSPPII-PFVPL-LLK-----------------------
----DMTFIHEGNR--TLA------------------------------EN-LINFE-KM
-------HM---MAKTVRVLQRCRG--DA----------------------HAPLSPLRN
RSPHRPEDPKAVRIST--
-----------C---SEQSLSV
RSPVSTWAY-LQHLKAIDSQKELLRLSHDLES----------------------------

>Rapgef3_[Apteryx_australis_mantelli]_926500193

---MTEG---------
---A-----EHALRS----------------------------PPPQQG-----------
----SGAGGGSR--SLAHC--------HVVGGCGWAR-------------TFX-----SL
PRIQG-------IRW--------------------------T------------------
-----PLLD-------SESALDYGH------------------SLTQAS---S-------
-------EKIW-------------RAGKLLF---------------S----H---LVITC
PS-L----------------IRDHKHH------L-RHH----------------RQC-CS
GK-----------------ELVDWLLSAG------------------------------L
----AVQ--------TRSQAIGVC---QVLVDGGVLT-----------------------
--------------H------------------------VKQ-EWHFQDKDTQFYRFAEL
E-----LSPEP-SNGLRDA---

----------EEL------LEALAFLAQLGPDAL-------------LTMALRKPPTQRT
EDELE-----LIFEELLHIKAVAHLS-NSVKRELA-SVLMFESHQRAGTV----------
---LFSQGDKG--TSWYIIW
KGSVNVVTHGK-----GL-------------------------------VATLHEG-DDF
GQLAL-----------VNDAPRA---ATIILREDNCHFLRVDKQDFNR------ILKD--
---V--------------

------------------------EANTMRLKEHGKVVLILQKNL-------------QG
G------SSQP------------------AAARSS-R------------YSVMAGTPEKI
LEHLLEF------------MRLDATLYDPV-D-------------------TLLG---D-
FLLTYTV--FM---------------PTSQLCRALL-----HHF------R---------
-----------------------------AEPLE---GSEQEKATYSLHKRRKILRLVSQ
WVLLYGRLLQGD-RSTTALLQ----NLAD--LASQDP--RLGGMVQ---EQ-AQDRRR--
PRP---LENGGGSI--SP------QPKAQS---------------------SAN------
------W-----------FASQE--------EA---------------------------
----------------IL--
----NSSC-AL-----------------RAQDK---------------------------

--------------VP-----------------------------YE-------------
IYRPDHSCLITVLPVNASVRDILR--------SLTPKLG-------------RDGE-HVL
VKVNSA----------------------GDKVG------LQMDAVGVFTSLGLNERLFAV
SMEE---L-CGLI-PHPEQL---------------------------GP------STGSS
ET--LDLISSKDLASHLTDYDWNLFKSIHQVEMIH-------YIIRPQKF-HDVTTANLE
RVMRR---------FNELQYWV-----ATELCLCSEVGRR------AQLLRKFIKLAAHL
---------------KEQKNLNSFFAVMF-GVSNTAVSRLAKTWE---------------
-------RLPHKIRKLHSA-LERML--------------------------DPSWNH---
-RVYRL----AVAK-------LSPPII-PFVPL-LLK-----------------------
----DMTFIHEGNR--TLA------------------------------EN-LINFE-KM
-------HM---MAKTVRVLQRCRG--HA----------------------HAPVSPLRS
RSPHRLEDPKAVRIST--
-----------C---SEQSLSV
RNTVSTWAY-LQHLKAIDSQKELLRLSRDLES----------------------------

>Rapgef3_[Aquila_chrysaetos_canadensis]_768403046

---MKVR---------
---G-TGAGGEAARRGPGLVPVARSRQRTRRTGLEPGTGAVRPPPPSSSRPPPPSSPPPP
GGPGTDGNRLSV--LLKKM--------HLFRSSSYEI-------------RLEGE-GSSL
PRIQG-------IRW--------------------------T------------------
-----PLLD-------TESTLDYGH------------------SLTQAS---S-------
-------EKIW-------------RAGKLLF---------------T----H---LTSTR
PG-L----------------IRDHKHH------L-RHH----------------RQC-CS
GK-----------------ELVDWLLSAG------------------------------L
----AVQ--------TRSQAIGIC---QVLVDGGVLT-----------------------
--------------H------------------------VKQ-EWHFQDKDTQFYRFAEL
E-----LSPEP-SAGLRDA---

----------EEL------LEALAFLAQLGPDAL-------------LTMALRKPPAQRT
EDELE-----LIFEELLHIKAVAHLS-NSVKRELA-SVLMFESHQRAGTV----------
---LFSQGDKG--TSWYIIW
KGSVNVVTHGK-----GL-------------------------------VATLHEG-DDF
GQLAL-----------VNDAPRA---ATIILREDNCHFLRVDKQDFNH------ILKD--
---V--------------

------------------------EANTMRLKEHGKVVLVLQKNL-------------QG

G------SSQP------------------ATARSS-R------------YFVMAGTPEKI
LEHLLEF------------MRLDATLYDPV-D-------------------TLLG---D-
FLLTYTV--FM---------------PTSQLCRALL-----HHF------R---------
-----------------------------AEPLE---GSEQEKATYSLHKRRKILRLVSQ
WVLLYGRLLQGD-RSTTALLQ----NLAD--LASRDP--RLGGLVQ---EQ-AQDRRR--
PRA---LENGDGSV--SP------QPKARS---------------------SGN------
------W-----------LTSQE--------EA---------------------------
----------------IL--
----NSSC-AL-----------------RAQDK---------------------------

--------------VP-----------------------------YE-------------
IYRPDHSCLITVLPVNASVRDVLR--------SLAPRLG-------------RDGE-HIL
VKVNSA----------------------GDKVG------LQLDAVGVFTALGLNERLFAV
SVEE---L-GSLT-PHPEQL---------------------------GP------HVGSS
ET--LDLISSKDLASHLTDYDWNLFKSIHQVEMIH-------YIVGPQKF-HDVTTANLE
RVMRR---------FNELQYWV-----ATELCLCPEVGRR------AQLLRKFIKLAAHL
---------------KEQKNLNSFFAVMF-GVSNTAVSRLAKTWE---------------
-------RLPHKIRKLHSA-LERML--------------------------DPSWNH---
-RVYRL----AVAK-------LSPPII-PFVPL-LLK-----------------------
----DMTFIHEGNR--TLA------------------------------EN-LINFE-KM
-------HM---MAKTVRVLQRCRG--HA----------------------HAPLSPLRN
RSPHRPEDPKAVRIST--
-----------C---SEQSLSV
RSPVSTWAY-LQHLKAIDSQKELLRLSHDLES----------------------------

>Rapgef3_[Austrofundulus_limnaeus]_928045558

--MHLFRSHNYQ-V
F----PH-----------------RHAVQ-T--PTVR-----------------------
------------------GIS-------------------WT------------------
-----PLPEALDS---KD-TMKQ---------------------------FLS-------
-------DRVV-------------KA---------------------ARSVY-SVMIEKN
PG-L----------------IRDRKHH------L-KTY----------------RQC-CS
GK-----------------ELVDWLMKQT------------------------------E
----CLQ--------SRSQAVGMW---QVLVDEGILL-----------------------
--------------H------------------------VKH-ELNFHDKDSQFYRFQDS
-------EFGLNHMTNEK--
DL--

---------EDEL------QEALSLLCQLGPDAL-------------LTMILRKSPSQRS
AEDTE-----VIYEELLHVKAAAHLS-ASVRKELA-AVLVFESHAKAGTVL---------
--FSQGDKG--TSWYIIW
KGSVNVITHGK-----GL-------------------------------VTTLHEG-EDF
GQLAL-----------LNDAPRA---ATIILREDNCHFLRVDKQDFIR------ILKD--
---V--------------

------------------------EANTVRLEEHGKTVLVLEK-------S---------
EN----SSEQG------------------GAGANS------------K-YTVMSGTPEKI
LEHLLET------------MKLDLNGNDPI-DP-------------------CIS---D-
FLLTYKV--FM---------------SSSQLCAALQ-----NQY------Q---------
-----------------------------AELSE---GSEQEKDAYVLNTKQKVVKLIGQ
WVALYSFLLKED-PVTLDFLE----RMKKE--VAADC--RLSNILK---EQ-FKERRR--
TKM---LENGYQSL--S-------R-N-QQFD----------------------------
------W-----------FSHCE--------EP---------------------------

---SGRV---

----LPIRSQDK--VL-----------------------------YE-------------
IYKSDYKALSLMLPVDSSVQEVMS--------AIVQPG---------------GDH--VL
VKMNSS----------------------G------ERAQLKLDASSVYTALGLNERLFIC
TSSE---V-EQLR-PLKEQQ---------------------------GP------EQGTA
DL--IEQMSSKDIGIELTNYDWELFTAMHEVELVY-------YIFGRHKF-PGAITANLE
RFVRH---------FNEVQYWV-----VTELCLCEDLVKR------AVLLKKFIKIASVL
---------------KDQKNLNLFFAVMF-GLGNSAVQRLYKTWE---------------
-------RIPSKTKRIYCT-YERLM--------------------------DPSRNH---
-RAYRL----AVTK-------LSPPYI-PFMP----------------------------
LLLKDMTFIHEGNP--NC-LD-----------------------------K-LVNFE-KM
-------RM---LAKTVKIVRGCRSQPY------------------------VPSSPQRG
LADRIFLEGP-TRLSTSSEHAFPLR-------T--------------PSNIRH-----YI
QNLKVIDNQRKL---TQLSRSM
-----------EC---

>Rapgef3_[Balaenoptera_acutorostrata_scammoni]_594662221

---------------------M--------------------------------------

---------------K---------------------------------VGWPGES----
-----RWQVGLAVED-SSVLGAPPVGG----LPDVVPEGTLLSMVLRRMHRSRSCSYQ-L
LLE------------------------HQRP-S---------CIQ---------------

```
------------------GLR-------------------WT------------------
-----PLTNSEESLDFSV-SLEQA-------------------S--------T-------
-------ERVL-------------RA---------------------GKQLH-RHLLATC
PN-L----------------IRDRKYH------L-RLH----------------RQC-CS
GR-----------------ELVDGILALG------------------------------L
----GVH--------SRSQAVGIC---QVLLDEGALC-----------------------
--------------H------------------------VKH-DWAFQDRDTQFYRFPGS
-------EPEP--AGIHEL-----------------------------------------
------------------------------------------------------------
---------EEEL------AEALALLSQRGPDAL-------------LTVALRKPPGQRT
DEELD-----LIFEELLHIKAVAHLS-NSVKRELA-AVLLFEPHSKAGTVL---------
--------------------------------------------FSQGDKG--TSWYIIW
KGSVNVVTHGK-----GL-------------------------------VTTLHEG-DDF
GQLAL-----------VNDAPRA---ATIILREDNCHFLRVDKQDFNR------IIKD--
---------------------------------------------V--------------
------------------------------------------------------------
------------------------------------------------------------
------------------------EAKTMRLEEHGKVVLVLER-------T------SQG
TG-----PSRP------------------PTPGRN------------R-YIVMSGTPEKI
LELLLEA------------MRPDSSAHDPT-ETF-------------------LS---D-
FLLTHSV--FM---------------PTAQLCAALL-----HHF------H---------
-----------------------------AEPA---GGSEQECSTYICNRRQQILRLVSQ
WVALYGPMLHTD-TVATSFLQ----KLSDL--VSRDA--RLSNLLR---EQ-WPERRR-H
HRL----ENGCGNA--SP------QMKAR----------------------NMP------
-----VW-----------LPSQD--------EP---------------------------
-----------------L------------------------------------------
---PSSN-----------------------------------------------------
------------------------------------------------------------
------------------------------------------------------------
------------------------------------------------------------
------------------------------------------------------------
----CAIRVGDK--VP-----------------------------YD-------------
ICRPDHSVLTLQLPVTASVREVMA--------ALAQEDG-----WT-------KGQ--VL
VKVNSA----------------------G------DAVGLQPDARGVAASLGLNERLFVV
SPQE---V-HKLT-PHPEQL---------------------------GP------AVGSA
EG--LDLVSTKDLAGQLTDHDWSLFNSIHQVELIH-------YVLGPQPL-RDVTTANLE
RFMRR---------FNELQYWV-----ATELCLCPVPGLR------AQLLRKFIKLAAHL
---------------KEQKNLNSFFAIMF-GLSNSAISRLAQTWE---------------
-------RLPHKVRKLYSA-LERLL--------------------------DPSWNH---
-RVYRL----ALTK-------LSPPLI-PFMP----------------------------
LLLKDMTFIHEGNH--TL-VE-----------------------------N-LINFE-KM
-------RM---MARAARMLHHCRSHSS------------------------VPLSPLRS
RVSHLHEDSQATRISMCSEQSLSTR-------S--------------PASTWA-----YV
QQLKVIDNQREL-----------------------------------------SRLSREL
-----------EP-----------------------------------------------
------------------------------------------------------------
------------------------------------------------------------
------------------------------------------------------------
------------------------------------------------------------
------------------------------------------------------------
------------------------------------------------------------
------------------------------------------------------------
------------------------------------------------------------
------------------------------------------------------------
```


>Rapgef3_[Bison_bison_bison]_742108280

---------------------MVGGVGQNSKE-----GPDLWYAAAAA-GWRPLSAPDRQ
APVKVFLS--LPRLLA--GAQHLRLTSPEDSKEVLLLGAAEPCFGP--------AAPDPA
SWL-----SAVLFPSQ---------------------------------VGWPGEG----
-----RWQVGLAVEG-DSILGAPPLGG----LPDVVPEGTLLNMVLRRMYRSRSCSYQ-L
LLE------------------------HQRP-S---------CIQ---------------
------------------GLR-------------------WT------------------
-----PLTSSEESLDFSV-SLEQA-------------------S--------V-------
-------ERVL-------------RA---------------------GKQLH-RHLLANC
PN-L----------------IRDRKYH------L-RLH----------------RQC-CS
GR-----------------ELVDGILALG------------------------------L
----GVH--------SRSQAVGIC---QVLLDEGALC-----------------------
--------------H------------------------VKH-DWAFQDRDTQFYRFPGP
-------EPEP--VGVHEL---

---------EEEL------VEALALLSQRGPDAL-------------LTVALRKPPGQRT
DEELD-----LIFEELLHIKAVAHLS-NSVKRELA-AVLLFEPHSKAGTVL---------
--FSQGDKG--TSWYIIW
KGSVNVVTHGK-----GL-------------------------------VTTLHEG-DDF
GQLAL-----------VNDAPRA---ATIILGEDNCHFLRVDKQDFNR------IIKD--
---V--------------

------------------------EAKTMRLEEHGKVVLVLER-------T------SQG
TG-----PSRP------------------PTPGRN------------R-YTVMSGTPEKI
LELLLEA------------MRPDSSAHDPT-ETF-------------------LS---D-
FLLTHSV--FM---------------PTAQLCAALL-----HHF------H---------
-----------------------------AEPA---GGSEQERSSYICNKRQQILRLVSQ
WVTLYGPTLRTD-PVATSFLK----KLSDL--VSRDT--RLSNLLR---EQ-WPERRR-H
HRL----ENGCGNA--SP------QMKVS----------------------DLP------
-----FG-----------PPARG--------EP---------------------------
-----------------L--
---PSSN---

----CAIRVGDK--VP-----------------------------YD-------------
ICRPDHSMLTLQLPVTASVREVMA--------ALAQEDG-----WT-------KGQ--VL
VKVNSA----------------------G------DAIGLQPDARGVATSLGLNERLFVV
NPQE---V-HELT-PHPDQL---------------------------GP------TVGSA
EG--LDLVSTKDLAGQLTDHDWSLFNSIHQVELIH-------YVLGPQHL-RDVTTANLE
RFMRR---------FNELQYWV-----ATELCLCSVPGLR------AQLLRKFIKLAAHL
---------------KEQKNLNSFFAIMF-GLSNSAISRLAHTWE---------------
-------RLPHKVRKLYSA-LERLL--------------------------DPSWNH---
-RVYRL----ALTK-------LSPPLI-PFMP----------------------------
LLLKDMTFIHEGNH--TL-VE-----------------------------N-LINFE-KM
-------RM---MARAARMLHHCRSHSN------------------------VPLSPLRS
RVSHLHEDSQAVRVSTCSEQSLSTR-------S--------------PASTWA-----YV
QQLKVIDNQREL---SRLSREL
-----------EP---

```
------------------------------------------------------------
------------------------------------------------------------
------------------------------------------------------------
------------------------------------------------------------
------------------------------------------------------------
------------------------------------------------------------
------------------------------------------------------------
------------------------------------------------------------
------------------------------------------------------------
------------------------------------------------------------
---
>Rapgef3_[Bos_mutus]_555987327
------------------------------------------------------------
---------------------MVGGVGQNSKE-----GPDLWYAAAAAAGWRPLSAPDRQ
APVKVFLS--LPRLLA--GAQHLRLTSPEDSKEVLLLGAAEPCFGP--------AAPDPA
SWL-----SAVLFPSQ---------------------------------VGWPGEG----
-----RWQVGLAVEG-DSILGAPPLGG----LPDVVPEGTLLNMVLRRMYRSRSCSYQ-L
LLE------------------------HQRP-S---------CIQ---------------
------------------GLR-------------------WT------------------
-----PLTSSEESLDFSV-SLEQA-------------------S--------V-------
-------ERVL-------------RA---------------------GKQLH-RHLLANC
PN-L----------------IRDRKYH------L-RLH----------------RQC-CS
GR-----------------ELVDGILALG------------------------------L
----GVH--------SRSQAVGIC---QVLLDEGALC-----------------------
--------------H------------------------VKH-DWAFQDRDTQFYRFPGP
-------EPEP--VGVHEL-----------------------------------------
------------------------------------------------------------
---------EEEL------VEALALLSQRGPDAL-------------LTVALRKPPGQRT
DEELD-----LIFEELLHIKAVAHLS-NSVKRELA-AVLLFEPHSKAGTVL---------
--------------------------------------------FSQGDKG--TSWYIIW
KGSVNVVTHGK-----GL-------------------------------VTTLHEG-DDF
GQLAL-----------VNDAPRA---ATIILGEDNCHFLRVDKQDFNR------IIKD--
---------------------------------------------V--------------
------------------------------------------------------------
------------------------------------------------------------
------------------------EAKTMRLEEHGKVVLVLER-------T------SQG
TG-----PSRP------------------PTPGRN------------R-YTVMSGTPEKI
LELLLEA------------MRPDSSAHDPT-ETF-------------------LS---D-
FLLTHSV--FM---------------PTAQLCAALL-----HHF------H---------
-----------------------------AEPA---GGSEQERSSYICNKRQQILRLVSQ
WVTLYGPTLRTD-PVATSFLK----KLSDL--VSRDT--RLSNLLR---EQ-WPERRR-H
HRL----ENGCGNA--SP------QMKAR----------------------NVP------
-----VW-----------LPSQD--------EP---------------------------
-----------------L------------------------------------------
---PSSN-----------------------------------------------------
------------------------------------------------------------
------------------------------------------------------------
------------------------------------------------------------
------------------------------------------------------------
----CAIRVGDK--VP-----------------------------YD-------------
ICRPDHSMLTLQLPVTASVREVMA--------ALAQEDG-----WT-------KGQ--VL
VKVNSA----------------------G------DAIGLQPDARGVATSLGLNERLFVV
NPQE---V-HELT-PHPDQL---------------------------GP------TVGSA
EG--LDLVSTKDLAGQLTDHDWSLFNSIHQVELIH-------YVLGPQHL-RDVTTANLE
```

RFMRR---------FNELQYWV-----ATELCLCSVPGLR------AQLLRKFIKLAAHL
---------------KEQKNLNSFFAIMF-GLSNSAISRLAHTWE---------------
-------RLPHKVRKLYSA-LERLL--------------------------DPSWNH---
-RVYRL----ALTK-------LSPPLI-PFMP----------------------------
LLLKDMTFIHEGNH--TL-VE-----------------------------N-LINFE-KM
-------RM---MARAARMLHHCRSHSN------------------------VPLSPLRS
RVSHLHEDSQAVRVSTCSEQSLSTR-------S--------------PASTWA-----YV
QQLKVIDNQREL---SRLSREL
-----------EP---

>Rapgef3_[Bos_taurus]_122692463

---------------------M--------------------------------------

---------------K---------------------------------VGWPGEG----
-----RWQVGLAVEG-DSILGAPPLGG----LPDVVPEGTLLNMVLRRMYRSRSCSYQ-L
LLE------------------------HQRP-S---------CIQ---------------
------------------GLR-------------------WT------------------
-----PLTSSEESLDFSV-SLEQA-------------------S--------A-------
-------ERVL-------------RA---------------------GKQLH-RHLLANC
PN-L----------------IRDRKYH------L-RLH----------------RQC-CS
GR-----------------ELVDGILALG------------------------------L
----GVH--------SRSQAVGIC---QVLLDEGALC-----------------------
--------------H------------------------VKH-DWAFQDRDTQFYRFPGP
-------EPEP--VGVHEL---

---------EEEL------VEALALLSQRGPDAL-------------LTVALRKPPGQRT
DEELD-----LIFEELLHIKAVAHLS-NSVKRELA-AVLLFEPHSKAGTVL---------
--FSQGDKG--TSWYIIW
KGSVNVVTHGK-----GL-------------------------------VTTLHEG-DDF
GQLAL-----------VNDAPRA---ATIILGEDNCHFLRVDKQDFNR------IIKD--
---V--------------

------------------------EAKTMRLEEHGKVVLVLER-------T------SQG
TG-----PSRP------------------PTPGRN------------R-YTVMSGTPEKI
LELLLEA------------MRPDSSAHDPT-ETF-------------------LS---D-
FLLTHSV--FM---------------PTAQLCAALL-----HHF------H---------
-----------------------------AEPA---GGSEQERSSYICNKRQQILRLVSQ
WVTLYGPTLRTD-PVATSFLK----KLSDL--VSRDT--RLSNLLR---EQ-WPERRR-H
HRL----ENGCGNA--SP------QMKAR----------------------NVP------
-----VW-----------LPSQD--------EP---------------------------
-----------------L--
---PSSN---

----CAIRVGDK--VP-----------------------------YD-------------
ICRPDHSMLTLQLPVTASVREVMA--------ALAQEDG-----WT-------KGQ--VL
VKVNSA----------------------G------DAIGLQPDARGVATSLGLNERLFVV
NPQE---V-HELT-PHPDQL---------------------------GP------TVGSA
EG--LDLVSTKDLAGQLTDHDWSLFNSIHQVELIH-------YVLGPQHL-RDVTTANLE
RFMRR---------FNQLQYWV-----ATELCLCSVPGLR------AQLLRKFIKLAAHL
---------------KEQKNLNSFFAIMF-GLSNSAISRLAHTWE---------------
-------RLPHKVRKLYSA-LERLL--------------------------DPSWNH---
-RVYRL----ALTK-------LSPPLI-PFMP----------------------------
LLLKDMTFIHEGNH--TL-VE-----------------------------N-LINFE-KM
-------RM---MARAARMLHHCRSHSN------------------------VPLSPLRS
RVSHLHEDSQAVRVSTCSEQSLSTR-------S--------------PASTWA-----YV
QQLKVIDNQREL---SRLSREL
-----------EP---

>Rapgef3_[Brugia_malayi]_1009341629

--------------------------------------MDRLVARIQRQSRSKQFSDALL
FTVLGNSDCKPKQIA--NGVLLFQSGDQIFYWYLLLSGEVQLFLSTADNE----------
------RIIETLQPIALFGE--LSIS-RHSCSAR-IIRPAEIVTINQSHFVAIYN-----
KHAD--NLQPHIVVMQDLIS--
----------------------DD--LDYSVAALVPQ-------DHKQRRNIRLE-----
GDVM------TDFNQPDGKMAYNFYEMLQRNNEIA--------------NDVRY------
----EPNTITADIDSEVE--
-CDWNSPSSYFELNNSASLESRIYEA---------------------GHILK-QTMELRD
PH-L----------------IRDRRGH--------LQ---------SFL------SC-MI
GT-----------------EMVDWLCSLT-------------------------------
---TKTLDSTKNISLTRFQVVGMW---QALLENGVIA-----------------------
--------------H------------------------VSN-ELQFADKQ-FFYRWNIG
TTANFNMRLIS----GV---
---MFD
GE-QETVPRISDI------AAAISFLNTIGPDSL-------------FRMILCKPSSERT
MQELE-----HVYRELLHVKALTHLS-TMVKRELA-AVVFFEQHQHAGHVL---------
--FRQGDAG--NCWYIVL
KGSVDVIIHGK-----GV-------------------------------VCTLREG-DDF
GKLAL-----------VNDAPRA---ATVALRKDKSQFLRVDKSDFNR------ILRD--
---V--------------

------------------------EANTVRLKEHGQDVLVLEK-----MNT---------

---------EYRTFNDTYHGSVAVAINATAIDATRNLSTDIRPRCC---YSVMAGMPEKT
VEYLLET-------------RIDA---------------QTDD----GLVDTFLE---D-
FILTHIT--HM---------------PVNILCSYLK-----N------------------
--------YYMR-G--------STVGV-----DG---TLPIDDYEHKIVAKRRVVAFLNL
WVQILGLRFFLD-PASNSFVE----ELYCY--VLEDS--------RQF-----PEMLSN-

--------------LEPMCTIRQ-LRENAMQTI---------------------------
----NR--------HSSV--------------------------------VLDCGIYCSH
A--PAPNP--

------VLPFDT--CN--QV
IYLSDTSSFVMSIRLDKTAADICE--------MAKTK-----------IRYSGSSEELHL
VEVKSN----------------------GERIV------FSPNDISIPTMISLNGLLYVA
HNDE---I-ETLA-PLPQQ---------------------------YSP------SEGIH
TS-LLDYLSSTDIAQQLLIFHTQLLEATDDIELIT-------QVFGREQF-PNRITSNLD
LLMRR---------FNEVQFWT-----TTEVLLAQSNTKR------LSVLKKFIKIAAH-
--------------AKENKDLLSLFAIIL-GLSNIAVSRISHLWD---------------
-------KLPSKLKQQYAE-FEELL--------------------------DPCRNH---
-RAYRM----LTAN-------MSAPTV-PFIP----------------------------
LLLKDLTFTHEGNK--TY-FA-----------------------------G-LINFE-KM
-------HM---IANILRGFRQCK--YP------VIAY-------------------E--
-----------------------RKV------F----------------KSKN-----LV
RNFKVIDNQRRM---MELSYQI
-----------EPSK----------RKHL-------------------------------

>Rapgef3_[Bubalus_bubalis]_594071307

---------------------M--------------------------------------

---------------K---------------------------------VGWPGES----
-----RWQVGLAVEG-DSILGAPPLGG----LPDVVPEGTLLNMVLRRMYRSRSCSYQ-L
LLE------------------------HQRP-S---------CIQ---------------
------------------GLR-------------------WT------------------
-----PLTSSEESLDFSV-SLEQA-------------------S--------T-------
-------ERVL-------------RA---------------------GKQLH-RHLLANC
PN-L----------------IRDRKYH------L-RLH----------------RQC-CS
GR-----------------ELVDGILALG------------------------------L
----GVH--------SRSQAVGIC---QVLLDEGALY-----------------------
--------------H------------------------VKH-DWAFQDRDTQFYRFPGP
-------EPEP--VGVHEL---

---------EEEL------VEALALLSQRGPDAL-------------LTVALRKPPGQRT
DEELD-----LIFEELLHIKAVAHLS-NSVKRELA-AVLLFEPHSKAGTVL---------
--FSQGDKG--TSWYIIW
KGSVNVVTHGK-----GL-------------------------------VTTLHEG-DDF
GQLAL-----------VNDAPRA---ATIILGEDNCHFLRVDKQDFNR------IIKD--
---V--------------

------------------------EAKTMRLEEHGKVVLVLER-------T------SQG
TG-----PSRP------------------PTPGRN------------R-YTVMSGTPEKI
LELLLEA------------MRPDSSAHDPT-ETF-------------------LS---D-
FLLTHSV--FM---------------PTAQLCAALL-----HHF------H---------
-----------------------------AEPA---GGSEQERSSYVCNKRQQILRLVSQ
WVTLYGPTLRTD-PVATSFLK----KLSDL--VSRDA--RLSNLLR---EQ-WPERRR-H
HRL----ENGCGNA--SP------QMKAR----------------------NVP------
-----VW-----------LPSQD--------EP---------------------------
-----------------L--
---PSSN---

----CAIRVGDK--VP-----------------------------YD-------------
ICRPDHSVLTLQLPVTASVREVMA--------ALAQEDG-----WT-------KGQ--VL
VKVNSA----------------------G------DAIGLQPDARGVATSLGLNERLFVV
NPQE---V-QELT-PHPDQL---------------------------GP------TVGSA
EG--LDLVSTKDLAGQLTDHDWSLFNSIHQVELIH-------YVLGPQHL-RDVTTANLE
RFMRR---------FNELQYWV-----ATELCLCSVPGLR------AQLLRKFIKLAAHL
---------------KEQKNLNSFFAVMF-GLSNSAISRLAHTWE---------------
-------RLPHKVRKLYSA-LERLL--------------------------DPSWNH---
-RVYRL----ALTK-------LSPPLI-PFMP----------------------------
LLLKDVTFIHEGNH--TL-VE-----------------------------N-LINFE-KM
-------RM---MARAARMLHHCRSHSN------------------------VPLSPLRS
RVSHLHEDSQAVRVSTCSEQSLSTR-------S--------------PASTWA-----YV
QQLKVIDNQREL---SRLSREL
-----------EP---

>Rapgef3_[Caenorhabditis_briggsae]_309361531

--------------------------------------MERMVSRVRRLSPLHTFSDALL
ISLFSESDFQPDNIQ--QGVVLFEKDEPTEYWYLLLSGEVQLYSKTYVSPSNFYLESSIF
VQVGDFKHLKTLRCGSLFGD--LSTL-THSCSCL-VSRPAQLIKIAQNHFLSVYN-----
KHGD--HLQPFIIIMHDILT--
----------------------DE--TPMDPQ--LPM-------HSSGLFNGQRS-----

```
NDLM------SSEINPN--------EIVSVSTEGIPTKMILPSLPHRREN-RSH------
----RPNQLNHNQNHRTE------------------------------------------
--DRN----FIEFHNPTGIEKQVLES---------------------GEVLH-RKMLTDN
HQ-V----------------IRDITTQ--------HTVIFFRSSQSEYLNFQRVQNC-MI
GA-----------------EMIDWLLTLF-------------------------------
---VST--STTSSSLSRIQMSAIW---QVLLNNGMIA-----------------------
--------------H------------------------IDG-EHEFMDKTNSYYRWVQP
Y------RNRN----KV-------------------------------------------
------------------------------------------------------------
------APTIEEV------SKSTALLSSIAPETL-------------FLMIVSKPGFERS
PEELE-----VVYEELTFIKALSHLS-TMVKRQLA-NFVKVEQYIHAGSVV---------
--------------------------------------------FRQREIG--VYWYIVL
KGAVEVNVNGK------V-------------------------------VCILREG-DDF
GKLAL-----------VNDLPRA---ATIVTYEDDSIFLVVDKHHFNQ------ILHQ--
---------------------------------------------V--------------
------------------------------------------------------------
------------------------------------------------------------
------------------------EANTVRLKDYGEDVLVLEK-----VDI---------
---------P----------------RGAALEN----SNSCNFNCG---YSVMAGKAEKI
LEYVLET-------------RIDA---------------LSDDI---SEIDLFVE---D-
FILTHDA--FM---------------PANTVCNFLK-----S------------------
--------YYFR-T--------PYRAT-----RD---SIT-DSCTEEVRCKKRVVQFVYI
WCSMLKINFFLN-PVTNSFVE----ELFCH--VIDDR--------KRL-----GGMEEI-
------------------------------------------------------------
--------------MTRIGAIRT-SRENMQLVL---------------------------
----AR--------HPAI--------------------------------VLDCGVLSAR
T--PCP------------------------------------------------------
------------------------------------------------------------
------------------------------------------------------------
------------------------------------------------------------
------------------------------------------------------------
------ILPSDV--CN------------------------------------------QI
IYLADTTCFVLPIRVDKTAEEICE--------LSRRR-----------MSFSA--EPLKL
VEVKSN----------------------GEKLI------FSPTDRAIPTVLSLNSKLYVV
NREE---I-PMLV-PMEDQ---------------------------NGP------TPTTH
SS-ILHLIDSQELAHQLFLFHLQLLRATDSNELLY-------QVIGRESF-PQSMPFNLD
LLVRR---------FNEVQHWS-----TTEILLA-TEDNR------VEILKKFIHIATML
VKNETEWFENIFYRAREYRDLLTVFAITL-GLSHTSVSRLTLTWN---------------
-------KLPPATFKTFSE-LENLL--------------------------DPTRNH---
-RMYRL----LVSK-------IATPYI-PFVP----------------------------
LILKDLMFIHQGNK--SF-YN-----------------------------G-LVNFE-KM
-------HM---FAKIFRNFRQCKS--H------MNDT-------------------T--
----------------------DHQY------V----------------EPQS-----LI
RNLRVIDNQKIL-----------------------------------------IQLSYGI
-----------EPKT----------AKRTVTKEIENNGKRAIDSICC-------------
------------------------------------------------------------
------------------------------------------------------------
------------------------------------------------------------
------------------------------------------------------------
------------------------------------------------------------
------------------------------------------------------------
------------------------------------------------------------
------------------------------------------------------------
------------------------------------------------------------
```

```
------------------------------------------------------------
---
>Rapgef3_[Caenorhabditis_remanei]_308501823
------------------------------------------------------------
---------------------------------MCHIQMERMVSRVRRLSPLHTFSDALL
ISLLSESDFQPDNIQ--QGVVLFEKDEPTEYWYLLLSGEVQLYSKNYV------------
---GDFKHLKTLRCGSLFGD--LSTT-THSCSCL-VSRPAQLIRISQNHFLSVYNFLEFQ
KHGD--HLQQFIIIMHDILT----------------------------------------
----------------------DE--TPMDP---IPT-------YSSGLFNGQRS-----
NDLM------TSEINPN--------EIVSVSTEGIPTKMILPSIPYRRDKVKNN------
----RPVT-----QKTVE------------------------------------------
--ERN----FIEFHNPTRIEKEIIES---------------------GGTLH-RKMLTDN
HQ-V----------------IRDITTQ--------HT---------------RVQNC-MI
GA-----------------EMIDWLLTLF-------------------------------
---VST--STTSSSLSRIQMSAIW---QVLLNYGIIA-----------------------
--------------H------------------------IDG-EHQFMDKTNSYYRWVQP
Y------RNRN----KV-------------------------------------------
------------------------------------------------------------
------APTMEEV------LKSISLLSSVAPETL-------------FLMIVSKPGFERS
PEELE-----VVYEELTFIKALSHLS-TMVKRQLA-NFVKVEQFVHAGSVV---------
--------------------------------------------FRQGEIG--VYWYIVL
KGAVEVNVNGK------V-------------------------------VCVLREG-DDF
GKLAL-----------VNDLPRA---ATIVTYEDDSIFLVVDKHNFNQ------ILHQ--
---------------------------------------------V--------------
------------------------------------------------------------
------------------------------------------------------------
------------------------EANTVRLKDYGEDVLVLEK-----VDI---------
---------P----------------RGAALEN----SSFSSLNCG---YSVMAGKAEKI
LEYVLET-------------RIDA---------------LSDDI---SELDLFVE---D-
FILTHDA--FM---------------PDNTVCNFLK-----T------------------
--------YYFR-T--------PYRAT-----RD---SIS-DSCTEEVRCKRRVVQFVYI
WCRMLKINFFLN-PVTNSFVE----ELFCH--VIDDR--------KRL-----GGMEEI-
------------------------------------------------------------
--------------MTRIGAIRT-SRENMQLVL---------------------------
----AR--------HPAI--------------------------------VLDCGVLSAH
T--PCP------------------------------------------------------
------------------------------------------------------------
------------------------------------------------------------
------------------------------------------------------------
------------------------------------------------------------
------ILPSDV--CN------------------------------------------QI
IYLADTTCFVLPIRVDKTAEEICE--------LSRRR-----------MSFSA--EPLNL
VEVKSN----------------------GEKLI------FSPNDRAIPTVLSLNSKLYVV
NREE---I-PLLV-PMEDQ---------------------------NGP------TPSSH
SS-ILHLIDSQELAHQLFLFHLQLLRATDSNELLY-------QVIGRESF-PLSMPFNLD
LLVRR---------FNEVQHWS-----TTEILLA-SEENR------VEILKKFIAIATI-
--------------AREYRDLLTVFAITL-GLSHTSVSRLTLTWS---------------
-------KLPPITLKTFSE-LEHLL--------------------------DPTRNH---
-RMYRL----MVSK-------MASPYI-PFVP----------------------------
LILKDLMFIHQGNK--SF-YN-----------------------------G-LVNFE-KM
-------HM---FAKIFRNFRQCKS--Q------MNDS-------------------S--
----------------------DHEY------V----------------EPQS-----LI
RNLRVIDNQKKL-----------------------------------------MQISYEI
-----------EPKT----------TRRNI-------GFH--------------------
```


>Rapgef3_[Callithrix_jacchus]_675676220

---------------------M--------------------------------------

---------------K---------------------------------VGWPGES----
-----CWQVGLAVED-SPALGAPQVGA----LPDLVPEGTLLNMVLRRMHRPRSCSYQ-L
LLE------------------------HRRP-S---------HIQ---------------
------------------GLR-------------------WT------------------
-----PLTNSEESLDFSE-SLEQA-------------------S--------T-------
-------ERVL-------------RA---------------------GKQLH-RHLLATC
PN-L----------------IRDRKYH------L-RLY----------------RQC-CS
GR-----------------ELVDGILALG------------------------------L
----GVH--------SRSQAVGIC---QVLLDEGVLC-----------------------
--------------H------------------------VKH-DWAFQDRDAQFYRFPGP
-------EPEP--MEAHEM---

---------EEEL------AEAVALLSQRGPDAL-------------LTVALRKPPGQRT
DEELD-----LIFEELLHVKAVAHLS-NSVKRELA-AVLLFEPHSKAGTVL---------
--FSQGDKG--TSWYIIW
KGSVNVVTHGK-----GL-------------------------------VTTLHEG-DDF
GQLAL-----------VNDAPRA---ATIILREDNCHFLRVDKQDFNR------IIKD--
---V--------------

------------------------EAKTMRLEEHGKVVLVLER-------A------SQG
VG-----SSRP------------------PTPGRN------------R-YTVMSGTPEKI
LELLLEA------------MGPDSSAHDPT-ETF-------------------LS---D-
FLLTHRV--FM---------------PSAHLCAALL-----HHF------H---------
-----------------------------AEPA---GGSEQERSTYVCNKRQQILRLVSQ
WVALYGSMLHTD-PVATSFLQ----KLSDL--VSGDA--RLSNLLR---EQ-WPERRR-H
HRL----ENGCGNA--SP------QMKAR----------------------NLP------
-----IW-----------LPNQD--------EP---------------------------
-----------------L--
---PGSS---

----CAIRVGDK--VP-----------------------------YD-------------
ICRPDHSVLTLQLPVTASVKEVMA--------ALAQEDG-----WT-------KGQ--VL
VKVNSA----------------------G------DAIGLQPDARGVATSLGLNERLFVV
NPQE---M-HELT-PHPEQL---------------------------GP------TVGSA
EG--LDLVSAKDLAGQLTDQDWSLFNSVHQVELIH-------YVLGPQHL-RDVTTANLE

RFMRR---------FNQLQYWV-----ATELCLCVAPGPR------AQLLRKFIKLAAHL
---------------KEQKNLNSFFAVMF-GLSNSAISRLAHTWE---------------
-------RLPHKVRKLYSA-LERLL--------------------------DPSWNH---
-RVYRL----ALAK-------LSPPVI-PFMP----------------------------
LLLKDMTFIHEGNH--TL-VE-----------------------------N-LINFE-KM
-------RM---MARASRMLHHCRSHNP------------------------VPLSPLRS
RVSHLHEDSQVARISTCSEQSLSTR-------S--------------PASTWA-----YV
QQLKVIDNQREL---SRLSREL
-----------EP---

>Rapgef3_[Callorhinchus_milii]_632985555

--------------------MNSFFAVAF-GLSNSAVSRLSKTWE---------------
-------GLTNKTRRVYQS-FERLM--------------------------DPSRNH---
-RAYRM----VAGK-------LSLPFL-PFMP----------------------------
LLIKDLTFIEEGNK--TF-VN-----------------------------N-LVNFE-KM
-------RM---IARTVNLFRRCRT--HSQMIPQKMEPEI-----SSAQI---STTSD--
-Q------------------SVSVKNP----------Q-----------PIRL-----YV
RSLKAIDNEQIL---SQLSQQL
-----------EP---

>Rapgef3_[Calypte_anna]_663267862

--------------M------------------------VKQ-EWHFQDKDSQFYRFAEL
E-----LSPEP-HVGPRDS---

----------EEL------LEALSFLAQLGPDAL-------------LTMALRKPPSQRT
EDELE-----LIFEELLHIKAVAHLS-NSVKRELA-SVLMFESHQRAGTV----------
---LFSQGDKG--TSWFIIW
KGSVNVVTHGK-----GL-------------------------------VAHLH-X-XXX
XXXXL-----------VNDAPRA---ATIILREDNCHFLRVDKQDFNH------ILKD--
---V--------------

------------------------EANTVRLKEHGKVVLVLQKNL-------------QG

```
G--------QA------------------GTGRGTGAL------A-TR-YFVVAGTPEKI
LEHLLEF------------MRLDATLYDPV-D-------------------TLLG---D-
FLLTYTL--FM---------------TTSQLCRALL-----HHF------R---------
-----------------------------AEPLE---GSQQEKVTFSLLKRRKILRLVSQ
WVLLYGRVLQGD-LSTTSLLQ---------------------------------------
------------------------------------------------------------
------------------------------------------------------------
------------------------------------------------------------
------------------------------------------------------------
------------------------------------------------------------
------------------------------------------------------------
------------------------------------------------------------
------------------------------------------------------------
------------------------------------------------------------
------------------------------------------------------------
------------------------------------------------------------
------------------------------------------------------------
------------------------------------------------------------
------------------------------------------------------------
------------------------------------------------------------
----------------PRT-----------------------------------------
------------------------------------------------------------
------------------------------------------------------------
------------------------------------------------------------
SGSWLGVQDE-TLPSS--------------------------------------------
-----------C-----------------------------------------ILRAQDR
---VPYEIY-RPDHSCLVTPLPINASVRDILGSLAPQLPPGREHLLVKDPSWNHRVYRLA
VAKLSPPLIPFVPLLLKGSELSLSLRSSLSSWDFPPLGAIDRQQELLRLSRELEP-----
------------------------------------------------------------
------------------------------------------------------------
------------------------------------------------------------
------------------------------------------------------------
------------------------------------------------------------
------------------------------------------------------------
------------------------------------------------------------
------------------------------------------------------------
------------------------------------------------------------
---
>Rapgef3_[Camelus_bactrianus]_743736343
------------------------------------------------------------
---------------------M--------------------------------------
------------------------------------------------------------
---------------K---------------------------------VGWPGES----
-----RWQVGLAVED-GSALGAPPVGG----LPDVVPEGTLLNMVLRRMHLPRSCSYQ-L
LLE------------------------HQRP-S---------RIQ---------------
------------------GLR-------------------WT------------------
-----PLTSSEESLDFSV-SLEQA-------------------S--------T-------
-------ERVL-------------RA---------------------GKQLH-RHLLANC
PN-L----------------IRDRKYH------L-RLH----------------RQC-CS
GR-----------------ELVDGILALG------------------------------L
----GVH--------SRSQAVGIC---QVLLDEGALC-----------------------
--------------H------------------------VKH-DWAFQDRDTQFYRFPGP
-------EPEP--AGTHEL-----------------------------------------
------------------------------------------------------------
```

---------EEEL------VEALALLSQRGPDAL-------------LTVALRKPPGQRT
DEELD-----LIFEELLHIKAVAHLS-NSVKRELA-AVLLFEPHNKAGTVL---------
--FSQGDKG--TSWYIIW
KGSVNVVTHGK-----GL-------------------------------VTTLHEG-DDF
GQLAL-----------VNDAPRA---ATIILREDNCHFLRVDKQDFNR------IIKD--
---V--------------

------------------------EAKTMRLEEHGKVVLVLER-------N------SQG
AG-----PSRP------------------PTPGRN------------R-YTVMSGTPEKI
LELLLEA------------MRPDSSAHDPT-ETF-------------------LS---D-
FLLTHTV--FM---------------PSTQLCAALL-----HHF------H---------
-----------------------------AEPA---GGNEQECSTYVCNKRQQILRLVSQ
WVALYGPMLHTD-PVATSFLQ----KLSEL--VSRDA--RLSNLLR---EQ-WPERRR-H
HRL----ENGCGNA--SP------QMKAR----------------------NMP------
-----VW-----------LPSQD--------EP---------------------------
-----------------L--
---PSSN---

----CAIRVGDK--VS-----------------------------YD-------------
ICRPDHSVLTLQLPVTASVREVMA--------ALAQEDG-----WT-------KGQ--VL
VKVNSA----------------------G------DAIGLQPDARGVATSLGLNERLFVV
NPQE---V-HKLT-PHPEQL---------------------------GP------TVGSA
EG--LDLVSTKDLAGQLTDHDWNLFNSIHQVELIH-------YVLGPQHL-RDVTTANLE
RFMRR---------FNELQYWV-----ATELCLCPVPGLR------AQLLRKFIKLAAHL
---------------KEQKNLNSFFAIMF-GLSNSAISRLAHTWE---------------
-------RLPHKVRKLYSA-LERLL--------------------------DPSWNH---
-RVYRL----ALTK-------LSPPVI-PFMP----------------------------
LLLKDMTFIHEGNH--TL-VE-----------------------------N-LINFE-KM
-------RM---MARAARMLHHCRSHSN------------------------VPLSPLRS
RVSHLHEDSQATRISTCSEQSLITR-------S--------------PGSTWA-----SI
QQLKVIDNQREL---SRLSREL
-----------EP---

>Rapgef3_[Camelus_dromedarius]_744616231

---------------------M--------------------------------------

---------------K---------------------------------VGWPGES----
-----RWQVGLAVED-GSALGAPPVGG----LPDVVPEGTLLNMVLRRMHLPRSCSYQ-L
LLE------------------------HQRP-S---------RIQ---------------

```
------------------GLR-------------------WT------------------
-----PLTSSEESLDFSV-SLEQA-------------------S--------T-------
-------ERVL-------------RA---------------------GKQLH-RHLLANC
PN-L----------------IRDRKYH------L-RLH----------------RQC-CS
GR-----------------ELVDGILALG------------------------------L
----GVH--------SRSQAVGIC---QVLLDEGALC-----------------------
--------------H------------------------VKH-DWAFQDRDTQFYRFPGP
-------EPEP--AGTHEL-----------------------------------------
------------------------------------------------------------
---------EEEL------VEALALLSQRGPDAL-------------LTVALRKPPGQRT
DEELD-----LIFEELLHIKAVAHLS-NSVKRELA-AVLLFEPHSKAGTVL---------
--------------------------------------------FSQGDKG--TSWYIIW
KGSVNVVTHGK-----GL-------------------------------VTTLHEG-DDF
GQLAL-----------VNDAPRA---ATIILREDNCHFLRVDKQDFNR------IIKD--
---------------------------------------------V--------------
------------------------------------------------------------
------------------------------------------------------------
------------------------EAKTMRLEEHGKVVLVLER-------N------SQG
AG-----PSRP------------------PTPGRN------------R-YTVMSGTPEKI
LELLLEA------------MRPDSSAHDPT-ETF-------------------LS---D-
FLLTHTV--FM---------------PSTQLCAALL-----HHF------H---------
-----------------------------AEPA---GGNEQECSTYVCNKRQQILRLVSQ
WVALYGPMLHTD-PVATSFLQ----KLSEL--VSRDA--RLSNLLR---EQ-WPERRR-H
HRL----ENGCGNA--SP------QMKAR----------------------NMP------
-----VW-----------LPSQD--------EP---------------------------
-----------------L------------------------------------------
---PSSN-----------------------------------------------------
------------------------------------------------------------
------------------------------------------------------------
------------------------------------------------------------
------------------------------------------------------------
----CAIRVGDK--VP-----------------------------YD-------------
ICRPDHSVLTLQLPVTASVREVMA--------ALAQEDG-----WT-------KGQ--VL
VKVNSA----------------------G------DAIGLQPDARGVATSLGLNERLFVV
NPQE---V-HKLT-PHPEQL---------------------------GP------TVGSA
EG--LDLVSTKDLAGQLTDHDWNLFNSIHQVELIH-------YVLGPQHL-RDVTTANLE
RFMRR---------FNELQYWV-----ATELCLCPVPGLR------AQLLRKFIKLAAHL
---------------KEQKNLNSFFAIMF-GLSNSAISRLAHTWE---------------
-------RLPHKVRKLYSA-LERLL--------------------------DPSWNH---
-RVYRL----ALTK-------LSPPVI-PFMP----------------------------
LLLKDMTFIHEGNH--TL-VE-----------------------------N-LINFE-KM
-------RM---MARAARMLHHCRSHSN------------------------VPLSPLRS
RVSHLHEDSQATRISTCSEQSLITR-------S--------------PGSTWA-----SI
QQLKVIDNQREL-----------------------------------------SRLSREL
-----------EP-----------------------------------------------
------------------------------------------------------------
------------------------------------------------------------
------------------------------------------------------------
------------------------------------------------------------
------------------------------------------------------------
------------------------------------------------------------
------------------------------------------------------------
------------------------------------------------------------
------------------------------------------------------------
```

```
------------------------------------------------------------
---
>Rapgef3_[Camelus_ferus]_946669812
------------------------------------------------------------
---------------------M--------------------------------------
------------------------------------------------------------
---------------K---------------------------------VGWPGES----
-----RWQVGLAVED-GSALGAPPVGG----LPDVVPEGTLLNMVLRRMHLPRSCSYQ-L
LLE------------------------HQRP-SXXLEHQRPSRIQ---------------
------------------GLR-------------------WT------------------
-----PLTSSEESLDFSV-SLEQA-------------------S--------T-------
-------ERVL-------------RA---------------------GKQLH-RHLLANC
PN-L----------------IRDRKYH------L-RLH----------------RQC-CS
GR-----------------ELVDGILALG------------------------------L
----GVH--------SRSQAVGIC---QVLLDEGALC-----------------------
--------------H------------------------VKH-DWAFQDRDTQFYRFPGP
-------EPEP--AGTHEL-----------------------------------------
------------------------------------------------------------
---------EEEL------VEALALLSQRGPDAL-------------LTVALRKPPGQRT
DEELD-----LIFEELLHIKAVAHLS-NSVKRELA-AVLLFEPHNKAGTVL---------
--------------------------------------------FSQGDKG--TSWYIIW
KGSVNVVTHGK-----GL-------------------------------VTTLHEG-DDF
GQLAL-----------VNDAPRA---ATIILREDNCHFLRVDKQDFNR------IIKD--
---------------------------------------------V--------------
------------------------------------------------------------
------------------------------------------------------------
------------------------EAKTMRLEEHGKVVLVLER-------N------SQG
AG-----PSRP------------------PTPGRN------------R-YTVMSGTPEKI
LELLLEA------------MRPDSSAHDPT-ETF-------------------LS---D-
FLLTHTV--FM---------------PSTQLCAALL-----HHF------H---------
-----------------------------AEPA---GGNEQECSTYVCNKRQQILRLVSQ
WVALYGPMLHTD-PVATSFLQ----KLSEL--VSRDA--RLSNLLR---EQ-WPERRR-H
HRL----ENGCGNA--SP------QMKAR----------------------NMP------
-----VW-----------LPSQD--------EP---------------------------
-----------------L------------------------------------------
---PSSN-----------------------------------------------------
------------------------------------------------------------
------------------------------------------------------------
------------------------------------------------------------
------------------------------------------------------------
----CAIRVGDK--VS-----------------------------YD-------------
ICRPDHSVLTLQLPVTASVREVMA--------ALAQEDG-----WT-------KGQ--VL
VKVNSA----------------------G------DAIGLQPDARGVATSLGLNERLFVV
NPQE---V-HKLT-PHPEQL---------------------------GP------TVGSA
EG--LDLVSTKDLAGQLTDHDWNLFNSIHQVELIH-------YVLGPQHL-RDVTTANLE
RFMRR---------FNELQYWV-----ATELCLCPVPGLR------AQLLRKFIKLAAHL
---------------KEQKNLNSFFAIMF-GLSNSAISRLAHTWE---------------
-------RLPHKVRKLYSA-LERLL--------------------------DPSWNH---
-RVYRL----ALTK-------LSPPVI-PFMP----------------------------
LLLKDMTFIHEGNH--TL-VE-----------------------------N-LINFE-KM
-------RM---MARAARMLHHCRSHSN------------------------VPLSPLRS
RVSHLHEDSQATRISTCSEQSLITR-------S--------------PGSTWA-----SI
QQLKVIDNQREL-----------------------------------------SRLSREL
-----------EP-----------------------------------------------
```


>Rapgef3_[Canis_lupus_familiaris]_153791885

---MVLKRMHRPRSCSYQ-L
LLE------------------------HQRP-S---------RIQ---------------
------------------GLR-------------------WT------------------
-----PLTDSEESLDFSE-SLEQA-------------------S--------T-------
-------ERVL-------------RA---------------------GKQLH-RHLLATS
PT-L----------------IRDRKYH------L-RLY----------------RQC-CS
GR-----------------ELVDGILALG------------------------------L
----GVH--------SRSQAVGIC---QVLLDEGALC-----------------------
--------------H------------------------VKH-DWTFQDRDTQFYRFPGP
-------EPEP--AGVHEL---

---------EEEL------VEAVALLSQRGPDAL-------------LTVALRKPPGQRT
DEELD-----LIFEELLHIKAVAHLS-NSVKRELA-AVLLFEPHSKAGTVL---------
--FSQGDKG--TSWYIIW
KGSVNVVTHGK-----GL-------------------------------VTTLHEG-DDF
GQLAL-----------VNDAPRA---ATIILREDNCHFLRVDKQDFNR------IIKD--
---V--------------

------------------------EAKTMRLEEHGKVVLVLER-------A------SQG
AG-----PSRP------------------PTPGRN------------R-YTVMSGTPEKI
LELLLEA------------MRPDSSAHDPT-ETF-------------------LS---D-
FLLTYSV--FM---------------PSAQLCAALL-----HHF------H---------
-----------------------------AEPS---GGSEQEHSTYICNKRQQILRLVSQ
WVALYGPMLYTD-PVATGFLQ----KLSDL--VSRDT--RLCILLR---EQ-WPERRR-H
HRL----ENGCGNA--SP------QMKAR----------------------NMP------
-----VW-----------LPGQD--------QP---------------------------
-----------------L--
---LCSN---

----RAIRVGDK--VP-----------------------------YD-------------
ICRPDHSVLTLKLPVTASVREVMA--------ALAQEDG-----WT-------KGQ--VL
VKVNSA----------------------G------DAIGLQPDARGVATSLGLNERLFVV
NPQE---V-HELT-PHPEQL---------------------------GP------TVGSA
EG--LDLVSAKDLAGQLTDHDWNLFNSIHQVELIH-------YVLGPQHL-RDVTTANLE

RFMRR---------FNELQYWV-----ATELCLCPVPGLR------AQLLRKFIKLAAHL
---------------KEQKNLNSFFAVMF-GLSNSAISRLAHTWE---------------
-------RLPHKVRKLYSA-LERLL--------------------------DPSWNH---
-RVYRL----ALTK-------LSPPII-PFMP----------------------------
LLLKDMTFIHEGNH--TL-VE-----------------------------N-LINFE-KM
-------RM---MARAVRMLHHCRSHSN------------------------VPLSPLRS
RVSHLHEDSQAARTSTCSEQSLSTR-------S--------------AASTWA-----YV
QQLKVIDNQREL---SRLSREL
-----------EP---

>Rapgef3_[Capra_hircus]_926691591

---------------------M--------------------------------------

---------------K---------------------------------VGWPGES----
-----RWQVGLAVEG-DSILGAPPLGG----LPDVVPEGTLLNMVLRRMYRSRSCSYQ-L
LLE------------------------HQRP-S---------CIQ---------------
------------------GLR-------------------WT------------------
-----PLTSSEESLDFSV-SLEQA-------------------S--------T-------
-------ERVL-------------RA---------------------GKQLH-RHLLANC
PN-L----------------IRDRKYH------L-RLH----------------RQC-CS
GR-----------------ELVDGILTVS------------------------------L
---------------SPGWVGTQC---QAVLGRGLCP-----------------------
--------------T------------------------VKH-DWAFQDRDTQFYRFPGL
-------EPEP--VGAHEL---

---------EEEL------VEALALLSQRGPDAL-------------LTVALRKPPGQRT
DEELD-----LIFEELLHIKAVAHLS-NSVKRELA-AVLLFEPHSKAGTVL---------
--FSQGDKG--TSWYIIW
KGSVNVVTHGK-----GL-------------------------------VTTLHEG-DDF
GQLAL-----------VNDAPRA---ATIILGEDNCHFLRVDKQDFNR------IIKD--
---V--------------

------------------------EAKTMRLEEHGKVVLVLER-------T------SQG
TG-----PSRP------------------PTPGGN------------R-YTVMSGTPEKI
LELLLEA------------MRPDSSAHDPT-ETF-------------------LS---D-
FLLTHSV--FM---------------PTAQLCAALL-----HHF------H---------
-----------------------------AEPA---GGSEQERSSYVCNKRQQILRLVSQ
WVTLYGPTLRTD-PVATSFLK----KLSDL--VSRDA--RLSNLLR---EQ-WPERRR-H
HRL----ENGCGNA--SP------QMKAR----------------------NVP------
-----VW-----------LPSQD--------EP---------------------------
-----------------L--
---PSSN---

----CAIRVGDK--VP-----------------------------YD-------------
ICRPDHSMLTLQLPVTASVREVMA--------ALAQEDG-----WI-------KGQ--VL
VKVNSA----------------------G------DAIGLQPDARGVATSLGLNERLFVV
NPQE---V-HKLT-PHPEQL---------------------------GP------TVGSA
EG--LDLVSTKDLAGQLTDHDWSLFNSIHQVELIH-------YVLGPQHL-RDVTTANLE
RFMRR---------FNELQYWV-----ATELCLCSVPGLR------AQLLRKFIKLAAHL
---------------KEQKNLNSFFAVMF-GLSNSAISRLAHTWE---------------
-------RLPHKVRKLYSA-LERLL--------------------------DPSWNH---
-RVYRL----ALTK-------LSPPLI-PFMP----------------------------
LLLKDMTFIHEGNH--TL-VE-----------------------------N-LINFE-KM
-------RM---MARAARMLHHCRSHSN------------------------VPLSPLRS
RVSHLHEDSQAVRVSTCSEQSLSTR-------S--------------PASTWA-----YV
QQLKGIDNQRMM---ARAARML
HHCRSHSNVPLSPLRSRVSHLHEDSQAGRVSTCSEQSLSTRSPASTWAYVQ---------

>Rapgef3_[Carlito_syrichta]_640792033

---MVLRRMHRPRSCSYQ-L
LLE------------------------HQRP-S---------RIQ---------------
------------------GLR-------------------WT------------------
-----PLTNSEESLDFSV-SLEQA-------------------S--------T-------
-------ERLL-------------RA---------------------GKQLH-RHLLATC
PN-L----------------IRDRKYH------L-RLY----------------RQC-CS
GR-----------------ELVDGILALG------------------------------L
----GVH--------SRSQAVGIC---QVLLDEGALC-----------------------
--------------H------------------------VKH-DWTFQDREAQFYRFPWP
-------ESEP--TGAHEM---

---------EEELL-----AEAVALLSQRGPDAL-------------LTVALRKFPGQRT
DEELD-----LIFEELLHIKAVAHLS-NSVKRELA-AVLVFEPHSKAGTVL---------
--FSQGDKG--TSWYIIW
KGSVNVVTHGK-----GL-------------------------------VTTLHEG-DDF
GQLAL-----------VNDAPRA---ATIILREDNCHFLRVDKQDFNR------IIKD--
---V--------------

------------------------EAKTMRLEEHGKVVLVLER-------A------FQG

AG-----PSRP------------------PTPGRN------------R-YTVMSGTPEKI
LELLLEA------------MRPDSSAHDPT-ETF-------------------LS---D-
FLLTHSV--FM---------------PSAQLCTALL-----HHF------H---------
-----------------------------AEPA---GSCEQERSTYICNKRQQILRLVSQ
WVALYGSMLHTD-PVATTFLQ----KLSDL--VSRDT--RLSNLLR---EQ-WPEKRR-H
HRL----ENGYGNA--SP------QMKAR----------------------NMP------
-----IW-----------LPGQD--------EP---------------------------
-----------------L--
---PSGN---

----SAIRVGDK--VP-----------------------------YD-------------
ICRPDHSVLTLQLPVTASVREVMA--------ALAQENG-----WT-------KGQ--VL
VKVNSA----------------------G------DAIGLQPDARGVATSLGLNERLFVV
SPQE---V-HELT-PHPEQL---------------------------GP------TVGSA
EG--LDLVSAKDLAGQLTDHDWSLFNSIHQVELIH-------YVLGPQHL-RDVTTANLE
RFMRR---------FNELQYWV-----ATELCLCPVPGPR------VQLLRKFIKLAAHL
---------------KEQKNLNSFFAIMF-GLSNSAISRLAHTWE---------------
-------RLPHKVRKLYSA-LERLL--------------------------DPSWNH---
-RVYRL----ALAK-------LSPPVI-PFMP----------------------------
LLLKDMTFIHEGNH--TL-VE-----------------------------N-LINFE-KM
-------RM---MARAVRMLHHCRGHST------------------------VPLSPLRS
RVSHLHEDSQAARISTCSEQSLSTR-------S--------------PASTWA-----HV
QQLKVIDNQREL---SRLSREL
-----------EP---

>Rapgef3_[Cavia_porcellus]_348580717

---------------------M--------------------------------------

---------------K---------------------------------VGWPGES----
-----HWQVGPAVEE-SPILGVPQIGS----LPDVVPEGTLLNMVLRRVHRSRSCSYQ-L
LLE------------------------HRRP-S---------RIQ---------------
------------------GLR-------------------WT------------------
-----PLTNSEESLDFSE-SIEQA-------------------S--------T-------
-------ERVL-------------RA---------------------GRYLY-RHLLATC
PT-L----------------IRDRKYH------L-RLH----------------RQC-CS
GR-----------------ELVDGILALG------------------------------L
----GVH--------SRSQAVGIC---QVLLDEGALC-----------------------
--------------H------------------------VKH-DWAFQDRDAQFYRFLGP
-------EPEP--AGAHEL---

---------EEEL------AEAVALLSQRGPDAL-------------LTVALRKPPGQRM
DEELD-----LIFEELLHIKAVAHLS-NSVKRELA-AVLLFEPHNKAGTVL---------
--FSQGDKG--TSWYIIW
KGSVNVVTHGK-----GL-------------------------------VTTLHEG-DDF
GQLAL-----------VNDAPRA---ATIILREDNCHFLRVDKQDFNR------IIKD--
---V--------------

------------------------EAKTMRLEEHGKVVLVLER-------T------SQG
AG-----PLRP------------------PTPGGN------------R-YTVMSGTPEKI
LELLLEA------------MRPDSSAHDPT-ETF-------------------LS---D-
FLLTHSV--FM---------------PNAQLCAALL-----HHF------H---------
-----------------------------AEPAESAGGSEPERSTYICNRRQQILRLVSQ
WVALYGPMLHQD-PVATSFLQ----KLSDL--VSRDA--RLSNLLR---EQ-WPERRR-H
HRL----ENGCGSA--SP------QLKAR----------------------NIP------
-----VW-----------LPSQD--------EP---------------------------
-----------------L--
---PSSN---

----CAIRVGDK--VP-----------------------------YD-------------
ICRPDHSVLTLQLPVTASVREVMA--------ALAQEDG-----WT-------KGQ--VL
VKVNSA----------------------G------DAIGLQPDARGVATSLGLNERLFVV
NPQE---V-HKLT-PHPKQL---------------------------GP------TVGSA
EG--LDMVSAKDLAGQLTDHDWSLFNSIHQVELIH-------YVLGPQHL-RDVTTANLE
RFMRR---------FNELQYWV-----ATELCLCSVPGLR------AQLLRKFIKLAAHL
---------------KEQKNLNSFFAIMF-GLSNSAISRLAHTWE---------------
-------RLPHKVRKLYSA-LERLL--------------------------DPSWNH---
-RVYRL----ALTK-------LSPPII-PFMP----------------------------
LLLKDMTFIHEGNH--TL-VE-----------------------------N-LINFE-KM
-------RM---MARAARLLHHCRNHST------------------------VPLSPLRS
RVSHLHEDSQASRISTCSEQSLSTR-------S--------------PASTWA-----YV
QQLKVIDNQREL---SRLSREL
-----------EP---

>Rapgef3_[Ceratotherium_simum_simum]_478509423

---------------------M--------------------------------------

---------------K---------------------------------VGWPGES----
-----RWQLGLAVED-SPALGAPPVGG----LPDVVPEGTLLNMVLRRMHRPRSCSYQ-L
LLE------------------------HQRP-S---------RIQ---------------

```
------------------GLR-------------------WT------------------
-----PLTDSEESLDFSV-SLEQA-------------------S--------T-------
-------ERVL-------------RA---------------------GKQLH-RHLLATC
PN-L----------------IRDRKYH------L-RLH----------------RQC-CS
GR-----------------ELVDGILALG------------------------------L
----GFH--------SRSQAVGIC---QVLLDEGALC-----------------------
--------------H------------------------VKH-DWTFQDRDTQFYRFPGP
-------EPEP--AGLHEL-----------------------------------------
------------------------------------------------------------
---------EEEL------VEAMALLSQRGPDAL-------------LTVALRKPPGQRT
DEELD-----LIFEELLHIKAVAHLS-NSVKRELA-AVLLFEPHSKAGTVL---------
--------------------------------------------FSQGDKG--TSWYIIW
KGSVNVVTHGK-----GL-------------------------------VTTLHEG-DDF
GQLAL-----------VNDAPRA---ATIILREDNCHFLRVDKQDFNR------IIKD--
---------------------------------------------V--------------
------------------------------------------------------------
------------------------------------------------------------
------------------------EAKTMRLEEHGKVVLVLER-------T------SQG
AG-----PSRP------------------PTPGRN------------R-YTVMSGTPEKI
LELLLEA------------MRPDSSAHDPT-ETF-------------------LS---D-
FLLTHSV--FM---------------PSTQLCAALL-----HHF------H---------
-----------------------------AEPA---GGSEQECSTYICNKRQQILRLVSQ
WVALYGPMLHND-PVATSFLQ----KLSDL--VSRDA--RLSNLLR---EQ-WPERRR-H
HRL----ENGCGNA--SP------QMKAR----------------------NIP------
-----VW-----------LPSQD--------EP---------------------------
-----------------L------------------------------------------
---PSSN-----------------------------------------------------
------------------------------------------------------------
------------------------------------------------------------
------------------------------------------------------------
------------------------------------------------------------
----CAIRVGDK--VP-----------------------------YD-------------
ICRPDHSVLTLRLPVTASVREVMA--------ALAQEDS-----WT-------KGQ--VL
VKVNSA----------------------G------DAVGLQPDARGVATSLGLNERLFVV
NPQE---V-HELT-PHPEQL---------------------------GP------TMGSA
EG--LDLVSAKDLAGQLTDHDWSLFNSIHQVELIH-------YVLGPQHL-RDVTTANLE
RFMRR---------FNELQYWV-----ATELCLCPVPGPR------AQLLRKFIKLAAHL
---------------KEQKNLNSFFAVMF-GLSNSAISRLAHTWE---------------
-------RLPHKVRKLHSA-LERLL--------------------------DPSWNH---
-RVYRL----ALTK-------LSPPII-PFMP----------------------------
LLLKDMTFIHEGNH--TL-VE-----------------------------N-LINFE-KM
-------RM---MARAVRMLHHCRSHSS------------------------VPLSPLRS
RVSHLHEDGQAARISTCSEQSLSTR-------S--------------PASTWA-----YV
QQLKVIDNQREL-----------------------------------------SRLSREL
-----------EP-----------------------------------------------
------------------------------------------------------------
------------------------------------------------------------
------------------------------------------------------------
------------------------------------------------------------
------------------------------------------------------------
------------------------------------------------------------
------------------------------------------------------------
------------------------------------------------------------
------------------------------------------------------------
```

```
------------------------------------------------------------
---
>Rapgef3_[Cercocebus_atys]_795538398
------------------------------------------------------------
---------------------M--------------------------------------
------------------------------------------------------------
---------------K---------------------------------VGWPGES----
-----CWQVGLAVED-SPALGAPRVGP----LPDVVPEGTLLDMVLRKMHRPRSCSYQ-L
LLE------------------------HQRP-S---------CIQ---------------
------------------GLR-------------------WT------------------
-----PLTNSEESLDFSE-SLEQA-------------------S--------T-------
-------ERVL-------------RA---------------------GRQLH-RQLLATC
PN-L----------------IRDRKYH------L-RLY----------------RQC-CS
GR-----------------ELVDGILALG------------------------------L
----GVH--------SRSQVVGIC---QVLLDEGALC-----------------------
--------------H------------------------VKH-DWAFQDRDAQFYRFPGP
-------EPEPEPVGAHEM-----------------------------------------
------------------------------------------------------------
---------EEEL------AEAVALLSQRGPDAL-------------LTVALRKPPGQRT
DEELD-----LIFEELLHIKAVAHLS-NSVKRELA-AVLLFEPHSKAGTVL---------
--------------------------------------------FSQGDKG--TSWYIIW
KGSVNVVTHGK-----GL-------------------------------VTTLHEG-DDF
GQLAL-----------VNDAPRA---ATIILREDNCHFLRVDKQDFNR------IIKD--
---------------------------------------------V--------------
------------------------------------------------------------
------------------------------------------------------------
------------------------EAKTMRLEEHGKVVLVLER-------A------SQG
AS-----PSRP------------------PTPGRN------------R-YTVMSGTPEKI
LELLLEA------------MGPDSSAHDPT-ETF-------------------LS---D-
FLLTHRV--FM---------------PSAQLCAALL-----HHF------H---------
-----------------------------AEPA---GGSEQERSTYICNKRQQILRLVSQ
WVALYGSMLHTD-PVATSFLQ----KLSDL--VGRDA--RLSNLLR---EQ-WPERRR-H
HRL----ENGCGNA--SP------QMKAR----------------------NLP------
-----VW-----------LPNQD--------EP---------------------------
-----------------L------------------------------------------
---PGSS-----------------------------------------------------
------------------------------------------------------------
------------------------------------------------------------
------------------------------------------------------------
------------------------------------------------------------
----CAIRVGDK--VP-----------------------------YD-------------
ICLPDHSVLTLQLPVTASVREVMA--------ALAQEDG-----WT-------KGQ--VL
VKVNSA----------------------G------DAIGLQPDARGVATSLGLNERLFVV
NPQE---V-HELT-PHPDQL---------------------------GP------TVGSA
EG--LDLVSAKDLAGQLTDHDWSLFNSIHQVELIY-------YALGPQHL-RDVTTANLE
RFMRR---------FNELQYWV-----ATELCLCPVPGPR------AQLLRKFIKLAAHL
---------------KEQKNLNSFFAVMF-GLSNSAISRLAHTWE---------------
-------RLPHKVRKLYSA-LERLL--------------------------DPSWNH---
-RVYRL----ALAK-------LSPPVI-PFMP----------------------------
LLLKDMTFIHEGNH--TL-VE-----------------------------N-LINFE-KM
-------RM---MARAARMLHHCRSHNP------------------------VPLSPLRS
RVSHLHEDSQVARISTCSEQSLSTR-------S--------------PASTWA-----YV
QQLKVIDNQREL-----------------------------------------SRLSREL
-----------EP-----------------------------------------------
```


>Rapgef3_[Charadrius_vociferus]_699656507

---MPGS---------
---G--WAQRQAQGQ--P
NSPEFAEAEIAV--LETCA--------YRFG-----------------------------
---T------------------
-----PLLD-------TESTLDYGH------------------SLTQAS---S-------
-------EKIW-------------RAGKLLF---------------I----H---LTSTR
PG-L----------------IRDHKHH------L-RHH----------------RQC-CS
GK-----------------ELVDWLLSAG------------------------------L
----AVQ--------MRSQAIGIC---QVLVDGGVLT-----------------------
--------------H------------------------VKQ-EWHFQDKDTQFYRFAEL
E-----LSPEP-GAGLRDA---

----------EEL------LEAVAFLAQLGPDAL-------------LTMALRKPPAQRT
EDELE-----LIFEELLHIKAVAHLS-NSVKRELA-AVLMFESHQRAGTVR---------
---EYLFSQGDKG--TSWYIIW
KGSVNVVTHGK-----GL-------------------------------VATLHEG-DDF
GQLAL-----------VNDAPRA---ATIILREDNCHFLRVDKQDFNH------ILKD--
---V--------------

------------------------EANTMRLKEHGKVVLVLQKNL-------------QG
G------SNQP------------------AAARSS-R------------YLVMAGTPEKI
LEHLLEF------------MRLDATLYDP-------------------------------
------------------------------LCRALL-----HHF------R---------
-----------------------------AEPLE---GSEQEKATYSLHKRRKILRLVSQ
WVLLYGRLLQGD-RSTTALLQ----NLAD--LASQDP--RLGGLVQ---EQ-AQDRRR--
PRA---LENGDGSV--SP------QPKARS---------------------SVN------
------W-----------LASQE--------EA---------------------------
----------------IL--
----NSSC-AL-----------------RAQDK---------------------------

--------------VP-----------------------------YE-------------
IYRPDHSCLITVLPVNASVRDILR--------SLAPRLG-------------RDRE-HIL
VKVNSA----------------------GDKVG------LQLDAVGVFTALGLNERLFAV
SVEE---L-GSLT-PHPEQL---------------------------GP------HVGSS
ET--LDLISSKDLASHLTDYDWNLFKSIHQVEMIH-------YIVGPQKF-HDVTTANLE

RVMRR---------FNELQYWV-----ATELCLCPEVGRR------AQLLRKFIKLAAHL
---------------KEQKNLNSFFAVMF-GVSNTAVSRLAKTWE---------------
-------RLPHKIRKLHSA-LERML--------------------------DPSWNH---
-RVYRL----AVAK-------LSPPII-PFVPL-LLK-----------------------
----DMTFIHEGNR--TLA------------------------------EN-LINFE-KM
-------HM---MAKTVRVLQRCRG--HA----------------------HAPLSPLRN
RSPHRPEDPKAIRIST--
-----------C---SEQSLSV
RSPVSTWAY-LQHLKAIDSQKELLRLSHDLES----------------------------

>Rapgef3_[Chelonia_mydas]_591349441

---MW-AVGQSGNA--
-GVA-ALHEEPELQFHIAKCDFNVYSRLAEFTG-------KLPAGDHAQ-----------
FQASVDERKLVVKVALQAMVDAADTASHSIATSDHEIIELVRETAQPDSDKKEGENAKKI
PYMQAEECFATCLQWLEQQPDATAMNLMILQELHTLAAKKRT------------------
-----PLPD-------SESAPDCGQ------------------SLTQAP---T-------
-------EKIW-------------RAGRLLY---------------S----H---LVSSR
PG-L----------------IRDHKYH------L-RHH----------------RHC-CS
GK-----------------ELVDWLLSAG------------------------------L
----SIQ--------TRGQAIGVC---QVLVDGAVLT-----------------------
--------------H------------------------VKQ-EWHFQDKDAQFYRFAEG
E-----LSLEP---GTWDT---

----------EEL------LEALAFLAQLGPDAL-------------LTMALRKPPAQRT
EDELE-----LIFEELLHIKAVAHLS-HSVKRELA-SVLLFESHPKAGTV----------
---LFSQGDKG--TSWYIIW
KGSVNVVTHGK-----GL-------------------------------VTTLHEG-DDF
GQLAL-----------VNDAPRA---ATIILREDNCHFLRVDKRDFNR------ILKD--
---V--------------

------------------------EANTMRLREHGKVVLVLEKNL-------------QG
SG----CSNQS------------------PSAGSS-R------------YSVMAGTPDKI
LEHLLEA------------MRLDATFSDPL-D-------------------TLIG---D-
FLLXXXX--FM---------------PTTQLCRALL-----HHF------H---------
-----------------------------AEPSE---GSEQEKAVYSLSKRQKILRLVSQ
WVLLYGHLLQAD-HSAITLLQ----NLSD--FVSRDP--RLCSLLR---DQ-TQDRRR--
NRT---LENGGGST--SP------QPKVRS---------------------TVN------
------W-----------IASLE--------DA---------------------------
----------------TL--
----NNSC-AI-----------------GAQDK---------------------------

--------------VP-----------------------------YD-------------
VYRADHSCLTVVLPVNASVRDVLQ--------SLAHREG-------------GHGE-RML
VKVNSA----------------------GDKAG------LQMDTISVYTSLGLNERLFVV
NVQE---L-HTLT-PHPEQL---------------------------GP------TVGSS
ET--LDLISSKDLASQLTDYDWNLFSSVHQVELIY-------YTFGQQTF-PSATTANLE
RVMRR---------FNELQYWI-----ATELCLCAEPGKR------AQLLRKFIKLAAHL
---------------KEQKNLNSFFAVMF-GLSNTSVSRLSKTWE---------------
-------RLPHKTRKLHSA-LERML--------------------------DPSWNH---
-RVYRL----AIAK-------LTPPII-PFMPL-LLK-----------------------
----DMTFIHEGNR--TLA------------------------------EN-LINFE-KM
-------RM---MAKAVRIIHHSQS--HT----------------------YGPISPLRS
RPPQLLEDPQALRIST--
-----------C---SEQSLSG
RSPAPTQAY-LQHLKVIDNQKELSRLSRDLES----------------------------

>Rapgef3_[Chinchilla_lanigera]_533187398

---------------------M--------------------------------------

---------------K---------------------------------VGWPGES----
-----RWQLGPAVEE-SLVLGVPAVGG----LPDVLPEGTLLDMVLRRVHRPRSCSYQ-L
LLQ------------------------HPRP-S---------RIQ---------------
------------------GLR-------------------WT------------------
-----PLTNGEESLDFSE-SLEQA-------------------S--------A-------
-------ERVL-------------RA---------------------GRHLH-RHLLAAC
PT-L----------------IRDRKHH------L-RLH----------------RQC-CS
GR-----------------ELVDGILALG------------------------------L
----GVH--------SRSQAVGIC---QVLLDEGALC-----------------------
--------------H------------------------VRH-DWTFQDRDAQFYRFLGP
-------EPEP--AGTHEL---

---------EEEL------VEAMALLSQRGPDAL-------------LTVALRKPPGQRL
DEELD-----LIFEELLHIKAVAHLS-NSVKRELA-AVLLFESHSKAGTVL---------
--FSQGDKG--TSWYIIW
KGSVNVVTHGK-----GL-------------------------------VTTLHEG-DDF
GQLAL-----------VNDAPRA---ATIILREDNCHFLRVDKQDFNR------IIKD--
---V--------------

------------------------EAKTMRLEEHGKVVLVLER-------T------SQG

AG-----PSRP------------------PTPGRN------------R-YTVMSGTPEKI
LELLLEA------------MRPDSSAHDPT-ETF-------------------LS---D-
FLLTHSV--FM---------------PTAQLCAALL-----HHF------H---------
-----------------------------AEPAEAAGGGELEHSSYICNRRQQILRLVGR
WVALYSPMLHKD-PVATSFLQ----KLSDL--VSRDA--RLSHLLR---EQ-WPERRR-H
HRL----ENGCGSA--SP------QLKAR----------------------NVP------
-----VW-----------LPSQD--------EP---------------------------
-----------------L--
---PSSS---

----CAIRVGDK--VP-----------------------------YD-------------
ICRPDHSVLTLQLPVTASVREVMA--------ALAQEDG-----WT-------KGQ--VL
VKVNST----------------------G------DAVGLQPDARGVATSLGLNERLFVV
TPQE---V-HKLT-PHPEQL---------------------------GP------AVGSA
EG--LDAVSARDLAGQLTDLDWSLFNSIHQVELIH-------YVLGPQRL-RDGTTANLE
RFMRR---------FNELQFWV-----ATELCLCPAPGLR------AQLLRKFIKLAAHL
---------------KEQKNLNSFFAIMF-GLSNSAISRLAHTWE---------------
-------RLPHKVRKLYSA-LERLL--------------------------DPSWNH---
-RVYRL----ALSK-------LSPPLI-PFMP----------------------------
LLLKDMTFIHEGNR--TL-VE-----------------------------N-LINFE-KM
-------RM---MARAARLLHRCRSHGA------------------------VPLSPLRS
RVSHLHEDSQASRVSTCSEQSLSTR-------S--------------SASTWA-----YV
QQLKVIDNQREL---SRLSREL
-----------EP---

>Rapgef3_[Chlorocebus_sabaeus]_635064864

---------------------M--------------------------------------
------------KLLA--GLQHLRLTSPEDSKEVVLPGAVE--LWS--------SCSTPV
PWL-----SAFLPVSQ---------------------------------VGWPGES----
-----CWQVGLAVED-SPALGAPRVGP----LPDVVPEGTLLNMVLRKMHRPRSCSYQ-L
LLE------------------------HQRP-S---------CIQ---------------
------------------GLR-------------------WT------------------
-----PLTNSEESLDFSE-SLEQA-------------------S--------T-------
-------ERVL-------------RA---------------------GRQLH-RQLLATC
PN-L----------------IRDRKYH------L-RLY----------------RQC-CS
GR-----------------ELVDGILALG------------------------------L
----GVH--------SRSQAVGIC---QVLLDEGALC-----------------------
--------------H------------------------VKH-DWAFQDRDAQFYRFPGP
-------EPEP--VGAHEI---

---------EEEL------AEAVALLSQRGPDAL-------------LTVALRKPPGQRT
DEELD-----LIFEELLHIKAVAHLS-NSVKRELA-AVLLFEPHSKAGTVL---------
--FSQGDKG--TSWYIIW
KGSVNVVTHGK-----GL-------------------------------VTTLHEG-DDF
GQLAL-----------VNDAPRA---ATIILREDNCHFLRVDKQDFNR------IIKD--
---V--------------

------------------------EAKTMRLEEHGKVVLVLER-------A------SQG
TG-----PSRP------------------PTPGRN------------R-YTVMSGTPEKI
LELLLEA------------MGPDSSAHDPT-ETF-------------------LS---D-
FLLTHGV--FM---------------PSAQLCAALL-----HHF------H---------
-----------------------------AEPA---GGSEQERSTYICNKRQQILRLVSQ
WVALYGSMLHTD-PVATSFLQ----KLSDL--VGRDA--RLSNLLR---EQ-WPERRR-H
HRL----ENGCGNA--SP------QMKAR----------------------NLP------
-----VW-----------LPNQD--------EP---------------------------
-----------------L--
---PGSS---

----CAIRVGDK--VP-----------------------------YD-------------
ICLPDHSVLTLQLPVTASVREVMA--------ALAQEDG-----WT-------KGQ--VL
VKVNSA----------------------G------DAIGLQPDARGVATSLGLNERLFVV
NPQE---V-HELT-PHPDQL---------------------------GP------TVGSA
EG--LDLVSAKDLAGQLTDHDWSLFNSIHQVELIY-------YALGPQHL-RDVTTANLE
RFMRR---------FNELQYWV-----ATELCLCPVPGPR------AQLLRKFIKLAAHL
---------------KEQKNLNSFFAVMF-GLSNSAISRLAHTWE---------------
-------RLPHKVRKLYSA-LERLL--------------------------DPSWNH---
-RVYRL----ALAK-------LSPPII-PFMP----------------------------
LLLKDMTFIHEGNH--TL-VE-----------------------------N-LINFE-KM
-------RM---MARAARMLHHCRSHNP------------------------VPLSPLRS
RVSHLHEDSQVARISTCSEQSLSTR-------S--------------PASTWA-----YV
QQLKVIDNQREL---SRLSREL
-----------EP---

>Rapgef3_[Chrysemys_picta_bellii]_530652169

---MQVAWGRQGRW--
-HVG-PLLED-------------------------------LPTPPGGL-----------
GDPTAEGHLLYL--LLKKM--------HLFRSYSYQI-------------QLEGE-AGSL

PRIQG-------IRW--------------------------T------------------
-----PLPD-------SESAPDCGQ------------------SLTQAH---A-------
-------EKVW-------------RAGRLLY---------------S----H---LVSSC
PG-L----------------IRDHKYH------L-RHH----------------RHC-CS
GK-----------------ELVDWLLSAG------------------------------L
----SIQ--------MRGQAIGVC---QVLVDGGVLT-----------------------
--------------H------------------------VKQ-EWHFQDKDAQFYRFAEG
E-----LTLEP---GTWDT---

----------EEL------LEALVFLAQLGPDAL-------------LTMALRKP-----
--------------------AKGCLS-LQVKRELA-SVLLFESHPKAGTV----------
---LFSQGDKG--TSWYIIW
KGSVNVVTHGK-----GL-------------------------------VTTLHEG-DDF
GQLAL-----------VNDAPRA---ATIILREDNCHFLRVDKRDFNR------ILKD--
---V--------------

------------------------EANTMRLKEHGKVVLVLEKNL-------------QG
SG----SSNQS------------------PSAGSS-R------------YSVMAGTPDKI
LEHLLEA------------MRLDATFSDPL-D-------------------TLVG---D-
FLLTYNV--FM---------------PTTQFCRALL-----HHF------H---------
-----------------------------AEPSE---GSEQEKAVYSLSKRQKIVRLVSQ
WVLLYGSLLQAE-HCAITLLQ----NLSD--FVSRDP--RLCNLLR---DQ-TQDRRR--
NRT---LENGGGSA--SP------QSKVRS---------------------TVN------
------W-----------IASLE--------DA---------------------------
----------------TL--
----NNSC-AI-----------------GAQDK---------------------------

--------------VP-----------------------------YD-------------
VYRADHSCLTVVLPVNASVRDVLQ--------SLTRQEG-------------GHGE-RML
VKVNSA----------------------GDKAG------LQMDTIGVFTSLGLNERLFVV
NVQE---L-HTLT-PHPEQL---------------------------GP------TVGSS
ET--LDLISSKDLASQLTDYDWNLFSSVHQVELIY-------YIFGQQTF-PSATTANLE
RVMRR---------FNELQYWI-----ATELCLCAEPGRR------AQLLRKFIKLAAHL
---------------KEQKNLNSFFAVMF-GLSNTAVSRLAKTWE---------------
-------RLPHKTRKLHSA-LERML--------------------------DPSWNH---
-RVYRL----AIAK-------LTPPII-PFVPL-LLK-----------------------
----DMTFIHEGNR--TLA------------------------------EN-LINFE-KM
-------RM---MAKAVRIIHHCQS--HT----------------------YAPMSPLRS
RAPQLLEDPQALRIST--
-----------C---SEQSLCG
RSPASTRAY-LQHLKVIDNQKELSRLSRDLES----------------------------

>Rapgef3_[Chrysochloris_asiatica]_586469584

---MVLRRMHRPRSCSYQ-L
LLE------------------------HQRP-S---------RIQ---------------
------------------GLR-------------------WT------------------
-----PLTDSEESLDFSM-SLEQA-------------------S--------T-------
-------ERVL-------------RA---------------------GKQLH-RHLLATC
PN-L----------------IRDRKYH------L-RLY----------------RQC-CS
GR-----------------ELVDGIFALG------------------------------L
----GVH--------SRSQAVGIY---QVLLDEGALC-----------------------
--------------H------------------------VKH-DWTFQDRDAQFYRFPGP
-------EPEP--VKAHEM---

---------GEEL------VEAMALLSQRGPDAL-------------LTVALRKPPGQRT
DEELD-----LIFEELLHIKAVAHLS-NSVKRELA-AVLLFEPHSKAGTVL---------
--FSQGDKG--TSWYIIW
KGSVNVVTHGK-----GL-------------------------------VTTLHEG-DDF
GQLAL-----------VNDAPRA---ATIILREDNCHFLRVDKQDFNR------IIKD--
---V--------------

------------------------EAKTMRLEEHGKVVLVLER-------A------SQG
TG-----PSHL------------------PTPGRN------------R-YTVMSGTPEKI
LELLLEA------------MQPDSSAHDPT-ETF-------------------LS---D-
FLLTHSV--FM---------------PSTQLCASLL-----HHF------H---------
-----------------------------AVPA---GGNEQERSTYVCNKRQQILRLVSQ
WVALYGPMLRTD-PVATSFFQ----KLSDL--VSRDA--RLSNLLK---EQ-WPERRR-H
HRL----ENGCGNA--SP------QMKAR----------------------NVP------
-----AW-----------LPTQD--------EP---------------------------
-----------------L--
----SNN---

----CSIRVGDK--VP-----------------------------YD-------------
ICRPDHSVLSLQLPVTASVREVMA--------ALAQEDG-----WT-------KGQ--VL
VKVNSA----------------------G------KAIGLQPEARGVATSLGLNERLFVV
NPQE---V-HELT-PHPEQL---------------------------GP------TMGSA
EG--LDVVSAKDLAGQLTDHDWNLFHSIHQVELIH-------YVLGPQHL-RTVTTANLE
RFLRR---------FNELQYWV-----ATELCLCPVPSLR------AQLLRKFIKLAAHL
---------------KEQKNLNSFFAIMF-GLSNSAISRLAHTWE---------------
-------RLPHKVRKLYSA-LERLL--------------------------DPSWNH---
-RVYRL----ALTK-------LSPPII-PFMP----------------------------
LLLKDMTFIHEGNH--TL-VE-----------------------------N-LINFE-KM
-------RM---MARAVRMLHQCRSHSN------------------------VPLSPLRS
RVSHLHEDSQAVRISTCSEQSLSTR-------S--------------PASTWA-----YV
QQLKVIDNQREL---SRLSREL
-----------EP---

>Rapgef3_[Clupea_harengus]_831315562

--MCPISFPSVTC---
--LC----A---NAFVFL-F
F---

-----PLF------------VSQ---------------------------FLS-------
-------ERIM-------------KA---------------------AKAVS-SALIERN
PV-L----------------FRDRKHH------L-KTY----------------RQC-GS
GK-----------------ELVDWLMKLN------------------------------D
----C-Q--------SRSQAVGMW---QVLVDEGILI-----------------------
--------------H------------------------VKQ-ELNFQDRDTQFYRFIDT
-------EFDLS--
---------------VDEKYSK--------------------------------------
---------EEEL------QESLSLLIQMGPDAL-------------LTMILRKQPNQRS
AEDLE-----VIYEELMHIKAAAHLS-TSVRKELA-AVLAFESHTKGGTVL---------
--FSQGDKG--TSWYIIW
KGSVNVITHGK-----GL-------------------------------VTTLHEG-EDF
GQLAL-----------VNDAPRS---ATIIMREDNCHFLRVDKQDFIR------ILKD--
---V--------------

------------------------EANTMRLEEHGKAVLVLEK-------S------SGQ
EL-----TNQG------------------GAGGSS------------K-YTVMSGTPEKI
LEHLLDT------------IKLDTNASDQI-DP-------------------SVS---D-
FLLTHQI--FM---------------PSGQLCTALL-----NHY------H---------
-----------------------------AEPSE---GSELEKAAYALNTKQKIVKLVLQ
WVSLYGLFLKDN-TPAVDFLE----SLNKE--VIGDS--RLSAMLR---EQ-LKERRK--
-KM---LDNGHQPW--I-------RQH-NQLD----------------------------
------W-----------FLGYD--------IP---------------------------

---VGKC---

----QAIKVNDK--VL-----------------------------HE-------------
IFRPDHTFVCLMLPVNSSVQDIME--------AMANTS---------------RDH--VL
VKMNSS----------------------G------DREELKLESRAVSTSLGLNERLFLC
SANQ---V-DKLT-PLKEQL---------------------------GP------EVNTM
DT--LEQMGSKDIASQLTNYDWELFTAMHETELLY-------YVFGREKF-PHTTTANLE

RFMRR---------FNEVQYWV-----VTELCLCVDLAKR------AALLKKFIKMAIVL
---------------KEQKNLNSFYAVMF-GLTNSAVTRLYKTWE---------------
-------RVPNKTRRIFTT-YERIL--------------------------DPARNH---
-RAYRL----AVVK-------LSPPYI-PFMP----------------------------
LILKDMTFIHEGNK--NY-TN-----------------------------T-LVNFE-KM
-------RM---IARTLKIVTGCRNQSYS-----------------------VPSSPQKG
MTERMFLETPAIRLSTYSEQSLTLR-------S--------------TSNIRH-----YV
QNLKVIDNQRKL---TQMSRDI
-----------ER---

>Rapgef3_[Colobus_angolensis_palliatus]_795136375

---------------------M--------------------------------------

---------------K---------------------------------VGWPGES----
-----CWQVGLAVED-SPALGAPRVGA----LPDVVPEGTLLNMVLRKMHRPRSCSYQ-L
LLE------------------------HQRP-S---------CIQ---------------
------------------GLR-------------------WT------------------
-----PLTNSEESLDFSE-SLEQA-------------------S--------T-------
-------ERVL-------------RA---------------------GRQLH-RQLLATC
PN-L----------------IRDRKYH------L-RLY----------------RQC-CS
GR-----------------ELVDGILALG------------------------------L
----GVH--------SRSQVVGIC---QVLLDEGALC-----------------------
--------------H------------------------VKH-DWAFQDRDAQFYRFPGP
-------EPEP--VGAHEM---

---------EEEL------AEAVALLSQRGPDAL-------------LTVALRKPPGQRT
DEELD-----LIFEELLHIKAVAHLS-NSVKRELA-AVLLFEPHSKAGTVL---------
--FSQGDKG--TSWYIIW
KGSVNVVTHGK-----GL-------------------------------VTTLHEG-DDF
GQLAL-----------VNDAPRA---ATIILREDNCHFLRVDKQDFNR------IIKD--
---V--------------

------------------------EAKTMRLEEHGKVVLVLER-------A------SQG
AG-----PSRP------------------PTLGRN------------R-YTVMSGTPEKI
LELLLEA------------MGPDSSAHDPT-ETF-------------------LS---D-
FLLTHRV--FM---------------PSAQLCAALL-----HHF------H---------
-----------------------------AEPA---GGSEQERSTYICNKRQQILRLVSQ
WVALYGSMLHTD-PVATSFLQ----KLSDL--VGRDA--RLSNLLR---EQ-WPERRR-H
HRL----ENGCGNA--SP------QMKAR----------------------NLP------
-----VW-----------LPNQD--------EP---------------------------
-----------------L--
---PGSS---

----CAIRVGDK--VP-----------------------------YD-------------
ICLPDHSVLTLQLPVTASVREVMA--------ALAQEDG-----WT-------KGQ--VL
VKVNSA----------------------G------DAIGLQPDARGVATSLGLNERLFVV
NPQE---V-HELT-PHPDQL---------------------------GP------TVGSA
EG--LDLVSAKDLAGQLTDHDWSLFNSIHQVELIY-------YALGPQHL-RDVTTANLE
RFMRR---------FNELQYWV-----ATELCLCPVPGPR------AQLLRKFIKLAAHL
---------------KEQKNLNSFFAVMF-GLSNSAISRLAHTWE---------------
-------RLPHKVRKLYSA-LERLL--------------------------DPSWNH---
-RVYRL----ALAK-------LSPPVI-PFMP----------------------------
LLLKDMTFIHEGNH--TL-VE-----------------------------N-LINFE-KM
-------RM---MARAARMLHHCRSHNP------------------------VPLSPLRS
RVSHLHEDSQVARISTCSEQSLSTR-------T--------------PASTCA-----YV
QQLKVIDNQREL---SRLSREL
-----------EP---

>Rapgef3_[Columba_livia]_543750387

-----------------------------VKRELA-SVLMFESHPRAGTVL---------
--FSQGDKG--TSWYIIW
KGSVNVVTHGK-----GL-------------------------------VATLHEG-DDF
GQLAL-----------VNDAPRA---ATIILREDNCHFLRVDKQDFNR------ILK---

>Rapgef3_[Condylura_cristata]_830194828

---------------------M--------------------------------------

---------------K---------------------------------VGWPGES----
-----RWEVGLAVED-GPALGAPAVGG----LPDLVPEGTLFNMVLRRMHRPRSCSYQ-L
LLE------------------------HQRP-S---------RIQ---------------
------------------GLR-------------------WT------------------
-----PLTSSEESLDFSG-SLEQT-------------------S--------T-------
-------ERLL-------------RT---------------------GRQLY-RHLLAAC
PN-L----------------IRDRKYH------L-RLY----------------RQC-CS
GR-----------------ELVDGILALG------------------------------L
----GVH--------SRSQAVGIC---QVLLDEGALY-----------------------
--------------H------------------------VKH-NWTFQDRDTQFYRFPGP
-------QPEP--VGVHEM---

---------EEEL------VEAVALLSQRGPDAL-------------LTVALRKLPGQRT
DEELD-----LIFEELLHIKAVAHLS-NSVKRELA-AVLLFEPHSKAGTVL---------
--FSQGDKG--TSWYIIW
KGSVNVVTHGK-----GL-------------------------------VTTLHEG-DDF
GQLAL-----------VNDAPRA---ATIILREDNCHFLRVDKQDFNR------IIKD--
---V--------------

------------------------EAKTMRLEEHGKVVLVMER-------T------SQS
AG-----PSRP------------------SPPGRN------------R-YTVMSGTPEKI
LELLLEA------------MRPDSSAHDPT-ETF-------------------LS---D-
FLLTYSV--FM---------------SSTQLCTALL-----HHF------H---------
-----------------------------AEPT---GSSEQERNTYICNKRQQILRLVSQ
WVALYGPTLHTD-PVATSFLQ----KLSDL--VSRDG--RLSHLLR---EQ-WPERRR-H
HRL----ENGCGNA--SP------QMKAR----------------------NVP------
-----SW-----------LPSQD--------EP---------------------------
-----------------L--
---PSSA---

----CAIRVGDK--VP-----------------------------YN-------------
ICRSDHSVLTLQLPVTATVREVMA--------ALAQEDG-----WT-------KGQ--VL
VKVNSA----------------------G------DAVGLQPEARGVATSLGLNERLFVV
NAQE---V-QELT-PHPEQL---------------------------GP------TVGSA
EG--LDLVSAKDLAVQLTEHDWSLFNSIHQVELIH-------YVLGPQHL-RDVTTANLE
RFLRR---------FNELQYWV-----ATELCLCPEPGPR------AQLLRKFIKLAAHL
---------------KEQRNLNSFFAIMF-GLSNSAISRLGHTWE---------------
-------RLPHKVRKLYSV-LERLL--------------------------DPSWNH---
-RVYRM----ALTK-------LSPPII-PFMP----------------------------
LLLKDMTFIHEGNH--TL-VE-----------------------------N-LINFE-KM
-------RM---MARAARTLHHCRSHSA------------------------VPLSPLRS
RVSHLHEDSQAARTSTCEWGGVARPPC-CQLFT--------------PRGRWS-----VI
RGSAGADRPKGL---S--EGHM
FSD---

>Rapgef3_[Coturnix_japonica]_1003995665

---MKVRGAGTATATG
TGTG-IGSGGETERRGAAPVGT-----HCQRTGLERCTGAVAPSTP----PL--S----P
GGSGGDGKKLSV--LLKKM--------HLFRSSSYEI-------------RLEGE-GGSL

```
PRIQG-------IRW--------------------------T------------------
-----PRLD-------AESSLDYGH------------------SLTQAS---S-------
-------DKIR-------------RAGKLLF---------------T----Y---LTNTS
PS-L----------------IRDHKHH------L-RHH----------------RRC-CS
GK-----------------ELVDWMLNAE------------------------------L
----GIQ--------TRSQAIGVG---QVLVDGGVLT-----------------------
--------------H------------------------VKQ-EWHFQDKDTQFYRFAEL
E-----LSPEP-RMGLRDA-----------------------------------------
------------------------------------------------------------
----------EEL------LEAVTFLVQLGPDAL-------------LTMALRKAPAQRT
EDELE-----LIFEELLHIKAVAHLS-NSVKRELA-SVLMFESHQRAGTV----------
-------------------------------------------LFSQGDKG--TSWYIVW
KGSVNVVTHGK-----GL-------------------------------VATLHEG-DDF
GXLAL-----------VNDAPRA---ASIILREDNCHFLRVDKQDFNR------ILKD--
---------------------------------------------V--------------
------------------------------------------------------------
------------------------------------------------------------
------------------------EANTMRLKEHGKVVLVLQKNL-------------QG
G------SSQP------------------ASMRSS-R------------YLVMAGTPEKI
LEHLLEF------------MRLDATLYDPV-D-------------------TLLG---D-
FLLTYTV--FM---------------PTSQLCRALL-----HHF------R---------
-----------------------------AEPLD---GSEQEKASYCLHKRRKILRLVSQ
WVLLYGRLLQGD-RSTTALLQ----NLAD--LVSQDP--RLGAMVQ---E---QERRR--
PRA---MENGDSSI--SP------QPKSRS---------------------SMS------
------W-----------LSSQD--------EA---------------------------
----------------AL------------------------------------------
----NSSC-AL-----------------RAQDK---------------------------
------------------------------------------------------------
------------------------------------------------------------
------------------------------------------------------------
------------------------------------------------------------
--------------VP-----------------------------YE-------------
IYRADHSCLVTVLPVNASVRDVLQ--------SLTPQLG-------------WDGE-HLL
VKVNSS----------------------GDKVG------LQLDAVGVFTSLGLNERLFAV
SMEE---L-GGLT-PHPEQL---------------------------GP------HIGSS
DT--LDLISSKDLASHLTDYDWNLFKSIHQVEMIH-------YIVGPQKF-HEVTTANLE
RMMRR---------FNELQYWV-----ATELCLCNELGRR------AQLLRKFIKLAAHL
---------------KEQKNLNSFFAVMF-GVSNTAVSRLAKTWE---------------
-------RLPHKIRKLHSA-LERML--------------------------DPSWNH---
-RVYRL----AVAK-------LSPPII-PFIPL-LLK-----------------------
----DMTFIHEGNR--TLA------------------------------EN-LINFE-KM
-------HM---MAKTVRILQRCRG--QA----------------------HAPLSPLRT
RSPHRPEDARAVRIST--------------------------------------------
-----------C-----------------------------------------SEQSLSV
RSPVSTWAY-LQHLKAIDSQKELLRLSRDLES----------------------------
------------------------------------------------------------
------------------------------------------------------------
------------------------------------------------------------
------------------------------------------------------------
------------------------------------------------------------
------------------------------------------------------------
------------------------------------------------------------
------------------------------------------------------------
------------------------------------------------------------
```


>Rapgef3_[Cricetulus_griseus]_625224026

---------------------M--------------------------------------

---------------K---------------------------------VGWPGDN----
-----HWQVGPAVVE-SPAVGAPQVES----LPDVVPEGTLLNMVLKRMHRPRCCSYQ-L
VFE------------------------HRRP-S---------CIQ---------------
------------------GLR-------------------WT------------------
-----PLTNSEESLDFTV-SLEQA-------------------T--------T-------
-------ERVL-------------KA---------------------GKLLH-RHLLATY
PT-L----------------IRDRKYH------L-RLY----------------RQC-CS
GR-----------------ELVDGILALG------------------------------L
----GVH--------SRSQAVGIC---QVLLDEGALC-----------------------
--------------H------------------------VKH-DWTFQDRDAQFYRFPGP
-------EPEP--AGTHDV---

---------EEEL------VEAMALLSQRGPDAL-------------LTVALRKPPGQRT
DEELD-----LIFEELLHIKAVAHLS-NSVKRELA-AVLLFEPHSKAGTVL---------
--FSQGDKG--TSWYIIW
KGSVNVVTHGK-----GL-------------------------------VTTLHEG-DDF
GQLAL-----------VNDAPRA---ATIILRENNCHFLRVDKQDFNR------IIKD--
---V--------------

------------------------EAKTMRLEEHGKVVLVLER-------S------SQG
TG-----PSRP------------------PTPGRN------------R-YTVMSGTPEKI
LELLLEA------------MRPDSSAHDPT-ETF-------------------LS---D-
FLLTHSV--FM---------------PSTQLFTALL-----HHF------H---------
-----------------------------VEPAEPAGGSEQERSTYICNKRQQILRLVSR
WVALYGPMLHSD-PVATSFLQ----KLSDL--VSRDA--RLSNLLK---EQ-WPERRR-H
HRL----ENGCGNA--SP------QTKAR----------------------NAP------
-----VW-----------LPSQE--------ES---------------------------
-----------------L--
---PSST---

----GAIRVGDK--VP-----------------------------YD-------------
ICRPDHSVVTLQLPVTASVREVMA--------ALAHEDH-----WT-------KGQ--VL
VKVNSA----------------------G------DVVGLQPDARGVATSLGLNERLFVV
DPQE---V-HELT-PHPEQL---------------------------GP------TLGSS
DM--LELVSAKDLAGQLTDHDWNLFNRIHQVELIH-------YVLGPQHL-RDVTTANLE
RFMRR---------FNELQYWV-----ATELCLCSVPGPR------AQLLRKFIKLAAHL
---------------KEQKNLNSFFAVMF-GLSNSAISRLAHTWE---------------
-------RLPHKVRKLYSA-LERLL--------------------------DPSWNH---
-RVYRL----ALTK-------LSPPVI-PFMP----------------------------
LLLKDMTFIHEGNH--TL-VE-----------------------------N-LINFE-KM
-------RM---MARAVRMLHHCRSHNT------------------------VPLSPLRS
RVSHIHEDSQASRISTCSEQSLSTR-------S--------------PASTWA-----YV
QQLKVIDNQREL---SRLSREL
-----------EP---

>Rapgef3_[Cynoglossus_semilaevis]_657766970

--MHLFRSHCYQ-V
F----PN-----------------RY-LD-T--PTIR-----------------------
------------------GVS-------------------WT------------------
-----PLPEALES---KD-TMKQ---------------------------FLS-------
-------DRIE-------------KA---------------------ARSVY-NVMIERN
PG-L----------------IRDRKYH------L-KTH----------------RQC-SS
GK-----------------ELVDWLMKQN------------------------------E
----CLQ--------SRSQAVGIW---QVLVDEGILI-----------------------
--------------H------------------------VKH-ELNFLDKDTQFYRFKNT
-------EFGLHHITDDR--
DS--
---------EDEL------HEGLSLLSQLGPDAL-------------LTMILRKCPSQRS
AEELE-----VIFEELLHVKAAAHLS-ASVRKELA-AVLVFESHAKAGTVL---------
--FSQGDKG--TSWYIIW
KGSVNVITHGK-----GL-------------------------------VTTLHEG-EDF
GQLAL-----------LNDAPRA---ATIILREDNCHFLRVDKQDFIR------ILKD--
---V--------------

------------------------EANTVRLEEHGKTVLVLEK-------S---------
AT----SVEPG------------------GAGNNSS-----------K-YTVMSGTPEKL
LEHLLDT------------VKLDANGNDAL-EP-------------------CVS---D-
FLLTHMV--FM---------------PSSQLCPALL-----HHY------Q---------
-----------------------------AQLSE---GSDQEKAAYVLNAKQKVVKFISQ
WVVLYGLLLKDD-PTALDFLE----RMKKE--VATDA--RLFNLLX---EQ-FRESRR--
TKVSILMENGYHSL--N-------R-N-QPFD----------------------------
------W-----------FSSVE--------EP---------------------------

---VGRL---

----QPIRTCDK--VL-----------------------------YE-------------
IYRPDNKPFTLMLPVNASVHDVMS--------AIVKPV---------------GDH--VL
VKMNSS----------------------G------ERAQLKLDATAVYTALGLNEKLFIC
TSSQ---V-EQLV-PMVEQQ---------------------------GP------ERGTA
DI--LELMSSKDLAYELTNYDWELFTAVHEVELVY-------YIFGRQKF-HGATTANLE

RFVRR---------FNEVQYWV-----LTEVCLCEDVVKR------ASLLKKFIKIAAVL
---------------KEQKNLNSFFAVMF-ALSNSAVQRLYKTWE---------------
-------RIPSKTKRIYCA-YERLM--------------------------DPSRNH---
-RAYRL----TVAK-------LSPPYI-PLMP----------------------------
LLLKDMTFIHEGNS--NY-VE-----------------------------K-LVNFE-KM
-------RM---MAKTVKIVRGCRSQPY------------------------VPSSPQRG
LADRMFLDGPANRLSTYSDHVFPLR-------S--------------VTSVRQ-----YI
QNLRVIDNQRKL---TQLSRLL
-----------EC---

>Rapgef3_[Cyprinodon_variegatus]_974052683

--MHLFRTHNYQ-V
F----PH-----------------RYSVE-K--PSIR-----------------------
------------------GIS-------------------WT------------------
-----PLPEALDP---KN-TMKQ---------------------------FLS-------
-------DRVV-------------KA---------------------ARSVY-SVMIEKN
PG-L----------------IRDRKHH------L-KTH----------------RHC-CS
GK-----------------ELVDWLMKQS------------------------------E
----CLQ--------SRSQAVGMW---QVLVDEGILV-----------------------
--------------H------------------------VKQ-DLNFLDKDTQFYRFQES
-------ELGLNHVVNEK--
DL--
---------EDEL------HEALSLLSQLGPDAL-------------LTMILRKSPSQRS
AEDIE-----VIYEELLHVKAAAHLS-SSVRKELA-AVLVFESHVKSGTVL---------
--FSQGDKG--TSWYIIW
KGSVNVITHGK-----GI-------------------------------VTTLHEG-EDF
GQLAL-----------LNDAPRA---ATIIILREDNCHFLRVDKQDFIR-----ILKD--
---V--------------

------------------------EANTVRLEEHGKTVLVLEQ-------S---------
AD----SNAQG------------------GAAANS------------K-YTVMSGTPEKI
LEHLLDT------------LKLDTNGNDPI-DP-------------------CVS---D-
FLLTHKV--FM---------------PSSQLCSALQ-----HHY------Q---------
-----------------------------AELTE---GSEQEKTAYIFNTKQKVVKLIVQ
WVALFGLLLKED-PDVLDFLE----RLKTE--VAADH--RLSNILR---EQ-FRERRR--
TKM---LENGYQSL--S-------K-N-QPFD----------------------------
------W-----------FSSCE--------EP---------------------------

---AGRL---

```
------------------------------------------------------------
------------------------------------------------------------
------------------------------------------------------------
------------------------------------------------------------
----QPIKAQDK--VL-----------------------------YE-------------
IFKPDSKPLSLMLPVNSAVHEVMS--------AIGKAD---------------GDH--VL
LKINSS----------------------G------DRAQLKLDATAVYTALGLNERLFIC
TSSE---V-EQLQ-PLKEQQ---------------------------GP------EQGTT
DL--IEQMSSKDIATELTNYDWELFTAMHEVELIY-------YIFGRHKF-PGATTANLE
RFVRH---------FNVVQYWV-----VTELCLCEDLVKR------AILLKKFIKIASVL
---------------KEQRNLNSFFAVMF-GLSNSAVQRLYKTWE---------------
-------RIPSKTKRIYCA-YERLM--------------------------DPSRNH---
-RAYRL----AVAK-------LNPPYI-PFMP----------------------------
LLLKDMTFIHEGNP--NY-VD-----------------------------K-LVNFE-KM
-------RM---LAKTVKIVLECRSQPY------------------------VPSSPQRG
LADRMFLDGPAARLSTYSDHALPLR-------T--------------LSNIRH-----YI
QNLKVIDNQRRL-----------------------------------------TQLSRTI
-----------EC-----------------------------------------------
------------------------------------------------------------
------------------------------------------------------------
------------------------------------------------------------
------------------------------------------------------------
------------------------------------------------------------
------------------------------------------------------------
------------------------------------------------------------
------------------------------------------------------------
------------------------------------------------------------
------------------------------------------------------------
---
>Rapgef3_[Danio_rerio]_688612296
------------------------------------------------------------
------------------------------------------------------------
------------------------------------------------------------
------------------------------------------------------------
------------------------------------------------MHLFRSYNYK-V
F----PD-----------------RCSNEKT--PQIR-----------------------
------------------GIS-------------------WT------------------
-----PLPDAVDT---QD-TIKQ---------------------------FLS-------
-------DRIL-------------KA---------------------ARVVY-STLMEGN
PG-L----------------IRDRKHH------L-KTY----------------RQC-CS
GK-----------------ELVDWLMKLN------------------------------D
----CFQ--------SRSQAVGMW---QVLVDEGILG-----------------------
--------------H------------------------VKQ-ELNFHDKDTQFYRFMEA
-------EF---------------------------------------------------
-D---------LNHTTNEKDSK--------------------------------------
---------DDEL------QESLSLLIQMGPDAL-------------LTMILRKCPSQRT
PEDLE-----VIYEELLHVKAVAHLS-TSVRKELA-SVLVFESHAKAGTVL---------
--------------------------------------------FSQGDKG--TSWYIIW
RGSVNVITHGK-----GL-------------------------------VTTLHEG-DDF
GQLAL-----------VNDAPRA---ATIILREDNCHFLRVDKQDFIR------ILKD--
---------------------------------------------V--------------
------------------------------------------------------------
------------------------------------------------------------
------------------------EANTVRLEEHGKVVLVLEK-------S---------
```

```
SA-----QD---------------------TPSCN------------K-YTVMSGTPEKI
LEHMLEA------------IKLETNGADFI-DP-------------------CVT---D-
FLLTHPV--FM---------------PCSQLCAALQ-----HHY------Q---------
-----------------------------AEPSE---GSDLEKAAYALNTKQKVVKLVCH
WVALFALLLRDD-PVASEFLE----KFREG--VMADS--RLSSMLR---EQ-LRDRRK--
TKI---MENGCHTL--T-------KLN-QKFD----------------------------
------W-----------FSAYE--------EP---------------------------
------------------------------------------------------------
---VGKL-----------------------------------------------------
------------------------------------------------------------
------------------------------------------------------------
------------------------------------------------------------
------------------------------------------------------------
----RSIKAQDK--VL-----------------------------YE-------------
IFKPDHKAVTVMLPVDASVNDILT--------TLVDPD---------------RNY--VL
VKMNSS----------------------G------DRVQLKLETTAVSASLGVNEKLFLC
TSSQ---V-EQLT-PDKEQL---------------------------GP------EKSTM
DT--LEQICSKDMASQHTSYDWELFMAMHEVELVY-------YVFGREKF-PGSTTANLE
RFVRR---------FNEVQYWV-----VTELCLCEDLGKR------AILLKKFIKMAVVL
---------------KEQKNLNSFFAVMF-GLSNSAVQRLNKTWE---------------
-------RLPNKTKRIYCA-YERLM--------------------------DPSRNH---
-RAYRL----TIAK-------LSPPYI-PFMP----------------------------
LLLKDMTFIHEGNK--NY-TD-----------------------------K-LVNFE-KM
-------RM---IARTVKTVRDCRSQPY------------------------VPSSPQKG
LTERMFLDAQAIRISTYSDQSLNLR-------S--------------ATSIRQ-----YI
QNLKVIDNQKKL-----------------------------------------TQLSRAL
-----------ER-----------------------------------------------
------------------------------------------------------------
------------------------------------------------------------
------------------------------------------------------------
------------------------------------------------------------
------------------------------------------------------------
------------------------------------------------------------
------------------------------------------------------------
------------------------------------------------------------
------------------------------------------------------------
------------------------------------------------------------
---
>Rapgef3_[Dasypus_novemcinctus]_488561334
------------------------------------------------------------
------------------------------------------------------------
------------------------------------------------------------
-----------------------------------------------MKVGWPGES----
-----RWQVGLAVDD-SPRVEALPVGG----LPDVVPEGTLLSMVLRRVHRPRSCSYQ-L
LLE------------------------HRRP-S---------CIQ---------------
------------------GLR-------------------WT------------------
-----PLTDSEESLDFSV-SLEQA-------------------S--------T-------
-------ERVL-------------RA---------------------GKQLH-RHLLATC
PN-L----------------IRDRKYH------L-RLY----------------RQC-CS
GR-----------------ELVDGLLALG------------------------------L
----GVH--------SRSQAVGLC---QVLLDDGALC-----------------------
--------------H------------------------VKH-DWTFQDRDAQFYRFPGP
-------EPEP--AGPHEL-----------------------------------------
------------------------------------------------------------
```

---------EEEL------AEAVALLSQRGPDAL-------------LAVALRKPPGQRT
DEELD-----LIFEELLHIKAVAHLS-NSVKRELA-AVLLFEPHSKAGTVL---------
--FSQGDKG--TSWYIIW
KGSVNVVTHGK-----GL-------------------------------VTTLHEG-DDF
GQLAL-----------VNDAPRA---ATIVLREDNCHFLRGDV-----------------

----------P------------------PAVVRP------------G-YTVMSGTPEKI
LELLLEA------------MRPDSSAHDPT-ETF-------------------LS---D-
FLLTHSV--FM---------------PSAQLCAALL-----HHF------H---------
-----------------------------AEPA---GGSEQERSSYICNKRLQILRLVGQ
WVALSGPLLHAD-PVAAGFLQ----KLSDL--VSRDA--RLSHLLR---EQ-GPERRR-L
PRL----ENGCGNA--SP------QMKAR----------------------NMP------
-----TW-----------LPSQD--------EP---------------------------
-----------------L--
---PSS--

----CAIRVGDK--VP-----------------------------YD-------------
ICRPDHSVLTLQLPVTASVREVMA--------ALAQQDG-----WT-------KGQ--VL
VKVNSA----------------------G------DAIRLQPDARGVATSLGLNERLFVV
SPQA---V-HELT-PHPEQL---------------------------GP------TVGSA
EG--LDLVSAKDLAGQLTEHDWTLFNSIQQVELIH-------FVLGPQHL-RDVTTANLE
RFMRR---------FNELQYWV-----ATELCLCPVPGPR------AQLLRKFIKLAAHL
---------------KEQKNLNSFFAVMF-GLSNSAISRLAHTWE---------------
-------RLPHKVRKLYSA-LERLL--------------------------DPSWNH---
-RVYRL----ALAK-------LSPPVI-PFMP----------------------------
LLLKDMTFIHEGNH--TL-VE-----------------------------N-LINFE-KM
-------RM---MARAMRMLHHCRNHSN--------------------------------
----------------GSEQSLSTR-------S--------------PASTWA-----YV
QQLKVIDNQREL---SRLSREL
-----------EP---

>Rapgef3_[Dipodomys_ordii]_852756250

---------------------M--------------------------------------

---------------K---------------------------------VGWPGES----
-----RWQVGLAVED-SPALGALQVGN----LPDVVPEGSLLEMVLKRMHRPRSCSYQ-L
LLQ------------------------HRRP-S---------RIQ---------------

------------------GLR-------------------WT------------------
-----PLTSSEESLDFSV-SLEQA-------------------S--------T-------
-------ERVL-------------RA---------------------GKHLY-RHLLVTF
PS-L----------------IRDRKYH------L-RLY----------------RQC-CS
GR-----------------ELVDGILALG------------------------------L
----GVH--------SRSQAVGIC---QVLLDEGALC-----------------------
--------------H------------------------VKH-DWTFQDREAQFYRFPGP
-------EPEP--AGAHEL---

---------EEEL------VEAMALLSQRGPDAL-------------LTVALRKPPGQRT
DEELD-----LIFEELLHIKAVAHLS-NSVKRELA-AVLLFEPHSKAGTVL---------
--FSQGDKG--TSWYIIW
KGSVNVVTHGK-----GL-------------------------------VTTLHEG-DDF
GQLAL-----------VNDAPRA---ATIILREDNCHFLRVDKQDFNR------IIKD--
---V--------------

------------------------EAKTMRLEEHGKVVLVLER-------A------SQG
TG-----SSRP------------------PTPGKN------------R-YTVMSGTPEKI
LELLLEA------------MRADSNAHDPT-ETF-------------------LS---D-
FLLTHSV--FM---------------PSAQLCAALL-----HHF------H---------
-----------------------------AEPAEPAGGSEQERSTYICNKRQQILRLVSQ
WVALYSPMLHKD-PVVASFLQ----KLSDL--VKMDP--RLSNLLK---EQ-WPERRR-H
HRL----ENGCGNA--SP------QIKAR----------------------SPS------
-----AW-----------LPSQD--------DS---------------------------
-----------------L--
---PSNH---

----GAIRAGDK--VP-----------------------------YE-------------
ICRPDHSVLTLQLPVTASVREVMA--------ALAREDG-----WT-------KGQ--VL
VKVNSA----------------------G------DAIGLKPEARGVATSLGLNERLFVV
NPQD---I-HELT-PHPEQL---------------------------GP------TVGSA
EV--LEVMSAKDLAGQLTDHDWNLFNSIHQVELIH-------YVLGPQHL-RDVTTANLE
RFMRR---------FNELQYWV-----ATELCLCPVPGPR------AQLLRKFIKLAAHL
---------------KEQKNLNSFFAVMF-GLSNSAISRLAHTWE---------------
-------RLPHKVRKLYSA-LERLL--------------------------DPSWNH---
-RVYRL----ALTK-------LSPPVI-PFMP----------------------------
LLLKDMTFIHEGNH--TL-VE-----------------------------N-LINFE-KM
-------RM---MARAVRMLHHCRSHNT------------------------VPLSPLRS
RVSHLHEDSQASRISTCSEQSLSIR-------S--------------PASTWA-----YV
QQLKVIDNQREL---SRLSREL
-----------EP---

>Rapgef3_[Echinops_telfairi]_850308220

---MVLRRMYRPRSCSYQ-L
LLK------------------------HQRP-S---------CIQ---------------
------------------GLR-------------------WT------------------
-----PLADSEESLDFSV-SLEQA-------------------P--------R-------
-------ERVL-------------RA---------------------GKQLH-RHLLATC
PN-L----------------IRDRKYH------L-RLY----------------RQC-CS
GR-----------------ELVDGILALR------------------------------L
----GVH--------SRSQAVGIC---QVLLDEGGLC-----------------------
--------------H------------------------VKH-DWAFQDRDAQFYRFPGP
-------EPQP--VGAQEV---

---------ED-L------LEAVALLAQRGPDAL-------------LTMALRKPPGQRT
DEELE-----LIFEELLHIKAVAHLS-NSVKRELA-AVLLFEPHSKAGTVL---------
--FSQGDKG--TSWYIIW
KGSVNVVTHGK-----GL-------------------------------VTTLHEG-DDF
GQLAL-----------VNDAPRA---ATIILGEDNCHFLRVDKHDFNR------IIKD--
---V--------------

------------------------EAKTMRLEEHGRVVLVLER-------A------SQG
TG-----PDPP------------------AASGRN------------R-YTVMSGTPEKI
LELLLEA------------MRPDSGAHDPT-ETF-------------------LS---D-
FLLTHSV--FM---------------PSAQLCAALL-----HHF------H---------
-----------------------------AEPA---GGSEQERSSYVCNKRQQILRLVSQ
WVALDGPMLHSD-PVVTSFLQ----KLSEL--VSRDA--RLSTQLK---EQ-WPERRR-H
HRL----ENGCGNM--SP------LIKAR----------------------NVP------
-----VW-----------LPIQD--------EP---------------------------
-----------------L--
---SSSN---

----CAIRAGDK--VP-----------------------------YD-------------
ICRPDHSVLTLHLPVTSSVREVMA--------ALAQEDG-----WT-------KGQ--VL
VKVNSA----------------------G------DATGLQPEARGVATSLGLNERLFVV
NPQE---V-HKLD-P---

---SWNH---
-RVYRL----ALTK-------LSPPII-PFMP----------------------------
LLLKDMTFIHEGNH--TL-VE-----------------------------N-LVNFE-KM
-------RM---MARAVRLLHHCRSHGN------------------------VPLSPLRS
RASQLHEDSQAMRVSTCSEQSVNSR-------S--------------PASALA-----YV
QQLKVIDNQREL---SRLSREL
-----------EP---

>Rapgef3_[Elephantulus_edwardii]_585667532

---MVLRRMYRPRSCSYQ-L
LLE------------------------HQRP-S---------RIQ---------------
------------------GLR-------------------WT------------------
-----PLTDSEESLDFSM-SLEQA-------------------S--------A-------
-------ERVL-------------RA---------------------GKQLH-RHLLATC
PN-L----------------IRDRKYH------L-RLY----------------RQC-CS
GR-----------------ELVDGILALG------------------------------L
----GVH--------SRSQAVGIC---QVLLDEGALC-----------------------
--------------H------------------------VKH-DWTFQDRDAQFYRFPGP
-------VPEP--AGPHEM---

---------EDEL------VEAVALLSQRGPDAL-------------LTVALRKSPGQRT
DEELD-----LIFEELLHIKAVAHLS-NSVKRELA-AVLLFEPHSKAGTVL---------
--FSQGDKG--TSWYIIW
KGSVNVVTHGK-----GL-------------------------------VTTLHEG-DDF
GQLAL-----------VNDAPRA---ATIILREDNCHFLRVDKQDFNR------IIKD--
---V--------------

------------------------EAKTMRLEEHGKVVLVLER-------A------SQG
AA-----ASHP------------------ATPGRN------------R-YTVMSGTPEKI
LELLLEA------------MRPDSGAHDPT-EMF-------------------LS---D-
FLLTHSV--FM---------------PSTQLCAALL-----HHF------H---------
-----------------------------AEPA---GGSEQERSTYICNKRQQILRLVSQ
WVALYGPMLRTD-PVATSFLQ----KLSDL--VSRDA--RLSNLLR---EQ-WPERRR-H
HRL----ENGCGNA--SP------QMKAR----------------------NVP------
-----AW-----------LPSQD--------EL---------------------------
-----------------L--
---PSSN---

----CAIRVGDK--VP-----------------------------YD-------------
ICRPDHSVLTLHLPVTASVREVMA--------ALAQEDS-----WT-------KGQ--VL
VKVNSA----------------------G------DAIGLQPDARGVATSLGLNERLFVV
NPQE---M-HKLT-PHPEQL---------------------------GP------TVGSA
EG--LDLVSVKELAGQLTDHDWSLFNSIHQVELIH-------YVLGPQHL-RTVTTANLE

RFMRR---------FNELQYWV-----ATELCLCPVPGLR------AQLLRKFIKLAAHL
---------------KEQKNLNSFFAIMF-GLSNSAISRLAHTWE---------------
-------RLPHKVRKLYSA-LERLL--------------------------DPSWNH---
-RVYRL----ALTK-------LSPPVI-PFMP----------------------------
LLLKDMTFIHEGNH--TL-VE-----------------------------N-LINFE-KM
-------RM---MARAVRMLHRCRSHSN------------------------VPLSPLRS
RVSHLHDDGQAARISTCSEQSLSTR-------S--------------PASTWA-----YV
QQLKVIDNQREL---SRLSREL
-----------EP---

>Rapgef3_[Eptesicus_fuscus]_641700539

---MVLRRMHLPRSCSYQ-L
LLE------------------------HQRP-S---------RIQ---------------
------------------GLR-------------------WT------------------
-----PLTNSEESLDFSV-SLEQT-------------------S--------T-------
-------ERVL-------------RA---------------------GKQLH-RHLLATC
QN-L----------------IRDRKYH------L-RLY----------------RQC-CS
GR-----------------ELVDGILALG------------------------------L
----GVH--------SRSQAVGIC---QVLLDEGALC-----------------------
--------------H------------------------VKH-DWTFQDRDTQFYRFPGP
-------EPEP--AGVHEL---

---------EEEL------VEAMALLSQRGPDAL-------------LTVALRKPPGQRT
DEELE-----LIFEELLHIKAVAHLS-NSVKRELA-AVLLFEPHSKAGTVL---------
--FSQGDKG--TSWYIIW
KGSVNVVTHGK-----GL-------------------------------VTTLHEG-DDF
GQLAL-----------VNDAPRA---ATIVLREDNCHFLRVDKQDFNR------IIKD--
---V--------------

------------------------EAKTMRLEEHGKVVLVLER-------T------SQG
AG-----SSCP------------------PTPGRN------------R-YTVMSGTPEKI
LELLLEA------------MRPDSSAHDPT-ETF-------------------LS---D-
FLLTHRV--FM---------------PSTQLCAALL-----HHF------H---------
-----------------------------AEPA---GGSEQERNIYICNKRQQILRLVSQ
WMALYGPMLHTD-PVASSFLQ----KLSDL--VSRDA--RLSNLLR---EH-WPERRRHH
HRL----ENGGGSA--SP------QIKAR----------------------NMS------
-----VW-----------LPSLD--------EP---------------------------
-----------------L--
---PSSN---

```
------------------------------------------------------------
------------------------------------------------------------
------------------------------------------------------------
------------------------------------------------------------
----CAIRVGDK--VP-----------------------------YD-------------
ICRPDHSVLTLQLPVTASVREVMA--------ALAQEDG-----WT-------KGQ--VL
VKLNSA----------------------G------DAIGLQPDARGVATSLGLNERLFVV
NPQE---V-HELT-PHPEQL---------------------------GP------TVGSA
EG--LDLVSAKDLAVQLTDHDWNLFNSIHQVELIH-------YVLGPQHL-RDVTTANLE
RFMRR---------FNELQYWV-----ATELCLCPVPGPR------AQLLRKFIKLAAHL
---------------KEQKNLNSFFAVMF-GLSNSAISRLAHTWE---------------
-------RLPHKVRKLYSA-LERLL--------------------------DPSWNH---
-RVYRL----ALTK-------LSPPVI-PFMP----------------------------
LLLKDMTFIHEGNH--TL-VE-----------------------------N-LINFE-KM
-------RM---MARAVRMLHHCRSHSN------------------------VPLSPLRS
RVSHLHEDSQAARVSTCSEQSLSTR-------S--------------PASTWA-----YV
QQLKVIDNQREL-----------------------------------------SRLSREL
-----------EP-----------------------------------------------
------------------------------------------------------------
------------------------------------------------------------
------------------------------------------------------------
------------------------------------------------------------
------------------------------------------------------------
------------------------------------------------------------
------------------------------------------------------------
------------------------------------------------------------
------------------------------------------------------------
------------------------------------------------------------
---
>Rapgef3_[Equus_asinus]_958727868
------------------------------------------------------------
------------------------------------------------------------
------------------------------------------------------------
------------------------------------------------------------
-------------------------------------------MVLRRMHRPRSCSYQ-L
LLE------------------------HQRP-S---------RIQ---------------
------------------GLR-------------------WT------------------
-----PLTNSEESLDFSV-SLEQA-------------------S--------T-------
-------ERVL-------------RA---------------------GKQLH-RHLLANC
PN-L----------------IRDRKYH------L-RLH----------------RQC-CS
GR-----------------ELVDGILALG------------------------------L
----GVH--------SRSQAVGIC---QVLLDEGALC-----------------------
--------------H------------------------VKH-DWTFQDRDTQFYRFPGP
-------EPEP--AGVHEL-----------------------------------------
------------------------------------------------------------
---------EEEL------VEAMALLSQRGPDAL-------------LTVALRKPPGQRT
DEELD-----LIFEELLHIKALAHLS-NSVKRELA-AVLLFEPHSKAGTVL---------
--------------------------------------------FSQGDKG--TSWYIIW
KGSVNVVTHGK-----GL-------------------------------VTTLHEG-DDF
GQLAL-----------VNDAPRA---ATIILREDNCHFLRVDKQDFNR------IIKD--
---------------------------------------------V--------------
------------------------------------------------------------
------------------------------------------------------------
------------------------EAKTMRLEEHGKVVLVLER-------T------SQG
```

AG-----PSRP------------------PTPGRN------------R-YTVMSGTPEKI
LELLLEA------------MRPDSSAHDPT-ETF-------------------LS---D-
FLLTHSV--FM---------------PSAQLCAALL-----HHF------H---------
-----------------------------AEPA---GGSEQESSSYICNKRQQILRLVSQ
WVALYGPMLHND-PVATSFLQ----KLSDL--VSRDA--RLSNLLR---EQ-WPERRR-H
HRL----ENGCGNA--SP------QMKAR----------------------NMP------
-----VW-----------LPSQD--------EP---------------------------
-----------------L--
---PSSN---

----CAIQVGDK--VP-----------------------------YD-------------
ICRPDHSVLTLQLPVTASVREVMA--------ALAQEDS-----WT-------KGQ--VL
VKVNSA----------------------G------DAVGLQPDARGVATSLGLNERLFVV
NPQE---V-HELT-PHPEQL---------------------------GP------TVGSA
EG--LDLVSAKDLAGQLTDHDWNLFNSIHQVELIH-------YVLGPQHL-RDVTTANLE
RFMRR---------FNELQYWV-----ATELCLCPVPGPR------AQLLRKFIKLAAHL
---------------KEQKNLNSFFAVMF-GLSNSAISRLAHTWE---------------
-------RLPHKVRKLYSA-LERLL--------------------------DPSWNH---
-RVYRL----ALTK-------LSPPVI-PFMP----------------------------
LLLKDMTFIHEGNH--TL-VE-----------------------------N-LINFE-KM
-------RM---MARAVRMLHHCRSHSS------------------------VPLSPLRS
RVSHFHEDGQAARISTCSEQSLSTR-------S--------------PASSWA-----YV
QQLKVIDNQREL---SRLSREL
-----------EP---

>Rapgef3_[Equus_caballus]_545219817

---MVLRRMHRPRSCSYQ-L
LLE------------------------HQRP-S---------RIQ---------------
------------------GLR-------------------WT------------------
-----PLTNSEESLDFSV-SLEQA-------------------S--------T-------
-------ERVL-------------RA---------------------GKQLH-RHLLANC
PN-L----------------IRDRKYH------L-RLH----------------RQC-CS
GR-----------------ELVDGILALG------------------------------L
----GVH--------SRSQAVGIC---QVLLDEGALC-----------------------
--------------H------------------------VKH-DWTFQDRDTQFYRFPGP
-------EPEP--AGVHEL---

---------EEEL------VEAMALLSQRGPDAV-------------LTVALRKPPGQRT
DEELD-----LIFEELLHIKAVAHLS-NSVKRELA-AVLLFEPHSKAGTVL---------
--FSQGDKG--TSWYIIW
KGSVNVVTHGK-----GL-------------------------------VTTLHEG-DDF
GQLAL-----------VNDAPRA---ATIILREDNCHFLRVDKQDFNR------IIKD--
---V--------------

------------------------EAKTMRLEEHGKVVLVLER-------T------SQG
AG-----PSRP------------------PTPGRN------------R-YTVMSGTPEKI
LELLLEA------------MRPDSSAHDPT-ETF-------------------LS---D-
FLLTHSV--FM---------------SSAQLCAALL-----HHF------H---------
-----------------------------AEPA---GGSEQESSSYICNKRQQILRLVSQ
WVALYGPMLHND-PVATSFLQ----KLSDL--VSRDA--RLSNLLR---EQ-WPERRR-H
HRL----ENGCGNA--SP------QMKAR----------------------NMP------
-----VW-----------LPSQD--------EP---------------------------
-----------------L--
---PSSN---

----CAIQVGDK--VP-----------------------------YD-------------
ICRPDHSVLTLQLPVTASVREVMA--------ALAQEDS-----WT-------KGQ--VL
VKVNSA----------------------G------DAVGLQPDARGVATSLGLNERLFVV
NPQE---V-HELT-PHPEQL---------------------------GP------TVGSA
EG--LDLVSAKDLAGQLTDHDWNLFNSIHQVELIH-------YVLGPQHL-RDVTTANLE
RFMRR---------FNELQYWV-----ATELCLCPVPGPR------AQLLRKFIKLAAHL
---------------KEQKNLNSFFAVMF-GLSNSAISRLAHTWE---------------
-------RLPHKVRKLYSA-LERLL--------------------------DPSWNH---
-RVYRL----ALTK-------LSPPVI-PFMP----------------------------
LLLKDMTFIHEGNH--TL-VE-----------------------------N-LINFE-KM
-------RM---MARAVRMLHHCRSHSS------------------------VPLSPLRS
RVSHFHEDGQAARISTCSEQSLSTR-------S--------------PASSWA-----YV
QQLKVIDNQREL---SRLSREL
-----------EP---

>Rapgef3_[Equus_przewalskii]_664709775

---MVLRRMHRPRSCSYQ-L
LLE------------------------HQRP-S---------RIQ---------------

```
------------------GLR-------------------WT------------------
-----PLTNSEESLDFSV-SLEQA-------------------S--------T-------
-------ERVL-------------RA---------------------GKQLH-RHLLANC
PN-L----------------IRDRKYH------L-RLH----------------RQC-CS
GR-----------------ELVDGILALG------------------------------L
----GVH--------SRSQAVGIC---QVLLDEGALC-----------------------
--------------H------------------------VKH-DWTFQDRDTQFYRFPGP
-------EPEP--AGVHEL-----------------------------------------
------------------------------------------------------------
---------EEEL------VEAMALLSQRGPDAV-------------LTVALRKPPGQRT
DEELD-----LIFEELLHIKAVAHLS-NSVKRELA-AVLLFEPHSKAGTVL---------
--------------------------------------------FSQGDKG--TSWYIIW
KGSVNVVTHGK-----GL-------------------------------VTTLHEG-DDF
GQLAL-----------VNDAPRA---ATIILREDNCHFLRVDKQDFNR------IIKD--
---------------------------------------------V--------------
------------------------------------------------------------
------------------------------------------------------------
------------------------EAKTMRLEEHGKVVLVLER-------T------SQG
AG-----PSRP------------------PTPGRN------------R-YTVMSGTPEKI
LELLLEA------------MRPDSSAHDPT-ETF-------------------LS---D-
FLLTHSV--FM---------------SSAQLCAALL-----HHF------H---------
-----------------------------AEPA---GGSEQESSSYICNKRQQILRLVSQ
WVALYGPMLHND-PVATSFLQ----KLSDL--VSRDA--RLSNLLR---EQ-WPERRR-H
HRL----ENGCGNA--SP------QMKAR----------------------NMP------
-----VW-----------LPSQD--------EP---------------------------
-----------------L------------------------------------------
---PSRN-----------------------------------------------------
------------------------------------------------------------
------------------------------------------------------------
------------------------------------------------------------
------------------------------------------------------------
----CAIQVGDK--VP-----------------------------YD-------------
ICRPDHSVLTLQLPVTASVREVMA--------ALAQEDS-----WT-------KGQ--VL
VKVNSA----------------------G------DAVGLQPDARGVATSLGLNERLFVV
NPQE---V-HELT-PHPEQL---------------------------GP------TVGSA
EG--LDLVSAKDLAGQLTDHDWNLFNSIHQVELIH-------YVLGPQHL-RDVTTANLE
RFMRR---------FNELQYWV-----ATELCLCPVPGPR------AQLLRKFIKLAAHL
---------------KEQKNLNSFFAVMF-GLSNSAISRLAHTWE---------------
-------RLPHKVRKLYSA-LERLL--------------------------DPSWNH---
-RVYRL----ALTK-------LSPPVI-PFMP----------------------------
LLLKDMTFIHEGNH--TL-VE-----------------------------N-LINFE-KM
-------RM---MARAVRMLHHCRSHSS------------------------VPLSPLRS
RVSHFHEDGQAARISTCSEQSLSTR-------S--------------PASSWA-----YV
QQLKVIDNQREL-----------------------------------------SRLSREL
-----------EP-----------------------------------------------
------------------------------------------------------------
------------------------------------------------------------
------------------------------------------------------------
------------------------------------------------------------
------------------------------------------------------------
------------------------------------------------------------
------------------------------------------------------------
------------------------------------------------------------
------------------------------------------------------------
```


>Rapgef3_[Erinaceus_europaeus]_1016600502

---------------------M--------------------------------------

---------------K---------------------------------VGWPGEG----
-----SWQVGLAVEDVGLALGAPPVGG----LPDALPEGTLLSMVLRRMHRPRSCSYQ-L
LLE------------------------PQRP-S---------RIQ---------------
------------------GLR-------------------WT------------------
-----PLTDSVESLDFSG-SLEQA-------------------S--------T-------
-------ERVL-------------RA---------------------GKQLH-RHLLATC
PN-L----------------IRDRKHH------L-RPY----------------RQC-CS
GR-----------------ELVDAVLALG------------------------------L
----GVH--------SRSQAVGIC---QVLLDDGALC-----------------------
--------------H------------------------VKH-DWTFQDRETQFYRFPGP
-------EPEP--VGIHEL---

---------EEEL------VEALALLSQRGPDAL-------------LTVALRKPPGQRT
DEELD-----LIFEELLHIKAVAHLS-NSVKRELA-AVLLFEPHSKAGTVL---------
--FSQGDKG--TSWYIIW
KGSVNVVTHGK-----GL-------------------------------VTTLHEG-DDF
GQLAL-----------VNDAPRA---ATIILREDNCHFLRVDKQDFNR------IIKD--
---V--------------

------------------------EAKTMRLEEHGKVVLVLER-------T------CQG
SS-----TSQP------------------PAPGRN------------R-YTVMSGTPEKI
LELLLEA------------MRPDSSAHDPT-ETF-------------------LS---D-
FLLTHSV--FM---------------PSTQLCTALL-----HHF------H---------
-----------------------------AEPA---GGSEQERSTYICNKRQQILRLVSQ
WVALYGPMLRTD-TVATSFLQ----KLSDL--VSRDA--RLSNLLR---EQ-WPERRR-H
HRL----ENGCGNA--SP------QMKAR----------------------NLP------
-----VW-----------LPSQD--------EP---------------------------
-----------------S--
---PSSN---

----CVIQVGDK--VP-----------------------------YD-------------
ICRPDHTVLTLQLPVTASVREVLA--------ALAQEDG-----WT-------KGQ--VL
VKVNSA----------------------G------DAVGLQPDARGVATSLGLNERLFVV
NPQE---M-NELT-PHPEQL---------------------------GP------TVGAA
EG--LDLVSAKDLAGQLTDHDWGLFNSIHQVELIH-------YVLGPQHL-RDVTTANLE
RFMRR---------FNELQYWV-----ATELCLCPLPGSR------AQLLRKFIKLAAHL
---------------KEQKNLNSFFAIMF-GLSNSAISRLAHTWE---------------
-------RLPHKVRKLYSA-LERLL--------------------------DPSWNH---
-RVYRL----ALSK-------LSPPII-PFMP----------------------------
LLLKDMTFIHEGNH--TL-VE-----------------------------N-LINFE-KM
-------RM---MARAVRMLHHCRSHSS------------------------VPLSPLRS
RVSHLHEDSQAARISTCSEQSLGTR-------S--------------PASTWA-----YV
QQLKVIDNQREL---SRLSREL
-----------EP---

>Rapgef3_[Esox_lucius]_742184352

--MHLFRSYNYR-V
F----PD-----------------RYSVE-S--STVR-----------------------
------------------GIS-------------------WT------------------
-----PLPEALDT---QD-TMKQ---------------------------FLS-------
-------DRIL-------------KA---------------------AKAVY-CALIERN
PG-L----------------IRDRKHH------L-KTY----------------RQC-CS
GK-----------------ELVDWLMKLN------------------------------E
----CFQ--------SRSQAVGMW---QVLVDEGIIV-----------------------
--------------H------------------------VKQ-EFNFHDKDTQLYRFMDS
-------EFGLNQLTNEKD--------------------------------PVTHMAKEK
LL---------INHTTNEKDQS--------------------------------------
---------DDDL------QEGLSLLLQMGPDAL-------------LTMILRKCPSQRS
AEDLE-----VIYEELLHVKAVAHLS-TSVRKELA-AVLVFESHAKAGTVL---------
--FSQGDKG--TSWYIIW
KGSVNVITHGK-----GL-------------------------------VTTLHEG-EDF
GQLAL-----------VNDAPRA---ATIIILREDNCHFLRVDKQDFIR-----ILKD--
---V--------------

------------------------EANTVRLEEHGKAVLVLEK-------T---------
ES-----TSQG------------------GAGTNS------------K-YTVMSGTPEKI
LEHLLET------------VKLDANSNDAI-DP-------------------CVT---D-
FLLTHRV--FM---------------PSSQLCPALQ-----HHY------H---------
-----------------------------SEPSE---GSELEKAAYALNTKQKIVKLIGQ
WVALFGQFLKDD-PIAVDFLE----RVRQE--VAGDT--RLSSMLK---EQ-LRDKRK--
TRV---LENGFGSL--T-------KLN-HQFD----------------------------
------W-----------FTSCE--------ET---------------------------

---MGNC---

----PPIRPQDT--VL-----------------------------YE-------------
LFRPDHTSVTVVLPVISSVQDLLS--------SLVNPG---------------GGH--IL
VKINSM----------------------G------DRVQLKLNATGVYTSLGLNERLFLC
TASQ---V-DQLT-PLREQQ---------------------------GP------EHSNT
EF--LEQMCSKDIAGQLTNYDWELFTAMHEVELVY-------YVFGQHNF-PGTTTANLE

RFVHR---------FNQVQYWV-----VTEVCLCDDLVKR------AMLLKKFIKIAAML
---------------KEQKNLNSFFAVMF-GLSNSAVQRLYKTWE---------------
-------RVPSKTKRVYCA-YERLM--------------------------DPSRNH---
-RAYRL----AVAK-------LTPPYI-PFMP----------------------------
LLLKDMTFMHEGNQ--NY-TE-----------------------------K-LVNFE-KM
-------RM---IAKIVKIVRGCRSTPY------------------------VPSSPQKG
LADRMFLETPSIRVSTYSEQSLPLR-------Y--------------SSNIRH-----YV
QNFEVIDNQRKL---TQLSRGL
-----------EC---

>Rapgef3_[Falco_cherrug]_929429028

---MVHG---------
---C--
--------RLSV--LFKKM--------HLFRSSSYEI-------------RLEGE-GGSL
PRIQG-------IRW--------------------------T------------------
-----PLLD-------TESTLDYGH------------------SLTQAS---S-------
-------EKIW-------------RAGKLLF---------------S----H---LTSTH
PS-L----------------IRDHKHH------L-RHY----------------RQC-CS
GK-----------------ELVDWLLSAG------------------------------L
----TIQ--------TRSQAIGIC---QVLVDGGVLT-----------------------
--------------H------------------------VKQ-EWHFQDKETQFYRFAEL
E-----LSPEL-GAGLRDA---

----------EEL------LEALAFLAQLGPDAL-------------LTMALRKPPAQRT
EDELE-----LIFEELLHIKAVAHLS-NSVKRELA-SVLMFESHQRAGTV----------
---LFSQGDKG--TSWYIIW
KGSVNVVTHGK-----GL-------------------------------VATLHEG-DDF
GQLAL-----------VNDAPRA---ATIILREDNCHFLRVDKQDFNH------ILKD--
---V--------------

------------------------EANTMRLKEHGKVVLVLQKNL-------------QG
G------SSQP------------------AEARSS-R------------YLVMAGTPEKI
LEHLLEF------------MRLDATLYDPV-D-------------------TLLG---D-
FLLTYTV--FM---------------PTSQLCRALL-----HHF------R---------
-----------------------------AEPLE---GSEQEKATYSLHKRRKILRLVSQ
WVLLYGRLLQGD-RSTTTLLQ----NLAD--LASQDP--RLGGLVQ---EQ-AQDRRR--
PRV---LENGHSSV--SP------QPKTRS---------------------SVN------
------W-----------LTSQE--------EA---------------------------
----------------VL--
----NSSC-AL-----------------RAQDK---------------------------

```
------------------------------------------------------------
------------------------------------------------------------
------------------------------------------------------------
------------------------------------------------------------
--------------VP-----------------------------YE-------------
IYRPDHSCLITVLPINASVQDILR--------SLAPQLG-------------QDGE-HIL
VKVNSA----------------------GDKVG------LQLDAVGVFTALGLNERLFAV
SVEE---L-DSLT-PHPEQL---------------------------GP------HVGSS
ET--LDLISSKDLASHLTDYDWNLFKSIHQVEMIH-------YIIGPQKF-HDVTTANLE
RVMRR---------FNELQYWV-----ATELCLCPEVGRR------AQLLRKFIKLAAHL
---------------KEQKNLNSFFAVMF-GVSNTAVSRLAKTWE---------------
-------RLPHKIRKLHSV-LERML--------------------------DPSWNH---
-RVYRL----AVAK-------LSPPII-PFVPL-LLK-----------------------
----DMTFIHEGNR--TLA------------------------------EN-LINFE-KM
-------HM---MAKTVRVLQRCRG--HA----------------------HAPLSPLRN
RSPHRTEDPRAVRIST--------------------------------------------
-----------C-----------------------------------------SEQSLSM
RSPVSTWAY-LQHLKAIDSQKELLRLSHDLES----------------------------
------------------------------------------------------------
------------------------------------------------------------
------------------------------------------------------------
------------------------------------------------------------
------------------------------------------------------------
------------------------------------------------------------
------------------------------------------------------------
------------------------------------------------------------
------------------------------------------------------------
------------------------------------------------------------
---
>Rapgef3_[Felis_catus]_410964201
------------------------------------------------------------
---------------------M--------------------------------------
------------------------------------------------------------
---------------K---------------------------------VGWPGES----
-----RWQVGLAVED-SPALGASQVGG----LPDVVPEGTLLNMVLKRMHRPRSCSYQ-L
LLE------------------------HQRP-S---------CIQ---------------
------------------GLR-------------------WT------------------
-----PLTDSEESLDFSV-SLEQA-------------------S--------T-------
-------ERVL-------------RA---------------------GKQLH-RHLLATC
PT-L----------------IRDRKYH------L-RLY----------------RQC-CS
GR-----------------ELVDGILALS------------------------------L
----GVH--------SRNQAVGIC---QVLLDEGALC-----------------------
--------------H------------------------VKH-DWTFQDRDTQFYRFPGP
-------EPEP--VGVREL-----------------------------------------
------------------------------------------------------------
---------EEEL------VEAMALLSQRGPDAL-------------LTVALRKPPGQRT
DEELD-----LIFEELLHIKAVAHLS-NSVKRELA-AVLLFEPHSRAGTVL---------
--------------------------------------------FSQGDKG--TSWYIIW
KGSVNVVTHGK-----GL-------------------------------VTTLHEG-DDF
GQLAL-----------VNDAPRA---ATIILREDNCHFLRVDKQDFNR------IIKD--
---------------------------------------------V--------------
------------------------------------------------------------
------------------------------------------------------------
------------------------EAKTMRLEEHGKVVLVLER-------A------SQG
```

AG-----PSRP------------------PTPGRN------------R-YTVMSGTPEKI
LELLLEA------------MRPDSSAHDPT-EKF-------------------LS---D-
FLLTHSV--FM---------------PSAQLCAALL-----HHF------H---------
-----------------------------AEPS---GGSEQEHSTYVCNKRQQVLRLVSQ
WVALYGPMLYAD-PVATSFLQ----KLSDL--VSRDA--RLCILLR---EQ-WPERRK-H
HRM----ENGCGNA--SP------QMKAR----------------------NMP------
-----VW-----------LPGQD--------QP---------------------------
-----------------L--
---PSSN---

----CAIRVGDK--VP-----------------------------YD-------------
IYRPDHSVLTLKLPVTASVREVMA--------ALAQEDG-----WT-------KGQ--EL
VKVNSA----------------------G------DAIGLQPDARGVATSLGLNERLFVV
NPQE---V-RELT-PLPEQL---------------------------GP------SVGSA
EG--LDLVSAKDLAGQLTDHDWNLFNSIHQVELIH-------YVLGPQHL-RDVTTANLE
RFMRR---------FNELQYWV-----ATELCLCPVPGLR------AQLLRKFIKLAAHL
---------------KEQKNLNSFFAVMF-GLSNSAISRLAHTWE---------------
-------RLPHKVRKLYSA-LERLL--------------------------DPSWNH---
-RVYRL----ALTK-------LSPPII-PFMP----------------------------
LLLKDMTFIHEGNH--TL-VE-----------------------------N-LINFE-KM
-------RM---MARAVRMLHHCRSHSN------------------------VPLSPLRS
RVSHFHEDSQAARTSTCSEQSLNTR-------S--------------PASTWA-----YV
QQLKVIDNQREL---SRLSREL
-----------EP---

>Rapgef3_[Ficedula_albicollis]_1020933356

---MAASERPAH---G
TGTR-PGSGNRSGSENGTRRGFRQRR----------------ALGQGGQRPFRARFC---
---SGSRGKQAR--LFRDL--------RLRRKSAPIR-------------SLRRP-GLDI
PWDPQ-DGPEL------------------------------P------------------
-----PLLGDSRAPSGARTALTCTHKGDK--------------NLLQPG---V-------
-------TPIPA---PTGDKPAAPRGDRQLWGDTPVPPPPPG--FRA----A---VARSE
PG-C----------------LGPRGHRGSATFAP-RSG----------------RGC-GD
GGSSLPPSLCFYPEHPRRGELGENEVQRG------------------------------S
----ESG--------SVREKWGFGFWIQDLVTGELPP-----------------------
--------------HLVKIWGHFGGVRTPSHGSVGCPPAVRQ-EWHFQDKESQFYRFAEL
E-----LSPEP-GAGPREP---

```
----------EEL------LEALGFLGQLGPDAL-------------LSMALRKPPAQRT
QDELE-----LIFEELLHIKAVAHLS-NSVKRELA-AVLMFEGHQRAGTV----------
-------------------------------------------LFSQGDKG--TSWYIIW
KGSVNVVTHGK-----GL-------------------------------VATLHEG-DDF
GQLAL-----------VNDAPRA---ATILLREDNCHFLRVDKHDFNR------ILKD--
---------------------------------------------V--------------
------------------------------------------------------------
------------------------------------------------------------
------------------------EANTVRLKEHGKVVLVLQKDL-------------QG
G------GGQA------------------ASAR-SSR------------YLVMAGTPEKI
LEHLLEF------------MRLDATVYDPV-D-------------------TLLG---D-
FLLTYTV--FM---------------PTSQLCRALL-----HHF------R---------
-----------------------------AEPLE---GSEQDKAAYSLRKRRKILRLVSQ
WVLLYGRLLQGD-RSTTALLQ----NLAD--LASRAP--Q-----------------G--
PPT---GWAGPGAG--PG------AAATPS---------------------CPP------
------S-----------VLQGP--------PV---------------------------
----------------FP------------------------------------------
----DATA-GV-----------------RGRPE---------------------------
------------------------------------------------------------
------------------------------------------------------------
------------------------------------------------------------
------------------------------------------------------------
--------------C---------------------------------------------
--RGWRVPGVTRIPGMTQTPGVTG--------G-DPNSG-------------GDPR-IPG
MTRTPG----------------------GS---------------GVFSR----------
----------AQT-PHPEQL---------------------------GP------HAGSS
DT--LDLISSKELASHLTDYDWSLFKSIHQVEMIH-------YIVGPQKF-HEVATANLA
RVMRR---------FNELQFWV-----ATELCLCPELGRR------AQLLRKFIKLAAHL
---------------KEQKNLNSFFAVMF-GVSNTAVTRLAKTWE---------------
-------RLPHKIRKLHSA-LERML--------------------------DPSWNH---
-RVYRL----AVAK-------LSPPII-PFVPL-LLKGLCP-------------------
----------------ALGT---------------------------------------L
W-----------------------------------------------------VCPTLS
GGPSDPRE--AVRIST--------------------------------------------
-----------C-----------------------------------------SEQSLSM
RSPVCTWAY-LQHLRAIDSQKELLRLSRNLEP----------------------------
------------------------------------------------------------
------------------------------------------------------------
------------------------------------------------------------
------------------------------------------------------------
------------------------------------------------------------
------------------------------------------------------------
------------------------------------------------------------
------------------------------------------------------------
------------------------------------------------------------
------------------------------------------------------------
---
>Rapgef3_[Fukomys_damarensis]_731247212
------------------------------------------------------------
------------------------------------------------------------
------------------------------------------------------------
------------------------------------------------------------
-------------------------------------------MVLRRMHLPRSCSYQ-L
LLE------------------------HRRP-A---------HIQ---------------
```

```
------------------GLR-------------------WT------------------
-----PLTNSEESLDFSD-SLEQV-------------------S--------T-------
-------ERVL-------------RA---------------------GRYLH-RHLLAAC
PT-L----------------IRDRKYH------L-RLY----------------RQC-CS
GR-----------------ELVDGILALG------------------------------V
----GVH--------SRSQAVGIC---QVLLDEGALC-----------------------
--------------H------------------------VKH-DWTFQDRDAQFYRFLGL
-------EPQP--AGPHEL-----------------------------------------
------------------------------------------------------------
---------EEEL------VEAMALLSQRGPDAL-------------LTVALRKPPGQRM
DEELD-----LIFEELLHIKAVAHLS-NSVKRELA-AVLVFEPHSKAGTVL---------
--------------------------------------------FSQGDKG--TSWYIIW
KGSVNVVTHGK-----GL-------------------------------VTTLHEG-DDF
GQLAL-----------VNDAPRA---ATIILREDNCHFLRVDKQDFNR------IIKD--
---------------------------------------------V--------------
------------------------------------------------------------
------------------------------------------------------------
------------------------EAKTMRLEEHGKVVLVLEK-------T------SQG
AG-----PSRP------------------QTPGQN------------R-YKVMSGTPEKI
LELLLEA------------MRPDSSAHDPT-EMF-------------------LS---D-
FLLTHSV--FM---------------PTAQLCTALL-----HHF------H---------
-----------------------------AGPAESAGGSEQECSTYICNRRQQILRLVSH
WVALYGPMLHKD-PVATSFLQ----KLSDL--VSKDS--RLSNLLR---EQ-GSERRR-H
HRL----ENGYGSA--SP------QLKVR----------------------NIP------
-----VW-----------LPSQD--------EP---------------------------
-----------------L------------------------------------------
---PSSN-----------------------------------------------------
------------------------------------------------------------
------------------------------------------------------------
------------------------------------------------------------
------------------------------------------------------------
----CAIRVGDK--VP-----------------------------YD-------------
ICRPDHSVLTLQLPVTASVREVMV--------ALAQEDG-----WT-------KGQ--VL
VKVNSA----------------------G------DAIGLQPDARGVATSLGLNERLFVV
NQQE---V-HKLT-PHPEQL---------------------------GP------TVGSA
EG--LDMVSAKDLAGQLTDHDWNLFNSIHQVELIH-------YVLGPQHL-RDVTTANLE
RLMRR---------FNELQYWV-----ATELCLCSVPGLR------AQLLRKFIKLAAHL
---------------KEQKNLNSFFAIMF-GLSNSAISRLAHTWE---------------
-------RLPHKVRKLYSA-LERLL--------------------------DPSWNH---
-RVYRL----ALTK-------LSPPII-PFMP----------------------------
LLLKDMTFIHEGNH--TL-VE-----------------------------N-LINFE-KM
-------RM---MARAVRLLHHCRSHST------------------------VPLSPLRS
RVSHLHEDSQASRISTCSEQSLSTR-------S--------------PASTWA-----YV
QQLKVIDNQREL-----------------------------------------SRLSREL
-----------EP-----------------------------------------------
------------------------------------------------------------
------------------------------------------------------------
------------------------------------------------------------
------------------------------------------------------------
------------------------------------------------------------
------------------------------------------------------------
------------------------------------------------------------
------------------------------------------------------------
------------------------------------------------------------
```


>Rapgef3_[Fundulus_heteroclitus]_831559262

--MHLFRTHNYQ-V
F----PH-----------------RYSVE-K--PTIR-----------------------
------------------GIS-------------------WT------------------
-----PLPEALDP---KD-TMKQ---------------------------FLS-------
-------DRVV-------------KA---------------------ARSVY-SVMIEKN
PG-L----------------IRDRKHH------L-KTH----------------RQC-CS
GK-----------------ELVDWLMKQS------------------------------E
----FLQ--------SRSQAVGMW---QVLVDERIIV-----------------------
--------------H------------------------VKQ-DLNFLDKDTQFYRFQDA
-------ELGLSHVSNEK--
DL--
---------EEEL------HEALSLLSQLGPDAL-------------LSMILRKCPTQRS
AEDIE-----VIYEELLHVKAAAHLS-SSVRKELA-AVLVFESHVKSGTVL---------
--FSQGDKG--TSWYIIW
KGSVNVITHGK-----GI-------------------------------VTTLHEG-EDF
GQLAL-----------LNDAPRA---ATIILREDNCHFLRVDKQDFIR------ILKD--
---V--------------

------------------------EANTVRLEEHGRTVVVLEK-------S---------
AD----CNGQG------------------GAAANS------------K-YTVMSGTPEKI
LEHLLDT------------LKLDSNGNDPI-DP-------------------CVS---D-
FLLTHKV--FM---------------PSSQLCSALQ-----HHY------Q---------
-----------------------------AELSE---GSEQEKTAYVFNTKQRVVKLIAQ
WVALYGLLLKED-PDVWDFLE----RLKKE--VGADN--RLSSILK---EQ-FRERRR--
TKV---LENGYQSL--S-------R-N-QQFD----------------------------
------W-----------FSSCE--------EP---------------------------

---AGRL---

----QPIKSQDK--VL-----------------------------YE-------------
VYTPDSKPLSLMLPVNTSVQEVMS--------AIVKAE---------------GDH--VL
VKMNST----------------------G------ERAQLKLDATAVYTALGVNERLFIC
TSSE---V-EQLR-PLKEQQ---------------------------GP------EQGTT
DL--MEQMSSKDVAIELTNYDWELFTAMHEVELVY-------YIFGRHKF-PGATTANLE
RFVRH---------FNVVQYWV-----VTELCLCEDLVKR------AILLKKFIKIASVL
---------------KEQRNLNSFFAVMF-GLGNSAVQRLYKTWD---------------
-------RIPSKTKRIYCA-YERLM--------------------------DPTRNH---
-RAYRL----AVAK-------LSPPYI-PFMP----------------------------
LLLKDMTFIHEGNP--NY-VD-----------------------------K-LVNFE-KM
-------RM---LAKTVKIVRECRSQPY------------------------VPSSPQRG
LADRMFLDGPATRLSTYSDHALPLR-------S--------------PSNIRH-----YI
QNLKVIDNQRRL---TQLSRTI
-----------EC---

>Rapgef3_[Galeopterus_variegatus]_667328784

-------MLHTD-PVATSFLQ----KLSDL--VSRDS--RLSNLLR---EQ-WPERRR-H
HRL----ENGCGNA--SP------QMKAW----------------------NVP------
-----VW-----------LPGQD--------EL---------------------------
-----------------L--
---HSSN---

----CAIRAGDK--VP-----------------------------YD-------------
ICRPDHSVLTLQLPVTASVREVMV--------ALAQEDG-----WT-------KGQ--VL
VKVNSA----------------------G------DAIGLQPDARGVATSLGLNERLFVV
NPQE---V-HEL--
------------------------------VELIH-------YVLGPQHL-RDVTTANLE

RFMHR---------FNELQYWV-----ATELCLCPVPGPR------AQLLRKFIKLAAHL
---------------KEQKNLNSFFAVMF-GLSNSAISRLAHTWE---------------
-------RLPHKIRKLYSA-LERLL--------------------------DPSWNH---
-RVYRL----ALTK-------LSPPII-PFMP----------------------------
LLLKDMTFIQEGNH--TL-VE-----------------------------N-LINFE-KM
-------RM---MARAVRMLHHCRSHSS------------------------VPLSPLRS
RASHLHEDSQVARISTCSEQSLSTR-------S--------------AANTWA-----YI
QQLKVIDNQREL---SRLSREL
-----------EP---

>Rapgef3_[Gallus_gallus]_971436898

---MKVRGAGTATATG
TGTG-IGSGGEAERRGAAPVGT-----HCGRTGLERCPGAVPPPTP----PPPPS----P
GGSGGDGKRLSV--LLKKM--------HLFRSSSYEI-------------RLEGE-GGSL
PRIQG-------IRW--------------------------T------------------
-----PLLD-------AESSLDYGH------------------SLTQAS---S-------
-------EKIW-------------RAGKLLF---------------T----Y---LTSTS
PS-L----------------IRDHKHH------L-RHH----------------RRC-CS
GK-----------------ELVDWMLNAE------------------------------L
----GIQ--------TRSQAIGVG---QVLVDGGVLT-----------------------
--------------H------------------------VKQ-EWHFQDKDTQFYRFAEL
E-----LSPEP-RVGLRDA---

----------EEL------LEAVTFLAQLGPDAL-------------LTMALRKPPAQRT
EDELE-----LIFEELLHIKAVAHLS-NSVKRELA-SVLMFESHQRAGTV----------
---LFSQGDKG--TSWYIVW
KGSVNVVTHGK-----GL-------------------------------VATLHEG-DDF
GKLAL-----------VNDAPRA---ASIVLREDNCHFLRVDKQDFNR------ILKD--
---V--------------

------------------------EANTMRLKEHGKVVLVLQKNL-------------QG
G------SSQP------------------AATRSS-R------------YLVMAGTPEKI
LEHLLEF------------MRLDATLYDPV-D-------------------TLLG---D-
FLLTYTV--FM---------------PTSQLCRALL-----HHF------R---------
-----------------------------AEPLD---GSEQEKATYCLHKRRKILRLVSQ
WVLLYGRLLQGD-RSTTALLQ----SLAD--LASQDP--RLGAMVQ---E---QERRR--
PRA---MENGDGSI--SP------QPKSRS---------------------SMS------
------W-----------LSSQD--------EA---------------------------
----------------AL--
----SSSC-AL-----------------RAHDK---------------------------

--------------VP-----------------------------YE-------------
VYRADHSCLVTVLPVNASVRDVLQ--------SLTPQLG-------------WDGE-HLL
VKVNSA----------------------GDKVG------LQLDAVGVFTSLGLNERLFAV
SVEE---L-GGLT-PHPEQL---------------------------GP------HVGSS
DT--LDLISSKDLASHLTDHDWNLFKSIHQVEMIH-------YIMGPQKF-HEVTTANLE
RMMRR---------FNELQYWV-----ATELCLCPELGRR------AQLLRKFIKLAAHL
---------------KEQKNLNSFFAVMF-GVSNTAVSRLAKTWE---------------
-------RLPHKIRKLHAA-LERML--------------------------DPSWNH---
-RVYRL----AVAK-------LSPPII-PFVPL-LLK-----------------------
----DMTFIHEGNR--TLA------------------------------EN-LINFE-KM
-------HM---MAKTVRILQRCRG--QA----------------------HAPLSPLRN
RSPHRPEDARAIRIST--
-----------C---SEQSLSV
RSPVSTWAY-LQHLRAIDSQKELLRLSRDLES----------------------------

>Rapgef3_[Geospiza_fortis]_930243948

---------------------MHLFRSSS------------------------------Y
----DVR--------LEGA-------------GGSLP-----------------------
--------------R------------------------IQGIRW---------------

-----------------------------VKRELA-AVLMFEGHQRAGTV----------
---LFSQGDKG--TSWYIIW
KGSVNVVTHGK-----GL-------------------------------VATLHEG-DDF
GQLAL-----------VNDAPRA---ATILLREDNCHFLRVDKQDFNR------ILKD--
---V--------------

------------------------EANTVRLKEHGKVVLVLQKDL-------------QG

G------SGQA------------------ASGR-----------S-SR-YLVMAGTPEKI
LEHLLEF------------MRLDATLYDPV-D-------------------TLLG---D-
FLLTYTV--FM---------------PTSQLCRALL-----HHF------R---------
-----------------------------AEPLE---GSEQDKASYSLRKRRKILRLVSQ
WVLLYGRLL-GE-RGS---LE----G-SGVLSRAQTP--HPEQLGP---HA-GSSDTL--
D---

------LISSKDLASHLTDYDWNLFKSIHQVEMIH-------YIVGPHKF-HEVATANLA
RVLRRGCQPRDPIPAQRLPHKIRKLHSALERMLDPSWNHRVYRLAVAKLSPPIIPFVPLL
--------------------LKDMTFIHE-GNRTLAENLINFEKM---------------
----------HMMAKTVRV-LQ--------------------------------------

---------------------RCRG--HA----------------------HAPLSPLRS
RSPHRPEDTKAVRIST--
-----------C---SEQSLSV
RNPVSTWAY-LQHLRAIDSQKELLRLSRELEP----------------------------

>Rapgef3_[Gorilla_gorilla_gorilla]_426372289

---------------------M--------------------------------------

---------------K---------------------------------VGWPGEN----
-----CWQVGLAVED-SPALGAPRVGA----LPDVVPEGTLLNMVLRRMHRPRSCSYQ-L
LLE------------------------HQRP-S---------CIQ---------------
------------------GLR-------------------WT------------------
-----PLTNSEESLDFSE-SLEQA-------------------S--------T-------
-------ERVL-------------RA---------------------GRQLH-RHLLATC
PN-L----------------IRDRKYH------L-RLY----------------RQC-CS
GR-----------------ELVDGILALG------------------------------L
----GVH--------SRSQVVGIC---QVLLDEGALC-----------------------
--------------H------------------------VKH-DWAFQDRDAQFYRFPGP
-------EPEP--VGTHEM---

---------EEEL------AEAVALLSQRGPDAL-------------LTVALRKPPGQRT
DEELD-----LIFEELLHIKAVAHLS-NSVKRELA-AVLLFEPHSKAGTVL---------
--FSQGDKG--TSWYIIW
KGSVNVVTHGK-----GL-------------------------------VTTLHEG-DDF
GQLAL-----------VNDAPRA---ATIILREDNCHFLRVDKQDFNR------IIKD--
---V--------------

------------------------EAKTMRLEEHGKVVLVLER-------A------SQG
AG-----PSRP------------------PTPGRN------------R-YTVMSGTPEKI
LELLLEA------------MGPDSSAHDPT-ETF-------------------LS---D-
FLLTHRV--FM---------------PSAQLCAALL-----HHF------H---------
-----------------------------VEPA---GGSEQERSTYVCNKRQQILRLVSQ
WVALYGSMLHTD-PVATSFLQ----KLSDL--VGRDT--RLSNLLR---EQ-WPERRR-C
HRL----ENGCGNA--SP------QMKAR----------------------NLP------
-----VW-----------LPNQD--------EP---------------------------
-----------------L--
---PGSS---

----CAIRVGDK--VP-----------------------------YD-------------
ICRPDHSVLTLQLPVTASVREVMA--------ALAQEDG-----WT-------KGQ--VL
VKVNSA----------------------G------DAIGLQPDARGVATSLGLNERLFVV
NPQE---V-HELI-PHPDQL---------------------------GP------TVGSA
EG--LDLVSAKDLAGQLTDHDWSLFNSIHQVELIH-------YVLGPQHL-RDVTTANLE
RFMRR---------FNELQYWV-----ATELCLCPVPGSR------AQLLRKFIKLAAHL
---------------KEQKNLNSFFAVMF-GLSNSAISRLAHTWE---------------
-------RLPHKVRKLYSA-LERLL--------------------------DPSWNH---
-RVYRL----ALAK-------LSPPVI-PFMP----------------------------
LLLKDMTFIHEGNH--TL-VE-----------------------------N-LINFE-KM
-------RM---MARAARMLHHCRSHNP------------------------VPLSPLRS
RVSHLHEDSQVARISTCSEQSLSTR-------S--------------PASTWA-----YV
QQLKVIDNQREL---SRLSREL
-----------EP---

>Rapgef3_[Haliaeetus_leucocephalus]_729737483

---MKVR---------
---G-TGAGGEAARSGPGSVPVARSRQRTRRTGLEPGTGAVRPPPPSSSRLPPPSSPPPP
GGPGTDGNRLSG--LLKKM--------HLFRSSSYEI-------------RLEGE-GSSL

```
PRIQG-------IRW--------------------------T------------------
-----PLLD-------TESTLDYGH------------------SLTQAS---S-------
-------EKIW-------------RAGKLLF---------------I----H---LTSTR
PG-L----------------IRDHKHH------L-RHH----------------RQC-CS
GK-----------------ELVDWLLSAG------------------------------L
----AVQ--------TRSQAIGIC---QVLVDGGVLT-----------------------
--------------H------------------------VKQ-EWHFQDKDTQFYRFAEL
E-----LSPEP-SAGLRDA-----------------------------------------
------------------------------------------------------------
----------EEL------LEALAFLAQLGPDAL-------------LTMALRKPPAQRT
EDELE-----LIFEELLHIKAVAHLS-NSVKRELA-SVLMFESHQRAGTV----------
-------------------------------------------LFSQGDKG--TSWYIIW
KGSVNVVTHGK-----GL-------------------------------VATLHEG-DDF
GQLAL-----------VNDAPRA---ATIILREDNCHFLRVDKQDFNH------ILKD--
---------------------------------------------V--------------
------------------------------------------------------------
------------------------------------------------------------
------------------------EANTMRLKEHGKVVLVLQKNL-------------QG
G------SSQS------------------ATARSS-R------------YFVMAGTPEKI
LEHLLEF------------MRLDATLYDPM-D-------------------TLLG---D-
FLLTYTV--FM---------------PTSQLCRALL-----HHF------R---------
-----------------------------AEPLE---GSEQEKATYSLHKRRKILRLVSQ
WVLLYGRLLQGD-RSTTALLQ----NLAD--LASQDP--RLGGLVQ---EQ-AQDRRR--
PRA---LENGDGSV--SP------QPKARS---------------------SGN------
------W-----------LTSQE--------EA---------------------------
----------------IL------------------------------------------
----NSSC-AL-----------------RAQDK---------------------------
------------------------------------------------------------
------------------------------------------------------------
------------------------------------------------------------
------------------------------------------------------------
--------------VP-----------------------------YE-------------
IYRPDHSCLITVLPVNASVRDVLR--------SLAPRLG-------------RDGE-HIL
VKVNSA----------------------GDKVG------LQLDAVGVFTALGLNERLFAV
SVEE---L-GSLT-PHPEQL---------------------------GP------HVGSS
ET--LDLISSKDLASHLTDYDWNLFKSIHQVEMIH-------YIVGPQKF-HDVTTANLE
RVMRR---------FNELQYWV-----ATELCLCPEVGRR------AQLLRKFIKLAAHL
---------------KEQKNLNSFFAVMF-GVSNTAVSRLAKTWE---------------
-------RLPHKIRKLHSA-LERML--------------------------DPSWNH---
-RVYRL----AVAK-------LSPPII-PFVPL-LLK-----------------------
----DMTFIHEGNR--TLA------------------------------EN-LINFE-KM
-------HM---MAKTVRVLQRCRG--HA----------------------HAPLSPLRN
RSPHRPEDPKAIRIST--------------------------------------------
-----------C-----------------------------------------SEQSLSV
RSPVSTWAY-LQHLKAIDSQKELLRLSHDLES----------------------------
------------------------------------------------------------
------------------------------------------------------------
------------------------------------------------------------
------------------------------------------------------------
------------------------------------------------------------
------------------------------------------------------------
------------------------------------------------------------
------------------------------------------------------------
------------------------------------------------------------
```


>Rapgef3_[Heterocephalus_glaber]_513003374

---MKVGWPGES----
-----HWQLDPAVED-SPVLGVPQIGG----LPHVVPEGTLLNMVLRRMHLPRSCSYQ-L
LLE------------------------HRRP-N---------HIQ---------------
------------------GLR-------------------WT------------------
-----PLANSEESLDFSE-SLEQV-------------------S--------T-------
-------ERVL-------------RA---------------------GRDLH-RHLLATC
PT-L----------------IRDRKYH------L-RLY----------------RQC-CS
GR-----------------ELVDGILALG------------------------------L
----GVH--------SRSQAVGIC---QVLLDEGALC-----------------------
--------------H------------------------VKH-DWTFQDRDAQFYRFLGP
-------EPQP--VGPHEL---

---------EEEL------VEAMALLSQRGPDAL-------------LTVALRKLPGQRT
DEELD-----LVFEELLHIKAVAHLS-NSVKRELA-AVLVFEPHSKAGTVL---------
--FSQGDKG--TSWYIIW
KGSVNVVTHGK-----GL-------------------------------VTTLHEG-DDF
GQLAL-----------VNDAPRA---ATIILREDNCHFLRVDKQDFNR------IIKD--
---V--------------

------------------------EAKTMRLEEHGKVVLVLER-------T------SQG
AG-----PSRL------------------PSPGRN------------R-YKVMSGTPEKI
LELLLEA------------MRPDSTAHDPT-ETF-------------------LS---D-
FLLTHSV--FM---------------PTAQLCTALL-----HHF------H---------
-----------------------------AGPADSAGGSELERSTYICNRRQQILRLVSQ
WMALYGPMLHKD-PVATSFLQ----KLSDL--VNKDA--RLSNLLR---EQ-WPERRR-H
HKL----ENGCGST--SP------QLKAR----------------------DTP------
-----VW-----------LPCQD--------EP---------------------------
-----------------L--
---PSSN---

----CAIRVGDK--VP-----------------------------YD-------------
ICRPDHSVLTLYLPVTASVREVMA--------ALAQEDG-----WT-------KGQ--VL
VKVNSA----------------------G------DATGLQPDARGVATSLGLNERLFVV
NQQE---V-HKLT-PHPEQL---------------------------GP------TVGSA
EG--LDMISAKDLAGQLTDQDWSLFNSIHQVELIH-------YVLGPQHL-RDVTTANLE
RFMRR---------FNQLQYWV-----ATELCLCSVAGLR------AQLLRKFIKLAAHL
---------------KEQKNLNSFFAVMF-GLSNSAISRLAHTWE---------------
-------RLPHKVRKLYSA-LERLL--------------------------DPSWNH---
-RVYRL----ALAK-------LSPPII-PFMP----------------------------
LLLKDMTFIHEGNH--TL-VE-----------------------------N-LINFE-KM
-------RM---MARTARLLHHCRSHST------------------------VPLSPLRS
RVSHLHEDGQASRIST--


```
------------------------------------------------------------
------------------------------------------------------------
------------------------------------------------------------
------------------------------------------------------------
------------------------------------------------------------
------------------------------------------------------------
------------------------------------------------------------
------------------------------------------------------------
------------------------------------------------------------
------------------------------------------------------------
---
>Rapgef3_[Homo_sapiens]_148747859
------------------------------------------------------------
---------------------M--------------------------------------
------------------------------------------------------------
---------------K---------------------------------VGWPGES----
-----CWQVGLAVED-SPALGAPRVGA----LPDVVPEGTLLNMVLRRMHRPRSCSYQ-L
LLE------------------------HQRP-S---------CIQ---------------
------------------GLR-------------------WT------------------
-----PLTNSEESLDFSE-SLEQA-------------------S--------T-------
-------ERVL-------------RA---------------------GRQLH-RHLLATC
PN-L----------------IRDRKYH------L-RLY----------------RQC-CS
GR-----------------ELVDGILALG------------------------------L
----GVH--------SRSQVVGIC---QVLLDEGALC-----------------------
--------------H------------------------VKH-DWAFQDRDAQFYRFPGP
-------EPEP--VGTHEM-----------------------------------------
------------------------------------------------------------
---------EEEL------AEAVALLSQRGPDAL-------------LTVALRKPPGQRT
DEELD-----LIFEELLHIKAVAHLS-NSVKRELA-AVLLFEPHSKAGTVL---------
--------------------------------------------FSQGDKG--TSWYIIW
KGSVNVVTHGK-----GL-------------------------------VTTLHEG-DDF
GQLAL-----------VNDAPRA---ATIIILREDNCHFLRVDKQDFNR-----IIKD--
---------------------------------------------V--------------
------------------------------------------------------------
------------------------------------------------------------
------------------------EAKTMRLEEHGKVVLVLER-------A------SQG
AG-----PSRP------------------PTPGRN------------R-YTVMSGTPEKI
LELLLEA------------MGPDSSAHDPT-ETF-------------------LS---D-
FLLTHRV--FM---------------PSAQLCAALL-----HHF------H---------
-----------------------------VEPA---GGSEQERSTYVCNKRQQILRLVSQ
WVALYGSMLHTD-PVATSFLQ----KLSDL--VGRDT--RLSNLLR---EQ-WPERRR-C
HRL----ENGCGNA--SP------QMKAR----------------------NLP------
-----VW-----------LPNQD--------EP---------------------------
-----------------L------------------------------------------
---PGSS-----------------------------------------------------
------------------------------------------------------------
------------------------------------------------------------
------------------------------------------------------------
------------------------------------------------------------
----CAIQVGDK--VP-----------------------------YD-------------
ICRPDHSVLTLQLPVTASVREVMA--------ALAQEDG-----WT-------KGQ--VL
VKVNSA----------------------G------DAIGLQPDARGVATSLGLNERLFVV
NPQE---V-HELI-PHPDQL---------------------------GP------TVGSA
EG--LDLVSAKDLAGQLTDHDWSLFNSIHQVELIH-------YVLGPQHL-RDVTTANLE
```

RFMRR---------FNELQYWV-----ATELCLCPVPGPR------AQLLRKFIKLAAHL
---------------KEQKNLNSFFAVMF-GLSNSAISRLAHTWE---------------
-------RLPHKVRKLYSA-LERLL--------------------------DPSWNH---
-RVYRL----ALAK-------LSPPVI-PFMP----------------------------
LLLKDMTFIHEGNH--TL-VE-----------------------------N-LINFE-KM
-------RM---MARAARMLHHCRSHNP------------------------VPLSPLRS
RVSHLHEDSQVARISTCSEQSLSTR-------S--------------PASTWA-----YV
QQLKVIDNQREL---SRLSREL
-----------EP---

>Rapgef3_[Ictidomys_tridecemlineatus]_914907256

---MVLKRMHRPRSCSYQ-L
LLE------------------------HQRP-S---------RIQ---------------
------------------GLR-------------------WT------------------
-----PLTNSEESLDFSV-SLEQA-------------------S--------T-------
-------ERVL-------------RA---------------------GKHLH-RHLLATC
PT-L----------------IRDRKYH------L-RLY----------------RQC-CS
GR-----------------ELVDWVLALG------------------------------L
----GVH--------SRSQAVGIC---QVLLDEGALC-----------------------
--------------H------------------------VKH-DWTFQDRDAQFYRFPGP
-------EPEP--TGAHEM---

---------EEEL------VEAMALLSQRGPDAL-------------LTVALRKPPGQRT
DEELE-----LIFEELLHIKAVAHLS-NSVKRELA-AVLLFEPHSKAGTVL---------
--FSQGDKG--TSWYIIW
KGSVNVVTHGK-----GL-------------------------------VTTLHEG-DDF
GQLAL-----------VNDAPRA---ATIILREDNCHFLRVDKQDFNR------IVKD--
---V--------------

------------------------EAKTMRLEEHGKVVLVLER-------A------SQG
TG-----PSRP------------------PTPGRN------------R-YTVMSGTPEKI
LELLLEA------------MRPDSSAHDPT-ETF-------------------LS---D-
FLLTHSV--FM---------------PSAQLCAALL-----HHF------H---------
-----------------------------VEPAEPAGGSEQERSTYICNKRQQILRLVSQ
WVALYGPMLHTD-PVATSFLQ----KLSDL--VSRDA--RLSNLLR---EQ-WPERRR-H
HRL----ENGCGNA--SP------MMKVPAQSQQGSP------------FFPLP------
-----IQ-----------LPYLE--------GP---------------------------
-----------------M--
---WGGS---

-------PPTDKPLIP-----------------------------YD-------------
ICLPDHSVLTLQLPVTASVREVMA--------ALAQKDG-----WT-------KGQ--VL
VKVNSA----------------------G------DAVGLQPDARGVATSLGLNERLFVV
DPQE---V-HELT-PHPEQL---------------------------GP------TVGSA
EG--LESVSAKDLAGQLTDHDWNLFNSIHQVELIH-------YVLGPQHL-RDVTTANLE
RFMRR---------FNELQYWV-----ATELCLCPVPGLR------AQLLRKFIKLAAHL
---------------KEQKNLNSFFAIMF-GLSNSAISRLAHTWE---------------
-------RLPHKVRKLYSA-LERLL--------------------------DPSWNH---
-RVYRL----ALTK-------LSPPVI-PFMP----------------------------
LLLKDMTFIHEGNH--TL-VE-----------------------------N-LINFE-KM
-------RM---MARAVRMLHHCRSHST------------------------VPLSPLRS
RVSHFHEDSQASRISTCSEQSLSTR-------S--------------PASTWA-----YV
QQLKVIDNQREL---SRLSREL
-----------EP---

>Rapgef3_[Jaculus_jaculus]_507569974

---------------------M--------------------------------------

---------------K---------------------------------VGWPGES----
-----GWQVGPAVED-SQALEAPQVGG----LPDVVPEGTLLNMVLKRMHLPRSCSYQ-L
LLE------------------------HRRP-S---------RIQ---------------
------------------GLR-------------------WT------------------
-----PLTNSEESLDFSM-SLEQA-------------------S--------T-------
-------ERVL-------------RA---------------------GKHLH-RQLLATC
PT-L----------------IRDRKYH------L-RLY-----------------RQC-CS
GR-----------------ELVDGILALG------------------------------L
----GVH--------SRSQAVGIC---QVLLDEGALC-----------------------
--------------H------------------------VKH-DWTFQDRDAQFYRFPGP
-------APEP--AGAHEA---

---------EEEL------AEAMALLSQRGPDAL-------------LTVALRKPPGQRT
DEELD-----LIFEELLHIKAVAHLS-NSVKRELA-AVLLFEPHSKAGTVL---------
--FSQGDKG--TSWYIIW
KGSVNVVTHGK-----GL-------------------------------VTTLHEG-DDF
GQLAL-----------VNDAPRA---ATIILREDNCHFLRVDKQDFNR------IIKD--
---V--------------

------------------------EAKTMRLEEHGKVVLVLER-------S------SQG

AG-----SSHP------------------PAPGRN------------R-YTVMSGTPEKI
LELLLEA------------MRPDSSAHDPT-ETF-------------------LS---D-
FLLTHSV--FM---------------PSAQLCTALL-----HHF------H---------
-----------------------------VEPAEPTGGSEQECSTYICNKRQQILRLVSR
WVALYGPMLHSD-PVATSFLQ----KLSDL--VSRDT--RLSNLLR---EQ-WPEKRR-H
HRL----ENGCGNA--SP------QIKAR----------------------NAP------
-----VW-----------LSSPD--------EP---------------------------
-----------------L--
---PSSI---

----RAIRAGDK--VP-----------------------------YD-------------
ICRPDHSILTLHLPVTASVREVMA--------ALACEDC-----WT-------KGQ--VL
VKVNSA----------------------G------DAIGLQPDARGVATSLGLNERLFVV
DPQE---V-HELT-PHPEQL---------------------------GP------TVGSA
EV--LDLVSAKDLAGQLTDHDWNLFNSIHQVELIH-------YVLGPQHL-RDVTTANLE
RFMRR---------FNELQYWV-----ATELCLCPVPGPR------AQLLRKFIKLAAHL
---------------KEQKNLNSFFAVMF-GLSNSAISRLAHTWE---------------
-------RLPHKVRKLYSA-LERLL--------------------------DPSWNH---
-RVYRL----ALTK-------LSPPVI-PFMP----------------------------
LLLKDMTFIHEGNH--TL-VE-----------------------------N-LINFE-KM
-------RM---MARAVRTLHHCRSHSS------------------------VPLSPLRS
RVSHLHEDSQASRISTCSEQSLGTR-------S--------------PASTWA-----YV
QQLKVIDNQREL---SRLSREL
-----------EP---

>Rapgef3_[Kryptolebias_marmoratus]_1041103289

------------MEN--------------------------------LVKLICSPTRR-C
T----PE-----------------MGE---------------------------------
---T------------------
-----PLPEALNS---KD-TMKQ---------------------------FLS-------
-------DRIV-------------KA---------------------ARSVY-SVMIEKN
PG-L----------------IRDRKHH------L-KTY----------------RQC-CS
GK-----------------ELVDWLMKQS-----------------------------E
----CLQ--------SRSQGVGMW---QVLVDEGILV-----------------------
--------------H------------------------VKH-ELNFHDKDSQFYRFQDS
-------EFGLNHMTNEK--
DM--

---------EDEL------QEALSLLCQLGPDAL-------------LTMILRKSPSQRS
AEDIE-----VIYEELLHVKAAAHLS-SSVRKELA-AVLVFESHTKAGTVL---------
--FSQGDKG--TSWYIIW
KGSVNVITHGK-----GL-------------------------------VTTLHEG-EDF
GQLAL-----------LNDAPRA---ATIILREDNCHFLRVDKQDFIR------ILKD--
---V--------------

------------------------EANTVRLEEHGKTVLVLEK-------T---------
AD----SAEQG-------------------AGANS------------K-YTVMSGTPEKI
LEHLLET------------IKLDSNGNDPI-DP-------------------CVN---D-
FLLTHKV--FM---------------SSSQLCAALL-----HHY------Q---------
-----------------------------AELSE---GSEQEKDAYVLNTKQKVVKLIGQ
WVALYSLLLKED-PVTLDFLE----RMKKE--VAADG--RLSSILK---EQ-FKERRR--
TKV---LENGYQSL--S-------R-N-QQFD---------------------------
------W-----------FSHCE--------EP---------------------------

---AGRV---

----LPIRAQDK--VL-----------------------------YE-------------
IYKSDNKALSLMLPVNSSVQEVMS--------AIVKPG---------------GDH--VL
VKMNSS----------------------G------ERAQLKLDASGVYTALGLNERLFIC
TSSE---V-EQLR-PLNEQQ---------------------------GP------ERGTA
DL--IEQMSSKDVAIELTNYDWELFTAMHEVELVH-------YIFGRHKF-PGAITANLE
RFVRH---------FNEVQYWV-----VTELCLCEDLVKR------ANLLKKFIKIASVL
---------------KDQKNLNLFFAVMF-GLGNSAVQRLYKTWE---------------
-------RIPSKTKRVYCT-YERMM--------------------------DPSRNH---
-RAYRL----AVTK-------LSPPYI-PFMP----------------------------
LLLKDMTFIHEGNP--DC-LD-----------------------------K-LVNFE-KM
-------RM---LAKTVKIVRECRSQPY------------------------VPSSPQRG
LADRIFLEGP-TRLSTSSEHAFPLR-------T--------------PSNIRH-----YI
QNLKVIDNQRKL---TQLSRTM
-----------ES---

>Rapgef3_[Larimichthys_crocea]_734606459

--MKRGSQQAEES---
--CA----SECDRDT--------------------------------VQAIFTMENVV-K
SICPAPR-----------------RCTPE-------------------------------

```
-------------------MG-------------------ET------------------
-----PLPEALNS---KD-TMKQ---------------------------FLS-------
-------DRVV-------------KA---------------------ARSVY-SVMIERN
PG-M----------------IRDRKHH------L-KTY----------------RQC-CS
GK-----------------ELVDWLMKQN------------------------------E
----CLQ--------SRSQAVGMW---QVLVDEGILV-----------------------
--------------H------------------------VKH-ELNFQDKDTQFYRFQDS
-------ECGPNHTTNEK------------------------------------------
DS----------------------------------------------------------
---------EDEL------QEGLSLLSQLGPDAL-------------LTMILRKCPSQRS
AEDLE-----VIYEELLHVKAAAHLS-TSVRKELA-AVLVFESHAKAGTVL---------
--------------------------------------------FSQGDKG--TSWYIIW
KGSVNVITHGK-----GL-------------------------------VTTLHEG-EDF
GQLAL-----------LNDAPRA---ATIILREDNCHFLRVDKQDFIR------ILKD--
---------------------------------------------V--------------
------------------------------------------------------------
------------------------------------------------------------
------------------------EANTVRLQEHGKTVLVLEK-------S---------
SD----WAEQG------------------GAGCNS------------K-YTVMSGTPEKI
LEHLLET------------IKLDSNGNDAI-DP-------------------CVS---D-
FLLTHKV--FM---------------PSSQLCEALQ-----HQYPAMCTYQ---------
-----------------------------AELSE---GSDQEKASYILTTKQKVVKLVGQ
WVVLYGLLLKED-PIALNFLE----RLKKE--VAGDF--RLSSVLK---E--FRERRR--
TRV---SENGYQSL--S-------R-N-QQFD----------------------------
------W-----------FSNCE--------EP---------------------------
------------------------------------------------------------
---VGRI-----------------------------------------------------
------------------------------------------------------------
------------------------------------------------------------
------------------------------------------------------------
------------------------------------------------------------
----QPIRAQDK--ML-----------------------------YE-------------
IYRPDNKPLTLMLPVNTSVQDVMS--------AIVKPG---------------GDH--IL
VKMNSA----------------------G------ERAQLKLDATAVYTALGLNEKLFIC
TSSQ---V-EQLM-PLKEQQ---------------------------GP------ERATT
DI--LEQMSSKDIANELTNYDWELFTAMHEVELVY-------YVFGRHKF-SGVITANLE
RFVRR---------FNEVQHWV-----VTELCLCEDLVKR------AILLKKFIKIAAVL
---------------KEQKNLNSFFAVMF-GLSNSAVHRLYKTWE---------------
-------RIPSKTKRIYCA-YERLM--------------------------DPSRNH---
-RAYRL----AVAK-------LSPPYI-PFMP----------------------------
LLLKDMTFINEGNP--NY-VE-----------------------------K-LVNFE-KL
-------RM---IAKTVKIVRGCRSQPY------------------------VPSSPQRG
LADRMFLEGPTNRISTYSDNGLPLR-------N--------------PSNIRH-----YI
QNLKVIDNQRKL-----------------------------------------TQLSRTT
-----------EC-----------------------------------------------
------------------------------------------------------------
------------------------------------------------------------
------------------------------------------------------------
------------------------------------------------------------
------------------------------------------------------------
------------------------------------------------------------
------------------------------------------------------------
------------------------------------------------------------
------------------------------------------------------------
```


>Rapgef3_[Latimeria_chalumnae]_556946159

---M
ET--LQPISSKDIANQLTDPDWDLFNCIHEVELVY-------YILGRHVF-GNITTANLE
RFVCR---------FNEIQFWV-----VTEIVLCSDLNKR------VTLLKKFIKVATI-
--------------LKEQKNLNSFFAIMF-GLSNSAVSRLSKTWE---------------
-------RLPNKTRKLYSG-FERLM--------------------------DPSKNH---
-RAYRL----AITK-------LSPPII-PFIP----------------------------
LLLKDMTFVHDGNK--TY-LE-----------------------------N-LVNFE-KM
-------RM---IATTVRIIQHCRS--Q-------------------------PYFSE--
-Q------------------SQSIKNL-----V-SIQA-----------YFQ--------
-QLKVIDQQKTL---TQHSREI
-----------EP---

>Rapgef3_[Lepisosteus_oculatus]_972964887

--MEDSEHHANP-T
PTPG----------------------TSADQ--ELPEKPD--CAS---------------
---T------------------
-----PLPEELDA---QD-TMRQ---------------------------FLF-------
-------QRVL-------------KA---------------------GRALY-AALIERS
PG-L----------------IRDRKYH------L-KTY----------------RRC-CT
GK-----------------ELVDWLMKVN------------------------------G
----SIQ--------SRSQAVGMW---QVLVDEGILV-----------------------
--------------H------------------------AKQ-ELNFQDKDAQFYHFLEA
-------EFGADHTATEK--
DS--
---------EDEL------QEAMALLAQMGPDAL-------------LTMILRKPPSQRG
PEDLE-----VIFEELLHIKAVAHLS-SSVRRELA-AVLVFESHAKAGTLL---------
--FSQGDKG--TSWYIIW
KGSVNVITHGK-----GL-------------------------------VTTLHEG-DDF
GQLAL-----------LNDAPRA---ATIIILREDNCHFLRVDKQDFIH-----ILKD--
---V--------------

------------------------EANTVRLEEHGKVVLVLEK-------S------SQS
EQ-----SSQG------------------GSANSN------------K-YTVMSGTPEKI
LEHLLET------------VRLDSNPSELP-DS-------------------CVT---D-
FLLTHCV--FM---------------PSSQLCPVLL-----HYY------H---------
-----------------------------TEPSE---GSELEKAAYSLNKKQKIIRLLYQ
WVGLYGKLLKED-PTAVEFLE----SLKGA--VVRDS--RLTVMLK---EQQLPDRRK--
TRA---LENGYSAA--S-------QSKANEFD----------------------------
------W-----------FSGQE--------EC---------------------------

---VRKC---

----RPIRSQDK--VL-----------------------------YE-------------
IFRPDRTPVSLMLPVDTSVQDVIS--------ALVNPG---------------GNH--VL
VKINSS----------------------G------ERAQLKLDTTAVFTSLGVNERLFLC
TANQ---V-DQLI-PLKEQQ---------------------------GP------DQGTL
DS--LEQMSSKDIACQLSDYDWELFGAMHEVELVY-------YIFGRHKF-RGATTANLE

RFVRR---------FNEVQHWV-----ATELCLCPDLGKR------ALLLKKFIKVATVL
---------------KEQKNLNSFFAVMF-GLSNSAVQRLSRTWE---------------
-------RLPSKTRRIYCA-YERLM--------------------------DPSRNH---
-RAYRL----AVAK-------LSSPYI-PFMP----------------------------
LLLKDMTFIHEGNK--NH-IE-----------------------------G-LVNFE-KM
-------RM---IAKTVKIVRECRSQPY------------------------VPASPQKG
LTDRVFLETTAVRVSTCSEQSVTLR-------S--------------PALVRL-----YI
QNLRVIDNQRRL---TQLSRDL
-----------EH---

>Rapgef3_[Leptonychotes_weddellii]_585154006

---------------------M--------------------------------------

---------------K---------------------------------VGWPGES----
-----PWRVGLAVDG-SPALGAAQVGG----LPDVVPEGTLLNMVLKRMHRPRSCSYQ-L
LLE------------------------HQRP-G---------RIQ---------------
------------------GLR-------------------WT------------------
-----PLTDSEESLDFSV-SLEQA-------------------S--------T-------
-------ERVL-------------RA---------------------GKQLH-RQLLATC
PT-L----------------IRDRKYH------L-RLY----------------RQC-CS
GR-----------------ELVDGILALG------------------------------L
----GVH--------SRSQAVGIC---QVLLDEGALC-----------------------
--------------H------------------------VKH-DWTFQDRDTQFYRFPGP
-------EPET--VGIHEL---

---------EEEL------VEAVALLSQRGPDAL-------------LTVALRKPPGQRT
DEELD-----LIFEELLHIKAVAHLS-NSVKRELA-AVLLFEPHSKAGTVL---------
--FSQGDKG--TSWYIIW
KGSVNVVTHGK-----GL-------------------------------VTTLHEG-DDF
GQLAL-----------VNDAPRA---ATIILREDNCHFLRVDKQDFNR------IIKD--
---V--------------

------------------------EAKTMRLEEHGKVVLVLER-------A------SQG
AG-----PSRP------------------PTPGRN------------R-YTVMSGTPEKI
LELLLEA------------MRPDSSAHDPT-ETF-------------------LS---D-
FLLTYSV--FM---------------PSAQLCAALL-----HHF------H---------
-----------------------------AEPS---GGSEQEHSTYICNKRQQILRLVSQ
WVALYGPMLHMD-PVATSFLQ----KLSDL--VSRDA--RLCLLLR---EQ-WPERRR-H
HRL----ENGCGNA--SP------QMKAR----------------------NVP------
-----VW-----------LPGQD--------QP---------------------------
-----------------L--
---PSSN---

----CAIRVGDK--VP-----------------------------YD-------------
ICRPDHSVLTLKLPVTASVREVMA--------ALAQEDG-----WT-------KGQ--VL
VKVNST----------------------G------HAIGLQPEARGVATSLGLNERLFVV
NPQE---V-HELT-PHPEQL---------------------------GP------TVGSA
EG--LDLVSAKDLAGQLTDHDWSLFNSIHQVELIH-------YVLGPQHL-RDVTTANLE
RFMRR---------FNELQYWV-----ATELCLCPVPSLR------AQLLRKFIKLAAHL
---------------KEQKNLNSFFAIMF-GLSNSAISRLAHTWE---------------
-------RLPHKVRKLYSA-LERLL--------------------------DPSWNH---
-RVYRL----LLTK-------LSPPII-PFMP----------------------------
LLLK--

>Rapgef3_[Lipotes_vexillifer]_602707263

---------------------M--------------------------------------

---------------K---------------------------------VSWPGES----
-----RWQVGLAVED-SSVLGAPPVGG----LPDVVPEGTLLSMVLRRMHRSRSCSYQ-L
LLE------------------------HQRP-S---------CIQ---------------
------------------GLR-------------------WT------------------
-----PLANSEESLDFSV-SLEQA-------------------S--------T-------
-------ERVL-------------RA---------------------GKQLH-RHLLATC
PN-L----------------IRDRKYH------L-RLH----------------RQC-CS
GR-----------------ELVDGILALG------------------------------L
----GVH--------SRSQAVGIC---QVLLDEGALC-----------------------
--------------H------------------------VKH-DWAFQDRDTQFYRFPGP
-------EPEP--AGIHEL---

---------EEEF------LEALALLSQRGPDAL-------------LTVALRKPPGQRT
DEELD-----LIFEELLHIKAVAHLS-NSVKRELA-AVLLFEPHSKAGTVL---------
--FSQGDKG--TSWYIIW
KGSVNVVTHGK-----GL-------------------------------VTTVHEG-DDF
GQLAL-----------VNDAPRA---ATIILREDNCHFLRVDKQDFNR------IIKD--
---V--------------

------------------------EAKTMRLEEHGKVVLVLER-------T------SQG

TG-----PSRP------------------PTPGRN------------R-YIVMSGTPEKI
LELLLEA------------MRPDSSAHDPT-ETF-------------------LS---D-
FLLTHSV--FM---------------PTAQLCAALL-----HHF------H---------
-----------------------------AEPV---GGSEQECSTYICNKRQQILRLVSQ
WVALYGPMLHTD-PVATSFLQ----KLSDL--VSRDA--RLSNLLR---EQ-WPERRR-H
HRL----ENGCGNA--SP------QMKAW----------------------NVP------
-----VW-----------LPSQD--------EP---------------------------
-----------------L--
---PSSN---

----CAIRVGDK--VP-----------------------------YD-------------
ICRPDHSVLTLQLPVTASVREVMA--------ALAQEDG-----WT-------KGQ--VL
VKVNSA----------------------G------DTVGLQPDAHGVATSLGLNERLFVV
NPQE---V-HKLT-PHPEQL--------------------------GP------TVGSA
EG--LDLVSTKDLAGQLTDHDWGLFNSIHQVELIH-------YVLGPQPL-RDVTTANLE
RFMRR---------FNELQYWV-----ATELCLCPVPGLR------AQLLRKFIKLAAHL
---------------KEQKNLNSFFAIMF-GLSNSAISRLAQTWE---------------
-------RLPHKVRKLYSA-LERLL--------------------------DPSWNH---
-RVYRL----ALTK-------LSPPLI-PFMP----------------------------
LLLKDMTFIHEGNH--TL-VE-----------------------------N-LINFE-KM
-------RM---MARAARMLHHCRSHSN------------------------VPLSPLRS
RVSHLHEDSQAARISTCSEQSLSTR-------S--------------PASTWA-----YV
QQLKVIDNQREL---SRLSREL
-----------EP---

>Rapgef3_[Loxodonta_africana]_731457673

---MVLRRMHRPRSCSYQ-L
LLE------------------------HRSP-S---------CIQ---------------
------------------GLR-------------------WT------------------
-----PLTDSEESLDFSV-SLEQA-------------------S--------T-------
-------ERVL-------------RA---------------------GKQLH-RHLLATC
PN-L----------------IRDRKYH------L-RLY----------------RQC-CS
GR-----------------ELVDGILALG------------------------------L
----GVH--------SRSQAVGVC---QVLLDEGALS-----------------------
--------------H------------------------VKH-DWTFQDRDAQFYRFPGL
-------EPEP--VGAHEM---

```
---------EEEL------VEAVSLLSQRGPDAL-------------LTVALRKPPGQRT
DEELD-----LVIEEVLNIEARGHLF-NSVKRELA-AVLLFEPHCKAGTVL---------
--------------------------------------------FSQGDKG--TSWYIIW
KGSVNVVTHGK-----CV------------------------------------------
---------------------------------DHWVWL---------------VLQD--
---------------------------------------------V--------------
------------------------------------------------------------
------------------------------------------------------------
------------------------EAKTMRLEEHGKVVLVLER-------T------SQG
AG-----PSCP------------------PTPGRN------------R-YTVMSGTPEKI
LELLLEA------------MRPDSSAHDPT-ETF-------------------LS---D-
FLLTHSV--FM---------------PSTQLCAALL-----HHF------H---------
-----------------------------AEPA---GGSEQERSTYVCNKRQQILRLVSQ
WVALYGPVLRTD-PVATSFLQ----KLSDL--VSQDA--RLSNLLK---EQ-WPERRR-H
HRL----ENGYGNA--SP------QMKAR----------------------NMA------
-----VW-----------LAGQD--------EP---------------------------
-----------------L------------------------------------------
---PNSN-----------------------------------------------------
------------------------------------------------------------
------------------------------------------------------------
------------------------------------------------------------
------------------------------------------------------------
----CAIRAGDK--VP-----------------------------YD-------------
ICRPDHSVLTLQLPVTASVREVMA--------ALAQEDS-----WT-------KEQ--VL
VKVNSA----------------------G------DAIGLQPDARGVATSLGLNERLFVV
NPQE---V-HKLT-PHPEQL---------------------------GP------TVGSA
EG--LDLVSAKDLAGQLTDHDWSLFNSIHQVELIH-------YVLGPQHL-RTVTTANLE
RFMRR---------FNELQYWV-----ATELCLCPVPGLR------AQLLRKFIKLAAHL
---------------KEQKNLNSFFAVMF-GLSNSAVSRLAHTWE---------------
-------RLPHKVRKLYSA-LERLL--------------------------DPSWNH---
-RVYRL----ALTK-------LSPPVI-PFMP----------------------------
LLLKDMTFIHEGNH--TL-VE-----------------------------N-LINFE-KM
-------RM---MARAVRMLHHCRGHSN------------------------VPLSPLRS
RVSHLHEDSQASRISTCSEQSLSTR-------S--------------PASTWA-----YV
QQLKVIDNQREL-----------------------------------------SRLSREM
-----------EP-----------------------------------------------
------------------------------------------------------------
------------------------------------------------------------
------------------------------------------------------------
------------------------------------------------------------
------------------------------------------------------------
------------------------------------------------------------
------------------------------------------------------------
------------------------------------------------------------
------------------------------------------------------------
------------------------------------------------------------
---
>Rapgef3_[Macaca_fascicularis]_544469453
------------------------------------------------------------
---------------------M--------------------------------------
------------------------------------------------------------
---------------K---------------------------------VGWPGES----
-----CWQVGLAVED-SPALGAPRVGP----LPDVVPEGTLLNMVLRKMHRPRSCSYQ-L
LLE------------------------HQPP-S---------CIQ---------------
```

```
------------------GLR-------------------WT------------------
-----PLTNSEESLDFSE-SLEQA-------------------S--------T-------
-------ERVF-------------RA---------------------GRQLH-RQLLATC
PN-L----------------IRDRKYH------L-RLY----------------RQC-CS
GR-----------------ELVDGILALG------------------------------L
----GVH--------SRSQVVGIC---QVLLDEGALC-----------------------
--------------H------------------------VKH-DWAFQDRDAQFYRFPGP
-------EPEP--VGAHEM-----------------------------------------
------------------------------------------------------------
---------EEEL------AEAVALLSQRGPDAL-------------LTVALRKPPGQRT
DEELD-----LIFEELLHIKAVAHLS-NSVKRELA-AVLLFEPHSKAGTVL---------
--------------------------------------------FSQGDKG--TSWYIIW
KGSVNVVTHGK-----GL-------------------------------VTTLHEG-DDF
GQLAL-----------VNDAPRA---ATIILREDNCHFLRVDKQDFNR------IIKD--
---------------------------------------------V--------------
------------------------------------------------------------
------------------------------------------------------------
------------------------EAKTMRLEEHGKVVLVLER-------A------SQG
AG-----PSRP------------------PTPGRN------------R-YTVMSGTPEKI
LELLLEA------------MGPDSSAHDPT-ETF-------------------LS---D-
FLLTHRV--FM---------------PSAQLCAALL-----HHF------H---------
-----------------------------AEPA---GGSEQERSTYICNKRQQILRLVSQ
WVALYGSMLHTD-PVATSFLQ----KLSDL--VGRDA--RLSNLLR---EQ-WPERRR-H
HRL----ENGCGNA--SP------QMKAR----------------------NLP------
-----VW-----------LPNQD--------EP---------------------------
-----------------L------------------------------------------
---PGSS-----------------------------------------------------
------------------------------------------------------------
------------------------------------------------------------
------------------------------------------------------------
------------------------------------------------------------
----CAIRVGDK--VP-----------------------------YD-------------
ICLPDHSVLTLQLPVTASVREVMA--------ALAQEDG-----WT-------KEQ--VL
VKVNSA----------------------G------DAIGLQPDARGVATSLGLNERLFVV
NPQE---V-HELT-PHPDQL---------------------------GP------TVGSA
EG--LDLVSAKDLAGQLTDHDWSLFNSIHQVELIY-------YVLGPQHL-RDVTTANLE
RFMRR---------FNELQYWV-----ATELCLCPVPGPR------AQLLRKFIKLAAHL
---------------KEQKNLNSFFAVMF-GLSNSAISRLAHTWE---------------
-------RLPHKVRKLYSA-LERLL--------------------------DPSWNH---
-RVYRL----ALAK-------LSPPVI-PFMP----------------------------
LLLKDMTFIHEGNH--TL-VE-----------------------------N-LINFE-KM
-------RM---MARAARMLHHCRSHNP------------------------VPLSPLRS
RVSHLHEDSQVARISTCSEQSLSTR-------S--------------PASTWA-----YA
QQLKVIDNQREL-----------------------------------------SRLSREL
-----------EP-----------------------------------------------
------------------------------------------------------------
------------------------------------------------------------
------------------------------------------------------------
------------------------------------------------------------
------------------------------------------------------------
------------------------------------------------------------
------------------------------------------------------------
------------------------------------------------------------
------------------------------------------------------------
```

```
------------------------------------------------------------
---
>Rapgef3_[Macaca_mulatta]_966971988
------------------------------------------------------------
---------------------M--------------------------------------
------------------------------------------------------------
---------------K---------------------------------VGWPGES----
-----CWQVGLAVED-SPALGAPRVGP----LPDVVPEGTLLNMVLRKMHRPRSCSYQ-L
LLE------------------------HQPP-S---------CIQ---------------
------------------GLR-------------------WT------------------
-----PLTNSEESLDFSE-SLEQA-------------------S--------T-------
-------ERVF-------------RA---------------------GRQLH-RQLLATC
PN-L----------------IRDRKYH------L-RLY----------------RQC-CS
GR-----------------ELVDGILALG------------------------------L
----GVH--------SRSQVVGIC---QLLDEGALC------------------------
--------------H------------------------VKH-DWAFQDRDAQFYRFPGP
-------EPEP--VGAHEM-----------------------------------------
------------------------------------------------------------
---------EEEL------AEAVALLSQRGPDAL-------------LTVALRKPPGQRT
DEELD-----LIFEELLHIKAVAHLS-NSVKRELA-AVLLFEPHSKAGTVL---------
--------------------------------------------FSQGDKG--TSWYIIW
KGSVNVVTHGK-----GL-------------------------------VTTLHEG-DDF
GQLAL-----------VNDAPRA---ATIILREDNCHFLRVDKQDFNR------IIKD--
---------------------------------------------V--------------
------------------------------------------------------------
------------------------------------------------------------
------------------------EAKTMRLEEHGKVVLVLER-------A------SQG
AG-----PSRP------------------PTPGRN------------R-YTVMSGTPEKI
LELLLEA------------MGPDSSAHDPT-ETF-------------------LS---D-
FLLTHRV--FM---------------PSAQLCAALL-----HHF------H---------
-----------------------------AEPA---GGSEQERSTYICNKRQQILRLVSQ
WVALYGSMLHTD-PVATSFLQ----KLSDL--VGRDA--RLSNLLR---EQ-WPERRR-H
HRL----ENGCGNA--SP------QMKAR----------------------NLP------
-----VW-----------LPNQD--------EP---------------------------
-----------------L------------------------------------------
---PGSS-----------------------------------------------------
------------------------------------------------------------
------------------------------------------------------------
------------------------------------------------------------
------------------------------------------------------------
----CAIRVGDK--VP-----------------------------YD-------------
ICLPDHSVLTLQLPVTASVREVMA--------ALAQEDG-----WT-------KEQ--VL
VKVNSA----------------------G------DAIGLQPDARGVATSLGLNERLFVV
NPQE---V-HELT-PHPDQL---------------------------GP------TVGSA
EG--LDLVSAKDLAGQLTDHDWSLFNSIHQVELIY-------YVLGPQHL-RDVTTANLE
RFMRR---------FNELQYWV-----ATELCLCPVPGPR------AQLLRKFIKLAAHL
---------------KEQKNLNSFFAVMF-GLSNSAISRLAHTWE---------------
-------RLPHKVRKLYSA-LERLL--------------------------DPSWNH---
-RVYRL----ALAK-------LSPPVI-PFMP----------------------------
LLLKDMTFIHEGNH--TL-VE-----------------------------N-LINFE-KM
-------RM---MARAARMLHHCRSHNP------------------------VPLSPLRS
RVSHLHEDSQVARISTCSEQSLSTR-------S--------------PASTWA-----YV
QQLKVIDNQREL-----------------------------------------SRLSREL
-----------EP-----------------------------------------------
```


>Rapgef3_[Macaca_nemestrina]_795561749

---------------------M--------------------------------------

---------------K---------------------------------VGWPGES----
-----CWQVGLAVED-SPALGAPRVGP----LPDVVPEGTLLNMVLRKMHRPRSCSYQ-L
LLE------------------------HQRP-S---------CIQ---------------
------------------GLR-------------------WT------------------
-----PLTNSEESLDFSE-SLEQA-------------------S--------T-------
-------ERVL-------------RA---------------------GRQLH-RQLLATC
PN-L----------------IRDRKYH------L-RLY----------------RQC-CS
GR-----------------ELVDGILALG------------------------------L
----GVH--------SRSQVVGIC---QVLLDEGALC-----------------------
--------------H------------------------VKH-DWAFQDRDAQFYRFPGP
-------EPEP--VGAHEM---

---------EEEL------AEAVALLSQRGPDAL-------------LTVALRKPPGQRT
DEELD-----LIFEELLHIKAVAHLS-NSVKRELA-AVLLFEPHSKAGTVL---------
--FSQGDKG--TSWYIIW
KGSVNVVTHGK-----GL-------------------------------VTTLHEG-DDF
GQLAL-----------VNDAPRA---ATIILREDNCHFLRVDKQDFNR------IIKD--
---V--------------

------------------------EAKTMRLEEHGKVVLVLER-------A------SQG
AG-----PSRP------------------PTPGRN------------R-YTVMSGTPEKI
LELLLEA------------MGPDSSAHDPT-ETF-------------------LS---D-
FLLTHRV--FM---------------PSAQLCAALL-----HHF-------H--------
-----------------------------AEPA---GGSEQERSTYICNKRQQILRLVSQ
WVALYGSMLHTD-PVATSFLQ----KLSDL--VGRDA--RLSNLLR---EQ-WPERRR-H
HRL----ENGCGNA--SP------QMKAR----------------------NLP------
-----VW-----------LPNQD--------EP---------------------------
-----------------L--
---PGSS---

----CAIRVGDK--VP-----------------------------YD-------------
ICLPDHSVLTLQLPVTASVREVMA--------ALAQEDG-----WT-------KEQ--VL
VKVNSA----------------------G------DAIGLQPDARGVATSLGLNERLFVV
NPQE---V-HELT-PHPDQL---------------------------GP------TVGSA
EG--LDLVSAKDLAGQLTDHDWSLFNGIHQVELIY-------YVLGPQHL-RDVTTANLE

RFMRR---------FNELQYWV-----ATELCLCPVPGPR------AQLLRKFIKLAAHL
---------------KEQKNLNSFFAVMF-GLSNSAISRLAHTWE---------------
-------RLPHKVRKLYSA-LERLL--------------------------DPSWNH---
-RVYRL----ALAK-------LSPPVI-PFMP----------------------------
LLLKDMTFIHEGNH--TL-VE-----------------------------N-LINFE-KM
-------RM---MARAARMLHHCRSHNP------------------------VPLSPLRS
RVSHLHEDSQVARISTCSEQSLESRG------S--------------PAAPGP-----YV
RQLKVIDNQRDS---PGLSREL
-----------EPWEEGAGTKAGGRALVGLETRACGPKYPQACATAGAQASVGVDVESLE
QAAVGGSVPCVTGG--

>Rapgef3_[Mandrillus_leucophaeus]_795173402

---MVLRKMHRPRSCSYQ-L
LLE------------------------HQRP-S---------CIQ---------------
------------------GLR-------------------WT------------------
-----PLTNSEESLDFSE-SLEQA-------------------S--------T-------
-------ERVL-------------RA---------------------GRQLH-RQLLATC
PN-L----------------IRDRKYH------L-RLY----------------RQC-CS
GR-----------------ELVDGILALG------------------------------L
----GVH--------SRSQVVGIC---QVLLDEGALC-----------------------
--------------H------------------------VKH-DWAFQDRDAQFYRFPGP
-------EPEPEPVGAHEM---

---------EEEL------AEAVALLSQRGPDAL-------------LTVALRKPPGQRT
DEELD-----LIFEELLHIKAVAHLS-NSVKRELA-AVLLFEPHSKAGTVL---------
--FSQGDKG--TSWYIIW
KGSVNVVTHGK-----GL-------------------------------VTTLHEG-DDF
GQLAL-----------VNDAPRA---ATIILREDNCHFLRVDKQDFNR------IIKD--
---V--------------

------------------------EAKTMRLEEHGKVVLVLER-------A------SQG
AG-----PSRP------------------PTPGRN------------R-YTVMSGTPEKI
LELLLEA------------MGPDSSAHDPT-ETF-------------------LS---D-
FLLTHRV--FM---------------PSAQLCAALL-----HHF------H---------
-----------------------------AEPA---GGSEQERSTYICNKRQQILRLVSQ
WVALYGSMLHTD-PVATSFLQ----KLSDL--VGRDA--RLSNLLR---EQ-WPERRR-H
HRL----ENGCGNA--SP------QMKAR----------------------NLP------
-----VW-----------LPNQD--------EP---------------------------
-----------------L--
---PGSS---

----CAIRVGDK--VP-----------------------------YD-------------
ICLPDHSVLTLQLPVTASVREVMA--------ALAQEDG-----WT-------KGQ--VL
VKVNSA----------------------G------DAIGLQPDARGVATSLGLNERLFVV
NPQE---V-HELT-PHPDQL---------------------------GP------TVGSA
EG--LDLVSAKDLAGQLTDHDWSLFNSIHQVELIY-------YALGPQHL-RDVTTANLE
RFMRR---------FNELQYWV-----ATELCLCPVPGPR------AQLLRKFIKLAAHL
---------------KEQKNLNSFFAVMF-GLSNSAISRLAHTWE---------------
-------RLPHKVRKLYSA-LERLL--------------------------DPSWNH---
-RVYRL----ALAK-------LSPPVI-PFMP----------------------------
LLLKDMTFIHEGNH--TL-VE-----------------------------N-LINFE-KM
-------RM---MARAARMLHHCRSHNP------------------------VPLSPLRS
RVSHLHEDSQVARISTCSEQSLSTR-------S--------------PASTWA-----YV
QQLKVIDNQREL---SRLSREL
-----------EP---

>Rapgef3_[Marmota_marmota_marmota]_984114365

---------------------M--------------------------------------

---------------K---------------------------------VGLPGES----
-----RWQVGPAVED-NLALGAPQVGG----LPDLVPEGTLLNMVLKRMHRPRSCSYQ-L
LLE------------------------HRRP-S---------RIQ---------------
------------------GLR-------------------WT------------------
-----PLTNSEESLDFSV-SLEQA-------------------S--------T-------
-------ERVL-------------RA---------------------GKHLH-RHLLATC
PT-L----------------IRDRKYH------L-RLY----------------RQC-CS
GR-----------------ELVDWVLALG------------------------------L
----GVH--------SRSQAVGIC---QVLLDEGALC-----------------------
--------------H------------------------VKH-DWTFQDRDAQFYRFPGP
-------EPEP--TGAHEM---

---------EEEL------VEAMALLSQRGPDAL-------------LTVALRKPPGQRT
DEELD-----LIFEELLHIKAVAHLS-NSVKRELA-AVLLFEPHSKAGTVL---------
--FSQGDKG--TSWYIIW
KGSVNVVTHGK-----GL-------------------------------VTTLHEG-DDF
GQLAL-----------VNDAPRA---ATIILREDNCHFLRVDKQDFNR------IVKD--
---V--------------

------------------------EAKTMRLEEHGKVVLVLER-------T------SQG

TG-----PSRP------------------PTPGRN------------R-YTVMSGTPEKI
LELLLEA------------MRPDSSAHDPT-ETF-------------------LS---D-
FLLTHSV--FM---------------PSAQLCAALL-----HHF------H---------
-----------------------------VEPAEPAGGSEQERSTYICNKRQQILRLVSQ
WVALYGPMLHTD-PVATNFLQ----KLSDL--VSRDA--RLSNLLR---EQ-WPERRR-H
HRL----ENGCGNA--SP------MMKAR----------------------NMP------
-----AW-----------LPSQE--------EP---------------------------
-----------------L--
---PSSN---

----CAIRAGDK--VP-----------------------------YD-------------
ICRPDHSVLTLQLPVTASVREVMA--------ALAQKDG-----WT-------KGQ--VL
VKVNSA----------------------G------DAIGLQPDARGVATSLGLNERLFVV
DPQE---V-HELT-PHPEQL---------------------------GP------TVGSA
EG--LESVSAKDLAGQLTDHDWNLFNSIHQVELIH-------YVLGPQHL-RDVTTANLE
RFMRR---------FNELQYWV-----ATELCLCPVPGLR------AQLLRKFIKLAAHL
---------------KEQKNLNSFFAIMF-GLSNSAISRLAHTWE---------------
-------RLPHKVRKLYSA-LERLL--------------------------DPSWNH---
-RVYRL----ALTK-------LSPPVI-PFMP----------------------------
LLLKDMTFIHEGNH--TL-VE-----------------------------N-LINFE-KM
-------RM---MARAVRMLHHCRSHST------------------------VPLSPLRS
RVSHFHEDSQASRISTCSEQSLSTR-------S--------------PASTWA-----YV
QQLKVIDNQREL---SRLSREL
-----------EP---

>Rapgef3_[Maylandia_zebra]_499019352

--MHLFRTHNYQ-V
F----PD-----------------RCSVE-K--QTIR-----------------------
------------------GIS-------------------WT------------------
-----PLPEALNS---KD-TMKQ---------------------------FLS-------
-------DRVV-------------KA---------------------ARSVY-SVMIEKN
PG-L----------------IRDRKHH------L-KTY----------------RQC-CS
GK-----------------DLVDWLMTQN------------------------------E
----GLQ--------SRSQAVGIW---QVLVDEGILL-----------------------
--------------H------------------------VKH-ELNFLDKDTQFYRFQDS
-------QFGLNHILNEK--
DS--

---------EDEL------GEALSLLSQLGPDAL-------------LTMILRKCPSQRS
PEDLE-----VIYEELLHVKAAAHLS-SSVRKELA-AVLVFESHAKAGTVL---------
--FSQGDKG--TSWYIIW
KGSVNVITHGK-----GL-------------------------------VTTLHEG-EDF
GQLAL-----------LNEQPRA---ATIILREDNCHFLRVDKQDFIR------ILKD--
---V--------------

------------------------EANTVRLEEHGKTVLVLEK-------S---------
AD----STEQG------------------GAGNSS------------K-YTVMSGTPEKI
LEHLLET------------IKLECNGNDAI-DS-------------------CVS---D-
FLLTHKV--FM---------------PSSQLCPALQ-----HHY------Q---------
-----------------------------AQLAE---GSEQEKTAYVLNIKQKVVKLVGQ
WVALFGLHLKED-PIALDFLE----RLKKD--MAPNS--QLFNVLK---D--FRERRR--
TKV---LENGYQSL--S-------R-N-HQFD----------------------------
------W-----------FSNFE--------ES---------------------------

---VGRV---

----QPIRAHDK--VL-----------------------------YE-------------
IYRPDNKPLTPMLPVNTSVQEIMS--------ALGKAG---------------EDH--VL
IKMNST----------------------G------ERAQLKLDATAVYTALGLNERLFIC
TISE---V-EQLT-PLKEQQ---------------------------GP------EQGSA
DV--LEQMSSKDIASELTNYDWELFTAMHEVELVY-------YIFGRHKF-PGAITANLE
RFVRR---------FNEVQHWV-----LTELCLCEDLVKR------AMLLKKFIKIASVL
---------------KEQKNLNSFFAVMF-GLSNSAIQRLYKTWE---------------
-------RIPSKTKRIYCS-LERLM--------------------------DPSRNH---
-RAYRL----AIAK-------LSPPYI-PFMP----------------------------
LLLKDMTFIHEGNA--NY-VD-----------------------------K-LVNFE-KM
-------RM---LAKTVKIVRGCRSQPY------------------------VPSSPQRG
LADRMFLEGAATRLSTYSDHAFPLR-------T--------------STNIRH-----YI
QNLKVIDNQRKL---TQLSRTI
-----------DC---

>Rapgef3_[Mesitornis_unicolor]_704529374


```
------------------------------------------------------------
------------------------------------------------------------
-----------T---PLLDTESTLDYGHSLTQASPERIWRAGKLLFS----H---LSTTR
PG-L----------------IRDHKHHLR-------HH----------------RQC-CS
GK-----------------ELVDWVLGAG------------------------------L
----AVQ--------TRSQAVGVC---QVLVDGGVLT-----------------------
--------------H------------------------VKQ-EWHFQDKDSQFYRFAEL
E-----LSPEA-GAGPRDA-----------------------------------------
------------------------------------------------------------
----------EEL------LEALAFLAQLGPDAL-------------LTMALRKPPAQRT
EDELE-----LIFEELLHIKAVAHLS-NSVKRELA-SVLMFEPHQRVGTV----------
-------------------------------------------LFSQGDKG--TSWYIIW
KGSVNVVTHGK-----GL-------------------------------VATLHEG-DDF
GQLAL-----------VNDAPRA---ATIILREDNCHFLRVDKQDFNR------ILKD--
---------------------------------------------V--------------
------------------------------------------------------------
------------------------------------------------------------
------------------------EANTMRLKEHGKVVLVLQKNL-------------QG
G-----SSQA-------------------ATAR-----------S-SR-YSVVAGTPEKI
LEHLLEF------------MRLDATLYDPL-D-------------------TLLK---RC
FLLSYTV--FM---------------PTSQLCRALL-----HHF------R---------
-----------------------------AEPLE---GSEQEKATYTLHKRRKILRLVSQ
WVLLYGRLLQGD-RSTTALLQ---------------------------------------
------------------------------------------------------------
------------------------------------------------------------
------------------------------------------------------------
------------------------------------------------------------
------------------------------------------------------------
------------------------------------------------------------
------------------------------------------------------------
------------------------------------------------------------
------------------------------------------------------------
------------------------------------------------------------
------------------------------------------------------------
------------------------------------------------------------
---------------------------------------------------------NLA
DLASQ----------------------------DPRLGALV-------------------
------------------------------------------QEQ---------------
----------GQERRRPRT-VE--------------------------------------
------------------------------------------------------------
------------------------------------------------------------
---------------------NGDG--SV-----------------------SPQPKAQS
SATWLPSHEEATVTSS--------------------------------------------
-----------C-----------------------------------------ALRAQDK
------------------------------------------------------------
------------------------------------------------------------
------------------------------------------------------------
------------------------------------------------------------
------------------------------------------------------------
------------------------------------------------------------
------------------------------------------------------------
------------------------------------------------------------
------------------------------------------------------------
------------------------------------------------------------
```


>Rapgef3_[Mesocricetus_auratus]_524925946

---------------------M--------------------------------------

---------------K---------------------------------VSWPGDN----
-----HWQVGPAVVE-SPAVEAPQVES----LPDVVPEDTLLNMVLKRMHRPRCCSYQ-L
VFE------------------------HRRP-S---------CIQ---------------
------------------GLR-------------------WT------------------
-----PLTNSEESLDFSV-SLEQA-------------------T--------T-------
-------ERVL-------------KA---------------------GRLLH-RHLLATY
PT-L----------------IRDRKYH------L-RLY----------------RQC-CS
GR-----------------ELVDGILALG------------------------------L
----GVH--------SRSQAVGIC---QVLLDEGALC-----------------------
--------------H------------------------VKH-DWTFQDRDAQFYRFPGP
-------EPEP--VGTHDV---

---------EEEL------VEAMALLSQRGPDAL-------------LTVALRKPPGQRT
DEELD-----LIFEELLHIKAVAHLS-NSVKRELA-AVLLFEPHSKAGTVL---------
--FSQGDKG--TSWYIIW
KGSVNVVTHGK-----GL-------------------------------VTTLHEG-DDF
GQLAL-----------VNDAPRA---ATIILRENNCHFLRVDKQDFNR------IIKD--
---V--------------

------------------------EAKTMRLEEHGKVVLVLER-------S------SQG
TG-----PSRP------------------PTPGRN------------R-YTVMSGTPEKI
LELLLEA------------MRPDSSAHDPT-ETF-------------------LS---D-
FLLTHSV--FM---------------PSAQLFTALL-----HHF------H---------
-----------------------------VEPAEPAGGSEQERNTYVCNKRQQILRLVSR
WVALYGPMLHSD-PVATSFLQ----KLSDL--VSRDA--RLSNLLK---EQ-CPERRR-H
HRL----ENGCGNA--SP------QTKAR----------------------NAP------
-----VW-----------LPNQE--------EP---------------------------
-----------------L--
---PSST---

----GAIRVGDK--VP-----------------------------YD-------------
ICRPDHSVLTLQLPVTASVREVMA--------ALAHEDH-----WT-------KGQ--VL
VKVNSA----------------------G------DVVGLQPDARGVATSLGLNERLFVV
DPQE---V-HELT-PHPEQL---------------------------GP------TLGSS
DM--LELVSAKDLAGQLTDHDWNLFNRIHQVELIH-------YVLGPQHL-RDVTTANLE
RFMRR---------FNELQYWV-----ATELCLCSVPGPR------AQLLRKFIKLAAHL
---------------KEQKNLNSFFAVMF-GLSNSAISRLAHTWE---------------
-------RLPHKVRKLYSA-LERLL---------------------------DPSWNH---
-RVYRL----ALTK-------LSPPVI-PFMP----------------------------
LLLKDMTFIHEGNH--TL-VE-----------------------------N-LINFE-KM
-------RM---MARAVRMLHHCRSHST------------------------VPLSPLRS
RVSHIHEDSQASRISTCSEQSLSTR-------S--------------PANTWA-----YV
QQLKVIDNQREL---SRLSREL
-----------EP---

>Rapgef3_[Microcebus_murinus]_829722072

---------------------M--------------------------------------

---------------K---------------------------------VGWPGES----
-----RWQVGLALEE-SPALGASQVGG----LPDVVPEGTLLNMVLRRMYRPRSCSYQ-L
LLE------------------------HRRP-S---------RIQ---------------
------------------GLR-------------------WT------------------
-----PLTSSEESLDFSV-SLEQA-------------------S--------T-------
-------ERVL-------------RA---------------------AKQLH-RHLLATC
PN-L----------------IRDRKYH------L-RLY----------------RQC-CS
GR-----------------ELVDGILALG------------------------------L
----GVH--------SRSQAVGIC---QVLLDEGALY-----------------------
--------------H------------------------VKH-DWTFQDRDAQFYRFPGP
-------EPEP--TGAHEM---

---------EEEL------VEAMALLSQRGPDAL-------------LTVALRKPPGQRT
DEELD-----LIFEELLHVKAVAHLS-NSVKRELA-AVLLFEPHSKAGTVL---------
--FSQGDKG--TSWYIIW
KGSVNVVTHGK-----GL-------------------------------VTTLHEG-DDF
GQLAL-----------VNDAPRA---ATIILREDNCHFLRVDKHDFNR------IIKD--
---V--------------

------------------------EAKTMRLEEHGKVVLVLER-------A------SQG
AG-----TARP------------------PTPGRN------------R-YTVMSGTPEKI
LELLLEA------------MRPDSSAHDPT-ETF-------------------LS---D-
FLLTHSV--FM---------------PSAQLCAALL-----HHF------H---------
-----------------------------AEPA---GGSEQERSTYICNKRQQILRLVSQ
WVALYGSMLHTD-PVATNFLQ----KLSDL--VSRDA--RLSNLLR---EQ-WPERRR-H
HRL----ENGFGNA--SP------QMKAR----------------------NAP------
-----VW-----------LPSQD--------EP---------------------------
-----------------L--
---PSSN---

----CAIRAGDK--VS-----------------------------YD-------------
ICRPDHSVLTLQLPVTASVREVMA--------ALAQEDG-----WT-------KGQ--VL
VKVNSA----------------------G------DATGLQPDARGVATSLGLNERLFVV
NSQE---V-HKLT-PHPEQL---------------------------GP------TVGSA
EG--LDLVSAKDLAGQLTDHDWNLFNSIHQVELIH-------YVLGPQHL-RDVTTANLE

RFMRR---------FNELQYWV-----ATELCLCSVPGLR------AQLLRKFIKLAAHL
---------------KEQKNLNSFFAVMF-GLSNSAISRLALTWQ---------------
-------RLPHKVRKLYSA-LERLL--------------------------DPSWNH---
-RVYRL----ALAK-------LSPPVI-PFMP----------------------------
LLLKDMTFIHEGNH--TL-VE-----------------------------N-LINFE-KM
-------RM---MARAARMLHHCRGHSS------------------------VPLSPLRS
RVSHLHEDGQAARISTCSEQSLSTR-------S--------------PASTWA-----YV
QQLKVIDNQREL---SRLSREL
-----------EP---

>Rapgef3_[Microtus_ochrogaster]_532021349

---------------------M--------------------------------------

---------------K---------------------------------VSWPGEN----
-----HWQVGPAVVE-SPAVGAPQVGS----LPDVVPEGTLLNMVLKRMHRPRCCSYQ-L
VFE------------------------HRRP-S---------CIQ---------------
------------------GLR-------------------WT------------------
-----PLTNSEESLDFSV-SLEQA-------------------T--------T-------
-------ERVL-------------KA---------------------GKLLH-RHLLATY
PT-L----------------IRDRKYH------L-RLY----------------RQC-CS
GR-----------------ELVDVILSLG------------------------------L
----GVH--------SRSQAVGVC---QVLLDEGALY-----------------------
--------------H------------------------VKH-DWTFQDRDAQFYRFPGP
-------EPEP--AGTHDV---

---------EEEL------VEAMALLSQRGPDAL-------------LTVALRKPPGQRT
DEELD-----LIFEELLHIKAVAHLS-NSVKRELA-AVLLFEPHSKAGTVL---------
--FSQGDKG--TSWYIIW
KGSVNVVTHGK-----GL-------------------------------VTTLHEG-DDF
GQLAL-----------VNDAPRA---ATIILRENNCHLGGGRKRSLGHWTPVWLLLQD--
---V--------------

------------------------EAKTMRLEEHGKVVLVLER-------S------SQG
TG-----PSRP------------------PTPGRN------------R-YTVMSGTPEKI
LELLLEA------------MRPDSSAHDPT-ETF-------------------LS---D-
FLLTHSV--FM---------------PSTQLFTALL-----HHF------H---------
-----------------------------VEPAEPAGGSEQERSTYVCNKRQQILRLVSR
WVALYGPMLHSD-PVATSFLQ----KLSDL--VSRDA--RLSNLLR---EQ-WPERRR-H
HRL----ENGCGNA--SP------QIKAR----------------------NAP------
-----VW-----------LPGQE--------EP---------------------------
-----------------L--
---PSSTS--

----GAIRVGDK--VP-----------------------------YD-------------
ICRPDHSVLTLQLPVTASVREVMA--------ALAHEDH-----WT-------KGQ--VL
VKVNSA----------------------G------DVVGLQPDARGVATSLGLNERLFVV
DPQE---V-HELT-PHPEQL---------------------------GP------TLGSS
EM--LDLVSAKDLAGQLTELDWSLFNRIHQVELIH-------YVLGPQHL-RDVTTANLE
RFMRR---------FNELQYWV-----ATELCLCSVPGPR------AQLLRKFIKLAAHL
---------------KEQKNLNSFFAVMF-GLSNSAISRLAHTWE---------------
-------RLPHKVRKLYSA-LERLL--------------------------DPSWNH---
-RVYRL----ALTK-------LSPPVI-PFMP----------------------------
LLLKDMTFIHEGNH--TL-VE-----------------------------N-LINFE-KM
-------RM---MARAMRMLHHCRSHST------------------------VPLSPLRS
RVSHIHEDSQASRISTCSEQSLSTR-------S--------------PASTWA-----YV
QQLKVIDNQREL---SRLSREL
-----------EP---

>Rapgef3_[Miniopterus_natalensis]_1016658187

---------------------M--------------------------------------

---------------K---------------------------------VGWPGES----
-----RWQVGLIVEY-SSALGAPQVGG----LPDLVPEATLLNMVLRRMHRPRSYSYQ-L
LLE------------------------HQRP-S---------RIQ---------------
------------------GLR-------------------WT------------------
-----PLTNSEESLDFSV-SLEQA-------------------S--------T-------
-------ERVL-------------RA---------------------GRQLH-RHLLATC
QN-L----------------IRDRKYH------L-RLY----------------RQC-CS
GR-----------------ELVDGILTLG------------------------------L
----GVH--------SRSQAVGIC---QVLLDEGALC-----------------------
--------------H------------------------VKH-DWTFQDRDTQFYRFPGP
-------EPEP--AGVHEL---

---------EEEL------VEALALLSQRGPDAL-------------LTVALRKPPGQRT
DEELE-----LIFEELLHIKAVAHLS-NSVKRELA-AVLLFEPHSKAGTVL---------
--FSQGDKG--TSWYIIW
KGSVNVVTHGK-----GL-------------------------------VTTLHEG-DDF
GQLAL-----------VNDAPRA---ATIILREDNCHFLRVDKQDFNR------IIKD--
---V--------------

------------------------EAKTIRLEEHGKVVLVLER-------T------SQG

AG-----SSCP------------------PTPGRN------------R-YTVMSGTPEKI
LELLLEA------------MRPDSSAHDPT-ETF-------------------LS---D-
FLLTHRV--FM---------------PSTQLCAALL-----HHF------H---------
-----------------------------AEPT---GGSEQERNTYICNKRQQILRLVSQ
WVALYGPMLHTD-PVASSFLQ----KLSDL--VSRDA--RLSNLLR---EQ-WPERRR-H
HRL----ENGCGNT--SP------QIKAR----------------------NMP------
-----VW-----------LPGQD--------EP---------------------------
-----------------L--
---PSSN---

----CAIRVGDK--VP-----------------------------YD-------------
ICRPDHSVLTLQLPVTASVREVMA--------ALAQEDG-----WT-------KGQ--VL
VKVNSA----------------------G------DAIGLQPDARGVATSLGLNERLFVV
NPQE---V-HELT-PHPEQL---------------------------GP------TVGSA
EG--LDLVSAKDLAGQLTDHDWNLFNSIHQVELIH-------YVLGPQHL-RDVTTANLE
RFMRR---------FNELQYWV-----ATELCLCPVPGPR------AQLLRKFIKLAAHL
---------------KEQKNLNSFFAVMF-GLSNSAISRLAHTWE---------------
-------RLPHKVRKLYSA-LERLL--------------------------DPSWNH---
-RVYRL----ALTK-------LSPPVI-PFMP----------------------------
LLLKDMTFIHEGNH--TL-VE-----------------------------N-LINFE-KM
-------RM---MARAVRMLHHCRSHSN------------------------VPLSPLRS
RVSHLHEDGQAARISTCSEQSLSTR-------S--------------PASTWA-----YV
QQLKVINNQREL---SRLSREL
-----------EP---

>Rapgef3_[Mus_musculus]_1039748753

---------------------M--------------------------------------
-------------------------------TSPSPEDAKPGVVVG--------RSSSSG
RWL-----SSFLPTSQ---------------------------------VSWPGEN----
-----HWQVGPAVVE-SPAVGAPQVGG----LPDVVPEGTLLNMVLKRMHRPRCCSYQ-L
VFE------------------------HRRP-S---------CIQ---------------
------------------GLR-------------------WT------------------
-----PLTNSEDSLDFRV-SLEQA-------------------T--------T-------
-------EHVH-------------KA---------------------GKLLH-RHLLATY
PT-L----------------IRDRKYH------L-RLY----------------RHC-CS
GR-----------------ELVDGILALG------------------------------L
----GVH--------SRSQAVGIC---QVLLDEGALC-----------------------
--------------H------------------------VKH-DWTFQDRDAQFYRFPGP
-------EPEP--TGTQDV---

---------EEEL------VEAMALLSQRGPDAL-------------LTVALRKPPGQRT
DEELD-----LIFEELLHIKAVAHLS-NSVKRELA-AVLLFEPHSKAGTVL---------
--FSQGDKG--TSWYIIW
KGSVNVVTHGK-----GL-------------------------------VTTLHEG-DDF
GQLAL-----------VNDAPRA---ATIILRENNCHFLRVDKQDFNR------IIKD--
---V--------------

------------------------EAKTMRLEEHGKVVLVLER-------T------SQG
AG-----PSRP------------------PTPGRN------------R-YTVMSGTPEKI
LELLLEA------------MRPDSSAHDPT-ETF-------------------LS---D-
FLLTHSV--FM---------------PSTQLFTALL-----HHF------H---------
-----------------------------VEPADPAGGSEQEHSTYICNKRQQILRLVGR
WVALYSPMLHSD-PVATSFLQ----KLSDL--VSRDA--RLSNLLR---EQ-YPERRR-H
HRL----ENGCGNV--SP------QTKAR----------------------NAP------
-----VW-----------LPNQE--------EP---------------------------
-----------------L--
---PSSA---

----GAIRVGDK--VP-----------------------------YD-------------
ICRPDHSVLTLHLPVTASVREVMA--------ALAHEDH-----WT-------KGQ--VL
VKVNSA----------------------G------DVVGLQPDARGVATSLGLNERLFVV
DPQE---V-HELT-PHPEQL---------------------------GP------TLGSS
EM--LDLVSAKDLAGQLTDHDWNLFNRIHQVELIH-------YVLGPQHL-RDVTTANLE
RFMRR---------FNELQYWV-----ATELCLCPVPGSR------AQLLRKFIKLAAHL
---------------KEQKNLNSFFAVMF-GLSNSAISRLAHTWE---------------
-------RLPHKVRKLYSA-LERLL--------------------------DPSWNH---
-RVYRL----ALTK-------LSPPVI-PFMP----------------------------
LLLKDVTFIHEGNH--TL-VE-----------------------------N-LINFE-KM
-------RM---MARAVRMLHHCRSHST------------------------APLSPLRS
RVSHIHEDSQGSRISTCSEQSLSTR-------S--------------PASTWA-----YV
QQLKVIDNQREL---SRLSREL
-----------EP---

>Rapgef3_[Mustela_putorius_furo]_511891954

---------------------M--------------------------------------

---------------K---------------------------------VGWPGES----
-----RWQVGLAVDD-TPSLGAAQVGG----LPDVVPEGTLFNMVLKRVHRPRSCSYQ-L
LLE------------------------HQRP-S---------RIQ---------------

```
------------------GLR-------------------WT------------------
-----PLTDSEESLDFSV-SLEQA-------------------S--------A-------
-------ERVL-------------RA---------------------GKQLH-RHLLATC
PT-L----------------IRDRKYH------L-RLY----------------RQC-CS
GR-----------------ELVDGILALG------------------------------L
----GVQ--------SRSQAVGIC---QVLLDEGALY-----------------------
--------------H------------------------VKH-DWTFQDRDTQFYRFPGP
-------EPET--AGVREL-----------------------------------------
------------------------------------------------------------
---------EEEL------VEAVALLSQRGPDAL-------------LTVALRKPPGQRT
DEELD-----LIFEELLHIKAVAHLS-NSVKRELA-AVLLFEPHSKAGTVL---------
--------------------------------------------FSQGDKG--TSWYIIW
KGSVNVVTHGK-----GL-------------------------------VTTLHEG-DDF
GQLAL-----------VNDAPRA---ATIILREDNCHFLRVDKQDFNR------IIKD--
---------------------------------------------V--------------
------------------------------------------------------------
------------------------------------------------------------
------------------------EAKTMRLEEHGKVVLVLER-------A------SQG
AG-----PSRP------------------PTPGRN------------R-YTVMSGTPEKI
LELLLEA------------MRPDSSTHDPT-ETF-------------------LS---D-
FLLTYSV--FM---------------PSAQLCAALL-----HHF------H---------
-----------------------------AEPS---GGSEQENSTYICNKRQQILRLVSQ
WMALYGPMLHAD-PVATSFLQ----KLSDL--VSRDT--RLCLLLR---EQ-WPERRR-H
HRL----ENGCGNA--SP------QMKTR----------------------NMP------
-----VW-----------LPGQD--------QP---------------------------
-----------------L------------------------------------------
---PSRN-----------------------------------------------------
------------------------------------------------------------
------------------------------------------------------------
------------------------------------------------------------
------------------------------------------------------------
----CAIRAGDK--VP-----------------------------YD-------------
ICRPDHSVLTLKLPVTASVREVMA--------ALAQEDG-----WT-------KGQ--VL
VKVNSA----------------------G------DAIGLQPEARGVATSLGLNERLFVV
NPQE---V-HELT-PHPEQL---------------------------GP------TMGSA
EG--LDLVSAKDLAGQLTDHDWSLFNSIHQVELIH-------YVLGPQHL-RDVTTANLE
RFMRR---------FNELQYWV-----ATELCLCPVPGLR------AQLLRKFIKLAAHL
---------------KEQKNLNSFFAVMF-GLSNSAISRLAHTWE---------------
-------RLPHKVRKLYSA-LERLL--------------------------DPSWNH---
-RVYRL----ALTK-------LSPPII-PFMP----------------------------
LLLKDMTFIHEGNH--TL-VE-----------------------------N-LINFE-KM
-------RM---MARAVRMLQHCRSHSN------------------------VPLSPLRS
RVSHLHEDSQVARMSTCSEQSLSTR-------S--------------PASPWA-----YV
QQLKVIDNQREL-----------------------------------------SRLSREL
-----------EP-----------------------------------------------
------------------------------------------------------------
------------------------------------------------------------
------------------------------------------------------------
------------------------------------------------------------
------------------------------------------------------------
------------------------------------------------------------
------------------------------------------------------------
------------------------------------------------------------
------------------------------------------------------------
```


>Rapgef3_[Myotis_brandtii]_554566307

---------------------M--------------------------------------

---------------K---------------------------------VGWPGES----
-----RWQVGLFVED-SPALGAPQVGS----LPDVVPEGTLLNMVLRRMHLPRSCSYR-L
LLE------------------------HQRP-S---------RIQ---------------
------------------GLR-------------------WT------------------
-----PLTNSEESLDFSV-SLEQA-------------------S--------T-------
-------ERVL-------------RA---------------------GKQLH-RHLLATC
QN-L----------------IRDRKYH------L-RLY----------------RQC-CS
GR-----------------ELVDGILALG------------------------------L
----GVH--------SRSQAVGIC---QVLLDEGALC-----------------------
--------------H------------------------VKH-DWTFQDRDTQFYRFPGP
-------EPEP--SGVHEL---

---------EEEL------VEAMALLSQRGPDAL-------------LTVALRKPPGQRT
DEELE-----LIFEELLHIKAVAHLS-NSVKRELA-AVLLFEPHSKAGTVL---------
--FSQGDKG--TSWYIIW
KGSVNVVTHGK-----GL-------------------------------VTTLHEG-DDF
GQLAL-----------VNDAPRA---ATIILREDNCHFLRVDKQDFNR------IIKD--
---V--------------

------------------------EAKTMRLEEHGKVVLVLER-------T------SQG
AA-----SSCP------------------PTPGRN------------R-YTVMSGTPEKI
LELLLEA------------MRPDSSAHDPT-ETF-------------------LS---D-
FLLTHRV--FM---------------PSTQLCTALL-----HHF------H---------
-----------------------------AEPA---GGSEQERNIYICNKRQQILRLVSQ
WVALYGPMLHTD-PVASSFLQ----KLSDL--VSKDA--RLSNLLR---EH-WPERRR-H
HRM----ENGGGSA--SP------QIKAR----------------------NMS------
-----VW-----------LPGPD--------ES---------------------------
-----------------L--
---PSSN---

----CAIRVGDK--VP-----------------------------YD-------------
ICRPDHSVLTLQLPVTASVREVMA--------ALAQEDG-----WT-------KGQ--VL
VKVNSA----------------------G------DAIGLQPDARGVATSLGLNERLFVV
NPEE---V-HELT-PHPEQL---------------------------GP------TVGSA
EG--LDLVSAKDLAVQLTDHDWNLFNSIHQVELIH-------YVLGPQHL-RDVTTANLE
RFMRR---------FNELQYWV-----ATELCLCPVPGPR------AQLLRKFIKLAAHL
---------------KEQKNLNSFFAVMF-GLSNSAISRLAHTWE---------------
-------RLPHKVRKLYSA-LERLL--------------------------DPSWNH---
-RVYRL----ALTK-------LSPPVI-PFMP----------------------------
LLLKDMTFIHEGNH--TL-VE-----------------------------N-LINFE-KM
-------RM---MARAVRMLHHCRSHSN------------------------VPLSPLRS
RVSHLHEDIQAARVSTCSEQSLSTR-------S--------------PASTWA-----YV
QQLKVIDNQREL---SRLSREL
-----------EP---

```
------------------------------------------------------------
------------------------------------------------------------
------------------------------------------------------------
------------------------------------------------------------
------------------------------------------------------------
------------------------------------------------------------
------------------------------------------------------------
------------------------------------------------------------
------------------------------------------------------------
------------------------------------------------------------
---
>Rapgef3_[Myotis_davidii]_432114532
------------------------------------------------------------
---------------------S--------------------------------------
------------------------------------------------------------
---------------Q---------------------------------VGWPGES----
-----RWQVGLIVED-SPALGAPQVGS----LPDVVPEGTLLNMVLRRMHLPRSCSYR-L
LLE------------------------HQRP-S---------RIQ---------------
------------------GLR-------------------WT------------------
-----PLTNSEESLDFSV-SLEQA-------------------S--------T-------
-------ERVL-------------RA---------------------GKQLH-RHLLATC
QN-L----------------IRDRKYH------L-RLY----------------RQC-CS
GR-----------------ELVDGILALG------------------------------L
----GVH--------SRSQAVGIC---QVLLDEGALC-----------------------
--------------H------------------------VKH-DWTFQDRDTQFYRFPGP
-------EPEP--SGVHEL-----------------------------------------
------------------------------------------------------------
---------EEEL------VEAMALLSQRGPDAL-------------LTVALRKLPGQRT
DEELE-----LIFEELLHIKAVAHLS-NSVKRELA-AVLLFEPHSKAGTVL---------
--------------------------------------------FSQGDKG--TSWYIIW
KGSVNVVTHGK-----GL-------------------------------VTTLHEG-DDF
GQLAL-----------VNDAPRA---ATIILREDNCHFLRVDKQDFNR------IIKD--
---------------------------------------------V--------------
------------------------------------------------------------
------------------------------------------------------------
------------------------EAKTMRLEEHGKVVLVLER-------T------SQG
TA-----SSCP------------------PTPGRN------------R-YTVMSGTPEKI
LELLLEA------------MRPDSSAHDPT-ETF-------------------LS---D-
FLLTHRV--FM---------------PSTQLCAALL-----HQY------P---------
-----------------------------SSPR---DGVEQERNIYICNKRQQILRLVSQ
WVALYGPMLHTD-PVASSFLQ----KLSDL--VSKDA--RLSNLLR---EH-WPERRR-H
HRL----ENGGGST--SP------QIKAR----------------------NMS------
-----VW-----------LPGPD--------ES---------------------------
-----------------L------------------------------------------
---PSSN-----------------------------------------------------
------------------------------------------------------------
------------------------------------------------------------
------------------------------------------------------------
------------------------------------------------------------
----CAIRVGDK--VP-----------------------------YD-------------
ICRPDHSVLTLQLPVTASVREVMA--------ALAQEDG-----WT-------KGQ--VL
VKVNSA----------------------G------DAIGLQPDARGVATSLGLNERLFVA
NPQE---V-HELT-PHPEQL---------------------------GP------TVGSA
EG--LDLVSAKDLAVQLTDHDWNLFNSIHQVELIH-------YVLGPQHL-RDVTTANLE
```

RFMRR---------FNELQYWV-----ATELCLCPVPGPR------AQLLRKFIKLAAHL
---------------KEQKNLNSFFAVMF-GLSNSAISRLAHTWE---------------
-------RLPHKVRKLYSA-LERLL--------------------------DPSWNH---
-RVYRL----ALTK-------LSPPVI-PFMP----------------------------
LLLKGSQLPHRPGRGSAL-RP-----------------------------G-LMSLLCPQ
-------RM---MARAVRMLHHCRSHSN------------------------VPLSPLRS
RVSHLHEDSQAARVSTCSEQSLSTR-------S--------------PASTWA-----YV
QQLKVIDNQREL---SRLSREL
-----------EP---

>Rapgef3_[Myotis_lucifugus]_940740350

---------------------M--------------------------------------

---------------K---------------------------------VGWPGES----
-----RWQVGLIVED-SPALGAPQVGS----LPDVVPEGTLLNMVLRRMHLPRSCSYR-L
LLE------------------------HQRP-S---------RIQ---------------
------------------GLR-------------------WT------------------
-----PLTNSEESLDFSV-SLEQA-------------------S--------T-------
-------ERVL-------------RA---------------------GKQLH-RHLLATC
QN-L----------------IRDRKYH------L-RLY----------------RQC-CS
GR-----------------ELVDGILALG------------------------------L
----GVH--------SRSQAVGIC---QVLLDEGALC-----------------------
--------------H------------------------VKH-DWTFQDRDTQFYRFPGP
-------EPEP--SGVHEL---

---------EEEL------VEAMALLSQRGPDAL-------------LTVALRKPPGQRT
DEELE-----LIFEELLHIKAVAHLS-NSVKRELA-AVLLFEPHSKAGTVL---------
--FSQGDKG--TSWYIIW
KGSVNVVTHGK-----GL-------------------------------VTTLHEG-DDF
GQLAL-----------VNDAPRA---ATIILREDNCHFLRVDKQDFNR------IIKD--
---V--------------

------------------------EAKTMRLEEHGKVVLVLER-------T------SQG
AG-----SSCP------------------PTPGRN------------R-YTVMSGTPEKI
LELLLEA------------MRPDSSAHDPT-ETF-------------------LS---D-
FLLTHRV--FM---------------PSTQLCAALL-----HHF------H---------
-----------------------------AEPA---GGSEQERNIYICNKRQQILRLVSQ
WVALYGPMLHTD-PVASSFLQ----KLSDL--VSKDA--RLSNLLR---EH-WPERRR-H
HRM----ENGSGSA--SP------QIKAR----------------------NMS------
-----VW-----------LPGPD--------ES---------------------------
-----------------L--
---PSSN---

----CAIRVGDK--VP-----------------------------YD-------------
ICRPDHSVLTLQLPVTASVREVMA--------ALAQEDG-----WT-------KGQ--VL
VKVNSA----------------------G------DAIGLQPDARGVATSLGLNERLFVV
NPEE---V-HELT-PHPEQL---------------------------GP------TVGSA
EG--LDLVSAKDLAVQLTDHDWNLFNSIHQVELIH-------YVLGPQHL-REVTTANLE
RFMRR---------FNELQYWV-----ATELCLCPVPGPR------AQLLRKFIKLAAHL
---------------KEQKNLNSFFAVMF-GLSNSAISRLAHTWE---------------
-------RLLHKVRKLYSA-LERLL--------------------------DPSWNH---
-RVYRL----ALTK-------LSPPVI-PFMP----------------------------
LLLKDMTFIHEGNH--TL-VE-----------------------------N-LINFE-KM
-------RM---MARAVRMLHHCRSHSN------------------------VPLSPLRS
RVSHLHEDIQAARVSTCSEQSLSTR-------S--------------PASTWA-----YV
QQLKVIDNQREL---SRLSREL
-----------EP---

>Rapgef3_[Neolamprologus_brichardi]_583998066

--MHLFRTHNYQ-V
F----PD-----------------RRSVE-K--QTIR-----------------------
------------------GIS-------------------WT------------------
-----PLPEALNS---KD-TIKQ---------------------------FLS-------
-------DRVV-------------KA---------------------ARSVY-SVMIEKN
PG-L----------------IRDRKHH------L-KTY----------------RQC-CS
GK-----------------DLVDWLMTQN------------------------------E
----GLQ--------SRSQAVGIW---QVLVDEGILL-----------------------
--------------H------------------------VKH-ELNFLDKDTQFYRFQDS
-------QFGLNHILNEK--
DS--
---------EDEL------QEALSLLSQLGPDAL-------------LTMILRKCPSQRS
PEDLE-----VIYEELLHVKAAAHLS-SSVRKELA-AVLVFESHAKAGTVL---------
--FSQGDKG--TSWYIIW
KGSVNVITHGK-----GL-------------------------------VTTLHEG-EDF
GQLAL-----------LNEQPRA---ATIILREDNCHFLRVDKQDFIR------ILKD--
---V--------------

------------------------EANTVRLEEHGKTVLVLEK-------S---------

AD----STEQG------------------GAGNSS------------K-YTVMSGSPEKI
LEHLLET------------IKLESNGNDAI-DS-------------------CVS---D-
FLLTHKV--FM---------------PSSQLCPALQ-----HHY------Q---------
-----------------------------AQLAE---GSEQEKTAYVLNIKQKVVKLVGQ
WVALFGLHLKED-PIALDFLE----RLKKD--MAANS--QLFNVLK---D--FRERKR--
TKV---LENGYQSL--S-------R-N-HQFD----------------------------
------W-----------FSNFE--------ES---------------------------

---VGRV---

----QPIRAHDK--VL-----------------------------YE-------------
IYRPDNKALTPMLPVNTSVQEIMS--------ALGKAG---------------EDH--VL
IKMNST----------------------G------ERAQLKLDATAVYTALGLNERLFIC
TISE---V-EQLT-PLKEQQ---------------------------GP------EQGTA
DV--LEQMSSKDIASELTNYDWELFTAMHEVELVY-------YIFGRHKF-PGAITANLE
RFVRR---------FNEVQHWV-----LTELCLCEDLMKR------AMLLKKFIKIASVL
---------------KEQKNLNSFFAVMF-GLSNSAIQRLYKTWE---------------
-------RIPSKTKRIYCS-LERLM--------------------------DPSRNH---
-RAYRL----AIAK-------LSPPYI-PFMP----------------------------
LLLKDMTFIHEGNA--NY-VD-----------------------------K-LVNFE-KM
-------RM---LAKTVKIVRGCRSQPY------------------------VPSSPQRG
LADRMFLEGAATRLSTYSDHAFPLR-------T--------------STNIRH-----YI
QNLKVIDNQRKL---TQLSRTI
-----------DC---

>Rapgef3_[Nothobranchius_furzeri]_1007768319

--MHLFRSHNYQ-V
F----PH-----------------RHAVQ-T--PTVR-----------------------
------------------GIS-------------------WT------------------
-----PLPEALNS---KD-TMKQ---------------------------FLS-------
-------DRVI-------------KA---------------------ARSVY-SIMTERN
PD-L----------------IRDRKHH------L-KTH----------------RQC-CS
GK-----------------ELVDWLMKQN------------------------------E
----CLQ--------SRSQAVGMW---QVLVDEGVLV-----------------------
--------------H------------------------VKQ-ELNFHDKDAQFYRFQDS
-------EFGLNHTSNEK--
DS--

---------EDEL------HEALSLLSQLGPDAL-------------LTMILRKCPSQRS
PEDIE-----VIYEELLHVKAAAHLS-SSVRKELA-AVLVFESHSKAGTVL---------
--FSQGDKG--TSWYIIW
KGSVNVITHGK-----GL-------------------------------VTTLHEG-EDF
GQLAL-----------LNDAPRA---ATIILREDNCHFLRVDKQDFIR------ILKD--
---V--------------

------------------------EANTVRLEEHGKTVLVLEK-------S---------
AD----SAEQG------------------GAGASS------------K-YTVMSGSPEKI
LEHLLET------------VKLDSNGNDPTADP-------------------CVS---D-
FLLTHRV--FM---------------PSSQLCAALL-----HHY------Q---------
-----------------------------AELSE---GSEQEKDAYAFNSKQKVVKLVGQ
WVALYSHLLKED-PAALDFLE----RMKKE--VAADY--RLSSILK---EQ-FRERRR--
AKM---LENGYQSL--S-------R-N-QPFD----------------------------
------W-----------FSLCE--------EC---------------------------

---VGRI---

----LPIKAQDK--VL-----------------------------YE-------------
VYRPDNKPLSLMLPVNSSVQDVMS--------ALVKPV---------------GDH--VL
VKMNSA----------------------G------ERAQLKLDACGVYTALGLNERLFIC
TSSE---V-EQLR-PLRDQQ---------------------------GP------ERGTA
DL--IEQMSSKDLAIELTNYDWELFNAMHEVELIY-------YIFGRHKF-PGAITANLE
RFVRH---------FNEVQHWV-----VTELCLCEDLVKR------AVLLKKFIKIASVL
---------------KEQKNLNLFFAVMF-GLSNSAVQRLYKTWE---------------
-------RIPSKTKRIYCT-YERLM--------------------------DPSRNH---
-RAYRL----AVAK-------LSPPYT-PFMP----------------------------
LLLKDMTFIHEGNP--NY-TD-----------------------------K-LVNFE-KM
-------RM---LAKTVKIVRGCRSQPY------------------------VPSSPQRG
LADRIFLEGP-TRLSTNSEHAFPLR-------T--------------PSNVRH-----YI
QNLKVIDNQRKL---TQLSRTM
-----------EC---

>Rapgef3_[Notothenia_coriiceps]_736177724


```
------------------------------------------------------------
-------------------MFMKF-------------------L--------S-------
-------DRVV-------------KA---------------------ARSVY-SVLMERD
SN-L----------------IRDRKHH------L-KTY----------------RQC-CS
GK-----------------ELVDWLMKQN------------------------------E
----CLA--------SRSQAVGMW---QVLLDEGILV-----------------------
--------------H------------------------VKH-ELNFLDKDTQFYRFQEC
-------EFGLHHVSDERE-----------------------------------------
--------------------E---------------------------------------
----------EEL------QEAVSLLAQLGPDAL-------------LTMILRKGPSQNS
GLGLLNWPLFCVYTDDAGIFALALIG----------------------------------
------------------------------------------------------------
------------------------------------------------------------
------------------------------------------------------------
------------------------------------------------------------
------------------------------------------------------------
------------------------------------------------------------
------------------------------------------------------------
------------------------------------------------------------
------------------------------------------------------------
------------------------------------------------------------
------------------------------------------------------------
------------------------------------------------------------
------------------------------------------------------------
------------------------------------------------------------
------------------------------------------------------------
------------------------------------------------------------
------------------------------------------------------------
------------------------------------------------------------
------------------------------------------------------------
------------------------------------------------------------
------------------------------------------------------------
------------------------------------------------------------
------------------------------------------------------------
------------------------------------------------------------
------------------------------------------------------------
------------------------------------------------------------
------------------------------------------------------------
------------------------------------------------------------
------------------------------------------------------------
------------------------------------------------------------
------------------------------------------------------------
------------------------------------------------------------
------------------------------------------------------------
------------------------------------------------------------
------------------------------------------------------------
------------------------------------------------------------
------------------------------------------------------------
------------------------------------------------------------
------------------------------------------------------------
------------------------------------------------------------
------------------------------------------------------------
------------------------------------------------------------
------------------------------------------------------------
```


>Rapgef3_[Ochotona_princeps]_504177543

-----------------------------------MVRVRVGFQQLLSKQGSCGPS----
----------------SFRDGPAKVGG----LADLPPEAQLLVAV-RRMRRPRSCSYQ-L
LLPP-----------------------HGRP-G---------RVQ---------------
------------------GLR-------------------WT------------------
-----PLTASEESLDFSG-SLEQA-------------------S--------T-------
-------ERVL-------------RA---------------------GKQLY-RHLLAAC
PG-L----------------IRDRKHH------L-RLY----------------RQC-CS
GQ-----------------ELVDGILTLG------------------------------L
----AVH--------SRSQAVGIC---QVLLDEGALY-----------------------
--------------H------------------------VKH-DWAFQDRDAQFYRFPWL
-------ELEP--TGSPGA---

---------EEEL------VEAMALLAQRGPDAL-------------LTMALRKPPNQRT
HEELE-----LIFEELLHIKAVAHLS-NSVKRELA-AVLLFEPHSKAGTVL---------
--FSQGDKG--TSWYIIW
KGSVNVVTHGK-----GL-------------------------------VTTLHEG-DDF
GQLAL-----------VNDAPRA---ATIILREDSCHFLRVDKQDFNR------IIKD--
---V--------------

------------------------EAKTMRLEEHGKVVLVLER-------A------SQG
AS-----PACP------------------PTPGRT------------R-YTVMSGTPEKI
LELLLEA------------MRPDSSSHDPT-ETF-------------------LS---D-
FLLTHSV--FM---------------PSAQLCPALL-----HHF------H---------
-----------------------------AQPA---GGSEQERSSYVCNKRQQIVRLVNQ
WVALYGPMLHVD-PAASNFLQ----GFSEL--VSGDV--QLSGLLK---GQ-WPERRR-L
HRL----ENGCGNA--SP------QLKAW----------------------NAP------
-----VW-----------LPSHE--------EP---------------------------
-----------------L--
---PSSN---

----CAIRAGDK-VS------------------------------YN-------------
IYRPDHSGLTLQLPVTASVREVLA--------ALAQADG-----RT-------SGQ--VL
VKVNSA----------------------G------DAVGLQPEARGVATSLGLNERLFVA
NPQQ---V-HELT-PHPEQL---------------------------GP------TVGSA
EA--LELVSAKDLAGQLTEQDRDLFHSIHQVELIH-------YVLGPQHL-RNVTTANLE
RFMRR---------FNQLQYWV-----ATELCLCPLPGPR------AQLLRKFIKLAAHL
---------------KEQKNLNSFFAIMF-GLSNSAITRLARTWE---------------
-------RLPQKVRKLHSA-LERLL--------------------------DPSWNH---
-RAYRV----ALAK-------LSPPVI-PFMP----------------------------
LLLKDMTFIHEGNR--TL-VE-----------------------------N-LINFE-KM
-------RM---LARAVRVLHHCQGHGH------------------------APLSPLRT
RAAHIHEDSQASRISTCSEQSLSTR-------N--------------PASAWA-----DI
QQLKVIDNQREL---SRLSREL
-----------EP---

>Rapgef3_[Octodon_degus]_820979431

---MVLRRMPRSRSCSYQ-L
LLE------------------------HRRP-S---------RIQ---------------
------------------GLR-------------------WT------------------
-----PLTTSEESLDFSE-SLEQA-------------------T--------T-------
-------ERVL-------------RA---------------------GRYLH-RHLLATY
PS-L----------------IRDRKHH------L-RLH----------------RQC-CS
GR-----------------ELVDGILALG------------------------------L
----GVH--------SRSQAVGIC---QVLLDEGALC-----------------------
--------------H------------------------VKH-DWTFQDRDAQFYRFLGP
-------EPEP--AGVHEL---

---------EEEL------VEAMALLSQRGPDAL-------------LTVALRKPPGQRV
EEELD-----LIFEELLHIKAVAHLS-NSVKRELA-SVLLFEPHSKAGTVL---------
--FSQGDKG--TSWYIIW
KGSVNVVTHGK-----GL-------------------------------VTTLHEG-DDF
GQLAL-----------MNDAPRA---ATIIILREDNCHFLRVDKQDFNR------IIKD--
---V--------------

------------------------EARTMRLEEHGKVVLVLER-------T------SQG
AG-----SSRP------------------PTPGRS------------R-YTVMSGTPEKI
LELLLEA------------MRPDSSAHDPT-ETF-------------------LG---D-
FLLTHSV--FM---------------PTAQLCAALL-----RHF------H---------
---MES-----------------------AESA---GDSEPERSSYTCNRRQQTLWLVGQ
WVALYGPTLRRD-PVATSFLQ----TLSDL--VSKDT--WLSQLLR---EQ-WPERRR-H
HRL----ENGCGST--SP------QLKAR----------------------NTP------
-----VW-----------LPSQD--------EP---------------------------
-----------------F--
---PSSS---

----CAIRVGDK--VP-----------------------------YD-------------
ICRPDHSVLTLKLPVTASVREVMA--------ALAQEDG-----WT-------KGQ--VL
VKLTSA----------------------G------DAIGLQPEARGVATSLGLNERLFVV
NPQE---V-HKLT-PHPEQL---------------------------GP------TVGSA
EG--LDAVSAKDLAGQLTERDWGLFKCIHQVELIH-------YVLDPQHL-RDGTTANLE

```
RVLRR---------FNELQYWV-----ATELCLCSAPGLR------AQLLRKFIKLAAHL
---------------KEQKNLNSFFAVMF-GLSNSAISRLARTWE---------------
-------RLPQKVRKLYSA-LERLL--------------------------DPSWNH---
-RVYRL----ALRK-------LSPPLI-PFMP----------------------------
LLLKDMTFIHEGNR--TL-VD-----------------------------S-LINFE-KM
-------RM---MARAARLLHYCRSHST------------------------APLSPLRS
RVSHLHEDSQALRMSTCSEQSLSTR-------S--------------PASTWA-----YI
QQPKVIDNQREL-----------------------------------------SRLSREL
-----------EP-----------------------------------------------
------------------------------------------------------------
------------------------------------------------------------
------------------------------------------------------------
------------------------------------------------------------
------------------------------------------------------------
------------------------------------------------------------
------------------------------------------------------------
------------------------------------------------------------
------------------------------------------------------------
------------------------------------------------------------
---
>Rapgef3_[Odobenus_rosmarus_divergens]_823398553
------------------------------------------------------------
---------------------M--------------------------------------
------------------------------------------------------------
---------------K---------------------------------VGWPGES----
-----PWRVGLAADG-SPALGAAQVGG----LPDVVPEGTLLNMVLKRMHRPRSCSYQ-L
LLE------------------------HQRP-S---------RIQ---------------
------------------GLR-------------------WT------------------
-----PLTDSEESLDFSV-SLEQA-------------------S--------M-------
-------ERVL-------------RA---------------------GKQLH-RQLLATC
PT-L----------------IRDRKYH------L-RLY----------------RQC-CS
GR-----------------ELVDGILALG------------------------------L
----GVH--------SRSQAVGIC---QVLLDEGALC-----------------------
--------------H------------------------VKH-DWTFQDRDTQFYRFPGP
-------EPEP--VGIREL-----------------------------------------
------------------------------------------------------------
---------EEEL------VEAVALLSQRGPDAL-------------LTVALRKPPGQRT
DEELD-----LIFEELLHIKAVAHLS-NSVKRELA-AVLLFEPHSKAGTVL---------
--------------------------------------------FSQGDKG--TSWYIIW
KGSVNVVTHGK-----GL-------------------------------VTTLHEG-DDF
GQLAL-----------VNDAPRA---ATIILREDNCHFLRVDKQDFNR------IIKD--
---------------------------------------------V--------------
------------------------------------------------------------
------------------------------------------------------------
------------------------EAKTMRLEEHGKVVLVLER-------T------SQG
AG-----PSRP------------------PTPGRN------------R-YTVMSGTPEKI
LELLLEA------------MRPDSSAHDPT-ETF-------------------LS---D-
FLLTYSV--FM---------------PSAQLCAALL-----HHF------H---------
-----------------------------AEPS---GGSEQEHSTYICNKRQQILRLVSQ
WVALYGPMLHTD-PVATSFLQ----KLSDL--VSRDA--RLCILLR---EQ-WPERRR-H
HRL----ENGCGNA--SP------LMKAR----------------------NMP------
-----VW-----------LPGQD--------QP---------------------------
-----------------L------------------------------------------
---PSSN-----------------------------------------------------
```


----CAIRVGDK--VP-----------------------------YD-------------
ICRPDHSVLTLKLPVTASVREVMA--------ALAQEDG-----WT-------KGQ--VL
VKVNST----------------------G------HAIGLQPEARGVATSLGLNERLFVV
NPQE---V-HELT-PHPEQL---------------------------GP------TVGSA
EG--LDLVSAKDLAGQLTDHDWSLFNSVHQVELIH-------YVLGPQHL-RDVTTANLE
RFMRR---------FNELQYWV-----ATELCLCPVPGLR------AQLLRKFIKLAAHL
---------------KEQKNLNSFFAVMF-GLSNSAISRLAHTWE---------------
-------RLPHKVRKLYSA-LERLL--------------------------DPSWNH---
-RVYRL----LLTK-------LSPPII-PFMP----------------------------
LLLKDMTFIHEGNH--TL-VE-----------------------------N-LINFE-KM
-------RM---LARAVRMLHHCRSHSN------------------------VPLSPLRS
RVSHLHEDSQAARTSTCSEQSLGTR-------S--------------PASTWA-----YV
QQLKVIDNQREL---SRLSREL
-----------EP---

>Rapgef3_[Opisthocomus_hoazin]_700398843

---QC-CS
GK-----------------ELVDWLLSAG------------------------------L
----AVQ--------TRSQAVGIC---QVLVDGGVLT-----------------------
--------------H------------------------APG--WGDPNSG---------

-----------------------HLL-PQVKQELA-SVLMFESHQRAGTV----------
---LFSQGDKG--TSWYIIW
KGSVNVVTHGK-----GL-------------------------------VATLHEG-DDF
GQLAL-----------VNDAPRA---ATIILREDNCHFLRVDKQDFNR------ILKD--
---V--------------

------------------------EANTMRLKEHGKVVLVLQKNL-------------QG

G------SNQP------------------ATAR-----------S-SR-YLVMAGTPEKI
LEHLLEF------------MRLDAT-HVPT-D-------------------TLLG---D-
FLLTYTV--FM---------------PTSQLCRALL-----HHF------R---------
-----------------------------AEPLE---GSEQEKATYSLHKRRKILRLVSQ
WVLLYGRLLQGD-RSTTALLQ----G-PG--AGRGTP--HPEQLGP---HV-SSSETL--
D---

------LISSKDLASHLTDYDWNLFKRIHQVEMIH-------YIVGPQKF-HDVTTANLE
RVMRR---------FNELQYWV-----ATELCLCPEVGRR------AQLLRKFIKLAAHL
---------------KEQKNLNSFFAVMF-GVSNTAVSRLAKTWE---------------
----------HMMAKTVRV-LQ--------------------------------------

---------------------RCRG--QA----------------------HAPLSPLRN
RSPHRPEDPKAVRIST--
-----------Y---CLQS---
----PGWL-----AKSVDVSRGFPFPGGRL------------------------------

>Rapgef3_[Orcinus_orca]_466031722

---------------------M--------------------------------------

---------------K---------------------------------VSWPGES----
-----RWQVGLAVED-SSVLGAPPVGG----LPDVVPEGTLFNMVLRRMHRSRSCSYQ-L
LLE------------------------HQRP-S---------CIQ---------------
------------------GLR-------------------WT------------------
-----PLANSEESLDFSV-SLEQA-------------------S--------T-------
-------ERVL-------------RA---------------------GRQLH-RHLLATC
PN-L----------------IRDRKYH------L-RLH----------------RQC-CS
GR-----------------ELVDGILALG------------------------------L
----GVH--------SRSQAVGIC---QVLLDEGALC-----------------------
--------------H------------------------VKH-DWAFQDRDTQFYRFPGP
-------EPEP--AGLHEL---

---------EEEL------VEALALLSQRGPDAL-------------LTVALRKPPGQRT
DEELD-----LIFEELLHIKAVAHLS-NSVKRELA-AVLLFEPHSKAGTVL---------
--FSQGDKG--TSWYIIW
KGSVNVVTHGK-----GL-------------------------------VTTVHEG-DDF
GQLAL-----------VNDAPRA---ATIILREDNCHFLRVDKQDFNR------IIKD--
---V--------------

------------------------EAKTMRLEEHGKVVLVLER-------T------SQG
TG-----PSRP------------------PTPGRN------------R-YIVMSGTPEKI
LELLLEA------------MRPDSSAHDPT-ETF-------------------LS---D-
FLLTHSV--FM---------------PTAQLCAALL-----HHF------H---------
-----------------------------AEPV---GGSEQECSTYICNKRQQILRLVSQ
WVALYGPMLHTD-PVATSFLQ----KLSDL--VSRDA--RLCNLLR---EQ-WPERRR-H
HRL----ENGCGNA--SP------QMKAR----------------------NMP------
-----VW-----------LPSQD--------EP---------------------------
-----------------L--
---PSSN---

----CAIRVGDK--VP-----------------------------YD-------------
ICRPDHSVLTLQLPVTASVREVMA--------ALAQEDG-----WT-------KGQ--VL
VKVNSA----------------------G------DTVGLQPDAHGVATSLGLNERLFVV
NPQE---V-HKLT-PHPEQL---------------------------GP------TVGSA
EG--LDLVSTKDLAGQLTDHDWSLFNSIHQVELIH-------YVLGPQPL-RDVTTANLE
RFMRR---------FNELQYWV-----ATELCLCPVPGLR------AQLLRKFIKLAAHL
---------------KEQKNLNSFFAIMF-GLSNSAISRLAQTWE---------------
-------RLPHKVRKLYSA-LERLL--------------------------DPSWNH---
-RVYRL----ALTK-------LSPPLI-PFMP----------------------------
LLLKDMTFIHEGNH--TL-VE-----------------------------N-LINFE-KM
-------RM---MARAARMLHHCRSHSN------------------------VPLSPLRS
RVSHLHEDSQAARISTCSEQSLSTR-------S--------------AASTWA-----YV
QQLKVIDNQREL---SRLSREL
-----------EP---

>Rapgef3_[Oreochromis_niloticus]_542216384

--MHLFRTHNYQ-V
F----PD-----------------RCSVE-K--QTIR-----------------------

```
------------------GIS-------------------WT------------------
-----PLPEALNS---KD-TMKQ---------------------------FLS-------
-------DRVV-------------KA---------------------ARSVY-SVMIEKN
PG-L----------------IRDRKHH------L-KTY----------------RQC-CS
GK-----------------DLVDWLMTQN------------------------------E
----GLQ--------SRSQAVGIW---QVLVDEGILL-----------------------
--------------H------------------------VKH-ELNFLDKDTQFYRFQDS
-------QFGLNHISNEK------------------------------------------
DS----------------------------------------------------------
---------EDEL------QEALSLLSQLGPDAL-------------LTMILRKCPSQRS
PEDLE-----VIYEELLHVKAAAHLS-SSVRKELA-AVLVFESHAKAGTVL---------
--------------------------------------------FSQGDKG--TSWYIIW
KGSVNVITHGK-----GL-------------------------------VTTLHEG-EDF
GQLAL-----------LNEQPRA---ATIILREDNCHFLRVDKQDFIR------ILKD--
---------------------------------------------V--------------
------------------------------------------------------------
------------------------------------------------------------
------------------------EANTVRLEEHGKTVLVLEK-------S---------
AD----STEQG------------------GAGNSS------------K-YTVMSGTPEKI
LEHLLET------------IKLESNGNDAL-DC-------------------CVS---D-
FLLTHKV--FM---------------PSSQLCHALQ-----HHY------Q---------
-----------------------------AQLAE---GSEQEKTAYVLNIKQKVVKLVGQ
WVALFGLHLKED-PIALDFLE----RLKKD--MAANS--QLFNVLK---D--FRERRR--
TKV---SENGYQSL--S-------R-N-HQFD----------------------------
------W-----------FSNFE--------ES---------------------------
------------------------------------------------------------
---VGRV-----------------------------------------------------
------------------------------------------------------------
------------------------------------------------------------
------------------------------------------------------------
------------------------------------------------------------
----QPIRAHDK--VL-----------------------------YE-------------
IYRPDNKPLTLMLPVNTSVQEIMS--------ALGKTG---------------EDH--VL
IKMNST----------------------G------ERAQLKLDATAVYTALGLNERLFIC
TISE---M-EQLT-PLKEQQ---------------------------GP------EQGTT
DV--LEQMSSKDIASELTNYDWELFTAMHEVELVY-------YIFGRHKF-PGAITANLE
RFVRR---------FNEVQHWV-----LTELCLCEDLVKR------AMLLKKFIKIASVL
---------------KEQKNLNSFFAVMF-GLSNSAIQRLYKTWE---------------
-------RIPSKTKRIYCS-LERLM--------------------------DPSRNH---
-RAYRL----AIAK-------LSPPYI-PFMP----------------------------
LLLKDMTFIHEGNA--NY-VD-----------------------------K-LVNFE-KM
-------RM---LAKTVKIVRGCRSQPY------------------------VASSPQRG
LADRMFLEGAATRLSTYSDHALPLR-------T--------------STNIRH-----YI
QNLKVIDNQRKL-----------------------------------------TQLSRTI
-----------DC-----------------------------------------------
------------------------------------------------------------
------------------------------------------------------------
------------------------------------------------------------
------------------------------------------------------------
------------------------------------------------------------
------------------------------------------------------------
------------------------------------------------------------
------------------------------------------------------------
------------------------------------------------------------
```


>Rapgef3_[Ornithorhynchus_anatinus]_620972164

---I
LELLLEA------------VRPDSCVHDPT--------------------DTFLG---D-
FLLTHGI--FM---------------PSTQLCAALL--------------H---------
--------QYP---
------------------LLE---------------------------------------
--------NGGGNG--SS------QMKAGTGS----------------------------
-----IW-----------FPGLD--------ETS--------------------------

---PSGCL--

-----AIRAQDR--VP-----------------------------YA-------------
VCRPDHEVLTVLLPVTASVREVTA--------ALAQVDSR-----T--------GD-PVL
VKVNSS----------------------GEAVK------LPPDARGVASTLGLNERLFLV
SAQEPAVLVRRYP-PHPDQL---------------------------GP------NVGSA
EL--LDAVSSRDLASLLSEYDWGLFHCVHQVEPIL-------YVLGPQRL-RDITTANLE
RLMRR---------FNELQYWV-----VTELCLCPAPAAR------ARLLSRFIKLAAQ-

>Rapgef3_[Orycteropus_afer_afer]_634839967

---MVLRRMHRPRSCSYQ-L
LLE------------------------HQRP-S---------RIQ---------------
------------------GLR-------------------WT------------------
-----PLTDSEESLDFSV-SLEQA-------------------S--------T-------
-------ERVL-------------RA---------------------GKQLH-RHLLATC
PN-L----------------IRDRKYH------L-RLY----------------RQC-CS
GR-----------------ELVDGILALG------------------------------L
----GVH--------SRSQAVGIC---QVLLDEGALC-----------------------
--------------H------------------------VKH-DWTFQDREAQFYRFFGT
-------EPDP--TGAHEM---

---------EEEL------VEGIALLSQRGPDAL-------------LTVALRKSPGQRT
DEELD-----LIFEELLHIKAVAHLS-NSVKRELA-AVLQFEPHSKAGTVL---------
--FSQGDKG--TSWYIIW
KGSVNVVTHGK-----GL-------------------------------VTTLHEG-DDF
GQLAL-----------VNDAPRA---ATIIILREDNCHFLRVDKQDFNR-----IIKD--
---V--------------

------------------------EAKTMRLEEHGKVVLVLER-------T------SQG
SG-----PSHP------------------PTPGRN------------R-YTVMSGTPEKI
LELLLEA------------MRPDSSAHDPT-ETF-------------------LS---D-
FLLTHSV--FM---------------PSTQLCAALL-----HHF------H---------
-----------------------------AEPA---GGSEQERSTYVCNKRQQILRLVSQ
WVALYGSMLHTD-SVATSFLQ----KLSDL--VSRDA--RLSNLLR---EQ-WPERRR-H
HRL----ENGCGNA--SP------QLKAR----------------------SAP------
-----VW-----------LPSQD--------EP---------------------------
-----------------L--
---PSSN---

----CAIRVGDK--VP-----------------------------YD-------------
ICRPDHSVLTLQLPVTASVREVMA--------ALAQEDG-----WT-------KGQ--VL
VKVNSS----------------------G------DTIGLQPDARGVATSLGLNERLFVV
NPQE---V-DALT-PHPEQL---------------------------GP------AVGSA
EG--LDLVSTKDLAGQLTDHDWSLFNSIHQVELIH-------YVLGPQHL-RTVTTANLE

```
RFMRR---------FNELQYWV-----ATELCLCPVPSLR------AQLLRKFIKLAAHL
---------------KEQKNLNSFFAVMF-GLSNSAISRLAHTWE---------------
-------RLPHKVRKLYSA-LERLL--------------------------DPSWNH---
-RVYRL----ALTK-------LSPPVI-PFMP----------------------------
LLLKDMTFIHEGNH--TL-VE-----------------------------N-LINFE-KM
-------RM---MARAVRMLHQCRSHSN------------------------VPLSPLRS
RVSHLHEDSQAARISTCSEQSLSTR-------S--------------PANTWA-----YV
QQLKVIDNQREL-----------------------------------------SRLSREL
-----------EP-----------------------------------------------
------------------------------------------------------------
------------------------------------------------------------
------------------------------------------------------------
------------------------------------------------------------
------------------------------------------------------------
------------------------------------------------------------
------------------------------------------------------------
------------------------------------------------------------
------------------------------------------------------------
------------------------------------------------------------
---
>Rapgef3_[Oryctolagus_cuniculus]_1040227932
------------------------------------------------------------
------------------------------------------------------------
------------------------------------------------------------
-----------------------------------------GRGPAAAVHG-CPPSF---
--SSHRWAAQVRAAC-KWAEPAAQVGG----LADLAPGAXLLDAV-RRMRRPRSCSYQ-L
LLA------------------------HGRP-G---------RVQ---------------
------------------GLR-------------------WT------------------
-----PLTTSQESLDFSG-SLEQA-------------------S--------T-------
-------ERVL-------------RA---------------------GKQLH-RHLLAAC
PG-L----------------IRDRKHH------L-RLY----------------RQC-CS
GR-----------------ELVDGILALG------------------------------L
----AVH--------SRSQAVGVC---QVLLDEGALC-----------------------
--------------H------------------------VRH-DWAFQDRDAQFYRFPGP
-------EPEP--AGRPEV-----------------------------------------
------------------------------------------------------------
---------EEDL------LEAVALLAQRGPDAL-------------LTVALRKPPAQRT
HEELE-----LIFEELLHIKAVAHLS-NSVKRELA-AVLLXEPHSKAGTVL---------
--------------------------------------------FSQGDKG--TSWYIIW
KGSVNVVTHGK-----GL-------------------------------VTTLHEG-DDF
GQLAL-----------VNDAPRA---ATIVLREDSCHFLRVDKQDFNR------IIKD--
---------------------------------------------V--------------
------------------------------------------------------------
------------------------------------------------------------
------------------------EAKTMRLEEHGKVVLVLER-------A------SQG
AG-----PACP------------------PTPGRT------------R-YTVMSGTPEKI
LELLLEA------------MRPDSSTHDPT-ETF-------------------LS---D-
FLLTHSV--FM---------------PSAQLCPALL-----HHF------H---------
-----------------------------AEPA---GSSEQEHSSYVCNKRQQILRLVSQ
WVALYGPMLHVD-PVASSFLQ----RLSEL--VSEDV--QLSSLLR---GQ-WPERRR-H
HRL----ENGCGNA--SP------QTKAQ----------------------NAP------
-----VW-----------LPGHD--------EL---------------------------
-----------------L------------------------------------------
---ASSN-----------------------------------------------------
```


----CAIRAGDK-GGP-----------------------------YD-------------
IYRPDHSGLTLQLPVTASVREVLV--------ALAQEDG-----GT-------RGQ--VL
VKVNSA----------------------G------DAVGLQPDARGVATSLGLNERLFVV
DPQQ---V-HELS-PHPEQL---------------------------GP------TVGSA
EA--LELVSAKDLASQLTEQDRDLFYSVHQVELIH-------YVLGPQHL-RDVTTANLE
RFMRR---------FNELQYWV-----ATELCLCLLPGPR------AQLLRKFIKLAAHL
---------------KEQKNLNSFFAVMF-GLSNSAISRLAHTWE---------------
-------RLPQKVRKLHSA-LERLL--------------------------DPSWNH---
-RAYRV----ALAK-------LSPPII-PFMP----------------------------
LLLKDMTFIHEGNR--TL-VE-----------------------------N-LINFE-KM
-------RM---MARAVRVLQHCRGHGH------------------------APLSPLRT
RASHVHEDSQASRISTCSEQSLSTR-------S--------------PASTWA-----YV
QQLKVIDNQREL---SRLSREL
-----------EP---

>Rapgef3_[Oryzias_latipes]_765126149

--------------------MKQ---------------------------FLS-------
-------DRVV-------------KA---------------------ARSVY-SVMNEKN
PG-L----------------IRDRKHH------L-KTH----------------RQC-CS
GK-----------------ELVDWLMKQN------------------------------E
----CLQ--------SRSQAVGMW---QVLVDEGILV-----------------------
--------------H------------------------VKH-ELNFQDKEAQFYRFQDS
-------DFGLSHIPNEK--
ES--
---------EEEL------QEALALLSQLGPDAL-------------LTMILRKTPSQRT
PEDIE-----VIYEELLHVKAAAHLS-TSVRKELA-AVLVFESHAKAGTVL---------
--FSQGDKG--TSWYIIW
KGSVNVITHGK-----GI-------------------------------VTTLHEG-EDF
GQLAL-----------LNDAPRA---ATIILREDNCHFLRVDKQDFIR------ILKD--
---V--------------

------------------------EANTVRLEEHGKTVLVLEQ-------S---------

TQ----SARQG------------------SAGTNS------------K-YTVMSGTPEKI
LEHLLET------------IKLDSNGYDPI-DP-------------------CVI---D-
FLLTHKV--FM---------------PTSQLCPALQ-----HHY------Q---------
-----------------------------AELSE---GSEQEKDAYIIDTKQKVVKLISH
WVAVYGFLLKED-PVAMDFLEA----MKKE--VAADS--RLSGMLK---EQ-YRERRR--
TKV---LENGHHSL--S-------R-N-QHFD----------------------------
------W-----------FSNCD--------EA---------------------------

---LGRI---

----QPIKAKDK--IL-----------------------------YE-------------
IYRPDHKALTLMLPVDSPVQDVLS--------GVVEPG---------------GGH--VL
VKMNST----------------------G------DRAELKLEASAVYTALGLNERLFIC
TSSE---V-EHLR-PLKEQQ---------------------------GP------EQGTT
DI--LEQIGSKDMAAELTNFDWELFTAVHEVELVY-------YIFGRNKF-PGAITANLE
RFVRH---------FNKLQYWV-----VTELCLCEDLMKR------ALLLKKFIKIASVL
---------------KEQKNLNSFFAVMF-GLSNSAVQRLYKTWE---------------
-------RIPSKIKRIYCT-YERLM--------------------------DPSRNH---
-RAYRL----AVAK-------LNPPYI-PFMP----------------------------
LLLKDMTFIHEGNP--SY-VD-----------------------------K-LVNFE-KM
-------RM---LAKTVNIVRGCRSQPY------------------------VPSSPQRG
LADRMFLEGPATRLSTYSDHSLPQR-------S--------------PTNIRL-----YI
QNLKVIDNQRQL---TQRSRAL
-----------EC---

>Rapgef3_[Otolemur_garnettii]_831232292

---MVLRRMHRPRSCSYQ-L
LLE------------------------HRRP-S---------RIQ---------------
------------------GLR-------------------WT------------------
-----PLTNSEESLDFSV-SLEQA-------------------S--------T-------
-------ERVL-------------RA---------------------GKQLQ-RHLLATC
PN-L----------------IRDRKYH------L-RLY----------------RQC-CS
GR-----------------ELVDGILSLG------------------------------L
----GVH--------SRSQAVGIC---QVLLDEGALC-----------------------
--------------H------------------------VKH-DWTFQDRDAQFYRFPGP
-------EPEP--TGAHEM---

---------EEEL------VEAMALLSQRGPDAL-------------LTVALRKPPGQRT
DEELD-----LIFEELLHIKAVAHLS-NSVKRELA-AVLLFEPHSKAGTVL---------
--FSQGDKG--TSWYIIW
KGSVNVVTHGK-----GL-------------------------------VTTLHEG-DDF
GQLAL-----------VNDAPRA---ATIILREDNCHFLRVDKQDFNR------IIKD--
---V--------------

------------------------EAKTIRLEEHGKVVLVLER-------A------SQG
AG-----SSRP------------------PTPGRN------------R-YTVMSGTPEKI
LELLLEA------------MRPDSSAHDPT-ETF-------------------LS---D-
FLLTHSV--FM---------------PSAQLCAALL-----HHF------H---------
-----------------------------AEPT---GGSEQERSTYICNKRQQILQLVSQ
WVALYGSMLHAD-PVATSFLQ----KLSDL--VSRDV--RLSNLLR---EQ-WPERRR-H
HRL----ENGYGNA--SP------QMKAP----------------------NVP------
-----IW-----------LPSQD--------EP---------------------------
-----------------L--
---PSSN---

----CAIRAGDK--VS-----------------------------YD-------------
ICRPDHSVLTLQLPVTASVREVMV--------ALAQEDS-----WT-------KGQ--VL
VKINSA----------------------G------HVTGLQPDARGVATSLGLNERLFVV
NPQE---V-HVLT-PHPEQL---------------------------GP------SVGSA
EG--LDLVSAKDLAGQLTDHDWNLFNSIHQVELIH-------YALGPQHL-RDVTTANLE
RFMRR---------FNELQYWV-----ATELCLCPVPGPR------AQLLRKFIKLAAHL
---------------KEQKNLNSFFAVMF-GLSNSAISRLAHTWE---------------
-------RLPHKVRKLYSA-LERLL--------------------------DPSWNH---
-RVYRL----ALAK-------LSPPAI-PFMP----------------------------
LLLKDMTFIHEGNH--TI-VE-----------------------------N-LINFE-KM
-------RM---MARAVRMLHHCRGHSS------------------------VPLSPLRT
RVSHFHEDNQVARISTCSEQSLSTR-------S--------------PASTWA-----YV
QQLKVINNQREL---SRLSREL
-----------EP---

>Rapgef3_[Ovis_aries_musimon]_803304007

---MVLRRMYRSRSCSYQ-L
LLE------------------------HQRP-S---------CIQ---------------

```
------------------GLR-------------------WT------------------
-----PLTSSEESLDFSV-SLEQA-------------------S--------T-------
-------ERVL-------------RA---------------------GKQLH-RHLLANC
PN-L----------------IRDRKYH------L-RLH----------------RQC-CS
GR-----------------ELVDGILALG------------------------------L
----GVH--------SRSQAVGIC---QVLLDEGALC-----------------------
--------------H------------------------VKH-DWAFQDRDTQFYRFPGP
-------EPEP--VGAHEL-----------------------------------------
------------------------------------------------------------
---------EEEL------VEALALLSQRGPDAL-------------LTVALRKPPGQRT
DEELD-----LIFEELLHIKAVAHLS-NSVKRELA-AVLLFEPHSKAGTVL---------
--------------------------------------------FSQGDKG--TSWYIIW
KGSVNVVTHGK-----GL-------------------------------VTTLHEG-DDF
GQLAL-----------VNDAPRA---ATIILGEDNCHFLRVDKQDFNR------IIKD--
---------------------------------------------V--------------
------------------------------------------------------------
------------------------------------------------------------
------------------------EAKTMRLEEHGKVVLVLER-------T------SQG
TG-----PSRP------------------PTPGGN------------R-YTVMSGTPEKI
LELLLEA------------MRPDSSAHDPT-ETF-------------------LS---D-
FLLTHSV--FM---------------PTAQLCAALL-----HHF------H---------
-----------------------------AEPA---GGSEQERSSYVCNKRQQILRLVSQ
WVTLYGPTLRTD-PVATSFLK----KLSDL--VSRDA--RLSNLLR---EQ-WPERRR-H
HRL----ENGCGNA--SP------QMKAR----------------------NVP------
-----VW-----------LPSQD--------EP---------------------------
-----------------L------------------------------------------
---PSNS-----------------------------------------------------
------------------------------------------------------------
------------------------------------------------------------
------------------------------------------------------------
------------------------------------------------------------
----CAIRVGDK--VP-----------------------------YD-------------
ICRPDHSVLTLQLPVTASVREVMA--------ALAQEDG-----WT-------KGQ--VL
VKVNSA----------------------G------DAIGLQPDARGVATSLGLNERLFVV
NPQE---V-HKLT-PHPEQL---------------------------GP------TVGSA
EG--LDLVSTKDLAGQLTDHDWSLFNSIHQVELIH-------YVLGPQHL-RDVTTANLE
RFMRR---------FNELQYWV-----ATELCLCSVPGLR------AQLLRKFIKLAAHL
---------------KEQKNLNSFFAVMF-GLSNSAISRLAHTWE---------------
-------RLPHKVRKLYSA-LERLL--------------------------DPSWNH---
-RVYRL----ALTK-------LSPPLI-PFMP----------------------------
LLLKDMTFIHEGNH--TL-VE-----------------------------N-LINFE-KM
-------RM---MARAARMLHHCRSHSN------------------------VPLSPLRS
RVSHLHEDSQAVRVSTCSEQSLSTR-------S--------------PASTWA-----YV
QQLKVIDNQREL-----------------------------------------SRLSREL
-----------EP-----------------------------------------------
------------------------------------------------------------
------------------------------------------------------------
------------------------------------------------------------
------------------------------------------------------------
------------------------------------------------------------
------------------------------------------------------------
------------------------------------------------------------
------------------------------------------------------------
------------------------------------------------------------
```


>Rapgef3_[Ovis_aries]_803044449

---------------------M--------------------------------------

---------------K---------------------------------VGWPGES----
-----RWQVGLAVEG-DSILGAPPLGG----LPDVVPEGTLLNMVLRRMYRSRSCSYQ-L
LLE------------------------HQRP-S---------CIQ---------------
------------------GLR-------------------WT------------------
-----PLTSSEESLDFSV-SLEQA-------------------S--------T-------
-------ERVL-------------RA---------------------GKQLH-RHLLANC
PN-L----------------IRDRKYH------L-RLH----------------RQC-CS
GR-----------------ELVDGILALG------------------------------L
----GVH--------SRSQAVGIC---QVLLDEGALC-----------------------
--------------H------------------------VKH-DWACQDRDTQFYRFPGP
-------EPEP--VGAHEL---

---------EEEL------VEALALLSQRGPDAL-------------LTVALRKPPGQRT
DEELD-----LIFEELLHIKAVAHLS-NSVKRELA-AVLLFEPHSKAGTVL---------
--FSQGDKG--TSWYIIW
KGSVNVVTHGK-----GL-------------------------------VTTLHEG-DDF
GQLAL-----------VNDAPRA---ATIILGEDNCHFLRVDKQDFNR------IIKD--
---V--------------

------------------------EAKTMRLEEHGKVVLVLER-------T------SQG
TG-----PSRP------------------PTPGGN------------R-YTVMSGTPEKI
LELLLEA------------MRPDSSAHDPT-ETF-------------------LS---D-
FLLTHSV--FM---------------PTAQLCAALL-----HHF------H---------
-----------------------------AEPA---GGSEQERSSYVCNKRQQILRLVSQ
WVTLYGPTLRTD-PVATSFLK----KLSDL--VSRDA--RLSNLLR---EQ-WPERRR-H
HRL----ENGCGNA--SP------QMKAR----------------------NVP------
-----VW-----------LPSQD--------EP---------------------------
-----------------L--
---PSNS---

----CAIRVGDK--VP-----------------------------YD-------------
ICRPDHSVLTLQLPVTASVREVMA--------ALAQEDG-----WT-------KGQ--VL
VKVNSA----------------------G------DAIGLQPDARGVATSLGLNERLFVV
NPQE---V-HKLT-PHPEQL---------------------------GP------TVGSA
EG--LDLVSTKDLAGQLTDHDWSLFNSIHQVELIH-------YVLGPQHL-RDVTTANLE
RFMRR---------FNELQYWV-----ATELCLCSVPGLR------AQLLRKFIKLAAHL
---------------KEQKNLNSFFAVMF-GLSNSAISRLAHTWE---------------
-------RLPHKVRKLYSA-LERLL--------------------------DPSWNH---
-RVYRL----ALTK-------LSPPLI-PFMP----------------------------
LLLKDMTFIHEGNH--TL-VE-----------------------------N-LINFE-KM
-------RM---MARAARMLHHCRSHSN------------------------VPLSPLRS
RVSHLHEDSQAVRVSTCSEQSLSTR-------S--------------PASTWA-----YV
QQLKVIDNQREL---SRLSREL
-----------EP---

>Rapgef3_[Pan_paniscus]_397510893

---------------------M--------------------------------------

---------------K---------------------------------VGWPGES----
-----CWQVGLAVED-SPALGAPRVGA----LPDVVPEGTLLNMVLRRMHRPRSCSYQ-L
LLE------------------------HQRP-S---------CIQ---------------
------------------GLR-------------------WT------------------
-----PLTNSEESLDFSE-SLEQA-------------------S--------T-------
-------ERVL-------------RA---------------------GRQLH-RHLLATC
PN-L----------------IRDRKYH------L-RLY----------------RQC-CS
GR-----------------ELVDGILALG------------------------------L
----GVH--------SRSQVVGIC---QVLLDEGALC-----------------------
--------------H------------------------VKH-DWAFQDRDAQFYRFPGP
-------EPEP--VGTHEM---

---------EEEL------AEAVALLSQRGPDAL-------------LTVALRKPPGQRT
DEELD-----LIFEELLHIKAVAHLS-NSVKRELA-AVLLFEPHSKAGTVL---------
--FSQGDKG--TSWYIIW
KGSVNVVTHGK-----GL-------------------------------VTTLHEG-DDF
GQLAL-----------VNDAPRA---ATIIILREDNCHFLRVDKQDFNR------IIKD--
---V--------------

------------------------EAKTMRLEEHGKVVLVLER-------A------SQG
AG-----PSRP------------------PTPGRN------------R-YTVMSGTPEKI
LELLLEA------------MGPDSSAHDPT-ETF-------------------LS---D-
FLLTHRV--FM---------------PSAQLCAALL-----HHF------H---------
-----------------------------VEPA---GGSEQERSTYVCNKRQQILRLVSQ
WVALYGSMLHTD-PVATSFLQ----KLSDL--VGRDT--RLSNLLR---EQ-WPERRR-C
HRL----ENGCGNA--SP------QMKAR----------------------NLP------
-----VW-----------LPNQD--------EP---------------------------
-----------------L--
---PGSS---

----CAIRVGDK--VP-----------------------------YD-------------
ICRPDHSVLTLQLPVTASVREVMA--------ALAQEDG-----WT-------KGQ--VL
VKVNSA----------------------G------DAIGLQPDARGVATSLGLNERLFVV
NPQE---V-HELI-PHPDQL---------------------------GP------TVGSA
EG--LDLVSAKDLAGQLTDHDWSLFNSIHQVELIH-------YVLGPQHL-RDVTTANLE

RFMRR---------FNELQYWV-----ATELCLCPVPGPR------AQLLRKFIKLAAHL
---------------KEQKNLNSFFAVMF-GLSNSAISRLAHTWE---------------
-------RLPHKVRKLYSA-LERLL--------------------------DPSWNH---
-RVYRL----ALAK-------LSPPVI-PFMP----------------------------
LLLKDMTFIHEGNH--TL-VE-----------------------------N-LINFE-KM
-------RM---MARAARMLHHCRSHNP------------------------VPLSPLRS
RVSHLHEDSQVARISTCSEQSLSTR-------S--------------PASTWA-----YV
QQLKVIDNQREL---SRLSREL
-----------EP---

>Rapgef3_[Pan_troglodytes]_1034096392

---------------------M--------------------------------------

---------------A---------------------QREEIKRPLWETVGWPGES----
-----CWQVGLAVED-SPALGAPRVGA----LPDVVPEGTLLNMVLRRMHRPRSCSYQ-L
LLE------------------------HQRP-S---------CIQ---------------
------------------GLR-------------------WT------------------
-----PLTNSEESLDFSE-SLEQA-------------------S--------T-------
-------ERVL-------------RA---------------------GRQLH-RHLLATC
PN-L----------------IRDRKYH------L-RLY-----------------RQC-CS
GR-----------------ELVDGILALG------------------------------L
----GVH--------SRSQVVGIC---QVLLDEGALC-----------------------
--------------H------------------------VKH-DWAFQDRDAQFYRFPGP
-------EPEP--VGTHEM---

---------EEEL------AEAVALLSQRGPDAL-------------LTVALRKPPGQRT
DEELD-----LIFEELLHIKAVAHLS-NSVKRELA-AVLLFEPHSKAGTVL---------
--FSQGDKG--TSWYIIW
KGSVNVVTHGK-----GL-------------------------------VTTLHEG-DDF
GQLAL-----------VNDAPRA---ATIILREDNCHFLRVDKQDFNR------IIKD--
---V--------------

------------------------EAKTMRLEEHGKVVLVLER-------A------SQG
AG-----PSRP------------------PTPGRN------------R-YTVMSGTPEKI
LELLLEA------------MGPDSSAHDPT-ETF-------------------LS---D-
FLLTHRV--FM---------------PSAQLCAALL-----HHF------H---------
-----------------------------VEPA---GGSEQERSTYVCNKRQQILRLVSQ
WVALYGSMLHTD-PVATSFLQ----KLSDL--VGRDT--RLSNLLR---EQ-WPERRR-C
HRL----ENGCGNA--SP------QMKAR----------------------NLP------
-----VW-----------LPNQD--------EP---------------------------
-----------------L--
---PGSS---

----CAIRVGDK--VP-----------------------------YD-------------
ICRPDHSVLTLQLPVTASVREVMA--------ALAQEDG-----WT-------KGQ--VL
VKVNSA----------------------G------DAIGLQPDARGVATSLGLNERLFVV
NPQE---V-HELI-PHPDQL---------------------------GP------TVGSA
EG--LDLVSAKDLAGQLTDHDWSLFNSIHQVELIH-------YVLGPQHL-RDVTTANLE
RFMCR---------FNELQYWV-----ATELCLCPVPGPR------AQLLRKFIKLAAHL
---------------KEQKNLNSFFAVMF-GLSNSAISRLAHTWE---------------
-------RLPHKVRKLYSA-LERLL--------------------------DPSWNH---
-RVYRL----ALAK-------LSPPVI-PFMP----------------------------
LLLKDMTFIHEGNH--TL-VE-----------------------------N-LINFE-KM
-------RM---MARAARMLHHCRSHNP------------------------VPLSPLRS
RVSHLHEDSQVARISTCSEQSLSTR-------S--------------PASTWA-----YV
QQLKVIDNQREL---SRLSREL
-----------EP---

>Rapgef3_[Panthera_tigris_altaica]_591291251

---------------------M--------------------------------------

---------------K---------------------------------VGWPGES----
-----RWQVGLAVED-SPALGASQVGG----LPDVVPEGTLLNMVLKRMHRPRSCSYQ-L
LLE------------------------HQRP-S---------CIQ---------------
------------------GLR-------------------WT------------------
-----PLTDSEESLDFSV-SLEQA-------------------S--------T-------
-------ERVL-------------RA---------------------GKQLH-RHLLATC
PT-L----------------IRDRKYH------L-RLY----------------RQC-CS
GR-----------------ELVDGILALS------------------------------L
----GVH--------SRNQAVGIC---QVLLDEGALC-----------------------
--------------H------------------------VKH-DWTFQDRDTQFYRFPGP
-------EPEP--VGVHEL---

---------EEEL------VEAMALLSQRGPDAL-------------LTVALRKPPGQRT
DEELD-----LIFEELLHIKAVAHLS-NSVKRELA-AVLLFEPHSRAGTVL---------
--FSQGDKG--TSWYIIW
KGSVNVVTHGK-----GL-------------------------------VTTLHEG-DDF
GQLAL-----------VNDAPRA---ATIILREDNCHFLRVDKQDFNR------IIKD--
---V--------------

------------------------EAKTMRLEEHGKVVLVLER-------A------SQG

AG-----PSRP------------------PTPGRN------------R-YTVMSGTPEKI
LELLLEA------------MRPDSSAHDPT-ETF-------------------LS---D-
FLLTHSV--FM---------------PSAQLCAALL-----HHF------H---------
-----------------------------AEPS---GGSEQEHSTYVCNKRQQVLRLVSQ
WVALYGPMLYAD-PVATSFLQ----KLSDL--VSRDA--RLCILLR---EQ-WPERRK-H
HRM----ENGCGNA--SP------QMKAR----------------------NMP------
-----VW-----------LPGQD--------QP---------------------------
-----------------L--
---PSSN---

----CAIRVGDK--VP-----------------------------YD-------------
IYRPDHSVLTLKLPVTASVREVMA--------ALAQEDG-----WT-------KGQ--EL
VKVNSA----------------------G------DAIGLQPDARGVATSLGLNERLFVV
NPQE---V-RELT-PLPEQL---------------------------GP------SVGSA
EG--LDLVSAKDLAGQLTDHDWNLFNSIHQVELIH-------YVLGPQHL-RDVTTANLE
RFMRR---------FNELQYWV-----ATELCLCPVPGLR------AQLLRKFIKLAAHL
---------------KEQKNLNSFFAVMF-GLSNSAISRLAHTWE---------------
-------RLPHKVRKLYSA-LERLL--------------------------DPSWNH---
-RVYRL----ALTK-------LSPPII-PFMP----------------------------
LLLKDMTFIHEGNH--TL-VE-----------------------------N-LINFE-KM
-------RM---MARAVRMLHHCRSHSN------------------------VPLSPLRS
RVSHLHEDSQTARTSTCSEQSLNTR-------S--------------PASTWA-----YV
QQLKVIDNQREL---SRLSREL
-----------EP---

>Rapgef3_[Pantholops_hodgsonii]_556771829

---------------------M--------------------------------------

---------------T-SELACRVAP-HAVGAARGTAQKEEITWQVWEPVSWPGES----
-----RWQVGLAVEG-DSILGAPPLGG----LPDVVPEGTLLNMVLRRMYRSRSCSYQ-L
LLE------------------------HQRP-S---------CIQ---------------
------------------GLR-------------------WT------------------
-----PLTSSEESLDFSV-SLEQA-------------------S--------T-------
-------ERVL-------------RA---------------------GKQLH-RHLLANC
PN-L----------------IRDRKYH------L-RLH----------------RQC-CS
GR-----------------ELVDGILALG------------------------------L
----GVH--------SRSQAVGIC---QVLLDEGALC-----------------------
--------------H------------------------VKH-DWAFQDRDTQFYRFPGP
-------EPEP--VGAHEL---

---------EEEL------VEALALLSQRGPDAL-------------LTVALRKPPGQRT
DEELD-----LIFEELLHIKAVAHLS-NSVKRELA-AVLLFEPHSKAGTVL---------
--FSQGDKG--TSWYIIW
KGSVNVVTHGK-----GL-------------------------------VTTLHEG-DDF
GQLAL-----------VNDAPRA---ATIILGEDNCHFLRVDKQDFNR------IIKD--
---V--------------

------------------------EAKTMRLEEHGKVVLVLER-------T------SQG
TG-----PSRP------------------PTPGGN------------R-YTVMSGTPEKI
LELLLEA------------MRPDSSAHDPT-ETF-------------------LS---D-
FLLTHSV--FM---------------PTAQLCAALL-----HHF------H---------
-----------------------------AEPA---RGSEQERSSYVCNKRQQILRLVSQ
WVTLYGPTLRTD-PVATSFLK----KLSDL--VSRDA--RLSNLLR---EQ-WPERRR-H
HRL----ENGCGNA--SP------QMKAR----------------------NVP------
-----VW-----------LPSQD--------EP---------------------------
-----------------L--
---PSSN---

----CAIRVGDK--VP-----------------------------YD-------------
ICRPDHSVLTLQLPVTASVREVMA--------ALAQEDG-----WT-------KGQ--VL
VKVNSA----------------------G------DAIGLQPDARGVATSLGLNERLFVV
NPQE---V-HKLT-PHPEQL---------------------------GP------TVGSA
EG--LDLVSTKDLAGQLTDHDWSLFNSIHQVELIH-------YVLGPQHL-RDVTAANLE
RFMRR---------FNELQYWV-----ATELCLCSVPGLR------AQLLRKFIKLAAHL
---------------KEQKNLNSFFAVMF-GLSNSAISRLAHTWE---------------
-------RLPHKVRKLYSA-LERLL--------------------------DPSWNH---
-RVYRL----ALTK-------LSPPLI-PFMP----------------------------
LLLKDMTFIHEGNH--TL-VE-----------------------------N-LINFE-KM
-------RM---MARAARMLHHCRSHSN------------------------VPLSPLRS
RVSHLHEDSQAVRVSTCSEQSLSTR-------S--------------PASTWA-----YV
QQLKVIDNQREL---SRLSREL
-----------EP---

>Rapgef3_[Papio_anubis]_402885758

---------------------M--------------------------------------

---------------K---------------------------------VGWPGES----
-----CWQVGLAVED-SPALGAPRVGP----LPDVVPEGTLLNMVLRKMHRPRSCSYQ-L
LLE------------------------HQRP-S---------CIQ---------------

```
------------------GLR-------------------WT------------------
-----PLTNSEESLDFSE-SLEQA-------------------S--------T-------
-------ERVL-------------RA---------------------GRQLH-RQLLATC
PN-L----------------IRDRKYH------L-RLY----------------RQC-CS
GR-----------------ELVDGILALG------------------------------L
----GVH--------SRSQVVGIC---QVLLDEGALC-----------------------
--------------H------------------------VKH-DWAFQDRDAQFYRFPGP
-------EPEPEPVGAHEM-----------------------------------------
------------------------------------------------------------
---------EEEL------AEAVALLSQRGPDAL-------------LTVALRKPPGQRT
DEELD-----LIFEELLHIKAVAHLS-NSVKRELA-AVLLFEPHSKAGTVL---------
--------------------------------------------FSQGDKG--TSWYIIW
KGSVNVVTHGK-----GL-------------------------------VTTLHEG-DDF
GQLAL-----------VNDAPRA---ATIILREDNCHFLRVDKQDFNR------IIKD--
---------------------------------------------V--------------
------------------------------------------------------------
------------------------------------------------------------
------------------------EAKTMRLEEHGKVVLVLER-------A------SQG
AG-----PSRP------------------PTPGRN------------R-YTVMSGTPEKI
LELLLEA------------MGPDSSAHDPT-ETF-------------------LS---D-
FLLTHRV--FM---------------PSAQLCAALL-----HHF------H---------
-----------------------------AEPA---GGSEQERSTYICNKRQQILRLVSQ
WVALYGSMLHTD-PVATSFLQ----KLSDL--VGRDA--RLSNLLR---EQ-WPERRR-H
HRL----ENGCGNA--SP------QMKAR----------------------NLP------
-----VW-----------LPNQD--------EP---------------------------
-----------------L------------------------------------------
---PGSS-----------------------------------------------------
------------------------------------------------------------
------------------------------------------------------------
------------------------------------------------------------
------------------------------------------------------------
----CAIRVGDK--VP-----------------------------YD-------------
ICLPDHSVLTLQLPVTASVREVMA--------ALAQEDG-----WT-------KGQ--VL
VKVNSS----------------------G------DAIGLQPDARGVATSLGLNERLFVV
NPQE---V-HELT-PHPDQL---------------------------GP------TVGSA
EG--LDLVSAKDLAGQLTDHDWSLFNSIHQVELIY-------YALGPQHL-RDVTTANLE
RFMRR---------FNELQYWV-----ATELCLCPVPGPR------AQLLRKFIKLAAHL
---------------KEQKNLNSFFAVMF-GLSNSAISRLAHTWE---------------
-------RLPHKVRKLYSA-LERLL--------------------------DPSWNH---
-RVYRL----ALAK-------LSPPVI-PFMP----------------------------
LLLKDMTFIHEGNH--TL-VE-----------------------------N-LINFE-KM
-------RM---MARAARMLHHCRSHNP------------------------VPLSPLRS
RVSHLHEDSQVARISTCSEQSLSTR-------S--------------PASTWA-----YV
QQLKVIDNQREL-----------------------------------------SRLSREL
-----------EP-----------------------------------------------
------------------------------------------------------------
------------------------------------------------------------
------------------------------------------------------------
------------------------------------------------------------
------------------------------------------------------------
------------------------------------------------------------
------------------------------------------------------------
------------------------------------------------------------
------------------------------------------------------------
```

```
------------------------------------------------------------
---
>Rapgef3_[Parus_major]_998744296
------------------------------------------------------------
------------------------------------------------------------
------------------------------------------------------------
-----------------------------------------------MGACSEGTS----
GWDG-NEDGERRRRSHDAPVPPLSF--HLLR--VFHLLTQNLPSGPSAL-PSPGSSC---
GVWVLDSGSAEF--PPPRV-----------KSWGCQG-------------PPTPE-GGTA
EPPQP-PTPSPLVFQ--------------------------T------------------
-----PLLD-------SESSLDCGH------------------SITQAS---P-------
-------EKIW-------------RAGKLLF---------------A----H---LSTSR
PG-L----------------IRDHKHH------L-RHH----------------RQC-CS
GK-----------------ELVDWLLSSG------------------------------A
----AVQ--------TRGQAVGIG---QVLLXVS--------------------------
------------------------------------------------------------
------------------------------------------------------------
------------------------------------------------------------
------------------------------------------------------------
---------------------------------------------RIQGI----------
-------------------------------------------LWGSGECG--E------
----RVPDPGN-----PL-------------------------------GEWGHEG-DDF
GQLAL-----------VNDAPRA---ATILLREDNCHFLRVDKQDFNR------ILKD--
---------------------------------------------V--------------
------------------------------------------------------------
------------------------------------------------------------
------------------------EANTVRLKEHGKVVLVLQKDL-------------QG
G------GGQA------------------ASARSS-------------------------
------------------------------------------------------------
----------R---------------VKRELAAVLMFEG---HQ------R---------
-----------------------------AGTVL---FSQGDKGT--------------S
WYIIW---------------K----GSVN--VVTHG---KLGGLGQ---EQ-GQERRR--
PRA---MENGDGSV--SP------QPKPRS---------------------SGM------
------W-----------LGTPE--------EA---------------------------
----------------IL------------------------------------------
----DSTS-AL-----------------RAQDK---------------------------
------------------------------------------------------------
------------------------------------------------------------
------------------------------------------------------------
------------------------------------------------------------
--------------VP-----------------------------YE-------------
IFRADHSCLVSVLPVNASVRDVLR--------ALAPRLG-------------RDRE-HVL
VKVNSA----------------------GERAV------LPQDAVGVFTALGLNERLFVV
TADE---L-GNLT-PHPEQL---------------------------GP------HAGSS
DS--LDLISSKDLASHLTDYDWNLFKSIHQVEMIH-------YIVGPQKF-HEVTTANLA
RVMRR---------FNELQFWV-----ATELCLCPELGRR------AQLLRKFIKLAAQC
---------------P--------------------------------------------
---------PH------------AQ--------------------------DPSWNH---
-RVYRL----AVAK-------MSPPII-PFMPL-LLK-----------------------
----DMTFIHEGNR--TLA------------------------------EN-LINFE-KM
-------HM---MAKTVRVLQRCRG--HA----------------------HAPLSPLRN
RSPHRPEDPKAVRIST--------------------------------------------
-----------C-----------------------------------------SEQSLSV
RNPVSTWAY-LQHLRAIDSQRELLRLSRDLEP----------------------------
```


>Rapgef3_[Pelodiscus_sinensis]_946688206

---MQVAWGAQGRW--
-HVG-LGPQD-------------------------------SPAQLHRL-----------
GDPTAEGHRLYL--LVKKM--------HLFRSYSYQI-------------RLEGE-AGSL
PSIQG-------IRW--------------------------K------------------
-----PLPD-------SESAPDFGQ------------------SLTQAP---A-------
-------EKIW-------------RAGKLLY---------------S----H---LMSTR
PG-L----------------IRDHKHH------L-RHH----------------RQC-LS
GK-----------------ELVDWLLSAG------------------------------L
----SAQ--------TRGQAIGVC---QVLVDGGV-------------------------
---KDKDAQFYRFAEG
E-----LSPEP---GTWGT---

----------EEL------LEALAFLAQLGPDAL-------------LTMALRKP-----
ED------------------AEGG-E-LPRQAGLA-SLLLFESHPKAGTV----------
---LFSQGDKG--TSWYIIW
KGSVNVVTHGK-----GL-------------------------------VTTLHEG-DDF
GQL---------------DAPRA---ATIILREDNCHFLRVDPQHXHW------VLXD--
---V--------------

------------------------EANTMRLKEHGKVVLVLEKNL-------------QG
WG----SGSPA------------------PSAGGR-KP------GRAG-YSVMAGTPDKI
LEYLLEA------------MRLDATFSDPL-D-------------------TLIG---D-
FLLTYKV--FM---------------PTAQLCRALL-----HHF------H---------
-----------------------------AEPSE---GSAQEKAVYSLSKRQKIL-AXSQ
WVLLYGHLLQAE-PSAMTLLQ----NLSD--FASRDP--GLCSSLQ---DQ-TQDRRR--
HRA---LENGGGSA--SP------QPKVRS---------------------TVN------
------W-----------FSTLE--------DA---------------------------
----------------AL--
----SNSY-TI-----------------GAQDKGSSSAS--VCHRKPAAAAGA-------
-------QTDRHLLPPHH---RDSFHCLPAHTTDSL--LRQPEVQ---------------

---TDRHL-----LPP--
------------PQVP-----------------------------YE-------------
VHRADPPCLTVVLPVNASVRDVLQ--------ALACQEG-------------EHGE-RVL
VKVNSA----------------------GDWPG------LPMDTVGVYTSLGLNERLFVV
DVQE---L-PTLT-PHHEQL---------------------------GP------TAGSS
EV--LDLISSKDLASQLTDYDWNLFISVHQVELIY-------YIFGQQTF-PSVTTANLE

RLMRR---------FNELQYWI-----ATELCLCAEPARR------TQLLRKFIKLAAHL
---------------KEQKNLNSFFAVMF-GLSNTAVSRLAKTWE---------------
-------RLPHKTRKLHSA-LERML--------------------------DPSWNH---
-RVYRL----AVAK-------LSPPII-PFIPL-LLK-----------------------
----DLTFIHEGNR--TLT------------------------------EN-LINFE-KM
-------RM---MAKAVRIIHHCQS--HT----------------------HAPISPLRS
RAPHVLEDPQALRIST--
-----------C---SELSLSG
RSPVSTWAY-LQHLKVIDNQKELSRLSRDLES----------------------------

>Rapgef3_[Peromyscus_maniculatus_bairdii]_589923021

---------------------M--------------------------------------

---------------K---------------------------------VSWPGEN----
-----HWQVGPAVVE-SPAVGTPPVGS----LPDVVPEGTLLDMVLKRMHRPRCCSYQ-L
VFE------------------------HRRP-S---------CIQ---------------
------------------GLR-------------------WT------------------
-----PLTNSEESLDFSV-SLEQA-------------------T--------T-------
-------ERVL-------------KA---------------------GKLLH-RHLLATY
PT-L----------------IRDRKYH------L-RLY----------------RQC-CS
GR-----------------ELVDGILALG------------------------------L
----GVH--------SRSQAVGIC---QVLLDEGALC-----------------------
--------------H------------------------VKH-DWTFQDRDAQFYRFPGP
-------EPEP--AGTHDV---

---------EEEL------VEATALLSQRGPDAL-------------LTVALRKPPGQRT
DEELD-----LIFEELLHIKAVAHLS-NSVKRELA-AVLLFEPHSKAGTVL---------
--FSQGDKG--TSWYIIW
KGSVNVVTHGK-----GL-------------------------------VTTLHEG-DDF
GQLAL-----------VNDAPRA---ATIIILRENNCHFLRVDKQDFNR------IIKD--
---V--------------

------------------------EAKTMRLEEHGKVVLVLER-------S------SQG
TG-----PSRP------------------PTPGRN------------R-YTVMSGTPEKI
LELLLEA------------MRPDSSAHDPT-ETF-------------------LS---D-
FLLTHSV--FM---------------PSTQLFTALL-----HHF------H---------
-----------------------------VEPAEPAGGSEQDRSTYICNKRQQILRLVSR
WVALYGPMLHSD-PVATSFLQ----KLSDL--VSRDA--RLSNLLR---EQ-WPERRR-H
HRL----ENGCGNA--SP------QTKAR----------------------NAP------
-----VW-----------LPGQE--------EP---------------------------
-----------------L--
---PSST---

----GAIRVGDK--VP-----------------------------YD-------------
ICRPDHTVLTLQLPVTASVREVMA--------ALAHEDH-----WT-------KGQ--VL
VKVNSA----------------------G------DVVGLQPEARGVATSLGLNERLFVV
NPQE---V-HELT-PHPEQL---------------------------GP------TLGSS
EM--LDLVSAKDLAGQLTDHDWNLFNKIHQVELIH-------YVLGPQHL-RDVTTANLE
RFMRR---------FNELQYWV-----ATELCLCPVPGPR------AQLLRKFIKLAAHL
---------------KEQKNLNSFFAVMF-GLSNSAISRLAHTWE---------------
-------RLPHKVRKLYSA-LERLL--------------------------DPSWNH---
-RVYRL----ALTK-------LSPPVI-PFMP----------------------------
LLLKDMTFIHEGNH--TL-VE-----------------------------N-LINFE-KM
-------RM---MARAVRMLHQCRSHST------------------------VPLSPLRS
RVSHIHEDSQASRISTCSEQSLSTR-------S--------------PASTWA-----YV
QQLKVIDNQREL---SRLSREL
-----------EP---

>Rapgef3_[Physeter_catodon]_593715976

---------------------M--------------------------------------

---------------K---------------------------------VGWPGES----
-----RWQVGLAVED-SSVLGAPLVGG----LPDVVPEGTLLNMVLRRMHRSRSCSYQ-L
LLE------------------------HQRP-S---------CIQ---------------
------------------GLR-------------------WT------------------
-----PLTNSEESLDFSV-SLEQA-------------------S--------T-------
-------ERVL-------------RA---------------------GRQLH-RHLLATC
PN-L----------------IRDRKYH------L-RLH----------------RQC-CS
GR-----------------ELVDGILALG------------------------------L
----GVH--------SRSQAVGIC---QVLLDEGALC-----------------------
--------------H------------------------VKH-DWAFQDRDTQFYRFPGS
-------EPEP--AGIHEL---

---------EEEF------VEALALLSQRGPDAL-------------LTVALRKPPGQRT
DEELD-----LIFEELLHIKAVAHLS-NSVKRELA-AVLLFEPHSKAGTVL---------
--FSQGDKG--TSWYIIW
KGSVNVVTHGK-----GL-------------------------------VTTLHEG-DDF
GQLAL-----------VNDAPRA---ATIILREDNCHFLRVDKQDFNR------IIKD--
---V--------------

------------------------EAKTMRLEEHGKVVLVLER-------T------SQG

TG-----PSRP------------------PTPGRN------------R-YIVMSGTPEKI
LELLLEA------------MRPDSSAHDPT-ETF-------------------LS---D-
FLLTHSV--FM---------------PTAQLCAALL-----HHF------H---------
-----------------------------AEPA---GGSEQECSTYICNKRQQILRLVSQ
WVALYGPMLHTD-PVATSFLQ----KLSDL--VSRDA--RLSNLLR---EQ-WPERRR-H
HRL----ENGCGNA--SP------QMKAW----------------------NMP------
-----VW-----------LPSQD--------EP---------------------------
-----------------L--
---PSSN---

----CAIRVGDK--VP-----------------------------YD-------------
ICRPDHSVLTLQLPVTASVREVMA--------ALAQEDG-----WT-------KGQ--VL
VKVNSA----------------------G------DTVGLQPDARGVATSLGLNERLFVV
NPQE---V-HELT-PHPEQL---------------------------GP------TVGSA
EG--LDLVSTKDLAGQLTDHDWSLFNSIHQVELIH-------YVLGPQPL-RDVTTANLE
RFMRR---------FNELQYWV-----ATELCLCPVPGLR------AQLLRKFIKLAAHL
---------------KEQKNLNSFFAIMF-GLSNSAISRLAQTWE---------------
-------RLPHKVRKLYSA-LERQL--------------------------DPSWNH---
-RVYRL----ALTK-------LSPPLI-PFMP----------------------------
LLLKDMTFIHEGNH--TL-VE-----------------------------N-LINFE-KM
-------RM---MARAARILHHCRSHSN------------------------VPLSPLRS
RVSHLHEDSQVARISTCSEQSLSTR-------S--------------PASTWA-----YV
QQLKVIDSQREL---SRLSREL
-----------EP---

>Rapgef3_[Poecilia_formosa]_617405394

--MHLFRTHNYQ-V
F----PH-----------------RYSVE-K--PTIR-----------------------
------------------GIS-------------------WT------------------
-----PLPEALDS---KD-SMKQ---------------------------FLS-------
-------DRVV-------------KA---------------------ARSVY-SVMVEKN
PG-L----------------IRDRKHH------L-KTH----------------RQC-CS
GK-----------------ELVDWLMKQS------------------------------E
----CLQ--------SRSQAAGMW---QVLVDEGILV-----------------------
--------------H------------------------VKQ-DLNFLDKDTQFYRFQDS
-------EFGLNHVSNEK--
EL--

---------EDEL------HEALSLLSQLGPDAL-------------LTMILRKCPSQRS
AEDIE-----VIYEELLHVKAAAHLS-SSVRKELA-AVLVFESHVKSGTVL---------
--FSQGDKG--TSWYIIW
KGSVNVITHGK-----GI-------------------------------VTTLHEG-EDF
GQLAL-----------LNDAPRA---ATIILREDNCHFLRVDKHDFIR------ILKD--
---V--------------

------------------------EANTVRLEEHGKTVLVLEK-------S---------
AD----CNGQG------------------GAAANS------------K-YTVMSGTPEKI
LEHLLDT------------LKLDSNGNDPI-DP-------------------CVS---D-
FLLTHKV--FM---------------PSSQLCSALQ-----HHY------Q---------
-----------------------------AELSK---GSDQEKTAYIFNTKQKVVKLIAQ
WVALYGLLLKED-PEVGDFLEQ---RLKKE--VAADY--RLSSILK---EQ-FRERRR--
TKV---MENGYQSL--S-------R-N-QQFD----------------------------
------W-----------FSSCE--------EP---------------------------

---VGRL---

----QPIKAQDK--VL-----------------------------YE-------------
IYKADSKPLSLMLPVSSSVQEVMS--------AIVKAD---------------GDH--VL
VKMNSS----------------------G------ERAQLKLDATAVYTALGLNERLFIC
TSSE---V-EQLR-PLKDQQ---------------------------GP------EQATT
DL--IEQMSSKDIATELTNYDWELFTAMHEVELVY-------YIFGRHKF-PGATTANLE
RFVRH---------FNVVQHWV-----VTELCLCEDLVKR------AILLKKFIKIASVL
---------------KEQRNLNSFFAVMF-GLSNSSVQRLYKTWE---------------
-------RIPSKTKRIYCA-YERLM--------------------------DPSRNH---
-RAYRL----AVAK-------LSPPYI-PFMP----------------------------
LLLKDMTFIHEGNP--NY-VD-----------------------------K-LVNFE-KM
-------RM---LAKTVKIVRGCRSQPY------------------------VPSSPQRG
LADRMFLDGPATRLSTYSDHAFPLR-------S--------------PSNIRQ-----YI
QNLKVIDNQRKL---TQLSRTI
-----------EC---

>Rapgef3_[Poecilia_latipinna]_961891077

--MHLFRTHNYQ-V
F----PH-----------------RYSVE-K--PTIR-----------------------

```
------------------GIS-------------------WT------------------
-----PLPEALDP---KD-TMKQ---------------------------FLS-------
-------DRVV-------------KA---------------------ARSVY-SVMVEKN
PG-L----------------IRDRKHH------L-KTH----------------RQC-CS
GK-----------------ELVDWLMKQS------------------------------E
----CLQ--------SRSQAAGMW---QVLVDEGILV-----------------------
--------------H------------------------VKQ-DLNFLDKDTQFYRFQDS
-------EFGLNHVSNEK------------------------------------------
EL----------------------------------------------------------
---------EDEL------HEALSLLSQLGPDAL-------------LTMILRKCPSQRS
AEDIE-----VIYEELLHVKAAAHLS-SSVRKELA-AVLVFESHVKSGTVL---------
--------------------------------------------FSQGDKG--TSWYIIW
KGSVNVITHGK-----GI-------------------------------VTTLHEG-EDF
GQLAL-----------LNDAPRA---ATIILREDNCHFLRVDKHDFIR------ILKD--
---------------------------------------------V--------------
------------------------------------------------------------
------------------------------------------------------------
------------------------EANTVRLEEHGKTVLVLEK-------S---------
AD----CNGQG------------------GAAANS------------K-YTVMSGTPEKI
LEHLLDT------------LKLDSNGNDPI-DP-------------------CVS---D-
FLLTHKV--FM---------------PSSQLCSALQ-----HHY------Q---------
-----------------------------AELSK---GSDQEKTAYIFNTKQKVVKLIAQ
WVALYGLLLKED-PEVGDFLE----RLKKE--VAADY--RLSSILK---EQ-FRERRR--
TKV---MENGYQSL--S-------R-N-QQFD----------------------------
------W-----------FSSCE--------EP---------------------------
------------------------------------------------------------
---VGRL-----------------------------------------------------
------------------------------------------------------------
------------------------------------------------------------
------------------------------------------------------------
------------------------------------------------------------
----QPIKAQDK--VL-----------------------------YE-------------
IYKADSKPLSLMLPVSSSVQEVMS--------AIVKAD---------------GDH--VL
VKMNSS----------------------G------ERAQLKLDATAVYTALGLNERLFIC
TSSE---V-EQLR-PLKDQQ---------------------------GP------EQATT
DL--IEQMSSKDIATELTNYDWELFTAMHEVELIY-------YIFGRHKF-PGATTANLE
RFVRH---------FNVVQHWV-----VTELCLCEDLVKR------AILLKKFIKIASVL
---------------KEQRNLNSFFAVMF-GLSNSAVQRLYKTWE---------------
-------RIPSKTKRIYCA-YERLM--------------------------DPSRNH---
-RAYRL----AVAK-------LSPPYI-PFMP----------------------------
LLLKDMTFIHEGNP--NY-VD-----------------------------K-LVNFE-KM
-------RM---LAKTVKIVRGCRSQPY------------------------VPSSPQRG
LADRMFLDGPATRLSTYSDHAFPLR-------S--------------PSNIRQ-----YI
QNLKVIDNQRKL-----------------------------------------TQLSRTI
-----------EC-----------------------------------------------
------------------------------------------------------------
------------------------------------------------------------
------------------------------------------------------------
------------------------------------------------------------
------------------------------------------------------------
------------------------------------------------------------
------------------------------------------------------------
------------------------------------------------------------
------------------------------------------------------------
```


>Rapgef3_[Poecilia_reticulata]_658859927

--MHLFRTHNYQ-V
F----PH-----------------RYSVE-K--PTIR-----------------------
------------------GIS-------------------WT------------------
-----PLPEALDP---KD-TMKQ---------------------------FLS-------
-------DRVV-------------KA---------------------ARSVY-SVMVEKN
PG-L----------------IRDRKHH------L-KTH----------------RQC-CS
GK-----------------ELVDWLMKQS------------------------------E
----CLQ--------SRSQAAGMW---QVLVDEGILV-----------------------
--------------H------------------------VKQ-DLNFLDKDTQFYRFQDS
-------EFGLSHXSNEK--
DL--
---------EDEL------HEALSLLSQLGPDAL-------------LTMILRKCPSQRS
AEDIE-----VIYEELLHVKAAAHLS-SSVRKELA-AVLVFESHVKSGTVL---------
--FSQGDKG--TSWYIIW
KGSVNVITHGK-----GI-------------------------------VTTLHEG-EDF
GQLAL-----------LNDAPRA---ATIILREDNCHFLRVDKQDFIR------ILKD--
---V--------------

------------------------EANTVRLEEHGKTVLVLEK-------S---------
AD----CNGQG------------------GAAANS------------K-YTVMSGTPEKI
LEHLLDT------------LKLDSNGNDPI-DP-------------------CVS---D-
FLLTHKV--FM---------------PSSQLCSALQ-----HHY------Q---------
-----------------------------AELSK---GSDQEKTAYIFNTKQKVVKLIAQ
WVALYGLLLKED-PEVGDFLEQ---RLKKE--VAADY--RLSSILK---EQ-FRERRR--
TKV---MENGYQSL--S-------R-N-QQFD----------------------------
------W-----------FPSCE--------EP---------------------------

---VGRL---

----QPIKAQDK--VL-----------------------------YE-------------
IYKADSKPLSLMLPVSSSVQEVMS--------AIVKAD---------------GDH--VL
VKMNSS----------------------G------DRAQLKLDATAVYTALGLNERLFIC
TSSD---V-EQLR-PLKDQQ---------------------------GP------EQATT
DL--IEQMSSKDIATELTNYDWELFTAMHEVELVY-------YIFGRHKF-PGATTANLE
RFVRH---------FNVVQYWV-----VTELCLCEDLVKR------AILLKKFIKIASVL
---------------KEQRNLNSFFAVMF-GLSNSAVQRLYKTWE---------------
-------RIPSKTKRIYCA-YERLM--------------------------DPSRNH---
-RAYRL----AVAK-------LSPPYI-PFMP----------------------------
LLLKDMTFIHEGNP--NY-VD-----------------------------K-LVNFE-KM
-------RM---LAKTVKIVRGCRSQPY------------------------VPSSPQRG
LADRMFLDGPATRLSTYSDHAFPLR-------S--------------PSNIRH-----YI
QTLKVIDNQRKL---TQLSRTI
-----------EC---

>Rapgef3_[Pongo_abelii]_197099402

-----------------------------------MAQKEEITQPLWETVGWPGEG----
-----CWQVGLAVED-SPALGAPRVGA----LPDVVPEGTLLNMVLRRMHRPRSCSYQ-L
LLE------------------------HQRP-S---------CIQ---------------
------------------GLR-------------------WT------------------
-----PLTNSEESLDFSE-SLEQA-------------------S--------T-------
-------ERVL-------------RA---------------------GRQLH-RHLLATC
PN-L----------------IRDRKYH------L-RLY----------------RQC-CS
GR-----------------ELVDGILALG------------------------------L
----GVH--------SRSQVVGIC---QVLLDEGALC-----------------------
--------------H------------------------VKH-DWAFQDRDAQFYRFPGP
-------EPEP--VGAHEM---

---------EEEL------AEAVALLSQRGPDAL-------------LTVALRKPPGQRT
DEELD-----LIFEELLHIKAVAHLS-NSVKRELA-AVLLFEPHSKAGTV----------

>Rapgef3_[Pristionchus_pacificus]_802732026

------------------------------------ISSQMVHDGENGPIP-----SGTS
VL---DLYGSAELATQLGFFHMELFHATNELETIA-------QVFGRDAF-PGHIPSNLD
LLLRR---------FNEVQYWA------TTEVLVSPAPKR------VQSLRKLIKIAHYS
---------------KQQGDLLSLFAIVL-GLSNVAVSRLSMTWE---------------
-------KLPSRVKRMFSE-LESLL--------------------------DPTRNH---
-RAYRS----LIAK-------MQPPFV-PFIP----------------------------
LLLKDLTFIHEGNK--TF--------------------------F-----NGLVNFE-KM
-------HM---IANVIRTFKECKASASGVT-----------------------------
--------------------GILHRDE-----K---------------PDSQL-----HI
RNLRVIDDQRRL---LEMSYLI
-----------EPKMSKR--

>Rapgef3_[Propithecus_coquereli]_826277148

---------------------M--------------------------------------

---------------AASELAGWATF-QAVGTAYVTAQRGEITWQVWATVGWPGES----
-----RWQVGLALED-SPALSASQVGG----LPDVVPEGTLLNMVLRRMYRSRSCSYQ-L
LLE------------------------HRRP-S---------RIQ---------------
------------------GLR-------------------WT------------------
-----PLTNSEESLDFTV-SLEQA-------------------S--------T-------
-------ERVL-------------RA---------------------GKQLH-RHLLATC
PN-L----------------IRDRKYH------L-RLY----------------RQC-CS
GR-----------------ELVDGILALG------------------------------L
----GVH--------SRSQAVGIC---QVLLDEGALC-----------------------
--------------H------------------------VKH-DWTFQDRDAQFYRFPGP
-------EPEP--TGAHEM---

---------EEEL------VEAMALLSQRGPDAL-------------LTVALRKPPGQRT
DEELD-----LIFEELLHVKAVAHLS-NSVKRELA-AVLLFEPHSKAGTVL---------
--FSQGDKG--TSWYIIW
KGSVNVVTHGK-----GL-------------------------------VTTLHEG-DDF
GQLAL-----------VNDAPRA---ATIILREDNCHFLRVDKQDFNR------IIKD--
---V--------------

------------------------EAKTMRLEEHGKVVLVLER-------A------SQG

AG-----TTRP------------------PTPGRN------------R-YTVMSGTPEKI
LELLLEA------------MRPDSSAHDPT-ETF-------------------LS---D-
FLLTHSV--FM---------------PSAQLCAALL-----HHF------H---------
-----------------------------AEPA---GGSEQECSTYICNKRQQILRLVSQ
WVALYGSMLHTD-PVATNFLQ----KLSDL--VSRDT--RLSNLLR---EQ-WPERRR-H
HRL----ENGCGNA--SP------QMKAQ----------------------NVP------
-----VW-----------LPSQE--------EP---------------------------
-----------------L--
---PSSN---

----CAIQVGDK--VS-----------------------------YD-------------
ICRPDHSVLTLQLPVTASVREVMA--------ALAQEDG-----WT-------KGQ--VL
VKVNSA----------------------G------DATGLQLDARGVATSLGLNERLFVV
NPQE---V-HKLT-PHPEQL---------------------------GP------TVGSA
EG--LDLVSAKDLAGQLTDHDWNLFNSIHQVELIH-------YVLGPQHL-RDVTTANLE
RFMRR---------FNELQYWV-----ATELCLCSVPGLR------AQLLRKFIKLAAHL
---------------KEQKNLNSFFAVMF-GLSNSAISRLAHTWQ---------------
-------RLPHKVRKLYSA-LERLL--------------------------DPSWNH---
-RVYRL----ALAK-------LSPPVI-PFMP----------------------------
LLLKDMTFIHEGNH--TL-VE-----------------------------N-LINFE-KM
-------RM---MARAVRMLHHCRGHSS------------------------VPLSPLRS
RVSHLHEDGQALRISTCSEQSLSTR-------S--------------PASTWA-----YV
QQLKVIDNQREL---SRLSREL
-----------EP---

>Rapgef3_[Protobothrops_mucrosquamatus]_1002588985

-------------------MKVAWSG-QGRWNVELPPGELADLSQFSTGLV-DPVPE---
--RN----LLYELWK--------------------------------KMHLFRSCSYR-I
Q------------------------LEGQMTSAPRIQ-----------------------
------------------GIR-------------------WT------------------
-----PLPESESTLVPGH-NLKQA------------------------S---S-------
-------NKIQ-------------RA---------------------GRLLC-AHLVIMH
PD-L----------------IRDHKHH------L-RLH----------------RQC-CS
GK-----------------ELVDALLSAG------------------------------L
----SVQ--------TRSQALGLC---QVLMDEGVLA-----------------------
--------------H------------------------GKG-QSV--------------

---PNQRT
DEELA-----LIFEELLHVKAVAHLS-SSVRRELA-AVLLFESHAKAGTVL---------
--FSQGDKG--TSWYIIW
KGSINVVTHDK-----GL-------------------------------VCTLHEG-DDF
GQLAL-----------VNDAPRA---ATIILREDSCHFLRVDKRDFNR------ILKD--
---V--------------

------------------------EANTLRLKEHGKVVLVLEK-------N---------
SQ-GTNSSHLA------------------GTPGSS------------R-YSVMAGTPEKI
LDYLLET------------MRSDSTLSDPV-DTF-------------------LG---D-
FLLTYSV--FM---------------PTPQLCRTLL-----QHF------H---------
-----------------------------SEPSE---GSEQDKAAYILGKRQKILWLVNQ
WVSLYGRLLQAE-ACAVAFLQ----TMSEY--ATQDA--RLGPLVR---EQ-IQDRRR--
NRV---IENGSNCA--SPK----LQVR-NVVN----------------------------
------R-----------FVIQE--------ET---------------------------
-----------------F--
---LNNS---

----YTMRIKDK--VS-----------------------------YD-------------
IYRPNYSCLTRLLQVNASVQEVLA--------SLVDCQDWN------------KDW--IL
VKVNSA----------------------G------DKVVLPMEAFGVFTSLGLNERLFAV
SVQE---L-HNLA-PHPEQL---------------------------GP------RIGSW
ES--LDFISSKDLANQITEHDWNLFKSLHQVELIY-------YVVGSQKF-PSATTVNVE
RFLRR---------FNELQFWI-----TTELCLCPDVIKR------AQLLRKFIKLAAHL
---------------KDQRNLNSFFAVMF-GLSHSAISRLSRTWE---------------
-------KLPYKTRKLYGT-MERML--------------------------DPSWNH---
-RVYRL----AVAK-------LSPPMI-PFMP----------------------------
LLLKDMTFIHEGNR--TL-VE-----------------------------N-LVNFE-KL
-------RM---MAKAVHLVHHCRSHPN------------------------LPLSPLRS
RPQHLLEEARAMRTSTCSVQSLSTR-------S--------------PAATWA-----HI
QPLRVIDRQKEL---LRLSRDL
-----------ES---

>Rapgef3_[Pseudopodoces_humilis]_929508805

------------------M--------HLFRSSSYEV-------------RLEGA-GGSL

```
PRIQG-------IRW--------------------------T------------------
-----PLLD-------SESSLDYGH------------------SITQAS---P-------
-------EKIW-------------RAGKLLF---------------A----H---LSTSR
PG-L----------------IRDHKHH------L-RHH----------------RQC-CS
GK-----------------ELVDWLLSSG------------------------------A
----AVQ--------TRGQAVGIG---QVLLDGGVLT-----------------------
--------------H------------------------VRQ-EWHFQDKETQFYRFAEL
E-----LSPEP-GAGPRDP-----------------------------------------
------------------------------------------------------------
----------EEL------LEALGFLAQLGPDAL-------------LSMALRKPPAQRT
QDELE-----LIFEELLHIKAVAHLS-NSVKRELA-AVLMFEGHQRAGTV----------
-------------------------------------------LFSQGDKG--TSWYIIW
KGSVNVVTHGK-----GL-------------------------------VATLHEG-DDF
GQLAL-----------VNDAPRA---ATILLREDNCHFLRVDKQDFNR------ILKD--
---------------------------------------------V--------------
------------------------------------------------------------
------------------------------------------------------------
------------------------EANTVRLKEHGKVVLVLQKDL-------------QG
G------GGQA------------------ASARSSSR------------YLVMAGTPEKI
LEHLLEF------------MRLDATLYDPV-D-------------------TLLG---D-
FLLTYTV--FM---------------PTSQLCRALL-----HHF------R---------
-----------------------------AEPLE---GSEQDKATYSLRKRRKILRLVSQ
WVLLYGRLLQGD-RSTTALLQ----NLAD--LASRDP--QLGGLGQ---EQ-GQERRR--
PRA---MENGDGSV--SP------QPKPRS---------------------SGM------
------W-----------LGTPE--------EA---------------------------
----------------IL------------------------------------------
----DSTS-AL-----------------RAQDK---------------------------
------------------------------------------------------------
------------------------------------------------------------
------------------------------------------------------------
------------------------------------------------------------
--------------VP-----------------------------YE-------------
IFRADHSCLVSVLPVNASVRDVLR--------ALAPRLG-------------RDRE-HVL
VKVNSA----------------------GERAV------LPQDAVGVFTALGLNERLFVV
TADE---L-GNLT-PHPEQL---------------------------GP------HAGSS
DS--LDLISSKDLASHLTDYDWNLFKSIHQVEMIH-------YIVGPQKF-HEVTTANLA
RVMRR---------FNELQFWV-----ATELCLCPELGRR------AQLLRKFIKLAAHL
---------------KEQKNLNSFFAVMF-GVSNTAVTRLAKTWE---------------
-------RLPHKIRKLHSA-LERML--------------------------DPSWNH---
-RVYRL----AVAK-------MSPPII-PFMPL-LLK-----------------------
----DMTFIHEGNR--TLA------------------------------EN-LINFE-KM
-------HM---MAKTVRVLQRCRG--HA----------------------HAPLSPLRN
RSPHRPEDAKAVRIST--------------------------------------------
-----------C-----------------------------------------SEQSLSV
RNPISTWAY-LQHLRAIDSQRELLRLSRDLEP----------------------------
------------------------------------------------------------
------------------------------------------------------------
------------------------------------------------------------
------------------------------------------------------------
------------------------------------------------------------
------------------------------------------------------------
------------------------------------------------------------
------------------------------------------------------------
------------------------------------------------------------
```


>Rapgef3_[Pteropus_alecto]_989976416

---MVLRRMHRPRSCSYQ-L
LLE------------------------HQRP-S---------RIQ---------------
------------------GLR-------------------WT------------------
-----PLTNSEESLDFSV-SLEQA-------------------S--------T-------
-------ERVL-------------RA---------------------GKQLH-RHLLATC
PN-L----------------IRDRKYH------L-RLY----------------RQC-CS
GR-----------------ELVDGILALG------------------------------L
----GVH--------SRSQAVGIC---QVLLDESALC-----------------------
--------------H------------------------VKH-DWTFQDRDTQFYRFPGP
-------EPEP--VGVHEL---

---------EEEL------VEAMALLSQRGPDAL-------------LTVALRKSPGQRT
DEELD-----LIFEELLHIKAVAHLS-NSVKRELA-AVLLFEPHSKAGTVL---------
--FSQGDKG--TSWYIVW
KGSVNVVTHGK-----GL-------------------------------VTTLHEG-DDF
GQLAL-----------VNDAPRA---ATIVLREDNCHFLRVDKQDFNR------IIKD--
---V--------------

------------------------EAKTMRLEEHGKVVLVLER-------T------SQG
AG-----SSCP------------------PTPGRN------------R-YTVMSGTPEKI
LELLLEA------------MRPDSSAHDPT-ETF-------------------LS---D-
FLLTHSV--FM---------------PSTQLCAALL-----HHF------H---------
-----------------------------VEPA---GGSEQERSTYICNKRQQILRLVSQ
WVALYGPMLHTD-PVASSFLQ----KLSDL--VSRDA--RLSNLLR---EQ-WPERRR-H
HRL----ENGCGNT--SP------QMKAR----------------------NMP------
-----AW-----------LPSQD--------EP---------------------------
-----------------L--
---PSSN---

----CAIRVGDK--VS-----------------------------YD-------------
ICRPDHSVLTLQLPVMASVREVMA--------ALAKEDS-----WT-------KGQ--VL
VKVNSA----------------------G------DAIGLQPDARGVATSLGLNERLFVV
NPQE---V-HELT-PHPEQL---------------------------GP------TVGSA
EG--LDLVSAKDLAGQLTDHDWNLFNSIHQVELIH-------YVLGPQHL-RDVTTANLE
RFMRR---------FNELQYWV-----ATELCLCAVPGPR------AQLLRKFIKLAAHL
---------------KEQKNLNSFFAVMF-GLSNSAISRLARTWE---------------
-------RLPHKVRKLYSA-LERLL--------------------------DPSWNH---
-RVYRL----ALTK-------LSPPVI-PFMP----------------------------
LLLKDMTFIHEGNH--TL-VE-----------------------------N-LINFE-KM
-------RM---MARAVRMLHHCRSHSN------------------------VPLSPLRS
RVSHLHEDSQAVRISTCSEQSLSTR-------S--------------PASTWA-----YV
QQLKVIDNQREL---SRLSREL
-----------EP---

>Rapgef3_[Pteropus_vampyrus]_759143390

---------------------M--------------------------------------

---------------K---------------------------------VGWPGES----
-----RWQVGLTVED-SPALGAPQVGG----LPDVVPEGTLLNMVLRRMHRPRSCSYQ-L
LLE------------------------HQRP-S---------RIQ---------------
------------------GLR-------------------WT------------------
-----PLTNSEESLDFSV-SLEQA-------------------S--------T-------
-------ERVL-------------RA---------------------GKQLH-RHLLATC
PN-L----------------IRDRKYH------L-RLY----------------RQC-CS
GR-----------------ELVDGILALG------------------------------L
----GVH--------SRSQAVGIC---QVLLDESALC-----------------------
--------------H------------------------VKH-DWTFQDRDTQFYRFPGP
-------EPEP--VGVHEL---

---------EEEL------VEAMALLSQRGPDAL-------------LTVALRKSPGQRT
DEELD-----LIFEELLHIKAVAHLS-NSVKRELA-AVLLFEPHSKAGTVL---------
--FSQGDKG--TSWYIVW
KGSVNVVTHGK-----GL-------------------------------VTTLHEG-DDF
GQLAL-----------VNDAPRA---ATIVLREDNCHFLRVDKQDFNR------IIKD--
---V--------------

------------------------EAKTMRLEEHGKVVLVLER-------T------SQG
AG-----PSCP------------------PTPGRN------------R-YTVMSGTPEKI
LELLLEA------------MRPDSSAHDPT-ETF-------------------LS---D-
FLLTHSV--FM---------------PSTQLCAALL-----HHF------H---------
-----------------------------VEPA---GGSEQERSTYICNKRQQILRLVSQ
WVALYGPMLHTD-PVASSFLQ----KLSDL--VSRDA--RLSNLLR---EQ-WPERRR-H
HRL----ENGCGNT--SP------QMKAR----------------------NMP------
-----AW-----------LPSQD--------EP---------------------------
-----------------L--
---PSSN---

----CAIRVGDK--VS-----------------------------YD-------------
ICRPDHSVLTLQLPVTASVREVMA--------ALAKEDS-----WT-------KGQ--VL
VKVNSA----------------------G------DAIGLQPDARGVATSLGLNERLFVV
NPQE---V-HELT-PHPEQL---------------------------GP------TVGSA
EG--LDLVSAKDLAGQLTDHDWNLFNSIHQVELIH-------YVLGPQHL-RDVTTANLE

RFMRR---------FNELQYWV-----ATELCLCAVPGPR------AQLLRKFIKLAAHL
---------------KEQKNLNSFFAVMF-GLSNSAISRLARTWE---------------
-------RLPHKVRKLYSA-LERLL--------------------------DPSWNH---
-RVYRL----ALTK-------LSPPVI-PFMP----------------------------
LLLKDMTFIHEGNH--TL-VE-----------------------------N-LINFE-KM
-------RM---MARAVRMLHHCRSHSN------------------------VPLSPLRS
RVSHLHEDSQAVRISTCSEQSLSTR-------S--------------PASTWA-----YV
QQLKVIDNQREL---SRLSREL
-----------EP---

>Rapgef3_[Pundamilia_nyererei]_548483151

--MHLFRTHNYQ-V
F----PD-----------------RCSVE-K--QTIR-----------------------
------------------GIS-------------------WT------------------
-----PLPEALNS---KD-TMKQ---------------------------FLS-------
-------DRVV-------------KA---------------------ARSVY-SVMIEKN
PG-L----------------IRDRKHH------L-KTY----------------RQC-CS
GK-----------------DLVDWLMTQN------------------------------E
----GLQ--------SRSQAVGIW---QVLVDEGILL-----------------------
--------------H------------------------VKH-ELNFLDKDTQFYRFQDS
-------QFGLNHILNEK--
DS--
---------EDEL------QEALSLLSQLGPDAL-------------LTMILRKCPSQRS
PEDLE-----VIYEELLHVKAAAHLS-SSVRKELA-AVLVFESHAKAGTVL---------
--FSQGDKG--TSWYIIW
KGSVNVITHGK-----GL-------------------------------VTTLHEG-EDF
GQLAL-----------LNEQPRA---ATIIILREDNCHFLRVDKQDFIR-----ILKD--
---V--------------

------------------------EANTVRLEEHGKTVLVLEK-------S---------
AD----STEQG------------------GAGNSS------------K-YTVMSGTPEKI
LEHLLET------------IKLECNGNDAI-DS-------------------CVS---D-
FLLTHKV--FM---------------PSSQLCPALQ-----HHY------Q---------
-----------------------------AQLAE---GSEQEKTAYVLNIKQKVVKLVGQ
WVALFGLHLKED-PIALDFLE----RLKKD--MAPNS--QLFNVLK---D--FRERRR--
TKV---LENGYQSL--S-------R-N-HQFD----------------------------
------W-----------FSNFE--------ES---------------------------

---VGRV---

```
------------------------------------------------------------
------------------------------------------------------------
------------------------------------------------------------
------------------------------------------------------------
----QPIRAHDK--VL-----------------------------YE-------------
IYRPDNKPLTPMLPVNTSVQEIMS--------ALGKAG---------------EDH--VL
IKMNST----------------------G------ERAQLKLDATAVYTALGLNERLFIC
TISE---V-EQLT-PLKEQQ---------------------------GP------EQGTA
DV--LEQMSSKDIASELTNYDWELFTAMHEVELVY-------YIFGRHKF-PGAITANLE
RFVRR---------FNEVQHWV-----LTELCLCEDLVKR------AMLLKKFIKIASVL
---------------KEQKNLNSFFAVMF-GLSNSAIQRLYKTWE---------------
-------RIPSKTKRIYCS-LERLM--------------------------DPSRNH---
-RAYRL----AIAK-------LSPPYI-PFMP----------------------------
LLLKDMTFIHEGNA--NY-VD-----------------------------K-LVNFE-KM
-------RM---LAKTVKIVRGCRSQPY------------------------VPSSPQRG
LADRMFLEGAATRLSTYSDHAFPLR-------T--------------STNIRH-----YI
QNLKVIDNQRKL-----------------------------------------TQLSRTI
-----------DC-----------------------------------------------
------------------------------------------------------------
------------------------------------------------------------
------------------------------------------------------------
------------------------------------------------------------
------------------------------------------------------------
------------------------------------------------------------
------------------------------------------------------------
------------------------------------------------------------
------------------------------------------------------------
------------------------------------------------------------
---
>Rapgef3_[Pygoscelis_adeliae]_690437383
------------------------------------------------------------
------------------------------------------------------------
------------------------------------------------------------
------------------------------------------------------------
------------------------------------------------------------
------------------------------------------------------------
-------------------------MAEPPWPPTPFFSVFQT------------------
-----PLLD-------TESTLDYGH------------------SLTQAS---S-------
-------EKIW-------------RAGKLLFI-------------------H---LTSTR
PG-L----------------IRDHKHH------L-RHH----------------RQC-CS
GK-----------------ELVDWLVGRG------------------------------V
----CTS--------GRGGERGNRQP---------FT-----------------------
--------------P---------------------------------------------
------LTPPR-------------------------------------------------
------------------------------------------------------------
-------------------------------------------------------PAQRT
EDELE-----LIFEELLHIKAVAHLS-NSVKRELA-SVLMFESHQRAGTVP---------
-----------------------------------------V------------------
-----------------------------------------------------------P
AALAA-----------HAACPPA---SSAALW----------------------------
------------------------------------------------------------
------------------------------------------------------------
------------------------------------------------------------
--------------------------------------LPLEE-----------------
```

--------SAG------------------TTVSPE------------G-YLVMAGTPEKI
LEHLLEF------------MRLDATLYDPV-GCN--------------------------
-----------------------------------L------------------------
-----------------------------ASPFR---GVQEQKVARN-------------

-------EHGHRGG--RP------Q-----------------------------------

---------AS-----------------ATQGT---------------------------

--------------VP-----------------------------YE-------------
IYRPDHSCLIAVLPVNASVRDILR--------SLAPRLG-------------RDGE-HVL
VKVNSA----------------------GDKVG------LQLDAVGVFTALGLNERLFAV
SVEE---L-GSL---HIGSS
ET--LDLISSKDLASHLTDYDWNLFKSIHQVEMIH-------YIVGPQKF-HDVTTANLE
RVMRR---------FNELQYWV-----ATELCLCPEVGRR------AQLLRKFIKLAAHL
---------------KEQKNLNSFFAVMF-GVSNTAVSRLAKTWE---------------
-------RLPHKIRKLHSA-LER-------------------------------------

-------HM---MAKTVRVLQRCRG--DA----------------------HAPLSPLRN
RSPHRPEDPKGVRIST--
-----------C---SEQSLSV
RSPVSTWAY-LQHLKAIDSQKELLRLSHDLES----------------------------

>Rapgef3_[Python_bivittatus]_602664072

--MFSE------------

---T------------------
-----PLPESESALVPGH-NLKQA------------------------S---S-------
-------NKIR-------------RA---------------------GRLLC-THLVVMY
PD-L----------------IRDHKHH------L-RLH----------------RQC-CS
GK-----------------ELVDALLSAG------------------------------L
----SVQ--------TRSQALGLC---QVLMDEGVLS-----------------------
--------------H------------------------VRQ-ESYFQDRDAQFFRFVAL
-------EP------SAE--
-----------------DKDG---------------------------------------

----------EEL------LEALALLTQLGPTAL-------------LTTILRKPPNQRT
DEELA-----LIFEELLHVKAVAHLS-SSVKRELA-AVLLFESHAKAGTVL---------
--FSQGDKG--TSWYIIW
KGSVNVVTHDK-----GL-------------------------------VSTLHEG-DDF
GQLAL-----------VNDAPRA---ATIILREDSCHFLRVDKRDFNR------ILKD--
---V--------------

------------------------EANTLRLKEHGKVVLVLEK-------N---------
SQ-GSNSGHLP------------------GTPGSS------------R-YSVMAGTPEKI
LDYLLET------------MRSDSTLSDPV-DTF-------------------LG---D-
FLLTYSV--FM---------------PTPQLCTTLL-----QHF------H---------
-----------------------------SESSK---GSEQDKAAYILGKRQKILWLVNQ
WVSLYGRLLQAE-ACAVAFLQ----TLSEC--TTQDA--RLGPLLR---EQ-IQDRRR--
NRV---IENGSNCA--SPK----LQAR-NVVN----------------------------
------W-----------FASQE--------EP---------------------------
-----------------F--
---LNSS---

----YTIRIKDK--VS-----------------------------YD-------------
IYRPDYSCLTTLLQVNASVQEVLA--------SLVDCQDWN------------KDW--IL
VKVNSA----------------------G------DKVVLPMEAFGVFTSLGLNERLFAA
SVQE---L-HNVA-PHPEQL---------------------------GP------SMGSW
ES--LDFMSSKDLANQFTEHDWNLFKSIHQVELIY-------YVVGPQKF-PSATTANVE
RFLRR---------FNELQFWV-----TTELCLCPDVIKR------AQLLRKFIKLAAHL
---------------KDQKNLNSFFAVMF-GLSHSAISRLSRTWE---------------
-------KLPHKTRKLYGT-MERML--------------------------DPSWNH---
-RVYRL----AVVK-------LSPPMI-PFMP----------------------------
LLLKDMTFIHEGNR--TL-VE-----------------------------N-LVNFE-KL
-------RM---MAKAVRLVHHCRSHPN------------------------LPLSPLRS
RPQHLMEEARAMRTSTCSEQSLSTR-------S--------------PAATWA-----YI
QHLRVIDRQKEL---LRLSRDL
-----------ES---

>Rapgef3_[Rhinopithecus_roxellana]_724952750

---------------------M--------------------------------------

---------------K---------------------------------VGWPGES----
-----CWQVGLAVED-SPALGAPQVGA----LPDVVPEGTLLNMVLRKMHRPRSCSYQ-L
LLE------------------------HQRP-S---------CIQ---------------

------------------GLR-------------------WT------------------
-----PLTNSEESLDFSE-TLEQA-------------------S--------T-------
-------ERVL-------------RA---------------------GRQLH-RQLLATC
PN-L----------------IRDRKYH------L-RLY----------------RQC-CS
GR-----------------ELVDGILALG------------------------------L
----GVH--------SRSQVVGIC---QVLLDEGALC-----------------------
--------------H------------------------VKH-DWAFQDRDAQFYRFPGP
-------EPEP--VGAHEM---

---------EEEL------AEAVALLSQRGPDAL-------------LTVALRKPPGQRT
DEELD-----LIFEELLHIKAVAHLS-NSVKRELA-AVLLFEPHSKAGTVL---------
--FSQGDKG--TSWYIIW
KGSVNVVTHGK-----GL-------------------------------VTTLHEG-DDF
GQLAL-----------VNDAPRA---ATIILREDNCHFLRVDKQDFNR------IIKD--
---V--------------

------------------------EAKTMRLEEHGKVVLVLER-------A------SQG
AG-----PSRP------------------PTPGRN------------R-YTVMSGTPEKI
LELLLEA------------MGPDSSAHDPT-ETF-------------------LS---D-
FLLTHRV--FM---------------PSAQLCAALL-----HHF------H---------
-----------------------------AEPA---GGSEQERSTYICNKRQQILRLVSQ
WVALYGSMLHTD-PVATSFLQ----KLSDL--VGRDA--RLSNLLR---EQ-WPERRR-H
HRL----ENGCGNA--SP------QMKAR----------------------NLP------
-----VW-----------LPNQD--------EP---------------------------
-----------------L--
---PGSS---

----CAIRVGDK--VS-----------------------------YD-------------
ICLPDHSVLTLQLPVTASVREVMA--------ALAQEDG-----WT-------KGQ--VL
VKVNSA----------------------G------DAIGLQPDARGVATSLGLNERLFVV
NPQE---V-HELT-PHPDQL---------------------------GP------TVGSA
EG--LDLVSAKDLAGQLTDHDWSLFNSIHQVELIY-------YVLGPQHL-RDVTTANLE
RFMRR---------FNELQYWV-----ATELCLCPVPGPR------AQLLRKFIKLAAHL
---------------KEQKNLNSFFAVMF-GLSNSAISRLAHTWE---------------
-------RLPHKVRKLYSA-LERLL--------------------------DPSWNH---
-RVYRL----ALAK-------LSPPVI-PFMP----------------------------
LLLKDMTFIHEGNH--TL-VE-----------------------------N-LINFE-KM
-------RM---MARAARMLHHCRSHNP------------------------VPLSPLRS
RVSHLHEDSQVARISTCSEQSLSTR-------T--------------PASTWA-----YV
QQLKVIDNQREL---SRLSREL
-----------EP---

>Rapgef3_[Rousettus_aegyptiacus]_1012262851

---------------------M--------------------------------------

---------------K---------------------------------VGWPGES----
-----RWQVGLTVED-SPALGAPLVGS----LTDVVAEGTLLNMVLRRMHRPRSCSYQ-L
LLE------------------------HQRP-S---------RIQ---------------
------------------GLR-------------------WT------------------
-----PLTNSEESLDFSV-SLEQA-------------------S--------T-------
-------ERVL-------------RA---------------------GKQLH-RHLLATC
PN-L----------------IRDRKYH------L-RLY----------------RQC-CS
GR-----------------ELVDGILALG------------------------------L
----GVH--------SRSQAVGIC---QVLLDEGALC-----------------------
--------------H------------------------VKH-DWTFQDRDTQFYRFPGP
-------EPEP--AGVHEL---

---------EEEL------VEAMALLSQRGPDAL-------------LTVALRKSPGQRT
DEELD-----LIFEELLHIKAVAHLS-NSVKRELA-AVLLFEPHSKAGTVL---------
--FSQGDKG--TSWYIIW
KGSVNVVTHGK-----GL-------------------------------VTTLHEG-DDF
GQLAL-----------VNDAPRA---ATIVLREDNCHFLRVDKQDFNR------IIKD--
---V--------------

------------------------EAKTMRLEEHGKVVLVLER-------T------SQG
AG-----PSHP------------------PTPGRN------------R-YTVMSGTPEKI
LELLLEA------------MRPDSSAHDPT-ETF-------------------LS---D-
FLLTHSV--FM---------------PSTQLCAALL-----HHF------H---------
-----------------------------VEPA---GGSEQERSTYICNKRQQILRLVSQ
WVALYGPMLHTD-PVASSFLQ----KLSEL--VSRDA--RLSNLLR---EQ-WPERRR-H
HRL----ENGCGNA--SP------QIKAR----------------------NMT------
-----AW-----------LPSQD--------EP---------------------------
-----------------L--
---PSSN---

----CAIRVGDK--VS-----------------------------YD-------------
ICRPDHSVLTLQLPVTASVREVMA--------ALAKEDS-----WT-------KGQ--VL
VKVNSA----------------------G------DAIGLQPEARGVATSLGLNERLFVV
NPQE---V-HELT-PHPEQL---------------------------GP------TVGSA
EG--LDLVSAKDLAGQLTDHDWNLFNSIHQVELIH-------YVLGPQHL-RDVTTANLE
RFMRR---------FNELQYWV-----ATELCLCAVPGPR------AQLLRKFIKLAAHL
---------------KEQKNLNSFFAVMF-GLSNSAISRLARTWE---------------
-------RLPHKVRKLYSA-LERLL--------------------------DPSWNH---
-RVYRL----ALTK-------LSPPII-PFMP----------------------------
LLLKDMTFIHEGNH--TL-VE-----------------------------N-LINFE-KM
-------RM---MARAVRMLHHCRSHSN------------------------VPLSPLRS
RVSHLHEDSQAVRISTCSEQSLSTR-------S--------------PASTWA-----YV
QQLKVIDNQREL---SRLSREL
-----------EP---

>Rapgef3_[Saimiri_boliviensis_boliviensis]_403301658

---------------------M--------------------------------------

---------------K---------------------------------VGWPGES----
-----CWQVGLAVED-SPALGAPQVGA----LPDVVPEGTLLNMVLRRMHRPRSCSYQ-L
LLE------------------------HRRP-S---------RIQ---------------
------------------GLR-------------------WT------------------
-----PLTNSEESLDFSE-SLEQA-------------------S--------T-------
-------ERVL-------------RA---------------------GKQLH-RHLLATC
PN-L----------------IRDRKYH------L-RLY----------------RQC-CS
GR-----------------ELVDGILALG------------------------------L
----GVH--------SRSQAVGIC---QVLLDEGALC-----------------------
--------------H------------------------VKH-DWAFQDRDAQFYRFPGP
-------EPEP--MGAHEM---

---------EEEL------AEAVALLSQRGPDAL-------------LSVALRKPPGQRT
DEELD-----LIFEELLHIKAVAHLS-NSVKRELA-AVLLFEPHSKAGTVL---------
--FSQGDKG--TSWYIIW
KGSVNVVTHGK-----GL-------------------------------VTTLHEG-DDF
GQLAL-----------VNDAPRA---ATIILREDNCHFLRVDKQDFNR------IIKD--
---V--------------

------------------------EAKTMRLEEHGKVVLVLER-------A------SRG
AG-----SSRP------------------PTPGRN------------R-YTVMSGTPEKI
LELLLEA------------MGPDSSAHDPT-ETF-------------------LS---D-
FLLTHRV--FM---------------PSAQLCTALL-----HHF------H---------
-----------------------------AEPA---GGSEQERSTYVCNKRQQILRLVSQ
WVALYGSMLHTD-PVATSFLQ----KLSDL--VSRDA--RLSNLLR---EQ-WPERRR-H
YRL----ENGCGNA--SP------QMKAR----------------------HLP------
-----IW-----------LPNQD--------EP---------------------------
-----------------L--
---PGSS---

----CAIRVGDK--VP-----------------------------YD-------------
ICRPDHSVLTLQLPVTASVREVMA--------ALAQEDG-----WT-------KGQ--VL
VKVNSA----------------------G------DATGLQPDARGVATSLGLNERLFVV
NPQE---V-HELT-PHPEQL---------------------------GP------TVGSA
EG--LDLVSAKDLAGQLTDHDWSLFNSIHQVELIH-------YVLGPQHL-RDVTTANLE

RFMRR---------FNELQYWV-----ATELCLCAVPGPR------AQLLRKFIKLAAHL
---------------KEQKNLNSFFAVMF-GLSNSAISRLAHTWE---------------
-------RLPHKVRKLYSA-LERLL--------------------------DPSWNH---
-RVYRL----ALAK-------LSPPVI-PFMP----------------------------
LLLKDMTFIHEGNH--TL-VE-----------------------------N-LINFE-KM
-------RM---MARAARMLHHCRSHNP------------------------VPLSPLRS
RVSHLHEDSQVARISTCSEQSLSTR-------S--------------PASTWA-----YV
QQLKVIDNQREL---SRLSREL
-----------EP---

>Rapgef3_[Salmo_salar]_929230175

--MHLFRSYNYR-I
F----PD-----------------HYSVE-T--STIR-----------------------
------------------GIS-------------------WT------------------
-----PLPEALDT---QD-TMKQ---------------------------FLS-------
-------DRIL-------------KA---------------------ARVVH-SALIVRS
PG-L----------------IRDRKHH------L-KTY----------------RQC-CS
GK-----------------ELVDWLMKLN------------------------------E
----CFQ--------SRSQAVGMW---QVLVDEGIIV-----------------------
--------------H------------------------VKQ-ELNFHDKDTQFYRFLDL
-------EFGLNHMTNEK--
IS---------LNHTTNEKDQL--------------------------------------
---------DDEL------QEGLSLLLQIGPDAL-------------LTMILRKCPSQRS
AEDLE-----VIYEELLHVKAAAHLS-TSVRKELA-AVLMFESHAKAGTVL---------
--FSQGDKG--TSWYIIW
KGSVNVITHGK-----GL-------------------------------VTTLHEG-EDF
GQLAL-----------VNDAPRA---ATIIILREDNCHFLRVDKQDFIR-----ILKD--
---V--------------

------------------------EANTMRLEEHGKAVLVLEK-------A---------
ES-----TNPG------------------GAGNSS------------K-YTVMAGTPEKI
LEHLLET------------VKLDTNGSDAI-DP-------------------CVS---D-
FLLTHRV--FM---------------PSSQLCPALQ-----HHY------H---------
-----------------------------AEPSE---GSDLEKAAYALNTKQKIVKLICQ
WVAVFGQLLKDD-PIAVDFLE----RLGEE--VAGDS--RLSSMLR---EQ-LRDRRR--
TRA---LENELGSL--T-------RLN-HQFD----------------------------
------W-----------FSSCE--------EP---------------------------

---VGKC---

```
------------------------------------------------------------
------------------------------------------------------------
------------------------------------------------------------
------------------------------------------------------------
----LPIRSKDK--VL-----------------------------YE-------------
IFRPDHTSITLMLPVNTSVQEMMS--------SLVNPG---------------GDH--VL
VKMNSM----------------------G------DRAQLKLDATAVHTSLGLNERLFLC
TASQ---V-EQLT-PLKEQQ---------------------------GP------DQSTI
DT--LEQMCSKDIASQLTNYDWELFTAMHEVELVY-------YIFGRHKF-PGATTANLE
RFVHR---------FNEVQYWV-----VTEVCLCADLMKR------AMLLKKFIKMAAML
---------------KEQKNLNSFFAVMF-GLSNSAVQRLYKTWE---------------
-------RVPSKTKRVYCA-YERLM--------------------------DPSRNH---
-RAYRL----AVAK-------LSPPYI-PFMP----------------------------
LLLKDMTFIHEGNK--NY-TE-----------------------------K-LVNFE-KM
-------RM---IAKTVKIVRGCRSTPY------------------------VPSSPQKG
LADRMFLEAPSIRVSTYSEQSLPLR-------N--------------PSNIRH-----YV
QNFEVIDNQRKL-----------------------------------------TQLSRGM
-----------ER-----------------------------------------------
------------------------------------------------------------
------------------------------------------------------------
------------------------------------------------------------
------------------------------------------------------------
------------------------------------------------------------
------------------------------------------------------------
------------------------------------------------------------
------------------------------------------------------------
------------------------------------------------------------
------------------------------------------------------------
---
>Rapgef3_[Sarcophilus_harrisii]_821489509
------------------------------------------------------------
------------------------------------------------------------
------------------------------------------------------------
------------------------------------------------------------
-------------------------------------------MVLRKIHRPWSCSYQ-L
QLE------------------------HRTPPS---------RIQ---------------
------------------GLR-------------------WT------------------
-----PLTNSNETLDFHV-NLEQA-------------------T--------S-------
-------EHVL-------------RA---------------------GKLLH-RHLLATF
PT-L----------------IRDRKYH------L-RLY----------------RQC-CS
GK-----------------ELVDGVLALG------------------------------L
----GVQ--------SRNQAMGIC---QVLLNEGALC-----------------------
--------------H------------------------VKH-EWAFHDRDTHFYRFPGP
-------ETDP--DNIQEM-----------------------------------------
------------------------------------------------------------
---------EE-L------AEAVTLLSQRGPDAL-------------LTVALRKPPGQRT
EEELD-----LIFEELLHIKAVAHLS-NSVKKELA-AVLLFEPSSKAGTVL---------
--------------------------------------------FSQGDKG--TSWYIIW
KGSVNVVTHGK-----GL-------------------------------VTTLHEG-DDF
GQLAL-----------VNDSPRA---ATIILREDNCQFLRVDKQDFNR------IIKD--
---------------------------------------------V--------------
------------------------------------------------------------
------------------------------------------------------------
------------------------EANTMRLEEHGKVVLVLER-------T------PQS
```

NG-----PDCS------------------LNPGRN------------R-YTVMSGTPEKI
LELLLEA------------VRPDSSAHDPT-ETF-------------------LG---D-
FLLTHSV--FM---------------PSAHLCAALL-----HHF------H---------
-----------------------------AEPA---GGTEQEHGIYICNKRQKILRLVSQ
WVTLYGPLLHID-PVAPSFLQ----KLSEL--VNRDT--QLISLLR---EQ-GPERRR-H
CGL----ENGGGNM--SP------QLKDR----------------------TIP------
-----IW-----------FPSYD--------DH---------------------------
-----------------F--
---LSNR---

----YAIQALDK--VP-----------------------------YD-------------
ICRLDHSILTLHLPVTASVREVMN--------TLAQEDG-----WT------QGQ--VL
VKVTSS----------------------GAIASPTDTISLQPDARGVATSLRLNERLFIV
NPQE---M-HKLT-PHPDQL--------------------------GP------NVGSA
EM--LDLLSAKDLANQLTDHDWNLFKSIHQVELIH-------YVLGPQQL-RNATTANLE
RFMRR---------FNELQYWV-----ATELCLCPVPGLR------TQLLRKFIKLASHL
---------------KEQKNLNSFFAIMF-GLSNSAISRLALTWE---------------
-------RLPHKVRKLYSA-LEQLL--------------------------DPSWNH---
-RVYRL----ALTK-------LSPPII-PFMP----------------------------
LLLKDMTFIHEGNH--TL-VD-----------------------------S-LINFE-KM
-------RM---MARTVRILHHCRSHSS------------------------APLSPLRN
RVSHIHEDSQAVKIYTCSEQSLSAR-------N--------------LASARA-----YI
QQLKVIDNQQEL---SRLSKEL
-----------EP---

>Rapgef3_[Scleropages_formosus]_938064599

--MHLFRSYNYR-V
F----PE-----------------RCSVD-TTSSCIR-----------------------
------------------GIS-------------------WT------------------
-----PLPETLET---QD-TMKQC-------------------LYLCLSQFLF-------
-------DRIL-------------KA---------------------ARSVY-NALIDRS
PG-L----------------IRDRKYH------L-KNY----------------RQC-VT
GK-----------------ELVDWLLKLN------------------------------C
----CVQ--------SRSQAVGLW---QVLMDEKILV-----------------------
--------------H------------------------VKH-ELNFQDREYQFYRFLDV
-------ELGLNRMNNEK--
DS--

---------EEEL------QESLLLLGQLGPDAL-------------LNMILRKCPSQRT
AEDVE-----VIYEELLHIKAVAHLS-SSVRKELA-AVLVFESHAKAGTVL---------
--FSQGDKG--TSWYIIW
KGSVNVVTHGK-----GL-------------------------------VTTLHEG-DDF
GQLAL-----------VNDAPRA---ATIILREDNCHFLRVDKQDFIH------ILKD--
---V--------------

------------------------EANTVRLEEHGRVVLVLEK-------S------CRN
ES-----ASQG------------------SSGNGN------------K-YTVMSGTPEKI
LEHVLES------------VKLDASSNDPV-GAFTACSMLQILSDSGCRDARCWL---D-
TVISDAV--VMVVLSAEFTKFTGEAKKSGQTVLNPL-----PTY------H---------
-----------------------------AEPSE---GSELEKAAYSLNKKQKIVRLVVQ
WVLLYGLLLKDD-PPVLEFLE----RLKAE--VAADS--RFASMLK---EQ-LRDKRR--
TRA---------------------QMT---------------------------------
------W-----------T---

--LC
SGPP---Q----T-PLKEQL---------------------------GP------EQGTM
DS--LEQMSSKDIASQLSDYDWELFGTMPEVELVY-------YVFGRHKF-PGATTANLE
RFVRR---------FNEVQYWV-----VTEMCLCADL-KR------ALLLKKFIKMATIL
---------------KEQKNLNSFFAVMF-GLSNCAVQRLSKTWE---------------
---DPSRNH---
-RAYRL----AVAK-------LNPPYI-PFMP----------------------------
LLLKDMTFIHEGNN--NY-TE-----------------------------N-LVNFE-KM
-------RM---IAKTVKTVRECRSQPY------------------------IPSSPQRS
LADRMFLDAPPVRISTYSEQSLSMR-------S--------------PTTVRQ-----YV
QNLQVIDNQRKL---TQLSRAV
-----------EH---

>Rapgef3_[Sinocyclocheilus_anshuiensis]_1025146268

--MEDIHARMLS-F
F----KQ-----------------ILSAKKK--NRLEAPAIMCSEDTFLRMSLR------

```
-----------------SSLE-------------------KT------------------
-----PLPEAEDT---QD-TIKQ---------------------------FLS-------
-------DRIL-------------KA---------------------ARVVY-STLMERN
PG-L----------------IRDRKHH------L-KTY----------------RQC-CS
GK-----------------ELVDWLMKLN------------------------------D
----CFQ--------SRSQAVGMW---QVLVDEGILS-----------------------
--------------H------------------------VKQ-ELNFHDKDTQFHRFMEA
-------EF---------------------------------------------------
-D---------LNHTTNEKDSK--------------------------------------
---------DDEL------QESLSLLVQMGPDAL-------------LTMILRKCPSQRT
PEDLE-----VIYEELLHVKAVAHLS-TSVRKELA-AVLVFESHAKAGTVL---------
--------------------------------------------FSQGDKG--TSWYIIW
RGSINVITHGK-----GL-------------------------------VTTLHEG-DDF
GQLAL-----------VNDAPRS---ATIILREDNCHFLRVDKQDFIR------ILKD--
---------------------------------------------V--------------
------------------------------------------------------------
------------------------------------------------------------
------------------------EANTVRLEEHGQVVLVHLS-------T---------
YQ-----GG---------------------SSSSS------------K-YTVMSGTPEKI
LEHMLEM------------IKLETNGTDFI-DP-------------------SVT---D-
FLLTHPV--FM---------------PCSQLCAALQ-----HHY------Q---------
-----------------------------VEPSE---GTDLEKAAYALNTKQKVVKLVAH
WVALYGPLLRDN-PVASEFLE----KFREA--VMADS--RLSSMLR---EQ-LRDRRK--
TKI---TENGCHTL--T-------KLN-QKFD----------------------------
------W-----------FSAYE--------EP---------------------------
------------------------------------------------------------
---VGKL-----------------------------------------------------
------------------------------------------------------------
------------------------------------------------------------
------------------------------------------------------------
------------------------------------------------------------
----RSIKAQDK--VL-----------------------------YE-------------
IFKPDHKAVTVILPVDASVKDILT--------TLVDPE---------------RDY--VL
VKMNSS----------------------G------DRVQLKLETTAVSASLGVNEKLFLC
SASQ---V-NQLT-PVKEQL---------------------------GP------EKTTM
DT--LEQMCSKDMASQHTSYDWELFMAMHEVELVY-------YVLGREKF-LGATTANLE
RFVRR---------FNEIQYWV-----VTELCLCEDLVKR------AILLKKFIKMAVVF
---------------KEQKNLNSFFAVMF-GLSNSAVQRLNKTWE---------------
-------RLPNKTKRIYCA-YERLM--------------------------DPSRNH---
-RAYRL----AVAK-------LSPPYI-PFMP----------------------------
LLLKDMTFIHEGNK--NY-TD-----------------------------K-LVNFE-KM
-------RM---IARTVKTVRQCRSQPY------------------------VPSSPQKG
LTERMFLDAQAIRLSTYSDQSLTLR-------S--------------PANIRQ-----YI
QNLKVIDNQKKL-----------------------------------------TQLSRAI
-----------ER-----------------------------------------------
------------------------------------------------------------
------------------------------------------------------------
------------------------------------------------------------
------------------------------------------------------------
------------------------------------------------------------
------------------------------------------------------------
------------------------------------------------------------
------------------------------------------------------------
------------------------------------------------------------
```


>Rapgef3_[Sinocyclocheilus_grahami]_1020398505

---MCSEDTFLLMSLR------
-----------------SSLE-------------------KT------------------
-----PLPEAEDT---QD-TIKQ---------------------------FLS-------
-------DRIL-------------KA---------------------ARVVY-STLMERN
PG-L----------------IRDRKHH------L-KTY----------------RQC-CS
GK-----------------ELVDWLMKLN------------------------------D
----CFQ--------SRSQAVGMW---QVLVDEGILS-----------------------
--------------H------------------------VKQ-ELNFHDKDTQFYRFMEA
-------EF---
-D---------LNHTTNEKDSK--------------------------------------
---------DDEL------QESLSLLVQMGSDAL-------------LTMILRKCPSQRT
PEDLE-----VIYEELLHVKAVAHLS-TSVRKELA-AVLVFESHAKAGTVL---------
--FSQGDKG--TSWYIIW
RGSINVITHGK-----GL-------------------------------VTTLHEG-DDF
GQLAL-----------VNDAPRS---ATIILREDNCHFLRVDKQDFIR------ILKD--
---V--------------

------------------------EANTVRLEEHGQVVLVHLS-------T---------
YQ-----GG---------------------SASSS------------K-YTVMSGTPEKI
LEHMLET------------IKLETNGTDFI-DP-------------------SVT---D-
FLLTHPV--FM---------------PCSQLCAALQ-----HHY------Q---------
-----------------------------VEPSE---GTDLEKAAYALNTKQKVVKLVAH
WVALYGPLLRDN-PVASEFLE----KFREA--VMADS--RLSSMLR---EQ-LRDRRK--
TKI---TENGCHTL--T-------KLN-QKFD----------------------------
------W-----------FSAYK--------EP---------------------------

---VGKL---

----RSIKAQDK--VL-----------------------------YE-------------
IFKPDHKAVTVMLPVDASVKDILT--------TLVDPE---------------SDY--VL
VKMNSS----------------------G------DRVQLKLETTAVSASLGVNEKLFLC
SASQ---V-KQLT-PVKEQL---------------------------GP------EKSTM
DS--LEQMCSKDMASQHTSYDWELFMAMHEVELVY-------YVLGREKF-LGATTANLE
RFVRR---------FNEIQYWV-----VTELCLCEDLVKR------AILLKKFIKMAVVF
---------------KEQKNLNSFFAVMF-GLSNSAVQRLNKTWE---------------
-------RLPNKTKRIYCA-YERLM--------------------------DPSRNH---
-RAYRL----AVAK-------LSPPYI-PFMP----------------------------
LLLKDMTFIHEGNK--NY-TD-----------------------------K-LVNFE-KM
-------RM---IARTVKTVRQCRSQPY------------------------VPSSPQKG
LTERMFLDAQAIRLSTYSDQSLTLR-------S--------------AANIRQ-----YI
QNLKVIDNQKKL---TQLSRAI
-----------ER---

>Rapgef3_[Sinocyclocheilus_rhinocerous]_1025211968

--MEDIHARMLS-F
F----KQ-----------------ILSAKKK--NRLEAPAIMCSEDTFLRMSLR------
-----------------SSLE-------------------KT------------------
-----PLPEAEDT---QD-TIKQ---------------------------FLS-------
-------DRIL-------------KA---------------------ARVVY-STLMERN
PG-L----------------IRDRKHH------L-KTY----------------RQC-CS
GK-----------------ELVDWLMKLN------------------------------D
----CFQ--------SRSQAVGLW---QVLVDEGILS-----------------------
--------------H------------------------VKQ-ELNFHDKDTQFYRFMEA
-------EF---
-D---------LNHTTNEKDSK--------------------------------------
---------DDEL------QESLSLLIQMGPDAL-------------LTMILRKCPSQRT
PEDLE-----VIYEELLHVKAVAHLS-TSVRKELA-AVLVFESHAKAGTVL---------
--FSQGDKG--TSWYIIW
RGSVNVITHGK-----GL-------------------------------VTTLHEG-DDF
GQLAL-----------VNDAPRS---ATIILREDKCHFLRVDKQDFIR------ILKD--
---V--------------

------------------------EANTVRLEEHGQVVLVHLS-------T---------
YQ-----GG---------------------SASSS------------K-YTVMSGTPEKT
LEHMLEM------------IKLETNGTDFI-DP-------------------SVT---D-
FLLTHPV--FM---------------PCSQLCAALQ-----HHY------Q---------
-----------------------------VEPSE---GTDLEKAAYALNTKQKVVKLVAH
WVALYGPLLRDN-PVASEFLE----KFREA--VMADS--RLSSMLR---EQ-LRDRRK--
TKI---TENGCHTL--T-------KLN-QKFD----------------------------
------W-----------FSAYE--------EP---------------------------

---VGKL---

----RSIKAQDK--VL-----------------------------YE-------------
IFKPDHKAVTVMLPVDASVNDILT--------TLVDPE---------------RDY--VL
VKMNSS----------------------G------DRVQLKLETTAVSASLGVNEKLFLC
SASQ---V-KQLT-PVKEQL---------------------------GP------EKSTM
DS--LEQMCSKDMASQHTSYDWELFMAMHEVELVY-------YVLGREKF-LGATTANLE

RFVRR---------FNEIQYWV-----VTELCLCEDLVKR------AILLKKFIKMAVVF
---------------KEQKNLNSFFAVMF-GLSNSAVQRLNKTWE---------------
-------RLPNKTKRIYCA-YERLM--------------------------DPSRNH---
-RAYRL----AVAK-------LSPPYI-PFMP----------------------------
LLLKDMTFIHEGNK--NY-TD-----------------------------K-LVNFE-KM
-------HM---IARTVKTVRQCRSQPY------------------------VPSSPQKG
LTERMFLDAQAIRLSTYSDQSLTLR-------S--------------AANIRQ-----YI
QNLKVIDNQKKL---TQLSRAI
-----------ER---

>Rapgef3_[Sorex_araneus]_836708039

---MVLRRMHRPRSCSYQ-L
LLE------------------------PQRP-S---------RIQ---------------
------------------GLR-------------------WT------------------
-----PLTDSEESLDFSG-SLEQA-------------------S--------P-------
-------ERVL-------------RA---------------------GKQLH-RHLLATC
PN-L----------------IRDRKYH------L-RLY----------------RQC-CS
GR-----------------ELVEGILALG------------------------------F
----GVH--------SRSQAVGIC---QVLLDEGALC-----------------------
--------------H------------------------VKH-DWTFQDRDTQFYRFPGP
-------EPEL--AGPREL---

---------EEEL------MEAVALLSQRGPDAL-------------LTVALRKPPGQRT
DEELD-----LIFEELLHIKAVAHLS-NSVKRELA-AVLLFEPHSKAGTVL---------
--FSQGDKG--TSWYIIW
KGSVNVVTHGK-----GL------------------------------VTTLHEG-EDF
GQLAL-----------VNDAPRA---ATIIILREDNCHFLRVDKQDFNR-----IIKD--
---V--------------

------------------------EAKTMRLEEHGKVVLVLER-------A------SQG
PA-----SSCP------------------PTPGRN------------R-YTVMSGTPEKI
LELLLEA------------MRPDSNAHDPT-ETF-------------------LS---D-
FLLTHSV--FM---------------PSAQLCTALL-----HHF------H---------
-----------------------------AEPA---GGSEQERSTYICNKRQQILRLVSR
WAALYGPMLHTD-AVATGFLQ----KLSEL--VSRDA--RLSHLLQ---EPLWPDRRRHH
HRL----ENGCGNA--SP------QMKAR----------------------NVP------
-----VW-----------LPGQD--------ER---------------------------
-----------------I--
---PSSN---

----CTIRVGDK--VS-----------------------------YD-------------
ICRRDHSVLSLQLPVTASVREVMA--------ALAQRDG-----WT-------KGQ--VL
VKVNSA----------------------G------DAIGLQLDARGVATSLGLNERLFVV
DPED---V-HELT-PHPEQL---------------------------GP------TVGSA
EG--LDLVSAKDLAGQLTEHDWSLFNSIHQVELIH-------YVLGPQHL-RDVTTANLE
RFLRR---------FNELQYWV-----ATELCLCPVPGPR------AQLLRKFIKLAAHL
---------------KEQKNLNSFFAVMF-GLSNSAISRLAHTWE---------------
-------RLPQKVRKLYSA-LERLL--------------------------DPSWNH---
-RVYRL----ALTK-------LSPPVI-PFMP----------------------------
LLLKDMTFIHEGNH--TL-VE-----------------------------N-LINFE-KM
-------RM---MARAVRMLHHCRSHSN------------------------VPLSPLRS
RVSHLHEDSQAARISTCSEQSLGTR-------S--------------PASTWA-----YV
QQLKVIDNQREL---SRLSREL
-----------EP---

>Rapgef3_[Struthio_camelus_australis]_697484424

---MS-----------

----------------------------------WQ------------------------
---T------------------
-----PLLD-------SESTLDYGH------------------SLTQAS---S-------
-------EKIW-------------RAGKLLF---------------S----H---LMITC
PG-L----------------IRDHKHH------L-RHH----------------RQC-CS
GK-----------------ELVDWLLSAG------------------------------L
----SIQ--------TRSQAIGVC---QVLVDGGVLT-----------------------
--------------H------------------------VKQ-EWHFQDKDTQFYRFAEL
E-----LSPEP-SNGLRDA---

----------EEL------LEALAFLAQLGPDAL-------------LTMALRKPPTQRT
EDELE-----LIFEELLHIKAVAHLS-NSVKRELA-SVMMFESHQRAGTV----------
---LFSQGDKG--TSWYIIW
KGSVNVVTHGK-----GL-------------------------------VATLHEG-DDF
GQLAL-----------VNDAPRA---ATIILREDNCHFLRVDKQDFNR------ILKD--
---V--------------

------------------------EANTMRLKEHGKVVLILQKNL-------------QG

G------SSQP------------------AAARSS-R------------YSVMAGTPEKI
LEHLLEF------------MRLDATLYDPV-D-------------------NLLG---D-
FLL---F--FL---------------PTSQLCRALL-----HHF------R---------
-----------------------------TEPLE---GLEQEKATYSLHKRRKILRLVSQ
WVLLYGRLLQGD-RTTTALLQ----SLAD--LASQDP--RLGGLIQ---EQ-AQDRRR--
PRP---LENGGGSI--SP------QPKARS---------------------SVN------
------W-----------FAGQE--------EA---------------------------
----------------IL--
----NSSC-AL-----------------RAQDK---------------------------

--------------VP-----------------------------YE-------------
IYRPDHSCLITVLPVNASVRDILR--------SLTPKLG-------------HEDE-HIL
VKVNSA----------------------GDKVG------LQMDAVGVFTSLGLNERLFAV
SMEE---L-CSLT-PHPEQL---------------------------GP------STGSS
ET--LDLISSKDLASHLTDYDWNLFKSIHQVEMIH-------YIIRPQKF-HDVTTANLE
RVMRR---------FNELQYWV-----ATELCLCPEVGRR------AQLLRKFIKLAAHL
---------------KEQKNLNSFFAVMF-GVSNTAVSRLAKTWE---------------
-------KLPHKIRKLHAA-LERML--------------------------DPSWNH---
-RVYRL----AVAK-------LSPPII-PFVPL-LLK-----------------------
----DMTFIHEGNR--TLA------------------------------EN-LINFE-KM
-------HM---MAKTVRVLQRCRG--HT----------------------HAPVSPLRS
RSPHRLEDPKAMRIST--
-----------C---SEQSLSV
RSPVSTWAY-LQHLKAIDSQKELLRLSRDLES----------------------------

>Rapgef3_[Sturnus_vulgaris]_959091038

--DKDSQFYRLAEL
E-----PSPER-GGGPRDP---

----------EEL------QEALGVLAQLGPDAL-------------LATALRKPPAQRT
QDELE-----LIFEELLHIKAVAHLS-NSVKRELA-AVLMFEGHQRA-------------
---EQRDRS---------
----GVTT--
--------------------PR-------------CHHPAVPPPPP--------------

---R-YLVMAGTPEKI
LEHLLEF------------MRLDATLHDPV-D-------------------TLLG---D-
FLLTYTV--FM---------------PTSQLCRALL-----HHF------R---------
-----------------------------AEPLE---GSEQDKVAYSLRKRRKILRLVSQ
WVLLYGRLLQGD-RSTTALLQ----NLAD--LASRDP--QLGGLGQ---EQ-GQERRR--
PRG---

------YLGVRGVGTALTC-------RLSQVEMIH-------YIVGPHKS-HEVATANLA
RVLRR---------FNELQFWV-----ATELCLCPELGRR------ARLLRKFIKLAAHL
---------------KEQKNLNSFFAVMF-GVSNTAVTRLAKTWE---------------
-------MLPHKIRKLHSA-LE--------------------------------------

---------------------R---------------------------------MLLWS
WWPWL-----LSMAPT--
-----------V---PLVAPTV
P------LV-APTVPLVAPALPLVAPTVPLVAPTVPLVAPALPLVAPTLPLVAPTVPLVA
------PTVPLVALGCPGAAPQVSSGQVRADLYTAPDSCEGRCGEPFSEEDECHCHRECH
SQHNCCQDHERHCGS---

>Rapgef3_[Sus_scrofa]_545893683

---------------------M--------------------------------------

---------------K---------------------------------VGWPGES----
-----RWQVGLAVED-GSALGAPTVGG----LPDVVPEGTLLXXXXXXXHRPRSCSYQ-L
LLE------------------------HQRP-S---------RIQ---------------

```
------------------GLR-------------------WT------------------
-----PLTNSEESLDFSV-SLEQA-------------------S--------T-------
-------ERVL-------------RA---------------------GKQLH-RHLLPS-
PG-W----------------DRDTQF-----------Y----------------R-----
---------------------FPGLEPEP------------------------------A
----GVH--------ELEEELVEA---LTLLSQ---------------------------
---------------------------------------------------------RGP
-------D----------------------------------------------------
------------------------------------------------------------
-----------------------ALL----------------------TVALRKPPGQRT
DEELD-----L--------------------------VLLFEPHSKAGTVL---------
--------------------------------------------FSQGDKG--TSWYIIW
KGSVNVVTHGK-----GL-------------------------------VTTLHEG-DDF
GQLXX-----------XXXXXXX---XXXXXXEDNCHFLRVDKQDFNR------IIKD--
---------------------------------------------V--------------
------------------------------------------------------------
------------------------------------------------------------
------------------------EAKTMRLEEHGKVVLVLER-------S------SQG
SG-----PSRP------------------PTPGRN------------R-YTVMSGTPEKI
LELLLEA------------MRPDSSAHDPT-ETF-------------------LS---D-
FLLTHSV--FM---------------PNAQLCAALL-----HHF------H---------
-----------------------------AEPA---GGSEQECSTYVCNRRQQILRLVSQ
WVALYGPMLHTD-PVATSFLQ----KLSDL--VSKDA--RLSNLLR---EQ-WPERRR-H
HRG----SEGCGDALLSPLGLPFSPLQAR----------------------HMP------
-----VW-----------LPSQD--------EP---------------------------
-----------------L------------------------------------------
---PSSN-----------------------------------------------------
------------------------------------------------------------
------------------------------------------------------------
------------------------------------------------------------
------------------------------------------------------------
----CAIRAGDK--VP-----------------------------YD-------------
ICRPDHSVGTLQLPVTASVREVMA--------ALAQEDG-----WT-------KGQ--VL
VKVNSA----------------------G------DAVGLQPDARGVATSLGLNERLFVV
NPQE---V-HELT-PHPEQL---------------------------GP------TVSSA
EG--LDLVSTKD------------------------------------------------
---RR---------FNELQYWV-----ATELCLCSVPSLR------AQLLRKFIKLAAHL
---------------KEQKNLNSFFAVMF-GLSNSAISRLAHTWE---------------
-------RLPHKVRKLYSA-LERLL--------------------------DPSWNH---
-RVYRV----ALTK-------LSPPVI-PFMP----------------------------
LLLK-------GKR-------------------------------------------TSA
-------RVP--ASTPSILVSVCLSDPP--------------------------------
-----------------SDSNVLTP--------------------------WS-----TS
SNGESIGWKRS-------------------------------------------------
------------------------------------------------------------
------------------------------------------------------------
------------------------------------------------------------
------------------------------------------------------------
------------------------------------------------------------
------------------------------------------------------------
------------------------------------------------------------
------------------------------------------------------------
------------------------------------------------------------
------------------------------------------------------------
```


>Rapgef3_[Takifugu_rubripes]_768914878

---MENIV-K
SICYPPR-----------------RYTPV-------------------------------
-------------------MR-------------------ET------------------
-----PLPESLGS---KD-TMKQ---------------------------FLS-------
-------DRVV-------------KA---------------------ARSVY-SVMIEKN
PG-L----------------IRDRKYH------L-KTY----------------RQC-CS
GK-----------------ELVDWLMKQS------------------------------D
----CLQ--------SRSQVVGMW---QVLVDEGILV-----------------------
--------------H------------------------VKQ-ELNFHDKDTQFFRFRDS
-------ECGLNHVTNER--
DA--
---------EDEL------QEGLSLLSQLGPDAL-------------LTMILRKCPSQRS
AEDLE-----VIYEELLHVKAAAHLS-TSVRKELA-AVLVFESHAKAGTVL---------
--FSQGDKG--TSWYIIW
KGSVNVITHGK-----GL-------------------------------VTTLHEG-EDF
GQLAL-----------LNDAPRA---ATIILREDNCHFLRVDKQDFIR------ILKD--
---V--------------

------------------------EANTVRLEEHGKTVLVLEK-------S---------
SD----RAEQT------------------GAGSNS------------K-YTVMSGTPEKI
LEHLLET------------IKLDSNGNDGI-DP-------------------CVS---D-
FLLTHKV--FM---------------SSSQLCSALL-----HQY------Q---------
-----------------------------AELSK---GSDQEKASYILTAKRKVVKLVCH
WVALYDLLLKED-PVALDFLE----RLKKE--VAADY--RLSGILK---EQ-FRERRR--
TRI---LDNGYQSL--C-------R-N-QKFD----------------------------
------W-----------FLHCE--------EP---------------------------

---VGRL---

----QPIRAHDK--VL-----------------------------YE-------------
ICRADSKALTVMLPVNTTVQDVIS--------AVVKPG---------------GDH--VL
VKMNSA----------------------G------ERAQLKLDAAAVYTALGVNERLFIC
TSSQ---V-EKLF-PLKEQQ---------------------------GP------ERGTA
DI--LGQMGSKDIATELTNYDWELFAAMHEAELIY-------YVFGRHKF-PGATTANLE
RFVRR---------FNEVQHWV-----VTELCLCEDLVKR------AVLLKKFIKIASVL
---------------KEQKNLNSFFAVMF-GLSNSAVRRLYKTWE---------------
-------RIPSKTKRIYCS-YERLM--------------------------DPSRNH---
-RAYRL----TVAK-------LGPPYI-PFMP----------------------------
LLLKDMTFINEGNP--NY-LE-----------------------------K-LVNFE-KM
-------RM---IAKTVKVVRGCRSHPY------------------------TPSSPQRS
LADRMFLDGPTTRMSTCSEHGLLLR-------S--------------PSNIRH-----YV
QNLKVIDNQRKL---TQLSRTL
-----------EC---

>Rapgef3_[Thamnophis_sirtalis]_927194898

--MY
PD-L----------------IRDHKHH------L-RLH----------------RQC-CS
GK-----------------ELVDALLSAG------------------------------L
----SVQ--------TRSQALGLC---QVLMDEGVLT-----------------------
--------------H------------------------VRQ-ESYFQDRDAQFFRFVAL
-------EP------SAE--
-----------------DRDG---------------------------------------
----------EDL------LEALALLTQLGPTAL-------------LTTILRKPPNQRT
DEELA-----LIFEELLHVKAVAHLS-SSVRRELA-AVLLFESHAKAGTVL---------
--FSQGDKG--TSWYIIW
KGSVNVVTHDK-----GL-------------------------------VCTLHEG-DDF
GQLAL-----------VNDAPRA---ATIIILREDSCHFLRVDKRDFNR-----ILKD--
---V--------------

------------------------EANTLRLKEHGKVVLVLEK-------N---------
SQ-GSNSSHLA------------------GTLGSS------------R-YSVIAGTPEKI
LDYLLET------------MRSDSTLSDPV-DTF-------------------LG---D-
FLLTYSV--FM---------------PTPQLCRTLL-----QHF------H---------
-----------------------------SEPSE---GSEQDKAAYILRKRQKILWLVNR
WVSLYGRLLQAE-TCAVAFLQ----TMSEC--ATQDA--RLGPLLR---EQ-IQDRRR--
NRV---IENGNNCA--SPK----LQVR-NAVN----------------------------
------W-----------FAVQE--------EP---------------------------
-----------------F--
---LNSS---

----YTIRMKDK--GH-----------------------------HD-------------
FPA---

>Rapgef3_[Tinamus_guttatus]_719772822

---MSER---------
---R-----GDLSRM----------------------------SAETQG----------P
VKVAADGNRLSV--LLKKM--------HLFRSSSYEI-------------RLEGE-GGSL
PRIQG-------IRW--------------------------T------------------
-----PLLD-------SESALDYGH------------------SLTQAS---S-------
-------EKIW-------------RAGKLLF---------------S----H---LVITC
PG-L----------------IRDHKHH------L-RHH----------------RQC-CS
GK-----------------ELVDWLLGAG------------------------------L
----GVQ--------TRSQAVGVC---QVLVDGGVLT-----------------------
--------------H------------------------VKQ-EWHFQDKDTQFYRFAEL
E-----LSPEP-GHGLRDA---

----------EEL------LEALGYLAQLGPDAL-------------LTMALRKPPTQRT
EDELE-----LIFEELLHIKAVAHLS-NSVKRELA-AVLMFESHPRAGTV----------
---LFSQGDKG--TSWYIIW
KGSVNVVTHGK-----GL-------------------------------VATLHEG-DDF
GQLAL-----------VNDAPRA---ATIILREDNCHFLRVDKQDFNR------ILKD--
---V--------------

------------------------EANTVRLKEHGKVVLILHKNL-------------QG
G------SSQS------------------VTARSS-R------------YSVMAGTPEKI
LEHLLEF------------MRLDATLYDPA-G-------------------E--G---D-
SLHLPLP--FS---------------EENRLCRALL-----HHF------R---------
-----------------------------AEPLE---GSEQEKATYSLHKRRKILRLVSQ
WVLLYGRLLQGD-RSTTALLQ----NLAD--LASQDP--RLGGMVQ---EQ-AQDQRR--
PRP---LENGGGSV--SP------QPKARS---------------------SVN------
------W-----------FSGQE--------EA---------------------------
----------------IL--
----NSSC-AL-----------------RAQDK---------------------------

--------------VP-----------------------------YE-------------
IYRPDHSCLITVLPVNASVRDILR--------ALAPKLG-------------RDGE-HLL
VKVNSA----------------------GDKVG------LQMDAVGVFTSLGLNERLFAV
SVEE---L-CGLI-PHPEQL---------------------------GP------STGSS
ET--LDLISSKDLASHLTDYDWNLFKSIHQVEMIH-------YIVRPQKF-HDVTTANLE
RVMRR---------FNELQYWV-----ATELCLCPEVGRR------AQLLRKFIKLAAHL
---------------KEQKNLNSFFAVMF-GVSNTAVSRLAKTWE---------------
-------RLPHKIRKLHSA-LERML--------------------------DPSWNH---
-RVYRL----AVAK-------LSPPII-PFVPL-LLK-----------------------
----DMTFIHEGNR--TLA------------------------------EN-LINFE-KM
-------HM---MAKTVRVLQRCQG--HA----------------------HAPLSPLRS
RSPHRLEDPKAMRIST--
-----------C---SEQSLSV
RSPVSTWAY-LQHLKAIDSQKELLRLSR--VA----------------------------

>Rapgef3_[Trichechus_manatus_latirostris]_823404739

---MVLRRMHRPRSCSYQ-L
LLE------------------------HQRP-S---------RIQ---------------
------------------GLR-------------------WT------------------
-----PLTDSEESLDFSV-SLEQA-------------------S--------T-------
-------ERVL-------------RA---------------------GKQLH-RHLLATC
PN-L----------------IRDRKYH------L-RLY----------------RQC-CS
GR-----------------ELVDGILALG------------------------------L
----GVH--------SRSQAVGIC---QVLLDEGALC-----------------------
--------------H------------------------VKH-DWTFQDRDAQFYRFPGP
-------EPEP--VGAHET---

---------EEEL------VEAVALLSQRGPDAL-------------LTVALRKPPGQRT
DEELD-----LIFEELLHIKAVAHLS-NSVKRELA-AVLLFEPHSKAGTVL---------
--FSQGDKG--TSWYIIW
KGSVNVVTHGK-----GL-------------------------------VTTLHEG-DDF
GQLAL-----------VNDAPRA---ATIILREDNCHFLRVDKQDFNR------IIKD--
---V--------------

------------------------EAKTMRLEEHGKVVLVLER-------T------SQG

AG-----PSRP------------------PTPGRN------------R-YTVMSGTPEKI
LELLLEA------------MRPDSSAHDPT-ETF-------------------LS---D-
FLLTYSV--FM---------------PSAQLCAALL-----HHF------H---------
-----------------------------AEPA---GGSEQERSTYVCNKRQQILRLVNQ
WVALYGPMLHSD-PVATSFLQ----KLSDL--LSRDA--RLSNLLK---EQ-WPERRR-H
HRL----ENGCGSA--SP------QMKAR----------------------NVP------
-----VW-----------LPSQD--------EP---------------------------
-----------------L--
---PSSN---

----CAIRVGDK--VP-----------------------------YD-------------
ICRPDHSVLTLQLPVTASVREVMA--------ALAQEDS-----WT-------KGQ--VL
VKVNSA----------------------G------DAIGLQPDARGVATSLGLNERLFVV
NPQE---V-HKLT-PHPEQL---------------------------GP------TVGSA
EG--LDLVSAKDLAGQLTDHDWSLFNSIHQVELIH-------YVLGPQHL-RTVTTANLE
RFMRR---------FNELQYWV-----ATELCLCPVPGLR------AQLLRKFIKLAAHL
---------------KEQKNLNSFFAVMF-GLSNSAISRLAHTWE---------------
-------RLPHKVRKLYSA-LERLL--------------------------DPSWNH---
-RVYRL----ALTK-------LSPPVI-PFMP----------------------------
LLLKDITFIHEGNH--TL-VE-----------------------------N-LINFE-KM
-------RM---MARAVRMLHHCRSHSN------------------------VPLSPLRS
RVSHLHEDSQPSRISMCSEQSLSTR-------S--------------PASTWA-----YV
QQLKVIDNQREL---SRLSREM
-----------EP---

>Rapgef3_[Tupaia_chinensis]_562882143

---------------------M--------------------------------------

---------------K---------------------------------VGWPGES----
-----RWQVGLGVES-NLALGAPQVGG----LPDVVPEGTLLNMVLRRMHRPRSCSYQ-L
LLE------------------------HRRP-S---------RIQ---------------
------------------GLR-------------------WT------------------
-----PLTNSEESLDFSV-SLEQA-------------------C--------T-------
-------ERVL-------------RA---------------------GKQLQ-RHLLATC
PM-L----------------IRDRKYH------L-RLY----------------RQC-CS
GR-----------------ELVDGILALG------------------------------L
----GVH--------SRSQAVGIC---QVLLDEGALC-----------------------
--------------H------------------------VKH-DWTFQDRDAQFYRFPGL
-------EPEP--MGAHEM---

```
---------EEEL------VEALALLSQRGPDAL-------------LTVALRKPPGQRT
DEELE-----LIFEELLHVKAVAHLS-NSVKRELA-AVLLFEPHSKAGTVL---------
--------------------------------------------FSQGDKG--TSWYIIW
KGSVNVVTHGK-----GL-------------------------------VTTLHEG-DDF
GQLAL-----------VNDAPRA---ATIILREDNCHFLRVDKQDFNR------IIKD--
---------------------------------------------V--------------
------------------------------------------------------------
------------------------------------------------------------
------------------------EAKTMRLEEHGKVVLVLER-------T------SQG
AG-----LSRP------------------PTPGRN------------R-YTVMSGTPEKI
LELLLEA------------MRPDSSAHDPT-ETF-------------------LS---D-
FLLTHSV--FM---------------PNAQLCAALL-----HHF------H---------
-----------------------------AEPA---GGSEQERSTYICNKRQQILRLVSQ
WVALYGPMLHTD-PVATSFLQ----KLSDL--VSRDT--RLSNLLR---EQ-WPERRR-H
HRL----ENGCGNA--SP------QMKAR----------------------NVP------
-----AW-----------LPSQD--------EP---------------------------
-----------------F------------------------------------------
---SSSA-----------------------------------------------------
------------------------------------------------------------
------------------------------------------------------------
------------------------------------------------------------
------------------------------------------------------------
----CAIRVGDK--VP-----------------------------YD-------------
ICRPDHSVLTLQLPVTASVREVMV--------ALSQEDI-----CT-------KGQ--VL
VKVNSA----------------------G------DAIGLQPDARGVATSLGLNERLFVV
NPQE---V-HELT-PHPEQL---------------------------GP------TVGSA
EG--LDLVSAKDLAGQLTDHDWNLFNSIHQVELIH-------YVLGPQHL-RDVTTANLE
RFMRR---------FNELQYWV-----ATELCLCPVPGPR------AQLLRKFIKLAAHL
---------------KEQKNLNSFFAVMF-GLSNSAISRLAHTWQ---------------
-------RLPHKVRKLYSA-LERLL--------------------------DPSWNH---
-RVYRL----ALTK-------LSPPII-PFMP----------------------------
LLLKDMTFIHEGNH--TL-VE-----------------------------N-LINFE-KM
-------RM---MARAVRMLHHCRSHSV------------------------VPLSPLRS
RVSHLHEDSQVARISTCSEPSLSTR-------S--------------PASTWA-----FV
QQLKVIDNQREL-----------------------------------------SRLSREL
-----------EP-----------------------------------------------
------------------------------------------------------------
------------------------------------------------------------
------------------------------------------------------------
------------------------------------------------------------
------------------------------------------------------------
------------------------------------------------------------
------------------------------------------------------------
------------------------------------------------------------
------------------------------------------------------------
------------------------------------------------------------
---
>Rapgef3_[Tursiops_truncatus]_470596594
------------------------------------------------------------
------------------------------------------------------------
------------------------------------------------------------
------------------------------------------------------------
-------------------------------------------MVLRRMHRSRSCSYQ-L
LLE------------------------HQRP-S---------CIQ---------------
```

------------------GLR-------------------WT------------------
-----PLANSEESLDFSV-SLEQA-------------------S--------T-------
-------ERVL-------------RA---------------------GRQLH-RHLLATC
PN-L----------------IRDRKYH------L-RLH----------------RQC-CS
GR-----------------ELVDGILALG------------------------------L
----GVH--------SRSQAVGIC---QVLLDEGALC-----------------------
--------------H------------------------VKH-DWAFQDRDTQFYRFPGP
-------EPEP--AGLHEL---

---------EEEL------LEALALLSQRGPDAL-------------LTVALRKPPGQRT
DEELD-----LIFEELLHIKAVAHLS-NSVKRELA-AVLLFEPHSKAGTVL---------
--FSQGDKG--TSWYIIW
KGSVNVVTHGK-----GL-------------------------------VTTVHEG-DDF
GQLAL-----------VNDAPRA---ATIILREDNCHFLRVDKQDFNR------IIKD--
---V--------------

------------------------EAKTMRLEEHGKVVLVLER-------T------SQG
TG-----PSRP------------------PTPGRN------------R-YIVMSGTPEKI
LELLLEA------------MRPDSSAHDPT-ETF-------------------LS---D-
FLLTHSV--FM---------------PTAQLCAALL-----HHF------H---------
-----------------------------AEPM---GGSEQECSTYICNKRQQILRLVSQ
WVALYGPMLHTD-PVATSFLQ----KLSDL--VSRDA--RLCNLLR---EQ-WPERRR-H
HRL----ENGCGNA--SP------QMKAR----------------------NMP------
-----VW-----------LPSQD--------EP---------------------------
-----------------L--
---PSSN---

----CAIRVGDK--VP-----------------------------YD-------------
ICRLDHSVLTLQLPVTASVREVMA--------ALAQEDG-----WT-------KGQ--VL
VKVNSA----------------------G------DTVGLQPDAHGVATSLGLNERLFVV
NPQE---V-HKLT-PHPEQL---------------------------GP------TVGSA
EG--LDLVSTKDLAGQLTDHDWSLFNSIHQVELIH-------YVLGPQPL-RDVTTANLE
RFMRR---------FNELQYWV-----ATELCLCPVPGLR------AQLLRKFIKLAAHL
---------------KEQKNLNSFFAIMF-GLSNSAISRLAQTWE---------------
-------RLPHKVRKLYSA-LERLL--------------------------DPSWNH---
-RVYRL----ALTK-------LSPPLI-PFMP----------------------------
LLLKDMTFIHEGNH--TL-VE-----------------------------N-LINFE-KM
-------RM---MARAARMLHHCRSHSN------------------------VPLSPLRS
RVSHLHEDSQAARISTCSEQSLSTR-------S--------------PASTWA-----YV
QQLKVIDNQREL---SRLSREL
-----------EP---

>Rapgef3_[Ursus_maritimus]_671024230

---------------------M--------------------------------------

---------------K---------------------------------VGWPGES----
-----RWQVGLAVED-SPALGASQVGG----LPAVVPEGTLLNMVLKRVHRPRSCSYQ-L
LLE------------------------HQRP-S---------RIQ---------------
------------------GLR-------------------WV------------------
-----TWEDSEESLDFSV-SLEQA-------------------S--------A-------
-------ERVL-------------RA---------------------GKQLH-RHLLATC
PT-L----------------IRDRKYH------L-RLY----------------RQC-CS
GR-----------------ELVDGILALG------------------------------L
----GVH--------SRSQAVGIC---QVLLDEGALC-----------------------
--------------H------------------------VKH-DWTFQDRDTQFYRFPGS
-------EPEP--VGIHEL---

---------EDEL------VEAVALLSQRGPDAL-------------LTVALRKPPGQRT
DEELD-----LIFEELLHIKAVAHLS-NSVKRELA-AVLLFEPHSKAGTVL---------
--FSQGDKG--TSWYIIW
KGSVNVVTHGK-----GL-------------------------------VTTLHEG-DDF
GQLAL-----------VNDAPRA---ATIILREDNCHFLRVDKQDFNR------IIKD--
---V--------------

------------------------EAKTMRLEEHGKVVLVLER-------A------SQG
AG-----PSRP------------------PTPGRN------------R-YTVMSGTPEKI
LELLLEA------------MRPDSSAHDPT-ETF-------------------LS---D-
FLLTYSV--FM---------------PSAQLCAALL-----HHF------H---------
-----------------------------AEPS---GGSEQEHSTYICNRRQQILRLVGQ
WVALYGPMLHTD-PVATSFLQ----KLSDL--VSRDT--RLCILLR---EQ-WPERRR-H
HRL----ENGCGNA--SP------QMKAR----------------------NMA------
-----VW-----------LPGQD--------QP---------------------------
-----------------L--
---PSSN---

----CAIRVGDK--VS-----------------------------YD-------------
ICRPDHSVLTLKLPVTASVREVMA--------ALAQEDG-----WT-------KGQ--VL
VKVNSA----------------------G------DAIGLQPEARGVATSLGLNKALF--
---------HLQT-PHPEQL---------------------------GP------TVGSA
EG--LDLVSAKDLAGQLTDHDWNLFNSIHQVELIH-------YVLGPQHL-RG-------
---------------RPAVVWV----GALGVWLFP--------------LRSSGSSAHSL
---------------KEQKNLNSFFAVMF-GLSNSAISRLAHTWE---------------
-------RPPHKVRKLYSA-LERLL--------------------------DPSWNH---
-RVYRL----ALTK-------LSPPII-PFMP----------------------------
LLLKDMTFIHEGNH--TL-VE-----------------------------N-LINFE-KM
-------RM---MARVRS
RVSHLHEDSQAARTSTCSEQSLSTR-------S--------------PASTWA-----YV
QQLKVIDNQREL---SRLSREL
-----------EP---

>Rapgef3_[Vicugna_pacos]_560961023

---------------------M--------------------------------------

---------------K---------------------------------VGWPGES----
-----RWQVGLAVED-GSALGAPLVGG----LPDVVPEGTLLNMVLRRMHLPRSCSYQ-L
LLE------------------------HQRP-S---------RIQ---------------
------------------GLR-------------------WT------------------
-----PLTSSEESLDFSV-SLEQA-------------------S--------T-------
-------ERVL-------------RA---------------------GKHLH-RHLLANC
PN-L----------------IRDRKYH------L-RLH----------------RQC-CS
GR-----------------ELVDGILTLG------------------------------L
----GVH--------SRSQAVGIC---QVLLDEGALC-----------------------
--------------H------------------------VKH-DWAFQDRDTQFYRFPGP
-------EPEP--AGAHEL---

---------EEEL------VEALALLSQRGPDAL-------------LTVALRKPPGQRT
DEELD-----LIFEELLHIKAVAHLS-NSVKRELA-AVLLFEPHSKAGTVL---------
--FSQGDKG--TSWYIIW
KGSVNVVTHGK-----GL-------------------------------VTTLHEG-DDF
GQLAL-----------VNDAPRA---ATIILREDNCHFLRVDKQDFNR------IIKD--
---V--------------

------------------------EAKTMRLEEHGKVVLVLER-------N------SQG
AG-----PSRP------------------PTPGRN------------R-YTVMSGTPEKI
LELLLEA------------MRPDSSAHDPT-ETF-------------------LS---D-
FLLTHTV--FM---------------PSTQLCAALL-----HHF------H---------
-----------------------------AEPA---GGNEQECSTYVCNKRQQILRLVSQ
WVALYGPMLHTD-PVATSFLQ----KLSEL--VSRDA--RLSNLLR---EQ-WPERRR-H
HRL----ENGCGNA--SP------QMKAR----------------------NMP------
-----VW-----------LPSQD--------EP---------------------------
-----------------L--
---PSSN---

----CAIRVGDK--VP-----------------------------YD-------------
ICRPDHSVLTLQLPVTASVREVMA--------ALAQEDG-----WT-------KGQ--VL
VKVNSA----------------------G------DAIGLQPDARGVATSLGLNERLFVV
NPQE---V-HKLT-PHPEQL---------------------------GP------TVGSA
EG--LDLVSTKDLAGQLTDHDWNLFNSIHQVELIH-------YVLGPQHL-RDVTTANLE

RFMRR---------FNELQYWV-----ATELCLCPVPGLR------AQLLRKFIKLAAHL
---------------KEQKNLNSFFAIMF-GLSNSAISRLAHTWE---------------
-------RLPHKVRKLYSA-LERLL--------------------------DPSWNH---
-RVYRL----ALTK-------LSPPVI-PFMP----------------------------
LLLKDMTFIHEGNH--TL-VE-----------------------------N-LINFE-KM
-------RM---MARAARMLHHCRSHSN------------------------VPLSPLRS
RVSHLHEDSQAMRISTCSEPSLITR-------S--------------PGSTWA-----SI
QQLKVIDNQREL---SRLSREL
-----------EP---

>Rapgef3_[Xenopus_laevis]_148227834

--MRH---
--RN---KMHFFRSYSYE-I
Q------------------------VQHRVDIHSHIQ-----------------------
------------------GIR-------------------WT------------------
-----PLPNIESQEEYSQ-TVKQV------------------------V---T-------
-------FKVW-------------RA---------------------AKVLH-TYLLTMS
PA-M----------------MRDRKHH------F-RSH----------------RQC-CS
GK-----------------ELVDWLLGIS------------------------------H
----GLQ--------SRSQALGIW---QVLVDEGALV-----------------------
--------------H------------------------VRQ-EPNFQDKDSQFYRFTRA
-------EP------YVDP--------------------------------LLS------
-----------------DRDT---------------------------------------
---------EEEM------QESLVFLSQLEPAAL-------------LTMILRKPPCQRT
EEELE-----VIFEELLHIKAVSHLS-NSVKKELA-SVLIFESHAKAGAVL---------
--FNQGDKG--TSWYIIW
KGSVNVITHGK-----GL-------------------------------VATLHEG-DDF
GQLAL-----------VNDAPRA---ATIVLREDNCHFLRVDKHDFNR------ILRD--
---V--------------

------------------------EANTVRLKEHEKVVLVLEK-------N---------
PQISTGNNLQH------------------AAASNL------------K-YTVMSGTSDKI
LEHLLET------------MKLDSYSGD----SF-------------------AG---D-
FLLTHSI--FM---------------PTAQLCQALL-----HQF------N---------
-----------------------------VEPSE---VNEPEKATYSLQKQQNILHLVSL
WVSLYGRLLEAE-PSAKSFLE----KLSEH--VSRDP--RLLAFCR---EN-LQDRRK--
TRT---LEVCNGNH--SPQ----IKIN-SYGN----------------------------
------L-----------FSNLE--------ED---------------------------
-----------------V--
---SSNT---

----YTIRAGDK--VP-----------------------------YD-------------
IYRPDHSCITTMLPVNASVQEVIS--------SLATKNGYN------------RDH--II
VKVNSS----------------------G------ERVCLKHEDTGVFTSLGLNERLFIC
YTHE---L-NNVT-ILPEQL---------------------------GP------NTSSF
DI--LELISSKDLAHQMTDNDWNLFKSIHQVELIY-------HTFGKQRF-RNATTANLE
RFMRR---------FNEVQFWV-----ATEVCLCQEEERR------AQLLKKFIKLAAYL
---------------KEQKNLNSFFAVMF-GLSNTAVSRLSRTWQ---------------
-------KLPNKIRKLYSI-FERLM--------------------------DPSWNH---
-RSYRL----AVAK-------LSAPLI-PFLP----------------------------
LILKDLTFLHEGNR--ST-LD-----------------------------N-LVNFE-KM
-------RM---IAKTVQMFHRCRSQAY------------------------VPLSPLRT
RPVYILEDPSAARISACSEHSMTLR-------N--------------PATTWA-----YI
QHMKVIDNQKKL---MELSRSL
-----------EPASSA---

>Rapgef3_[Xenopus_tropicalis]_847104409

-------------------MRII------GQQLRNPCG----------------MRH---
--RN---KMHFFRTYSYE-I
Q------------------------VEQRLDIHSRIQ-----------------------
------------------GIR-------------------WT------------------
-----PLPDIKSQEEYKQ-TVKQV------------------------V---T-------
-------HKVW-------------RA---------------------AKVLH-TYLLTVS
PA-M----------------MRDRKHH------F-RSH----------------RQC-CS
GK-----------------ELVDWLLGIS------------------------------H
----GLQ--------SRSQAVGIW---QVLVDEGALV-----------------------
--------------H------------------------VRQ-EPNFHDKDSQFYRFVKA
-------EL------HVDP--------------------------------LLT------
-----------------DKDT---------------------------------------
---------EEEV------QESLVLLSQLGPAAL-------------LTMILRKPPCQRT
EEELE-----VIFEELLHIKAVSHLS-NSVKKELA-SVLIFESHAKAGTVL---------
--FNQGDKG--TSWYIIW
KGSVNVITHGK-----GL-------------------------------VATLHEG-DDF
GQLAL-----------VNDAPRA---ATIVLREDNCHFLRVDKHDFNR------ILRD--
---V--------------

------------------------EANTVRLKEHGKVVLVLEK-------N---------

```
PQISAGNNHQP------------------AGASNQ------------K-YTVMSGTADKI
LEHLLET------------IKLDSCTED----SF-------------------AG---D-
FLLTHSI--FM---------------PTTQLCQALL-----HQF------N---------
-----------------------------VEPSE---VNEPEKATYSLHKRQNILHLVSL
WVALYGKLLEAE-PSAKSFLE----KLSEH--VSRDP--RLLAFWR---EN-LQDRRK--
TRT---LEVSSGNH--SPQ----IKIN-SFGN----------------------------
------L-----------FSSLE--------ED---------------------------
-----------------A------------------------------------------
---SSNT-----------------------------------------------------
------------------------------------------------------------
------------------------------------------------------------
------------------------------------------------------------
------------------------------------------------------------
----YTIRAGDK--VP-----------------------------YD-------------
IYRPDHSCITTMLPVNASVQEVIS--------SLATKNGYN------------RDQ--IV
VKVNSS----------------------G------ERVCLKHEDTGVFTSLGLNERIFIC
YNHE---L-NNVT-PLPEQL---------------------------GP------NTSSS
DI--LELISSKDLAHQMTDNDWNLFKSIHQVELIY-------HTFGKQRF-RNATTANLE
RFMRR---------FNEVQFWV-----ATEVCLCQEEERR------ALLLKKFIKLAAYL
---------------KEQKNLNSFFAVMF-GLSNTAVSRLSRTWQ---------------
-------KLPNKFRKLYSI-FERLM--------------------------DPSWNH---
-RSYRL----AVAK-------LSAPLI-PFLP----------------------------
LILKDLTFLHEGNR--ST-LD-----------------------------S-LVNFE-KM
-------RM---IAKTVQMFHSCRSQAY------------------------IPLSPLRP
RPVNILEDPSAARISACSEHSMTLR-------S--------------PATTWA-----YI
QHMKVIDNQKKL-----------------------------------------MELSRSL
-----------EPASST-------------------------------------------
------------------------------------------------------------
------------------------------------------------------------
------------------------------------------------------------
------------------------------------------------------------
------------------------------------------------------------
------------------------------------------------------------
------------------------------------------------------------
------------------------------------------------------------
------------------------------------------------------------
------------------------------------------------------------
---
>Rapgef4_[Acanthisitta_chloris]_677973646
------------------------------------------------------------
------------------------------------------------------------
------------------------------------------------------------
------------------------------------------------------------
-------MSGLLTPP----YGVMETGS----NNDRMPDKDS-------------------
------------------------------MSSSALCQVSK---------NSSK------
-----------------TPLI-------------------EP------------------
-----HIPHR--P---TK-TITQV-------------------P--------S-------
-------EKIL-------------RA---------------------GKILR-NTILSRA
PH-M----------------IRDRKYH------L-KTY----------------RQC-CV
GT-----------------ELVDWMMQQS------------------------------P
----CVH--------LRTQAVGMW---QVLLEEGVLN-----------------------
--------------H------------------------VDQ-EHHFQDK-YLFYRFLDD
-------EHEDAPLPTEEE-----------------------------------------
------------------KKEC--------------------------------------
```

---------DEEL------QDTMLLLSQIGPDAH-------------MRMILRKPPGQRT
VDDLE-----FIYEELLHIKALSHLS-TTVKRELA-GVLIFESHPKAGTVL---------
--FNQGEEG--TSWYIIL
KGSVNVVIYGK-----GV-------------------------------VCTLHEG-DDF
GKLAL-----------VNDAPRA---ASIVLREDNCHFLRVDKEDFNR------ILRD--
---V--------------

------------------------EANTVRLKEHDQDVLVLEK-------I------PAG
NR----VSNQG------------------NSQPQH------------K-YIVMSGTPEKI
LEHFLET------------MRLEATLNEATDSVLNDFIMMHCVFMPNSQLCPALMAQYP-
F---

>Rapgef4_[Acinonyx_jubatus]_961712463

---MAG-L
LAPPYG--VMETGS--N-N--------DRIPDKE--------------------------

------------------N---
---TPLIEPHVPLRP-------
---ANTITKVMG---C--------RA---------------------GKILR-NAILSRA
PH-M----------------IRDRKYH------L-KTY----------------RQC-CV
GT-----------------ELVDWMLQQT-------------------------------
---PCVH--------SRTQAVGMW---QVLVEDGVLN-----------------------
--------------H------------------------VDQ-EHHFQDKY-LFYRFLDD
-------EHED----AP-L---
--PT
EEEK--KECDEEL------QDTMLLLSQMGPDAH-------------MRMTLRKPPGQRT
VDDLE-----IIYEELIHIKALSHLS-TTVKRELA-GVLIFESHAKGGTVL---------
--FNQGEEG--TSWYIIL
KGSVNVVIYGK-----GV-------------------------------VCTLHEG-DDF
GKLAL-----------VNDAPRA---ASIVLREDNCHFLRVDKEDFNR------ILRD--
---V--------------

------------------------EANTVRLKEHDQDVLVLEK-----VPA-GNRASNQG
NS----QP-QQ------------------------------------K-YTVMSGTPEKI
LEHFLET------------IRLEP-ALN--------------EA-----TDSILN---D-
FVMMHCV--FM---------------PNTQLCPALV-----A------------------
--------H-----------Y-------HAQPSQ---GTEQEKTDYTLNNKRRVIRLVLQ
WAAVHGDVLQED-DVAMAFLE----EFYVS--VSDDA--RMIAALK---EQ-LPELEKIV
KQI---SED--AK---------APQKK---------------------------------
---HKV--------LLQQFNTGD-ERAQ-KRQ----------------------------

-----PVRGSDE--VL-----------------------------FK-------------
VYCMDHTYTTIRVPVAASVKEVIS--------AVADK-----------LGSG-EG--LII
VKMSSG----------------------GEKVV------LKPNDVSVFTTLTINGRLFAC
PREQ---F-DSLT-PLPEQ---------------------------EGP------TVGTM
GT--FELMSSKDLAYQMTIYDWELFNCVHELELIY-------HTFGRHNF-KK-TTANLD
LFLRR---------FNEIQFWV-----VTEICLCSQPSKR------VQLLKKFIKIAAH-
--------------CKEYKNLNSFFAIVM-GLSNVAVSRLALTWE---------------
-------KLPSKFKKFYAE-FESLM---------------------------DPSRNH---
-RAYRL----TVAK-------LEPPLI-PFMP----------------------------
LLIKDMTFTHEGNK--TF-ID-----------------------------N-LVNFE-KM
-------RM---IANTARTVRYCRS--Q-------------------------PFNPD--
-A------------------AQANKNH-----Q----------------DVRS-----YV
RQLNVIDNQRTL---SQMSHRL
-----------EPRR----------P----------------------------------

>Rapgef4_[Acropora_digitifera]_1005470793
-----------------------------MEA----------------------------
--DWIS---CLEK-----------RSAD-----RTEKDLDIIYSKLKVLEGFERFHSSVL
LKICQYGY--YQDLE--EGITLFRQGDIGCYWYAVLSGTVDVNLSE-NGKFEDAE-----
-------TICSLGPGKVFGESVLDDS-PRHATVV-TSEFCELLFVKKRDFREIWERE---
--KE--HLEHLISSP--S--
---------VETST----SYFE---------------------IQ---------------
------------------GKN---------------------------------------
-----------DKLFEGNNSQEHL-------------------SVIVCR-----------
------DEAFLA-------------A---------------------GRVLHYRMTLSMS
PK-L----------------ICDRIIE------N-RTE----------------ERC-FI
GS-----------------QAIDWLAKVS-------------------------------
---PLVD--------GRFHALNML---QALLEEDIII-----------------------
--------------N------------------------VSGSECEFKDTD-NLYRFSIN
E---W-------------
--PG
NEVY----AEEDL------DDALLMLTRSGPDAQ-------------LRLALRKRSCDRT
PDDLA-----FIYEELLHIKALHYLS-TTVKKELA-AVVVFESCDNMGTVL---------
--FKEGEEG--NSWYVII
KGIVNVIVHGK-----GV-------------------------------VCQLNEG-DDF
GKLAL-----------VNNALRT---ATIETAKDSCHFLKIGKSDFNR------IFQD--
---V--------------

------------------------ETNTVHLKEHGQDVLVLEKMN-----S----RILFG
QS-GCHASSQM--------------------------------------YSVMAGTPSKM
VEYLLET-------------RIDS------------------KC---SLDDHFLN---D-
FLVAFPI--FM---------------TTESLCYALF-----ANYS-----R---------
---------------TTF--F--DCGP---EFSSC---TIREHSDAI---KKRVVQVTQR
WCTLAGNLFLED-KSICIFIE----DLLQS--LEKDKLNDEYNILK-------TEMSLLH
ERF-SQEKNVDGSM--------GPQRDGLQ-----------------------S------
----------------RRMGLGDIAKLEVERKP---------------------------

------IRGNDQD--V-----------------------------FP-------------
VFYADHSYVTLALPYNVTAQTIVD--------SIEDHTIL--------------GEDCLL
CEVTST----------------------GELIP------FKADEICVTTGLSVNGKLFIA
PSEH---L-DSLT-PTPEQK---------------------------GPVC------SSL
VF--LETVGAKELAYFISQYDFALFNAVNVYEFIY-------HVFGKEKY--NQITANLD
MLLRR---------LNEVRFWI-----ETQVLLTQQLSKR------VYLLKTFIKLAAHC
---------------KELNNWNSFFAILM-ALNSVAVSRLTQTWE---------------
-------KVPRKFRNILEQ-FDSIL--------------------------EPTRNH---
-RVYRL----LVGKQ-------KPPIL-PFVP----------------------------
LLIKDMTFANEGNK--TY-LD-----------------------------D-LVNFE-KM
-------RM---IASSVRFFHYCRS--E-------------------------PFTADC-
-------------------PSLGRPSQ----------------------EIAA-----FV
RDWKVIDNERTL---TQLSHGL
QPKKAS--

>Rapgef4_[Acyrthosiphon_pisum]_641671152
---------M----LYRQGEI---------GSSWYAVL----------------------
------GGSLEARL-------------T-----HTTQ-----------------------
---TSSTNTDK-------
-------AVVSLDVGATFGESIVHDL-PRDMTVC-TKTTCELLRIHQNDFKKIWD-----
KHSN--LMKDLI--------------A-SNKLRNGMASGN-L--------SKCQSPP--P
VMTQA------------------R--RTITPDNPDPAE----------------------

--VITE--------------
-----------------NPSMPMARA---------------------GWVLR-TLLLSDQ
NT-V----------------LRDRKTGG-----G-RSV---------------VRRC-AS
GS-----------------ELIDWLMNLV-------------------------------
---STDH-----DSFSRHDVIGMW---QALLEEGVIS-----------------------
--------------H------------------------ATG-EHPFKDKC-LFYNFWQD
R---------E----G--
--ALNTPT
IQDV--AEAEEHL------DEALQELVHRGPDAH-------------LRLILRSPSSERT
PEDLE-----LIFEELSEQKALAHLP-NSVKRELA-SVVVFEAHPKAGHTL---------
--FRQGDDG--KAWYVIM
QGAVSVETYSK-----GI-------------------------------VESLYEG-EDF
GGLAL-----------IHNVPRS---ATITVKEDNTHLLRVDKDSYNK------IVRD--
---I--------------

------------------------EANTVRLKELGSDVLVLE--------K---------
------SPRLH----------------------K---------------YMIIAGTPQKI
LEHLLEH-------------RLST-----T-------NVAGSRN------DPCLD---D-
FLLTHIV--FM---------------PTRTLVSELN-----K------------------
--------YYHM-------------------------ENPAQDREYTINCKRKVVQFVYR
WVVTIRHPVFDD-VVSMSFLE----ELASD--VEADC--------QLLGGK-SQALQEEA
SLM---
---HHI--------MSRLRRYQD-ERLETSGLK---------------------------
----WKLPP---GGQPIC--------------------------------LFSAISN---
---PSKNNRPI---

------IRPNDD--II-----------------------------FR-------------
VYCADHTFCTLRLAVNATAETIKV--------GAYEK-----------CSLRHPVQDLRI
VEVKSN----------------------GERVP------FRDSEHSIPTTVALNSCIFLT
PKDH---M-DALT-CVPEQ---------------------------E-K------SEGAE
ISGDFDSLSTKELAYHMTLFDWELFWNVHEYELIY-------LTFGRHRF--QQITANLD

IFLRR---------FNEIQYWV-----ITEICTSQNISKR------TNILKKMIKLATY-
--------------CKEYYNFNAFFAILM-GLSDVAVSRLSTTWD---------------
-------KLPSKSRKQFTE-YETLI--------------------------DPSRNH---
-RAYRI----TVGK-------LPSPMI-PFMP----------------------------
LLIKDMKFTHDGNK--TH-VN-----------------------------G-LVNFE-KM
-------HM---LAQTMRTLRYCRA--R------HLVL-----------------DPP--
----------------------VSKNE-----A----------------DVKS-----YV
SCLRCVNNERTL---MSMSQKL
-----------EPRR----------T----------------------------------

>Rapgef4_[Ailuropoda_melanoleuca]_752414091
---M
VAAHA-AHSSSSAEWIA--C-LDKRPLE-----RSSEDVDIIFTRLKEVK----------

--------------------AFEKFH-PNLLHQIC--LCGYYENLEKGITL---------
--FRQGDIG--TNWYAVL
AGSLDVKVSETSSHQDAVT------------------------------ICTLGIG-TAF
GE-SI-----------LDNTPRH---ATIVTRE-SSELLRIEQKDFKA------LWEG--
------LTPLSSSFSMEWRNDSNPERKGVKLLGGN-------------------------

>Rapgef4_[Alligator_mississippiensis]_564252547

---MSG-L
LAPPYG--VMETGS--N-N--------DRMPDKE--------------------------
------------------NMS-------SNALCPVSKNCNKT------------------
-----PLIEPHIPLRPRK-TITQ------------------VPS----------------
-------EKIL-------------RA---------------------GKILR-NAILSRA
PH-M----------------IRDRKYH------L-KTY----------------RQC-CV
GT-----------------ELVDWMMQQS-------------------------------
---PCVH--------SRTQAVGMW---QVLLEEGVLN-----------------------
--------------H------------------------VDQ-EHHFQDKY-LFYRFLDD
-------EQED----AP-M---
--PT
EEEK--KECDEEL------QDTLLLLSQIGPDAH-------------MRMILRKPPGQRT
VDDLE-----IIYEELIHIKALSHLS-TTVKRELA-GVLIFESHPKAGTVL---------
--FNQGEEG--TSWYIIL
KGSVNVVIYGK-----GV-------------------------------VCTLHEG-DDF
GKLAL-----------VNDAPRA---ASIVLREDNCHFLRVDKEDFNR------ILRD--
---V--------------

------------------------EANTVRLKEHDQDVLVLEK-----IPA-GNRASNQG

NS----QP-QH------------------------------------K-YIVMSGTPEKI
LEHFLET------------MRLES-TVN--------------EA-----TDSVLN---D-
FVLMHCV--FM---------------PNSQLCPALM-----A------------------
--------H-----------Y-------HAQPSQ---GSEQEKLDYAINNKKRVIRLVLQ
WAALYGDLLQED-EAAVAFLE----EFYVS--VSDDT--RMIAALK---EQ-LPELEKVV
KQI---SEE--GK---------TPQKK---------------------------------
---HKV--------LLQLFNTTD-DRAQ-KRQ----------------------------

-----PIRGSDE--IL-----------------------------FK-------------
VYCIDHTYTTIRVPVLSSVKEVVS--------AVTDK-----------LGSG-EG--LII
VKMSSG----------------------GEKVV------LKPNDVSVFTTLTVNGRLFAC
PRDQ---F-DSLT-PLPEQ---------------------------EGP------STGTV
GT--FELMSSKDLAYQMTIYDWELFNCVHELELIY-------HTFGRHHF-KK-TTANLD
LFLRR---------FNEIQFWV-----VTEISLCSQLSKR------VQLLKKYIKIAAH-
--------------CKEYKNLNSFFAIIM-GLSNVAVSRLTLTWE---------------
-------KLPSKFKKIYAE-FESLM--------------------------DPSRNH---
-RAYRL----TVAK-------LDPPII-PFMP----------------------------
LLIKDMTFTHEGNK--TF-ID-----------------------------S-LVNFE-KM
-------RM---IANTIRTVKFCRS--Q-------------------------SFNPD--
-A------------------ALTNKNH-----Q----------------DVRS-----YV
RQLNVIDNQRTL---SQMSHRL
-----------EPRR----------A----------------------------------

>Rapgef4_[Amazona_aestiva]_944296700
----------------------------MVAA-------HT------SH--------SSS
SGEWIA---CLDK-----------RPLE-----RSSEDVDIIFTRLKEVKAFEKFHPNLL
QQICLCGY--YENLE--KGITLFRQGDIGMNWYAVLTGSLDVKVSD-TSNHQDA------
------VTICTLGIGTAFGESILDNT-PRHATIV-TREYSELL-----------------
----------------------------RIEQKD-------FKALW---EKYRQYMSG-L
LTPPYG--VMETGS--N-N--------DRMPDKD--------------------------
------------------SMS-------SGALCQVSKNCSKT------------------
-----PLIEPHIPHRSTK-TITQ------------------VPS----------------
-------EKIL-------------RA---------------------GKILR-NTILSRA
PH-M----------------IRDRKYH------L-KTY----------------RQC-CV
GT-----------------ELVDWMMQQS-------------------------------
---PCVH--------LRTQAVGMW---QVLLEEGVLN-----------------------
--------------H------------------------VDQ-EHHFQDKY-LFYRFLDD
-------EHED----AP-L---
--PT

EEEK--KECDEEL------QDTMLLLSQIGPDAH-------------MRMILRKPPGQRT
VDDLE-----FIYEELLHIKALSHLS-TTVKRELA-GVLIFESHPKAGTVL---------
--FNQGEEG--TSWYIIL
KGSVNVVIYGK-----GV-------------------------------VCTLHEG-DDF
GKLAL-----------VNDAPRA---ASIVLREDNCHFLRVDKEDFNR------ILRD--
---V--------------

------------------------EANTVRLKEHDQDVLVLEK-----IPA-GNRVSNQG
NS----QP-QH------------------------------------K-YIVMSGTPEKI
LEHFLET------------MRLEA-TLN--------------EA-----TE---------
-----------------------------QLKEHLK-----G------------------
--------M-----------T-------DFAPSQ---GTEQEKMDYALNNKRRVIRLVLQ
WAALYGDLLQED-EAAMAFLE---------------------------------------

---HKV--------LLQLFNTSD-DRAQ-KRQ----------------------------

-----PIRGSDE--VL-----------------------------FK-------------
VYCIDQTYTTIRVPVSSSVKEVIS--------AVADK-----------LGSG-EG--LII
VKMSSG----------------------GEKVV------LKPHDVSVFTTLSVNGRLFAC
PRDQ---F-DSLA-PLPEQ---------------------------EGP------SAGTV
GT--FELMSSKDLAHQMTIYDWELFNCVHEDPSRN-------H----RAY-RL-TVAKLD
-------------------------------------PPI------IPFMPLLIKVK---
------------------EDFGDLIAFML-GCSDRTVVSAAMGDI---------------
-------TL-----KSIVE-LYLLLPL---------------------------------

>Rapgef4_[Amphimedon_queenslandica]_761901406


```
------------------------------------------------------------
------------------------------------------------------------
------------------------------------------------------------
--------------------------------------------------MYTYKRC-CS
GQ-----------------SLVNWVIKQS-------------------------------
----AVN-------RSRQQVIAMW---QALLCDGIIE-----------------------
--------------H-----------------------TLNE-HNQFLDDD-KIYYRFVD
RPI-----PES-----AKLANSPGASGRFEY-----------------------RRTQSC
DSSI-------LESPIMSRSSTH-----------------NSISSGVEGGGGGGGGGSPT
SSPR------SSLSS--LLEDCFELIAQLGPEAL-------------IYATLIKSPDNRS
DDDIQ-----LIYEELLHVKAFGHLS-NAIKEELA-SVVQLENHPVAGKYL---------
--------------------------------------------FKEGDAG--TSWYIIL
KGSVNVLV-GKD-----V-------------------------------MCTLHEG-DEF
GKLAL-----------LNNAPRT---TSVQLREPNCVFLRVDRDDFTR------ILLS--
---------------------------------------------V--------------
------------------------------------------------------------
------------------------------------------------------------
------------------------EKNTVKIKEHGKEVLLLEKS------T---------
GG---------------------------------------------K-YLVVKGTPQKM
LEYLLTLDISPGG-------RDAAIDESFA------------------------C---D-
FFLTYPA--FI---------------SVSELCDGLI-----KCYDS----QAPPTPSPL-
--------ERPR----SM------STE--SLPAE-------ATEEQLLTRRKRVVWAVSI
WVSMAKAEVVKD-PTFQKLLQYLQKSLAKD-GLEEDQR-NLNMCLM---DT----IKRSS
YHE--GTKGGGSSGLSIPF----PMKK--------------------------P------
-------------------SVSE-----IKIELG--------------------------
-----------------I------------------------------------------
-------C-AV-------------------------------------------------
------------------------------------------------------------
------------------------------------------------------------
------------------------------------------------------------
---------------------------------------------------------P--
-----PCQPNDT--VN-----------------------------LR-------------
IHTIDKKCCRLHVKLSSTSRDILTE--------ACSKLGLD-------LSSH---E---L
CEIKST----------------------GEKII------FKETDLSIATEMTVNGRLYAL
PKDS---E-AAIS-PLPDQNQKPI-----------------------APFP---------
-----ESEGSREIAAHLTSYDWNLFSNIQQMELIY-------QVFGRHRF--SRITSNLD
VMIRR---------FNEVQYWT-----VTEICKESNLQKR------VKIIQKFIKIASHC
---------------KSFNNLNCFFAIVV-GLMNGAITRLKQTWE---------------
-------KVSVKLRRRYEQ-FEALM--------------------------DPSRNH---
-RVLRA----YQQK-------LQPPII-PFMP----------------------------
LIVKDAFFLQEGNE--TF-VD-----------------------------G-LVNFE-KM
-------RM---VASKVNDFSYYRKGSLANEI----------------------------
-------------------KMLSNKNS----------------------ELQR-----YI
RDFKVIDSQQVL-----------------------------------------MQMSHAI
ESSKRTQAT---PTS---------------------------------------------
------------------------------------------------------------
------------------------------------------------------------
------------------------------------------------------------
------------------------------------------------------------
------------------------------------------------------------
------------------------------------------------------------
------------------------------------------------------------
------------------------------------------------------------
------------------------------------------------------------
```


>Rapgef4_[Anas_platyrhynchos]_874471711

-------------------MRRE-------------------------------------

--RIV-----------------
----------------------------SSILQW-------GDTSV---QKYRQYISG-L
LTPPYG--VMETGS--N-N--------DRMPDKD--------------------------
------------------SMS-------GSSLCQVSKNCNKT------------------
-----PLIEPHIPHRPTK-TITQ------------------VPS----------------
-------EKIL-------------RA---------------------GKILR-NAILSRA
PH-M----------------IRDRKYH------L-KTY----------------RQC-CV
GT-----------------ELVDWMMQQS-------------------------------
---PCVH--------SRSQAVGMW---QVLLEEGVLN-----------------------
--------------H------------------------VDQ-EHYFQDKY-LFYRFLDD
-------ERED----AP-L---
--PT
EEEK--KECDEEL------QDTMLFLSQIGPDAH-------------MRMILRKPPGQRT
IDDLE-----IIYEELLHIKALSHLS-TTVKRELA-GVLIFESHPKAGTVL---------
--FNQGEEG--TSWYIIL
KGSVNVVIYGK-----GV-------------------------------VCTLHEG-DDF
GKLAL-----------VNDAPRA---ASIVLREDNCHFLRVDKEDFNR------ILRD--
---V--------------

------------------------EANTVRLKEHDQDVLVLEK-----IPA-GNRVSNQG
NS----QP-QH------------------------------------K-YIVMSGTPEKI
LEHFLET------------MRLEV-TLN--------------EA-----TDSALN---D-
FIMMHCV--FM---------------PNSQLCPALM-----A------------------
--------H-----------Y-------HAQPSQ---GTEQEKMDYALNNKRRVIRLVLQ
WAALYGDLLQED-EAAMAFLE----EFYVS--VSDDT--RMIAALK---EQ-LPELEKIV
KQV---SEE--PK---------APQKK---------------------------------
---HKV--------LLQLFNTSD-DRAQ-KRQ----------------------------

-----PIRGSDE--VL-----------------------------FK-------------
VYCIDQTYTTIRVPVSSSVKEVIS--------AVADK-----------LGSG-EG--LII
VKMSSG----------------------GEKVV------LKPHDVSVFTTLSVNGRLFAC
PRDQ---F-DSLA-PLPEQ---------------------------EGP------STGTV
GT--FELMSSKDLAHQMTIYDWELFNCVHELELIY-------HTFGRHNF-KK-TTANLD
LFLRR---------FNEIQFWV-----VTEICLCSQLSKR------VQLLKKYIKIAAH-
--------------CKEYKNLNSFFAIIM-GLSNVAVSRLSLTWE---------------
-------KLPSKFKKIYAE-FESLM--------------------------DPSRNH---
-RAYRL----TVAK-------LDPPII-PFMP----------------------------
LLIKDMTFTHEGNK--TL-TD-----------------------------N-LVNFE-KM
-------RM---IANTVRTVKFCRS--Q-------------------------SFNPD--
-A------------------ALTNKNH-----Q----------------DVRS-----YV
RQLNVIDNQRTL---SQMSHRL
-----------EPRR----------A----------------------------------

>Rapgef4_[Anolis_carolinensis]_637371027
---M
VAAHT-SHAASSSEWIA--C-LDKRPLE-----RSSEDLDIIFTRLKEVK----------

--------------------AFEKFH-PNLLQQIC--LCGYYENLEKGITL---------
--FRQGDIG--TNWYAVL
TGSLDVKVSDTSNHQDAVT------------------------------ICTLGIG-TAF
GE-SI-----------LDNTPRH---ATIVTRE-NSELLRIEQKDFKA------LWE---
------IPKLEQG----------PIPHGTSGARRH-------------------------

>Rapgef4_[Anser_cygnoides_domesticus]_902873580

---MSG-L
LTPPYG--VMETGS--N-N--------DRMPDKD--------------------------
------------------SMS-------GSSLCQVSKNCNKT------------------
-----PLIEPHIPHRPTK-TITQ------------------VPS----------------
-------EKIL-------------RA---------------------GKILR-NAILSRA
PH-M----------------IRDRKYH------L-KTY----------------RQC-CV
GT-----------------ELVDWMMQQS-------------------------------
---PCVH--------SRSQAVGMW---QVLLEEGVLN-----------------------
--------------H------------------------VDQ-EHYFQDKY-LFYRFLDD
-------ERED----AP-L---
--PT
EEEK--KECDEEL------QDTMLFLSQIGPDAH-------------MRMILRKPPGQRT
IDDLE-----IIYEELLHIKALSHLS-TTVKRELA-GVLIFESHPKAGTVL---------
--FNQGEEG--TSWYIIL
KGSVNVVIYGK-----GV-------------------------------VCTLHEG-DDF
GKLAL-----------VNDAPRA---ASIVLREDNCHFLRVDKEDFNR------ILRD--
---V--------------

------------------------EANTVRLKEHDQDVLVLEK-----IPA-GNRVSNQG
NS----QP-QH------------------------------------K-YIVMSGTPEKI
LEHFLET------------MRLEA-TLN--------------EA-----TDSALN---D-
FIMMHCV--FM---------------PNSQLCPALM-----A------------------
--------H-----------Y-------HAQPSQ---GTEQEKMDYALNNKRRVIRLVLQ
WAALYGDLLQED-EAALAFLE----EFYVS--VSDDT--RMIAALK---EQ-LPELEKIV
KQV---SEE--PK---------APQKK---------------------------------
---HKV--------LLQLFNTSD-DRAQ-KRQ----------------------------

-----PIRGSDE--VL-----------------------------FK-------------
VYCIDQTYTTIRVPVSSSVKEVIS--------AVADK-----------LGSG-EG--LII
VKMSSG----------------------GEKVV------LKPHDVSVFTTLSVNGRLFAC
PRDQ---F-DSLA-PLPEQ---------------------------EGP------STGTV
GT--FELMSSKDLAHQMTIYDWELFNCVHELELIY-------HTFGRHNF-KK-TTANLD
LFLRR---------FNEIQFWV-----VTEICLCSQLSKR------VQLLKKYIKIAAH-
--------------CKEYKNLNSFFAIIM-GLSNVAVSRLSLTWE---------------
-------KLPSKFKKIYAE-FESLM--------------------------DPSRNH---
-RAYRL----TVAK-------LDPPII-PFMP----------------------------
LLIKDMTFTHEGNK--TL-TD-----------------------------N-LVNFE-KM
-------RM---IANTVRTVKFCRS--Q-------------------------SFNPD--
-A------------------ALTNKNH-----Q----------------DVRS-----YV
RQLNVIDNQRTL---SQMSHRL
-----------EPRR----------A----------------------------------

>Rapgef4_[Aotus_nancymaae]_817294746
----------------------------MISLLMLDGNQYS------RH--------SEL
CNLWCGGEGCIQG------------PLE-----RSSEDVDIIFTRLKEVKAFEKFHPNLL
HQICLCGY--YENLE--KGITLFRQGDIGTNWYAVLAGSLDVKVSE-TSSHQDA------
------VTICTLGIGTAFGESILDNT-PRHATIV-TRESSELL-----------------
----------------------------RIEQKD-------FKALW---EKYRQYMAG-L
LAPPYG--VMETGS--N-N--------DRIPDKE--------------------------
------------------N---
---VPS----------------
-------EKIL-------------KA---------------------GKILR-NAILSRA
PH-M----------------IRDRKYH------L-KTY----------------RQC-CV
GT-----------------ELVDWMMQQT-------------------------------
---PCVH--------SRTQAVGMW---QVLLEDGVLN-----------------------
--------------H------------------------VDQ-EHHFQDKY-LFYRFLDD
-------EHED----AP-L---
--PT
EEEK--KECDEEL------QDTMLLLSQMGPDAH-------------MRMILRKPPGQRT
VDDLE-----IIYEELLHIKALSHLS-TTVKRELA-GVLIFESHAKGGTVL---------
--FNQGEEG--TSWYIIL
KGSVNVVIYGK-----GV-------------------------------VCTLHEG-DDF
GKLAL-----------VNDAPRA---ASIVLREDNCHFLRVDKEDFNR------ILRD--
---V--------------

------------------------EANTVRLKEHDQDVLVLEK-----VPA-GNRASNQG

NS----QP-QQ------------------------------------K-YTVMSGTPEKI
LEHFLET------------IRLEA-SLN--------------EA-----TDSVLN---D-
FIMMHCV--FM---------------PNTQLCPALV-----A------------------
--------H-----------Y-------HAQPSQ---GTEQEKMDYALNNKRRVIRLVLQ
WAAMYGDLLQED-DVSMAFLE----EFYVS--VSDDA--RMIATLK---EQ-LPELEKIV
KQI---SED--AK---------APQKK---------------------------------
---HKV--------LLQQFNTGD-ERAQ-KRQ----------------------------

-----PIRGSDE--VL-----------------------------FK-------------
VYCMDHTYTTIRVPVAASVKEVIS--------AVADK-----------LGSG-EG--LII
VKMSSG----------------------GEKVV------LKPNDVSVFTTLTINGRLFAC
PREQ---F-DSLT-PLPEQ---------------------------EGP------TVGTV
GT--FELMSSKDLAYQMTIYDWELFNCVHELELIY-------HTFGRHNF-KK-TTANLD
LFLRR---------FNEIQFWV-----VTEICLCSQLSKR------VQLLKKFIKIAAH-
--------------CKEYKNLNSFFAIVM-GLSNVAVSRLALTWE---------------
-------KLPSKFKKFYAE-FESLM--------------------------DPSRNH---
-RAYRL----TVAK-------LEPPLI-PFMP----------------------------
LLIKDMTFTHEGNK--TF-ID-----------------------------N-LVNFE-KM
-------HM---IANTARTVRYYRS--Q-------------------------PFNPD--
-A------------------AQANKNH-----Q----------------DVRS-----YV
RQLNVIDNQRTL---SQMSHRL
-----------EPRR----------P----------------------------------

>Rapgef4_[Apaloderma_vittatum]_699612538

---MSG-L
LTPPYG--VMETGS--N-N--------DRMPDKD--------------------------
------------------N----------------------T------------------
-----PLIEPHIPHRPTK-TIAQ------------------VPS----------------
-------EKIL-------------RA---------------------GKILR-NTILSRA
PH-M----------------IRDRKYH------L-KTY----------------RQC-CV
GT-----------------ELVDWMMQQS-------------------------------
---PCVH--------SRTQAVGMW---QVLLEEGVLN-----------------------
--------------H------------------------VDQ-EHHFQDKY-LFYRFLDD
-------ERED----AP-L---
--PT

EEEK--KECDEEL------QDTMLLLSQIGPDAH-------------MRMILRKPPGQRT
VDDLE-----FIYEELLHIKALSHLS-TTVKRELA-GVLIFESHPKAGTVL---------
--FNQGEEG--TSWYIIL
KGSVNVVIYGK-----GV-------------------------------VCTLHEG-DDF
GKLAL-----------VNDAPRA---ASIVLREDNCHFLRVDKEDFNR------ILRD--
---V--------------

------------------------EANTVRLKEHDQDVLVLEK-----VPA-GNRVSNQG
NS----QP-QH------------------------------------K-YIVMSGTPEKI
LEHFLET------------MRLEA-TLN--------------EA-----TDSVLN---D-
FIMMHCV--FM---------------PNSQLCPALM-----A------------------
--------H-----------Y-------HAQPSQ---GTEQEKMDYALNNKRRVIRLVLQ
WAALYGDLLQED-EAAMAFLE----EFYVS--VSDDT--RMIAALK---EQ-LPELEKIV
KQV---SEE--PK---------APQKK---------------------------------
---HKV--------LLQLFNTSD-DRAQ-KRQ----------------------------

-----PIRGSDE--VL-----------------------------FK-------------
VYCIDQTYTTIRVPVSSSVKEVIS--------AVADK-----------LGSG-ES--LII
VKMSSG----------------------GEKVV------LKPHDVSVFTTLSVNGRLFVC
PRDQ---F-DSLV-PLPEQ---------------------------EGP------STGTI
GT--FELMSSKDLAHQMTIYDWELFNCVHELELIY-------HTFGRHNF-KK-TTANLD
LFLRR---------FNEIQFWV-----VTEICLCSQLSKR------VQLLKKYIKIAAH-
--------------CKEYKNLNSFFAIIM-GLSNVAVSRLSLTWE---------------
-------KLPSKFKKIYAE-FESLM--------------------------DPSRNH---
-RAYRL----TVAK-------LDPPII-PFMP----------------------------
LLIKDMTFTHEGNK--TF-TD-----------------------------N-LVNFE-KM
-------RM---IANTVRTVKFCRS--Q-------------------------SFNPD--
-A------------------ALTNKNH-----Q----------------DVRS-----YV
RQLNVIDNQRTL---SQMSHRL
-----------EPRR----------A----------------------------------

>Rapgef4_[Aptenodytes_forsteri]_686601170
----------------------------MVAA-------HT------SH--------SSS
SGEWIA---CLDK-----------RPLE-----RSSEDVDIIFTRLKEVKAFEKFHPNLL
QQICLCGY--YENLE--KGITLFRQGDIGTNWYAVLTGSLDVKVSD-TSNHQDA------
------VTICTLGIGTAFGESILDNT-PRHATIV-TREYSELL-----------------
----------------------------RIEQKD-------FKALW---EKYRQYMSG-L
LTPPYG--VMEAGS--N-N--------DRLPDKD--------------------------

```
------------------N----------------------T------------------
-----PLIEPHIPHRPTK-TITQ------------------VPS----------------
-------EKIL-------------RA---------------------GKILR-NTILSRA
PH-M----------------IRDRKYH------L-KTY----------------RQC-CV
GT-----------------ELVDWMMQQS-------------------------------
---TCVH--------SRTQAVGMW---QVLLEEGVLN-----------------------
--------------H------------------------VDQ-EHHFQDKY-LFYRFLDD
-------ERED----AP-L-----------------------------------------
----------------------------------------------------------PT
EEEK--KECDEEL------QDTMLLLSQIGPDAH-------------MRMILRKPPGQRT
VDDLE-----IIYEELLHIKALSHLS-TTVKRELA-GVLIFESHPKAGTVL---------
--------------------------------------------FNQGEEG--TSWYIIL
KGSVNVVIYGK-----GV-------------------------------VCTLHEG-DDF
GKLAL-----------VNDAPRA---ASIVLREDNCHFLRVDKEDFNR------ILRD--
---------------------------------------------V--------------
------------------------------------------------------------
------------------------------------------------------------
------------------------EANTVRLKEHDQDVLVLEK-----VPA-GNRVSNQG
NS----QP-QH------------------------------------K-YIVMSGTPEKI
LEHFLET------------MRPEA-TLN--------------EA-----TDSVLN---D-
FIMMHCV--FM---------------PNSQLCPALM-----A------------------
--------H-----------Y-------HAQPSQ---GTEQEKMDYALNNKRRVIRLVLQ
WAALYGDLLQED-EAAMAFLE----EFYVS--VSDDT--RMIAALK---EQ-LPELEKIV
KQV---SEE--SK---------APQKK---------------------------------
---HKV--------LLQLFNTSD-DRAQ-KRQ----------------------------
------------------------------------------------------------
------------------------------------------------------------
------------------------------------------------------------
------------------------------------------------------------
------------------------------------------------------------
------------------------------------------------------------
-----PIRGSDE--VL-----------------------------FK-------------
VYCIDQTYTTIRVPVSSSVKEVIS--------AVADK-----------LGSG-EG--LII
VKMSSG----------------------GEKVV------LKPHDVSVFTTLSVNGRLFAC
PRDQ---F-DSLA-PLPEQ---------------------------EGP------STGTV
GT--FELMCSKDLAHQMTIYDWELFNCVHELELIY-------HTFGRHNF-KK-TTANLD
LFLRR---------FNEIQFWV-----VTEICLCSQLSKR------VQLLKKYIKIAAH-
--------------CKEYKNLNSFFAIIM-GLSNVAVSRLSLTWE---------------
-------KLPSKFKKIYAE-FESLM--------------------------DPSRNH---
-RAYRL----TVAK-------LDPPII-PFMP----------------------------
LLIKDMTFTHEGNK--TF-TD-----------------------------N-LVNFE-KM
-------RM---IANTVRTVKFCRS--Q-------------------------SFNPD--
-A------------------ALTNKNH-----Q----------------DVRS-----YV
RQLNVIDNQRTL-----------------------------------------SHMSHRL
-----------ELRR----------A----------------------------------
------------------------------------------------------------
------------------------------------------------------------
------------------------------------------------------------
------------------------------------------------------------
------------------------------------------------------------
------------------------------------------------------------
------------------------------------------------------------
------------------------------------------------------------
------------------------------------------------------------
```


>Rapgef4_[Apteryx_australis_mantelli]_926533754

---M----------------IRDRKYH------L-KTY----------------RQC-CV
GT-----------------ELVDWMMQQS-------------------------------
---PCVH--------SRTQAIGMW---QVLLEEGVLN-----------------------
--------------H------------------------VDQ-EHHFQDKY-LFYRFLDD
-------EHED----AP-L---
--PT
EEEK--KECDEEL------QDTMLLLSQIGPDAH-------------MRMILRKPPGQRT
VDDLE-----IIYEELLHIKALSHLS-TTVKRELA-GVLIFESHPKAGTVL---------
--FNQGEEG--TSWYIIL
KGSVNVVIYGK-----GV-------------------------------VCTLHEG-DDF
GKLAL-----------VNDAPRA---ASIVLREDNCHFLRVDKEDFNR------ILRD--
---V--------------

------------------------EANTVRLKEHDQDVLVLEK-----IPA-GNRVSNQG
NS----QP-QH------------------------------------K-YIVMSGTPEKI
LEHFLET------------MRLEA-TLN--------------EA-----TDSVLN---D-
FVMMHCV--FM---------------PNSQLCPALM-----A------------------
--------H-----------Y-------HAQPSQ---GTEQEKMDYALNNKRRVIRLVLQ
WAALYGDLLHED-EAAMAFLE----EFYVS--VSDDT--RMIAALK---EQ-LLELEKIV
KQV---SEE--PK---------APQKK---------------------------------
---HKV--------LLQLFNTSD-DRAQ-KRQ----------------------------

-----PIRGSDE--VL-----------------------------FK-------------
VYCIDQTYTTIRVPVSSSVKEVIG--------AVADK-----------LGSG-EG--LTL
VKMSSG----------------------GEKVV------LKPHDVSVFTTLSVNGRLFAC
PRDQ---F-DSLA-PLPEQ---------------------------EGP------SAGTV
GT--FELMSSKDLAYQMTIYDWEFFNCVHELELIY-------HTFGRHNF-KK-TTANLD
LFLRR---------FNEIQFWV-----VTEICLCSQLSKR------VQLLKKYIKIAAH-
--------------CKEYKNLNSFFAIIM-GLSNVAVSRLSLTWE---------------
-------KLPSKFKKIYAE-FESLM--------------------------DPSRNH---
-RAYRL----TVTK-------LDPPII-PFMP----------------------------
LLIKDMTFTHEGNK--TL-TD-----------------------------N-LVNFE-KM
-------RM---IANTVRTVKFCRS--Q-------------------------SFNPD--
-A------------------ALANKNH-----Q----------------DVRS-----YV
RQLNVIDNQRTL---SQMSHRL
-----------EPRR----------A----------------------------------

>Rapgef4_[Aquila_chrysaetos_canadensis]_768339852

---MSG-L
LTPPYG--VMETGS--N-N--------DRMPDKD--------------------------
------------------SMS-------SSALCQVSKNCNKT------------------
-----PLIEPHIPHRPTK-TITQ------------------VPS----------------
-------EKIL-------------RA---------------------GKILR-NTILSRA
PH-M----------------IRDRKYH------L-KTY----------------RQC-CV
GT-----------------ELVDWMMQQS-------------------------------
---PCVH--------SRTQAVGMW---QVLLEEGVLN-----------------------
--------------H------------------------VDQ-EHHFQDKY-LFYRFLDD
-------ECED----AP-L---
--PT
EEEK--KECDEEL------QDTMLLLSQIGPDAH-------------MRMILRKPPGQRT
VDDLE-----FIYEELLHIKALSHLS-TTVKRELA-GVLIFESHPKAGTVL---------
--FNQGEEG--TSWYIIL
KGSVNVVIYGK-----GV-------------------------------VCTLHEG-DDF
GKLAL-----------VNDAPRA---ASIVLREDNCHFLRVDKEDFNR------ILRD--
---V--------------

------------------------EANTVRLKEHDQDVLVLEK-----IPA-GNRVSNQG
NS----QP-QH------------------------------------K-YIVMSGTPEKI
LEHFLET------------MRLEA-TLN--------------EA-----TDSVLN---D-
FIMMHCV--FM---------------PNSQLCPALM-----A------------------
--------H-----------Y-------HAQPSQ---GTEQEKMDYALNNKRRVIRLVLQ
WAALYGDLLQED-EAAMAFLE----EFYVS--VSDDT--RMIAALK---EQ-LPELEKIV
KQV---SEE--PK---------AQQKK---------------------------------
---HKV--------LLQLFNTSD-DRTQ-KRQ----------------------------

-----PIRGSDE--VL-----------------------------FK-------------
VYCIDQTYTTIRVPVSSSVKEVIS--------AVADK-----------LGSG-EG--LII
VKMSSG----------------------GEKVV------LKPHDVSVFTTLSVNGRLFAC
PRDQ---F-DSLA-PLPEQ---------------------------EGP------STGTV
GT--FELMSSKDLAHQMTIYDWELFNCVHELELIY-------HTFGRHNF-KK-TTANLD

```
LFLRR---------FNEIQFWV-----VTEICLCSQLSKR------VQLLKKYIKIAAH-
--------------CKEYKNLNSFFAIIM-GLSNVAVSRLSLTWE---------------
-------KLPSKFKKIYAE-FESLM--------------------------DPSRNH---
-RAYRL----TVAK-------LDPPII-PFMP----------------------------
LLIKDMTFTHEGNK--TF-TD-----------------------------N-LVNFE-KM
-------RM---IANTVRTVKFCRS--Q-------------------------SFNPD--
-A------------------ALTNKNH-----Q----------------DVRS-----YV
RQLNVIDNQRTL-----------------------------------------SQMSHRL
-----------EPRR----------A----------------------------------
------------------------------------------------------------
------------------------------------------------------------
------------------------------------------------------------
------------------------------------------------------------
------------------------------------------------------------
------------------------------------------------------------
------------------------------------------------------------
------------------------------------------------------------
------------------------------------------------------------
------------------------------------------------------------
---
>Rapgef4_[Astyanax_mexicanus]_992223431
------------------------------------------------------------
------------------------------------------------------------
------------------------------------------------------------
------------------------------------------------------------
-----------------------------------MEDEESCLIAWSKKRTEQRRSAE-A
ATRD----------------------SITTMASAGICSPDPLVVSS--LLAPGR------
-----------------TASD-------------------QT------------------
-----PLPEALDT---QD-TMKQF-------------------L--------S-------
-------DRIL-------------KA---------------------ARVVF-SALMERN
PG-L----------------IRDRKHH------L-KTY----------------RQC-CS
GK-----------------ELVDWLIKLN------------------------------D
----CFQ--------SRSQAAGMW---QVLVDEGILV-----------------------
--------------H------------------------MKQ-ELNFHDKDTQFYRFMET
-------EFDLNPSTNEKD-----------------------------------------
------------------SKE---------------------------------------
----------DEL------QEGLALLVQMGPDAL-------------LNMILRK------
----------CVRR----------------------------------------------
------------------------------------------------------------
------------------------------------------------------------
------------------------------------------------------------
------------------------------------------------------------
------------------------------------------------------------
------------------------------------------------------------
------------------------------------------------------------
------------------------------------------------------------
------------------------------------------------------------
------------------------------------------------------------
------------------------------------------------------------
------------------------------------------------------------
------------------------------------------------------------
------------------------------------------------------------
------------------------------------------------------------
------------------------------------------------------------
```


>Rapgef4_[Bactrocera_cucurbitae]_751444012
-----------------------------MAMEWITAM----------------------
----------------------DKRPCD-----RNLRDAELISCRLRRVEPLCRLPSSAL
QQLAMCGF--YEDLE--KGVTLFRAGEQGRFWYAVLGGSLEVRYHA-SDTDAK------A
P-----VTLCNLSVGATFGESILHEL-PRDSTVV-TKTTCELLRVEQQDFRLIWE-----
KNKE--LMNDII--------------T-TCKLKNGFGSGV---SP-----VA-SS-----
---PT------------------K--RPLSPDHPNPAL----------------------

--PISE--------------
-----------------YPSPTMNRM---------------------GWALR-TLLIADC
SS-C----------------LKDRKVA------G-K-F---------------IRKC-AP
GT-----------------ELVDWLLNLS-------------------------------
---SIVH--------TRAQAAGMW---QALLEEGVLS-----------------------
--------------H------------------------VNK-EQPFKDKC-FLYRFRVD
E---------D----G--
---TASSYST
TEDI--NAANEHV------RESLSILFQRGPDAI-------------LRMILRKPSHERT
PEELE-----LIFEELLHITALSHLS-TSIKRELS-SIIVFESHAKAGTIL---------
--FNQGDEG--RSWYILL
KGSVDVVIHGK-----GT-------------------------------VATLKNG-DDF
GKLAL-----------INDAPRA---ATIVLKENDCHLLRVDKEHFNR------ILRD--
---V--------------

------------------------EANTLRLQEHGKDVLVLER-----VAK---------

------QRGQ----HS---------------AFK---------------YTVMSGTPSKM
LEHLLET-------------RL---G---N-------QVSG--------LDPFLD---D-
FLLTHIV--FM---------------PVVQLVDELA-----N------------------
--------YF---HCDS---------N-----NG---SQTPEDREYIINFKKRVVQFMQK
WVTAVRHAAFDE-PSVCDFIE----DLAAE--IELD-----------------QELSEET
SII---
---HNV--------LTQMTRYQE-DRNQNTGQK---------------------------
----WKLPP---NGQPIC--------------------------------LFSGNTT---
---PSKT---I---

------IRPDDD--II-----------------------------FR-------------
VYCADHTYCTLRFPLHTTAEIIKA--------CAADK-----------LQLNRGPEDLVL
VEVKSN----------------------GERSV------YKDNDVSVPTGLSLNGRLFVS
VKDH---L-DALT-SLPEQ---------------------------EGP------IEGID
I--DLEVMSTKEIAYQMTLFDWDLFWAVHEYELLY-------QTFGRHHF--GKITANLD
VFLRR---------FNELQFWI-----VTEIVSTSSMCKR------VGLLRKFVKLAAY-
--------------CKEYQNLNAFFAIIM-GLSNMAVSRLTQTWE---------------
-------KVPSKFRKLFQE-FEALI--------------------------DPSRNH---
-RAYRV----YVGK-------LQPPVI-PFMP----------------------------
LLLKDMTFAHEGNK--TS-LE-----------------------------G-LVNFE-KM
-------HM---MAQTMRTIRFCRS--R------SLGL-----------------EPP--
----------------------SPKSE-----G----------------EVRS-----YI
SCLRVIDNQRVL---TAMSQKI
-----------EPIR----------KA---------------------------------

>Rapgef4_[Bactrocera_dorsalis]_751799864

----------------------MW---QALLEEGVLS-----------------------
--------------H------------------------VNK-EQPFKDKC-FLYRFRVD
E---------D----G--
---SAGSYST

```
TEDT--NAANEYV------RESLSTLFQRGPDAI-------------LRMILRKPSHERT
PEELE-----LIFEELLHITALSHLS-TSIKRELS-SIIVFESHAQAGTIL---------
--------------------------------------------FNQGDEG--RSWYILL
KGSVDVVIHGK-----GT-------------------------------VATLKNG-DDF
GKLAL-----------INDAPRA---ATIVLKENNCHLLRVDKEHFNR------ILRD--
---------------------------------------------V--------------
------------------------------------------------------------
------------------------------------------------------------
------------------------EANTLRLQEHGKDVLVLER-----VAK---------
------QRGQ----HS---------------AFK---------------YTVMSGTPSKM
LEHLLET-------------RL---G---N-------QVSG--------LDPFLD---D-
FLLTHIV--FM---------------PVVQLVDELA-----N------------------
--------YF---HCDS---------N-----NG---SQTPEDREYIINFKKRVVQFMQK
WVTAVRHAAFDE-PSVCDFIE----DLATE--IEAD-----------------QELSEET
SII---------------------------------------------------------
---HNV--------LTQMTRYQE-DRNQNTGQK---------------------------
----WKLPP---NGQPIC--------------------------------LFSGNAT---
---PSKT---I-------------------------------------------------
------------------------------------------------------------
------------------------------------------------------------
------------------------------------------------------------
------------------------------------------------------------
------IRPDDD--II-----------------------------FR-------------
VYCADHTYCTLRFPLHTTAGIIKA--------CAADK-----------LQLNRGPDDLVL
IEVKSN----------------------GERSV------YKDNDVSIPTSLSLNGRLFVS
VKDH---L-DALT-PLPEQ---------------------------EGP------TEGID
I--DLEILSTKEIAYQMTLFDWDLFWAVHEYELLY-------QTFGRHHF--GKITANLD
VFLRR---------FNELQFWI-----ATEIVTTSSMCKR------VGLLRKFIKLAAY-
--------------CKEYQNLNAFFAITM-GLSNMAVLRLTQTWE---------------
-------KIPSKFRKLFQE-FEALI--------------------------DPSRNH---
-RAYRV----YVGK-------LQPPVI-PFIP----------------------------
LLLKDMTFAHEGNK--TS-LE-----------------------------G-LVNFE-KM
-------HM---MAQTMRTIRFCRS--R------SLGL-----------------EPP--
----------------------SPKSE-----G----------------EVRS-----YI
SCLRVIDNQRVL-----------------------------------------TAMSQKI
-----------EPIR----------KV---------------------------------
------------------------------------------------------------
------------------------------------------------------------
------------------------------------------------------------
------------------------------------------------------------
------------------------------------------------------------
------------------------------------------------------------
------------------------------------------------------------
------------------------------------------------------------
------------------------------------------------------------
------------------------------------------------------------
---
>Rapgef4_[Balaenoptera_acutorostrata_scammoni]_594670782
------------------------------------------------------------
------------------------------------------------------------
------------------------------------------------------------
------------------------------------------------------------
-------------------------------------------------------MAG-L
LAPPYG--VMETGS--N-N--------DRIPDKE--------------------------
```

```
------------------N----------------------T------------------
-----PLIEPHVPLRPAN-TITK------------------VPS----------------
-------EKIL-------------RA---------------------GKILR-NAILSRA
PH-M----------------IRDRKYH------L-KTY----------------RQC-CV
GT-----------------ELVDWMLQQT-------------------------------
---PCVH--------SRVQAVGMW---QVLVEDGVLN-----------------------
--------------H------------------------VDQ-EHHFQDKY-LFYRFLDD
-------EHED----AP-L-----------------------------------------
----------------------------------------------------------PT
EEEK--KECDEEL------QDTMLLLSQMGPDAH-------------MRMILRKPPGQRT
VDDLE-----IIYEELLHIKALSHLS-TTVKRELA-GVLIFESHAKGGTVL---------
--------------------------------------------FNQGEEG--TSWYIIL
KGSVNVVIYGK-----GV-------------------------------VCTLHEG-DDF
GKLAL-----------VNDAPRA---ASIVLREDNCHFLRVDKEDFNR------ILRD--
---------------------------------------------V--------------
------------------------------------------------------------
------------------------------------------------------------
------------------------EANTVRLKEHDQDVLVLEK-----VPA-GNRASNQG
NS----QP-QQ------------------------------------K-YTVMSGTPEKI
LEHFLET------------IRLEP-ALN--------------EA-----TDSVLN---D-
FVMMHCV--FM---------------PNTQLCPALV-----A------------------
--------H-----------Y-------HAQPSQ---GTEQEKMDYALNNKRRVIRLVLQ
WAAVYGDVLQED-DVAVAFLE----EFYVS--VSDDA--RMIAALK---EQ-LPELEKIV
KQI---SED--AK---------APQKK---------------------------------
---HKV--------LLQQFNTGD-ERAQ-KRQ----------------------------
------------------------------------------------------------
------------------------------------------------------------
------------------------------------------------------------
------------------------------------------------------------
------------------------------------------------------------
------------------------------------------------------------
-----PIRGSDE--VL-----------------------------FK-------------
VYCMDHTYTTIRVPVAASVKEVLS--------AVADK-----------LGSG-EG--LII
IKMSSG----------------------GEKVV------LKPNDVSVFTTLTINGRLFAC
PREK---F-DSLT-PLPEQ---------------------------EGP------TVGTM
GT--FELMSSKDLAYQMTVYDWDLFSCVHELELIY-------HTFGRHHF-KK-TTANLD
LFLRR---------FNEIQFWV-----VTEICLCSQPSKR------VQLLKKFIKIAAH-
--------------CKEYKNLNSFFAIVM-GLSNVAVSRLALTWE---------------
-------KLPSKFKKFYAE-FESLM--------------------------DPSRNH---
-RAYRL----TVAK-------LDPPLI-PFMP----------------------------
LLIKDMTFTHEGNK--TF-ID-----------------------------N-LVNFE-KM
-------RM---IANTARTVRYCRS--Q-------------------------PFNPD--
-A------------------AQANKNH-----Q----------------DVRS-----YV
RQLNVIDNQRTL-----------------------------------------SQMSHRL
-----------EPRR----------P----------------------------------
------------------------------------------------------------
------------------------------------------------------------
------------------------------------------------------------
------------------------------------------------------------
------------------------------------------------------------
------------------------------------------------------------
------------------------------------------------------------
------------------------------------------------------------
------------------------------------------------------------
```


>Rapgef4_[Balearica_regulorum_gibbericeps]_723568217
----------------------------MGAR-------PV------SEVWLLQQLLKLF
LGLYPA---DVMEIQQQLPHKDPERPLE-----RSSEDVDIIFTRLKEVKAFEKFHPNLL
QQICLCGY--YENLE--KGITLFRQGDIGTNWYAVLTGSLDVKVSD-TSNHQDA------
------VTICTLGIGTAFGESILDNT-PRHATIV-TREYSELL-----------------
----------------------------RIEQKD-------FKALW---EKYRQYMSG-L
LTPPYG--VMETGS--N-N--------DRMPDKD--------------------------
------------------N----------------------T------------------
-----PLIEPHIPHRPTK-TITQ------------------VPS----------------
-------EKIL-------------RA---------------------GKILR-NTILSRA
PH-M----------------IRDRKYH------L-KTY----------------RQC-CV
GT-----------------ELVDWMMQQS-------------------------------
---PCVH--------SRTQAVGMW---QVLLEEGVLN-----------------------
--------------H------------------------VDQ-EHHFQDKY-LFYRFLDD
-------ERED----AP-L---
--PT
EEEK--KECDEEL------QDTVLLLSQIGPDAH-------------MRMILRKPPGQRT
VDDLE-----IIYEELLHIKALSHLS-TTVKRELA-GVLIFESHPKAGTVL---------
--FNQGEEG--TSWYIIL
KGSVNVVIYGK-----GV-------------------------------VCTLHEG-DDF
GKLAL-----------VNDAPRA---ASIVLREDNCHFLRVDKEDFNR------ILRD--
---V--------------

------------------------EANTVRLKEHDQDVLVLEK-----IPA-GNRVSNQG
NS----QP-QH------------------------------------K-YIVMSGTPEKI
LEHFLET------------MRLEA-TLN--------------EA-----TDSVLN---D-
FIMMHCV--FM---------------PNSQLCPALM-----A------------------
--------HY------------------HAQPSQ---GTEQEKMDYALNNKRRVIRLVLQ
WAALYGDLLQED-EAAMAFLE----EFYVS--VSDDT--RMIAALK---EQ-LPELEKIV
KQV---SEE--PK---------APQKK---------------------------------
---HKV--------LLQLFNASD-DRAQ-KRQ----------------------------

-----PIRGTDE--IL-----------------------------FK-------------
VYCIDQTYTTIRVPVSSSVKEVIS--------AVADK-----------LGSG-EG--LII
VKMSSG----------------------GEKVV------LKPHDVSVFTTLSVNGRLFAC
PRDQ---F-DSLA-PLPEQ---------------------------EGP------STGTV
GT--FELMSSKDLAHQMTIYDWELFNCVHELELIY-------HIFGRHNF-KK-TTANLD
LFLRR---------FNEIQFWV-----VTEICLCSQLSKR------VQLLKKYIKIAAH-
--------------CKEYKNLNSFFAIIM-GLSNVAVSRLSLTWE---------------
-------KLPSKFKKIYAE-FESLM--------------------------DPSRNH---
-RAYRL----TVAK-------LDPPII-PFMP----------------------------
LLIKDMTFTHEGNK--TF-TD-----------------------------N-LVNFE-KM

>Rapgef4_[Bison_bison_bison]_742139618
----------------------------M-------------------------------

--DV--------IMAV-Y
PA----------------------------PSSF--------------------------
------------------R----------------------T------------------
-----PLIEPHVPLRPAN-TITK------------------VPS----------------
-------EKIL-------------RA---------------------GKILR-NAILSRA
PH-M----------------IRDRKYH------L-KTY----------------RQC-CV
GT-----------------ELVDWMLQQT-------------------------------
---PCVH--------SRTQAVGMW---QVLVEDSVLN-----------------------
--------------H------------------------VDQ-EHHFQDKY-LFYRFLDD
-------EHED----AP-L---
--PT
EEEK--KECDEEL------QDTMLLLSQMGPDAH-------------MRMVLRKPPGQRT
MDDLE-----IIYEELLHIKALSHLS-TTVKRELA-AVLIFESHAKGGTVL---------
--FNQGEEG--TSWYIIL
KGSVNVVIYGK-----GV-------------------------------VCTLHEG-DDF
GKLAL-----------VNDAPRA---ASIVLREDNCHFLRVDKEDFNR------ILRD--
---V--------------

------------------------EANTVRLKEHDQDVLVLEK-----VPA-GNRASNQG
NS----QP-QQ------------------------------------K-YTVMSGTPEKI
LEHFLET------------IRLEP-ALN--------------EA-----TDSVLN---D-
FVMMHCV--FM---------------PNTQLCPALV-----A------------------
--------H-----------Y-------HAQPSQ---GTEQEKMDYALNNKRRVIRLVLQ
WAAMYGDILQED-DVAMAFLE----EFYVS--VSDDA--RMLAALK---EQ-LPELEKIV
KQI---SED--AK---------APQKK---------------------------------
---HKV--------LLQQFNTGD-ERAQ-KRQ----------------------------

-----PIRGSDE--VL-----------------------------FK-------------
VYCVDHTYTTIRVPVAASVKEVLS--------AVADK-----------LGSG-EG--LII
VKMSSG----------------------GEKVV------LKPNDVSVFTTLTINGRLFAC
PREQ---F-DSLT-PLPEQ---------------------------EGP------TVGTM
GT--FELMSSKDLAYQMTIYDWELFNCVHELELIY-------HTFGRHHF-KK-TTANLD

LFLRR---------FNEIQFWV-----VTEICLCSQPSKR------VQLLKKFIKIAAH-
--------------CKEYKNLNSFFAIVM-GLSNVAVSRLALTWE---------------
-------KLPSKFKKFYAE-FESLM--------------------------DPSRNH---
-RAYRL----TVAK-------LDPPLI-PFMP----------------------------
LLIKDMTFTHEGNK--TF-ID-----------------------------N-LVNFE-KM
-------RM---IANTARTVRYCRS--Q-------------------------PFNPD--
-A------------------AQANKNH-----Q----------------DVRS-----YV
RQLNVIDNQRTL---SQMSHRL
-----------EPRR----------P----------------------------------

>Rapgef4_[Bos_mutus]_555950853

---MAG-L
LAPPYG--VMETGS--N-N--------DRIPDKE--------------------------
------------------N---
---VPS----------------
-------EKIL-------------RA---------------------GKILR-NAILSRA
PH-M----------------IRDRKYH------L-KTY----------------RQC-CV
GT-----------------ELVDWMLQQT-------------------------------
---PCVH--------SRTQAVGMW---QVLVEDSVLN-----------------------
--------------H------------------------VDQ-EHHFQDKY-LFYRFLDD
-------EHED----AP-L---
--PT
EEEK--KECDEEL------QDTMLLLSQMGPDAH-------------MRMVLRKPPGQRT
MDDLE-----IIYEELLHIKALSHLS-TTVKRELA-AVLIFESHAKGGTVL---------
--FNQGEEG--TSWYIIL
KGSVNVVIYGK-----GV-------------------------------VCTLHEG-DDF
GKLAL-----------VNDAPRA---ASIVLREDNCHFLRVDKEDFNR------ILRD--
---V--------------

------------------------EANTVRLKEHDQDVLVLEK-----VPA-GNRASNQG
NS----QP-QQ------------------------------------K-YTVMSGTPEKI
LEHFLET------------IRLEP-ALN--------------EA-----TDSVLN---D-
FVMMHCV--FM---------------PNTQLCPALV-----A------------------
--------H-----------Y-------HAQPSQ---GTEQEKMDYALNNKRRVIRLVLQ
WAAMYGDILQED-DVAMAFLE----EFYVS--VSDDA--RMLAALK---EQ-LPELEKIV
KQI---SED--AK---------APQKK---------------------------------
---HKV--------LLQQFNTGD-ERAQ-KRQ----------------------------


```
------------------------------------------------------------
------------------------------------------------------------
------------------------------------------------------------
------------------------------------------------------------
-----PIRGSDE--VL-----------------------------FK-------------
VYCVDHTYTTIRVPVAASVKEVLS--------AVADK-----------LGSG-EG--LII
VKMSSG----------------------GEKVV------LKPNDVSVFTTLTTNGRLFAC
PREQ---F-DSLT-PLPEQ---------------------------EGP------TVGTM
GT--FELMSSKDLAYQMTIYDWELFNCVHELELIY-------HTFGRHHF-KK-TTANLD
LFLRR---------FNEIQFWV-----VTEICLCSQPSKR------VQLLKKFIKIAAH-
--------------CKEYKNLNSFFAIVM-GLSNVAVSRLALTWE---------------
-------KLPSKFKKFYAE-FESLM--------------------------DPSRNH---
-RAYRL----TVAK-------LDPPLI-PFMP----------------------------
LLIKDMTFTHEGNK--TF-ID-----------------------------N-LVNFE-KM
-------RM---IANTARTVRYCRS--Q-------------------------PFNPD--
-A------------------AQANKNH-----Q----------------DVRS-----YV
RQLNVIDNQRTL-----------------------------------------SQMSHRL
-----------EPRR----------P----------------------------------
------------------------------------------------------------
------------------------------------------------------------
------------------------------------------------------------
------------------------------------------------------------
------------------------------------------------------------
------------------------------------------------------------
------------------------------------------------------------
------------------------------------------------------------
------------------------------------------------------------
------------------------------------------------------------
---
>Rapgef4_[Bos_taurus]_741916201
----------------------------M-------------------------------
------------------------------------------------------------
------------------------------------------------------------
------------------------------------------------------------
--------------------------------------------PC---KKYRQYMAG-L
LAPPYG--VMETGS--N-N--------DRIPDKE--------------------------
------------------N----------------------T------------------
-----PLIEPHVPLRPAN-TITK------------------VPS----------------
-------EKIL-------------RA---------------------GKILR-NAILSRA
PH-M----------------IRDRKYH------L-KTY----------------RQC-CV
GT-----------------ELVDWMLQQT-------------------------------
---PCVH--------SRTQAVGMW---QVLVEDSVLN-----------------------
--------------H------------------------VDQ-EHHFQDKY-LFYRFLDD
-------EHED----AP-L-----------------------------------------
----------------------------------------------------------PT
EEEK--KECDEEL------QDTMLLLSQMGPDAH-------------MRMVLRKPPGQRT
MDDLE-----IIYEELLHIKALSHLS-TTVKRELA-AVLIFESHAKGGTVL---------
--------------------------------------------FNQGEEG--TSWYIIL
KGSVNVVIYGK-----GV-------------------------------VCTLHEG-DDF
GKLAL-----------VNDAPRA---ASIVLREDNCHFLRVDKEDFNR------ILRD--
---------------------------------------------V--------------
------------------------------------------------------------
------------------------------------------------------------
------------------------EANTVRLKEHDQDVLVLEK-----VPA-GNRASNQG
```

NS----QP-QQ------------------------------------K-YTVMSGTPEKI
LEHFLET------------IRLEP-ALN--------------EA-----TDSVLN---D-
FVMMHCV--FM---------------PNTQLCPALV-----A------------------
--------H-----------Y-------HAQPSQ---GTEQEKMDYALNNKRRVIRLVLQ
WAAMYGDILQED-DVAMAFLE----EFYVS--VSDDA--RMLAALK---EQ-LPELEKIV
KQI---SED--AK---------APQKK---------------------------------
---HKV--------LLQQFNTGD-ERAQ-KRQ----------------------------

-----PIRGSDE--VL-----------------------------FK-------------
VYCVDHTYTTIRVPVAASVKEVLS--------AVADK-----------LGSG-EG--LII
VKMSSG----------------------GEKVV------LKPNDVSVFTTLTINGRLFAC
PREQ---F-DSLT-PLPEQ---------------------------EGP------TVGTM
GT--FELMSSKDLAYQMTIYDWELFNCVHELELIY-------HTFGRHHF-KK-TTANLD
LFLRR---------FNEIQFWV-----VTEICLCSQPSKR------VQLLKKFIKIAAH-
--------------CKEYKNLNSFFAIVM-GLSNVAVSRLALTWE---------------
-------KLPSKFKKFYAE-FESLM--------------------------DPSRNH---
-RAYRL----TVAK-------LDPPLI-PFMP----------------------------
LLIKDMTFTHEGNK--TF-ID-----------------------------N-LVNFE-KM
-------RM---IANTARTVRYCRS--Q-------------------------PFNPD--
-A------------------AQANKNH-----Q----------------DVRS-----YV
RQLNVIDNQRTL---SQMSHRL
-----------EPRR----------P----------------------------------

>Rapgef4_[Bubalus_bubalis]_594078793
----------------------------M-------------------------------

--PC---KKYRQYMAG-L
LAPPYG--VMETGS--N-N--------DRIPDKE--------------------------
------------------N---
---VPS----------------
-------EKIL-------------RA---------------------GKILR-NAILSRA
PH-M----------------IRDRKYH------L-KTY----------------RQC-CV
GT-----------------ELVDWMLQQT-------------------------------
---PCVH--------SRTQAVGMW---QVLVEDSVLN-----------------------
--------------H------------------------VDQ-EHHFQDKY-LFYRFLDD
-------EHED----AP-L---
--PT

EEEK--KECDEEL------QDTMLLLSQMGPDAH-------------MRMVLRKPPGQRT
MDDLE-----IIYEELLHIKALSHLS-TTVKRELA-AVLIFESHAKGGTVL---------
--FNQGEEG--TSWYIIL
KGSVNVVIYGK-----GV-------------------------------VCTLHEG-DDF
GKLAL-----------VNDAPRA---ASIVLREDNCHFLRVDKEDFNR------ILRD--
---V--------------

------------------------EANTVRLKEHDQDVLVLEK-----VPA-GNRASNQG
NS----QP-QQ------------------------------------K-YTVMSGTPEKI
LEHFLET------------IRLEP-ALN--------------EA-----TDSVLN---D-
FVMMQCV--FM---------------PNTQLCPALV-----A------------------
--------H-----------Y-------HAQPSQ---GTEQEKMDYALNNKRRVIRLVLQ
WAAMYGDILQED-DVAMAFLE----EFYVS--VSDDA--RMLATLK---EQ-LPELEKIV
KQI---SED--AK---------APQKK---------------------------------
---HKV--------LLQQFNTGD-ERAQ-KRQ----------------------------

-----PIRGSDE--VL-----------------------------FK-------------
VYCMDHTYTTIRVPVAASVKEVLS--------AVADK-----------LGSG-EG--LII
VKMSSG----------------------GEKVV------LKPNDVSVFTTLTINGRLFAC
PREQ---F-DSLT-PLPEQ---------------------------EGP------TVGTM
GT--FELMSSKDLAYQMTIYDWELFNCVHELELIY-------HTFGRHHF-KK-TTANLD
LFLRR---------FNEIQFWV-----VTEICLCSQPSKR------VQLLKKFIKIAAH-
--------------CKEYKNLNSFFAIVM-GLSNVAVSRLALTWE---------------
-------KLPSKFKKFYAE-FESLM--------------------------DPSRNH---
-RAYRL----TVAK-------LDPPLI-PFMP----------------------------
LLIKDMTFTHEGNK--TF-ID-----------------------------N-LVNFE-KM
-------RM---IANTARTVRYCRS--Q-------------------------PFNPD--
-A------------------AQANKNH-----Q----------------DVRS-----YV
RQLNVIDNQRTL---SQMSHRL
-----------EPRR----------P----------------------------------

>Rapgef4_[Buceros_rhinoceros_silvestris]_676701775

---F------H---------
-----------------------------AQPSQ---GTEQEKMDYALNNKRRVIRLVLQ
WAALYGDLLQED-EAAMAFLE----EFYVS--VSDDT--RMIAALK---EQ-LPE-----
------LEKIVKQV--SE------EPKASQ---------------------KKH------
------K-----------VLLQ--------------------------------------
-----------------L--
----FNTS-DD-----------------RAQKR---------------------------
--QPIRG---------------

---SD-------------
-------------EVL-----------------------------FK-------------
VYCVDQTYTTIRVPVSSSVKEVIS--------AVADKLG-------------SGEG-LII
VKMSSG----------------------G-------------------------------


```
------------------------------------------------------------
---
>Rapgef4_[Callorhinchus_milii]_632945696
------------------------------------------------------------
------------------------------------------------------------
------------------------------------------------------------
------------------------------------------------------------
-----------------------------------------------M-NKYRQYMAG-L
LAPPYG--VMETGS--S-N--------NRISDKE--------------------------
------------------NMN-------TNVLCPVSKNTNKT------------------
-----SIIETRLALQPAK-SITQ------------------VPS----------------
-------EKIL-------------RA---------------------GKILR-NAIVTQA
PH-M----------------IRDRKYH------L-KTY----------------KQC-CV
GT-----------------ELVDWMMQQS-------------------------------
---TCVH--------SRSQAVGMW---QALLEEGVVN-----------------------
--------------H-------------------------VDQ-EYNFQDKY-LFYRFLDD
-------EQED----AL-V-----------------------------------------
----------------------------------------------------------PT
DGEM--KECEEEL------QETMLLLSQIGPDAH-------------MRMILRKLPGQRT
VDDLE-----FIYEELLHIKALSHLS-TTVKRELA-GVLIFESHAKAGTVL---------
--------------------------------------------FNQGEEG--TSWYIIL
KGSVNVVIYGK-----GV-------------------------------VCTLHEG-DDF
GKLAL-----------VNDAPRA---ASIVLHEDNCHFLRVDKEDFNR------ILRD--
---------------------------------------------V--------------
------------------------------------------------------------
------------------------------------------------------------
------------------------EANTVRLKEHEQDVLVLEK-----IPT-CSRASVQG
ST----QT-QY------------------------------------K-YTVMSGTPEKI
LEHFLET------------LRLEL-PSN--------------ET-IVSQTDKFLD---D-
FVLMHCV--FM---------------PNSQLCPALM-----A------------------
--------H-----------Y-------HSEAFQ---GSEQEKTDYALNNKRRVIRLILK
WTNVYLDLLQED-EIAMAFLE----EFYVS--VSDDS--RMIPALK---EQ-LPELEKLV
KPN---LEEASSK---------TSQKK---------------------------------
---HKI--------ILRQFSIGE-ERLQ-KRQ----------------------------
------------------------------------------------------------
------------------------------------------------------------
------------------------------------------------------------
------------------------------------------------------------
------------------------------------------------------------
------------------------------------------------------------
-----PIRSIDE--IL-----------------------------FK-------------
VYCADHTYITIRVPVAASVKEVIS--------AVADK-----------LGSG-ED--LIL
VKISSA----------------------GERVV------LKPNDVSVFTSLNVNGRLFVC
PREH---F-DSLT-PLPEQ---------------------------EGL------STGTM
TI--FELMSSKDLAHQITIYDWELFNCVHELELVY-------HTFGRQNF-KK-TTANLD
LFLRR---------FNEIQFWV-----VTETCLCPQLSKR------VQLLKKFIKVAAH-
--------------CKEYRNLNSFFAIVM-GLSNVAVSRLSQTWE---------------
-------KLPSKFKKIYSE-FESLM--------------------------DPSRNH---
-RAYRL----TVAK-------LESPII-PFMP----------------------------
LLIKDMTFTHEGNK--TF-ID-----------------------------N-LVNFE-KM
-------HM---IGNTIRTVRYCRS--Q-------------------------SFNTD--
--------------------SIANKNH-----Q----------------DVRV-----YV
RQLNVIDNQRTL-----------------------------------------SQLSHRL
-----------EPRR----------S----------------------------------
```


>Rapgef4_[Calypte_anna]_663246129

---MSG-L
LTPPYG--VMETGS--N-N--------DRMPDKD--------------------------
------------------SMS-------SNALCQVSKNCNKT------------------
-----PLIEPHNPHRPTK-TITQ------------------VPS----------------
-------EKIL-------------RA---------------------GKILR-NTILSRA
PH-M----------------IRDRKYH------L-KTY----------------RQC-CV
GT-----------------ELVDWMMQQS-------------------------------
---PCVH--------SRTQAVGMW---QVLLEEGVLN-----------------------
--------------H------------------------VDQ-EHHFQDKY-LFYRFLDD
-------ERED----AP-L---
--PT
EEEK--KECDEEL------QDTMLLLSQIGPDAH-------------MRMLLRKPPGQRT
VDDLE-----IIYEELLHIKALSHLS-TTVKRELA-GVLIFESHPKAGTVL---------
--FNQGEEG--TSWYIIL
KGSVNVVIYGK-----GV-------------------------------VCTLHEG-DDF
GKLAL-----------VNDAPRA---ASIVLREDNCHFLRVDKEDFNR------ILRD--
---V--------------

------------------------EANTVRLKEHDQDVLVLEK-----IPA-GSRVSAQG
NS----QP-QH------------------------------------K-YIVMSGTPEKI
LEHFLET------------MRPEA-TSN--------------EA-----TDSVLN---D-
FIMMHCV--FM---------------PNSQLCPALM-----A------------------
--------H-----------Y-------HAQPSQ---GTEQEKMDYALNNKRRVIRLVLQ
WAALYGDLLQED-EAAMAFLE----EFYVS--VSDDT--RMIAALK---EQ-LAELEKIV
KQV---SEE--PK---------ATQKK---------------------------------
---HKV--------LLQLFNTSD-DRAQ-KRQ----------------------------

-----PIKGSDE--VL-----------------------------FK-------------
VYCIDQTYTTIRVPVSSSVKEVIS--------AVADK-----------LGSG-EG--LII
VKMSSG----------------------GEKVV------LKPHDVSVFTTLSVNGRLFAC
PRDQ---F-DSLA-PLPEQ---------------------------EGP------STGTV
GT--FELMSSKDLAHQMTIYDWELFNCVHELELIY-------HTFGRHNF-KK-TTANLD

LFLRR---------FNEIQFWV-----VTEICLCSQLSKR------VQLLKKYIKIAAH-
--------------CKEYKNLNSFFAIIM-GLSNVAVSRLSLTWE---------------
-------KLPSKFKKIYAE-FESLM--------------------------DPSRNH---
-RAYRL----TVAK-------LDPPVI-PFMP----------------------------
LLIKDMTFTHEGNK--TF-TD-----------------------------N-LVNFE-KM
-------RM---IANTVRTVKFCRS--Q-------------------------SFNPD--
-A------------------ALTNKNH-----Q----------------DVRS-----YV
RQLNVIDNQRTL---SQMSHRL
-----------EPRR----------A----------------------------------

>Rapgef4_[Camelus_bactrianus]_743729779
----------------------------M-------------------------------

--SENV-EKKTKKD----
VCPTRN--IDSTWQ--D-FWLLLMVLWKRALTMT--------------------------
---T------------------
-----PLIEPHVPLRPAN-TITK------------------VPS----------------
-------EKIL-------------RA---------------------GKILR-NAILSRA
PH-M----------------IRDRKYH------L-KTY----------------RQC-CV
GT-----------------ELVDWMLQQT-------------------------------
---PCVH--------SRTQAVGMW---QVLVEDGVLN-----------------------
--------------H------------------------VDQ-EHHFQDKY-LFYRFLDD
-------EHED----AP-L---
--PT
EE-K--KECDEEL------QDTMLLLSQMGPDAH-------------MRMILRKPPGQRT
VDDLE-----IIYEELLHIKALSHLS-TTVKRELA-GVLIFESHAKGGTVL---------
--FNQGEEG--TSWYIIL
KGSVNVVIYGK-----GV-------------------------------VCTLHEG-DDF
GKLAL-----------VNDAPRA---ASIVLREDNCHFLRVDKEDFNR------ILRD--
---V--------------

------------------------EANTVRLKEHDQDVLVLEK-----VPA-GNRASNQG
NS----QP-QQ------------------------------------K-YTVMSGTPEKI
LEHFLET------------IRLEP-ALN--------------EA-----TDSVLN---D-
FVMMHCV--FM---------------PNTQLCPALV-----A------------------
--------H-----------Y-------HAQPSQ---GTEQEKMDYALNNKRRVIRLVLQ
WAAMYGDVLQED-DVAMAFLE----EFYVS--VSDDA--RMIATLK---EQ-LPELEKIV
KQI---SED--AK---------TPQKK---------------------------------
---HKV--------LLQQFNTGD-ERAQ-KRQ----------------------------


```
------------------------------------------------------------
------------------------------------------------------------
------------------------------------------------------------
------------------------------------------------------------
-----PIRGSDE--VL-----------------------------FK-------------
VYCMDHTYTTIRVPVAASVKEVLS--------AVADK-----------LGSG-DG--LII
VKMSSG----------------------GEKVV------LKPNDVSVFTTLTINGRLFAC
PREQ---F-DSLT-PLPEQ---------------------------EGP------TVGTV
GT--FELMSSKDLAYQMTIYDWELFNCVHELELIY-------HTFGRHHF-KK-TTANLD
LFLRR---------FNEIQFWV-----VTEICLCSQPSKR------VQLLKKFIKIAAH-
--------------CKEYKNLNSFFAIVM-GLSNVAVSRLALTWE---------------
-------KLPSKFKKFYAE-FESLM--------------------------DPSRNH---
-RAYRL----TVAK-------LEPPLI-PFMP----------------------------
LLIKDMTFTHEGNK--TF-ID-----------------------------N-LVNFE-KM
-------RM---IANTARMVRYCRS--Q-------------------------PFNPD--
-A------------------AQANKNH-----Q----------------DVRS-----YV
RQLNVIDNQRTL-----------------------------------------SQMSHRL
-----------EPRR----------P----------------------------------
------------------------------------------------------------
------------------------------------------------------------
------------------------------------------------------------
------------------------------------------------------------
------------------------------------------------------------
------------------------------------------------------------
------------------------------------------------------------
------------------------------------------------------------
------------------------------------------------------------
------------------------------------------------------------
---
>Rapgef4_[Camelus_dromedarius]_744571826
----------------------------M-------------------------------
------------------------------------------------------------
------------------------------------------------------------
------------------------------------------------------------
--------------------------------------------WRRK-QKYRQYMAG-L
LAPPYG--VMETGS--N-N--------DRIPDKE--------------------------
------------------N----------------------T------------------
-----PLIEPHVPLRPAN-TITK------------------VPS----------------
-------EKIL-------------RA---------------------GKILR-NAILSRA
PH-M----------------IRDRKYH------L-KTY----------------RQC-CV
GT-----------------ELVDWMLQQT-------------------------------
---PCVH--------SRTQAVGMW---QVLVEDGVLN-----------------------
--------------H------------------------VDQ-EHHFQDKY-LFYRFLDD
-------EHED----AP-L-----------------------------------------
----------------------------------------------------------PT
EEEK--KECDEEL------QDTMLLLSQMGPDAH-------------MRMILRKPPGQRT
MDDLE-----IIYEELLHIKALSHLS-TTVKRELA-GVLIFESHAKGGTVL---------
--------------------------------------------FNQGEEG--TSWYIIL
KGSVNVVIYGK-----GV-------------------------------VCTLHEG-DDF
GKLAL-----------VNDAPRA---ASIVLREDNCHFLRVDKEDFNR------ILRD--
---------------------------------------------V--------------
------------------------------------------------------------
------------------------------------------------------------
------------------------EANTVRLKEHDQDVLVLEK-----VPA-GNRASNQG
```

NS----QP-QH------------------------------------K-YTVMSGTPEKI
LEHFLET------------IRLEP-ALN--------------EA-----TDSVLN---D-
FVMMHCV--FM---------------PNTQLCPALV-----A------------------
--------H-----------Y-------HAQPSQ---GTEQEKMDYALNNKRRVIRLVLQ
WAAMYGDVLQED-DVAMAFLE----EFYVS--VSDDA--RMIATLK---EQ-LPELEKIV
KQI---SED--AK---------TPQKK---------------------------------
---HKV--------LLQQFNTGD-ERAQ-KRQ----------------------------

-----PIRGSDE--VL-----------------------------FK-------------
VYCMDHTYTTIRVPVAASVKEVLS--------AVADK-----------LGSG-DG--LII
VKMSSG----------------------GEKVV------LKPNDVSVFTTLTINGRLFAC
PREQ---F-DSLT-PLPEQ---------------------------EGP------TVGTV
GT--FELMSSKDLAYQMTIYDWELFNCVHELELIY-------HTFGRHHF-KK-TTANLD
LFLRR---------FNEIQFWV-----VTEICLCSQPSKR------VQLLKKFIKIAAH-
--------------CKEYKNLNSFFAIVM-GLSNVAVSRLALTWE---------------
-------KLPSKFKKFYAE-FESLM--------------------------DPSRNH---
-RAYRL----TVAK-------LEPPLI-PFMP----------------------------
LLIKDMTFTHEGNK--TF-ID-----------------------------N-LVNFE-KM
-------RM---IANTARMVRYCRS--Q-------------------------PFNPD--
-A------------------AQANKNH-----Q----------------DVRS-----YV
RQLNVIDNQRTL---SQMSHRL
-----------EPRR----------P----------------------------------

>Rapgef4_[Capra_hircus]_926684811

---MAG-L
LAPPYG--VMETGS--N-N--------DRIPDKE--------------------------
------------------N---
---VPS----------------
-------EKIL-------------RA---------------------GKILR-NAILSRA
PH-M----------------IRDRKYH------L-KTY----------------RQC-CV
GT-----------------ELVDWMLQQT-------------------------------
---PCVH--------SRTQAVGMW---QVLVEDSVLN-----------------------
--------------H------------------------VDQ-EHHFQDKY-LFYRFLDD
-------EHED----AP-L---
--PT

EEEK--KECDEEL------QDTMLLLSQMGPDAH-------------MRMVLRKPPGQRT
MDDLE-----IIYEELLHIKALSHLS-TTVKRELA-AVLIFESHAKGGTVL---------
--FNQGEEG--TSWYIIL
KGSVNVVIYGK-----GV-------------------------------VCTLHEG-DDF
GKLAL-----------VNDAPRA---ASIVLREDNCHFLRVDKEDFNR------ILRD--
---V--------------

------------------------EANTVRLKEHDQDVLVLEK-----VPA-GNRASNQG
NS----QP-QQ------------------------------------K-YTVMSGTPEKI
LEHFLET------------IRLEP-ALN--------------EA-----TDSVLN---D-
FVMMHCV--FM---------------PNTQLCPALV-----A------------------
--------H-----------Y-------HAQPSQ---GTEQEKMDYALNNKRRVVRLVLQ
WAAMYGDILQED-DVAMAFLE----EFYVS--VSDDA--RMLAALK---EQ-LPELEKIV
KQI---SED--AK---------APQKK---------------------------------
---HKV--------LLQQFNTGD-ERAQ-KRQ----------------------------

-----PIRGSDE--VL-----------------------------FK-------------
VYCLDHTYTTIRVPVAASVKEVLS--------AVADK-----------LGSG-EG--LII
VKMSSG----------------------GEKVV------LKPNDVSVFTTLTINGRLFAC
PREQ---F-DSLT-PLPEQ---------------------------EGP------TVGTM
GT--FELMSSKDLAYQMTIYDWELFNCVHELELIY-------HTFGRHHF-KK-TTANLD
LFLRR---------FNEIQFWV-----VTEICLCSQPSKR------VQLLKKFIKIAAH-
--------------CKEYKNLNSFFAIVM-GLSNVAVSRLALTWE---------------
-------KLPSKFKKFYAE-FESLM--------------------------DPSRNH---
-RAYRL----TVAK-------LDPPLI-PFMP----------------------------
LLIKDMTFTHEGNK--TF-ID-----------------------------N-LVNFE-KM
-------RM---IANTARTVRYCRS--Q-------------------------PFNPD--
-A------------------AQANKNH-----Q----------------DVRS-----YV
RQLNVIDNQRTL---SQMSHRL
-----------EPRR----------P----------------------------------

>Rapgef4_[Caprimulgus_carolinensis]_704296106

---MSG-L
LTPPYG--VMETGS--N-N--------DRMPDKD--------------------------

------------------GVS-------SSALCQV-------------------------
----------------SK-NCNK------------------VPS----------------
-------EKIL-------------RA---------------------GKILR-NTILSRA
PH-M----------------IRDRKYH------L-KTY----------------RQC-CV
GT-----------------ELVDWMMQQS-------------------------------
---PCVH--------SRTQAIGMW---QVLLEEGVLN-----------------------
--------------H------------------------VDQ-EHHFQDKY-LFYRFLDD
-------EHED----AP-L---
--PT
EEEK--KECDEEL------QDTMLLLSQIGPDAH-------------MRMILRKPPGQRT
IDDLE-----IIYEELLHIKALSHLS-TTVKRELA-GVLIFESHPKAGTVL---------
--FNQGEEG--TSWYIIL
KGSVNVVIYGK-----GV-------------------------------VCTLHEG-DDF
GKLAL-----------VNDAPRA---ASIVLREDNCHFLRVDKEDFNR------ILRD--
---V--------------

------------------------EANTVRLKEHDQDVLVLEK-----IPA-GNRVSNQG
NP----QP-QH------------------------------------K-YIVMSGTPEKI
LEHFLET------------MRPEA-TLN--------------EA-----TDSVLN---D-
FIMMHCV--FM---------------PNSQLCPALM-----A------------------
--------H-----------Y-------HAQPSQ---GTEQEKMDYALNNKRRVIRLVLQ
WAALYGDLLQED-EAAMAFLE----EFYVS--VSDDT--RMIAALK---EQ-LPELEKVV
KQV---SEE--PK---------APQKK---------------------------------
---HKV--------LLQLFNTSD-DRAQ-KRQ----------------------------

-----PIRGSDE--VL-----------------------------FK-------------
VYCIDQTYTTIRVPVSSSVKEVIS--------AVADK-----------LGSG-EG--LII
VKMSSG----------------------GEKVV------LKPHDVSVFTTLSVNGRLFAC
PRDQ---F-DSLA-PLPEQ---------------------------EGP------STGTI
GT--FELMSSKDLAHQMTVYDWELFNCVHELELIY-------HTFGRHNF-KK-TTANLD
LFLRR---------FNEIQFWV-----VTEICLCSQLSKR------VQLLKKYIKIAAH-
--------------CKEYKNLNSFFAIIM-GLSNVAVSRLSLTWE---------------
-------KLPSKFKKIYAE-FESLM--------------------------DPSRNH---
-RAYRL----TVAK-------LDPPII-PFMP----------------------------
LLIKDMTFTHEGNK--TF-TD-----------------------------N-LVNFE-KM
-------RM---IANTVRTVKFCRS--Q-------------------------SFNPD--
-A------------------ALTNKNH-----Q----------------DVRS-----YV
RQLNVIDNQRTL---SQMSHRL
-----------EPRR----------A----------------------------------

>Rapgef4_[Capsaspora_owczarzaki_ATCC_30864]_470326013

-------------------------------------MSANDEESSPLTRRHGGRHHHKP
TKPATNTTPATGAASAAASNGNGEMSNHSDTTSGGFATTPATATVTTAAATTTTAAAAAA
ATAGGGAATSGSSTSLTNTVLTAPSSADLAAMSPATRRR-MLNINPSGRPSGKQAIVLLL
DDAEDEHDNDDDDDDDNDGSS--GGEPVAAVLATPTAAPAPAPAPAPAPAPAPVVVAASN
SY-RDSFDEDEMTNRRASQPRTAAAQQHPPPPPAGTVPFTLTPKVQTPTLIVELEASPAQ
--RRRDARKLSAPIEVAPEVLTPGDASSLRYSYSASASPAHSP-LGSSERIQSHNSDTNI
PA---RQPIRRSASQGME---EDNRHSTLFGVEA-KALGHHPSSVSATVSPLGSTEL-LE
GKPPKTTAAAAFAARFSGTASPDSPIRHNELLALAASMGCNPPKSPAPLSPVNIQFGDNL
LV-PEVHTRVPRVDAARRKQLGRSVSNPDAIAEAIAANAALAAQLADNGRAAGDAPQAQA
SSSSSSSSSDAAQQHKIVP--SKTKMQAVAARAKMQRRTTNR--SLTNDKRGGLMALLRD
E-----TSRKQGGISSNGFLTVPGEDGISQASSVSSLSDEEEEEEEFHLSFGGIPEDSQT
SNSSNDLVGG--NAGVDDDDDDL-IAPSRLGNLGLGGMGLDFSSGMDFADAVRSSMDFDT
TMEF-TSSAGADFA--STSVEDLLATSEEVVESEDLSPLEARERRL---ALLDIPPASRT
PEQVTE-----LCRLLQDFPAFAELNGDTPRRELC--AIAKFVRFEESDSI---------
--VDVMDELCETQWYFVH
QGALDVIIDGE------I-------------------------------VVTTLEG-EGF
GQKPL----------VVDGRVRLD--AIVLTRGPDVELLNVPLQHYER------ISRL--
---VE-------------

------------------------STTEKIVDDAGEVVMILDKRTRT-APL-------QF
GL-----PSQR-----------------------N-----------VS-VLVTQATLEKL
IERLYLAD-------------LQALDEFYH------------------------Q---D-
FLLSHRT--FT---------------SSENLLGAMI-----DRL------RLPGA-----
-------------------AFVPKAPKGAAAPIT--------SPEQLRSPAHVIGDILLD
WMADYPKDFCRP-----SMWD-----LLAQ------AQ-KLLTALEPKLAE-VRELATAV
LAL-RPAESGAPPT--SGT---APLTREPSLGL---------------------------
---HAVHANLAPL----GFALGP----DPRRRASSAASSIIVPKSANAQP----------
-----HTPPSG------L--QSARFKRFFGRGNSSEER--------------AGSAAASP
A-APSTAT-AS--------------PAGAAKET---------------------------

--NG------
----------------------------------VPAAK---------------------
-----TVRTQRH-RLL---------------------DEDSDFEVIR-------------
VYRGDQTFRYVAVYPHTTAAEVVS--------ELLESFSI-----Q--LKSASDRTEYVL
VMAVAA---------------------LGSAPG--RVSRLSPAADHLAQTLSIHSRYYLR
NNAN---LAERLLP--PEPIAVEG------------------------------------
ES-LILAMDPIELARQITLEDSELFRSISPSEYVD-------YLFKLDNNK----CENLH
KFIER---------FNLINFWV-----VTQIVSTRDLRKR------AMVIRFLVLMAKCF
---------------KEWNNFNSLFALVS-GFANSAVTRLHNTHS---------------
-------KLSKRTVAAMQG-FEALM--------------------------NPTRNM---
-GAYRSMLLQ-VPE-------G-TPLV-PFFP----------------------------
LLMKDLVFIHEGNP--SK--------------------------F-----NGLINFD-KR
-------RL---IGRTLRNVKRMAEVGYL-DANFKFELDE-AKELDAFGSEQDDAKILRD
TVKRSLVASKTANVNTLSIGSAGAAMR-----QLFEKTRTKFF-------VQEYLGE-LV
KHVDL--DQNRL---SVRSAEL
EHRP--

>Rapgef4_[Cariama_cristata]_698410374

---------------L-----------------------------FK-------------
VYCVDQTYTTIRVPVSCSVKEVIS--------AVADK-----------LGSG-EG--LII
VKMSSG----------------------GEKVV------LKPHDVSVFTTLSVNGRLFAC
PRDQ---F-DSLA-PLPEQ---------------------------EGP------STGTV
GT--FELMSSKDLAHQMTIYDWELFNCVHELELIY-------HTFGRHNF-KK-TTANLD

LFLRR---------FNEIQFWV-----VTEICLCSQLSKR------VQLLKKYIKIAAH-
--------------CKEYKNLNSFFAIIM-GLSNVAVSRLSLTWE---------------
-------KLPSKFKKIYAE-FESLM--------------------------DPSRNH---
-RAYRL----TVAK-------LDPPII-PFMP----------------------------
LLIKDMTFTHEGNK--TI-TD-----------------------------N-LVNFE-KM
-------RM---IANTVRTVKFCRS--Q-------------------------SFNPD--
-A------------------ALTNKN----------HQ-----------DVRS-----YV
RQLNVIDNQRTL---SQMSHRL
-----------EPRR----------A----------------------------------

>Rapgef4_[Carlito_syrichta]_640812551
----------------------------MVAA-------HA------AH--------SSS
SAEWIA---CLDK-----------RPLE-----RSSEDVDIIFTRLKEVKAFEKFHPNLL
HQICLCGY--YENLE--KGITLFRQGDIGTNWYAVLAGSLDVKVSE-TSSHQDA------
------VTICTLGIGTAFGESILDNT-PRHATIV-TRESSELL-----------------
----------------------------RIEQKD-------FKALW---EKYRQYMAG-L
LAPPYG--VMETGS--N-N--------DRIPDKE--------------------------
------------------N---
---VPS----------------
-------EKIL-------------RA---------------------GKILR-NAILSRA
PH-M----------------IRDRKYH------L-KTY----------------RQC-CV
GT-----------------ELVDWMMQQT-------------------------------
---PCVH--------SRTQAVGMW---QVLLEDGVLN-----------------------
--------------H------------------------VDQ-EHHFQDKY-LFYRFLDD
-------EHED----AP-L---
--PT
EEEK--KECDEEL------QDTMLLLSQMGPDAH-------------MRMILRKPPGQRT
VDDLE-----IIYEELLHIKALSHLS-TTVKRELA-GVLIFESHAKGGTVL---------
--FNQGEEG--TSWYIIL
KGSVNVVIYGK-----GV-------------------------------VCTLHEG-DDF
GKLAL-----------VNDAPRA---ASIVLREDNCHFLRVDKEDFNR------ILRD--
---V--------------

------------------------EANTVRLKEHDQDVLVLEK-----VPA-GNRASNQG
NS----QP-QQ------------------------------------K-YTVMSGTPEKI
LEHFLET------------IRLEP-ALN--------------EA-----TDSVLN---D-
FVMMHCV--FM---------------PNTQLCPALV-----A------------------
--------H-----------Y-------HAQPSQ---GTEQEKMDYAFNNKRRVIRLVLQ
WAAMCGDLLQED-DVAMAFLE----EFYVS--VSDDA--RMIAALK---EQ-LPELEKIV
KQI---SED--TK---------APQKK---------------------------------
---HKV--------LLQQFNTGD-ERAQ-KRQ----------------------------


```
------------------------------------------------------------
------------------------------------------------------------
------------------------------------------------------------
------------------------------------------------------------
-----PIRGSDE--VL-----------------------------FK-------------
VYCLDHTYTTIRVPVAASVKEVIS--------AVADK-----------LGSG-EG--LII
VKMSSG----------------------GEKVV------LKPNDVSVFTTLTINGRLFAC
PREQ---F-DSLT-PLPEQ---------------------------EGP------TVGTV
GT--FELMSSKDLAFQMTIYDWELFNCVHELELIY-------HTFGRHNF-KK-TTANLD
LFLRR---------FNEIQFWV-----VTEICLCSQLSKR------VQLLKKFIKIAAH-
--------------CKEYKNLNSFFAIVM-GLSNIAVSRLALTWE---------------
-------KLPSKFKKFYAE-FESLM--------------------------DPSRNH---
-RAYRL----TVAK-------LDPPLI-PFTP----------------------------
LLIKDMTFTHEGNK--TF-VD-----------------------------N-LVNFE-KM
-------RM---IANTARTVRYYRS--Q-------------------------PFNPD--
-A------------------AQANKNH-----Q----------------DVRS-----YV
RQLNVIDNQRTL-----------------------------------------SQMSHRL
-----------EPRR----------P----------------------------------
------------------------------------------------------------
------------------------------------------------------------
------------------------------------------------------------
------------------------------------------------------------
------------------------------------------------------------
------------------------------------------------------------
------------------------------------------------------------
------------------------------------------------------------
------------------------------------------------------------
------------------------------------------------------------
---
>Rapgef4_[Cathartes_aura]_677231281
------------------------------------------------------------
------------------------------------------------------------
------------------------------------------------------------
------------------------------------------------------------
------------------------------------------------------------
------------------------------------------------------------
-----------------------------------------T------------------
-----PLIEPHIPHRPTK-TITQ------------------VPS----------------
-------EKIL-------------RA---------------------GKILR-NTILSRA
PH-M----------------IRDRKYH------L-KTY----------------RQC-CV
GT-----------------ELVDWMMQQS-------------------------------
---PCVH--------SRTQAVGMW---QVLLEEGVLN-----------------------
--------------H------------------------VDQ-EHHFQDKY-LFYRFLDD
-------ERED----AS-L-----------------------------------------
----------------------------------------------------------PT
EEEK--KECDEEL------QDTMLLLSQIGPDAH-------------MRMILRKPPGQRT
IDDLE-----FIYEELLHIKALSHLS-TTVKRELA-GVLIFESHPKAGTVL---------
--------------------------------------------FNQGEEG--TSWYIIL
KGSVNVVIYGK-----GV-------------------------------VCTLHEG-DDF
GKLAL-----------VNDAPRA---ASIVLREDNCHFLRVDKEDFNR------ILRD--
---------------------------------------------V--------------
------------------------------------------------------------
------------------------------------------------------------
------------------------EANTVRLKEHDQDVLVLEK-----IPA-GNRVSNQG
```

NS----QP-QH------------------------------------K-YIVMSGTPEKI
LEHFLET------------MRLEA-TLN--------------EA-----TDSVLN---D-
FIMMHCV--FM---------------PNSQLCPALM-----A------------------
--------Q-----------YPFHRGTYHAQPSQ---GTEQEKMDYALNNKRRVIRLVLQ
WAALYGDLLQED-EAAMAFLE----EFYVS--VSDDT--RMIAALK---EQ-LPELEKIV
KQV---SEE--PK---------APQKK---------------------------------
---HKV--------LLQLFNTSD-DRAQ-KRQ----------------------------

-----PIRGSDE--VL-----------------------------FK-------------
VYCIDQTYTTIRVPVSSSVKEVIS--------AVADK-----------LGSG-EG--LII
VKMSSG----------------------GEKVV------LKPHDVSVFTTLSVNGRLFAC
PRDQ---F-DSLA-PLPEQ---------------------------EGP------SAGTV
GT--FELMSSKDLAHQMTIYDWELFNCVHELELIY-------HTFGRHNF-KK-TTANLD
LFLRR---------FNEIQFWV-----VTEICLCSQLSKR------VQLLKKYIKIAAH-
--------------CKEYKNLNSFFAIIM-GLSNVAVSRLSLTWE---------------
-------KLPSKFKKIYAE-FESLM--------------------------DPSRNH---
-RAYRL----TVAK-------LDPPII-PFMP----------------------------
LLIKDMTFTHEGNK--TF-TD-----------------------------N-LVNFE-KM
-------RM---IANTVRTVKFCRS--Q-------------------------SFNPD--
-A------------------ALTNKNH-----Q----------------DVRS-----YV
RQLNVIDNQRTL---SQMSHRL
-----------EPRR----------A----------------------------------

>Rapgef4_[Cavia_porcellus]_884941756

---MAG-L
LAPPYG--VMETGS--N-N--------DRIPDKE--------------------------
------------------N---
---VPS----------------
-------EKIL-------------RA---------------------GKILR-NAILSRA
PH-M----------------IRDRKYH------L-KTY----------------RQC-CV
GT-----------------ELVDWMLQQT-------------------------------
---PCVH--------SRTQAVGMW---QVLLEDGVLN-----------------------
--------------H------------------------VDQ-EHHFQDKY-LFYRFLDD
-------EHED----AP-L---
--PT

EEEK--KECDEEL------QDTMLLLSQMGPDAH-------------MRMILRKPPGQRT
VDDLE-----IIYEELLHIKALSHLS-TTVKRELA-GVLIFESHAKGGTVL---------
--FNQGEEG--TSWYIIL
KGSVNVVIYGK-----GV-------------------------------VCTLHEG-DDF
GKLAL-----------VNDAPRA---ASIVLREDNCHFLRVDKEDFNR------ILRD--
---V--------------

------------------------EANTVRLKEHDQDVLVLEK-----VPA-GSRASNQG
NS----QP-QQ------------------------------------K-YTVMSGTPEKI
LEHFLET------------IRLEP-ALN--------------EA-----TDSILN---D-
FIMMHCV--FM---------------PNTQLCPALV-----A------------------
--------H-----------Y-------HAQPSQ---GTEQEKLDYALNNKRRVIRLVLQ
WASTYGDLLQED-DVAMAFLE----EFYVS--VSDDA--RMIAALK---EQ-LPELERIV
KQI---SED--AK---------APQKK---------------------------------
---HKV--------LLQQFNTSD-ERAQ-KRQ----------------------------

-----PIRGSDE--VL-----------------------------FK-------------
VYCMDHTYTTIRVPVAASVKEVIS--------AVADK-----------LGSG-EG--LII
VKMSSG----------------------GEKVV------LKPNDVSVFTTLTINGRLFAC
PREQ---F-DSLT-PLPEQ---------------------------EGP------TVGTV
GT--FELMSSKDLAYQMTIYDWELFNCVHELELIY-------HTFGRHNF-KK-TTANLD
LFLRR---------FNEIQFWV-----VTEICLCSQLSKR------VQLLKKFIKIAAH-
--------------CKEYKNLNSFFAIVM-GLSNVAVSRLALTWE---------------
-------KLPSKFKKFYAE-FESLM--------------------------DPSRNH---
-RAYRL----TAAK-------LEPPLI-PFMP----------------------------
LLIKDMTFTHEGNK--TF-ID-----------------------------N-LVNFE-KM
-------RM---IANTARTVRYYRS--Q-------------------------PFSPD--
-A------------------AQANKNH-----Q----------------DVRS-----YV
RQLNVIDNQRTL---SQMSHRL
-----------EPRR----------P----------------------------------

>Rapgef4_[Ceratitis_capitata]_499006314
-----------------------------MAMEWITAM----------------------
----------------------DKRPCD-----RNLRDVELISCRLRRVEPLCRLPSSAL
QQLAMCGF--YEDLE--KGVTLFRAGEQGRFWYAVLGGSLEVRYHA-SDADAK------A
P-----VTLCTLGVGATFGESILHDL-PRDSTVV-TKTTCELLRVEQQDFRLIWE-----
KNKE--LINDII--------------T-TCKLKNGFGSGV---SP-----VA-SS-----
---PT------------------K--RPLSPDHPNPAL----------------------

```
------------------------------------------------------------
------------------------------------------PISE--------------
-----------------SPSPAMNRM---------------------GRALR-TLLLADS
SS-C----------------LKDRKVA------G-K-L---------------IRKC-AP
GT-----------------ELVDWLLNLS-------------------------------
---PIVH--------TRAQAAGMW---QALLEEGVLS-----------------------
--------------H------------------------VNK-EQPFKDKC-FLYRFRVD
E---------D----S--------------------------------------------
-----------------------------------------------------ATSSYST
SEDI--NTANEHI------RESISALLQRGPDAT-------------LRMILRKPSHERT
PEELE-----LIFEELLHITALSHLS-TSIKRELS-SIIVFESHAQAGTIL---------
--------------------------------------------FNQGDEG--RSWYILL
KGSVDVVIHGK-----GT-------------------------------VATLKNG-DDF
GKLAL-----------INDAPRA---ATIVLKENNCHLLRVDKEHFNR------ILRD--
---------------------------------------------V--------------
------------------------------------------------------------
------------------------------------------------------------
------------------------EANTLRLQEHGKDVLVLER-----VAK---------
------QRGQ----HS---------------AFK---------------YTVMSGTPSKI
LEHLLET-------------RL---G---N-------QVSS--------LDPFLD---D-
FLLTNMV--FM---------------PVIQLVDELA-----N------------------
--------YF---HCDV---------N-----DA---AQTPEDREYIINFKKRVIQFMHK
WVLVARHTALDE-PCVCDFIE----EMALE--VEAN-----------------PELSEET
SNI---------------------------------------------------------
---HNL--------LTQKARYQE-DRKQNSAQK---------------------------
----WKLPP---NGQPVC--------------------------------LFSGNTT---
---SSRN---T-------------------------------------------------
------------------------------------------------------------
------------------------------------------------------------
------------------------------------------------------------
------------------------------------------------------------
------MHPDDD--II-----------------------------FR-------------
VYCADHTYCTLRFPLHTTAEIIKA--------CAAEK-----------LQLNRGAEDLVL
VEVKSN----------------------GERAV------FKDNDVSIPTGLSLNGRLFVT
VKDH---V-DAVT-PLPEQ---------------------------EGP------TEGID
I--DLEILSTKDLAFYITIYDWDLFWVVHEYELLY-------RTFGRHHF--GKITANLD
VFLRR---------FNELQYWI-----VTDIVSASSMSKR------VGLLRKFIKLAAY-
--------------CKEYNNLNAFFAIVM-GLSNMAVSRLTQTWD---------------
-------KIPSKFRKLFQE-FEALI--------------------------DPSRNH---
-RAYRV----YVGK-------LQPPLI-PFMP----------------------------
LLLKDMTFAHEGNK--TS-LD-----------------------------G-LVNFE-KM
-------HM---MAQTMRTMRYCRS--R------TISL-----------------DPP--
----------------------SPKSE-----G----------------DARS-----YI
CCLRAIDNQRVL-----------------------------------------TAMSQKL
-----------EPTR----------KV---------------------------------
------------------------------------------------------------
------------------------------------------------------------
------------------------------------------------------------
------------------------------------------------------------
------------------------------------------------------------
------------------------------------------------------------
------------------------------------------------------------
------------------------------------------------------------
------------------------------------------------------------
```


>Rapgef4_[Ceratotherium_simum_simum]_955513373
---M
VAAHA-AHSSSSAEWIA--C-LDKRPLE-----RSSEDVDIIFTRLKEVK----------

--------------------AFEKFH-PNLLHQIC--LCGYYENLEKGITL---------
--FRQGDIG--TNWYAVL
AGSLDVKVSETSSHQDAVT------------------------------ICTLGIG-TAF
GE-SI-----------LDNTPRH---ATIVTRE-SSELLRIEQKDFQA------LWE---

>Rapgef4_[Chaetura_pelagica]_701389426
----------------------------MGTA-------FG------ESILDNTPR----
-------------HATIVTREYS-------------------------------------

--ELL-----------------
----------------------------RIEQKD-------FKALW---EKYRQYMSG-L
LTPPYG--VMETGS--N-N--------DRMPDKD--------------------------
------------------SMS------SSALCQV--------------------------
----------------SK-NCNK------------------VPS----------------
-------EKIL-------------RA---------------------GKILR-NTILSRA
PH-M----------------IRDRKYH------L-KTY----------------RQC-CV
GT-----------------ELVDWMMQQS-------------------------------
---PCVH--------SRTQAVGMW---QVLLEEGVLN-----------------------
--------------H------------------------VDQ-EHHFQDKY-LFYRFLDD
-------ERED----AP-L---
--PT
EEEK--KECDEEL------QDTMLLLSQIGPDAH-------------MRMLLRKLPGQRT
VDDLE-----IIYEELLHIKALSHLS-TTVKRELA-GVLIFESHPKAGTVL---------
--FNQGEEG--TSWYIIL
KGSVNVVIYGK-----GV-------------------------------VCTLHEG-DDF
GKLAL-----------VNDAPRA---ASIVLREDNCHFLRVDKEDFNR------ILRD--
---V--------------

------------------------EANTVRLKEHDQDVLVLEK-----IPA-GSRVSNQG
NS----QP-QH------------------------------------K-YTVMSGTPEKI
LEHFLET------------MRPET-TLN--------------EA-----TDSVLN---D-
FIMMHCV--FM---------------PNSQLCPALM-----A------------------
--------H-----------Y-------HAQPSQ---GTEQEKMDYALNNKRRVIRLVLQ
WAALYGDLLQED-EAAMAFLE----EFYVS--VSDDT--RMIAALK---EQ-LPELEKIV
KQV---SEE--PK---------APQKK---------------------------------
---HKV--------LLQLFNTSD-DRTQ-KRQ----------------------------

-----PIRGSDE--VL-----------------------------FK-------------
VYCIDQTYTTIRVPVASSVKEVIS--------AVADK-----------LSSG-EG--LII
VKMSSG----------------------GEKVV------LKPHDVSVFTTLSVNGRLFAC
PRDQ---F-DSLA-PLPEQ---------------------------EGP------STGTV
GT--FELMSSKDLAHQMTIYDWELFNCVHELELIY-------HTFGRHNF-KK-TTANLD

LFLRR---------FNEIQFWV-----VTEICLCSQLSKR------VQLLKKYIKIAAH-
--------------CKEYKNLNSFFAIIM-GLSNVAVSRLSLTWE---------------
-------KLPSKFKKIYAE-FESLM--------------------------DPSRNH---
-RAYRL----TVAK-------LDPPII-PFMP----------------------------
LLIKDMTFTHEGNK--TF-TD-----------------------------N-LVNFE-KM
-------RM---IASTVRTVKFCRS--Q-------------------------SFNPD--
-A------------------ALTNKNH-----Q----------------DVRS-----YV
RQLNVIDNQRTL---SQMSHRL
-----------EPRR----------A----------------------------------

>Rapgef4_[Charadrius_vociferus]_699632739

---MSG-L
LTPPYG--VMETGS--N-N--------DRMPDKD--------------------------
------------------SMS-------SSALCQV-------------------------
----------------SK-NCNK------------------VPS----------------
-------EKIL-------------RA---------------------GKILR-NTILSRA
PH-M----------------IRDRKYH------L-KTY----------------RQC-CV
GT-----------------ELVDWMMQQS-------------------------------
---TCVH--------SRTQAVGMW---QVLLEEGVLN-----------------------
--------------H------------------------VDQ-EHHFQDKY-LFYRFLDD
-------ERED----AP-L---
--PT
EEEK--KECDEEL------QDTMLLLSQIGPDAH-------------MRMILRKPPGQRT
VDDLE-----IIHEELLHIKALSHLS-TTVKRELA-GVLIFESHPKAGTVL---------
--FNQGEEG--TSWYIIL
KGSVNVVIYGK-----GV-------------------------------VCTLHEG-DDF
GKLAL-----------VNDAPRA---ASIVLREDNCHFLRVDKEDFNR------ILRD--
---V--------------

------------------------EANTVRLKEHDQDVLVLEK-----IPA-GNRVSNQG
NS----QP-QH------------------------------------K-YIVMSGTPEKI
LEHFLET------------MRLEA-TLN--------------EA-----TDSVLN---D-
FIMMHCV--FM---------------PNSQLCPALM-----A------------------
--------H-----------Y-------HAQPSQ---GTEQEKMDYALNNKRRVIRLVLQ
WAALYGDLLQED-EAAMAFLE----EFYVS--VSDDT--RMIAALK---EQ-LPELEKIV
KQV---SEE--PK---------APQKK---------------------------------
---HKV--------LLQLFNTSD-DRAQ-KRQ----------------------------


```
------------------------------------------------------------
------------------------------------------------------------
------------------------------------------------------------
------------------------------------------------------------
-----PIRGSDE--VL-----------------------------FK-------------
VYCIDQTYTTIRVPVSSSVKEVIS--------AVADK-----------LGSG-EG--LII
VKMSSG----------------------GEKVV------LKPHDVSVFTTLSVNGRLFAC
PRDQ---F-DSLA-PLPEQ---------------------------EGP------STGTV
GT--FELMSSKDLAHQMTIYDWELFNCVHELELIY-------HTFGRHNF-KK-TTANLD
LFLRR---------FNEIQFWV-----VTEICLCSQLSKR------VQLLKKYIKIAAH-
--------------CKEYKNLNSFFAIIM-GLSNVAVSRLSLTWE---------------
-------KLPSKFKKIYAE-FESLM--------------------------DPSRNH---
-RAYRL----TVAK-------LDPPII-PFMP----------------------------
LLIKDMTFTHEGNK--TF-TD-----------------------------N-LVNFE-KM
-------RM---IANTVRTVKFCRS--Q-------------------------SFNPD--
-A------------------ALTNKNH-----Q----------------DVRS-----YV
RQLNVIDNQRTL-----------------------------------------SQMSHRL
-----------EPRR----------A----------------------------------
------------------------------------------------------------
------------------------------------------------------------
------------------------------------------------------------
------------------------------------------------------------
------------------------------------------------------------
------------------------------------------------------------
------------------------------------------------------------
------------------------------------------------------------
------------------------------------------------------------
------------------------------------------------------------
---
>Rapgef4_[Chelonia_mydas]_591371593
----------------------------MVAA-------HA------SH--------SSS
SSEWIA---CLDK-----------RPLE-----RSSEDVDIIFTRLKEVKAFEKFHPNLL
QQICLCGY--YENLE--KGITLFRQGDIGTNWYAVLAGSLDVKVSD-TSNYQDA------
------VTICTLGIGTAFGESILDNT-PRHATIV-TREYSELL-----------------
----------------------------RIEQKD-------FKALW---EKYRQYMSG-L
LAPPYG--VMETGS--N-N--------DRMPDKE--------------------------
------------------NMS-------SNTLCPVSKNCNKT------------------
-----PLIEPHIPLRPAK-TITQ------------------VPS----------------
-------EKIL-------------RA---------------------GKILR-NAILSRA
PH-M----------------IRDRKYH------L-KTY----------------RQC-CV
GT-----------------ELVDWMMQQS-------------------------------
---PCVH--------SRTQAVGMW---QVLLEEGVLN-----------------------
--------------H------------------------VDQ-EHHFQDKY-LFYRFLDD
-------EHED----AP-M-----------------------------------------
----------------------------------------------------------PT
EEEK--KECDEEL------QDTLLLLSQIGPDAH-------------MRMILRKPPGQRT
VDDLE-----IIYEELIHIKALSHLS-TTVKRELA-GVLIFESHPKAGTVL---------
--------------------------------------------FNQGEEG--TSWYIIL
KGSVNVVIYGK-----TIDHLNITQRSYR-------------------HTCTNPKGIDDF
GKLAL-----------VNDAPRA---ASIVLREDNCHFLRVDKEDFNR------ILRD--
---------------------------------------------V--------------
------------------------------------------------------------
------------------------------------------------------------
------------------------EANTVRLKEHDQDVLVLEK-----IPA-GNRASNQG
```

NS----QP-QH------------------------------------K-YTVMSGTPEKI
LEHFLET------------MRLES-TLN--------------EA-----TDSVLN---D-
FVMMHCV--FM---------------PNSQLCPALM-----A------------------
--------H-----------Y-------HAQPSQ---GSEQEKMDYALNNKRRVLRLVLQ
WAASYGDLLQED-EAAVAFLE----EFYVS--VSDDA--RIITALK---EQ-LSELEKTV
KQI---SEE--TK---------APQKK---------------------------------
---HKI--------LLQQFNTTD-DRAQ-KRQ----------------------------

-----PIRGSDE--IL------------------------------FK------------
VYCIDHTYTTIRVPVMASVKEVIS--------AVADK-----------LGSG-EG--LII
VKMSSG----------------------GEKVV------LKPNDGSAFTTLSVNGRLFAC
PRDQ---F-DSLT-PLPEQ---------------------------EGP------SAGTV
GT--FELMSSKDLAYQMTIYDWELFNCVHELELIY-------HTFGTHNF-KK-TTANLD
LFLRR---------FNEIQFWV-----VTEICLCSQLSKR------VQLLKKFIKIAAH-
--------------CKEYKNLNSFFAIIM-GLSNVAVSRLSLTWE---------------
-------KLPSKFKKIYAE-FESLM-------------------------DPSRNH---
-RAYRL----TVAK-------LDPPII-PFMP----------------------------
LLIKDMTFTHEGNK--TF-ID-----------------------------N-LVNFE-KM
-------RM---IANTARTVKFCRS--Q------------------------SFNPD--
-A------------------ALTNKNH-----Q----------------DVRT-----YV
RQLNVIDNQRTL--SQMSHRL
-----------EPRR----------A----------------------------------

>Rapgef4_[Chinchilla_lanigera]_918612485

---MDA-I
TAVPPGPSCFRTPL--I-E--------PHVPLRP--------------------------
------------------A---
-----------------N-TITK------------------VPS----------------
-------EKIL-------------RA---------------------GKILR-NAILSRA
PH-M-----------------IRDRKYH------L-KTY----------------RQC-CV
GT------------------ELVDWMLQQT------------------------------
---PCVH--------SRTQAVGMW---QVLLEDGVLN-----------------------
--------------H------------------------VDQ-EHHFQDKY-LFYRFLDD
-------EHED----AP-L---
--PT

EEEK--KECDEEL------QDTMLLLSQMGPDAH-------------MRMILRKPPGQRT
VDDLE-----IIYEELLHIKALSHLS-TTVKRELA-GVLIFESHAKGGTVL---------
--FNQGEEG--TSWYIIL
KGSVNVVIYGK-----GV-------------------------------VCTLHEG-DDF
GKLAL-----------VNDAPRA---ASIVLREDNCHFLRVDKEDFNR------ILRD--
---V--------------

------------------------EANTVRLKEHDQDVLVLEK-----VPA-GNRASNQG
NS----QP-QQ------------------------------------K-YTVMSGTPEKI
LEHFLET------------IRLEP-ALN--------------EA-----TDSVLN---D-
FIMMHCV--FM---------------PNTQLCPALV-----A------------------
--------H-----------Y-------HAQPSQ---GTEQEKLDYALNNKRRVIRLVLQ
WASTYGDLLQED-DVAMAFLE----EFYVS--VSDDA--RMIAALK---EQ-LPELEKIV
KQI---SED--AK---------APQKK---------------------------------
---HKV--------LLQQFNTSD-ERAQ-KRQ----------------------------

-----PIRGSDE--VL------------------------------FK-------------
VYCMDHTYTTIRVPVAASVKEVIS--------AVADK-----------LGSG-EG--LII
VKMSSG----------------------GEKVV------LKPNDVSVFTTLTINGRLFAC
PREQ---F-DSLT-PLPEQ---------------------------EGP------TVGTV
GT--FELMSSKDLAYQMTLYDWELFNCVHELELIY-------HTFGRHNF-KK-TTANLD
LFLRR---------FNEIQFWV-----VTEICLCSQLSKR------VQLLKKFIKIAAH-
--------------CKEYKNLNSFFAIVM-GLSNVAVSRLALTWE---------------
-------KLPSKFKKFYAE-FESLM--------------------------DPSRNH---
-RAYRL----TAAK-------LEPPLI-PFMP----------------------------
LLIKDMTFTHEGNK--TF-ID-----------------------------N-LVNFE-KM
-------RM---IANTARTVRYYRS--Q-------------------------PFSPD--
-A------------------AQANKNH-----Q----------------DVRS-----YV
RQLNVIDNQRTL---SQMSHRL
-----------EPRR----------P----------------------------------

>Rapgef4_[Chlamydotis_macqueenii]_705667844

-----------------------------VKRELA-GVLIFESHPKAGTVL---------
--FNQGEEG--TSWYIIL
KGSVNVVIYGK-----GV-------------------------------VCTLHEG-DDF
GKLAL-----------VNDAPRA---ASIVLREDNCHFLRVDKEDFNR------ILRD--
---V--------------

------------------------EANTVRLKEHDQDVLVLEK-----IPA-GNRISNQG
NS----QP-QH------------------------------------K-YIVMSGTPEKI
LEHFLET------------MRLEA-TLN--------------EA-----TDSVLN---D-
FIMMHCV--FM---------------PNSQLCPALM-----A------------------
--------H-----------Y-------HAQPSQ---GTEQEKMDYALNNKRRVIRLVLQ
WAALYGDLLQED-EAAMAFLE----EFYVS--VSDDT--RMIAALK---EQ-LPELEKIV
KQV---SEE--PK---------APQKK---------------------------------
---HKV--------LLQLFNTSD-DRAQ-KRQ----------------------------

-----PIRGSDE--VL-----------------------------FK-------------
VYCIDQTYTTIRVPVSSSVKEVIS--------AVADK-----------LGSG-EG--LII
VKMSSG----------------------GEKVV------LKPHDVSVFTTLSVNGRLFAC
PRDQ---F-DSLA-PLPEQ---------------------------EGP------STGTV
GT--FELMSSKDLAHQMTIYDWELFNCVHELELIY-------HTFGRHNF-KK-TTANLD
LFLRR---------FNEIQFWV-----VTEICLCSQLSKR------VQLLKKYIKIAAH-
--------------CKEYKNLNSFFAIIM-GLSNVAVSRLSLTWE---------------
-------KLPSKFKKIYAE-FESLM--------------------------DPSRNH---
-RAYRL----TVAK-------LEPPII-PFMP----------------------------
LLIKDMTFTHEGNK--TF-AD-----------------------------N-LVNFE-KM
-------RM---IANTVRTVKFCRS--Q-------------------------SFNPD--
-A------------------ALTNKNH-----Q----------------DVRS-----YV
RQLNVIDNQRTL---SQMSHRL
-----------EPRR----------A----------------------------------

>Rapgef4_[Chrysochloris_asiatica]_586472254

---MAG-L
LAPPYG--VMETGS--N-N--------DRIPDKE--------------------------
------------------N---
---VPS----------------
-------EKIL-------------RA---------------------GKILR-NAILSRA
PH-M----------------IRDRKYH------L-KTY----------------RQC-CV
GT-----------------ELVDWMTQQT-------------------------------
---QCVH--------SRTQAVGMW---QVLLEDGVLN-----------------------
--------------H------------------------VDQ-EHHFQDKY-LFYRFLDD
-------EQED----AP-L---
--PS
EEEK--KECDEEL------QDTMLLLSQMGPDAH-------------MRMILRKPPGQRT
ADELE-----IIYEELLHIKALSHLS-TTVKRELA-GVLIFESHAKGGTVL---------
--FNQGEEG--TSWYIIL
KGSVNVVIYGK-----GV-------------------------------VCTLHEG-DDF
GKLAL-----------VNDAPRA---ASIVLREDNCHFLRVDKEDFNR------ILRD--
---V--------------

------------------------EANTVRLKEHDQDVLVLEK-----VPA-GNRTSNQG
NS----QP-QQ------------------------------------K-YTVMSGTPEKI
LEHFLET------------IRLEP-ALN--------------EA-----TDSVLN---D-
FVMMHCV--FM---------------PNTQLCPGLV-----A------------------
--------H-----------Y-------HAQPSQ---GSEQEKMDYAVNNKRRVIRLVLQ
WAAMYGDLLQDD-GVAMAFLE----EFYVS--VSDDA--RMIASLK---EQ-LPDLEKIV
KEI---LED--AK---------APQKK---------------------------------
---HKV--------LLQQFNTGD-ERAQ-KRL----------------------------

-----PIRGSDE--VL-----------------------------FK-------------
VYCVDHTYTTIRVPVAASVKEVVS--------AVADK-----------LGSG-EG--LTL
VKMSSG----------------------GEKVV------LKPNDVSVFTTLTINGRLFAC
PREQ---F-DSLT-PLPEQ---------------------------EGP------TVGTV
GT--FELMSSKDLAYQMTIYDWELFNCVHELELIY-------HTFGRHHF-KK-TTANLD
LFLRR---------FNEIQFWV-----VTEICLCSQLSKR------VQLLKKFIKIAAH-
--------------CKEYKNLNSFFAIVM-GLSNVAVSRLALTWE---------------
-------KLPSKFKKFYAE-FESSM--------------------------DPSRNH---
-RAYRL----TVAK-------LEPPLI-PFMP----------------------------
LLIKDMTFTHEGNK--TF-ID-----------------------------N-LVNFE-KM
-------RM---IANTARTVRYCRS--Q-------------------------PFNPD--
-A------------------AQANKNH-----Q----------------DVRS-----YV
RQLNVIDNQRTL---SQMSHRV
-----------EPRR----------P----------------------------------

>Rapgef4_[Cimex_lectularius]_939265221
-------MA---------------------ATEWITAF----------------------
----------------------DKRPSE-----RTCRDVDLICGRLRRIDSLARIPQSLL
NNLAHLAF--YEDLE--KGVTLFRQGEIGTSWYVILTGSVEVKVNQ-----EK------N
I-----VSLCTLSVGSSFGESVLQDS-PRETTVV-TKTSCELLRLSQHDLKALAE-----
KNKD--VMTELI--------------S-NCKMKNGLGGVG-K-TPQ--------SP----
-PPVVQQG---------------K--RTLSPDQTNPAE----------------------

--PITE--------------
-----------------VPSVVMTRA---------------------AWVLR-TLLLNEA
ES-I----------------LRERKTAG-----G-R-L---------------VVPCCAS
GS-----------------ELVTWLVSLA-------------------------------
---PDI---------DRHQGTTMW---QALLEEGLIY-----------------------
--------------H------------------------VTG-EHPFKDKC-VLYQFRQD
R---------D----IT---
--CIPRPP
PQDI--AEAEEHL------HEAIADLSDRGPDAW-------------LRMILRKPSNDRT
PEDLE-----IIYEELLHMKPLSHLS-NSMKRELA-SVIMFEAHPRSGSIV---------
--FEQGEEG--RSWYLVL
NGSVDVVIHGK-----GV-------------------------------VATLHQG-EDF
GKLAL-----------INDVPRA---ASIIVREDGTHLLRVDKEDFNR------ILRD--
---V--------------

------------------------EANTVRLKEHGKDVLVLE--------K---------
------SSSP----NS---------------QYK---------------YVVMACTAMRM
VEHLLDS-------------RLDT-N-----------TAAH---------DPALD---D-
FLLTHVL--FI---------------STRHLVQELR-----K------------------
--------HY----------------------TM---DTPLHDKEQSDGHKRKVILFVYR
WVTTIRHPVLAD-SNAVAFIE----ELASD--CEHEG--------W-----------EES
GLM---
---HRV--------TAQITRHRE---SSTVVNR---------------------------
----WKLPP---SGQPIC--------------------------------LFSPSET---
---GG-T---L---

------IKSHDD--II-----------------------------FR-------------
VYCADHTYCTLRMQVSATAEQIKV--------AAADK-----------LGIRHRPEDLLL
AEVKSN----------------------GERIT------FRDSEVSIPTALTLNGKIFVA
PKEH---L-DALT-IIPEQ---------------------------EIP------TEGLE
L--DLEDVSAKEMAYHLTMFDWELFWNIHEYELLY-------HTFGRHRF--GQNTANLD

VFLRR---------FNELQYWV-----VTEICLTQSLSKR------VHVLRKIIKIASY-
--------------CKEFHNINGMFALVL-GLSNVAVSRLSATWD---------------
-------KLPSKSRKLYTQ-LEATI--------------------------DPSRNH---
-RAYRA----VVTS-------MSSPLI-PFMP----------------------------
LLLKDMTFIHEGNK--TM-VD-----------------------------G-LVNFE-KM
-------HM---LAQTLRTLRYCRN--R------HMVI-----------------DPP--
----------------------TPKNE-----S----------------EVRA-----YV
RCLRTIDNQRTL---NSHSQKL
-----------EPRR----------S----------------------------------

>Rapgef4_[Ciona_intestinalis]_699251985

----------------------------------MLHTEIDNEALTVARIMY-SYLIQTK
PD-I----------------ITEHQSDEEISG-L---------ADDDVV---TYRNC-CF
GH-----------------ELVAWLCGYIKTT--------------------------NL
AL---IS------ELSRFHVNGLW---QVLLEEGLVY-----------------------
--------------H------------------------VRH-EQYFEDTR-YLYQFHNE
V-----TEYAQ--TAEEEI---
---P
DKSKLLEVTEDKMGW------VLAYLNQSSPDTM-------------LRTALRKLPEHRT
SEELE-----LIYDEILHMKALTQLS-DQVKQQLA-AVLQFEFYHKSGETI---------
--FHQGDEG--RSWYIIS
KGSVDVVIQGK-----GV-------------------------------VCTLHEG-DDF
GSLAI-----------VNDAPRA---ATIVTTKPNCQFLRVDKYDFNK------IHEE--
---V--------------

------------------------ESKTVRLKEHGKDVLILQKSLK----T------KEG
GT------SAYV-----------------GSRGQK------------R-YNISAGTAEKI
VNHILES-------------------LQPWVKPRH------EEN---------LE---D-
FLLTFRF--FT---------------TTQKLCDLLL-----QRY------NSEVVNG---
-----------------------------AECSR--------------SLKKRVVYLVVR
WSYISTHLFIQD-LVVQNFLQV----LREN--IAEDIQ-KNLAYLEL---Q--SDLNLLY
KSP---EHDEVGNV--------------------------------------LT------
-----VGE------TIQRYPSPS---SHDEQCA---------------------------


```
------------------------------------------------------------
------------------------------------------------------------
------------------------------------------------------------
------------------------------------------------------------
------IHDNDE--VL-----------------------------LK-------------
IYDLQSSYTTVKVPICLTCEETVN--------AACEKIN-------------RSPNKMLL
FEVQENND------------------------IH----AIPKHQMSVATSTKLNSRLYMC
IPDE---R-DELA-SIQLPP----------------------------P------TIQPT
TC--METVSSLEIAYHVTQHDWQLMNNIH-------------------------------
----------------------------------------------------------LL
---------------KMYGNMFGFMAVMI-GIGNQAISRLHGTWD---------------
-------KLPTKYRKLVDE-FETIL--------------------------DPSRKH---
-RNYRVF----VSS-------TNSARI-PYFP----------------------------
LLMKDLTFQHEGND--TF--N----------------------------KNGLVNFE-KM
-------HM---LSNSIRLVVHGCSRSLDN------QFFE--------------------
----------------------STKTA-----E----------------ESRN-----YT
REVSVITNQILL-----------------------------------------NEMSNKL
-----------EPKRT--------------------------------------------
------------------------------------------------------------
------------------------------------------------------------
------------------------------------------------------------
------------------------------------------------------------
------------------------------------------------------------
------------------------------------------------------------
------------------------------------------------------------
------------------------------------------------------------
------------------------------------------------------------
------------------------------------------------------------
---
>Rapgef4_[Clonorchis_sinensis]_358335756
------------------------------------------------------------
------------------------------------------------------------
------------------------------------------------------------
------------------------------------------------------------
------------------------------------------------------------
------------------------------------------------------------
------------------------------------------------------------
------------------------------------------------------------
------------------------------------------------------------
------------------------------------------------------------
------------------------------------------------------------
-----------------MLASGVW---QALLEESAIQ-----------------------
-------PFDVMGTRKVAD--------------------VPE----FMDSPQEMYRCTCP
LDVSLSLEEDLEAVSTNTLRK---------------------------------------
----------------QEKPADF-----------LGRKSMF-----------SRSHTLST
DADD-AVTAEDRF------QLLIERLYCLASAAL-------------LRRILAKSPNQRT
GVEVE-----FVSQELLMVPALANFP-NSVRNELS-KCIKYEVHPLSNKLV---------
--------------------------------------------FRQGDPG--VCWYIIY
KGSVWVHIKSQ-----GY-------------------------------VCRLYDG-DDF
GKLAL-----------VTGDPRA---ASIVLAENNCHFLRVDKTDYDR------VLVD--
---------------------------------------------V--------------
------------------------------------------------------------
------------------------------------------------------------
------------------------EANIVRLREGDSDVLVLECVKFTCRES---ASSVHG
```

FE--QTATTEPE--NKRL------------TRANN------------Q--LIIAGTIDKI
LRHILDARL--SIYDDQL-----PNLVSRLEKLYRLCPFPQVEI------DIVVE---E-
FILTLTM--FT---------------TNSDVFNLLF-----AYLS-----QD--------
-------------RSNGA-----------GKADQLTSTQNENPEVYQDCFSRRVLILLYL
WCMCIGLLAFASLDGLRKFLSDIK-QLISC-RTNCDLEKKAVEVLLKECDQIWNPTHHDA
RST---TEHDSLKKNNSRK---KPSQKCGVLSGTRAL-----FTRGKQIVKSLP--C---
--------------ALQQLSHYT-HHVQ--RSMYYT------------------------
--------PYH------L--
------NR--

------LRIEPH-SLP-----------------FTPRTHTITFLVYLG-----E------
----DHRKVILTLPVDTTVQRIKSR--------LSSELP-----------SGAEINDYEF
VEVRST----------------------GERIT------YTDGERGILFSLSPNSCLFFV
PRQD---R-AKLV-PLPIQLNVAMSFLDQTDPGRSMTEIQRKESSLSSPKMKKSISVSQH
FS-ALEELSVDEIASVFTFSHIQLAMCITASELLN-------YTMGSEKS--GCATPHIR
LLVAQ---------FSLIHAWT-----ITQLVTTPVLNKR------VQFLKRLIKLADRL
IS-------------SPFHDQHTCFAIVM-ALQSAPISRLTQTWD---------------
-------RLPVRWKRLYQNRLLPLI--------------------------DPARNH---
-RAARN----WIAE-------NIQNCL-PFLA----------------------------
ILLKDLRFAEDGNS--TIYAQQQPSKV-----------------------R-LINFE-KM
-------RL---IARTLHVWNMAISGYEE-------------------------LHPFAK
RMH-------------CPPRELTATGE----LQFIPQTL---------QSISSEVLELFY
DHMDCIDDLQLL---SQLAWRI
-----------EPKK---

>Rapgef4_[Clupea_harengus]_831318321
----------------------------MISPKMV-----A------AH----TSTHSSA
SAEWIV---CLDK-----------RPTE-----RSGEDVDIILTRLKNVKAFERFNPNLL
QEICLCGF--YECLE--KGITLYRQGDIGTSWYAVLSGSLDVKVSE-TANHQDA------
------VTICTLGIGTAFGESILDNT-PRHATIV-TREVSELL-----------------
----------------------------RIEQRE-------FKALW---EKYRQCMAG-L
LAPPYG--VMESG---P-SS-------DRMPDKD--------------------------
------------------NLN-------NNTLGSMSKHLNK-------------------
---VPS----------------
-------EKIL-------------RA---------------------GKVLR-NVILSRV
PH-M----------------IRDRKYH------L-KTY----------------KQC-CV
GT-----------------ELVDWQMQQS-------------------------------
---SCVH--------SRVQAVGMW---QVLLEEGVLN-----------------------
--------------H------------------------VDL-ELNFQDKY-LFYRFLDD
-------EKDG----AP-Q---
--PS

EEEK--KESEDEL------QDTLLLLSQMGPDAH-------------MRMILRKPPGQRT
ADDLE-----IIYEELLHIKALSHLS-TTVKRELA-GVLIFESHAKAGTVL---------
--FNQGEEG--TSWYIIL
KGSVNVVIYGK-----GV-------------------------------VCTLHEG-DDF
GKLAL-----------VNDAPRA---ASIVLREDNCHFLRVDKEDFNR------ILRD--
---V--------------

------------------------EANTVRLKEHDQDVLVLEK-----T---T----GRT
SS-HGASS-HH------------------------------------K-YTVMAGTPEKI
LEHFLET------------MKLDS-QFT--------------ES------DQALD---D-
FVLIHSV--LL---------------PNSQLCPLLM-----A------------------
--------H-----------Y-------HAQASQ---GSEQEQLDFALNNKRRVVRLALQ
WATFHGHHLQEE-EASLAFLE----ELYVA--VSDDG--RLFPALK---EQ-LPVLEMMV
KNN---SDD--SR---------SSQKK---------------------------------
---QKV--------LLRQFSMGD-EKHQ-KRQ----------------------------

-----PIKGNDE--IL-----------------------------FK-------------
VYCIDHTYTTIRVPVSASVREVIS--------AVADK-----------LGSA-ED--LLL
VSLSSA----------------------GDKVV------AKPNDVSVFSTLSVNGRLFVC
PREK---L-DSLT-PLPEQ---------------------------EGP------STGSL
PT--FELMSSKDLAYQMTLCDWELFNCVHELEVVY-------HTFGRQHF-KK-TTANLD
LFLRR---------FNEIQLWV-----ITEVCLCPQLSKR------VQLLKKFIKIAAH-
--------------CKEYRNLNSFFAIIM-GMSNPAVSRLSQTWE---------------
-------KLPSKFKKFYGE-FENLM--------------------------DPSRNH---
-RAYRL----TMAK-------LHPPII-PFMP----------------------------
LLIKDMTFTHEGNK--TY-ID-----------------------------S-LVNFE-KM
-------RM---IANTVRILRYCRS--Q-------------------------PFSVDA-
-S------------------Q-VNKNH-----Q----------------DVRS-----YM
HQLNVIDNQRTL---SQLSHKL
-----------EPRR----------T----------------------------------

>Rapgef4_[Colius_striatus]_706115657

---MSG-L
LTPPYG--VMETGS--N-N--------DRMPDKD--------------------------

```
------------------SMS-------SNALCQV-------------------------
----------------SK-NCNK------------------VPS----------------
-------EKIL-------------RA---------------------GKILR-NTILSRA
PH-M----------------IRDRKYH------L-KTY----------------RQC-CV
GT-----------------ELVDWMTQQS-------------------------------
---PCVH--------SRTQAVGMW---QVLLEEGVLN-----------------------
--------------H------------------------VDQ-EHHFQDKY-LFYRFLDD
-------ERED----AP-L-----------------------------------------
----------------------------------------------------------PT
EEEK--KECDEEL------QDTMLLLSQIGPDAH-------------MRMILRKPPGQRT
VDDLE-----FIYEELLHIKALSHLS-TTVKRELA-GVLIFESHPKAGTVL---------
--------------------------------------------FNQGEEG--TSWYIIL
KGSVNVVIYGK-----GV-------------------------------VCTLHEG-DDF
GKLAL-----------VNDAPRA---ASIVLREDNCHFLRVDKEDFNR------ILRD--
---------------------------------------------V--------------
------------------------------------------------------------
------------------------------------------------------------
------------------------EANTVRLKEHDQDVLVLEK-----IPA-GNRVSNQG
NS----QP-QH------------------------------------K-YIVMSGTPEKI
LEHFLET------------MRLEA-TLN--------------EA-----TDSILN---D-
FIMMHCV--FM---------------PNSQLCPALM-----A------------------
--------H-----------Y-------HAQPSQ---GTEQEKMDYALNNKRRVIWLVLQ
WAALYGDLLQED-EAAMAFLE----EFYVS--VSDDT--RMITALK---EQ-LPELEKIV
KQV---SEE--AK---------GPQKK---------------------------------
---HKV--------LLQLFNTSD-DRAQ-KRQ----------------------------
------------------------------------------------------------
------------------------------------------------------------
------------------------------------------------------------
------------------------------------------------------------
------------------------------------------------------------
------------------------------------------------------------
-----PIRGSDE--VL-----------------------------FK-------------
VYCIDQTYTTIRVPVSSSVKEVIS--------AVADK-----------LGSG-ES--LII
VKMSSG----------------------GEKVV------LKPHDVSVFTTLSVNGRLFAC
PRDQ---F-DSLA-PLPEQ---------------------------EGP------SAGTV
GT--FELMSSKDLAHQMTIYDWELFNCVHELELIY-------HTFGRHNF-KK-TTANLD
LFLRR---------FNEIQFWV-----VTEICLCSQLSKR------VQLLKKYIKIAAH-
--------------CKEYKNLNSFFAIIM-GLSNVAVSRLSLTWE---------------
-------KLPSKFKKIYAE-FESLM--------------------------DPSRNH---
-RAYRL----TVAK-------LDPPII-PFMP----------------------------
LLIKDMTFTHEGNK--TF-TD-----------------------------N-LVNFE-KM
-------RM---IANTVRTVKFCRS--Q-------------------------SFNPD--
-A------------------ALTNKNH-----Q----------------DVRS-----YV
RQLNVIDNQRTL-----------------------------------------SQMSHRL
-----------EPRR----------A----------------------------------
------------------------------------------------------------
------------------------------------------------------------
------------------------------------------------------------
------------------------------------------------------------
------------------------------------------------------------
------------------------------------------------------------
------------------------------------------------------------
------------------------------------------------------------
------------------------------------------------------------
```


>Rapgef4_[Colobus_angolensis_palliatus]_795183836
----------------------------M-------------------------------

--LY---KKYRQYMAG-L
LAPPYG--VMETGS--N-N--------DRIPDKE--------------------------
------------------N----------------------T------------------
-----PLIEPHVPLRPAN-TITK------------------VPS----------------
-------EKIL-------------RA---------------------GKILR-NAILSRA
PH-M----------------IRDRKYH------L-KTY----------------RQC-CV
GT-----------------ELVDWMMQQT-------------------------------
---PCVH--------SRTQAVGMW---QVLLEDGVLN-----------------------
--------------H------------------------VDQ-EHHFQDKY-LFYRFLDD
-------EHED----AP-L---
--PT
EEEK--KECDEEL------QDTMLLLSQMGPDAH-------------MRMILRKPPGQRT
VDDLE-----IIYEELLHIKALSHLS-TTVKRELA-GVLIFESHAKGGTVL---------
--FNQGEEG--TSWYIIL
KGSVNVVIYGK-----GV-------------------------------VCTLHEG-DDF
GKLAL-----------VNDAPRA---ASIVLREDNCHFLRVDKEDFNR------ILRD--
---V--------------

------------------------EANTVRLKEHDQDVLVLEK-----VPA-GNRASNQG
NS----QP-QQ------------------------------------K-YTVMSGTPEKI
LEHFLET------------IRLEA-TLN--------------EA-----TDSVLN---D-
FIMMHCV--FM---------------PNTQLCPALV-----A------------------
--------H-----------Y-------HAQPSQ---GTEQEKMDYALNNKRRVIRLVLQ
WAAMYGDLLQED-DVSMAFLE----EFYVS--VSDDA--RMIAALK---EQ-LPELEKIV
KQI---SED--AK---------APQKK---------------------------------
---HKV--------LLQQFNTGD-ERAQ-KRQ----------------------------

-----PIRGSDE--VL-----------------------------FK-------------
VYCMDHTYTTIRVPVAASVKEVIS--------AVADK-----------LGSG-EG--LII
VKMSSG----------------------GEKVV------LKPNDVSVFTTLTINGRLFAC
PREQ---F-DSLT-PLPEQ---------------------------EGP------TIGTV
GT--FELMSSKDLAYQMTIYDWELFNCMHELELIY-------HTFGRHNF-KK-TTANLD
LFLRR---------FNEIQFWV-----VTEICLCSQLSKR------VQLLKKFIKIAAH-
--------------CKEYKNLNSFFAIVM-GLSNVAVSRLALTWE---------------
-------KLPSKFKKFYAE-FESLM--------------------------DPSRNH---
-RAYRL----TVAK-------LEPPLI-PFMP----------------------------
LLIKDMTFTHEGNK--TF-ID-----------------------------N-LVNFE-KM
-------RM---IANTARTVRYYRS--Q-------------------------PFNPD--
-A------------------AQANKNH-----Q----------------DVRS-----YV
RQLNVIDNQRTL---SQMSHRL
-----------EPRR----------P----------------------------------

>Rapgef4_[Columba_livia]_915555508
----------------------------MVAA-------HT------SH--------SSS
SGEWIA---CLDK-----------RPLE-----RSSEDVDIIFTRLKEVKAFEKFHPNLL
QQICLCGY--YENLE--KGITLFRQGDIGTNWYAVLTGSLDVKVSD-TSNHQDA------
------VTICTLGVGTAFGESILDNT-PRHATIV-TREYSELL-----------------
----------------------------RIEQKD-------FKALW---EKYRQYMSG-L
LTPPYG--VMETGS--N-N--------DRMPDKD--------------------------
------------------SMS-------SSALCQVSKNCNKT------------------
-----PPIEPHIPPRPTK-TITQ------------------VPS----------------
-------EKIL-------------RA---------------------GKILR-NTILSRA
PH-M----------------IRDRKYH------L-KTY----------------RQC-CV
GT-----------------ELVDWMMQQS-------------------------------
---PCVH--------SRSQAVGMW---QVLLEEGVLN-----------------------
--------------H------------------------VDQ-EHYFQDKY-LFYRFLDD
-------EHED----AP-L---
--PT
EEEK--KECDEEL------QDTMLFLSQIGPDAH-------------MRMILRKPPGQRS
VDDLE-----IIYEELLHIKALSHLS-TTVKRELA-GVLIFESHPKAGTVL---------
--FNQGEEG--TSWYIIL
KGSVNVVIYGK-----GV-------------------------------VCTLHEG-DDF
GKLAL-----------VNDAPRA---ASIVLREDNCHFLRVDKEDFNR------ILRD--
---V--------------

------------------------EANTVRLKEHDQDVLVLEK-----IPA-GNRISNQG
NS----QP-QH------------------------------------K-YIVMSGTPEKI
LEHFLET------------MRLEA-TLN--------------EA-----TDSVLN---D-
FIMMHCV--FM---------------PNSQLCPALM-----A------------------
--------H-----------Y-------HAQPSQ---GTEQEKMDYALNNKRRVIRLVLQ
WAALYGDLLQED-EAAMAFLE----EFYVS--VSDDT--RMIAALK---EQ-LPELEKIV
KQV---SEE--PK---------APQKK---------------------------------
---HKV--------LLQLFNTSD-DRAQ-KRQ----------------------------

-----PIRGSDE--VL-----------------------------FK-------------
VYCIDQTYTTIRVPVSSSVKEVIS--------AVADK-----------LGSG-EG--LII
VKMSSG----------------------GEKVV------LKPHDVSVFTTLSVNGRLFAC
PRDQ---F-DSLA-PLPEQ---------------------------EGP------SAGTV
GT--FELMSSKDLAHQMTIYDWELFNCVHELELIY-------HTFGRHNF-KK-TTANLD

```
LFLRR---------FNEIQFWV-----VTEICLCSQLSKR------VQLLKKYIKIAAH-
--------------CKEYKNLNSFFAIIM-GLSNVAVSRLSLTWE---------------
-------KLPSKFKKIYAE-FESLM--------------------------DPSRNH---
-RAYRL----TVAK-------LDPPII-PFMP----------------------------
LLIKDMTFTHEGNK--TF-TD-----------------------------N-LVNFE-KM
-------RM---IANTVRTVKFCRS--Q-------------------------SFNPD--
-A------------------ALTNKNH-----Q----------------DVRS-----YV
RQLNVIDNQRTL-----------------------------------------SQMSHRL
-----------EPRR----------A----------------------------------
------------------------------------------------------------
------------------------------------------------------------
------------------------------------------------------------
------------------------------------------------------------
------------------------------------------------------------
------------------------------------------------------------
------------------------------------------------------------
------------------------------------------------------------
------------------------------------------------------------
------------------------------------------------------------
---
>Rapgef4_[Condylura_cristata]_829983197
----------------------------M-------------------------------
------------------------------------------------------------
------------------------------------------------------------
------------------------------------------------------------
--------------------------------------------DI-------------I
TAVHPG------------------------PSSF--------------------------
------------------R-----------------------------------------
-----------------------------------------VPS----------------
-------EKIL-------------RA---------------------GKILR-NAILSRA
PH-M----------------IRDRKYH------L-KTY----------------RQC-CV
GT-----------------ELVDWMMQQT-------------------------------
---PCVH--------SRTQSVGMW---QVLVEDGVLN-----------------------
--------------H------------------------VDQ-EHHFQDKY-LFYRFLDD
-------EHED----AP-L-----------------------------------------
----------------------------------------------------------PT
EEEK--KECDEEL------QDTMLLLSQMGPDAH-------------MRMILRKPPGQRT
VDDLE-----IIYEELLHIKALSHLS-TTVKRELA-GVLIFESHAKGGTVL---------
--------------------------------------------FNQGEEG--TSWYIIL
KGSVNVVIYGK-----GV-------------------------------VCTLHEG-DDF
GKLAL-----------VNDAPRA---ASIVLREDNCHFLRVDKEDFNR------ILRD--
---------------------------------------------V--------------
------------------------------------------------------------
------------------------------------------------------------
------------------------EANTVRLKEHDQDVLVLEK-----VPA-GNRASNQG
NS----QP-QQ------------------------------------K-YTVMSGTPEKI
LEHFLET------------IRLEP-TLN--------------EA-----TDSILN---D-
FVMMHCV--FM---------------PNTQLCPALV-----A------------------
--------H-----------Y-------HAQPSQ---GTEQEKMDYALNNKRRVIRLVLQ
WAAMYGDLLQDD-DVALAFLE----EFYVS--VSDDA--RMIAALK---EQ-VPELEKIV
KQI---SED--AK---------APQKK---------------------------------
---HKV--------LLQQFNTSD-ERAQ-KRQ----------------------------
------------------------------------------------------------
------------------------------------------------------------
```


-----PVRGSDE--VL-----------------------------FK-------------
VYCLDHTYTTIRVPVAASVKEVIS--------AVADK-----------LGSG-EG--LVI
VKMSSG----------------------GEKVV------LKPNDVSVFTTLTINGRLFAC
PREQ---F-DSLT-PLPEQ---------------------------EGP------TVGTV
GT--FELMSSKDLAYQMTIYDWELFNCVHELELIY-------HTFGRHNF-KR-TTANLD
LFLRR---------FNEIQFWV-----VTEICLCSQLSKR------VQLLKKFIKIAAH-
--------------CKEYKNLNSFFAIVM-GLSNVAVSRLALTWE---------------
-------KLPSKFKKFYAE-FESLM--------------------------DPSRNH---
-RTYRL----TVAK-------LEPPLI-PFMP----------------------------
LLIKDMTFTHEGNK--TF-ID-----------------------------N-LVNFE-KM
-------RM---IANTARTVRYYRS--Q-------------------------PFNPD--
-A------------------AQANKNH-----Q----------------DVRS-----YV
RQLNVIDNQRTL---SQMSHRL
-----------EPRR----------P----------------------------------

>Rapgef4_[Corvus_brachyrhynchos]_669280985

---MSG-L
LTPPYG--VMETGS--N-N--------DRMPDKD--------------------------
------------------N----------------------T------------------
-----PLIDPHIPHHPTK-TITQ------------------VPS----------------
-------EKIL-------------RA---------------------GKILR-NTILSRA
PH-M----------------IRDRKYH------L-KTY----------------RQC-CV
GT-----------------ELVDWMMQQS-------------------------------
---PCVH--------LRTQAVGMW---QVLLEEGVLN-----------------------
--------------H------------------------VDQ-EHHFQDKY-LFYRFLDD
-------ERED----AP-L---
--PT
EEEK--KECDEEL------QDTMLLLSQIGPDAH-------------MRMILRKPPGQRT
VDDLE-----FIYEELLHIKALSHLS-TTVKRELA-GVLIFESHPKAGTVL---------
--FNQGEEG--TSWYIIL
KGSVNVVIYGK-----GV-------------------------------VCTLHEG-DDF
GKLAL-----------VNDAPRA---ASIVLREDNCHFLRVDKEDFNR------ILRD--
---V--------------

------------------------EANTVRLKEHDQDVLVLEK-----IPA-GNRISNQG

NS----QP-QH------------------------------------K-YIVMSGTPEKI
LEHFLET------------MRLEA-TLS--------------EA-----TDSVLN---D-
FIMMHCV--FM---------------PNSQLCPALM-----A------------------
--------H-----------Y-------HAQPSQ---GTEQEKMDYALNNKRRVIRLVLQ
WAALYGDLLQED-EAAMAFLE----EFYVS--VSDDT--RMIAALK---EQ-LPELEKIV
KQI---SEE--PK---------ASQKK---------------------------------
---HKV--------LLQLFNTSD-DRAQ-KRQ----------------------------

-----PIRGSDE--VL-----------------------------FK-------------
VYCIDQTYTTIRVPVSSSVKEVIS--------AVADK-----------LGSG-EG--LII
VKMSSG----------------------GEKVV------LKPHDVSVFTTLSVNGRLFAC
PRDQ---F-DSLA-PLPEQ---------------------------EGP------STGTV
GT--FELMSSKDLAHQMTVYDWELFNCVHELELIY-------HTFGRHNF-KK-TTANLD
LFLRR---------FNEIQFWV-----VTEICLCSQLSKR------VQLLKKYIKIAAH-
--------------CKEYKNLNSFFAIIM-GLSNVAVSRLSLTWE---------------
-------KLPSKFKKIYAE-FESLM--------------------------DPSRNH---
-RAYRL----TVAK-------LDPPII-PFMP----------------------------
LLIKDMTFTHEGNK--TF-TD-----------------------------N-LVNFE-KM
-------RM---IANTVRTVKFCRS--Q-------------------------SFNPD--
-A------------------ALTNKNH-----Q----------------DVRS-----YV
RQLNVIDNQRTL---SQMSHRL
-----------EPRR----------A----------------------------------

>Rapgef4_[Corvus_cornix_cornix]_727051291
----------------------------MWT-----------------------------
---W---------------------PLE-----RSSEDVDIIFTRLKEVKAFEKFHPNLL
QQICLCGY--YENLE--KGITLFRQGDIGTNWYAVLTGSLDVKVSD-TSNHQDA------
------VTICTLGIGTAFGESILDNT-PRHATIV-TREYSELL-----------------
----------------------------RIEQKD-------FKALW---EKYRQYMSG-L
LTPPYG--VMETGS--N-N--------DRMPDKD--------------------------
------------------SMS-------SSALCQVSKNCNKT------------------
-----PLIDPHIPHHPTK-TITQ------------------VPS----------------
-------EKIL-------------RA---------------------GKILR-NTILSRA
PH-M----------------IRDRKYH------L-KTY----------------RQC-CV
GT-----------------ELVDWMMQQS-------------------------------
---PCVH--------LRTQAVGMW---QVLLEEGVLN-----------------------
--------------H------------------------VDQ-EHHFQDKY-LFYRFLDD
-------ERED----AP-L---
--PT

EEEK--KECDEEL------QDTMLLLSQIGPDAH-------------MRMILRKPPGQRT
VDDLE-----FIYEELLHIKALSHLS-TTVKRELA-GVLIFESHPKAGTVL---------
--FNQGEEG--TSWYIIL
KGSVNVVIYGK-----GV-------------------------------VCTLHEG-DDF
GKLAL-----------VNDAPRA---ASIVLREDNCHFLRVDKEDFNR------ILRD--
---V--------------

------------------------EANTVRLKEHDQDVLVLEK-----IPA-GNRISNQG
NS----QP-QH------------------------------------K-YIVMSGTPEKI
LEHFLET------------MRLEA-TLS--------------EA-----TDSVLN---D-
FIMMHCV--FM---------------PNSQLCPALM-----A------------------
--------H-----------Y-------HAQPSQ---GTEQEKMDYALNNKRRVIRLVLQ
WAALYGDLLQED-EAAMAFLE----EFYVS--VSDDT--RMIAALK---EQ-LPELEKIV
KQI---SEE--PK---------ASQKK---------------------------------
---HKV--------LLQLFNTSD-DRAQ-KRQ----------------------------

-----PIRGSDE--VL-----------------------------FK-------------
VYCIDQTYTTIRVPVSSSVKEVIS--------AVADK-----------LGSG-EG--LII
VKMSSG----------------------GEKVV------LKPHDVSVFTTLSVNGRLFAC
PRDQ---F-DSLA-PLPEQ---------------------------EGP------STGTV
GT--FELMSSKDLAHQMTVYDWELFNCVHELELIY-------HTFGRHNF-KK-TTANLD
LFLRR---------FNEIQFWV-----VTEICLCSQLSKR------VQLLKKYIKIAAH-
--------------CKEYKNLNSFFAIIM-GLSNVAVSRLSLTWE---------------
-------KLPSKFKKIYAE-FESLM--------------------------DPSRNH---
-RAYRL----TVAK-------LDPPII-PFMP----------------------------
LLIKDMTFTHEGNK--TF-TD-----------------------------N-LVNFE-KM
-------RM---IANTVRTVKFCRS--Q-------------------------SFNPD--
-A------------------ALTNKNH-----Q----------------DVRS-----YV
RQLNVIDNQRTL---SQMSHRL
-----------EPRR----------A----------------------------------

>Rapgef4_[Coturnix_japonica]_1003923803

---MSG-L
LTPPYG--VMETGS--N-N--------DRMPDKD--------------------------

```
------------------SMS-------SGALCQVSKNCNKT------------------
-----PLIDSHIPHRPSK-TITQ------------------VPS----------------
-------EKIL-------------RA---------------------GKILR-NTILSRA
PH-M----------------IRDRKYH------L-KTY----------------RQC-CV
GT-----------------ELVDWMMQQS-------------------------------
---TCVH--------SRSQAVGMW---QVLLEEGVLN-----------------------
--------------H------------------------VDQ-EHHFQDKY-LFYRFLDD
-------ERED----AP-L-----------------------------------------
----------------------------------------------------------PT
EEEK--KECDEEL------QDTMLLLSQIGPDAH-------------MRMILRKPPGQRS
MDDLE-----IIYEELLHIKALSHLS-TTVKRELA-GVLIFESHPKAGTVL---------
--------------------------------------------FNQGEEG--TSWYIIL
KGSVNVVIYGK-----GV-------------------------------VCTLHEG-DDF
GKLAL-----------VNDAPRA---ASIVLREDNCHFLRVDKEDFNR------ILRD--
---------------------------------------------V--------------
------------------------------------------------------------
------------------------------------------------------------
------------------------EANTVRLKEHDQDVLVLEK-----IPA-GNRVSNQG
SS----QP-QH------------------------------------K-YIVMSGTPEKI
LEHFLET------------MRLEA-TLN--------------EA-----TDSALN---D-
FIMMHCV--FM---------------PNSQLCPALM-----A------------------
--------H-----------Y-------HAQPSQ---GTEQEKMDYALNNKRRVIRLVLQ
WAALYGDLLQED-EAAMAFLE----EFYVS--VSDDT--RMIAALK---EQ-LPELEKIV
KQV---AEE--PK---------GPQKK---------------------------------
---HKV--------LLQLFNTSD-DRAQ-KRQ----------------------------
------------------------------------------------------------
------------------------------------------------------------
------------------------------------------------------------
------------------------------------------------------------
------------------------------------------------------------
------------------------------------------------------------
-----PIRGSDE--VL-----------------------------FK-------------
VYCIDQTYTTIRVPVSSSVKEVIS--------AVADK-----------LGSG-EG--LII
VKMSSG----------------------GEKVV------LKPHDVSVFTTLSVNGRLFAC
PRDQ---F-DSLA-PLPEQ---------------------------EGP------SAGTV
GT--FELMSSKDLAHQMTIYDWELFNCVHELELIY-------HTFGRHNF-KK-TTANLD
LFLRR---------FNEIQFWV-----VTEICLCSQLSKR------VQILKKYIKIAAH-
--------------CKEYKNLNSFFAIIM-GLSNVAVSRLSLTWE---------------
-------KLPSKFKKIYAE-FESLM--------------------------DPSRNH---
-RAYRL----TVAK-------LDPPII-PFMP----------------------------
LLIKDMTFTHEGNK--TL-TD-----------------------------N-LVNFE-KM
-------RM---IANTVRTVKFCRS--Q-------------------------SFNPD--
-A------------------ALANKNH-----Q----------------DVRS-----YV
RQLNVIDNQRTL-----------------------------------------SQMSHRL
-----------EPRR----------A----------------------------------
------------------------------------------------------------
------------------------------------------------------------
------------------------------------------------------------
------------------------------------------------------------
------------------------------------------------------------
------------------------------------------------------------
------------------------------------------------------------
------------------------------------------------------------
------------------------------------------------------------
```

```
------------------------------------------------------------
---
>Rapgef4_[Crassostrea_gigas]_762156185
----------------------------MMVSE---------------------------
---WIAS---LDK-----------RPCD-----RTGEELDLIYSHLKNLKAFEKFHPSLL
QQICVYGY--YEDLD--KGVTLFRQGDIGTNWYIVLTGSLEVLVSE-TGDHKDA------
------IIVSTLAPGTCFGESILTNK-PRYGTVT-TREFTELIRVEQKDFKILWEGS---
--KK--LLEGVIT----PLERLST-TA---------------------------------
------------------------------------------------------------
-----------------------------------NQLQY--------------------
-----------SPAISRQ-GQGRRKSSA----VARLNGQSNPAAP---------IT----
---SIPSEKLK-------------RA---------------------GRVLR-TVLLTRA
PH-M----------------VRDRKYH------L-RTY----------------RKC-MV
GT-----------------EMVEWLLQQS-------------------------------
---PIVH--------SRNQAVGIW---QALCEEGIIV-----------------------
--------------H------------------------VCR-EHQFKDKY-LFYRFCED
-------DQGV----GT-V-----------------------------------------
----------------------------------------------------------PN
HAQK--KECEEEL------QDTLITLAQIGPDAM-------------MRMILRKLPHDRT
LDDLE-----IIYEELLHIKALSHLS-TSVKRELA-SVLVFESHAKAGTVL---------
--------------------------------------------FNQGDEG--KSWYIIL
KGSVNVVIYGK-----GA-------------------------------VCTLHEG-DDF
GKLAL-----------LNDAPRA---ATIVLREDNCHFLRVDKEDFNR------ILRD--
---------------------------------------------V--------------
------------------------------------------------------------
------------------------------------------------------------
------------------------EANTVRLKEHGQDVLVLEK-----IPT-KSQA-ADG
TM----QS-HY------------------------------------K-YSVMAGTPEKM
LEHLLETRLDNTKSEETT--------------------------------DSFLE---D-
FLLTHVI--FM---------------PTEKLCPALL-----S------------------
--------YYDAKSLKTDEQDAG--DY------G-------------LTQKKSVVQFVQE
WHNLAEDIFNED-EQVQVFLQ----ELHSY--VDEDC--ASFPCLA---SE-LTVLEGMM
SCE-KGFQFINTP------------EV---------------------------------
---GKRRISPSVMLTKKMANELYLDSIN-KKQPIKANDENIV------------------
------------------------------------------------------------
------------------------------------------------------------
------------------------------------------------------------
------------------------------------------------------------
------------------------------------------------------------
------------------------------------------------------------
----------------------------------------------K-------------
IYCADHTYSTLRLPMSSSVHSLIE--------HAREK-----------LGLGSEE--LVL
CEIKSS----------------------GDRVL------YKEDDLCVTTGLSLNGLLFIS
PRKH---L-DALV-SESTQ---------------------------NQC------LPASL
SH--LIAVQNSDCKLPREI---DLFYDSCQYELIY-------QVLGRSNF-NK-ITANLD
LFLRR---------FNEVQYWV-----VTEMVLAQNVGKR------VQLLRKFIKVAAH-
--------------CKEFQNLHSFFAIVM-GLSNIAVSRLSQTWE---------------
-------KLPGKFKKMFAD-FETLM--------------------------DPSRNH---
-RVYRL----SVSK-------LTPPII-PFMP----------------------------
LLMKDLTFTHDGNK--TY-FD-----------------------------G-LVNFE-KM
-------HM---IAQTIRNVRICRS--R-------------------------RLDLEP-
-P------------------NT-AKSS-----T----------------EVQD-----YI
RNLQVIDNQRVL-----------------------------------------TQLSYKL
-----------EPRR----------T----------------------------------
```

```
------------------------------------------------------------
------------------------------------------------------------
------------------------------------------------------------
------------------------------------------------------------
------------------------------------------------------------
------------------------------------------------------------
------------------------------------------------------------
------------------------------------------------------------
------------------------------------------------------------
------------------------------------------------------------
---
>Rapgef4_[Cricetulus_griseus]_344239574
------------------------------------------------------MSCGLF
FTKTLQVL-CLLPTPPLTPC----RPLE-----RSSEDVDIIFTRLKEVKAFEKFHPNLL
HQICLCGY--YESLE--KGITLFRQGDIGTNWYAVLAGSLDVKVSE-TSSHQDA------
------VTICTLGIGTAFGESILDNT-PRHATIV-TRESSELL-----------------
----------------------------RIEQED-------FKALW---E----------
------------------------------------------------------------
------------------------------------------------------------
-----------------------------------------VPS----------------
-------EKIL-------------RA---------------------GKILR-IAILSRA
PH-M----------------IRDRKYH------L-KTY----------------RQC-CV
GT-----------------ELVDWMIQQT-------------------------------
---SCVH--------SRTQAVGMW---QVLLEDGVLN-----------------------
--------------H------------------------VDQ-ERHFQDKY-LFYRFLDD
-------EHED----AP-L-----------------------------------------
----------------------------------------------------------PT
EEEK--KECDEEL------QDTMLLLSQMGPDAH-------------MRMILRKPPGQRT
VDDLE-----IIYDELLHIKALSHLS-TTVKRELA-GVLIFESHAKGGTVL---------
--------------------------------------------FNQGEEG--TSWYIIL
KGSVNVVIYGK-----GV-------------------------------VCTLHEG-DDF
GKLAL-----------VNDAPRA---ASIVLREDNCHFLRVDKEDFNR------ILR---
------------------------------------------------------------
------------------------------------------------------------
------------------------------------------------------------
------------------------------------------------------------
------------------------------------------------------------
------------------------------------------------------------
------------------------------------------------------------
-------------------------------PSQ---GTEQERMDYALNNKRRVIRLVLQ
WAAMYGDLLQED-DVAMAFLE----EFYVS--VSDDA--RMMVAFK---EQ-LPELEKIV
KQI---SED--VK---------APQKK---------------------------------
---HKV--------LLQQFNTGD-ERAQ-KRQ----------------------------
------------------------------------------------------------
------------------------------------------------------------
------------------------------------------------------------
------------------------------------------------------------
------------------------------------------------------------
------------------------------------------------------------
-----PIRGSDE--VL-----------------------------FK-------------
VYCMDHTYTTIRVPVAASVKEVIS--------AVADK-----------LGSG-EG--LII
VKMNSG----------------------GEKVV------LKPNDVSVFTTLTINGRLFAC
PREQ---F-DSLT-PLPEQ---------------------------EGP------TTGTV
GT--FELMSSKDLAYQMTTYDWELFNCVHELELIY-------HTFGRHNF-KK-TTANLD
```

LFLRR---------FNEIQFWV-----VTEICLCSQLSKR------VQLLKKFIKIAAH-
--------------CKEYKNLNSFFAIIM-GLSNVAVSRLALTWE---------------
-------QRSTCLCFPSAG-IKGVHYYHPA------------------------------

>Rapgef4_[Cuculus_canorus]_696966096

---MSG-L
LTPPYG--VMETGS--N-N--------DRMPDKD--------------------------
------------------SMS-------SSALCQV-------------------------
----------------SK-NCNK------------------VPS----------------
-------EKIL-------------RA---------------------GKILR-NTILSRA
PH-M----------------IRDRKYH------L-KTY----------------RQC-CV
GT-----------------ELVDWMMQQS-------------------------------
---PCVH--------SRTQAVGMW---QVLVEEGVLN-----------------------
--------------H------------------------VDQ-EHHFQDKY-LFYRFLDD
-------ERED----AP-L---
--PT
EEEK--KECDEEI------QDTMLLLSQIGPDAH-------------MRMILRKPPGQRT
VDDLE-----IIYEELLHIKALSHLS-TTVKRELA-GVLIFESHPKAGTVL---------
--FNQGEEG--TSWYIIL
KGSVNVVIYGK-----GV-------------------------------VCTLHEG-DDF
GKLAL-----------VNDAPRA---ASIVLREDNCHFLRVDKEDFNR------ILRD--
---V--------------

------------------------EANTVRLKEHDQDVLVLEK-----IPA-GNRVSNQG
NS----QP-QH------------------------------------K-YIVMSGTPEKI
LEHFLET------------MRLDT-TLN--------------EA-----TDSGLN---D-
FIMMHCV--FM---------------PNIQLCPALM-----A------------------
--------H-----------Y-------HAQPSQ---GTEQEKMDYALNNKRRVIRLVLQ
WAALYGDLLQED-EAAMAFLE----EFYVS--VSDDT--RMIAALK---EQ-LPELEKIV
KQV---SEE--PK---------ASQKK---------------------------------
---HKV--------LLQLFNTND-DRAQ-KRL----------------------------

-----PIRGSDE--VL-----------------------------FK-------------
VYCIDQTYTTIRVPVSSSVKEVIS--------AVADK-----------LGSG-EG--LII
VKMSSG----------------------GEKVV------LKPHDVSVFTTLTVNGRLFAC
PRDQ---F-DSLT-PLPEQ---------------------------EGP------STGTV
GT--FELMSSKDLAHQMTIYDWELFNCMHELELIY-------HTFGRHNF-KK-TTANLD
LFLRR---------FNEIQFWV-----VTEICLCSQLSKR------VQLLKKYIKIAAH-
--------------CKEYKNLNSFFAIIM-GLSNVAVSRLSLTWE---------------
-------KLPSKFKKIYAE-FESLM--------------------------DPSRNH---
-RAYRL----TVAK-------LEPPII-PFMP----------------------------
LLIKDMTFTHEGNK--TF-TD-----------------------------N-LVNFE-KM
-------RM---IANTVRTVKFCRS--Q-------------------------SFNPD--
-A------------------ALTNKNH-----Q----------------DVRS-----YV
RQLNVIDNQRTL---SQMSHRL
-----------EPRR----------A----------------------------------

>Rapgef4_[Cynoglossus_semilaevis]_1035265328

---------MESGS----NN-------DRLTDKD--------------------------
------------------NIH-------S-------------------------------
--------DSA---TKAH-----N----------------KIPS----------------
-------EKLQ-------------RA---------------------GKVVR-NAILSRA
PH-M----------------IRDRKYH------L-KTY----------------RQC-CV
GT-----------------ELVDWLVLQS-------------------------------
---ACVL--------TRSHAVGMW---QALVEEGGLN-----------------------
--------------H------------------------VDQ-ELGFQDKY-LFYRFLDD
-------DDED----TP-L---
--PS
EEEK--RESEEEL------PETILFLAQIGPDAL-------------LRMILRKPPGQRT
GDDLE-----IIYDELLHIKALAHLS-NTVKRELA-SVVIFESHAKAGTVL---------
--FNQGEEG--TSWYIIQ
KGSVNVVIYGK-----GV-------------------------------VCTLHEG-DDF
GKLAL-----------VTDSPRA---ASIVLREDNCHFLRVDKEDFNR------ILRD--
---V--------------

------------------------EANTVRLKEHDQAVLVLEK-----S----PRASTLG

S---------I------------------------------------K-YTVISGTPEKI
LDHFLET------------MRVDI-HHS--------------EP------DPAVD---D-
FVLMHCV--FM---------------ANSQFCPLLM-----A------------------
--------H-----------Y-------HAVSPP---GSEQERLEYALNNKRRALILALR
WANTHSYLLQEE-PAALSFLE----ELYGS--LSNDS--RVLRALK---DF-VPDLEKIV
QLH---SEE--AK---------ATKKK---------------------------------
-----T--------LIRQFSNGE-ERLQ-KKQ----------------------------

-----PIRNQDD--IL-----------------------------LK-------------
VYCSDHTYTTIRIAVAATGREVIN--------SVADK-----------LGTS-DE--LML
IHLSSA----------------------GEKQM------LKPNDVSVFSSLNINGRLFAC
SRDQ---L-NSLS-PLPDQ---------------------------EGP------SAGSM
ST--FELMSSKDLAYQMTMFDWELFSCVHEHELLY-------HTFGRQSF-RR-TTANLD
LFLRR---------FNQVQLWV-----VTEVCLCGQLSKR------VQLLKKFIKIAAH-
--------------CREFKNLNSFFAIIM-GMSNPAVSRLSQTWE---------------
-------KLPTKFKKFYAE-FESMM--------------------------DPSRNH---
-RSYRL----TVTK-------LEPPII-PFTP----------------------------
LLLKDMTFTHEGNK--TF-ID-----------------------------N-MVNFE-KM
-------RM---IANTIRQVRHCRG--Q-------------------------PFNPD--
-I------------------CQPNKNQ-----A----------------EVRG-----YV
RKLCVIDNQRAL---TQLSYRL
-----------EPRR----------T----------------------------------

>Rapgef4_[Cyprinodon_variegatus]_974068430

---MAG-L
LVPPYG--VME--S--S-GS-------DRMPEKE--------------------------
------------------NIS-------NSSFVSMSSHPNKK------------------
-----SFHESP---AKLQ-NKSPS----------------QVPS----------------
-------EKIL-------------RA---------------------GKVLR-NAILSRA
PH-M----------------IRDRKYH------L-KTY----------------RQC-CV
GT-----------------ELVDWQMLQS-------------------------------
---SCVH--------SRLQAVGMW---QVLLEEGVLN-----------------------
--------------H------------------------VDG-ETSFQDKY-LFYRFLDD
-------EQED----AL-I---
--PS

DDDM--RESEGEL------QDTLLFLSQIGPDAL-------------MRMILRKPPSERS
PDDLE-----IIYEELLHIKALSHLS-TTVKRELA-GVLIFESHERAGTVL---------
--FHQGEEG--TSWYIIL
KGSVNVVIYGK-----GV-------------------------------VCTLHEG-DDF
GKLAL-----------VNDAPRA---ASIVLREDNCHFLRVDKEDFNR------ILRD--
---V--------------

------------------------EANTVRLKEHGQDVLVLEK-----SLS-SSRNSVHG
SS----AA-HH------------------------------------K-YNIISGTPEKI
LEHLLEM------------MRLDS-QFT--------------ES------DSALD---D-
FVLTHCV--FI---------------PNSQLCPVLR-----D------------------
--------H-----------Y-------HAQASQ---GSELEKLDYTLNNKRRVIRLLMK
WASVHGEHLQQE-DMV--FLE----EFVLA--VSEDA--KVLPDLR---DQ-LTELERIT
KSS---AED--AR---------SAQRK---------------------------------
---HKV--------LLQHFNLGD-EKPQ-RRQ----------------------------

-----PIKGDDE--IL-----------------------------FK-------------
VFCCDHTYTTIRVSVASSVGEVLE--------AVADK-----------LGSA-ED--LIL
VGLSSA----------------------GEKVV------FKPNDVSVFSTLSINGRLFAC
QRDQ---L-DVLM-PLPEQ---------------------------EGP------SVGSL
AN--FELMSSKDVAYHMTFYDWELFHCVHELELIY-------HTFGRQNV-NK-TTVNLD
LFLQR---------FNEIQFWV-----ITEVCLCSQLNKR------VQLLKKFIKIAAH-
--------------CKEYKNLNAFFAIIM-GLSNPAVSRLSQTWE---------------
-------KLPSKFKKFFGE-FENLM--------------------------DPSRNH---
-RAYRL----TVAK-------LDPPVI-PFMP----------------------------
LMIKDMTFTHEGNK--TF-ID-----------------------------N-LVNFE-KM
-------RM---MAKTVKTMRYCRS--Q-------------------------TFSPE--
-S------------------PLAGKHH-----P----------------DVWT-----YV
RQLSVIDNQRTL---SQLSHSL
-----------EPRR----------S----------------------------------

>Rapgef4_[Danio_rerio]_688569964

---MAG-L
LVPPYG--VMDSG---P-NA-------DRMPDKE--------------------------

```
------------------NLS-------NSSPGLSPKHLNKK------------------
-----SFIENPIKVHP-K-PINQ------------------VPS----------------
-------EKIL-------------RA---------------------GKVLR-NIILSRA
PH-M----------------IRDRKYH------L-KTY----------------RRS-CI
GT-----------------ELVDWLLQHT-------------------------------
---SCVH--------SRVQAVGMW---QVLLEEGVLN-----------------------
--------------H------------------------VDQ-EPTFQDKY-LFYRFLDD
-------ESED----SG-M-----------------------------------------
----------------------------------------------------------PS
EEDK--KESDEEL------QDTLLLLSQIGPDAH-------------LRMILRKQPGQRT
VDDLE-----IIYEELLHIKALSHLS-TTVKRELA-GVLIFESHAKAGTVL---------
--------------------------------------------FNQGEEG--TSWYIIL
KGSVNVVIYGK-----GV-------------------------------VCTLHEG-DDF
GKLAL-----------VNDAPRA---ASIVLREDFCHFLRVDKEDFNR------ILRD--
---------------------------------------------V--------------
------------------------------------------------------------
------------------------------------------------------------
------------------------EANTVRLKEHEEDVLVLEK-----A---N----NRS
SS----SS-PL------------------------------------K-YTVMSGTPEKI
LEHLLET------------MKLDC-QFS--------------ES------DPALD---D-
FLLMHCV--FV---------------PNSQLCPLLM-----T------------------
--------H-----------Y-------HAQASQ---GSEQERMDYTLNNKRRVVRLVLQ
WASINTDHLQEE-DSSFSFIQ----EFYEM--VCEDS--RLTPALK---DH-LPELEKII
KQN---SDD--GR---------PSQKK---------------------------------
---HKV--------LLRQFSTGN-DRLQ-KRQ----------------------------
------------------------------------------------------------
------------------------------------------------------------
------------------------------------------------------------
------------------------------------------------------------
------------------------------------------------------------
------------------------------------------------------------
-----PIKSTDE--IL-----------------------------FK-------------
VYCSDHTYSTIRVPVSASVREVIA--------AVSDK-----------LSSG-DD--LLL
IHLNSA----------------------GDKEL------LKPSDVSIFSSLSINGRLFVC
PRDQ---I-DSLT-PLPEQ---------------------------EGP------SAGSM
ST--FELMSSKDLAHQMTLYDWELFHCVHEHELIY-------HTFGRHHF-KK-TTANLD
LFLRR---------FNEVQLWV-----VTEVCLCPALSKR------VQLLKKFIKIAAH-
--------------CKEFKNLNSFFAIIM-GLGNPAVCRLSQTWE---------------
-------KLPSKFKKFYGE-FENLM--------------------------DPSRNH---
-RAYRL----TMAK-------LEPPII-PFMP----------------------------
LLIKDMTFTHEGNK--TF-TD-----------------------------R-LVNFE-KM
-------RM---IANTVRIIRYCRS--Q-------------------------PFDQEA-
-P------------------QATGKSH-----Q----------------DVRT-----YV
RHISVIDNQRSL-----------------------------------------SQLSHRL
-----------EPRR----------T----------------------------------
------------------------------------------------------------
------------------------------------------------------------
------------------------------------------------------------
------------------------------------------------------------
------------------------------------------------------------
------------------------------------------------------------
------------------------------------------------------------
------------------------------------------------------------
------------------------------------------------------------
```


>Rapgef4_[Dasypus_novemcinctus]_488582878
----------------------------MVAA-------HA------AH--------SSS
SAEWIA---CLDK-----------RPLE-----RSSEDVDIIFTRLKEVKAFEKFHPNLL
HQICLCGY--YENLE--KGITLFRQGDIGTNWYAVLAGSLDVKVSE-TSSYQDA------
------VTICTLGIGTAFGESILDNT-PRHATIV-TRECSELL-----------------
----------------------------RIEQKD-------FKALW---EKYRQYMAG-L
LAPPYG--VMETGS--N-N--------DRIPDKE--------------------------
------------------NVS-------NYSLCLPSKNSHKT------------------
-----PLIEPHVPLRPAN-TITK------------------VPS----------------
-------EKIL-------------RA---------------------GKILR-NAILSRA
PH-M----------------IRDRKYH------L-KTY----------------RQC-CV
GT-----------------ELVDWMMQQT-------------------------------
---PCVH--------SRTQAVGMW---QVLLEDGVLN-----------------------
--------------H------------------------VDQ-EHHFQDKY-LFYRFLDD
-------EHED----AP-L---
--PT
EEEK--KECDEEL------QDTMLLLSQMGPDAH-------------MRMILRKPPGQRT
VDDLE-----IIYEELLHIKALSHLS-TTVKRELA-GVLIFESHAKGGTVL---------
--FNQGEEG--TSWYIIL
KGSVNVVIYGK-----GV-------------------------------VCTLHEG-DDF
GKLAL-----------VNDAPRA---ASIVLREDNCHFLRVDKEDFNR------ILRD--
---V--------------

------------------------EANTVRLKEHDQDVLVLEK-----VPA-GNRASNQG
NS----QP-QQ------------------------------------K-YTVMSGTPEKI
LEHFLET------------IRLEP-ALN--------------EA-----TDSVLN---D-
FVMMHCV--FM---------------PNTQLCPALV-----A------------------
--------H-----------Y-------HAQPSQ---GTEQEKMDYALNSKRRVIRLVLQ
WAAVYGDLLQED-DVAMAFLE----EFYVS--VSDDA--RMVAALK---EQ-LPELEKIV
KQI---SED--AK---------APQKK---------------------------------
---HKV--------LLQQFNTGD-ERAQ-KRQ----------------------------

-----PIWGSDE--VL-----------------------------FK-------------
VYCMDHTYTTIRVPVAASVREIIS--------AVADK-----------LGSG-EG--LIV
VKMSSG----------------------GEKVV------LKPNDVSVFTTLTINGRLFAC
PREQ---F-DSLT-PLPEQ---------------------------EGP------TVGTV
GT--FELMSSKDLAYQMTIYDWELFNCVHEMELIY-------HTFGRHNF-KK-TTANLD
LFLRR---------FNEIQFWV-----VTEICLCSQLSKR------VQLLKKFIKIAAH-
--------------CKEYKNLNSFFAIVM-GLSNVAVSRLALTWE---------------
-------KLPSKFKKFYAE-FESLM--------------------------DPSRNH---
-RAYRL----TAAK-------LEPPLI-PFMP----------------------------
LLIKDMTFTHEGNK--TF-TD-----------------------------N-LVNFE-KM
-------RM---IANTARTVRYCRS--Q-------------------------PFNLD--
-A------------------AQGNKNH-----Q----------------DVRS-----YV
RQLNVIDNQRTL---SQLSHRL
-----------EPRR----------P----------------------------------

>Rapgef4_[Diaphorina_citri]_662208416

----------------------LNQNDS-----GSSWYIVLGGSLEVRLITTNTQTTQTN
TIEKASVY--LKRYASSRDDIFLNQNDSGSSWYIVLGGSLEVRLITTNTQTTQTNTIEKT
I-----ITLTYLGVGATFTETLLGDL-PRGAHII-TKTTCELLRIQYRDFVEIKE-----
RNKD--VISELQ--------------M-NTKIKNGFEPTI-S----------KSSPS---
--MPT-QA---------------R--RTSTPDCPNPAE----------------------

--PIVE--------------
-----------------SPSVPMARA---------------------GWVLR-TLLLNDE
SG-T----------------LRDRKTSG-----G-RTI---------------ARRC-AS
GS-----------------ELVDWLMSLA-------------------------------
---PSLA-------VSRQITTGMW---QALLEEGVIY-----------------------
--------------H------------------------VNG-EQAFRDKC-ILYNFWQD
K---------E----GS---
---SSQAT
AQDI--AEAEEHL------EEALLALARRAPDAI-------------LRYILRKQPLDRT
SEDLE-----QIYEELLHLKPLHHLS-NSVRRELA-GVVMFEAHPRKGEILFHQGDEGKS
WYIIIQGSVDVVIYGKGCVTSLYAGEDFGKLALVNNAPRKYSYVFHQGDEG--KSWYIII
QGSVDVVIYGK-----GC-------------------------------VTSLYAG-EDF
GKLAL-----------VNNAPR--------------------------------------

---------Y--

-----KFR--

---GPNRF--QQITANLD

VFLRR---------FNEIQYWV-----ITEILLVTSLNKR------VQILRKMIKLAAY-
--------------CKEYRNINALFAVLM-GLSNVAVSRLSLTWD---------------
-------KLPSKSKKTYTE-LEALI--------------------------DPSKNH---
-RAYRQ----AVSK-------LQSPVI-PFMP----------------------------
LLLKDLAFTHDGNK--TV-VD-----------------------------G-LVNFE-KM
-------HM---FAQTLRTLRYCRT--R------HLVL-----------------DPP--
----------------------TPKCE-----S----------------EVKS-----YV
SCLRAMDNQRIL---TSMSQKL
-----------EPRR----------S----------------------------------

>Rapgef4_[Dipodomys_ordii]_852730826
----------------------------M-------------------------------

--FV--------------
-------FVL-TPL--I-E--------PHVPLRP--------------------------
------------------A---
-----------------N-TITK------------------VPS----------------
-------EKIL-------------RA---------------------GKILR-NAILSRA
PH-M----------------IRDRKYH------L-KTY----------------RQC-CV
GT-----------------ELVDWIMQQT-------------------------------
---SCVH--------SRTQAVGMW---QVLLEDGVLN-----------------------
--------------H------------------------VDQ-EHHFQDKY-LFYRFLDD
-------EHED----AP-L---
--PT
EEEK--KECEEEL------QDTMLLLSQMGPDAH-------------MRMILRKPPGQRT
VDDLE-----IIYEELLHIKALSHLS-TTVKRELA-GVLIFESHAKGGTVL---------
--FNQGEEG--TSWYIIL
KGSVNVVIYGK-----GV-------------------------------VCTLHEG-DDF
GKLAL-----------VNDAPRA---ASIVLREDNCHFLRVDKEDFNR------ILRD--
---V--------------

------------------------EANTVRLKEHDQDVLVLEK-----VPA-GNRASNQG
NS----QP-QQ------------------------------------K-YTVMSGTPEKI
LEHFLET------------IRLEP-ALN--------------EA-----TDAILN---D-
FVMMHCV--FM---------------PNTQLCPALV-----A------------------
--------H-----------Y-------HAQPSQ---GTEQEKMDYALNNKRRVIRLVLQ
WAATYGDLLQED-DVAMAFLE----EFYVS--VSDDA--RMIAVLK---EQ-LPELEKIV
KQI---SED--AK---------APQKK---------------------------------
---HRV--------LLQQFNTGD-ERSQ-KRQ----------------------------

-----PIRGSDE--VL-----------------------------FK-------------
VYCMDHTYTTIRVPVSASVKEVIS--------AVADK-----------LGSG-ES--LII
VKMSSG----------------------GEKVL------LKPNDVSVFTTLTINGRLFAC
PREQ---F-DSLT-PLPEQ---------------------------EGP------TTGTV
GT--FELMSSKDLAYQMTIYDWELFNCVHELELIY-------HTFGRHNF-KK-TTANLD
LFLRR---------FNEIQFWV-----VTEICLCSQLSKR------VQLLKKFIKIAAH-
--------------CKEYKNLNSFFAIIM-GLSNVAVSRLALTWE---------------
-------KLPSKFKKFYAE-FESLM--------------------------DPSRNH---
-RAYRL----TAAK-------LDPPLI-PFMP----------------------------
LLIKDMTFTHEGNK--TF-ID-----------------------------N-LVNFE-KM
-------RM---IANSARTVRYYRS--Q-------------------------PFNLD--
-A------------------AQANKNH-----Q----------------DVRS-----YV
RQLSVIDNQRTL---SQMSHRL
-----------EPRR----------P----------------------------------

>Rapgef4_[Diuraphis_noxia]_985397275
-------MAM----FFKRS------------TDMFRYT----------------------
------EKLLYGRV-------------V-----RI-K-----------------------
---PKLESWDK-------
I-----CAVVSLDVGATFGESIVHDL-PRDMTVC-TKTTCELLRIHQNDFKKIWD-----
KHSN--LMKDLF--------------A-SNKLRNGMASGS-L--------AKCQSPP--P
VMSQT------------------R--RTITPDIPDPAE----------------------

--VITE--------------
-----------------NPSMPMARA---------------------GWVLR-TLLLSDQ
NT-V----------------LRDRKTGG-----G-RSV---------------VRRC-AS
GS-----------------ELIDWLMNLV-------------------------------
---STDH-----DSFSRHDVIGMW---QALLEEGVIS-----------------------
--------------H------------------------ATG-EHPFKDKC-LFYNFWQD
R---------E----G--
--ALNTPT
IQDV--AEAEEHL------DEALQELVHRGPDAH-------------LRLILRSP-----

----------------IF--------------------------------LFS-------

-----------D--ST-----------------------------KY-------------
LYCI---IC---

------------T-CVPEQ---------------------------E-K------SEGVD
ISGDFDSLSTKELAYHMTLFDWELFWNVHEYELIY-------LTFGRHRF--QQITANLD
IFLRR---------FNEIQYWV-----ITEICTSQNISKR------TNILKKMIKLATY-
--------------CKEYYNFNAFFAILM-GLSDVAVSRLSTTWD---------------
-------KLPSKSRKQFTE-YETLI--------------------------DPSRNH---
-RAYRI----TVGK-------LPSPMI-PFMP----------------------------
LLIKDMKFTHDGNK--TH-VD-----------------------------G-LVNFE-KM
-------HM---LAQTMRTLRYCRA--R------HLVL-----------------DPP--
----------------------VSKNE-----A----------------DVKS-----YV
SCLRCVNNERTL---MSMSQKL
-----------EPRR----------T----------------------------------

>Rapgef4_[Drosophila_bipectinata]_1037099488
------------------------------------ML----------------------
----------------------FRRSCT-----YNV------------------------
--------------------------EQSTRHYA------------------K------S
S-----VTLCNLGVGATFGESVLHDL-PRDSTVV-TKTTCELLRVEQQDFRLIWE-----
KNKE--LMNDIF--------------T-NCKLKNGFGPGV-Q-TA-----AAATS-----
---PT------------------K--RPVSPDHPNPAL----------------------

--PITE--------------
-----------------TPSPAMSRM---------------------GWALR-TLLVADN
SS-C----------------LKDRKVS------G-K-L---------------IRKC-AP
GT-----------------ELVDWLVNLS-------------------------------
---PIVH--------TRAQAAGMW---QALVEEGVLS-----------------------
--------------H------------------------VNK-EQPFKDKC-FLYRFRLD
E---------E----GG-A---
---AAAGAPQ

TEDI--GAANEHI------REALSALFQRGPDAT-------------LRMILRKPSHERT
SEELE-----LVFEELVHIAALSHLS-TSIKRELS-SIFVFEAHAQAGTIL---------
--FNQGDEG--RSWYILL
KGSVDVVIHGK-----GT-------------------------------VATLKTG-DDF
GKLAL-----------INDAPRA---ATIVLKENNCHLLRVDKEHFNR------ILRD--
---V--------------

------------------------EANTLRLQEHGKDVLVLER-----VAK---------
------QRGQ----HS---------------AFK---------------YTVMSGTPAKM
LEHLLET-------------RL---G---Q-------SVGG--------MDPFLD---D-
FLLTHIV--FM---------------PVVQLVDELA-----N------------------
--------YF---HCDA---------H-----EE---AQTPEDREYIINFKKRVILFMQK
WVMAVRHAAFEE-PSVCDFIE----DLAAE--VEAD-----------------PDLNEET
SIV---
---HNV--------LTQMARYQE-DRNQNAGQK---------------------------
----WKLPP---NGQPIC--------------------------------LFSGNAA---
---PSKT---V---

------IRPDDD--II-----------------------------FR-------------
VYCADHTYCTLRFPMHTTAELIKA--------CAADK-----------LQLNRGPEDLVL
VEVKSN----------------------GERSV------FKDNDVSIPTGLTLNGRLFVS
VKDH---L-DALT-PLQEQ---------------------------EGP------TEGVD
I--DLEILSTKELAYHITLFEWDLFWAVHEYELLY-------HTFGRHHF--GKITANLD
VFLRR---------FNEVQYWI-----VTELVSTPSLSKR------VGLVRKFIKLAAY-
--------------CKEYQNLNAFFAVVM-GLSNMAVSRLQQTWE---------------
-------KIPSKFRKIFQE-FEALI--------------------------DPSRNH---
-RAYRV----FVGK-------LQPPLI-PFMP----------------------------
LLLKDMTFAHEGNK--TS-LD-----------------------------G-LVNFE-KM
-------HM---MAQTMRTIRFCRS--R------SLGL-----------------EPP--
----------------------SPKSE-----G----------------EVRS-----YI
SSFRVIDNQRVL---TAMSQKV
-----------EPTR----------KL---------------------------------

>Rapgef4_[Drosophila_ficusphila]_1036935367
------------------------------------ML----------------------
----------------------FRRSCT-----YNV------------------------
--------------------------EQSTRHYA------------------K------S
A-----VTLCNLGVGATFGESVLHDL-PRDSTVV-TKTTCELLRVEQQDFRLIWE-----
KNKE--LMNDIF--------------T-NCKLKNGFGPSV-Q-TA-----AA-TS-----
---PT------------------K--RPLSPDHPNPAL----------------------

```
------------------------------------------------------------
------------------------------------------PITE--------------
-----------------TPSPAMNRM---------------------GWALR-TLLVADN
SS-C----------------LKDRKVS------G-K-L---------------IRKC-AP
GT-----------------ELVDWLVNLS-------------------------------
---PIVH--------TRAQAAGMW---QALLEEGVLA-----------------------
--------------H------------------------VNK-EQPFKDKC-FLYRFRLD
E---------E----GG-T-----------------------------------------
-----------------------------------------------------AAAGVPQ
AEDL--GAANEHI------RETLSALFQRGPDAT-------------LRMILRKPSHERT
SEELE-----LVFEELVHIAALSHLS-TSIKRELS-AIFVFEAHAQAGTIL---------
--------------------------------------------FNQGDEG--RSWYILL
KGSVDVVIHGK-----GT-------------------------------VATLKTG-DDF
GKLAL-----------INDAPRA---ATIVLKENNCHLLRVDKEHFNR------ILRD--
---------------------------------------------V--------------
------------------------------------------------------------
------------------------------------------------------------
------------------------EANTLRLQEHGKDVLVLER-----VAK---------
------QRGQ----HS---------------AFK---------------YTVMSGTPAKM
LEHLLET-------------RL---G---Q-------SVGG--------MDPFLD---D-
FLLTHIV--FM---------------PVVQLVDELA-----N------------------
--------YF---HCDA---------H-----ED---AQTPEDREYIINFKKRVIQFMQK
WVMAVRHAAFEE-PSVCDFIE----DLAAE--VEAD-----------------PDLNEET
SIV---------------------------------------------------------
---HNV--------LTQMARYQE-DRNQNAGQK---------------------------
----WKLPP---NGQPIC--------------------------------LFSGNAT---
---PSKT---V-------------------------------------------------
------------------------------------------------------------
------------------------------------------------------------
------------------------------------------------------------
------------------------------------------------------------
------IRPDDD--II-----------------------------FR-------------
VYCADHTYCTLRFPMHTTAELIKA--------CAADK-----------LQLNRGPEDLVL
VEVKSN----------------------GERSV------FKDNDVSIPTGLSLNGRLFVS
VKDH---L-DALT-QLQEQ---------------------------ECP------TEGVD
I--DLEILSTKELAYHITLFEWDLFWAVHEYELLY-------HTFGRHHF--GKITANLD
VFLRR---------FNEVQYWI-----VTELVSTPSLSKR------VGLVRKFIKLAAY-
--------------CKEYQNLNAFFAVVM-GLSNMAVSRLQQTWE---------------
-------KIPSKFRKIFQE-FEALI--------------------------DPSRNH---
-RAYRV----FVGK-------LQPPLI-PFMP----------------------------
LLLKDMTFAHEGNK--TS-LD-----------------------------G-LVNFE-KM
-------HM---MAQTMRTIRFCRS--R------SLGL-----------------EPP--
----------------------SPKSE-----G----------------EVRS-----YI
SSFRVIDNQRVL-----------------------------------------TAMSQKV
-----------EPTR----------KL---------------------------------
------------------------------------------------------------
------------------------------------------------------------
------------------------------------------------------------
------------------------------------------------------------
------------------------------------------------------------
------------------------------------------------------------
------------------------------------------------------------
------------------------------------------------------------
------------------------------------------------------------
```


>Rapgef4_[Drosophila_kikkawai]_1036730951
------------------------------------ML----------------------
----------------------FRRSCT-----YNV------------------------
--------------------------EQSTRHYA------------------K------S
A-----VTLCNLGVGATFGESVLHDL-PRDSTVV-TKTTCELLRVEQQDFRLIWE-----
KNKE--LMNDIF--------------T-NCKFKNGFGPGV-Q-TA-----AAATS-----
---PT------------------K--RPLSPDHPNPAL----------------------

--PITE--------------
-----------------TPSPAMSRM---------------------GWALR-TLLVADN
SS-C----------------LKDRKVS------G-K-L---------------IRKC-AP
GT-----------------ELVDWLVNLS-------------------------------
---PIVH--------TRAQAAGMW---QALVEEGVLA-----------------------
--------------H------------------------VNK-EQPFKDKC-FLYRFRLD
E---------E----GG-A---
---AAAGVPQ
SDDL--GAANEHI------REALSALFQRGPDAT-------------LRMILRKPSHERT
SEELE-----LVFEELVHIAALSHLS-TSIKRELS-SIFVFEAHAQAGTIL---------
--FNQGDEG--RSWYILL
KGSVDVVIHGK-----GT-------------------------------VATLKTG-DDF
GKLAL-----------INDAPRA---ATIVLKENNCHLLRVDKEHFNR------ILRD--
---V--------------

------------------------EANTLRLQEHGKDVLVLER-----VAK---------
------QRGQ----HS---------------AFK---------------YTVMSGTPAKM
LEHLLET-------------RL---G---Q-------SVGG--------MDPFLD---D-
FLLTHIV--FM---------------PVVQLVDELA-----N------------------
--------YF---HCDA---------H-----EE---AQTPEDREYIINFKKRVIQFMQK
WVMAVRHAAFEE-PSVCDFIE----DLAAE--VEAD-----------------PDLNEET
SIV---
---HNV--------LTQMARYQE-DRNQNAGQK---------------------------
----WKLPP---NGQPIC--------------------------------LFSGNAT---
---PSKT---V---

------IRPDDD--II-----------------------------FR-------------
VYCADHTYCTLRFPMHTTAELIKA--------CAADK-----------LQLNRGPEDLVL
VEVKSN----------------------GERSV------FKDNDVSIPTGLSLNGRLFVS
VKDH---L-DALT-QLQEQ---------------------------ECP------TEGVD
I--DLEILSTKELAYHITLFEWDLFWAVHEYELLY-------HTFGRHHF--GKITANLD
VFLRR---------FNEVQYWI-----VTELVSTPSLSKR------VGLVRKFIKLAAY-
--------------CKEYQNLNAFFAVVM-GLSNMAVTRLQQTWE---------------
-------KIPSKFKKIFQE-FEALI--------------------------DPSRNH---
-RAYRV----FVGK-------LQPPLI-PFMP----------------------------
LLLKDMTFAHEGNK--TS-LD-----------------------------G-LVNFE-KM
-------HM---MAQTMRTIRFCRS--R------SLGL-----------------EPP--
----------------------SPKSE-----G----------------EVRS-----YI
SSFRVIDNQRVL---TAMSQKV
-----------EPTR----------KL---------------------------------

>Rapgef4_[Drosophila_miranda]_1037006446
-------MSAAQQQRQGQRQRWSAQIVASDEAAWCVLLAYRRHSDSHEHEVLESERTAGA
PTAGATSAVIKRRLRLERGQPARPQSTA-----FEM------------------------
--------------------------SN--------------------------------

KNKE--LMNDIF--------------T-NCKLKNGFGPSV-Q-QT-----AAAPS-----
---PT------------------K--RPLSPDHPNPAL----------------------

--PITE--------------
-----------------TPSPAMSRM---------------------GWALR-TLLVADN
SS-C----------------LKDRKVS------G-K-L---------------IRKC-AP
GT-----------------ELVDWLVNLA-------------------------------
---PIVH--------TRAQAAGMW---QALVEEGVLT-----------------------
--------------H------------------------VNK-EQPFKDKC-FLYRFRLD
E---------E----GG-A---
---PAVGIPQ
AEDQ--HAANEHI------REALSALFQRGPDAT-------------LRMILRKPSHERT
SEELE-----LVFEELVHIAALSHLS-TSIKRELS-SIFVFEAHAKAGTIL---------
--FNQGDEG--RSWYILL
KGSVDVVIHGK-----GT-------------------------------VATLKTG-DDF
GKLAL-----------INDAPRA---ATIVLKENNCHLLRVDKEHFNR------ILRD--
---V--------------

------------------------EANTLRLQEHGKDVLVLER-----VAK---------
------QRGQ----HS---------------AFK---------------YTVMSGTPAKM
LEHLLET-------------RL---G---Q-------SVGG--------MDPFLD---D-
FLLTHIV--FM---------------PVVQLVDELA-----N------------------
--------YF---HCDT---------H-----ED---AQTPEDREYIVNFKKRVIQFMQK
WVMAVRHAAFEE-PSVCDFIE----DLAAE--VEAD-----------------PDLNEET
SIV---
---HNV--------LTQMARYQE-DRNQNAGQK---------------------------
----WKLPP---NGQPIC--------------------------------LFSGNAT---
---PSKT---V---

------IRPDDD--II-----------------------------FR-------------
VYCADHTYCTLRFPMHTTAELIKA--------CAADK-----------LQLNRGPEDLCL
VEVRSN----------------------GERSV------FKDNDVSIPTGLSLNGRLFVS
VKDH---L-DALT-QLQEQ---------------------------ECP------TEGVD
I--DLEILSTKELAYHITLFEWDLFYAVHEYELLY-------HTFGRHHF--GKITANLD

VFLRR---------FNEVQYWI-----VTELVSTPSLSKR------VGLVRKFIKLAAY-
--------------CKEYQNLNAFFAVVM-GLSNMAVSRLHQTWE---------------
-------KIPSKFRKIFQE-FEALI--------------------------DPSRNH---
-RAYRV----FVGK-------LQPPLI-PFMP----------------------------
LLLKDMTFAHEGNK--TS-LD-----------------------------G-LVNFE-KM
-------HM---MAQTMRTIRFCRS--R------SLGL-----------------EPP--
----------------------SPKSE-----G----------------EVRS-----YI
SSFRVIDNQRVL---TAMSQKV
-----------EPTR----------KL---------------------------------

>Rapgef4_[Drosophila_rhopaloa]_1036871102
-----------------------------MAMEWITAM----------------------
----------------------DKRPCD-----RNLRDVELISCRLRRVEPLCRLPGSAL
QQLAMCGF--YEDLE--KGVTLFRAGEQGRFWYAVLGGSLEVRYHAHADADGK------S
A-----VTLCNLGVGATFGESVLHDL-PRDSTVV-TKTTCELLRVEQQDFRLIWE-----
KNKE--LMNDIF--------------T-NCKLKNGFGPTV-QASA-----AA-TS-----
---PT------------------K--RPLSPDHPNPAL----------------------

--PITE--------------
-----------------TPSPAMNRM---------------------GWALR-ILLVADN
SS-C----------------LKDRKVS------G-K-L---------------IRKC-AP
GT-----------------ELVDWLVNLS-------------------------------
---PIVH--------TRAQAAGMW---QALVEEGVLA-----------------------
--------------H------------------------VNK-EQPFKDKC-FLYRFRLD
E---------E----GG-T---
---AAAGVPQ
AEDL--GAANEHI------REGLSALFQRGPDAT-------------LRMILRKPSHERT
SEELE-----LVFEELVHIAALSHLS-TSIKRELS-SIFVFEAHAQAGTIL---------
--FNQGDEG--RSWYILL
KGSVDVVIHGK-----GT-------------------------------VATLKTG-DDF
GKLAL-----------INDAPRA---ATIVLKENNCHLLRVDKEHFNR------ILRD--
---V--------------

------------------------EANTLRLQEHGKDVLVLER-----VAK---------
------QRGQ----HS---------------AFK---------------YTVMSGTPAKM
LEHLLET-------------RL---G---Q-------SVGG--------MDPFLD---D-
FLLTHIV--FM---------------PVVQLVDELA-----N------------------
--------YF---HCDA---------H-----ED---AQTPEDREYIINFKKRVIQFMQK
WVMAVRHAAFEE-PSVCDFIE----DLAAE--VEAD-----------------PDLNEET
SIV---
---HNV--------LTQMARYQE-DRNQNAGQK---------------------------
----WKLPP---NGQPIC--------------------------------LFSGNAT---
---PSKT---V---

------IRPDDD--II-----------------------------FR-------------
VYCADHTYCTLRFPMHTTAELIKA--------CAADK-----------LQLNRGPEDLVL
VEVKSN----------------------GERSV------FKDNDVSIPTGLSLNGRLFVS
VKDH---L-DALT-QLQEQ---------------------------EGP------TEGVD
I--DLEILSTKELAYHITLFEWDLFWAVHEYELLY-------HTFGRHHF--GKITANLD
VFLRR---------FNEVQYWI-----VTELVSTPSLSKR------VGLVRKFIKLAAY-
--------------CKEYQNLNAFFAVVM-GXSNMAVSRLQQTWE---------------
-------KIPSKFRKIFQE-FEALI--------------------------DPSRNH---
-RAYRV----FVGK-------LQPPLI-PFMP----------------------------
LLLKDMTFAHEGNK--TS-LD-----------------------------G-LVNFX-KM
-------HM---MAQTMRTIRFCRS--R------SLGL-----------------EPP--
----------------------SPKSE-----G----------------EVRS-----YI
SSFRVIDNQRVL---TAMSQKV
-----------EPTR----------KL---------------------------------

>Rapgef4_[Drosophila_suzukii]_1036073995

-------MNDIF--------------T-NCKLKNGFGPSV-Q-AS-----ATATS-----
---PT------------------K--RPLSPDHPNPAL----------------------

--PITE--------------
-----------------TPSPAMNRM---------------------GWALR-TLLVADN
SS-C----------------LKDRKVS------G-K-L---------------IRKC-AP
GT-----------------ELVDWLVNLS-------------------------------
---PIVH--------TRAQAAGMW---QALVEEGVLA-----------------------
--------------H------------------------VNK-EQPFKDKC-FLYRFRID
E---------E----GG-T---
---AAAGVPQ
AEDL--GAANEHI------REALSALFQRGPDAT-------------LRMILRKPSHERT
SEELE-----LVFEELVHIAALSHLS-TSIKRELS-SIFVFEAHAQAGTIL---------
--FNQGDEG--RSWYILL
KGSVDVVIHGK-----GT-------------------------------VATLKTG-DDF
GKLAL-----------INDAPRA---ATIVLKENNCHLLRVDKEHFNR------ILRD--
---V--------------

------------------------EANTLRLQEHGKDVLVLER-----VAK---------

------QRGQ----HS---------------AFK---------------YTVMSGTPAKM
LEHLLET-------------RL---G---Q-------SVGG--------MDPFLD---D-
FLLTHIV--FM---------------PVVQLVDELA-----N------------------
--------YF---HCDA---------H-----ED---AQTPEDREYIINFKKRVIQFMQK
WVMAVRHAAFEE-PSVCDFIE----DLAAE--VEAD-----------------PDLNEET
SIV---
---HNV--------LTQMARYQE-DRNQNAGQK---------------------------
----WKLPP---NGQPIC--------------------------------LFSGNAT---
---PSKT---V---

------IRPDDD--II-----------------------------FR-------------
VYCADHTYCTLRFPMHTTAELIKA--------CAADK-----------LQLNRGPEDLVL
VEVRSN----------------------GERSV------FKDNDVSIPTGLSLNGRLFVS
VKDH---L-DALT-QLQEQ---------------------------EGP------TEGVD
I--DLEILSTKELAYHITLFEWDLFWAVHEYELLY-------HTFGRHHF--GKITANLD
VFLRR---------FNEVQYWI-----VTELVSTPSLSKR------VGLVRKFIKLAAY-
--------------CKEYQNLNAFFAVVM-GLSNMAVSRLQQTWE---------------
-------KIPSKFRKIFQE-FEALI--------------------------DPSRNH---
-RAYRV----FVGK-------LQPPLI-PFMP----------------------------
LLLKDMTFAHEGNK--TS-LD-----------------------------G-LVNFE-KM
-------HM---MAQTMRTIRFCRS--R------SLGL-----------------EPP--
----------------------SPKSE-----G----------------EVRS-----YI
SCFRVIDNQRVL---TAMSQKV
-----------EPTR----------KL---------------------------------

>Rapgef4_[Drosophila_takahashii]_1036922041
------------------------------------MT----------------------
----------------------RPQSTA-----FEM------------------------
--------------------------SN--------------------------------

KNKE--LMNDIF--------------T-NCKLKNGFGPSV-Q-TA-----AAATS-----
---PT------------------K--RPLSPDHPNPAL----------------------

--PITE--------------
-----------------TPSPAMNRM---------------------GWALR-TLLVADN
SS-C----------------LKDRKVS------G-K-L---------------IRKC-AP
GT-----------------ELVDWLVNLA-------------------------------
---PIVH--------TRAQAAGMW---QALVEEGVLA-----------------------
--------------H------------------------VNK-EQPFKDKC-FLYRFRLD
E---------E----GG-T---
---AAAGVPQ

```
AEDL--GAANEHI------REALSALFQRGPDAT-------------LRMILRKPSHERT
SEELE-----LVFEELVHIAALSHLS-TSIKRELS-SIFVFEAHAQAGTIL---------
--------------------------------------------FNQGDEG--RSWYILL
KGSVDVVIHGK-----GT-------------------------------VATLKTG-DDF
GKLAL-----------INDAPRA---ATIVLKENNCHLLRVDKEHFNR------ILRD--
---------------------------------------------V--------------
------------------------------------------------------------
------------------------------------------------------------
------------------------EANTLRLQEHGKDVLVLER-----VAK---------
------QRGQ----HS---------------AFK---------------YTVMSGTPAKM
LEHLLET-------------RL---G---Q-------SVGG--------MDPFLD---D-
FLLTHIV--FM---------------PVVQLVDELA-----N------------------
--------YF---HCDA---------H-----ED---AQTPEDREYIINFKKRVIQFMQK
WVMAVRHAAFEE-PSVCDFIE----DLAAE--VEAD-----------------PDLNEET
SIV---------------------------------------------------------
---HNV--------LTQMARYQE-DRNQNAGQK---------------------------
----WKLPP---NGQPIC--------------------------------LFSGNAT---
---PAKT---V-------------------------------------------------
------------------------------------------------------------
------------------------------------------------------------
------------------------------------------------------------
------------------------------------------------------------
------IRPDDD--II-----------------------------FR-------------
VYCADHTYCTLRFPMHTTAELIKA--------CAADK-----------LQLNRGPEDLVL
VEVKSN----------------------GERSV------FKDNDVSIPTGLSLNGRLFVS
VKDH---L-DALT-QLQEQ---------------------------ECP------TEGVD
I--DLEILSTKELAYHITLFEWDLFWAVHEYELLY-------HTFGRHHF--GKITANLD
VFLRR---------FNEVQYWI-----VTELVSTPSLSKR------VGLIRKFIKLAAY-
--------------CKEYQNLNAFFAVVM-GLSNMAVSRLQQTWE---------------
-------KIPSKFRKIFQE-FEALI--------------------------DPSRNH---
-RAYRV----FVGK-------LQPPLI-PFMP----------------------------
LLLKDMTFAHEGNK--TS-LD-----------------------------G-LVNFE-KM
-------HM---MAQTMRTIRFCRS--R------SLGL-----------------EPP--
----------------------SPKSE-----G----------------EVRT-----YI
SSFRVIDNQRVL-----------------------------------------TAMSQKV
-----------EPTR----------KL---------------------------------
------------------------------------------------------------
------------------------------------------------------------
------------------------------------------------------------
------------------------------------------------------------
------------------------------------------------------------
------------------------------------------------------------
------------------------------------------------------------
------------------------------------------------------------
------------------------------------------------------------
------------------------------------------------------------
---
&gt;Rapgef4_[Echinops_telfairi]_507650135
----------------------------MVAA-------HA------TH--------SSS
SAEWIA---CLDK-----------RPLE-----RSSEDVDIIFTRLKEVKAFEKFHPNLL
HQICLCGY--YENLE--KGITLFRQGDIGTNWYAVLAGSLDVKVSE-TSSHQDA------
------VTICTLGIGTAFGESILDNT-PRHATIV-TRECSELL-----------------
----------------------------RIEQKD-------FRALW---EKYRQYMAG-L
LAPPYG--VMETGS--N-N--------DRIPDKE--------------------------
```

------------------N----------------------T------------------
-----PLIEPHVPLHPAN-SITK------------------VPS----------------
-------EKIL-------------RA---------------------GKILR-NAILSRA
PH-M----------------IRDRKYH------L-ETY----------------RQC-CV
GT-----------------ELVDWMVQQT-------------------------------
---QCVH--------SRTQAVGMW---QVLLEDGVLN-----------------------
--------------H------------------------VDQ-EHHFQDKY-LFYRFLDD
-------ERED----AP-L---
--PT
EEEK--TQGDEEL------QDTVLLLSQLGPDAH-------------MRMILRKPPGQRT
MDDLE-----IIYEELLHIKALSHLS-TTVKRELA-GVLIFESHAKGGTVL---------
--FNQGEEG--TSWYIIL
KGSVNVVIYGK-----GV-------------------------------VCTLHEG-DDF
GKLAL-----------VNDAPRA---ASIVLREDNCHFLRVDKEDFNR------ILRD--
---V--------------

------------------------EANTVRLKEHDQDVLVLEK-----VPA-GNRASNQG
NS----QP-QQ------------------------------------K-YTVMSGTPEKI
LEHFLET------------IRLEP-ALN--------------EA-----TESVLN---D-
FVMMHCV--FM---------------PNTQLCPGLV-----A------------------
--------H-----------Y-------HAQPSQ---GTQQEKMDYALNNKRRVIRLVLQ
WAAVHGDLLQED-DVAVAFLE----EFYVS--VSDDA--RMIAALK---EQ-LPELEKIV
KQI---LEE--AK---------APQKK---------------------------------
---HKV--------LLQQFNTGD-ERAQ-KRQ----------------------------

-----PIRGSDE--VL-----------------------------FK-------------
VYCMDHTYTTIRVPVGASVKEVIS--------AVADK-----------LGSG-EG--LII
VKMSSG----------------------GEKVV------LKPNDVSVFTTLTINGRLFAC
PREQ---F-DSLT-PLPEQ---------------------------EGP------TVGTM
GT--FELMSSKDLAYQMTIYDWELFHCVHELELIY-------HTFGRHNF-KK-TTANLD
LFLRR---------FNEIQFWV-----VTEVCLCSQLSKR------VQLLKKFIKIAAH-
--------------CKEYKNLNAFFAIVM-GLSNVAVSRLALTWE---------------
-------KLPSKFKKFYAE-FESLM--------------------------DPSRNH---
-RAYRL----TVAK-------LEAPLI-PFMP----------------------------
LLIKDMTFIHEGNK--TF-ID-----------------------------N-LVNFE-KM
-------RM---IANTARTVRYCRS--Q-------------------------PFNPD--
-A------------------AQANKNH-----Q----------------DVRS-----YV
RQLNVIDNQRTL---SQMSHRL
-----------EPRR----------P----------------------------------


```
------------------------------------------------------------
---
>Rapgef4_[Egretta_garzetta]_697837442
----------------------------MVAA-------HT------SH--------SSS
SGEWIA---CLDK-----------RPLE-----RSSEDVDIIFTRLKEVKAFEKFHPNLL
QQICLCGY--YENLE--KGITLFRQGDIGTNWYAVLTGSLDVKVSD-TSNHQDA------
------VTICTLGIGTAFGESILDNT-PRHATIV-TREYSELL-----------------
----------------------------RIEQKD-------FKALW---EKYRQYMSG-L
LTPPYG--VMETGS--N-N--------DRIPTKQ--------------------------
------------------N----------------------T------------------
-----PLIEPHIPHRATK-TITQ------------------VPS----------------
-------EKIL-------------RA---------------------GKILR-NTILSRA
PH-M----------------IRDRKYH------L-KTY----------------RQC-CV
GT-----------------ELVDWMMQQS-------------------------------
---PCVH--------SRTQAVGMW---QVLLEEGVLN-----------------------
--------------H------------------------VDQ-EHHFQDKY-LFYRFLDD
-------ERED----AP-L-----------------------------------------
----------------------------------------------------------PT
EEEK--KECDEEL------QDTMLLLSQIGPDAH-------------MRMILRKPPGQRT
VDDLE-----IIYEELLHIKALSHLS-TTVKRELA-GVLIFESHPKAGTVL---------
--------------------------------------------FNQGEEG--TSWYIIL
KGSVNVVIYGK-----GV-------------------------------VCTLHEG-DDF
GKLAL-----------VNDAPRA---ASIVLREDNCHFLRVDKEDFNR------ILRD--
---------------------------------------------V--------------
------------------------------------------------------------
------------------------------------------------------------
------------------------EANTVRLKEHDQDVLVLEK-----IPA-GNRVSNQG
NS----QP-QH------------------------------------K-YIVMSGTPEKI
LEHFLET------------MRPEA-TLN--------------EA-----TDSVLN---D-
FIMMHCV--FM---------------PNSQLCPALM-----A------------------
--------H-----------Y-------HAQPSQ---GTEQEKMDYALNNKRRVIRLVLQ
WAALYGDLLQED-EAAMAFLE----EFYVS--VSDDT--RMIAALK---EQ-LPELEKIV
KQV---SEE--PK---------APQKK---------------------------------
---HKV--------LLQLFNTSD-DRAQ-KRQ----------------------------
------------------------------------------------------------
------------------------------------------------------------
------------------------------------------------------------
------------------------------------------------------------
------------------------------------------------------------
------------------------------------------------------------
-----PIRGSDE--VL-----------------------------FK-------------
VYCIDQTYTTIRVPVSSSVKEVIS--------AVADK-----------LGSG-EG--LII
VKMSSG----------------------GEKVV------LKPHDVSVFTTLSVNGRLFAC
PRDQ---F-DSLA-PLPEQ---------------------------EGP------STGTV
GT--FELMSSKDLAHQMTIYDWELFNCVHELELIY-------HTFGRHNF-KK-TTANLD
LFLRR---------FNEIQFWV-----VTEICLCSQLSKR------VQLLKKYIKIAAH-
--------------CKEYKNLNSFFAIIM-GLSNVAVSRLSLTWE---------------
-------KLPSKFKKIYAE-FESLM--------------------------DPSRNH---
-RAYRL----TVAK-------LDPPII-PFMP----------------------------
LLIKDMTFTHEGNK--TF-TD-----------------------------N-LVNFE-KM
-------RM---IANTVRTVKFCRS--Q-------------------------SFNPD--
-A------------------ALTNKNH-----Q----------------DVRS-----YV
RQLNVIDNQRTL-----------------------------------------SHMSHRL
-----------EPRR----------A----------------------------------
```


>Rapgef4_[Elephantulus_edwardii]_585637321

---MAG-L
LAPPYG--VMETGS--N-N--------DRIPDKE--------------------------
------------------N---
---VPS----------------
-------EKIL-------------RA---------------------GKILR-NAILSRA
PH-M----------------IRDRKYH------L-KTY----------------RQC-CV
GT-----------------ELVDWMMQQT-------------------------------
---QCVH--------SRAQAVGMW---QVLLEDGVLN-----------------------
--------------H------------------------VDQ-EHHFQDKY-LFYRFLDD
-------EHED----AP-L---
--PT
EEEK--KECDEEL------QDTMLLLSQMGPDAH-------------MRMILRKPPGQRT
VDDLE-----IIYEELLHIKALSHLS-TTVKRELA-GVLIFESHAKGGTVL---------
--FNQGEEG--TSWYIIL
KGSVNVVIYGK-----GV-------------------------------VCTLHEG-DDF
GKLAL-----------VNDAPRA---ASIVLREDNCHFLRVDKEDFNR------ILRD--
---V--------------

------------------------EANTVRLKEHDQDVLVLEK-----VPA-GNRASNQG
NS----QP-QQ------------------------------------K-YTVMSGTPEKI
LEHFLET------------IRLEP-ALN--------------EA-----TDSILN---D-
FVMMHCI--FM---------------PNTQLCPALV-----A------------------
--------H-----------Y-------HAQPSQ---GTEQEKMDYALNNKRRVIRLVLQ
WAAMNGDLLQED-DVAVAFLE----EFYVS--VSDDA--RMIAALK---EQ-LPELEKIV
KQI---LEE--AK---------APQKK---------------------------------
---HKV--------LLQQFNTGD-ERAQ-KRQ----------------------------

-----PIRGSDE--VL-----------------------------FK-------------
VYCMDHTYTTIRVPVAASVKEVIS--------AVADK-----------LGSG-EG--MII
VKMSSG----------------------GEKVV------LKPNDVSVFTTLTINGRLFAC
PREQ---F-DSLT-PLPEQ---------------------------EGP------TVGTV
GT--FELMSSKDLAYQMTIYDWELFNCVHELELIY-------HTFGRHNF-KK-TTANLD

LFLRR---------FNEIQFWV-----VTEICLCSQLSKR------VQLLKKFIKIAAH-
--------------CKEYKNLNSFFAIVM-GLSNVAVSRLALTWE---------------
-------KLPSKFKKFYAE-FESLM--------------------------DPSRNH---
-RAYRL----TVAK-------LEPPLI-PFMP----------------------------
LLIKDMTFTHEGNK--TF-ID-----------------------------N-LVNFE-KM
-------RM---IANTARTVRYCRS--Q-------------------------PFNPD--
-A------------------AQANKNH-----Q----------------DVRS-----YV
RQLNVIDNQRTL---SQMSHRL
-----------EPRR----------P----------------------------------

>Rapgef4_[Eptesicus_fuscus]_641696743

---MAG-L
LAPPYG--VMETGS--N-N--------DRIPDKE--------------------------
------------------N---
---VPS----------------
-------EKIL-------------RA---------------------GKILR-NTILSRA
PH-M----------------IRDRKYH------L-KTY----------------RQC-CV
GT-----------------ELVDWMLQQT-------------------------------
---PCVH--------SRTQAVGMW---QVLVEDGVLN-----------------------
--------------H------------------------VDQ-EHHFQDKY-LFYRFLDD
-------EHED----AP-L---
--PT
EEEK--KECDEEL------QDTMLLLSQMGPDAH-------------MRMILRKSPGQRS
MDDLE-----IIYDELLHIKALSHLS-TTVKRELA-GVLIFESHAKGGTVL---------
--FNQGEEG--TSWYIIL
KGSVNVVIYGK-----GV-------------------------------VCTLHEG-DDF
GKLAL-----------VNDAPRA---ASIVLREDNCHFLRVDKEDFNR------ILRD--
---V--------------

------------------------EANTVRLKEHDQDVLVLEK-----VPA-GSRASNQG
NS----QP-QQ------------------------------------K-YTVMSGTPEKI
LEHFLET------------IRLEP-ALN--------------EA-----TDSFLN---D-
FVMMHCV--FM---------------PNTQLCPALM-----A------------------
--------H-----------Y-------HAQPSQ---GTEQEKMDYALNNKRRVVRLVLQ
WAAVYGDLLQED-DVAMAFLE----EFYVS--VSDDA--RMIAALK---EQ-LPELERIV
KQI---SED--AK---------NPQKK---------------------------------
---HKV--------LLQHFNTSD-ERAQ-KRQ----------------------------

-----PIRGSDE--VL-----------------------------FK-------------
VYCMDHTYTTIRVPVAASVREVIS--------AVADK-----------LGSG-EG--LII
VKMSSG----------------------GEKVV------LKPNDVSVFTTLTINGRLFAC
PREQ---F-DSLT-PLPEQ---------------------------EGP------TVGTV
GT--FELMSSKDLAYQMTIYDWELFNCVHELELIY-------HTFGRHNF-KK-TTANLD
LFLRR---------FNEIQFWV-----VTEICLCSQPSKR------VQLLKKFIKIAAH-
--------------CKEYKNLNSFFAIVM-GLSNVAVSRLALTWE---------------
-------KLPSKFKKFYAE-FESLM--------------------------DPSRNH---
-RAYRL----TVAK-------LEPPLI-PFMP----------------------------
LLIKDMTFTHEGNK--TF-ID-----------------------------N-LVNFE-KM
-------RM---IANTARTVRYCRS--Q-------------------------PFNLD--
-A------------------AQANKNH-----Q----------------DVRS-----YV
RQLNVIDNQRTL---SQMSHRL
-----------EPRR----------P----------------------------------

>Rapgef4_[Equus_asinus]_958716126

---MAG-L
LAPPYG--VMETSS--N-N--------DRIPDKE--------------------------
------------------N---
---VPS----------------
-------EKIL-------------RA---------------------GKILR-NAILSRA
AH-M----------------IRDRKYH------L-KTY----------------RQC-CV
GT-----------------ELVDWMLQQT-------------------------------
---PCAH--------SRTQAVGMW---QVLVEDGVLN-----------------------
--------------H------------------------VDQ-EHHFQDKY-LFYRFLDD
-------EHED----AP-L---
--PT
EEEK--KECDEEL------QDTMLLLSQMGPDAH-------------MRMILRKPPGQRT
VDDLE-----IIYEELLHIKALSHLS-TTVKRELA-GVLIFESHAKGGTVL---------
--FNQGEEG--TSWYIIL
KGSVNVVIYGK-----GV-------------------------------VCTLHEG-DDF
GKLAL-----------VNDAPRA---ASIVLREDNCHFLRVDKEDFNR------ILRD--
---V--------------

------------------------EANTVRLKEHDQDVLVLEK-----VPA-GNRASNQG

NS----QP-QQ------------------------------------K-YTVMSGTPEKI
LEHFLET------------IRLEP-ALN--------------EA-----TDSVLN---D-
FVMMHCV--FM---------------PNIQLCPALV-----A------------------
--------H-----------Y-------HAQPSQ---GTEQEKMDYALNNKRRVIRLVLQ
WAAMYGDVLQED-DVAMAFLE----EFYVS--VSDDA--RMIAALK---EQ-LPELEKIV
KQI---SED--AK---------APQKK---------------------------------
---HKV--------LLQQFNTGD-ERAQ-KRQ----------------------------

-----PIRGSDE--VL-----------------------------FK-------------
VYCMDHTYTTIRVPVAASVKEVIS--------AVADK-----------LGSG-EG--LII
VKMSSG----------------------GEKVV------LKPNDVSVFTTLTINGRLFAC
PREQ---F-DSLT-PLPEQ---------------------------EGP------TVGTV
GT--FELMSSKDLAYQMTIYDWELFNCVHELELIY-------HTFGRHNF-KK-TTANLD
LFLRR---------FNEIQFWV-----VTEICLCPQPSKR------VQLLKKFIKIAAH-
--------------CKEYKNLNSFFAIVM-GLSNVAVSRLALTWE---------------
-------KLPSKFKKFYAE-FESLM--------------------------DPSRNH---
-RAYRL----TVAK-------LDPPLI-PFMP----------------------------
LLIKDMTFTHEGNK--TF-ID-----------------------------N-LVNFE-KM
-------RM---IANTARTVRYCRS--Q-------------------------PFNPD--
-A------------------AQANKNH-----Q----------------DVRS-----YV
RQLNVIDNQRTL---SQMSHRL
-----------EPRR----------P----------------------------------

>Rapgef4_[Equus_caballus]_953879137

---MAG-L
LAPPYG--VMETGS--N-N--------DRIPDKE--------------------------
------------------N----------------------T------------------
-----PLIEPHVPLRPAN-TITK------------------VPS----------------
-------EKIL-------------RA---------------------GKILR-NAILSRA
PH-M----------------IRDRKYH------L-KTY----------------RQC-CV
GT-----------------ELVDWMLQQT-------------------------------
---PCAH--------SRTQAVGMW---QVLVEDGVLN-----------------------
--------------H------------------------VDQ-EHHFQDKY-LFYRFLDD
-------EHED----AP-L---
--PT

```
EEEK--KECDEEL------QDTMLLLSQMGPDAH-------------MRMILRKPPGQRT
VDDLE-----IIYEELLHIKALSHLS-TTVKRELA-GVLIFESHAKGGTVL---------
--------------------------------------------FNQGEEG--TSWYIIL
KGSVNVVIYGK-----GV-------------------------------VCTLHEG-DDF
GKLAL-----------VNDAPRA---ASIVLREDNCHFLRVDKEDFNR------ILRD--
---------------------------------------------V--------------
------------------------------------------------------------
------------------------------------------------------------
------------------------EANTVRLKEHDQDVLVLEK-----VPA-GNRASNQG
NS----QP-QQ------------------------------------K-YTVMSGTPEKI
LEHFLET------------IRLEP-ALN--------------EA-----TDSVLN---D-
FVMMHCV--FM---------------PNIQLCPALV-----A------------------
--------H-----------Y-------HAQPSQ---GTEQEKMDYALNNRRRVIRLVLH
WAAMYGDVLQED-DVAMAFLE----EFYVS--VSDDA--RMIAALK---EQ-LPELEKIV
KQI---SED--AK---------APQKK---------------------------------
---HKV--------LLQQFNTGD-ERAQ-KRQ----------------------------
------------------------------------------------------------
------------------------------------------------------------
------------------------------------------------------------
------------------------------------------------------------
------------------------------------------------------------
------------------------------------------------------------
-----PIRGSDE--VL-----------------------------FK-------------
VYCMDHTYTTIRVPVAASVKEVIS--------AVADK-----------LGSG-EG--LII
VKMSSG----------------------GEKVV------LKPNDVSVFTTLTINGRLFAC
PREQ---F-DSLT-PLPEQ---------------------------EGP------TVGTV
GT--FELMSSKDLAYQMTIYDWELFNCVHELELIY-------HTFGRHNF-KK-TTANLD
LFLRR---------FNEIQFWV-----VTEICLCPQPSKR------VQLLKKFIKIAAH-
--------------CKEYKNLNSFFAIVM-GLSNVAVSRLALTWE---------------
-------KLPSKFKKFYAE-FESLM--------------------------DPSRNH---
-RAYRL----TIAK-------LDPPLI-PFMP----------------------------
LLIKDMTFTHEGNK--TF-ID-----------------------------N-LVNFE-KM
-------RM---IANTARTVRYCRS--Q-------------------------PFNPD--
-A------------------AQANKNH-----Q----------------DVRS-----YV
RQLNVIDNQRTL-----------------------------------------SQMSHRL
-----------EPRR----------P----------------------------------
------------------------------------------------------------
------------------------------------------------------------
------------------------------------------------------------
------------------------------------------------------------
------------------------------------------------------------
------------------------------------------------------------
------------------------------------------------------------
------------------------------------------------------------
------------------------------------------------------------
------------------------------------------------------------
---
>Rapgef4_[Esox_lucius]_884949900
---------------------------------MV-----A------VE---TSPNSSPS
-AEWVS---CLDK-----------RPAE-----RSGEDVDIILTRLKGVKAFRRFCPSLL
LQICSCAF--YEYLE--KGITLFRQGDIGTSWYAVLSGSLDVKVSE-TANHQDA------
------VTICTLGIGTAFGESILDNT-PRHATIV-SRETSELL-----------------
----------------------------RIEQRE-------FKSLWE---KYRQSMAG-L
LAPPYG--AMESGS----NN-------DRLADKD--------------------------
```

```
------------------SLG-------S-------------------------------
--------DSLSLMNKSL-----N----------------KVPS----------------
-------EKLQ-------------RG---------------------GKVMR-NAILSRA
PH-M----------------IRDRKYH------L-KTY----------------RQC-CV
GT-----------------ELVDWLVQQS-------------------------------
---TCVH--------TRSHAVGMW---QALLEEGVLN-----------------------
--------------H------------------------VDQ-ELGFQDKY-LFYRFLDD
-------EEED----TP-L-----------------------------------------
----------------------------------------------------------PS
EEEK--RESEEEL------PETILFLAQIGPDAL-------------LRMILRKPPGQRT
GDDLE-----IIYDELLHIKALSQLS-NTVKRELA-SVLIFESHAKAGTVL---------
--------------------------------------------FNQGEEG--TSWYIIQ
KGSVNVVIYGK-----GV-------------------------------VCMLHEG-DDF
GKLAL-----------VTDSPRA---ASIVLREDNCHFLRVDKEDFNR------ILRD--
---------------------------------------------V--------------
------------------------------------------------------------
------------------------------------------------------------
------------------------EANTVRLKEHEQAVLVLEK-----S----PRASTLG
N---------I------------------------------------K-YTVMSGTPEKI
LDHFLET------------MRMDT-HHS--------------DP------DPAVD---D-
FVLMHCV--FM---------------PNSQLCQLLM-----A------------------
--------H-----------Y-------HAVSPP---GSEQERMEYAVTCKRRVLNLAMR
WAAVHTYYLQEE-PAALTFLE----ELYGS--VSNDS--RILRALK---DF-VPDLEKVV
KLH---SGE--AK----------LRKK---------------------------------
-----V--------LLRQFSNGD-ERLA-KKQ----------------------------
------------------------------------------------------------
------------------------------------------------------------
------------------------------------------------------------
------------------------------------------------------------
------------------------------------------------------------
------------------------------------------------------------
-----PIRNSDD--IL-----------------------------LK-------------
VYCSDHTYTTIRVAVAATGREVTS--------AVGDK-----------LASN-ED--LLL
VHLSSA----------------------GEKHV------LKPSDVSVFSTLSINGRMFAC
PRDQ---L-NALT-PLPDQ---------------------------EGP------SAGSM
SS--FELMSSKDLAYQMTLFDWELFSCVHEHELLY-------HTFGRLSF-RR-TTANLD
LFIRR---------FNQVQLWV-----VTEVCLCGQLSKR------VQLLKKFIKIAAH-
--------------CREFKNLNSFFAIIM-GMSNPAVSRLTQTWE---------------
-------KLPSKFKKFYAE-FESMM--------------------------DPSRNH---
-RAYRL----TVTK-------IEPPII-PFMP----------------------------
LLLKDMTFSHEGNK--TF-ID-----------------------------N-MVNFE-KM
-------RM---IANTIRAVRHCRS--Q-------------------------PFNPD--
-V------------------CQPNKNH-----A----------------EVRG-----YV
RKLCVIDNQRTL-----------------------------------------TTLSYRL
-----------EPRR----------T----------------------------------
------------------------------------------------------------
------------------------------------------------------------
------------------------------------------------------------
------------------------------------------------------------
------------------------------------------------------------
------------------------------------------------------------
------------------------------------------------------------
------------------------------------------------------------
------------------------------------------------------------
```


>Rapgef4_[Eurypyga_helias]_679008399

--ILQD--
---V--------------

------------------------EANTVRLKEHDQDVLVLEK-----IPA-GSRVSNQG
NS----QP-QH------------------------------------K-YIVMSGTPEKI
LEHFLET------------MRPEP-TLN--------------EA-----TDSVLN---D-
FIMMHCV--FM---------------PNSQLCPALM-----A------------------
--------Q-----------YPFHRGTYHAQPSQ---GTEQEKMDYALNNKRRVIRLVLQ
WAALYGDLLQED-EAAMAFLE----EFYVS--VSDDT--RMIAALK---EQ-LPELEKIV
KQL---SEE--PK---------APQKK---------------------------------
---QKV--------LLQLFNTGD-DRAQ-KRQ----------------------------

-----PIRGSDE--VL-----------------------------FK-------------
VYCIDQTYTTIRVPVSSSVKEVIS--------AVADK-----------LGSG-EG--LII
VKMSSG----------------------GEKVV------LKPHDVSVFTTLSINGRLFAC
PRDQ---F-DSLA-PLPEQ---------------------------EGP------SAGTV
GT--FELMSSKDLAHQMTVYDWELFNCVHELELIY-------HTFGRHNF-KK-TTANLD
LFLRR---------FNEIQFWV-----VTEICLCSQLSKR------VQLLKKYIKIAAH-
--------------CKEYKNLNSFFAIIM-GLSNVAVSRLLLTWE---------------
-------KLPSKFKKIYAE-FESLM--------------------------DPSRNH---
-RAYRL----TVAK-------LDPPII-PFMP----------------------------
LLIKDMTFTHEGNK--TF-TD-----------------------------N-LVNFE-KM
-------RM---IANTVRTVKFCRS--Q-------------------------SFNPD--
-A------------------ALTSKNH-----Q----------------DVRS-----YV
RQLNVIDNQRTL--SQMSHRL
-----------EPRR----------A----------------------------------

>Rapgef4_[Falco_peregrinus]_909796784

---MSG-L
LTPPYG--VMETGS--N-N--------DRMPDKD--------------------------
------------------N---
---VPS----------------
-------EKIL-------------RA---------------------GKILR-NTILSRA
PH-M----------------IRDRKYH------L-KTY----------------RQC-CV
GT-----------------ELVDWMMQQS-------------------------------
---PCVH--------SRTQAVGMW---QVLLEEGVLN-----------------------
--------------H------------------------VDQ-EHHFQDKY-LFYRFLDD
-------ERED----AP-L---
--PT
EEEK--KECDEEL------QDTMLLLSQIGPDAH-------------MRMILRKPPGQRT
VDDLE-----FIYEELLHIKALSHLS-TTVKRELA-GVLIFESHPKAGTVL---------
--FNQGEEG--TSWYIIL
KGSVNVVIYGK-----GV-------------------------------VCTLHEG-DDF
GKLAL-----------VNDAPRA---ASIVLREDNCHFLRVDKEDFNR------ILRD--
---V--------------

------------------------EANTVRLKEHDQDVLVLEK-----IPA-GNRVSNQG
NS----QP-QH------------------------------------K-YIVMSGTPEKI
LEHFLET------------MRLEA-TLN--------------EA-----TDSVLN---D-
FIMMHCV--FM---------------PNSQLCPALM-----A------------------
--------H-----------Y-------HAQPSQ---GTEQEKMDYALNNKRRVIRLVLQ
WAALYGDLLQED-EAAMAFLE----EFYVS--VSDDT--RMIAALK---EQ-LPELEKTV
KQV---SEE--PK---------APQKK---------------------------------
---HKV--------LLQLFNTSD-DRAQ-KRQ----------------------------

-----PIRGSDE--VL-----------------------------FK-------------
VYCIDQTYTTIRVPVSSSVKEVIS--------AVADK-----------LGSG-EG--LII
VKMSSG----------------------GEKVV------LKPHDVSVFTTLSVNGRLFAC
PRDQ---F-DSLA-PLPEQ---------------------------EGP------STGTV
GT--FELMSSKDLAHQMTIYDWELFNCVHELELIY-------HTFGRHNF-KK-TTANLD

```
LFLRR---------FNEIQFWV-----VTEICLCSQLSKR------VQLLKKYIKIAAH-
--------------CKEYKNLNSFFAIIM-GLSNVAVSRLSLTWE---------------
-------KLPSKFKKIYAE-FESLM--------------------------DPSRNH---
-RAYRL----TVAK-------LDPPII-PFMP----------------------------
LLIKDMTFTHEGNK--TF-TD-----------------------------N-LVNFE-KM
-------RM---IANTVRTVKFCRS--Q-------------------------SFNPD--
-V------------------ALTNKNH-----Q----------------DVRS-----YV
RQLNVIDNQRTL-----------------------------------------SQMSHRL
-----------EPRR----------A----------------------------------
------------------------------------------------------------
------------------------------------------------------------
------------------------------------------------------------
------------------------------------------------------------
------------------------------------------------------------
------------------------------------------------------------
------------------------------------------------------------
------------------------------------------------------------
------------------------------------------------------------
------------------------------------------------------------
---
>Rapgef4_[Felis_catus]_755759060
------------------------------------------------------------
------------------------------------------------------------
------------------------------------------------------------
------------------------------------------------------------
-------------------------------------------------------MAG-L
LAPPYG--VMETGS--N-N--------DRIPDKE--------------------------
------------------N-----------------------------------------
-----------------------------------------VPS----------------
-------EKIL-------------RA---------------------GKILR-NAILSRA
PH-M----------------IRDRKYH------L-KTY----------------RQC-CV
GT-----------------ELVDWMLQQT-------------------------------
---PCVH--------SRTQAVGMW---QVLVEDGVLN-----------------------
--------------H------------------------VDQ-EHHFQDKY-LFYRFLDD
-------EHED----AP-L-----------------------------------------
----------------------------------------------------------PT
EEEK--KECDEEL------QDTMLLLSQMGPDAH-------------MRMTLRKPPGQRT
VDDLE-----IIYEELIHIKALSHLS-TTVKRELA-GVLIFESHAKGGTVL---------
--------------------------------------------FNQGEEG--TSWYIIL
KGSVNVVIYGK-----GV-------------------------------VCTLHEG-DDF
GKLAL-----------VNDAPRA---ASIVLREDNCHFLRVDKEDFNR------ILRD--
---------------------------------------------V--------------
------------------------------------------------------------
------------------------------------------------------------
------------------------EANTVRLKEHDQDVLVLEK-----VPA-GNRASNQG
NS----QP-QQ------------------------------------K-YTVMSGTPEKI
LEHFLET------------IRLEP-ALN--------------EA-----TDSILN---D-
FVMMHCV--FM---------------PNTQLCPALV-----A------------------
--------H-----------Y-------HAQPSQ---GTEQEKTDYTLNNKRRVIRLVLQ
WAAVHGDVLQED-DVAVAFLE----EFYVS--VSDDA--RMIAALK---EQ-LPELEKIV
KQI---SED--AK---------APQKK---------------------------------
---HKV--------LLQQFNTGD-ERAQ-KRQ----------------------------
------------------------------------------------------------
------------------------------------------------------------
```


-----PVRGSDE--VL-----------------------------FK-------------
VYCMDHTYTTIRVPVAASVKEVIS--------AVADK-----------LGSG-EG--LII
VKMSSG----------------------GEKVV------LKPNDVSVFTTLTINGRLFAC
PREQ---F-DSLT-PLPEQ---------------------------EGP------TVGTM
GT--FELMSSKDLAYQMTIYDWELFNCVHELELIY-------HTFGRHNF-KK-TTANLD
LFLRR---------FNEIQFWV-----VTEICLCSQPSKR------VQLLKKFIKIAAH-
--------------CKEYKNLNSFFAIVM-GLSNVAVSRLALTWE---------------
-------KLPSKFKKFYAE-FESLM--------------------------DPSRNH---
-RAYRL----TVAK-------LEPPLI-PFMP----------------------------
LLIKDMTFTHEGNK--TF-ID-----------------------------N-LVNFE-KM
-------RM---IANTARTVRYCRS--Q-------------------------PFNPD--
-A------------------AQANKNH-----Q----------------DVRS-----YV
RQLNVIDNQRTL---SQMSHRL
-----------EPRR----------P----------------------------------

>Rapgef4_[Ficedula_albicollis]_1020984796
----------------------------MVAA-------HA------SH--------SSS
SGEWIA---CLDK-----------RPLE-----RSSEDVDIIFTRLKEVKAFEKFHPNLL
QQICLCGY--YENLE--KGITLFRQGDIGTNWYAVLTGSLDVKVSD-TSNHQDA------
------VTICTLGIGTAFGESILDNT-PRHATIV-TREHSELL-----------------
----------------------------RIEQKD-------FKALW---EKYRQYMSG-L
LTPPYG--VMETGS--N-N--------DRMPDKD--------------------------
------------------SMS-------SSALCQVSKNCSKT------------------
-----PLIGPHIPHHPTK-TITQ------------------VPS----------------
-------EKIL-------------RA---------------------GKILR-NTILSRA
PH-M----------------IRDRKYH------L-KTY----------------RQC-CV
GT-----------------ELVDWMMQQS-------------------------------
---PCVH--------LRSQAVGMW---QVLLEEGVLN-----------------------
--------------H------------------------VDQ-EHHFQDKY-LFYRFLDD
-------ERED----AP-L---
--PT
EEEK--KECDEEL------QDTMLLLSQIGPDAH-------------MRMILRKPPGQRT
VDDLE-----FIYEELLHIKALSHLS-TTVKRELA-GVLIFESHPKAGTVL---------
--FNQGEEG--TSWYIIL
KGSVNVVIYGK-----GV-------------------------------VCTLHEG-DDF
GKLAL-----------VNDAPRA---ASIVLREDNCHFLRVDKEDFNR------ILRD--
---V--------------

------------------------EANTVRLKEHDQDVLVLEK-----IPA-GNRISNQG

SS----QP-QH------------------------------------K-YIVMSGTPEKI
LEHFLET------------MRLEA-TLN--------------EA-----TDSVLN---D-
FIMMHCV--FM---------------PNSQLCPALM-----A------------------
--------H-----------Y-------HAQPSQ---GTEQEKMDYALNNKRRVIRLVLQ
WAALYGDLLQED-EAAMAFLE----EFYVS--VSDDT--RMIAALK---EQ-LPELEKIV
KQI---SEE--PK---------APQKK---------------------------------
---HKV--------LLQLFNTSD-DRAQ-KRQ----------------------------

-----PIRGSDE--VL-----------------------------FK-------------
VYCIDQTYTTIRVPVSSSVKEVIS--------AVADK-----------LGSG-EG--LII
VKMSSG----------------------GEKVV------LKPHDVSVFTTLSVNGRLFAC
PRDQ---F-DSLA-PLPEQ---------------------------EGP------SAGTV
GT--FELMSSKDLAHQMTIYDWELFNCVHELELIY-------HTFGRHNF-KK-TTANLD
LFLRR---------FNEIQFWV-----VTEICLCTQLSKR------VQLLKKYIKIAAH-
--------------CKEYKNLNSFFAIIM-GLSNVAVSRLSLTWE---------------
-------KLPSKFKKIYAE-FESLM--------------------------DPSRNH---
-RAYRL----TVAK-------LDPPII-PFMP----------------------------
LLIKDMTFTHEGNK--TF-TD-----------------------------N-LVNFE-KM
-------RM---IANTVRTVKFCRS--Q-------------------------SFNPD--
-A------------------ALTNKNH-----Q----------------DVRS-----YV
RQLNVIDNQRTL---SQMSHRL
-----------EPRR----------A----------------------------------

>Rapgef4_[Fukomys_damarensis]_731272285

---MAG-L
LAPPYG--AMETGS--N-N--------DRIPDKE--------------------------
------------------N---
---VPS----------------
-------EKIL-------------RA---------------------GKILR-NAILSRA
PH-M----------------IRDRKYH------L-KTY----------------RQC-CV
GT-----------------ELVDWMLQQT-------------------------------
---PCVH--------SRTQAVGMW---QVLLEDGVLN-----------------------
--------------H------------------------VDQ-EHHFQDKY-LFYRFLDD
-------EHED----AP-L---
--PT

```
EEEK--KECDEEL------QDTMLLLSQMGPDAH-------------MRMILRKPPGQRT
VDDLE-----IIYEELLHIKALSHLS-TTVKRELA-GVLIFESHAKGGTVL---------
--------------------------------------------FNQGEEG--TSWYIIL
KGSVNVVIYGK-----GV-------------------------------VCTLHEG-DDF
GKLAL-----------VNDAPRA---ASIVLREDNCHFLRVDKEDFNR------ILRD--
---------------------------------------------V--------------
------------------------------------------------------------
------------------------------------------------------------
------------------------EANTVRLKEHDQDVLVLEK-----VPA-GNRASNQG
NS----QP-QQ------------------------------------K-YTVMSGTPEKI
LEHFLET------------IRLEP-ALN--------------EA-----ADSVLN---D-
FVMMHCV--FM---------------PNTQLCPALV-----A------------------
--------H-----------Y-------HAQPSQ---GTEQERLDYALNNKRRVIRLVLQ
WAATYGDLLQED-DVSMAFLE----EFYVS--VSDDA--RMIAAFK---EQ-LPELEKIV
KQT---SED--AK---------GPQKK---------------------------------
---HKV--------LLQQFNTSD-ERAQ-KRQ----------------------------
------------------------------------------------------------
------------------------------------------------------------
------------------------------------------------------------
------------------------------------------------------------
------------------------------------------------------------
------------------------------------------------------------
-----PIRGSDE--VL-----------------------------FK-------------
VYCMDHTYTTIRVPVAASVKEVIS--------AVADK-----------LGSG-EG--LII
VKMSSG----------------------GEKVV------LKPNDVSVFTTLTINGRLFAC
PREQ---F-DSLT-PLPEQ---------------------------DGP------TVGTV
GT--FELMSSKDLAYQMTIYDWELFNCVHELELIY-------HTFGRHNF-KK-TTANLD
LFLRR---------FNEIQFWV-----VTEICLCSQLSKR------VQLLKKFIKIAAH-
--------------CKEYKNLNSFFAIVM-GLSNVAVSRLALTWE---------------
-------KLPSKFKKFYAE-FESLM--------------------------DPSRNH---
-RAYRL----TAAK-------LEPPLI-PFMP----------------------------
LLIKDMTFTHEGNK--TF-ID-----------------------------N-LVNFE-KM
-------RM---IANTARTVRYYRS--Q-------------------------PFNPD--
-A------------------AQANKNH-----Q----------------DVRS-----YV
RQLNVIDNQRTL-----------------------------------------SQMSHRL
-----------EPRR----------P----------------------------------
------------------------------------------------------------
------------------------------------------------------------
------------------------------------------------------------
------------------------------------------------------------
------------------------------------------------------------
------------------------------------------------------------
------------------------------------------------------------
------------------------------------------------------------
------------------------------------------------------------
------------------------------------------------------------
---
>Rapgef4_[Fundulus_heteroclitus]_831555062
------------------------------------------------------------
------------------------------------------------------------
------------------------------------------------------------
------------------------------------------------------------
------------------------------------------------------------
---------MESGS----NN-------DRLTDKD--------------------------
```

```
------------------NMN-------S-------------------------------
--------DSG---NKAH-----S----------------KVPS----------------
-------EKLQ-------------RA---------------------GKVLR-NAILSRA
PH-M----------------IRDRKYH------L-KTY----------------RQC-CV
GT-----------------ELVDWLVMQS-------------------------------
---ACVL--------TRSHAVGMW---QALLEEGVLN-----------------------
--------------H------------------------VDQ-DLGFHDKY-LFYRFLDD
-------EEED----TP-L-----------------------------------------
----------------------------------------------------------PS
EEEK--RESEEEL------PETILFLAQIGPDAL-------------LRMILRKPPGQRT
ADDLE-----IIYDELLHIKALSHLS-NTVKRELA-SVVIFESHAKAGTVL---------
--------------------------------------------FNQGEEG--TSWYIIQ
KGSVNVVIYGK-----GV-------------------------------VCTLHEG-DDF
GKLAL-----------VTDSPRA---ASIVLREDNCHFLRVDKEDFNR------ILRD--
---------------------------------------------V--------------
------------------------------------------------------------
------------------------------------------------------------
------------------------EANTVRLKEHEQAVLVLEK-----S----PRSSTLG
S---------I------------------------------------K-YTVISGTPEKI
LEHFLET------------MRLDI-HHN--------------EP------DPAVD---D-
FVLMHCV--FL---------------PNSQLCPLLM-----A------------------
--------H-----------Y-------HVVSPP---GSEQERLEYTLNSKRRALILTLR
WANSHTYLLQEE-PAAISFLE----ELYGS--VSNDS--RTLRGLK---DL-VLDLEKVV
KLH---SEE--VK---------STKKR---------------------------------
-----T--------LIRQFSNGE-ERLQ-KKQ----------------------------
------------------------------------------------------------
------------------------------------------------------------
------------------------------------------------------------
------------------------------------------------------------
------------------------------------------------------------
------------------------------------------------------------
-----PIRNQDD--IL-----------------------------LK-------------
VYCSDHTYTTIRITVAATGREVIS--------AVSEK-----------LGTT-QE--LVL
VHLSSA----------------------GEKQL------LKPNDVSVFSTLSINGRLFAC
PREQ---L-SSLA-PLPDQ---------------------------EGP------SAGSM
ST--FELMSSKDLAYQMTMFDWELFSCVHEHELLY-------HTFGRHSF-RR-TTANMD
LFLRR---------FNQVQLWV-----VTEVCLCTQLSKR------VQLLKKFIKIAAH-
--------------CREFKNLNSFFAIIM-GMSNPAVSRLSQTWE---------------
-------KLPTKFKKFYAE-FESMM--------------------------DPSRNH---
-RSYRL----TVTK-------LEPPII-PFMP----------------------------
LLLKDMTFTHEGNK--TF-ID-----------------------------N-MVNFE-KM
-------RI---IANTIRQVRHCRS--Q-------------------------PFNPD--
-I------------------CQPNKNQ-----A----------------DVRG-----YV
RKLCVIDNQRAL-----------------------------------------TQLSYRL
-----------EPRR----------T----------------------------------
------------------------------------------------------------
------------------------------------------------------------
------------------------------------------------------------
------------------------------------------------------------
------------------------------------------------------------
------------------------------------------------------------
------------------------------------------------------------
------------------------------------------------------------
------------------------------------------------------------
```


>Rapgef4_[Galeopterus_variegatus]_667257960

---M----------------IRDRKYH------L-KTY----------------RQC-CV
GT-----------------ELVDWMMQQT-------------------------------
---PFVH--------SRTQAVGMW---QVLLEDGVLN-----------------------
--------------H------------------------VDQ-EHHFQDKY-LFYRFLDD
-------EHED----AP-L---
--PT
EEEK--KECDEEL------QDTMLLLSQMGPDAH-------------MRMILRKPPGQRT
VDDLE-----IIYEELLHIKALSHLS-TTVKRELA-GVLIFESHAKGGTVL---------
--FNQGEEG--TSWYIIL
KGSVNVVIYGK-----GV-------------------------------VCTLHEG-DDF
GKLAL-----------VNDAPRA---ASIVLREDNCHFLRVDKEDFNR------ILRD--
---V--------------

------------------------EANTVRLKEHDQDVLVLEK-----VPA-GNRASNQG
NS----QP-QQ------------------------------------K-YTVMSGTPEKI
LEHFLET------------VRLEP-ALN--------------EA-----TDSVLS---D-
FVMMHCV--FM---------------PNTQLCPALV-----A------------------
--------H-----------Y-------HAQPSQ---GTEQEKLDYALNNKRRVVRLVLQ
WAAVYGDLLQED-DVAMAFLE----EFYVS--VSDDA--RMMAALK---EQ-LPDLEKIV
KQI---SED--AK---------TPQKK---------------------------------
---HKV--------LLQQFNTSD-ERAQ-RRQ----------------------------

-----PIRGSDE--VL-----------------------------FK-------------
VYCMDHTYTTIRVPVAASVKEVLS--------AVTDK-----------LGSG-EG--LII
VKMSSG----------------------GEKVV------LKPNDVSVFTTLTINGRLFAC
PREQ---F-DSLT-PLPEQ---------------------------EGP------TVGTV
GT--FELMSSKDLAYQMTIYDWELFNCVHELELIY-------HTFGRHNF-KK-TTANLD
LFLRR---------FNEIQFWV-----VTEICLCSQLSKR------VQLLKKFIKIAAH-
--------------CKEYKNLNSFFAIVM-GLSNVAVSRLALTWE---------------
-------VSYMRVQNQSGK-PDDGMTRLGVLGSQSVYMSRVTDQHLSVSPKDPSRNH---
-RAYRL----TVAK-------LEPPLI-PFMP----------------------------
LLIKDMTFTHEGNK--TF-TD-----------------------------N-LVNFE-KM
-------RM---IANTARTVRYYRS--Q-------------------------PFNPD--
-A------------------AQANKNH-----Q----------------DVRS-----YV
RQLNVIDNQRTL---SQMSHRL
-----------EPRR----------P----------------------------------

>Rapgef4_[Gallus_gallus]_971405295

---MSG-L
LTPPYG--VMETGS--N-N--------DRMPDKD--------------------------
------------------SMS-------SGALCQVSKNCNKT------------------
-----PLIDSHIPHRPSK-TITQ------------------VPS----------------
-------EKIL-------------RA---------------------GKILR-NTILSRA
PH-M----------------IRDRKYH------L-KTY----------------RQC-CV
GT-----------------ELVDWMMQQS-------------------------------
---TCVH--------SRSQAVGMW---QVLLEEGVLN-----------------------
--------------H------------------------VDQ-EHHFQDKY-LFYRFLDD
-------ERED----AP-L---
--PT
EEEK--KECDEEL------QDTMLLLSQIGPDAH-------------MRMILRKPPGQRS
IDDLE-----IIYEELLHIKALSHLS-TTVKRELA-GVLIFESHPKAGTVL---------
--FNQGEEG--TSWYIIL
KGSVNVVIYGK-----GV-------------------------------VCTLHEG-DDF
GKLAL-----------VNDAPRA---ASIVLREDNCHFLRVDKEDFNR------ILRD--
---V--------------

------------------------EANTVRLKEHDQDVLVLEK-----IPA-GNRVSNQG
SS----QP-QH------------------------------------K-YIVMSGTPEKI
LEHFLET------------MRLEA-TLN--------------EA-----TDSVLN---D-
FIMMHCV--FM---------------PNSQLCPALM-----A------------------
--------H-----------Y-------HAQPSQ---GTEQEKMDYALNNKRRVIRLVLQ
WAALYGDLLQED-EAAMAFLE----EFYVS--VSDDT--RMIAALK---EQ-LPELEKIV
KQV---AEE--PK---------APQKK---------------------------------
---HKV--------LLQLFNTSD-DRAQ-KRQ----------------------------

-----PIRGSDE--VL-----------------------------FK-------------
VYCIDQTYTTIRVPVSSSVKEVIS--------AVADK-----------LGSG-EG--LII
VKMSSG----------------------GEKVV------LKPHDVSVFTTLSVNGRLFAC
PRDQ---F-DSLA-PLPEQ---------------------------EGP------SAGTV
GM--FELMSSKDLAHQMTIYDWELFNCVHELELIY-------HTFGRHNF-KK-TTANLD

LFLRR---------FNEIQFWV-----VTEICLCSQLSKR------VQLLKKYIKIAAH-
--------------CKEYKNLNSFFAIIM-GLSNVAVSRLSLTWE---------------
-------KLPSKFKKIYAE-FESLM--------------------------DPSRNH---
-RAYRL----TVAK-------LDPPII-PFMP----------------------------
LLIKDMTFTHEGNK--TL-TD-----------------------------N-LVNFE-KM
-------RM---IANTVRTVKFCRS--Q-------------------------SFNPD--
-A------------------ALANKNH-----Q----------------DVRS-----YV
RQLNVIDNQRTL---SQMSHRL
-----------EPRR----------A----------------------------------

>Rapgef4_[Gavia_stellata]_698431451

------------MTSFE--N-ITARPLE-----RSSEDVDIIFTRLKEVK----------

--------------------AFEKFH-PNLLQQIC--LCGYYENLEKGITL---------
--FRQGDIG--TNWYAVL
TGSLDVKVSDTSNHQDAVT------------------------------ICTLGIG-TAF
GE-SI-----------LDNTPRH---ATIVTRE-YSELLRIEQKDFKA------LWE---

>Rapgef4_[Gorilla_gorilla_gorilla]_426337729

---MAG-L
LAPPYG--VMETGS--N-N--------DRIPDKE--------------------------
------------------N----------------------T------------------
-----PLIEPHVPLRPAN-TITK------------------VPS----------------
-------EKIL-------------RA---------------------GKILR-NAILSRA
PH-M----------------IRDRKYH------L-KTY----------------RQC-CV
GT-----------------ELVDWMMQQT-------------------------------
---PCVH--------SRTQAVGMW---QVLLEDGVLN-----------------------
--------------H------------------------VDQ-EHHFQDKY-LFYRFLDD
-------EHED----AP-L---
--PT
EEEK--KECDEEL------QDTMLLLSQMGPDAH-------------MRMILRKPPGQRT
VDDLE-----IIYEELLHIKALSHLS-TTVKRELA-GVLIFESHAKGGTVL---------
--FNQGEEG--TSWYIIL
KGSVNVVIYGK-----GV-------------------------------VCTLHEG-DDF
GKLAL-----------VNDAPRA---ASIVLREDNCHFLRVDKEDFNR------ILRD--
---V--------------

------------------------EANTVRLKEHDQDVLVLEK-----VPA-GNRASNQG

NS----QP-QQ------------------------------------K-YTVMSGTPEKI
LEHFLET------------IRLEA-TLN--------------EA-----TDSVLN---D-
FIMMHCV--FM---------------PNTQLCPALV-----A------------------
--------H-----------Y-------HAQPSQ---GTEQEKMDYALNNKRRVIRLVLQ
WAAMYGDLLQED-DVSMAFLE----EFYVS--VSDDA--RMIAALK---EQ-LPELEKIV
KQI---SED--AK---------APQKK---------------------------------
---HKV--------LLQQFSTGD-ERAQ-KRQ----------------------------

-----PIRGSDE--VL-----------------------------FK-------------
VYCMDHTYTTIRVPVATSVKEVIS--------AVADK-----------LGSG-EG--LII
VKMSSG----------------------GEKVV------LKPNDVSVFTTLTINGRLFAC
PREQ---F-DSLT-PLPEQ---------------------------EGP------TVGTV
AT--FELMSSKDLAYQMTIYDWELFNCVHELELIY-------HTFGRHNF-KK-TTANLD
LFLRR---------FNEIQFWV-----VTEICLCSQLSKR------VQLLKKFIKIAAH-
--------------CKEYKNLNSFFAIVM-GLSNVAVSRLALTWE---------------
-------KLPSKFKKFYAE-FESLM--------------------------DPSRNH---
-RAYRL----TVAK-------LEPPLI-PFMP----------------------------
LLIKDMTFTHEGNK--TF-ID-----------------------------N-LVNFE-KM
-------RM---IANTARTVRYYRS--Q-------------------------PFNPD--
-A------------------AQANKNH-----Q----------------DVRS-----YV
RQLNVIDNQRTL---SQMSHRL
-----------EPRR----------P----------------------------------

>Rapgef4_[Haliaeetus_albicilla]_700359609

---MSG-L
LTPPYG--VMETGS--N-N--------DRMPDKD--------------------------
------------------N----------------------T------------------
-----PLIEPHIPHRPTK-TNTQ------------------VPS----------------
-------EKIL-------------RA---------------------GKILR-NTILSRA
PH-M----------------IRDRKYH------L-KTY----------------RQC-CV
GT-----------------ELVDWMMQQS-------------------------------
---PCVH--------SRTQAVGMW---QVLLEEGVLN-----------------------
--------------H------------------------VDQ-EHHFQDKY-LFYRFLDD
-------ECED----AP-L---
--PT

EEEK--KECDEEL------QDTMLLLSQIGPDAH-------------MRMILRKPPGQRT
VDDLE-----FIYEELLHIKALSHLS-TTVKRELA-GVLIFESHPKAGTVL---------
--FNQGEEG--TSWYIIL
KGSVNVVIYGK-----GV-------------------------------VCTLHEG-DDF
GKLAL-----------VNDAPRA---ASIVLREDNCHFLRVDKEDFNR------ILRD--
---V--------------

------------------------EANTVRLKEHDQDVLVLEK-----IPA-GNRVSNQG
NS----QP-QH------------------------------------K-YIVMSGTPEKI
LEHFLET------------MRLEA-TLN--------------EA-----TDSVLN---D-
FIMMHCV--FM---------------PNSQLCPALM-----A------------------
--------H-----------Y-------HAQPSQ---GTEQEKMDYALNNKRRVIRLVLQ
WAALYGDLLQED-EAAMAFLE----EFYVS--VSDDT--RMIAALK---EQ-LPELEKIV
KQV---SEE--PK---------APQKK---------------------------------
---HKV--------LLQLFNTSD-DRTQ-KRQ----------------------------

-----PIRGSDE--VL-----------------------------FK-------------
VYCIDQTYTTIRVPVSSSVKEVIS--------AVADK-----------LGSG-EG--LII
VKMSSG----------------------GEKVV------LKPHDVSVFTTLSVNGRLFAC
PRDQ---F-DSLA-PLPEQ---------------------------EGP------STGTV
GT--FELMSSKDLAHQMTIYDWELFNCVHELELIY-------HTFGRHNF-KK-TTANLD
LFLRR---------FNEIQFWV-----VTEICLCSQLSKR------VQLLKKYIKIAAH-
--------------CKEYKNLNSFFAIIM-GLSNVAVSRLSLTWE---------------
-------KLPSKFKKIYAE-FESLM--------------------------DPSRNH---
-RAYRL----TVAK-------LDPPII-PFMP----------------------------
LLIKDMTFTHEGNK--TF-TD-----------------------------N-LVNFE-KM
-------RM---IANTVRTVKFCRS--Q-------------------------SFNPD--
-A------------------ALTNKNH-----Q----------------DVRS-----YV
RQLNVIDNQRTL---SQMSHRL
-----------EPRR----------A----------------------------------

>Rapgef4_[Haliaeetus_leucocephalus]_729721424
----------------------------MVAA-------HA------SH--------SSS
SGEWIA---CLDK-----------RPLE-----RSSEDVDIIFTRLKEVKAFEKFHPNLL
QQICLCGY--YENLE--KGITLFRQGDIGTNWYAVLTGSLDVKVSD-TSNHQDA------
------VTICTLGIGTAFGESILDNT-PRHATIV-TREYSELL-----------------
----------------------------RIEQKD-------FKALW---EKYRQYMSG-L
LTPPYG--VMETGS--N-N--------DRMPDKD--------------------------

```
------------------N----------------------T------------------
-----PLIEPHIPHRPTK-TNTQ------------------VPS----------------
-------EKIL-------------RA---------------------GKILR-NTILSRA
PH-M----------------IRDRKYH------L-KTY----------------RQC-CV
GT-----------------ELVDWMMQQS-------------------------------
---PCVH--------SRTQAVGMW---QVLLEEGVLN-----------------------
--------------H------------------------VDQ-EHHFQDKY-LFYRFLDD
-------ECED----AP-L-----------------------------------------
----------------------------------------------------------PT
EEEK--KECDEEL------QDTMLLLSQIGPDAH-------------MRMILRKPPGQRT
VDDLE-----FIYEELLHIKALSHLS-TTVKRELA-GVLIFESHPKAGTVL---------
--------------------------------------------FNQGEEG--TSWYIIL
KGSVNVVIYGK-----GV-------------------------------VCTLHEG-DDF
GKLAL-----------VNDAPRA---ASIVLREDNCHFLRVDKEDFNR------ILRD--
---------------------------------------------V--------------
------------------------------------------------------------
------------------------------------------------------------
------------------------EANTVRLKEHDQDVLVLEK-----IPA-GNRVSNQG
NS----QP-QH------------------------------------K-YIVMSGTPEKI
LEHFLET------------MRLEA-TLN--------------EA-----TDSVLN---D-
FIMMHCV--FM---------------PNSQLCPALM-----A------------------
--------H-----------Y-------HAQPSQ---GTEQEKMDYALNNKRRVIRLVLQ
WAALYGDLLQED-EAAMAFLE----EFYVS--VSDDT--RMIAALK---EQ-LPELEKIV
KQV---SEE--PK---------APQKK---------------------------------
---HKV--------LLQLFNTSD-DRTQ-KRQ----------------------------
------------------------------------------------------------
------------------------------------------------------------
------------------------------------------------------------
------------------------------------------------------------
------------------------------------------------------------
------------------------------------------------------------
-----PIRGSDE--VL-----------------------------FK-------------
VYCIDQTYTTIRVPVSSSVKEVIS--------AVADK-----------LGSG-EG--LII
VKMSSG----------------------GEKVV------LKPHDVSVFTTLSVNGRLFAC
PRDQ---F-DSLA-PLPEQ---------------------------EGP------STGTV
GT--FELMSSKDLAHQMTIYDWELFNCVHELELIY-------HTFGRHNF-KK-TTANLD
LFLRR---------FNEIQFWV-----VTEICLCSQLSKR------VQLLKKYIKIAAH-
--------------CKEYKNLNSFFAIIM-GLSNVAVSRLSLTWE---------------
-------KLPSKFKKIYAE-FESLM--------------------------DPSRNH---
-RAYRL----TVAK-------LDPPII-PFMP----------------------------
LLIKDMTFTHEGNK--TF-TD-----------------------------N-LVNFE-KM
-------RM---IANTVRTVKFCRS--Q-------------------------SFNPD--
-A------------------ALTNKNH-----Q----------------DVRS-----YV
RQLNVIDNQRTL-----------------------------------------SQMSHRL
-----------EPRR----------A----------------------------------
------------------------------------------------------------
------------------------------------------------------------
------------------------------------------------------------
------------------------------------------------------------
------------------------------------------------------------
------------------------------------------------------------
------------------------------------------------------------
------------------------------------------------------------
------------------------------------------------------------
```


>Rapgef4_[Halyomorpha_halys]_939661167
-------MA---------------------TTQWILAF----------------------
----------------------DRRPGD-----RSVRDVELICGRLRRLEALSRLPQQVI
AHLAHHAF--YEDVE--RGVTLFRQGDIGTSWYMILAGKVEVRAIQ-----DK------S
M-----VTLCTLGVGGSFGEGVLHDL-PRDSTVI-TRTSCELLRLTHQDLKALAE-----
KNKE--IMCDLI--------------S-NCKMKNGLGGVV-K-S-Q--------SP----
-SPPLPQA---------------K--RGLSPDESDPAR----------------------

--PITE--------------
-----------------MPSMEMARA---------------------GWVLR-TLLLNEQ
DN-V----------------LRERKTAG-----G-R-L---------------SVPMAAS
GS-----------------ELVTWLISLE-------------------------------
---AGL---------DRHRATTMW---QALLEEGIIY-----------------------
--------------H------------------------VTG-EHEFKDKC-VLYQFRSD
R---------D----SG---
--GGGCPT
PQDI--AEAEEHL------QECLADLADRGPDAW-------------LRMVLRKPSNDRT
ADDLE-----IIYEELLHMKPLSHLS-NSMKRELA-SVILFEAHPRPGTVV---------
--FEQGEEG--RSWYLVL
CGSVDVVIHGK-----GV-------------------------------VATLHSG-EDF
GKLAL-----------INEAPRA---ASIIVREENTHLLRVDKDDFNR------ILRD--
---V--------------

------------------------EANTVRLKEHGKDVLILE--------K---------
------SQGP----HS---------------SVK---------------YVVMACTPARM
VEHLLDS-------------RLDS-G-----------SI-----------DPALD---D-
FLLTHVI--FI---------------TTRHLVQELK-----K------------------
--------HY----------------------NM---ESPHHDKEHTDNYKRRVLLFVYR
WVTTIRYPALSD-PIIISFIE----ELSND--ASSEG--------W-----------EEA
ALM---
---HRL--------SAQVARQQE---INSNLNK---------------------------
----WKLPP---TGQPIC--------------------------------LFSPSET---
---GS-T---L---

------IKPKDD--II-----------------------------FR-------------
VYCADHTYCTLRLPVSATAEQIKV--------CAADK-----------LGLRVRPEDLIL
AELKSN----------------------GERVN------FRDVEVSVPTSLSLNGRIFVA
PKDH---L-DALT-VIAEQ---------------------------EAP------TEGVE
L--DLEDVSAKEMAYHMTMFDWELFWNIHEYELLY-------HTFGRHRF--GQSTANLD
AFLRR---------FNELQYWV-----VTEICLTQSLSKR------VQVLRKIIKLAAY-
--------------CKQYHNINGMFALVL-GLSNMAVSRLSLTWD---------------
-------KLPSKSRKLYMQ-LEATI--------------------------DPSKNH---
-RAYRG----VVSS-------MDSPLI-PFMP----------------------------
LLLKDMTFIHEGNK--TM-VD-----------------------------G-LVNFE-KM
-------HM---LAQTLRTLRYCRS--R------QLIIE----------------DPP--
----------------------TPKNE-----S----------------EVRA-----YV
RCLRTIDNQRTL---TSLSQKL
-----------EPRR----------S----------------------------------

>Rapgef4_[Haplochromis_burtoni]_554821688

---MAG-L
LAPPYG--VME--S--GCNA-------DRLPDKE--------------------------
------------------NIS-------NNSFV-VSKPLNKK------------------
-----SFIQSP---AKPH-PKCFS----------------QVPS----------------
-------EKIL-------------RA---------------------GQILR-NAILSRA
PH-M----------------IRDRKYH------L-KTY----------------RRC-CV
GT-----------------ELVDWQLQQS-------------------------------
---SCVH--------SRVQAVGMW---QVLLEEGVLN-----------------------
--------------H------------------------VDQ-ELSFQDKY-LFYRFLDD
-------EQED----AS-F---
--PT
EDER--RESQEEL------QDTLLLLSQIGPDAH-------------LRMILRKPPSERT
ADDLE-----IIYEELLHIKALSHLS-TTVKRELA-GVLIFESHAKAGTVL---------
--FNQGEEG--TSWYIIL
KGSVNVVIYGK-----GV-------------------------------VCTLHEG-DDF
GKLAL-----------VNDAPRA---ASIVLREDNCHFLRVDKEDFNR------ILRD--
---V--------------

------------------------EANTVRLKEHGQDVLVLEK-----SLS-SSRTSVQG
SS----SS-HY------------------------------------K-YNVMSGTPEKI
LEHLLEM------------MRLDS-QFT--------------ES------DSALD---D-
FVLMHCV--FI---------------PNSQLCPVLM-----S------------------
--------H-----------Y-------HAQASQ---GSEQEKLDYTLNNKRRVIRLVMH
WAAVNGDHLQEE-DVSGLFLE----EFFMA--VSNDA--KAFPALR---DQ-LTELERIV
KRN---AED--AR---------SSQKK---------------------------------
---HKV--------LLRHFSVGE-EKLQ-KRQ----------------------------

-----PIRSNDE--IL-----------------------------FK-------------
VYCCDHTYITIRVPVASSVREVIG--------AVADK-----------LGSA-ED--LLL
VGLSSA----------------------GEKTI------FKPSDMSVFSTLSINGRLFVC
RRDQ---L-DSLT-TLPEQ---------------------------EGP------STGSL
AS--FELMSSKDVAYHMTSYDWELFHCVHELELIY-------HTFGRQYV-KK-TTVNLD

LFLRR---------FNEIQFWV-----ITEMCLCPQLSKR------VQLLKKFIKIAAH-
--------------CKEYRNLNAFFAIIM-GLSNPAVCRLSQTWE---------------
-------KLPSKFKKFYVE-FENLM--------------------------DPSRNH---
-RAYRL----TVAK-------LEPPII-PFMP----------------------------
LLIKDMTFTHEGNK--TF-ID-----------------------------S-LVNFE-KM
-------RM---IANTVKIVRYCRS--Q-------------------------RFSPE--
-S------------------PLPSKNH-----P----------------DAWT-----YI
RQLNVIDNQRML---TQLSHGL
-----------EPRR----------S----------------------------------

>Rapgef4_[Heterocephalus_glaber]_512992186
----------------------------MVAA-------HA------AH--------SSS
SAEWIA---CLDK-----------RPLE-----RSSEDVDIIFTRLKEVKAFEKFHPNLL
HQICLCGY--YENLE--KGITLFRQGDIGTNWYAVLAGSLDVKVSE-TSSHQDA------
------VTICTLGIGTAFGESILDNT-PRHATIV-TRESSELL-----------------
----------------------------RIEQKD-------FKALW---EKYRQYMAG-L
LAPPYG--VMETGS--N-N--------DRIPDKE--------------------------
------------------N----------------------T------------------
-----PLIEPHVPLRPAN-TITK------------------VPS----------------
-------EKIL-------------RA---------------------GKILR-NAILSRA
PH-M----------------IRDRKYH------L-KTY----------------RQC-CV
GT-----------------ELVDWMLQQT-------------------------------
---PCVH--------SRTQAVGMW---QVLLEDGVLN-----------------------
--------------H------------------------VDQ-EHHFQDKY-LFYRFLDD
-------EHED----AP-L---
--PT
EEEK--KECDEEL------QDTMLLLSQMGPDAH-------------MRMILRKPPGQRT
VDDLE-----IIYEELLHIKALSHLS-TTVKRELA-GVLIFESHAKGGTVL---------
--FNQGEEG--TSWYIIL
KGSVNVVIYGK-----GV-------------------------------VCTLHEG-DDF
GKLAL-----------VNDAPRA---ASIVLREDNCHFLRVDKEDFNR------ILRD--
---V--------------

------------------------EANTVRLKEHDQDVLVLEK-----VPA-GNRASNQG
NS----QP-QQ------------------------------------K-YTVMSGTPEKI
LEHFLET------------IRLEP-ALN--------------EA-----TDSVLN---D-
FVMMHCV--FM---------------PNTQLCPALV-----A------------------
--------H-----------Y-------HAQPSQ---GTEQEKLDYGLNNKRRVIRLVLQ
WAATYGDLLQED-DVAMAFLE----EFYVS--VSDDA--RMIAAFK---EQ-LPELEKIV
KQI---SED--AK---------APQKK---------------------------------
---HKV--------LLQQFNTSD-ERAQ-KRQ----------------------------

-----PIRGSDE--VL-----------------------------FK-------------
VYCMDHTYTTIRVPVAASVKEVIS--------AVADK-----------LGSG-EG--LII
VKMSSG----------------------GEKVV------LKPNDVSVFTTLTINGRLFAC
PREQ---F-DSLT-PLPEQ---------------------------EGP------TVGTV
GT--FELMSSKDLAYQMTIYDWELFNCVHELELIY-------HTFGRHNF-KK-TTANLD
LFLKR---------FNEIQFWV-----VTEICLCSQLSKR------VQLLKKFIKIAAH-
--------------CKEYKNLNSFFAIVM-GLSNVAVSRLALTWE---------------
-------KLPSKFKKFYAE-FESLM--------------------------DPSRNH---
-RAYRL----TAAK-------LEPPLI-PFMP----------------------------
LLIKDMTFTHEGNK--TF-ID-----------------------------N-LVNFE-KM
-------RM---IANTARTVRYYRS--Q-------------------------PFNPD--
-A------------------AQANKNH-----Q----------------DVRS-----YV
RQLNVIDNQRTL---SQMSHRL
-----------EPRR----------P----------------------------------

>Rapgef4_[Homo_sapiens]_608785455
----------------------------MVAA-------HG------AH--------SSS
SAEWIA---CLD----------------------------IIFTRLKEVKAFEKFHPNLL
HQICLCGY--YENLE--KGITLFRQGDIGTNWYAVLAGSLDVKVSE-TSSHQDA------
------VTICTLGIGTAFGESILDNT-PRHATIV-TRESSELL-----------------
----------------------------RIEQKD-------FKALW---EKYRQYMAG-L
LAPPYG--VMETGS--N-N--------DRIPDKE--------------------------
------------------N----------------------T------------------
-----PLIEPHVPLRPAN-TITK------------------VPS----------------
-------EKIL-------------RA---------------------GKILR-NAILSRA
PH-M----------------IRDRKYH------L-KTY----------------RQC-CV
GT-----------------ELVDWMMQQT-------------------------------
---PCVH--------SRTQAVGMW---QVLLEDGVLN-----------------------
--------------H------------------------VDQ-EHHFQDKY-LFYRFLDD
-------EHED----AP-L---
--PT
EEEK--KECDEEL------QDTMLLLSQMGPDAH-------------MRMILRKPPGQRT
VDDLE-----IIYEELLHIKALSHLS-TTVKRELA-GVLIFESHAKGGTVL---------
--FNQGEEG--TSWYIIL
KGSVNVVIYGK-----GV-------------------------------VCTLHEG-DDF
GKLAL-----------VNDAPRA---ASIVLREDNCHFLRVDKEDFNR------ILRD--
---V--------------

------------------------EANTVRLKEHDQDVLVLEK-----VPA-GNRASNQG

NS----QP-QQ------------------------------------K-YTVMSGTPEKI
LEHFLET------------IRLEA-TLN--------------EA-----TDSVLN---D-
FIMMHCV--FM---------------PNTQLCPALV-----A------------------
--------H-----------Y-------HAQPSQ---GTEQEKMDYALNNKRRVIRLVLQ
WAAMYGDLLQEG-DVSMAFLE----EFYVS--VSDDA--RMIAALK---EQ-LPELEKIV
KQI---SED--AK---------APQKK---------------------------------
---HKV--------LLQQFNTGD-ERAQ-KRQ----------------------------

-----PIRGSDE--VL-----------------------------FK-------------
VYCMDHTYTTIRVPVATSVKEVIS--------AVADK-----------LGSG-EG--LII
VKMSSG----------------------GEKVV------LKPNDVSVFTTLTINGRLFAC
PREQ---F-DSLT-PLPEQ---------------------------EGP------TVGTV
GT--FELMSSKDLAYQMTIYDWELFNCVHELELIY-------HTFGRHNF-KK-TTANLD
LFLRR---------FNEIQFWV-----VTEICLCSQLSKR------VQLLKKFIKIAAH-
--------------CKEYKNLNSFFAIVM-GLSNVAVSRLALTWE---------------
-------KLPSKFKKFYAE-FESLM--------------------------DPSRNH---
-RAYRL----TVAK-------LEPPLI-PFMP----------------------------
LLIKDMTFTHEGNK--TF-ID-----------------------------N-LVNFE-KM
-------RM---IANTARTVRYYRS--Q-------------------------PFNPD--
-A------------------AQANKNH-----Q----------------DVRS-----YV
RQLNVIDNQRTL---SQMSHRL
-----------EPRR----------P----------------------------------

>Rapgef4_[Ictidomys_tridecemlineatus]_532077758
----------------------------MVAA-------HA------AH--------SSS
SAEWIA---CLDK-----------RPLE-----RSSEDVDIIFTRLKEVKAFEKFHPNLL
HQICLCGY--YENLE--KGITLFRQGDIGTNWYAVLAGSLDVKVSE-TSSHQDA------
------VTICTLGIGTAFGESILDNT-PRHATIV-TRESSELL-----------------
----------------------------RIEQKD-------FKALW---EKYRQYMAG-L
LAPPYG--VMETGS--N-N--------DRIPDKE--------------------------
------------------N----------------------T------------------
-----PLIEPHVPLRPAN-TITK------------------VPS----------------
-------EKIL-------------RA---------------------GKILR-NAILSRA
PH-M----------------IRDRKYH------L-KTY----------------RQC-CV
GT-----------------ELVDWMMQQT-------------------------------
---PCVH--------SRTQAVGMW---QVLLEDGVLN-----------------------
--------------H------------------------VDQ-EHHFQDKY-LFYRFLDD
-------EHED----AP-L---
--PT

EEEK--KECDEEL------QDTMLLLSQMGPDAH-------------MRMILRKPPGQRT
MDDLE-----IIYEELLHIKALSHLS-TTVKRELA-GVLIFESHAKGGTVL---------
--FNQGEEG--TSWYIIL
KGSVNVVIYGK-----GV-------------------------------VCTLHEG-DDF
GKLAL-----------VNDAPRA---ASIVLREDNCHFLRVDKEDFNR------ILRD--
---V--------------

------------------------EANTVRLKEHDQDVLVLEK-----VPA-GSRASNQG
NS----QP-QQ------------------------------------K-YTVMSGTPEKI
LEHFLET------------IRLEP-ALN--------------EA-----TDSVLN---D-
FIMMHCV--FM---------------PNTQLCPALV-----A------------------
--------H-----------Y-------HAQPSQ---GTEQERMDYALNNKRRVIRLVLQ
WAAMYGDLLQED-DVAMAFLE----EFYVS--VSDDA--RMIAALK---EQ-LPELEKIV
KQI---SED--AK---------APQKK---------------------------------
---HKV--------LLQQFNTSD-ERAQ-KRQ----------------------------

-----PVRGSDE--VL-----------------------------FK-------------
VYCMDHTYTTIRVPVAASVKEVIS--------AVADK-----------LGSG-EG--LII
VKMSSG----------------------GEKVV------LKPNDVSVFTTLTINGRLFAC
PXEQ---F-DSLT-PLPEQ---------------------------EGP------TVGTV
GT--FELMSSKDLAYQMTIYDWELFNCVHELELIY-------HTFGRHNF-KK-TTANLD
LFLRR---------FNEIQFWV-----VTEICLCSQLSKR------VQLLKKFIKIAAH-
--------------CKEYRNLNSFFAIVM-GLSNVAVSRLALTWE---------------
-------KLPSKFKKFYAE-FESLM--------------------------DPSRNH---
-RAYRL----TAAK-------LEPPLI-PFMP----------------------------
LLIKDMTFTHEGNK--TF-ID-----------------------------N-LVNFE-KM
-------RM---IANTARTVRYYRS--Q-------------------------PFNPD--
-A------------------AQANKNH-----Q----------------DVRS-----YV
RQLNVIDNQRTL---SQMSHRL
-----------EPRR----------P----------------------------------

>Rapgef4_[Kryptolebias_marmoratus]_1041109518

-----------------------------MPEKE--------------------------

------------------NIT-------NNVFVSVSKHPNKK------------------
-----SFLESP---AKVL-PKCTA----------------QVLS----------------
-------EKIL-------------RA---------------------GKTLR-YAILSSA
PH-M----------------IRDRKYH------L-KTY----------------RQC-CV
GT-----------------ELVDWQMQQS-------------------------------
---SCVH--------SRIQAVGMW---QVLLEEGVLN-----------------------
--------------H------------------------VDG-ETSFQDKY-LFYRFLDD
-------ERED----AC-V---
--PD
EEER--RESQEEL------QDTLLFLSQIGPDAL-------------MRMILRKPPSERT
ADELE-----IIYEELLHIKALSHLS-TTVKRELA-GVLIFESHEKAGTVL---------
--FNQGEEG--TSWYIIL
KGSVNVVIYGK-----GV-------------------------------VCTLHEG-DDF
GKLAL-----------VNDAPRA---ASIVLREDNCHFLRVDKEDFNR------ILRD--
---V--------------

------------------------EANTVRLKEHGQDVLVLEK-----S-----RNSVHG
SS----TS-HY------------------------------------K-YNIMSGTPEKI
LEHLLEM------------MRLDS-QFT--------------KS------DSALD---D-
FVLMHCV--FM---------------PNSQLCPVLT-----A------------------
--------H-----------Y-------HAEALQ---GSEQEQLDYTLNNKRRVIRLVVK
WAAVNGDHLQQE-DRA--FLE----EFLVM--VSNDV--KVLPALR---DQ-LSELERIL
KSN---AED--AR---------SSQKK---------------------------------
---HKV--------LLRQFSMGD-EKHQ-KCQ----------------------------

-----PIKGDDE--IL-----------------------------FK-------------
VHCCDHTYTTIRVPVAASVREVID--------AVADK-----------LGSA-EG--LIL
VGLSSA----------------------GEKSI------FKPNDVSVFSTLSTNGRLFVC
QRDQ---L-DLLT-PLPEQ---------------------------EGP------SSGSL
AS--FELMSSKDVAYHITSYDWELFHCVHELELIY-------HTFGRQYV-KK-TTVNLD
LFLRR---------FNEIQFWV-----ITEMCLCPQLNKR------VQLLKKFIKIAAH-
--------------CKEYKNLNAFFAVIM-GLSNPAVSRLSQTWE---------------
-------KLPSKFKKFYEE-FENLM--------------------------DPTRNH---
-RAYRL----TVSK-------LDPPLI-PFMP----------------------------
LMIKDMTFTHEGNK--TF-ID-----------------------------N-LVNFE-KM
-------RM---IANTVKIVRYCRS--Q-------------------------MFSPD--
-S------------------PLAGKHH-----P----------------DVWT-----YV
RQLNVIDNQRTL---TQLSHGL
-----------EPRR----------S----------------------------------

>Rapgef4_[Lepisosteus_oculatus]_973161213

-----------------------------MPDKD--------------------------
------------------NMT-------NNALGSISKNLNK-------------------
---VPS----------------
-------EKIL-------------RA---------------------GKILR-NSILSRA
PP-M----------------IRDRKYH------L-KTY----------------RQC-CV
GT-----------------ELVDWLMQQS-------------------------------
---PCVH--------SRTQAVGMW---QVLLEEGVLN-----------------------
--------------H------------------------VDQ-EHNFQDKY-LFYRFLDD
-------ERED----AP-L---
--LS
EEEK--KESEEEL------QDTLLLLSQTGPDAH-------------MRMILRKPPGQRT
VDDLE-----IIYEELLHIKALSHLS-TTVKRELA-GVLIFESHAKAGTVL---------
--FNQGEEG--TSWYIIL
KGSVNVVIYGK-----GV-------------------------------VCTLHEG-DDF
GKLAL-----------VNDAPRA---ASIVLREDNCHFLRVDKEDFNR------ILRD--
---V--------------

------------------------EANTVRLKEHEQDVLVLEK-----IST-GSRVLNHG
NA----SS-QY------------------------------------K-YMVMSGTPEKI
LEHFLET------------MRLDS-NFT--------------ES------DPALD---D-
FVLMHCV--FI---------------PNTQLCPILM-----A------------------
--------H-----------Y-------HAQASQ---GSEQEKLDYTFNNKRRVIRLVLQ
WASVYGDHLQEE-EAALAFLE----EFYVS--VSDDS--RVIPALK---EH-LPELEKIV
KQN---TEE--AK---------SSQKK---------------------------------
---HKV--------LLRQFSIGD-ERLQ-KRQ----------------------------

-----PIKSNDE--IL-----------------------------FK-------------
VYCIDHTYTTIRVSVAASVKEVIS--------AVADK-----------LGSG-EE--LIL
VHVNSA----------------------GDKVV------LKPNDISVFTTLSINGRLFVC
PRDQ---F-DSLA-PLPEQ---------------------------EGP------STGSI
GT--FELMSSKDLAYQMTVYDWELFNCVHEHELIY-------HTFGRQHF-KK-TTANLD
LFLRR---------FNELQLWV-----VTEICLCSQLSKR------VQLLKKFIKIAAH-
--------------CKEYRNLNSFFAIIM-GLSNPAASRLSQTWE---------------
-------KLPSKFKKFYSE-FESLM--------------------------DPSRNH---
-RAYRL----TVAK-------LDPPII-PFMP----------------------------
LLMKDMTFTHEGNK--TF-ID-----------------------------S-LVNFE-KM
-------RM---IANTVRIVRYCRS--Q-------------------------PFNPD--
-A------------------SLANKNH-----Q----------------EVRS-----YV
RQLNVVDNQRTL---SQLSHRL
-----------EPRR----------N----------------------------------

>Rapgef4_[Leptonychotes_weddellii]_585180432

---MAG-L
LAPPYG--VMETGS--N-N--------DRIPDKE--------------------------
------------------N---
---VPS----------------
-------EKIL-------------RA---------------------GKILR-NAILSRA
PH-M----------------IRDRKYH------L-KTY----------------RQC-CV
GT-----------------ELVDWMLQQT-------------------------------
---SCVH--------SRTQAVGMW---QVLVEDSVLN-----------------------
--------------H------------------------VDQ-EHHFQDKY-LFYRFLDD
-------EHED----AP-L---
--PT
EEEK--KECDEEL------QDTMLLLSQMGPDAH-------------MRMILRKPPGQRT
VDDLE-----IIYEELIHIKALSHLS-TTVKRELA-GVLIFESHAKGGTVL---------
--FNQGEEG--TSWYIIL
KGSVNVVIYGK-----GV-------------------------------VCTLHEG-DDF
GKLAL-----------VNDAPRA---ASIVLREDNCHFLRVDKEDFNR------ILRD--
---V--------------

------------------------EANTVRLKEHDQDVLVLEK-----VPA-GNRASNQG
NS----QP-QQ------------------------------------K-YTVMSGTPEKI
LEHFLET------------IRLEP-ALN--------------EA-----TDSVLN---D-
FVMMHCV--FM---------------PNTQLCPALV-----A------------------
--------H-----------Y-------HAQASQ---GTEQEKMDYALNNKRRVIRLVLQ
WTAVYGDALQED-DVAVAFLE----EFYVS--VSDDA--RMIAALK---DQ-LPELEKIV
KQI---SED--AK---------APQKK---------------------------------
---HKV--------LLQQFNTGD-ERAQ-KRQ----------------------------

-----PIRGSDE--VL-----------------------------FK-------------
VYCMDHTYTTIRVPVAASVKEVIS--------AVADK-----------LGSG-EG--LII
VKMSSG----------------------GEKVV------LKPNDVSVFTTLTINGRLFAC
PREQ---F-DSLT-PLPEQ---------------------------EGP------TVGTV
GT--FELMSSKDLAYQMTIYDWELFNCVHELELIY-------HTFGRHNF-KK-TTANLD

LFLRR---------FNEIQFWV-----VTEICLCSQPSKR------VQLLKKFIKIAAH-
--------------CKEYKNLNSFFAIVM-GLSNVAVSRLALTWE---------------
-------KLPSKFKKFYAE-FESLM--------------------------DPSRNH---
-RAYRL----TVAK-------LEPPLI-PFMP----------------------------
LLIKDMTFTHEGNK--TF-ID-----------------------------N-LVNFE-KM
-------RM---IANTARTVRYCRS--Q-------------------------AFNPD--
-A------------------AQANKNH-----Q----------------DVRS-----YV
RQLNVIDNQRTL---SQMSHRL
-----------EPRR----------P----------------------------------

>Rapgef4_[Leptosomus_discolor]_700415863

---MSG-L
LTPPYG--VMETGS--N-N--------DRMPDKD--------------------------
------------------SVS-------SSALCQV-------------------------
----------------SK-NCNK------------------VPS----------------
-------EKIL-------------RA---------------------GKILR-NTILSRA
PH-M----------------IRDRKYH------L-KTY----------------RQC-CV
GT-----------------ELVDWLMQQS-------------------------------
---PCVH--------SRTQAVGMW---QVLLEESVLN-----------------------
--------------H------------------------VDQ-EHHFQDKY-LFYRFLDD
-------EHED----AP-L---
--PT
EEEK--KECDEEL------QDTMLLLSQIGPDAH-------------MRMILRKPPGQRT
VDDLE-----FIYEELLHIKALSHLS-TTVKRELA-GVLIFESHPKAGTVL---------
--FNQGEEG--TSWYIIL
KGSVNVVIYGK-----GV-------------------------------VCTLHEG-DDF
GKLAL-----------VNDAPRA---ASIVLREDNCHFLRVDKEDFNR------ILRD--
---V--------------

------------------------EANTVRLKEHDQDVLVLEK-----IPA-GNRVSNQG
NS----QP-QH------------------------------------K-YIVMSGTPEKI
LEHFLET------------MRLEA-TLN--------------EA-----TDSVLN---D-
FIMMHCV--FM---------------PNSQLCPALM-----A------------------
--------H-----------Y-------HAQPSQ---GTEQEKMDYALNNKRRVIRLILQ
WAALYGDLLQED-EAAMAFLE----EFYVS--VSDDT--RMIAALK---EQ-LPELEKIV
KQV---SEE--PK---------APQKK---------------------------------
---HKV--------LLQLFNTSD-DRAQ-KRQ----------------------------


```
------------------------------------------------------------
------------------------------------------------------------
------------------------------------------------------------
------------------------------------------------------------
-----PIRGSDE--VL-----------------------------FK-------------
VYCIDQTYTTIRVPVSSSVKEVIS--------AVADK-----------LGSG-EG--LII
VKMSSG----------------------GEKVV------LKPHDVSVFTTLSVNGRLFAC
PRDQ---F-DSLA-PLPEQ---------------------------EGP------STGTV
GT--FELMSSKDLAYQMTIYDWELFNCVHELELVY-------HTFGRHNF-KK-TTANLD
LFLRR---------FNEIQFWV-----VTEICLCSQLSKR------VQLLKKYIKIAAH-
--------------CKEYKNLNSFFAIIM-GLSNVAVSRLSLTWE---------------
-------KLPSKFKKIYAE-FESLM--------------------------DPSRNH---
-RAYRL----TVAK-------LDPPII-PFMP----------------------------
LLIKDMTFTHEGNK--TF-TD-----------------------------N-LVNFE-KM
-------RM---IANTVRTVKFCRS--Q-------------------------SFNPD--
-A------------------ALTNKNH-----Q----------------DVRS-----YV
RQLNVIDNQRTL-----------------------------------------SQMSHRL
-----------EPRR----------A----------------------------------
------------------------------------------------------------
------------------------------------------------------------
------------------------------------------------------------
------------------------------------------------------------
------------------------------------------------------------
------------------------------------------------------------
------------------------------------------------------------
------------------------------------------------------------
------------------------------------------------------------
------------------------------------------------------------
---
>Rapgef4_[Limulus_polyphemus]_926609137
-----------------------------MVTE---------------------------
---WMSS---MDK-----------RPLD-----RTEEDVNAIYSRLRNVQAFQQFHPTLL
HYLCYYGY--YEDLD--KGITLFREGDQGSNWYLVLAGSLEVHTSQSS-RSQDS------
------LTLCTLGIGTAFGDGVLNNS-PHTATVI-TNEHCELLRIEQRDFQELWERN---
--PH--LMEDIV------------------------SPLNTVRSLT--------------
DLHRRGEKKDTNGTSRNLPPSNKHPEPSSPEARNP-------------------------
------------------------------------------------------------
---------------------------------------ALP------------IT----
---QEPSAKLR-------------RA---------------------GKVLR-TMIFSHA
PH-L----------------IRDRKYH------M-KTI----------------RRC-LV
GT-----------------EMVDWLLQQA------------------------------I
GGAVRIH--------SRAQAIGMW---QALLEEGVIS-----------------------
--------------H------------------------VSQ-EYQFKDKH-LFYRFRED
-------QDGV----GT-T-----------------------------------------
----------------------------------------------------------PS
QDDR--HLAEKEF------QESLTVLLQLSPEAI-------------LRTIIRKPPEDRT
ADDIE-----VIFEELLHIKALSHLS-NSVKRELA-GVLFFESHLRTGTVL---------
--------------------------------------------FNQGDEG--TSWYIIL
RGSVDVVIYGK-----GV-------------------------------VCTLHEG-DDF
GKLAL-----------VNDAPRA---ATIVTREDNCHFLRVDKIDFNR------ILRD--
---------------------------------------------A--------------
------------------------------------------------------------
------------------------------------------------------------
------------------------EANTVRLKEHGQDVLLLQK-----ILT-----DVRG
```

```
VE--GSHC-HY------------------------------------K-YAVMAGTPQKM
LEHLLETRIDIRVED------GTG-PLT--------------YS-K----DTFLE---D-
FLLTHVI--FM---------------PSHQLCPELM-----K------------------
--------HYKVDSAN-----------------------SRHEKEFIVANKRRAVQFVHT
WAIVVREAFFED-PIICSFLD----DLQAL--LLKDF--ELY--VGDLQME-VKLLAKMI
DAK-QRFQVDLAS--------SAS-QR---------------------------------
---WKISNSGQLERLTSSGSAEE-DEDE-DR-----------------------------
------------------------------------------------------------
------------------------------Q-----------------------------
------------------------------------------------------------
------------------------------------------------------------
------------------------------------------------------------
------------------------------------------------------------
-----PIKSTDE--II-----------------------------FR-------------
VYRADQTYTTLKTPTAATAETIKR--------SAADK-----------LGLK-DD--VLL
VEVRST----------------------GEKVP------FKDAEVSVPTGLSVNGKIFVC
TADQ---I-DSLR-PLPEQ---------------------------EGP------SEGTG
DV--LETFSSHDLAHQMSLYDWELFTVVHEYELTY-------QVFGRHQF-RK-SMSNLD
VFLRR---------FNELQFWV-----VTELCLASSLSRR------VQLLRKFIKVAAY-
--------------CRDYQNLNAFFAIVM-GLSNVAVSRMSQTWE---------------
-------RLPGKLRKLYAD-FEALI--------------------------DPSRNH---
-RRYRI----IVAK-------LAPPII-PFMP----------------------------
LLLKDMTFSHEGNK--TY-LE-----------------------------G-LVNFE-KM
-------HM---IAQTLRTLRYCRS--Q-------------------------VLALEP-
-P------------------PI-SKVQ-----Q----------------EVRE-----YI
RNIKVIDNQRRL-----------------------------------------TQLSNTL
-----------EPRR----------PC---------------------------------
------------------------------------------------------------
------------------------------------------------------------
------------------------------------------------------------
------------------------------------------------------------
------------------------------------------------------------
------------------------------------------------------------
------------------------------------------------------------
------------------------------------------------------------
------------------------------------------------------------
------------------------------------------------------------
---
>Rapgef4_[Lingula_anatina]_919011582
------------------------------------------------------------
------------------------------------------------------------
------------------------------------------------------------
-------------------------------------------------MKISNERN---
--QQ--LMEGAFN------PAIST----LPQLDTTYRRVA--------------------
---------------------------------DPRRR----S-----------------
-----------------VK---EGENTLTNT-----------------------------
------------------------------------------ATP---------IT----
---STPSSRLL-------------KA---------------------GYVLR-TIIQTRS
PS-M----------------IRDRKHH------L-RTY----------------RRC-MV
GS-----------------EMTDWMLQVS-------------------------------
---PSVH--------SKIQAIGMW---QALLEEGVLV-----------------------
--------------H------------------------VCR-EHPFKDAD-LFYRFHDD
-------DQGV----GT-V-----------------------------------------
----------------------------------------------------------PT
```

SGMK--KECEELL------PEVVNNLVQIGPDAL-------------MRMILRKLPEDRS
PEDLE-----IIYEELLHIKALSHLS-TMVKRELA-SVLMFEAHERAGTVL---------
--FNQGDEG--TSWYIIL
KGSVNVVIYGK-----GV-------------------------------VCTLHEG-DDF
GKLAL-----------VNDAPRA---ATIVLREDDCHFLRVDKEDFNR------ILRD--
---V--------------

------------------------EANTVRLKEHGQDVLVLEK-----IPT---KQPGQQ
TT--SGQT-HY------------------------------------K-YSVMAGTPEKM
LEYLLETIE-SKH-ENVS--------------------------------DTFLE---D-
FLLTHII--FM---------------PSNHLCPALL-----M------------------
--------HYNAEGAN-----------------G---NSEQESPDLMLANKKRVVKFVQE
WQNVAGSVFFED-ATVTAFLE---------------------------------------

------------------------------EMRVAVNEDSGFFDSLKEEANTIKQIQHRQ
QGHLNSLHPKRVSLPDSC--------------------------------SSDPQASPSR
SPHPSPSRSPLLQRFTRRGSNFETVIDDKVQ-----------------------------

-----PLRAIDE--NI-----------------------------FK-------------
IYCADHTYTTLRQRMGASVAEVLQ--------NAANK-----------LCLG-ND--LLL
CEVKSN----------------------GERVI------FSEDDLCVTTGLSVNGRLFIV
PKEH---L-DALT-PLPEQ---------------------------DGP------TENTY
AT--FEIMGSKDIAYHLTMYDWELLNTMHEYEMIY-------LIFGRSKF-QR-ISANFD
RFLQR---------FNVVQYWV-----LTEMCLTPNLGKR------VQMLKKFIKIAEY-
--------------CLEYQNLNSFFAIVM-GLSNLAVSRLSQTWE---------------
-------KLPSKMKKLFSD-FESMM--------------------------DPSRNH---
-RVYRL----TVAK-------MDAPFI-PFMP----------------------------
LLMKDLTFTHDGNK--SY-FD-----------------------------G-LVNFE-KM
-------HM---VALTMRSIKFCRS--K-------------------------PLEVEP-
-P------------------TV-GKNA-----Q----------------DVKN-----YV
RNIRVIDNQRIL---TRLSQRL
-----------EPRR----------S----------------------------------

>Rapgef4_[Lipotes_vexillifer]_602693655

---MAG-L
LAPPYG--VMETGS--N-N--------DRIPDKE--------------------------

```
------------------N-----------------------------------------
-----------------------------------------VPS----------------
-------EKIL-------------RA---------------------GKILR-NAILSRA
PH-M----------------IRDRKYH------L-KTY----------------RQC-CV
GT-----------------ELVDWMLQQT-------------------------------
---PCVH--------SRIQAVGMW---QVLVEDGVLN-----------------------
--------------H------------------------VDQ-EHHFQDKY-LFYRFLDD
-------EHED----AP-L-----------------------------------------
----------------------------------------------------------PT
EEEK--KECDEEL------QDTMLLLSQMGPDAH-------------MRMILRKPPGQRT
VDDLE-----IIYEELLHIKALSHLS-TTVKRELA-GVLVFESHAKGGTVL---------
--------------------------------------------FNQGEEG--TSWYIIL
KGSVNVVIYGK-----GV-------------------------------VCTLHEG-DDF
GKLAL-----------VNDAPRA---ASIVLREDNCHFLRVDKEDFNR------ILRD--
---------------------------------------------V--------------
------------------------------------------------------------
------------------------------------------------------------
------------------------EANTVRLKEHDQDVLVLEK-----VPA-GNRASNQG
NS----QP-QQ------------------------------------K-YTVMSGTPEKI
LEHFLET------------IRLEP-TLN--------------EA-----TDSVLN---D-
FVMMHCV--FM---------------PNTQLCPALV-----A------------------
--------H-----------Y-------HAQPSQ---GTEQEKMDYALNNKRRVIRLVLQ
WAAMYGDVLQED-DVAMAFLE----EFYVS--VSDDA--RMIAALK---EQ-LPELEQIV
KQI---SED--AK---------APQKK---------------------------------
---HKV--------LLQQFNTGD-ERAQ-KRQ----------------------------
------------------------------------------------------------
------------------------------------------------------------
------------------------------------------------------------
------------------------------------------------------------
------------------------------------------------------------
------------------------------------------------------------
-----PIRGSDE--VL-----------------------------FK-------------
VYCMDHTYTTIRVPVAASVKEVLS--------AVADK-----------LGSG-EG--LII
IKMSSG----------------------GEKVV------LKPNDVSVFTTLTINGRLFAC
PREK---F-DSLT-PLPEQ---------------------------EGP------TVGTM
GT--FELMSSKDLAYQMTVYDWDLFSCVHELELIY-------HTFGRHHF-KK-TTANLD
LFLRR---------FNEIQFWV-----VTEICLCSQPSKR------VQLLKKFIKIAAH-
--------------CKEYKNLNSFFAIVM-GLSNVAVSRLALTWE---------------
-------KLPSKFKKFYAE-FESLM--------------------------DPSRNH---
-RAYRL----TVAK-------LDPPLI-PFMP----------------------------
LLIKDMTFTHEGNK--TF-ID-----------------------------N-LVNFE-KM
-------RM---IANTARTVRYCRS--Q-------------------------PFNPD--
-A------------------AQANKNH-----Q----------------DVRS-----YV
RQLNVIDNQRTL-----------------------------------------SQMSHRL
-----------EPRR----------P----------------------------------
------------------------------------------------------------
------------------------------------------------------------
------------------------------------------------------------
------------------------------------------------------------
------------------------------------------------------------
------------------------------------------------------------
------------------------------------------------------------
------------------------------------------------------------
------------------------------------------------------------
```


>Rapgef4_[Loxodonta_africana]_731458747

---MAG-L
LAPPYG--VMETGS--N-N--------DRIPDKE--------------------------
------------------N----------------------T------------------
-----PLIEPHVPLRPAN-TITK------------------VPS----------------
-------EKIL-------------RA---------------------GKILR-NVILSRA
PH-M----------------IRDRKYH------L-KTY----------------RQC-CV
GT-----------------ELVDWMMQQT-------------------------------
---QCVH--------SRTQAVGMW---QVLLEDGVLN-----------------------
--------------H------------------------VDQ-EHHFQDKY-LFYRFLDD
-------EHED----AP-L---
--PT
EEEK--KECDEEL------QDTMLLLSQMGPDAH-------------MRMILRKPPGQRT
VDDLE-----IIYEELLHIKALSHLS-TTVKRELA-GVLIFESHAKGGTVL---------
--FNQGEEG--TSWYIIL
KGSVNVVIYGK-----GV-------------------------------VCTLHEG-DDF
GKLAL-----------VNDAPRA---ASIVLREDNCHFLRVDKEDFNR------ILRD--
---V--------------

------------------------EANTVRLKEHDQDVLVLEK-----VPA-GNRASNQG
NS----QS-QQ------------------------------------K-YTVMSGTPEKI
LEHFLET------------IRLEP-ALN--------------EA-----TDSVLN---D-
FVMMHCV--FM---------------PNTQLCPALV-----A------------------
--------H-----------Y-------HAQPSQ---GTEQEKMDYTLNNKKRVIRLVLQ
WAATYGDLLQED-DVAMAFLE----EFYVS--VSDDA--RMIAALK---EQ-LPDLEKIV
KQI---LEE--AK---------APQKK---------------------------------
---HKV--------LLQQFNTGD-ERAQ-KRQ----------------------------

-----PIRGSDE--VL-----------------------------FK-------------
VYCMDHTYTTIRVPVAASVKEVIS--------AVADK-----------LGSG-EG--LII
VKMSSG----------------------GEKVL------LKPNDVSVFTTLTINGRLFAC
PREQ---F-DSLT-PLPEQ---------------------------EGP------TVGTV
GT--FELMSSKDLAYQMTIYDWELFNCVHELELIY-------HTFGRHNF-KK-TTANLD
LFLRR---------FNEIQFWV-----VTEICLCSQLSKR------VQLLKKFIKIAAH-
--------------CKEYKNLNSFFAIVM-GLSNVAVSRLALTWE---------------
-------KLPSKFKKFYAE-FESLM--------------------------DPSRNH---
-RAYRL----TVAK-------LEPPLI-PFMP----------------------------
LLIKDMTFTHEGNK--TF-TD-----------------------------N-LVNFE-KM
-------RM---IANTARTVRYCRS--Q-------------------------PFNPD--
-A------------------AQANKNH-----Q----------------DVRS-----YV
RQLNVIDNQRTL---SQMSHRL
-----------EPRR----------P----------------------------------

```
------------------------------------------------------------
------------------------------------------------------------
------------------------------------------------------------
------------------------------------------------------------
------------------------------------------------------------
------------------------------------------------------------
------------------------------------------------------------
------------------------------------------------------------
------------------------------------------------------------
------------------------------------------------------------
---
>Rapgef4_[Manacus_vitellinus]_675430502
------------------------------------------------------------
------------------------------------------------------------
------------------------------------------------------------
------------------------------------------------------------
-------------------------------------------------------MSG-L
LTPPYG--VMETGS--N-N--------DRMPDKD--------------------------
------------------N----------------------T------------------
-----PLIEPHIPHRPTK-TITQ------------------VPS----------------
-------EKIL-------------RA---------------------GKILR-NTILSRA
PH-M----------------IRDRKYH------L-KTY----------------RQC-CV
GT-----------------ELVDWMMQQS-------------------------------
---PCVH--------LRTQAVGMW---QVLLEEGVLN-----------------------
--------------H------------------------VDQ-EHHFQDKY-LFYRFLDD
-------ERED----AP-L-----------------------------------------
----------------------------------------------------------PT
EEEK--KECDEEL------QDTMLLLSQIGPDAH-------------MRMILRKPPGQRT
VDDLE-----FIYEELLHIKALSHLS-TTVKRELA-GVLIFESHPKAGTVL---------
--------------------------------------------FNQGEEG--TSWYIIL
KGSVNVVIYGK-----GV-------------------------------VCTLHEG-DDF
GKLAL-----------VNDAPRA---ASIVLREDNCHFLRVDKEDFNR------ILRD--
---------------------------------------------V--------------
------------------------------------------------------------
------------------------------------------------------------
------------------------EANTVRLKEHDQDVLVLEK-----IPA-GNRISNQG
NS----QL-QH------------------------------------K-YIVMSGTPEKI
LEHFLET------------MRLEA-TLN--------------EA-----TDSVLN---D-
FIMMHCV--FM---------------PNSQLCPALM-----A------------------
--------H-----------Y-------HAQPSQ---GTEQEKMDYALNNKRRVIRLVLQ
WAALYGDLLQED-EAAMAFLE----EFYVS--VSDDT--RMIAALK---EQ-LPELEKIV
KQV---SEE--PK---------APQKK---------------------------------
---HKV--------LLQLFNTSD-DRAQ-KRQ----------------------------
------------------------------------------------------------
------------------------------------------------------------
------------------------------------------------------------
------------------------------------------------------------
------------------------------------------------------------
------------------------------------------------------------
-----PIRGSDE--VL------------------------------FK-------------
VYCIDQTYTTIRVPVSSSVKEVIS--------AVADK-----------LGSG-EG--LII
VKMSSG----------------------GEKVV------LKPHDVSVFTTLSVNGRLFAC
PRDQ---F-DSLA-PLPEQ---------------------------EGP------STGTV
GT--FELMSSKDLAHQMTIYDWELFNCVHELELIY-------HTFGRHNF-KK-TTANLD
```

LFLRR---------FNEIQFWV-----VTEICLCSQLSKR------VQLLKKYIKIAAH-
--------------CKEYKNLNSFFAIIM-GLSNVAVSRLSLTWE---------------
-------KLPSKFKKIYAE-FESLM--------------------------DPSRNH---
-RAYRL----TVAK-------LDPPII-PFMP----------------------------
LLIKDMTFTHEGNK--TF-TD-----------------------------N-LVNFE-KM
-------RM---IANTVRTVKFCRS--Q-------------------------SFNPD--
-A------------------ALANKNH-----Q----------------DVRS-----YV
RQLNVIDNQRTL---SQMSHRL
-----------EPRR----------A----------------------------------

>Rapgef4_[Mandrillus_leucophaeus]_795195949

---M----------------IRDRKYH------L-KTY----------------RQC-CV
GT-----------------ELVDWMMQQT-------------------------------
---PCVH--------SRTQAVGMW---QVLLEDGVLN-----------------------
--------------H------------------------VDQ-EHHFQDKY-LFYRFLDD
-------EHED----AP-L---
--PT
EEEK--KECDEEL------QDTMLLLSQMGPDAH-------------MRMILRKPPGQRT
VDDLE-----IIYEELLHIKALSHLS-TTVKRELA-GVLIFESHAKGGTVL---------
--FNQGEEG--TSWYIIL
KGSVNVVIYGK-----GV-------------------------------VCTLHEG-DDF
GKLAL-----------VNDAPRA---ASIVLREDNCHFLRVDKEDFNR------ILRD--
---V--------------

------------------------EANTVRLKEHDQDVLVLEK-----VPA-GNRASNQG
NS----QP-QQ------------------------------------K-YTVMSGTPEKI
LEHFLET------------IRLEA-TLN--------------EA-----TDSVLN---D-
FIMMHCV--FM---------------PNTQLCPALV-----A------------------
--------H-----------Y-------HAQPSQ---GTEQEKMDYALNNKRRVIRLVLQ
WAAMYGDLLQED-DVSMAFLE----EFYVS--VSDDA--RMIAALK---EQ-LPELEKIV
KQI---SED--AK---------APQKK---------------------------------
---HKV--------LLQQFNTGD-ERAQ-KRQ----------------------------

-----PIRGSDE--VL-----------------------------FK-------------
VYCMDHTYTTIRVPVAASVKEVIS--------AVADK-----------LGSG-EG--LII
VKMSSG----------------------GEKVV------LKPNDVSVFTTLTINGRLFAC
PREQ---F-DSLT-PLPEQ---------------------------EGP------TIGTV
GT--FELMSSKDLAYQMTIYDWELFNCVHELELIY-------HTFGRHNF-KK-TTANLD
LFLRR---------FNEIQFWV-----VTEICLCSQLSKR------VQLLKKFIKIAAH-
--------------CKEYKNLNSFFAIVM-GLSNVAVSRLALTWE---------------
-------KLPSKFKKFYAE-FESLM--------------------------DPSRNH---
-RAYRL----TVAK-------LEPPLI-PFMP----------------------------
LLIKDMTFTHEGNK--TF-ID-----------------------------N-LVNFE-KM
-------RM---IANTARTVRYYRS--Q-------------------------PFNPD--
-A------------------AQANKNH-----Q----------------DVRS-----YV
RQLNVIDNQRTL---SQMSHRL
-----------EPRR----------P----------------------------------

>Rapgef4_[Marmota_marmota_marmota]_984087885

---MAG-L
LAPPYG--VMETGS--N-N--------DRIPDKE--------------------------
------------------N----------------------T------------------
-----PLIEPHVPLRPAN-TITK------------------VPS----------------
-------EKIL-------------RA---------------------GKILR-NAILSRA
PH-M----------------IRDRKYH------L-KTY----------------RQC-CV
GT-----------------ELVDWMMQQT-------------------------------
---PCVH--------SRTQAVGMW---QVLLEDSVLN-----------------------
--------------H------------------------VDQ-EHHFQDKY-LFYRFLDD
-------EHED----AP-L---
--PT
EEEK--KECDEEL------QDTMLLLSQMGPDAH-------------MRMILRKPPGQRT
MDDLE-----IIYEELLHIKALSHLS-TTVKRELA-GVLIFESHAKGGTVL---------
--FNQGEEG--TSWYIIL
KGSVNVVIYGK-----GV-------------------------------VCTLHEG-DDF
GKLAL-----------VNDAPRA---ASIVLREDNCHFLRVDKEDFNR------ILRD--
---V--------------

------------------------EANTVRLKEHDQDVLVLEK-----VPA-GSRASNQG

NS----QP-QQ------------------------------------K-YTVMSGTPEKI
LEHFLET------------IRLEP-ALN--------------EA-----TDSVLN---D-
FVMMHCV--FM---------------PNTQLCPALV-----A------------------
--------H-----------Y-------HAQPSQ---GTEQERMDYALNNKRRVIRLVLQ
WAAMYGDLLQED-DVAMAFLE----EFYVS--VSDDA--RMIAALK---EQ-LPELEKIV
KQI---SED--AK---------APQKK---------------------------------
---HKV--------LLQQFNTSD-ERAQ-KRQ----------------------------

-----PVRGSDE--VL-----------------------------FK-------------
VYCMDHTYTTIRVPVAASVKEVIS--------AVADK-----------LGSG-EG--LII
VKMSSG----------------------GEKVV------LKPNDVSVFTTLTINGRLFAC
PREQ---F-DSLT-PLPEQ---------------------------EGP------TVGTV
GT--FELMSSKDLAYQMTIYDWELFNCVHELELIY-------HTFGRHNF-KK-TTANLD
LFLRR---------FNEIQFWV-----VTEICLCSQLSKR------VQLLKKFIKIAAH-
--------------CKEYRNLNSFFAIVM-GLSNVAVSRLALTWE---------------
-------KLPSKFKKFYAE-FESLM--------------------------DPSRNH---
-RAYRL----TAAK-------LEPPLI-PFMP----------------------------
LLIKDMTFTHEGNK--TF-ID-----------------------------N-LVNFE-KM
-------RM---IANTARTVRYYRS--Q-------------------------PFNPD--
-A------------------AQANKNH-----Q----------------DVRS-----YV
RQLNVIDNQRTL---SQMSHRL
-----------EPRR----------P----------------------------------

>Rapgef4_[Maylandia_zebra]_499012713

---MAG-L
LAPRYG--VME--S--GCNA-------DRLPDKE--------------------------
------------------NIS-------NNSFV-VSKPLNKK------------------
-----SFIQSP---AKPH-PKCFS----------------QVPS----------------
-------EKIL-------------RA---------------------GQILR-NAILSRA
PH-M----------------IRDRKYH------L-KTY----------------RRC-CV
GT-----------------ELVDWQLQQS-------------------------------
---SCVH--------SRVQAVGMW---QVLLEEGVLN-----------------------
--------------H------------------------VDQ-ELSFQDKY-LFYRFLDD
-------EQED----AS-F---
--PT

EDER--RESQEEL------QDTLLLLSQIGPDAH-------------LRMILRKPPSERT
ADDLE-----IIYEELLHIKALSHLS-TTVKRELA-GVLIFESHAKAGTVL---------
--FNQGEEG--TSWYIIL
KGSVNVVIYGK-----GV-------------------------------VCTLHEG-DDF
GKLAL-----------VNDAPRA---ASIVLREDNCHFLRVDKEDFNR------ILRD--
---V--------------

------------------------EANTVRLKEHGQDVLVLEK-----SLS-SSRTSVQG
SS----SS-HY------------------------------------K-YNVMSGTPEKI
LEHLLEM------------MRLDS-QFT--------------ES------DSALD---D-
FVLMHCV--FI---------------PNTQLCPVLM-----S------------------
--------H-----------Y-------HAQASQ---GSEQEKLDYTLNNKRRVIRLVMH
WAAVNGDHLQEE-DVSGLFLE----EFFMA--VSNDA--KAFPALR---DQ-LTELERIV
KRN---AED--AR---------SSQKK---------------------------------
---HKV--------LLRHFSVGE-EKLQ-KRQ----------------------------

-----PIRSNDE--IL-----------------------------FK-------------
VYCCDHTYTTIRVPVAASVREVIG--------AVADK-----------LGSA-ED--LLL
VGLSSA----------------------GEKTI------FKPSDMSVFSTLSINGRLFVC
RRDQ---L-DSLT-TLPEQ---------------------------EGP------STGSL
AS--FELMSSKDVAYHMTSYDWELFHCVHELELIY-------HTFGRQYV-KK-TTVNLD
LFLRR---------FNEIQFWV-----ITEMCLCPQLSKR------VQLLKKFIKIAAH-
--------------CKEYRNLNAFFAIIM-GLSNPAVCRLSQTWE---------------
-------KLPSKFKKFYVE-FENLM--------------------------DPSRNH---
-RAYRL----TVAK-------LEPPII-PFMP----------------------------
LLIKDMTFTHEGNK--TF-ID-----------------------------S-LVNFE-KM
-------RM---IANTVKIVRYCRS--Q-------------------------RFSPE--
-S------------------PLPSKNH-----P----------------DVWT-----YI
RQLNVIDNQRML---TQLSHGL
-----------EPRR----------S----------------------------------

>Rapgef4_[Meleagris_gallopavo]_733895485

---MPLSDWE----------


```
------------------------------------------------------------
------------------------------------------------------------
------------------------------------------------------------
------------------------------------------------------------
------------------------------------------------------------
------------------------------------------------------------
------------------------------------------------------------
------------------------------------------------------------
------------------------------------------------------------
------------------------------------------------------------
--------------------GWSQDP-P--------------------------------
--------------------------------------------FPRPHLK--VGW---R
QGNLD---GDSC---DAVT------------------------------ICTLGIG-TAF
GE-SI-----------LDNTPRH---ATIVTRE-YSELLRIEQKDFKA------LWEKYR
QYMSELLTPPYGVMETGSNNDRMPDKDSMSSGALCQVSKNCNKSLM--------------
------------------------------------------------------------
------------------------------------------------------------
------------------------------------------------------------
------------------------------------------------------------
------------------------------------------------------------
------------------------------------------------------------
------------------------------------------------------------
------------------------------------------------------------
------------------------------------------------------------
------------------------------------------------------------
------------------------------------------------------------
------------------------------------------------------------
------------------------------------------------------------
------------------------------------------------------------
------------------------------------------------------------
------------------------------------------------------------
------------------------------------------------------------
------------------------------------------------------------
------------------------------------------------------------
------------------------------------------------------------
------------------------------------------------------------
------------------------------------------------------------
------------------------------------------------------------
------------------------------------------------------------
------------------------------------------------------------
------------------------------------------------------------
------------------------------------------------------------
------------------------------------------------------------
------------------------------------------------------------
------------------------------------------------------------
------------------------------------------------------------
------------------------------------------------------------
------------------------------------------------------------
------------------------------------------------------------
------------------------------------------------------------
------------------------------------------------------------
------------------------------------------------------------
------------------------------------------------------------
------------------------------------------------------------
```


>Rapgef4_[Melopsittacus_undulatus]_527269285
----------------------------MVAA-------HA------SH--------SSS
SGEWIA---CLDK-----------RPLE-----RSSEDVDIIFTRLKEVKAFEKFHPNLL
QQICLCGY--YENLE--KGITLFRQGDIGTNWYAVLTGSLDVKVSD-TSNHQDA------
------VTICTLGIGTAFGESILDNT-PRHATIV-TREYSELL-----------------
----------------------------RIEQKD-------FKALW---EKYRQYMSG-L
LTPPYG--VMETGS--N-N--------DRMPDKD--------------------------
------------------N----------------------T------------------
-----PLIEPHIPHRSTK-TITQ------------------VPS----------------
-------EKIL-------------RA---------------------GKILR-NTILSRA
PH-M----------------IRDRKYH------L-KTY----------------RQC-CV
GT-----------------ELVDWMMQQS-------------------------------
---PCVH--------LRTQAVGMW---QVLLEEGVLN-----------------------
--------------H------------------------VDQ-EHHFQDKY-LFYRFLDD
-------EHED----AP-L---
--PT
EEEK--KECDEEL------QDTMLLLSQIGPDAH-------------MRMILRKPPGQRT
VDDLE-----FIYEELLHIKALSHLS-TTVKRELA-GVLIFESHPKAGTVL---------
--FNQGEEG--TSWYIIL
KGSVNVVIYGK-----GV-------------------------------VCTLHEG-DDF
GKLAL-----------VNDAPRA---ASIVLREDNCHFLRVDKEDFNR------ILRD--
---V--------------

------------------------EANTVRLKEHDQDVLVLEK-----IPA-GNRVSNQG
NS----QP-QH------------------------------------K-YIVMSGTPEKI
LEHFLET------------MRLEA-TLN--------------EA-----TDSVLN---D-
FIMMHCV--FM---------------PNSQLCPALM-----A------------------
--------H-----------Y-------HAQPSQ---GTEQEKMDYALNNKRRVIRLVLQ
WAALYGDLLQED-EAAMAFLE----EFYVS--VSDDT--RMIAALK---EQ-LPELEKIV
KQV---SEE--PK---------APQKK---------------------------------
---HKV--------LLQLFNTSD-DRAQ-KRQ----------------------------

-----PIRGSDE--VL-----------------------------FK-------------
VYCIDQTYTTIRVPVSSSVKEVIS--------AVADK-----------LGSG-EG--LII
VKMSSG----------------------GEKVV------LKPHDVSVFTTLSVNGRLFAC
PRDQ---F-DSLA-PLPEQ---------------------------EGP------STGTV
GT--FELMSSKDLAHQMTIYDWELFNCVHELELIY-------HTFGRHNF-KK-TTANLD
LFLRR---------FNEIQFWV-----VTEICLCAQLSKR------VQLLKKYIKIAAH-
--------------CKEYKNLNSFFAIIM-GLSNVAVSRLSLTWE---------------
-------KLPSKFKKIYAE-FESLM--------------------------DPSRNH---
-RAYRL----TVAK-------LDPPII-PFMP----------------------------
LLIKDMTFTHEGNK--TF-TD-----------------------------N-LVNFE-KM
-------RM---IANTVRTVKFCRS--Q-------------------------SFNPD--
-A------------------ALTNKNH-----Q----------------DVRS-----YV
RQLNVIDNQRTL---SQMSHRL
-----------EPRR----------A----------------------------------

>Rapgef4_[Merops_nubicus]_675626705

---MSG-L
LTPPYG--VMETGS--N-N--------DRMPDKD--------------------------
------------------SMS-------SSALCQV-------------------------
----------------SK-NCNK------------------VPS----------------
-------EKIL-------------RA---------------------GKILR-NTVLSRA
PH-M----------------IRDRKYH------L-KTY----------------RQC-CV
GT-----------------ELVDWMMQQS-------------------------------
---SCVH--------SRTQAVGMW---QVLLEEGVLN-----------------------
--------------H------------------------VDQ-EHHFQDKY-LFYRFLDD
-------ERED----AP-L---
--PT
EEEK--KECDEEL------QDTMLLLSQIGPDAH-------------MRMILRKPPGQRT
VDDLE-----FIYEELLHIKALSHLS-TTVKRELA-GVLIFESHPKAGTVL---------
--FNQGEEG--TSWYIIL
KGSVNVVIYGK-----GV-------------------------------VCTLHEG-DDF
GKLAL-----------VNDAPRA---ASIVLREDNCHFLRVDKEDFNR------ILRD--
---V--------------

------------------------EANTVRLKEHDQDVLVLEK-----IPA-GNRVSNQG
NS----QP-QH------------------------------------K-YIVMSGTPEKI
LEHFLET------------MRLEA-TLN--------------EA-----TGSVLN---D-
FIMMHCV--FM---------------PNSQLCPALM-----A------------------
--------H-----------Y-------HAQPSQ---GTEQEKMDYALNNKRRVIRLVLQ
WAALYGDLLQED-EAAMAFLE----EFYVS--VSDDT--RMIEALK---EQ-LPELEKIV
KQV---SEE--PK---------APQKK---------------------------------
---HKV--------LLQLFNTND-DRAQ-KRQ----------------------------

-----PIRGSDE--VL-----------------------------FK-------------
VYCIDQTYTTIRVPVSSSVKEVIS--------AVADK-----------LGSG-EG--LII
VKMSSG----------------------GEKVV------LKPHDVSVFTTLSVNGRLFAC
PRHQ---F-DSLA-PLPEQ---------------------------EGP------SAGTV
GT--FELMSSKDLAHQMTIYDWELFNCVHELELIY-------HTFGRHNF-KK-TTANLD

LFLRR---------FNEIQFWV-----VTEICLCSQLSKR------VQLLKKYIKIAAH-
--------------CKEYKNLNSFFAIIM-GLSNVAVSRLSLTWE---------------
-------KLPSKFKKIYAE-FESLM--------------------------DPSRNH---
-RAYRL----TVAK-------LDPPII-PFMP----------------------------
LLIKDMTFTHEGNK--TF-TD-----------------------------N-LVNFE-KM
-------RM---IANTVRTVKFCRS--Q-------------------------SFNPD--
-T------------------ALANKNH-----Q----------------DVRS-----YV
RQLNVIDNQRTL---SQMSHRL
-----------EPRR----------A----------------------------------

>Rapgef4_[Mesitornis_unicolor]_704578686

---------------M-----------------------------FT-------------

-----------------------------EKVV------LKPHDVSVFTTLSVNGRLFAC
PRDQ---F-DSLA-PLPEQ---------------------------EGP------SAGTV
GT--FELMSSKDLAHQMTIYDWELFNCVHELELIY-------HTFGRHNF-KK-TTANLD
LFLRR---------FNEIQFWV-----VTEICLCSQLSKR------VQLLKKYIKIAAH-
--------------CKEYKNLNSFFAIIM-GLSNVAVSRLSLTWE---------------
-------KLPSKFKKIYAE-FESLM--------------------------DPSRNH---
-RAYRL----TVAK-------LDPPII-PFMP----------------------------
LLIKDMTFTHEGNK--TF-TD-----------------------------N-LVNFE-KM
-------RM---IANTVRTVKFCRS--Q-------------------------SFNPD--
-A------------------ALTNKN----------HQ-----------DVRS-----YV
RQLNVIDNQRTL---SQMSHRL
-----------EPRR----------A----------------------------------

>Rapgef4_[Mesocricetus_auratus]_880859282
----------------------------M-------------------------------

--LY---KKYRQYMAG-L
LAPPYG--VMETGS--N-N--------DRIPDKE--------------------------
------------------N---
---VPS----------------
-------EKIL-------------RA---------------------GKILR-IAILSRA
PH-M----------------IRDRKYH------L-KTY----------------RQC-CV
GT-----------------ELVDWMIQQT-------------------------------
---SCVH--------SRTQAVGMW---QVLLEDGVLN-----------------------
--------------H------------------------VDQ-ERHFQDKY-LFYRFLDD
-------EHED----AP-L---
--PT
EEEK--KECDEEL------QDTMLLLSQMGPDAH-------------MRMILRKPPGQRT
VDDLE-----IIYDELLHIKALSHLS-TTVKRELA-GVLIFESHAKGGTVL---------
--FNQGEEG--TSWYIIL
KGSVNVVIYGK-----GV-------------------------------VCTLHEG-DDF
GKLAL-----------VNDAPRA---ASIVLREDNCHFLRVDKEDFNR------ILRD--
---V--------------

------------------------EANTVRLKEHDQDVLVLEK-----VPA-GSRASNQG

NS----QP-QQ------------------------------------K-YTVMSGTPEKI
LEHFLET------------IRLEP-SLN--------------EA-----TDAVLN---D-
FVMMHCV--FM---------------PNTQLCPALV-----A------------------
--------H-----------Y-------HAQPSQ---GTEQERMDYALNNKRRVIRMVLQ
WAAMYGDLLQED-DVAMAFLE----EFYVS--VSDDA--RMMAAFK---EQ-LPELEKIV
KQI---SED--AK---------APQKK---------------------------------
---HKV--------LLQQFNTGD-ERAQ-KRQ----------------------------

-----PIRGSDE--VL-----------------------------FK-------------
VYCMDHTYTTIRVPVAASVKEVIS--------AVADK-----------LGSG-EG--LII
VKMNSG----------------------GEKVV------LKPNDVSVFTTLTINGRLFAC
PREQ---F-DSLT-PLPEQ---------------------------EGP------TTGTV
GT--FELMSSKDLAYQMTTYDWELFNCVHELELIY-------HTFGRHNF-KK-TTANLD
LFLRR---------FNEIQFWV-----VTEICLCSQLSKR------VQLLKKFIKIAAH-
--------------CKEYKNLNSFFAIVM-GLSNVAVSRLALTWE---------------
-------KLPSKFKKFYAE-FESLM--------------------------DPSRNH---
-RAYRL----TAAK-------LDPPLI-PFMP----------------------------
LLIKDMTFTHEGNK--TF-ID-----------------------------N-LVNFE-KM
-------RM---IANTARTVRYCRS--Q-------------------------PFNPD--
-A------------------AQANKNH-----Q----------------DVRS-----YV
RQLNVIDNQRTL---SQMSHRL
-----------EPRR----------P----------------------------------

>Rapgef4_[Metaseiulus_occidentalis]_391334871

----------MDK-----------RCTD-----RTEDDLEMIYAKLRSMPPFSQLHPVLL
QQLSFYGY--YEDID--AGVTLFREGDKGKNWYAVLKGSLDVQIANPSGASNDN------
------VTLCTLGVGRAFGESISGLSLPHSATVV-TNECCELLRIDQRDFQLLWERN---
--RH--LMEDMI------------------------SPLSTLRGITHH-PVHPSLASQ-Q
ATQQQSQSSIHQGSHQTLNKMTSLGAPEHIGPPEPIRRASTNS-----------------
-----------------VRLSGEGQFQLQDTHPQLNNKFYPS------------------
-----SKVIPPVPQVAGL-QQNQQSMQQSQQLHLHQAQQQQPQPQQQFHLQQ-------
---QNSASALA-------------RA---------------------SWVLR-TVLLYQA
PQ-L----------------VRDRRVT------PGRAF----------------KRC-MV
GT-----------------EMVDWMLQMG-------------------------------
CVHVRLS--------SRAQAAGMW---QVLLEEGCLN--------------------
--------------H------------------------VTG-ERQFEDKY-LFYRFRDD
-------DAEH----PR-L---
--PT

AEER--PIAEAEL------QDWLVYLMQLAPDAN-------------LRMILRKPPSERR
QDDLD-----TIYEELTRIKALSHLS-NSVKRELS-GVMVFESHPKRDTAL---------
--FNQGDEG--KSWYIIL
KGSVNVVIYGK-----GV-------------------------------VCELHEG-DDF
GKLAL-----------VNDAPRA---ATIITREENCHFLRVDKHDFNR------ILRD--
---V--------------

------------------------EANTVRLKEHGQDVLVLQK-----NAQ-----DVSI
-----SHV-HS------------------------------------RTYTVMAGTPQKM
LEHLLETRIGERNDEMQTAASAA---------------------------DSFLE---D-
FLLTHVI--FM---------------PSHDLCPELM-----R------------------
--------HYRLDAVN-----------------------QRQEREFILASKRRVVQFTAQ
WAAIVREPFYQD-PVIQCFLE----ELLSA--IEEDR--QKYLGA--LQDQ-LSIVQRLF
EIV-DKYRSDPSM---------TSGQK---------------------------------
---WKVDTNGQIRHLSSGSTQTD-ENSQ-EHI----------------------------

-----------------------------IR-----------------------------

-----AIRDTDE--II------------------------------FR-------------
VYCADHTYVTLKTAVKATANSIKK--------LAAEK-----------IALK-DE--VIL
VEVKSN----------------------GEKLP------FNESEVSVPTGLSINGRIFIS
PIDH---I-DALT-PLQEQ---------------------------DGP------NEGTA
NI--LEALSSQDIAYHMTVYEWQLFTTVHEYELIY-------QVFGRNQF-RR-IMSNLD
VFQRR---------FNQVQFWV-----VTEMCLANSLSRR------VTLLRKFIKIAQH-
--------------CREYQNLNALFAIVM-GLGNAAVSRLGQTWE---------------
-------RLPSKLRRSFEE-LENLI--------------------------EPSRNH---
-KRYRN----AVAK-------LQPPLI-PFIP----------------------------
LLLKDMTFCHVGNK--SY-ID-----------------------------G-LVNFE-KM
-------HM---IGQTLRHLRHSRS--Q-------------------------RLAMEP-
-P------------------QGSSKSH-----H----------------NVAD-----YI
RNLKVIDNQRTL---TKLSHQL
-----------EPRR----------S----------------------------------

>Rapgef4_[Microtus_ochrogaster]_532005567
----------------------------MVAA-------HA------AH--------SPS
SAEWIA---CLDK-----------RPLE-----RSSEDVDIIFTRLKEVKAFEKFHPNLL
HQICLCGY--YENLE--KGITLFRQGDIGTNWYAVLAGSLDVKVSE-TSSHQDA------
------VTICTLGIGTAFGESILDNT-PRHATIV-TRESSELL-----------------
----------------------------RIEQED-------FKTLW---EKYRQYMAG-L
LAPPYG--VMETGS--N-N--------DRIPDKE--------------------------

```
------------------N-----------------------------------------
-----------------------------------------VPS----------------
-------EKIL-------------RA---------------------GKILR-IAILSRA
PH-M----------------IRDRKYH------L-KTY----------------RQC-CV
GT-----------------ELVDWMIQQT-------------------------------
---SCVH--------SRTQAVGMW---QVLLEDGVLN-----------------------
--------------H------------------------VDQ-ERHFQDKY-LFYRFLDD
-------EHED----AP-L-----------------------------------------
----------------------------------------------------------PT
DEEK--KECDEEL------QDTMLLLSQMGPDAH-------------MRMILRKPPGQRT
VDDLE-----IIYDELLHIKALSHLS-TTVKRELA-GVLIFESHAKGGTVL---------
--------------------------------------------FNQGEEG--TSWYIIL
KGSVNVVIYGK-----GV-------------------------------VCTLHEG-DDF
GKLAL-----------VNDAPRA---ASIVLREDNCHFLRVDKEDFNR------ILRD--
---------------------------------------------V--------------
------------------------------------------------------------
------------------------------------------------------------
------------------------EANTVRLKEHDQDVLVLEK-----VPA-GSRASNQG
NS----QP-QQ------------------------------------K-YTVMSGTPEKI
LEHFLET------------IRLEP-SLN--------------EA-----TDSVLN---D-
FVMMHCV--FM---------------PNTQLCPALV-----A------------------
--------H-----------Y-------HAQPSQ---GTEQERMDYALNNKRRVIRLVLQ
WAALYGDLLQED-DVAMAFLE----EFYVS--VSDDA--RMMAAFK---EQ-LPELEKIV
KQI---SED--AK---------APQKK---------------------------------
---HKV--------LLQQFNTGD-ERAQ-KRQ----------------------------
------------------------------------------------------------
------------------------------------------------------------
------------------------------------------------------------
------------------------------------------------------------
------------------------------------------------------------
------------------------------------------------------------
-----PIRGSDE--VL-----------------------------FK-------------
VYCMDHTYTTIRVPVAASVKEVIS--------AVADK-----------LGSG-EG--LII
VKMNSG----------------------GEKVV------LKPNDVSVFTTLTINGRLFAC
PREQ---F-DSLT-PLPEQ---------------------------EGP------TTGTV
GT--FELMSSKDLAYQMTTYDWELFNCVHELELIY-------HTFGRHNF-KK-TTANLD
LFLRR---------FNEIQFWV-----VTEICLCSQLSKR------VQLLKKFIKIAAH-
--------------CKEYKNLNSFFAIVM-GLSNVAVSRLALTWE---------------
-------KLPSKFKKFYAE-FESLM--------------------------DPSRNH---
-RAYRL----TAAK-------LDPPII-PFMP----------------------------
LLIKDMTFTHEGNK--TF-ID-----------------------------N-LVNFE-KM
-------RM---IANTARTVRYYRS--Q-------------------------PFNPD--
-A------------------AQANKNH-----Q----------------DVRS-----YV
RQLNVIDNQRTL-----------------------------------------SQMSHRL
-----------EPRR----------P----------------------------------
------------------------------------------------------------
------------------------------------------------------------
------------------------------------------------------------
------------------------------------------------------------
------------------------------------------------------------
------------------------------------------------------------
------------------------------------------------------------
------------------------------------------------------------
------------------------------------------------------------
```


>Rapgef4_[Miniopterus_natalensis]_1016626283

---MAG-L
LAPPYG--VMETGS--N-N--------DRIPDKE--------------------------
------------------N----------------------T------------------
-----PPIEPHVPPRPAN-TIPK------------------VPS----------------
-------EKIL-------------RA---------------------GKILR-NAILSRA
PH-M----------------IRDRKYH------L-KTY----------------RQC-CV
GT-----------------ELVDWMLQQT-------------------------------
---PCVH--------SRTQAVGMW---QVLVEDGVLN-----------------------
--------------H------------------------VDQ-EHHFQDKY-LFYRFLDD
-------DHED----AP-L---
--PT
EEEK--KECDEEL------QDTMLLLSQMGPDAH-------------MRMILRKSPGQRN
MDDLE-----IIYDELLHIKALSHLS-TTVKRELA-GVLIFESHAKGGTVL---------
--FNQGEEG--TSWYIIL
KGSVNVVIYGK-----GV-------------------------------VCTLHEG-DDF
GKLAL-----------VNDAPRA---ASIVLREDNCHFLRVDKEDFNR------ILRD--
---V--------------

------------------------EANTVRLKEHDQDVLVLEK-----VPA-GSRASNQG
NS----QP-QQ------------------------------------K-YTVMSGTPEKI
LEHFLET------------IRLEP-ALN--------------EA-----TDSFLN---D-
FVMMHCV--FM---------------PNTQLCPALM-----A------------------
--------H-----------Y-------HAQPSQ---GTEQEKVDYALNNKRRVVRLVLQ
WASAYGDLLQED-SVAMAFLE----EFYVS--VSDDA--RMIAALK---EQ-LPELERIV
KQI---SED--AK---------NPQKK---------------------------------
---HKV--------LLQHFNTGD-ERAQ-KRQ----------------------------

-----PIRGSDE--VL-----------------------------FK-------------
VYCMDHTYTTIRVPVAASVKEVIS--------AVADK-----------LGSG-EG--LII
VKMSSG----------------------GEKVV------LKPNDVSVFTTLTINGRLFAC
PREQ---F-DSLT-PLPEQ---------------------------EGP------TVGTV
GT--FELMSSKDLAYQMTIYDWELFNCVHELELIY-------HTFGRHNF-KK-TTANLD
LFLRR---------FNEIQFWV-----ITEICLCSQPSKR------VQLLKKFIKIAAH-
--------------CKEYKNLNSFFAIVM-GLSNVAVSRLALTWE---------------
-------KLPSKFKKFYAE-FESLM--------------------------DPSRNH---
-RAYRL----TVAK-------LEPPLI-PFMP----------------------------
LLIKDMTFTHEGNK--TF-ID-----------------------------N-LVNFE-KM
-------RM---IANTARMVRYCRS--Q-------------------------PFNLD--
-A------------------AQANKNH-----Q----------------DVRS-----YV
RQLNVIDNQRTL---SQMSHRL
-----------EPRR----------P----------------------------------

>Rapgef4_[Monodelphis_domestica]_1023002080

---MAG-L
LAPPYG--VMETSA--N-N--------DRMPDKE--------------------------
------------------N---
-------VSSSNLCSASK-NCNK------------------VPS----------------
-------EKIL-------------RA---------------------GKILR-NAILSRA
PH-M----------------IRDRKYH------L-KTY----------------RQC-CV
GT-----------------ELVDWMIQQT-------------------------------
---PCVH--------SRAQAVGMW---QVLLEEGVLN-----------------------
--------------H------------------------VDQ-EHHFQDKY-LFYRFLDD
-------EHED----AP-L---
--PT
EEEK--KECDEEL------QDTMLLLSQMGPDAH-------------MRMILRKPPGQRT
VDDLE-----IIYEELIHIKALSHLS-TTVKRELA-GVLIFESHAKGGTVL---------
--FNQGEEG--TSWYIIL
KGSVNVVIYGK-----GV-------------------------------VCTLHEG-DDF
GKLAL-----------VNDAPRA---ASIVLREDNCHFLRVDKEDFNR------ILRD--
---V--------------

------------------------EANTVRLKEHDQDVLVLEK-----VPA-GNRASNQG
NS----QP-QQ------------------------------------K-YTVMSGTPEKI
LEHFLET------------VRLEQ-PLN--------------EV-----TDSVLN---D-
FIMMHCV--FM---------------PNSQLCPALV-----A------------------
--------H-----------Y-------HAQPSQ---GTEQDKMDYALNNKRRVIRLVLQ
WAALYGDLLQED-EVAMAFLE----EFYVS--VSDDS--RMIAALK---DQ-LPELEKMV
KQI---SED--AK---------APQKK---------------------------------
---HKV--------LLQQFNPVE-DRAQ-KRQ----------------------------

-----PVRGSDE--IL-----------------------------FK-------------
VYCLDHTYTTIRVPVSASVKEVIS--------AIADK-----------LGSG-EG--MII
VKMSSG----------------------GEKVV------LKPNDVSVFTTLTVNGRLFAC
PREQ---F-DSLT-PLPEQ---------------------------EGP------TTGTV
GT--FELMSSKDLAYQMTIYDWELFNCVHELELIY-------HTFGRHNF-KK-TTANLD

LFLRR---------FNEIQFWV-----VTEICLCAQLSKR------VQLLKKFIKIAAH-
--------------CKEYKNLNSFFAIVM-GLSNVAVSRLALTWE---------------
-------KLPSKFKKIYAE-FESLM--------------------------DPSRNH---
-RAYRL----TVTK-------LDPPLI-PFMP----------------------------
LLIKDMTFTHEGNK--TF-ID-----------------------------N-LVNFE-KM
-------RM---IANTVRTVRYCRS--Q-------------------------TFNPD--
-A------------------ALANKNH-----Q----------------DVRG-----YV
RQLNVIDNQRTL---SQMSHRL
-----------EPRR----------A----------------------------------

>Rapgef4_[Mus_musculus]_1039761433

---MAG-L
LAPPYG--VMETGS--N-N--------DRIPDKE--------------------------
------------------N---
---VPS----------------
-------EKIL-------------RA---------------------GKILR-IAILSRA
PH-M----------------IRDRKYH------L-KTY----------------RQC-CV
GT-----------------ELVDWMIQQT-------------------------------
---SCVH--------SRTQAVGMW---QVLLEDGVLN-----------------------
--------------H------------------------VDQ-ERHFQDKY-LFYRFLDD
-------ERED----AP-L---
--PT
EEEK--KECDEEL------QDTMLLLSQMGPDAH-------------MRMILRKPPGQRT
VDDLE-----IIYDELLHIKALSHLS-TTVKRELA-GVLIFESHAKGGTVL---------
--FNQGEEG--TSWYIIL
KGSVNVVIYGK-----GV-------------------------------VCTLHEG-DDF
GKLAL-----------VNDAPRA---ASIVLREDNCHFLRVDKEDFNR------ILRD--
---V--------------

------------------------EANTVRLKEHDQDVLVLEK-----VPA-GNRAANQG
NS----QP-QQ------------------------------------K-YTVMSGTPEKI
LEHFLET------------IRLEP-SLN--------------EA-----TDSVLN---D-
FVMMHCV--FM---------------PNTQLCPALV-----A------------------
--------H-----------Y-------HAQPSQ---GTEQERMDYALNNKRRVIRLVLQ
WAAMYGDLLQED-DVAMAFLE----EFYVS--VSDDA--RMMAAFK---EQ-LPELEKIV
KQI---SED--AK---------APQKK---------------------------------
---HKV--------LLQQFNTGD-ERAQ-KRQ----------------------------

-----PIRGSDE--VL-----------------------------FK-------------
VYCIDHTYTTIRVPVAASVKEVIS--------AVADK-----------LGSG-EG--LII
VKMNSG----------------------GEKVV------LKSNDVSVFTTLTINGRLFAC
PREQ---F-DSLT-PLPEQ---------------------------EGP------TTGTV
GT--FELMSSKDLAYQMTTYDWELFNCVHELELIY-------HTFGRHNF-KK-TTANLD
LFLRR---------FNEIQFWV-----VTEVCLCSQLSKR------VQLLKKFIKIAAH-
--------------CKEYKNLNSFFAIVM-GLSNVAVSRLALTWE---------------
-------KLPSKFKKFYAE-FESLM--------------------------DPSRNH---
-RAYRL----TAAK-------LEPPLI-PFMP----------------------------
LLIKDMTFTHEGNK--TF-ID-----------------------------N-LVNFE-KM
-------RM---IANTARTVRYYRS--Q-------------------------PFNPD--
-A------------------AQANKNH-----Q----------------DVRS-----YV
RQLNVIDNQRTL---SQMSHRL
-----------EPRR----------P----------------------------------

>Rapgef4_[Musca_domestica]_557781731
-------MASNSSSISSESLR-SAASSKLSSSSSATKRWSQHIVASTYDDVCVLLKYQRR
PHSQENLSQISGG------------ECA-----MTNKYPNSYATLPTPHHHYHHHNNSHH
HTPSLPAP--PPPYN--NNNNTIKCNNIG----LVANGCCNTKSKPTTTSMAT------S
V-----LTLSSSVLLRNGGPSSNH-Y-RRGETPR-RRLIRPVSCFEPGDH----------
KNKE--LMNDII-------------T-SCKFKNGFGPNT-Q-QQQQQQQSN-SS-----
---PT------------------K--RPLSPDHPNPAL----------------------

--PITE--------------
-----------------SPSPAMNRM---------------------GLALR-TLLVADN
SS-C----------------LKDRKVS------G-K-L---------------IRKC-AP
GT-----------------EFVDWLVNLS-------------------------------
---SIVH--------TRAQAAGMW---QALLEEGILS-----------------------
--------------H------------------------VNK-EQPFKDKC-FLYRFRVD
E---------E----G--
---TAGGSPS
ADDI--NAANEHI------RENLSTLFQRGPDAT-------------LRMILRKPSHERT
PEELE-----LVFEELLHIAALSHLS-TSIKRELS-SIIVFEAHAQAGTIL---------
--FSQGDEG--RSWYIIL
KGSVDVVIHGK-----GT-------------------------------VATLKSG-DDF
GKLAL-----------INDAPRA---ATIVLKENNCHLLRVDKEHFNR------ILRD--
---V--------------

------------------------EANTLRLQEHGKDVLVLER-----VAK---------

------QRGQ----HS---------------AFK---------------YTVMSGTPSKM
LEHLLET-------------RL---G---G-------QISG--------VDPFLD---D-
FLLTHIV--FM---------------PVVQLVDELA-----N------------------
--------YF---HCDS---------H-----DG---SQTPEERDYIINFKKRVIQFMQK
WVMAVRHAAFDE-PSVCDFIE----DLAAE--VEAD-----------------PELQEET
SII---
---HNI--------LTQMARYQD-DRNQNAGQK---------------------------
----WKLPP---NGQPIC--------------------------------LFSGNIT---
---PSKT---V---

------IRPDDD--II-----------------------------FR-------------
VYCADHTYCTLRFPLHTTAEIIKA--------CAADK-----------LQLNRGPEDLCL
VEVKSN----------------------GERSV------FKDNDVSIPTGLSLNGRLFVS
VKDH---I-DGLT-PLPEQ---------------------------EGP------TEGID
M--DLEMLSTKELAYHITIFDWDLFWAVHEYELLY-------HTFGRHHF--GKITANLD
VFLRR---------FNELQYWI-----VTEMVSTSSMSKR------VGLLRKFIKLAAY-
--------------CKEYQNLNAFFAIVM-GLSNMAVSRLQQTWE---------------
-------KIPSKFRKLYQE-FEALI--------------------------DPSRNH---
-RAYRV----FVGK-------LQPPLI-PFMP----------------------------
LLLKDMTFAHEGNK--TS-ID-----------------------------G-LVNFE-KM
-------HM---MAQTMRTLRFCRS--R------SLGL-----------------DPP--
----------------------SPKSE-----G----------------EVRS-----FI
SCLRVIDNQRVL---TAMSQKI
-----------EPTR----------KI---------------------------------

>Rapgef4_[Myotis_davidii]_432107330

--------------------------------------MFNSLMTS-FGDSQDA------
------VTICTLGIGTAFGESILDNT-PRHATIV-TRESSELL-----------------
----------------------------RIEQKD-------FKALW---EKYRQYMAG-L
LAPPYG--VMETGS--N-N--------DRIPDKE--------------------------
------------------N----------------------T------------------
-----PLIEPPAPLRPAN-TITK------------------VPS----------------
-------EKIL-------------RA---------------------GKILR-NTILSRA
PH-M----------------IRDRKYH------L-KTY----------------RQC-CV
GT-----------------ELVDWMLQQT-------------------------------
---PCVH--------SRTQAVGMW---QVLVEDGVLN-----------------------
--------------H------------------------ADQ-EHHFQDKY-LFYRFLDD
-------EHED----AP-L---
--PT

EEEK--KECDEEL------QDTMLLLSQMGPDAH-------------MRMILRKSPGQRS
MDDLE-----IIYDELLHIKALSHLS-TTVKRELA-GVLIFESHAKGGTVL---------
--FNQGEEG--TSWYIIL
KGSVNVVIYGK-----GV-------------------------------VCTLHEG-DDF
GKLAL-----------VNDAPRA---ASIVLREDNCHFLRVDKEDFNR------ILRD--
---V--------------

------------------------EANTVRLKEHDQDVLVLEK-----VPA-GSRASNQG
NS----QP-QQ------------------------------------K-YTVMSGTPEKI
LEHFLET------------IRLEP-ALN--------------EA-----TDSVLN---D-
FVMMHCV--FM---------------PNTQLCPALV-----AQY------PFSVKSLYSL
QDTLHVHGH-----------Y-------LTPASE---GTEQEKMDYALNNKRRVVRLVLQ
WAAVYGDLLQED-EVAMAFLE----EFYVS--VSDDA--RMIAALK---EQ-LPELERIV
KQI---SED--AK---------NPQKK---------------------------------
---HKV--------LLQHFNTSD-ERAQ-KRQ----------------------------

-----PIRGSDE--VL-----------------------------FK-------------
VYCMDHTYTTIRVPVAASVREVIS--------AVADK-----------LGSG-EG--LII
VKMSSG----------------------GEKVV------LKPNDVSVFTTLTINGRLFAC
PREQ---F-DSLT-PLPEQ---------------------------EGP------TVGTV
GT--FELMSSKDLAYQMTIYDWELFNCVHE------------------------------
---VSRLIESVSS-
--------------CKEYKNLNSFFAIVM-GLSNVAVSRLALTWE---------------
-------KLPSKFKKFYAE-FESLM--------------------------DPSRNH---
-RAYRL----TVAK-------LEPPLI-PFMP----------------------------
LLIKDMTFTHEGNK--TF-ID-----------------------------N-LVNFE-KM
-------RM---IANTARTVRYCRS--Q-------------------------PFNLD--
-A------------------AQANKNH-----Q----------------DVRS-----YV
RQLNVIDNQRTL---SQMSHRL
-----------EPRR----------P----------------------------------

>Rapgef4_[Myotis_lucifugus]_558101259

---MAG-L
LAPPYG--VMETGS--N-N--------DRIPDKE--------------------------

```
------------------N-----------------------------------------
-----------------------------------------VPS----------------
-------EKIL-------------RA---------------------GKILR-NTILSRA
PH-M----------------IRDRKYH------L-KTY----------------RQC-CV
GT-----------------ELVDWMLQQT-------------------------------
---PCVH--------SRTQAVGMW---QVLVEDGVLN-----------------------
--------------H------------------------VDQ-EHHFQDKY-LFYRFLDD
-------EHED----AP-L-----------------------------------------
----------------------------------------------------------PT
EEEK--KECDEEL------QDTMLLLSQMGPDAH-------------MRMILRKSPGQRS
MDDLE-----IIYDELLHIKALSHLS-TTVKRELA-GVLIFESHAKGGTVL---------
--------------------------------------------FNQGEEG--TSWYIIL
KGSVNVVIYGK-----GV-------------------------------VCTLHEG-DDF
GKLAL-----------VNDAPRA---ASIVLREDNCHFLRVDKEDFNR------ILRD--
---------------------------------------------V--------------
------------------------------------------------------------
------------------------------------------------------------
------------------------EANTVRLKEHDQDVLVLEK-----VPA-GSRASNQG
NS----QP-QQ------------------------------------K-YTVMSGTPEKI
LEHFLET------------IRLEP-ALN--------------EA-----TDSVLN---D-
FVMMHCV--FM---------------PNTQLCPALV-----A------------------
--------H-----------Y-------HAQPSQ---GTEQEKMDYALNNKRRVVRLVLQ
WAAVYGDLLQED-EVAMAFLE----EFYVS--VSDDA--RMITALK---EQ-LPELERIV
KQI---SED--AK---------NPQKK---------------------------------
---HKV--------LLQHFNTSD-ERAQ-KRQ----------------------------
------------------------------------------------------------
------------------------------------------------------------
------------------------------------------------------------
------------------------------------------------------------
------------------------------------------------------------
------------------------------------------------------------
-----PIRGSDE--VL-----------------------------FK-------------
VYCMDHTYTTIRVPVAASVREVIS--------AVADK-----------LGSG-EG--LII
VKMSSG----------------------GEKVV------LKPNDVSVFTTLTINGRLFAC
PREQ---F-DSLT-PLPEQ---------------------------EGP------TVGTV
GT--FELMSSKDLAYQMTIYDWELFNCVHELELIY-------HTFGRHNF-KK-TTANLD
LFLRR---------FNEIQFWV-----VTEICLCSQPSKR------VQLLKKFIKIAAH-
--------------CKEYKNLNSFFAIVM-GLSNVAVSRLALTWE---------------
-------KLPSKFKKFYAE-FESLM--------------------------DPSRNH---
-RAYRL----TVAK-------LEPPLI-PFMP----------------------------
LLIKDMTFTHEGNK--TF-ID-----------------------------N-LVNFE-KM
-------RM---IANTARTVRYCRS--Q-------------------------PFNLD--
-A------------------AQANKNH-----Q----------------DVRS-----YV
RQLNVIDNQRTL-----------------------------------------SQMSHRL
-----------EPRR----------P----------------------------------
------------------------------------------------------------
------------------------------------------------------------
------------------------------------------------------------
------------------------------------------------------------
------------------------------------------------------------
------------------------------------------------------------
------------------------------------------------------------
------------------------------------------------------------
------------------------------------------------------------
```


>Rapgef4_[Neolamprologus_brichardi]_583982936
---------------------------------MV-----A------VQ---TSPNSSPS
-AEWIC---CLDK-----------RPAE-----RSGEDVDIILTRLREVKAFQRFPPPLL
LQICACAF--YECLE--KGITLFRQGDIGTSWYAVLSGSLDVKVSE-TANHQDA------
------VTICTLGIGTAFGESILDNT-PRHATIV-SRETSELL-----------------
----------------------------RIEQRE-------FKTLWE---KYRQSLAA-L
LAPPYG--AMESGS----NN-------DRLTDKD--------------------------
------------------NMN-------S-------------------------------
--------DSA---NKAH-----N----------------KIPS----------------
-------EKLQ-------------RA---------------------GKVLR-NAILSRA
PH-M----------------IRDRKYH------L-KTY----------------RQC-CV
GT-----------------ELVDWLVLQS-------------------------------
---ACVL--------TRSHAVGMW---QALLEEGVLN-----------------------
--------------H------------------------VDQ-ELGFYDKY-LFYRFLDD
-------EEED----TP-L---
--PS
EEEK--RESEEEL------PETILFLAQIGPDAL-------------LRMILRKSPGQRT
GDDLE-----IIYDELLHIKALAHLS-NTVKRELA-SVVIFESHAKAGTVL---------
--FNQGEEG--TSWYIIL
KGSVNVVIYGK-----GV-------------------------------VCTLHEG-DDF
GKLAL-----------VTDSPRA---ASIVLREDNCHFLRVDKEDFNR------ILRD--
---V--------------

------------------------EANTVRLKEHDHPVLVLEK-----S----PRASTLG
N---------I------------------------------------K-YTVISGTPEKI
LEHFLET------------MRMDI-HHS--------------DP------DPAVD---D-
FVLMHCV--FM---------------PNSQLCPLLL-----S------------------
--------Q-----------Y-------HVTSPP---GSEQERLEYALNSKRRALIMTLR
WANAHTYLLQEE-PAAIFFLE----ELYGS--LSNDS--RMLRALK---DL-VPDLEKVV
KLH---SEE--AK---------ATKKK---------------------------------
-----T--------LIRQFSNGE-ERLQ-KKQ----------------------------

-----PIRNQDD--IL-----------------------------LK-------------
VYCSDHTYTTIRIPVAATGREVIS--------AVTDK-----------LGTT-DE--LLL
VHLSSA----------------------GEKQI------LKPNDVSVFSTLSINGRLFAC
PREQ---L-NSLT-PLPDQ---------------------------EGP------TAGSM
ST--FELMSSKDLAYQMTMFDWELFSCVHEHELLY-------HTFGCQSF-KR-TTANLD
LFLRR---------FNQVQLWV-----VTEVCLCGQLSKR------VQLLKKFIKIAAH-
--------------CREFKNLNSFFAIIM-GMSNPAVSRLSQTWE---------------
-------KLPTKFKKFYAE-FENMM--------------------------DPSRNH---
-RSYRL----TVTK-------LEPPII-PFVP----------------------------
LLLKDMTFTHEGNK--TF-ID----------------------------N-MVNFE-KM
-------RI---IANTIRQVRHCRS--Q-------------------------PFNPD--
-I------------------CQPNKNQ-----A----------------EVRG-----YV
RKLCVIDNQRAL---TQLSYRL
-----------EPRR----------T----------------------------------

>Rapgef4_[Nestor_notabilis]_677450494

---Y------H---------
-----------------------------AQPSQ---GTEQEKMDYALNNKRRVIRLVLQ
WAALYGDLLQED-EAAMAFLE----EFYVS--VSDDT--RMIAALK---EQ-LPE-----
------LEKIVKQV--SE------EPKAPQ---------------------KKH------
------K-----------VLLQ--------------------------------------
-----------------L--
----FNTS-DD-----------------RAQKR---------------------------
--QPIRG---------------

---SD-------------
-------------EVL-----------------------------FK-------------
VYCIDQTYTTIRVPVSSSVKEVIS--------AVADKLG-------------SGEG-LII
VKMSSG----------------------G-------------------------------

>Rapgef4_[Nipponia_nippon]_694839384
----------------------------MVAA-------HT------SH--------SSS
SGEWIA---CLDK-----------RPLE-----RSSEDVDIIFTRLKEVKAFEKFHPNLL
QQICLCGY--YENLE--KGITLFRQGDIGTNWYAVLTGSLDVKVSD-TSNHQDA------
------VTICTLGIGTAFGESILDNT-PRHATIV-TRECSELL-----------------
----------------------------RIEQKD-------FKALW---EKYRQYMSG-L
LTPPYG--VMETGS--N-N--------DRMPDKD--------------------------
------------------N----------------------T------------------
-----PLIEPHIPHRPTK-TITQ------------------VPS----------------
-------EKIL-------------RA---------------------GKILR-NTILSRA
PH-M----------------IRDRKYH------L-KTY----------------RQC-CV
GT-----------------ELVDWMMQQS-------------------------------
---PCVH--------SRTQAVGMW---QVLLEEGVLN-----------------------
--------------H------------------------VDQ-EHHFQDKY-LFYRFLDD
-------EHED----AP-L---
--PT
EEEK--KECDEEL------QDTMLLLSQIGPDAH-------------MRMILRKPPGQRT
VDDLE-----IIYEELLHIKALSHLS-TTVKRELA-GVLIFESHPKAGTVL---------
--FNQGEEG--TSWYIIL
KGSVNVVIYGK-----GV-------------------------------VCTLHEG-DDF
GKLAL-----------VNDAPRA---ASIVLREDNCHFLRVDKEDFNR------ILRD--
---V--------------

------------------------EANTVRLKEHDQDVLVLEK-----IPA-GNRVSNQG
NS----QP-QH------------------------------------K-YIVMSGTPEKI
LEHFLET------------MRPEA-TLN--------------EA-----TDSVLN---D-
FIMMHCV--FM---------------PNSQLCPALM-----A------------------
--------H-----------Y-------HAQPSQ---GTEQEKMDYALNNKRRVIRLVLQ
WAALYGDLLQED-EAAMAFLE----EFYVS--VSDDT--RMIAALK---EQ-LPELEKVV
KQV---SEE--PK---------APQKK---------------------------------
---HKV--------LLQLFNTSD-DRAQ-KRQ----------------------------

-----PIRGSDE--VL-----------------------------FK-------------
VYCIDQTYTTIRVPVSSSVKEVIS--------AVADK-----------LGSG-EG--LII
VKMSSG----------------------GEKVV------LKPHDVSVFTTLSVNGRLFAC
PRDQ---F-DSLA-PLPEQ---------------------------EGP------STGTV
GT--FELMSSKDLAYQMTIYDWELFNCVHELELIY-------HTFGRHNF-KK-TTANLD
LFLRR---------FNEIQFWV-----VTEICLCSQLSKR------VQLLKKYIKIAAH-
--------------CKEYKNLNSFFAIIM-GLSNVAVSRLSLTWE---------------
-------KLPSKFKKIYAE-FESLM--------------------------DPSRNH---
-RAYRL----TVAK-------LDPPII-PFMP----------------------------
LLIKDMTFTHEGNK--TF-TD-----------------------------N-LVNFE-KM
-------RM---IANTVRTVKFCRS--Q-------------------------SFNPD--
-A------------------ALTNKNH-----Q----------------DVRS-----YV
RQLNVIDNQRTL---SHMSHRL
-----------EPRR----------A----------------------------------

>Rapgef4_[Nothobranchius_furzeri]_1007766827
---------------------------------MV-----A------AH---ASSHSPES
-AEWIV---CLDK-----------RPAE-----RSGEDVDIILARLKHVKALEGFHPSLL
QQICLCGF--YECLE--KGITLFRQGDIGTCWYAVFSGSLDVKVSE-TSRYQDA------
------VTICTLGTGTAFGESILDNT-PRHATIV-TREFSELL-----------------
----------------------------RVEQKE-------FRSLWE---KFYHCMAG-L
LSPPYG--VMD--S--SCGS-------DRMPGKE--------------------------
------------------NLS-------NSLFVSVSKHPNK-------------------
-----SFTESP---SKLR-PKCSS----------------QVFS----------------
-------ERIL-------------HA---------------------GKILR-NTILSAA
PH-M----------------IRDRKYH------L-KTY----------------RQC-CV
GT-----------------ELVDWQMQQN-------------------------------
---SCVH--------SRVQAVGMW---QVLLEEGVLN-----------------------
--------------H------------------------VDR-ETSFQDKY-IFYRFLDD
-------ERED----AP-V---
--PS
EEER--MESHEEL------QDTLLFLSQIGPDAL-------------MRMILRKPHSERT
AEELE-----IIYEELLHIKALSHLS-TTVKRELA-GVLIFESHGKAGTVL---------
--FNQGEEG--TSWYIIL
KGSVNVFIYGK-----GV-------------------------------VCTLHEG-DDF
GKLAL-----------VNDAPRA---ASIILREDNCHFLRVDKEDFNR------ILRD--
---V--------------

------------------------EANTVRLKEHDQDVLVLEK-----SLN-SGRNSIHG

SS----AS-HY------------------------------------K-YNIMSGTPEKI
LEHLLEM------------MRLDS-QLT--------------ES------DSALD---D-
FVLMHCV--FI---------------PNSQLCPVLR-----S------------------
--------H-----------Y-------QAQALQ---GSEQEKLDYTLNSKRRVIRLVMK
WAAVHGEHLQQE-NTA--FLE----EFLRA--VSDDA--KVLPALR---DQ-LTELERIV
KNN---AED--FK---------ASQKK---------------------------------
---RKI--------LLQQFSTGD-EKLQ-KRQ----------------------------

-----PIKGNDE--IL-----------------------------FK-------------
VYCCDHTYTTIRVPVAASVREVIS--------AVADK-----------LGSA-ED--LIL
VGLSSA----------------------GEKTI------FKPNDVSVFSTLGINGRLFTC
QTDQ---L-DLLT-PLPEQ---------------------------EGP------ISGSL
AS--FELMSSKDVAYHMTSYDWELFHCVHELELIY-------HTFGRQYV-KK-TTVNLD
LFLRR---------FNEIQFWV-----ITEVCLCSQLNKR------VQLLKKFIKIAAQ-
--------------CKEYKNLNAFFAIIM-GLSNPAVSRLSQTWE---------------
-------KLPSKYKKFYGE-FENLM--------------------------DPTRNH---
-RAYRL----TVSK-------LEPPII-PFMP----------------------------
LMIKDMTFTHEGNK--TL-ID-----------------------------S-LVNFE-KM
-------RM---IAKTVKIVRYCRS--L-------------------------TFSPD--
-S------------------PLAGKHH-----L----------------DVLA-----YV
RQLHIIDNQRTL---TQLSHSL
-----------EPRR----------S----------------------------------

>Rapgef4_[Notothenia_coriiceps]_736302842

--MRVDSCIG
-------DREQ----PG-Y---
--GC

EVKG--GGSQDEM------ESRAVEGC----------------------------PSERA
ADDLE-----IIYEELLHIKALSHLS-TTVKRELA-GVLIFESHAKAGTVL---------
--FSQGEEG--TSWYIIL
KGSVNVVIYGK-----GV-------------------------------VCTLHEG-DDF
GKLAL-----------VNDAPRA---ASIVLREDNCHFLRVDKEDFNR------ILRD--
---V--------------

------------------------EANTVRLKEHGQDVLVLEK-----S---HGRTSSHG
AS----SS-HY------------------------------------K-YKVMSGTPEKI
LEHLLEM------------IRLDS-QFS--------------ES------DAALE---D-
FVLMHCV--FI---------------PNTQLCPVLL-----A------------------
--------H-----------Y-------HAQALQ---GSDQEKLDYTVNNKRRVIRLVMQ
WAAVHGDQLQQE-DVPVAFLE----EFLVA--VSNDA--ESIPALR---DQ-LAELERRL
KQH---TED--AS---------VSQKK---------------------------------
---HKV--------LLRQFSMGD-EKLQ-KRQ----------------------------

-----PIKSIDE--IL-----------------------------LK-------------
VYCCDHTYTTIRAPVAASVREVIG--------AVADK-----------LGSA-ED--LLL
VSLSSA----------------------GEKVI------FKPNDVSVFSTLSVNGRLFVC
RRDQ---L-DSLT-PLPEQ---------------------------DGS------STALL
AG--FELMSSKDVAYHMTSYDWELFHCVHELELIY-------HTFGRQNV-KK-TTVNLD
LFLRR---------FNEIQFWV-----ITEVCLCSQLSKR------VQLLKKFIKIAAH-
--------------CKEYRNLNAFFAIIM-GLSNPAVCRLSQTWE---------------
-------KLPSKFKKFYGE-FENFM--------------------------DPSRNH---
-RAYRL----TVSK-------LEPPII-PFMP----------------------------
LLIKDMTFTHEGNK--TF-ID-----------------------------C-LVNFE-KM
-------RM---IANTVKIVRYCRS--Q-------------------------PFSPD--
-S------------------PLASKSH-----P----------------EVRT-----YV
RHLNVIDNQRTL---TQLSHGL
-----------EPRR----------S----------------------------------

>Rapgef4_[Octopus_bimaculoides]_961078509

----------------------------MVKRELA-SVLVFESHSKKGTVL---------
--FRQGDEG--KSWYIIL
KGSVNVSIYGK-----GT-------------------------------VCQLQEG-DDF
GKLAL-----------VNDAPRS---ATIFLCEDNCHFLRVDKDDFNR------ILRD--
---V--------------

------------------------EANTVRLNEHGQDVLVLEKM-----PT---------
----------------------------------NIQAQDGSIQSHYK-YSVMAGTPEKI
LEHLLETRMEKTDSYDHF-----------------------------------LE---S-
FLLTHVI--FM---------------PTKKLCPALR-----M------------------
--------HYEATAGAKHESHDITVSH-----------------------KCRVIQFVQE
WQSISGDEFLED-NTISAFLK----ELHQA--LTRDCV----------------------
---------EYPE---
---LK---NDLIVIEHMI
QLRASNV----CTDPAKY--------------------------------SVKKRLTPNV
TLARKGSNENLIDDSKFRRG--

-----PIKATDE--TI-----------------------------FR-------------
VYCADHSYCTLRVPMTTTVSHIIA--------QAQEK-----------LSLG-ED--LVL
CEVKSN----------------------GERIL------FRENDLGIVNSLSLNGRLFLT
PREH---L-DAVT-PLPEQ---------------------------DDI------LSGTA
SS--LELVGSQELAYHMTIYDWELFTSVPEYELIY-------QVFGRYKF-AK-MTANLD
LFLRR---------FNEVQYWV-----VTEILLTQNLNKR------VHLLRKFIKLASY-
--------------CKEYQNMSSFFAIVL-GLSNIAVSRLSQTWE---------------
-------KLPGKLKKMFSE-FETIM--------------------------DPSRNH---
-RVYRL----SISK-------MRPPII-PFMP----------------------------
LMMKDMTFTHEGNK--TY-YD-----------------------------G-LVNFE-KM
-------HM---IAQTLRTVRCCRS--M----PLNLDIP---------------------
--------------------SSSVKPP-----F-EVKA-----------YVR--------
-NLCVMDNQRTL---TQLSHKL
-----------EPRR----------T----------------------------------


```
------------------------------------------------------------
---
>Rapgef4_[Odobenus_rosmarus_divergens]_472376522
----------------------------MVAA-------HA------AH--------SSS
SAEWIA---CLDK-----------RPLE-----RSSEDVDIIFTRLKEVKAFEKFHPNLL
HQICLCGY--YENLE--KGITLFRQGDIGTNWYAVLAGSLDVKVSE-TSSHQDA------
------VTICTLGIGTAFGESILDNT-PRHATIV-TRESSELL-----------------
----------------------------RIEQKD-------FKALW---EKYRQYMAG-L
LAPPYG--VMETGS--N-N--------DRIPDKE--------------------------
------------------N-----------------------------------------
-----------------------------------------VPS----------------
-------EKIL-------------RA---------------------GKILR-NAILSRA
PH-M----------------IRDRKYH------L-KTY----------------RQC-CV
GT-----------------ELVDWMLQQT-------------------------------
---SCVH--------SRTQAVGMW---QVLVEDSVLN-----------------------
--------------H------------------------VDQ-EHHFQDKY-LFYRFLDD
-------EHED----AP-L-----------------------------------------
----------------------------------------------------------PT
EEEK--KECDEEL------QDTMLLLSQMGPDAH-------------MRMILRKPPGQRT
VDDLE-----IIYEELIHIKALSHLS-TTVKRELA-GVLIFESHAKGGTVL---------
--------------------------------------------FNQGEEG--TSWYIIL
KGSVNVVIYGK-----GV-------------------------------VCTLHEG-DDF
GKLAL-----------VNDAPRA---ASIVLREDNCHFLRVDKEDFNR------ILRD--
---------------------------------------------V--------------
------------------------------------------------------------
------------------------------------------------------------
------------------------EANTVRLKEHDQDVLVLEK-----VPA-GNRASNQG
NS----QP-QQ------------------------------------K-YTVMSGTPEKI
LEHFLET------------IRLEP-ALN--------------EA-----TDSVLN---D-
FVMMHCV--FM---------------PNTQLCPALV-----A------------------
--------H-----------Y-------HAQASQ---GTEQEKMDYALNNKRRVIRLVLQ
WATVYGDALQED-DVAVAFLE----EFYVS--VSDDA--RMIAALK---DQ-LPELEKIV
KQI---SED--AK---------APQKK---------------------------------
---HKV--------LLQQFNTGD-ERAQ-KRQ----------------------------
------------------------------------------------------------
------------------------------------------------------------
------------------------------------------------------------
------------------------------------------------------------
------------------------------------------------------------
------------------------------------------------------------
-----PIRGSDE--VL-----------------------------FK-------------
VYCMDHTYTTIRVPVAASVKEVIS--------AVADK-----------LGSG-EG--LII
VKMSSG----------------------GEKVV------LKPNDVSVFTTLTINGRLFAC
PREQ---F-DSLT-PLPEQ---------------------------EGP------TVGTV
GT--FELMSSKDLAYQMTIYDWELFNCVHELELIY-------HTFGRHNF-KK-TTANLD
LFLRR---------FNEIQFWV-----VTEICLCSQPSKR------VQLLKKFIKIAAH-
--------------CKEYKNLNSFFAIVM-GLSNVAVSRLALTWE---------------
-------KLPSKFKKFYAE-FESLM--------------------------DPSRNH---
-RAYRL----TVAK-------LEPPLI-PFMP----------------------------
LLIKDMTFTHEGNK--TF-ID-----------------------------N-LVNFE-KM
-------RM---IANTARTVRYCRS--Q-------------------------AFNPD--
-A------------------AQANKNH-----Q----------------DVRS-----YV
RQLNVIDNQRTL-----------------------------------------SQMSHRL
-----------EPRR----------P----------------------------------
```


>Rapgef4_[Opisthocomus_hoazin]_700384684

---MSG-L
LTPPYG--VMETVS--N-N--------DRMPDKD--------------------------
------------------SMS-------SSALCQV-------------------------
----------------SK-NCNK------------------VPS----------------
-------EKIL-------------RA---------------------GKILR-NTILSRA
PH-M----------------IRDRKYH------L-KTY----------------RQC-CV
GT-----------------ELVDWMMQQS-------------------------------
---PCVH--------SRTQAVGMW---QVLLEEGVLN-----------------------
--------------H------------------------VDQ-EHHFQDKY-LFYRFLDD
-------ERED----AS-L---
--PT
EEEK--KECDEEL------QDTMLLLSQIGPDAH-------------MRMILRKPPGQRT
VDDLE-----IIYEELLHIKALSHLS-TTVKRELA-GVLIFESHPKAGTVL---------
--FNQGEEG--TSWYIIL
KGSVNVVIYGK-----GV-------------------------------VCTLHEG-DDF
GKLAL-----------VNDAPRA---ASIVLREDNCHFLRVDKEDFNR------ILRD--
---V--------------

------------------------EANTVRLKEHDQDVLVLEK-----IPA-GNRVSNQV
NS----QP-QH------------------------------------K-YIVMSGTPEKI
LEHFLET------------MRPEA-TLN--------------EA-----TDSVLN---D-
FIMMHCV--FM---------------PNSQLCPALM-----A------------------
--------H-----------Y-------HAQPSQ---GTEQEKMDYALNNKRRVIRLVLQ
WAALYGDLLQED-EAAMAFLE----EFYVS--VSDDT--RMIAALK---EQ-LPELEKIV
KQV---SEE--PK---------ALQKK---------------------------------
---HKV--------LLQLFNTSD-DRAQ-KRQ----------------------------

-----PIRGSDE--VL-----------------------------FK-------------
VYCIDQTYTTIRVPVSSSVKELIS--------AVADK-----------LGSG-EG--LII
VKMSSG----------------------GEKVV------LKPHDVSVFTTLSVNGRLFAC
PRDQ---F-DSLA-PLPEQ---------------------------EGP------SAGTV
GT--FELMSSKDLAHQMTVYDWELFNCVHELELIY-------HTFGRHNF-KK-TTANLD

LFLRR---------FNEIQFWV-----ATEICLCSQLSKR------VQLLKKYIKIAAH-
--------------CKEYKNLNSFFAIIM-GLSNVAVSRLSLTWE---------------
-------KLPSKFKKIYAE-FESSM--------------------------DPSRNH---
-RAYRL----TVAK-------LDPPII-PFMP----------------------------
LLIKDMTFTHEGNK--TF-TD-----------------------------N-LVNFE-KM
-------RM---IANTVRTVKFCRS--Q-------------------------SFNPD--
-A------------------ALTNKNH-----Q----------------DVRS-----YV
RQLNVIDNQRTL---SQMSHRL
-----------EPRR----------A----------------------------------

>Rapgef4_[Orcinus_orca]_465994873

---MAG-L
LAPPYG--VMETGS--N-N--------DRIPDKE--------------------------
------------------N---
---VPS----------------
-------EKIL-------------RA---------------------GKILR-NAILSRA
PH-M----------------IRDRKYH------L-KTY----------------RQC-CV
GT-----------------ELVDWMLQQT-------------------------------
---PCVH--------SRMQAVGMW---QVLVEDGVLN-----------------------
--------------H------------------------VDQ-EHHFQDKY-LFYRFLDD
-------EHED----AP-L---
--PT
EEEK--KECDEEL------QDTMLLLSQMGPDAH-------------MRMILRKPPGQRT
VDDLE-----IIYEELLHIKALSHLS-TTVKRELA-GVLIFESHAKGGTVL---------
--FNQGEEG--TSWYIIL
KGSVNVVIYGK-----GV-------------------------------VCTLHEG-DDF
GKLAL-----------VNDAPRA---ASIVLREDNCHFLRVDKEDFNR------ILRD--
---V--------------

------------------------EANTVRLKEHDQDVLVLEK-----VPA-GNRASNQG
NS----QP-QQ------------------------------------K-YTVMSGTPEKI
LEHFLET------------IRLEP-ALN--------------EA-----TDSVLN---D-
FVMMHCV--FM---------------PNTQLCPALV-----A------------------
--------H-----------Y-------HAQPSQ---GTEQEKMDYALNNKRRVIRLVLQ
WAAMYGDILQED-DVAMAFLE----EFYVS--VSDDA--RMIAALK---EQ-LPELEKIV
KQI---SED--AK---------APQKK---------------------------------
---HKV--------LLQQFNTGD-DRAQ-KRQ----------------------------

-----PIRGSDE--VL-----------------------------FK-------------
VYCMDHTYTTIRVPVAASVKEVLS--------AVADK-----------LGSG-EG--LII
IKMSSG----------------------GEKVV------LKPNDVSVFTTLTINGRLFAC
PREK---F-DSLT-PLPEQ---------------------------EGP------TVGTM
GT--FELMSSKDLAYQMTVYDWDLFSCVHELELIY-------HTFGRHHF-KK-TTANLD
LFLRR---------FNEIQFWV-----VTEICLCSQPSKR------VQLLKKFIKIAAH-
--------------CKEYKNLNSFFAIVM-GLSNVAVSRLALTWE---------------
-------KLPSKFKKFYAE-FESLM--------------------------DPSRNH---
-RAYRL----TVAK-------LDPPLI-PFMP----------------------------
LLIKDMTFTHEGNK--TF-ID-----------------------------N-LVNFE-KM
-------RM---IANTARTVRYCRS--Q-------------------------PFNPD--
-A------------------AQANKNH-----Q----------------DVRS-----YV
RQLNVIDNQRTL---SQMSHRL
-----------EPRR----------P----------------------------------

>Rapgef4_[Oreochromis_niloticus]_908516742

---MAG-L
LAPPYG--VME--S--GCNA-------DRLPDKE--------------------------
------------------NIS-------NNSFV-LSKPLNKK------------------
-----SFIESP---AKPH-PKCFS----------------QVPS----------------
-------EKIL-------------RA---------------------GQILR-NAILCRA
PH-M----------------IRDRKYH------L-KTY----------------RRC-CV
GT-----------------ELVDWQLQQS-------------------------------
---SCVH--------SRVQAVGMW---QVLLEEGVLN-----------------------
--------------H------------------------VDQ-ELSFQDKY-LFYRFLDD
-------EQED----AS-F---
--PT
EDER--RESQEEL------QDTLLLLSQIGPDAH-------------LRMILRKPPSERT
ADDLE-----IIYEELLHIKALSHLS-TTVKRELA-GVLIFESHAKAGTVL---------
--FNQGEEG--TSWYIIL
KGSVNVVIYGK-----GV-------------------------------VCTLHEG-DDF
GKLAL-----------VNDAPRA---ASIVLREDNCHFLRVDKEDFNR------ILRD--
---V--------------

------------------------EANTVRLKEHGQDVLVLEK-----SLS-SSRTSVQG

SS----SS-HY------------------------------------K-YNVMSGTPEKI
LEHLLEM------------MRLDS-QFT--------------ES------DSALD---D-
FVLMHCV--FI---------------PNSQLCPVLM-----S------------------
--------H-----------Y-------HAQASQ---GSEQEKLDYTLNNKRRVIRLVMH
WAAVHGDHLQEE-DVSVVFLE----EFFMA--VSNDA--KAFPALR---DQ-LTELERIV
KRN---AED--AR---------SSQKK---------------------------------
---HKV--------LLRHFSMGE-EKLQ-KRQ----------------------------

-----PIRSNDE--IL-----------------------------FK-------------
VYCCDHTYTTIRVPVAASVREVIG--------AVADK-----------LGSA-ED--LLL
VSLSSA----------------------GEKTI------FKPNDVSVFSTLSINGRLFVC
RRDQ---L-DSLT-PLPEQ---------------------------EGP------STGSL
AS--FELMSSKDVAYHMTSYDWELFHCVHELELIY-------HTFGRQYV-KK-TTVNLD
LFLRR---------FNEIQFWV-----ITEMCLCPQLSKR------VQLLKKFIKIAAH-
--------------CKEYRNLNAFFAIIM-GLSNPAVCRLSQTWE---------------
-------KLPSKFKKFYVE-FENLM--------------------------DPSRNH---
-RAYRL----TVAK-------LEPPII-PFMP----------------------------
LLIKDMTFTHEGNK--TF-ID-----------------------------S-LVNFE-KM
-------RM---IANTVKIVRYCRS--Q-------------------------RFSPE--
-S------------------PLPSKNH-----P----------------DVWT-----YI
RQLNVIDNQRML---TQLSHGL
-----------EPRR----------S----------------------------------

>Rapgef4_[Ornithorhynchus_anatinus]_620978658

---MAG-L
LAPPYG--VMETGS--N-N--------DRMPDKE--------------------------
------------------N---
-------MSGSTLCPASK-NCNKTTLIEPHIPLRPAKTIAQVPS----------------
-------EKIL-------------RS---------------------GKILR-NAILSRA
PH-M----------------IRDRKYH------L-KTY----------------RQC-CV
GT-----------------ELVDWMVQQT-------------------------------
---PCVH--------SRTQAVGMW---QVLLEEGVLN-----------------------
--------------H------------------------VDQ-EHYFQDKY-LFYRFLDD
-------EHED----AP-M---
--PT

```
EEEK--KECDEEL------QDTMLLLSQIGPDAH-------------MRMILRKLPGQRT
GDDLE-----IIYEELLHIKALSHLS-TTVKRELA-GVLIFESHAKGGTVL---------
--------------------------------------------FNQGEEG--TSWYIIL
KGSVNVVIYGK-----GV-------------------------------VCTLHEG-DDF
GKLAL-----------VNDAPRA---ASIVLREDHCHFLRVDKEDFNR------ILRD--
---------------------------------------------V--------------
------------------------------------------------------------
------------------------------------------------------------
------------------------EANTVRLKEHDQDVLVLEK-----VPA-GNRAANQG
NS----QP-QQ------------------------------------K-YTVMSGTPEKI
LEHFLET------------MRLEL-ALN--------------EA-----ADSVVN---D-
FVMMHCV--FM---------------PNSQLCPALM-----T------------------
--------H-----------Y-------HAQPSQ---GSEQEKMDYALNNKRRVIRLVLQ
WTALYGDLLQED-EVALAFLE----EFYVS--VSDDT--RIIASLK---EQ-LPELERTV
KQI---SED--SK---------APQKK---------------------------------
---HKV--------LLQQFNTGE-ERTQ-KRQ----------------------------
------------------------------------------------------------
------------------------------------------------------------
------------------------------------------------------------
------------------------------------------------------------
------------------------------------------------------------
------------------------------------------------------------
-----PVRGSDE--IL-----------------------------FK-------------
VYCIDHTYTTIRVPVSASVKEVIG--------AVADK-----------LGSG-EG--LII
VKMSSG----------------------GEKVV------LKPNDVSVFTTLTVNGRLFAC
PREQ---F-DSLT-PLPEQ---------------------------EGP------TTGTI
GT--FELMSSKDLAYQMTIYDWELFNCVHELELIY-------NTFGRHNF-KK-TTANLD
LFLRR---------FNEIQFWV-----VTEICLCSQLSKR------VQLLKKFTKIAAH-
--------------CKEYKNLNSFFAIVM-GLSNVAVSRLTLTWE---------------
-------KLPSKFKKIYAE-FENLM--------------------------DPSRNH---
-RAYRL----TIAK-------LDPPLI-PFMP----------------------------
LLIKDMTFTHEGNK--TF-ID-----------------------------N-LVNFE-KM
-------RM---IANTARTVRYCRS--Q-------------------------PFNPD--
-A------------------ALANKNH-----Q----------------DVRS-----YV
RQLNVIDNQRTL-----------------------------------------SQMSHRL
-----------EPRR----------A----------------------------------
------------------------------------------------------------
------------------------------------------------------------
------------------------------------------------------------
------------------------------------------------------------
------------------------------------------------------------
------------------------------------------------------------
------------------------------------------------------------
------------------------------------------------------------
------------------------------------------------------------
------------------------------------------------------------
---
>Rapgef4_[Orycteropus_afer_afer]_634820942
----------------------------MVAA-------HA------AH--------SSS
SAEWIA---CLDK-----------RPLE-----RSSEDVDIIFTRLKEVKAFEKFHPNLL
HQICLCGY--YENLE--KGITLFRQGDIGTNWYAVLAGSLDVKVSE-TSSHQDA------
------VTICTLGIGTAFGESILDNT-PRHATIV-TRECSELL-----------------
----------------------------RIEQKD-------FKALW---EKYRQYMAG-L
LAPPYG--VMETGS--N-N--------DRIPDKE--------------------------
```

```
------------------N-----------------------------------------
-----------------------------------------VPS----------------
-------EKIL-------------RA---------------------GKILR-NVILSRA
PH-M----------------IRDRKYH------L-KTY----------------RQC-CV
GT-----------------ELVDWMMQQT-------------------------------
---QCVH--------SRTQAVGMW---QVLLEDGVLN-----------------------
--------------H------------------------VDQ-EHHFQDKY-LFYRFLDD
-------EHED----AP-L-----------------------------------------
----------------------------------------------------------PT
EEEK--KECDEEL------QDTMLLLSQMGPDAH-------------MRMILRKPPGQRT
VDDLE-----IIYEELLHIKALSHLS-TTVKRELA-GVLIFESHAKGGTVL---------
--------------------------------------------FNQGEEG--TSWYIIL
KGSVNVVIYGK-----GV-------------------------------VCTLHEG-DDF
GKLAL-----------VNDAPRA---ASIVLREDNCHFLRVDKEDFNR------ILRD--
---------------------------------------------V--------------
------------------------------------------------------------
------------------------------------------------------------
------------------------EANTVRLKEHDQDVLVLEK-----VPA-GNRASNQG
NS----QP-QQ------------------------------------K-YTVMSGTPEKI
LEHFLET------------IRLEP-ALN--------------EA-----TDSVLN---D-
FVMMHCI--FM---------------PNTQLCSALA-----A------------------
--------H-----------Y-------HAQPSQ---GTEQEKMDYALNNKRRVIRLVLQ
WAAMYGDLLQED-DVAMAFLE----EFYVS--VSDDA--RMIAALK---EQ-LPELEKIV
KQI---LEE--AK---------VPQKK---------------------------------
---HKV--------LLQQFNTGD-ERAQ-KRQ----------------------------
------------------------------------------------------------
------------------------------------------------------------
------------------------------------------------------------
------------------------------------------------------------
------------------------------------------------------------
------------------------------------------------------------
-----PIRGSDE--VL-----------------------------FK-------------
VYCMDHTYTTIRVPVAASVKEVIS--------AVADK-----------LGSG-EG--LII
VKMSSG----------------------GEKVV------LKPNDVSVFTTLTINGRLFAC
PREQ---F-DSLT-PLPEQ---------------------------EGP------TVGTV
GT--FELMSSKDLAYQMTIYDWELFHCVHELELIY-------HTFGRHNF-KK-TTANLD
LFLRR---------FNEIQFWV-----VTEICLCSQLSKR------VQLLKKFIKIAAH-
--------------CKEYKNLNSFLAIVM-GLSNVAVSRLALTWE---------------
-------KLPSKFKKFYAE-FESLM--------------------------DPSRNH---
-RAYRL----TVAK-------LDPPLI-PFMP----------------------------
LLIKDMTFTHEGNK--TF-ID-----------------------------N-LVNFE-KM
-------RM---IANTARTVRYCRS--Q-------------------------PFNPD--
-A------------------TQANKNH-----Q----------------DVRS-----YV
RQLNVIDNQRTL-----------------------------------------SQMSHRL
-----------EPRR----------P----------------------------------
------------------------------------------------------------
------------------------------------------------------------
------------------------------------------------------------
------------------------------------------------------------
------------------------------------------------------------
------------------------------------------------------------
------------------------------------------------------------
------------------------------------------------------------
------------------------------------------------------------
```


>Rapgef4_[Oryctolagus_cuniculus]_1040225784
----------------------------MVAA-------HA------AH--------SSS
SAEWIA---CLDK-----------RPLE-----RSSEDVDIIFTRLKEVKAFEKFHPNLL
HQICLCGY--YENLE--KGITLFRQGDIGTNWYAVLAGSLDVKVSE-TSSHQDA------
------VTICTLGIGTAFGESILDNT-PRHATIV-TRESSELL-----------------
----------------------------RIEQKD-------FKALW---EKYRQYMAG-L
LAPPYG--VMETGS--N-N--------DRIPDKE--------------------------
------------------N---
---VPS----------------
-------EKIL-------------RA---------------------GKILR-NAILSRA
PH-M----------------IRDRKYH------L-KTY----------------RQC-CV
GT-----------------ELVDWMMQQT-------------------------------
---PCAH--------SRTQAVGMW---QVLLEDGVLN-----------------------
--------------H------------------------VDQ-EHHFQDKY-LFYRFLDD
-------EQED----AP-L---
--PT
EEEK--KECDEEL------QDTMLLLSQMGPDAH-------------MRMILRKPPGQRT
VDDLE-----IIYEELLHIKALSHLS-TTVKRELA-GVLIFESHAKGGTVL---------
--FNQGEEG--TSWYIIL
KGSVNVVIYGK-----GV-------------------------------VCTLHEG-DDF
GKLAL-----------VNDAPRA---ASIVLREDNCHFLRVDKEDFNR------ILRD--
---V--------------

------------------------EANTVRLKEHDQDVLVLEK-----VPA-GNRASNQG
NS----QP-QQ------------------------------------K-YTVMSGTPEKI
LEHFLET------------IRLEP-ALN--------------EA-----ADSVLS---D-
FVLMHCV--FM---------------PNTQLCPALV-----A------------------
--------H-----------Y-------HAQPSQ---GTEQEKMDYSLNNKRRVIRLVLQ
WAATYGDLLQED-DVAVAFLE----EFYVS--VSDDA--RVIATLK---EQ-LPELEKIV
KQI---SED--AK---------APQKK---------------------------------
---HKV--------LLQQFNTSD-ERSQ-KRQ----------------------------

-----PIRGSDE--VL-----------------------------FK-------------
VYCMDHTYTTIRVPVAASVKEVIS--------AVADK-----------LGSG-EG--LII
VKMSSG----------------------GEKVV------LKPNDVSVFTTLTINGRLFAC
PREQ---F-DSLT-PLAEQ---------------------------EGP------TVGTV
AT--FELMSSKDLAYQMTIYDWELFNCVHELELIY-------HTFGRHNF-KK-TTANLD
LFLRR---------FNEIQFWV-----ITEICLCSQLSKR------VQLLKKFIKIAAH-
--------------CKEYRNLNSFFAIVM-GLSNVAVSRLALTWE---------------
-------KLPSKFKKFYAE-FESLM--------------------------DPSRNH---
-RAYRL----TVAK-------LEAPLI-PFMP----------------------------
LLIKDMTFTHEGNK--TF-TD-----------------------------N-LVNFE-KM
-------RM---IANTARTVRYYRS--Q-------------------------PFNLD--
-A------------------AQANKNH-----Q----------------DVRS-----YV
RQLNVIDNQRTL---SQMSHRL
-----------EPRR----------P----------------------------------

>Rapgef4_[Oryzias_latipes]_765148738
----------------------------MQPRKMV-----A------LQ---PSPSSSPS
LAEWIC---CLDK-----------RPSE-----RSGEDVDIILTRLREVKAFQRFPPPLL
LQICACAF--YECLE--KGITLFRQGDIGTSWYAVLSGSLDVKVSE-TANHQDA------
------VTICTLGIGTAFGESILDNT-PRHATIV-SRETSELL-----------------
----------------------------RIEQRE-------FKTLWE---KYRQSLAG-L
LAPPYG--AMESGS----NN-------DRLADKD--------------------------
------------------NMN-------S-------------------------------
--------ESG---SKAH-----N----------------KIPS----------------
-------EKLQ-------------RA---------------------GKVLR-NAILSRA
PH-M----------------IRDRKYH------L-KTY----------------RQC-CV
GT-----------------ELVDWLVMQS-------------------------------
---ACVL--------TRSHAVGMW---QVLLEEGVLN-----------------------
--------------H------------------------VDQ-DLGFQDKY-LFYRFLDD
-------EDED----TP-L---
--PS
EEEK--RESEEEL------PETILFLAQIGPDAL-------------LRMILRKSPGQRT
GDDLE-----IIYDELLHIKALAHLS-NTVKRELA-SVVIFESHAKAGTVL---------
--FNQGEEG--TSWYIIL
KGSVNVVIYGK-----GV-------------------------------VCTLHEG-DDF
GKLAL-----------VTDSPRA---ASIVLREDNCHFLRVDKEDFNR------ILRD--
---V--------------

------------------------EANTVRLKEHEQSVLVLEK-----S----PRASTLG
G---------I------------------------------------K-YTVISGTPEKI
LEHFLEA------------MRLDI-HHN--------------EP------DPAVD---D-
FVLMHCV--FM---------------PNSQLCPLLM-----A------------------
--------H-----------Y-------HAASPP---GSEQERLEYALNCKRRVLILALR
WVNAHTYLLQEE-PAAISFLE----ELYGS--VSNDS--RILRGLK---DL-AVDLEKVV
KLH---SEE--AK---------SSKRK---------------------------------
-----T--------LIRQFSNGE-ERLQ-KKQ----------------------------

-----PIKNQDD--IL-----------------------------LK-------------
VYCSDHTYTTIRIAVAATGREVIS--------AVTDK-----------LGTA-DE--LLL
IHLSSA----------------------GEKQI------LKPNDVSVFSTLSINGRLFAC
PRDQ---L-SGLT-PHPDQ---------------------------EGP------SAGSM
ST--FELMSSKDLAYQMTMFDWELFSCVHEHELLY-------HTFGRQSF-RR-TTANLD

LFLRR---------FNQVMLWV-----VTEVCLCNQLSKR------VQLLKKFIKIAAH-
--------------CREFKNLNSFFAIIM-GMSNPAVSRLSQTWE---------------
-------KLPTKFKKFYAE-FESMM--------------------------DPSRNH---
-RTYRI----TVTK-------LEPPII-PFMP----------------------------
LLLKDMTFTHEGNK--TF-ID-----------------------------N-MVNFE-KM
-------RI---IANTIRQVRHCRS--Q-------------------------PFNPD--
-I------------------CQPNKNQ-----A----------------EVRG-----YV
RKLCVIDNQRAL---TQLSYRL
-----------EPRR----------T----------------------------------

>Rapgef4_[Ovis_aries_musimon]_803230360
----------------------------MVAA-------HA------AH--------SSS
SAEWIA---CLDK-----------RPLE-----RSSEDVDIIFTRLKEVKAFEKFHPNLL
HQICLCGY--YENLE--KGITLFRQGDIGTNWYAVLAGSLDVKVSE-TSSHQDA------
------VTICTLGIGTAFGESILDNT-PRHATIV-TRESSELL-----------------
----------------------------RIEQKD-------FKALW---EKYRQYMAG-L
LAPPYG--VMETGS--N-N--------DRIPDKE--------------------------
------------------N----------------------T------------------
-----PLIEPHVPLRPAN-TITK------------------VPS----------------
-------EKIL-------------RA---------------------GKILR-NAILSRA
PH-M----------------IRDRKYH------L-KTY----------------RQC-CV
GT-----------------ELVDWMLQQT-------------------------------
---PCVH--------SRTQAVGMW---QVLVEDSVLN-----------------------
--------------H------------------------VDQ-EHHFQDKY-LFYRFLDD
-------EHED----AP-L---
--PT
EEEK--KECDEEL------QDTMLLLSQMGPDAH-------------MRMVLRKPPGQRT
MDDLE-----IIYEELLHIKALSHLS-TTVKRELA-AVLIFESHAKGGTVL---------
--FNQGEEG--TSWYIIL
KGSVNVVIYGK-----GV-------------------------------VCTLHEG-DDF
GKLAL-----------VNDAPRA---ASIVLREDNCHFLRVDKEDFNR------ILRD--
---V--------------

------------------------EANTVRLKEHDQDVLVLEK-----VPA-GNRASNQG
NS----QP-QQ------------------------------------K-YTVMSGTPEKI
LEHFLET------------IRLEP-ALN--------------EA-----TDSVLN---D-
FVMMHCV--FM---------------PNTQLCPALV-----A------------------
--------H-----------Y-------HAQPSQ---GTEQEKMDYALNNKRRVIRLVLQ
WAAMYGDILQED-DVAMAFLE----EFYVS--VSDDA--RMLAALK---EQ-LPELEKIV
KQI---SED--AK---------APQKK---------------------------------
---HKV--------LLQQFNTGD-ERAQ-KRQ----------------------------

-----PIRGSDE--VL-----------------------------FK-------------
VYCLDHTYTTIRVPVAASVKEVLS--------AVADK-----------LGSG-EG--LII
VKMSSG----------------------GEKVV------LKPNDVSVFTTLTINGRLFAC
PREQ---F-DSLT-PLPEQ---------------------------EGP------TVGTM
GT--FELMSSKDLAYQMTIYDWELFNCVHELELIY-------HTFGRHHF-KK-TTANLD
LFLRR---------FNEIQFWV-----VTEICLCSQPSKR------VQLLKKFIKIAAH-
--------------CKEYKNLNSFFAIVM-GLSNVAVSRLALTWE---------------
-------KLPSKFKKFYAE-FESLM--------------------------DPSRNH---
-RAYRL----TVAK-------LDPPLI-PFMP----------------------------
LLIKDMTFTHEGNK--TF-ID-----------------------------N-LVNFE-KM
-------RM---IANTARTVRYCRS--Q-------------------------PFNPD--
-A------------------AQANKNH-----Q----------------DVRS-----YV
RQLNVIDNQRTL---SQMSHRL
-----------EPRR----------P----------------------------------

>Rapgef4_[Ovis_aries]_426220871

---MAG-L
LAPPYG--VMETGS--N-N--------DRIPDKE--------------------------
------------------N---
---VPS----------------
-------EKIL-------------RA---------------------GKILR-NAILSRA
PH-M----------------IRDRKYH------L-KTY----------------RQC-CV
GT-----------------ELVDWMLQQT-------------------------------
---PCVH--------SRTQAVGMW---QVLVEDSVLN-----------------------
--------------H------------------------VDQ-EHHFQDKY-LFYRFLDD
-------EHED----AP-L---
--PT
EEEK--KECDEEL------QDTMLLLSQMGPDAH-------------MRMVLRKPPGQRT
MDDLE-----IIYEELLHIKALSHLS-TTVKRELA-AVLIFESHAKGGTVL---------
--FNQGEEG--TSWYIIL
KGSVNVVIYGK-----GV-------------------------------VCTLHEG-DDF
GKLAL-----------VNDAPRA---ASIVLREDNCHFLRVDKEDFNR------ILRD--
---V--------------

------------------------EANTVRLKEHDQDVLVLEK-----VPA-GNRASNQG

NS----QP-QQ------------------------------------K-YTVMSGTPEKI
LEHFLET------------IRLEP-ALN--------------EA-----TDSVLN---D-
FVMMHCV--FM---------------PNTQLCPALV-----A------------------
--------H-----------Y-------HAQPSQ---GTEQEKMDYALNNKRRVIRLVLQ
WAAMYGDILQED-DVAMAFLE----EFYVS--VSDDA--RMLAALK---EQ-LPELEKIV
KQI---SED--AK---------APQKK---------------------------------
---HKV--------LLQQFNTGD-ERAQ-KRQ----------------------------

-----PIRGSDE--VL-----------------------------FK-------------
VYCLDHTYTTIRVPVAASVKEVLS--------AVADK-----------LGSG-EG--LII
VKMSSG----------------------GEKVV------LKPNDVSVFTTLTINGRLFAC
PREQ---F-DSLT-PLPEQ---------------------------EGP------TVGTM
GT--FELMSSKDLAYQMTIYDWELFNCVHELELIY-------HTFGRHHF-KK-TTANLD
LFLRR---------FNEIQFWV-----VTEICLCSQPSKR------VQLLKKFIKIAAH-
--------------CKEYKNLNSFFAIVM-GLSNVAVSRLALTWE---------------
-------KLPSKFKKFYAE-FESLM--------------------------DPSRNH---
-RAYRL----TVAK-------LDPPLI-PFMP----------------------------
LLIKDMTFTHEGNK--TF-ID-----------------------------N-LVNFE-KM
-------RM---IANTARTVRYCRS--Q-------------------------PFNPD--
-A------------------AQANKNH-----Q----------------DVRS-----YV
RQLNVIDNQRTL---SQMSHRL
-----------EPRR----------P----------------------------------

>Rapgef4_[Panthera_tigris_altaica]_987393874
----------------------------M-------------------------------

---R-PSLPGRAVN-C
SASSRR--TSRLYG--R-N--------IDSTWQD--------------------------
------------------C----------------------W------------------
-----LLLM--VLWKRAL-TMT-------------------TPLIEPHVPLRP-------
---ANTITKVPSEKIL--------RA---------------------GKILR-NAILSRA
PH-M----------------IRDRKYH------L-KTY----------------RQC-CV
GT-----------------ELVDWMLQQT-------------------------------
---PCVH--------SRTQAVGMW---QVLVEDGVLN-----------------------
--------------H------------------------VDQ-EHHFQDKY-LFYRFLDD
-------EHED----AP-L---
--PT

EEEK--KECDEEL------QDTMLLLSQMGPDAH------------MRMTLRKPPGQRT
VDDLE-----IIYEELIHIKALSHLS-TTVKRELA-GVLIFESHAKGGTVL---------
---FNQGEEG--TSWYIIL
KGSVNVVIYGK-----GV-------------------------------VCTLHEG-DDF
GKLAL-----------VNDAPRA---ASIVLREDNCHFLRVDKEDFNR------ILRD--
--V--------------

-----------------------EANTVRLKEHDQDVLVLEK-----VPA-GNRASNQG
NS----QP-QQ-----------------------------------K-YTVMSGTPEKI
LEHFLET------------IRLEP-ALN--------------EA-----TDSILN---D-
FVMMHCV--FM---------------PNTQLCPALV-----A-----------------
--------H-----------Y-------HAQPSQ---GTEQEKTDYTLNNKRRVIRLVLQ
WAAVHGDVLQED-DVAVAFLE----EFYVS--VSDDA--RMIAALK---EQ-LPELEKIV
KQI---SED--AK---------APQKK---------------------------------
---HKV--------LLQQFNTGD-ERAQ-KRQ----------------------------

-----PVRGSDE--VL----------------------------FK-------------
VYCMDHTYTTIRVPVAASVKEVIS--------AVADK-----------LGSG-EG--LII
VKMSSG----------------------GEKVV------LKPNDVSVFTTLTINGRLFAC
PREQ---F-DSLT-PLPEQ---------------------------EGP------TVGTM
GT--FELMSSKDLAYQMTIYDWELFNCVHELELIY-------HTFGRHNF-KK-TTANLD
LFLRR---------FNEIQFWV-----VTEICLCSQPSKR------VQLLKKFIKIAAH-
--------------CKEYKNLNSFFAIVM-GLSNVAVSRLALTWE---------------
-------KLPSKFKKFYAE-FESLM-------------------------DPSRNH---
-RAYRL----TVAK-------LEPPLI-PFMP----------------------------
LLIKDMTFTHEGNK--TF-ID------------------------------N-LVNFE-KM
-------RM---IANTARTVRYCRS--Q-------------------------PFNPD--
-A------------------AQANKNH-----Q----------------DVRS-----YV
RQLNVIDNQRTL---SQMSHRL
-----------EPRR----------P----------------------------------

>Rapgef4_[Papio_anubis]_402888638
----------------------------MVAA-------HA------AH--------SSS
SAEWIA---CLDK-----------RPLE-----RSSEDVDIIFTRLKEVKAFEKFHPNLL
HQICLCGY--YENLE--KGITLFRQGDIGTNWYAVLAGSLDVKVSE-TSSHQDA------
------VTICTLGIGTAFGESILDNT-PRHATIV-TRESSELL-----------------
----------------------------RIEQKD-------FKALW---EKYRQYMAG-L
LAPPYG--VMETGS--N-N--------DRIPDKE-------------------------

```
------------------N----------------------T------------------
-----PLIEPHVPLRPAN-TITK------------------VPS----------------
-------EKIL-------------RA---------------------GKILR-NAILSRA
PH-M----------------IRDRKYH------L-KTY----------------RQC-CV
GT-----------------ELVDWMMQQT-------------------------------
---PCVH--------SRTQAVGMW---QVLLEDGVLN-----------------------
--------------H------------------------VDQ-EHHFQDKY-LFYRFLDD
-------EHED----AP-L-----------------------------------------
----------------------------------------------------------PT
EEEK--KECDEEL------QDTMLLLSQMGPDAH-------------MRMILRKPPGQRT
VDDLE-----IIYEELLHIKALSHLS-TTVKRELA-GVLIFESHAKGGTVL---------
--------------------------------------------FNQGEEG--TSWYIIL
KGSVNVVIYGK-----GV-------------------------------VCTLHEG-DDF
GKLAL-----------VNDAPRA---ASIVLREDNCHFLRVDKEDFNR------ILRD--
---------------------------------------------V--------------
------------------------------------------------------------
------------------------------------------------------------
------------------------EANTVRLKEHDQDVLVLEK-----VPA-GNRASNQG
NS----QP-QQ------------------------------------K-YTVMSGTPEKI
LEHFLET------------IRLEA-TLN--------------EA-----TDSVLN---D-
FIMMHCV--FM---------------PNTQLCPALV-----A------------------
--------H-----------Y-------HAQPSQ---GTEQEKMDYALNNKRRVIRLVLQ
WAAMYGDLLQED-DVSMAFLE----EFYVS--VSDDA--RMIAALK---EQ-LPELEKIV
KQI---SED--AK---------APQKK---------------------------------
---HKV--------LLQQFNTGD-ERAQ-KRQ----------------------------
------------------------------------------------------------
------------------------------------------------------------
------------------------------------------------------------
------------------------------------------------------------
------------------------------------------------------------
------------------------------------------------------------
-----PIRGSDE--VL-----------------------------FK-------------
VYCMDHTYTTIRVPVAASVKEVIS--------AVADK-----------LGSG-EG--LII
VKMSSG----------------------GEKVV------LKPNDVSVFTTLTINGRLFAC
PREQ---F-DSLT-PLPEQ---------------------------EGP------TIGTV
GT--FELMSSKDLAYQMTIYDWELFNCVHELELIY-------HTFGRHNF-KK-TTANLD
LFLRR---------FNEIQFWV-----VTEICLCSQLSKR------VQLLKKFIKIAAH-
--------------CKEYKNLNSFFAIVM-GLSNVAVSRLALTWE---------------
-------KLPSKFKKFYAE-FESLM--------------------------DPSRNH---
-RAYRL----TVAK-------LEPPLI-PFMP----------------------------
LLIKDMTFTHEGNK--TF-ID-----------------------------N-LVNFE-KM
-------RM---IANTARTVRYYRS--Q-------------------------PFNPD--
-A------------------AQANKNH-----Q----------------DVRS-----YV
RQLNVIDNQRTL-----------------------------------------SQMSHRL
-----------EPRR----------P----------------------------------
------------------------------------------------------------
------------------------------------------------------------
------------------------------------------------------------
------------------------------------------------------------
------------------------------------------------------------
------------------------------------------------------------
------------------------------------------------------------
------------------------------------------------------------
------------------------------------------------------------
```

```
------------------------------------------------------------
---
>Rapgef4_[Parasteatoda_tepidariorum]_1009531414
-----------------------------MVTE---------------------------
---WAHV---MDK-----------RPAD-----RTEEELEILYNRLRSIEAFEKYHPTLL
QQMCCFGY--YEDLD--KGVTLFRQGDKGTNWYAVLAGSLDVQVLQTS-PEQDT------
------VTLCTLGIGTAFGESILDNS-PHSATVV-TNEHCELLRIEQRDFKTIWERN---
--KH--LMEDIV------------------------SPISTLRSLT--------------
GLPRQDECKASNGNIRSITPLGKK-ESLTPDGPNP-------------------------
------------------------------------------------------------
---------------------------------------ALP------------IT----
---QEPSSTLS-------------RA---------------------GWVLR-TLILSQS
PH-L----------------IRDRKYH------V-RTF----------------RKC-LV
GS-----------------EMVDWLMQQG-------------------------------
GSYVRIH--------SRSQAVGMW---QALMEEGVIS-----------------------
--------------H------------------------VTN-EHQFKDKY-LFYRFRED
-------QIDS----GS-F-----------------------------------------
----------------------------------------------------------PS
ADDR--QQAELEL------AETLAILLQLAPDAI-------------FRMILRKPSCERT
EEDLE-----IIYEELLHIKALSHLS-NSVKRELA-GVLVFESHPKAGTVL---------
--------------------------------------------FNQGDEG--KSWFIIL
KGAVNVVIYGK-----GV-------------------------------VCSLHEG-DDF
GKLAL-----------VNDAPRA---ATIVTRENNCHFLRVDKDDFNN------IIRD--
---------------------------------------------V--------------
------------------------------------------------------------
------------------------------------------------------------
------------------------EANTVRLKEHGQDVLLLQK-----IPT-----DSRA
PD--GTHS-HY------------------------------------K-YMVMAGTPQKM
LEHLLETRIDQRNED------AS---------------------------DTFLE---D-
FLLTHVI--FM---------------PSHQLCPELM-----R------------------
--------HYRIDATN-----------------------CKQEKEFIIANKKRVLQFVQI
WATVIREAFFED-ASIASFLQ----ELHDS--VVEDC--KKF--T--LHEE-LSIISRIL
ETK-HKYDKEVIG---------VT-YR---------------------------------
---WKVGPYGQIRLLSASESQDE-ERTE-FR-----------------------------
------------------------------------------------------------
------------------------------R-----------------------------
------------------------------------------------------------
------------------------------------------------------------
------------------------------------------------------------
------------------------------------------------------------
-----CIKYSDE--VV------------------------------FR-------------
VYCADHTYTTIKTGVGATAEAIKR--------SAAEK-----------LGFK-DE--LLL
VEVKST----------------------SERVI------FKDSEVSVPTGLSVNGRIFLT
PSDQ---V-DLLT-PLPEQ---------------------------EGP------QDGTG
AI--LETLNSLDIAYGMTIYEWDLFSCVHEYELIY-------QVFGRHQF-RK-IMSNLD
VFLRR---------FNEIQHWV-----VTEMVLCNNLSRR------VALLRKFIKIAAH-
--------------CREHQNMNSFFAIVM-GLSNVAVSRMTQTWE---------------
-------KLPSKLKRTFSE-FEALI--------------------------DPSRNH---
-RRYRL----TVSK-------MVPPII-PFMP----------------------------
LLLKDMTFSHEGNK--TY-ME-----------------------------G-LLNFE-KM
-------HM---IAQSLRTIRHCRS--Q-------------------------PLELDP-
-P------------------PG-NKPQ-----Q----------------DVRN-----YI
RNLRIIDNQKRL-----------------------------------------TQLSDVL
-----------EPRR----------TA---------------------------------
```


>Rapgef4_[Parus_major]_998705924
----------------------------MVAA-------HA------SH--------SSS
SGEWIA---CLDK-----------RPLE-----RSSEDVDIIFTRLKEVKAFEKFHPNLL
QQICLCGY--YENLE--KGITLFRQGDIGTNWYAVLTGSLDVKVSD-TSNHQDA------
------VTICTLGIGTAFGESILDNT-PRHATIV-TREHSELL-----------------
----------------------------RIEQKD-------FKALW---EKYRQYMSG-L
LTPPYG--VMETGS--N-N-------DRMPDKD--------------------------
------------------SMS-------SSALCQVSKNCNKT------------------
-----PLIEPYIPHHPTK-TITQ------------------VPS----------------
-------EKIL-------------RA---------------------GKILR-NTILSRA
PH-M----------------IRDRKYH------L-KTY----------------RQC-CV
GT-----------------ELVDWMMQQS-------------------------------
---PCVH--------LRTQAVGMW---QVLLEEGVLN-----------------------
--------------H------------------------VDQ-EHHFQDKY-LFYRFLDD
-------ERED----AP-L---
--PT
EEEK--KECDEEL------QDTMLLLSQIGPDAH-------------MRMILRKPPGQRT
VDDLE-----FIYEELLHIKALSHLS-TTVKRELA-GVLIFESHPKAGTVL---------
--FNQGEEG--TSWYIIL
KGSVNVVIYGK-----GV-------------------------------VCTLHEG-DDF
GKLAL-----------VNDAPRA---ASIVLREDNCHFLRVDKEDFNR------ILRD--
---V--------------

------------------------EANTVRLKEHDQDVLVLEK-----IPA-GNRISNQG
NP----QP-QH------------------------------------K-YIVMSGTPEKI
LEHFLET------------MRLEA-TLN--------------EA-----TDSVLN---D-
FIMMHCV--FM---------------PNSQLCPALM-----A------------------
--------H-----------Y-------HAQPSQ---GTEQEKMDYALNNKRRVIRLVLQ
WAALYGDLLQED-EAAMAFLE----EFYVS--VSDDT--RMIAALK---EQ-LPELEKIV
KQI---SEE--PK---------APQKK---------------------------------
---HKV--------LLQLFNTSD-DRAQ-KRQ----------------------------

-----PIRGSDE--VL-----------------------------FK-------------
VYCIDQTYTTIRVPVSSSVKEVIS--------AVADK-----------LGSG-EG--LII
VKMSSG----------------------GEKVV------LKPHDVSVFTTLSVNGRLFAC
PRDQ---F-DSLA-PLPEQ---------------------------EGP------STGTI
GT--FELMSSKDLAHQMTIYDWELFNCVHELELIY-------HTFGRHNF-KK-TTANLD

LFLRR---------FNEIQFWV-----VTEICLCSQLSKR------VQLLKKYIKIAAH-
--------------CKEYKNLNSFFAIIM-GLSNVAVSRLSLTWE---------------
-------KLPSKFKKIYAE-FESLM--------------------------DPSRNH---
-RAYRL----TVAK-------LDPPII-PFMP----------------------------
LLIKDMTFTHEGNK--TF-TD-----------------------------N-LVNFE-KM
-------RM---IANTVRTVKFCRS--Q-------------------------SFNPD--
-A------------------ALTNKNH-----Q----------------DVRS-----YV
RQLNVIDNQRTL---SQMSHRL
-----------EPRR----------A----------------------------------

>Rapgef4_[Pelecanus_crispus]_694663624
--MTSIVKTVLPDKTYYA
LKRQISNINKELDKAFSGETKQKGLPLERSTE-----DVDIIFTRLKEVKAFEKFHPNLL
QQICLCGY--YENLEK--GITLFRQGDIGTNWYAVLTGSLDVKVSD--------TSNHQD
AVT-----ICTLGIGTAFGESILDNT-PRHATIV-TREYSELLRIEQKDFKALWEKY---
--RQ--YMSGLLTPP----YGVMETGS----NNDRMPDKEN-------------------

-----------------TPLI-------------------EP------------------
-----HIPHR--P---TK-TITQV-------------------P--------S-------
-------EKIL-------------RA---------------------GKILR-NTILSRA
PH-M----------------IRDRKYH------L-KTY----------------RQC-CV
GT-----------------ELVDWMMQQS------------------------------P
----CVH--------SRTQAVGMW---QVLLEEGVLN-----------------------
--------------H------------------------VDQ-EHHFQDK-YLFYRFLDD
-------ECEDAPLPTEEE---
------------------KKEC--------------------------------------
---------DEEL------QDTMLLLSQIGPDAH-------------MRMILRKPPGQRT
VDDLE-----IIYEELLHIKALSHLS-TTVKRELA-GVLIFESHPKAGTVL---------
--FNQGEEG--TSWYIIL
KGSVNVVIYGK-----GV-------------------------------VCTLHEG-DDF
GKLAL-----------VNDAPRA---ASIVLREDNCHFLRVDKEDFNR------ILRD--
---V--------------

------------------------EANTVRLKEHDQDVLVLEK-------I------PAG
NR----VSNQG------------------NSQPQH------------K-YIVMSGTPEKI
LEHFLET------------MRPEATLNEAT------------------------------

>Rapgef4_[Peromyscus_maniculatus_bairdii]_1008739307
----------------------------M-------------------------------

--HH---LALTP------
LIEPLA--PLRPAN--T-I--------TK-------------------------------

---VPS----------------
-------EKIL-------------RA---------------------GKILR-IAILSRA
PH-M----------------IRDRKYH------L-KTY----------------RQC-CV
GT-----------------ELVDWMIQQT-------------------------------
---SCVH--------SRTQAVGMW---QVLLEDGVLN-----------------------
--------------H------------------------VDQ-ERHFQDKY-LFYRFLDD
-------EHED----GP-L---
--PT
EEEK--KECDEEL------QDTMLLLSQMGPDAH-------------MRMILRKPPGQRT
VDDLE-----IIYDELLHIKALSHLS-TTVKRELA-GVLIFESHAKGGTVL---------
--FNQGEEG--TSWYIIL
KGSVNVVIYGK-----GV-------------------------------VCTLHEG-DDF
GKLAL-----------VNDAPRA---ASIVLREDNCHFLRVDKEDFNR------ILRD--
---V--------------

------------------------EANTVRLKEHDQDVLVLEK-----VPA-GSRASNQG

NS----QP-QQ------------------------------------K-YTVMSGTPEKI
LEHFLET------------IRLEP-SLN--------------EA-----TDSLLN---D-
FVMMHCV--FM---------------PNTQLCPALV-----A------------------
--------H-----------Y-------HAQPSQ---GTEQERLDYALNNKRRVIRLVLQ
WAAMYGDLLQED-DVAMAFLE----EFYVS--VSDDA--RMMAAFK---EQ-LPELEKIV
KQI---SED--AK---------APQKK---------------------------------
---HKV--------LLQQFNTGD-ERAQ-KRQ----------------------------

-----PIRGSDE--VL-----------------------------FK-------------
VYCVDHTYTTIRVPVAASVKEVIS--------AVADK-----------LSSG-EG--LLI
VKMNSG----------------------GEKVV------LKPNDVSVFTTLTINGRLFAC
PREQ---F-DSLT-PLPEQ---------------------------EGP------TTGTV
GT--FELMSSKDLAYQMTTYDWELFNCVHELELIY-------HTFGRHNF-KK-TTANLD
LFLRR---------FNEIQFWV-----VTEICLCSQLSKR------VQLLKKFIKIAAH-
--------------CKEYKNLNSFFAIVM-GLSNVAVSRLALTWE---------------
-------KLPSKFKKFYAE-FESLM--------------------------DPSRNH---
-RAYRL----TAAK-------LDPPLI-PFMP----------------------------
LLIKDMTFTHEGNK--TF-ID-----------------------------N-LVNFE-KM
-------RM---IANTARTVRYYRS--Q-------------------------PFNPD--
-A------------------AQANKNH-----Q----------------DVRS-----YV
RQLNVIDNQRTL---SQMSHRL
-----------EPRR----------P----------------------------------

>Rapgef4_[Phaethon_lepturus]_723122997

-------------------------PLE-----RSSEDVDIIFTRLKEVK----------

--------------------AFEKFH-PNLLQQIC--LCGYYENLEKGITL---------
--FRQGDIG--TNWYAVL
TGSLDVKVSDTSNHQDAVT------------------------------ICTLGIG-TAF
GE-SI-----------LDNTPRH---ATIVTRE-HSELLRIEQKDFKA------LWE---

>Rapgef4_[Phalacrocorax_carbo]_679181721

------------------------RPLE-----RSSEDVDIIFTRLKEVKAFEKFHPNLL
QQICLCGY--YENLE--KGITLFRQGDIGTNWYAVLTGSLDVKVSD-TSNHQDA------
------VTICTLGIGTAFGESILDNT-PRHATIV-TREFSELL-----------------
----------------------------RIEQKD-------FKALW---EKYRQYMSG-L
LTPPYG--VMETSS--N-N--------DKVTDRR--------------------------

------------------N---------------------KT------------------
-----PLIEPHIPHRPTK-TITQ------------------VPS----------------
-------EKIL-------------RA---------------------GKILR-NTILSRA
PH-M----------------IRDRKYH------L-KTY----------------RQC-CV
GT-----------------ELVDWMMQQS-------------------------------
---PCVH--------SRTQAVGMW---QVLLEEGVLN-----------------------
--------------H------------------------VDQ-EHHFQDKY-LFYRFLDD
-------ERED----AP-L---
--PT
EEEK--KECDEEL------QDTMLLLSQIGPDAH-------------MRMILRKPPGQRT
VDDLE-----IIYEELLHIKALSHLS-TTVKRELA-GVLIFESHPKAGTVL---------
--FNQGEEG--TSWYIIL
KGSVNVVIYGK-----GV-------------------------------VCTLHEG-DDF
GKLAL-----------VNDAPRA---ASIVLREDNCHFLRVDKEDFNR------ILRD--
---V--------------

------------------------EANTVRLKEHDQDVLVLEK-----IPA-GNRVSNQG
NS----QP-QH------------------------------------K-YIVMSGTPEKI
LEHFLET------------MRPEA-TLN--------------EG-----TDSVLS---D-
FIMMHCV--FM---------------PNSQLCPALM-----A------------------
--------QYPFLREKHRGTY-------HAQPSQ---GTEQEKMDYALNNKRRVIRLVLQ
WAALYGDLLQED-EAAMAFLE----EFYVS--VSDDT--RMIAALK---EQ-LPELEKIV
KQV---SEE--PK---------APQKK---------------------------------
---HKV--------LLQLFNTSD-DRSQ-KRQ----------------------------

-----PIRGSDE--VL-----------------------------FK-------------
VYCIDQTYTTIRVPVSSSVKEVIS--------AVADK-----------LGSG-EG--LII
VKMSSG----------------------GEKVV------LKPHDVSVFTTLSVNGRLFAC
PRDQ---F-DSLA-PLPEQ---------------------------EGP------STGTV
GT--FELMSSKDLAHQMTIYDWELFNCVHELELIY-------HTFGRHNF-KK-TTANLD
LFLRR---------FNEIQFWV-----VTEICLCSQLSKR------VQLLKKYIKIAAH-
--------------CKEYKNLNSFFAIIM-GLSNVAVSRLSLTWE---------------
-------KLPSKFKKIYAE-FESLM--------------------------DPSRNH---
-RAYRL----TVAK-------LDPPII-PFMP----------------------------
LLIKDMTFTHEGNK--TF-TD-----------------------------N-LVNFE-KM


```
------------------------------------------------------------
---
>Rapgef4_[Physeter_catodon]_593753582
----------------------------M-------------------------------
------------------------------------------------------------
------------------------------------------------------------
------------------------------------------------------------
--------------------------------------------PY---QKYRQYMAG-L
LAPPYG--VMETGS--N-N--------DRIPDKE--------------------------
------------------N----------------------T------------------
-----PLIEPHVPLRPAN-TITK------------------VPS----------------
-------EKIL-------------RA---------------------GKILR-NAILSRA
PH-M----------------IRDRKYH------L-KTY----------------RQC-CV
GT-----------------ELVDWMLQQT-------------------------------
---PCVH--------SRIQAVGMW---QVLVEDSVLN-----------------------
--------------H------------------------VDQ-EHHFQDKY-LFYRFLDD
-------EHED----AP-L-----------------------------------------
----------------------------------------------------------PT
EEEK--KECDEEL------QDTMLLLSQMGPDAH-------------MRMILRKPPGQRT
VDDLE-----IIYEELLHIKALSHLS-TTVKRELA-GVLIFESHAKGGTVL---------
--------------------------------------------FNQGEEG--TSWYIIL
KGSVNVVIYGK-----GV-------------------------------VCTLHEG-DDF
GKLAL-----------VNDAPRA---ASIVLREDNCHFLRVDKEDFNR------ILRD--
---------------------------------------------V--------------
------------------------------------------------------------
------------------------------------------------------------
------------------------EANTVRLKEHDQDVLVLEK-----VPA-GNRASNQG
NS----QP-QQ------------------------------------K-YTVMSGTPEKI
LEHFLET------------IRLEP-ALN--------------EA-----TDSILN---D-
FVMMHCV--FM---------------PNTQLCPALV-----A------------------
--------H-----------Y-------HAQPSQ---GTEQEKMDYALNNKRRVIRLVLQ
WAAMYGDVLQED-DVALAFLE----EFYVS--VSDDA--RMIAALK---EQ-LPELEKIV
KQI---SED--AK---------APQKK---------------------------------
---HKV--------LLQQFNTGD-ERAQ-KRQ----------------------------
------------------------------------------------------------
------------------------------------------------------------
------------------------------------------------------------
------------------------------------------------------------
------------------------------------------------------------
------------------------------------------------------------
-----PIRGSDE--VL-----------------------------FK-------------
VYCMDHTYTTIRVPVAASVKEVLS--------AVADK-----------LGSG-EG--LII
IKMSSG----------------------GEKVV------LKPNDVSVFTTLTINGRLFAC
PREK---F-DSLT-PLPEQ---------------------------EGP------TVGTM
GT--FELMSSKDLAYQMTVYDWDLFSCVHELELIY-------HTFGRHHF-KK-TTANLD
LFLRR---------FNEIQFWV-----VTEICLCSQPSKR------VQLLKKFIKIAAH-
--------------CKEYKNLNSFFAIVM-GLSNVAVSRLALTWE---------------
-------KLPSKFKKFYAE-FESLM-------------------------DPSRNH---
-RAYRL----TVAK-------LDPPLI-PFMP----------------------------
LLIKDMTFTHEGNK--TF-ID-----------------------------N-LVNFE-KM
-------RM---IANTARTVRYCRS--Q-------------------------PFNPD--
-A------------------AQANKNH-----Q----------------DVRS-----YV
RQLNVIDNQRTL-----------------------------------------SQMSHRL
-----------EPRR----------P----------------------------------
```


>Rapgef4_[Picoides_pubescens]_678205164

--VF---QKYRQYMSA-L
LTPPYG--VMETGS--N-N--------DSNLYFTLLLNLSFFF-----------------
-----------------FQ----------------------T------------------
-----PLIEPHIPHRPTK-IISQ------------------VPS----------------
-------EKIL-------------RA---------------------GKILR-NTILSRA
PH-M----------------IRDRKYH------L-KTY----------------RQC-CV
GT-----------------ELVDWMIQQS-------------------------------
---PCVH--------SRTQAVGMW---QVLLEEGVLN-----------------------
--------------H------------------------VDQ-EHHFQDKY-LFYRFLDD
-------ERED----AP-L---
--PT
EEEK--KECDEEL------QDTMLLLSQIGPDAH-------------MRMILRKPPGQRT
VDDLE-----FIYEELLHIKALSHLS-TTVKRELA-GVLIFESHPKAGTVL---------
--FNQGEEG--TSWYIIL
KGSVNVVIYGK-----GV-------------------------------VCTLHEG-DDF
GKLAL-----------VNDAPRA---ASIVLREDNCHFLRVDKEDFNR------ILRD--
---V--------------

------------------------EANTVRLKEHDQDVLVLEK-----IPA-GNRVSNQG
NS----QP-QH------------------------------------K-YIVMSGTPEKI
LEHFLET------------MRLEA-TLS--------------EA-----TDSVLN---D-
FIMMHCV--FM---------------PNSQLCPALM-----A------------------
--------Q-----------YPFHHGTYHAQPSQ---GTEQEKMDYALNNKRRVIRLVLQ
WAALYGDLLQED-EAAVAFLE----EFYVS--VSDDT--RMIAALK---EQ-LPELEKIV
KQV---SEE--PK---------APQKK---------------------------------
---HKV--------LLQLFNASD-DRAQ-KRQ----------------------------

-----PIRGSDE--VL-----------------------------FK-------------
VYCIDQTYTTIRVPVSSSVKEVIS--------AVADK-----------LGSG-EG--LII
VKMSSG----------------------GEKVL------LKPHDVSVFTTLSVNGRLFAC
PRDQ---F-DSLA-PLPEQ---------------------------EGP------STGTV
GT--FELMSSKDLAYQMTIYDWELFSCVHELELIY-------HTFGRHNF-KK-TTANLD

LFLRR---------FNEIQFWV-----VTEICLCSQLSKR------VQLLKKYIKIAAH-
--------------CKEYKNLNSFFAIIM-GLSNVAVSRLLLTWE---------------
-------KLPSKFKKIYAE-FESLM--------------------------DPSRNH---
-RAYRL----TVAK-------LDPPLI-PFMP----------------------------
LLIKDMTFTHEGNK--TF-TD-----------------------------K-LVNFE-KM
-------RM---IANTVRTVKFCRS--Q-------------------------PFNPD--
-A------------------ALANKNH-----Q----------------DVRS-----YV
RQLNVIDNQRTL---SQMSHRL
-----------EPRR----------A----------------------------------

>Rapgef4_[Poecilia_formosa]_617381047
---------------------------------MV-----A------AH---TSSHSSES
-AEWII---CLDK-----------RPAE-----RSGEDVDIILARLRNVKALERFHPRLL
QQICLCGF--YECLE--KGITLFRQGDIGTSWYAVLSGSLDVKVSE-TSSYQDA------
------VTICTLGTGTAFGESILDNT-PRHATIV-TREFSELL-----------------
----------------------------RIEQKE-------FRSLWEVCYKFHHCMAG-L
LAPPYG--VMD--S--N-GS-------DRMPEKE--------------------------
------------------NIS-------SNSFVSVSKHPNKK------------------
-----SFPESP---AKLH-TKSPT----------------QVPS----------------
-------EKIL-------------RA---------------------GKILR-HAILSRV
PH-M----------------IRDRKYH------L-KTY----------------RQC-CV
GT-----------------ELVDWQMLQC-------------------------------
---SCVH--------SRIQAVGMW---QVLLEEGVLN-----------------------
--------------H------------------------VDG-ETVFQDKY-LFYRFLDD
-------EQED----AL-I---
--PS
EEER--RESQEEL------QDTLLFLSQIGPDAL-------------MRMILRKPPSERT
PDDLE-----IIYEELLHIKALSHLS-TTVKRELA-GVLIFESHERAGTVL---------
--FHQGEEG--TSWYIIL
KGSVNVVIYGK-----GV-------------------------------VCTLYEG-DDF
GKLAL-----------VNDAPRA---ASIVLREDDCHFLRVDKEDFNR------ILRD--
---V--------------

------------------------EANTVRLKEHGQDVLVLEK-----SLS-SSRNSVQG
SS----AA-HH------------------------------------K-YNIMSGTPEKI
LEHLLEM------------MRSDS-QFT--------------ES------DSALD---D-
FVLTHCV--FI---------------PNSRLCPVLS-----D------------------
--------H-----------Y-------HAQASQ---DSEMEKLDYTLNNKRRVIRLVMK
WAAVHGEHLQQQ-DMV--FLE----EFLVA--VSEDA--KVLPALR---DQ-LTELEKII
KSS---ADD--AR---------SSQRK---------------------------------
---HKV--------LLQHFNMVD-EKLQ-KRQ----------------------------

-----PIKGDDE--IL-----------------------------FK-------------
VYCCDHTYTTIRVPVASSVREVLE--------AVADK-----------LGSA-ED--LIL
VGLSSA----------------------GEKVV------FKPNDVSIFSTLSINGRLFAC
QRDQ---L-DVLV-PLSEQ---------------------------EGL------SLGSL
AN--FELMSSKDVAYHMTFYDWELFHCVHELELIY-------HTFGRQHV-KK-TTVNLD
LFLQR---------FNEIQFWV-----ITEVCLCSQLNKR------VQLLKKFIKIAAH-
--------------CKEYKNLNAFFAVIL-GLSHPAVSRLSQTWE---------------
-------KLPSKFKKFYGE-FENLM--------------------------DPSRNH---
-RAYRL----TVAK-------LAPPVI-PFMP----------------------------
LMIKDMTFTHEGNK--TF-ID-----------------------------N-LVNFE-KM
-------RM---IAKTVKTVRYYRS--Q-------------------------TFSPD--
-S------------------PQAGKHH-----P----------------DVWT-----YV
RQLSVIDNQRTL---TQLSHGL
-----------EPRR----------S----------------------------------

>Rapgef4_[Poecilia_mexicana]_961931386
---------------------------------MV-----A------AH---TSSHSSES
-AEWII---CLDK-----------RPAE-----RSGEDVDIILARLRNVKALEGFHPRLL
QQICLCGF--YECLE--KGITLFRQGDIGTSWYAVLSGSLDVKVSE-TSSYRDA------
------VTICTLGTGTAFGESILDNT-PRHATIV-TREFSELL-----------------
----------------------------RIEQKE-------FRSLWE---KFHHCMAG-L
LAPPYG--VMD--S--N-GS-------DRMPEKE--------------------------
------------------NIS-------SNSFVSVSKHPNKK------------------
-----SFPESP---AKLH-TKSPT----------------QVPS----------------
-------EKIL-------------RA---------------------GKILR-HAILSRV
PH-M----------------IRDRKYH------L-KTY----------------RQC-CV
GT-----------------ELVDWQMLQC-------------------------------
---SCVH--------SRIQAVGMW---QVLLEEGVLN-----------------------
--------------H------------------------VDG-ETVFQDKY-LFYRFLDD
-------EQED----AL-I---
--PS
EEER--RESQEEL------QDTLLFLSQIGPDAL-------------MRMILRKPPSERT
PDDLE-----IIYEELLHIKALSHLS-TTVKRELA-GVLIFESHERAGTVL---------
--FHQGEEG--TSWYIIL
KGSVNVVIYGK-----GV-------------------------------VCTLYEG-DDF
GKLAL-----------VNDAPRA---ASIVLREDDCHFLRVDKEDFNR------ILRD--
---V--------------

------------------------EANTVRLKEHGQDVLVLEK-----SLS-SSRNSVQG

SS----AA-HH------------------------------------K-YNIMSGTPEKI
LEHLLEM------------MRSDS-QFT--------------ES------DSALD---D-
FVLTHCV--FI---------------PNSRLCPVLS-----D------------------
--------H-----------Y-------HAQASQ---DSEMEKLDYTLNNKRRVIRLVMK
WAAVHGEHLQQQ-DMV--FLE----EFLVA--VSEDA--KVLPALR---DQ-LTELEKII
KSS---ADD--AR---------SSQRK---------------------------------
---HKV--------LLQHFNMVD-EKLQ-KRQ----------------------------

-----PIKGDDE--IL-----------------------------FK-------------
VYCCDHTYTTIRVPVASSVREVLE--------AVADK-----------LGSA-ED--LIL
VGLSSA----------------------GEKVV------FKPNDVSIFSTLSINGRLFAC
QRDQ---L-DVLV-PLSEQ---------------------------EGP------SLGSL
AN--FELMSSKDVAYHMTFYDWELFHCVHELELIY-------HTFGRQHV-KK-TTVNLD
LFLQR---------FNEIQFWV-----ITEVCLCSQLNKR------VQLLKKFIKIAAH-
--------------CKEYKNLNAFFAVIL-GLSHPAVSRLSQTWE---------------
-------KLPSKFKKFYGE-FENLM--------------------------DPSRNH---
-RAYRL----TVAK-------LAPPVI-PFMP----------------------------
LMIKDMTFTHEGNK--TF-ID-----------------------------N-LVNFE-KM
-------RM---IAKTVKTVRYFRS--Q-------------------------TFSPD--
-S------------------PQAGKHH-----P----------------DVWT-----YV
RQLSVIDNQRTL---TQLSHGL
-----------EPRR----------S----------------------------------

>Rapgef4_[Poecilia_reticulata]_658838439

---MAG-L
LAPPYG--VME--S--S-AS-------DRMPEKE--------------------------
------------------NMS-------SNSFASVSKHPNKK------------------
-----SFPESP---AKLH-TKRPT----------------QVPS----------------
-------EKIL-------------QA---------------------GKILR-HAILSRA
PH-M----------------IRDRKYH------L-KTY----------------RQC-CV
GT-----------------ELVDWQMLQS-------------------------------
---SCVH--------SRIQAVGMW---QVLLEEGVLN-----------------------
--------------H------------------------VDG-ETVFQDKY-LFYRFLDD
-------EQED----AL-I---
--PS

EEER--RKSQEEL------QDTLLFLSQIGPDAL-------------MRMILRKPPSERT
PDDLE-----IIYEELLHIKALSHLS-TTVKRELA-GVLIFESHERAGTVL---------
--FHQGEEG--TSWYIIL
KGSVNVVIYGK-----GV-------------------------------VCTLHEG-DDF
GKLAL-----------VNDAPRA---ASIVLREDDCHFLRVDKEDFNR------ILRD--
---V--------------

------------------------EANTVRLKEHGQDVLVLEK-----SLS-SSRNSVQG
SS----AA-HH------------------------------------K-YNIMSGTPEKI
LEHLLEM------------MRSDS-QFT--------------ES------DSALD---D-
FVLTHCV--FI---------------PNSRLCPVLS-----D------------------
--------H-----------Y-------HAQASQ---GSEMEKLDYTLNNKRRVIRLVMK
WAAVHGEHLQQQ-DMV--FLE----EFFVA--VSEDA--KVLPALR---DQ-LTELEKII
KSS---ADD--AR---------SSQRK---------------------------------
---HKV--------LLQHFSMVD-EKLQ-KRQ----------------------------

-----PIKGDDE--IL-----------------------------FK-------------
VYCCDHTYTTIRVPVASSVREVLE--------AVADK-----------LGSA-ED--LIL
VGLSSA----------------------GEKVV------FKPNDVSIFSTLSINGRLFAC
QRXQ---L-DVLM-PLSEQ---------------------------EGP------SXGSL
AN--FELMSSKDVAYHMTFYDWELFHCVHELELIY-------HTFGRQHV-KK-TTVNLD
LFLQR---------FNEIQLWV-----ITEVCLCSQLSKR------VQLLKKFIKIAAH-
--------------CKEYKNLNAFFAVIM-GLSHPAVSRLSQTWE---------------
-------KLPSKFKKFYGE-FESLM--------------------------DPSRNH---
-RAYRL----TVAK-------LTPPVI-PFMP----------------------------
LMIKDMTFTHEGNK--TF-ID-----------------------------N-LVNFE-KM
-------RM---MAKTVKTVRYCRS--Q-------------------------TFNPD--
-S------------------PQAGKHH-----P----------------DVWT-----YV
RQLSVIDNQRTL---TQLSHGL
-----------EPRR----------S----------------------------------

>Rapgef4_[Pongo_abelii]_686709095

---MAG-L
LAPPYG--VMETGS--N-N--------DRIPDKE--------------------------

```
------------------N-----------------------------------------
-----------------------------------------VPS----------------
-------EKIL-------------RA---------------------GKILR-NAILSRA
PH-M----------------IRDRKYH------L-KTY----------------RQC-CV
GT-----------------ELVDWMMQQT-------------------------------
---PCVH--------SRTQAVGMW---QVLLEDGVLN-----------------------
--------------H------------------------VDQ-EHHFQDKY-LFYRFLDD
-------ENED----AP-L-----------------------------------------
----------------------------------------------------------PT
EEEK--KECDEEL------QDTMLLLSQMGPDAH-------------MRMILRKPPGQRT
VDDLE-----IIYEELLHIKALSHLS-TTVKRELA-GVLIFESHAKGGTVL---------
--------------------------------------------FNQGEEG--TSWYIIL
KGSVNVVIYGK-----GV-------------------------------VCTLHEG-DDF
GKLAL-----------VNDAPRA---ASIVLREDNCHFLRVDKEDFNR------ILRD--
---------------------------------------------V--------------
------------------------------------------------------------
------------------------------------------------------------
------------------------EANTVRLKEHDQDVLVLEK-----VPA-GNRASNQG
NS----QP-QQ------------------------------------K-YTVMSGTPEKI
LEHFLET------------IRLEA-TLN--------------EA-----TDSVLN---D-
FIMMHCV--FM---------------PNTQLCPALV-----A------------------
--------H-----------Y-------HAQPSQ---GTEQEKMDYALNNKRRVIRLVLQ
WAAMYGDLLQED-DVSMAFLE----EFYVS--VSDDA--RMIAALK---EQ-LPELEKIV
KQI---SED--AK---------APQKK---------------------------------
---HKV--------LLQQFNTGD-ERAQ-KRQ----------------------------
------------------------------------------------------------
------------------------------------------------------------
------------------------------------------------------------
------------------------------------------------------------
------------------------------------------------------------
------------------------------------------------------------
-----PIRGSDE--VL-----------------------------FK-------------
VYCMDHTYTTIRVPVAASVKEVIS--------AVADK-----------LGSG-EG--LIV
VKMSSG----------------------GEKVV------LKPNDVSVFTTLTINGRLFAC
PREQ---F-DSLT-PLPEQ---------------------------EGP------TVGTV
GT--FELMSSKDLAYQMTIYDWELFNCVHELELIY-------HTFGRHNF-KK-TTANLD
LFLRR---------FNEIQFWV-----VTEICLCSQLSKR------VQLLKKFIKIAAH-
--------------CKEYKNLNSFFAIAM-GLSNVAVSRLALTWE---------------
-------KLPSKFKKFYAE-FESLM--------------------------DPSRNH---
-RAYRL----TVAK-------LEPPVI-PFMP----------------------------
LLIKDMTFTHEGNK--TF-ID-----------------------------N-LVNFE-KM
-------RM---IANTARTVRYYRS--Q-------------------------PFNPD--
-A------------------AQANKNH-----Q----------------DVWS-----YV
RQLNVIDNQRTL-----------------------------------------SQMSHRL
-----------EPRR----------P----------------------------------
------------------------------------------------------------
------------------------------------------------------------
------------------------------------------------------------
------------------------------------------------------------
------------------------------------------------------------
------------------------------------------------------------
------------------------------------------------------------
------------------------------------------------------------
------------------------------------------------------------
```


>Rapgef4_[Priapulus_caudatus]_957848036
----------------------------MIAAYSANSN---------SSIN---------
---WIQC---LDK-----------RPSD-----RSSEDLEIIYTRLKNVKAFEKLNPMLL
QQICYYGY--YEDLE--KGVVLYRQGDLGTNWYTVLAGTLDVNVSE-TGHTDDS------
------VTVCTFGIGIAFGESVLDNS-PRHATVV-TSDYCELLRVEEKDFRLLWEQN---
--RE--FMEGVLSQLSGPLAELGL------------------------------------

-----------------------------------EKLNI--------------------
-----------EPQLP-----ARRPEHA----AS-----VNPALP---------IT----
---QEPSEKLS-------------RA---------------------GKVLR-TVLLSRA
PT-M----------------IRDRKFH------L-RTY----------------RRC-MV
AS-----------------DMVDWLLQQS-------------------------------
---TVVH--------SRGQAVGMW---QALLEEGVVA-----------------------
--------------H------------------------VSH-EHQFKDKY-LFYRFRED
-------DLGI----ST-V---
--PT
SAEK--KESEDEL------PETLNLLSQIGPDSM-------------LRMILRKTPQERT
ADDLE-----IIYEELLHIKALSHLS-TMVKRELA-SVLVFESHAHAGEVL---------
--FNQGDEG--KSWYIIL
KGSVNVVIYGK-----GV-------------------------------VCTLHEG-DDF
GKLAL-----------VNDAPRA---ASIVLRENNCHFLRVDKDDFNR------ILRD--
---V--------------

------------------------EANTVRLKEHGQDVLILEK-----IAAGGGGG-GGG
GS----QP-HY------------------------------------K-YSVMAGTPDKM
LEHLLETRL-GAAADDQS--------------------------------DTYLQ---D-
FLLTYVI--FM---------------PSGVLCPALM-----Q------------------
--------HYHTTANHGDDTAGGATDG------G----NQEDPVALILANKKRVIEFVQQ
WFNTAGDAFKED-QTIRAFLE----ELYHS--VLVDC--QSYTLLR---EQ-LTVLNGII
N----GINDKAQE------------DK---------------------------------
---SSRRSRGMPGTSIKKTKE---DKSA-AHQTYKSADYNIF------------------

--K-------------
VYCADHTYSTLKMRVDATAGQIVH--------VAREK-----------LCLG-ED--LQL
VEVKST----------------------GERVV------FKEEEVSVTTGLSVNGRLFIA
PREH---L-DSLT-PLPEQ---------------------------DGP------STSTC
SQ--LELMNSRDIAYQLAVFDWDLFTCVHKFELIY-------HVFGRDKF-GK-IMSNLD
VFLRR---------FNEVQYWV-----VTELCLTPTVSKR------VQLLRKYIKVAAH-
--------------CKEYQDLNSFFAIVM-GLSNIAISRLSQTWE---------------
-------KLPGKFKKMFAE-FEALM--------------------------DPSRNH---
-RVYRL----AVAK-------MSPPIV-PFMP----------------------------
LLMKDMTFTHEGNK--TY-FE-----------------------------A-LINFE-KM
-------HM---IAQTIRTIQYCRS--K-------------------------PFRIEQ-
-P------------------SS-LKNT-----T----------------DVRT-----YI
RNLKVIDNQRRL---TQLSHKL
-----------EPRR----------S----------------------------------

>Rapgef4_[Protobothrops_mucrosquamatus]_1002568461

---M----------------IRDRKYH------L-KTY----------------RQC-CV
GT-----------------ELVDWIMQQS-------------------------------
---SCVH--------LRTQAVGMW---QVLLEEGVLN-----------------------
--------------H------------------------VDQ-EQYFQDKY-LFYRFLDD
-------EHED----APVM---
--PT
EEEK--KECDEEL------QDTILLLAQIGPDAH-------------MRMILRKPPGQRT
VDDLE-----IIYEELLHIKALSHLS-TTVKRELA-GVLVFESHPKAGTVL---------
--FNQGEEG--TSWYIIL
KGSVNVVIYGK-----GV-------------------------------VCTLHEG-DDF
GKLAL-----------VNDAPRA---ASIVLREENCHFLRVDKEDFNR------ILRD--
---V--------------

------------------------EANTVRLKEHDQDVLVLEK-----VPA-GNRPFNQG
NA----QPLQH------------------------------------K-YIVMSGTPEKI
LEHFLET------------MRLES-TLN--------------EG-----MDAGVN---D-
FVTMHCV--FM---------------PNNQLCPALM-----A------------------
--------H-----------Y-------HSQPSQ---GTEQEKLDYALNNKRRVVRLVLL
WAAFYGDVLPEE-EAAVAFLE----EFYVS--VSDDA--RVITALK---EQ-FPELEKTM
KQI---SEE--PK---------GPQKK---------------------------------
---HKV--------LLQHFNMSD-ERTQ-KRQ----------------------------

-----PIRGSDE--IL-----------------------------FK-------------
VYCVDHTYTTIRIPVFSSVKEVLR--------AVADK-----------LGSG-DG--LIL
VKMSSA----------------------GEKVV------LKPQDVSVFTTLSVNGRLFAC
PRDQ---F-DSLV-PLQEQ---------------------------EGP------STGTM
GT--FELMSSKDLAYQMTIFEWELFNCVHELELVY-------HTFGRHHF-KK-TTANLE

LFLRR---------FNEIQFWV-----VTEICLCSQLSKR------VQLLKKFIKIAAH-
--------------CKEYKNLNSFFALIM-GLSNVAVSRLTMTWE---------------
-------KLPSKFKKIYAE-FESLM--------------------------DPSRNH---
-RAYRL----TVAK-------LEPPII-PFMP----------------------------
LLIKDMTFTYEGNK--TF-ID-----------------------------N-LVNFE-KM
-------RM---ISNTIKTVRFCRS--L-------------------------PFNPD--
-A------------------SQANKNH-----Q----------------DLRG-----YV
RQLNVIDNQRTL---SQMSHRL
-----------EPRR----------T----------------------------------

>Rapgef4_[Pseudopodoces_humilis]_929420255
----------------------------MVAA-------HA------SH--------SSS
SGEWIA---CLDK-----------RPLE-----RSSEDVDIIFTRLKEVKAFEKFHPNLL
QQICLCGY--YENLE--KGITLFRQGDIGTNWYAVLTGSLDVKVSD-TSNHQDA------
------VTICTLGIGTAFGESILDNT-PRHATIV-TSEHSELL-----------------
----------------------------RIEQKD-------FKALW---EKYRQYMSG-L
LTPPYG--VMETGS--N-N--------DRMPDKD--------------------------
------------------SMS-------SSALCQVSKNCNKT------------------
-----PLIEPHIPHHPTK-TITQ------------------VPS----------------
-------EKIL-------------RA---------------------GKILR-NTILSRA
PH-M----------------IRDRKYH------L-KTY----------------RQC-CV
GT-----------------ELVDWMMQQS-------------------------------
---PCVH--------LRTQAVGMW---QVLLEEGVLN-----------------------
--------------H------------------------VDQ-EHHFQDKY-LFYRFLDD
-------ERED----AP-L---
--PT
EEEK--KECDEEL------QDTMLLLSQIGPDAH-------------MRMILRKPPGQRT
VDDLE-----FIYEELLHIKALSHLS-TTVKRELA-GVLIFESHPKAGTVL---------
--FNQGEEG--TSWYIIL
KGSVNVVIYGK-----GV-------------------------------VCTLHEG-DDF
GKLAL-----------VNDAPRA---ASIVLREDNCHFLRVDKEDFNR------ILRD--
---V--------------

------------------------EANTVRLKEHDQDVLVLEK-----IPA-GNRISNQG
NP----QP-QH------------------------------------K-YIVMSGTPEKI
LEHFLET------------MRLEA-TLN--------------EA-----TDSVLN---D-
FIMMHCV--FM---------------PNSQLCPALM-----A------------------
--------H-----------Y-------HAQPSQ---GTEQEKMDYALNNKRRVIRLVLQ
WAALYGDLLQED-EAAMAFLE----EFYVS--VSDDT--RMIAALK---EQ-LPELEKIV
KQI---SEE--PK---------APQKK---------------------------------
---HKV--------LLQLFNTSD-DRAQ-KRQ----------------------------

-----PIRGSDE--VL-----------------------------FK-------------
VYCIDQTYTTIRVPVSSSVKEVIS--------AVADK-----------LGSG-EG--LII
VKMSSG----------------------GEKVV------LKPHDVSVFTTLSVNGRLFAC
PRDQ---F-DSLA-PLPEQ---------------------------EGP------STGTI
GT--FELMSSKDLAHQMTIYDWELFNCVHELELIY-------HTFGRHNF-KK-TTANLD
LFLRR---------FNEIQFWV-----VTEICLCSQLSKR------VQLLKKYIKIAAH-
--------------CKEYKNLNSFFAIIM-GLSNVAVSRLSLTWE---------------
-------KLPSKFKKIYAE-FESLM--------------------------DPSRNH---
-RAYRL----TVAK-------LDPPII-PFMP----------------------------
LLIKDMTFTHEGNK--TF-TD-----------------------------N-LVNFE-KM
-------RM---IANTVRTVKFCRS--Q-------------------------SFNPD--
-A------------------ALTNKNH-----Q----------------DVRS-----YV
RQLNVIDNQRTL---SQMSHRL
-----------EPRR----------A----------------------------------

>Rapgef4_[Pterocles_gutturalis]_704489718

-------------------------PLE-----RSSEDVDIIFTRLKEVKAFEKFHPNLL
QQICLCGY--YENLE--KGITLFRQGDIGTNWYAVLTGSLDVKVSD-TSNHQDA------
------VTICTLGIGTAFGESILDNT-PRHATIV-TRECSELL-----------------
----------------------------RIEQKD-------FKALW---EKYRQYMSG-L
LTPPYG--VMETGS--N-N--------DRMPDKD--------------------------
------------------N----------------------T------------------
-----PLTEPHIPHHPTK-TITQ------------------VPS----------------
-------EKIL-------------RA---------------------GKILR-NTILSRA
PH-M----------------IRDRKYH------L-KTY----------------RQC-CV
GT-----------------ELVDWMMQQS-------------------------------
---PCVH--------SRPQAVGMW---QVLLEEGVLN-----------------------
--------------H------------------------VDQ-EYYFQDKY-LFYRFLDD
-------EHED----AH-L---
--PT
EEEK--KECDEEL------QDTMLLLSQIGPDAH-------------MRMILRKPPGQRT
VDDLE-----IIYEELLHIKALSHLS-TTVKRELA-GVLIFESHPKAGTVL---------
--FNQGEEG--TSWYIIL
KGSVNVVIYGK-----GV-------------------------------VCTLHEG-DDF
GKLAL-----------VNDAPRA---ASIVLREDNCHFLRVDKEDFNR------ILRD--
---V--------------

------------------------EANTVRLKEHDQDVLVLEK-----IPA-GNRISNQG

NS----QP-QH------------------------------------K-YIVMSGTPEKI
LEHFLET------------MRLEA-TLN--------------EA-----TDSALN---D-
FIMMHCV--FM---------------PNSQLCPALM-----A------------------
--------HY------------------HAQPSQ---GTEQEKMDYALNNKRRVIRLVLQ
WAALYGDLLQED-EAAMAFLE----EFYVS--VSDDT--RMIAPLK---DQ-LPELEKIV
KQV---SED--PK---------ASQKK---------------------------------
---HKV--------LLQLFNTSD-DRAQ-KRQ----------------------------

-----PIRGSDE--VL-----------------------------FK-------------
VYCIDQTYTTIRVPVSSSVKEVIS--------AVADK-----------LGSG-EG--LVI
VKMSSG----------------------GEKVM------LKPHDVSVFTTLSVNGRLFAC
PRDQ---F-NSLA-PLPEQ---------------------------EGP------SAGTV
GT--FELMSSKDLAYQMTIYDWELFNCVHEMELIY-------HTFGRHNF-KK-TTANLD
LFLRR---------FNEIQFWV-----VTEICLCSQLSKR------VQLLKKYIKIAAH-
--------------CKEYKNLNSFFAIIM-GLSNVAVSRLSLTWE---------------
-------KLPSKFKKIYAE-FESLM--------------------------DPSRNH---
-RAYRL----TVTK-------LDPPII-PFMP----------------------------
LLIKDMTFTHEGNK--TF-TD-----------------------------N-LVNFE-KM

-------------------------V----------------------------------

>Rapgef4_[Pteropus_alecto]_586532736
----------------------------MVAA-------HA------AH--------SSS
SAEWIA---CLDK-----------RPLE-----RSSEDVDIIFTRLKEVKAFEKFHPNLL
HQICLCGY--YENLE--KGITLFRQGDIGTNWYAVLAGSLDVKVSE-TSSHQDA------
------VTICTLGIGTAFGESILDNT-PRHATIV-TRESSELL-----------------
----------------------------RIEQKD-------FKALW---EKYRQYMAG-L
LAPPYG--VMETGS--N-N--------DRIPDKE--------------------------
------------------N---
---VPS----------------
-------EKIL-------------RA---------------------GKILR-NAILSRA
PH-M----------------IRDRKYH------L-KTY----------------RQC-CV
GT-----------------ELVDWMLQQT-------------------------------
---PCVH--------SRTQAVGMW---QVLVEDGVLN-----------------------
--------------H------------------------VDQ-EHHFQDKY-LFYRFLDD
-------EHED----AP-L---
--PT

EEEK--KECDEEL------QDTMLLLSQTGPDAH-------------MRMILRKPPGQRT
VDDLE-----IIYEELLHIKALSHLS-TTVKRELA-GVLIFESHAKGGTVL---------
--FNQGEEG--TSWYIIL
KGSVNVVIYGK-----GV-------------------------------VCTLHEG-DDF
GKLAL-----------VNDAPRA---ASIVLREDNCHFLRVDKEDFNR------ILRD--
---V--------------

------------------------EANTVRLKEHDQDVLVLEK-----VPA-GNRAPNQG
NS----QP-QQ------------------------------------K-YTVMSGTPEKI
LEHFLET------------IRLEP-ALN--------------EA-----TDSILN---D-
FVMMHCV--FM---------------PNTQLCPALV-----A------------------
--------H-----------Y-------HAQPSQ---GTEQEKMDYALNNKRRVIRLVLQ
WAAMYGDVLQED-DVAMAFLE----EFYVS--VSDDA--RMITAFK---EQ-LPELEKIV
KQI---SED--AK---------NPQKK---------------------------------
---HKV--------LLQQFNTGD-ERAQ-KHQ----------------------------

-----PIRGSDE--VL-----------------------------FK-------------
VYCMDHTYTTIRVPVAASVKEVIS--------AVADK-----------LGSG-EG--LII
VKMSSA----------------------GEKVV------LKPNDVSVFTTLTINGRLFAC
PREQ---F-DSLT-PLPEQ---------------------------EGP------TVGTV
GT--FELMSSKDLAYQMTIYDWELFNCVHELELIY-------HTFGRHNF-KK-TTANLD
LFLRR---------FNEIQFWV-----VTEICLCSQPSKR------VQLLKKFIKIAAH-
--------------CKEYKNLNSFFAIVM-GLSNVAVSRLALTWE---------------
-------KLPSKFKKFYAE-FESLM--------------------------DPSRNH---
-RAYRL----TVAK-------LEPPLI-PFMP----------------------------
LLIKDMTFTHEGNK--TF-ID-----------------------------N-LVNFE-KM
-------RM---IANTARTVRYCRS--Q-------------------------PFNLD--
-A------------------AQANKNH-----Q----------------DVRS-----YV
RQLNVIDNQRTL---SQMSHRL
-----------EPRR----------P----------------------------------

>Rapgef4_[Pundamilia_nyererei]_548336931

---------MESGS----NN-------DRLTDKD--------------------------

```
------------------NMN-------S-------------------------------
--------DSA---NKAH-----N----------------KIPS----------------
-------EKLQ-------------RA---------------------GKVLR-NAILSRA
PH-M----------------IRDRKYH------L-KTY----------------RQC-CV
GT-----------------ELVDWLVLQS-------------------------------
---ACVL--------TRSHAVGMW---QALLEEGVLN-----------------------
--------------H------------------------VDQ-ELGFYDKY-LFYRFLDD
-------EDED----TP-L-----------------------------------------
----------------------------------------------------------PS
EEEK--RESEEEL------PETILFLAQIGPDAL-------------LRMILRKSPGQRT
GDDLE-----IIYDELLHIKALAHLS-NTVKRELA-SVVIFESHAKAGTVL---------
--------------------------------------------FNQGEEG--TSWYIIL
KGSVNVVIYGK-----GV-------------------------------VCTLHEG-DDF
GKLAL-----------VTDSPRA---ASIVLREDNCHFLRVDKEDFNR------ILRD--
---------------------------------------------V--------------
------------------------------------------------------------
------------------------------------------------------------
------------------------EANTVRLKEHDHPVLVLEK-----S----PRASTLG
N---------I------------------------------------K-YTVISGTPEKI
LEHFLET------------MRMDI-HHS--------------DP------DPAVD---D-
FVLMHCV--FM---------------PNSQLCPLLL-----S------------------
--------Q-----------Y-------HVTSPP---GSEQERLEYALNSKRRALIMTLR
WANAHTYLLQEE-PAAIFFLE----ELYGS--LSNDS--RMLRALK---DL-VPDLEKVV
KLH---SEE--AK---------ATKKK---------------------------------
-----T--------LIRQFSNGE-ERLQ-KKQ----------------------------
------------------------------------------------------------
------------------------------------------------------------
------------------------------------------------------------
------------------------------------------------------------
------------------------------------------------------------
------------------------------------------------------------
-----PIRNQDD--IL-----------------------------LK-------------
VYCSDHTYTTIRIPVAATGREVIS--------AVTDK-----------LGTT-DE--LLL
VHLSSA----------------------GEKQI------LKPNDVSVFSTLSINGRLFAC
PREQ---L-NSLT-PLPDQ---------------------------EGP------TAGSM
ST--FELMSSKDLAYQMTMFDWELFSCVHEHELLY-------HTFGCQSF-KR-TTANLD
LFLRR---------FNQVQLWV-----VTEVCLCGQLSKR------VQLLKKFIKIAAH-
--------------CREFKNLNSFFAIIM-GMSNPAVSRLSQTWE---------------
-------KLPTKFKKFYAE-FENMM--------------------------DPSRNH---
-RSYRL----TVTK-------LEPPII-PFVP----------------------------
LLLKDMTFTHEGNK--TF-ID-----------------------------N-MINFE-KM
-------RI---IANTIRQVRHCRS--Q-------------------------PFNPD--
-I------------------CQPNKNQ-----A----------------EVRA-----YV
RKLCVIDNQRAL-----------------------------------------TQLSYRL
-----------EPRR----------T----------------------------------
------------------------------------------------------------
------------------------------------------------------------
------------------------------------------------------------
------------------------------------------------------------
------------------------------------------------------------
------------------------------------------------------------
------------------------------------------------------------
------------------------------------------------------------
------------------------------------------------------------
```


>Rapgef4_[Pygoscelis_adeliae]_679131078

------------------------RPLE-----RSSEDVDIIFTRLKEVKAFEKFHPNLL
QQICLCGY--YENLE--KGITLFRQGDIGTNWYAVLTGSLDVKVSD-TSNHQDA------
------VTICTLGIGTAFGESILDNT-PRHATIV-TREYSELL-----------------
----------------------------RIEQKD-------FKALW---EKYRQYMSG-L
LTPPYG--VMEASS--N-N--------DSKYEKI--------------------------
------------------D----------------------T------------------
-----PLIEPHIPHRPTK-TITQ------------------VPS----------------
-------EKIL-------------RA---------------------GKILR-NTILSRA
PH-M----------------IRDRKYH------L-KTY----------------RQC-CV
GT-----------------ELVDWMMQQS-------------------------------
---TCVH--------SRTQAVGMW---QVLLEEGVLN-----------------------
--------------H------------------------VDQ-EHHFQDKY-LFYRFLDD
-------ERED----AP-L---
--PT
EEEK--KECDEEL------QDTMLLLSQIGPDAH-------------MRMILRKPPGQRT
VDDLE-----IIYEELLHIKALSHLS-TTVKRELA-GVLVFESHPKAGTVL---------
--FNQGEEG--TSWYIIL
KGSVNVVIYGK-----GV--------------------------------VCTLHEG-DDF
GKLAL-----------VNDAPRA---ASIVLREDNCHFLRVDKEDFNR------ILRD--
---V--------------

------------------------EANTVRLKEHDQDVLVLEK-----VPA-GNRVSNQG
NS----QP-QH------------------------------------K-YIVMSGTPEKI
LEHFLET------------MRPEA-TLN--------------EA-----TDSVLN---D-
FIMMHCV--FM---------------PNSQLCPALM-----A------------------
--------QYPF----HRGTY-------HAQPSQ---GTEQEKMDYALNNKRRVIRLVLQ
WAALYGDLLQED-EAAMAFLE----EFYVS--VSDDT--RMIAALK---EQ-LPELEKIV
KQV---SEE--SK---------APQKK---------------------------------
---HKV--------LLQLFNTSD-DRAQ-KRQ----------------------------

-----PIRGSDE--VL-----------------------------FK-------------
VYCIDQTYTTIRVPVSSSVKEVIS--------AVADK-----------LGSG-EG--LII
VKMSSG----------------------GEKVV------LKPHDVSVFTTLSVNGRLFAC
PRDQ---F-DSLA-PLPEQ----------------------------EGP------STGTV
GT--FELMSSKDLAHQMTIYDWELFNCVHELELIY-------HTFGRHNF-KK-TTANLD
LFLRR---------FNEIQFWV-----VTEICLCSQLSKR------VQLLKKYIKIAAH-
--------------CKEYKNLNSFFAIIM-GLSNVAVSRLSLTWE---------------
-------KLPSKFKKIYAE-FESLM--------------------------DPSRNH---
-RAYRL----TVAK-------LDPPII-PFMP----------------------------
LLIKDMTFTHEGNK--TF-TD------------------------------N-LVNFE-KM
-------RM---IANTVRTVKFCRS--Q--------------------------SFNPD--
-A------------------ALTNKNH-----Q----------------DVRS-----YV
RQLNVIDNQRTL---SHMSHRL
-----------ELRR----------A----------------------------------

>Rapgef4_[Python_bivittatus]_1004668304
---M
VAVHI-SHSSSSTEWIA--C-LDKRPLE-----RSSEDVDIIFTRLKEVK----------

--------------------AFEKFH-PNLLQQIC--LCGYYENLEKGITL---------
--FRQGDIG--TNWYAVL
TGSLDVKVSDTSNHQDAVT------------------------------ICTLGIG-TAF
GE-SV-----------LDNTPRH---ATIVTRE-NSELLRIEQKDFKA------LWE---
------IPKLEQS----------TIPYGTSGARRH-------------------------

>Rapgef4_[Rattus_norvegicus]_293346052
----------------------------MVAA-------HA------AH--------SPS
SAEWIA---CLDK-----------RPLE-----RSSEDVDIIFTRLKGVKAFERFHPNLL
RQICLCSY--YENLE--KGITLFRQGDIGTNWYAVLAGSLDVKVSE-TSSHQDA------
------VTICTLGIGTAFGESILDNT-PRHATIV-TRENSELL-----------------
----------------------------RIEQED-------FKALW---EKYRQYMAG-L
LAPPYG--VMETGS--N-N--------DRIPDKE--------------------------
------------------N----------------------T------------------
-----PLIEPHVPLRPAH-TITK------------------VPS----------------
-------EKIL-------------RA---------------------GKILR-IAILSRA
PH-M----------------IRDRKYH------L-KTY----------------RQC-CV
GT-----------------ELVDWMIQQT-------------------------------
---SCVH--------SRTQAVGMW---QVLLEDGVLN------------------------
--------------H------------------------VDQ-ERHFQDKY-LFYRFLDD
-------EHED----AP-L---
--PT
EEEK--KECDEEL------QDTMLLLSQMGPDAH-------------MRMILRKPPGQRT
ADDLE-----IIYDELLHIKALSHLS-TTVKRELA-GVLIFESHAKGGTVL---------
--FNQGEEG--TSWYIIL
KGSVNVVIYGK-----GV-------------------------------VCTLHEG-DDF
GKLAL-----------VNDAPRA---ASIVLREDNCHFLRVDKEDFNR------ILRD--
---V--------------

------------------------EANTVRLKEHDQDVLVLEK-----VPA-GNRASNQG
NS----QP-QQ------------------------------------K-YTVMSGTPEKI
LEHFLET------------IRLEP-SLN--------------EA-----TDSVLN---D-
FVMMHCV--FM---------------PNTQLCPALV-----A------------------
--------H-----------Y-------HAQPSQ---GTEQERMDYALNNKRRVIRLVLQ
WAAMYGDLLQED-DVAMAFLE----EFYVS--VSDDA--RMMVAFK---EQ-LAELEKTV
KQI---SED--AK---------APQKK---------------------------------
---HKV--------LLQQFNTGD-ERAQ-KRQ----------------------------

-----PIRGSDE--VL-----------------------------FK-------------
VYCIDHTYTTIRVPVAASVKEVIS--------AVADK-----------LGSG-EG--LII
VKMNSG----------------------GEKVV------LKPNDVSVFTTLTINGRLFAC
PREQ---F-DSLT-PLPEQ---------------------------EGP------TTGTV
GT--FELMSSKDLAYQMTTYDWELFNCVHELELIY-------HTFGRHNF-KK-TTANLD
LFLRR---------FNEIQFWV-----VTEICLCSQLSKR------VQLLKKFIKIAAH-
--------------CKEYKNLNSFFAIVM-GLSNVAVSRLALTWE---------------
-------KLPSKFKKFYAE-FESLM--------------------------DPSRNH---
-RAYRL----TAAK-------LEPPLI-PFMP----------------------------
LLIKDMTFTHEGNK--TF-ID-----------------------------N-LVNFE-KM
-------RM---IANTARTVRYYRS--Q-------------------------PFNPD--
-A------------------AQANKNH-----Q----------------DVRS-----YV
RQLNVIDNQRTL---SQMSHRL
-----------EPRR----------P----------------------------------

>Rapgef4_[Rhinopithecus_roxellana]_724894120
----------------------------MRFL-------MP------TV--------MAM
S-----------------------RPLE-----RSSEDVDIIFTRLKEVKAFEKFHPNLL
HQICLCGY--YENLE--KGITLFRQGDIGTNWYAVLAGSLDVKVSE-TSSHQDA------
------VTICTLGIGTAFGESILDNT-PRHATIV-TRESSELL-----------------
----------------------------RIEQKD-------FKALW---EKYRQYMAG-L
LAPPYG--VMETGS--N-N--------DRIPDKE--------------------------
------------------N----------------------T------------------
-----PLIEPHVPLRPAN-TITK------------------VPS----------------
-------EKIL-------------RA---------------------GKILR-NAILSRA
PH-M----------------IRDRKYH------L-KTY----------------RQC-CV
GT-----------------ELVDWMMQQT-------------------------------
---PCVH--------SRTQAVGMW---QVLLEDGVLN-----------------------
--------------H------------------------VDQ-EHHFQDKY-LFYRFLDD
-------EHED----AP-L---
--PT
EEEK--KECDEEL------QDTMLLLSQMGPDAH-------------MRMILRKPPGQRT
VDDLE-----IIYEELLHIKALSHLS-TTVKRELA-GVLIFESHAKGGTVL---------
--FNQGEEG--TSWYIIL
KGSVNVVIYGK-----GV-------------------------------VCTLHEG-DDF
GKLAL-----------VNDAPRA---ASIVLREDNCHFLRVDKEDFNR------ILRD--
---V--------------

------------------------EANTVRLKEHDQDVLVLEK-----VPA-GNRASNQG

NS----QP-QQ------------------------------------K-YTVMSGTPEKI
LEHFLET------------IRLEA-TLN--------------EA-----TDSVLN---D-
FIMMHCV--FM---------------PNTQLCPALV-----A------------------
--------H-----------Y-------HAQPSQ---GTEQEKMDYALNNKRRVIRLVLQ
WAAMYGDLLQED-DVSMAFLE----EFYVS--VSDDA--RMIAALK---EQ-LPELEKIV
KQI---SED--AK---------APQKK---------------------------------
---HKV--------LLQQFNTGD-ERAQ-KRQ----------------------------

-----PIRGSDE--VL-----------------------------FK-------------
VYCMDHTYTTIRVPVAASVKEVIS--------AIADK-----------LGSG-EG--LII
VKMSSG----------------------GEKVV------LKPNDVSVFTTLTINGRLFAC
PREQ---F-DSLT-PLPEQ---------------------------EGP------TIGTV
GT--FELMSSKDLAYQMTIYDWELFNCVHELELIY-------HTFGRHNF-KK-TTANLD
LFLRR---------FNEIQFWV-----VTEICLCSQLSKR------VQLLKKFIKIAAH-
--------------CKEYKNLNSFFAIVM-GLSNVAVSRLALTWE---------------
-------KLPSKFKKFYAE-FESLM--------------------------DPSRNH---
-RAYRL----TVAK-------LEPPLI-PFMP----------------------------
LLIKDMTFTHEGNK--TF-ID-----------------------------N-LVNFE-KM
-------RM---IANTARTVRYYRS--Q-------------------------PFNPD--
-A------------------AQANKNH-----Q----------------DVRS-----YV
RQLNVIDNQRTL---SQMSHRL
-----------EPRR----------P----------------------------------

>Rapgef4_[Rousettus_aegyptiacus]_1012008377

---MAG-L
LAPPYG--VMETGS--N-N--------DRIPDKE--------------------------
------------------N---
---VPS----------------
-------EKIL-------------RA---------------------GKILR-NAILSRA
PH-M----------------IRDRKYH------L-KTY----------------RQC-CV
GT-----------------ELVDWMLQQT-------------------------------
---PCVH--------SRTQAVGMW---QVLVEDGVLN-----------------------
--------------H------------------------VDQ-EHHFQDKY-LFYRFLDD
-------EHED----AP-L---
--PT

EEEK--KECDEEL------QDTMLLLSQTGPDAH-------------MRMILRKPPGQRT
VDDLE-----IIYEELLHIKALSHLS-TTVKRELA-GVLIFESHAKGGTVL---------
--FNQGEEG--TSWYIIL
KGSVNVVIYGK-----GV-------------------------------VCTLHEG-DDF
GKLAL-----------VNDAPRA---ASIVLREDNCHFLRVDKEDFNR------ILRD--
---V--------------

------------------------EANTVRLKEHDQDVLVLEK-----VPA-GNRAPNQG
NS----QP-QQ------------------------------------K-YTVMSGTPEKI
LEHFLET------------IRLEP-ALN--------------EA-----TDSILN---D-
FVMMHCV--FM---------------PNTQLCPALV-----A------------------
--------H-----------Y-------HAQPSQ---GTEQEKMDYALNNKRRVIRLVLQ
WAAMYGDVLQED-DVAIAFLE----EFYVS--VSDDA--RMIAAFK---EQ-LPELEKIV
KQI---SED--AK---------NPQKK---------------------------------
---HKV--------LLQQFNTGD-ERAQ-KHQ----------------------------

-----PIRGSDE--VL-----------------------------FK-------------
VYCMDHTYTTIRVPVAASVKEVIS--------AVADK-----------LGSG-ES--LII
VKMSSA----------------------GEKVV------LKPNDVSVFTTLTINGRLFAC
PREQ---F-DSLT-PLPEQ---------------------------EGP------TVGTV
GT--FELMSSKDLAYQMTIYDWELFNCVHELELIY-------HTFGRHNF-KK-TTANLD
LFLRR---------FNEIQFWV-----VTEICLCSQPSKR------VQLLKKFIKIAAH-
--------------CKEYKNLNSFFAIVM-GLSNVAVSRLALTWE---------------
-------KLPSKFKKFYAE-FESLM--------------------------DPSRNH---
-RAYRL----TVAK-------LEPPLI-PFMP----------------------------
LLIKDMTFTHEGNK--TF-ID-----------------------------N-LVNFE-KM
-------RM---IANTARTVRYCRS--Q-------------------------PFNLD--
-A------------------AQANKNH-----Q----------------DVRS-----YV
RQLNVIDNQRTL---SQMSHRL
-----------EPRR----------P----------------------------------

>Rapgef4_[Saccoglossus_kowalevskii]_585695958

---------------------------MGTNWYAVLTGTVDVNVSE-TGDPKDA------
------ITICTLGVGTAFGESVLSDT-PRHATVI-TTEYCEFLRIEQKDFRSLWERN---
--KQ--FMEGMIN--SNPVPALPSNTAYLQQQEITGSPIT--------------------
---------------------------------PRRAY----S-----------------

```
-----------------LD---RGSPALISRDSLNESLEI--------------------
-----------IPKLS-----QSPKPHGEDGFGA-----QHPALP---------LT----
---DTPSERLA-------------RA---------------------GYVIR-SVILTRS
PH-M----------------IRDRKYH------L-RTY----------------RRC-LV
GS-----------------EMVDWLVQQS-------------------------------
---SFVH--------SRNQAVGMW---QALLEEGVIT-----------------------
--------------H------------------------VCH-EHQFKDKY-LFYRFRED
-------DLGI----GT-V-----------------------------------------
----------------------------------------------------------PT
NNEK--KECEDEL------QDTLVMLAQISPDAM-------------MRMILRKPPPERT
VDDLE-----IIYEELLHIKALSHLS-TMVKRELA-SVLKFESHQKAGTVL---------
--------------------------------------------FNQGDEG--KSWYIIL
RGSVNVVIYGK-----GV-------------------------------VCTLHEG-DDF
GKLAL-----------VNDAPRA---ASIVLREDNCHFLRVDKDDFNR------ILRD--
---------------------------------------------V--------------
------------------------------------------------------------
------------------------------------------------------------
------------------------EANTVRLKEHGQDVLVLEK-----ILT---NR-GED
GT----PT-HY------------------------------------K-YSVMAGTPEKM
LEHLLEAHI-DKY--NEL--------------------------------DTFVE---D-
FLLTHVV--FM---------------ESSELCPTLM-----S------------------
--------HYHAKASQ-----------------G----TEQEMADYALNNKRRVIHLVLQ
WSSVAGDDFRED-TTIAAFID---------------------------------------
------------------------------------------------------------
------------------------------RKSIKPNDENIF------------------
------------------------------------------------------------
------------------------------------------------------------
------------------------------------------------------------
------------------------------------------------------------
------------------------------------------------------------
------------------------------------------------------------
----------------------------------------------K-------------
VYCADHTYTTLRLPIDSKASVIAQ--------SAADK-----------ICIG-ED--LVL
CEVKST----------------------GERVV------IKDNEVGVTTTLSLNGRLFIA
TREH---L-DALT-PIPEQ---------------------------EGP------SQGTS
SV--FELMSTKEVAYQMTLYDWELLNCLHEYELIY-------HTFGRHKF-GK-ITCNLD
MLLRR---------FNEVQFWV-----VTEMCLTNTLGKR------VQLLRKFIKIAAF-
--------------CKEYKNMNSFFSIVM-GLSNIAVSRLSQTWE---------------
-------KLPSKFKRMFAE-FETTL--------------------------DPSRNH---
-RVYRL----AVAK-------MQPPII-PFMP----------------------------
LLMKDMTFTHEGNK--TY-FD-----------------------------G-LVNFE-KM
-------HM---IATTIRTIKYCRS--E-------------------------PFKVEP-
-P------------------PA-VKNV-----Q----------------EVRA-----YV
RNLSVIDNQRTL-----------------------------------------TQLSHKL
-----------EPRR----------S----------------------------------
------------------------------------------------------------
------------------------------------------------------------
------------------------------------------------------------
------------------------------------------------------------
------------------------------------------------------------
------------------------------------------------------------
------------------------------------------------------------
------------------------------------------------------------
------------------------------------------------------------
```


>Rapgef4_[Saimiri_boliviensis_boliviensis]_403258758

---MAG-L
LAPPYG--VMETGS--N-N--------DRIPDKE--------------------------
------------------N---
---VPS----------------
-------EKIL-------------KA---------------------GKILR-NAILSRA
PH-M----------------IRDRKYH------L-KTY----------------RQC-CV
GT-----------------ELVDWMMQQT-------------------------------
---PCVH--------SRTQAVGMW---QVLLEDGVLN-----------------------
--------------H------------------------VDQ-EHHFQDKY-LFYRFLDD
-------EHED----AP-L---
--PT
EEEK--KECDEEL------QDTMLLLSQMGPDAH-------------MRMILRKPPGQRT
VDDLE-----IIYEELLHIKALSHLS-TTVKRELA-GVLIFESHAKGGTVL---------
--FNQGEEG--TSWYIIL
KGSVNVVIYGK-----GV-------------------------------VCTLHEG-DDF
GKLAL-----------VNDAPRA---ASIVLREDNCHFLRVDKEDFNR------ILRD--
---V--------------

------------------------EANTVRLKEHDQDVLVLEK-----VPA-GNRASNQG
NS----QP-QQ------------------------------------K-YTVMSGTPEKI
LEHFLET------------IRLEA-SLN--------------EA-----TDSVLN---D-
FIMMHCV--FM---------------PNTQLCPALV-----A------------------
--------H-----------Y-------HAQPSQ---GTEQEKMDYALNNKRRVIRLVLQ
WAAMYGDLLQED-DVSMAFLE----EFYVS--VSDDA--RMIAALK---EQ-LPELEKIV
KQI---SED--AK---------APQKK---------------------------------
---HKV--------LLQQFNTGD-ERAQ-KRQ----------------------------

-----PIRGSDE--VL-----------------------------FK-------------
VYCMDHTYTTIRVPVAASVKEVIS--------AVADK-----------LGSG-EG--LIV
VKMSSG----------------------GEKVV------LKPNDVSFTTLTINGRLFAC
PREQ---F-DSLT-PLPEQ---------------------------EGP------TVGTV
GT--FELMSSKDLAYQMTIYDWELFHCVHELELIY-------HTFGRHNF-KK-TTANLD
LFLRR---------FNEIQFWV-----VTEICLCSQLSKR------VQLLKKFIKIAAH-
--------------CKEYKNLNSFFAIVM-GLSNVAVSRLALTWE---------------
-------KLPSKFKKFYAE-FESLM--------------------------DPSRNH---
-RAYRL----TVAK-------LEPPLI-PFMP----------------------------
LLIKDMTFTHEGNK--TF-ID-----------------------------N-LVNFE-KM
-------RM---IANTARTVRYYRS--Q-------------------------PFNPD--
-A------------------AQANKNH-----Q----------------DVRS-----YV
RQLNVIDNQRTL---SQMSHRL
-----------EPRR----------P----------------------------------

>Rapgef4_[Salmo_salar]_929302579

--------------------------------MK-------ITRLG-T-RKYRQCMAG-L
LAPSYG--VMESGG--P-SP-------DRMPDKE--------------------------
------------------NIS-------NNSFGSISKHLSKK------------------
-----SFTENPAKLHP-K-SISQ------------------VPS----------------
-------EKIM-------------RA---------------------GKVLR-DAILSRA
PH-I----------------IRDRKYH------L-KTY----------------RQC-CV
GT-----------------ELVDWQMQQS-------------------------------
---SCVH--------TRIQAVGMW---QVLLEEGVLN-----------------------
--------------H------------------------VDQ-ELTFQDKY-LFYRFLDD
-------EQED----AP-L---
--PT
EKEA--RESNEEL------QDTLLLLSQIGPDAH-------------MRMILRKQPGQRT
ADDLE-----IIYEELLHIKALSHLS-TTVKKELA-GVLIFESHAKAGTVL---------
--FNQGEEG--TSWYIIL
KGSVNVVIYGK-----GV-------------------------------VCTLHEG-DDF
GKLAL-----------VNDAPRA---ASIVLREDNCHFLRVDKEDFNR------ILKD--
---V--------------

------------------------EANTVRLKEHYQDVLVLEK-----SPS-S----NST
TS----SS-HY------------------------------------K-YTVMSGTPEKI
LEHFLEM------------MRLDS-HLN--------------ES------DPALD---D-
FVLMHCV--FI---------------PNSQLCPVLM-----A------------------
--------H-----------Y-------HAEASQ---GSEQERLDYTLNNKRRVIRLVLQ
WASVHGDHLQEE-DASLAFLE----EFFVS--VSDDA--RVIPALK---DQ-LPELEKIV
KNT---SDD--AR---------SSQQK---------------------------------
---HKV--------LLRQFSMGD-EKLQ-KRQ----------------------------

-----AIKSNDE--IL-----------------------------FK-------------
VYCSDHTYTTIRVPMAASVREVIS--------AVADK-----------LGSA-ED--LLL
VNLSSA----------------------GDKVV------LKPNDISVFSTLSINGRLFVC
PGDQ---L-DSLT-PLPEQ---------------------------EGP------STGSL
SS--FELMSSKDLAYQMTLYDWELFHCVHEHELIY-------YTFGRQNF-KK-TTANMD

LFLRR---------FNEIQLWV-----ITEICLCAQLSKR------VQLLKKFIKIAAH-
--------------CKEYKNLNSFFAIIM-GMSNPAVSRLSQTWE---------------
-------KLPSKFKKFYSE-FESLM--------------------------DPSRNH---
-RAYRL----TVAK-------LDPPII-PFMP----------------------------
LLIKDMTFTHDGNK--TF-ID-----------------------------S-LVNFE-KM
-------RM---IANTVRIVRYCRS--L-------------------------PFSAE--
-P------------------SQTSKNH-----P----------------DVRS-----YV
RQLTVIDNQRTL---SQFSHRL
-----------EPRR----------T----------------------------------

>Rapgef4_[Sarcophilus_harrisii]_395519780
----------------------------MVAA-------HA------AH--------SSS
SAEWIA---CLDK-----------RPLE-----RSSEDVDIIFTRLKEVKAFEKFHPNLL
HQICLCGY--YENLE--KGITLFRQGDIGTNWYAVLAGSLDVKVSE-TSSHQDA------
------VTICTLGIGTAFGESILDNT-PRHATIV-TRECSELL-----------------
----------------------------RIEQKD-------FKALW---EKYRQYMAG-L
LAPPYG--VMETGA--N-N--------DRMPDKE--------------------------
------------------N---
---VSSSN--------------
----------L---

----CSA--------SKN-----------------CN-----------------------
--------------K------------------------VKN-NNNFTSR----------
----------E----SP-L---

---------------------SFFI------------------------------PGQRT
VDDLE-----IIYEELIHIKALSHLS-TTVKRELA-GVLIFESHAKGGTVL---------
--FNQGEEG--TSWYIIL
KGSVNVVIYGK-----GV-------------------------------VCTLHEG-DDF
GKLAL-----------VNDAPRA---ASIVLREDNCHFLRVDKEDFNR------ILRD--
---V--------------

------------------------EANTVRLKEHDQDVLVLEK-----VPA-GNRTSNQG
NS----QP-QQ------------------------------------K-YTVMSGTPEKI
LEHFLET------------IRLEQ-SLN--------------EV-----TDSVLN---D-
FIMMHCV--FM---------------PNSQLCPALV-----A------------------
--------H-----------Y-------HAQPSQ---GTEQDKMDYALNNKRRVIRLVLQ
WATLYGDLLQED-EVAMAFLE----EFYVS--VSDDS--RLIAALK---EQ-LPELEKTV
KQI---SED--AK---------APQKK---------------------------------
---HKV--------LLQQFNPVE-DKAQ-KRQ----------------------------

-----PVRGSDE--IL-----------------------------FK-------------
VYCLDHTYTTIRVPVSASVKEVIS--------AVADK-----------LGSG-EG--LII
VKMSSG----------------------GEKVV------LKPNDVSVFTTLTVNGRLFAC
PREQ---F-DSLT-PLPEQ---------------------------EGP------TTGTV
GT--FELMSSKDLAYQMTIYDWELFNCVHELELIY-------HTFGRHNF-KK-TTANLD
LFLRR---------FNEIQFWV-----VTEICLCAQLSKR------VQLLKKFIKIAAH-
--------------CKEYKNLNSFFAIVM-GLSNVAVSRLALTWE---------------
-------KLPSKFKKIYAE-FESLM--------------------------DPSRNH---
-RAYRL----TVAK-------LDPPLI-PFMP----------------------------
LLIKDMTFTHEGNK--TF-ID-----------------------------N-LVNFE-KM
-------RM---IANTVRTVRYCRS--Q-------------------------PFNPD--
-A------------------ALANKNH-----Q----------------DIRG-----YV
RQLNVIDNQRTL---SQMSHRL
-----------EPRR----------A----------------------------------

>Rapgef4_[Sarcoptes_scabiei]_934156037
-----------------------------MLA----------------------------
--EWIKS---LDK-----------KTTE-----RTDEDLEIIYKKLKTLKLFRRVHPSVI
QQFCFVAI--IEHIE--KGVMLYKQGDEALNWYLLVYGSLDVQITH-TGKRKDV------
------VTVCTLGSGTAFGEYVLNDG-LHSVSVV-SNEPCTLLRVPKQSFRDIWQKS---
--SQ--FM--

-------EEII--T
PS-F---------------SLPDVEPN------I---YP---------------------
----------------------------S-------------------------------

--DE-MNSFLDRY-AFYRFCFD
DVID---ERMQ------TLL--
-NLF----------------QQ----------------------------------NIPL
DDNR----IYDYLY------ENLTFLLHLAPQAT-------------LRQILRKRPENRT
EDEID-----IVYEEIMNIKAFSHLS-NSVKKELS-GVIAFESYEQKSTIW---------

--SVNVVIASK-----GI-------------------------------VCTLHEG-DDF
GKLAL-----------VNEAPRA---ATIITNEPDCQFLRVDKVHFDR------ILKD--

-----------------------NEASIVRLKECGKEVLILQK-----MSI--KQLKNEG

QP------SHF------------------------------------K-YMVMAGTPEKV
IDYLLETRMNNNN-------NSEEQFNKEN----------------------LFE---D-
FLLTFPI--FI---------------TWKQMTNSLL-----RHY----------------
------------------------------KIDEL---NFRQMEDYIIDNKKRVTRFIML
WYRISQDSFLNS-QTTGHFLD----ELIDL--LDKDN-------LKHN-NQ---------
--F---------EA--
----------------------E-------------------------------------
-----------------L--
----NSLK--

-----SIKETKE---I--
VYCADHTYTTLKITMDTKAEIIKN--------QAAEKLN---------LDNRMDYS---L
VELKSD----------------------NERYV------YNETEYNVATTLSLNGRIFIS
HKDH---L-DALT-TLPEQE---------------------------GP------TIGSM
FK--LESFSSQEIAYFLTNSTWDLFTNVHPYEFIYV-------VFGRHNF--KAITSNLD
LCRRT---------FNELQYWA-----ITEVLLEKSLSRR------VQILKKLIKIAG--

>Rapgef4_[Schistosoma_haematobium]_844834006

---MFQ
HNESIIDRKTCLINNSLNMETINNQLFNNNNNHNHNYTSSSMRIDKSDKLPSINDCKLSI
PK-LYKAKITPDWIVTICSEIWTRLLSSSSPS--SSSFIL-DSNENNID-----HQI--S
GS-----------------KLVNYLMITE-------------------------------
------N------KTDRNLICGLW---QLLLDENAIEPIL--------------------
-------SLNNNNDNNDKS--------------------VNS--VEFYDRDDVFYICKCI
K--NKRLSIDE-ALIINEISLNNNQ----------------------------SNLEQNH
DLLK------LINNTSNDPMTQINLHNNNNISNDKSNQYINII-----------------

-NDH-----NHEIDGYHI---LIDKLYRLSPEAL-------------FRKILQKLPSNRT
ENELQ-----YVYQELLLVPALSSFS-ISVKQELC-KCLYYEVHENALDIV---------
--FYQGDPG--ISWYIIY
HGSVWVHINNQ-----GY-------------------------------ICLLNEG-DDF
GKLSL-----------ITDKPRA---ASILLAENNCHFLKLNKNDFNR------ILCN--
---V--------------

------------------------EANTVHLKNNNSDVLILER-----IPDNKLLIQLQN
NQ-LHNHSHHQI------------------NDNDNQLI---NKLTDHN-YSIIAGTIEYI
LYHLLENCL----NELNN--QLQSIINNQL---YP--------SYLYPHTPYNIDQTWQI
FFLTYKL--FT---------------TNNEILLFLY------KYL---------------
-----------------------------ATSSE--------------HGDSSVTATTTT
TTTTTTSTTSTT----ANYVK----SLDNQ-----DT-----TIVH--NEQYNPEMKSIP
NLLF--------DLHISPN--------------------------------TLP------
--------------LIQSIHTVT-------------------------------------
-----------------I--

-----------KIYIT------------------------------ELT-----------
------KQIYMTVPIEANVYEIKQL-------LITNQMIQS----NI-------NE-LKL
VEVFSK----------------------GDWII------YQDYECGIMFSLSSNSRLFLT
KESQ---I-QNLI-PLAEQLIVTMNFLELNDIDRIKNFNQHDHLL----LRKNRLMQTSI
RL--LDELTIDELATILSYCHVQLAICIHPQELLDYVIDN-----NNHNK-ANKPCSHIR
TLVKR---------FSLLHAWT-----ITQIVTTCNLTKR------VNIIKKLIKLANCL
LS-------------SPLYDLYSSFAIIL-GLQNSSITRLTHTWE---------------
-------RVPNRWRRLFNDTLLPLT--------------------------DPSKNH---
-RTARLWNNYNENNNYIMHNIINLPRL-PFLP----------------------------
LILKDLRFAEDANQ-----TDYHKTDNNNNNNNN---------------NNRLINFN-KM
-------RL---ISTSLRSWINCISGYEL-------------------------LHPYNQ
FID------------------LNNNNNNKINNSLINNIT--VQKLL---EVTNESKIFVL
EHLHCIDDPKII---TQLSLRL
-----------EPKK---

>Rapgef4_[Sinocyclocheilus_anshuiensis]_1025001496
---------------------------------MV-----A------AH----TSSHPSA
SAEWII---CLDK-----------RLEE-----RSGEDVDIILARLKSVKAFEKFHPALL
QQICLCGF--YECLE--KGITLYRQGDIGTNWYTVLSGSLDVKVSE-TANHQDA------
------VTICTLGIGTAFGESILDNT-PRHATIV-TREFSELL-----------------
----------------------------WIEQKD-------FRTLW---ENYRQCMAG-L
LVPPYG--VMESG---P-NA-------DRMPDKD--------------------------

```
------------------NPN-------NSSPGSSSKHLNKK------------------
-----SFIQSPSKLHP-E-SITQ------------------VPS----------------
-------EKIL-------------RA---------------------GKVLR-NTILSRA
PH-M----------------IRDRKYH------L-KTY----------------RQC-CM
GM-----------------ELVDWLMQHT-------------------------------
---SCVH--------SRVQAVGMW---QVLLEEGVLN-----------------------
--------------H------------------------VDQ-ELSFQDKY-LFYRFLDD
-------ETED----AP-L-----------------------------------------
----------------------------------------------------------PT
EEEK--KESDEEL------QDTLLLLSQIGPDAH-------------LRMILSKQPGQRT
VDDLE-----IIYEELLHIKALSHLS-TTVKRELA-GVLIFESHAKAGTVL---------
--------------------------------------------FNQGEEG--TSWYIIL
KGSINVVIYGK-----GV-------------------------------VCTLHEG-DDF
GKLAL-----------VNDAPRA---ASIVLREDNCHFLRVDKEDFNR------ILRD--
---------------------------------------------V--------------
------------------------------------------------------------
------------------------------------------------------------
------------------------EANTVRLKEHEEDVLVLQK-----T---N----NRS
SS----SS-PL------------------------------------K-YTVMSGTPEKI
LEHLLET------------MKLDS-QFT--------------ES------DPALD---D-
FLLMHCV--FV---------------PNSQLCPLLM-----A------------------
--------H-----------Y-------HAQPSQ---GSEQERMDYALNNKRRVIRLVLQ
WATINTDHLQEE-DSSFNFLQ----EFNET--VCEDS--RLIPALK---DQ-LPELEKII
KQN---CED--GR---------PSQKK---------------------------------
---HKV--------LLRQFSTGN-DRLQ-KKQ----------------------------
------------------------------------------------------------
------------------------------------------------------------
------------------------------------------------------------
------------------------------------------------------------
------------------------------------------------------------
------------------------------------------------------------
-----PIRSTDE--IL-----------------------------FK-------------
VYCSDHTYSTIRVPVAASVREVIA--------AVTDK-----------LGSG-DD--LLL
IHLNSA----------------------GDKEL------LKPSDVSIFSSMSINGRLFVC
PREQ---I-DSLT-PLPEQ---------------------------EGP------SAGSM
ST--FELMSSKDLAHQMTLYDWELFDCVHEHELIY-------HTFGRQHF-KK-TTANLD
LFLRR---------FNEVQLWV-----VTEVCLCPTLSKR------VQLLKKFIKISAH-
--------------CKEFKNLNSFFAIIM-GLGNPAVCRLSQTWE---------------
-------KLPSKFKKFYGE-FENLM--------------------------DPSRNH---
-RAYRL----TVAK-------LEPPII-PFMP----------------------------
LLIKDMTFTHEGNK--TF-TD-----------------------------R-LVNFE-KM
-------RM---IANTVRIMRYCRS--Q-------------------------PFNQEA-
-P------------------QATGKSH-----Q----------------DVRS-----YV
RHISVIDNQRTL-----------------------------------------SQLSHRL
-----------EPRR----------T----------------------------------
------------------------------------------------------------
------------------------------------------------------------
------------------------------------------------------------
------------------------------------------------------------
------------------------------------------------------------
------------------------------------------------------------
------------------------------------------------------------
------------------------------------------------------------
------------------------------------------------------------
```


>Rapgef4_[Sinocyclocheilus_grahami]_1020489043
---M
VAAHTCSHPSASAEWII--C-LDKRPEE-----RSGEDVDIILSHLKSVK----------

--------------------AFEKFH-PALLQQIC--LCGLYECLEKSITL---------
--YRQGDIG--TNWYTVL
SGSLDVKVSETANHQDAVT------------------------------ICTLGIG-TAF
GE-SI-----------LDNTPRH---ATIVTRE-FSELLRIEQKDFRS------LWENYR
QCMAGLLVPPYGVMESGPNTDS---KYKHLSLFAQQ------------------------

>Rapgef4_[Sinocyclocheilus_rhinocerous]_1025414289

---MAG-L
LVPPYG--VMESG---P-NT-------DRMPDKE--------------------------
------------------NLN-------NSSPGSSSKHLNKK------------------
-----SFIQSPTKRPP-E-SITQ------------------VPS----------------
-------EKIL-------------RA---------------------GKVLR-NTILSRA
PH-I----------------IRDRKYH------L-KTY----------------RQC-CM
GM-----------------ELVDWLMQHT-------------------------------
---SCVH--------ARVQAVGMW---QVLLEEGVLN-----------------------
--------------H------------------------VDQ-ELSFQDKY-LFYRFLED
-------ETED----AP-L---
--PT
EEEK--KESDEEL------QDTLLLLSQIGPDAH-------------LRMILSKQPGQRT
ADDLE-----IIYEELLHIKALSLLS-TTVKRELA-GVLIFESHAKAGTVL---------
--FNQGEEG--TSWYIIL
KGSVNVVIYGK-----GV-------------------------------VCTLHEG-DDF
GKLAL-----------VNDAPRA---ASIVLREDNCHFLRVDKEDFNR------VLRD--
---V--------------

------------------------EANTVRLKEHEEDVLVLEK-----T---N----NRG
SS----SS-PL------------------------------------K-YTVMSGTPEKI
LEHLLET------------MKLDS-QFT--------------ES------DPALD---D-
FLLMHCV--FV---------------PNSQLCPLLM-----A------------------
--------H-----------Y-------HAQPSQ---GSEQERMDYSLNNKRRVIRLVLQ
WAAINTDHLQEE-DSSFNFLQ----EFYET--VCEDS--RLIPALK---DQ-LPELEKII
KQN---CDD--GR---------PSQKK---------------------------------
---HKV--------LLRQFSTGN-DRLQ-KKQ----------------------------

-----PIKSTDE--IL-----------------------------FK-------------
VYCSDHTYSTIRVPVAASVREVIA--------AVTDK-----------LGSG-DD--LLL
IHLNSA----------------------GDKEL------LKPADVSIFSSLSINGRLFVC
PRDQ---I-DSLT-PLPEQ---------------------------EGP------SAGSM
ST--FELMSSKDLAHQMTLYDWELFDCVHEHELIY-------HTFGRQHF-KK-TTANLD

```
LFLRR---------FNEVQLWV-----VTEVCLCLTLSKR------VQLLKKFIKIAAH-
--------------CKEFKNLNSFFAIIM-GLGNPAVCRLSHTWE---------------
-------KLPSKFKKFYGE-FENLM--------------------------DPSRNH---
-RAYRL----TVAK-------LEPPII-PFMP----------------------------
LLIKDMTFTHEGNK--TF-TD-----------------------------R-LVNFE-KM
-------RM---IANTVRIMRYCRS--Q-------------------------PFNQEA-
-P------------------QATGKSH-----Q----------------DVRS-----YM
RHISVIDNQRTL-----------------------------------------SQLSHRL
-----------EPRR----------T----------------------------------
------------------------------------------------------------
------------------------------------------------------------
------------------------------------------------------------
------------------------------------------------------------
------------------------------------------------------------
------------------------------------------------------------
------------------------------------------------------------
------------------------------------------------------------
------------------------------------------------------------
------------------------------------------------------------
---
>Rapgef4_[Sorex_araneus]_505771648
------------------------------------------------------------
------------------------------------------------------------
------------------------------------------------------------
------------------------------------------------------------
-------------------------------------------------------MAG-L
LAPPYG--VMETGS--N-N--------DRIPDKE--------------------------
------------------N-----------------------------------------
-----------------------------------------VPS----------------
-------EKIL-------------RA---------------------GKILR-NAILSRA
PH-M----------------IRDRKYH------L-KTY----------------RQC-CV
GT-----------------ELVDWMMQQT-------------------------------
---PCVH--------SRTQAVGMW---QVLVEDGVLN-----------------------
--------------H------------------------VDQ-EHHFQDKY-LFYRFLDD
-------EHED----AP-L-----------------------------------------
----------------------------------------------------------PT
EEEK--KECDEEL------QDTLLLLSQMGPDAH-------------MRMILRKPPGQRT
LDDLE-----IIYEELLHIKALSHLS-TTVKRELA-GVLIFESHAKGGTVL---------
--------------------------------------------FNQGEEG--TSWYIIL
KGSVNVVIYGK-----GV-------------------------------VCTLHEG-DDF
GKLAL-----------VNDAPRA---ASIVLREDNCHFLRVDKEDFNR------ILRD--
---------------------------------------------V--------------
------------------------------------------------------------
------------------------------------------------------------
------------------------EANTVRLKEHDQDVLVLEK-----VPA-GNRASNQG
NS----QP-QQ------------------------------------K-YTVMSGTPEKI
LEHFLET------------IRLEP-ALI--------------EA-----TDSVLN---D-
FVMMHCV--FM---------------PNAQLCPALV-----A------------------
--------H-----------Y-------HAQPSQ---GTEQEKMDYALNNKRRVIRLVLQ
WATMYGDLLQED-EVAMAFLE----EFYVS--VSDDA--RMIAALK---DQ-LPELEKIV
KQI---LED--AK---------APQKK---------------------------------
---HKV--------LLQQFNTGD-ERTQ-KRL----------------------------
------------------------------------------------------------
------------------------------------------------------------
```


-----PVRGSDE--VL-----------------------------FK-------------
VYCLDHTYTTIRVPVAASVKEVIS--------AVADK-----------LGSG-EG--LIV
VKMSSG----------------------GEKVV------LKPNDVSIFTTLTINGRLFAC
PREQ---F-DSLT-PLPEQ---------------------------EGP------TVGTV
GT--FELMSSKDLAYQMTIYDWELFNCVHELELIY-------HTFGRHNF-KK-TTANLD
LFLRR---------FNEIQFWV-----VTEICLCSQLSKR------VQLLKKFIKIAAH-
--------------CKEYKNLNSFFAIVM-GLSNVAVSRLALTWE---------------
-------KLPSKFKKFFAE-FESLM--------------------------DPSRNH---
-RAYRL----TVAK-------LDPPLT-PFMP----------------------------
LLIKDMTFTHEGNK--TF-VD-----------------------------N-LVNFE-KM
-------RM---IANTARTVRYCRS--Q-------------------------PFNPD--
-A------------------AQANKNH-----Q----------------DVRS-----YV
RQLNVIDNQRTL---SQMSHRL
-----------EPRR----------P----------------------------------

>Rapgef4_[Stegastes_partitus]_657542364

---MAG-L
LAPPYG--VME--S----SA-------DRMPDKE--------------------------
------------------NIS-------NNSFVSVSKHQNKN------------------
-----SFIDSP---AKPH-PKCIS----------------QVPS----------------
-------EKIL-------------HA---------------------GKILR-NAILSRA
PH-M----------------IRDRKYH------L-KTY----------------KQC-CV
GT-----------------ELVDWQMQQS-------------------------------
---SCVH--------SRVQAVGMW---QVLLEEGVLN-----------------------
--------------H------------------------VDQ-ELSFQDKY-LFYRFLDD
-------EQED----AP-L---
--PS
EEER--KESQEEL------QDTLLLLSQIGPDAH-------------MRMILKKHPSERT
ADDLE-----IIYEELLHIKALSHLS-TTVKRELA-GVLIFESHAKAGTVL---------
--FNQGEEG--TSWYIIL
KGSVNVVIYGK-----GV-------------------------------VCTLHEG-DDF
GKLAL-----------VNDAPRA---ASIVLREDNCHFLRVDKEDFNR------ILRD--
---V--------------

------------------------EANTVRLKEHGLDVLVLEK-----SLS-SSRTSIQG

```
SS----AS-HY------------------------------------K-YKVMSGTPEKI
LEHLLEM------------MRLDS-QFT--------------ES------DSALD---D-
FVLMHCV--FI---------------PNSQLCPVLL-----A------------------
--------H-----------Y-------HAQASQ---GSDQEKLDYTLNNKRRVIRLVMR
WAAVLGDQLQQE-EVSVAFLE----EFFLA--VSNDA--KVLPTLR---DQ-LTELERTV
KRK---AED--AR---------SLQKK---------------------------------
---HKI--------LLRQFSMGD-EKLQ-KRQ----------------------------
------------------------------------------------------------
------------------------------------------------------------
------------------------------------------------------------
------------------------------------------------------------
------------------------------------------------------------
------------------------------------------------------------
-----PIKSNDE--IL-----------------------------FK-------------
VYCCDHTYTTIRVSVATSVREVIG--------AVADK-----------LGSA-ED--LLL
VNLSSA----------------------GEKSV------FKPNDVSVFSTLSINGRLFVC
RRDQ---L-DSLT-PLPEQ---------------------------EGP------STGSL
AT--FELMSSKDVAYHMTSYDWELFHCVHELELIY-------HTFGRQYV-KK-TTVNLD
LFLRR---------FNEIQFWV-----ITEMCLCSQLSKR------VQLLKKFIKIAAH-
--------------CKEYRNLNAFFAVIM-GLSNPAVSRLSQTWE---------------
-------KLPSKFKKFYVE-FENLM--------------------------DPSRNH---
-RAYRL----TVAK-------LEPPII-PFMP----------------------------
LLIKDMTFTHEGNK--TF-ID-----------------------------N-LVNFE-KM
-------RM---IANTVKIVRYCRS--Q-------------------------TFSPD--
-S------------------PLASKNH-----P----------------DVWS-----YV
RQLSVIDNQRTL-----------------------------------------TQLSHEL
-----------EPRR----------S----------------------------------
------------------------------------------------------------
------------------------------------------------------------
------------------------------------------------------------
------------------------------------------------------------
------------------------------------------------------------
------------------------------------------------------------
------------------------------------------------------------
------------------------------------------------------------
------------------------------------------------------------
------------------------------------------------------------
---
>Rapgef4_[Stegodyphus_mimosarum]_675363725
------------------------------------------------------------
------------------------------------------------------------
------------------------------------------------------------
------------------------------------------------------------
------------------------------------------------------------
------------------------------------------------------------
------------------------------------------------------------
------------------------------------------------------------
------------------------------------------------------------
------------------------------------------------------------
--------------------MVDWLMQQG-------------------------------
AVYVRIH--------SRSQAVGMW---QALMEEGVIT-----------------------
--------------H------------------------VTN-EHQFKDKY-LFYRFRED
-------QQEG----VP-P-----------------------------------------
----------------------------------------------------------PT
```

FEDK--QQAEAEL------PETLGLLLQLAPDAI-------------FRMILRKPLGDRS
TEDLE-----IIYEELLHIKALSHLS-NSVKRELA-GVLVFESHPKAGTVL---------
--FNQGDEG--KSWFIIL
RGSVNVVIYGK-----GI-------------------------------VCSLHEG-DDF
GKLAL-----------VNDAPRA---ATIITRENNCHFLRVDKEDFNN------IIRD--
---V--------------

------------------------EANTVRLKEHGQDVLLLQK-----IPT-----DGRA
AD--GTHS-HY------------------------------------K-YMVMAGTPQKM
LEHLLETRIDQRNED------AS---------------------------DTFLE---D-
FLLTHVI--FM---------------PSHQLCPELM-----R------------------
--------HYRIDAAN-----------------------SRQEKEFIVANKKRVLQFVQI
WANLIRDAFFED-ASIAAFLQ----ELQEA--VAEDC--KKY--G--LHEE-LSAISKII
EKK-QKFDEEVTG---------VC-RR---------------------------------
---WKVGAYGQIRLLLASESQDE-ERTE-FK-----------------------------

------------------------------R-----------------------------

-----CIKASDE--VV-----------------------------FR-------------
VYCADHTYTTIKTPVSATAEAIKR--------SAAEK-----------LGFK-DD--LLL
VEVKST----------------------SERVI------FKDSEVSVPTGLSVNGRIFLT
PADQ---V-DLLT-PLPEQ---------------------------DGP------TDGTG
VI--LETLNSLDIAYNMTIYEWDLFSCVHEYELIY-------QVFGRHQF-RK-IMSNLD
VFLRR---------FNEVQFWV-----VTEMTLATTLSRR------VALLRKFIKIAAH-
--------------CREYQNMNSFFAIVM-GLSNVAVSRMSQTWE---------------
-------KLPSKLKRTFAD-FEALI--------------------------DPSRNH---
-RRYRL----TVAK-------MVPPII-PFMP----------------------------
LLLKDMTFSHEGNK--TF-ME-----------------------------G-LLNFE-KM
-------HM---IAQTLRTIRHCRS--Q-------------------------PLVFDP-
-P------------------PG-NKSQ-----Q----------------DVRN-----YI
RNLRIIDNQKRL---TQLSDAL
-----------EPRR----------P----------------------------------

>Rapgef4_[Strongylocentrotus_purpuratus]_390363651

---------------------------------------MEML-----------------
----------------------------LSHRVI-------TVPPPPPKTALLHPESS-P
SVDSQGR------S--RRSSRK-----SRSPSPQ--------------------------

-------------------------------------RTSPA------------------
-----RTHDGLDPIHPAL-PIVK------------------VPS----------------
-------DKQS-------------HT---------------------GTIFR-TLILAKS
PN-L----------------IKDRKNN------L-RTY----------------RKC-MV
GS-----------------EMVDWLMQQG-------------------------------
-QMTLVR--------SRPQAIGMW---QALLEEGVIT-----------------------
--------------E------------------------VNR-EHPFQDAE-HQYRFRED
-------DLGI----TS-T---
--PT
SSQK--KAAEEEL------PEIVGILAKVAPDAM-------------MRLILRKPPQERS
VDDLE-----IIYEELLHIKALAHLS-GMVKRQLT-SVLVFECHEKAGTVL---------
--FHQGDEG--RSWFIIL
RGSVNVVKYGK-----GV-------------------------------VCTLHDG-DDF
GKLAL-----------VNDAPRA---ASIVLREDNCQFLRVDKYDFNR------ILRD--
---V--------------

------------------------EANTVRLKEHGQDSLVLEK-----IPT-STQHSNNN
KH-HH---VSY------------------------------------R-YSVMAGVPEKM
LEHLLETRI---------DSKYDP-------------------------TDTFLE---D-
FLLTHIV--FM---------------PSEKLCPALL-----Q------------------
--------H-----------YH-----SERTSSR---GPEQETIDHIVNNKRRVVQLVCL
WHVVAGYAFKQD-TTIMQFLE----TLFSA--VCDDC--PAYPSLQ---DP-LASLEQVL
KAV-KSTKD-SGS---------HTWQV---------------------------------
---QTQ--------VLSESRTHQ-EDEL-RRT----------------------------

-----PIKAKDE--TI-----------------------------FK-------------
VYCADHTYTTLRVHMDTSVTNIIQ--------TVSEK-----------MCLG-ND--LVL
CEVKSS----------------------GERLS------FHQKDVCIATSLSCNGRIFIT
LRDH---L-DALT-PLPEQ---------------------------EGP------SEGSV
SF--LEPTSSRELAYQMTMYDWQLFLCTHECEFIY-------HTFGRHKY-RR-ITANLD
VFLRR---------FNEVQSWI-----VTELCLTSHISKR------VHLLKKFIKIAAH-
--------------CKEYQNMNSFYAIIM-GLSHMSVSRLAQTWD---------------
-------KLPNKLKRVFSD-FEALM--------------------------DPSRNH---
-RVYRL----ALSK-------MRPPII-PFMP----------------------------
LLIKDMLFTHEGNK--TY-FE-----------------------------G-LVNFE-KM
-------HL---VASIMRVVKYCRS--E-------------------------NFKLD--
-S------------------PPAVKNV-----K----------------EIVS-----YV
RNLQVIDNTKRL---MQLSYTL
-----------EPPK----------T----------------------------------

>Rapgef4_[Struthio_camelus_australis]_697502539

---MSG-L
LTPPYG--VMETGS--N-N--------DRMPDKE--------------------------
------------------NMP-------S-ALCQVSKNCNKT------------------
-----PLIEPHIPHRPTK-TITQ------------------VPS----------------
-------EKIL-------------RA---------------------GKILR-NAILSRA
PH-M----------------IRDRKYH------L-KTY----------------RQC-CV
GT-----------------ELVDWMMQQS-------------------------------
---PCVH--------SRTQAIGMW---QVLLEEGVLN-----------------------
--------------H------------------------VDQ-EHHFQDKY-LFYRFLDD
-------EHED----AP-L---
--PT
EEEK--KECDEEL------QDTMLLLSQIGPDAH-------------MRMILRKPPGQRT
VDDLE-----IIYEELLHIKALSHLS-TTVKRELA-GVLIFESHPKAGTVL---------
--FNQGEEG--TSWYIIL
KGSVNVVIYGK-----GV-------------------------------VCTLHEG-DDF
GKLAL-----------VNDAPRA---ASIVLREDNCHFLRVDKEDFNR------ILRD--
---V--------------

------------------------EANTVRLKEHDQDVLVLEK-----IPA-GNRVSNQG
NS----QP-QH------------------------------------K-YIVMSGTPEKI
LEHFLET------------MRLEA-TLN--------------EA-----TDSVLN---D-
FIMMHCV--FM---------------PNSQLCPALM-----A------------------
--------H-----------Y-------HAQPSQ---GTEQEKMDYALNNKRRVIRLVLQ
WAALYGDLLQED-EAAMAFLE----EFYVS--VSDDT--RMIAALK---EQ-LLELEKIV
KQV---SEE--PK---------APQKK---------------------------------
---HKV--------LLQLFNTSD-DRAQ-KRQ----------------------------

-----PIRGSDD--VL-----------------------------FK-------------
VYCIDQTYTTIRVPVSSSVKEVIS--------AVADK-----------LGSG-EG--LIL
VKMSSG----------------------GEKVV------LKPHDVSVFTTLSVNGRLFAC
PRDQ---F-DSLA-PLPEQ---------------------------EGP------SAGTV
GT--FELMSSKDLAYQMTIYDWELFNCVHELELIY-------HTFGRHNF-KK-TTANLD
LFLRR---------FNEIQFWV-----VTEICLCSQLSKR------VQLLKKYIKIAAH-
--------------CKEYKNLNSFFAIIM-GLSNVAVSRLSLTWE---------------
-------KLPSKFKKIYAE-FESLM--------------------------DPSRNH---
-RAYRL----TVAK-------LDPPII-PFMP----------------------------
LLIKDMTFTHEGNK--TL-TD-----------------------------N-LVNFE-KM
-------RM---IANTVRTVKFCRN--Q-------------------------SFNPD--
-A------------------ALANKNH-----Q----------------DVRS-----YV
RQLNVIDNQRTL---SQMSHRL
-----------EPRR----------A----------------------------------

>Rapgef4_[Sturnus_vulgaris]_959065034
----------------------------MVAA-------HA------SH--------SSS
SGEWIA---CLDK-----------RPLE-----RSSEDVDIIFTRLKEVKAFEKFHPNLL
QQICLCGY--YENLE--KGITLFRQGDIGTNWYAVLTGSLDVKVSD-TSNHQDA------
------VTICTLGIGTAFGESILDNT-PRHATIV-TREHSELL-----------------
----------------------------RIEQKD-------FKALW---EKYRQYMSG-L
LTPPYG--VMETGS--N-N--------DRMPDKD--------------------------
------------------SMS-------SSALCQVSKNCSKT------------------
-----PLIDPHISHHPTK-IITQ------------------VPS----------------
-------EKIL-------------RA---------------------GKILR-NTILSRA
PH-M----------------IRDRKYH------L-KTY----------------RQC-CV
GT-----------------ELVDWMMQQS-------------------------------
---PCVH--------LRTQAVGMW---QVLLEEGVLN-----------------------
--------------H------------------------VDQ-EHHFQDKY-LFYRFLDD
-------ERED----AP-L---
--PT
EEEK--KECDEEL------QDTMLLLSQIGPDAH-------------MRMILRKPPGQRT
VDDLE-----FIYEELLHIKALSHLS-TTVKRELA-GVLIFESHPKAGTVL---------
--FNQGEEG--TSWYIIL
KGSVNVVIYGK-----GV-------------------------------VCTLHEG-DDF
GKLAL-----------VNDAPRA---ASIVLREDNCHFLRVDKEDFNR------ILRD--
---V--------------

------------------------EANTVRLKEHDQDVLVLEK-----IPA-GNRISNQG
NS----QP-QH------------------------------------K-YIVMSGTPEKI
LEHFLET------------MRLEA-TLN--------------EA-----TDSVLN---D-
FIMMHCV--FM---------------PNSQLCPALM-----A------------------
--------H-----------Y-------HAQPSQ---GTEQEKMDYALNNKRRVIRLVLQ
WAALYGDLLQED-EAAMAFLE----EFYVS--VSDDT--RMIAALK---EQ-LPELEKIV
KQI---SEE--PK---------APQKK---------------------------------
---HKV--------LLQLFNTSD-DRAQ-KRQ----------------------------

-----PIRGSDE--VL-----------------------------FK-------------
VYCIDQTYTTIRVPVSSSVKEVIS--------AVADK-----------LGSG-EG--LII
VKMSSG----------------------GEKVV------LKPHDVSVFTTLSVNGRLFAC
PRDQ---F-DSLA-PLPEQ---------------------------EGP------STGTV
GT--FELMSSKDLAHQMTIYDWELFNCVHELELIY-------HTFGRHNF-KK-TTANLD

LFLRR---------FNEIQFWV-----VTEICLCSQLSKR------VQLLKKYIKIAAH-
--------------CKEYKNLNSFFAIIM-GLSNVAVSRLSLTWE---------------
-------KLPSKFKKIYAE-FESLM--------------------------DPSRNH---
-RAYRL----TVAK-------LDPPII-PFMP----------------------------
LLIKDMTFTHEGNK--TF-TD-----------------------------N-LVNFE-KM
-------RM---IANTVRTVKFCRS--Q-------------------------SFNPD--
-A------------------ALTNKNH-----Q----------------DVRS-----YV
RQLNVIDNQRTL---SQMSHRL
-----------EPRR----------A----------------------------------

>Rapgef4_[Sus_scrofa]_927210429
----------------------------M-------------------------------

--PS---KKYRQYMAG-L
LAPPYG--VMETGS--N-N--------DRLPDKE--------------------------
------------------N----------------------T------------------
-----PLIEPHVPLRPAN-TITK------------------VPS----------------
-------EKIL-------------RA---------------------GKILR-NAILSRA
PH-M----------------IRDRKYH------L-KTY----------------RQC-CV
GT-----------------ELVDWMLQQT-------------------------------
---PCVH--------SRTQAVGMW---QVLVEDGVLN-----------------------
--------------H------------------------VDQ-EHHFQDKY-LFYRFLDD
-------EHED----AP-L---
--PT
EEEK--KECDEEL------QDTLLLLSQMGPDAH-------------MRMILRKPPGQRT
VDDLE-----IIYEELLHIKALSHLS-TTVKRELA-GVLIFESHAKGGTVL---------
--FNQGEEG--TSWYIIL
KGSVNVVIYGK-----GV-------------------------------VCTLHEG-DDF
GKLAL-----------VNDAPRA---ASIVLREDNCHFLRVDKEDFNR------ILRD--
---V--------------

------------------------EANTVRLKEHDQDVLVLEK-----VPA-GNRASNQG
NS----QP-QQ------------------------------------K-YTVMSGTPEKI
LEHFLET------------IRLEP-ALN--------------EA-----TESVLN---D-
FVMMHCV--FM---------------PNTQLCPALM-----A------------------
--------H-----------Y-------HAQPSQ---GTEQEKMDYALNNKRRVIRLVLQ
WAAMYGDVLQED-DVAMAFLE----EFYVS--VSDDA--RMIAALK---EQ-LPELEKTV
KQI---SED--AK---------APQKK---------------------------------
---HKV--------LLQQFNTGD-ERSQ-KRQ----------------------------

-----PIRGSDE--VL-----------------------------FK-------------
VYCMDHTYTTIRVPVAASVKEVLS--------AVADK-----------LGSG-EG--LII
VKMSSG----------------------GEKVV------LKPNDVSVFTTLTINGRLFAC
PREQ---F-DSLT-PLPEQ---------------------------EGP------TVGTV
GT--FELMSSKDLAYQMTIYDWELFNCVHELELIY-------HTFGRHHF-KK-TTANLD
LFLRR---------FNEIQFWV-----VTEICLCSQPSKR------VQLLKKFIKIAAH-
--------------CKEYKNLNSFFAIVM-GLSNVAVSRLALTWE---------------
-------KLPSKFKKFYAE-FESLM--------------------------DPSRNH---
-RAYRL----TVAK-------LDPPLI-PFMP----------------------------
LLIKDMTFTHEGNK--TF-ID-----------------------------S-LVNFE-KM
-------RM---IANTARTVRYCRS--Q-------------------------PFNPD--
-A------------------AQANKNH-----Q----------------DVRS-----YV
RQLNVIDNQRTL---SQMSHRL
-----------EPRR----------P----------------------------------

>Rapgef4_[Takifugu_rubripes]_410896946
---------------------------------MV-----A------AH---PSSHSSES
-AQWII---CLDK-----------RPAE-----RSGEDVDIILARLKKAKAFEHFHPSLL
HQICLCGF--YECLE--KGITLYRQGDIGTSWYAVLSGSLDVKVSE-TSYHQDA------
------VVICTLGTGTAFGESILDNT-PRRATIV-TREFTELL-----------------
----------------------------RIEQRE-------FRCLWE---RYHHCMAG-L
LVPSYG--VME--S--ACSA-------DRMPNKE--------------------------
------------------NVS-------DNPFVSIGKHQNK-------------------
---VPS----------------
-------EDIL-------------RA---------------------GKILR-NAILSRA
PH-M----------------IRDRKYH------L-KTY----------------RQC-CV
GT-----------------ELVDWQMQQS-------------------------------
---SCVR--------SRLQAVGMW---QVLLEEGVLN-----------------------
--------------H------------------------VDQ-ELSFQDKY-LFYRFLDD
-------EQED----AL-F---
--PS
EEER--RESQEEL------QDTLLLLSKIGPDAH-------------MRMILRKHPSERT
ADDLE-----IIYEELLHIKALSHLS-TTVKRELA-GVLIFESHAKAGTVL---------
--FSQGEEG--TSWYIIL
KGSVNVVIYGK-----GV-------------------------------VCTLHEG-DDF
GKLAL-----------VNDAPRA---ASIVLREDNCHFLRVDKEDFNR------ILRD--
---V--------------

------------------------EANTVRLKEHGQDVLVLER-----SLG-NSRSSIHG

AS----SS-QY------------------------------------K-YTIMSGTPEKI
LEHLLEM------------MRLDS-QFT--------------ES-G----NSALD---D-
FVLTHPV--FI---------------PNSQLCPVLM-----A------------------
--------H-----------Y-------HAQASQ---GCEQERMDYTLNNKRRVIRLVMR
WAAVHGDHLLEE-EDVVAFLQ----EFLVA--VSNDA--KVLPALR---EQ-LAKLEITV
KHY---TED--AR---------ISRKK---------------------------------
---HKV--------FLRQFSTGE-EKLQ-KRQ----------------------------

-----PIKSNDE--IL-----------------------------FK-------------
VYCCDNTYTTIRAPVAASVRELIS--------SLAEK-----------LGST-ED--LLL
VSLGSA----------------------GEKVI------FKPNDVSVFSTLSINGRLFVC
RRDE---L-DSLT-PLPEQ---------------------------VEP------SAGLS
SN--FELMSSKDVACHMTSYDWELFNCVHELELIY-------HTFGRQNI-QK-TTVNLD
LFLRR---------FNEIQFWV-----ITEMCLCSQLSKR------VQLLKKFIKIAAH-
--------------CKEYKNLNGFFAITM-GLSNPAVCRLNMTWE---------------
-------KLPSKFRKFYRE-FENLM--------------------------DPSRNH---
-RAYRL----TVAK-------LDPPII-PFMP----------------------------
LLIKDMTFTQEGNK--TF-ID-----------------------------T-LVNFE-KM
-------RM---IANTVKILRYCRS--Q-------------------------PFSPD--
-S------------------PQAGKSH-----S----------------DVRA-----YV
RQFNVIDNQRTL---SQLSHSL
-----------EPPR----------S----------------------------------

>Rapgef4_[Tetranychus_urticae]_1005954532

-----------------------------------MDDVDNIVGKLKLTRFFQRMHPSLL
QQICKHAI--VERLD--KSVVVFRQGDKAFNWYAVVTGSLDVQVNA---KAKEV------
------VTLCTLGVGTAFGESVLTGK-PHSVSII-TNEPCTLLRVRKDEFQEIWDQN---
--SH--LM---------------T---------D-------------------------I
VTPLVGLRCLKRASF---NKSLSI----RITSTNDNCN----S-----------------
-----------------TNQSSES----NNNSNNSSKNSS--------------------
--------NNNCNIITTK-NSTKSN--------------NSLDSLSYRFKSIQ-------
---RDQSVELL-------------QI---------------------GRILR-ALIIAHD
PS-M----------------IRDRKYN------VGLVY----------------RNC-LV
GS-----------------ELVDWLISAS------------------------------S
VTPKRIH--------SRSQAAAML---QVLLEENVLT-----------------------
--------------S------------------------PTG-DSQFWDKY-LFYRFIFD
G-EDESLRSED----LPSL---
--PS

FEGK--RRTESAL------VHCLTTLHSLAPEAN------------FRLILCKSPKERS
AEEVD-----LVYEELMHIKALSHLG-NSVRKELA-SVIAFEKHPRKGTVL---------
--FNQGDPG--KSWYIIL
KGTVNVVIVGK-----GV------------------------------VCTLCEG-DDF
GKLAL-----------VNDAPRA---ATIITNEDNCHFLRVDKDNFNR------ILRD--
---V--------------

------------------------EANTVHLKEHGRDVLLLRKMVPKVIPS-STSSTING
SS------HHYPPTAQPVTSGSPQPTVNPQVEQQKVTSKYSSSCSHYK-YMVLAGTPEKM
LEHLLETRIDVTSPPEIA-CALPS-SVQLR-------GGGPLEL-IYS-VDTFLE---D-
FVLTHIV--FL---------------KTDALCVYLL-----K-----------------
--------H-----------Y---------KIDL---LNTRQEKEFIIGNKKRVIRFVKI
WFNLISEVLHRD-SYVTTFVD----KLYNL--VKSDL--IKYEGFL--EDE-LALLESIC
KDR-KRFEDDLVL---------RGTQK---------------------------------
---WKNDLPGP----IRRLSTNG---LT-DNLIAFTNIPDDWL-----------------

------------------------LINLRFN-----------------------------

-----SIQPKDE--II----------------------------SR-------------
IYCADHTYTTLKMTIDSTAGAIKI--------AAADK-----------LGLNKEEGELIL
AEVKST----------------------GERVL------FDNQDVSIQTGLSVNGRIFVS
LIDH---I-DALT-PLPEQ----------------------------EGP------KIESF
SS-ALEDISSQDIAYYLTYYSWLLFQNVHEYEFIY-------HVFGRVKF-GH-ITANLD
LFLRL---------FNEIQYWV-----VTEIVLTTSLSKR------VQILRKFIKVAGL-
--------------CKEHQNLSIFFAITM-GLSNIAVSRLTQTWE---------------
-------RLPNKLKRTFSQ-YESLI--------------------------DPSRNH---
-RKYRV----YLSK-------LEAPII-PFTP----------------------------
LILKDMTFSHEGNK--TY-LE------------------------------NNLINFE-KM
-------VM---LSQSLRTFRFCRS--K-------------------------PMKLDS-
-L------------------INGSSNS-----NYSNRGIIPSTRKAALIKIDQ-----YI
KDLKVIDNQRLL---LHLSHSL
-----------EHRR----------I----------------------------------

>Rapgef4_[Tinamus_guttatus]_719773627

---MSG-L
LTPPYG--VMETGS--N-N--------DRMPDKE--------------------------

```
------------------NMS-------S-ALCQFSKNCNK-------------------
-----------------------------------------VPS----------------
-------EKIL-------------RA---------------------GKILR-NAILSRA
PH-M----------------IRDRKYH------L-KTY----------------RQC-CV
GT-----------------ELVDWMMQQS-------------------------------
---PCVH--------SRSQAIGMW---QVLLEEGVLN-----------------------
--------------H------------------------VDQ-ELHFQDKY-LFYRFLDD
-------EHED----AP-L-----------------------------------------
----------------------------------------------------------PT
EEEK--KECDEEL------QDTMLLLSQIGPDAH-------------MRMILRKPPGQRT
VDDLE-----IIYEELLHIKALSHLS-TTVKRELA-GVLIFESHPKAGTVL---------
--------------------------------------------FNQGEEG--TSWYIIL
KGSVNVVIYGK-----GV-------------------------------VCTLHEG-DDF
GKLAL-----------VNDAPRA---ASIVLREDNCHFLRVDKEDFNR------ILRD--
---------------------------------------------V--------------
------------------------------------------------------------
------------------------------------------------------------
------------------------EANTVRLKEHDQDVLVLEK-----IPA-GNRASNQG
NS----QP-QH------------------------------------K-YIVMSGTPEKI
LEHFLET------------MRLEA-TLN--------------EA-----TDSVLN---D-
FIMMHCV--FM---------------PNSQLCPALM-----A------------------
--------H-----------Y-------HAQPSQ---GTEQEKMDYALTNKRRVIRLVLQ
WAALYGDLLQED-EAAMAFLE----EFYVS--VSDDT--RMIAALK---DQ-LLELEKIV
KQA---SEE--PK---------APQKK---------------------------------
---HKV--------LLQLFNTSD-DRAQ-KRQ----------------------------
------------------------------------------------------------
------------------------------------------------------------
------------------------------------------------------------
------------------------------------------------------------
------------------------------------------------------------
------------------------------------------------------------
-----PIRGSDE--VL-----------------------------FK-------------
VYCIDQTYTTIRVPVSSSVKEVIS--------AVADK-----------LGSG-EG--LIL
VKMSSG----------------------GEKVV------LKPHDVSVFTTLSVNGRLFAC
PRDQ---F-DALA-PLPEQ---------------------------EGP------STGTV
GT--FELMSSKDLAYQMTIYDWDLFNCVHELELIY-------HTFGRHNF-KK-TTANLD
LFLRR---------FNEIQFWV-----VTEICLCSQLSKR------VQLLKKYIKIAAH-
--------------CKEYKNLNSFFAIIM-GLSNVAVSRLSLTWE---------------
-------KLPSKFKKIYAE-FESLM--------------------------DPSRNH---
-RAYRL----TVAK-------LDPPII-PFMP----------------------------
LLIKDMTFTHEGNK--TL-TD-----------------------------N-LVNFE-KM
-------RM---IANTVRTVKFCRS--Q-------------------------SFNPD--
-A------------------ALANKNH-----Q----------------DVRS-----YV
RQLNVIDNQRTL-----------------------------------------SQMSHRL
-----------EPRR----------A----------------------------------
------------------------------------------------------------
------------------------------------------------------------
------------------------------------------------------------
------------------------------------------------------------
------------------------------------------------------------
------------------------------------------------------------
------------------------------------------------------------
------------------------------------------------------------
------------------------------------------------------------
```


>Rapgef4_[Tribolium_castaneum]_642935500

-----------------------------------MAGTLEVRIIP-PESDTK------V
P-----VTLCTLGVGATFGESILQDL-PRDTTVV-TQTTCELLRVEQHDFKLIWE-----
KNKE--LMNDLV--------------S-NCKLKNGFGTAV-S-GS-----KG-VGMQQ-P
ISPPP------------------R--RSMSPDQPNPAE----------------------

--PITE--------------
-----------------SPSTIIGRI---------------------GWALR-TLLLSQN
T--C----------------LKDRKVS------G-R-L---------------VRRC-AP
GT-----------------ELVDWLLGLS-------------------------------
---SSIH--------TRAQAAGMW---QALLEEGVIS-----------------------
--------------H------------------------VSK-EQPFKDKC-FLYRFWQD
E---------E----GP-T---
---S---LPP
LEDV--ATAEEQI------QDSLGTLIHRGPDAV-------------LRMILRKQSHERT
PDDLE-----TIYEELLHIRALAHLS-NSVKRELS-SVIVFEAHPRAGTVL---------
--FHQGDEG--RSWYIIV
RGSVDVVIHGK-----GT-------------------------------VNTLHEG-DDF
GKLAL-----------INDAPRA---ATIVLRENNCHFLRVDKENFNR------ILRD--
---V--------------

------------------------EANTVRLKEHGKDVLILEK-----INT---------
------PTKQI--FSS---------------HFK---------------YTVMAGTPQKM
LEHLLET-------------RLDGRG---T-------MGGGDNFIITSAQDPFLD---D-
FLLTHIV--FI---------------PTHQLIAELD-----N------------------
--------YY---RIDS---------S-----N--------QDKEFVLACKRRVIQFVYR
WVTTIRHPVFED-DVAVEFLD----DLASE--LESDC--------I----Q-WNALQEEA
SLM---
---HHV--------MSQLRRYQE-DRKAHEGQK---------------------------
----WKLPP---CGQPIS--------------------------------LFSGNET---
----NRT---I---

------IMPQDD--II-----------------------------FR-------------
VYCADHTYCTLRLPVDTTAETIKI--------VAAEK-----------LKM-RSTDELLL
VEVKSN----------------------GERVT------FKDNDISIPTALSLNGRIFVS
PKDH---L-DALT-CLAEQ---------------------------EES------TQGID
A--DIELFSTKELAYYMTLFDWDLFWCVHEYELLY-------HTFGRHHF--GQITANLD
VFLRR---------FNEIQFWV-----VTEICMTTSLSKR------VALLRKFIKLAAY-
--------------CKEYQNLNAFCAIVM-GLSNVAVSRLSLTWE---------------
-------KLPSKFRKLYTE-FESLI--------------------------DPSRNH---
-RAYRV----SVGK-------LQPPVV-PFMP----------------------------
LLLKDMTFTHEGNK--TS-LD-----------------------------G-LVNFE-KM
-------HM---LAQTMRTIRFCRS--R------HLVL-----------------EPP--
----------------------SPKSE-----G----------------EVKS-----YI
SCLRVIDNQRVL---TSMSQKL
-----------EPRR----------S----------------------------------

>Rapgef4_[Trichechus_manatus_latirostris]_823389193
----------------------------MVAA-------HA------AH--------SSS
SAEWIA---CLDK-----------RPLE-----RSSEDVDIIFTRLKEVKAFEKFHPNLL
HQICLCGY--YENLE--KGITLFRQGDIGTNWYAVLAGSLDVKVSE-TSSHQDA------
------VTICTLGIGTAFGESILDNT-PRHATIV-TRECSELL-----------------
----------------------------RIEQKD-------FKALW---EKYRQYMAG-L
LAPPYG--VMETGS--N-N--------DRIPDKE--------------------------
------------------N---
---VPS----------------
-------EKIL-------------RA---------------------GKILR-NAILCRA
PH-M----------------IRDRKYH------L-KTY----------------RQC-CV
GT-----------------ELVDWMMQQT-------------------------------
---QCVH--------SRTQAVGMW---QVLLEDSVLN-----------------------
--------------H------------------------VDQ-EHHFQDKY-LFYRFLDD
-------EHED----AP-L---
--PT
EEEK--KECDEEL------QDTMLLLSQMGPDAH-------------MRMILRKPPGQRT
VDDLE-----IIYEELLHIKALSHLS-TTVKRDVC-GIAIFSSFL---CLV---------
--FNQGEEG--TSWYIIL
KGSVNVVIYGK-----GV-------------------------------VCTLHEG-DDF
GKLAL-----------VNDAPRA---ASIVLREDNCHFLRVDKEDFNR------ILRD--
---V--------------

------------------------EANTVRLKEHDQDVLVLEK-----VPA-GNRASNQG
NS----QP-QQ------------------------------------K-YTVMSGTPEKI
LEHFLET------------IRLEP-ALN--------------EA-----TDSVLN---D-
FIMMHCV--FM---------------PNTQLCPALV-----A------------------
--------H-----------Y-------HAQPSQ---GTEQEKMDYSLNNKRRVIRLVLQ
WAVMYGDLLQED-DVAMAFLE----EFYVS--VSDDA--RMIAALK---EQ-LPELEKIV
KQI---LEE--AK---------APRKK---------------------------------
---HKV--------LLQQFNTGD-ERAQ-KRQ----------------------------

-----PIRGSDE--VL-----------------------------FK-------------
VYCMDHTYTTIRVQVAASVKEVIS--------AVADK-----------LSSG-EG--LMI
VKMSSG----------------------GEKVV------LKPNDVSVFTTLTINGRLFAC
PREQ---F-DSLT-PLPEQ---------------------------EGP------TVGTV
GT--FELMSSKDLAYQMTIYDWELFNCVHELELIY-------HTFGRHNF-KK-TTANLD

LFLRR---------FNEIQFWV-----VTEICLCSQLSKR------VQLLKKFIKIAAH-
--------------CKECKNLNSFFAIVM-GLSNVAVSRLALTWE---------------
-------KLPSKFKKFYAE-FESLM--------------------------DPSRNH---
-RAYRL----TVAK-------LEPPLI-PFMP----------------------------
LLIKDMTFTHEGNK--TF-ID-----------------------------N-LVNFE-KM
-------RM---IANTARTVRYCRS--Q-------------------------PFNPD--
-A------------------AQANKNH-----Q----------------DVRS-----YV
RQLNVIDNQRTL---SQMSHRL
-----------EPRR----------P----------------------------------

>Rapgef4_[Trichinella_britovi]_954388359
----------MRNVICCKFDYFGRISVACKNLEWLFAV----SVTFEKEEAFI-------
------------IFYFI------------SIKKVSHADCNSVADRIRGVAAFEHFSQQLV
ELLASRG--LYEELD--AGIVLFCEGKPSSNWYALLDGSVDLYCYDPEKPS---------
----ELVKIKTLHSGCTFGEGALNKQ-LQPCTAI-VSQRSQFIRLDFSSLSLIWE-----
QYEN--ELMQNTVSLESIVKNKFVKAS-SSKMSKGVKQAD-SVSLSAFKSETSAFAAA-T
NYKPSKEDAVNCDPKQTAASVGEQ--MSLKANQPFPMSRGVVASDSSALVSSRAK-----
YSPLMDTTSSTSSFLSDTDQSTTDSQVSSKFSIQIKKKKKKKKKPEKRKGLKNNFQSFHS
IYCTMPADSSVDPQREVS-TLRKSSSVSDKLEVKNPATGIGDPMAEPANGITTNATATAR
PDDSQVNNNLIELKNLLTPDMQISWA---------------------SRVLR-SAMVVRS
PN-M----------------IRDRKFQ--------MTL---------YK------RC-MV
GT-----------------EMVDWLLRQS-------------------------------
---NSVV-------QNRMQAAGIW---QVLLEEGIIH-----------------------
--------------H------------------------VTH-EYPFLDKY-LFYRWVED
E------RSTD----AA-V---
---RALNLSD
DD---GIYSENDF------LLAISNLSIMGPDAL-------------FRMVLRKAPNERS
NEELE-----VVFEELLNIKALSNLS-TMVKRELA-TVIMFEQHQNAGTVL---------
--FRQGDQG--KSWYIIL
KGSVNVVIHGK-----GV-------------------------------VCTLQEG-DDF
GKLAL-----------VNDAPRA---ATIIILNENCCQFLRVDKHDFDR-----ILRD--
---V--------------

------------------------EANTVRLKEHGHDVLVLEK-----VPI---------
---------R---------------VNRSPIAYR---------------YSVMAGTPEKM
VEYLLET-------------RIDC---------------AKDE----DIADTFME---D-
FILTHLI--FM---------------PTNELCNALT-----I------------------
--------YYQQ-GCSSI---NGNSAL-----SE---SILTESVEHQLSFRKRVIAFLQA
WIQISGHMFYED-PVGAAFIE----EMYCA--ILEDS--------RDH-----PQLLPE-

--------------LTVVEKLMR-EREQALGSS---------------------------
----AR--------PPVI--------------------------------LSDNNFI-RE
L--PANSP--

```
------------------------------------------------------------
------------------------------------------------------------
------------------------------------------------------------
------------------------------------------------------------
------VLPFDL--C-----------------------------------------IVKV
NSSDSLRTSTLKIRVDQTCETIKR--------MASQK-----------LGLGN-GEGVEL
VEVKSN----------------------REKNI------FTPNDTGIPTSMSHNGRLFAC
FKDQ---V-ESLS-PLPEQ---------------------------AGP------SESVH
SV-ILETLTSTELAQHLLIYHWQLFINTHEYELLY-------HVVGRNLF-PGKVPVNLD
LLIRR---------FNELQFWV-----ITEILLCGSVSKR------AHVVKKFIKIALH-
--------------CKANQDLFSFFAITL-GLSNVAISRLSQTWE---------------
-------KVNAKFRKLFFE-FESLL--------------------------DPSRNH---
-RAYRL----LVAK-------MKPPTI-PFIP----------------------------
LLLKDLLFAHEGNK--TY-FD-----------------------------G-MVNFE-KM
-------HM---MAQTLRNHRMYKS--Q------MLQI-------------------D--
----------------------SSKIP-----T----------------DAQH-----LV
RNFRIIDDQRKF-----------------------------------------LRLSHLL
-----------EPKQ----------KDTT-------------------------------
------------------------------------------------------------
------------------------------------------------------------
------------------------------------------------------------
------------------------------------------------------------
------------------------------------------------------------
------------------------------------------------------------
------------------------------------------------------------
------------------------------------------------------------
------------------------------------------------------------
------------------------------------------------------------
---
>Rapgef4_[Trichinella_nativa]_954534912
----------MRNVICCKFDYFGRISVACKNLEWLFAV----SGDMLKKELYMSSPKSNS
RDEQAMPPKTKNIFKTALFAMAFKRACSKSIKKVSHADCNSVADRIRGVAAFEHFSQQLV
ELLASRG--LYEELD--AGIVLFCEGKPSSNWYALLDGSVDLYCYDPEKPS---------
----ELVKIKTLHSGCTFGEGALNKQ-LQPCTAI-VSQRSQFIRLDFSSLSLIWE-----
QYEN--ELMQNTVSLESIVKNKFVKAS-SSKMSKGVKQAD-SVSLSAFKSETSAFAAA-T
NYKPSKEDAVNCDPKQTAASVGEQ--MSLKANQPFPMSRGVVASDSSALLSSRAK-----
YSPLMDTTSSTSSFLSDTDQSTTDSQVSSKFSIQIKKKKKKKKKPERRKGLKNNFQSFHS
IYCTMPADSSVDPQREVS-TLRKSSSVSDKLEVKNPATSIGDPMAEPANGITTNATATAR
PDDSQVNNNLIELKNLLTPDMQISWA---------------------SRVLR-SAMVVRS
PN-M----------------IRDRKFQ--------MTL---------YK------RC-MV
GT-----------------EMVDWLLRQS-------------------------------
---NSVV-------QNRMQAAGIW---QVLLEEGIIH-----------------------
--------------H------------------------VTH-EYPFLDKY-LFYRWVED
E------RSTD----AA-V-----------------------------------------
-----------------------------------------------------RALNLSD
DD---GIYSENDF------LLAISNLSIMGPDAL-------------FRMVLRKAPNERS
NEELE-----VVFEELLNIKALSNLS-TMVKRELA-TVIMFEQHQNAGTVL---------
--------------------------------------------FRQGDQG--KSWYIIL
KGSVNVVIHGK-----GV-------------------------------VCTLQEG-DDF
GKLAL-----------VNDAPRA---ATIIILNENCCQFLRVDKHDFDR-----ILRD--
---------------------------------------------V--------------
------------------------------------------------------------
------------------------------------------------------------
------------------------EANTVRLKEHGHDVLVLEK-----VPI---------
```

---------R---------------VNRSPIAYR---------------YSVMAGTPEKM
VEYLLET-------------RIDC---------------AKDE----DIADTFME---D-
FILTHLI--FM---------------PTNELCNALT-----I------------------
--------YYQQ-GCSSI---NGNSAL-----SE---SILTESVEHQLSFRKRVIAFLQA
WIQISGHMFYED-PVGAAFIEVVVVEMYCA--ILEDS--------RDH-----PQLLPE-

--------------LTVVEKLMR-EREQALGSS---------------------------
----AR--------PPVI--------------------------------LSDNNFI-R-

--IVKV
NSSDSLRTSTLKIRVDQTCETIKR--------MASQK-----------LGLGN-GEGVEL
VEVKSN----------------------REKNI------FTPNYTGIPTSMSHNGRLFAC
FKDQ---V-ESLS-PLPEQ---------------------------AGP------SESVH
SV-ILETLTSTELAQHLLIYHWQLFINTHEYELLY-------HVVGRNLF-PGKVPVNLD
LLIRR---------FNELQFWV-----ITEILLCGSVSKR------AHVVKKFIKIALH-
--------------CKANQDLFSFFAITL-GLSNVAISRLSQTWE---------------
-------KVNAKFRKLFFE-FESLL--------------------------DPSRNH---
-RAYRL----LVAK-------MKPPTI-PFIP----------------------------
LLLKDLLFAHEGNK--TY-FD-----------------------------G--------M
-------HM---MAQTLRNHRMYKS--Q------MLQI-------------------D--
----------------------SSKIP-----T----------------DAQH-----LV
RNFRIIDDQRKF---LRLSHLL
-----------EPKQ----------KDTT-------------------------------

>Rapgef4_[Trichinella_papuae]_954590902
LQFQLCCLVIMRNVICCKFDYFGRISVACKNLKWLFAV----SVTFEKEEAFI-------
------------IFYFI------------SIKKVSHAEYNLVADRIRGVAAFEHFSQQLV
ELLASHG--LYEELD--AGIVLFCEGKPSSNWYALLDGSVDLYCYDPEKSL---------
----ELVKIKTLHSGCTFGEGALNKQ-RQPCTAI-VSQRSQFIRLDFSSLSLIWE-----
QYEN--ELMQNTVSLESIVKNKFVKAS-SSKMSKGVKQAD-SVSSSAFKSETSAFAAA-A
NYKPSKEEAVNCDPKQTTTSVGEQ--MPLKANQPFPTSRGVAASDSSALLSGRVK-----
YSPLMDTTSSASSFLSDTDQSTTDSQLSSKFSIKIKKKKKKK--PEKRKGPKNNFQPFHS
IYCTAPADSSVDPEREVNNTLRKSSSMDDKLEAKNPTSNIGDPVTEPANGITTNATATVR
PDDSQINNNLIELKNLLTPDMQISWA---------------------SRVLR-SAMVVRS
PN-M----------------IRDRKFQ--------MTL---------YK------RC-MV
GT-----------------EMVDWLLRQS-------------------------------
---NSIV-------QNRMQAAGIW---QVLLEEGIIH-----------------------
--------------H------------------------VTH-EYPFLDKY-LFYRWVED
E------RSTD----AA-V---
---RALNLSD

DD---GIYSENDF------LLAISNLSIMGPDAL------------FRMVLRKAPNERS
NEELE-----VVFEELLNIKALSNLS-TMVKRELA-TVIMFEQHQNAGTVL---------
--FRQGDQG--KSWYIIL
KGSVNVVIHGK-----GV------------------------------VCTLQEG-DDF
GKLAL-----------VNDAPRA---ATIILNENCCQFLRVDKHDFDR------ILRD--
---V--------------

-------------------------EANTVRLKEHGHDVLVLEK-----VPI---------
---------R---------------VNRPPIAYR---------------YSVMAGTPEKM
IEYLLET-------------RIDC---------------AKDE----DIADTFME---D-
FILTHLI--FM---------------PTNELCNALT-----V-----------------
--------YYQQ-GCSSI---NGNSAL-----SE---SIMTESVEHQLSFRKRVIAFLQA
WVQISGHMFFED-PVGAAFIEVVVVEMYCA--ILEDS--------RDH-----PQLLPE-

--------------LTVVEKLMR-EREQALGSS---------------------------
----AR--------PPVI--------------------------------LSDNNFI-RE
L--PANSP---

------VLPFDL--C---IVKV
NSFDSLRTSTLKIRVDQTCETIKR--------MASQK-----------LGLGN-GEGVEL
VEVKSS----------------------REKNI------FTPNDTGIPTSMSHNGRLFAC
FKDQ---V-ESLS-PLPEQ---------------------------SGP------SESVH
SV-ILETLTSTELAQHLLIYHWQLFINTHEYELLY-------HVVSRNLF-PGKVPVNLD
LLIRR---------FNELQFWV-----ITEILLCGSVSKR------AHVVKKFIKIALH-
--------------CKTNQDLFSFFAITL-GLSNVAISRLSQTWE---------------
-------KVNAKFRKLFFE-FESLL--------------------------DPSRNH---
-RAYRL----LVAK-------MKPPTI-PFIP----------------------------
LLLKDLLFAHEGNK--TY-FD------------------------------G-MVNFE-KM
-------HM---MAQTLRNHRMYKS--Q------MPQI-------------------D--
----------------------SSKIP-----M----------------DAQH-----LV
RNFRIIDDQRKF---LRLSQLL
-----------EPKQ----------KDTT-------------------------------

>Rapgef4_[Trichinella_patagoniensis]_954341164
---MSSPKSNS
RDEQAMPPKTKNIFKTALFAMAFKRACSKSIKKVSHADCNSVADRIRGVAAFEHFSQQLV
ELLASRG--LYEELD--AGIVLFCEGKPSSNWYALLDGSVDLYCYDPEKPS---------
----ELVKIKTLHSGCTFGEGALNKQ-LQPCTAI-VSQRSQFIRLDFSSLSLIWE-----
QYEN--ELMQNTVSLESIVKNKFVKAS-SSKMSKGVKQAD-SVSLSAFKSETSAFAAA-T
NYKPSKEDAVNCDPKQTAASVGEQ--MSLKANQPFPMSRGVVASDSSALLSSRAK-----

```
YSPLMDTTSSTSSFLSDTDQSTTDSQVSSKFSIQIKKKKKKKKKPEKRKGLKNNFQSFHS
IYCTMPADSSVDPQREVS-TLRKSSSVSDKLEVKNPATSIGDPMAEPANGITTNATATAR
PDDSQVNNNLIELKNLLTPDMQISWA---------------------SRVLR-SAMVVRS
PN-M----------------IRDRKFQ--------MTL---------YK------RC-MV
GT-----------------EMVDWLLRQS-------------------------------
---NSVV-------QNRMQAAGIW---QVLLEEGIIH------------------------
--------------H------------------------VTH-EYPFLDKY-LFYRWVED
E------RSTD----AA-V-----------------------------------------
-----------------------------------------------------RALNLSD
DD---GIYSENDF------LLAISNLSIMGPDAL-------------FRMVLRKAPNERS
NEELE-----VVFEELLNIKALSNLS-TMVKRELA-TVIMFEQHQNAGTVL---------
--------------------------------------------FRQGDQG--KSWYIIL
KGSVNVVIHGK-----GV-------------------------------VCTLQEG-DDF
GKLAL-----------VNDAPRA---ATIIILNENCCQFLRVDKHDFDR-----ILRD--
---------------------------------------------V--------------
------------------------------------------------------------
------------------------------------------------------------
------------------------EANTVRLKEHGHDVLVLEK-----VPI---------
---------R---------------VNRSPIAYR---------------YSVMAGTPEKM
VEYLLET-------------RIDC---------------AKDE----DIADTFME---D-
FILTHLI--FM---------------PTNELCNALT-----I------------------
--------YYQQ-GCSSI---NGNSAL-----SE---SILTESVEHQLSFRKRVIAFLQA
WIQISGHMFYED-PVGAAFIE----EMYCA--ILEDS--------RDH-----PQLLPE-
------------------------------------------------------------
--------------LTVVEKLMR-EREQALGSS---------------------------
----AR--------PPVI--------------------------------LSDNNFI-RE
L--PANSP----------------------------------------------------
------------------------------------------------------------
------------------------------------------------------------
------------------------------------------------------------
------------------------------------------------------------
------VLPFDL--C-----------------------------------------IVKV
NSSDSLRTSTLKIRVDQTCETIKR--------MASQK-----------LGLGN-GEGVEL
VEVKSN----------------------REKNI------FTPNDTGIPTSMSHNGRLFAC
FKDQ---V-ESLS-PLPEQ---------------------------AGP------SESVH
SV-ILETLTSTELAQHLLIYHWQLFINTHEYELLY-------HVVGRNLF-PGKVPVNLD
LLIRR---------FNELQFWV-----ITEILLCGSVSKR------AHVVKKFIKIALH-
--------------CKANQDLFSFFAITL-GLSNVAISRLSQTWE---------------
-------KVNAKFRKLFFE-FESLL--------------------------DPSRNH---
-RAYRL----LVAK-------MKPPTI-PFIP----------------------------
LLLKDLLFAHEGNK--TY-FD-----------------------------G-MVNFE-KM
-------HM---MAQTLRNHRMYKS--Q------MLQI-------------------D--
----------------------SSKIP-----T----------------DAQH-----LV
RNFRIIDDQRKF-----------------------------------------LRLSQLL
-----------EPKQ----------KDTT-------------------------------
------------------------------------------------------------
------------------------------------------------------------
------------------------------------------------------------
------------------------------------------------------------
------------------------------------------------------------
------------------------------------------------------------
------------------------------------------------------------
------------------------------------------------------------
------------------------------------------------------------
```


>Rapgef4_[Trichinella_sp._T6]_954280930
---MSMLKKELYMSSPKSNS
RDEQAMPPKTKNIFKTALFAMAFKRACSKSIKKVSHADCNSVADRIRGVAAFEHFSQQLV
ELLASRG--LYEELD--AGIVLFCEGKPSSNWYALLDGSVDLYCYDPEKPS---------
----ELVKIKTLHSGCTFGEGALNKQ-LQPCTAI-VSQRSQFIRLDFSSLSLIWE-----
QYEN--ELMQNTVSLESIVKNKFVKTS-SSKMSKGVKQAD-SVSLSAFKSETSAFAAA-T
NYKPSKEDAVNCEPKQTAASVGEQ--MSLKANQPFPMSRGVVASDSSALLSSRAK-----
YSPLMDTTSSTSSFLSDTDQSTTDSQVSSKFSIQIKKKKKKKKKPEKRKGLKNNFQSFHS
IYCTMPADSSVDPQREVS-TLRKSSSVSDKLEVKNPATSIGDPMAEPANGITTNATATAR
PDDSQVNNNLIELKNLLTPDMQITWA---------------------SRVLR-SAMVVRS
PN-M----------------IRDRKFQ--------MTL---------YK------RC-MV
GT-----------------EMVDWLLRQS-------------------------------
---NSVV-------QNRMQAAGIW---QVLLEEGIIH-----------------------
--------------H------------------------VTH-EYPFLDKY-LFYRWVED
E------RSTD----AA-V---
---RALNLSD
DD---GIYSENDF------LLAISNLSIMGPDAL-------------FRMVLRKAPNERS
NEELE-----VVFEELLNIKALSNLS-TMVKRELA-TVIMFEQHQNAGTVL---------
--FRQGDQG--KSWYIIL
KGSVNVVIHGKVPISPGV-------------------------------VCTLQEG-DDF
GKLAL-----------VNDAPRA---ATIILNENCCQFLRVDKHDFDR------ILRD--
---V--------------

------------------------EANTVRLKEHGHDVLVLEK-----VPI---------
---------R---------------VNRSPIAYR---------------YSVMAGTPEKM
VEYLLET-------------RIDC---------------AKDE----DIADTFME---D-
FILTHLI--FM---------------PTNELCNALT-----I------------------
--------YYQQ-GCSSI---NGNSAL-----SE---SILTESVEHQLSFRKRVIAFLQA
WIQISGHMFYED-PVGAAFIEVVMVEMYCA--ILEDS--------RDH-----PQLLPE-

--------------LTVVEKLMR-EREQALGSS---------------------------
----AR--------PPVI--------------------------------LSDNNFI-RE
L--PANSP--

------VLPFDL--C---IVKV
NSSDSLRTSTLKIRVDQTCETIKR--------MASQK-----------LGLGN-GEGVEL
VEVKSN----------------------REKNI------FTPNDTGIPTSMSHNGRLFAC
FKDQ---V-ESLS-PLPEQ---------------------------AGP------SESVH
SV-ILETLTSTELAQHLLIYHWQLFINTHEYELLY-------HVVGRNLF-PGKVPVNLD
LLIRR---------FNELQFWV-----ITEILLCGSVSKR------AHVVKKFIKIALH-
--------------CKANQDLFSFFAITL-GLSNVAISRLSQTWE---------------
-------KVNAKFRKLFFE-FESLL--------------------------DPSRNH---
-RAYRL----LVAK-------MKPPTI-PFIP----------------------------
LLLKDLLFAHEGNK--TY-FD-----------------------------G-MVNFE-KM
-------HM---MAQTLRNHRMYKS--Q------MLQI-------------------D--
----------------------SSKIP-----T----------------DAQH-----LV
RNFRIIDDQRKF---LRLSHLL
-----------EPKQ----------KDTT-------------------------------

>Rapgef4_[Trichinella_sp._T8]_954617710
----------MRNVICCKFDYFGRISVACKNLEWLFAV----SGDMLKKELYMSSPKSNS
RDEQAMPPKTKNIFKTALFAMAFKRACSKSIKKVSHADCNSVADRIRGVAAFEHFSQQLV
ELLASRG--LYEELD--AGIVLFCEGKPSSNWYALLDGSVDLYCYDPEKPS---------
----ELVKIKTLHSGCTFGEGALNKQ-LQPCTAI-VSQRSQFIRLDFSSLSLIWE-----
QYEN--ELMQNTVSLESIVKNKFVKAS-SSKMSKGVKQAD-SVSLSAFKSETSAFAAA-T
NYKPSKEDAVNCDPKQTAASVGEQ--MSLKANQPFPMSRGVVASDSSALLSSRAK-----
YSPLMDTTSSTSSFLSDTDQSTTDSQVSSKFSIQIKKKKKKKKKPEKRKGLKNNFQSFHS
IYCTMPADSSVDPQREVS-TLRKSSSVSDKLEVKNPATSIGDPMAEPANGITTNATATAR
PDDSQVNNNLIELKNLLTPDMQISWA---------------------SRVLR-SAMVVRS
PN-M----------------IRDRKFQ--------MTL---------YK------RC-MV
GT-----------------EMVDWLLRQS-------------------------------
---NSVV-------QNRMQAAGIW---QVLLEEGIIH-----------------------
--------------H------------------------VTH-EYPFLDKY-LFYRWVED
E------RSTD----AA-V---
---RALNLSD
DD---GIYSENDF------LLAISNLSIMGPDAL-------------FRMVLRKAPNERS
NEELE-----VVFEELLNIKALSNLS-TMVKRELA-TVIMFEQHQNAGTVL---------
--FRQGDQG--KSWYIIL
KGSVNVVIHGK-----GV-------------------------------VCTLQEG-DDF
GKLAL-----------VNDAPRA---ATIILNENCCQFLRVDKHDFDR------ILRD--
---V--------------

------------------------EANTVRLKEHGHDVLVLEK-----VPI---------
---------R---------------VNRSPIAYR---------------YSVMAGTPEKM
VEYLLET-------------RIDC---------------AKDE----DIADTFME---D-
FILTHLI--FM---------------PTNELCNALT-----I------------------
--------YYQQ-GCSSI---NGNSAL-----SE---SILTESVEHQLSFRKRVIAFLQA
WIQISGHMFYED-PVGAAFIE----EMYCA--ILEDS--------RDH-----PQLLPE-

--------------LTVVEKLMR-EREQALGSS---------------------------
----AR--------PPVI--------------------------------LSDNNFI-R-

--IVKV
NSSDSLRTSTLKIRVDQTCETIKR--------MASQK-----------LGLGN-GEGVEL
VEVKSN----------------------REKNI------FTPNDTGIPTSMSHNGRLFAC
FKDQ---V-ESLS-PLPEQ---------------------------AGP------SESVH
SV-ILETLTSTELAQHLLIYHWQLFINTHEYELLY-------HVVGRNLF-PGKVPVNLD

LLIRR---------FNELQFWV-----ITEILLCGSVSKR------AHVVKKFIKIALH-
--------------CKANQDLFSFFAITL-GLSNVAISRLSQTWE---------------
-------KVNAKFRKLFFE-FESLL--------------------------DPSRNH---
-RAYRL----LVAK-------MKPPTI-PFIP----------------------------
LLLKDLLFAHEGNK--TY-FD-----------------------------G--------M
-------HM---MAQTLRNHRMYKS--Q------MLQI-------------------D--
----------------------SSKIP-----T----------------DAQH-----LV
RNFRIIDDQRKF---LRLSQLL
-----------EPKQ----------KDTT-------------------------------

>Rapgef4_[Trichinella_sp._T9]_954260542
LQFHLCCLVIMRNVICCKFDYFGRISVACKNLEWLFAV----S---VPTELYMSSPKSNS
RDEQAMPPKTKNIFKTALFAMAFKRACSKSIKKVSHADCNSVADRIRGVAAFEHFSQQLV
ELLASRG--LYEELD--AGIVLFCEGKPSSNWYALLDGSVDLYCYDPEKPS---------
----ELVKIKTLHSGCTFGEGALNKQ-LQPCTAI-VSQRSQFIRLDFSSLSLIWE-----
QYEN--ELMQNTVSLESIVKNKFVKAS-SSKMSKGVKQAD-SVSLSAFKSETSAFAAA-T
NYKPSKEDAVNCDPKQTAASVGEQ--MSLKANQPFPMSRGVVASDSSALLSSRAK-----
YSPLMDTTSSTSSFLSDTDQSTTDSQVSSKFSIQIKKKKKKKK-PEKRKGLKNNFQSFHS
IYCTMPADSSVDPQREVS-TLRKSSSVSDKLEVKNPATSIGDPMAEPANGITTNATATAR
PDDSQVNNNLIELKNLLTPDMQISWA---------------------SRVLR-SAMVVRS
PN-M----------------IRDRKFQ--------MTL---------YK------RC-MV
GT-----------------EMVDWLLRQS-------------------------------
---NSVV-------QNRMQAAGIW---QVLLEEGIIH-----------------------
--------------H------------------------VTH-EYPFLDKY-LFYRWVED
E------RSTD----AA-V---
---RALNLSD
DD---GIYSENDF------LLAISNLSIMGPDAL-------------FRMVLRKAPNERS
NEELE-----VVFEELLNIKALSNLS-TMVKRELA-TVIMFEQHQNAGTVL---------
--FRQGDQG--KSWYIIL
KGSVNVVIHGK-----GV-------------------------------VCTLQEG-DDF
GKLAL-----------VNDAPRA---ATIILNENCCQFLRVDKHDFDR------ILRD--
---V--------------

------------------------EANTVRLKEHGHDVLVLEK-----VPI---------
---------R---------------VNRSPIAYR---------------YSVMAGTPEKM
VEYLLET-------------RIDC---------------AKDE----DIADTFME---D-
FILTHLI--FM---------------PTNELCNALT-----I------------------
--------YYQQ-GCSSI---NGNSAL-----SE---SILTESVEHQLSFRKRVIAFLQA
WIQISGHMFYED-PVGAAFIEVVVVEMYCA--ILEDS--------RDH-----PQLLPE-

--------------LTVVEKLMR-EREQALGSS---------------------------
----AR--------PPVI--------------------------------LSDNNFI-RE
L--PANSP--

------VLPFTF--DK-----------------------------NETLLLLTHAGIVKV
NSSDSLRTSTLKIRVDQTCETIKR--------MASQK-----------LGLGN-GEGVEL
VEVKSN----------------------REKNI------FTPNDTGIPTSMSHNGRLFAC
FKDQ---V-ESLS-PLPEQ---------------------------AGP------SESVH
SV-ILETLTSTELAQHLLIYHWQLFINTHEYELLY-------HVVGRNLF-PGKVPVNLD
LLIRR---------FNELQFWV-----ITEILLCGSVSKR------AHVVKKFIKIALH-
--------------CKANQDLFSFFAITL-GLSNVAISRLSQTWE---------------
-------KVNAKFRKLFFE-FESLL--------------------------DPSRNH---
-RAYRL----LVAK-------IKPPTI-PFIP----------------------------
LLLKDLLFAHEGNK--TY-FD------------------------------G-MVNFE-KM
-------HM---MAQTLRNHRMYKS--Q------MLQI-------------------D--
----------------------SSKIP-----T----------------DAQH-----LV
RNFRIIDDQRKF---LRLSHLL
-----------EPKQ----------KDTT-------------------------------

>Rapgef4_[Trichinella_spiralis]_954362210
---MSMLKKELYMSSPKSNS
CDEQAMPPKTKNIFKTALFAMAFKRACSKSIKKVSHADCNSVADRIRGVAAFEHFSQQLV
ELLASRG--LYEELD--AGIVLFCEGKPSSNWYALLDGSVDLYCYDPEKPS---------
----ELVKIKTLHSGCTFGEGALNKQ-LQPCTAI-VSQRSQFIRLDFSSLSLIWE-----
QYEN--ELMQNTVSLESIVKNKFVKAS-SSKISKGVKQAD-SVSLSAFKSET-AFAAA-S
NYKPSKEEAVNCDPKQTAASVGEQ--MSLKANQPFPMSRGVAASDSSALLSSRAK-----
YSPLMDTTSSTSSFLSDTDQSTTDSQVSSKFSIQIKKKKKKKK-PEKRKGLKNNFQSFHS
IYCTMPADSSVDPQREVS-TLRKSSSVGDKLEAKNPATSIGDPMAEPANGITTNATATAR
PDDSQVNNNLIELKNLLTPDMQISWA---------------------SRVLR-SAMVVRS
PN-M----------------IRDRKFQ--------MTL---------YK------RC-MV
GT-----------------EMVDWLLRQS-------------------------------
---NSVV-------QNRMQAAGIW---QVLLEEGIIH-----------------------
--------------H------------------------VTH-EYPFLDKY-LFYRWVED
E------RSTD----AA-V---
---RALNLSD
DD---GIYSENDF------LLAISNLSIMGPDAL-------------FRMVLRKAPNERS
NEELE-----VVFEELLNIKALSNLS-TMVKRELA-TVIMFEQHQNAGTVL---------
--FRQGDQG--KSWYIIL
KGSVNVVIHGK-----GV-------------------------------VCTLQEG-DDF
GKLAL-----------VNDAPRA---ATIILNENCCQFLRVDKHDFDR------ILRD--
---V--------------

------------------------EANTVRLKEHGHDVLVLEK-----VPI---------

---------R---------------VNRSPIAYR---------------YSVMAGTPEKM
VEYLLET-------------RIDC---------------AKDE----DIADTFME---D-
FILTHLI--FM---------------PTNELCNALT-----I------------------
--------YYQQ-GCSSI---NGNSAL-----SE---SILTESVEHQLSFRKRVIAFLQA
WIQISGHMFYED-PVGAAFIE----EMYCA--ILEDS--------RDH-----PQLLPE-

--------------LTIVEKLMR-EREQALGSS---------------------------
----AR--------PPVI--------------------------------LSDNNFI-RE
L--PANSP--

------VLPFDL--C---IVKV
NSSDSLRTSTLKIRVDQTCETIKR--------MASQK-----------LGLGN-GEGVEL
VEVKSN----------------------REKNI------FAPNDTGIPTSMSHNGRLFAC
FKDQ---V-ESLS-PLPEQ---------------------------AGP------SESVH
SV-ILETLTSTELAQHLLIYHWQLFINTHEYELLY-------HVVGRNLF-PGKVPVNLD
LLIRR---------FNELQFWV-----ITEILLCGSVSKR------AHVVKKFIKIALH-
--------------CKANQDLFSFFAITL-GLSNVAISRLSQTWEVNANLLDFFGYHLLL
TNQRSIQKVNAKFRKLFFE-FESLL--------------------------DPSRNH---
-RAYRL----LVAK-------MKPPTI-PFIP----------------------------
LLLKDLLFAHEGNK--TY-FD-----------------------------G-MVNFE-KM
-------HM---MAQTLRNHRMYKS--Q------MLQI-------------------D--
----------------------SSKIP-----T----------------DAQH-----LV
RNFRIIDDQRKF---LRLSQLL
-----------EPKQ----------KDTT-------------------------------

>Rapgef4_[Trichinella_zimbabwensis]_954489455
-------MSSPKSNPCDAQAMSPKTKNIFKTALLTMAL----KRLCSK------------
-----------------------------SIKKVSHAEYNLVADRIRGVAAFEHFSQQLV
ELLASHG--LYEELD--AGIVLFCEGKPSSNWYALLDGSVDLYCYDPEKSS---------
----ELVKIKTLHSGCTFGEGALNKQ-RQPCTAI-VSQRSQFIRLDFSSLSLIWE-----
QYEN--ELMQNTVSLESIVKNKFVKAS-SSKMSKGVKQAD-SVSSSAFKSETSAFAAA-A
NYKPSKEEAVNCDPKQTTTNVGEQ--MPLKANQPFPTSRGVAASDSSALLSGRAK-----
YSPLMDTTSSASSFLSDTDQSTTDSQLSSKFSIKIKKKKKKKKKPEKRKGPKNNFQPFHS
IYCTAPADSSVDPEHEVNNTLRKSSSMGDKLEAKNPTSNIGDPVTEPANGITTNATATTR
PDDSQINNNLIELKNLLTPDMQISWA---------------------SRVFR-SAMVVRS
PN-M----------------IRDRKFQ--------MTL---------YK------RC-MV
GT-----------------EMVDWLLRQS-------------------------------
---NSIV-------QNRMQAAGIW---QVLLEEGIIH-----------------------
--------------H------------------------VTH-EYPFLDKY-LFYRWVED
E------RSTD----AA-V---
---RALNLSD

DD---GIYSENDF------LLAISNLSIMGPDAL-------------FRMVLRKAPNERS
NEELE-----VVFEELLNIKALSNLS-TMVKRELA-TVIMFEQHQNAGTVL---------
--FRQGDQG--KSWYIIL
KGSVNVVIHGK-----GV-------------------------------VCTLQEG-DDF
GKLAL-----------VNDAPRA---ATIILNENCCQFLRVDKHDFDR------ILRD--
---V--------------

------------------------EANTVRLKEHGHDVLVLEK-----VPI---------
---------R---------------VNRPPIAYR---------------YSVMAGTPEKM
IEYLLET-------------RIDC---------------AKDE----DIADTFME---D-
FILTHLI--FM---------------PTNELCNALT-----V------------------
--------YYQQ-GCSSI---NGNSAL-----SE---SIMTESVEHQLSFRKRVIAFLQA
WVQISGHMFFED-PVGAAFIE----EMYCA--ILEDS--------RDH-----PQLLPE-

--------------LTVVEKLMR-EREQALGSS---------------------------
----AR--------PPVI--------------------------------LSDNNFI-RE
L--PANSP--

---V
NSFDSLRTSTLKIRVDQTCETIKR--------MASQK-----------LGLGN-GEGVEL
VEVKSS----------------------REKNI------FTPNDTGIPTSMSHNGRLFAC
FKDQ---V-ESLS-PLPEQ---------------------------SGP------SESVH
SV-ILETLTSTELAQHLLIYHWQLFINTHEYELLY-------HVVGRNLF-PGKVPVNLD
LLIRR---------FNELQFWV-----ITEILLCGSVSKR------AHVVKKFIKIALH-
--------------CKTNQDLFSFFAITL-GLSNVAISRLSQTWE---------------
-------KVNAKFRKLFFE-FESLL--------------------------DPSRNH---
-RAYRL----LVAK-------MKPPTI-PFIP----------------------------
LLLKDLLFAHEGNK--TY-FD-----------------------------G--------M
-------HM---MAQTLRNHRMYKS--Q------MPQI-------------------D--
----------------------SSKIP-----M----------------DAQH-----LV
RNFRIIDDQRKF---LRLSQLL
-----------EPKQ----------KDTT-------------------------------

>Rapgef4_[Tupaia_chinensis]_562844413
----------------------------MMGS---------------------------K
TALPAGS--C--------------RPLE-----RSSEDVDIIFTRLKEVKAFEKFHPNLL
HQICLCGY--YENLE--KGITLFRQGDIGTNWYAVLAGSLDVKVSE-TSSHQDA------
------VTICTLGIGTAFGESILDNT-PRHATIV-TRESSELL-----------------
----------------------------RIEQKD-------FKALW---EKYRQYMAG-L
LAPPYG--VMETGS--N-N--------DRIPDKE--------------------------

```
------------------N-----------------------------------------
-----------------------------------------VPS----------------
-------EKIL-------------RA---------------------GKILR-NAILSRA
PH-M----------------IRDRKYH------L-KTY----------------RQC-CV
GT-----------------ELVDWMMQQT-------------------------------
---PCVH--------SRTQAVGMW---QVLLEDSVLN-----------------------
--------------H------------------------VDQ-EHHFQDKY-LFYRFLDD
-------EHED----AP-L-----------------------------------------
----------------------------------------------------------PT
EEEK--KEWDEEL------QDTMLLLSQMGPDAH-------------MRMILRKPPGQRT
VDDLE-----IIYEELLHIKALSHLS-TTVKRELA-GVLVFESHAKGGTVL---------
--------------------------------------------FNQGEEG--TSWYIIL
KGSVNVVIYGK-----GV-------------------------------VCTLHEG-DDF
GKLAL-----------VNDAPRA---ASIVLREDNCHFLRVDKEDFNR------ILRD--
---------------------------------------------V--------------
------------------------------------------------------------
------------------------------------------------------------
------------------------EANTVRLKEHDQDVLVLEK-----VPA-GNRASNQG
NS----QP-QQ------------------------------------K-YTVMSGTPEKI
LEHFLET------------IRLEP-ALN--------------GA-----TDSVLN---D-
FIMMHCV--FM---------------PNTQLCPALV-----A------------------
--------H-----------Y-------HAQPSQ---GTEQEKMDYALNNKRRVIRLVLQ
WAAMYGDLLQED-DVAVAFLE----EFYVS--VSDDA--RMIAALK---EQ-LPELEKIV
KQI---SED--AK---------APQKK---------------------------------
---HKV--------LLQQFNTGD-ERAQ-KRQ----------------------------
------------------------------------------------------------
------------------------------------------------------------
------------------------------------------------------------
------------------------------------------------------------
------------------------------------------------------------
------------------------------------------------------------
-----PIRGSDE--VL-----------------------------FK-------------
VYCMDHTYTTIRVPVAASVKEVIS--------AVADK-----------LGSG-EG--MII
VKMSSG----------------------GEKVV------LKPNDVSVFTTLTINGRLFAC
PREQ---F-DSLT-PLPEQ---------------------------EGP------TVGTV
GT--FELMSSKDLAYQMTIYDWELFNCVHELELIY-------HTFGRHNF-KK-TTANLD
LFLRR---------FNEIQFWV-----VTEICLCSQLSKR------VQLLKKFIKIAAH-
--------------CKEYKNLNSFFAIVM-GLSNVAVSRLALTWE---------------
-------KLPSKFKKFYAE-FESLM--------------------------DPSRNH---
-RAYRL----TVAK-------LEPPLI-PFMP----------------------------
LLIKDMTFTHEGNK--TF-ID-----------------------------N-LVNFE-KM
-------RM---IANTARTVRYYRS--Q-------------------------PFNPD--
-A------------------AQANKNH-----Q----------------DVRS-----YV
RQLNVIDNQRTL-----------------------------------------SQMSHRL
-----------EPRR----------P----------------------------------
------------------------------------------------------------
------------------------------------------------------------
------------------------------------------------------------
------------------------------------------------------------
------------------------------------------------------------
------------------------------------------------------------
------------------------------------------------------------
------------------------------------------------------------
------------------------------------------------------------
```


>Rapgef4_[Tursiops_truncatus]_470618433
---M
R-ARF-VHSPLARTWLQHNC-KGSRPLE-----RSSEDVDIIFTRLKEVK----------

--------------------AFEKFH-PNLLHQIC--LCGYYENLEKGITL---------
--FRQGDIG--TNWYAVL
AGSLDVKVSETSSHQDAVT------------------------------ICTLGIG-TAF
GE-SI-----------LDNTPRH---ATIVTRE-SSELLRIEQKDFKA------LWEKYR
QYMAGLLAPPYGVMETGSNNDRIPDKENVSSYSVSA------------------------

>Rapgef4_[Tyto_alba]_701342931

-------------------------PLE-----RSSEDVDIIFTRLKEVK----------

--------------------AFEKFH-PNLLHQIC--LCGYYENLEKGITL---------
--FRQGDIG--TNWYAVL
SGSLDVKVSDTSNHQDAVT------------------------------ICTLGIG-TAF
GE-SI-----------LDNTPRH---ATIVTRE-YSELLRIEQKDFKA------LWE---

>Rapgef4_[Ursus_maritimus]_670988641
----------------------------M-------------------------------

--HGYK-QKYRQYMAG-L
LAPPYG--VMETGS--N-N--------DRIPDKE--------------------------
------------------N----------------------T------------------
-----PLIEPHVPLRPAN-TISK------------------VPS----------------
-------EKIL-------------RA---------------------GKILR-NAILSRA
PH-M----------------IRDRKYH------L-KTY----------------RQC-CV
GT-----------------ELVDWMLQQT-------------------------------
---SCVH--------SRTQAVGMW---QVLVEDSVLN-----------------------
--------------H------------------------VDQ-EHHFQDKY-LFYRFLDD
-------EHED----AP-L---
--PT
EEEK--KECDEEL------QDTMLLLSQMGPDAH-------------MRMILRKPPGQRT
VDDLE-----IIYEELIHIKALSHLS-TTVKRELA-GVLIFESHAKGGTVL---------
--FNQGEEG--TSWYIIL
KGSVNVVIYGK-----GV-------------------------------VCTLHEG-DDF
GKLAL-----------VNDAPRA---ASIVLREDNCHFLRVDKEDFNR------ILRD--
---V--------------

------------------------EANTVRLKEHDQDVLVLEK-----VPA-GNRASNQG
NS----QP-QQ------------------------------------K-YTVMSGTPEKI
LEHFLET------------IRLEP-ALN--------------EA-----TDSVLN---D-
FVMMHCV--FM---------------PNTQLCPALV-----A------------------
--------H-----------Y-------HAQPSQ---GTEQEKMDYALNSKRRVIRLVLQ
WAAVYGDVLQED-DVAVAFLE----EFYVS--VSDDA--RMIAALK---DQ-LPELEKIV
KQI---SED--AK---------APQKK---------------------------------
---HKV--------LLQQFNTGD-ERAQ-KRQ----------------------------

-----PIRGSDE--VL-----------------------------FK-------------
VYCMDHTYTTIRVPVAASVKEVIS--------AVADK-----------LGSG-EG--LII
VKMSSG----------------------GEKVV------LKPNDVSVFTTLTINGRLFAC
PREQ---F-DSLT-PLPEQ---------------------------EGP------TVGTV
GT--FELMSSKDLAYQMTIYDWELFNCVHELELIY-------HTFGRHNF-KK-TTANLD
LFLRR---------FNEIQFWV-----VTEICLCSQPSKR------VQLLKKFIKIAAH-
--------------CKEYKNLNSFFAIVM-GLSNVAVSRLALTWE---------------
-------KLPSKFKKFYAE-FESLM--------------------------DPSRNH---
-RAYRL----TVAK-------LEPPLI-PFMP----------------------------
LLIKDMTFTHEGNK--TF-ID-----------------------------N-LVNFE-KM
-------RM---IANTARTVRYCRS--Q-------------------------AFNPD--
-A------------------AQANKNH-----Q----------------DVRS-----YV
RQLNVIDNQRTL---SQMSHRL
-----------EPRR----------P----------------------------------

>Rapgef4_[Vicugna_pacos]_560983554
----------------------------M-------------------------------

--PY---KKYRQYMAG-L
LAPPYG--VMETGS--N-N--------DRIPDKE--------------------------
------------------N----------------------T------------------
-----PLIEPHVPLRPAN-TITK------------------VPS----------------
-------EKIL-------------RA---------------------GKILR-NAILSRA
PH-M----------------IRDRKYH------L-KTY----------------RQC-CV
GT-----------------ELVDWMLQQT-------------------------------
---PCVH--------SRTQAVGMW---QVLVEDGVLN-----------------------
--------------H------------------------VDQ-EHHFQDKY-LFYRFLDD
-------EHED----AP-L---
--PT
EEEK--KECDEEL------QDTMLLLSQMGPDAH-------------MRMILRKPPGQRT
VDDLE-----IIYEELLHIKALSHLS-TTVKRELA-GVLIFESHAKGGTVL---------
--FNQGEEG--TSWYIIL
KGSVNVVIYGK-----GV-------------------------------VCTLHEG-DDF
GKLAL-----------VNDAPRA---ASIVLREDNCHFLRVDKEDFNR------ILRD--
---V--------------

------------------------EANTVRLKEHDQDVLVLEK-----VPA-GNRASNQG

NS----QP-QQ------------------------------------K-YTVMSGTPEKI
LEHFLET------------IRLEP-ALN--------------EA-----TDSVLN---D-
FVMMHCV--FM---------------PNTQLCPALV-----A------------------
--------H-----------Y-------HAQPSQ---GTEQEKVDYALNNKRRVIRLVLQ
WAAMYGDILQED-DVAVAFLE----EFYVS--VSDDA--RMIAALK---EQ-LPELEKIV
KQI---SED--AK---------TPQKK---------------------------------
---HKV--------LLQQFNTGD-ERAQ-KRQ----------------------------

-----PIRGSDE--VL-----------------------------FK-------------
VYCMDHTYTTIRVPVAASVKEVLS--------AVADK-----------LGSG-DG--LII
VKMSSG----------------------GEKVV------LKPNDVSVFTTLTINGRLFAC
PREQ---F-DSLT-PLPEQ---------------------------EGP------TVGTV
GT--FELMSSKDLAYQMTTYDWELFNCVHELELIY-------HTFGRHHF-KK-TTANLD
LFLRR---------FNEIQFWV-----VTEICLCSQPSKR------VQLLKKFIKIAAH-
--------------CKEYKNLNSFFAIVM-GLSNVAVSRLALTWE---------------
-------KLPSKFKKFYAE-FESLM--------------------------DPSRNH---
-RAYRL----TVAK-------LEPPLI-PFMP----------------------------
LLIKDMTFTHEGNK--TF-ID-----------------------------N-LVNFE-KM
-------RM---IANTARMVRYCRS--Q-------------------------PFNPD--
-A------------------AQANKNH-----Q----------------DVRS-----YV
RQLNVIDNQRTL---SQMSHRL
-----------EPRR----------P----------------------------------

>Rapgef4_[Xenopus_tropicalis]_512866132
----------------------------MVAA-------HT------AH--------STS
SAEWIA---CLDK-----------RPLE-----RSGEDVDIIFARLKEVKAFERFHPNLL
QQICFCGY--YENLE--KGITLYRQGDIGTSWYAVLTGSLDVKVSE-TNNHQDA------
------VVICTLGVGTAFGESILDNT-PRHATIV-TRENSELL-----------------
----------------------------RIEQKD-------FKTLW---EKYRQYMAG-F
LAPPYG--VLETGS--N----------DRLSDKE--------------------------
------------------NMN-------S-ALGPGSKNCNKT------------------
-----PLIENQIPIRPAK-TITE------------------VPS----------------
-------ERIL-------------RA---------------------GKTLR-NAILTRA
PH-M----------------IRDRKYH------L-KTY----------------RQC-CV
GT-----------------ELVDWLMQQS-------------------------------
---SCVH--------SRTQAVGMW---QVLLEEGVLN-----------------------
--------------H------------------------VDQ-EYYFQDKY-LFYRFLDD
-------EHED----AP-M---
--PT

EEEK--KESDEEL------QDTLLLLSQIGPDAH-------------MRMILRKPPGQRS
ADDLE-----IIFEELIHIKALSHLS-TTVKRELA-GFLIFESHPKAGTVL---------
--FNQGEEG--TSWYIIL
KGSVNVVIYGK-----GV-------------------------------VCTLHEG-DDF
GKLAL-----------VNDAPRA---ASIVLREDNCHFLRVDKEDFNR------ILRD--
---V--------------

------------------------EANTVRLKEHDQDVLVLEK-----ILA-GSQGSAQG
NT----QS-PY------------------------------------K-YTVMSGNPEKL
LEHFLET------------MRLDP-SLN--------------DA-----TDTALD---D-
FILMHCV--FM---------------PNSQLCPALM-----S------------------
--------H-----------Y-------HAQPSQ---GTEQEKLDYTLNNKRRVIRLVQQ
WATLYGDLLRED-EVSMAFLE----EFYVS--VSDDT--RTTAALK---EQ-LPDLERAI
KQI---SED--AK---------GQQKK---------------------------------
---HKV--------LLRQFSTGD-ERLQ-KRQ----------------------------

-----AIRSSDE--LL-----------------------------FK-------------
VYCIDHTYTTIRVAVAASVKEVLS--------AVADK-----------LGSG-DS--LIL
VKISSA----------------------GEKVV------LKPNEISVFTTLSVNGRLFAC
PRDQ---F-DSLT-PLPEQ---------------------------EGP------SAGTM
ST--FELMSSKDLAYQMTIHDWDLFSCVHELELIY-------HTFGRQNF-KK-TTANLD
LFLRR---------FNEIQFWV-----VTEICLCPQLSKR------VQLLKKFIKIAAH-
--------------CKEYKNLNSFFAIVM-GLSNVAVSRLSLTWE---------------
-------KLPSKFKKIYAE-FENLM--------------------------DPSRNH---
-RAYRL----TVAK-------LDPPII-PFTP----------------------------
LLIKDMTFTHEGNK--TF-ID-----------------------------N-LVNFE-KM
-------RM---IANTLRTIRYCRS--V-------------------------PFNPD--
-A------------------SLAGKNH-----Q----------------DVRN-----YV
RQFNVIDNQRTL---SQMSHRL
-----------EPRR----------A----------------------------------

>Rapgef4_[Xiphophorus_maculatus]_941773030
---------------------------------MV-----A------VQ---TSLNSSPS
-AEWIC---CLDK-----------RPSE-----RSGEDVDIILTRLRGVKTFQRFPPPLL
LQICTCAF--YECLE--KGITLFRQGDIGTSWYAVLSGSLDVKVSE-TANHQDA------
------VTICTLGIGTAFGESILDNT-PRHATIV-SRETSELL-----------------
----------------------------RIEQRE-------FKTLWE---KYRQSLAG-L
LAPPYG--AMESGS----SN-------DRLTDKD--------------------------

```
------------------NMN-------S-------------------------------
--------DSG---HKAH-----N----------------KVPS----------------
-------EKLQ-------------RA---------------------GKVLR-NAILSRA
PH-M----------------IRDRKYH------L-KTY----------------RQC-CV
GT-----------------ELVDWLVMQS-------------------------------
---ACVL--------TRSHAVGMW---QALLEEGVLN-----------------------
--------------H------------------------VDQ-ELGFHDKY-LFYRFLDD
-------EEED----TP-L-----------------------------------------
----------------------------------------------------------PS
EEEK--RESEEEL------PETILFLAQIGPDAL-------------LRMILRKPPGQRT
ADDLE-----IIYDELLHIKALSHLS-NTVKRELA-SVVIFESHAKAGTVL---------
--------------------------------------------FNQGEEG--TSWYIIQ
KGSVNVVIYGK-----GV-------------------------------VCTLHEG-DDF
GKLAL-----------VTDSPRA---ASIVLREDNCHFLRVDKEDFNR------ILRD--
---------------------------------------------V--------------
------------------------------------------------------------
------------------------------------------------------------
------------------------EANTVRLKEHEQAVLVLEK-----S----HRVSTLG
S---------I------------------------------------K-YTVISGTPEKI
LEHFLET------------MRLDI-HHN--------------EP------DPAVD---D-
FVLMHCV--FM---------------PNSQLCPLLM-----A------------------
--------H-----------Y-------HVVSPP---GSEQERLEYTLNNKRRALILTLR
WANTHTYLLQEE-PAAVSFLE----ELYGS--VSNDS--RTLRGLK---DL-VPDLEKVV
KLH---SEE--VK---------STKKK---------------------------------
-----T--------LIRQFSNGE-ERLQ-KKQ----------------------------
------------------------------------------------------------
------------------------------------------------------------
------------------------------------------------------------
------------------------------------------------------------
------------------------------------------------------------
------------------------------------------------------------
-----PIRNQDD--IL-----------------------------LK-------------
VYCSDHTYTTIRIPVVASGKEVIS--------AVSDK-----------LGTT-EE--LLL
VHLSSA----------------------GEKQM------LKPNDVSVFSTLSINGRLFAC
PREQ---L-GSLA-PLPDQ---------------------------EGP------SAGSM
ST--FELMSSKDLAYQMTMFDWELFSCVHEHELLY-------HTFGRHSF-RR-TTANLD
LFLRR---------FNQVQLWV-----VTEVCLCTQLSKR------VQLLKKFIKIAAH-
--------------CREFKNLNSFFAIIM-GMSNPAVSRLSQTWE---------------
-------KLPTKFKKFYAE-FESMM--------------------------DPSRNH---
-RSYRL----TVTK-------LEPPII-PFMP----------------------------
LLLKDMTFTHEGNK--TF-ID-----------------------------N-MVNFE-KM
-------RI---IANTIRQVRHCRS--Q-------------------------PFNPD--
-I------------------CQPNKNQ-----A----------------EVRG-----YV
RKLCVIDNQRAL-----------------------------------------TQLSYRL
-----------EPRR----------T----------------------------------
------------------------------------------------------------
------------------------------------------------------------
------------------------------------------------------------
------------------------------------------------------------
------------------------------------------------------------
------------------------------------------------------------
------------------------------------------------------------
------------------------------------------------------------
------------------------------------------------------------
```


>Rapgef4_[Zonotrichia_albicollis]_929451867

---MSG-L
LTPPYG--VMETGS--N-N--------DRMPDKD--------------------------
------------------SMS-------NSALCQVSKNCNKT------------------
-----PLIDPHIPHHPTK-TITQ------------------VPS----------------
-------EKIL-------------RA---------------------GKILR-NTILSRA
PH-M----------------IRDRKYH------L-KTY----------------RQC-CV
GT-----------------ELVDWMMQQS-------------------------------
---PCVH--------LRTQAVGMW---QVLLEEGVLN-----------------------
--------------H------------------------VDQ-EHHFQDKY-LFYRFLDD
-------ERED----AP-L---
--PT
EEEK--KECDEEL------QDTMLLLSQIGPDAH-------------MRMILRKPPGQRT
VDDLE-----FIYEELLHIKALSHLS-TTVKRELA-GVLIFESHPKAGTVL---------
--FNQGEEG--TSWYIIL
KGSVNVVIYGK-----GV-------------------------------VCTLHEG-DDF
GKLAL-----------VNDAPRA---ASIVLREDNCHFLRVDKEDFNR------ILRD--
---V--------------

------------------------EANTVRLKEHDQDVLVLEK-----IPA-GNRISNQG
NS----QP-QH------------------------------------K-YIVMSGTPEKI
LEHFLET------------MRLEA-TLN--------------EA-----TDSVLN---D-
FIMMHCV--FM---------------PNSQLCPALM-----A------------------
--------H-----------Y-------HAQPSQ---GTEQEKMDYALNNKRRVIRLVLQ
WAALYGDLLQED-EAAMAFLE----EFYVS--VSDDT--RMIAALK---EQ-LPELEKIV
KQI---SEE--PK---------GPQKK---------------------------------
---HKV--------LLQLFNTSD-DRAQ-KRQ----------------------------

-----PIRGSDE--VL-----------------------------FK-------------
VYCIDQTYTTIRVPVSSSVKEVIG--------AVADK-----------LGSG-EG--LII
VKMSSG----------------------GEKVV------LKPHDVSVFTTLSVNGRLFAC
PRDQ---F-DALA-PLPEQ---------------------------EGP------STGTV
GT--FELMSSKDLAHQMTIYDWELFNCVHELELIY-------HTFGRHNF-KK-TTANLD
LFLRR---------FNEIQFWV-----VTEICLCSQLSKR------VQLLKKYIKIAAH-
--------------CKEYKNLNSFFAIIM-GLSNVAVSRLSLTWE---------------
-------KLPSKFKKIYAE-FESLM--------------------------DPSRNH---
-RAYRL----TVAK-------LDPPII-PFMP----------------------------
LLIKDMTFTHEGNK--TF-TD-----------------------------N-LVNFE-KM
-------RM---IANTVRTVKFCRS--Q-------------------------SFNPE--
-A------------------ALTNKNH-----Q----------------DVRS-----YV
RQLNVIDNQRTL---SQMSHRL
-----------EPRR----------A----------------------------------

>Rapgef4_[Zootermopsis_nevadensis]_646703516

------------------------RPSD-----RNHKDVDVISLRLKRVELLDRLPTSVL
QQLAFYGY--YEDLE--KGVTLFRQGDLGSSWYAVLSGQLDVRLEQ-TGKDSK----DKT
P-----VSLCSLGVGATFGESILQDL-PRDSTVV-TRSTCELLRVEQQDFKLIWE-----
KHKE--FMGDIV--------------A-NSKLKNGFGPAV-L-AK-----SP---SPP-P
RSPPPSSS---------------R--RPQSPDNPSPCG----------------------

--PITE--------------
-----------------SPSPQMAVI---------------------GQTLR-TLLLAHC
SPPV----------------LRDHKSS------N-R-M---------------YRHS-AS
GT-----------------ELVDWIMTLS-------------------------------
---PSIH--------TRHQAAGMW---QALLEEGVIS-----------------------
--------------H------------------------VTG-EHPFRDKA-LLYRFWQD
E---------E----GA-T---
---G---LPS
QDEL--VWAEDQL------NASLNTLTQKGPDAI-------------MRMILRKPSHERT
VDDLE-----IIYEELLHIKALSHLS-NSVKRELA-AVIVFEAHPHAGTVL---------
--FNQGDEG--RSWYIIL
RGSVDVVIHGK-----GT-------------------------------VTTLQEG-DDF
GKLAL-----------INDAPRA---ATIVLRENNCHFLRVDKENFNR------ILRD--
---V--------------

------------------------EANTVRLKEHGQDVLVLEK-----IS----------
------PTHSV--AYS---------------HFK---------------YTVMAGTPQKM
LEHLLET-------------RLDGRGGSGS-------VGGQDS--LPSSSDPFLE---D-
FLLTHIV--FM---------------PTHQLVTELA-----R------------------
--------QYPTYRIDS---------P-----S--------QDREFLLACKRRVVQFVHC
WVITIRHPVFDD-TNSLAFLE----ELATE--LEADC--------I----Q-WSSLQEEA
SLM---
---HHV--------MSQLHRYQD-ERALSAGQK---------------------------
----WKLPP---CGQPIS--------------------------------LFSGSSE---
----SRT---V---

------IRATDD--II-----------------------------FR-------------
VYCADHTYCTLRFNVNATAETIKV--------SAADK-----------LKL-RH-DDLVL
VEVKSS----------------------GERVV------FRDSDVSIPTGLSLNGRIFVS
PKDH---L-DALT-CLAEQ---------------------------EGP------TEGMD
V--DLELFSTKEVAYHMTQFDWDLFWSVHEYELIY-------HTFGRHHF--GQITANLD

```
VFLRR---------FNEIQFWV-----VTELCLTPSLSKR------VQILRKFIKLAAY-
--------------CKEYQNLNAFFAIVM-GLSNVAVSRLSLTWE---------------
-------KLPSKFRKLYTE-FESLI--------------------------DPSRNH---
-RAYRI----SVGK-------LQPPVV-PFMP----------------------------
LLLKDMTFTHEGNK--TC-LD-----------------------------G-LVNFE-KM
-------HM---LAQTMRTIRYCRS--R------HLLL-----------------EPP--
----------------------SPKSE-----A----------------EVRS-----YI
SCLRIIDNQRVL-----------------------------------------TSLSQKL
-----------EPRR----------S----------------------------------
------------------------------------------------------------
------------------------------------------------------------
------------------------------------------------------------
------------------------------------------------------------
------------------------------------------------------------
------------------------------------------------------------
------------------------------------------------------------
------------------------------------------------------------
------------------------------------------------------------
------------------------------------------------------------
```

>Supplementary data 2: Sequence alignment of CBD of PKA/PKG, RAPGEF2/RAPGEF6 and EPACs

```
>PK_[Penicillium_expansum]_1027044025
--SHLDDDQ---FLTVL-NALVEKPIPAKDIK--VISQGDAGDY---FYIVEKGNFDVYI
--HPSGAV---QPGP-DGLGNKV-A---S-TGPG-GSFGELALMYNAPRAATVISTEP--
-----K-STLWALDRITFRRI-LMDSAFQRRRMYE----------
>PK_[Penicillium_griseofulvum]_995936297
--SHLDDDQ---FLTVL-NALVEKPIPAKDIK--VISQGDAGDY---FYIVEKGNFDVYI
--HSSGSV---QPGP-DGLGNKV-A---S-IGPG-GSFGELALMYNAPRAATVISTEP--
-----K-SNLWALDRITFRRI-LMDSAFQRRRMYE----------
>PK_[Penicillium_italicum]_700494201
--SHLDDDQ---FLTVL-NALVEKPIPAKDIK--VISQGDAGDY---FYIVEKGNFDIYI
--HPSGAV---QSGP-DGLGNKV-A---S-TGPG-GSFGELAL-----------------
-------------------------------------------
>PKA_[Acremonium_chrysogenum_ATCC_11550]_672795300
---------------------------GDV---IIKEGDPGHS---FYLLESGEADAYK
--GD--N-----K---------V-L---H-YKKG-DYFGELALLNDAPRAASVIASS---
-----E-VKVATLGKSGFQRL-LGPVEGIMRR-------------
>PKA_[Alternaria_alternata]_399971489
-LSTLTPYE---RSKIA-DALETKKYPPGTT---IIQEGDVGES---FFLLESGEAQVFK
----RGV-------D-----SAV-N---Q-YKKG-DYFGELALLNDAPRAASVISRT---
-----E-VKVATLGKNGFQRL-LGPVEG-----------------
>PKA_[Ascaris_suum]_541044944
---------------------------GTH---VVEQGQPGDE---FFIIVEGEANVLQ
--KRSDD-----APF-----ENV-G---H-LSSS-DYFGEIALLLDRPRAATVVAK----
---T-H-LKCVKLDRARFERV-M----------------------
>PKA_[Aspergillus_calidoustus]_972231926
--------------------------AGST---IISEGDPGDA---FYLLESGEAEAFK
----NGL-----EGP-------V-K---N-YKRG-DFFGELALLDEKPRAASIVA-RT--
-----E-VKVARLGRDGFKRL-LGPVEEIMRRAEYEEK-------
>PKA_[Aspergillus_clavatus_NRRL_1]_121705476
--------------------------ANST---IIAEGDPGDA---FYLLEAGDAEAFM
----KGV-----EGP-------V-K---T-YKRG-DYFGELALLDEKPRAASVVA-KT--
-----D-VKVARLGRDGFKRL-LGPVQDIMRRAEYQAK-------
>PKA_[Aspergillus_fumigatus_Af293]_70999804
--------------------------AGST---IIEEGAPGDA---FYLLESGEAEAFK
----KDV-----EGP-------V-K---S-YRRG-DFFGELALLDDKPRAASVVA-KT--
-----D-VKVARLGRDGFKRL-LGPVEDIMRRAEYSAK-------
>PKA_[Aspergillus_niger_CBS_513.88]_145252346
--------------------------AGST---IIAEGDPGDA---FYLLESGEADAFK
----NGV-----EGP-------V-K---S-YKRG-DYFGELALLDDKPRAASIVA-KT--
-----E-VKVAKLGRDGFKRL-LGPVEDIMRRAEYESKPV-----
>PKA_[Aspergillus_ochraceoroseus]_816333989
--------------------------AGST---IITEGDPGDA---FYLLESGEADAFK
----NGV-----DGP-------V-K---S-YKRG-DYFGELALLDDKPRAASIIT-RT--
-----D-VKVARLGRDGFKRL-LGPVEEIMRRAEY----------
>PKA_[Aspergillus_oryzae_RIB40]_169770467
--------------------------AGSS---IIKEGDPGDA---FYLLESGEAEAFK
----EGV-----DRP-------V-K---S-YQRG-DYFGELALLDDQPRAASIVA-KT--
-----D-VKVAKLGRDGFKRL-LGPVEDIMRRAEYE-Q-------
>PKA_[Aspergillus_ruber_CBS_135680]_599159193
--------------------------AGST---IITEGDPGDA---FYLLESGEAEAYK
----EGE-----EGS-------V-K---D-YKRG-DYFGELALLDDKPRAASVIA-KT--
-----D-VKVARLGRDGFKRL-LGPVEKILRRTEYG---------
```

```
>PKA_[Aspergillus_terreus_NIH2624]_115396126
---------------------------SGST---IITEGDPGDA---FYLLESGEAEAFK
----EGV-----EGN-------V-K---S-YKRG-DFFGELALLDDKPRAASVVA-KT--
-----D-VKVARLGRDGFKRL-LGPVEDIMRRADYQTK-------
>PKA_[Beauveria_bassiana_D1-5]_701775066
----------------------------GAV---IIKEGDPGYS---FYLLEDGTADAYK
--GD--I-----S-------NKV-L---Q-YKKG-DFFGELALLNDAPRAASVVATT---
-----D-VKVATLGKNAFQRL-LGPVEGILRRTKYQG--------
>PKA_[Biomphalaria_glabrata]_908424905
--------------------MFPVHRHAGEV---IIQQGDEGDN---FYVIDQGEVDVYV
----N---------G-----VHV-T---S-IGEG-GSFGELALIYGTPRAATVKAKN---
-----D-VKLWGIDRDSYRRI-L----------------------
>PKA_[Brettanomyces_bruxellensis_AWRI1499]_385302590
-LKSLSSYE---RSKLA-DALNTENYVEGKN---IVTQGEGGEN---FYFIENGTADVIK
--DGK-V---------------V-Q---K-LXKG-DYFGELALLYDSPRQATV-------
---------------------------------------------
>PKA_[Caenorhabditis_elegans]_115534999
--------------------MFPVEKSAGET---IIEQGEEGDN---FYVIDKGTVDVYV
----N---------H-----EYV-L---T-INEG-GSFGELALIYGTPRAATVIAKT---
-----D-VKLWAIDRLTYRRI-L----------------------
>PKA_[Caenorhabditis_remanei]_71835967
--------------------MFPVEKAAQET---IIEQGEEGDN---FYVIDKGTVDVYV
----N---------H-----EYV-L---T-INEG-GSFGELALIYGTPRAATVIAKT---
-----D-VKLWAIDRLTYRRI------------------------
>PKA_[Calliphora_vicina]_158935664
------------------------------------------------------------
----------------------V-A---T-LKSG-DDFGKLALINDAPRAATIVLKEN--
-----N-CHLLRVDKEHFNRI-LRDVEANTLRLQEHGKDVLV---
>PKA_[Candida_albicans_P87]_712863295
-LKSLSDHA---RSKLA-DALSTEMYHKGDK---IVTEGEQGEN---FYLIESGNCQVYN
--EKLGN---------------I-K---Q-LTKG-DYFGELALIKDLPRQATVEALDN--
-------VIVATLGKSGFQRL-LGPVVEVLKEQD-----------
>PKA_[Candida_glabrata]_961821097
---------------------------AGDV---IIREGDRGEN---FYLIEYGACDVTK
--EKEGL---------------V-T---Q-LKDH-DYFGEVALLNDLPRQATV-------
---------------------------------------------
>PKA_[Capronia_coronata_CBS_617.96]_628231581
--------------------------EAGTN---IITEGEPGDS---FYLLESGEAVAYK
----HGV-----DKP-------V-K---E-YKRG-DFFGELALLDDKPRAASVVA-KT--
-----N-VKVARLGRDGFKRL-LGPVESIMRREEY----------
>PKA_[Capronia_epimyces_CBS_606.96]_628273410
---------------------------AGTN---IITEGEPGDS---FYLLESGEAVAYK
----QGI-----DKP-------V-K---E-YKRG-DFFGELALLDDKPRAASVVA-QT--
-----D-VKVARLGRDGFKRL-LGPVESIMRREEY----------
>PKA_[Capsaspora_owczarzaki_ATCC_30864]_754346028
--------------------MVERHVPAGTT---IIKQGDEGDY---FYVVESGKFSVHV
----ERD-----GVS-----KKV-V---E-VGPG-GGFGELALMYNSPRAATVIAD-E--
-----D-STVWGVDRVTFRRI-L----------------------
>PKA_[Ceraceosorus_bombacis]_939395459
---------------------------AGTR---VIEQGERGTE---FFIILDGHAEVQK
--RREQD-----GVE-----EAI-G---K-LAEG-DYFGELALLNNAPRAASIVAS----
---SPA-LRLATLSESAFKRL-V----------------------
>PKA_[Ceratocystis_platani]_814601286
-LSSLTPYE---RSKIA-DALEQERFSAGQS---IITEGDVGQT---FYIVESGEADALK
```

----G---------D-----TIV-K---N-YKKG-EFFGELALLNDAPRAATVVART---
-----N-TKVVSLGKSAFQRL-LGPVEG-----------------
>PKA_[Chaetomium_thermophilum_var._thermophilum_DSM_1495]_576043404
---------------------------AGHT---IIREGDPGYD---FYLLESGEAVAYR
--SDNDQ-----P---------V-K---H-YKKG-DYFGELALLNDAPRAASVVSTT---
-----E-VKVARLGKSAFQRL-LGPVESIMRRQKY----------
>PKA_[Chrysochromulina_sp._CCMP291]_922861303
----------------------------------VFEQGDQGST---FYIIYQGGVKVWV
N---DGKGVSKVNDG--GYGTCV-A---T-LLEG-DSFGELALIGNGTRSGTAITTG---
-----P-TQLLRVEKDAYD-------------------------
>PKA_[Cladophialophora_psammophila_CBS_110553]_628318878
-LSSLKPYERAKIADAL-ETVK---YESGQN---IITEGEPGDA---FYLLESGHAVAYK
----HGV-----DHP-------V-K---E-YGRG-DFFGELALLDDKPRAASVVA-KD--
-----N-VTVAKLGRDGFKRL-LGPVESIMRREEYE---------
>PKA_[Cladophialophora_yegresii_CBS_114405]_628289763
-------------------------FEPGQN---IITEGEPGDA---FYLLESGHAVAYK
----HGV-----DHA-------V-K---E-YGRG-DFFGELALLDDKPRAASVIA-KD--
-----N-VTVAKLGRDGFKRL-LGPVEGIMRREEY----------
>PKA_[Colletotrichum_gloeosporioides_Nara_gc5]_596690365
----------------------------GET---IIKEGDPGHS---FYLLESGEADAYI
--GD--S-----K-------EAV-K---H-YSKG-DFFGELALLNDAPRAASIVATT---
-----D-VKVASLGKSAFQRL-LGPVEGIMRR-------------
>PKA_[Colletotrichum_gloeosporioides]_375005170
----------------------------GET---IIKEGDPGHS---FYLLESGEADAYI
--GD--S-----K-------EAV-K---H-YSKG-DFFGELALLNDAPRAASIVATT---
-----D-VKVASLGKSAFQRL-LGPVEGIMRR-------------
>PKA_[Colletotrichum_nymphaeae_SA-01]_996635092
----------------------------GET---IIKEGDPGHA---FFLLESGEADAYK
--GG--V-----K-------ESV-K---H-YSKG-DFFGELALLNDAPRAASIVATT---
-----D-VKVASLGKNAFQRL-LGPVEGIMRRTKYEEV-------
>PKA_[Cordyceps_confragosa_RCEF_1005]_1025692434
----------------------------GAV---IIKEGDPGYS---FYLLEDGTADAYK
--GD--T-----N-------NKV-L---Q-YKKG-DFFGELALLNDAPRAASVVATT---
-----D-VKVATLGKNAFQRL-LGPVEGILRRTKYQG--------
>PKA_[Cordyceps_militaris_CM01]_573987763
----------------------------GAV---IIKEGDPGYS---FYLLEDGTADAYK
--GD--T-----S-------NKV-L---Q-YKKG-DFFGELALLNDAPRAASVVATT---
-----D-VKVATLGKNAFQRL-LGPVEGILRRTKYQG--------
>PKA_[Crassostrea_gigas]_762095734
--SHLDDNE---RSDIF-DAMFPVHRHAGEV---IIQQGDEGDN---FYVIDQGEVDIFV
----N---------D-----EHV-T---T-IGEG-GSFGELALIYGTPRAATVKAKG---
-----D-VKLWGIDRDSYRRI-LMGSTIRKRKMYEEFLGKV----
>PKA_[Cryptococcus_bacillisporus_CA1280]_757342465
----------------------EVKANAGQM---VIEQGAAGDF---FYIVESGKLDVFI
--KREGQILDLEKGDRPGLGMKI-A---E-CSEG-SSFGELALMHNAPRAASIISV-T--
-----P-CTLWALDRVSFRTI-LLDHTSRKRRLYE----------
>PKA_[Cryptococcus_tetragattii_IND107]_757381277
----------------------EVKANAGQM---VIEQGAAGDF---FYIVESGKLDVFI
--QREGQILDLEKGDRPGLGMKV-A---E-CSEG-SSFGELALMHNAPRAASIVSV-T--
-----P-CTLWALDRVSFRTI-LLDHTSRKRRLYE----------
>PKA_[Cryptosporidium_muris_RN66]_209881331
-----------------------------TV---IINQGDDGDK---LYIIEKGQVDCFK
--EFKDS-----SER-----KHL-C---S-LRSG-DAFGELALLYNCPRAATVIASS---
-----D-CLLWALDRETFNHI-VKGAAAKRIETY-----------

>PKA_[Cryptosporidium_parvum_Iowa_II]_126651910
------------------------IFEDGQE---IIKQGEQGDT---FYLIITGNAVALK
--DNVE--------------VM------S-YKRG-DYFGELALLRNAPRAATVKARG---
-----R-CKVAYLDRKAFKRV-LGPIE------------------
>PKA_[Curvularia_lunata]_565619410
-LSTLTPYE---RSKIA-DALETKKYPPGTT---IIQEGDVGES---FFLLEAGEAQVFK
----RGV-------D-----GPV-N---Q-YQKG-DYFGELALLNDAPRAASVISKT---
-----E-VKVATLGKNGFQRL-LGPVEG-----------------
>PKA_[Danaus_plexippus]_357624754
--------------------MFPVQCLQGET---VIRQGDEGDN---FYIIDSGEVEVLV
----N---------G-----EPV-T---T-IGEG-GSFGELALIYGTPRAATVRART---
-----P-LKLWGLDRDSYRRI-L----------------------
>PKA_[Diaporthe_ampelina]_821076706
----------------------------GTA---VIKEGDPGYD---FYLLESGECDAYK
--EGVHK-----ERI-----N-L-R---H-YEKG-DFFGELALLNDAPRAATVVSVT---
-----E-VKVAKLGKNAFNRL-LGPVESIMRR-------------
>PKA_[Dictyostelium_purpureum]_330841828
--------------------------KAGDI---IIKQGDEGDL---FYVVDKGICDIYV
--STNGS------TP-----TLV-M---E-VFEG-GSFGELALIYGSPRAATVIART---
-----D-VRLWALNGSTYRRI-LMDQTIRKRRLYEE---------
>PKA_[Drosophila_melanogaster]_28574658
--AHLDESE---RSDIF-DAMFPVNHIAGEN---IIQQGDEGDN---FYVIDVGEVDVFV
----N---------S-----ELV-T---T-ISEG-GSFGELALIYGTPRAATVRAKT---
-----D-VKLWGIDRDSYRRI-L----------------------
>PKA_[Echinococcus_granulosus]_576698744
--------------------MFPLHRSRGDI---IIKQGDDGDN---FYIIDQGKVDIYV
----N---------G-----EFV-S---C-IGEG-GSFGELALIYGTPRAATVKAHS---
-----DEVKLWGIDRDSYRRI-LMGSTIRRRKMYQDFL-------
>PKA_[Eimeria_necatrix]_921116540
---------------------------AGDT---IIREGEAGDT---FYLLLDGEAEAVK
--GGKV----------------V-M---K-YSRG-SYFGELALLKNQPRAATVTAK----
---T-D-CKVAYMDRRSFKRL-LGPLEGLLMRNMDHYRAV-----
>PKA_[Eimeria_tenella]_916419130
---------------------------AGDT---IIREGEAGDT---FYLLLDGEAEAVK
--GGKV----------------V-M---K-YSRG-SYFGELALLKNQPRAATVTAK----
---T-D-CKVAYMDRRSFKRL-LGPLEGLLMRNMDHYRAV-----
>PKA_[Exophiala_dermatitidis_NIH/UT8656]_684170162
---------------------------AGTN---IITEGEPGDS---FYLLEAGEAAAYK
----QGI-----DGP-------V-K---E-YKRG-DFFGELALLDDKPRAASVVA-KT--
-----D-VKVAKLGREGFKRL-LGPVESIMRREEY----------
>PKA_[Fusarium_avenaceum]_751358440
----------------------------GEV---IINEGDPGHA---FYLLESGEADAYI
--GQ--P-----D-------NKV-R---H-YKKG-DYFGELALLNDAPRAASIVASS---
-----A-VKVGSLGKNAFQRL-LGPVEGILRR-------------
>PKA_[Fusarium_langsethiae]_927760275
----------------------------GEI---IINEGDPGHA---FYLLESGEADAYI
--GQ--S-----D-------NKV-R---H-YKKG-DYFGELALLNDAPRAASIVATS---
-----T-VKVGSLGKNAFQRL-LGPVEGILRRTKYQG--------
>PKA_[Fusarium_oxysporum_f._sp._cubense_race_4]_475665949
-LASLNPYE---RSKIA-DALETKKFAAGEV---IINEGDPGHA---FYLLESGEADAYI
--GQ--P-----D-------NKV-R---H-YKKG-DYFGELALLNDAPRAASIVATS---
-----P-VKVGSLGKNAFQRL-LGPVEGILRRTK-----------
>PKA_[Gaeumannomyces_graminis_var._tritici_R3-111a-1]_685408583
-------------------------------------QGDVGDY---FYVVEKGSFNVYK

--NPTGA---MQPGP-DGLGKHE-G---T-VAEG-GYFGEIALMHNALRNATVMSAEA--
-----N-CVLWALDRVTFNRI-LMETNSARYQMYDK---------
>PKA_[Hammondia_hammondi]_675125108
----------------------------GDV---IIREGETGDT---FYILLEGAAEAIK
--NDKV----------------V-M---E-YKKG-GFFGELALLKDQPRAATVVAK----
---S-H-VQVAYMDRKSFKRL-LGPVEQILMRNQDN---------
>PKA_[Hanseniaspora_valbyensis_NRRL_Y-1626]_1037355805
-LSNLTESE---RNKLI-DVLKIKTVPKGSV---IIRQGEMGEN---FYIIEQGSCVVIK
----DN--------V-----E-L-K---A-LHKN-DYFGELALLDDSPRAATVVASSEE-
-----G-CKLVYLGKSEFKRL-L---------------------
>PKA_[Kluyveromyces_marxianus_DMKU3-1042]_574141192
----LSTYD---RAKLA-DALDTEYYEAGDV---IIKEGDTGEN---FYFIEYGEAEVSK
----EN--------Q-----GVI-T---K-LGKG-DYFGEVALLNDLPRQATV-------

>PKA_[Lasius_niger]_861608592
---------------------------PGEA---IIRQGDEGDN---FYVIDQGEVEIFV
----N---------G-----ELA-T---T-IGEG-GSFGELALIYGTPRAATVRAKT---
-----D-VKLWGIDRDSYRRI-L---------------------
>PKA_[Leptosphaeria_maculans_JN3]_396496360
-LSTLTPYE---RSKIA-DALETKKYPPGST---IIQEGDVGES---FYLLESGDAQVFK
----RGI-------E-----TAV-K---E-YTKG-DYFGELALLNDAPRAASVVSRT---
-----E-VKVATLGKNGFQRL-LGPVEG-----------------
>PKA_[Lichtheimia_corymbifera_JMRC:FSU:9682]_661180422
--------------------MAEKRVKSGEE---VIEQGAQGDY---YYVVEQGTFDCLI
----NGNK--------------V-T---S-YGPG-GSFGELALMYNAPRAATIVATS---
-----D-GVLWALDRITFRSI-LMDSTARKRRMYEQ---------
>PKA_[Limulus_polyphemus]_926620498
------------------------------V---IIHQGDEGDN---FYVIDQGEVEVFV
----N---------G-----QMV-T---T-IGEA-GSFGELALIYGTPRAATVKAKT---
-----N-VKLWAIDRDTYRRI-LMGSTIRKRKMYEEFL-------
>PKA_[Lingula_anatina]_919061114
---------------------------SGEV---IIQQGDEGDN---FYVIDQGEVDIYV
----N---------N-----EHV-T---N-IGEG-GSFGELALIYGTPRAATVKAKT---
-----D-VKLWGIDRQTYRRI-LMGSTMR----------------
>PKA_[Loa_loa]_312084002
--------------------MFPVEKKKGET---IIEQGEEGDN---FYVIDSGEVDVFV
----N---------G-----EYA-L---S-IKEG-GSFGELALIYGTPRAATVVAKSD--
-----V-VKCWAIDRITYRQI-L---------------------
>PKA_[Lycosa_singoriensis]_161669170
--AHLDDTE---RSDIF-DAMFPVVHKAGEV---IITQGDQGDN---FYVIDQGEVEVFV
----N---------G-----QLV-V---T-IGEA-GSFGELALIYGTPRAATVKAKI---
-----D-CKLWAIDRDTYRRI-L---------------------
>PKA_[Madurella_mycetomatis]_1003743647
-LSSLTPYE---RSKIA-DALESQKFPAGHT---IIREGDPGHS---FFLLESGEAVAYR
--AGNET-----P---------V-K---H-YKKG-DFFGELALLNDAPRAASVVSET---
-----E-VKVATLGKSAFQRL-LGPVEGIMRRTK-----------
>PKA_[Magnaporthe_oryzae_70-15]_389635839
----------------------------GTV---VIKEGDPGED---FYLLECGEAEAFK
----AGI-------D-----QPV-K---L-YKKG-DFFGELALLNDAPRAASVVSKT---
-----E-VKVAALGKSAFQRL-LGPVE------------------
>PKA_[Magnetospirillum_gryphiswaldense_MSR-1]_144898590
---------------------------AGQK---VFGEGDSGDR---AYLIQEGSVEISK
----HGLV--------------L-A---T-LNKG-ELFGEMALVDDQPRMAT--------

```
>PKA_[Marssonina_brunnea_f._sp._'multigermtubi'_MB_m1]_597579793
---------------------------AGTT---IIKEGDAGEA---FYLLESGEAEAYK
----MDV-------Q-----NPV-K---S-YKKG-DFFGELALLNDAPRAASVVSKT---
-----E-VKVATLGKDGFQRL-LGPVESIMRRTTYDHG-------
>PKA_[Metarhizium_album_ARSEF_1941]_734659332
----------------------------GDV---IIREGDQGHA---FFLLESGEADAFK
--GDQSN-----K---------V-L---H-YKKG-DFFGELALLNDKPRAASVVAST---
-----E-VKVATLGKHAFQRL-LGPLEG-----------------
>PKA_[Metarhizium_anisopliae]_672378012
-LSSLTPYE---RSKIA-DALETKKYTPGEI---IIREGDPGHS---FFLLESGEADAFK
--GDQSN-----K---------V-L---H-YKKG-DFFGELALLNDQPRAASVMAST---
-----E-VKVATLGKNAFQRL-LGPVEG-----------------
>PKA_[Metarhizium_robertsii_ARSEF_23]_629717739
-LSSLTPYE---RSKIA-DALETKKYAPGEI---IIREGDPGHS---FFLLESGEADAFK
--GDQSN-----K---------V-L---H-YKKG-DFFGELALLNDQPRAASIMAST---
-----E-VKVATLGKNAFQRL-LGPVEG-----------------
>PKA_[Mitosporidium_daphniae]_914960361
------------------------------V---VIREGDIGDS---FYIVSQGNANVCI
----NGK-----QEK-----SLV-I--------G-DYFGEMSLITKQPRAATVIAQSN--
-----P-LSVLRIEVDAFNRL-L----------------------
>PKA_[Mucor_ambiguus]_758351316
----------------------EKRVAKGTT---VIEQGDVGDF---FYVVESGTLDCFI
----GQNK--------------V-T---N-YEAG-GSFGELALMYNAPRAATIITTS---
-----D-SVLWALDRITFRTI-LMENTSRKRRMYEY---------
>PKA_[Mucor_circinelloides_f._circinelloides_1006PhL]_511004461
----------------------------GQE---VVKQGDVGDQ---FYIIESGEAIVLK
--EENGIQQQ------------V-N---Q-LERG-SYFGELALLNDAPRAATVV------
---------------------------------------------
>PKA_[Mucor_racemosus]_119712284
----------------------------GQE---VVKQGDVGDQ---FYIIESGEAIVLK
--EENGVQQQ------------V-N---Q-LERG-SYFGELALLNDAPRAATVV------
---------------------------------------------
>PKA_[Neofusicoccum_parvum_UCRNP2]_615406214
-LSSLTPYE---RSKIA-DALETKKYPAGTT---IIQEGDVGES---FYILETGEAEVYK
----RGI-------D-----KPV-K---R-YSKG-DYFGELALLNDAPRAASVVSTR---
-----E-IKVATLGKDGFQRL-LGPVEG-----------------
>PKA_[Neonectria_ditissima]_936414681
---------------------------SGEI---IIKEGDPGHA---FYLLENGQAAAYK
--GD--P-----S-------NKV-L---D-YDKG-DYFGELALLNDAPRAASVVATT---
-----D-VKVASLGKNAFQRL-LGPVEGIMRRTE-----------
>PKA_[Neospora_caninum_Liverpool]_401405056
----------------------------GDV---IIKEGETGDT---FYILLEGTAEAIK
--NDKV----------------V-M---E-YKKG-GFFGELALLKDQPRAATVVAK----
---S-H-VQVAYMDRKSFKRL-LGPVEQILMRNQDN---------
>PKA_[Neurospora_tetrasperma_FGSC_2508]_698982218
---------------------------AGHE---IILEGDPGHS---FFLLEAGEAAAFK
--RG--N-----D-------SPV-K---N-YKKG-DFFGELALLNDAPRAASVISQT---
-----E-VKVARLGKNAFQRL-LGPIESILRR-------------
>PKA_[Octopus_bimaculoides]_961135686
--SHLDDNE---RSDIF-DAMFPVHRHAGEV---IIQQGDDGDN---FYVIDQGEVDVYV
----N---------N-----EHV-T---T-IGEG-GSFGELALIYGIPRAATVKAKN---
-----D-VKLWGIDRDSYRRI-L----------------------
>PKA_[Ogataea_parapolymorpha_DL-1]_927375419
----LSSYE---RSKLA-DALNSEFYSVGQN---VVTEGEAGEN---FYFIESGTADVIK
```

--SGEGV---------------V-S---K-LNKG-DYFGELALLYDSPRQATV-------

>PKA_[Ophiocordyceps_sinensis_CO18]_531867378
----------------------------GEI---IIKEGDPGHS---FYLLESGEAVAYK
--GD--P-----S-------NRL-L---Q-YKKG-DYFGELALLNDAPRAASIMAST---
-----D-VKVASLGKNAFQRL-LGPVEGILRRTK-----------
>PKA_[Ophiostoma_piceae_UAMH_11346]_512187331
----------------------------GTN---IISEGDPGHA---FFLVESGEADAIK
--SG--V-----P-------DPV-L---H-YKKG-DFFGELALLNDAPRAATIVAKT---
-----D-VKVATLGKSAFQRL-LGPVEGIMRR-------------
>PKA_[Oryctolagus_cuniculus]_6002552
---------------------------AGET---VIQQGDEGDN---FYVIDQGEMDVYV
----N---------N-----EWA-T---S-VGEG-GSFGELALIYGTPRAATVKAKT---
-----N-VKLWGIDRDSYRRI-L---------------------
>PKA_[Paramecium_tetraurelia]_1513244
------------ELAIVIDAMEEKKFKAGDF---VIKQGDDGDV---LYVVDQGQLDCFK
VFKKGEP-------E-----KHL-K---V-YQPG-ESFGELALLYNVPRAATIKAKT---
-----D-AICFSLDRETFNHI-VKDAAAK----------------
>PKA_[Pediculus_humanus_corporis]_242003576
---------------------------AGSV---ILKQGDEGSL---FYIITGGTVSVTI
--TQPD--------------GTV-KQGPI-LKTG-DFFGEKALLKDEKRAATVIAQEP--
-----G-VECLTLDRIHF---------------------------
>PKA_[Penicillium_digitatum_Pd1]_953384115
---------------------------ANST---IIHEGDPGDA---FYLLESGEAEATK
----NGV-------S-----GPV-K---N-YHRG-DYFGELALLDDKPRQASITT-KT--
-----D-VKVARLGRDGFKRL-LGPVEGMMRGAEY----------
>PKA_[Penicillium_roqueforti_FM164]_584417054
-----------------------------SI---IIHEGDPGDA---FYLLESGEAEATK
----NGV-------S-----GPV-K---S-YHRG-DYFGELALLDDKPRQASITT-KT--
-----D-VKVARLGRDGFKRL-LGPVEGMMRGAEYELD-------
>PKA_[Perkinsus_marinus_ATCC_50983]_294899358
----------------------------GEV---VCHKGEEGNE---FFLIFHGAVNIFP
--VPGEKP--------------V-A---T-YVAG-ETFGELALLHDAPRAATVV------

>PKA_[Phaeoacremonium_minimum_UCRPA7]_631232375
-LSTLTPYE---RSKIA-DALETQKYPPGTE---IIREGDPGTD---FFLLESGECDAYK
----TGN-------P-----NSV-K---H-YARG-DFFGELALLNDAPRLASVVSKT---
-----E-VKVAALGKSAFQRL-LGPVESIMRRTK-----------
>PKA_[Phaeomoniella_chlamydospora]_821066414
---------------------------AGSK---IINEGDPGDA---FYLLESGEAEAFK
----AGV-----EKP-------V-K---A-YSRG-DYFGELALLDDKPRAASVIA-KT--
-----S-VKVARLDREGFKRL-LGPVESIMRREDY----------
>PKA_[Plasmodium_berghei_ANKA]_675229101
---------------------------AGDV---IINEGEQGDT---FYILTDGNATALK
--NCQ----------------II-K---T-YTKG-DYFGELALLRNQPRAATVKAES---
-----T-CQVVYLERKGFKRL-LGPIEKILIRNVENYKKVL----
>PKA_[Plasmodium_chabaudi_chabaudi]_56522589
---------------------------AGDI---IINEGERGDT---FYILTYGNATALK
--SDQ----------------VI-K---T-YTKG-DYFGELALLRNKPRAATVKADG---
-----V-CQVVYLERKGFKRL-LGPIEKILIRNVENYKQVL----
>PKA_[Plasmodium_coatneyi]_1042343877
------------------------------I---IIKEGEPGDT---FYIIIEGNALAIK
--GKT----------------VI-K---T-YSKG-DYFGELALLKNKPRAATVKAKD---
-----S-CQVVYLDRKSFKRL-LGPIEEILHRNVENYRKVL----

>PKA_[Plasmodium_gaboni]_1011103433
----------------------------GEI---IIKEGEEGDT---FFILIDGNAVASK
--DNK----------------VI-K---T-YAKG-DYFGELALLKNKPRAATIKAQN---
-----F-CQVVYLDRKSFKRL-LGPIEDILHRNVENYKQVL----
>PKA_[Plasmodium_inui_San_Antonio_1]_672187601
----------------------------------IIKEGEQGDT---FYIIIDGKALAIK
--DKK----------------VI-K---T-YSKG-DYFGELALLKNQPRAATVKAKD---
-----T-CQVVYLDRKSFKRL-LGPIEEILYRNVENYRKVL----
>PKA_[Plasmodium_knowlesi_strain_H]_1041816653
------------------------------V---IIKEGEPGDT---FYIIVDGSALAIK
--DKT----------------VI-K---T-YSKG-DYFGELALLKNQPRAATVKAKD---
-----S-CQVVYLDRKSFKRL-LGPIEEILHRNVENYKKVL----
>PKA_[Plasmodium_malariae]_1037137315
----------------------------GDI---IINEGEQGDT---FYILIEGNATALK
--DNK----------------VI-K---T-YNKG-DYFGELALLKNQPRAATVKAQN---
-----T-CQVVYLDRKSFKRL-LGPIEEILHRNVENYKKVL----
>PKA_[Plasmodium_ovale_curtisi]_1036545630
----------------------------GDI---IINEGEQGNT---FYILIDGNATALK
--DNK----------------VI-K---T-YNKG-DYFGELALLKNQPRAATVKAQN---
-----N-CQVVYLDRKSFKRL-LGPIEKILYRNVENYKKVL----
>PKA_[Plasmodium_vinckei_petteri]_577151418
--------------------------KTGDI---IINEGEQGDT---FYILIDGKATALK
--SDQ----------------VI-K---T-YAKG-DYFGELALLRNQPRAATVKAES---
-----T-CQVVYLERKGFKRL-LGPIEKILIRNVENYKQVL----
>PKA_[Plasmodium_vivax_Sal-1]_156103253
----------------------------------IIKEGEPGDT---FYIIVEGNALAIK
--DKT----------------VI-K---T-YGKG-DYFGELALLKNKPRAATVKAKD---
-----T-CQVVYLDRKSFKRL-LGPIEEILHRNVENYRQVL----
>PKA_[Plasmodium_yoelii_yoelii_17XNL]_83318121
----------------------------GDI---IINEGEQGDT---FYILIDGKATALK
--NGQ----------------VI-K---T-YTKG-DYFGELALLRNQPRAATVKAES---
-----T-CQVVHLERKGFKRL-LGPIEKILIRNVENYKKVL----
>PKA_[Plasmodium_yoelii]_675234964
----------------------------GDI---IINEGEQGDT---FYILIDGKATALK
--NGQ----------------VI-K---T-YTKG-DYFGELALLRNQPRAATVKAES---
-----T-CQVVHLERKGFKRL-LGPIEKILIRNVENYKKVL----
>PKA_[Priapulus_caudatus]_957826758
--------------------MFPVHHKAGEV---VIQQGDEGDN---FYVIDDGQVDIYV
----N---------D-----KHV-V---A-VSEG-GSFGELALIYGTPRAATVKAKT---
-----D-LKLWGIDRDSYRRI-LMGSTIRKRKMYEDFL-------
>PKA_[Pyrenophora_tritici-repentis_Pt-1C-BFP]_189192997
----------------------------GTT---IIQEGDVGES---FFLLESGEAQVFK
----RGI-------D-----SAV-R---E-YHKG-DYFGELALLNDAPRAASVVSKT---
-----E-VKVATLGKNGFQRL-LGPVEGIMRR-------------
>PKA_[Riptortus_pedestris]_501292686
----------------------------GET---IIQQGDEGDN---FYVIDQGEVEVYV
----N---------S-----ELV-T---T-IGEG-SSFGELALIYGTPRAATVKAKT---
-----A-VKLWGIDRDSYRRI-LMGSTIRKRKMYE----------
>PKA_[Rosellinia_necatrix]_949385799
---------------------------AGTT---IIRQGDIGQE---FYLVESGEADAFK
--NG--E-----P-------DSV-K---H-YEKG-DFFGELALLNDAPRAASVISAT---
-----E-IKVATLGKSAFARL-LGPVEGIMRRTRYIGVV------
>PKA_[Saccharomyces_cerevisiae_S288c]_6322156
--------------------------QPGET---IIREGDQGEN---FYLIEYGAVDVSK

--KGQGV---------------I-N---K-LKDH-DYFGEVALLNDLPRQATV-------

>PKA_[Schistocerca_gregaria]_311359099
--------------------MFPVNALPGET---IIQQGDEGDN---FYVIDQGEVEVFV
----N---------S-----ELV-T---T-IGDG-GSFGELALIYGTPRAATVRAKT---
-----D-VKLWGIDRDSYRRI-L----------------------
>PKA_[Schistosoma_haematobium]_844855164
--------------------MFPVHRNSGDV---IIQQGDEGDN---FYIIDQGEVDIFL
----N---------N-----EYS-S---T-IGEG-GSFGELALIYGTPRAATVRAKT---
-----E-VKLWGIDRDSYRRI-L----------------------
>PKA_[Schizosaccharomyces_cryophilus_OY26]_891585810
---------------------------AGGI---VIRQGDIGNQ---FYLIEDGEAEVCK
----EG--------K-----GVV-V---T-LGKG-DYFGELALIHETERTATVRAKT---
-----R-LKLATFDKPTFNRL-L----------------------
>PKA_[Schizosaccharomyces_japonicus_yFS275]_213403454
-LSKLSNYE---RQKIA-DALQTVVYPEGSI---VVRQGDEGEN---FYLIESGEAEVIK
----EG--------Q-----GVI-A---I-LTKG-EYFGELALIYKTVRNATVRART---
-----R-LKLATFDKAAFNRL-LGNVIDTMR--------------
>PKA_[Spathaspora_passalidarum_NRRL_Y-27907]_598068477
----LSDHE---RSKLA-DALWTEMYHAGDK---IVTEGDQGEN---FYLIESGNCDVYS
--QSGGH---------------L-A---R-LTKG-DYFGEVALLNDLPRQATVQALDN--
-------VIVATLGKSGFQRL-L----------------------
>PKA_[Sporothrix_brasiliensis_5110]_748537701
-LATLTPYE---RSKIA-DALETQKFAAGAT---IIREGDLGHS---FYLVESGEATVYK
--SGTES-----P---------V-K---H-YQKG-DFFGELALLNDAPRAATIVAKT---
-----D-IKVATLGKSAFQRL-LGPVEGIMRRTR-----------
>PKA_[Stegodyphus_mimosarum]_675373908
--AHLDDTE---RSDIF-DAMFPVVHSAGET---IIQQGDQGDN---FYVIDQGEVEVFV
----N---------G-----QLV-T---T-IGEG-GSFGELALIYGTPRAATVRAKT---
-----D-CKLWAIDRDTYRRI-L----------------------
>PKA_[Sugiyamaella_lignohabitans]_1026313839
------------------------VYEPGSV---VIREGDKGDE---FYLIESGTATVSK
--ANEGT---------------V-S---E-LKKG-DYFGEVALLHDSPRQATV-------

>PKA_[Talaromyces_cellulolyticus]_748554364
---------------------------AGST---IIREGDPGDA---FYLLESGEAEAVK
----AGT-------T-------V-K---E-YSRG-DYFGELALLDDKPRAASVMA-KT--
-----D-VKVARLGRDGFKRL-LGPVEQIMRRTEY----------
>PKA_[Talaromyces_marneffei_ATCC_18224]_212528198
---------------------------AGST---IIHEGDPGDA---FYLLEAGEAEAVK
----AGT-------R-------V-K---D-YSRG-DYFGELALLDDKPRAASVMA-KT--
-----D-VKVARLGRDGFKRL-LGPVEQIMRRTEY----------
>PKA_[Talaromyces_stipitatus_ATCC_10500]_242767083
---------------------------AGST---IIREGDPGDS---FYLLESGEAEAVK
----AGT-------T-------V-K---E-YSRG-GYFGELALLDEKPRAASVIA-KT--
-----D-VKVARLGRDGFKRL-LGPVEQIMRRTEY----------
>PKA_[Tetrahymena_thermophila_SB210]_146161423
----------------------EKKFKAGET---IIKQGDDGDE---LYVVDSGLLDCYK
--EKANQ-------E-----KILLK---T-YKEG-EAFGELALLYNAPRAATIIAKT---
-----D-CILFSLDRPTFNHI-VKDAAAK----------------
>PKA_[Theileria_annulata_strain_Ankara]_84994998
-------------------------------------QGEPGSS---LFMVLEGQAESFV
--ENK-L---------------V-K---S-YNPG-DYFGEIGFILKKPRASTVKAKGK--
-------CLFVELERENFINL-LGPME------------------

```
>PKA_[Theileria_orientalis_strain_Shintoku]_697890559
-------------------------ANAGDV---LVKQGDDGDK---LYLIESGTADVTR
--SSKLS-----G-N-----EFL-T---T-LKDG-DYFGELALMYNAPRAATVTA-KT--
-----E-MRLWTLDRTTFNYV-VK--------------------
>PKA_[Theileria_parva_strain_Muguga]_71030836
-------------------------------------QGEPGSS---LFMVLEGQAESFV
--ENK-L---------------V-K---S-YNPG-DYFGEIGFILKKPRASTVKAK----
------------LERENFINL-----------------------
>PKA_[Tolypocladium_ophioglossoides_CBS_100239]_908388170
-LSSLTPYE---RSKIA-DALETQKFAPGEI---IIKEGDPGHS---FYLLESGEADAYK
--GDSNN-----K---------V-L---H-YKKG-DFFGELALLNDAPRAASVVASS---
-----D-VKVATLGKNAFQRL-LGPVEG----------------
>PKA_[Torrubiella_hemipterigena]_729181934
---------------------------AGEL---IIKEGDPGYS---FYLLESGEADAYI
--GEPGN-----K---------V-K---H-YTKG-DYFGELALLNDEPRAASVVAGT---
-----D-VKVATLGKNAFQRL-LGPVEGIMRR------------
>PKA_[Toxocara_canis]_734555419
--------------------MFPVEKKKGET---IIEQGEEGDN---FYVIDSGEVDVFV
----N---------N-----EYA-V---S-IKEG-GSFGELALIYGTPRAATVIAKSD--
-----V-VKCWAIDRITYRQI-L---------------------
>PKA_[Toxoplasma_gondii_ARI]_1005150459
----------------------------GDV---IIKEGETGDT---FYILLEGAAEAIK
--NDKV----------------V-M---E-YKKG-GFFGELALLKDQPRAATVVAK----
---S-H-VQVAYMDRKSFKRL-LGPVEQILMRNQDN---------
>PKA_[Toxoplasma_gondii_FOU]_672257803
----------------------------GDV---IIKEGETGDT---FYILLEGAAEAIK
--NDKV----------------V-M---E-YKKG-GFFGELALLKDQPRAATVVAK----
---S-H-VQVAYMDRKSFKRL-LGPVEQILMRNQDN---------
>PKA_[Toxoplasma_gondii_GAB2-2007-GAL-DOM2]_672257581
----------------------------GDV---IIKEGETGDT---FYILLEGAAEAIK
--NDKV----------------V-M---E-YKKG-GFFGELALLKDQPRAATVVAK----
---S-H-VQVAYMDRKSFKRL-LGPVEQILMRNQDN---------
>PKA_[Toxoplasma_gondii_MAS]_672560367
----------------------------GDV---IIKEGETGDT---FYILLEGAAEAIK
--NDKV----------------V-M---E-YKKG-GFFGELALLKDQPRAATVVAK----
---S-H-VQVAYMDRKSFKRL-LGPVEQILMRNQDN---------
>PKA_[Toxoplasma_gondii_ME49]_237834899
----------------------------GDV---IIKEGETGDT---FYILLEGAAEAIK
--NDKV----------------V-M---E-YKKG-GFFGELALLKDQPRAATVVAK----
---S-H-VQVAYMDRKSFKRL-LGPVEQILMRNQDN---------
>PKA_[Toxoplasma_gondii]_12698442
----------------------------GDV---IIKEGETGDT---FYILLEGAAEAIK
--NDKV----------------V-M---E-YKKG-GFFGELALLKDQPRAATVVAK----
---S-H-VQVAYMDRKSFKRL-LGPVEQILMRNQDN---------
>PKA_[Tribolium_castaneum]_642916971
--------------------MFPVTCLPGEA---IIQQGDEGDN---FYVIDQGEVEVYV
----N---------N-----ELV-T---T-IGDG-GSFGELALIYGTPRAATVKAKT---
-----D-VKLWGIDRDSYRRI-LMGSTIRKRKMYEE---------
>PKA_[Trichinella_papuae]_954602153
--AHLDENE---KKDIF-NAMFPVEANAGEV---IIQQGDEGDN---FYVIDSGEVEVFV
----N---------N-----KSV-T---T-IKEN-GSFGELALIYGTPRAATVLAKT---
-----R-VKLWALDRDTYRRI-L---------------------
>PKA_[Trichinella_patagoniensis]_954351355
--AHLDENE---KKDIF-NAMFPVEANAGEV---IIQQGDEGDN---FYVIDSGEVEVFV
```

----N---------N-----KSV-T---T-IKES-GSFGELALIYGTPRAATVLAKT---
-----R-VKLWALDRDTYRRI-L----------------------
>PKA_[Trichinella_sp._T8]_954620704
--AHLDENE---KKDIF-NAMFPVEANAGEV---IIQQGDEGDN---FYVIDSGEVEVFV
----N---------N-----KSV-T---T-IKES-GSFGELALIYGTPRAATVLAKT---
-----R-VKLWALDRDTYRRI-L----------------------
>PKA_[Trichinella_sp._T9]_954270144
--AHLDENE---KKDIF-NAMFPVEANAGEV---IIQQGDEGDN---FYVIDSGEVEVFV
----N---------N-----KSV-T---T-IKES-GSFGELALIYGTPRAATVLAKT---
-----R-VKLWALDRDTYRRI-L----------------------
>PKA_[Trichinella_zimbabwensis]_954489314
--AHLDENE---KKDIF-NAMFPVEANAGEV---IIQQGDEGDN---FYVIDSGEVEVFV
----N---------N-----KSV-T---T-IKEN-GSFGELALIYGTPRAATVLAKT---
-----R-VKLWALDRDTYRRI-L----------------------
>PKA_[Valsa_mali_var._pyri]_972126529
----------------------------GTA---IINQGDPGYD---FYLLESGECDAYK
--EGVHK-----ERI-----N-L-R---H-YEKG-DFFGELALLNDAPRAATVSSVT---
-----E-VKVAKLGKNAFQRL-LGPVESIMRR-------------
>PKA_[Valsa_mali]_972146471
----------------------------GTA---IINQGDPGYD---FYLLESGECDAYK
--EGVHK-----ERI-----N-L-R---H-YEKG-DFFGELALLNDAPRAATVSSVT---
-----E-VKVAKLGKNAFQRL-LGPVESIMRR-------------
>PKA_[Vicugna_pacos]_560996168
-------------------------K-AGET---IIEQGEEGDN---FYVIDKGVVDVFV
----N---------N-----EFV-L---T-INEG-GSFGELALIYGTPRAATVVAKT---
-----D-VKLWAIDRLSYRRI-L----------------------
>PKA_[Wallemia_mellicola_CBS_633.66]_588257012
------------------------------V---VISQGDTGDA---FYFVEQGEADIIK
--NGEK----------------V-G---S-YKKG-DYFGELALLNSAPRAATVKASENQS
TAEN-Q-LKVVALDAPAFTRL-LGPVRDIMAR-------------
>PKA_[Wickerhamomyces_ciferrii]_754419701
-LSRLSLFE---RSKLA-DALETESYKSGDV---IIKEGEVGEN---FYLVENGEADVIK
----NQ--------G-----GLI-G---H-VKRG-DYFGEVALLNDTPRQASIVAKT---
-----D-VQVATLDKRGFQRL-L----------------------
>PKA_[Xenopus_laevis]_147898518
-FAHLDDTE---RSDIF-DAMFSVTYISGET---VIQQGDEGDN---FYVVDQGEMDVYV
----N---------N-----EWM-T---S-IGEG-GSFGELALIYGTPRAATVKAKT---
-----N-VKLWGIDRDSYRRI-L----------------------
>PKA_[Xylona_heveae_TC161]_1018267199
-LSSLTPYE---RSKIA-DALDAQKYPPGST---IIKEGDPGEA---FYIIENGEAEVFK
----RGT-------E-----KAV-H---K-YKKG-DYFGELALLNDAPRAASVISKT---
-----E-VKVATLGKDGFQRL-LGPVEG-----------------
>PKA_[Zygosaccharomyces_bailii_ISA1307]_578050454
------------------------LYEPGQV---IIREGDVGEN---FYLIEYGECDVSK
--KGKV----------------I-N---H-LKSH-DYFGEIALLKDLPRQATV-------

>PKA1_[Acanthisitta_chloris]_677990740
------------------------THIAGET---VIQQGDEGDN---FYVIDQGEVDVYV
----N---------G-----EWV-T---S-IGEG-GSFGELALIYGTPRAATVKAKT---
-----D-LKLWGIDRDSYRRI-LMGSTLRKRKMYEEFL-------
>PKA1_[Acinonyx_jubatus]_961715909
-FAHLDDNE---RSDIF-DAMFPVTHIAGET---VIQQGDEGDN---FYVIDQGEVDVYV
----N---------G-----EWV-T---S-ISEG-GSFGELALIYGTPRAATVKAKT---
-----D-LKLWGIDRDSYRRI-L----------------------

>PKA1_[Acropora_digitifera]_1005430447
-------------------------------------QGEHGDE---FYIIVDGVAVVLQ
--RRSIN-----EDF-----IEV-S---R-LGQS-DYFGEIALVLNRPRAATVRAK----
---G-T-LTCVKLDRQRFERV-L----------------------
>PKA1_[Alligator_mississippiensis]_951062238
--AHLDDNE---RSDIF-DAMFPVTHIAGET---VIQQGDEGDN---FYVIDQGEVDVYV
----N---------G-----EWV-T---S-IGEG-GSFGELALIYGTPRAATVKAKT---
-----D-LKLWGIDRDSYRRI-L----------------------
>PKA1_[Alligator_sinensis]_944356250
--AHLDDNE---RSDIF-DAMFPVTHIAGET---VIQQGDEGDN---FYVIDQGEVDVYV
----N---------G-----EWV-T---S-IGEG-GSFGELALIYGTPRAATVKAKT---
-----D-LKLWGIDRDSYRRI-L----------------------
>PKA1_[Amazona_aestiva]_944298418
--AHLDDNE---RSDIF-DAMFPVTHIAGET---VIQQGDEGDN---FYVIDQGEVDVYV
----N---------G-----EWV-T---S-IGEG-GSFGELALIYGTPRAATVKAKT---
-----D-LKLWGIDRDSYRRI-L----------------------
>PKA1_[Anas_platyrhynchos]_514748288
--AHLDDNE---RSDIF-DAMFPVTHIAGET---VIQQGDEGDN---FYVIDQGEVDVYV
----N---------G-----EWV-T---S-IGEG-GSFGELALIYGTPRAATVKAKT---
-----D-LKLWGIDRDSYRRI-L----------------------
>PKA1_[Anolis_carolinensis]_327283663
--AHLDDNE---RSDIF-DAMFPVTHIAGEI---VIQQGDEGDN---FYVIDQGEVDVYV
----N---------G-----EWV-T---S-IGEG-GSFGELALIYGTPRAATVKAKT---
-----D-LKLWGIDRDSYRRI-L----------------------
>PKA1_[Aotus_nancymaae]_817273831
--AHLDDNE---RSDIF-DAMFPVTHIAGET---VIQQGNEGDN---FYVIDQGEVDVYV
----N---------G-----EWV-T---N-ISEG-GSFGELALIYGTPRAATVKAKT---
-----D-LKLWGIDRDSYRRI-L----------------------
>PKA1_[Apis_cerana]_1035586152
----------------------------GEA---IIRQGDEGDN---FYVIDQGEVEIFV
----N---------G-----ELA-T---T-IGEG-GSFGELALIYGTPRAATVRAKT---
-----D-VKLWGIDRDSYRRI-L----------------------
>PKA1_[Apis_florea]_820837013
----------------------------GEA---IIRQGDEGDN---FYVIDQGEVEIFV
----N---------G-----ELA-T---T-IGEG-GSFGELALIYGTPRAATVRAKT---
-----D-VKLWGIDRDSYRRI-L----------------------
>PKA1_[Apis_mellifera]_571549118
---------------------------PGEA---IIRQGDEGDN---FYVIDQGEVEIFV
----N---------G-----ELA-T---T-IGEG-GSFGELALIYGTPRAATVRAKT---
-----D-VKLWGIDRDSYRRI-L----------------------
>PKA1_[Apteryx_australis_mantelli]_926514690
-FAHLDDNE---RSDIF-DAMFPVTHIAGET---VIQQGDEGDN---FYVIDQGEVDVYV
----N---------G-----EWV-T---S-IGEG-GSFGELALIYGTPRAATVKAKT---
-----D-LKLWGIDRDSYRRI-L----------------------
>PKA1_[Astyanax_mexicanus]_597743064
---------------------------AGEI---VIQQGDEGDN---FYVIDQGEMDVYV
----N---------N-----EWA-T---S-IGEG-GSFGELALIYGTPRAATVRAKT---
-----N-AKLWGIDRDSYRRI-LMGSTLRKRKMYE----------
>PKA1_[Athalia_rosae]_817088793
---------------------------PGEP---IIRQGDEGDN---FYVIDQGEVEIFV
----N---------S-----ELV-T---T-IGEG-GSFGELALIYGTPRAATVRAKT---
-----D-VKLWGIDRDSYRRI-L----------------------
>PKA1_[Balaenoptera_acutorostrata_scammoni]_594652405
--AHLDDNE---RSDIF-DAMFPVMHIAGET---VIQQGDEGDN---FYVIDQGEVDVYV

----N---------G-----EWV-T---S-ISEG-GSFGELALIYGTPRAATVKAKT---
-----D-LKLWGIDRDSYRRI-LMGSTLRKRRMYEE---------
>PKA1_[Balearica_regulorum_gibbericeps]_723541872
------------------------THIAGET---VIQQGDEGDN---FYVIDQGEVDVYV
----N---------G-----EWV-T---S-IGEG-GSFGELALIYGTPRAATVKAKT---
-----D-LKLWGIDRDSYRRI-LMGSTLRKRKMYEEFL-------
>PKA1_[Bison_bison_bison]_742197730
--SHLDDNE---RSDIF-DAMFPVTHIAGET---VIQQGDEGDN---FYVIDQGEVDVYV
----N---------G-----EWV-T---S-ISEG-GSFGELALIYGTPRAATVKAKT---
-----D-LKLWGIDRDSYRRI-LMGSTLRKRRMYEE---------
>PKA1_[Bombus_terrestris]_808127992
----------------------------GEA---IIRQGDEGDN---FYVIDQGEVEIFV
----N---------G-----ELA-T---T-IGEG-GSFGELALIYGTPRAATVRAKT---
-----D-VKLWGIDRDSYRRI-L---------------------
>PKA1_[Bos_mutus]_555997402
-FSHLDDNE---RSDIF-DAMFPVTHIAGET---VIQQGDEGDN---FYVIDQGEVDVYV
----N---------G-----EWV-T---S-ISEG-GSFGELALIYGTPRAATVKAKT---
-----D-LKLWGIDRDSYRRI-L---------------------
>PKA1_[Bos_taurus]_296472916
-FSHLDDNE---RSDIF-DAMFPVTHIAGET---VIQQGDEGDN---FYVIDQGEVDVYV
----N---------G-----EWV-T---S-ISEG-GSFGELALIYGTPRAATVKAKT---
-----D-LKLWGIDRDSYRRI-L---------------------
>PKA1_[Calidris_pugnax]_960983349
--AHLDDNE---RSDIF-DAMFPVTHIAGET---VIQQGDEGDN---FYVIDQGEVDVYV
----N---------G-----EWV-T---S-IGEG-GSFGELALIYGTPRAATVKAKT---
-----D-LKLWGIDRDSYRRI-L---------------------
>PKA1_[Callorhinchus_milii]_632943104
---------------------------AGET---VIQQGDEGDN---FYVIDQGEMDVYV
----N---------N-----EWV-T---S-IGEG-GSFGELALIYGTPRAATVRAKT---
-----N-VKLWGIDRDSYRRI-LMGSTLRKRKMYE----------
>PKA1_[Calypte_anna]_663276586
--AHLDDNE---RSDIF-DAMFPVTHIAGET---VIQQGDEGDN---FYVIDQGEVDVYV
----N---------G-----EWV-T---S-IGEG-GSFGELALIYGTPRAATVKAKT---
-----D-LKLWGIDRDSYRRI-L---------------------
>PKA1_[Camelus_dromedarius]_744610170
-FAHLDDSE---RSDIF-DAMFPVTHIAGET---VIQQGDEGDN---FYVIDQGEVDVYV
----N---------G-----EWV-T---S-ISEG-GSFGELALIYGTPRAATVKAKT---
-----D-LKVWGIDRDSYRRI-L---------------------
>PKA1_[Camelus_ferus]_946601670
-FAHLDDSE---RSDIF-DAMFPVTHIAGET---VIQQGDEGDN---FYVIDQGEVDVYV
----N---------G-----EWV-T---S-ISEG-GSFGELALIYGTPRAATVKAKT---
-----D-LKVWGIDRDSYRRI-L---------------------
>PKA1_[Camponotus_floridanus]_752878646
---------------------------PGEA---IIRQGDEGDN---FYVIDQGEVEIFV
----N---------G-----ELA-T---T-IGEG-GSFGELALIYGTPRAATVRAKT---
-----D-VKLWGIDRDSYRRI-L---------------------
>PKA1_[Capra_hircus]_926724592
--SHLDDNE---RSDIF-DAMFPVTHIAGET---VIQQGDEGDN---FYVIDQGEVDVYV
----N---------G-----EWV-T---S-ISEG-GSFGELALIYGTPRAATVKAKT---
-----D-LKLWGIDRDSYRRI-LMGSTLRKRRMYE----------
>PKA1_[Carlito_syrichta]_640778685
--AHLDDNE---RSDIF-DAMFPVTHIAGET---VIQQGNEGDN---FYVIDQGEVDVFV
----N---------G-----EWV-T---S-ISEG-GSFGELALIYGTPRAATVKAKT---
-----D-LKLWGIDRDSYRRI-L---------------------

>PKA1_[Cerapachys_biroi]_759046834
---------------------------PGEA---IIRQGDEGDN---FYVIDQGEVEIFV
----N---------G-----ELA-T---T-IGEG-GSFGELALIYGTPRAATVRAKT---
-----D-VKLWGIDRDSYRRI-L----------------------
>PKA1_[Ceratosolen_solmsi_marchali]_766930771
----------------------------GEA---IIRQGDEGDN---FYVIDQGEVEIFV
----S---------G-----ELV-T---T-IGEG-GSFGELALIYGTPRAATVRAKT---
-----D-VKLWGIDRDSYRRI-LMGSTIRKRKMYEEFL-------
>PKA1_[Ceratotherium_simum_simum]_955539393
--AHLDDNE---RSDIF-DAMFPVTHIAGET---VIQQGDEGDN---FYVIDQGEVDVYV
----N---------G-----EWV-T---S-ISEG-GSFGELALIYGTPRAATVKAKT---
-----D-LKLWGIDRDSYRRI-L----------------------
>PKA1_[Chaetura_pelagica]_701423906
--AHLDDNE---RSDIF-DAMFPVTHIAGET---VIQQGDEGDN---FYVIDQGEVDVYV
----N---------G-----EWV-T---S-IGEG-GSFGELALIYGTPRAATVKAKT---
-----D-LKLWGIDRDSYRRI-L----------------------
>PKA1_[Charadrius_vociferus]_699626517
--AHLDDNE---RSDIF-DAMFPVTHIAGET---VIQQGDEGDN---FYVIDQGEVDVYV
----N---------G-----EWV-T---S-IGEG-GSFGELALIYGTPRAATVKAKT---
-----D-LKLWGIDRDSYRRI-L----------------------
>PKA1_[Chelonia_mydas]_591381780
--AHLDDNE---RSDIF-DAMFPVMHIAGET---VIQQGDEGDN---FYVIDQGEVDVYV
----N---------G-----EWV-T---S-IGEG-GSFGELALIYGTPRAATVKAKT---
-----D-LKLWGIDRDSYRRI-L----------------------
>PKA1_[Chlamydotis_macqueenii]_705673756
--AHLDDNE---RSDIF-DAMFPVTHIAGET---VIQQGDEGDN---FYVIDQGEVDVYV
----N---------G-----EWV-T---S-IGEG-GSFGELALIYGTPRAATVKAKT---
-----D-LKLWGIDRDSYRRI-L----------------------
>PKA1_[Clupea_harengus]_831283318
---------------------------AGEI---VIQQGDEGDN---FYVIDQGEMDVYV
----N---------G-----EWA-T---N-IGEG-GSFGELALIYGTPRAATVRART---
-----N-VKLWGIDRDSYRRI-L----------------------
>PKA1_[Colius_striatus]_706102549
--AHLDDNE---RSDIF-DAMFPVTHIAGET---VIQQGDEGDN---FYVIDQGEVDVYV
----N---------G-----EWV-T---S-IGEG-GSFGELALIYGTPRAATVKAKT---
-----D-LKLWGIDRDSYRRI-L----------------------
>PKA1_[Condylura_cristata]_830089745
--AHLDDNE---RSDIF-DAMFPVTHIAGET---VIQQGDEGDN---FYVIDQGEVDVYV
----N---------G-----EWV-T---S-ISEG-GSFGELALIYGTPRAATVKAKT---
-----D-LKLWGIDRDSYRRI-L----------------------
>PKA1_[Copidosoma_floridanum]_936601194
----------------------------GEA---IIRQGDEGDN---FYVIDQGEVEIFV
----N---------G-----ELV-T---T-IGEG-GSFGELALIYGTPRAATVRAKT---
-----D-VKLWGIDRDSYRRI-LMNSTIRKRKMYEEFL-------
>PKA1_[Coturnix_japonica]_1003958536
--AHLDDNE---RSDIF-DAMFPVTHIAGET---VIQQGDEGDN---FYVIDQGEVDVYV
----N---------G-----EWV-T---S-IGEG-GSFGELALIYGTPRAATVKAKT---
-----D-LKLWGIDRDSYRRI-L----------------------
>PKA1_[Cricetulus_griseus]_625241285
---------------------------AGET---VIQQGNEGDN---FYVVDQGEVDVYV
----N---------G-----DWV-T---T-ISEG-GSFGELALIYGTPRAATVQAKT---
-----D-LKLWGIDRDSYRRI-L----------------------
>PKA1_[Cuculus_canorus]_697001501
--AHLDDNE---RSDIF-DAMFPVTHIAGET---VIQQGDEGDN---FYVIDQGEVDVYV

----N---------G-----EWV-T---S-IGEG-GSFGELALIYGTPRAATVKAKT---
-----D-LKLWGIDRDSYRRI-L----------------------
>PKA1_[Danio_rerio]_115495407
--AHLDDNE---RSDIF-DAMFPVTHIAGET---VIQQGDEGDN---FYVIDQGEVDVYV
----N---------G-----EWV-T---S-IGEG-GSFGELALIYGTPRAATVKAKT---
-----D-LKLWGIDRDSYRRI-L----------------------
>PKA1_[Daphnia_magna]_1022768684
--------------------MFPVSAHSGEV---IIQQGDEGDN---FYVIDQGEVEVFV
----D---------G-----NMV-T---V-IGEG-GSFGELALIYGTPRAATVKAKT---
-----D-VKLWGLDRDSYRRI-L----------------------
>PKA1_[Diaphorina_citri]_1041547186
----------------------------------IVRQGQPGDD---FYIIVEGTALVLQ
--NTVEE-----ESP-----VEV-G---K-LGPS-DYFGEIALLLDRPRAATVVAK----
---G-P-LKCVKLDRARFERV-L----------------------
>PKA1_[Dipodomys_ordii]_852770109
--AHLDDNE---RSDIF-DAMFPVTHIAGET---VIQQGNEGDN---FYVIDQGEADVYV
----N---------G-----EWV-T---N-ISEG-GSFGELALIYGTPRAATVKAKT---
-----D-LKLWGIDRDSYRRI-L----------------------
>PKA1_[Drosophila_eugracilis]_1037046479
--AHLDESE---RSDIF-DAMFPVNHIAGEN---IIQQGDEGDN---FYVIDVGEVDVFV
----N---------S-----ELV-T---T-ISEG-GSFGELALIYGTPRAATVRAKT---
-----D-VKLWGIDRDSYRRI-L----------------------
>PKA1_[Drosophila_kikkawai]_1037045534
--AHLDESE---RSDIF-DAMFPVNHIAGED---IIRQGDEGDN---FYVIDVGEVDVFV
----N---------A-----ELV-T---T-ISEG-GSFGELALIYGTPRAATVRAKT---
-----D-VKLWGIDRDSYRRI-L----------------------
>PKA1_[Drosophila_suzukii]_1036061260
--AHLDESE---RSDIF-DAMFPVNHIAGEN---IIQQGDEGDN---FYVIDVGEVDVFV
----N---------S-----ELV-T---T-ISEG-GSFGELALIYGTPRAATVRAKT---
-----D-VKLWGIDRDSYRRI-L----------------------
>PKA1_[Echinococcus_multilocularis]_961439633
--------------------MFPLHRSRGDI---IIKQGDDGDN---FYIIDQGKVDIYV
----N---------G-----EFV-S---C-IGEG-GSFGELALIYGTPRAATVKAHS---
-----DEVKLWGIDRDSYRRI-LMGSTIRRRKMYQDFL-------
>PKA1_[Echinops_telfairi]_507657555
--AHLDDNE---RSDIF-DAMFPVTHIAGET---VIQQGDEGDN---FYVVDQGEVDVYV
----N---------G-----EWV-T---S-ISEG-GSFGELALIYGTPRAATVKAKT---
-----D-LKLWGIDRDSYRRI-L----------------------
>PKA1_[Eptesicus_fuscus]_641728295
--AHLDDNE---RSDIF-DAMFPVTHIAGET---VIQQGDEGDN---FYVIDQGEVDVYV
----N---------G-----EWV-T---N-ISEG-GSFGELALIYGTPRAATVRAKT---
-----D-LKLWGIDRDSYRRI-L----------------------
>PKA1_[Equus_asinus]_958716155
--AHLDDNE---RSDIF-DAMFPVTHIAGET---VIQQGDEGDN---FYVIDQGEVDVYV
----N---------G-----EWV-T---S-ISEG-GSFGELALIYGTPRAATVKAKT---
-----D-LKLWGIDRDSYRRI-L----------------------
>PKA1_[Equus_caballus]_953870329
-FAHLDDNE---RSDIF-DAMFPVTHIAGET---VIQQGDEGDN---FYVMDQGEVDVYV
----N---------G-----EWV-T---S-ISEG-GSFGELALIYGTPRAATVKAKT---
-----D-LKLWGIDRDSYRRI-L----------------------
>PKA1_[Esox_lucius]_742179557
--AHLDDNE---RSDIF-DAMFPVTHIAGET---VIQQGDEGDN---FYVIDQGEVDVYV
----N---------G-----EWV-T---S-IGEG-GSFGELALIYGTPRAATVKAKT---
-----D-MKLWGIDRDSYRRI-L----------------------

```
>PKA1_[Eurypyga_helias]_704267282
------------------------THIAGET---VIQQGDEGDN---FYVIDQGEVDVYV
----N---------G-----EWV-T---S-IGEG-GSFGELALIYGTPRAATVKAKT---
-----D-LKLWGIDRDSYRRI-LMGSTLRKRKMYEEFL-------
>PKA1_[Falco_cherrug]_541967630
--AHLDDNE---RSDIF-DAMFPVTHIAGET---VIQQGDEGDN---FYVIDQGEVDVYV
----N---------G-----EWV-T---S-IGEG-GSFGELALIYGTPRAATVKAKT---
-----D-LKLWGIDRDSYRRI-L---------------------
>PKA1_[Felis_catus]_755801765
--AHLDDNE---RSDIF-DAMFPVTHIAGET---VIQQGDEGDN---FYVIDQGEVDVYV
----N---------G-----EWV-T---S-ISEG-GSFGELALIYGTPRAATVKAKT---
-----D-LKLWGIDRDSYRRI-L---------------------
>PKA1_[Fopius_arisanus]_755956134
---------------------------PGEP---IIRQGDEGDN---FYVIDQGEVEIFV
----N---------G-----ELV-T---T-IGEG-GSFGELALIYGTPRAATVRAKT---
-----D-VKLWGIDRDSYRRI-L---------------------
>PKA1_[Fukomys_damarensis]_676285698
---------------------------AGET---VIQQGNEGDN---FYVIDQGEVDVYV
----N---------G-----EWV-T---S-ISEG-GSFGELALIYGTPRAATVKAKT---
-----D-LKLWGIDRDSYRRI-L---------------------
>PKA1_[Gallus_gallus]_50755469
--AHLDDNE---RSDIF-DAMFPVTHIAGET---VIQQGDEGDN---FYVIDQGEVDVYV
----N---------G-----EWV-T---S-IGEG-GSFGELALIYGTPRAATVKAKT---
-----D-LKLWGIDRDSYRRI-L---------------------
>PKA1_[Gekko_japonicus]_975135243
--AHLDDNE---RSDIF-DAMFPVTHIAGET---VIQQGDEGDN---FYVIDQGEVDVYV
----N---------G-----DLV-T---N-ISEG-GSFGELALIYGTPRAATVKAKT---
-----D-LKLWGIDRDSYRRI-L---------------------
>PKA1_[Habropoda_laboriosa]_915658220
----------------------------GEA---IIRQGDEGDN---FYVIDQGEVEIFV
----N---------G-----ELA-T---T-IGEG-GSFGELALIYGTPRAATVRAKT---
-----D-VKLWGIDRDSYRRI-LMGSTIRKRKMYEEFL-------
>PKA1_[Haliaeetus_albicilla]_700352967
------------------------THIAGET---VIQQGDEGDN---FYVIDQGEVDVYV
----N---------G-----EWV-T---S-IGEG-GSFGELALIYGTPRAATVKAKT---
-----D-LKLWGIDRDSYRRI-L---------------------
>PKA1_[Halyomorpha_halys]_939649870
--------------------MFPVNFLPGET---IIQQGDEGDN---FYVIDQGEVEVYV
----N---------S-----ELV-T---T-IGEG-SSFGELALIYGTPRAATVKAKT---
-----A-VKLWGIDRDSYRRI-L---------------------
>PKA1_[Heterocephalus_glaber]_512956089
--AHLDDNE---RSDIF-DAMFPVTHIAGET---VIQQGNEGDN---FYVIDQGEVDVYV
----N---------G-----EWV-T---S-ISEG-GSFGELALIYGTPRAATVKAKT---
-----D-LKLWGIDRDSYRRI-L---------------------
>PKA1_[Homo_sapiens]_1034656282
------------------------THIAGET---VIQQGNEGDN---FYVVDQGEVDVYV
----N---------G-----EWV-T---N-ISEG-GSFGELALIYGTPRAATVKAKT---
-----D-LKLWGIDRDSYRRI-LMGSTLRKRKMYEEFL-------
>PKA1_[Ictidomys_tridecemlineatus]_532110346
--AHLDDNE---RSDIF-DAMFPVTHIAGET---VIQQGNEGDN---FYVIDQGEVDVYV
----N---------G-----EWV-T---N-ISEG-GSFGELALIYGTPRAATVKAKT---
-----D-LKLWGIDRDSYRRI-L---------------------
>PKA1_[Intoshia_linei]_1026916177
--------------------MFLSNFSTDDV---IMNQGDEGDN---FYVINTGTVDIIV
```

----N---------N-----VKV-T---T-ITDG-SSFGELALIYGTPRAATVIAKSD--
-----I-T-LFGIDRMSYRKI-L----------------------
>PKA1_[Ixodes_scapularis]_241117494
--SHLDDNE---RSDIF-DAMFPVVHRAGEV---IIQQGDEGDN---FYVLDQGEVDVYV
----N---------G-----QLV-T---T-IAES-GSFGELALIYGTPRAATVKAKT---
-----D-VKLWAIDRDTYRRI-LMGSTIRKRKLYE----------
>PKA1_[Jaculus_jaculus]_847034118
--AHLDDNE---RSDIF-DAMFPVTHIAGET---VIQQGNEGDN---FYVLDQGEVDVYV
----N---------G-----EWV-T---S-ISEG-GSFGELALIYGTPRAATVKAKT---
-----D-LKLWGIDRDSYRRI-L----------------------
>PKA1_[Lepeophtheirus_salmonis]_290462751
-FSHLDENE---RSDIF-DAMFPINALPGEV---VIKQGDEGDN---FYIIDAGEVEIYV
----H---------N-----EKV-L---T-IREG-GSFGELALIYGTPRAATVKAHS---
-----N-VKLWGIDRDSYRRI-LMGSTIRKRKMYEEFL-------
>PKA1_[Limulus_polyphemus]_926651500
-------------------------HSAGEK---VIQQGDEGDN---FYVIDQGEVEVFV
----N---------D-----HLV-T---T-IGEG-GSFGELALIYGTPRAATVIAKT---
-----D-VKLWAIDRDTYRRI-L----------------------
>PKA1_[Linepithema_humile]_815765982
---------------------------PGEA---IIRQGDEGDN---FYVIDQGEVEIFV
----N---------G-----ELA-T---T-IGEG-GSFGELALIYGTPRAATVRAKT---
-----D-VKLWGIDRDSYRRI-L----------------------
>PKA1_[Lipotes_vexillifer]_602713819
--AHLDDNE---RSDIF-DAMFPVMHIAGET---VIQQGDEGDN---FYVIDQGEVDVYV
----N---------G-----EWV-T---S-ISEG-GSFGELALIYGTPRAATVKAKT---
-----D-LKLWGIDRDSYRRI-LMGSTLRKRRMYEEFLGKV----
>PKA1_[Loxodonta_africana]_731500532
--AHLDDNE---RSDIF-DAMFPVTHIAGEI---VIQQGDEGDN---FYVIDQGEVDVYV
----N---------G-----EWV-T---S-ISEG-GSFGELALIYGTPRAATVKAKT---
-----D-LKLWGIDRDSYRRI-L----------------------
>PKA1_[Lucilia_cuprina]_906473136
----------------------------GEN---IIQQGDEGDN---FYVIDQGEVEVFV
----N---------S-----EMV-T---T-IGEG-GSFGELALIYGTPRAATVRAKT---
-----D-VKLWGIDRDSYRRI-L----------------------
>PKA1_[Macaca_mulatta]_967510169
---------------------------AGET---VIQQGNEGDN---FYVIDQGEVDVYV
----N---------G-----EWV-T---N-ISEG-GSFGELALIYGTPRAATVRAKT---
-----D-LKLWGIDRDSYRRI-L----------------------
>PKA1_[Manacus_vitellinus]_675425077
--AHLDDNE---RSDIF-DAMFPVTHIAGET---VIQQGDEGDN---FYVIDQGEVDVYV
----N---------G-----EWV-T---S-IGEG-GSFGELALIYGTPRAATVKAKT---
-----D-LKLWGIDRDSYRRI-L----------------------
>PKA1_[Maylandia_zebra]_499017241
--AHLDDNE---RSDIF-DAMFPVTHIAGET---VIQQGDEGDN---FYVIDQGEVDVYV
----N---------G-----EWV-T---S-IGEG-GSFGELALIYGTPRAATVKAKT---
-----D-LKLWGIDRDSYRRI-L----------------------
>PKA1_[Megachile_rotundata]_805821312
---------------------------PGEA---IIRQGDEGDN---FYVIDQGEVEIFV
----N---------G-----ELV-T---T-IGEG-GSFGELALIYGTPRAATVRAKT---
-----D-VKLWGIDRDSYRRI-L----------------------
>PKA1_[Melipona_quadrifasciata]_925673659
----------------------------GEA---IIRQGDEGDN---FYVIDQGEVEIFV
----N---------G-----ELA-T---T-IGEG-GSFGELALIYGTPRAATVRAKT---
-----D-VKLWGIDRDSYRRI-LMGSTIRKRKMYEEFL-------

>PKA1_[Merops_nubicus]_677431645
--AHLDDNE---RSDIF-DAMFPVTHIAGET---VIQQGDEGDN---FYVIDQGEVDVYV
----N---------G-----EWV-T---S-IGEG-GSFGELALIYGTPRAATVKAKT---
-----D-LKLWGIDRDSYRRI-L----------------------
>PKA1_[Microplitis_demolitor]_939681039
---------------------------PGEP---IIRQGDEGDN---FYVIDQGEVEIFV
----N---------G-----ELV-T---T-IGEG-GSFGELALIYGTPRAATVRAKT---
-----D-VKLWGIDRDSYRRI-L----------------------
>PKA1_[Microtus_ochrogaster]_532048746
---------------------------AGET---VIQQGNEGDN---FYVIDQGEVDVYV
----N---------G-----EWV-T---N-ISEG-GSFGELALIYGTPRAATVKAKT---
-----D-LKLWGIDRDSYRRI-L----------------------
>PKA1_[Monodelphis_domestica]_1023021855
--AHLDDNE---RSDIF-DAMFPVTHIAGET---VILQGDEGDN---FYVIDQGEVDVYV
----N---------M-----EWV-T---N-IGEG-GSFGELALIYGTPRAATVKAKT---
-----D-LKLWGIDRDSYRRI-L----------------------
>PKA1_[Mus_musculus]_254675178
--SHLDDNE---RSDIF-DAMFPVTHIGGET---VIQQGNEGDN---FYVIDQGEVDVYV
----N---------G-----EWV-T---N-ISEG-GSFGELALIYGTPRAATVKAKT---
-----D-LKLWGIDRDSYRRI-L----------------------
>PKA1_[Mustela_putorius_furo]_511914083
--AHLDDNE---RSDIF-DAMFPVTHIAGET---VIQQGDEGDN---FYVIDQGEVDVYV
----N---------G-----EWV-T---S-ISEG-GSFGELALIYGTPRAATVKAKT---
-----D-LKLWGIDRDSYRRI-L----------------------
>PKA1_[Myotis_brandtii]_946750609
--AHLDDNE---RSDIF-DAMFPVTHIAGET---VIQQGDEGDN---FYVVDQGEVDVYV
----N---------G-----EWV-T---N-ISEG-GSFGELALIYGTPRAATVKAKT---
-----D-LKLWGIDRDSYRRI-L----------------------
>PKA1_[Myotis_davidii]_432100476
--AHLDDNE---RSDIF-DAMFPVTHIAGET---VIQQGDEGDN---FYVVDQGEVDVYV
----N---------G-----EWV-T---N-ISEG-GSFGELALIYGTPRAATVKAKT---
-----D-LKLWGIDRDSYRRI-L----------------------
>PKA1_[Myotis_lucifugus]_940782762
---------------------------AGET---VIQQGDEGDN---FYVVDQGEVDVYV
----N---------G-----EWV-T---N-ISEG-GSFGELALIYGTPRAATVKAKT---
-----D-LKLWGIDRDSYRRI-L----------------------
>PKA1_[Nannospalax_galili]_674083910
---------------------------AGET---VIQQGNEGDN---FYVIDQGEVDVYV
----N---------G-----EWV-T---N-ISEG-GSFGELALIYGTPRAATVKAKT---
-----D-LKLWGIDRDSYRRI-L----------------------
>PKA1_[Nasonia_vitripennis]_1032738335
----------------------------GEA---IIRQGDEGDN---FYVIDQGEVEIFV
----N---------G-----EHV-T---T-IGEG-GSFGELALIYGTPRAATVRAKT---
-----D-VKLWGIDRDSYRRI-L----------------------
>PKA1_[Nestor_notabilis]_701290125
------------------------THIAGET---VIQQGDEGDN---FYVIDQGEVDVYV
----N---------G-----EWV-T---S-IGEG-GSFGELALIYGTPRAATVKAKT---
-----D-LKLWGIDRDSYRRI-L----------------------
>PKA1_[Ochotona_princeps]_504178228
---------------------------AGET---VIQQGNEGDN---FYVIDQGEVDVYV
----N---------G-----EWV-T---N-ISEG-GSFGELALIYGTPRAATVRAKT---
-----D-LKLWGIDRDSYRRI-L----------------------
>PKA1_[Octodon_degus]_507638327
--AHLDDNE---RSDIF-DAMFPVTHIAGET---VIQQGNEGDN---FYVIDQGEVDVYV

----N---------G-----EWV-T---N-ISEG-GSFGELALIYGTPRAATVKAKT---
-----D-LKLWGIDRDSYRRI-L---------------------
>PKA1_[Odobenus_rosmarus_divergens]_823433139
--AHLDDNE---RSDIF-DAMFPVTHIAGET---VIQQGDEGDN---FYVIDQGEVDVYV
----N---------G-----EWV-T---S-ISEG-GSFGELALIYGTPRAATVKAKT---
-----D-LKLWGIDRDSYRRI-L---------------------
>PKA1_[Oikopleura_dioica]_188593370
----------------------------GTV---IIKQGDSGNF---FYVIDQGEVEVLV
----N---------D-----KQV-A---L-ISEW-GTFGELALIHGRPRQATVVAKTD--
-----V-VKLWAIDRETYRKI-LMSLQ-----------------
>PKA1_[Orcinus_orca]_821392390
----------------------------GEK---IVVQGEPGDD---FFIITEGTASVLQ
--RRSPG-----EEY-----VEV-G---R-LGPS-DYFGEIALLLNRPRAATVVAR----
---G-P-LKCVKLDRPRFERV-L---------------------
>PKA1_[Ornithorhynchus_anatinus]_620942137
------------------------------------SSGDEGDN---FYVIDQGEVDVYV
----N---------G-----EWV-T---S-IGEG-GSFGELALIYGTPRAATVKAKT---
-----D-LKLWGIDRDSYRRI-LMGSTLRKRKMYEE---------
>PKA1_[Orycteropus_afer_afer]_634823522
--AHLDDNE---RSDIF-DAMFPVTHIAGET---VIQQGDEGDN---FYVIDQGEVDVYV
----N---------G-----EWV-T---G-ISEG-GSFGELALIYGTPRAATVKAKT---
-----D-LKLWGIDRDSYRRI-L---------------------
>PKA1_[Oryzias_latipes]_765127750
--AHLDDNE---RSDIF-DAMFPVTHIAGET---VIQQGDEGDN---FYVIDQGEVDVYV
----N---------G-----ELV-T---N-IGEG-GSFGELALIYGTPRAATVKAKT---
-----D-LKLWGIDRDSYRRI-L---------------------
>PKA1_[Otolemur_garnettii]_395845587
---------------------------AGET---VIQQGNEGDN---FYVIDQGEVDVYV
----N---------G-----EWV-T---S-ISEG-GSFGELALIYGTPRAATVKAKT---
-----D-LKLWGIDRDSYRRI-L---------------------
>PKA1_[Ovis_aries_musimon]_803256005
---------------------------AGET---VIQQGDEGDN---FYVIDQGEVDVYV
----N---------G-----EWV-T---S-ISEG-GSFGELALIYGTPRAATVKAKT---
-----D-LKLWGIDRDSYRRI-LMGSTLRKRRMYE----------
>PKA1_[Ovis_aries]_966012556
---------------------------AGET---VIQQGDEGDN---FYVIDQGEVDVYV
----N---------G-----EWV-T---S-ISEG-GSFGELALIYGTPRAATVKAKT---
-----D-LKLWGIDRDSYRRI-LMGSTLRKRRMYE----------
>PKA1_[Pan_paniscus]_397497932
--AHLDDNE---RSDIF-DAMFPVTHIAGET---VIQQGNEGDN---FYVIDQGEVDVYV
----N---------G-----EWV-T---N-ISEG-GSFGELALIYGTPRAATVKAKT---
-----D-LKLWGIDRDSYRRI-L---------------------
>PKA1_[Panthera_tigris_altaica]_987417036
-FAHLDDNE---RSDIF-DAMFPVTHIAGET---VIQQGDEGDN---FYVIDQGEVDVYV
----N---------G-----EWV-T---S-ISEG-GSFGELALIYGTPRAATVKAKT---
-----D-LKLWGIDRDSYRRI-L---------------------
>PKA1_[Papilio_polytes]_909581713
-FTHLDESE---RADMF-DAMFPVQCLQGET---VIRQGDEGDN---FYIIDSGEVEVLV
----N---------G-----ELV-T---T-IGEG-GSFGELALIYGTPRAATVRARS---
-----R-LKLWGLDRDSYRRI-L---------------------
>PKA1_[Papilio_xuthus]_910336798
-FTHLDESE---RADMF-DAMFPVQCLQGET---VIRQGDEGDN---FYIIDSGEVEVLV
----N---------G-----ELV-T---T-IGEG-GSFGELALIYGTPRAATVRARS---
-----R-LKLWGLDRDSYRRI-L---------------------

```
>PKA1_[Papio_anubis]_685620580
------------------------THIAGET---VIQQGNEGDN---FYVIDQGEVDVYV
----N---------G-----EWV-T---N-ISEG-GSFGELALIYGTPRAATVRAKT---
-----D-LKLWGIDRDSYRRI-LMGSTLRKRKMYEEFL-------
>PKA1_[Peromyscus_maniculatus_bairdii]_589957158
-FSHLDDNE---RSDIF-DAMFPVTHIAGET---VIQQGNEGDN---FYVIDQGEVDVYV
----N---------G-----EWV-T---N-ISEG-GSFGELALIYGTPRAATVKAKT---
-----D-LKLWGIDRDSYRRI-L----------------------
>PKA1_[Physeter_catodon]_593751936
-FAHLDDNE---RSDIF-DAMFPVTHIAGET---VIQQGDEGDN---FYVIDQGEVDVYV
----N---------G-----EWV-T---S-ISEG-GSFGELALIYGTPRAATVKAKT---
-----D-LKLWGIDRDSYRRI-L----------------------
>PKA1_[Picoides_pubescens]_678195321
--AHLDDNE---RSDIF-DAMFPVTHIAGET---VIQQGDEGDN---FYVIDQGEVDVYV
----N---------G-----EWV-T---S-IGEG-GSFGELALIYGTPRAATVKAKT---
-----D-LKLWGIDRDSYRRI-L----------------------
>PKA1_[Podiceps_cristatus]_683472431
------------------------THIAGET---VIQQGDEGDN---FYVIDQGEVDVYV
----N---------G-----EWV-T---S-IGEG-GSFGELALIYGTPRAATVKAKT---
-----D-LKLWGIDRDSYRRI-LMGSTLRKRKMYEEFL-------
>PKA1_[Poecilia_formosa]_617414431
--AHLDDNE---RSDIF-DAMFPVTHIAGET---VIQQGDEGDN---FYVIDQGEVDVYV
----N---------G-----ELV-T---N-IGEG-GSFGELALIYGTPRAATVKAKT---
-----D-LKLWGIDRDSYRRI-L----------------------
>PKA1_[Polistes_canadensis]_954558836
---------------------------PGEA---IIRQGDEGDN---FYVIDQGEVEIFV
----N---------G-----ELV-T---T-IGEG-GSFGELALIYGTPRAATVRAKT---
-----D-VKLWGIDRDSYRRI-L----------------------
>PKA1_[Polistes_dominula]_972186183
----------------------------GEA---IIRQGDEGDN---FYVIDQGEVEIFV
----N---------G-----ELV-T---T-IGEG-GSFGELALIYGTPRAATVRAKT---
-----D-VKLWGIDRDSYRRI-LMGSTIRKRKMYEEFL-------
>PKA1_[Pongo_abelii]_686770134
------------------------THIAGET---VIQQGNEGDN---FYVIDQGEVDVYV
----N---------G-----EWV-T---N-ISEG-GSFGELALIYGTPRAATVKAKT---
-----D-LKLWGIDRDSYRRI-L----------------------
>PKA1_[Propithecus_coquereli]_826278761
-FAHLDDNE---RSDIF-DAMFPVTHIAGET---VIQQGNEGDN---FYVIDQGEVDVYV
----N---------G-----DWV-T---S-ISEG-GSFGELALIYGTPRAATVKAKT---
-----D-LKLWGIDRDSYRRI-L----------------------
>PKA1_[Protobothrops_mucrosquamatus]_1002594974
--AHLDDNE---RSDIF-DAMFPVTHIAGEI---VIQQGDEGDN---FYVIDHGEVYVYV
----N---------G-----ELV-T---S-IGEG-GSFGELALIYGTPRAATVKAKT---
-----D-LKLWGIDRDSYRRI-L----------------------
>PKA1_[Pteropus_vampyrus]_759198126
--------------------MFPVSFIAGET---VIQQGDEGDN---FYVIDQGEMDVYV
----N---------S-----EWA-T---S-VGEG-GSFGELALIYGTPRAATVKAKT---
-----N-VKLWGIDRDSYRRI-L----------------------
>PKA1_[Python_bivittatus]_602638945
--AHLDDNE---RSDIF-DAMFPVTHIAGET---VIQQGDEGDN---FYVIDHGEVYVYV
----N---------G-----ELV-T---S-IGEG-GSFGELALIYGTPRAATVKAKT---
-----D-LKLWGIDRDSYRRI-L----------------------
>PKA1_[Rhinopithecus_roxellana]_724841325
--AHLDDNE---RSDIF-DAMFPVTHIAGET---VIQQGNEGDN---FYVIDQGEVDVYV
```

```
----N---------G-----EWV-T---N-ISEG-GSFGELALIYGTPRAATVRAKT---
-----D-LKLWGIDRDSYRRI-L----------------------
>PKA1_[Salmo_salar]_929094637
-------------------------HIAGET---VIQQGDEGDN---FYVIDQGEVDVYV
----N---------G-----EWV-T---S-IGEG-GSFGELALIYGTPRAATVKAKT---
-----D-MKLWGIDRDSYRRI-LMGSTLRKRKMYEEFL-------
>PKA1_[Schistosoma_mansoni]_350855186
--------------------MFPVHRNSGDV---IIQQGDEGDN---FYIIDQGEVDIFL
----N---------N-----EYS-S---T-IGEG-GSFGELALIYGTPRAATVKAKT---
-----E-VKLWGIDRDSYRRI-L----------------------
>PKA1_[Scleropages_formosus]_938062607
-FSHLDDNE---RSDIF-DAMFPVTYIAGET---VILQGDEGDN---FYVIDQGEMDVYV
----N---------N-----EWV-T---S-IGEG-GSFGELALIYGTPRAATVRAKT---
-----N-VKLWGIDRDSYRRI-L----------------------
>PKA1_[Sinocyclocheilus_anshuiensis]_1024949926
--AHLDDNE---RSDIF-DAMFPVTHIAGET---VIQQGDEGDN---FYVIDQGEVDVYV
----N---------G-----EWV-T---S-IGEG-GSFGELALIYGTPRAATVKAKT---
-----D-LKLWGIDRDSYRRI-L----------------------
>PKA1_[Sinocyclocheilus_rhinocerous]_1025300960
--AHLDDNE---RSDIF-DAMFPVTHIAGET---VIQQGDEGDN---FYVIDQGEVDVYV
----N---------G-----EWV-T---S-IGEG-GSFGELALIYGTPRAATVKAKT---
-----D-LKLWGIDRDSYRRI-L----------------------
>PKA1_[Solenopsis_invicta]_751208003
---------------------------PGEA---IIRQGDEGDN---FYVIDQGEVEIFV
----N---------G-----ELA-T---T-IGEG-GSFGELALIYGTPRAATVRAKT---
-----D-VKLWGIDRDSYRRI-L----------------------
>PKA1_[Sphaeroforma_arctica_JP610]_929755800
--------------------------KAGDK---IVTQGEDGDT---FFIIVEGEAIVTQ
--EMEGK-----AV-------EV-A---R-LHSS-DYFGEIALLTNKPRAATVTAS----
---G-A-LKCVKLDKERFERV-LGSCE------------------
>PKA1_[Stegastes_partitus]_657555081
--AHLDDNE---RSDIF-DAMFPVTHIAGET---VIQQGDEGDN---FYVIDQGEVDVYV
----N---------G-----EWV-T---S-IGEG-GSFGELALIYGTPRAATVKAKT---
-----D-LKLWGIDRDSYRRI-L----------------------
>PKA1_[Stomoxys_calcitrans]_907700118
----------------------------GEN---IIQQGDEGDN---FYVIDQGEVEVFV
----N---------S-----EMV-T---T-IGDG-GSFGELALIYGTPRAATVRAKT---
-----D-VKLWGIDRDSYRRI-L----------------------
>PKA1_[Strongylocentrotus_purpuratus]_780113006
----------------------------GQK---IVVQGEQGDD---FFMIIEGQAAVLQ
--RHGND-----SEL-----IEV-G---K-LGPS-DYFGEIALLLDRPRAATVVAR----
---G-N-LKCVKLDRQRFERV-L----------------------
>PKA1_[Taeniopygia_guttata]_224070450
--AHLDDNE---RSDIF-DAMFPVTHIAGET---VIQQGDEGDN---FYVIDQGEVDVYV
----N---------G-----EWV-T---S-IGEG-GSFGELALIYGTPRAATVKAKT---
-----D-LKLWGIDRDSYRRI-L----------------------
>PKA1_[Theileria_equi]_510900715
------------------------------V---IIQEGDAGTS---LFMILEGKAEAYC
--QGKLV-----------------K---S-YSKD-DYFGEIALIKQTPRASTV-------
---------------------------------------------
>PKA1_[Tinamus_guttatus]_719789282
--AHLDDNE---RSDIF-DAMFPVTHIAGET---VIQQGDEGDN---FYVIDQGEVDVYV
----N---------G-----EWV-T---S-IGEG-GSFGELALIYGTPRAATVKAKT---
-----D-LKLWGIDRDSYRRI-L----------------------
```

>PKA1_[Trichechus_manatus_latirostris]_471408822
-FAHLDDNE---RSDIF-DAMFPVTHIAGEI---VIQQGDEGDN---FYVIDQGEVDVYV
----N---------G-----EWV-T---S-ISEG-GSFGELALIYGTPRAATVKAKT---
-----D-LKLWGIDRDSYRRI-L---------------------
>PKA1_[Trichogramma_pretiosum]_936716301
----------------------------GEA---IIRQGDEGDN---FYVIDQGEVEIFV
----N---------G-----ELV-T---T-IGEG-GSFGELALIYGTPRAATVRAKT---
-----D-VKLWGIDRDSYRRI-LMGSTIRKRKMYEEFL-------
>PKA1_[Tupaia_chinensis]_562823219
-------------------------HIAGET---VIQQGDEGDN---FYVIDQGEVDVYV
----N---------G-----EWV-T---S-ISEG-GSFGELALIYGTPRAATVKAKT---
-----D-LKLWGIDRDSYRRI-LMGSTLRKRKMYEEFL-------
>PKA1_[Tursiops_truncatus]_470639443
----------------------------GEK---IVVQGEPGDD---FFIITEGTASVLQ
--RRSPG-----EEY-----VEV-G---R-LGPS-DYFGEIALLLNRPRAATVVAR----
---G-P-LKCVKLDRPRFERV-L---------------------
>PKA1_[Ursus_maritimus]_671011360
--AHLDDNE---RSDIF-DAMFPVTHIAGET---VIQQGDEGDN---FYVIDQGEVDVYV
----N---------G-----EWV-T---S-ISEG-GSFGELALIYGTPRAATVKAKT---
-----D-LKLWGIDRDSYRRI-L---------------------
>PKA1_[Vicugna_pacos]_560976338
--AHLDDSE---RSDIF-DAMFPVTHIAGET---VIQQGDEGDN---FYVIDQGEVDVYV
----N---------G-----EWV-T---S-ISEG-GSFGELALIYGTPRAATVKAKT---
-----D-LKVWGIDRDSYRRI-LMGSTLRKRRMYEE---------
>PKA1_[Vollenhovia_emeryi]_795049279
---------------------------PGEA---IIRQGDEGDN---FYVIDQGEVEIFV
----N---------G-----ELA-T---T-IGEG-GSFGELALIYGTPRAATVRAKT---
-----D-VKLWGIDRDSYRRI-L---------------------
>PKA1_[Xiphophorus_maculatus]_551515663
--AHLDDNE---RSDIF-DAMFPVTHIAGEI---VIQQGDEGDN---FYVIDQGEVDVYV
----N---------C-----ELV-T---N-IGEG-GSFGELALIYGTPRAATVKAKT---
-----D-LKLWGIDRDSYRRI-L---------------------
>PKA2_[Cimex_lectularius]_939264753
------------KLQLA-DALVQKSYKMGER---ILKQGDAADG---MYFVMQGEVEISI
--INDKG-----ED------VVL-K---V-LKSG-DYFGELALVNHQPRAASAFARK---
-----K-ADLAYLDAEAFERL-L---------------------
>PKA2_[Copidosoma_floridanum]_936574405
---------KEQMAGVL-DAMFEKTVKPGEF---IIRQGDDGDN---FYVIEKGQYEVYV
----KD--------------ALI-H---T-YDNS-GFFGELALLYNMPRAATVKAKT---
-----P-GTLWAMDRQTFRRI-LLKSAYKKRKMYE----------
>PKA2_[Danio_rerio]_47085741
--------Q---LAEVL-DSMFEVLVKPGEC---IINQGDDGDN---FYVIERGVYEIVI
--QQDGLQ------------HSV-G---R-YDHK-GSFGELALMYNTPRAATIRAL-Q--
-----E-GALWALDRATFHRL-IVKNNAKKRRMYE----------
>PKA2_[Fopius_arisanus]_755982687
---------KEQMTDVL-DAMFEKHVQPGDF---IIRQGDDGDN---FYVIERGKFEVYV
----KDPT----GAP-----TLI-H---T-YDNG-GAFGELALLYNMPRAATIKALT---
-----Q-GTLWAMDRQTFRRI-I---------------------
>PKA2_[Hydra_vulgaris]_449662454
--------------------MFEKKVNKGDV---IIKQGDDGDN---FYVIEKGIFDVHV
----KKD-----SAE-----KIV-A---T-LEDK-GFFGDLALLYNCPRNATIIAKS---
-----E-GVLWGLDQKTFKRI-VVKATAKKRLLFEE---------
>PKA2_[Maylandia_zebra]_498997757
--------------------MFESIVKPQEH---VIDQGHDGDN---FYVIERGVYDIVV

--SGK----------------CV-G---Q-YNNK-GSFGELALMYNTPRAATIVAT-Q--
-----D-GALWGLDRATFRRL-IVKNNAKKRRMYE----------
>PKA2_[Neolamprologus_brichardi]_583976161
--------------------MFEVLVKPQDH---IIDQGDDGDN---FYIIEKGVYDIFV
--QKDGVS------------ICV-G---K-YDNK-GSFGELALMYNTPRAATIVAT-Q--
-----D-GALWGLDRATFHRL-IVKNNAKKRRMYE----------
>PKA2_[Takifugu_rubripes]_410899138
--------------------MFESRVQAQEH---VIDQGDDGDN---FYVIERGVFDIVV
--SGN----------------CV-G---Q-YNNK-GSFGELALMYNTPRAATIIAT-Q--
-----E-GALWGLDRATFRRL-IVKNNAKKRRMYE----------
>PKA2_[Xenopus_tropicalis]_847114511
------QEQ---MSQVL-DAMFEKLVKCGEH---VIDQGDDGDN---FYVIDRGTYDIFV
--KSDGVV------------RCV-G---A-YDNR-GSFGELALMYNTPRAATIVAT-S--
-----V-GSIWGLDRATFRRI-IVKNNAKKKRMYEN---------
>PKG_[Alligator_mississippiensis]_950917339
----------------------------GDY---VIREGEEGST---FFILAKGKVRVTQ
--STDGH-----AQP-----QLI-K---T-LQKG-DYFGEKALISDDVRSANIIADEN--
-----D-VECLVIDRETFNQT-VGTFE------------------
>PKG_[Alligator_sinensis]_944338233
----------------------------GDY---VIREGEEGST---FFILAKGKVRVTQ
--SSDGH-----AQP-----QLI-K---T-LQKG-DYFGEKALISDDVRSANIIADEN--
-----D-VECLVIDRETFNQT-VGTFE------------------
>PKG_[Amazona_aestiva]_944214442
-----------------------EYYDKGDY---VIREGEEGNT---FFIIAKGKVIVTQ
--STSDH-----SQP-----QLI-K---N-LHKG-DYFGEKALISDDVRSANVIADEY--
-----N-VECLVIDRETFNQT-VGTYE------------------
>PKG_[Amyelois_transitella]_913311658
------------LAKIS-ECLKREFFSAGTP---VVRQGDRGDK---FYIIRGGTVLVTK
--R-EGD-----DGE-----RRI-G---T-LHRG-DYFGEQALLHEDRRLATVTAQPP--
-----G-VECLTLDRTQFTDL-LGDLD------------------
>PKG_[Anolis_carolinensis]_637302463
----------------------------GDY---IIREGEEGNT---FFVIAKGKVKVTQ
--TTDGQ-----PQT-----QLI-K---T-LQKG-DYFGERALISDDVRSANIIADEN--
-----D-VECLVVDRETFNQT-VGTFE------------------
>PKG_[Aquila_chrysaetos_canadensis]_768399953
-----------------------EYYDKGDY---VIREGEEGNT---FFIIAKGKVIVTQ
--SSADH-----LQP-----QLI-K---N-LHKG-DYFGEKALIRDDVRSANVIADEY--
-----N-VECLVIDRETFNQI-VGTYE------------------
>PKG_[Austrofundulus_limnaeus]_928051910
----------------------------GEY---VIRQGEEGST---FYIIAQGKVNVTQ
--TTEAH-----KMP-----QII-N---T-LQTG-DYFGEKALISDDVRSANIIADDN--
-----G-VECLVIDRETFDQT-V---------------------
>PKG_[Bos_mutus]_555982500
----------------------------GDY---IIREGEEGST---FFILAKGKVKVTQ
--STEGH-----DQP-----QLI-K---T-LQKG-EYFGEKALISEDVRSANIIAEEN--
-----D-VACLVIDRETFNQT-VGTFEELQKYLE-----------
>PKG_[Bos_taurus]_296486400
----------------------------GDY---IIREGEEGST---FFILAKGKVKVTQ
--STEGH-----DQP-----QLI-K---T-LQKG-EYFGEKALISEDVRSANIIAEEN--
-----D-VACLVIDRETFNQT-VGTFEELQKYLE-----------
>PKG_[Camelus_bactrianus]_743701208
----------------------------GDY---IIREGEEGST---FFILAKGKVKVTQ
--STEGH-----DQP-----QLI-K---T-LQKG-EYFGEKALISEDVRSANIIAEEN--
-----D-VACLVIDRETFNQT-VGTFEELQKYLE-----------

>PKG_[Camelus_ferus]_560928512
----------------------------GDY---IIREGEEGST---FFILAKGKVKVTQ
--STEGH-----DQP-----QLI-K---T-LQKG-EYFGEKALISEDVRSANIIAEEN--
-----D-VACLVIDRETFNQT-VGTFEELQKYLE-----------
>PKG_[Capra_hircus]_548470773
SVSLLKNLPEDKLSKII-DCLEVEYYDKGDY---IIREGEEGST---FFILAKGKVKVTQ
--STEGH-----DQP-----QLI-K---T-LQKG-EYFGEKALISEDVRSANIIAEEN--
-----D-VACLVIDRETFNQT-VGTFEELQKYLE-----------
>PKG_[Caprimulgus_carolinensis]_704316565
----------------------------GDY---IIREGEEGNT---FFIIAKGKVIVTQ
--STADH-----SQP-----QVI-K---N-LHKG-DYFGEKALISDDVRSANVIADEY--
-----N-VECLVIDRETFNQT-VGTYE------------------
>PKG_[Carlito_syrichta]_640823046
----------------------------GDY---IIREGEEGST---FFILAKGKVKVTQ
--STEGH-----DQP-----QLI-K---T-LQKG-EYFGEKALISDDVRSANIIAEEN--
-----D-IACLVIDRETFNQT-VGTFEELQKYLE-----------
>PKG_[Cebus_capucinus_imitator]_1044360559
----------------------------GDY---IIREGEEGST---FFILAKGKVKVTQ
--STEGH-----DQP-----QLI-K---T-LQKG-EYFGEKALISDDVRSANIIAEEN--
-----D-VACLVIDRETFNQT-VGTFEELQKYLE-----------
>PKG_[Cercocebus_atys]_795495021
----------------------------GDY---IIREGEEGST---FFILAKGKVKVTQ
--STEGH-----DQP-----QLI-K---T-LQKG-EYFGEKALISDDVRSANIIAEEN--
-----D-VACLVIDRETFNQT-VGTFEELQKYLE-----------
>PKG_[Chinchilla_lanigera]_533147780
----------------------------GDY---IIREGEEGST---FFILAKGKVKVTQ
--STEGH-----DQP-----QLI-K---T-LQTG-EYFGEKALISDDVRSANIIAEEN--
-----D-VACLVIDRETFNQT-VGTFEELQKYLE-----------
>PKG_[Chlamydotis_macqueenii]_677160399
-----------------------EYYDKGDY---VIREGEEGNT---FFIIAKGKVIVTQ
--STADH-----LQP-----QLI-K---N-LHKG-DYFGEKALISDDVRSANVIADEY--
-----N-VECLVIDRETFNQT-VGTYE------------------
>PKG_[Chrysochloris_asiatica]_586489323
----------------------------GDY---IIREGEEGST---FYILAKGKVQVTQ
--STEGH-----DQP-----QLI-K---I-LQKG-DYFGEKALISDDVRSANIIAEEN--
-----D-VACLVIDRETFNQT-VGTFE------------------
>PKG_[Chrysochromulina_sp._CCMP291]_922865428
----------------------------------IITEGEEGTH---FYVIARGEVVITK
--AGQG---------------EL-A---R-RREG-DYFGELSLKTGAPTMASVTAA----
---AAG-TTVVRMDRGAFQRL-L----------------------
>PKG_[Clupea_harengus]_831282541
----------------------------GEY---VIREGEEGNT---FFIIAQGGVCVTQ
--TMEGC-----PDP-----QQI-K---T-LGVG-DYFGEKALISEDVRSANIIATDD--
-----N-TQCLVLDRDNFNQM-VGTYEELQAYLREY---------
>PKG_[Columba_livia]_915525022
-----------------------EYYNKGDY---VIREGEEGNT---FFIIAKGKVIVTQ
--STADH-----VQP-----QVI-K---N-LHKG-DYFGEKALISDDVRSANVIADEY--
-----N-VECLVIDRETFNQT-VGTYE------------------
>PKG_[Condylura_cristata]_507942718
SVSLLKNLPEEKLTKII-DCLEVEYYDKGDY---IIREGEEGST---FFILAKGKVKVTQ
--STEGH-----DQP-----QLI-K---T-LQKG-EYFGEKALISEDVRSANIIAEEN--
-----D-VACLVIDRETFNQT-VGTFEELQKYLE-----------
>PKG_[Corvus_brachyrhynchos]_669292792
-----------------------EYYVKGDY---VIREGEEGNT---FFIIAKGRVIVTQ

```
--STADH-----SQP-----QVI-K---N-LHKG-DYFGEKALISDDVRSANVIADEY--
-----N-VECLVIDRETFNQT-VGTYE------------------
>PKG_[Corvus_cornix_cornix]_727064776
-----------------------EYYVKGDY---VIREGEEGNT---FFIIAKGRVIVTQ
--STADH-----SQP-----QVI-K---N-LHKG-DYFGEKALISDDVRSANVIADEY--
-----N-VECLVIDRETFNQT-VGTYE------------------
>PKG_[Coturnix_japonica]_1003773094
-----------------------EYYNKGDY---VIREGEEGNT---FFIIAKGKVKVTQ
--STTDH-----SQP-----LLI-K---N-LHKG-DYFGEKALISDDVRSANVIADEY--
-----D-VECLVIDRETFNQT-VGTYE------------------
>PKG_[Cricetulus_griseus]_354504910
----------------------------GDY---IIREGEEGST---FFILAKGKVKVTQ
--STEGH-----EQP-----QLI-K---T-LQKG-EYFGEKALISDDVRSANIIAEEN--
-----D-VACLVIDRETFNQT-VGTFEELQKYLE-----------
>PKG_[Cynoglossus_semilaevis]_657784751
----------------------------GEY---IIREGEEGNT---FFIIAKGEVIVTQ
--STEGF-----IEP-----QEI-K---T-LTVG-DYFGEKALISEDVRSANIVSNQN--
-----D-THCLVVDRDNFNQI-VGTYEELQAYLKEYVKEL-----
>PKG_[Danio_rerio]_528497262
----------------------------GEY---IIREGEEGNT---FFIIAKGEVSVTQ
--TTEGF-----TEP-----QEI-K---T-LRVG-DYFGEKALISEDVRSANIIAKEN--
-----D-TQCLVVDRDNFNEM-VGTYEELQAYLREYVEEL-----
>PKG_[Elephantulus_edwardii]_585657006
----------------------------GDY---IIREGEEGST---FYILAKGKVKVTQ
--STEGH-----DQP-----QLI-K---V-LQKG-EYFGEKALISDDVRSANIIAEEN--
-----A-VACLVIDRETFNQT-VGTFEELQKYLE-----------
>PKG_[Equus_caballus]_194209030
----------------------------GDY---IIREGEEGST---FFILAKGKVKVTQ
--STEGH-----DQP-----QLI-K---T-LQKG-EYFGEKALISEDVRSANIIAEEN--
-----D-VACLVIDRETFNQT-VGTFEELQKYLE-----------
>PKG_[Esox_lucius]_742098783
----------------------------GEY---IIREGEEGNT---FFIIAKGEVCVTQ
--TTEGW-----TEP-----QEI-K---T-LGVG-DYFGEKALISEDVRSANIISTEN--
-----D-TQCLVVDRDNFNQI-VGTYEELQAYLKEYVKEL-----
>PKG_[Felis_catus]_755722898
----------------------------GDY---IIREGEEGST---FFILAKGKVKVTQ
--STEGH-----DQP-----QLI-K---T-LQKG-EYFGEKALISEDVRSANIIAEEN--
-----D-VACLVIDRETFNQT-VGTFEELQKYLE-----------
>PKG_[Fukomys_damarensis]_676286342
----------------------------GDY---IIREGEEGST---FFILAKGKVKVTQ
--STEGH-----DQP-----QLI-K---T-LQTG-EYFGEKALISDDVRSANIIAEEN--
-----D-VACLVIDRETFSQT-VGTFEELQKYLE-----------
>PKG_[Gekko_japonicus]_975113523
-----------------------EYYDKGDY---VIREGEEGNT---FFVIAKGKVKVTQ
--TAEGC-----TQA-----HLI-K---I-LQKG-DYFGEKALISDDVRSANIIADEN--
-----D-VECLVIDRETFNQT-VGTFE------------------
>PKG_[Haplochromis_burtoni]_554822682
----------------------------GEY---VIREGEEGST---FYIIAQGKVKVTQ
--TTEAH-----KLP-----QII-N---T-LQKG-DYFGEKALISDDVRSANIIADEN--
-----G-VECLVIDRETFDQT-V---------------------
>PKG_[Heterocephalus_glaber]_512971868
----------------------------GDY---IIREGEEGST---FFILAKGKVKVTQ
--STEGH-----DQP-----QLI-K---T-LQTG-EYFGEKALISDDVRSANIIAEEN--
-----D-VACLVIDRETFNQT-VGTFEELQKYLE-----------
```

>PKG_[Homo_sapiens]_1034640577
----------------------------GDY---IIREGEEGST---FFILAKGKVKVTQ
--STEGH-----DQP-----QLI-K---T-LQKG-EYFGEKALISDDVRSANIIAEEN--
-----D-VACLVIDRETFNQT-VGTFEELQKYLE-----------
>PKG_[Jaculus_jaculus]_507538849
----------------------------GDY---IIREGEEGST---FFILAKGKVKVTQ
--STEGH-----DQP-----QLI-K---T-LQRG-EYFGEKALISDDVRSANIIAEEN--
-----D-VACLVIDRETFNQT-VGTFEELQKYLE-----------
>PKG_[Kryptolebias_marmoratus]_1041107755
----------------------------GEY---IIREGEEGNT---FFIIAKGEVIVTQ
--STEGF-----AEP-----QEI-K---T-LGVG-DYFGEKALISEDVRSANIICSEN--
-----D-TQCLVVDRDNFIQM-VGTYEELQVYLKEYVKEL-----
>PKG_[Lepisosteus_oculatus]_972953834
-----------------------EYYDKGDY---VIREGEEGST---FYIIAKGKVKVTQ
--STQDH-----VEP-----QTI-K---T-LQKG-DYFGEKALISDDVRSANIIADEN--
-----D-VECLVIDRETFNQT-V---------------------
>PKG_[Lipotes_vexillifer]_602724288
----------------------------GDY---IIREGEEGST---FFILAKGKVKVTQ
--STEGH-----DQP-----QLI-K---T-LQKG-DYFGEKALISEDVRSANIIAEEN--
-----D-VACLVIDRETFNQT-VGTFE-----------------
>PKG_[Loa_loa]_393910958
--------------------MFPVEKKKGET---IIEQGEEGDN---FYVIDSGEVDVFV
----N---------G-----EYA-L---S-IKEG-GSFGELALIYGTPRAATVVAKSD--
-----V-VKCWAIDRITYRQI-L---------------------
>PKG_[Macaca_mulatta]_966943263
----------------------------GDY---IIREGEEGST---FFILAKGKVKVTQ
--STEGH-----DQP-----QLI-K---T-LQKG-EYFGEKALISDDVRSANIIAEEN--
-----D-VACLVIDRETFNQT-VGTFEELQKYLE-----------
>PKG_[Macaca_nemestrina]_795492120
----------------------------GDY---IIREGEEGST---FFILAKGKVKVTQ
--STEGH-----DQP-----QLI-K---T-LQKG-EYFGEKALISDDVRSANIIAEEN--
-----D-VACLVIDRETFNQT-VGTFEELQKYLE-----------
>PKG_[Manacus_vitellinus]_675407196
-----------------------EYYNKGDY---VIREGEEGNT---FFIIAKGKVIVTQ
--STADH-----SQP-----QLI-K---N-LHKG-DYFGEKALISDDVRSANVIADEY--
-----N-VECLVIDRETFNQT-VGTYEELQTYLEGYVADL-----
>PKG_[Maylandia_zebra]_499004466
----------------------------GEY---VIREGEEGST---FYIIAQGKVKVTQ
--TTEAH-----KLP-----QII-N---T-LQKG-DYFGEKALISDDVRSANIIADEN--
-----G-VECLVIDRETFDQT-V---------------------
>PKG_[Monodelphis_domestica]_126331034
----------------------------GDY---IIREGEEGST---FFILAKGVVKVTQ
--STEGY-----DQP-----QLI-K---T-LKKG-DYFGEKALISDDVRSANIIAEEN--
-----D-VACLVIDRETFSQT-VGTFE-----------------
>PKG_[Mus_musculus]_568935385
----------------------------GDY---IIREGEEGST---FFILAKGKVKVTQ
--STEGH-----DQP-----QLI-K---T-LQKG-EYFGEKALISDDVRSANIIAEEN--
-----D-VACLVIDRETFNQT-V---------------------
>PKG_[Mustela_putorius_furo]_511916269
----------------------------GDY---IIREGEEGST---FFILAKGKVKVTQ
--STEGH-----DQP-----QLI-K---T-LQKG-EYFGEKALISEDVRSANIIAEEN--
-----D-VACLVIDRETFNQT-VGTFEELQKYLE-----------
>PKG_[Nannospalax_galili]_674054426
----------------------------GDY---IIREGEEGST---FFILAKGKVKVTQ

--STEGH-----DQP-----QLI-K---T-LQKG-EYFGEKALISDDVRSANIIAEEN--
-----D-VACLVIDRETFNQT-VGTFEELQKYLE-----------
>PKG_[Nomascus_leucogenys]_332233332
----------------------------GDY---IIREGEEGST---FFILAKGKVKVTQ
--STEGH-----DQP-----QLI-K---T-LQKG-EYFGEKALISDDVRSANIIAEEN--
-----D-VACLVIDRETFNQT-VGTFEELQKYLE-----------
>PKG_[Nothobranchius_furzeri]_1007752306
----------------------------GEY---VIRQGEEGST---FYIIAQGKVKVTQ
--TTEAH-----KPP-----QII-N---T-LQKG-DYFGEKALISDDLRSANIIADEN--
-----G-VECLVIDRETFD--------------------------
>PKG_[Notothenia_coriiceps]_736227441
----------------------------GEY---IIREGEEGNT---FFIIAKGEVIVTQ
--STEGL-----AEP-----QEI-K---T-LGVG-DYFGEKALISEDVRSANIICNEN--
-----D-THCLVVDRDNFNQM-VGTYEELQSYLKEY---------
>PKG_[Ochotona_princeps]_504158458
----------------------------GDY---IIREGEEGST---FFILAKGKVKVTQ
--STEGH-----DQP-----QLI-K---T-LQKG-EYFGEKALISDDVRSANIIAEEN--
-----D-VACLVIDRETFNQT-VGTFEDLQKYLE-----------
>PKG_[Octodon_degus]_507695791
----------------------------GDY---IIREGEEGST---FFILAKGKVKVTQ
--STEGH-----DQP-----QLI-K---T-LQTG-EYFGEKALISDDVRSANIIAEEN--
-----D-VACLVIDRETFNQT-VGTFEELQKYLE-----------
>PKG_[Orcinus_orca]_466060154
----------------------------GDY---IIREGEEGST---FFILAKGKVKVTQ
--STEGH-----DQP-----QLI-K---T-LQKG-EYFGEKALISEDVRSANIIAEEN--
-----D-VACLVIDRETFNQT-VGTFEELQKYLE-----------
>PKG_[Oryzias_latipes]_432901822
----------------------------GEY---IIREGEEGNT---FFIIAKGQVTVTQ
--SSEGF-----TQP-----QEI-K---T-LGVG-DYFGERALISEDVRSANIICNEN--
-----D-TQCLVVDRDNFNQM-VGTYEELQTYLKEY---------
>PKG_[Ostreococcus_tauri]_308800796
---------------IS-NAARRETYEARDE---VFRQGDPGHC---FYIIERGEVSVRV
-------------NG-----AEV-V---K-LSRG-DFFGERALVNNEPRAATIYA-MT--
-----E-VSCLVLNRQTFVSM-LGSIE------------------
>PKG_[Otolemur_garnettii]_395834200
----------------------------GDY---IIREGEEGST---FFILAKGKVKVTQ
--STEGH-----DQP-----QLI-K---T-LQKG-EYFGEKALISDDVRSANIIAEEN--
-----D-VACLVIDRETFNQT-VGTFEELQKYLE-----------
>PKG_[Ovis_aries]_426231932
----------------------------GDY---IIREGEEGST---FFILAKGKVKVTQ
--STEGH-----DQP-----QLI-K---T-LQKG-EYFGEKALISEDVRSANIIAEEN--
-----D-VACLVIDRETFNQT-VGTFEELQKYLE-----------
>PKG_[Panthera_tigris_altaica]_591295261
----------------------------GDY---IIREGEEGST---FFILAKGKVKVTQ
--STEGH-----DQP-----QLI-K---T-LQKG-EYFGEKALISEDVRSANIIAEEN--
-----D-VACLVIDRETFNQT-VGTFEELQKYLE-----------
>PKG_[Papio_anubis]_685541174
----------------------------GDY---IIREGEEGST---FFILAKGKVKVTQ
--STEGH-----DQP-----QLI-K---T-LQKG-EYFGEKALISDDVRSANIIAEEN--
-----D-VACLVIDRETFNQT-VGTFEELQKYLE-----------
>PKG_[Paramecium_tetraurelia_strain_d4-2]_124088638
----------------------------GEM---VFKQGDKASS---YFLIERGQCQIII
----NNE--------------VKK---T-LKQG-EAFGELALLYNAPRSASVKAVG---
-----D-CAFWAIDRNTVRKA-IEAISQRDYEQNK----------

>PKG_[Paramecium_tetraurelia]_74832323
----------------------------GQF---VFKQGDKASS---YFLIERGQCQIII
----NGE---------------LKK---T-LKSG-DAFGELAMLYNAPRSASVRAVG---
-----D-CAFWAIDRNTFRKV-VE---------------------
>PKG_[Parus_major]_998687631
-----------------------EYYDKGDY---VIREGEEGNT---FFIIAKGKVIVTQ
--STTDH-----SQP-----QVI-K---N-LHKG-DYFGEKALISDDVRSANVIADEY--
-----N-VECLVIDRETFNQT-VGTYE------------------
>PKG_[Peromyscus_maniculatus_bairdii]_589925120
----------------------------GDY---IIREGEEGST---FFILAKGKVKVTQ
--STEGH-----DQP-----QLI-K---T-LQKG-EYFGEKALISDDVRSANIIAEEN--
-----D-VACLVIDRETFNQT-VGTFEELQKYLE-----------
>PKG_[Physeter_catodon]_593728959
----------------------------GDY---IIREGEEGST---FFILAKGKVKVTQ
--STEGH-----DQP-----QLI-K---T-LQKG-EYFGEKALISEDVRSANIIAEEN--
-----D-VACLVIDRETFNQT-VGTFEELQKYLE-----------
>PKG_[Poecilia_formosa]_617384217
-LANLPDDKLSKIVDCL-EVEY---YDKGEY---VIRQGEEGST---FYIIAQGKVKVTQ
--TTEAY-----RLP-----QTI-N---T-LQKG-DYFGEKALISDDVRSANIIADEN--
-----G-VECLVIDRETFDHT-VGNFNELQKHLEGYVATLDRDDK
>PKG_[Pongo_abelii]_297673859
----------------------------GDY---IIREGEEGST---FFILAKGKVKVTQ
--STEGH-----DQP-----QLI-K---T-LQKG-EYFGEKALISDDVRSANIIAEEN--
-----D-VACLVIDRETFNQT-VGTFEELQKYLE-----------
>PKG_[Protobothrops_mucrosquamatus]_1002572554
-----------------------EYYDKGDY---VIREGEEGST---FFVISRGKVKVTQ
--TPEGQ-----TQP-----QLI-K---T-LQKG-DSFGEKALISDDVRSANIIADEN--
-----D-VECLVIDRETFNQT-VGTFE------------------
>PKG_[Pseudopodoces_humilis]_929494223
-----------------------EYYDKGDY---VIREGEEGNT---FFIIAKGKVIVTQ
--STTDH-----SQP-----QVI-K---N-LHKG-DYFGEKALISDDVRSANVIADEY--
-----N-VECLVIDRETFNQT-VGTYE------------------
>PKG_[Pundamilia_nyererei]_548356986
----------------------------GEY---VIREGEEGST---FYIIAQGKVKVTQ
--TTEAH-----KLP-----QII-N---T-LQKG-DYFGEKALISDDVRSANIIADEN--
-----G-VECLVIDRETFDQT-V----------------------
>PKG_[Rattus_norvegicus]_149046836
----------------------------GDY---IIREGEEGST---FFILAKGKVKVTQ
--STEGH-----DQP-----QLI-K---T-LQKG-EYFGEKALISDDVRSANIIAEEN--
-----D-VACLVIDRETFNQT-V----------------------
>PKG_[Rhinopithecus_roxellana]_724952715
----------------------------GDY---IIREGEEGST---FFILAKGKVKVTQ
--STEGH-----DQP-----QLI-K---T-LQKG-EYFGEKALISDDVRSANIIAEEN--
-----D-VACLVIDRETFNQT-VGTFEELQKYLE-----------
>PKG_[Saimiri_boliviensis_boliviensis]_403263339
----------------------------GDY---IIREGEEGST---FFILAKGKVKVTQ
--STEGH-----DQP-----QLI-K---T-LQKG-EYFGEKALISDDVRSANIIAEEN--
-----D-VACLVIDRETFNQT-VGTFEELQKYLE-----------
>PKG_[Salmo_salar]_929265821
----------------------------GEY---IIREGEEGNT---FFIIAKGEVCVTQ
--TTEGC-----TEP-----QEI-K---T-LGVG-DYFGEKALISEDVRSANIISTEN--
-----D-TQCLVVDRDNFNQM-VGTYEELQAYLKEYVEEL-----
>PKG_[Sarcophilus_harrisii]_821490471
----------------------------GDY---IIREGEEGST---FFILAKGMVKVTQ

--STEGN-----DKP-----QVI-K---T-LQKG-DYFGEKALISDDVRSANIIAEEN--
-----D-VACLVIDRETFSQT-VGTFE------------------
>PKG_[Scleropages_formosus]_938085719
----------------------------GDY---IIREGEEGNT---FFIIAKGEVSVTQ
--TVEGF-----PEP-----QEI-K---T-LGVG-DYFGEKALVSEDVRSANIICKEN--
-----D-TECLVLDRDNFNQM-VGTYEELQAYLREY---------
>PKG_[Sinocyclocheilus_anshuiensis]_1025161141
----------------------------GEY---IIREGEEGNT---FFIIAKGEVLVTQ
--TTEGF-----SEP-----QEI-K---T-LGVG-DYFGEKALISEDVRSANIIAKEN--
-----D-TQCLVVDRDNFNQM-VGTYEELQAYLREY---------
>PKG_[Sinocyclocheilus_grahami]_1020420739
----------------------------GEY---IIREGEEGNT---FFIIAKGEVLVTQ
--TTEGF-----SEP-----QEI-K---T-LVVG-DYFGEKALISEDVRSANIIAKEN--
-----D-TQCLVVDRDNFNQM-VGTYEELQAYLREY---------
>PKG_[Sinocyclocheilus_rhinocerous]_1025246920
----------------------------GEY---IIREGEEGNT---FFIIAKGEVLVTQ
--TTEGF-----SEP-----QEI-K---T-LGVG-DYFGEKALISEDVRSANIIAKEN--
-----D-TQCLVVDRDNFNQM-VGTYEELQAYLREY---------
>PKG_[Stegastes_partitus]_657591087
----------------------------GEY---IIREGEEGNT---FFIIAKGEVIVTQ
--RTEGF-----AEP-----QEI-N---T-LGVG-DYFGEKALISEDVRSANIICNEN--
-----D-THCLVVDRENFNQM-VGTYEELQAYLKEYVEEL-----
>PKG_[Sturnus_vulgaris]_959065620
-----------------------EYYDKGDY---VIREGEEGNT---FFIIAKGKVIVTQ
--STTDH-----SQP-----QVI-K---N-LHKG-DYFGEKALISDDVRSANVIADEY--
-----N-VECLVIDRETFNQT-VGTYE------------------
>PKG_[Sus_scrofa]_350588031
----------------------------GDY---IIREGEEGST---FFILAKGKVKVTQ
--STEGH-----DQP-----QLI-K---T-LQKG-EYFGEKALISEDVRSANIIAEED--
-----D-VACLVIDRETFNQT-VGTFEELQKYLE-----------
>PKG_[Takifugu_rubripes]_410922467
----------------------------GEF---IIREGEEGST---FYIIAQGKVKVTQ
--TTEAH-----TFP-----QVI-N---T-LQKG-DYFGEKALVSDDVRSASILAEEN--
-----G-VECLVIDRETFDQT-V----------------------
>PKG_[Tetrahymena_thermophila_SB210]_829108302
----------------------------------VFKQEDKASS---YFIIESGQVDIII
----NDE---------------VKR---T-LNPG-ESFGELALLYNAPRSASIRCKG---
-----N-CHFWAIDRNSFRKT-IEDMVQK----------------
>PKG_[Theileria_orientalis_strain_Shintoku]_697892214
----------------------------GST---VTKQGTNGSY---FFIINEGVFDVFV
--DDKLV-----------------N---T-MERG-ASFGEIALINDMPRTATVKVRD---

>PKG_[Vicugna_pacos]_560951478
----------------------------GDY---IIREGEEGST---FFILAKGKVKVTQ
--STEGH-----DQP-----QLI-K---T-LQKG-EYFGEKALISEDVRSANIIAEEN--
-----D-VACLVIDRETFNQT-VGTFEELQKYLE-----------
>PKG_[Xenopus_tropicalis]_512852469
----------------------------GDY---IIREGEEGNT---FFIIAKGKVCVTQ
--AVEGS-----QEP-----QEI-K---T-LGVG-DYFGEKALISEDVRSANIIAEED--
-----D-TQCLVIDRDTFNQM-VGTYQELQTYLREY---------
>Rapgef1_[Ascaris_suum]_541042023
ALAHLSTMV---KRELASVVFFEQHQHAGTV---LFRQGDEGNS---WYIIILKGSVHVSI
H----GKGI-------------V-C---T-LQEG-DDFGKLALVNDAPRAATIALSED--
-----N-SQFLRVDKTDFNRI-LRDVEANTVRLKEHGHDVLV---

>Rapgef1_[Loa_loa]_393908590
ALTHLSTMV---KRELAAVVFFEQHQHAGHV---LFRQGDVGIC---WYIILKGSVDVII
H----GKGV-------------V-C---T-LREG-DDFGKLALVNDAPRAATVALRQD--
-----K-SQFLRVDKDDFNRI-LRDVEANTVRLKEHGQDVLV---
>Rapgef1_[Toxocara_canis]_734558230
ALAHLSTMV---KRELASVVFFEQHQHAGTV---LFRQGDEGNC---WYIILKGSVNVSI
H----GKGV-------------V-C---T-LQEG-DDFGKLALVNDAPRAATIALCED--
-----N-SQFLRVDKTDFNRI-LRDVEANTVRLKEHGHDVLV---
>Rapgef2_[Acanthisitta_chloris]_677303776
AFANMTMSV---RRELCAVMVFAVVERAGTI---VLNDGEELDS---WSVILNGSVEVTY
---PDGR---------------T-E---I-LCMG-NSFGVSPTMEKEYMKGVMRTKVD--
-----D-CQFVCIAQQDYCRI-LNQVEKNMQKVEE-EGEIV----
>Rapgef2_[Acromyrmex_echinatior]_746838636
AFTNMTLAV---RRALCAVMVFAVVDRAGMV---VLNDGEELDS---WSVLINGAVEIEH
---SNGE---------------I-E---Q-LGLG-DSFGILPTMERLLHRGVMRTKCD--
-----D-CQFVCVTQADYFRI-QHQGEENTRRHE-ENGRVI----
>Rapgef2_[Alligator_mississippiensis]_950917983
AFANMTMSV---RRELCAVMVFAVVERAGTI---VLNDGEELDS---WSVILNGSVEVTY
---PDGR---------------T-E---I-LCMG-NSFGVSPTLDKEYMKGVMRTKVD--
-----D-CQFVCIAQQDYCRI-LNQVEKNMQKVEE-EGEIV----
>Rapgef2_[Alligator_sinensis]_557280478
AFANMTMSV---RRELCAVMVFAVVERAGTI---VLNDGEELDS---WSVILNGSVEVTY
---PDGR---------------M-E---I-LCMG-NSFGVSPTLDKEYMKGVMRTKVD--
-----D-CQFVCIAQQDYCRI-LNQVEKNMQKVEE-EGEIV----
>Rapgef2_[Anser_cygnoides_domesticus]_902881430
AFANMTMSV---RRELCAVMVFAVVERAGTI---VLNDGEELDS---WSVILNGSVEVTY
---PDGR---------------T-E---I-LCMG-NSFGVSPTMDKEYMKGVMRTKVD--
-----D-CQFVCIAQQDYCRI-LNQVEKNMQKVEE-EGEIV----
>Rapgef2_[Apis_cerana]_1035602537
AFTNMTLAV---RRALCAVMVFAVVERAGMI---VLNDGEELDS---WSVLINGAVEIEH
---SNGE---------------I-E---Q-LHLG-DSFGILPTMERLLHRGVMRTKCD--
-----D-CQFVCVTQADYFRI-QHQGEENTRRHE-ENGRVI----
>Rapgef2_[Apis_florea]_820851409
AFTNMTLAV---RRALCAVMVFAVVERAGMI---VLNDGEELDS---WSVLINGAVEIEH
---SNGE---------------I-E---Q-LHLG-DSFGILPTMERLLHRGVMRTKCD--
-----D-CQFVCVTQADYFRI-QHQGEENTRRHE-ENGRVI----
>Rapgef2_[Apis_mellifera]_1032018827
AFTNMTLAV---RRALCAVMVFAVVERAGMI---VLNDGEELDS---WSVLINGAVEIEH
---SNGE---------------I-E---Q-LHLG-DSFGILPTMERLLHRGVMRTKCD--
-----D-CQFVCVTQADYFRI-QHQGEENTRRHE-ENGRVI----
>Rapgef2_[Aptenodytes_forsteri]_675315011
AFANMTMSV---RRELCAVMVFAVVERAGTI---VLNDGEELDS---WSVILNGSVEVTY
---PDGR---------------T-E---I-LCMG-NSFGVSPTMEKEYMKGVMRTKVD--
-----D-CQFVCIAQQDYCRI-LNQVEKNMQKVEE-EGEIV----
>Rapgef2_[Apteryx_australis_mantelli]_926516120
AFANMTMSV---RRELCAVMVFAVVERAGTI---VLNDGEELDS---WSVILNGSVEVTY
---PDGR---------------T-E---I-LCMG-NSFGVSPTMEKEYMKGVMRTKVD--
-----D-CQFVCIAQQDYCRI-LNQVEKNMQKVEE-EGEIV----
>Rapgef2_[Aquila_chrysaetos_canadensis]_768351324
AFANMTMSV---RRELCAVMVFAVVERAGTI---VLNDGEELDS---WSVILNGSVEVTY
---PDGR---------------T-E---I-LCMG-NSFGVSPTMEKEYMKGVMRTKVD--
-----D-CQFVCIAQQDYCRI-LNQVEKNMQKVEE-EGEIV----
>Rapgef2_[Bactrocera_dorsalis]_751774256
AFTNITLAV---RRALCAVMVFAVVDKAGTV---VMSDGEELDS---WSVLINGAVEIEH

---ANGT---------------R-E---E-LQMG-DSFGILPTMDKLYHRGVMRTKCD--
-----D-CQFVCITQTDYYRI-QHQGEENTRRHEDEDGRIV----
>Rapgef2_[Bactrocera_oleae]_929380230
AFTNITLAV---RRALCAVMVFAVVDKAGTV---VMSDGEELDS---WSVLINGAVEIEH
---ANGT---------------R-E---E-LQMG-DSFGILPTMDKLYHRGVMRTKCD--
-----D-CQFVCITQTDYYRI-QHQGEENTRRHEDEDGRIV----
>Rapgef2_[Balaenoptera_acutorostrata_scammoni]_594672686
AFANMTMSV---RRELCAVMVFAVVERAGTI---VLNNGEELDS---WSVILNGSVEVTY
---PDGK---------------A-E---I-LCMG-NSFGVSPTMDKEYMKGVMRTKVD--
-----D-CQFVCIAQQDYCRI-LNQVEKNMQKVEE-EGEIV----
>Rapgef2_[Balearica_regulorum_gibbericeps]_676249633
AFANMTMSV---RRELCAVMVFAVVERAGTI---VLNDGEELDS---WSVILNGSVEVTY
---PDGR---------------T-E---I-LCMG-NSFGVSPTMEKEYMKGVMRTKVD--
-----D-CQFVCIAQQDYCRI-LNQVEKNMQKVEE-EGEIV----
>Rapgef2_[Bos_mutus]_440905105
AFANMTMSV---RRELCAVMVFAVVERAGTI---VLNDGEELDS---WSVILNGSVEVTY
---PDGK---------------A-E---I-LCMG-NSFGVSPTMDKEYMKGVMRTKVD--
-----D-CQFVCIAQQDYCRI-LNQVEKNMQKVEE-EGEIV----
>Rapgef2_[Bos_taurus]_982949932
AFANMTMSV---RRELCAVMVFAVVERAGTI---VLNDGEELDS---WSVILNGSVEVTY
---PDGK---------------A-E---I-LCMG-NSFGVSPTMDKEYMKGVMRTKVD--
-----D-CQFVCIAQQDYCRI-LNQVEKNMQKVEE-EGEIV----
>Rapgef2_[Bubalus_bubalis]_594101752
AFANMTMSV---RRELCAVMVFAVVERAGTI---VLNDGEELDS---WSVILNGSVEVTY
---PDGK---------------A-E---I-LCMG-NSFGVSPTMDKEYMKGVMRTKVD--
-----D-CQFVCIAQQDYCRI-LNQVEKNMQKVEE-EGEIV----
>Rapgef2_[Buceros_rhinoceros_silvestris]_676706740
AFANMTMSV---RRELCAVMVFAVVERAGTI---VLNDGEELDS---WSVILNGSVEVTY
---PDGR---------------T-E---I-LCMG-NSFGVSPTMEKEYMKGVMRTKVD--
-----D-CQFVCIAQQDYCRI-LNQVEKNMQKVEE-EGEIV----
>Rapgef2_[Calypte_anna]_676772321
AFANMTMSV---RRELCAVMVFAVVERAGTI---VLNDGEELDS---WSVILNGSVEVTY
---PDGR---------------T-E---I-LCMG-NSFGVSPTMEKEYMKGVMRTKVD--
-----D-CQFVCIAQQDYCRI-LNQVEKNMQKVEE-EGEIV----
>Rapgef2_[Capra_hircus]_926712293
AFANMTMSV---RRELCAVMVFAVVERAGTI---VLNDGEELDS---WSVILNGSVEVTY
---PDGK---------------A-E---I-LCMG-NSFGVSPTMDKEYMKGVMRTKVD--
-----D-CQFVCIAQQDYCRI-LNQVEKNMQKVEE-EGEIV----
>Rapgef2_[Caprimulgus_carolinensis]_683464460
AFANMTMSV---RRELCAVMVFAVVERAGTI---VLNDGEELDS---WSVILNGSVEVTY
---PDGR---------------T-E---I-LCMG-NSFGVSPTMEKEYMKGVMRTKVD--
-----D-CQFVCIAQQDYCRI-LNQVEKNMQKVEE-EGEIV----
>Rapgef2_[Cariama_cristata]_677270084
AFANMTMSV---RRELCAVMVFAVVERAGTI---VLNDGEELDS---WSVILNGSVEVTY
---PDGR---------------T-E---I-LCMG-NSFGVSPTMEKEYMKGVMRTKVD--
-----D-CQFVCIAQQDYCRI-LNQVEKNMQKVEE-EGEIV----
>Rapgef2_[Cathartes_aura]_677207033
AFANMTMSV---RRELCAVMVFAVVERAGTI---VLNDGEELDS---WSVILNGSVEVTY
---PDGR---------------T-E---I-LCMG-NSFGVSPTMEKEYMKGVMRTKVD--
-----D-CQFVCIAQQDYCRI-LNQVEKNMQKVEE-EGEIV----
>Rapgef2_[Cerapachys_biroi]_759046383
AFTNMTLAV---RRALCAVMVFAVVDRAGMV---VLNDGEELDS---WSVLINGAVEIEH
---SNGE---------------I-E---Q-LGLG-DSFGILPTMERLLHRGVMRTKCD--
-----D-CQFVCVTQADYFRI-QHQGEENTRRHE-ENGRVI----

>Rapgef2_[Ceratotherium_simum_simum]_955490023
AFANMTMSV---RRELCAVMVFAVVERAGTI---VLNDGEELDS---WSVILNGSVEVTY
---PDGK---------------A-E---I-LCMG-NSFGVSPTMDKEYMKGVMRTKVD--
-----D-CQFVCIAQQDYCRI-LNQVEKNMQKVEE-EGEIV----
>Rapgef2_[Chelonia_mydas]_591353969
AFANMTMSV---RRELCAVMVFAVVERAGTI---VLNDGEELDS---WSVILNGSVEVTY
---PDGR---------------T-E---I-LCMG-NSFGVSPTMDKEYMKGVMRTKVD--
-----D-CQFVCIAQQDYCRI-LNQVEKNMQKVEE-EGEIV----
>Rapgef2_[Chlamydotis_macqueenii]_677153119
AFANMTMSV---RRELCAVMVFAVVERAGTI---VLNDGEELDS---WSVILNGSVEVTY
---PDGR---------------T-E---I-LCMG-NSFGVSPTMEKEYMKGVMRTKVD--
-----D-CQFVCIAQQDYCRI-LNQVEKNMQKVEE-EGEIV----
>Rapgef2_[Chlorocebus_sabaeus]_635045457
AFANMTMSV---RRELCAVMVFAVVERAGTI---VLNDGEELDS---WSVILNGSVEVTY
---PDGK---------------A-E---I-LCMG-NSFGVSPTMDKEYMKGVMRTKVD--
-----D-CQFVCIAQQDYCRI-LNQVEKNMQKVEE-EGEIV----
>Rapgef2_[Chrysochloris_asiatica]_586474854
AFANMTMSV---RRELCAVMVFAVVERAGTI---VLNDGEELDS---WSVILNGSVEVTY
---PDGK---------------A-E---I-LCMG-NSFGVSPTMDKEYMKGIMRTKVD--
-----D-CQFVCIAQQDYCRI-LNQVEKNMQKVEE-EGEIV----
>Rapgef2_[Clupea_harengus]_831319817
AFANMTMSV---RRELCAVMVFAVVERAGTV---VLNDGEELDS---WSVILNGSVEVTY
---PEGR---------------A-E---I-LCMG-NSFGVSPTMDKEYMKGVMKTKVD--
-----D-CQFVCIAQQDYCCI-LNQVEKNMQKVEE-EGEIV----
>Rapgef2_[Colius_striatus]_677095238
AFANMTMSV---RRELCAVMVFAVVERAGTI---VLNDGEELDS---WSVILNGSVEVTY
---PDGR---------------T-E---I-LCMG-NSFGVSPTMEKEYMKGVMRTKVD--
-----D-CQFVCIAQQDYCRI-LNQVEKNMQKVEE-EGEIV----
>Rapgef2_[Colobus_angolensis_palliatus]_795294381
AFANMTMSV---RRELCAVMVFAVVERAGTI---VLNDGEELDS---WSVILNGSVEVTY
---PDGK---------------A-E---I-LCMG-NSFGVSPTMDKEYMKGVMRTKVD--
-----D-CQFVCIAQQDYCRI-LNQVEKNMQKVEE-EGEIV----
>Rapgef2_[Columba_livia]_543733807
AFANMTMSV---RRELCAVMVFAVVERAGTI---VLNDGEELDS---WSVILNGSVEVTY
---PDGR---------------T-E---I-LCMG-NSFGVSPTMEKEYMKGVMRTKVD--
-----D-CQFVCIAQQDYCRI-LNQVEKNMQKVEE-EGEIV----
>Rapgef2_[Corvus_cornix_cornix]_727064564
AFANMTMSV---RRELCAVMVFAVVERAGTI---VLNDGEELDS---WSVILNGSVEVTY
---PDGR---------------T-E---I-LCMG-NSFGVSPTMEKEYMKGVMRTKVD--
-----D-CQFVCIAQQDYCRI-LNQVEKNMQKVEE-EGEIV----
>Rapgef2_[Crassostrea_gigas]_762094124
AFANMTQPI---RRELCAVMVFAVIEQRGTV---VMKDGEELDS---WSVILNGQVEIVH
---PEGS---------------A-E---F-LQMG-DSFGISPTLDKMYHKGTMKTLLD--
-----D-CQFVCIAQEDYHRI-LDKGKENTEKHVE-EGQVV----
>Rapgef2_[Cricetulus_griseus]_1032849679
AFANMTMSV---RRELCAVMVFAVVERAGTI---VLNDGEELDS---WSVILNGSVEVTY
---PDGK---------------A-E---I-LCMG-NSFGVSPTMDKEYMKGVMRTKVD--
-----D-CQFVCIAQQDYCCI-LNQVEKNMQKVEE-EGEIV----
>Rapgef2_[Culex_quinquefasciatus]_170039216
AFTNMTFAV---RRDLCSVMVFAVVEKAGTI---VMNDGEELDS---WSVLINGHVEIEH
---ANGE---------------I-E---Y-LHIG-DSFGIMPTMDKLYHRGIMRTKCD--
-----D-CQFVCITQTDYYRI-QHQGEDNIRKIEK-DGQVI----
>Rapgef2_[Cynoglossus_semilaevis]_1035251284
AFANMTMSV---RRELCAVMVFAVVERAGTI---VLNDGEELDS---WSVILNGSVEVTY

```
---PDNR---------------T-E---I-LCMG-NSFGVSPTMEKEYMKGVMKTKVD--
-----D-CQFVCIAQQDYCCI-LNQVEKNMQKVEE-EGEIV----
>Rapgef2_[Cyphomyrmex_costatus]_1009369324
AFTNMTLAV---RRALCAVMVFAVVDRAGMV---VLNDGEELDS---WSVLINGAVEIEH
---SNSE---------------I-E---Q-LGLG-DSFGILPTMERLLHRGVMRTKCD--
-----D-CQFVCVTQADYFRI-QHQGEENTRRHE-ENGRVI----
>Rapgef2_[Danio_rerio]_688532444
AFASLSVSV---RRELCAVMVFAVVERAGTI---VLNHGEELDS---WSVILNGAVEVIY
---LDGR---------------S-E---S-VCMG-GSFGVCPSMEKQLMTGVMRTKVD--
-----D-CQFVCIAQQDYCCI-LNQVEQNTQRVEE-EGEIV----
>Rapgef2_[Diaphorina_citri]_1041553037
AFTNMTLAV---RKALCGVMVFAVVEKAGTI---VMNDGEELDS---WSVVINGCVEVEL
---SDGR---------------S-Q---M-LQVG-DSFGILPTMEKLYHEGVMRTRCD--
-----D-CQFVCITQTDYYRI------------------------
>Rapgef2_[Diuraphis_noxia]_985398052
AFTNMTLAV---RRALCSVMVFAVVEKAGTV---VMNDGEELDS---WSVLINGHVEVEH
---ADGI---------------P-D---Q-LHMG-DSFGIVPTMERLYHRGVMRTKCD--
-----D-CQFVCITQTDYYRI-LHQGEENTKRHED-DG-------
>Rapgef2_[Drosophila_takahashii]_1036740816
AFTNITLAV---RRALCSVMVFAVVDKAGTV---VMSDGEELDS---WSVLINGAVEIEH
---AHGS---------------R-E---E-LQMG-DSFGILPTMDKLYHRGVMRTKCD--
-----D-CQFVCITQTDYYRI-QHQGEENTRRHED----------
>Rapgef2_[Dufourea_novaeangliae]_1016162772
AFTNMTLAV---RRALCAVMVFAVVERAGMI---VLNDGEELDS---WSVLINGAVEIDH
---SNGD---------------I-E---Q-LHLG-DSFGILPTMERLLHRGVMRTKCD--
-----D-CQFVCVTQADYFRI-QHQGEENTRRHE-ENGRVI----
>Rapgef2_[Echinops_telfairi]_507671420
AFANMTMSV---RRELCAVMVFAVVERAGTI---VLNDGEELDS---WSVILNGSVEVTY
---PDGK---------------A-E---I-LCMG-NSFGVSPTMDKEYMKGVMRTKVD--
-----D-CQFVCIAQQDYCRI-LNQVEKNMQKVEE-EGEIV----
>Rapgef2_[Eptesicus_fuscus]_641691941
AFANMTMSV---RRELCAVMVFAVVERAGTI---VLNDGEELDS---WSVILNGSVEVTY
---PDGK---------------A-E---I-LCMG-NSFGVSPTMDKEYMKGVMRTKVD--
-----D-CQFVCIAQQDYCRI-LNQVEKNMQKVEE-EGEIV----
>Rapgef2_[Equus_asinus]_958803741
AFANMTMSV---RRELCAVMVFAVVERAGTI---VLNDGEELDS---WSVILNGSVEVTY
---PDGK---------------A-E---I-LCMG-NSFGVSPTMDKEYMKGVMRTKVD--
-----D-CQFVCIAQQDYCRI-LNQVEKNMQKVEE-EGEIV----
>Rapgef2_[Equus_caballus]_953847560
AFANMTMSV---RRELCAVMVFAVVERAGTI---VLNDGEELDS---WSVILNGSVEVTY
---PDGK---------------A-E---I-LCMG-NSFGVSPTMDKEYMKGVMRTKVD--
-----D-CQFVCIAQQDYCRI-LNQVEKNMQKVEE-EGEIV----
>Rapgef2_[Esox_lucius]_742092552
AFANMTMSV---RRELCAVMVFAVVERAGTI---VLNDGEELDS---WSVILNGSVEVTY
---PEGR---------------P-E---I-LCMG-NSFGVSPTMEKEYMKGVMKTKVD--
-----D-CQFVCIAQQDYCCI-LNQVEKNMQKVEE-EGEIV----
>Rapgef2_[Falco_cherrug]_541968958
AFANMTMSV---RRELCAVMVFAVVERAGTI---VLNDGEELDS---WSVILNGSVEVTY
---PDGR---------------T-E---I-LCMG-NSFGVSPTMEKEYMKGVMRTKVD--
-----D-CQFVCIAQQDYCRI-LNQVEKNMQKVEE-EGEIV----
>Rapgef2_[Felis_catus]_755716579
AFANMTMSV---RRELCAVMVFAVVERAGTI---VLNDGEELDS---WSVILNGSVEVTY
---PDGK---------------A-E---I-LCMG-NSFGVSPTMDKEYMKGVMRTKVD--
-----D-CQFVCIAQQDYCRI-LNQVEKNMQKVEE-EGEIV----
```

>Rapgef2_[Ficedula_albicollis]_1020979017
AFANMTMSV---RRELCAVMVFAVVERAGTI---VLNDGEELDS---WSVILNGSVEVTY
---PDGR---------------T-E---I-LCMG-NSFGVSPTMEKEYMKGVMRTKVD--
-----D-CQFVCIAQQDYCRI-LNQVEKNMQKVEE-EGEIV----
>Rapgef2_[Fukomys_damarensis]_731224407
AFANMTMSV---RRELCAVMVFAVVERAGTI---VLNDGEELDS---WSVILNGSVEVTY
---PDGK---------------A-E---I-LCMG-NSFGVSPTMDKEYMKGVMRTKVD--
-----D-CQFVCIAQQDYCRI-LNQVEKNMQKVEE-EGEIV----
>Rapgef2_[Fundulus_heteroclitus]_831545540
AFANMTMSV---RRELCAVMVFAVVERAGTI---VLNDGEELDS---WSVILNGSVEVTH
---PDGQ---------------T-E---M-LCMG-NSFGVSPTMEKEFMKGVMRTKVD--
-----D-CQFVCIAQQEYCCI-LNQVEKNMQKVEE-EGEIV----
>Rapgef2_[Galeopterus_variegatus]_667288983
AFANMTMSV---RRELCAVMVFAVVERAGTI---VLNDGEELDS---WSVILNGSVEVTY
---PDGK---------------A-E---I-LCMG-NSFGVSPTMDKEYMKGVMRTKVD--
-----D-CQFVCIAQQDYCRI-LNQVEKNMQKVEE-EGEIV----
>Rapgef2_[Geospiza_fortis]_543244460
AFANMTMSV---RRELCAVMVFAVVERAGTI---VLNDGEELDS---WSVILNGSVEVTY
---PDGR---------------T-E---I-LCMG-NSFGVSPTMEKEYMKGVMRTKVD--
-----D-CQFVCIAQQDYCRI-LNQVEKNMQKVEE-EGEIV----
>Rapgef2_[Haliaeetus_albicilla]_700326780
AFANMTMSV---RRELCAVMVFAVVERAGTI---VLNDGEELDS---WSVILNGSVEVTY
---PDGR---------------T-E---I-LCMG-NSFGVSPTMEKEYMKGVMRTKVD--
-----D-CQFVCIAQQDYCRI-LNQVEKNMQKVEE-EGEIV----
>Rapgef2_[Haliaeetus_leucocephalus]_729726817
AFANMTMSV---RRELCAVMVFAVVERAGTI---VLNDGEELDS---WSVILNGSVEVTY
---PDGR---------------T-E---I-LCMG-NSFGVSPTMEKEYMKGVMRTKVD--
-----D-CQFVCIAQQDYCRI-LNQVEKNMQKVEE-EGEIV----
>Rapgef2_[Harpegnathos_saltator]_749732955
AFTNMTLAV---RRALCAVMVFAVVERAGMV---VLNDGEELDS---WSVLINGAVEVEQ
---SNGE---------------I-Q---Q-LCLG-DCFGILPTMDRLLHCGVMRTKCD--
-----D-CQFVCVTQADYFRI-QHQGEENTRRHE-ENGRVI----
>Rapgef2_[Heterocephalus_glaber]_513014560
AFANMTMSV---RRELCAVMVFAVVERAGTT---VLNGGEELDS---WSVILNGSVEVTY
---PDGK---------------A-E---I-LCMG-NSFGVSPTMDKEYMKGVMRTKVD--
-----D-CQFVCIAQQDYCRI-LNQVEKNMQKVEE-EGEIVMV--
>Rapgef2_[Ictalurus_punctatus]_1042371527
AFANMTMSV---RRELCAVMVFAVVERAGTI---VLNDGEELDS---WSVILNGSVEVTY
---PEGR---------------T-E---I-LCMG-NSFGVSPSREKEFMKGVMKTKVD--
-----D-CQFVCIAQQDYCCI-LNQVEKNMQKVEE-EGEIV----
>Rapgef2_[Ictidomys_tridecemlineatus]_914915197
AFANMTMSV---RRELCAVMVFAVVERAGTI---VLNDGEELDS---WSVILNGSVEVTY
---PDGK---------------A-E---I-LCMG-NSFGVSPTMDKEYMKGVMRTKVD--
-----D-CQFVCIAQQDYCRI-LNQVEKNMQKVEE-EGEIV----
>Rapgef2_[Kryptolebias_marmoratus]_1041100152
AFANMTMSV---RRELCAVMVFAVVERAGTI---VLNDGEELDS---WSVILNGSVEVTY
---PDGR---------------T-E---I-LCMG-NSFGVSPTMEKEYMKGVMKTKVD--
-----D-CQFVCIAQQDYCCI-LNQVEKNMQKVEE-EGEIV----
>Rapgef2_[Latimeria_chalumnae]_556992865
AFANMTMSV---RKELCAVMVFAVVERAGTI---VLNDGEELDS---WSVILNGSVEVTY
---PDGR---------------T-E---I-LCMG-NSFGVSPTMEKEYMKGVMKTKVD--
-----D-CQFVCIAQQDYCRI-LNQVEKNMQKVEE-EGEIV----
>Rapgef2_[Lepisosteus_oculatus]_972965615
AFANMTMSV---RRELCAVMVFAVVERAGTI---VLNDGEELDS---WSVILNGSVEVTY

---PDGR---------------T-E---I-LCMG-NSFGVSPTLEKEYMKGVMKTKVD--
-----D-CQFVCIAQQDYCCI-LNQVEKNMQKVEE-EGEIV----
>Rapgef2_[Leptonychotes_weddellii]_585176488
AFANMTMSV---RRELCAVMVFAVVERAGTI---VLNDGEELDS---WSVILNGSVEVTY
---PDGK---------------A-E---I-LCMG-NSFGVSPTMDKEYMKGVMRTKVD--
-----D-CQFVCIAQQDYCRI-LNQVEKNMQKVEE-EGEIV----
>Rapgef2_[Limulus_polyphemus]_926648335
AFANMTIAV---RRALCAVMVFAVVQKAGTV---VMNDGEELDS---WSVIVNGQVEVDF
---PDGT---------------S-H---E-LHVG-DSFGITATTEKMYHEGVMRTMLD--
-----D-CQFVCIAQSDYHKI-LHQGEENTRKHEE-NGQVV----
>Rapgef2_[Lipotes_vexillifer]_602704040
AFANMTMSV---RRELCAVMVFAVVERAGTI---VLNNGEELDS---WSVILNGSVEVTY
---PDGK---------------A-E---I-LCMG-NSFGVSPTMDKEYMKGVMRTKVD--
-----D-CQFVCIAQQDYCRI-LNQVEKNMQKVEE-EGEIV----
>Rapgef2_[Macaca_fascicularis]_544435351
AFANMTMSV---RRELCAVMVFAVVERAGTI---VLNDGEELDS---WSVILNGSVEVTY
---PDGK---------------A-E---I-LCMG-NSFGVSPTMDKEYMKGVMRTKVD--
-----D-CQFVCIAQQDYCRI-LNQVEKNMQKVEE-EGEIV----
>Rapgef2_[Macaca_mulatta]_966945521
AFANMTMSV---RRELCAVMVFAVVERAGTI---VLNDGEELDS---WSVILNGSVEVTY
---PDGK---------------A-E---I-LCMG-NSFGVSPTMDKEYMKGVMRTKVD--
-----D-CQFVCIAQQDYCRI-LNQVEKNMQKVEE-EGEIV----
>Rapgef2_[Macaca_nemestrina]_795381724
AFANMTMSV---RRELCAVMVFAVVERAGTI---VLNDGEELDS---WSVILNGSVEVTY
---PDGK---------------A-E---I-LCMG-NSFGVSPTMDKEYMKGVMRTKVD--
-----D-CQFVCIAQQDYCRI-LNQVEKNMQKVEE-EGEIV----
>Rapgef2_[Mandrillus_leucophaeus]_795325379
AFANMTMSV---RRELCAVMVFAVVERAGTI---VLNDGEELDS---WSVILNGSVEVTY
---PDGK---------------A-E---I-LCMG-NSFGVSPTMDKEYMKGVMRTKVD--
-----D-CQFVCIAQQDYCRI-LNQVEKNMQKVEE-EGEIV----
>Rapgef2_[Melipona_quadrifasciata]_925684072
AFTNMTLAV---RRALCAVMVFAVVERAGMI---VLNDGEELDS---WSVLINGAVEIEH
---SNGE---------------I-E---Q-LHLG-DSFGILPTMERLLHRGVMRTKCD--
-----D-CQFVCVTQADYFRI-QHQGEENTRRHE-ENGRVI----
>Rapgef2_[Melopsittacus_undulatus]_884809301
AFANMTMSV---RRELCAVMVFAVVERAGTI---VLNDGEELDS---WSVILNGSVEVTY
---PDGR---------------T-E---I-LCMG-NSFGVSPTMEKEYMKGVMRTKVD--
-----D-CQFVCIAQQDYCRI-LNQVEKNMQKVEE-EGEIV----
>Rapgef2_[Miniopterus_natalensis]_1016655590
AFANMTMSV---RRELCAVMVFAVVERAGTI---VLNDGEELDS---WSVILNGSVEVTY
---PDGK---------------A-E---I-LCMG-NSFGVSPTMDKEYMKGVMRTKVD--
-----D-CQFVCIAQQDYCRI-LNQVEKNMQKVEE-EGEIV----
>Rapgef2_[Monodelphis_domestica]_612030789
AFANMTMSV---RRELCAVMVFAVVERAGTI---VLNDGEELDS---WSVILNGSVEVTY
---PDGR---------------T-E---I-LCMG-NSFGVSPTMDKEYMKGVMRTKVD--
-----D-CQFVCIAQQDYCRI-LNQVEKNMQKVEE-EGEIV----
>Rapgef2_[Mus_musculus]_887221696
AFANMTMSV---RRELCAVMVFAVVERAGTI---VLNDGEELDS---WSVILNGSVEVTY
---PDGK---------------A-E---I-LCMG-NSFGVSPTMDKEYMKGVMRTKVD--
-----D-CQFVCIAQQDYCRI-LNQVEKNMQKVEE-EGEIV----
>Rapgef2_[Mustela_putorius_furo]_859933733
AFANMTMSV---RRELCAVMVFAVVERAGTI---VLNDGEELDS---WSVILNGSVEVTY
---PDGK---------------A-E---I-LCMG-NSFGVSPTMDKEYMKGVMRTKVD--
-----D-CQFVCIAQQDYCRI-LNQVEKNMQKVEE-EGEIV----

>Rapgef2_[Myotis_davidii]_584034002
AFANMTMSV---RRELCAVMVFAVVERAGTI---VLNDGEELDS---WSVILNGSVEVTY
---PDGK---------------A-E---I-LCMG-NSFGVSPTMDKEYMKGVMRTKVD--
-----D-CQFVCIAQQDYCRI-LNQVEKNMQKVEE-EGEIV----
>Rapgef2_[Nannospalax_galili]_674089174
AFANMTMSV---RRELCAVMVFAVVERAGTI---VLNDGEELDS---WSVILNGSVEVTY
---PDGK---------------A-E---I-LCMG-NSFGVSPTMDKEYMKGVMRTKVD--
-----D-CQFVCIAQQDYCRI-LNQVEKNMQKVEE-EGEIV----
>Rapgef2_[Nasonia_vitripennis]_1032757814
AFTNMTLAV---RRALCAVMVFAVVERAGMV---VLTDGEELDS---WSVLINGTVEVEH
----NGT---------------V-D---Q-LHVG-DSFGILPTMETLLHRGIMRTKCD--
-----D-CQFVCVTQVDYYRI-QHQGEENTRRHE-EDGRVI----
>Rapgef2_[Nothobranchius_furzeri]_1007706565
AFANMTMSV---RRELCAVMVFAVVERAGTI---VLNDGEELDS---WSVILNGSVEVTH
---PDSR---------------T-E---I-LCMG-NSFGVSPTMEKEYMKGVMKTKVD--
-----D-CQFVCIAQQEYCCI-LNQVEKNMQKVEE-EGEIV----
>Rapgef2_[Notothenia_coriiceps]_736169539
AFANMTMSV---RRELCAVMVFAVVERAGTI---VLNDGEELDS---WSVILNGSVEVTY
---PEGR---------------T-E---I-LCMG-NSFGVSPTMEKEYMKGVMKTKVD--
-----D-CQFVCIAQQDYCCI-LNQVEKNMQKVEE-EGEIV----
>Rapgef2_[Ochotona_princeps]_837819646
AFANMTMSV---RRELCAVMVFAVVERAGTI---VLNDGEELDS---WSVILNGSVEVTY
---PDGK---------------A-E---I-LCMG-NSFGVSPTMDKEYMKGVMRTKVD--
-----D-CQFVCIAQQDYCRI-LNQVEKNMQKVEE-EGEIV----
>Rapgef2_[Octodon_degus]_820993488
AFANMTMSV---RRELCAVMVFAVVERAGTI---VLNDGEELDS---WSVILNGSVEVTY
---PDGK---------------A-E---I-LCMG-NSFGVSPTMDKEHMKGVMRTKVD--
-----D-CQFVCIAQQDYCRI-LNQVEKNMQKVEE-EGEIV----
>Rapgef2_[Odobenus_rosmarus_divergens]_823395200
AFANMTMSV---RRELCAVMVFAVVERAGTI---VLNDGEELDS---WSVILNGSVEVTY
---PDGK---------------A-E---I-LCMG-NSFGVSPTMDKEYMKGVMRTKVD--
-----D-CQFVCIAQQDYCRI-LNQVEKNMQKVEE-EGEIV----
>Rapgef2_[Opisthocomus_hoazin]_700389884
AFANMTMSV---RRELCAVMVFAVVERAGTI---VLNDGEELDS---WSVILNGSVEVTY
---PDGR---------------T-E---I-LCMG-NSFGVSPTMEKEYMKGVMRTKVD--
-----D-CQFVCIAQQDYCRI-LNQVEKNMQKVEE-EGEIV----
>Rapgef2_[Ornithorhynchus_anatinus]_1019060572
AFANMTMSV---RRELCAVMVFAVVERAGTI---VLNDGEELDS---WSVILNGSVEVTY
---PDGR---------------T-E---I-LCMG-NSFGVSPTMDKEYMKGVMRTKVD--
-----D-CQFVCIAQQDYCRI-LNQVEKNMQKVEE-EGEIV----
>Rapgef2_[Orycteropus_afer_afer]_634878373
AFANMTMSV---RRELCAVMVFAVVERAGTI---VLNDGEELDS---WSVILNGSVEVTY
---PDGK---------------A-E---I-LCMG-NSFGVSPTMDKEYMKGVMRTKVD--
-----D-CQFVCIAQQDYCRI-LNQVEKNMQKVEE-EGEIV----
>Rapgef2_[Oryctolagus_cuniculus]_655879031
AFANMTMSV---RRELCAVMVFAVVERAGTI---VLNDGEELDS---WSVILNGSVEVTY
---PDGK---------------A-E---I-LCMG-NSFGVSPTMDKEYMKGVMRTKVD--
-----D-CQFVCIAQQDYCRI-LNQVEKNMQKVEE-EGEIV----
>Rapgef2_[Ovis_aries_musimon]_803287889
AFANMTMSV---RRELCAVMVFAVVERAGTI---VLNDGEELDS---WSVILNGSVEVTY
---PDGK---------------A-E---I-LCMG-NSFGVSPTMDKEYMKGVMRTKVD--
-----D-CQFVCIAQQDYCRI-LNQVEKNMQKVEE-EGEIV----
>Rapgef2_[Pan_paniscus]_931570497
AFANMTMSV---RRELCAVMVFAVVERAGTI---VLNDGEELDS---WSVILNGSVEVTY

---PDGK---------------A-E---I-LCMG-NSFGVSPTMDKEYMKGVMRTKVD--
-----D-CQFVCIAQQDYCRI-LNQVEKNMQKVEE-EGEIV----
>Rapgef2_[Pan_troglodytes]_1034166384
AFANMTMSV---RRELCAVMVFAVVERAGTI---VLNDGEELDS---WSVILNGSVEVTY
---PDGK---------------A-E---I-LCMG-NSFGVSPTMDKEYMKGVMRTKVD--
-----D-CQFVCIAQQDYCRI-LNQVEKNMQKVEE-EGEIV----
>Rapgef2_[Panthera_tigris_altaica]_987408704
AFANMTMSV---RRELCAVMVFAVVERAGTI---VLNDGEELDS---WSVILNGSVEVTY
---PDGK---------------A-E---I-LCMG-NSFGVSPTMDKEYMKGVMRTKVD--
-----D-CQFVCIAQQDYCRI-LNQVEKNMQKVEE-EGEIV----
>Rapgef2_[Papio_anubis]_685543722
AFANMTMSV---RRELCAVMVFAVVERAGTI---VLNDGEELDS---WSVILNGSVEVTY
---PDGK---------------A-E---I-LCMG-NSFGVSPTMDKEYMKGVMRTKVD--
-----D-CQFVCIAQQDYCRI-LNQVEKNMQKVEE-EGEIV----
>Rapgef2_[Parus_major]_998685098
AFANMTMSV---RRELCAVMVFAVVERAGTI---VLNDGEELDS---WSVILNGSVEVTY
---PDGR---------------T-E---I-LCMG-NSFGVSPTMEKEYMKGVMRTKVD--
-----D-CQFVCIAQQDYCRI-LNQVEKNMQKVEE-EGEIV----
>Rapgef2_[Pelecanus_crispus]_677469268
AFANMTMSV---RRELCAVMVFAVVERAGTI---VLNDGEELDS---WSVILNGSVEVTY
---PDGR---------------T-E---I-LCMG-NSFGVSPTMEKEYMKGVMRTKVD--
-----D-CQFVCIAQQDYCRI-LNQVEKNMQKVEE-EGEIV----
>Rapgef2_[Peromyscus_maniculatus_bairdii]_1008761808
AFANMTMSV---RRELCAVMVFAVVERAGTI---VLNDGEELDS---WSVILNGSVEVTY
---PDGK---------------A-E---I-LCMG-NSFGVSPTMDKEYMKGVMRTKVD--
-----D-CQFVCIAQQDYCRI-LNQVEKNMQKVEE-EGEIV----
>Rapgef2_[Phaethon_lepturus]_723143940
AFANMTMSV---RRELCAVMVFAVVERAGTI---VLNDGEELDS---WSVILNGSVEVTY
---PDGR---------------T-E---I-LCMG-NSFGVSPTMEKEYMKGVMRTKVD--
-----D-CQFVCIAQQDYCRI-LNQVEKNMQKVEE-EGEIV----
>Rapgef2_[Phalacrocorax_carbo]_679211993
AFANMTMSV---RRELCAVMVFAVVERAGTI---VLNDGEELDS---WSVILNGSVEVTY
---PDGR---------------T-E---I-LCMG-NSFGVSPTMEKEYMKGVMRTKVD--
-----D-CQFVCIAQQDYCRI-LNQVEKNMQKVEE-EGEIV----
>Rapgef2_[Poecilia_formosa]_1025435394
AFANMTMSV---RRELCAVMVFAVVERAGTI---VLNDGEELDS---WSVILNGSVEVTY
---PDSR---------------T-E---M-LCMG-NSFGVSPTMEKEFMKGVMRTKVD--
-----D-CQFVCIAQQDYCCI-LNQVEKNMQKVEE-EGEIV----
>Rapgef2_[Propithecus_coquereli]_826315269
AFANMTMSV---RRELCAVMVFAVVERAGTI---VLNDGEELDS---WSVILNGSVEVTY
---PDGK---------------A-E---I-LCMG-NSFGVSPTMDKEYMKGVMRTKVD--
-----D-CQFVCIAQQDYCRI-LNQVEKNMQKVEE-EGEIV----
>Rapgef2_[Protobothrops_mucrosquamatus]_1002581465
AFANMTMSV---RRELCAVMVFAVVERAGTI---VLNDGEELDS---WSVILNGSVEVTY
---PDGR---------------T-E---I-LCMG-NSFGVSPTMDKEYMKGIMRTKVD--
-----D-CQFVCIAQQDYCRI-LNQVEKNMQKVEE-EGEIV----
>Rapgef2_[Pterocles_gutturalis]_704510117
AFANMTMSV---RRELCAVMVFAVVERAGTI---VLNDGEELDS---WSVILNGSVEVTY
---PDGR---------------T-E---I-LCMG-NSFGVSPTMEKEYMKGVMRTKVD--
-----D-CQFVCIAQQDYCRI-LNQVEKNMQKVEE-EGEIV----
>Rapgef2_[Python_bivittatus]_602665199
AFANMTMSV---RRELCAVMVFAVVERAGTI---VLNDGEELDS---WSVILNGSVEVTY
---PDGR---------------T-E---I-LCMG-NSFGVSPTMDKEYMKGIMRTKVD--
-----D-CQFVCIAQQDYCRI-LNQVEKNMQKVEE-EGEIV----

>Rapgef2_[Rhinopithecus_roxellana]_724803574
AFANMTMSV---RRELCAVMVFAVVERAGTI---VLNDGEELDS---WSVILNGSVEVTY
---PDGK---------------A-E---I-LCMG-NSFGVSPTMDKEYMKGVMRTKVD--
-----D-CQFVCIAQQDYCRI-LNQVEKNMQKVEE-EGEIV----
>Rapgef2_[Rousettus_aegyptiacus]_1012013897
AFANMTMSV---RRELCAVMVFAVVERAGTI---VLNDGEELDS---WSVILNGSVEVTY
---PDGK---------------A-E---I-LCMG-NSFGVSPTMDKEYMKGVMRTKVD--
-----D-CQFVCIAQQDYCRI-LNQVEKNMQKVEE-EGEIV----
>Rapgef2_[Saimiri_boliviensis_boliviensis]_403272230
AFANMTMSV---RRELCAVMVFAVVERAGTI---VLNDGEELDS---WSVILNGSVEVTY
---PDGK---------------A-E---I-LCMG-NSFGVSPTMDKEYMKGVMRTKVD--
-----D-CQFVCIAQQDYCRI-LNQVEKNMQKVEE-EGEIV----
>Rapgef2_[Sarcophilus_harrisii]_821490713
AFANMTMSV---RRELCAVMVFAVVERAGTI---VLNDGEELDS---WSVILNGSVEVTY
---PDGR---------------T-E---I-LCMG-NSFGVSPTMDKEYMKGVMRTKVD--
-----D-CQFVCIAQQDYCRI-LNQVEKNMQKVEE-EGEIV----
>Rapgef2_[Scleropages_formosus]_938067446
AFANMTMSV---RRELCAVMVFAVVERAGTV---VLNDGEELDS---WSVILNGSVEVTH
---PDGR---------------T-E---I-LCMG-NSFGVSPTMEKEFMKGVMKTKVD--
-----D-CQFVCIAQQDYCCI-LNQVEKNMQKVEE-EGEIV----
>Rapgef2_[Sinocyclocheilus_anshuiensis]_1024980578
AFANMTMSV---RRELCAVMVFAVVERAGTV---VLNDGEELDS---WSVILNGSVEVTH
---PDGR---------------T-E---I-LCMG-NSFGVSPTMEKEYMKGVMKTKVD--
-----D-CQFVCIAQQDYCCI-LNQVEKNMQKVEE-EGEIV----
>Rapgef2_[Sinocyclocheilus_grahami]_1020470969
AFANMTMSV---RRELCAVMVFAVVERAGTV---VLNDGEELDS---WSVILNGSVEVTH
---PDGR---------------T-E---I-LCMG-NSFGVSPTMEKEYMKGVMKTKVD--
-----D-CQFVCIAQQDYCCI-LNQVEKNMQKVEE-EGEIV----
>Rapgef2_[Sinocyclocheilus_rhinocerous]_1025212222
AFANMTMSV---RRELCAVMVFAVVERAGTV---VLNDGEELDS---WSVILNGSVEVTH
---PDGR---------------T-E---I-LCMG-NSFGVSPTMEKEYMKGVMKTKVD--
-----D-CQFVCIAQQDYCCI-LNQVEKNMQKVEE-EGEIV----
>Rapgef2_[Sorex_araneus]_505792953
AFANMTMSV---RRELCAVMVFAVVERAGTI---VLNDGEELDS---WSVILNGSVEVTY
---PDGK---------------A-E---I-LCMG-NSFGVSPTMDKEYMKGVMRTKVD--
-----D-CQFVCIAQQDYCRI-LNQVEKNMQKVEE-EGEIV----
>Rapgef2_[Strongylocentrotus_purpuratus]_780127327
AFANMTMSV---RRALCSVMVFAVVSKAGTV---VMKDGEELDS---WSVILNGHVEVSK
---PDES---------------V-D---E-LHLG-DSFGVKPTMEKQTHSGVMRTTVD--
-----D-CQFVCIAQEHYYRI-LTQGEENIRRISE-DGVLV----
>Rapgef2_[Struthio_camelus_australis]_678215500
AFANMTMSV---RRELCAVMVFAVVERAGTI---VLNDGEELDS---WSVILNGSVEVTY
---PDGR---------------T-E---I-LCMG-NSFGVSPTMEKEYMKGVMRTKVD--
-----D-CQFVCIAQQDYCRI-LNQVEKNMQKVEE-EGEIV----
>Rapgef2_[Sturnus_vulgaris]_959071480
AFANMTMSV---RRELCAVMVFAVVERAGTI---VLNDGEELDS---WSVILNGSVEVTY
---PDGR---------------T-E---I-LCMG-NSFGVSPTMEKEYMKGVMRTKVD--
-----D-CQFVCIAQQDYCRI-LNQVEKNMQKVEE-EGEIV----
>Rapgef2_[Takifugu_rubripes]_768938240
AFANMTMSV---RRELCAVMVFAVVERAGTI---VLNDGEELDS---WSVILNGSVEVTY
---PEGR---------------T-E---I-LCMG-NSFGVSPTMEKEYMKGVMKTKVD--
-----D-CQFVCIAQQDYCCI-LNQVEKNMQKVEE-EGEIV----
>Rapgef2_[Trachymyrmex_septentrionalis]_1009425222
AFTNMTLAV---RRALCAVMVFAVVDRAGMV---VLNDGEELDS---WSVLINGAVEIEH

---SNGE---------------I-E---Q-LGLG-DSFGILPTMERLLHRGVMRTKCD--
-----D-CQFVCVTQADYFRI-QHQGEENTRRHE-ENGRVI----
>Rapgef2_[Trichechus_manatus_latirostris]_471397359
AFANMTMSV---RRELCAVMVFAVVERAGTI---VLNDGEELDS---WSVILNGSVEVTY
---PDGK---------------A-E---I-LCMG-NSFGVSPTMDKEYMKGIMRTKVD--
-----D-CQFVCIAQQDYCRI-LNQVEKNMQKVEE-EGEIV----
>Rapgef2_[Tyto_alba]_701382803
AFANMTMSV---RRELCAVMVFAVVERAGTI---VLNDGEELDS---WSVILNGSVEVTY
---PDGR---------------T-E---I-LCMG-NSFGVSPTMEKEYMKGVMRTKVD--
-----D-CQFVCIAQQDYCRI-LNQVEKNMQKVEE-EGEIV----
>Rapgef2_[Ursus_maritimus]_670985380
AFANMTMSV---RRELCAVMVFAVVERAGTI---VLNDGEELDS---WSVILNGSVEVTY
---PDGK---------------A-E---I-LCMG-NSFGVSPTMDKEYMKGVMRTKVD--
-----D-CQFVCIAQQDYCRI-LNQVEKNMQKVEE-EGEIV----
>Rapgef2_[Vicugna_pacos]_970736793
AFANMTMSV---RRELCAVMVFAVVERAGTI---VLNDGEELDS---WSVILNGSVEVTY
---PDGK---------------A-E---I-LCMG-NSFGVSPTMDKEYMKGVMRTKVD--
-----D-CQFVCIAQQDYCRI-LNQVEKNMQKVEE-EGEIV----
>Rapgef2_[Vollenhovia_emeryi]_795102090
AFTNMTLAV---RRALCAVMVFAVVDRAGMV---VLNDGEELDS---WSVLINGAVEIEH
---TSGE---------------I-E---Q-LGLG-DSFGILPTMERLLHRGVMRTKCD--
-----D-CQFVCVTQADYFRI-QHQGEENTRRHE-ENGRVI----
>Rapgef2_[Xenopus_laevis]_147907136
AFANMTMSV---RRELCAVMVFAVVERAGTV---VLNDGEELDS---WSVILNGSVEVTY
---PDGR---------------T-E---I-LCMG-NSFGVSPTMEKEYMKGVMRTKVD--
-----D-CQFVCIAQQDYCRI-LNQVEKNMQKVEE-EGEIV----
>Rapgef2_[Zonotrichia_albicollis]_929469278
AFANMTMSV---RRELCAVMVFAVVERAGTI---VLNDGEELDS---WSVILNGSVEVTY
---PDGR---------------T-E---I-LCMG-NSFGVSPTMEKEYMKGVMRTKVD--
-----D-CQFVCIAQQDYCRI-LNQVEKNMQKVEE-EGEIV----
>Rapgef2_[Zootermopsis_nevadensis]_646721548
AFTNMTLAV---RRALCSVMVFAVVEKAGTV---VMNDGEELDS---WSVLINGHVEVEH
---VNGE---------------T-E---Q-LQVG-DSFGILPTMDKLYHRGVMRTRCD--
-----D-CQFVCITQTDYYRI-QHQGEENIRRHEE-DGRVV----
>Rapgef3_[Acinonyx_jubatus]_961722103
AVAHLSNSV---KRELAAVLLFEPHSRAGTV---LFSQGDKGTS---WYIIWKGSVNVVT
H----GKGL-------------V-T---T-LHEG-DDFGQLALVNDAPRAATIILRED--
-----N-CHFLRVDKQDFNRI-IKDVEAKTMRLEEHGKVV-----
>Rapgef3_[Ailuropoda_melanoleuca]_752406281
AVAHLSNSV---KRELAAVLLFEPHSKAGTV---LFSQGDKGTS---WYIIWKGSVNVVT
H----GKGL-------------V-T---T-LHEG-DDFGQLALVNDAPRAATIILRED--
-----N-CHFLRVDKQDFNRI-IKDVEAKTMRLEEHGKVV-----
>Rapgef3_[Alligator_mississippiensis]_950944900
AVAYLSSSV---KRELASVVLFESHARAGTV---LFSQGDKGTS---WYILWKGSVNVVT
H----GKGL-------------V-T---T-LHEG-DDFGQLALVNDAPRAATIILRED--
-----S-CHFLRVDKHDFNRI-LKDVEANTIRLKEHGKVV-----
>Rapgef3_[Alligator_sinensis]_944358354
AVAYLSSSV---KRELASVVLFESHARAGTV---LFSQGDKGTS---WYILWKGSVNVVT
H----GKGL-------------V-T---T-LHEG-DDFGQLALVNDAPRAATIILRED--
-----S-SHFLRVDKHDFNRI-LKDVEANTIRLKEHGKVV-----
>Rapgef3_[Anolis_carolinensis]_1033369210
AVAHLSSSV---KRELAAVLLFESHTKAGTV---LFSQGDKATS---WYIIWKGSVNVVT
H----DKGL-------------V-S---T-LHEG-DEFGQLALVNDAPRAATIILRED--
-----N-CHFLRVDKRDFNRI-LKDVEANTMRLKEHGKVV-----

>Rapgef3_[Anser_cygnoides_domesticus]_902933497
AVAHLSNSV---KRELAAVLMFESHQRAGTV---LFSQGDKGTS---WYIVWKGSVNVVT
H----GKGL-------------V-A---T-LHEG-DDFGQLALVNDAPRAASIILRED--
-----N-CHFLRVDKQDFNHI-LKDVEANTMRLKEHGKVV-----
>Rapgef3_[Aotus_nancymaae]_817288343
AVAHLSNSV---KRELAAVLLFEPHSKAGTV---LFSQGDKGTS---WYIIWKGSVNVVT
H----GKGL-------------V-T---T-LHEG-DDFGQLALVNDAPRAATIILRED--
-----N-CHFLRVDKQDFNRI-IKDVEAKTMRLEEHGKVV-----
>Rapgef3_[Aptenodytes_forsteri]_675309006
AVAHLSNSV---KRELASVLMFESHQRAGTV---LFSQGDKGTS---WYIVWKGSVNVVT
H----GKGL-------------V-A---T-LHEG-DDFGQLALVNDAPRAATIILRED--
-----N-CHFLRVDKQDFNHI-LKDVEANTMRLKEHGKVV-----
>Rapgef3_[Apteryx_australis_mantelli]_926500193
AVAHLSNSV---KRELASVLMFESHQRAGTV---LFSQGDKGTS---WYIIWKGSVNVVT
H----GKGL-------------V-A---T-LHEG-DDFGQLALVNDAPRAATIILRED--
-----N-CHFLRVDKQDFNRI-LKDVEANTMRLKEHGKVV-----
>Rapgef3_[Aquila_chrysaetos_canadensis]_768403048
AVAHLSNSV---KRELASVLMFESHQRAGTV---LFSQGDKGTS---WYIIWKGSVNVVT
H----GKGL-------------V-A---T-LHEG-DDFGQLALVNDAPRAATIILRED--
-----N-CHFLRVDKQDFNHI-LKDVEANTMRLKEHGKVV-----
>Rapgef3_[Austrofundulus_limnaeus]_928045560
AAAHLSASV---RKELAAVLVFESHAKAGTV---LFSQGDKGTS---WYIIWKGSVNVIT
H----GKGL-------------V-T---T-LHEG-EDFGQLALLNDAPRAATIILRED--
-----N-CHFLRVDKQDFIRI-LKDVEANTVRLEEHGKTV-----
>Rapgef3_[Balaenoptera_acutorostrata_scammoni]_594662223
AVAHLSNSV---KRELAAVLLFEPHSKAGTV---LFSQGDKGTS---WYIIWKGSVNVVT
H----GKGL-------------V-T---T-LHEG-DDFGQLALVNDAPRAATIILRED--
-----N-CHFLRVDKQDFNRI-IKDVEAKTMRLEEHGKVV-----
>Rapgef3_[Bison_bison_bison]_742108280
AVAHLSNSV---KRELAAVLLFEPHSKAGTV---LFSQGDKGTS---WYIIWKGSVNVVT
H----GKGL-------------V-T---T-LHEG-DDFGQLALVNDAPRAATIILGED--
-----N-CHFLRVDKQDFNRI-IKDVEAKTMRLEEHGKVV-----
>Rapgef3_[Bos_mutus]_555987327
AVAHLSNSV---KRELAAVLLFEPHSKAGTV---LFSQGDKGTS---WYIIWKGSVNVVT
H----GKGL-------------V-T---T-LHEG-DDFGQLALVNDAPRAATIILGED--
-----N-CHFLRVDKQDFNRI-IKDVEAKTMRLEEHGKVV-----
>Rapgef3_[Bos_taurus]_528949062
AVAHLSNSV---KRELAAVLLFEPHSKAGTV---LFSQGDKGTS---WYIIWKGSVNVVT
H----GKGL-------------V-T---T-LHEG-DDFGQLALVNDAPRAATIILGED--
-----N-CHFLRVDKQDFNRI-IKDVEAKTMRLEEHGKVV-----
>Rapgef3_[Bubalus_bubalis]_594071309
AVAHLSNSV---KRELAAVLLFEPHSKAGTV---LFSQGDKGTS---WYIIWKGSVNVVT
H----GKGL-------------V-T---T-LHEG-DDFGQLALVNDAPRAATIILGED--
-----N-CHFLRVDKQDFNRI-IKDVEAKTMRLEEHGKVV-----
>Rapgef3_[Callithrix_jacchus]_675676227
AVAHLSNSV---KRELAAVLLFEPHSKAGTV---LFSQGDKGTS---WYIIWKGSVNVVT
H----GKGL-------------V-T---T-LHEG-DDFGQLALVNDAPRAATIILRED--
-----N-CHFLRVDKQDFNRI-IKDVEAKTMRLEEHGKVV-----
>Rapgef3_[Calypte_anna]_663267862
AVAHLSNSV---KRELASVLMFESHQRAGTV---LFSQGDKGTS---WFIIWKGSVNVVT
H----GKGL-------------V-A---H-LH-X-XXXXXXXLVNDAPRAATIILRED--
-----N-CHFLRVDKQDFNHI-LKDVEANTVRLKEHGKVV-----
>Rapgef3_[Camelus_bactrianus]_743736343
AVAHLSNSV---KRELAAVLLFEPHNKAGTV---LFSQGDKGTS---WYIIWKGSVNVVT

H----GKGL-------------V-T---T-LHEG-DDFGQLALVNDAPRAATIILRED--
-----N-CHFLRVDKQDFNRI-IKDVEAKTMRLEEHGKVV-----
>Rapgef3_[Camelus_dromedarius]_744616231
AVAHLSNSV---KRELAAVLLFEPHSKAGTV---LFSQGDKGTS---WYIIWKGSVNVVT
H----GKGL-------------V-T---T-LHEG-DDFGQLALVNDAPRAATIILRED--
-----N-CHFLRVDKQDFNRI-IKDVEAKTMRLEEHGKVV-----
>Rapgef3_[Camelus_ferus]_946669809
AVAHLSNSV---KRELAAVLLFEPHNKAGTV---LFSQGDKGTS---WYIIWKGSVNVVT
H----GKGL-------------V-T---T-LHEG-DDFGQLALVNDAPRAATIILRED--
-----N-CHFLRVDKQDFNRI-IKDVEAKTMRLEEHGKVV-----
>Rapgef3_[Canis_lupus_familiaris]_153791885
AVAHLSNSV---KRELAAVLLFEPHSKAGTV---LFSQGDKGTS---WYIIWKGSVNVVT
H----GKGL-------------V-T---T-LHEG-DDFGQLALVNDAPRAATIILRED--
-----N-CHFLRVDKQDFNRI-IKDVEAKTMRLEEHGKVV-----
>Rapgef3_[Capra_hircus]_926691591
AVAHLSNSV---KRELAAVLLFEPHSKAGTV---LFSQGDKGTS---WYIIWKGSVNVVT
H----GKGL-------------V-T---T-LHEG-DDFGQLALVNDAPRAATIILGED--
-----N-CHFLRVDKQDFNRI-IKDVEAKTMRLEEHGKVV-----
>Rapgef3_[Carlito_syrichta]_640792033
AVAHLSNSV---KRELAAVLVFEPHSKAGTV---LFSQGDKGTS---WYIIWKGSVNVVT
H----GKGL-------------V-T---T-LHEG-DDFGQLALVNDAPRAATIILRED--
-----N-CHFLRVDKQDFNRI-IKDVEAKTMRLEEHGKVV-----
>Rapgef3_[Cavia_porcellus]_348580717
AVAHLSNSV---KRELAAVLLFEPHNKAGTV---LFSQGDKGTS---WYIIWKGSVNVVT
H----GKGL-------------V-T---T-LHEG-DDFGQLALVNDAPRAATIILRED--
-----N-CHFLRVDKQDFNRI-IKDVEAKTMRLEEHGKVV-----
>Rapgef3_[Cebus_capucinus_imitator]_1044333210
AVAHLSNTV---KRELAAVLLFEPHSKAGTV---LFSQGDKGTS---WYIIWKGSVNVVT
H----GKGL-------------V-T---T-LHEG-DDFGQLALVNDAPRAATIILRED--
-----N-CHFLRVDKQDFNRI-IKDVEAKTMRLEEHGKVV-----
>Rapgef3_[Ceratotherium_simum_simum]_478509423
AVAHLSNSV---KRELAAVLLFEPHSKAGTV---LFSQGDKGTS---WYIIWKGSVNVVT
H----GKGL-------------V-T---T-LHEG-DDFGQLALVNDAPRAATIILRED--
-----N-CHFLRVDKQDFNRI-IKDVEAKTMRLEEHGKVV-----
>Rapgef3_[Cercocebus_atys]_795538404
AVAHLSNSV---KRELAAVLLFEPHSKAGTV---LFSQGDKGTS---WYIIWKGSVNVVT
H----GKGL-------------V-T---T-LHEG-DDFGQLALVNDAPRAATIILRED--
-----N-CHFLRVDKQDFNRI-IKDVEAKTMRLEEHGKVV-----
>Rapgef3_[Charadrius_vociferus]_699656507
AVAHLSNSV---KRELAAVLMFESHQRAGTVREYLFSQGDKGTS---WYIIWKGSVNVVT
H----GKGL-------------V-A---T-LHEG-DDFGQLALVNDAPRAATIILRED--
-----N-CHFLRVDKQDFNHI-LKDVEANTMRLKEHGKVV-----
>Rapgef3_[Chelonia_mydas]_465956413
AVAHLSHSV---KRELASVLLFESHPKAGTV---LFSQGDKGTS---WYIIWKGSVNVVT
H----GKGL-------------V-T---T-LHEG-DDFGQLALVNDAPRAATIILRED--
-----N-CHFLRVDKRDFNRI-LKDVEANTMRLREHGKVV-----
>Rapgef3_[Chinchilla_lanigera]_533187398
AVAHLSNSV---KRELAAVLLFESHSKAGTV---LFSQGDKGTS---WYIIWKGSVNVVT
H----GKGL-------------V-T---T-LHEG-DDFGQLALVNDAPRAATIILRED--
-----N-CHFLRVDKQDFNRI-IKDVEAKTMRLEEHGKVV-----
>Rapgef3_[Chlorocebus_sabaeus]_635064864
AVAHLSNSV---KRELAAVLLFEPHSKAGTV---LFSQGDKGTS---WYIIWKGSVNVVT
H----GKGL-------------V-T---T-LHEG-DDFGQLALVNDAPRAATIILRED--
-----N-CHFLRVDKQDFNRI-IKDVEAKTMRLEEHGKVV-----

>Rapgef3_[Chrysochloris_asiatica]_586469584
AVAHLSNSV---KRELAAVLLFEPHSKAGTV---LFSQGDKGTS---WYIIWKGSVNVVT
H----GKGL-------------V-T---T-LHEG-DDFGQLALVNDAPRAATIILRED--
-----N-CHFLRVDKQDFNRI-IKDVEAKTMRLEEHGKVV-----
>Rapgef3_[Clupea_harengus]_831315562
AAAHLSTSV---RKELAAVLAFESHTKGGTV---LFSQGDKGTS---WYIIWKGSVNVIT
H----GKGL-------------V-T---T-LHEG-EDFGQLALVNDAPRSATIIMRED--
-----N-CHFLRVDKQDFIRI-LKDVEANTMRLEEHGKAV-----
>Rapgef3_[Colobus_angolensis_palliatus]_795136380
AVAHLSNSV---KRELAAVLLFEPHSKAGTV---LFSQGDKGTS---WYIIWKGSVNVVT
H----GKGL-------------V-T---T-LHEG-DDFGQLALVNDAPRAATIILRED--
-----N-CHFLRVDKQDFNRI-IKDVEAKTMRLEEHGKVV-----
>Rapgef3_[Columba_livia]_543750387
--------V---KRELASVLMFESHPRAGTV---LFSQGDKGTS---WYIIWKGSVNVVT
H----GKGL-------------V-A---T-LHEG-DDFGQLALVNDAPRAATIILRED--
-----N-CHFLRVDKQDFNRI-LK--------------------
>Rapgef3_[Condylura_cristata]_830194828
AVAHLSNSV---KRELAAVLLFEPHSKAGTV---LFSQGDKGTS---WYIIWKGSVNVVT
H----GKGL-------------V-T---T-LHEG-DDFGQLALVNDAPRAATIILRED--
-----N-CHFLRVDKQDFNRI-IKDVEAKTMRLEEHGKVV-----
>Rapgef3_[Coturnix_japonica]_1003995665
AVAHLSNSV---KRELASVLMFESHQRAGTV---LFSQGDKGTS---WYIVWKGSVNVVT
H----GKGL-------------V-A---T-LHEG-DDFGXLALVNDAPRAASIILRED--
-----N-CHFLRVDKQDFNRI-LKDVEANTMRLKEHGKVV-----
>Rapgef3_[Cricetulus_griseus]_625224024
AVAHLSNSV---KRELAAVLLFEPHSKAGTV---LFSQGDKGTS---WYIIWKGSVNVVT
H----GKGL-------------V-T---T-LHEG-DDFGQLALVNDAPRAATIILREN--
-----N-CHFLRVDKQDFNRI-IKDVEAKTMRLEEHGKVV-----
>Rapgef3_[Cynoglossus_semilaevis]_657766970
AAAHLSASV---RKELAAVLVFESHAKAGTV---LFSQGDKGTS---WYIIWKGSVNVIT
H----GKGL-------------V-T---T-LHEG-EDFGQLALLNDAPRAATIILRED--
-----N-CHFLRVDKQDFIRI-LKDVEANTVRLEEHGKTV-----
>Rapgef3_[Danio_rerio]_688612301
AVAHLSTSV---RKELASVLVFESHAKAGTV---LFSQGDKGTS---WYIIWRGSVNVIT
H----GKGL-------------V-T---T-LHEG-DDFGQLALVNDAPRAATIILRED--
-----N-CHFLRVDKQDFIRI-LKDVEANTVRLEEHGKVV-----
>Rapgef3_[Dasypus_novemcinctus]_488561334
AVAHLSNSV---KRELAAVLLFEPHSKAGTV---LFSQGDKGTS---WYIIWKGSVNVVT
H----GKGL-------------V-T---T-LHEG-DDFGQLALVNDAPRAATIVLRED--
-----N-CHFLRGD------------------------------
>Rapgef3_[Dipodomys_ordii]_852756250
AVAHLSNSV---KRELAAVLLFEPHSKAGTV---LFSQGDKGTS---WYIIWKGSVNVVT
H----GKGL-------------V-T---T-LHEG-DDFGQLALVNDAPRAATIILRED--
-----N-CHFLRVDKQDFNRI-IKDVEAKTMRLEEHGKVV-----
>Rapgef3_[Echinops_telfairi]_850308220
AVAHLSNSV---KRELAAVLLFEPHSKAGTV---LFSQGDKGTS---WYIIWKGSVNVVT
H----GKGL-------------V-T---T-LHEG-DDFGQLALVNDAPRAATIILGED--
-----N-CHFLRVDKHDFNRI-IKDVEAKTMRLEEHGRVV-----
>Rapgef3_[Egretta_garzetta]_697847879
AVAHLSNSV---KRELASVLMFESHQRAGTV---LFSQGDKGTS---WYIIWKGSVNVVT
H----GKGL-------------V-A---T-LHEG--------------------------

>Rapgef3_[Elephantulus_edwardii]_585667536
AVAHLSNSV---KRELAAVLLFEPHSKAGTV---LFSQGDKGTS---WYIIWKGSVNVVT

H----GKGL-------------V-T---T-LHEG-DDFGQLALVNDAPRAATIILRED--
-----N-CHFLRVDKQDFNRI-IKDVEAKTMRLEEHGKVV-----
>Rapgef3_[Eptesicus_fuscus]_641700539
AVAHLSNSV---KRELAAVLLFEPHSKAGTV---LFSQGDKGTS---WYIIWKGSVNVVT
H----GKGL-------------V-T---T-LHEG-DDFGQLALVNDAPRAATIVLRED--
-----N-CHFLRVDKQDFNRI-IKDVEAKTMRLEEHGKVV-----
>Rapgef3_[Equus_asinus]_958727868
ALAHLSNSV---KRELAAVLLFEPHSKAGTV---LFSQGDKGTS---WYIIWKGSVNVVT
H----GKGL-------------V-T---T-LHEG-DDFGQLALVNDAPRAATIILRED--
-----N-CHFLRVDKQDFNRI-IKDVEAKTMRLEEHGKVV-----
>Rapgef3_[Equus_caballus]_545219817
AVAHLSNSV---KRELAAVLLFEPHSKAGTV---LFSQGDKGTS---WYIIWKGSVNVVT
H----GKGL-------------V-T---T-LHEG-DDFGQLALVNDAPRAATIILRED--
-----N-CHFLRVDKQDFNRI-IKDVEAKTMRLEEHGKVV-----
>Rapgef3_[Equus_przewalskii]_664709775
AVAHLSNSV---KRELAAVLLFEPHSKAGTV---LFSQGDKGTS---WYIIWKGSVNVVT
H----GKGL-------------V-T---T-LHEG-DDFGQLALVNDAPRAATIILRED--
-----N-CHFLRVDKQDFNRI-IKDVEAKTMRLEEHGKVV-----
>Rapgef3_[Erinaceus_europaeus]_1016600502
AVAHLSNSV---KRELAAVLLFEPHSKAGTV---LFSQGDKGTS---WYIIWKGSVNVVT
H----GKGL-------------V-T---T-LHEG-DDFGQLALVNDAPRAATIILRED--
-----N-CHFLRVDKQDFNRI-IKDVEAKTMRLEEHGKVV-----
>Rapgef3_[Esox_lucius]_742184366
AVAHLSTSV---RKELAAVLVFESHAKAGTV---LFSQGDKGTS---WYIIWKGSVNVIT
H----GKGL-------------V-T---T-LHEG-EDFGQLALVNDAPRAATIILRED--
-----N-CHFLRVDKQDFIRI-LKDVEANTVRLEEHGKAV-----
>Rapgef3_[Falco_cherrug]_929429028
AVAHLSNSV---KRELASVLMFESHQRAGTV---LFSQGDKGTS---WYIIWKGSVNVVT
H----GKGL-------------V-A---T-LHEG-DDFGQLALVNDAPRAATIILRED--
-----N-CHFLRVDKQDFNHI-LKDVEANTMRLKEHGKVV-----
>Rapgef3_[Felis_catus]_410964201
AVAHLSNSV---KRELAAVLLFEPHSRAGTV---LFSQGDKGTS---WYIIWKGSVNVVT
H----GKGL-------------V-T---T-LHEG-DDFGQLALVNDAPRAATIILRED--
-----N-CHFLRVDKQDFNRI-IKDVEAKTMRLEEHGKVV-----
>Rapgef3_[Ficedula_albicollis]_1020933356
AVAHLSNSV---KRELAAVLMFEGHQRAGTV---LFSQGDKGTS---WYIIWKGSVNVVT
H----GKGL-------------V-A---T-LHEG-DDFGQLALVNDAPRAATILLRED--
-----N-CHFLRVDKHDFNRI-LKDVEANTVRLKEHGKVV-----
>Rapgef3_[Fukomys_damarensis]_731247212
AVAHLSNSV---KRELAAVLVFEPHSKAGTV---LFSQGDKGTS---WYIIWKGSVNVVT
H----GKGL-------------V-T---T-LHEG-DDFGQLALVNDAPRAATIILRED--
-----N-CHFLRVDKQDFNRI-IKDVEAKTMRLEEHGKVV-----
>Rapgef3_[Fundulus_heteroclitus]_831559266
AAAHLSSSV---RKELAAVLVFESHVKSGTV---LFSQGDKGTS---WYIIWKGSVNVIT
H----GKGI-------------V-T---T-LHEG-EDFGQLALLNDAPRAATIILRED--
-----N-CHFLRVDKQDFIRI-LKDVEANTVRLEEHGRTV-----
>Rapgef3_[Galeopterus_variegatus]_667289153
AVAHLSNSV---KRELAAVLLFEPHSKAGTV---LFSQGDKGTS---WYIIWKGSVNVVT
H----GKGL-------------V-T---T-LHEG-DDFGQLALVNDAPRAATIVLRED--
-----N-CHFLRVDKQDFNRI-IKDVEAKTMRLEEHGKVV-----
>Rapgef3_[Geospiza_fortis]_930243948
--------V---KRELAAVLMFEGHQRAGTV---LFSQGDKGTS---WYIIWKGSVNVVT
H----GKGL-------------V-A---T-LHEG-DDFGQLALVNDAPRAATILLRED--
-----N-CHFLRVDKQDFNRI-LKDVEANTVRLKEHGKVV-----

>Rapgef3_[Gorilla_gorilla_gorilla]_426372291
AVAHLSNSV---KRELAAVLLFEPHSKAGTV---LFSQGDKGTS---WYIIWKGSVNVVT
H----GKGL-------------V-T---T-LHEG-DDFGQLALVNDAPRAATIILRED--
-----N-CHFLRVDKQDFNRI-IKDVEAKTMRLEEHGKVV-----
>Rapgef3_[Haliaeetus_leucocephalus]_729737483
AVAHLSNSV---KRELASVLMFESHQRAGTV---LFSQGDKGTS---WYIIWKGSVNVVT
H----GKGL-------------V-A---T-LHEG-DDFGQLALVNDAPRAATIILRED--
-----N-CHFLRVDKQDFNHI-LKDVEANTMRLKEHGKVV-----
>Rapgef3_[Heterocephalus_glaber]_351710665
AVAHLSNSV---KRELAAVLVFEPHSKAGTV---LFSQGDKGTS---WYIIWKGSVNVVT
H----GKGL-------------V-T---T-LHEG-DDFGQLALVNDAPRAATIILRED--
-----N-CHFLRVDKQDFNRI-IKDVEAKTMRLEEHGKVV-----
>Rapgef3_[Homo_sapiens]_1034577577
AVAHLSNSV---KRELAAVLLFEPHSKAGTV---LFSQGDKGTS---WYIIWKGSVNVVT
H----GKGL-------------V-T---T-LHEG-DDFGQLALVNDAPRAATIILRED--
-----N-CHFLRVDKQDFNRI-IKDVEAKTMRLEEHGKVV-----
>Rapgef3_[Ictidomys_tridecemlineatus]_914907256
AVAHLSNSV---KRELAAVLLFEPHSKAGTV---LFSQGDKGTS---WYIIWKGSVNVVT
H----GKGL-------------V-T---T-LHEG-DDFGQLALVNDAPRAATIILRED--
-----N-CHFLRVDKQDFNRI-VKDVEAKTMRLEEHGKVV-----
>Rapgef3_[Jaculus_jaculus]_507569974
AVAHLSNSV---KRELAAVLLFEPHSKAGTV---LFSQGDKGTS---WYIIWKGSVNVVT
H----GKGL-------------V-T---T-LHEG-DDFGQLALVNDAPRAATIILRED--
-----N-CHFLRVDKQDFNRI-IKDVEAKTMRLEEHGKVV-----
>Rapgef3_[Kryptolebias_marmoratus]_1041103289
AAAHLSSSV---RKELAAVLVFESHTKAGTV---LFSQGDKGTS---WYIIWKGSVNVIT
H----GKGL-------------V-T---T-LHEG-EDFGQLALLNDAPRAATIILRED--
-----N-CHFLRVDKQDFIRI-LKDVEANTVRLEEHGKTV-----
>Rapgef3_[Latimeria_chalumnae]_557028580
AVAHLSNSV---KRELASVLEFESHAKAETV---LFSQGDKGTS---WYIIWKGSVNVLT
H----GKGL-------------V-T---T-LSEG-DDFGQLALVNDAPRSATIILRED--
-----N-CHFLRVHKQDFIRI-LKDVEAKTIRLKEHGKVV-----
>Rapgef3_[Lepisosteus_oculatus]_972964891
AVAHLSSSV---RRELAAVLVFESHAKAGTL---LFSQGDKGTS---WYIIWKGSVNVIT
H----GKGL-------------V-T---T-LHEG-DDFGQLALLNDAPRAATIILRED--
-----N-CHFLRVDKQDFIHI-LKDVEANTVRLEEHGKVV-----
>Rapgef3_[Leptonychotes_weddellii]_585154006
AVAHLSNSV---KRELAAVLLFEPHSKAGTV---LFSQGDKGTS---WYIIWKGSVNVVT
H----GKGL-------------V-T---T-LHEG-DDFGQLALVNDAPRAATIILRED--
-----N-CHFLRVDKQDFNRI-IKDVEAKTMRLEEHGKVV-----
>Rapgef3_[Lipotes_vexillifer]_602707263
AVAHLSNSV---KRELAAVLLFEPHSKAGTV---LFSQGDKGTS---WYIIWKGSVNVVT
H----GKGL-------------V-T---T-VHEG-DDFGQLALVNDAPRAATIILRED--
-----N-CHFLRVDKQDFNRI-IKDVEAKTMRLEEHGKVV-----
>Rapgef3_[Loxodonta_africana]_731457673
ARGHLFNSV---KRELAAVLLFEPHCKAGTV---LFSQGDKGTS---WYIIWKGSVNVVT
H----GKCV-------------D-H---W-VW----------------------------
-------------------LV-LQDVEAKTMRLEEHGKVV-----
>Rapgef3_[Macaca_fascicularis]_544469453
AVAHLSNSV---KRELAAVLLFEPHSKAGTV---LFSQGDKGTS---WYIIWKGSVNVVT
H----GKGL-------------V-T---T-LHEG-DDFGQLALVNDAPRAATIILRED--
-----N-CHFLRVDKQDFNRI-IKDVEAKTMRLEEHGKVV-----
>Rapgef3_[Macaca_mulatta]_966971988
AVAHLSNSV---KRELAAVLLFEPHSKAGTV---LFSQGDKGTS---WYIIWKGSVNVVT

H----GKGL------------V-T---T-LHEG-DDFGQLALVNDAPRAATIILRED--
-----N-CHFLRVDKQDFNRI-IKDVEAKTMRLEEHGKVV-----
>Rapgef3_[Macaca_nemestrina]_795561749
AVAHLSNSV---KRELAAVLLFEPHSKAGTV---LFSQGDKGTS---WYIIWKGSVNVVT
H----GKGL------------V-T---T-LHEG-DDFGQLALVNDAPRAATIILRED--
-----N-CHFLRVDKQDFNRI-IKDVEAKTMRLEEHGKVV-----
>Rapgef3_[Mandrillus_leucophaeus]_795173394
AVAHLSNSV---KRELAAVLLFEPHSKAGTV---LFSQGDKGTS---WYIIWKGSVNVVT
H----GKGL------------V-T---T-LHEG-DDFGQLALVNDAPRAATIILRED--
-----N-CHFLRVDKQDFNRI-IKDVEAKTMRLEEHGKVV-----
>Rapgef3_[Marmota_marmota_marmota]_984114363
AVAHLSNSV---KRELAAVLLFEPHSKAGTV---LFSQGDKGTS---WYIIWKGSVNVVT
H----GKGL------------V-T---T-LHEG-DDFGQLALVNDAPRAATIILRED--
-----N-CHFLRVDKQDFNRI-VKDVEAKTMRLEEHGKVV-----
>Rapgef3_[Maylandia_zebra]_499019354
AAAHLSSSV---RKELAAVLVFESHAKAGTV---LFSQGDKGTS---WYIIWKGSVNVIT
H----GKGL------------V-T---T-LHEG-EDFGQLALLNEQPRAATIILRED--
-----N-CHFLRVDKQDFIRI-LKDVEANTVRLEEHGKTV-----
>Rapgef3_[Mesitornis_unicolor]_704529374
AVAHLSNSV---KRELASVLMFEPHQRVGTV---LFSQGDKGTS---WYIIWKGSVNVVT
H----GKGL------------V-A---T-LHEG-DDFGQLALVNDAPRAATIILRED--
-----N-CHFLRVDKQDFNRI-LKDVEANTMRLKEHGKVV-----
>Rapgef3_[Mesocricetus_auratus]_880869538
AVAHLSNSV---KRELAAVLLFEPHSKAGTV---LFSQGDKGTS---WYIIWKGSVNVVT
H----GKGL------------V-T---T-LHEG-DDFGQLALVNDAPRAATIILREN--
-----N-CHFLRVDKQDFNRI-IKDVEAKTMRLEEHGKVV-----
>Rapgef3_[Miniopterus_natalensis]_1016658187
AVAHLSNSV---KRELAAVLLFEPHSKAGTV---LFSQGDKGTS---WYIIWKGSVNVVT
H----GKGL------------V-T---T-LHEG-DDFGQLALVNDAPRAATIILRED--
-----N-CHFLRVDKQDFNRI-IKDVEAKTIRLEEHGKVV-----
>Rapgef3_[Mus_musculus]_755551528
AVAHLSNSV---KRELAAVLLFEPHSKAGTV---LFSQGDKGTS---WYIIWKGSVNVVT
H----GKGL------------V-T---T-LHEG-DDFGQLALVNDAPRAATIILREN--
-----N-CHFLRVDKQDFNRI-IKDVEAKTMRLEEHGKVV-----
>Rapgef3_[Mustela_putorius_furo]_511891954
AVAHLSNSV---KRELAAVLLFEPHSKAGTV---LFSQGDKGTS---WYIIWKGSVNVVT
H----GKGL------------V-T---T-LHEG-DDFGQLALVNDAPRAATIILRED--
-----N-CHFLRVDKQDFNRI-IKDVEAKTMRLEEHGKVV-----
>Rapgef3_[Myotis_brandtii]_521032880
AVAHLSNSV---KRELAAVLLFEPHSKAGTV---LFSQGDKGTS---WYIIWKGSVNVVT
H----GKGL------------V-T---T-LHEG-DDFGQLALVNDAPRAATIILRED--
-----N-CHFLRVDKQDFNRI-IKDVEAKTMRLEEHGKVV-----
>Rapgef3_[Myotis_davidii]_432114532
AVAHLSNSV---KRELAAVLLFEPHSKAGTV---LFSQGDKGTS---WYIIWKGSVNVVT
H----GKGL------------V-T---T-LHEG-DDFGQLALVNDAPRAATIILRED--
-----N-CHFLRVDKQDFNRI-IKDVEAKTMRLEEHGKVV-----
>Rapgef3_[Myotis_lucifugus]_940740350
AVAHLSNSV---KRELAAVLLFEPHSKAGTV---LFSQGDKGTS---WYIIWKGSVNVVT
H----GKGL------------V-T---T-LHEG-DDFGQLALVNDAPRAATIILRED--
-----N-CHFLRVDKQDFNRI-IKDVEAKTMRLEEHGKVV-----
>Rapgef3_[Neolamprologus_brichardi]_583998066
AAAHLSSSV---RKELAAVLVFESHAKAGTV---LFSQGDKGTS---WYIIWKGSVNVIT
H----GKGL------------V-T---T-LHEG-EDFGQLALLNEQPRAATIILRED--
-----N-CHFLRVDKQDFIRI-LKDVEANTVRLEEHGKTV-----

>Rapgef3_[Nomascus_leucogenys]_820994222
AVAHLSNSV---KRELAAVLQFEPHSKAGTV---LFSQGDKGTS---WYIIWKGSVNVVT
H----GKGL-------------V-T---T-LHEG-DDFGQLALVNDAPRAATIILRED--
-----N-CHFLRVDKQDFNRI-IKDVEAKTMRLEEHGKVV-----
>Rapgef3_[Nothobranchius_furzeri]_1007768327
AAAHLSSSV---RKELAAVLVFESHSKAGTV---LFSQGDKGTS---WYIIWKGSVNVIT
H----GKGL-------------V-T---T-LHEG-EDFGQLALLNDAPRAATIILRED--
-----N-CHFLRVDKQDFIRI-LKDVEANTVRLEEHGKTV-----
>Rapgef3_[Ochotona_princeps]_504177543
AVAHLSNSV---KRELAAVLLFEPHSKAGTV---LFSQGDKGTS---WYIIWKGSVNVVT
H----GKGL-------------V-T---T-LHEG-DDFGQLALVNDAPRAATIILRED--
-----S-CHFLRVDKQDFNRI-IKDVEAKTMRLEEHGKVV-----
>Rapgef3_[Octodon_degus]_507675869
AVAHLSNSV---KRELASVLLFEPHSKAGTV---LFSQGDKGTS---WYIIWKGSVNVVT
H----GKGL-------------V-T---T-LHEG-DDFGQLALMNDAPRAATIILRED--
-----N-CHFLRVDKQDFNRI-IKDVEARTMRLEEHGKVV-----
>Rapgef3_[Odobenus_rosmarus_divergens]_823398556
AVAHLSNSV---KRELAAVLLFEPHSKAGTV---LFSQGDKGTS---WYIIWKGSVNVVT
H----GKGL-------------V-T---T-LHEG-DDFGQLALVNDAPRAATIILRED--
-----N-CHFLRVDKQDFNRI-IKDVEAKTMRLEEHGKVV-----
>Rapgef3_[Opisthocomus_hoazin]_700398843
G--HLLPQV---KQELASVLMFESHQRAGTV---LFSQGDKGTS---WYIIWKGSVNVVT
H----GKGL-------------V-A---T-LHEG-DDFGQLALVNDAPRAATIILRED--
-----N-CHFLRVDKQDFNRI-LKDVEANTMRLKEHGKVV-----
>Rapgef3_[Orcinus_orca]_466031722
AVAHLSNSV---KRELAAVLLFEPHSKAGTV---LFSQGDKGTS---WYIIWKGSVNVVT
H----GKGL-------------V-T---T-VHEG-DDFGQLALVNDAPRAATIILRED--
-----N-CHFLRVDKQDFNRI-IKDVEAKTMRLEEHGKVV-----
>Rapgef3_[Oreochromis_niloticus]_542216386
AAAHLSSSV---RKELAAVLVFESHAKAGTV---LFSQGDKGTS---WYIIWKGSVNVIT
H----GKGL-------------V-T---T-LHEG-EDFGQLALLNEQPRAATIILRED--
-----N-CHFLRVDKQDFIRI-LKDVEANTVRLEEHGKTV-----
>Rapgef3_[Orycteropus_afer_afer]_634839964
AVAHLSNSV---KRELAAVLQFEPHSKAGTV---LFSQGDKGTS---WYIIWKGSVNVVT
H----GKGL-------------V-T---T-LHEG-DDFGQLALVNDAPRAATIILRED--
-----N-CHFLRVDKQDFNRI-IKDVEAKTMRLEEHGKVV-----
>Rapgef3_[Oryctolagus_cuniculus]_1040227932
AVAHLSNSV---KRELAAVLLXEPHSKAGTV---LFSQGDKGTS---WYIIWKGSVNVVT
H----GKGL-------------V-T---T-LHEG-DDFGQLALVNDAPRAATIVLRED--
-----S-CHFLRVDKQDFNRI-IKDVEAKTMRLEEHGKVV-----
>Rapgef3_[Oryzias_latipes]_765126149
AAAHLSTSV---RKELAAVLVFESHAKAGTV---LFSQGDKGTS---WYIIWKGSVNVIT
H----GKGI-------------V-T---T-LHEG-EDFGQLALLNDAPRAATIILRED--
-----N-CHFLRVDKQDFIRI-LKDVEANTVRLEEHGKTV-----
>Rapgef3_[Otolemur_garnettii]_831232292
AVAHLSNSV---KRELAAVLLFEPHSKAGTV---LFSQGDKGTS---WYIIWKGSVNVVT
H----GKGL-------------V-T---T-LHEG-DDFGQLALVNDAPRAATIILRED--
-----N-CHFLRVDKQDFNRI-IKDVEAKTIRLEEHGKVV-----
>Rapgef3_[Ovis_aries_musimon]_803304001
AVAHLSNSV---KRELAAVLLFEPHSKAGTV---LFSQGDKGTS---WYIIWKGSVNVVT
H----GKGL-------------V-T---T-LHEG-DDFGQLALVNDAPRAATIILGED--
-----N-CHFLRVDKQDFNRI-IKDVEAKTMRLEEHGKVV-----
>Rapgef3_[Ovis_aries]_803044458
AVAHLSNSV---KRELAAVLLFEPHSKAGTV---LFSQGDKGTS---WYIIWKGSVNVVT

H----GKGL-------------V-T---T-LHEG-DDFGQLALVNDAPRAATIILGED--
-----N-CHFLRVDKQDFNRI-IKDVEAKTMRLEEHGKVV-----
>Rapgef3_[Pan_paniscus]_675783058
AVAHLSNSV---KRELAAVLLFEPHSKAGTV---LFSQGDKGTS---WYIIWKGSVNVVT
H----GKGL-------------V-T---T-LHEG-DDFGQLALVNDAPRAATIILRED--
-----N-CHFLRVDKQDFNRI-IKDVEAKTMRLEEHGKVV-----
>Rapgef3_[Pan_troglodytes]_1034096396
AVAHLSNSV---KRELAAVLLFEPHSKAGTV---LFSQGDKGTS---WYIIWKGSVNVVT
H----GKGL-------------V-T---T-LHEG-DDFGQLALVNDAPRAATIILRED--
-----N-CHFLRVDKQDFNRI-IKDVEAKTMRLEEHGKVV-----
>Rapgef3_[Panthera_tigris_altaica]_591291251
AVAHLSNSV---KRELAAVLLFEPHSRAGTV---LFSQGDKGTS---WYIIWKGSVNVVT
H----GKGL-------------V-T---T-LHEG-DDFGQLALVNDAPRAATIILRED--
-----N-CHFLRVDKQDFNRI-IKDVEAKTMRLEEHGKVV-----
>Rapgef3_[Pantholops_hodgsonii]_556771829
AVAHLSNSV---KRELAAVLLFEPHSKAGTV---LFSQGDKGTS---WYIIWKGSVNVVT
H----GKGL-------------V-T---T-LHEG-DDFGQLALVNDAPRAATIILGED--
-----N-CHFLRVDKQDFNRI-IKDVEAKTMRLEEHGKVV-----
>Rapgef3_[Papio_anubis]_402885758
AVAHLSNSV---KRELAAVLLFEPHSKAGTV---LFSQGDKGTS---WYIIWKGSVNVVT
H----GKGL-------------V-T---T-LHEG-DDFGQLALVNDAPRAATIILRED--
-----N-CHFLRVDKQDFNRI-IKDVEAKTMRLEEHGKVV-----
>Rapgef3_[Parus_major]_998744296

-------------------------------HEG-DDFGQLALVNDAPRAATILLRED--
-----N-CHFLRVDKQDFNRI-LKDVEANTVRLKEHGKVV-----
>Rapgef3_[Peromyscus_maniculatus_bairdii]_589923021
AVAHLSNSV---KRELAAVLLFEPHSKAGTV---LFSQGDKGTS---WYIIWKGSVNVVT
H----GKGL-------------V-T---T-LHEG-DDFGQLALVNDAPRAATIILREN--
-----N-CHFLRVDKQDFNRI-IKDVEAKTMRLEEHGKVV-----
>Rapgef3_[Physeter_catodon]_593715976
AVAHLSNSV---KRELAAVLLFEPHSKAGTV---LFSQGDKGTS---WYIIWKGSVNVVT
H----GKGL-------------V-T---T-LHEG-DDFGQLALVNDAPRAATIILRED--
-----N-CHFLRVDKQDFNRI-IKDVEAKTMRLEEHGKVV-----
>Rapgef3_[Poecilia_formosa]_617405404
AAAHLSSSV---RKELAAVLVFESHVKSGTV---LFSQGDKGTS---WYIIWKGSVNVIT
H----GKGI-------------V-T---T-LHEG-EDFGQLALLNDAPRAATIILRED--
-----N-CHFLRVDKHDFIRI-LKDVEANTVRLEEHGKTV-----
>Rapgef3_[Poecilia_latipinna]_961891079
AAAHLSSSV---RKELAAVLVFESHVKSGTV---LFSQGDKGTS---WYIIWKGSVNVIT
H----GKGI-------------V-T---T-LHEG-EDFGQLALLNDAPRAATIILRED--
-----N-CHFLRVDKHDFIRI-LKDVEANTVRLEEHGKTV-----
>Rapgef3_[Poecilia_reticulata]_658859927
AAAHLSSSV---RKELAAVLVFESHVKSGTV---LFSQGDKGTS---WYIIWKGSVNVIT
H----GKGI-------------V-T---T-LHEG-EDFGQLALLNDAPRAATIILRED--
-----N-CHFLRVDKQDFIRI-LKDVEANTVRLEEHGKTV-----
>Rapgef3_[Pongo_abelii]_197099402
AVAHLSNSV---KRELAAVLLFEPHSKAGTV-----------------------------

>Rapgef3_[Propithecus_coquereli]_826277148
AVAHLSNSV---KRELAAVLLFEPHSKAGTV---LFSQGDKGTS---WYIIWKGSVNVVT
H----GKGL-------------V-T---T-LHEG-DDFGQLALVNDAPRAATIILRED--
-----N-CHFLRVDKQDFNRI-IKDVEAKTMRLEEHGKVV-----

>Rapgef3_[Protobothrops_mucrosquamatus]_1002588985
AVAHLSSSV---RRELAAVLLFESHAKAGTV---LFSQGDKGTS---WYIIWKGSINVVT
H----DKGL-------------V-C---T-LHEG-DDFGQLALVNDAPRAATIILRED--
-----S-CHFLRVDKRDFNRI-LKDVEANTLRLKEHGKVV-----
>Rapgef3_[Pseudopodoces_humilis]_929508811
AVAHLSNSV---KRELAAVLMFEGHQRAGTV---LFSQGDKGTS---WYIIWKGSVNVVT
H----GKGL-------------V-A---T-LHEG-DDFGQLALVNDAPRAATILLRED--
-----N-CHFLRVDKQDFNRI-LKDVEANTVRLKEHGKVV-----
>Rapgef3_[Pteropus_alecto]_989976416
AVAHLSNSV---KRELAAVLLFEPHSKAGTV---LFSQGDKGTS---WYIVWKGSVNVVT
H----GKGL-------------V-T---T-LHEG-DDFGQLALVNDAPRAATIVLRED--
-----N-CHFLRVDKQDFNRI-IKDVEAKTMRLEEHGKVV-----
>Rapgef3_[Pteropus_vampyrus]_759143390
AVAHLSNSV---KRELAAVLLFEPHSKAGTV---LFSQGDKGTS---WYIVWKGSVNVVT
H----GKGL-------------V-T---T-LHEG-DDFGQLALVNDAPRAATIVLRED--
-----N-CHFLRVDKQDFNRI-IKDVEAKTMRLEEHGKVV-----
>Rapgef3_[Pundamilia_nyererei]_548483158
AAAHLSSSV---RKELAAVLVFESHAKAGTV---LFSQGDKGTS---WYIIWKGSVNVIT
H----GKGL-------------V-T---T-LHEG-EDFGQLALLNEQPRAATIILRED--
-----N-CHFLRVDKQDFIRI-LKDVEANTVRLEEHGKTV-----
>Rapgef3_[Python_bivittatus]_602664072
AVAHLSSSV---KRELAAVLLFESHAKAGTV---LFSQGDKGTS---WYIIWKGSVNVVT
H----DKGL-------------V-S---T-LHEG-DDFGQLALVNDAPRAATIILRED--
-----S-CHFLRVDKRDFNRI-LKDVEANTLRLKEHGKVV-----
>Rapgef3_[Rhinopithecus_roxellana]_724952752
AVAHLSNSV---KRELAAVLLFEPHSKAGTV---LFSQGDKGTS---WYIIWKGSVNVVT
H----GKGL-------------V-T---T-LHEG-DDFGQLALVNDAPRAATIILRED--
-----N-CHFLRVDKQDFNRI-IKDVEAKTMRLEEHGKVV-----
>Rapgef3_[Rousettus_aegyptiacus]_1012262855
AVAHLSNSV---KRELAAVLLFEPHSKAGTV---LFSQGDKGTS---WYIIWKGSVNVVT
H----GKGL-------------V-T---T-LHEG-DDFGQLALVNDAPRAATIVLRED--
-----N-CHFLRVDKQDFNRI-IKDVEAKTMRLEEHGKVV-----
>Rapgef3_[Saimiri_boliviensis_boliviensis]_403301658
AVAHLSNSV---KRELAAVLLFEPHSKAGTV---LFSQGDKGTS---WYIIWKGSVNVVT
H----GKGL-------------V-T---T-LHEG-DDFGQLALVNDAPRAATIILRED--
-----N-CHFLRVDKQDFNRI-IKDVEAKTMRLEEHGKVV-----
>Rapgef3_[Salmo_salar]_929230181
AAAHLSTSV---RKELAAVLMFESHAKAGTV---LFSQGDKGTS---WYIIWKGSVNVIT
H----GKGL-------------V-T---T-LHEG-EDFGQLALVNDAPRAATIILRED--
-----N-CHFLRVDKQDFIRI-LKDVEANTMRLEEHGKAV-----
>Rapgef3_[Sarcophilus_harrisii]_821489513
AVAHLSNSV---KKELAAVLLFEPSSKAGTV---LFSQGDKGTS---WYIIWKGSVNVVT
H----GKGL-------------V-T---T-LHEG-DDFGQLALVNDSPRAATIILRED--
-----N-CQFLRVDKQDFNRI-IKDVEANTMRLEEHGKVV-----
>Rapgef3_[Scleropages_formosus]_938064599
AVAHLSSSV---RKELAAVLVFESHAKAGTV---LFSQGDKGTS---WYIIWKGSVNVVT
H----GKGL-------------V-T---T-LHEG-DDFGQLALVNDAPRAATIILRED--
-----N-CHFLRVDKQDFIHI-LKDVEANTVRLEEHGRVV-----
>Rapgef3_[Sinocyclocheilus_anshuiensis]_1025002748
AVAHLSTSV---RKELAAVLVFESHAKAGTV---LFSQGDKGTS---WYIIWRGSVNVIT
H----GKGL-------------V-T---T-LHEG-EDFGQLALVNDAPRSATIILRED--
-----N-CHFLRVDKQDFIRI-LKDVEANTVRLEEHGKVV-----
>Rapgef3_[Sinocyclocheilus_grahami]_1020398505
AVAHLSTSV---RKELAAVLVFESHAKAGTV---LFSQGDKGTS---WYIIWRGSINVIT

H----GKGL-------------V-T---T-LHEG-DDFGQLALVNDAPRSATIILRED--
-----N-CHFLRVDKQDFIRI-LKDVEANTVRLEEHGQVV-----
>Rapgef3_[Sinocyclocheilus_rhinocerous]_1025325482
AVAHLSTSV---RKELAAVLVFESHAKAGTV---LFSQGDKGTS---WYIIWRGSVNVIT
H----GKGL-------------V-T---T-LHEG-EDFGQLALVNDAPRSATIILRED--
-----N-CHFLRVDKQDFIRI-LKDVEANTVRLEEHGKVV-----
>Rapgef3_[Sorex_araneus]_836708039
AVAHLSNSV---KRELAAVLLFEPHSKAGTV---LFSQGDKGTS---WYIIWKGSVNVVT
H----GKGL-------------V-T---T-LHEG-EDFGQLALVNDAPRAATIILRED--
-----N-CHFLRVDKQDFNRI-IKDVEAKTMRLEEHGKVV-----
>Rapgef3_[Struthio_camelus_australis]_678214569
AVAHLSNSV---KRELASVMMFESHQRAGTV---LFSQGDKGTS---WYIIWKGSVNVVT
H----GKGL-------------V-A---T-LHEG-DDFGQLALVNDAPRAATIILRED--
-----N-CHFLRVDKQDFNRI-LKDVEANTMRLKEHGKVV-----
>Rapgef3_[Sus_scrofa]_545893683
--------------ELDLVLLFEPHSKAGTV---LFSQGDKGTS---WYIIWKGSVNVVT
H----GKGL-------------V-T---T-LHEG-DDFGQLXXXXXXXXXXXXXXXXED--
-----N-CHFLRVDKQDFNRI-IKDVEAKTMRLEEHGKVV-----
>Rapgef3_[Takifugu_rubripes]_768914882
AAAHLSTSV---RKELAAVLVFESHAKAGTV---LFSQGDKGTS---WYIIWKGSVNVIT
H----GKGL-------------V-T---T-LHEG-EDFGQLALLNDAPRAATIILRED--
-----N-CHFLRVDKQDFIRI-LKDVEANTVRLEEHGKTV-----
>Rapgef3_[Thamnophis_sirtalis]_927194898
AVAHLSSSV---RRELAAVLLFESHAKAGTV---LFSQGDKGTS---WYIIWKGSVNVVT
H----DKGL-------------V-C---T-LHEG-DDFGQLALVNDAPRAATIILRED--
-----S-CHFLRVDKRDFNRI-LKDVEANTLRLKEHGKVV-----
>Rapgef3_[Tinamus_guttatus]_719772822
AVAHLSNSV---KRELAAVLMFESHPRAGTV---LFSQGDKGTS---WYIIWKGSVNVVT
H----GKGL-------------V-A---T-LHEG-DDFGQLALVNDAPRAATIILRED--
-----N-CHFLRVDKQDFNRI-LKDVEANTVRLKEHGKVV-----
>Rapgef3_[Trichechus_manatus_latirostris]_823404739
AVAHLSNSV---KRELAAVLLFEPHSKAGTV---LFSQGDKGTS---WYIIWKGSVNVVT
H----GKGL-------------V-T---T-LHEG-DDFGQLALVNDAPRAATIILRED--
-----N-CHFLRVDKQDFNRI-IKDVEAKTMRLEEHGKVV-----
>Rapgef3_[Tupaia_chinensis]_562882145
AVAHLSNSV---KRELAAVLLFEPHSKAGTV---LFSQGDKGTS---WYIIWKGSVNVVT
H----GKGL-------------V-T---T-LHEG-DDFGQLALVNDAPRAATIILRED--
-----N-CHFLRVDKQDFNRI-IKDVEAKTMRLEEHGKVV-----
>Rapgef3_[Tursiops_truncatus]_470596594
AVAHLSNSV---KRELAAVLLFEPHSKAGTV---LFSQGDKGTS---WYIIWKGSVNVVT
H----GKGL-------------V-T---T-VHEG-DDFGQLALVNDAPRAATIILRED--
-----N-CHFLRVDKQDFNRI-IKDVEAKTMRLEEHGKVV-----
>Rapgef3_[Ursus_maritimus]_671024230
AVAHLSNSV---KRELAAVLLFEPHSKAGTV---LFSQGDKGTS---WYIIWKGSVNVVT
H----GKGL-------------V-T---T-LHEG-DDFGQLALVNDAPRAATIILRED--
-----N-CHFLRVDKQDFNRI-IKDVEAKTMRLEEHGKVV-----
>Rapgef3_[Vicugna_pacos]_970713237
AVAHLSNSV---KRELAAVLLFEPHSKAGTV---LFSQGDKGTS---WYIIWKGSVNVVT
H----GKGL-------------V-T---T-LHEG-DDFGQLALVNDAPRAATIILRED--
-----N-CHFLRVDKQDFNRI-IKDVEAKTMRLEEHGKVV-----
>Rapgef3_[Xenopus_laevis]_148227834
AVSHLSNSV---KKELASVLIFESHAKAGAV---LFNQGDKGTS---WYIIWKGSVNVIT
H----GKGL-------------V-A---T-LHEG-DDFGQLALVNDAPRAATIVLRED--
-----N-CHFLRVDKHDFNRI-LRDVEANTVRLKEHEKVV-----

>Rapgef3_[Xenopus_tropicalis]_847104409
AVSHLSNSV---KKELASVLIFESHAKAGTV---LFNQGDKGTS---WYIIWKGSVNVIT
H----GKGL-------------V-A---T-LHEG-DDFGQLALVNDAPRAATIVLRED--
-----N-CHFLRVDKHDFNRI-LRDVEANTVRLKEHGKVV-----
>Rapgef4_[Acanthisitta_chloris]_677973646
ALSHLSTTV---KRELAGVLIFESHPKAGTV---LFNQGEEGTS---WYIILKGSVNVVI
Y----GKGV-------------V-C---T-LHEG-DDFGKLALVNDAPRAASIVLRED--
-----N-CHFLRVDKEDFNRI-LRDVEANTVRLKEHDQDVLV---
>Rapgef4_[Acinonyx_jubatus]_961712463
ALSHLSTTV---KRELAGVLIFESHAKGGTV---LFNQGEEGTS---WYIILKGSVNVVI
Y----GKGV-------------V-C---T-LHEG-DDFGKLALVNDAPRAASIVLRED--
-----N-CHFLRVDKEDFNRI-LRDVEANTVRLKEHDQDVLV---
>Rapgef4_[Acropora_digitifera]_1005470793
ALHYLSTTV---KKELAAVVVFESCDNMGTV---LFKEGEEGNS---WYVIIKGIVNVIV
H----GKGV-------------V-C---Q-LNEG-DDFGKLALVNNALRTATIETAKD--
-----S-CHFLKIGKSDFNRI-FQDVETNTVHLKEHGQDVLV---
>Rapgef4_[Acyrthosiphon_pisum]_641671152
ALAHLPNSV---KRELASVVVFEAHPKAGHT---LFRQGDDGKA---WYVIMQGAVSVET
--YSKGI---------------V-E---S-LYEG-EDFGGLALIHNVPRSATITVKED--
-----N-THLLRVDKDSYNKI-VRDIEANTVRLKELGSDVLV---
>Rapgef4_[Ailuropoda_melanoleuca]_752414095
ALSHLSTTV---KRELAGVLIFESHAKGGTV---LFNQGEEGTS---WYIILKGSVNVVI
Y----GKGV-------------V-C---T-LHEG-DDFGKLALVNDAPRAASIVLRED--
-----N-CHFLRVDKEDFNRI-LRDVEANTVRLKEHDQDVLV---
>Rapgef4_[Alligator_mississippiensis]_564252547
ALSHLSTTV---KRELAGVLIFESHPKAGTV---LFNQGEEGTS---WYIILKGSVNVVI
Y----GKGV-------------V-C---T-LHEG-DDFGKLALVNDAPRAASIVLRED--
-----N-CHFLRVDKEDFNRI-LRDVEANTVRLKEHDQDVLV---
>Rapgef4_[Amazona_aestiva]_944296700
ALSHLSTTV---KRELAGVLIFESHPKAGTV---LFNQGEEGTS---WYIILKGSVNVVI
Y----GKGV-------------V-C---T-LHEG-DDFGKLALVNDAPRAASIVLRED--
-----N-CHFLRVDKEDFNRI-LRDVEANTVRLKEHDQDVLV---
>Rapgef4_[Amphimedon_queenslandica]_761901406
AFGHLSNAI---KEELASVVQLENHPVAGKY---LFKEGDAGTS---WYIILKGSVNVLV
-----GKDV-------------M-C---T-LHEG-DEFGKLALLNNAPRTTSVQLREP--
-----N-CVFLRVDRDDFTRI-LLSVEKNTVKIKEHGKEVLL---
>Rapgef4_[Anas_platyrhynchos]_874471726
ALSHLSTTV---KRELAGVLIFESHPKAGTV---LFNQGEEGTS---WYIILKGSVNVVI
Y----GKGV-------------V-C---T-LHEG-DDFGKLALVNDAPRAASIVLRED--
-----N-CHFLRVDKEDFNRI-LRDVEANTVRLKEHDQDVLV---
>Rapgef4_[Anolis_carolinensis]_637340171
ALSHLSTTV---KRELAGVLVFESHPKAGTV---LFNQGEEGTS---WYIILKGSVNVVI
Y----GKGV-------------V-C---T-LHEG-DDFGKLALVNDAPRAASIVLRED--
-----N-CHFLRVDKEDFNRI-LRDVEANTVRLKEHEQDVLV---
>Rapgef4_[Anser_cygnoides_domesticus]_902873580
ALSHLSTTV---KRELAGVLIFESHPKAGTV---LFNQGEEGTS---WYIILKGSVNVVI
Y----GKGV-------------V-C---T-LHEG-DDFGKLALVNDAPRAASIVLRED--
-----N-CHFLRVDKEDFNRI-LRDVEANTVRLKEHDQDVLV---
>Rapgef4_[Aotus_nancymaae]_817294750
ALSHLSTTV---KRELAGVLIFESHAKGGTV---LFNQGEEGTS---WYIILKGSVNVVI
Y----GKGV-------------V-C---T-LHEG-DDFGKLALVNDAPRAASIVLRED--
-----N-CHFLRVDKEDFNRI-LRDVEANTVRLKEHDQDVLV---
>Rapgef4_[Apaloderma_vittatum]_677309553
ALSHLSTTV---KRELAGVLIFESHPKAGTV---LFNQGEEGTS---WYIILKGSVNVVI

Y----GKGV-------------V-C---T-LHEG-DDFGKLALVNDAPRAASIVLRED--
-----N-CHFLRVDKEDFNRI-LRDVEANTVRLKEHDQDVLV---
>Rapgef4_[Aptenodytes_forsteri]_686601170
ALSHLSTTV---KRELAGVLIFESHPKAGTV---LFNQGEEGTS---WYIILKGSVNVVI
Y----GKGV-------------V-C---T-LHEG-DDFGKLALVNDAPRAASIVLRED--
-----N-CHFLRVDKEDFNRI-LRDVEANTVRLKEHDQDVLV---
>Rapgef4_[Apteryx_australis_mantelli]_926533754
ALSHLSTTV---KRELAGVLIFESHPKAGTV---LFNQGEEGTS---WYIILKGSVNVVI
Y----GKGV-------------V-C---T-LHEG-DDFGKLALVNDAPRAASIVLRED--
-----N-CHFLRVDKEDFNRI-LRDVEANTVRLKEHDQDVLV---
>Rapgef4_[Aquila_chrysaetos_canadensis]_768339852
ALSHLSTTV---KRELAGVLIFESHPKAGTV---LFNQGEEGTS---WYIILKGSVNVVI
Y----GKGV-------------V-C---T-LHEG-DDFGKLALVNDAPRAASIVLRED--
-----N-CHFLRVDKEDFNRI-LRDVEANTVRLKEHDQDVLV---
>Rapgef4_[Astyanax_mexicanus]_597779974
ALSHLSTTV---KRELAGVLIFESHAKAGTV---LFNQGEEGTS---WYIILKGSVNVVI
Y----GKGV-------------V-C---T-LHEG-DDFGKLALVNDAPRAASIVLRED--
-----N-CHFLRVDKEDFNRI-LRDVEANTVRLKEHEEDVLV---
>Rapgef4_[Bactrocera_cucurbitae]_751444012
ALSHLSTSI---KRELSSIIVFESHAKAGTI---LFNQGDEGRS---WYILLKGSVDVVI
H----GKGT-------------V-A---T-LKNG-DDFGKLALINDAPRAATIVLKEN--
-----D-CHLLRVDKEHFNRI-LRDVEANTLRLQEHGKDVLV---
>Rapgef4_[Bactrocera_dorsalis]_751799864
ALSHLSTSI---KRELSSIIVFESHAQAGTI---LFNQGDEGRS---WYILLKGSVDVVI
H----GKGT-------------V-A---T-LKNG-DDFGKLALINDAPRAATIVLKEN--
-----N-CHLLRVDKEHFNRI-LRDVEANTLRLQEHGKDVLV---
>Rapgef4_[Balaenoptera_acutorostrata_scammoni]_594670782
ALSHLSTTV---KRELAGVLIFESHAKGGTV---LFNQGEEGTS---WYIILKGSVNVVI
Y----GKGV-------------V-C---T-LHEG-DDFGKLALVNDAPRAASIVLRED--
-----N-CHFLRVDKEDFNRI-LRDVEANTVRLKEHDQDVLV---
>Rapgef4_[Balearica_regulorum_gibbericeps]_723568217
ALSHLSTTV---KRELAGVLIFESHPKAGTV---LFNQGEEGTS---WYIILKGSVNVVI
Y----GKGV-------------V-C---T-LHEG-DDFGKLALVNDAPRAASIVLRED--
-----N-CHFLRVDKEDFNRI-LRDVEANTVRLKEHDQDVLV---
>Rapgef4_[Bison_bison_bison]_742139618
ALSHLSTTV---KRELAAVLIFESHAKGGTV---LFNQGEEGTS---WYIILKGSVNVVI
Y----GKGV-------------V-C---T-LHEG-DDFGKLALVNDAPRAASIVLRED--
-----N-CHFLRVDKEDFNRI-LRDVEANTVRLKEHDQDVLV---
>Rapgef4_[Bos_mutus]_555950849
ALSHLSTTV---KRELAAVLIFESHAKGGTV---LFNQGEEGTS---WYIILKGSVNVVI
Y----GKGV-------------V-C---T-LHEG-DDFGKLALVNDAPRAASIVLRED--
-----N-CHFLRVDKEDFNRI-LRDVEANTVRLKEHDQDVLV---
>Rapgef4_[Bos_taurus]_528938929
ALSHLSTTV---KRELAAVLIFESHAKGGTV---LFNQGEEGTS---WYIILKGSVNVVI
Y----GKGV-------------V-C---T-LHEG-DDFGKLALVNDAPRAASIVLRED--
-----N-CHFLRVDKEDFNRI-LRDVEANTVRLKEHDQDVLV---
>Rapgef4_[Bubalus_bubalis]_594078793
ALSHLSTTV---KRELAAVLIFESHAKGGTV---LFNQGEEGTS---WYIILKGSVNVVI
Y----GKGV-------------V-C---T-LHEG-DDFGKLALVNDAPRAASIVLRED--
-----N-CHFLRVDKEDFNRI-LRDVEANTVRLKEHDQDVLV---
>Rapgef4_[Buceros_rhinoceros_silvestris]_704172716
ALSHLSTTV---KRELAGVLIFESHPKAGTV---LFNQGEEGTS---WYIILKGSVNVVI
Y----GK---

>Rapgef4_[Callorhinchus_milii]_632945696
ALSHLSTTV---KRELAGVLIFESHAKAGTV---LFNQGEEGTS---WYIILKGSVNVVI
Y----GKGV-------------V-C---T-LHEG-DDFGKLALVNDAPRAASIVLHED--
-----N-CHFLRVDKEDFNRI-LRDVEANTVRLKEHEQDVLV---
>Rapgef4_[Calypte_anna]_663246129
ALSHLSTTV---KRELAGVLIFESHPKAGTV---LFNQGEEGTS---WYIILKGSVNVVI
Y----GKGV-------------V-C---T-LHEG-DDFGKLALVNDAPRAASIVLRED--
-----N-CHFLRVDKEDFNRI-LRDVEANTVRLKEHDQDVLV---
>Rapgef4_[Camelus_bactrianus]_743729783
ALSHLSTTV---KRELAGVLIFESHAKGGTV---LFNQGEEGTS---WYIILKGSVNVVI
Y----GKGV-------------V-C---T-LHEG-DDFGKLALVNDAPRAASIVLRED--
-----N-CHFLRVDKEDFNRI-LRDVEANTVRLKEHDQDVLV---
>Rapgef4_[Camelus_dromedarius]_744571829
ALSHLSTTV---KRELAGVLIFESHAKGGTV---LFNQGEEGTS---WYIILKGSVNVVI
Y----GKGV-------------V-C---T-LHEG-DDFGKLALVNDAPRAASIVLRED--
-----N-CHFLRVDKEDFNRI-LRDVEANTVRLKEHDQDVLV---
>Rapgef4_[Camelus_ferus]_560926398
ALSHLSTTV---KRELAGVLIFESHAKGGTV---LFNQGEEGTS---WYIILKGSVNVVI
Y----GKGV-------------V-C---T-LHEG-DDFGKLALVNDAPRAASIVLRED--
-----N-CHFLRVDKEDFNRI-LRDVEANTVRLKEHDQDVLV---
>Rapgef4_[Canis_lupus_familiaris]_545555228
ALSHLSTTV---KRELAGVLIFESHAKGGTV---LFNQGEEGTS---WYIILKGSVNVVI
Y----GKGV-------------V-C---T-LHEG-DDFGKLALVNDAPRAASIVLRED--
-----N-CHFLRVDKEDFNRI-LRDVEANTVRLKEHDQDVLV---
>Rapgef4_[Capra_hircus]_548453219
ALSHLSTTV---KRELAAVLIFESHAKGGTV---LFNQGEEGTS---WYIILKGSVNVVI
Y----GKGV-------------V-C---T-LHEG-DDFGKLALVNDAPRAASIVLRED--
-----N-CHFLRVDKEDFNRI-LRDVEANTVRLKEHDQDVLV---
>Rapgef4_[Caprimulgus_carolinensis]_683458636
ALSHLSTTV---KRELAGVLIFESHPKAGTV---LFNQGEEGTS---WYIILKGSVNVVI
Y----GKGV-------------V-C---T-LHEG-DDFGKLALVNDAPRAASIVLRED--
-----N-CHFLRVDKEDFNRI-LRDVEANTVRLKEHDQDVLV---
>Rapgef4_[Cariama_cristata]_698411398
ALSHLSTTV---KRELAGVLIFESHPKAGTV---LFNQGEEGTS---WYIILKGSVNVVI
Y----GKGV-------------V-C---T-LHEG-DDFGKLALVNDAPRAASIVLRED--
-----N-CHFLRVDKEDFNRI-LRDVEANTVRLKEHDQDVLV---
>Rapgef4_[Carlito_syrichta]_640812553
ALSHLSTTV---KRELAGVLIFESHAKGGTV---LFNQGEEGTS---WYIILKGSVNVVI
Y----GKGV-------------V-C---T-LHEG-DDFGKLALVNDAPRAASIVLRED--
-----N-CHFLRVDKEDFNRI-LRDVEANTVRLKEHDQDVLV---
>Rapgef4_[Cathartes_aura]_677231281
ALSHLSTTV---KRELAGVLIFESHPKAGTV---LFNQGEEGTS---WYIILKGSVNVVI
Y----GKGV-------------V-C---T-LHEG-DDFGKLALVNDAPRAASIVLRED--
-----N-CHFLRVDKEDFNRI-LRDVEANTVRLKEHDQDVLV---
>Rapgef4_[Cavia_porcellus]_884941742
ALSHLSTTV---KRELAGVLIFESHAKGGTV---LFNQGEEGTS---WYIILKGSVNVVI
Y----GKGV-------------V-C---T-LHEG-DDFGKLALVNDAPRAASIVLRED--
-----N-CHFLRVDKEDFNRI-LRDVEANTVRLKEHDQDVLV---
>Rapgef4_[Cebus_capucinus_imitator]_1044377132
ALSHLSTTV---KRELAGVLIFESHAKGGTV---LFNQGEEGTS---WYIILKGSVNVVI
Y----GKGV-------------V-C---T-LHEG-DDFGKLALVNDAPRAASIVLRED--
-----N-CHFLRVDKEDFNRI-LRDVEANTVRLKEHDQDVLV---
>Rapgef4_[Ceratitis_capitata]_499006314
ALSHLSTSI---KRELSSIIVFESHAQAGTI---LFNQGDEGRS---WYILLKGSVDVVI

H----GKGT-------------V-A---T-LKNG-DDFGKLALINDAPRAATIVLKEN--
-----N-CHLLRVDKEHFNRI-LRDVEANTLRLQEHGKDVLV---
>Rapgef4_[Ceratotherium_simum_simum]_478504778
ALSHLSTTV---KRELAGVLIFESHAKGGTV---LFNQGEEGTS---WYIILKGSVNVVI
Y----GKGV-------------V-C---T-LHEG-DDFGKLALVNDAPRAASIVLRED--
-----N-CHFLRVDKEDFNRI-LRDVEANTVRLKEHDQDVLV---
>Rapgef4_[Chaetura_pelagica]_701389410
ALSHLSTTV---KRELAGVLIFESHPKAGTV---LFNQGEEGTS---WYIILKGSVNVVI
Y----GKGV-------------V-C---T-LHEG-DDFGKLALVNDAPRAASIVLRED--
-----N-CHFLRVDKEDFNRI-LRDVEANTVRLKEHDQDVLV---
>Rapgef4_[Charadrius_vociferus]_699632734
ALSHLSTTV---KRELAGVLIFESHPKAGTV---LFNQGEEGTS---WYIILKGSVNVVI
Y----GKGV-------------V-C---T-LHEG-DDFGKLALVNDAPRAASIVLRED--
-----N-CHFLRVDKEDFNRI-LRDVEANTVRLKEHDQDVLV---
>Rapgef4_[Chelonia_mydas]_591371593
ALSHLSTTV---KRELAGVLIFESHPKAGTV---LFNQGEEGTS---WYIILKGSVNVVI
Y----GKTID-HLNITQRSYRHT-C---T-NPKGIDDFGKLALVNDAPRAASIVLRED--
-----N-CHFLRVDKEDFNRI-LRDVEANTVRLKEHDQDVLV---
>Rapgef4_[Chinchilla_lanigera]_918612485
ALSHLSTTV---KRELAGVLIFESHAKGGTV---LFNQGEEGTS---WYIILKGSVNVVI
Y----GKGV-------------V-C---T-LHEG-DDFGKLALVNDAPRAASIVLRED--
-----N-CHFLRVDKEDFNRI-LRDVEANTVRLKEHDQDVLV---
>Rapgef4_[Chlamydotis_macqueenii]_677141915
--------V---KRELAGVLIFESHPKAGTV---LFNQGEEGTS---WYIILKGSVNVVI
Y----GKGV-------------V-C---T-LHEG-DDFGKLALVNDAPRAASIVLRED--
-----N-CHFLRVDKEDFNRI-LRDVEANTVRLKEHDQDVLV---
>Rapgef4_[Chlorocebus_sabaeus]_635058795
ALSHLSTTV---KRELAGVLIFESHAKGGTV---LFNQGEEGTS---WYIILKGSVNVVI
Y----GKGV-------------V-C---T-LHEG-DDFGKLALVNDAPRAASIVLRED--
-----N-CHFLRVDKEDFNRI-LRDVEANTVRLKEHDQDVLV---
>Rapgef4_[Chrysemys_picta_bellii]_530621676
ALSHLSTTV---KRELAGVLIFESHPKAGTV---LFNQGEEGTS---WYIILKGSVNVVI
Y----GKGV-------------V-C---T-LHEG-DDFGKLALVNDAPRAASIVLRED--
-----N-CHFLRVDKEDFNRI-LRDVEANTVRLKEHDQDVLV---
>Rapgef4_[Chrysochloris_asiatica]_586472252
ALSHLSTTV---KRELAGVLIFESHAKGGTV---LFNQGEEGTS---WYIILKGSVNVVI
Y----GKGV-------------V-C---T-LHEG-DDFGKLALVNDAPRAASIVLRED--
-----N-CHFLRVDKEDFNRI-LRDVEANTVRLKEHDQDVLV---
>Rapgef4_[Cimex_lectularius]_939265223
-LSHLSNSM---KRELASVIMFEAHPRSGSI---VFEQGEEGRS---WYLVLNGSVDVVI
H----GKGV-------------V-A---T-LHQG-EDFGKLALINDVPRAASIIVRED--
-----G-THLLRVDKEDFNRI-LRDVEANTVRLKEHGKDVLV---
>Rapgef4_[Ciona_intestinalis]_699251985
ALTQLSDQV---KQQLAAVLQFEFYHKSGET---IFHQGDEGRS---WYIISKGSVDVVI
Q----GKGV-------------V-C---T-LHEG-DDFGSLAIVNDAPRAATIVTTKP--
-----N-CQFLRVDKYDFNKI-HEEVESKTVRLKEHGKDV-----
>Rapgef4_[Clupea_harengus]_831318321
ALSHLSTTV---KRELAGVLIFESHAKAGTV---LFNQGEEGTS---WYIILKGSVNVVI
Y----GKGV-------------V-C---T-LHEG-DDFGKLALVNDAPRAASIVLRED--
-----N-CHFLRVDKEDFNRI-LRDVEANTVRLKEHDQDVLV---
>Rapgef4_[Colius_striatus]_706115654
ALSHLSTTV---KRELAGVLIFESHPKAGTV---LFNQGEEGTS---WYIILKGSVNVVI
Y----GKGV-------------V-C---T-LHEG-DDFGKLALVNDAPRAASIVLRED--
-----N-CHFLRVDKEDFNRI-LRDVEANTVRLKEHDQDVLV---

>Rapgef4_[Colobus_angolensis_palliatus]_795183836
ALSHLSTTV---KRELAGVLIFESHAKGGTV---LFNQGEEGTS---WYIILKGSVNVVI
Y----GKGV-------------V-C---T-LHEG-DDFGKLALVNDAPRAASIVLRED--
-----N-CHFLRVDKEDFNRI-LRDVEANTVRLKEHDQDVLV---
>Rapgef4_[Columba_livia]_449270176
ALSHLSTTV---KRELAGVLIFESHPKAGTV---LFNQGEEGTS---WYIILKGSVNVVI
Y----GKGV-------------V-C---T-LHEG-DDFGKLALVNDAPRAASIVLRED--
-----N-CHFLRVDKEDFNRI-LRDVEANTVRLKEHDQDVLV---
>Rapgef4_[Condylura_cristata]_507926658
ALSHLSTTV---KRELAGVLIFESHAKGGTV---LFNQGEEGTS---WYIILKGSVNVVI
Y----GKGV-------------V-C---T-LHEG-DDFGKLALVNDAPRAASIVLRED--
-----N-CHFLRVDKEDFNRI-LRDVEANTVRLKEHDQDVLV---
>Rapgef4_[Corvus_cornix_cornix]_727051291
ALSHLSTTV---KRELAGVLIFESHPKAGTV---LFNQGEEGTS---WYIILKGSVNVVI
Y----GKGV-------------V-C---T-LHEG-DDFGKLALVNDAPRAASIVLRED--
-----N-CHFLRVDKEDFNRI-LRDVEANTVRLKEHDQDVLV---
>Rapgef4_[Coturnix_japonica]_1003923803
ALSHLSTTV---KRELAGVLIFESHPKAGTV---LFNQGEEGTS---WYIILKGSVNVVI
Y----GKGV-------------V-C---T-LHEG-DDFGKLALVNDAPRAASIVLRED--
-----N-CHFLRVDKEDFNRI-LRDVEANTVRLKEHDQDVLV---
>Rapgef4_[Crassostrea_gigas]_405960606
ALSHLSTSV---KRELASVLVFESHAKAGTV---LFNQGDEGKS---WYIILKGSVNVVI
Y----GKGA-------------V-C---T-LHEG-DDFGKLALLNDAPRAATIVLRED--
-----N-CHFLRVDKEDFNRI-LRDVEANTVRLKEHGQDVLV---
>Rapgef4_[Cricetulus_griseus]_1032830925
ALSHLSTTV---KRELAGVLIFESHAKGGTV---LFNQGEEGTS---WYIILKGSVNVVI
Y----GKGV-------------V-C---T-LHEG-DDFGKLALVNDAPRAASIVLRED--
-----N-CHFLRVDKEDFNRI-LRDVEANTVRLKEHDQDVLV---
>Rapgef4_[Cynoglossus_semilaevis]_657798823
ALSHLTTTV---KQELAGVLMFESRAKTGTV---LFNQGEEGTS---WYIILKGSVNVVI
H----GKGV-------------V-C---T-LHEG-DDFGKLALVNDAPRAASIVLRED--
-----N-CHFLRVDKEDFNRI-LRDVEANTVRLKEHGQDVLV---
>Rapgef4_[Cyprinodon_variegatus]_974068430
ALSHLSTTV---KRELAGVLIFESHERAGTV---LFHQGEEGTS---WYIILKGSVNVVI
Y----GKGV-------------V-C---T-LHEG-DDFGKLALVNDAPRAASIVLRED--
-----N-CHFLRVDKEDFNRI-LRDVEANTVRLKEHGQDVLV---
>Rapgef4_[Danio_rerio]_1040698669
ALSHLSTTV---KRELAGVLIFESHAKAGTV---LFNQGEEGTS---WYIILKGSVNVVI
Y----GKGV-------------V-C---T-LHEG-DDFGKLALVNDAPRAASIVLRED--
-----F-CHFLRVDKEDFNRI-LRDVEANTVRLKEHEEDVLV---
>Rapgef4_[Dasypus_novemcinctus]_488582886
ALSHLSTTV---KRELAGVLIFESHAKGGTV---LFNQGEEGTS---WYIILKGSVNVVI
Y----GKGV-------------V-C---T-LHEG-DDFGKLALVNDAPRAASIVLRED--
-----N-CHFLRVDKEDFNRI-LRDVEANTVRLKEHDQDVLV---
>Rapgef4_[Diaphorina_citri]_1041549174
----------------------------------MFHQGDEGKS---WYIIIQGSVDVVI
Y----GKGC-------------V-T---S-LYAG-EDFGKLALVNNAPRAATIVTRED--
-----N-CHFLRVDKDDFIRI-MRDVEANTVRLKEHGKDVLV---
>Rapgef4_[Dipodomys_ordii]_852730824
ALSHLSTTV---KRELAGVLIFESHAKGGTV---LFNQGEEGTS---WYIILKGSVNVVI
Y----GKGV-------------V-C---T-LHEG-DDFGKLALVNDAPRAASIVLRED--
-----N-CHFLRVDKEDFNRI-LRDVEANTVRLKEHDQDVLV---
>Rapgef4_[Diuraphis_noxia]_985425009
-----------------------------------FRQGDEGKA---WYVIMQGAVNVET

--YSKGV---------------V-E---S-LYEG-EDFGGLALIHNVPRSATITVKED--
-----N-THLLRVDKDSYNKI-VRDIETNTVRLKELGSDVLV---
>Rapgef4_[Drosophila_bipectinata]_1037099499
ALSHLSTSI---KRELSSIFVFEAHAQAGTI---LFNQGDEGRS---WYILLKGSVDVVI
H----GKGT-------------V-A---T-LKTG-DDFGKLALINDAPRAATIVLKEN--
-----N-CHLLRVDKEHFNRI-LRDVEANTLRLQEHGKDVLV---
>Rapgef4_[Drosophila_ficusphila]_1036935383
ALSHLSTSI---KRELSAIFVFEAHAQAGTI---LFNQGDEGRS---WYILLKGSVDVVI
H----GKGT-------------V-A---T-LKTG-DDFGKLALINDAPRAATIVLKEN--
-----N-CHLLRVDKEHFNRI-LRDVEANTLRLQEHGKDVLV---
>Rapgef4_[Drosophila_kikkawai]_1036730969
ALSHLSTSI---KRELSSIFVFEAHAQAGTI---LFNQGDEGRS---WYILLKGSVDVVI
H----GKGT-------------V-A---T-LKTG-DDFGKLALINDAPRAATIVLKEN--
-----N-CHLLRVDKEHFNRI-LRDVEANTLRLQEHGKDVLV---
>Rapgef4_[Drosophila_miranda]_1037006478
ALSHLSTSI---KRELSSIFVFEAHAKAGTI---LFNQGDEGRS---WYILLKGSVDVVI
H----GKGT-------------V-A---T-LKTG-DDFGKLALINDAPRAATIVLKEN--
-----N-CHLLRVDKEHFNRI-LRDVEANTLRLQEHGKDVLV---
>Rapgef4_[Drosophila_rhopaloa]_1036871102
ALSHLSTSI---KRELSSIFVFEAHAQAGTI---LFNQGDEGRS---WYILLKGSVDVVI
H----GKGT-------------V-A---T-LKTG-DDFGKLALINDAPRAATIVLKEN--
-----N-CHLLRVDKEHFNRI-LRDVEANTLRLQEHGKDVLV---
>Rapgef4_[Drosophila_suzukii]_1036073993
ALSHLSTSI---KRELSSIFVFEAHAQAGTI---LFNQGDEGRS---WYILLKGSVDVVI
H----GKGT-------------V-A---T-LKTG-DDFGKLALINDAPRAATIVLKEN--
-----N-CHLLRVDKEHFNRI-LRDVEANTLRLQEHGKDVLV---
>Rapgef4_[Drosophila_takahashii]_1036922041
ALSHLSTSI---KRELSSIFVFEAHAQAGTI---LFNQGDEGRS---WYILLKGSVDVVI
H----GKGT-------------V-A---T-LKTG-DDFGKLALINDAPRAATIVLKEN--
-----N-CHLLRVDKEHFNRI-LRDVEANTLRLQEHGKDVLV---
>Rapgef4_[Echinops_telfairi]_850287185
ALSHLSTTV---KRELAGVLIFESHAKGGTV---LFNQGEEGTS---WYIILKGSVNVVI
Y----GKGV-------------V-C---T-LHEG-DDFGKLALVNDAPRAASIVLRED--
-----N-CHFLRVDKEDFNRI-LRDVEANTVRLKEHDQDVLV---
>Rapgef4_[Egretta_garzetta]_697837447
ALSHLSTTV---KRELAGVLIFESHPKAGTV---LFNQGEEGTS---WYIILKGSVNVVI
Y----GKGV-------------V-C---T-LHEG-DDFGKLALVNDAPRAASIVLRED--
-----N-CHFLRVDKEDFNRI-LRDVEANTVRLKEHDQDVLV---
>Rapgef4_[Elephantulus_edwardii]_585637319
ALSHLSTTV---KRELAGVLIFESHAKGGTV---LFNQGEEGTS---WYIILKGSVNVVI
Y----GKGV-------------V-C---T-LHEG-DDFGKLALVNDAPRAASIVLRED--
-----N-CHFLRVDKEDFNRI-LRDVEANTVRLKEHDQDVLV---
>Rapgef4_[Eptesicus_fuscus]_641696739
ALSHLSTTV---KRELAGVLIFESHAKGGTV---LFNQGEEGTS---WYIILKGSVNVVI
Y----GKGV-------------V-C---T-LHEG-DDFGKLALVNDAPRAASIVLRED--
-----N-CHFLRVDKEDFNRI-LRDVEANTVRLKEHDQDVLV---
>Rapgef4_[Equus_asinus]_958716129
ALSHLSTTV---KRELAGVLIFESHAKGGTV---LFNQGEEGTS---WYIILKGSVNVVI
Y----GKGV-------------V-C---T-LHEG-DDFGKLALVNDAPRAASIVLRED--
-----N-CHFLRVDKEDFNRI-LRDVEANTVRLKEHDQDVLV---
>Rapgef4_[Equus_caballus]_953879137
ALSHLSTTV---KRELAGVLIFESHAKGGTV---LFNQGEEGTS---WYIILKGSVNVVI
Y----GKGV-------------V-C---T-LHEG-DDFGKLALVNDAPRAASIVLRED--
-----N-CHFLRVDKEDFNRI-LRDVEANTVRLKEHDQDVLV---

>Rapgef4_[Equus_przewalskii]_664748190
ALSHLSTTV---KRELAGVLIFESHAKGGTV---LFNQGEEGTS---WYIILKGSVNVVI
Y----GKGV-------------V-C---T-LHEG-DDFGKLALVNDAPRAASIVLRED--
-----N-CHFLRVDKEDFNRI-LRDVEANTVRLKEHDQDVLV---
>Rapgef4_[Erinaceus_europaeus]_1016663854
ALSHLSTTV---KRELAGVLIFESHAKGGTV---LFNQGEEGTS---WYIILKGSVNVVI
Y----GKGV-------------V-C---T-LHEG-DDFGKLALVNDSPRAASIVLRED--
-----N-CHFLRVDKEDFNRI-LRDVEANTVRLKEHDQDVLV---
>Rapgef4_[Esox_lucius]_742138478
ALSHLSTSV---KRELAGVLIFESHAKAGTV---LFNQGEEGTS---WYIILKGSVNVVI
Y----GKGV-------------V-C---T-LHEG-DDFGKLALVNDAPRSASIVLRED--
-----N-CHFLRVDKEDFNRI-LRDVEANTVRLKEHDQDVLV---
>Rapgef4_[Eurypyga_helias]_704253784
---------------------YYENLEKGIT---LFRQGDIGTN---WYAVLTGSLDVKV
--SDTSNH----QDA-----VTI-C---T-LGIG-TAFGE-SILDNTPRHATIVTRE---
-----Y-SELLRIEQKDFKTL-----------------------
>Rapgef4_[Falco_peregrinus]_909796782
ALSHLSTTV---KRELAGVLIFESHPKAGTV---LFNQGEEGTS---WYIILKGSVNVVI
Y----GKGV-------------V-C---T-LHEG-DDFGKLALVNDAPRAASIVLRED--
-----N-CHFLRVDKEDFNRI-LRDVEANTVRLKEHDQDVLV---
>Rapgef4_[Felis_catus]_586997380
ALSHLSTTV---KRELAGVLIFESHAKGGTV---LFNQGEEGTS---WYIILKGSVNVVI
Y----GKGV-------------V-C---T-LHEG-DDFGKLALVNDAPRAASIVLRED--
-----N-CHFLRVDKEDFNRI-LRDVEANTVRLKEHDQDVLV---
>Rapgef4_[Ficedula_albicollis]_525003360
ALSHLSTTV---KRELAGVLIFESHPKAGTV---LFNQGEEGTS---WYIILKGSVNVVI
Y----GKGV-------------V-C---T-LHEG-DDFGKLALVNDAPRAASIVLRED--
-----N-CHFLRVDKEDFNRI-LRDVEANTVRLKEHDQDVLV---
>Rapgef4_[Fukomys_damarensis]_731272280
ALSHLSTTV---KRELAGVLIFESHAKGGTV---LFNQGEEGTS---WYIILKGSVNVVI
Y----GKGV-------------V-C---T-LHEG-DDFGKLALVNDAPRAASIVLRED--
-----N-CHFLRVDKEDFNRI-LRDVEANTVRLKEHDQDVLV---
>Rapgef4_[Fulmarus_glacialis]_679000713
ALSHLSTTV---KRELAGVLIFESHPKAGTV---LFNQGEEGTS---WYIILKGSVNVVI
Y----GKGV-------------V-C---T-LHEG-DDFGKLALVNDAPRAASIVLRED--
-----N-CHFLRVDKEDFNRI-LRDVEANTVRLKEHDQDVLV---
>Rapgef4_[Fundulus_heteroclitus]_831511149
ALSHLSTTV---KRQLAGVLIFESHERAGTV---LFHQGEEGTS---WYIILKGSVNVVI
Y----GKGV-------------V-C---T-LHEG-DDFGKLALVNDAPRAASIVLRED--
-----N-CHFLRVDKEDFNSI-LRDVEANTVRLKEHGQDVLV---
>Rapgef4_[Galeopterus_variegatus]_667257960
ALSHLSTTV---KRELAGVLIFESHAKGGTV---LFNQGEEGTS---WYIILKGSVNVVI
Y----GKGV-------------V-C---T-LHEG-DDFGKLALVNDAPRAASIVLRED--
-----N-CHFLRVDKEDFNRI-LRDVEANTVRLKEHDQDVLV---
>Rapgef4_[Gallus_gallus]_971405305
ALSHLSTTV---KRELAGVLIFESHPKAGTV---LFNQGEEGTS---WYIILKGSVNVVI
Y----GKGV-------------V-C---T-LHEG-DDFGKLALVNDAPRAASIVLRED--
-----N-CHFLRVDKEDFNRI-LRDVEANTVRLKEHDQDVLV---
>Rapgef4_[Gavia_stellata]_678191532
ALSHLSTTV---KRELAGVLIFESHPKAGTV---LFNQGEEGTS---WYIILKGSVNVVI
Y----GKGV-------------V-C---T-LHEG-DDFGKLALVNDAPRAASIVLRED--
-----N-CHFLRVDKEDFNRI-LRDVEANTVRLKEHDQDVLV---
>Rapgef4_[Gorilla_gorilla_gorilla]_426337733
ALSHLSTTV---KRELAGVLIFESHAKGGTV---LFNQGEEGTS---WYIILKGSVNVVI

Y----GKGV-------------V-C---T-LHEG-DDFGKLALVNDAPRAASIVLRED--
-----N-CHFLRVDKEDFNRI-LRDVEANTVRLKEHDQDVLV---
>Rapgef4_[Haliaeetus_albicilla]_700359609
ALSHLSTTV---KRELAGVLIFESHPKAGTV---LFNQGEEGTS---WYIILKGSVNVVI
Y----GKGV-------------V-C---T-LHEG-DDFGKLALVNDAPRAASIVLRED--
-----N-CHFLRVDKEDFNRI-LRDVEANTVRLKEHDQDVLV---
>Rapgef4_[Haliaeetus_leucocephalus]_729721422
ALSHLSTTV---KRELAGVLIFESHPKAGTV---LFNQGEEGTS---WYIILKGSVNVVI
Y----GKGV-------------V-C---T-LHEG-DDFGKLALVNDAPRAASIVLRED--
-----N-CHFLRVDKEDFNRI-LRDVEANTVRLKEHDQDVLV---
>Rapgef4_[Halyomorpha_halys]_939661167
-LSHLSNSM---KRELASVILFEAHPRPGTV---VFEQGEEGRS---WYLVLCGSVDVVI
H----GKGV-------------V-A---T-LHSG-EDFGKLALINEAPRAASIIVREE--
-----N-THLLRVDKDDFNRI-LRDVEANTVRLKEHGKDVLI---
>Rapgef4_[Heterocephalus_glaber]_351695053
ALSHLSTTV---KRELAGVLIFESHAKGGTV---LFNQGEEGTS---WYIILKGSVNVVI
Y----GKGV-------------V-C---T-LHEG-DDFGKLALVNDAPRAASIVLRED--
-----N-CHFLRVDKEDFNRI-LRDVEANTVRLKEHDQDVLV---
>Rapgef4_[Homo_sapiens]_544583481
ALSHLSTTV---KRELAGVLIFESHAKGGTV---LFNQGEEGTS---WYIILKGSVNVVI
Y----GKGV-------------V-C---T-LHEG-DDFGKLALVNDAPRAASIVLRED--
-----N-CHFLRVDKEDFNRI-LRDVEANTVRLKEHDQDVLV---
>Rapgef4_[Ictalurus_punctatus]_1042363418
ALSHLSTTV---KRELAGVLIFESHAKAGTV---LFNQGEEGTS---WYIILKGSVNVVI
Y----GKGV-------------V-C---T-LHEG-DDFGKLALVNDAPRAASIVLRED--
-----N-CHFLRVDKEDFNRI-LRDVEANTVRLKEHEADVLV---
>Rapgef4_[Ictidomys_tridecemlineatus]_532077758
ALSHLSTTV---KRELAGVLIFESHAKGGTV---LFNQGEEGTS---WYIILKGSVNVVI
Y----GKGV-------------V-C---T-LHEG-DDFGKLALVNDAPRAASIVLRED--
-----N-CHFLRVDKEDFNRI-LRDVEANTVRLKEHDQDVLV---
>Rapgef4_[Kryptolebias_marmoratus]_1041109516
ALSHLSTTV---KRELAGVLIFESHEKAGTV---LFNQGEEGTS---WYIILKGSVNVVI
Y----GKGV-------------V-C---T-LHEG-DDFGKLALVNDAPRAASIVLRED--
-----N-CHFLRVDKEDFNRI-LRDVEANTVRLKEHGQDVLV---
>Rapgef4_[Larimichthys_crocea]_808857288

-------GV-------------V-C---T-LHEG-DDFGKLALVNDAPRAASIVLRDD--
-----N-CHFLRVDKEDFNRI-LRDVEANTVRLKEHGQDVLV---
>Rapgef4_[Lepisosteus_oculatus]_973161163
ALSHLSTTV---KRELAGVLIFESHAKAGTV---LFNQGEEGTS---WYIILKGSVNVVI
Y----GKGV-------------V-C---T-LHEG-DDFGKLALVNDAPRAASIVLRED--
-----N-CHFLRVDKEDFNRI-LRDVEANTVRLKEHEQDVLV---
>Rapgef4_[Leptonychotes_weddellii]_585180428
ALSHLSTTV---KRELAGVLIFESHAKGGTV---LFNQGEEGTS---WYIILKGSVNVVI
Y----GKGV-------------V-C---T-LHEG-DDFGKLALVNDAPRAASIVLRED--
-----N-CHFLRVDKEDFNRI-LRDVEANTVRLKEHDQDVLV---
>Rapgef4_[Leptosomus_discolor]_700415865
ALSHLSTTV---KRELAGVLIFESHPKAGTV---LFNQGEEGTS---WYIILKGSVNVVI
Y----GKGV-------------V-C---T-LHEG-DDFGKLALVNDAPRAASIVLRED--
-----N-CHFLRVDKEDFNRI-LRDVEANTVRLKEHDQDVLV---
>Rapgef4_[Limulus_polyphemus]_926607017
ALSHLSNSI---KRELAGVLLFETHLRAGTV---LFNQGDEGTS---WYIILRGSVDVVI
Y----GKGV-------------V-C---T-LHEG-DDFGKLALVNDAPRAATIVTRED--
-----N-CHFLRVDKNDFNRI-LRDAEANTVRLKEHGQDVL----

>Rapgef4_[Lingula_anatina]_919011590
ALSHLSTMV---KRELASVLMFEAHERAGTV---LFNQGDEGTS---WYIILKGSVNVVI
Y----GKGV-------------V-C---T-LHEG-DDFGKLALVNDAPRAATIVLRED--
-----D-CHFLRVDKEDFNRI-LRDVEANTVRLKEHGQDVLV---
>Rapgef4_[Lipotes_vexillifer]_602693651
ALSHLSTTV---KRELAGVLVFESHAKGGTV---LFNQGEEGTS---WYIILKGSVNVVI
Y----GKGV-------------V-C---T-LHEG-DDFGKLALVNDAPRAASIVLRED--
-----N-CHFLRVDKEDFNRI-LRDVEANTVRLKEHDQDVLV---
>Rapgef4_[Loxodonta_africana]_731458745
ALSHLSTTV---KRELAGVLIFESHAKGGTV---LFNQGEEGTS---WYIILKGSVNVVI
Y----GKGV-------------V-C---T-LHEG-DDFGKLALVNDAPRAASIVLRED--
-----N-CHFLRVDKEDFNRI-LRDVEANTVRLKEHDQDVLV---
>Rapgef4_[Macaca_fascicularis]_544475839
ALSHLSTTV---KRELAGVLIFESHAKGGTV---LFNQGEEGTS---WYIILKGSVNVVI
Y----GKGV-------------V-C---T-LHEG-DDFGKLALVNDAPRAASIVLRED--
-----N-CHFLRVDKEDFNRI-LRDVEANTVRLKEHDQDVLV---
>Rapgef4_[Macaca_nemestrina]_795659619
ALSHLSTTV---KRELAGVLIFESHAKGGTV---LFNQGEEGTS---WYIILKGSVNVVI
Y----GKGV-------------V-C---T-LHEG-DDFGKLALVNDAPRAASIVLRED--
-----N-CHFLRVDKEDFNRI-LRDVEANTVRLKEHDQDVLV---
>Rapgef4_[Manacus_vitellinus]_675430502
ALSHLSTTV---KRELAGVLIFESHPKAGTV---LFNQGEEGTS---WYIILKGSVNVVI
Y----GKGV-------------V-C---T-LHEG-DDFGKLALVNDAPRAASIVLRED--
-----N-CHFLRVDKEDFNRI-LRDVEANTVRLKEHDQDVLV---
>Rapgef4_[Mandrillus_leucophaeus]_795195949
ALSHLSTTV---KRELAGVLIFESHAKGGTV---LFNQGEEGTS---WYIILKGSVNVVI
Y----GKGV-------------V-C---T-LHEG-DDFGKLALVNDAPRAASIVLRED--
-----N-CHFLRVDKEDFNRI-LRDVEANTVRLKEHDQDVLV---
>Rapgef4_[Marmota_marmota_marmota]_984087885
ALSHLSTTV---KRELAGVLIFESHAKGGTV---LFNQGEEGTS---WYIILKGSVNVVI
Y----GKGV-------------V-C---T-LHEG-DDFGKLALVNDAPRAASIVLRED--
-----N-CHFLRVDKEDFNRI-LRDVEANTVRLKEHDQDVLV---
>Rapgef4_[Maylandia_zebra]_499012713
ALSHLSTTV---KRELAGVLIFESHAKAGTV---LFNQGEEGTS---WYIILKGSVNVVI
Y----GKGV-------------V-C---T-LHEG-DDFGKLALVNDAPRAASIVLRED--
-----N-CHFLRVDKEDFNRI-LRDVEANTVRLKEHGQDVLV---
>Rapgef4_[Melopsittacus_undulatus]_527269285
ALSHLSTTV---KRELAGVLIFESHPKAGTV---LFNQGEEGTS---WYIILKGSVNVVI
Y----GKGV-------------V-C---T-LHEG-DDFGKLALVNDAPRAASIVLRED--
-----N-CHFLRVDKEDFNRI-LRDVEANTVRLKEHDQDVLV---
>Rapgef4_[Merops_nubicus]_675626701
ALSHLSTTV---KRELAGVLIFESHPKAGTV---LFNQGEEGTS---WYIILKGSVNVVI
Y----GKGV-------------V-C---T-LHEG-DDFGKLALVNDAPRAASIVLRED--
-----N-CHFLRVDKEDFNRI-LRDVEANTVRLKEHDQDVLV---
>Rapgef4_[Mesitornis_unicolor]_677439496
ALSHLSTTV---KRELAGVLIFESHPKAGTV---LFNQGEEGTS---WYIILKGSVNVVI
Y----GKGV-------------V-C---T-LHEG-DDFGKLALVNDAPRAASIVLRED--
-----N-CHFLRVDKEDFNRI-LRDVEANTVRLKEHDQDVLV---
>Rapgef4_[Mesocricetus_auratus]_880859278
ALSHLSTTV---KRELAGVLIFESHAKGGTV---LFNQGEEGTS---WYIILKGSVNVVI
Y----GKGV-------------V-C---T-LHEG-DDFGKLALVNDAPRAASIVLRED--
-----N-CHFLRVDKEDFNRI-LRDVEANTVRLKEHDQDVLV---
>Rapgef4_[Metaseiulus_occidentalis]_391334871
ALSHLSNSV---KRELSGVMVFESHPKRDTA---LFNQGDEGKS---WYIILKGSVNVVI

Y----GKGV-------------V-C---E-LHEG-DDFGKLALVNDAPRAATIITREE--
-----N-CHFLRVDKHDFNRI-LRDVEANTVRLKEHGQDVLV---
>Rapgef4_[Microcebus_murinus]_829851277
ALSHLSTTV---KRELAGVLIFESHAKGGTV---LFNQGEEGTS---WYIILKGSVNVVI
Y----GKGV-------------V-C---T-LHEG-DDFGKLALVNDAPRAASIVLRED--
-----N-CHFLRVDKEDFNRI-LRDVEANTVRLKEHDQDVLV---
>Rapgef4_[Microtus_ochrogaster]_913490047
ALSHLSTTV---KRELAGVLIFESHAKGGTV---LFNQGEEGTS---WYIILKGSVNVVI
Y----GKGV-------------V-C---T-LHEG-DDFGKLALVNDAPRAASIVLRED--
-----N-CHFLRVDKEDFNRI-LRDVEANTVRLKEHDQDVLV---
>Rapgef4_[Miniopterus_natalensis]_1016626283
ALSHLSTTV---KRELAGVLIFESHAKGGTV---LFNQGEEGTS---WYIILKGSVNVVI
Y----GKGV-------------V-C---T-LHEG-DDFGKLALVNDAPRAASIVLRED--
-----N-CHFLRVDKEDFNRI-LRDVEANTVRLKEHDQDVLV---
>Rapgef4_[Monodelphis_domestica]_612019670
ALSHLSTTV---KRELAGVLIFESHAKGGTV---LFNQGEEGTS---WYIILKGSVNVVI
Y----GKGV-------------V-C---T-LHEG-DDFGKLALVNDAPRAASIVLRED--
-----N-CHFLRVDKEDFNRI-LRDVEANTVRLKEHDQDVLV---
>Rapgef4_[Mus_musculus]_323637463
ALSHLSTTV---KRELAGVLIFESHAKGGTV---LFNQGEEGTS---WYIILKGSVNVVI
Y----GKGV-------------V-C---T-LHEG-DDFGKLALVNDAPRAASIVLRED--
-----N-CHFLRVDKEDFNRI-LRDVEANTVRLKEHDQDVLV---
>Rapgef4_[Musca_domestica]_557781733
ALSHLSTSI---KRELSSIIVFEAHAQAGTI---LFSQGDEGRS---WYIILKGSVDVVI
H----GKGT-------------V-A---T-LKSG-DDFGKLALINDAPRAATIVLKEN--
-----N-CHLLRVDKEHFNRI-LRDVEANTLRLQEHGKDVLV---
>Rapgef4_[Myotis_brandtii]_554525588
ALSHLSTTV---KRELAGVLIFESHAKGGTV---LFNQGEEGTS---WYIILKGSVNVVI
Y----GKGV-------------V-C---T-LHEG-DDFGKLALVNDAPRAASIVLRED--
-----N-CHFLRVDKEDFNRI-LRDVEANTVRLKEHDQDVLV---
>Rapgef4_[Myotis_davidii]_584081691
ALSHLSTTV---KRELAGVLIFESHAKGGTV---LFNQGEEGTS---WYIILKGSVNVVI
Y----GKGV-------------V-C---T-LHEG-DDFGKLALVNDAPRAASIVLRED--
-----N-CHFLRVDKEDFNRI-LRDVEANTVRLKEHDQDVLV---
>Rapgef4_[Myotis_lucifugus]_558101247
ALSHLSTTV---KRELAGVLIFESHAKGGTV---LFNQGEEGTS---WYIILKGSVNVVI
Y----GKGV-------------V-C---T-LHEG-DDFGKLALVNDAPRAASIVLRED--
-----N-CHFLRVDKEDFNRI-LRDVEANTVRLKEHDQDVLV---
>Rapgef4_[Neolamprologus_brichardi]_583986590
ALSHLSTTV---KRELAGVLIFESHAKAGTV---LFNQGEEGTS---WYIILKGSVNVVI
Y----GKGV-------------V-C---T-LHEG-DDFGKLALVNDAPRAASIVLRED--
-----N-CHFLRVDKEDFNRI-LRDVEANTVRLKEHGQDVLV---
>Rapgef4_[Nestor_notabilis]_701316307
ALSHLSTTV---KRELAGVLIFESHPKAGTV---LFNQGEEGTS---WYIILKGSVNVVI
Y----GKGV-------------V-C---T-LHEG-DDFGKLALVNDAPRAASIVLRED--
-----N-CHFLRVDKEDFNRI-LRDVEANTVRLKEHDQDVLV---
>Rapgef4_[Nomascus_leucogenys]_821032839
ALSHLSTTV---KRELAGVLIFESHAKGGTV---LFNQGEEGTS---WYIILKGSVNVVI
Y----GKGV-------------V-C---T-LHEG-DDFGKLALVNDAPRAASIVLRED--
-----N-CHFLRVDKEDFNRI-LRDVEANTVRLKEHDQDVLV---
>Rapgef4_[Nothobranchius_furzeri]_1007766827
ALSHLSTTV---KRELAGVLIFESHGKAGTV---LFNQGEEGTS---WYIILKGSVNVFI
Y----GKGV-------------V-C---T-LHEG-DDFGKLALVNDAPRAASIILRED--
-----N-CHFLRVDKEDFNRI-LRDVEANTVRLKEHDQDVLV---

>Rapgef4_[Notothenia_coriiceps]_736302842
ALSHLSTTV---KRELAGVLIFESHAKAGTV---LFSQGEEGTS---WYIILKGSVNVVI
Y----GKGV-------------V-C---T-LHEG-DDFGKLALVNDAPRAASIVLRED--
-----N-CHFLRVDKEDFNRI-LRDVEANTVRLKEHGQDVLV---
>Rapgef4_[Ochotona_princeps]_504128086
ALSHLSTTV---KRELAGVLIFESHAKGGTV---LFNQGEEGTS---WYIILKGSVNVVI
Y----GKGV-------------V-C---T-LHEG-DDFGKLALVNDAPRAASIVLRED--
-----N-CHFLRVDKEDFNRI-LRDVEANTVRLKEHDQDVLV---
>Rapgef4_[Octopus_bimaculoides]_961078509
--------V---KRELASVLVFESHSKKGTV---LFRQGDEGKS---WYIILKGSVNVSI
Y----GKGT-------------V-C---Q-LQEG-DDFGKLALVNDAPRSATIFLCED--
-----N-CHFLRVDKDDFNRI-LRDVEANTVRLNEHGQDVLV---
>Rapgef4_[Odobenus_rosmarus_divergens]_472376524
ALSHLSTTV---KRELAGVLIFESHAKGGTV---LFNQGEEGTS---WYIILKGSVNVVI
Y----GKGV-------------V-C---T-LHEG-DDFGKLALVNDAPRAASIVLRED--
-----N-CHFLRVDKEDFNRI-LRDVEANTVRLKEHDQDVLV---
>Rapgef4_[Opisthocomus_hoazin]_700384682
ALSHLSTTV---KRELAGVLIFESHPKAGTV---LFNQGEEGTS---WYIILKGSVNVVI
Y----GKGV-------------V-C---T-LHEG-DDFGKLALVNDAPRAASIVLRED--
-----N-CHFLRVDKEDFNRI-LRDVEANTVRLKEHDQDVLV---
>Rapgef4_[Orcinus_orca]_465994869
ALSHLSTTV---KRELAGVLIFESHAKGGTV---LFNQGEEGTS---WYIILKGSVNVVI
Y----GKGV-------------V-C---T-LHEG-DDFGKLALVNDAPRAASIVLRED--
-----N-CHFLRVDKEDFNRI-LRDVEANTVRLKEHDQDVLV---
>Rapgef4_[Oreochromis_niloticus]_908516742
ALSHLSTTV---KRELAGVLIFESHAKAGTV---LFNQGEEGTS---WYIILKGSVNVVI
Y----GKGV-------------V-C---T-LHEG-DDFGKLALVNDAPRAASIVLRED--
-----N-CHFLRVDKEDFNRI-LRDVEANTVRLKEHGQDVLV---
>Rapgef4_[Ornithorhynchus_anatinus]_620978658
ALSHLSTTV---KRELAGVLIFESHAKGGTV---LFNQGEEGTS---WYIILKGSVNVVI
Y----GKGV-------------V-C---T-LHEG-DDFGKLALVNDAPRAASIVLRED--
-----H-CHFLRVDKEDFNRI-LRDVEANTVRLKEHDQDVLV---
>Rapgef4_[Orycteropus_afer_afer]_634820945
ALSHLSTTV---KRELAGVLIFESHAKGGTV---LFNQGEEGTS---WYIILKGSVNVVI
Y----GKGV-------------V-C---T-LHEG-DDFGKLALVNDAPRAASIVLRED--
-----N-CHFLRVDKEDFNRI-LRDVEANTVRLKEHDQDVLV---
>Rapgef4_[Oryctolagus_cuniculus]_655827993
ALSHLSTTV---KRELAGVLIFESHAKGGTV---LFNQGEEGTS---WYIILKGSVNVVI
Y----GKGV-------------V-C---T-LHEG-DDFGKLALVNDAPRAASIVLRED--
-----N-CHFLRVDKEDFNRI-LRDVEANTVRLKEHDQDVLV---
>Rapgef4_[Oryzias_latipes]_765156385
ALSHLSTTV---KRELAGVLIFESHAKAGTV---LFNQGDEGTS---WYIILKGSVDVVI
Y----GKGV-------------V-C---T-LHEG-DDFGKLALVNDAPRAASIVLRED--
-----N-CHFLRVDKEDFNRI-LRDVEANTVRLEEHGQDVLV---
>Rapgef4_[Otolemur_garnettii]_831228731
ALSHLSTTV---KRELAGVLIFESHAKGGTV---LFNQGEEGTS---WYIILKGSVNVVI
Y----GKGV-------------V-C---T-LHEG-DDFGKLALVNDAPRAASIVLRED--
-----N-CHFLRVDKEDFNRI-LRDVEANTVRLKEHDQDVLV---
>Rapgef4_[Ovis_aries_musimon]_803230360
ALSHLSTTV---KRELAAVLIFESHAKGGTV---LFNQGEEGTS---WYIILKGSVNVVI
Y----GKGV-------------V-C---T-LHEG-DDFGKLALVNDAPRAASIVLRED--
-----N-CHFLRVDKEDFNRI-LRDVEANTVRLKEHDQDVLV---
>Rapgef4_[Ovis_aries]_803018389
ALSHLSTTV---KRELAAVLIFESHAKGGTV---LFNQGEEGTS---WYIILKGSVNVVI

Y----GKGV-------------V-C---T-LHEG-DDFGKLALVNDAPRAASIVLRED--
-----N-CHFLRVDKEDFNRI-LRDVEANTVRLKEHDQDVLV---
>Rapgef4_[Pan_troglodytes]_1034150403
ALSHLSTTV---KRELAGVLIFESHAKGGTV---LFNQGEEGTS---WYIILKGSVNVVI
Y----GKGV-------------V-C---T-LHEG-DDFGKLALVNDAPRAASIVLRED--
-----N-CHFLRVDKEDFNRI-LRDVEANTVRLKEHDQDVLV---
>Rapgef4_[Panthera_tigris_altaica]_987393874
ALSHLSTTV---KRELAGVLIFESHAKGGTV---LFNQGEEGTS---WYIILKGSVNVVI
Y----GKGV-------------V-C---T-LHEG-DDFGKLALVNDAPRAASIVLRED--
-----N-CHFLRVDKEDFNRI-LRDVEANTVRLKEHDQDVLV---
>Rapgef4_[Pantholops_hodgsonii]_556763448
ALSHLSTTV---KRELAAVLIFESHAKGGTV---LFNQGEEGTS---WYIILKGSVNVVI
Y----GKGV-------------V-C---T-LHEG-DDFGKLALVNDAPRAASIVLRED--
-----N-CHFLRVDKEDFNRI-LRDVEANTVRLKEHDQDVLV---
>Rapgef4_[Papio_anubis]_402888642
ALSHLSTTV---KRELAGVLIFESHAKGGTV---LFNQGEEGTS---WYIILKGSVNVVI
Y----GKGV-------------V-C---T-LHEG-DDFGKLALVNDAPRAASIVLRED--
-----N-CHFLRVDKEDFNRI-LRDVEANTVRLKEHDQDVLV---
>Rapgef4_[Parasteatoda_tepidariorum]_1009531414
ALSHLSNSV---KRELAGVLVFESHPKAGTV---LFNQGDEGKS---WFIILKGAVNVVI
Y----GKGV-------------V-C---S-LHEG-DDFGKLALVNDAPRAATIVTREN--
-----N-CHFLRVDKDDFNNI-IRDVEANTVRLKEHGQDVLL---
>Rapgef4_[Parus_major]_998705928
ALSHLSTTV---KRELAGVLIFESHPKAGTV---LFNQGEEGTS---WYIILKGSVNVVI
Y----GKGV-------------V-C---T-LHEG-DDFGKLALVNDAPRAASIVLRED--
-----N-CHFLRVDKEDFNRI-LRDVEANTVRLKEHDQDVLV---
>Rapgef4_[Pelecanus_crispus]_677474558
ALSHLSTTV---KRELAGVLIFESHPKAGTV---LFNQGEEGTS---WYIILKGSVNVVI
Y----GKGV-------------V-C---T-LHEG-DDFGKLALVNDAPRAASIVLRED--
-----N-CHFLRVDKEDFNRI-LRDVEANTVRLKEHDQDVLV---
>Rapgef4_[Peromyscus_maniculatus_bairdii]_1008739312
ALSHLSTTV---KRELAGVLIFESHAKGGTV---LFNQGEEGTS---WYIILKGSVNVVI
Y----GKGV-------------V-C---T-LHEG-DDFGKLALVNDAPRAASIVLRED--
-----N-CHFLRVDKEDFNRI-LRDVEANTVRLKEHDQDVLV---
>Rapgef4_[Phaethon_lepturus]_723138393
ALSHLSTTV---KRELAGVLIFESHPKAGTV---LFNQGEEGTS---WYIILKGSVNVVI
Y----GKGV-------------V-C---T-LHEG-DDFGKLALVNDAPRAASIVLRED--
-----N-CHFLRVDKEDFNRI-LRDVEANTVRLKEHDQDVLV---
>Rapgef4_[Phalacrocorax_carbo]_695135744
ALSHLSTTV---KRELAGVLIFESHPKAGTV---LFNQGEEGTS---WYIILKGSVNVVI
Y----GKGV-------------V-C---T-LHEG-DDFGKLALVNDAPRAASIVLRED--
-----N-CHFLRVDKEDFNRI-LRDVEANTVRLKEHDQDVLV---
>Rapgef4_[Physeter_catodon]_593753582
ALSHLSTTV---KRELAGVLIFESHAKGGTV---LFNQGEEGTS---WYIILKGSVNVVI
Y----GKGV-------------V-C---T-LHEG-DDFGKLALVNDAPRAASIVLRED--
-----N-CHFLRVDKEDFNRI-LRDVEANTVRLKEHDQDVLV---
>Rapgef4_[Poecilia_formosa]_617381053
ALSHLSTTV---KRELAGVLIFESHERAGTV---LFHQGEEGTS---WYIILKGSVNVVI
Y----GKGV-------------V-C---T-LYEG-DDFGKLALVNDAPRAASIVLRED--
-----D-CHFLRVDKEDFNRI-LRDVEANTVRLKEHGQDVLV---
>Rapgef4_[Poecilia_latipinna]_961931725
ALSHLSTTV---KRELAGVLIFESHERAGTV---LFHQGEEGTS---WYIILKGSVNVVI
Y----GKGV-------------V-C---T-LYEG-DDFGKLALVNDAPRAASIVLRED--
-----D-CHFLRVDKEDFNRI-LRDVEANTVRLKEHGQDVLV---

>Rapgef4_[Poecilia_reticulata]_1039383673
ALSHLSTTV---KRELAGVLIFESHERAGTV---LFHQGEEGTS---WYIILKGSVNVVI
Y----GKGV-------------V-C---T-LHEG-DDFGKLALVNDAPRAASIVLRED--
-----D-CHFLRVDKEDFNRI-LRDVEANTVRLKEHGQDVLV---
>Rapgef4_[Pongo_abelii]_686709088
ALSHLSTTV---KRELAGVLIFESHAKGGTV---LFNQGEEGTS---WYIILKGSVNVVI
Y----GKGV-------------V-C---T-LHEG-DDFGKLALVNDAPRAASIVLRED--
-----N-CHFLRVDKEDFNRI-LRDVEANTVRLKEHDQDVLV---
>Rapgef4_[Propithecus_coquereli]_826312078
ALSHLSTTV---KRELAGVLIFESHAKGGTV---LFNQGEEGTS---WYIILKGSVNVVI
Y----GKGV-------------V-C---T-LHEG-DDFGKLALVNDAPRAASIVLRED--
-----N-CHFLRVDKEDFNRI-LRDVEANTVRLKEHDQDVLV---
>Rapgef4_[Protobothrops_mucrosquamatus]_1002568461
ALSHLSTTV---KRELAGVLVFESHPKAGTV---LFNQGEEGTS---WYIILKGSVNVVI
Y----GKGV-------------V-C---T-LHEG-DDFGKLALVNDAPRAASIVLREE--
-----N-CHFLRVDKEDFNRI-LRDVEANTVRLKEHDQDVLV---
>Rapgef4_[Pseudopodoces_humilis]_929420255
ALSHLSTTV---KRELAGVLIFESHPKAGTV---LFNQGEEGTS---WYIILKGSVNVVI
Y----GKGV-------------V-C---T-LHEG-DDFGKLALVNDAPRAASIVLRED--
-----N-CHFLRVDKEDFNRI-LRDVEANTVRLKEHDQDVLV---
>Rapgef4_[Pterocles_gutturalis]_704489718
ALSHLSTTV---KRELAGVLIFESHPKAGTV---LFNQGEEGTS---WYIILKGSVNVVI
Y----GKGV-------------V-C---T-LHEG-DDFGKLALVNDAPRAASIVLRED--
-----N-CHFLRVDKEDFNRI-LRDVEANTVRLKEHDQDVLV---
>Rapgef4_[Pteropus_alecto]_586532740
ALSHLSTTV---KRELAGVLIFESHAKGGTV---LFNQGEEGTS---WYIILKGSVNVVI
Y----GKGV-------------V-C---T-LHEG-DDFGKLALVNDAPRAASIVLRED--
-----N-CHFLRVDKEDFNRI-LRDVEANTVRLKEHDQDVLV---
>Rapgef4_[Pundamilia_nyererei]_548342431
ALSHLSTTV---KRELAGVLIFESHAKAGTV---LFNQGEEGTS---WYIILKGSVNVVI
Y----GKGV-------------V-C---T-LHEG-DDFGKLALVNDAPRAASIVLRED--
-----N-CHFLRVDKEDFNRI-LRDVEANTVRLKEHGQDVLV---
>Rapgef4_[Pygoscelis_adeliae]_690425254
ALSHLSTTV---KRELAGVLVFESHPKAGTV---LFNQGEEGTS---WYIILKGSVNVVI
Y----GKGV-------------V-C---T-LHEG-DDFGKLALVNDAPRAASIVLRED--
-----N-CHFLRVDKEDFNRI-LRDVEANTVRLKEHDQDVLV---
>Rapgef4_[Python_bivittatus]_1004668302
ALSHLSTTV---KRELAGVLVFESHPKAGTV---LFNQGEEGTS---WYIILKGSVNVVI
Y----GKGV-------------V-C---T-LHEG-DDFGKLALVNDAPRAASIVLREE--
-----N-CHFLRVDKEDFNRI-LRDVEANTVRLKEHNQDVLV---
>Rapgef4_[Rhinopithecus_roxellana]_724894124
ALSHLSTTV---KRELAGVLIFESHAKGGTV---LFNQGEEGTS---WYIILKGSVNVVI
Y----GKGV-------------V-C---T-LHEG-DDFGKLALVNDAPRAASIVLRED--
-----N-CHFLRVDKEDFNRI-LRDVEANTVRLKEHDQDVLV---
>Rapgef4_[Rousettus_aegyptiacus]_1012008359
ALSHLSTTV---KRELAGVLIFESHAKGGTV---LFNQGEEGTS---WYIILKGSVNVVI
Y----GKGV-------------V-C---T-LHEG-DDFGKLALVNDAPRAASIVLRED--
-----N-CHFLRVDKEDFNRI-LRDVEANTVRLKEHDQDVLV---
>Rapgef4_[Saccoglossus_kowalevskii]_585695958
ALSHLSTMV---KRELASVLKFESHQKAGTV---LFNQGDEGKS---WYIILRGSVNVVI
Y----GKGV-------------V-C---T-LHEG-DDFGKLALVNDAPRAASIVLRED--
-----N-CHFLRVDKDDFNRI-LRDVEANTVRLKEHGQDVLV---
>Rapgef4_[Saimiri_boliviensis_boliviensis]_403258754
ALSHLSTTV---KRELAGVLIFESHAKGGTV---LFNQGEEGTS---WYIILKGSVNVVI

Y----GKGV-------------V-C---T-LHEG-DDFGKLALVNDAPRAASIVLRED--
-----N-CHFLRVDKEDFNRI-LRDVEANTVRLKEHDQDVLV---
>Rapgef4_[Salmo_salar]_929285053
ALSHLSTTV---KKELAGVLIFESHAKAGTV---LFNQGEEGTS---WYIILKGSVNVVI
Y----GKGV-------------V-C---T-LHEG-DDFGKLALVNDAPRAASIVLRED--
-----N-CHFLRVDKEDFNRI-LRDVEANTVRLKEHDQDVLV---
>Rapgef4_[Sarcophilus_harrisii]_395519780
ALSHLSTTV---KRELAGVLIFESHAKGGTV---LFNQGEEGTS---WYIILKGSVNVVI
Y----GKGV-------------V-C---T-LHEG-DDFGKLALVNDAPRAASIVLRED--
-----N-CHFLRVDKEDFNRI-LRDVEANTVRLKEHDQDVLV---
>Rapgef4_[Sarcoptes_scabiei]_934156037
AFSHLSNSV---KKELSGVIAFE-SYEQKSTIW----------S-----------VNVVI
A----SKGI-------------V-C---T-LHEG-DDFGKLALVNEAPRAATIITNEP--
-----D-CQFLRVDKVHFDRI-LKDNEASIVRLKECGKEVLI---
>Rapgef4_[Schistosoma_haematobium]_844834006
ALSSFSISV---KQELCKCLYYEVHENALDI---VFYQGDPGIS---WYIIYHGSVWVHI
--NNQGY---------------I-C---L-LNEG-DDFGKLSLITDKPRAASILLAEN--
-----N-CHFLKLNKNDFNRI-LCNVEANTVHLKNNNSDVLI---
>Rapgef4_[Scleropages_formosus]_938054751
ALSHLSTTV---KRELAGVLIFESHAKAGTV---LFNQGEEGTS---WYIILKGSVNVVI
Y----GKGV-------------V-C---T-LHEG-DDFGKLALVNDAPRAASIVLRED--
-----N-CHFLRVDKEDFNRI-LRDVEANTVRLKEHQQDVLV---
>Rapgef4_[Sinocyclocheilus_anshuiensis]_1025001496
ALSHLSTTV---KRELAGVLIFESHAKAGTV---LFNQGEEGTS---WYIILKGSINVVI
Y----GKGV-------------V-C---T-LHEG-DDFGKLALVNDAPRAASIVLRED--
-----N-CHFLRVDKEDFNRI-LRDVEANTVRLKEHEEDVLV---
>Rapgef4_[Sinocyclocheilus_grahami]_1020426833
-------------------------------------EGDDFGKRLQWYIILKGSVNVVI
Y----GKGV-------------V-C---T-LHEG-DDFGKLALVNDAPRAASIVLRED--
-----N-CHLLRVDKEDFNRI-LRDVEANTVRLKEH---------
>Rapgef4_[Sinocyclocheilus_rhinocerous]_1025300063
ALSHLSTTV---KRELAGVLIFESHAKAGTV---LFNQGEEGTS---WYIILKGSINVVI
Y----GKGV-------------V-C---T-LHEG-DDFGKLALVNDAPRAASIVLRED--
-----N-CHFLRVDKEDFNRI-LRDVEANTVRLKEHEEDVLV---
>Rapgef4_[Sorex_araneus]_505771640
ALSHLSTTV---KRELAGVLIFESHAKGGTV---LFNQGEEGTS---WYIILKGSVNVVI
Y----GKGV-------------V-C---T-LHEG-DDFGKLALVNDAPRAASIVLRED--
-----N-CHFLRVDKEDFNRI-LRDVEANTVRLKEHDQDVLV---
>Rapgef4_[Stegodyphus_mimosarum]_675363725
ALSHLSNSV---KRELAGVLVFESHPKAGTV---LFNQGDEGKS---WFIILRGSVNVVI
Y----GKGI-------------V-C---S-LHEG-DDFGKLALVNDAPRAATIITREN--
-----N-CHFLRVDKEDFNNI-IRDVEANTVRLKEHGQDVLL---
>Rapgef4_[Strongylocentrotus_purpuratus]_390363651
ALAHLSGMV---KRQLTSVLVFECHEKAGTV---LFHQGDEGRS---WFIILRGSVNVVK
Y----GKGV-------------V-C---T-LHDG-DDFGKLALVNDAPRAASIVLRED--
-----N-CQFLRVDKYDFNRI-LRDVEANTVRLKEHGQDSLV---
>Rapgef4_[Struthio_camelus_australis]_697502539
ALSHLSTTV---KRELAGVLIFESHPKAGTV---LFNQGEEGTS---WYIILKGSVNVVI
Y----GKGV-------------V-C---T-LHEG-DDFGKLALVNDAPRAASIVLRED--
-----N-CHFLRVDKEDFNRI-LRDVEANTVRLKEHDQDVLV---
>Rapgef4_[Sturnus_vulgaris]_959065034
ALSHLSTTV---KRELAGVLIFESHPKAGTV---LFNQGEEGTS---WYIILKGSVNVVI
Y----GKGV-------------V-C---T-LHEG-DDFGKLALVNDAPRAASIVLRED--
-----N-CHFLRVDKEDFNRI-LRDVEANTVRLKEHDQDVLV---

>Rapgef4_[Sus_scrofa]_927210429
ALSHLSTTV---KRELAGVLIFESHAKGGTV---LFNQGEEGTS---WYIILKGSVNVVI
Y----GKGV-------------V-C---T-LHEG-DDFGKLALVNDAPRAASIVLRED--
-----N-CHFLRVDKEDFNRI-LRDVEANTVRLKEHDQDVLV---
>Rapgef4_[Takifugu_rubripes]_410896946
ALSHLSTTV---KRELAGVLIFESHAKAGTV---LFSQGEEGTS---WYIILKGSVNVVI
Y----GKGV-------------V-C---T-LHEG-DDFGKLALVNDAPRAASIVLRED--
-----N-CHFLRVDKEDFNRI-LRDVEANTVRLKEHGQDVLV---
>Rapgef4_[Tinamus_guttatus]_719773624
ALSHLSTTV---KRELAGVLIFESHPKAGTV---LFNQGEEGTS---WYIILKGSVNVVI
Y----GKGV-------------V-C---T-LHEG-DDFGKLALVNDAPRAASIVLRED--
-----N-CHFLRVDKEDFNRI-LRDVEANTVRLKEHDQDVLV---
>Rapgef4_[Trichechus_manatus_latirostris]_471370093
ALSHLSTTV---KRDVCGIAIFSSFL---CL---VFNQGEEGTS---WYIILKGSVNVVI
Y----GKGV-------------V-C---T-LHEG-DDFGKLALVNDAPRAASIVLRED--
-----N-CHFLRVDKEDFNRI-LRDVEANTVRLKEHDQDVLV---
>Rapgef4_[Trichinella_britovi]_954388359
ALSNLSTMV---KRELATVIMFEQHQNAGTV---LFRQGDQGKS---WYIILKGSVNVVI
H----GKGV-------------V-C---T-LQEG-DDFGKLALVNDAPRAATIILNEN--
-----C-CQFLRVDKHDFDRI-LRDVEANTVRLKEHGHDVLV---
>Rapgef4_[Trichinella_nativa]_954534912
ALSNLSTMV---KRELATVIMFEQHQNAGTV---LFRQGDQGKS---WYIILKGSVNVVI
H----GKGV-------------V-C---T-LQEG-DDFGKLALVNDAPRAATIILNEN--
-----C-CQFLRVDKHDFDRI-LRDVEANTVRLKEHGHDVLV---
>Rapgef4_[Trichinella_papuae]_954590899
ALSNLSTMV---KRELATVIMFEQHQNAGTV---LFRQGDQGKS---WYIILKGSVNVVI
H----GKGV-------------V-C---T-LQEG-DDFGKLALVNDAPRAATIILNEN--
-----C-CQFLRVDKHDFDRI-LRDVEANTVRLKEHGHDVLV---
>Rapgef4_[Trichinella_patagoniensis]_954341164
ALSNLSTMV---KRELATVIMFEQHQNAGTV---LFRQGDQGKS---WYIILKGSVNVVI
H----GKGV-------------V-C---T-LQEG-DDFGKLALVNDAPRAATIILNEN--
-----C-CQFLRVDKHDFDRI-LRDVEANTVRLKEHGHDVLV---
>Rapgef4_[Trichinella_sp._T6]_954280927
ALSNLSTMV---KRELATVIMFEQHQNAGTV---LFRQGDQGKS---WYIILKGSVNVVI
H----GKGV-------------V-C---T-LQEG-DDFGKLALVNDAPRAATIILNEN--
-----C-CQFLRVDKHDFDRI-LRDVEANTVRLKEHGHDVLV---
>Rapgef4_[Trichinella_sp._T8]_954617710
ALSNLSTMV---KRELATVIMFEQHQNAGTV---LFRQGDQGKS---WYIILKGSVNVVI
H----GKGV-------------V-C---T-LQEG-DDFGKLALVNDAPRAATIILNEN--
-----C-CQFLRVDKHDFDRI-LRDVEANTVRLKEHGHDVLV---
>Rapgef4_[Trichinella_spiralis]_954362209
ALSNLSTMV---KRELATVIMFEQHQNAGTV---LFRQGDQGKS---WYIILKGSVNVVI
H----GKGV-------------V-C---T-LQEG-DDFGKLALVNDAPRAATIILNEN--
-----C-CQFLRVDKHDFDRI-LRDVEANTVRLKEHGHDVLV---
>Rapgef4_[Trichinella_zimbabwensis]_954489457
ALSNLSTMV---KRELATVIMFEQHQNAGTV---LFRQGDQGKS---WYIILKGSVNVVI
H----GKGV-------------V-C---T-LQEG-DDFGKLALVNDAPRAATIILNEN--
-----C-CQFLRVDKHDFDRI-LRDVEANTVRLKEHGHDVLV---
>Rapgef4_[Tupaia_chinensis]_562844419
ALSHLSTTV---KRELAGVLVFESHAKGGTV---LFNQGEEGTS---WYIILKGSVNVVI
Y----GKGV-------------V-C---T-LHEG-DDFGKLALVNDAPRAASIVLRED--
-----N-CHFLRVDKEDFNRI-LRDVEANTVRLKEHDQDVLV---
>Rapgef4_[Tursiops_truncatus]_470618433
----------------------------GIT---LFRQGDIGTN---WYAVLAGSLDVKV

```
--SETSSH----QDA-----VTI-C---T-LGIG-TAFGE-SILDNTPRHATIVTRE---
-----S-SELLRIEQKDF--------------------------
>Rapgef4_[Tyto_alba]_678181601
ALSHLSTTV---KRELAGVLIFESHPKAGTV---LFNQGEEGTS---WYIILKGSVNVVI
Y----GKGV-------------V-C---T-LHEG-DDFGKLALVNDAPRAASIVLRED--
-----N-CHFLRVDKEDFNRI-LRDVEANTVRLKEHDQDVLV---
>Rapgef4_[Ursus_maritimus]_670988645
ALSHLSTTV---KRELAGVLIFESHAKGGTV---LFNQGEEGTS---WYIILKGSVNVVI
Y----GKGV-------------V-C---T-LHEG-DDFGKLALVNDAPRAASIVLRED--
-----N-CHFLRVDKEDFNRI-LRDVEANTVRLKEHDQDVLV---
>Rapgef4_[Vicugna_pacos]_560983554
ALSHLSTTV---KRELAGVLIFESHAKGGTV---LFNQGEEGTS---WYIILKGSVNVVI
Y----GKGV-------------V-C---T-LHEG-DDFGKLALVNDAPRAASIVLRED--
-----N-CHFLRVDKEDFNRI-LRDVEANTVRLKEHDQDVLV---
>Rapgef4_[Xenopus_tropicalis]_512866132
ALSHLSTTV---KRELAGFLIFESHPKAGTV---LFNQGEEGTS---WYIILKGSVNVVI
Y----GKGV-------------V-C---T-LHEG-DDFGKLALVNDAPRAASIVLRED--
-----N-CHFLRVDKEDFNRI-LRDVEANTVRLKEHDQDVLV---
>Rapgef4_[Xiphophorus_maculatus]_941779216
ALSHLSTTV---KRELAGVLIFESHERAGTV---LFHQGEEGTS---WYIILKGSVNVVI
Y----GKGV-------------V-C---T-LHEG-DDFGKLALVNDAPRAASIVLRED--
-----D-CHFLRVDKEDFNRI-LRDVEANTVRLKEHGQDVLV---
>Rapgef4_[Zonotrichia_albicollis]_929451867
ALSHLSTTV---KRELAGVLIFESHPKAGTV---LFNQGEEGTS---WYIILKGSVNVVI
Y----GKGV-------------V-C---T-LHEG-DDFGKLALVNDAPRAASIVLRED--
-----N-CHFLRVDKEDFNRI-LRDVEANTVRLKEHDQDVLV---
>Rapgef4_[Zootermopsis_nevadensis]_646703516
ALSHLSNSV---KRELAAVIVFEAHPHAGTV---LFNQGDEGRS---WYIILRGSVDVVI
H----GKGT-------------V-T---T-LQEG-DDFGKLALINDAPRAATIVLREN--
-----N-CHFLRVDKENFNRI-LRDVEANTVRLKEHGQDVLV---
>Rapgef5_[Acinonyx_jubatus]_961711358
-LARLTSAV---QRELA-AVIALKARKSAIE------QDEDNGD---KHVIVAEAEGV--
---PDSQA------G------VMCK-----LQER-DDIGRIELVQKLARE----------
-----N-CQFLQTDKKEQEKSEHLDEEVATVQVKEQERNVLV---
>Rapgef5_[Ailuropoda_melanoleuca]_752425000
-LARLTSAV---QRELA-AVIALKARKSAIE------QDEENGD---KHVIVTEAGGV--
---PDSQA------G------VMCK-----LQER-DDIGRIELVQKLARE----------
-----N-CQFLQTDKKEQEKSEHQDDEVTTVQVKEQEQNVLV---
>Rapgef5_[Balaenoptera_acutorostrata_scammoni]_594672128
-LCRLTSAV---QRELA-AVIALKARKSAIE------QDEENSD---KHVTVTEAEGV--
---ADPQA------G------VICK-----LQER-DDIGRIELVQKLARE----------
-----N-CQFLQTDKKEQEKSEHQDDEVTTVQVKEQDRDVLV---
>Rapgef5_[Bison_bison_bison]_742114507
-LARLTSAV---QRELA-AVIALKARKSALA------QDEENSD---KHVTVTEAEAA--
---ADPQA------G------MMCK-----LQER-DDIGRIELVQKLARE----------
-----S-CQFLQTDKKEQEKSEH-DDEVTMVEVKEQDQNVLV---
>Rapgef5_[Carlito_syrichta]_640775670
-LARLTSAV---QRELA-AVIALKARKSALE------QDEENND---KHVTVTEADGI--
---PDSQA------G------VMCK-----LQER-DDIGRIELVQKLARE----------
-----N-YQFLQSDKKEQEKSEHQDDEVATVQVKEQDRSVLV---
>Rapgef5_[Ceratotherium_simum_simum]_478489217
-LARLTSAV---QRELA-AVIALKARKSAID------QDEENSD---KHVTVTEAESV--
---PDSQA------G------VMCK-----LQER-DDIGRIELVQKLARE----------
-----N-CQFLQTDKKEQEKSEHQDDEVTTVRVKEQERNVLV---
```

```
>Rapgef5_[Chinchilla_lanigera]_533124335
-LARLTSAV---QRELA-AVIALKARKSALE------QDDEDND---KHVPVTEAEGV--
---PDSQA------G------VMCK-----LQER-DDIGRIELVQKLARE----------
-----N-CQFLQTDKKELEKSEHQ-DEVTTLQVKEQDQTVLV---
>Rapgef5_[Chrysemys_picta_bellii]_641751788
------------------------------------------------------------
--------------G------VLCK-----LQER-DDIGRIELVQKLARE----------
-----N-CQFLQVDRKEPEKTE-QNDEVSTIEVKEQDQDVLV---
>Rapgef5_[Chrysochloris_asiatica]_586448209
-LARLTSAM---QRELA-AVIALKARKSAVE------QDEETGD---QHVALIEAEGV--
---PESQA------G------VMCK-----LQER-DDIGRIELVQKLARE----------
-----N-CQLLQTDKKEQEKSEHQEDEVTTVQVKEQDQNVLV---
>Rapgef5_[Dasypus_novemcinctus]_488539542
-LARLTSAV---QRELA-AVIALKARKSAIE------QEEENSD---KHVAVTETEGV--
---PESQA------G------VMCK-----LHER-DDIGRIELVQKLARE----------
-----N-CQFLQTDKKEQEKSEHQDDDLTTVQVKEQDQSVLV---
>Rapgef5_[Echinops_telfairi]_850284753
-LARLTSAV---QRELA-AVIALKARKSALE------QEEESGD---KHIAGTEAEGV--
---PESQA------G------VMCK-----LHER-DDLGRIELVQKLARE----------
-----N-CHLLQTDRKEHEKSEHPEDEVSTIQVKEQDRDVLV---
>Rapgef5_[Equus_asinus]_958806827
-LARLTSAV---QRELA-AVIALKARKSALE------QDEENSD---KHVTVTEAEGV--
---PDSQA------G------VMCK-----LQER-DDIGRIELVQKLARE----------
-----N-CQFLQTDKKEQEKSEHQDDEVTTVQVKDQEQNVLV---
>Rapgef5_[Equus_caballus]_194209713
-LARLTSAV---QRELA-AVIALKARKSALE------QDEENSD---KHVTVTEAEGV--
---PDSQA------G------VMCK-----LQER-DDIGRIELVQKLARE----------
-----N-CQFLQTDKKEQEKSEHQDDEVMTVQVKEQEQNVLV---
>Rapgef5_[Equus_przewalskii]_664704332
-LARLTSAV---QRELA-AVIALKARKSALE------QDEENSV---KHVTVTEAEGV--
---PDSQA------G------VMCK-----LQER-DDIGRIELVQKLARE----------
-----N-CQFLQTDKKEQEKSEHQDDEVMTVQVKEQEQNVLV---
>Rapgef5_[Erinaceus_europaeus]_1016649020
-LARLTSAV---QRELA-AVIALKARKSALE------QDEENSD---KHVPVTEAGGV--
---PDSKA------G------VMCK-----LQER-DDTGKIELVQKLARE----------
-----N-CQILQMDKKEQEKSEQQDDEVTTVGVKEQNQNVLV---
>Rapgef5_[Felis_catus]_755702256
-LARLTSAV---QRELA-AVIALKARKSAIE------QDEDNGD---KHVIVAEAEGV--
---PDSQA------G------VMCK-----LQER-DDIGRIELVQKLTRE----------
-----N-CQFLQTDKKEQEKSEHLDEEVATVQVKEQERNVLV---
>Rapgef5_[Leptonychotes_weddellii]_585182469
-LARLTSAV---QRELA-AVIALKARKSAIE------QDEENRD---KHVIVTEAGGV--
---PDSQA------G------VMCK-----LQER-DDIGRIELVQKLARE----------
-----N-CQFLQTDKKEQEKSEHQDDEVRTVQVKEQEHNVLV---
>Rapgef5_[Loxodonta_africana]_731461513
-LARLTSAV---QRELA-AVIALKARKSAIE------QDEENSD---KHIAVPEAEGV--
---PQSQA------G------VMCK-----LQER-DDIGRIELVQKLARE----------
-----N-CQLLQTDKKELEKSEHQDDEVTTVQVKEQDQNVLV---
>Rapgef5_[Mus_musculus]_568979472
-LARLTSAV---QRELA-AVIALKARKSAIE------QDDENAD---KHVTVTEANNG--
---PDPQA------G------VMCK-----LQER-DDIGRIELVHKLARE----------
-----N-CQFLQTEKKESEKLEQQDDEVTMVQVKEQGQSVLV---
>Rapgef5_[Odobenus_rosmarus_divergens]_472355446
-LARLTSAV---QRELA-AVIALKARKSAVE------QDEENSD---KHDIVTEAGGV--
```

---PDSQA------G------VMCK-----LQER-DDIGRIELVQKLARE----------
-----N-CQFLQTDKKEQEKSEHQDDEVRTVRVKEQEHNVLV---
>Rapgef5_[Orcinus_orca]_465971427
-LCRLTSAV---QRELA-AVIALKARKSAIE------QDEENSD---KHVTVTEAEGV--
---ADPQA------G------VICK-----LQER-DDIGRIELVQKLARE----------
-----N-CQFLQTDKKEQEKSEHQDDEVTTVQVKEQDRDVLV---
>Rapgef5_[Panthera_tigris_altaica]_987416040
-LARLTSAV---QRELA-AVIALKARKSAIE------QDEDNGD---KHVIVAEAEGV--
---PDSQA------G------VMCK-----LQER-DDIGRIELVQKLARE----------
-----N-CQFLQTDKKEQEKSEHLDEEVATVQVKEQERNVLV---
>Rapgef5_[Peromyscus_maniculatus_bairdii]_1008780728
-LARLTSAV---QRELA-AVIALKARKSAVE------QDDENAD---KHIAVTEANDG--
---PDPQA------G------VMCK-----LQER-DDIGRIELVQKLARE----------
-----N-CQFLQTDKKEPEKSEQQDDEVTTVQVKEQGQSVLV---
>Rapgef5_[Physeter_catodon]_593712772
-LCRLTSAV---QRELA-AVIALKARKSAVE------QDEENSD---KHVTVTEAEGV--
---ADPQA------G------VICK-----LQER-DDIGRIELVQKLARE----------
-----N-CQFLQTDKKEQEKSEHQDDEVTTVQVKEQDRDVLV---
>Rapgef5_[Pteropus_vampyrus]_759124282
-LARLTSAV---QRELA-AVIALKAKKSAMA------QDEENSD---KHVTVTEAEGV--
---PDSQA------G------VMCK-----LQER-DDMGRIELVQKLAKE----------
-----N-CQFLQTEKKEREKSEHQDDEVTTVQ--EQEQDVLV---
>Rapgef5_[Rousettus_aegyptiacus]_1012209897
-LARLTSAV---QRELA-AVIALKAKKSALE------QDEDNSE---KHVTVAEAGGA--
---PDSQA------G------VMCK-----LQER-DDIGRIELVQKLARE----------
-----N-CQFLQTEKKEREKSEH-DDEVTTVQ--EQEQDVLV---
>Rapgef5_[Sorex_araneus]_836703300
-LARLTSAM---QRELA-AVIALKARKSALE------QDEENSE---KHVPGTEGENV--
---LDSRA------G------VMCK-----LQER-DDIGRIELVQKLARD----------
-----N-CQCLQTEKKEQEKSEQQDEEAPRVQVREQEENVLV---
>Rapgef5_[Sus_scrofa]_927180328
-LARLTSAV---QRELA-AVIALKAKKSAIE------QDEDNSD---KRETVTEADCI--
---PDPQA------G------VMCK-----LQER-DDIGRIELVQKLARE----------
-----N-CQFLQTDKKEQEKSDNQDDEVTTVQVKEQDRNVLV---
>Rapgef5_[Trichechus_manatus_latirostris]_471379407
-LARLTSAM---QRELA-AVIALKARKSAIE------QDEENSD---KHIAVPEAEDG--
---PESQA------G------VMCK-----LQER-DDIGRIELVQKLARE----------
-----N-CQLLQTDKKEQEKSEHQDDEVTTVQVTEQDQNVLV---
>Rapgef5_[Ursus_maritimus]_670987468
-LARLTSAV---QRELA-AVIALKARKSAIE------QDEENSD---KHVIVTEAGGV--
---PDSQA------G------VMCK-----LQER-DDIGRIELVQKLARE----------
-----N-CQFLQTDKKEQEKSEHQDDEVTTVQVKEQERNVLV---
>Rapgef5_[Vicugna_pacos]_970711077
-LARLTSAV---QRELA-AVIALKARKSAVE------RDEDNNG---KHVTVTEAEGV--
---PGPQA------G------VMCK-----LQEK-DDIGRIELVQKLARE----------
-----N-CQCLQTDKKEQEKSEHQDDEVTTIQVKEQDQNVLV---
>Rapgef6_[Acanthisitta_chloris]_678007447
AFANMTMSV---RRELCSVMIFEVVEQAGAI---ILEDGQELDS---WYVILNGTVEISY
---PDGK---------------S-E---S-LCMG-NSFGITPSLDKQYMNGVVRTKVD--
-----D-CQFVCIAQQDYWRI-LNHVEKNTHKVEE-EGEIV----
>Rapgef6_[Acinonyx_jubatus]_961725169
AFANMTMSV---RRELCSVMIFEVVEQAGAV---ILEDGQELDS---WYVILNGTVEITH
---PDGK---------------V-E---N-LFMG-NSFGITPTLDKQYMHGVVRTKVD--
-----D-CQFVCIAQQDYWRI-LNHVEKNTHKVEE-EGEIV----

>Rapgef6_[Acromyrmex_echinatior]_746838626
AFTNMTLAV---RRALCAVMVFAVVDRAGMV---VLNDGEELDS---WSVLINGAVEIEH
---SNGE---------------I-E---Q-LGLG-DSFGILPTMERLLHRGVMRTKCD--
-----D-CQFVCVTQADYFRI-QHQGEENTRRHE-ENGRVI----
>Rapgef6_[Ailuropoda_melanoleuca]_301782691
AFANMTMSV---RRELCSVMIFEVVEQAGAI---ILEDGQELDS---WYVILNGTVEITH
---PDGK---------------V-E---N-LFMG-NSFGITPTLDKQYMHGVIRTKVD--
-----D-CQFVCIAQQDYWRI-LNHVEKNTHKVEE-EGEIV----
>Rapgef6_[Alligator_mississippiensis]_950961539
AFANMTMSV---RRELCSVMIFEVVEQAGAI---ILEDGQELDS---WYVILNGTVEISY
---PDGK---------------T-E---N-LCMG-NSFGITPSLEKQYMNGVVRTKVD--
-----D-CQFVCIAQQDYWRI-LNHVEKNTHKVEE-EGEIV----
>Rapgef6_[Anolis_carolinensis]_637269198
AFANMTMSV---RRELCSVMIFEVVEQAGAI---ILEDGQELDS---WYVILNGTVEISH
---PDGK---------------T-E---S-LCMG-NSFGITPSLEKQYMNGVVRTKVD--
-----D-CQFVCIAQQDYWRI-LNHVEKNTHKVEE-EGEIV----
>Rapgef6_[Anser_cygnoides_domesticus]_902883087
AFANMTMSV---RRELCSVMIFEVVEQAGAI---ILEDGQELDS---WYVILNGTVEISY
---PDGK---------------S-E---S-LCMG-NSFGITPSLEKQYMNGVVRTKVD--
-----D-CQFVCIAQQDYWRI-LNHVEKNTHKVEE-EGEIV----
>Rapgef6_[Aotus_nancymaae]_817253569
AFANMTMSV---RRELCSVMIFEVVEQAGAV---ILEDGQELDS---WYVILNGTVEISH
---PDGK---------------V-E---N-LFMG-NSFGITPTLDKQYMHGIVRTKVD--
-----D-CQFVCIAQQDYWRI-LNHVEKNTHKVEE-EGEIV----
>Rapgef6_[Apis_cerana]_1035602523
AFTNMTLAV---RRALCAVMVFAVVERAGMI---VLNDGEELDS---WSVLINGAVEIEH
---SNGE---------------I-E---Q-LHLG-DSFGILPTMERLLHRGVMRTKCD--
-----D-CQFVCVTQADYFRI-QHQGEENTRRHE-ENGRVI----
>Rapgef6_[Apis_florea]_820851405
AFTNMTLAV---RRALCAVMVFAVVERAGMI---VLNDGEELDS---WSVLINGAVEIEH
---SNGE---------------I-E---Q-LHLG-DSFGILPTMERLLHRGVMRTKCD--
-----D-CQFVCVTQADYFRI-QHQGEENTRRHE-ENGRVI----
>Rapgef6_[Aptenodytes_forsteri]_686614740
AFANMTMSV---RRELCSVMIFEVVEQAGAI---ILEDSQELDS---WYVILNGTVEITY
---PDGK---------------S-E---S-LCMG-NSFGITPSLDKQYMNGVVRTKVD--
-----D-CQFVCIAQQDYWRI-LNHVEKNTHKVEE-EGEIV----
>Rapgef6_[Aquila_chrysaetos_canadensis]_768345498
AFANMTMSV---RRELCSVMIFEVVEQAGAI---ILEDGQELDS---WYVILNGTVEISY
---PDGK---------------S-E---S-LCMG-NSFGITPSLDKQYMNGVVKTKVD--
-----D-CQFVCIAQQDYWRI-LNHVEKNTHKVEE-EGEIV----
>Rapgef6_[Athalia_rosae]_817056259
AFTNMTLAV---RRALCAVMVFAVVERAGMV---VLNDGEELDS---WSVLINGEVEVEH
---SNGE---------------V-E---Q-LTMG-DSFGILPTMERLLHQGVMRTKCD--
-----D-CQFVCVTQADYFQI-QSQGEENTRRHE-ENGRVI----
>Rapgef6_[Balaenoptera_acutorostrata_scammoni]_594681834
AFANMTMSV---RRELCSVMIFEVVEQAGAI---ILEDGQELDS---WYVILNGTVEISH
---PDGK---------------V-E---N-LFMG-NSFGITPTLDKQYMHGVVRTKVD--
-----D-CQFVCIAQQDYWRI-LNHVEKNTHKVEE-EGEIV----
>Rapgef6_[Balearica_regulorum_gibbericeps]_723546616
AFANMTMSV---RRELCSVMIFEVVEQAGAI---ILEDGQELDS---WYVILNGTVEISY
---PDGK---------------S-E---S-LCMG-NSFGITPSLDKQYMNGVVRTKVD--
-----D-CQFVCIAQQDYWRI-LNHVEKNTHKVEE-EGEIV----
>Rapgef6_[Bison_bison_bison]_742217352
AFANMTMSV---RRELCSVMIFEVVEQAGAV---ILEDGQELDS---WYVILNGTVEISH

---PDGK---------------V-D---N-LFMG-NSFGITPTLDKQYMHGVVRTKVD--
-----D-CQFVCIAQQDYWRI-LNHVEKNTHKVEE-EGEIV----
>Rapgef6_[Bos_mutus]_440912203
AFANMTMSV---RRELCSVMIFEVVEQAGAV---ILEDGQELDS---WYVILNGTVEISH
---PDGK---------------V-D---N-LFMG-NSFGITPTLDKQYMHGVVRTKVD--
-----D-CQFVCIAQQDYWRI-LNHVEKNTHKVEE-EGEIV----
>Rapgef6_[Bos_taurus]_359067234
AFANMTMSV---RRELCSVMIFEVVEQAGAV---ILEDGQELDS---WYVILNGTVEISH
---PDGK---------------V-D---N-LFMG-NSFGITPTLDKQYMHGVVRTKVD--
-----D-CQFVCIAQQDYWRI-LNHVEKNTHKVEE-EGEIV----
>Rapgef6_[Buceros_rhinoceros_silvestris]_676722949
AFANMTMSV---RRELCSVMIFEVVEQAGAI---ILEDGQELDS---WYVILNGTVEISY
---PDGK---------------S-E---S-LCMG-NSFGITPSLEKQYMNGVVRTKVD--
-----D-CQFVCIAQQDYWRI-LNHVEKNTHKVEE-EGEIV----
>Rapgef6_[Callithrix_jacchus]_675644732
AFANMTMSV---RRELCSVMIFEVVEQAGAV---ILEDGQELDS---WYVILNGTVEISH
---PDGK---------------V-E---N-LFMG-NSFGITPTLDKQYMHGIVRTKVD--
-----D-CQFVCIAQQDYWRI-LNHVEKNTHKVEE-EGEIV----
>Rapgef6_[Callorhinchus_milii]_632966528
AFANMTMFV---RRELCSVMIFEVVEHAGAI---IIDEGKELES---WYVILNGSVEITD
---SSSR---------------I-E---I-LCMG-NSFGILPSLEKQYMHGAVRTKTD--
-----D-CQFVSIAQQDYCRI-LNQVEKNTHKVEE-EGEIVMV--
>Rapgef6_[Camelus_ferus]_560915805
AFANMTMSV---RRELCSVMIFEVVEQAGAV---ILEDGQELDS---WYVILNGTVEISH
---PDGK---------------V-E---N-LFMG-NSFGITPTLDKQYMRGVVRTKVD--
-----D-CQFVCIAQQDYWRI-LNHVEKNTHKVEE-EGEIV----
>Rapgef6_[Capra_hircus]_548473191
AFANMTMSV---RRELCSVMIFEVVEQAGAV---ILEDGQELDS---WYVILNGTVEISY
---PDGK---------------V-D---N-LFMG-NSFGITPTLDKQYMHGVVRTKVD--
-----D-CQFVCIAQQDYWRI-LNHVEKNTHKVEE-KGEIV----
>Rapgef6_[Caprimulgus_carolinensis]_683447165
AFANMTMSV---RRELCSVMIFEVVEQAGAI---ILEDGQELDS---WYVILNGTVEISY
---PDGK---------------S-E---S-LCMG-NSFGITPSLDKQYMNGVVRTKVD--
-----D-CQFVCIAQQDYWRI-LNHVEKNTHKVEE-EGEIV----
>Rapgef6_[Cariama_cristata]_698452567
AFANMTMSV---RRELCSVMIFEVVEQAGAI---ILEDGQELDS---WYVILNGTVEISY
---PDGK---------------S-E---S-LCMG-NSFGITPSLDKQYMNGVVRTKVD--
-----D-CQFVCIAQQDYWRI-LNHVEKNTHKVEE-EGEIV----
>Rapgef6_[Carlito_syrichta]_640806927
AFTNMTMSV---RRELCSVMIFEVVEQAGAI---ILEDGQELDS---WYVILNGTVEISH
---PDGK---------------I-E---N-LFMG-NSFGITPTLDKQYMHGIVRTKVD--
-----D-CQFVCIAQQDYWRI-LNHVEKNTHKVEE-EGEIV----
>Rapgef6_[Cathartes_aura]_677228612
AFANMTMSV---RRELCSVMIFEVVEQAGAI---ILEDGQELDS---WYVILNGTVEISY
---PDGK---------------S-E---S-LCMG-NSFGITPSLDKQYMNGVVRTKVD--
-----D-CQFVCIAQQDYWRI-LNHVEKNTHKVEE-EGEIV----
>Rapgef6_[Cebus_capucinus_imitator]_1044364594
AFANMTMSV---RRELCSVMIFEVVEQAGAV---ILEDGQELDS---WYVILNGTVEISH
---PDGK---------------V-E---N-LFMG-NSFGITPTLDKQYMHGIVRTKVD--
-----D-CQFVCIAQQDYWRI-LNHVEKNTHKVEE-EGEIV----
>Rapgef6_[Cephus_cinctus]_1000734963
AFTNMTLAV---RRALCAVMVFAVVERAGMV---VLNDGEELDS---WSVLINGAVEVEH
---SNGE---------------V-E---Q-LHLG-DSFGILPTMERLLHRGIMRTKCD--
-----D-CQFVCVTQADYFRI-QHQGEENTRRHE-ENGRVI----

>Rapgef6_[Ceratotherium_simum_simum]_478491851
AFANMTMSV---RRELCSVMIFEVVEQAGAV---ILDDGQELDS---WYVILNGTVEISH
---PDGK---------------V-E---N-LFMG-NSFGITPTLDKQYMHGVVRTKVD--
-----D-CQFVCIAQQDYWRI-LNHVEKNTHKVEE-EGEIV----
>Rapgef6_[Cercocebus_atys]_795413582
AFANMTMSV---RRELCSVMIFEVVEQAGAI---ILEDGQELDS---WYVILNGTVEISH
---PDGK---------------V-E---N-LFMG-NSFGITPTLDKQYMHGIVRTKVD--
-----D-CQFVCIAQQDYWRI-LNHVEKNTHKVEE-EGEIV----
>Rapgef6_[Chaetura_pelagica]_701407210
AFANMTMSV---RRELCSVMIFEVVEQAGAI---ILEDGQELDS---WYVILNGTVEISY
---PDGK---------------S-E---S-LCMG-NSFGITPSLDKQYMNGVVRTKVD--
-----D-CQFVCIAQQDYWRI-LNHVEKNTHKVEE-EGEIV----
>Rapgef6_[Chlamydotis_macqueenii]_705691314
AFANMTMSV---RRELCSVMIFEVVEQAGAI---ILEDGQELDS---WYVILNGTVEISY
---PDGK---------------T-E---S-LCMG-NSFGITPSLDKQYMNGVVRTKVD--
-----D-CQFVCIAQQDYWRI-LNHVEKNTHKVEE-EGEIV----
>Rapgef6_[Chlorocebus_sabaeus]_635123469
AFANMTMSV---RRELCSVMIFEVVEQAGAI---ILEDGQELDS---WYVILNGTVEISH
---PDGK---------------V-E---N-LFMG-NSFGITPTLDKQYMHGIVRTKVD--
-----D-CQFVCIAQQDYWRI-LNHVEKNTHKVEE-EGEIV----
>Rapgef6_[Chrysemys_picta_bellii]_530612721
AFANMTMSV---RRELCSVMIFEVVEQAGAI---ILEDGQELDS---WYVILNGTVEISH
---PDGK---------------T-E---S-LCMG-NSFGITPSLDKQYMNGVVRTKVD--
-----D-CQFVCIAQQDYWRI-LNHVEKNTHKVEE-EGEIV----
>Rapgef6_[Chrysochloris_asiatica]_586485010
AFANMTMSV---RRELCSVMIFEVVEQAGAI---ILEDGEELDS---WYVILNGTVEISH
---PDGK---------------I-E---N-LFMG-NSFGITPTLDKQYMHGVVRTKVD--
-----D-CQFVCIAQQDYWRI-LNHVEKNTHKVEE-EGEIV----
>Rapgef6_[Colobus_angolensis_palliatus]_795224884
AFANMTMSV---RRELCSVMIFEVVEQAGAI---ILEDGQELDS---WYVILNGTVEISH
---PDGK---------------V-E---N-LFMG-NSFGITPTLDKQYMHGIVRTKVD--
-----D-CQFVCIAQQDYWRI-LNHVEKNTHKVEE-EGEIV----
>Rapgef6_[Columba_livia]_915537474
AFANMTMSV---RRELCSVMIFEVVEQAGAI---ILEDGQELDS---WYVILNGTVEISY
---PDGK---------------S-E---S-LCMG-NSFGITPSLDKQYMNGVVRTKVD--
-----D-CQFVCIAQQDYWRI-LNHVEKNTHKVEE-EGEIV----
>Rapgef6_[Condylura_cristata]_507959751
AFANMTMSV---RRELCSVMIFEVVEQAGAV---ILEDGQELDS---WYVILNGTVEISH
---PDGK---------------V-E---N-LFMG-NSFGITPTLDKQYMRGVVRTKVD--
-----D-CQFVCIAQQDYWRI-LNHVEKNTHKVEE-EGEIV----
>Rapgef6_[Corvus_cornix_cornix]_726993272
AFANMTMSV---RRELCSVMIFEVVEQAGAI---ILEDGQELDS---WYVILNGTVEISY
---PDGK---------------S-E---S-LCMG-NSFGITPSLDKQYMNGVVRTKVD--
-----D-CQFVCIAQQDYWRI-LNHVEKNTHKVEE-EGEIV----
>Rapgef6_[Crassostrea_gigas]_762094130
AFANMTQPI---RRELCAVMVFAVIEQRGTV---VMKDGEELDS---WSVILNGQVEIVH
---PEGS---------------A-E---F-LQMG-DSFGISPTLDKMYHKGTMKTLLD--
-----D-CQFVCIAQEDYHRI-LDKGKENTEKHVE-EGQVV----
>Rapgef6_[Cynoglossus_semilaevis]_1035260107
AFANMTMSV---RRDLCSVMVFEVVEQAGTV---ILHDKQELDL---WYVILNGAVEISH
---QEGR---------------V-E---V-LCMG-NSFGISPSLDKQYMNGEVRTKGD--
-----D-CQFVCIAQEDYWRI-LNHVEKNMHKVEE-EGEIVMV--
>Rapgef6_[Cyprinodon_variegatus]_974056205
AFANMTMSV---RRELCSVMVFEVVEQSGTI---ILHNKQELDH---WYVILNGAVEISH

---PEAR---------------T-E---T-LCMG-NSFGISPSLDKQYMNGEVRTKGD--
-----D-CQFVCIAQEDYWRI-LNHVEKNTHKVEE-EGEIV----
>Rapgef6_[Daphnia_magna]_1022775171
AFANMTLSV---RRALCRVMVFAVVEKAGTV---VMNDGEELDS---WSVIINGHVEIDG
G--ASGE---------------P-G---RALHLG-DSFGITPTMDKLYHRGVMRTKCD--
-----D-CQFVCITQTDYFSI-LHKGEENTRRHEE-NGRVV----
>Rapgef6_[Echinops_telfairi]_507622381
AFANMTMSV---RRELCSVMIFEVVEQAGAV---ILEDGEELDS---WYVILNGTVEISH
---SGGK---------------I-E---N-LFMG-NSFGITPTLDKQYMHGVVRTKVD--
-----D-CQFVCIAQQDYWRI-LNHVEKNTHKVEE-EGEIV----
>Rapgef6_[Egretta_garzetta]_697849644
AFANMTMSV---RRELCSVMIFEVVEQAGAI---ILEDGQELDS---WYVILNGTVEISY
---PDGK---------------S-E---S-LCMG-NSFGITPSLDKQYMNGVVRTKVD--
-----D-CQFVCIAQQDYWRI-LNHVEKNTHKVEE-EGEIV----
>Rapgef6_[Elephantulus_edwardii]_585707696
AFANMTMSV---RRELCSVMIFEVVEQAGAI---ILEDGEELDS---WYVILNGTVEISH
---PDGK---------------I-E---N-LFMG-NSFGITPTLDKQYMHGVVRTKVD--
-----D-CQFVCIAQQDYWRI-LNHVEKNTHKVEE-EGEIV----
>Rapgef6_[Equus_asinus]_958795644
AFANMTMSV---RRELCSVMIFEVVEQAGAI---ILEDGQELDS---WYVILNGTVEIIH
---PDGK---------------V-E---N-LFMG-NSFGITPTLDKQYMHGVVRTKVD--
-----D-CQFVCIAQQDYWRI-LNHVEKNTHKVEE-EGEIV----
>Rapgef6_[Equus_caballus]_545185041
AFANMTMSV---RRELCSVMIFEVVEQAGAI---ILEDGQELDS---WYVILNGTVEIIH
---PDGK---------------V-E---N-LFMG-NSFGITPTLDKQYMHGVVRTKVD--
-----D-CQFVCIAQQDYWRI-LNHVEKNTHKVEE-EGEIV----
>Rapgef6_[Esox_lucius]_884954379
AFANMTMSV---RRDLCTVMVFEVVEQAGTI---ILQDQQELDL---WYVILNGAVEIGH
---PDSR---------------M-E---T-LCMG-NSFGISPSLDKQYMSGVVRTKGD--
-----D-CQFVCIAQEDYWRI-LNHVEKNTHKVEE-EGEIVMV--
>Rapgef6_[Falco_peregrinus]_909808308
AFANMTMSV---RRELCSVMIFEVVEQAGAI---ILEDGQELDS---WYVILNGTVEISY
---PDGK---------------S-E---S-LCMG-NSFGITPSLDKQYMNGVVRTKVD--
-----D-CQFVCIAQQDYWRI-LNHVEKNTHKVEE-EGEIV----
>Rapgef6_[Felis_catus]_755689979
AFANMTMSV---RRELCSVMIFEVVEQAGAV---ILEDGQELDS---WYVILNGTVEITH
---PDGK---------------V-E---N-LFMG-NSFGITPTLDKQYMHGVVRTKVD--
-----D-CQFVCIAQQDYWRI-LNHVEKNTHKVEE-EGEIV----
>Rapgef6_[Ficedula_albicollis]_1020955556
AFANMTMSV---RRELCSVMIFEVVEQAGAI---ILEDGQELDS---WYVILNGTVEISY
---PDGK---------------S-E---S-LCMG-NSFGITPSLDKQYMNGVVRTKVD--
-----D-CQFVCIAQQDYWRI-LNHVEKNTHKVEE-EGEIV----
>Rapgef6_[Fulmarus_glacialis]_697019310
AFANMTMSV---RRELCSVMIFEVVEQAGAI---ILEDGQELDS---WYVILNGTVEISY
---PDGK---------------S-E---S-LCMG-NSFGITPSLDKQYMNGVVRTKVD--
-----D-CQFVCIAQQDYWRI-LNHVEKNTHKVEE-EGEIV----
>Rapgef6_[Fundulus_heteroclitus]_831570944
AFANMTMSV---RRDLCSVMVFEVVEQSGTV---ILHNKQELDH---WYVILNGAVEISH
---PESR---------------S-E---T-LCMG-NSFGISPSLDKQYMNGEVRTKGD--
-----D-CQFVCIAQEDYWRI-LNHVEKNTHKVEE-EGEIVMV--
>Rapgef6_[Galeopterus_variegatus]_667332237
AFANMTMSV---RRELCSVMIFEVVEQAGAI---ILEDGQELDS---WYVILNGTVEISH
---PDGK---------------V-E---N-LFMG-NSFGITPTLDKQHMHGVVRTKVD--
-----D-CQFVCIAQQDYWRI-LNHVEKNTHKVEE-EGEIV----

>Rapgef6_[Gallus_gallus]_971418561
AFANMTMSV---RRELCSVMIFEVVEQAGAI---ILEDGQELDS---WYVILNGTVEISY
---PDGK---------------S-E---S-LCMG-NSFGITPSLDKQYMNGVVRTKVD--
-----D-CQFVCIAQQDYWRI-LNHVEKNTHKVEE-EGEIV----
>Rapgef6_[Gavia_stellata]_678177468
AFANMTMSV---RRELCSVMIFEVVEQAGAI---ILEDGQELDS---WYVILNGTVEISY
---PDGK---------------S-E---S-LCMG-NSFGITPSLDKQYMNGVVRTKVD--
-----D-CQFVCIAQQDYWRI-LNHVEKNTHKVEE-EGEIV----
>Rapgef6_[Gekko_japonicus]_975091751
AFANMTMSV---RRELCSVMIFEVVEQAGTI---ILEDGQELDS---WYVILNGTVEISH
---PDGK---------------T-E---S-LCMG-NSFGITPSLEKQYMNGVVRTKVD--
-----D-CQFVCIAQQDYWRI-LNHVEKNTHKVEE-EGEIV----
>Rapgef6_[Haliaeetus_albicilla]_700361031
AFANMTMSV---RRELCSVMIFEVVEQAGAI---ILEDGQELDS---WYVILNGTVEISY
---PDGK---------------S-E---S-LCMG-NSFGITPSLDKQYMNGVVRTKVD--
-----D-CQFVCIAQQDYWRI-LNHVEKNTHKVEE-EGEIV----
>Rapgef6_[Haliaeetus_leucocephalus]_729760461
AFANMTMSV---RRELCSVMIFEVVEQAGAI---ILEDGQELDS---WYVILNGTVEISY
---PDGK---------------S-E---S-LCMG-NSFGITPSLDKQYMNGVVRTKVD--
-----D-CQFVCIAQQDYWRI-LNHVEKNTHKVEE-EGEIV----
>Rapgef6_[Harpegnathos_saltator]_749732965
AFTNMTLAV---RRALCAVMVFAVVERAGMV---VLNDGEELDS---WSVLINGAVEVEQ
---SNGE---------------I-Q---Q-LCLG-DCFGILPTMDRLLHCGVMRTKCD--
-----D-CQFVCVTQADYFRI-QHQGEENTRRHE-ENGRVI----
>Rapgef6_[Heterocephalus_glaber]_512948458
AFANMTMSV---RRELCSVMIFEVVEQAGAV---ILEDGQELDS---WYVILNGTVEISH
---PDGK---------------V-E---N-LFMG-NSFGITPTLDKQYMHGIVRTKVD--
-----D-CQFVCIAQRDYWRI-LNHVEKNTHKVEE-EGEIV----
>Rapgef6_[Ictalurus_punctatus]_1042302955
AFANMTMSV---RRELCTVMVFEVVEHAGTI---ILHDKQELNL---WYVILNGAVEISY
---GDGR---------------T-E---I-LCMG-NSFGISASLVKQCMNGEVRTKGD--
-----D-CQLVCIAQEDYWRI-LNHVEKNMHKVEE-EGEIVMV--
>Rapgef6_[Ictidomys_tridecemlineatus]_532100447
AFANMTMSV---RRELCSVMIFEVVDQAGAI---ILEDGQELDS---WYVILNGTVEINH
---PDGK---------------V-E---N-LFMG-NSFGITPTLDKQYMRGIVRTKVD--
-----D-CQFVCIAQQDYWRI-LNHVEKNTHKVEE-EGEIV----
>Rapgef6_[Larimichthys_crocea]_808885705
AFANMTMSV---RRDLCSVMVFEVVEQAGTV---ILHNKQELDL---WYVILNGAVEISH
---PEGR---------------V-E---T-LCMG-NSFGISPSLDKQYMNGEVRTKGD--
-----D-CQFVCIAQEDYWRI-LNHVEKNTHKVEE-EGEIVMV--
>Rapgef6_[Lepisosteus_oculatus]_972976862
AFANMTMSV---RRELCNVMKFEVIEHAGKV---ILQDGQELDM---WYVILNGSVEVFH
---PDGR---------------L-E---I-LCMG-NSFGISPSLDKQFMSGLVRTKVE--
-----D-CQFVCIAQEDYWRI-LNHVEKNTHKVEE-EGEIVMV--
>Rapgef6_[Leptosomus_discolor]_700411390
AFANMTMSV---RRELCSVMIFEVVEQAGAI---ILEDGQELDS---WYVILNGTVEISY
---PDGK---------------S-E---S-LCMG-NSFGITPSLDKQYMNGVVRTKVD--
-----D-CQFVCIAQQDYWRI-LNHVEKNTHKVEE-EGEIV----
>Rapgef6_[Limulus_polyphemus]_926627213
AFANMTLAV---RRALCAVMVFAVVEKAGTV---VMNDGEELDS---WSVIVNGQVEVEL
---PDGN---------------I-Q---E-LHFG-DSFGITATTEKMYHQGTMRTKVD--
-----D-CQFVCIAQSDYHKI-LHQGEENTKKHEE-DGQVI----
>Rapgef6_[Lipotes_vexillifer]_602699629
AFANMTMSV---RRELCSVMIFEVVEQAGAI---ILEDGQELDS---WYVILNGTVEISH

---PDGK---------------V-E---N-LFMG-NSFGITPTLDKQYTHGVVRTKVD--
-----D-CQFVCIAQQDYWRI-LNHVEKNTHKVEE-EGEIV----
>Rapgef6_[Macaca_nemestrina]_795530958
AFANMTMSV---RRELCSVMIFEVVEQAGAI---ILEDGQELDS---WYVILNGTVEISH
---PDGK---------------V-E---N-LFMG-NSFGITPTLDKQYMHGIVRTKVD--
-----D-CQFVCIAQQDYWRI-LNHVEKNTHKVEE-EGEIV----
>Rapgef6_[Mandrillus_leucophaeus]_795275466
AFANMTMSV---RRELCSVMIFEVVEQAGAI---ILEDGQELDS---WYVILNGTVEISH
---PDGK---------------V-E---N-LFMG-NSFGITPTLDKQYMHGIVRTKVD--
-----D-CQFVCIAQQDYWRI-LNHVEKNTHKVEE-EGEIV----
>Rapgef6_[Marmota_marmota_marmota]_984113345
AFANMTMSV---RRELCSVMIFEVVDQAGAI---ILEDGQELDS---WYVILNGTVEINH
---PDGK---------------V-E---N-LFMG-NSFGITPTLDKQYMRGIVRTKVD--
-----D-CQFVCIAQQDYWRI-LNHVEKNTHKVEE-EGEIV----
>Rapgef6_[Maylandia_zebra]_499022069
AFANMTMSV---RRDLCSVMMFEVVEQAGTV---ILHDKQELDH---WYVILNGAVEISH
---HDGR---------------T-E---T-LCMG-NSFGISPSLDKQYMNGEVRTKGD--
-----D-CQFVCIAQEDYWRI-LNHVERNTHKVEE-EGEIV----
>Rapgef6_[Melopsittacus_undulatus]_527258131
AFANMTMSV---RRELCSVMIFEVVEQAGAI---ILEDGQELDS---WYVILNGTVEISY
---PDGK---------------S-E---S-LCMG-NSFGITPSLDKQYMNGVVRTKVD--
-----D-CQFVCIAQQDYWRI-LNHVEKNTHKVEE-EGEIV----
>Rapgef6_[Microcebus_murinus]_829753475
AFANMTMSV---RRELCSVMIFEVVEQAGAI---ILEDGQELDS---WYVILNGTVEISH
---PDGR---------------V-E---N-LFMG-NSFGITPTLDKQYMHGVVRTKVD--
-----D-CQFVCIAQQDYWRI-LNHVEKNTHKVEE-EGEIV----
>Rapgef6_[Microtus_ochrogaster]_532012666
AFANMTMSV---RRELCSVMIFEVVEQAGAV---ILEDGQELDS---WYVILNGTVEISH
---PDGR---------------I-E---N-LFMG-NSFGIVPTLDKQHMHGVVRTKVD--
-----D-CQFVCIAQQDYWRI-LNHVEKNTHKVEE-EGEIV----
>Rapgef6_[Mus_musculus]_357394799
AFANMTMSV---RRELCSVMVFEVVEQAGAV---ILEDGQELDS---WYVILNGTVEISH
---PDGK---------------I-E---N-LFMG-NSFGIVPTLDKQHMHGAVRTKVD--
-----D-CQFVCIAQQDYWRI-LNHVEKNTHKVEE-EGEIV----
>Rapgef6_[Mustela_putorius_furo]_511825338
AFANMTMSV---RRELCTVMIFEVVEQAGAI---ILEDGQELDS---WYVILNGTVEITH
---PDGK---------------V-E---N-LFMG-NSFGITPTLDKQYMHGVVRTKVD--
-----D-CQFVCIAQQDYWRI-LNHVEKNTHKVEE-EGEIV----
>Rapgef6_[Myotis_brandtii]_554541927
AFANMTMSV---RRELCSVMIFEVVEQAGAI---ILEDGQELDS---WYVILNGTVEISH
---SEGK---------------V-E---N-LFMG-NSFGITPTLDKQYMHGVVRTKVD--
-----D-CQFVCIAQQDYWRI-LNHVEKNTHKVEE-EGEIV----
>Rapgef6_[Myotis_lucifugus]_558176189
AFANMTMSV---RRELCSVMIFEVVEQAGAI---ILEDGQELDS---WYVILNGTVEISH
---SDGK---------------V-E---N-LFMG-NSFGITPTLDKQYMHGVVRTKVD--
-----D-CQFVCIAQQDYWRI-LNHVEKNTHKVEE-EGEIV----
>Rapgef6_[Neolamprologus_brichardi]_583979497
AFANMTMSV---RRDLCSVMMFEVVEQAGTV---ILHDKQELDH---WYVILNGAVEISY
---HDGR---------------T-E---T-LCMG-NSFGISPSLDKQYMNGEVRTKGD--
-----D-CQFVCIAQEDYWRI-LNHVERNTHKVEE-EGEIVMV--
>Rapgef6_[Nestor_notabilis]_701323631
AFANMTMSV---RRELCSVMIFEVVEQAGAI---ILEDGQELDS---WYVILNGTVEISY
---PDGK---------------S-E---S-LCMG-NSFGITPSLDKQYMNGVVRTKVD--
-----D-CQFVCIAQQDYWRI-LNHVEKNTHKVEE-EGEIV----

>Rapgef6_[Nomascus_leucogenys]_332221644
AFANMTMSV---RRELCSVMIFEVVEQAGAI---ILEDGQELDS---WYVILNGTVEISH
---PDGK---------------V-E---N-LFMG-NSFGITPTLDKQYMHGIVRTKVD--
-----D-CQFVCIAQQDYWRI-LNHVEKNTHKVEE-EGEIV----
>Rapgef6_[Odobenus_rosmarus_divergens]_472377734
AFANMTMSV---RRELCSVMIFEVVEQAGAI---ILEDGQELDS---WYVILNGTVEITH
---PDGK---------------V-E---N-LFMG-NSFGITPTLDKQYMHGVVRTKVD--
-----D-CQFVCIAQQDYWRI-LNHVEKNTHKVEE-EGEIV----
>Rapgef6_[Orcinus_orca]_466058779
AFANMTMSV---RRELCSVMIFEVVEQAGAI---ILEDGQELDS---WYVILNGTVEISH
---PDGK---------------V-E---N-LFMG-NSFGITPTLDKQYTHGVVRTKVD--
-----D-CQFVCIAQQDYWRI-LNHVEKNTHKVEE-EGEIV----
>Rapgef6_[Oreochromis_niloticus]_542236526
AFANMTMSV---RRDLCSVMMFEVVEQAGTV---ILHDKQELDH---WYVILNGAVEISH
---HDGR---------------M-E---T-LCMG-NSFGISPTLDKQYMNGEVRTKGD--
-----D-CQFVCIAQEDYWRI-LNHVERNTHKVEE-EGEIV----
>Rapgef6_[Ornithorhynchus_anatinus]_1019073942
AFANMTMSV---RRELCSVMIFEVVEQAGAT---ILEHGQELDS---WYVILNGTVEISH
---PDGK---------------S-E---S-LCMG-NSFGIIPSLDKQYMNGVVRTKVD--
-----D-CQFVCIAQQDYWRI-LNHVEKNTHKVEE-EGEIV----
>Rapgef6_[Orycteropus_afer_afer]_634856153
AFANMTMSV---RRELCSVMIFEVVEQAGAI---ILEDGQELDS---WYVILNGTVEISH
---PDGK---------------I-E---N-LFMG-NSFGITPTLDKQYMHGVVRTKVD--
-----D-CQFVCIAQQDYWRI-LNHVEKNTHKVEE-EGEIV----
>Rapgef6_[Otolemur_garnettii]_831213177
AFANMTMSV---RRELCSVMIFEVVEQAGAI---ILEDGQELDS---WYVILNGTVEISH
---PDGK---------------V-E---N-LFMG-NSFGITPTLDKQYMHGIVRTKVD--
-----D-CQFVCIAQQDYWRI-LNHVEKNTHKVEE-EGEIV----
>Rapgef6_[Ovis_aries_musimon]_803320911
AFANMTMSV---RRELCSVMIFEVVEQAGAV---ILEDGQELDS---WYVILNGTVEISY
---PDGK---------------V-D---N-LFMG-NSFGITPTLDKQYMHGVVRTKVD--
-----D-CQFVCIAQQDYWRI-LNHVEKNTHKVEE-KGEIV----
>Rapgef6_[Ovis_aries]_803073498
AFANMTMSV---RRELCSVMIFEVVEQAGAV---ILEDGQELDS---WYVILNGTVEISY
---PDGK---------------V-D---N-LFMG-NSFGITPTLDKQYMHGVVRTKVD--
-----D-CQFVCIAQQDYWRI-LNHVEKNTHKVEE-KGEIV----
>Rapgef6_[Pan_troglodytes]_694912260
AFANMTMSV---RRELCSVMIFEVVEQAGAI---ILEDGQELDS---WYVILNGTVEISH
---PDGK---------------V-E---N-LFMG-NSFGITPTLDKQYMHGIVRTKVD--
-----D-CQFVCIAQQDYWRI-LNHVEKNTHKVEE-EGEIV----
>Rapgef6_[Panthera_tigris_altaica]_591323548
AFANMTMSV---RRELCSVMIFEVVEQAGAV---ILEDGQELDS---WYVILNGTVEITH
---PDGK---------------V-E---N-LFMG-NSFGITPTLDKQYMHGVVRTKVD--
-----D-CQFVCIAQQDYWRI-LNHVEKNTHKVEE-EGEIV----
>Rapgef6_[Pantholops_hodgsonii]_556768279
AFANMTMSV---RRELCSVMIFEVVEQAGAV---ILEDGQELDS---WYVILNGTVEISY
---PDGK---------------V-D---N-LFMG-NSFGITPTLDKQYMHGVVRTKVD--
-----D-CQFVCIAQQDYWRI-LNHVEKNTHKVEE-KGEIV----
>Rapgef6_[Papio_anubis]_685547351
AFANMTMSV---RRELCSVMIFEVVEQAGAI---ILEDGQELDS---WYVILNGTVEISH
---PDGK---------------V-E---N-LFMG-NSFGITPTLDKQYMHGIVRTKVD--
-----D-CQFVCIAQQDYWRI-LNHVEKNTHKVEE-EGEIV----
>Rapgef6_[Parasteatoda_tepidariorum]_1009601738
AFANMTLTI---RRALCAVMVFAVVEKAGTI---VMNDGEELDS---WSVIVNGHVEVEQ

---ADGR---------------I-E---E-LHLG-DSFGITPTTEKMYHQGVMRTKVD--
-----D-CQFVCIAQNDYYRI-LHQGEENTRKHEE-DGEVV----
>Rapgef6_[Parus_major]_998718768
AFANMTMSV---RRELCSVMIFEVVEQAGAI---ILEDGQELDS---WYVILNGTVEISY
---PDGK---------------S-E---S-LCMG-NSFGITPSLEKQYMNGVVRTKVD--
-----D-CQFVCIAQQDYWRI-LNHVEKNTHKVEE-EGEIV----
>Rapgef6_[Peromyscus_maniculatus_bairdii]_1008772020
AFANMTMSV---RRELCSVMIFEVVEQAGAV---ILEDGQELDS---WYVILNGTVEISH
---PDGK---------------S-E---N-LFMG-NSFGIVPTLDKQYMHGVVRTKVD--
-----D-CQFVCIAQQDYWRI-LNHVEKNTHKVEE-EGEIV----
>Rapgef6_[Phaethon_lepturus]_723128522
AFANMTMSV---RRELCSVMIFEVVEQAGAI---ILEDGQELDS---WYVILNGTVEISY
---PDGK---------------S-E---S-LCMG-NSFGITPSLDKQYMTGVVRTKVD--
-----D-CQFVCIAQQDYWRI-LNHVEKNTHKVEE-EGEIV----
>Rapgef6_[Phalacrocorax_carbo]_695144964
AFANMTMSV---RRELCSVMIFEVVEQAGAI---ILEDGQELDS---WYVILNGTVEISY
---PDGK---------------S-E---S-LCMG-NSFGITPSLDKQYMHGVVRTKVD--
-----D-CQFVCIAQQDYWRI-LNHVEKNTHKVEE-EGEIV----
>Rapgef6_[Physeter_catodon]_593723309
AFANMTMSV---RRELCSVMIFEVVEQAGAI---ILEDGQELDS---WYVILNGTVEISH
---PDGK---------------V-E---N-LFMG-NSFGITPTLDKQYMHGVVRTKVD--
-----D-CQFVCIAQQDYWRI-LNHVEKNTHKVEE-EGEIV----
>Rapgef6_[Poecilia_formosa]_617462884
AFANMTMSV---RRDLCSVMVFEVVEQSGTV---ILHNKQELDH---WYVILNGAVEISH
---AESR---------------T-E---T-LCMG-NSFGISPSLDKQYMNGEVRTKGD--
-----D-CQFVCIAQEDYWRI-LNHVEKNTHKVEE-EGEIVMV--
>Rapgef6_[Poecilia_reticulata]_658884514
AFANMTMSV---RRELCSVMVFEVVEQSGTV---ILHNKQELDH---WYVILNGAVEISR
---AESR---------------T-E---T-LCMG-NSFGISPSLDKQYMNGEVRTKGD--
-----D-CQFVCIAQEDYWRI-LNHVEKNTHKVEE-EGEIV----
>Rapgef6_[Pongo_abelii]_686719316
AFANMTMSV---RRELCSVMIFEVVEQAGAI---ILEDGQELDS---WYVILNGTVEISH
---PDGK---------------V-E---N-LFMG-NSFGITPTLDKQYMRGIVRTKVD--
-----D-CQFVCIAQQDYWRI-LNHVEKNTHKVEE-EGEIV----
>Rapgef6_[Protobothrops_mucrosquamatus]_1002581315
AFANMTMSV---RRELCSVMIFEVVEQAGAI---ILEDGQELDS---WYVILNGTVEISY
---PDGK---------------T-E---S-LCMG-NSFGITPSLEKQHMNGVVRTKVD--
-----D-CQFVCIAQQDYWRI-LNHVEKNTHKVEE-EGEIV----
>Rapgef6_[Pseudopodoces_humilis]_929446440
AFANMTMSV---RRELCSVMIFEVVEQAGAI---ILEDGQELDS---WYVILNGTVEISY
---PDGK---------------S-E---S-LCMG-NSFGITPSLEKQYMNGVVRTKVD--
-----D-CQFVCIAQQDYWRI-LNHVEKNTHKVEE-EGEIV----
>Rapgef6_[Pteropus_alecto]_431892688
AFANMTMSV---RRELCSVMIFEVVEQAGAI---ILEDGQELDS---WYVILNGTVEISH
---SDGK---------------V-E---N-LFMG-NSFGITPTLDKQYMRGVVRTKVD--
-----D-CQFVCIAQQDYWRI-LNHVEKNTHKVEE-EGEIV----
>Rapgef6_[Pygoscelis_adeliae]_690439934
AFANMTMSV---RRELCSVMIFEVVEQAGAI---ILEDSQELDS---WYVILNGTVEISY
---PDGK---------------S-E---S-LCMG-NSFGITPSLDKQYMNGVVRTKVD--
-----D-CQFVCIAQQDYWRI-LNHVEKNTHKVEE-EGEIV----
>Rapgef6_[Python_bivittatus]_602628151
AFANMTMSV---RRELCSVMIFEVVEQAGAI---ILEDGQELDS---WYVILNGTVEISH
---PDGK---------------T-E---S-LCMG-NSFGITPSLEKQHMNGVVRTKVD--
-----D-CQFVCIAQQDYWRI-LNHVEKNTHKVEE-EGEIV----

>Rapgef6_[Rhinopithecus_roxellana]_724913891
AFANMTMSV---RRELCSVMIFEVVEQAGAI---ILEDGQELDS---WYVILNGTVEISH
---PDGK---------------V-E---N-LFMG-NSFGITPTLDKQYMHGIVRTKVD--
-----D-CQFVCIAQQDYWRI-LNHVEKNTHKVEE-EGEIV----
>Rapgef6_[Rousettus_aegyptiacus]_1012239480
AFANMTMSV---RRELCSVMIFEVVEQAGAI---ILEDGQELDS---WYVILNGTVEISH
---SDGK---------------V-E---N-LFMG-NSFGIIPTLDKQYMRGVVRTKVD--
-----D-CQFVCIAQQDYWRI-LNHVEKNTHKVEE-EGEIV----
>Rapgef6_[Saccoglossus_kowalevskii]_585647408
AFSNMTLAV---RRALCAVMVFAVVEKAATV---VMKDGEELDS---WSVILNGHVEISR
---PDGT---------------L-E---H-LHLG-DSFGCVPSLTRQTHRGLMKTKVD--
-----D-CQFVCIAQTDYYRI-LHQGEENTRKVEE----------
>Rapgef6_[Saimiri_boliviensis_boliviensis]_725550658
AFANMTMSV---RRELCSVMIFEVVEQAGAV---ILEDGQELDS---WYVILNGTVEISH
---PDGK---------------V-E---N-LFMG-NSFGITPTLDKQYMHGIVRTKVD--
-----D-CQFVCIAQQDYWRI-LNHVEKNTHKVEE-EGEIV----
>Rapgef6_[Sarcophilus_harrisii]_821455239
AFANMTMSV---RRELCSVMIFEVVDQAGAV---ILADGQELDS---WYVILNGTVEISY
---PDGK---------------S-E---S-LCMG-NSFGIIPTLDKQYMNGVVRTKVD--
-----D-CQFVCIAQEDYWRI-LNHVEKNTHKVEE-EGEIV----
>Rapgef6_[Scleropages_formosus]_938080831
AFANMTMSV---RRELCTVMVFEVVERAGTV---ILHDRQELDL---WYVILNGSVEVIH
---TDGR---------------A-E---V-LCMG-NSFGISPSLDKQYMDGEVRTKGD--
-----D-CQFVCIAQEDYWRI-LNHVEKNMHKVEE-EGEIVMV--
>Rapgef6_[Serinus_canaria]_683916584
AFANMTMSV---RRELCSVMIFEVVEQAGAI---ILEDGQELDS---WYVILNGTVEISY
---PDGK---------------S-E---S-LCMG-NSFGITPSLDKQYMNGVVRTKVD--
-----D-CQFVCIAQQDYWRI-LNHVEKNTHKVEE-EGEIV----
>Rapgef6_[Sinocyclocheilus_anshuiensis]_1024970981
AFANMTMSV---RRELCRVMMFEVVEQEGTI---ILQDKQELDL---WYVILNGAVEISY
---GDGR---------------T-E---I-LCMG-NSFGISPSLDRQFMNGVVCTKGD--
-----D-CQFVCIAQEDYCRI-LNHVEKNTHKVEE-EGEIVMV--
>Rapgef6_[Sinocyclocheilus_grahami]_1020491327
AFANMTMSV---RRELCRVMMFEVVEQEGTI---ILQDKQELDL---WYVILNGSVEISY
---GDGR---------------T-E---I-LCMG-NSFGISPSLDRQFMNGVVCTKGD--
-----D-CQFVCIAQEDYCRI-LNHVEKNTHKVEE-EGEIVMV--
>Rapgef6_[Sinocyclocheilus_rhinocerous]_1025412073
AFANMTMSV---RRELCRVMMFEVVEQEGTI---ILQDKQELDL---WYVILNGAVEISY
---GDGR---------------T-E---I-LCMG-NSFGISPSLDRQFMNGVVCTKGD--
-----D-CQFVCIAQEDYCRI-LNHVEKNTHKVEE-EGEIVMV--
>Rapgef6_[Sorex_araneus]_505829179
AFANMTMSV---RRELCSVMIFEVVEQAGAI---ILEDGQELDS---WYVILNGTVEISH
---PDGR---------------V-E---N-LFMG-NSFGITPTLDKQYMHGVVRTKVD--
-----D-CQFVCIAQQDYCRI-LNHVEKNTHKVEE-EGEIV----
>Rapgef6_[Struthio_camelus_australis]_697433805
AFANMTMSV---RRELCSVMIFEVVEQAGAI---ILEDGQELDS---WYVILNGTVEISY
---PDGK---------------S-E---S-LCMG-NSFGITPSLDKQYMNGVVRTKVD--
-----D-CQFVCIAQQDYWRI-LNHVEKNTHKVEE-EGEIV----
>Rapgef6_[Sturnus_vulgaris]_959086177
AFANMTMSV---RRELCSVMIFEVVEQAGAI---ILEDGQELDS---WYVILNGTVEISY
---PDGK---------------S-E---S-LCMG-NSFGITPSLDKQYMNGVVRTKVD--
-----D-CQFVCIAQQDYWRI-LNHVEKNTHKVEE-EGEIV----
>Rapgef6_[Sus_scrofa]_927105974
AFANMTMSV---RRELCSVMIFEVVEQAGAI---ILEDGQELDS---WYVILNGTVEISH

---PDGK---------------V-E---N-LFMG-NSFGVTPTLDKQYMHGVVRTKVD--
-----D-CQFVCIAQQDYWRI-LNHVEKNTHKVEE-EGEIV----
>Rapgef6_[Taeniopygia_guttata]_823480934
AFANMTMSV---RRELCSVMIFEVVEQAGAI---ILEDGQELDS---WYVILNGTVEISY
---PDGK---------------S-E---S-LCMG-NSFGITPSLDKQYMNGVVRTKVD--
-----D-CQFVCIAQQDYWRI-LNHVEKNTHKVEE-EGEIV----
>Rapgef6_[Takifugu_rubripes]_768939119
AFANMTMSV---RRELCSVMVFEVVEQAGTV---ILHNKQELDL---WYVILNGAVEFSY
---PDGR---------------M-E---T-LCMG-NSFGISPSLDKQYMNGEVRTKGD--
-----D-CQFVCIAQEDYWRI-LNHVEKNTHKVEE-EGEIV----
>Rapgef6_[Thamnophis_sirtalis]_927123537
AFANMTMSV---RRELCSVMIFEVVEQAGAI---ILEDGQELDS---WYVILNGTVEISY
---PDGK---------------T-E---S-LCMG-NSFGITPSLEKQHMNGVVRTKVD--
-----D-CQFVCIAQQDYWRI-LNHVEKNTHKVEE-EGEIV----
>Rapgef6_[Tinamus_guttatus]_697413352
AFANMTMSV---RRELCSVMIFEVVEQAGAI---ILEDGQELDS---WYVILNGTVEISY
---PDGK---------------S-E---S-LCMG-NSFGITPSLDKQYMNGMVRTKVD--
-----D-CQFVCIAQQDYWRI-LNHVEKNTHKVEE-EGEIV----
>Rapgef6_[Trichechus_manatus_latirostris]_471406079
AFANMTMSV---RRELCSVMIFEVVEQAGAI---ILEDGEELDS---WYVILNGTVEISH
---PDGK---------------I-E---N-LFMG-NSFGITPSLDKQYMHGVVRTKVD--
-----D-CQFVCIAQQDYWRI-LNHVEKNTHKVEE-EGEIV----
>Rapgef6_[Tyto_alba]_678189604
AFANMTMSV---RRELCSVMIFEVVEQAGAI---ILEDGQELDS---WYVILNGTVEISY
---PDGK---------------S-E---S-LCMG-NSFGITPSLDKQYMNGVVRTKVD--
-----D-CQFVCIAQQDYWRI-LNHVEKNTHKVEE-EGEIV----
>Rapgef6_[Vicugna_pacos]_560981325
AFANMTMSV---RRELCSVMIFEVVEQAGAV---ILEDGQELDS---WYVILNGTVEISH
---PDGK---------------V-E---N-LFMG-NSFGITPTLDKQYMRGVVRTKVD--
-----D-CQFVCIAQQDYWRI-LNHVEKNTHKVEE-EGEIV----
>Rapgef6_[Xiphophorus_maculatus]_941798194
AFANMTMSV---RRDLCSVMVFEVVEQSGTV---ILHNKQELDH---WYVILNGAVEISH
---AESR---------------T-E---T-LCMG-NSFGISPSLNKQYMNGEVRTKGD--
-----D-CQFVCIAQEDYWRI-LNHVEKNTHKVEE-EGEIVMV--

>Supplementary data 3: Sequence alignment of GEF of RAPGEF1-6.
>Rapgef0_[Absidia_glauca]_1021066824
---ELARQLTVMDSKLYSRIKPV----ECL-----DKNWG-RADS--Q-H-----TAA--
-------------------NVKASIEYSNQVTAWVTDSILSKEELKKRSAVVKHWIYVA-
EKCRL-LHNYNTCMAILSAFDNSSVGRLKRTWETMSARTTAILTGIRRLMGANRNFTEYR
DIIHK--VNP-------PCIPF-LGIYLQDLTFI-------------------------
-----EDGNS--NFLKKT----N---NLINFA-KRMKTGEVI---RELQQYQSTPYIL--
-----QM---------------VPDIQEFIKTHLQSSRDEETLYNLSLA---VEPR
>Rapgef0_[Acanthamoeba_castellanii_str._Neff]_470420624
--EELARQLTLMEFETFRAIR----PSELL-----NQVWN-KPK-----Q--RH-RAP--
-------------------NVVKMIRRFNEISNWVATSIVGSEKIRQRVKVMTKFLRLA-
DILRK-MNNFNTMVAVVAGINASAVHRLKWTKEEVMKGIWPQFAECERLMSNEGSYKTYR
GALFQ--ARP-------PCLPY-LGVYLTDLTFI-------------------------
-----EDGNP--DYV-------N---ELINFS-KRSLI---------------------

>Rapgef0_[Acytostelium_subglobosum_LB1]_831776467
--EEVARQLTLIDFEIFSSIK----PSELL-----NQSWN-KPK-----L--RH-RSP--
-------------------NVMLIINRFNEISQWVASIILSFDKVKDRARVMSKIVKIA-
EYLMKPLNNFNTSMAILSGLNAASVHRLKFTKEEMPKHIQVTLADLQGQLSSAQAYKTYR
DILAK--SNP-------PCLPY-LGVCLTDLTFI-------------------------
-----EEGNP--DLI-------K---GFINFS-KRKLIYNAI---FTVQSFQNTR-----
------------------YN------------------------------------
>Rapgef0_[Aedes_aegypti]_157125916
-TKDVAYHMTLFDWDLFWAVHEY----ELL-----YHTFG-RHHF--N-K-----ITS--
-------------------NLDVFIRRFNEIQYWVVTEIVSTSNLTKRMSLVKKFIKLA-
AFCKE-YQNLNAFFAIVMGLSNMAVSRLSQTWDKLPSKFRKLFTEYEALIDPSRNHRAYR
MSVGK--LQP-------PVIPF-MPLLLKDMTFA-------------------------
-----HEGNK--TSL-------D---GLVNFE-KMHMMAQTM---RTVRYCRSRHLVL-D
PPSP-KN---------------ENEIRQYISC-FRTIDNQRVLTAMSQK---VEPR
>Rapgef0_[Aedes_albopictus]_1000202901

MSVGK--LQP-------PVIPF-MPLLLKDMTFA-------------------------
-----HEGNK--TSL-------D---GLVNFE-KMHMMAQTM---RTVRYCRSRHLVLDP
-PSP-KN---------------ENEIRQYISC-FRTIDNQRVLTAMSQK---VEPR
>Rapgef0_[Ancylostoma_ceylanicum]_597883026
GSHELAQQLLVLHTQLFEATDEI----ELV-----TQVIG-RDQF--PGR-----VPS--
-------------------NLDLLMRRFNEVQYWATTEVLLA-LPQKRVNTLRKFIKIA-
MYAKE-NRDLMTLFAITLGLSNIAVSRLTHLWERLPAKLRRQFAEFESLLDPSRNHRPYR
ALVAK--MSP-------PLIPF-VPLLLKDLTFI-------------------------
-----HEGNK--TYY-------N---GLVNFE-KM------------------------

>Rapgef0_[Ancylostoma_duodenale]_748380306
NAQVVAAQLTLQDFAVFASIEPT----EYI-----DNLFQ-L-ES----R-YGS-P----
-------------------RLEEFERIFNREMWWVATEVCSERNVQKRAKLIKKFIKVA-
RHCRE-LRNFNSMFAIMSGLDKPAVRRLHSTWERISGKYIRMLEDVQQLVDPSRNMSKYR
QHLAEVSQEP-------PVVPI-YPVIKKDLTFS-------------------------
-----HEGNP--TYC-------D---KLVNFE-KLRLIAKSV---RAVSKLSSAPYEISS
MAER--
>Rapgef0_[Anopheles_darlingi]_568252345
--TS--
-------------------NLDVFIRRFNEIQYWVVTEIVSTTNLTKRMSLVKKFIKLA-
AFCKE-YQNLNAFFAIVMGLSNTAVSRLSLTWDKLPSKFRKLFTEYEALIDPSRNHRAYR

MSVGK--LQP-------PVIPF-MPLLLKDMTFA-------------------------
-----HEGNK--TSL-------D---GLVNFE-KMHMMAQTM---RTVRFCRSRHLVL-D
PPSP-KN---------------ENEIRNYIRC-FRTIDNQRVLNSMSQK---VEPR
>Rapgef0_[Anopheles_sinensis]_668451108
STKDVAYHMTLFDWDLFWAVHEY----ELL-----YHTFG-RHHF--N-K-----ITS--
-------------------NLDVFIRRFNEIQYWVVTEIVSTTNMTKRMSLVKKFIKLA-
AFCKE-YQNLNAFFAIVMGLSNTAVSRLSGTWDKLPSKFRKLFTEYEALIDPSRNHRAYR
MSVGK--LQP-------PVIPF-MPLLLKDMTFA-------------------------
-----HEGNK--TSL-------D---GLVNFE-KMHMMAQTM---RAVRFCRSRHLVL-D
PPSP-KN---------------ENEIRNYIRC-FRTIDNQRVLNSMSQK-------
>Rapgef0_[Batrachochytrium_dendrobatidis_JAM81]_575477186
---EVAQQLCIHNSGIFRSIQPI----EFL-----NEIWS-G-DI----D-----SSP--
-------------------SFKFFVERFDKESYWTATELVKVKDLKKRTLILKKFIQLI-
KESLS-LNNFFTTFSLIAGLNLTPVQRLKKTWEALPEKSKKLWSEVEKIADPSKNMKIYR
DQLAA--SVP-------PMVPF-LPIYLKDLTFI-------------------------
-----NDGNQ--SKA----------RGMINIE-KLRMMSS--------------------

>Rapgef0_[Batrachochytrium_dendrobatidis_JEL423]_1028569995
---EVAQQLCIHNSGIFRSIQPI----EFL-----NEIWS-G-DI----D-----SSP--
-------------------SFKFFVERFDKESYWTATELVKVKDLKKRTLILKKFIQLI-
KESLS-LNNFFTTFSLIAGLNLTPVQRLKKTWEALPEKSKKLWSEVEKIADPSKNMKIYR
DQLAA--SVP-------PMVPF-LPIYLKDLTFI-------------------------
-----NDGNQ--SKA----------RGMINIE-KLRMMSS--------------------

>Rapgef0_[Branchiostoma_floridae]_260811986
SSREMAYQMTLYDWELFNCVHEN----ELI-----YHIFG-RHKF--G-K-----ITS--
-------------------NLDLLLRRFNEVQFWVVTEMCLTPQVGKRVQLLRKFIKIA-
AYCKE-YQNLNAFFAIIMGLGNIAVSRLSQTWERLPNKFKRMFAEFESFMDPSRNHRVYR
LAMSK--LQP-------PIIPF-IPLLMKDMTFT-------------------------
-----HEGNK--TYY-------D---GLVNFE-KMHMISSTI---RTIRQCGSEPFNFD-
PPPVVKN---------------IQEVRNYVRS-LNVIDNQRTLTQLSHK---LEPR
>Rapgef0_[Caenorhabditis_brenneri]_341877847
--------------------------ELL-----YQVIG-RESF--PLS-----MPF--
-------------------NLDLLVRRFNEIQHWSTTEILLA-SEENRIEILKKFITIA-
TIARE-YRDLLTVFAITLGLSHTSVSRLTLTWSKLPPVTLKTFSELENLLDPTRNHRMYR
LMVSK--MTS-------PYIPF-VPLILKDLMFI-------------------------
-----HQGNK--SFY-------N---GLVNFE-KMHMFAKIF---RSFRQCKT--Q----

>Rapgef0_[Calliphora_vicina]_158935664
STKELAYYITTFDWDLFWAVHEY----ELL-----YHTFG-RHHF--G-K-----ITA--
-------------------NLDVFLRRFNELQYWIVTEIVSAASMSKRVGLLRKFIKLA-
AYCKE-YQNLNAFFAIVMGLSNMAVSRLQQTWEKIPSKFRKLYQEFEALIDPSRNHRAYR
VFVGK--LQP-------PLIPF-MPLLLKDMTFA-------------------------
-----HEGNK--TSL-------D---GLVNFE-KMHMMAQTM---RTLRFCRSRSLGL-E
PPSP-KS---------------EGEVRSFISC-LRVI-------------------
>Rapgef0_[Capitella_teleta]_443707267
SSREVAYQLTLYDWELFNCVHEY----ELI-----YLVFG-RQNF--G-R-----ITA--
-------------------NLDIFLRRFNEVQYWVASEMCLTQPVNKRVQLLRKLIKIA-
AYCKE-MQNLNSFFAIAMGLSNIAVSRLSQTWEKLPNKFKKIFADFEMSMDPSRNHRVYR
LSVAK--MQP-------PIVPF-MPLLMKDMTFT-------------------------
-----HEGNK--THF-------D---GLINFE-KMHMIAQTL---RTIRFCRSKSLDLE-
PPQSLKN---------------SQEVKSYART-LKVIDNQRVLTQLSHK---LEPR
>Rapgef0_[Cyprinus_carpio]_966662070
SSKDLAFQMTQYDWELFSCVHEY----ELV-----YHTFG-RQAY--R-R-----STA--

```
-------------------NLELFLKRFNQVQLWVVTEVCLCGTLSKRVQLLKKFIKIA-
AHCRE-FKNLNSFFAIIMGMCNPAVSRLSQTWEKLPSKFKKFYSEFESFLDPSRNHRAYR
LTVAK--MEP-------PIIPF-MPLLIKDMTFT--------------------------
-----HEGNK--TFV-------D---GLVNFE-KMRLIANTI---RAVRHCRSQLFS---
--------------------------------------------------------
>Rapgef0_[Danaus_plexippus]_357608300
---EVAVQLTLQDFCIFRQIEST----EYV-----DDLFD-L-KS----R-YGT-PM---
--------------------LSQFAGLVNKEMFWVVTEVVNEQNIVRRSKIIKQFIKVA-
RHCKE-CKNFNSMFAIISGLGHGAVSRLRMTWEKLPTKYNKLFSDLQMLMDPSRNMSKYR
QLVTIEQGRS-------PVIPF-YPVVRKDLTFI--------------------------
-----HLGND--TKV-------E---GMVNFE-KLRMIAKEV---RTLTNMCSSPYD---
--------------------------------------------------------
>Rapgef0_[Daphnia_pulex]_321471019
STRELAYYITLIDWDLFCSVHEY----ELL-----YHVAG-AQPF--R-K-----IKS--
-------------------NLDLFLRRFNEIQYWVVTEICLANTLGKRVQLLRKFIKLA-
AYCKE-FQNLNAFFALVMGLSNVAVSRLTQTWERLPSKLRKMFTEFDGLIEPSRNHRAYR
IAVGK--LQP-------PILPF-MPLLLKDMTFT--------------------------
-----HEGNR--TLL-------DS-AGLINFE-KMHMLAQTM---RTLRYCRSRQLLL-Q
PPTP-RS---------------EQEVRNYIRN-LRVIDNQRILTGLSQR---LEPK
>Rapgef0_[Dictyocaulus_viviparus]_768188889
GSYELAQQLYYLHTQLFEATDEI----ELV-----TQVIG-RDQF--PGR-----IPS--
-------------------NLDLLMRRFNEVQYWATTEVLLA-SPQKRVHTLRKFIKIA-
LYAKE-NRDLMTLFAITLGLSNVAVSRLTNLWDRLPNKLRRQFAEFESLLNPSRNHRPYR
ALVSK--MSP-------PMIPF-VPLLLKDLTFI--------------------------
-----HEGNK--TYY-------N---GLVNFE-KMHMIANIL---RSFRQCKS-RCTGSQ
VESK--K---------------ISETQNFIRN-FCVVDNQR---------------
>Rapgef0_[Dictyostelium_fasciculatum]_470247515
--EEIARQLTLIDFEMFAAIK----PSELL-----NQSWN-KPK-----L--RH-RSP--
-------------------NVLALISRFNEISSWTASMILNHDKVKDRARVMAKFVKIG-
EFLLKQLNNYNTAMAILSGLNQSAIHRLKFTREEMPKAVQQSYTDLQAQLSNAFSYKVYR
ELLAK--ANP-------PLLPY-LGVCLTDLTFI--------------------------
-----EDGNP--DFI-------G---NLINFS-KRRLV----------------------
--------------------------------------------------------
>Rapgef0_[Dictyostelium_purpureum]_330795492
--EEIARQLTLMDFEIFSNIK----STELL-----NQSWN-KPK-----L--RY-RSP--
-------------------NVLTLINRFNEISQWTATSILSYERVKDRARIMAKFIRIA-
EYSMKLLNNFNTSMAILSGLNASSVHRLKFTKEEMPKHTQQVYAELQQQLSSSQSYKEYR
ALLAK--ANP-------PCLPY-LGVYLTDLTFF--------------------------
-----EEGNP--DFI-------Q---GYINFG-KRKLIYGSI---SNVQSFQNAK-----
------------------YN------------------------------------
>Rapgef0_[Drosophila_busckii]_924553109
---ELAIQLTLQDFANFRQIEST----EYI-----DELFE-L-QS----K-YGV-PM---
--------------------LSKFSELVNREMFWVVSEICGEHNIVRRMKIVKQFIKIA-
RHCKE-CRNFNSMFAIISGLGHGAVSRLRLTWEKVPSKYQRLFNDLQDLMDPSRNMSKYR
QLVSAELLAQH------PIIPF-YPIVKKDLTFI--------------------------
-----HLGND--TRV-------D---GLINFE-KLRMLSKEV---RLLTHMCSSPYD---
--------------------------------------------------------
>Rapgef0_[Drosophila_erecta]_968029732
STKELAYHITLFEWDLFWAVHEY----ELL-----YHTFG-RHHF--G-K-----ITA--
-------------------NLDVFLRRFNEVQYWIVTELVSTPSLSKRVGLVRKFIKLA-
AYCKE-YQNLNAFFAVVMGLSNMAVSRLQQTWEKIPSKFRKIFQEFEALIDPSRNHRAYR
VFVGK--LQP-------PLIPF-MPLLLKDMTFA--------------------------
-----HEGNK--TSL-------D---GLVNFE-KMHMMAQTM---RTIRFCRSRSLGL-E
PPSP-KS---------------EGEVRSYISS-FRVIDNQRVLTAMSQK---VEP-
```

>Rapgef0_[Drosophila_melanogaster]_24586077
STKELAYHITLFEWDLFWAVHEY----ELL-----YHTFG-RHHF--G-K-----ITA--
-------------------NLDVFLRRFNEVQYWIVTELVSTPSLSKRVGLVRKFIKLA-
AYCKE-YQNLNAFFAVVMGLSNMAVSRLQQTWEKIPSKFRKIFQEFEALIDPSRNHRAYR
VFVGK--LQP-------PLIPF-MPLLLKDMTFA--------------------------
-----HEGNK--TSL-------D---GLVNFE-KMHMMAQTM---RTIRFCRSRSLGL-E
PPSP-KS---------------EGEVRSYISS-FRVIDNQRVLTAMSQK---VEP-
>Rapgef0_[Drosophila_mojavensis]_968046798
STKELAYHITIFEWDLFWAVHEY----ELL-----YHTFG-RHHF--G-K-----ITA--
-------------------NLDVFLRRFNEVQYWIVTELVSTPSLSKRVGLVRKFIKLA-
AYCKE-YQNLNAFFAIVMGLSNMAVTRLHQTWEKIPSKFRKIFQEFEALIDPSRNHRAYR
VFVGK--LQP-------PLIPF-MPLLLKDMTFA--------------------------
-----HEGNK--TSL-------D---GLVNFE-KMHMMAQTM---RTIRFCRSRSLGL-E
PPSP-KS---------------EGEVRSYISS-FRVIDNQRVLTAMSQK---VEP-
>Rapgef0_[Drosophila_persimilis]_195149149
STKELAYHITLFEWDLFYAVHEY----ELL-----YHTFG-RHHF--G-K-----ITA--
-------------------NLDVFLRRFNEVQYWIVTELVSTPSLSKRVGLVRKFIKLA-
AYCKE-YQNLNAFFAVVMGLSNMAVSRLQQTWEKIPSKFRKIFQEFEALIDPSRNHRAYR
VFVGK--LQP-------PLIPF-MPLLLKDMTFA--------------------------
-----HEGNK--TSL-------D---GLVNFE-KMHMMAQTM---RTIRFCRSRSLGL-E
PPSP-KS---------------EGEVRSYISS-FRVIDNQRVLTAMSQK---VEP-
>Rapgef0_[Drosophila_pseudoobscura_pseudoobscura]_198455898
STKELAYHITLFEWDLFYAVHEY----ELL-----YHTFG-RHHF--G-K-----ITA--
-------------------NLDVFLRRFNEVQYWIVTELVSTPSLSKRVGLVRKFIKLA-
AYCKE-YQNLNAFFAVVMGLSNMAVSRLQQTWEKIPSKFRKIFQEFEALIDPSRNHRAYR
VFVGK--LQP-------PLIPF-MPLLLKDMTFA--------------------------
-----HEGNK--TSL-------D---GLVNFE-KMHMMAQTM---RTIRFCRSRSLGL-E
PPSP-KS---------------EGEVRSYISS-FRVIDNQRVLTAMSQK---VEP-
>Rapgef0_[Drosophila_sechellia]_195331917
STKELAYHITLFEWDLFWAVHEY----ELL-----YHTFG-RHHF--G-K-----ITA--
-------------------NLDVFLRRFNEVQYWIVTELVSTPSLSKRVGLVRKFIKLA-
AYCKE-YQNLNAFFAVVMGLSNMAVSRLQQTWEKIPSKFRKIFQEFEALIDPSRNHRAYR
VFVGK--LQP-------PLIPF-MPLLLKDMTFA--------------------------
-----HEGNK--TSL-------D---GLVNFE-KMHMMAQTM---RTIRFCRSRSLGL-E
PPSP-KS---------------EGEVRSYISS-FRVIDNQRVLTAMSQK---VEP-
>Rapgef0_[Drosophila_simulans]_1013905440
STKELAYHITLFEWDLFWAVHEY----ELL-----YHTFG-RHHF--G-K-----ITA--
-------------------NLDVFLRRFNEVQYWIVTELVSTPSLSKRVGLVRKFIKLA-
AYCKE-YQNLNAFFAVVMGLSNMAVSRLQQTWEKIPSKFRKIFQEFEALIDPSRNHRAYR
VFVGK--LQP-------PLIPF-MPLLLKDMTFA--------------------------
-----HEGNK--TSL-------D---GLVNFE-KMHMMAQTM---RTIRFCRSRSLGL-E
PPSP-KS---------------EGEVRSYISS-FRVIDNQRVLTAMSQK---VEP-
>Rapgef0_[Drosophila_virilis]_968111026
STKELAYHITIFEWDLFWAVHEY----ELL-----YHTFG-RHHF--G-K-----ITA--
-------------------NLDVFLRRFNEVQYWIVTELVSTPSLSKRVGLVRKFIKLA-
AYCKE-YQNLNAFFAIVMGLSNMAVTRMHQTWEKIPSKFRKIFQEFEALIDPSRNHRAYR
VFVGK--LQP-------PLIPF-MPLLLKDMTFA--------------------------
-----HEGNK--TSL-------D---GLVNFE-KMHMMAQTM---RTIRFCRSRSLGL-E
PPSP-KS---------------EGEVRAYISS-FRVIDNQRVVTAMSQK---VEP-
>Rapgef0_[Drosophila_yakuba]_969526469
STKELAYHITLFEWDLFWAVHEY----ELL-----YHTFG-RHHF--G-K-----ITA--
-------------------NLDVFLRRFNEVQYWIVTELVSTPSLSKRVGLVRKFIKLA-
AYCKE-YQNLNAFFAVVMGLSNMAVSRLQQTWEKIPSKFRKIFQEFEALIDPSRNHRAYR
VFVGK--LQP-------PLIPF-MPLLLKDMTFA--------------------------

```
-----HEGNK--TSL-------D---GLVNFE-KMHMMAQTM---RTIRFCRSRSLGL-E
PPSP-KS---------------EGEVRTYISS-FRVIDNQRVLTAMSQK---VEP-
>Rapgef0_[Lottia_gigantea]_676496085
STKELAYQITLYDWELFNALHEF----ELI-----YLVFG-RHRF----N---K-ITA--
-------------------NLDLFLRRFNEIQYWVATEMVLCSNVSKRVQLLKKFIKLA-
AICKE-NKNLHSFFAIVMGLSNIAVSRLSQTWEKLPGKFNKLFAEFETVMDPSRNHRVYR
LTVAK--MNP-------PILPF-MPLLMK-------------------------------
------------------------------------------------------------
--------------------------------------------------------
>Rapgef0_[Naegleria_gruberi]_290987038
--QEVARQMSLIDYTMFKKIEPK----ECL-----NQAWN-K-EH----RVTKA-P----
-------------------NISRMIQHFNQFSGFVATEILKQEDHEKRVKCVEKFIELA-
NHCKG-LNNFNAVFSVMSGLNSSSIHRLSKTWEAISEEAKKTREELLSITNTNGNFANLR
NMLKTVN--P-------PCVPY-NGVFLTDLTFI--------------------------
-----EDGSP--KYI-------N---GLINFG-KCRLFAKVI---RDIQ-----------
--------------------------------------------------------
>Rapgef0_[Necator_americanus]_915250685
NAQVVAAQLTLQDFAVFASIEPT----EYI-----DNLFQ-L-ES----R-YGS-P----
-------------------RLEEFERIFNREMWWVATEVCSERVIQKRAKLIKKFIKVA-
RYCRE-LRNFNSMFAIMSGLDKPAVRRLHSTWERISGKYIRMLEDIQQLVDPSRNMSKYR
QHLAEVSQEP-------PVVPI-YPVIKKDLTFS--------------------------
-----HEGNP--TYC-------D---KLVNFE-KLRLIAKSV---RAVSKLSSAPYEISS
MAER----------------------------------------------------
>Rapgef0_[Neotoma_lepida]_1040239113
---DLALELMSFDWSLFNSIHEQ----ELI-----YFTFS-RQGS--G-E-----HTV--
-------------------NLSLLLQRCNEVQLWVATEILLCSQLGKRVQLVKKFIKIA-
AHCKA-QQNLNSFFAIVMGLNTASVSRLSQTWEKIPGKFKKLFSELESLTDPSLNHKAYR
DAFKK--MKP-------PKIPF-MPLLLKDVTFI--------------------------
-----HEGNK--TFL-------D---NLVNFE-KLHMIADTV---RTLRHCRSNQFGSDM
SPKE------------------QQELKSYVNH-LYVIDSQQALFELSHR---LEP-
>Rapgef0_[Oikopleura_dioica]_313238569
---ELAFQLTIRDYNIFRNVLPT----DYV-----NDIFH-RIGG----QKDPS-HIK--
-------------------EMSALESLVNEEMFWVVHEVLQETNLHRRANSLKQFIQTC-
IHLKE-LKNYNSLFAIISGLDHTAVARLKQTWAAVSPKNRKKFEELKSLMEPSRNFSTYR
-SLKQKNEVP-------PLIPF-VPVIKKDLTFI--------------------------
-----HLGND--TKV-------D---GLINFE-KMRDFAREI---RTLRKYC--------
--------------------------------------------------------
>Rapgef0_[Oncorhynchus_mykiss]_642080378
SSKDLAYQMTLYDWELFSCVHEH----ELL-----YHTFG-RQSF--R-R-----TTA--
-------------------NLDLFLRRFNQVQLWVVTEVCLCGQLSKRVQLLKKFIKIA-
AHCRE-FKNLNSFFAIIMGMSNPAVSRLTQTWEKLPSKFKKFYAEFESMMDPSRNHRAYR
LTVTK--IEP-------PIIPF-MPLLLKDMTFS--------------------------
-----HEGNK--TFI-------D---NMVNFE-KMRVIANTI---RAVRQCRSQPFS---
--------------------------------------------------------
>Rapgef0_[Pediculus_humanus_corporis]_242021899
STKELAYHMTHFDWELFCCINEY----EML-----YHVIG-KHHF---GE---L--TA--
-------------------NLEMFLRRFNEIQYWVATEILHTQSLSKRVTVLRKLIKLA-
SYCKE-YQNFNAFLAIITGLNNQAVSRLSLTWEKLPNKFRKMFTEFEILIDSSRNHRAYR
AAVGK--LQP-------PVLPF-MPLLIKDMNCA--------------------------
-----QENHK--TFT-------D---GLVNFE-KMHLFAQTI---RTMRYSRSRHLTLEP
PSPKSEG-----------------NIRSYISC-LRVIDNQKQLMSLSQK---LEPR
>Rapgef0_[Spizellomyces_punctatus_DAOM_BR117]_1026975868
SVEDLSQQLCIHNFKLFHNIHPI----EYL-----NQIWQ-K-TN----E-----SSP--
-------------------SMIYFIERFDKESYWASTEILKEKDLKKRTSLLRKFILTA-
```

KMCQE-LSNFFTMFALIAGLNMPPIQRLKKTWEALPDKAKKAWAELEKVADPSRNMKNYR
DLLNA--ATP-------PIVPF-LPIYLKDLTFM-------------------------
-----NDGNE--SKI-------GTERQMINFD-KLRMMGNRV---KDI------------

>Rapgef0_[Trichuris_suis]_669314000
SSAELAQHLLIYHWQLFHNTHEY----ELL-----YQTIG-RNRF--PGR-----LPV--
-------------------NLDLFVRRFNELQFWVITEILLCTALSKRAHLVKKFIKIA-
LHAKN-SHDLFSFFAITLGLSNVAISRLSQTWEKVSTKFRKQFFEFEGLLDPSRNHRAYR
LLVAK--MSP-------PVIPF-IPLLLKDLLFI-------------------------
-----HEGNK--TSF-------E---GMVNFE-KMHMMAHTL---RNLRAYKAHAPAID-
---ASKV---------------SPEAQHLVHN-FRVIDDQRKFIQMSKA---LEPR
>Rapgef0_[Wuchereria_bancrofti]_402583412
SSIDIAQQLLIFHTQLLEATDDI----ELI-----TQVFG-REQF--PNR-----ITS--
-------------------NLDLLMRRFNEVQFWTTTEVLLAQSNTKRLSILKKFIKIA-
AHAKE-NKDLLSLFAIILGLSNIAVSRITHLWDKLPSKMKQQYAEFEELLDPCRNHRAYR
MLTAN--MSA-------PTVPF-IPLLLKXLTFT-------------------------
-----HEGNK--T--

>Rapgef1_[Acanthisitta_chloris]_677291549
-SHEIAEQLTLLDAELFYKIE---IPEVLL--------WA-KEQ-----N--EE-KSP--
-------------------NLTQFTEHFNNMSYWVRSIIMLQEKAQDRERLLLKFIKIM-
KHLRK-LNNFNSYLAILSALDSAPIRRLEWQKQTS-----EGLAEYCTLIDSSSSFRAYR
AALAD--VEP-------PCIPY-LGLILQDLTFV-------------------------
-----HLGNP--DYI-------D---SKVNFS-K-------------------------

>Rapgef1_[Acinonyx_jubatus]_961760290
-SHEIAEQLTLLDAELFYKIE---IPEVLL--------WA-KEQ-----N--EE-KSP--
-------------------NLTQFTEHFNNMSYWVRSIIMLQEKAQDRERLLLKFIKIM-
KHLRK-LNNFNSYLAILSALDSAPIRRLEWQKQTS-----EGLAEYCTLIDSSSSFRAYR
AALSE--VEP-------PCIPY-LGLILQDLTFV-------------------------
-----HLGNP--DYI-------D---GKVNFS-KRWQQFNIL---DSMRCFQ--------

>Rapgef1_[Ailuropoda_melanoleuca]_752388953
-SHEIAEQLTLLDAELFYKIE---IPEVLL--------WA-KEQ-----N--EE-KSP--
-------------------NLTQFTEHFNNMSYWVRSIIMLQEKAQDRERLLLKFIKIM-
KHLRK-LNNFNSYLAILSALDSAPIRRLEWQKQTS-----EGLAEYCTLIDSSSSFRAYR
AALSE--VEP-------PCIPY-LGLILQDLTFV-------------------------
-----HLGNP--DYI-------D---GKVNFS-KRWQQFNIL---DSMRCFQQAH-----
------------------YDIRRNDDIINFFNDFSDHLAEEALWELSLK---IKPR
>Rapgef1_[Alligator_mississippiensis]_951018134
-AEDIANHLTAFHWELFRCIHEL----EFV-----DYVFHGE-RG----R-RET-A----
-------------------NLELMLQRCSEVQHWVATEILLCEALGKRAHLLKKFVKIA-
AICKQ-NQDMLSFYAVVIGLNNAAVSRLRLTWEKLPGKFKNLFRKFENLTDPCRNHKTYR
EVLTK--MKP-------PIIPF-VPLILKDLTFL-------------------------
-----HEGSK--TLL-------D---GLVNIE-KLHSIAEKV---RTIRKYRS-------

>Rapgef1_[Alligator_sinensis]_557283170
---EIAEQLTLLDAELFYKIE---IPEVLL--------WA-KEQ-----N--EE-KSP--
-------------------NLTQFTEHFNNMSYWVRSIIMLQEKAQDRERLLLKFIKIM-
KHLRK-LNNFNSYLAILSALDSAPIRRLEWQKQTS-----EGLAEYCTLIDSSSSFRAYR
AALAE--VEP-------PCIPY-LGLILQDLTFV-------------------------
-----HLGNP--DYI-------D---GKVNFS-K-------------------------

>Rapgef1_[Anser_cygnoides_domesticus]_902922233

-SHEIAEQLTLLDAELFYKIE---IPEVLL--------WA-KEQ-----N--EE-KSP--
-------------------NLTQFTEHFNNMSYWVRSIIMLQEKAQDRERLLLKFIKIM-
KHLRK-LNNFNSYLAILSALDSAPIRRLEWQKQTS-----EGLAEYCTLIDSSSSFRAYR
AALAD--VEP-------PCIPY-LGLILQDLTFV--------------------------
-----HLGNP--DYI-------D---SKVNFS-K--------------------------

>Rapgef1_[Aquila_chrysaetos_canadensis]_768361773
-SHEIAEQLTLLDAELFYKIE---IPEVLL--------WA-KEQ-----N--EE-KSP--
-------------------NLTQFTEHFNNMSYWVRSIIMLQEKAQDRERLLLKFIKIM-
KHLRK-LNNFNSYLAILSALDSAPIRRLEWQKQTS-----EGLAEYCTLIDSSSSFRAYR
AALAD--VEP-------PCIPY-LGLILQDLTFV--------------------------
-----HLGNP--DYI-------D---SKVNFS-K--------------------------

>Rapgef1_[Ascaris_suum]_541042023
SSVDIAQQLSIFHTQLFEATDEI----ELV-----TQVFG-RDQF--PGR-----IPS--
-------------------NLDLLMRRFNEVQFWTTTEVLLAHGPSKRVAMLKKFIKIA-
AHAKE-NRDLMSLFAIILGLSNVAVSRITHIWDKLPSKMRRQYAEFEALLDPSRNHRAYR
MLVAK--MTS-------PTVPF-VPLLLKDLTFT--------------------------
-----HEGNK--TYF-------A---GLVNFE-KMHMIANVL---RSFRQCKS-KYPVTS
FDQK--K---------------VFESKNLIRN-FKVIDNQRRLMEISYQ---IEP-
>Rapgef1_[Balaenoptera_acutorostrata_scammoni]_594694590
-SHEIAEQLTLLDAELFYKIE---IPEVLL--------WA-KEQ-----N--EE-KSP--
-------------------NLTQFTEHFNNMSYWVRSIIMLQEKAQDRERLLLKFIKIM-
KHLKK-LNNFNSYLAILSALDSAPIRRLEWQKQTS-----EGLAEYCTLIDSSSSFRAYR
AALSE--VEP-------PCIPY-LGLILQDLIFL--------------------------
-----HLGNP--DYI-------D---GKVNFS-KRWQQFNIL---DSMRCFQQAH-----
------------------YDIRRNDDIINFFNDFSDHLAEEALWELSLK---IKPR
>Rapgef1_[Balearica_regulorum_gibbericeps]_676242958
-SHEIAEQLTLLDAELFYKIE---IPEVLL--------WA-KEQ-----N--EE-KSP--
-------------------NLTQFTEHFNNMSYWVRSIIMLQEKAQDRERLLLKFIKIM-
KHLRK-LNNFNSYLAILSALDSAPIRRLEWQKQTS-----EGLAEYCTLIDSSSSFRAYR
AALAD--VEP-------PCIPY-LGLILQDLTFV--------------------------
-----HLGNP--DYI-------D---SKVNFS-K--------------------------

>Rapgef1_[Bos_mutus]_440900078
---EIAEQLTLLDAELFYKIE---IPEVLL--------WA-KEQ-----N--EE-KSP--
-------------------NLTQFTEHFNNMSYWVRSIIMLQEKAQDRERLLLKFIKIM-
KHLRK-LNNFNSYLAILSALDSAPIRRLEWQKQTS-----EGLAEYCTLIDSSSSFRAYR
AALSE--VEP-------PCIPY-LGLILQDLTFV--------------------------
-----HLGNP--DYI-------D---GKVNFS-K--------------------------

>Rapgef1_[Bos_taurus]_982939844
---EIAEQLTLLDAELFYKIE---IPEVLL--------WA-KEQ-----N--EE-KSP--
-------------------NLTQFTEHFNNMSYWVRSIIMLQEKAQDRERLLLKFIKIM-
KHLRK-LNNFNSYLAILSALDSAPIRRLEWQKQTS-----EGLAEYCTLIDSSSSFRAYR
AALSE--VEP-------PCIPY-LGLILQDLTFV--------------------------
-----HLGNP--DYI-------D---GKVNFS-K--------------------------

>Rapgef1_[Buceros_rhinoceros_silvestris]_704153824
---EIAEQLTLLDAELFYKIE---IPEVLL--------WA-KEQ-----N--EE-KSP--
-------------------NLTQFTEHFNNMSYWVRSIIMLQEKAQDRERLLLKFIKIM-
KHLRK-LNNFNSYLAILSALDSAPIRRLEWQKQTS-----EGLAEYCTLIDSSSSFRAYR
AALAD--VEP-------PCIPY-LGLILQDLTFV--------------------------
-----HLGNP--DYI-------D---SKVNFS-K--------------------------

>Rapgef1_[Calypte_anna]_663253808
---EIAEQLTLLDAELFYKIE---IPEVLL--------WA-KEQ-----N--EE-KSP--
-------------------NLTQFTEHFNNMSYWVRSIIMLQEKAQDRERLLLKFIKIM-
KHLRK-LNNFNSYLAILSALDSAPIRRLEWQKQTS-----EGLAEYCTLIDSSSSFRAYR
AALAD--VDP-------PCIPY-LGLILQDLTFV--------------------------
-----HLGNP--DYI-------D---SKVNFS-K--------------------------

>Rapgef1_[Caprimulgus_carolinensis]_704288312
-SHEIAEQLTLLDAELFYKIE---IPEVLL--------WA-KEQ-----N--EE-KSP--
-------------------NLTQFTEHFNNMSYWVRSIIMLQEKAQDRERLLLKFIKIM-
KHLRK-LNNFNSYLAILSALDSAPIRRLEWQKQTS-----EGLAEYCTLIDSSSSFRAYR
AALAD--VEP-------PCIPY-LGLILQDLTFV--------------------------
-----HLGNP--DYI-------D---NKVNFS-K--------------------------

>Rapgef1_[Cariama_cristata]_698401251
---EIAEQLTLLDAELFYKIE---IPEVLL--------WA-KEQ-----N--EE-KSP--
-------------------NLTQFTEHFNNMSYWVRSIIMLQEKAQDRERLLLKFIKIM-
KHLRK-LNNFNSYLAILSALDSAPIRRLEWQKQTS-----EGLAEYCTLIDSSSSFRAYR
AALAD--VEP-------PCIPY-LGLILQDLTFV--------------------------
-----HLGNP--DYI-------D---SKVNFS-K--------------------------

>Rapgef1_[Carlito_syrichta]_640785364
---EIAEQLTLLDAELFYKIE---IPEVLL--------WA-KEQ-----N--EE-KSP--
-------------------NLTQFTEHFNNMSYWVRSIIMLQEKAQDRERLLLKFIKIM-
KHLRK-LNNFNSYLAILSALDSAPIRRLEWQKQTS-----EGLAEYCTLIDSSSSFRAYR
AALSE--VEP-------PCIPY-LGLILQDLTFV--------------------------
-----HLGNP--DYI-------D---GKVNFS-K--------------------------

>Rapgef1_[Ceratotherium_simum_simum]_955502909
-SHEIAEQLTLLDAELFYKIE---IPEVLL--------WA-KEQ-----N--EE-KSP--
-------------------NLTQFTEHFNNMSYWVRSIIMLQEKAQDRERLLLKFIKIM-
KHLRK-LNNFNSYLAILSALDSAPIRRLEWQKQTS-----EGLAEYCTLIDSSSSFRAYR
AALSE--VEP-------PCIPY-LGLILQDLTFV--------------------------
-----HLGNP--DYI-------D---GKVNFS-KRWQQFNIL---DSMRCFQQAH-----
------------------YDIRRNDDIINFFNDFSDHLAEEALWELSLK---IKPR
>Rapgef1_[Charadrius_vociferus]_699697996
-SHEIAEQLTLLDAELFYKIE---IPEVLL--------WA-KEQ-----N--EE-KSP--
-------------------NLTQFTEHFNNMSYWVRSIIMLQEKAQDRERLLLKFIKIM-
KHLRK-LNNFNSYLAILSALDSAPIRRLEWQKQTS-----EGLAEYCTLIDSSSSFRAYR
AALAD--VEP-------PCIPY-LGLILQDLTFV--------------------------
-----HLGNP--DYI-------D---SKVNFS-K--------------------------

>Rapgef1_[Chlamydotis_macqueenii]_705662410
---EIAEQLTLLDAELFYKIE---IPEVLL--------WA-KEQ-----N--EE-KSP--
-------------------NLTQFTEHFNNMSYWVRSIIMLQEKAQDRERLLLKFIKIM-
KHLRK-LNNFNSYLAILSALDSAPIRRLEWQKQTS-----EGLAEYCTLIDSSSSFRAYR
AALAD--VEP-------PCIPY-LGLILQDLTFV--------------------------
-----HLGNP--DYI-------D---SKVNFS-K--------------------------

>Rapgef1_[Chlorocebus_sabaeus]_635070519
-SHEIAEQLTLLDAELFYKIE---IPEVLL--------WA-KEQ-----N--EE-KSP--
-------------------NLTQFTEHFNNMSYWVRSIIMLQEKAQDRERLLLKFIKIM-
KHLRK-LNNFNSYLAILSALDSAPIRRLEWQKQTS-----EGLAEYCTLIDSSSSFRAYR

AALSE--VEP-------PCIPY-LGLILQDLTFV--------------------------
-----HLGNP--DYI-------D---GKVNFS-KRWQQFNIL---DSMRCFQQAH-----
------------------YDIRRNDDIINFFNDFSDHLAEEALWELSLK---IKPR
>Rapgef1_[Clupea_harengus]_831306567
---EVADQLTLLDAELFYKIE---IPEVLL--------WA-KEQ-----N--EE-KSP--
-------------------NLTQFTEHFNNMSYWVRSVIIQQERAQDREKLLLKFIKIM-
KHLRK-LNNFNSYLAILSALDSAPIRRLEWQKQTS-----EGLEEYCTLIDSSSSFRAYR
AALAD--VEP-------PCIPY-LGLILQDLTFV--------------------------
-----HLGNP--DLV-------E---GKVNFS-K--------------------------

>Rapgef1_[Colius_striatus]_706146434
-SHEIAEQLTLLDAELFYKIE---IPEVLL--------WA-KEQ-----N--EE-KSP--
-------------------NLTQFTEHFNNMSYWVRSIIMLQEKAQDRERLLLKFIKIM-
KHLRK-LNNFNSYLAILSALDSAPIRRLEWQKQTS-----EGLAEYCTLIDSSSSFRAYR
AALAD--VEP-------PCIPY-LGLILQDLTFV--------------------------
-----HLGNP--DYI-------D---SKVNFS-K--------------------------

>Rapgef1_[Columba_livia]_543744181
---EIAEQLTLLDAELFYKIE---IPEVLL--------WA-KEQ-----N--EE-KSP--
-------------------NLTQFTEHFNNMSYWVRSIIMLQEKAQDRERLLLKFIKIM-
KHLRK-LNNFNSYLAILSALDSAPIRRLEWQKQTS-----EGLAEYCTLIDSSSSFRAYR
AALAD--VEP-------PCIPY-LGLILQDLTFV--------------------------
-----HLGNP--DYI-------D---NKVNFS-K--------------------------

>Rapgef1_[Corvus_cornix_cornix]_727012745
-SHEIAEQLTLLDAELFYKIE---IPEVLL--------WA-KEQ-----N--EE-KSP--
-------------------NLTQFTEHFNNMSYWVRSIIMLQEKAQDRERLLLKFIKIM-
KHLRK-LNNFNSYLAILSALDSAPIRRLEWQKQTS-----EGLAEYCTLIDSSSSFRAYR
AALAD--VEP-------PCIPY-LGLILQDLTFV--------------------------
-----HLGNP--DYI-------D---SKVNFS-K--------------------------

>Rapgef1_[Cricetulus_griseus]_1032921614
---EIAEQLTLLDAELFYKIE---IPEVLL--------WA-KEQ-----N--EE-KSP--
-------------------NLTQFTEHFNNMSYWVRSIIMLQEKAQDRERLLLKFIKIM-
KHLRK-LNNFNSYLAILSALDSAPIRRLEWQRQTS-----EGLAEYCTLIDSSSSFRAYR
AALSE--VEP-------PCIPY-LGLILQDLTFV--------------------------
-----HLGNP--DYI-------D---GKVNFS-K--------------------------

>Rapgef1_[Danio_rerio]_688566951
---EIADQLTLLDAELFYKIE---IPEVLL--------WA-KEQ-----N--EE-KSP--
-------------------NLTQFTEHFNNMSYWVRSIIILQEKAQDREKLLLKFIKIM-
KHLRK-LNNFNSYLAILSALDSAPIRRLEWQKQTS-----EGLEEYCTLIDSSSSFRAYR
AALAD--VEP-------PCIPY-LGLILQDLTFV--------------------------
-----HLGNP--DHI-------E---GKINFS-K--------------------------

>Rapgef1_[Dasypus_novemcinctus]_488553799
-SHEIAEQLTLLDAELFYKIE---IPEVLL--------WA-KEQ-----N--EE-KSP--
-------------------NLTQFTEHFNNMSYWVRSIIMLQEKAQDRERLLLKFIKIM-
KHLRK-LNNFNSYLAILSALDSAPIRRLEWQKQTS-----EGLAEYCTLIDSSSSFRAYR
AALSE--VEP-------PCIPY-LGLILQDLTFV--------------------------
-----HLGNP--DYI-------D---GKVNFS-KRWQQFNIL---DSMRCFQQAH-----
------------------YDIRRNDDIINFFNDFSDHLAEEALWELSLK---IKPR
>Rapgef1_[Eptesicus_fuscus]_641722335
-SHEIAEQLTLLDAELFYKIE---IPEVLL--------WA-KEQ-----N--EE-KSP--

-------------------NLTQFTEHFNNMSYWVRSIIMLQEKAQDRERLLLKFIKIM-
KHLRK-LNNFNSYLAILSALDSAPIRRLEWQKQTS-----EGLAEYCTLIDSSSSFRAYR
AALSE--VEP-------PCIPY-LGLILQDLTFV--------------------------
-----HLGNP--DYI-------D---GKVNFS-KRWQQFNIL---DSMRCFQQAH-----
------------------YDIRRNDDIINFFNDFSDHLAEEALWELSLK---IKPR
>Rapgef1_[Equus_asinus]_958773773
---EIAEQLTLLDAELFYKIE---IPEVLL--------WA-KEQ-----N--EE-KSP--
-------------------NLTQFTEHFNNMSYWVRSIIMLQEKAQDRERLLLKFIKIM-
KHLRK-LNNFNSYLAILSALDSAPIRRLEWQKQTS-----EGLAEYCTLIDSSSSFRAYR
AALSE--VEP-------PCIPY-LGLILQDLTFV--------------------------
-----HLGNP--DYI-------D---GKVNFS-K--------------------------

>Rapgef1_[Equus_caballus]_194225954
---EIAEQLTLLDAELFYKIE---IPEVLL--------WA-KEQ-----N--EE-KSP--
-------------------NLTQFTEHFNNMSYWVRSIIMLQEKAQDRERLLLKFIKIM-
KHLRK-LNNFNSYLAILSALDSAPIRRLEWQKQTS-----EGLAEYCTLIDSSSSFRAYR
AALSE--VEP-------PCIPY-LGLILQDLTFV--------------------------
-----HLGNP--DYI-------D---GKVNFS-K--------------------------

>Rapgef1_[Esox_lucius]_742225031
---EIADQLTLLDAELFYKIE---IPEVLL--------WA-KEQ-----N--EE-KSP--
-------------------NLTQFTEHFNNMSYWVRSLIIQQEKAQDREKLLLKFIKIM-
KHLRK-LNNFNSYLAILSALDSAPIRRLEWQKQTS-----EGLEEYCTLIDSSSSFRAYR
AALAD--VEP-------PCIPY-LGLILQDLTFV--------------------------
-----HLGNP--DLI-------E---GKVNFS-K--------------------------

>Rapgef1_[Eurypyga_helias]_704286424
---EIAEQLTLLDAELFYKIE---IPEVLL--------WA-KEQ-----N--EE-KSP--
-------------------NLTQFTEHFNNMSYWVRSIIMLQEKAQDRERLLLKFIKIM-
KHLRK-LNNFNSYLAILSALDSAPIRRLEWQKQTS-----EGLAEYCTLIDSSSSFRAYR
AALAD--VEP-------PCIPY-LGLILQDLTFV--------------------------
-----HLGNP--DYI-------D---SKVNFS-K--------------------------

>Rapgef1_[Ficedula_albicollis]_1020958161
-SHEIAEQLTLLDAELFYKIE---IPEVLL--------WA-KEQ-----N--EE-KSP--
-------------------NLTQFTEHFNNMSYWVRSIIMLQEKAQDRERLLLKFIKIM-
KHLRK-LNNFNSYLAILSALDSAPIRRLEWQKQTS-----EGLAEYCTLIDSSSSFRAYR
AALAD--VEP-------PCIPY-LGLILQDLTFV--------------------------
-----HLGNP--DYI-------D---SKVNFS-K--------------------------

>Rapgef1_[Fukomys_damarensis]_731223266
---EIAEQLTLLDAELFYKIE---IPEVLL--------WA-KEQ-----N--EE-KSP--
-------------------NLTQFTEHFNNMSYWVRSIIMLQEKAQDRERLLLKFIKIM-
KHLRK-LNNFNSYLAILSALDSAPIRRLEWQKQTS-----EGLAEYCTLIDSSSSFRAYR
AALSE--VEP-------PCIPY-LGLILQDLTFV--------------------------
-----HLGNP--DYV-------D---GKVNFS-K--------------------------

>Rapgef1_[Fundulus_heteroclitus]_831537766
---EIADQLTLLDAELFYKIE---IPEVLL--------WA-KEQ-----N--EE-KSP--
-------------------NLTQFTEHFNNMSYWVRSLIIQQEKAQDREKLLLKFIKIM-
KHLRK-LNNFNSYLAILSALDSAPIRRLEWQKQTS-----DGLEEYCTLIDSSSSFRAYR
AALAE--VEP-------PCIPY-LGLILQDLTFV--------------------------
-----HLGNP--DLI-------D---GKVNFS-K--------------------------

>Rapgef1_[Galeopterus_variegatus]_667337446
---EIAEQLTLLDAELFYKIE---IPEVLL--------WA-KEQ-----N--EE-KSP--
-------------------NLTQFTEHFNNMSYWVRSIIMLQEKAQDRERLLLKFIKIM-
KHLRK-LNNFNSYLAILSALDSAPIRRLEWQKQTS-----EGLAEYCTLIDSSSSFRAYR
AALSE--VEP-------PCIPY-LGLILQDLTFV--------------------------
-----HLGNP--DYI-------D---GKVNFS-K--------------------------

>Rapgef1_[Geospiza_fortis]_543261235
-SHEIAEQLTLLDAELFYKIE---IPEVLL--------WA-KEQ-----N--EE-KSP--
-------------------NLTQFTEHFNNMSYWVRSIIMLQEKAQDRERLLLKFIKIM-
KHLRK-LNNFNSYLAILSALDSAPIRRLEWQKQTS-----EGLAEYCTLIDSSSSFRAYR
AALAD--VEP-------PCIPY-LGLILQDLTFV--------------------------
-----HLGNP--DYI-------D---SKVNFS-K--------------------------

>Rapgef1_[Haliaeetus_leucocephalus]_729745536
-SHEIAEQLTLLDAELFYKIE---IPEVLL--------WA-KEQ-----N--EE-KSP--
-------------------NLTQFTEHFNNMSYWVRSIIMLQEKAQDRERLLLKFIKIM-
KHLRK-LNNFNSYLAILSALDSAPIRRLEWQKQTS-----EGLAEYCTLIDSSSSFRAYR
AALAD--VEP-------PCIPY-LGLILQDLTFV--------------------------
-----HLGNP--DYI-------D---SKVNFS-K--------------------------

>Rapgef1_[Heterocephalus_glaber]_512974084
---EIAEQLTLLDAELFYKIE---IPEVLL--------WA-KEQ-----N--EE-KSP--
-------------------NLTQFTEHFNNMSYWVRSIIMLQEKAQDRERLLLKFIKIM-
KHLRK-LNNFNSYLAILSALDSAPIRRLEWQKQTS-----EGLAEYCTLIDSSSSFRAYR
AALSE--VEP-------PCIPY-LGLILQDLTFV--------------------------
-----HLGNP--DYV-------E---GKVNFS-K--------------------------

>Rapgef1_[Ictidomys_tridecemlineatus]_532070648
-SHEIAEQLTLLDAELFYKIE---IPEVLL--------WA-KEQ-----N--EE-KSP--
-------------------NLTQFTEHFNNMSYWVRSIIMLQEKAQDRERLLLKFIKIM-
KHLRK-LNNFNSYLAILSALDSAPIRRLEWQKQTS-----EGLAEYCTLIDSSSSFRAYR
AALSE--VEP-------PCIPY-LGLILQDLTFV--------------------------
-----HLGNP--DYI-------D---GKVNFS-KRWQQFNIL---DSMRCFQQAH-----
------------------YDIRRNDDIINFFNDFSDHLAEEALWELSLK---IKPR
>Rapgef1_[Jaculus_jaculus]_507540166
---EIAEQLTLLDAELFYKIE---IPEVLL--------WA-KEQ-----N--EE-KSP--
-------------------NLTQFTEHFNNMSYWVRSIIMLQEKAQDRERLLLKFIKIM-
KHLRK-LNNFNSYLAILSALDSAPIRRLEWQRQTS-----EGLAEYCTLIDSSSSFRAYR
AALSE--VEP-------PCIPY-LGLILQDLTFV--------------------------
-----HLGNP--DYI-------D---GKVNFS-K--------------------------

>Rapgef1_[Kryptolebias_marmoratus]_1041071222
---EVADQLTLLDAELFYKIE---IPEVLL--------WA-KEQ-----N--EE-KSP--
-------------------NLTQFTEHFNNMSYWVRSLIIQQEKAQDREKLLLKFIKIM-
KHLRK-LNNFNSYLAILSALDSAPIRRLEWQKQTS-----EGLEEYCTLIDSSSSFRAYR
AALAE--VEP-------PCIPY-LGLILQDLTFV--------------------------
-----HLGNP--DLI-------D---GKVNFS-K--------------------------

>Rapgef1_[Leptonychotes_weddellii]_585165118
-SHEIAEQLTLLDAELFYKIE---IPEVLL--------WA-KEQ-----N--EE-KSP--
-------------------NLTQFTEHFNNMSYWVRSIIMLQEKAQDRERLLLKFIKIM-
KHLRK-LNNFNSYLAILSALDSAPIRRLEWQKQTS-----EGLAEYCTLIDSSSSFRAYR
AALSE--VEP-------PCIPY-LGLILQDLTFV--------------------------

```
-----HLGNP--DYI-------D---GKVNFS-KRWQQFNIL---DSMRCFQQAH-----
------------------YDIRRNDDIINFFNDFSDHLAEEALWELSLK---IKPR
>Rapgef1_[Loa_loa]_393908590
SSIDIAQQLLIFHTQLFEATDDI----ELI-----TQVFG-REQF--PNR-----ITS--
-------------------NLDLLMRRFNEVQFWTTTEVLLAQGNAKRLSILKKFIKIA-
AHAKE-NKDLLSLFAIILGLSNVAVSRITHLWDKLPSKMKQQYAEFEELLDPCRNHRAYR
MLTAN--MSA-------PTVPF-IPLLLKDLTFT--------------------------
-----HEGNK--TYF-------A---GLINFE-KMHMIANVL---RGFRQCK---YPAFA
FE-R--K---------------VFKSKNLVRN-FKVIDNQRRLMELSYQ---IEP-
>Rapgef1_[Macaca_mulatta]_966990136
-SHEIAEQLTLLDAELFYKIE---IPEVLL--------WA-KEQ-----N--EE-KSP--
-------------------NLTQFTEHFNNMSYWVRSIIMLQEKAQDRERLLLKFIKIM-
KHLRK-LNNFNSYLAILSALDSAPIRRLEWQKQTS-----EGLAEYCTLIDSSSSFRAYR
AALSE--VEP-------PCIPY-LGLILQDLTFV--------------------------
-----HLGNP--DYI-------D---GKVNFS-KRWQQFNIL---DSMRCFQQAH-----
------------------YDIRRNDDIINFFNDFSDHLAEEALWELSLK---IKPR
>Rapgef1_[Maylandia_zebra]_835915294
---EIADQLTLLDAELFYKIE---IPEVLL--------WA-KEQ-----N--EE-KSP--
-------------------NLTQFTEHFNNMSYWVRSLIIQQEKAQDREKLLLKFIKIM-
KHLRK-LNNFNSYLAILSALDSAPIRRLEWQKQTS-----EGLEEYCTLIDSSSSFRAYR
AALAE--VEP-------PCIPY-LGLILQDLTFV--------------------------
-----HLGNP--DLI-------D---GKVNFS-K--------------------------
--------------------------------------------------------
>Rapgef1_[Melopsittacus_undulatus]_527256067
-SHEIAEQLTLLDAELFYKIE---IPEVLL--------WA-KEQ-----N--EE-KSP--
-------------------NLTQFTEHFNNMSYWVRSIIMLQEKAQDRERLLLKFIKIM-
KHLRK-LNNFNSYLAILSALDSAPIRRLEWQKQTS-----EGLAEYCTLIDSSSSFRAYR
AALAD--VEP-------PCIPY-LGLILQDLTFV--------------------------
-----HLGNP--DYI-------D---SKVNFS-K--------------------------
--------------------------------------------------------
>Rapgef1_[Merops_nubicus]_677440357
-SHEIAEQLTLLDAELFYKIE---IPEVLL--------WA-KEQ-----N--EE-KSP--
-------------------NLTQFTEHFNNMSYWVRSIIMLQEKAQDRERLLLKFIKIM-
KHLRK-LNNFNSYLAILSALDSAPIRRLEWQKQTS-----EGLAEYCTLIDSSSSFRAYR
AALAD--VEP-------PCIPY-LGLILQDLTFV--------------------------
-----HLGNP--DYI-------D---SKVNFS-K--------------------------
--------------------------------------------------------
>Rapgef1_[Monodelphis_domestica]_611977953
-SHEIAEQLTLLDAELFYKIE---IPEVLL--------WA-KEQ-----N--EE-KSP--
-------------------NLTQFTEHFNNMSYWVRSIIMLQEKAQDRERLLLKFIKIM-
KHLRK-LNNFNSYLAILSALDSAPIRRLEWQKQTS-----EGLAEYCTLIDSSSSFRAYR
AALSE--VEP-------PCIPY-LGLILQDLTFV--------------------------
-----HLGNP--DYI-------D---GKVNFS-KRWQQFNIL---DSMRCFQQAH-----
------------------YEIRRNEDIVNFFNDFSDHLAEEALWELSLK---IKPR
>Rapgef1_[Mus_musculus]_568912752
---EIAEQLTLLDAELFYKIE---IPEVLL--------WA-KEQ-----N--EE-KSP--
-------------------NLTQFTEHFNNMSYWVRSIIMLQEKAQDRERLLLKFIKIM-
KHLRK-LNNFNSYLAILSALDSAPIRRLEWQRQTS-----EGLAEYCTLIDSSSSFRAYR
AALSE--VEP-------PCIPY-LGLILQDLTFV--------------------------
-----HLGNP--DYI-------D---GKVNFS-K--------------------------
--------------------------------------------------------
>Rapgef1_[Mustela_putorius_furo]_511874071
-SHEIAEQLTLLDAELFYKIE---IPEVLL--------WA-KEQ-----N--EE-KSP--
-------------------NLTQFTEHFNNMSYWVRSIIMLQEKAQDRERLLLKFIKIM-
```

KHLRK-LNNFNSYLAILSALDSAPIRRLEWQKQTS-----EGLAEYCTLIDSSSSFRAYR
AALSE--VEP-------PCIPY-LGLILQDLTFV-------------------------
-----HLGNP--DYI-------D---GKVNFS-KRWQQFNIL---DSMRCFQQAH-----
------------------YDIRRNEDIINFFNDFSDHLAEEALWELSLK---IKPR
>Rapgef1_[Myotis_lucifugus]_940778357
-SHEIAEQLTLLDAELFYKIE---IPEVLL--------WA-KEQ-----N--EE-KSP--
-------------------NLTQFTEHFNNMSYWVRSIIMLQEKAQDRERLLLKFIKIM-
KHLRK-LNNFNSYLAILSALDSAPIRRLEWQKQTS-----EGLAEYCTLIDSSSSFRAYR
AALSE--VEP-------PCIPY-LGLILQDLTFV-------------------------
-----HLGNP--DYI-------D---GKVNFS-KRWQQFNIL---DSMRCFQQAH-----
------------------YDIRRNDDIINFFNDFSDHLAEEALWELSLK---IKPR
>Rapgef1_[Neolamprologus_brichardi]_584003669
---EIADQLTLLDAELFYKIE---IPEVLL--------WA-KEQ-----N--EE-KSP--
-------------------NLTQFTEHFNNMSYWVRSLIIQQEKAQDREKLLLKFIKIM-
KHLRK-LNNFNSYLAILSALDSAPIRRLEWQKQTS-----EGLEEYCTLIDSSSSFRAYR
AALAE--VEP-------PCIPY-LGLILQDLTFV-------------------------
-----HLGNP--DLI-------D---GKVNFS-K-------------------------

>Rapgef1_[Nothobranchius_furzeri]_1007713379
---EIADQLTLLDAELFYKIE---IPEVLL--------WA-KEQ-----N--EE-KSP--
-------------------NLTQFTEHFNNMSYWVRSLIIQQEKAQDREKLLLKFIKIM-
KHLRK-LNNFNSYLAILSALDSAPIRRLEWQKQTS-----EGLEEYCTLIDSSSSFRAYR
AALAE--VEP-------PCIPY-LGLILQDLTFV-------------------------
-----HLGNP--DLI-------D---GKVNFS-K-------------------------

>Rapgef1_[Notothenia_coriiceps]_736310365
---EIADQLTLLDAELFYKIEVLEIPEVLL--------WA-KEQ-----N--EE-KSP--
-------------------NLTQFTEHFNNMSYWVRSLIIQQEKAQDREKLLLKFIKIM-
KHLRK-LNNFNSYLAILSALDSAPIRRLEWQKQTS-----EGLEEYCTLIDSSSSFRAYR
AALSE--VEP-------PCIPY-LGLILQDLTFV-------------------------
-----HLGNP--DFI-------E---GKVNFS-K-------------------------

>Rapgef1_[Ochotona_princeps]_837824864
-SHEIAEQLTLLDAELFYKIE---IPEVLL--------WA-KEQ-----N--EE-KSP--
-------------------NLTQFTEHFNNMSYWVRSIIMLQEKAQDRERLLLKFIKIM-
KHLRK-LNNFNSYLAILSALDSAPIRRLEWQKQTS-----EGLAEYCTLIDSSSSFRAYR
AALSE--VEP-------PCIPY-LGLILQDLTFV-------------------------
-----HLGNP--DHI-------D---GKVNFS-KRWQQFNIL---DSMRCFQQAH-----
------------------YDIRRNDDIINFFNDFSDHLAEEALWELSLK---IKPR
>Rapgef1_[Octodon_degus]_820983544
---EIAEQLTLLDAELFYKIE---IPEVLL--------WA-KEQ-----N--EE-KSP--
-------------------NLTQFTEHFNNMSYWVRSIIMLQEKAQDRERLLLKFIKIM-
KHLRK-LNNFNSYLAILSALDSAPIRRLEWQKQTS-----EGLAEYCTLIDSSSSFRAYR
AALSE--VEP-------PCIPY-LGLILQDLTFV-------------------------
-----HLGNP--DYV-------D---GKVNFS-K-------------------------

>Rapgef1_[Odobenus_rosmarus_divergens]_823391946
-SHEIAEQLTLLDAELFYKIE---IPEVLL--------WA-KEQ-----N--EE-KSP--
-------------------NLTQFTEHFNNMSYWVRSIIMLQEKAQDRERLLLKFIKIM-
KHLRK-LNNFNSYLAILSALDSAPIRRLEWQKQTS-----EGLAEYCTLIDSSSSFRAYR
AALSE--VEP-------PCIPY-LGLILQDLTFV-------------------------
-----HLGNP--DYI-------D---GKVNFS-KRWQQFNIL---DSMRCFQQAH-----
------------------YDIRRNDDIINFFNDFSDHLAEEALWELSLK---IKPR
>Rapgef1_[Opisthocomus_hoazin]_700373687

-SHEIAEQLTLLDAELFYKIE---IPEVLL--------WA-KEQ-----N--EE-KSP--
-------------------NLTQFTEHFNNMSYWVRSIIMLQEKAQDRERLLLKFIKIM-
KHLRK-LNNFNSYLAILSALDSAPIRRLEWQKQTS-----EGLAEYCTLIDSSSSFRAYR
AALAD--VEP-------PCIPY-LGLILQDLTFV--------------------------
-----HLGNP--DYI-------D---SKVNFS-K--------------------------

>Rapgef1_[Ornithorhynchus_anatinus]_620943894
---EIAEQLTLLDAELFYKIE---IPEVLL--------WA-KEQ-----N--EE-KSP--
-------------------NLTQFTEHFNNMSYWVRSIIMLQEKAQDRERLLLKFIKIM-
KHLRK-LNNFNSYLAILSALDSAPIRRLEWQKQTS-----EGLAEYCTLIDSSSSFRAYR
AALSE--VEP-------PCIPY-LGLILQDLTFV--------------------------
-----HLGNP--DYI-------D---GKVNFS-K--------------------------

>Rapgef1_[Ovis_aries_musimon]_803226629
---EIAEQLTLLDAELFYKIE---IPEVLL--------WA-KEQ-----N--EE-KSP--
-------------------NLTQFTEHFNNMSYWVRSIIMLQEKAQDRERLLLKFIKIM-
KHLRK-LNNFNSYLAILSALDSAPIRRLEWQKQTS-----EGLAEYCTLIDSSSSFRAYR
AALSE--VEP-------PCIPY-LGLILQDLTFV--------------------------
-----HLGNP--DYI-------D---GKVNFS-K--------------------------

>Rapgef1_[Pan_paniscus]_675769199
-SHEIAEQLTLLDAELFYKIE---IPEVLL--------WA-KEQ-----N--EE-KSP--
-------------------NLTQFTEHFNNMSYWVRSIIMLQEKAQDRERLLLKFIKIM-
KHLRK-LNNFNSYLAILSALDSAPIRRLEWQKQTS-----EGLAEYCTLIDSSSSFRAYR
AALSE--VEP-------PCIPY-LGLILQDLTFV--------------------------
-----HLGNP--DYI-------D---GKVNFS-KRWQQFNIL---DSMRCFQQAH-----
------------------YDIRRNDDIINFFNDFSDHLAEEALWELSLK---IKPR
>Rapgef1_[Papio_anubis]_685591345
-SHEIAEQLTLLDAELFYKIE---IPEVLL--------WA-KEQ-----N--EE-KSP--
-------------------NLTQFTEHFNNMSYWVRSIIMLQEKAQDRERLLLKFIKIM-
KHLRK-LNNFNSYLAILSALDSAPIRRLEWQKQTS-----EGLAEYCTLIDSSSSFRAYR
AALSE--VEP-------PCIPY-LGLILQDLTFV--------------------------
-----HLGNP--DYI-------D---GKVNFS-KRWQQFNIL---DSMRCFQQAH-----
------------------YDIRRNDDIINFFNDFSDHLAEEALWELSLK---IKPR
>Rapgef1_[Parus_major]_998724189
-SHEIAEQLTLLDAELFYKIE---IPEVLL--------WA-KEQ-----N--EE-KSP--
-------------------NLTQFTEHFNNMSYWVRSIIMLQEKAQDRERLLLKFIKIM-
KHLRK-LNNFNSYLAILSALDSAPIRRLEWQKQTS-----EGLAEYCTLIDSSSSFRAYR
AALAD--VEP-------PCIPY-LGLILQDLTFV--------------------------
-----HLGNP--DYI-------D---SKVNFS-K--------------------------

>Rapgef1_[Pelecanus_crispus]_694646979
---EIAEQLTLLDAELFYKIE---IPEVLL--------WA-KEQ-----N--EE-KSP--
-------------------NLTQFTEHFNNMSYWVRSIIMLQEKAQDRERLLLKFIKIM-
KHLRK-LNNFNSYLAILSALDSAPIRRLEWQKQTS-----EGLAEYCTLIDSSSSFRAYR
AALAD--VEP-------PCIPY-LGLILQDLTFV--------------------------
-----HLGNP--DYI-------D---NKVNFS-K--------------------------

>Rapgef1_[Peromyscus_maniculatus_bairdii]_1008767945
---EIAEQLTLLDAELFYKIE---IPEVLL--------WA-KEQ-----N--EE-KSP--
-------------------NLTQFTEHFNNMSYWVRSIIMLQEKAQDRERLLLKFIKIM-
KHLRK-LNNFNSYLAILSALDSAPIRRLEWQRQTS-----EGLAEYCTLIDSSSSFRAYR
AALSE--VEP-------PCIPY-LGLILQDLTFV--------------------------
-----HLGNP--DYI-------D---GKVNFS-K--------------------------

>Rapgef1_[Phaethon_lepturus]_723138787
-SHEIAEQLTLLDAELFYKIE---IPEVLL--------WA-KEQ-----N--EE-KSP--
-------------------NLTQFTEHFNNMSYWVRSIIMLQEKAQDRERLLLKFIKIM-
KHLRK-LNNFNSYLAILSALDSAPIRRLEWQKQTS-----EGLAEYCTLIDSSSSFRAYR
AALAD--VEP-------PCIPY-LGLILQDLTFV--------------------------
-----HLGNP--DYI-------D---SKVNFS-K--------------------------

>Rapgef1_[Phalacrocorax_carbo]_695141004
---EIAEQLTLLDAELFYKIE---IPEVLL--------WA-KEQ-----N--EE-KSP--
-------------------NLTQFTEHFNNMSYWVRSIIMLQEKGQDRERLLLKFIKIM-
KHLRK-LNNFNSYLAILSALDSAPIRRLEWQKQTS-----EGLAEYCTLIDSSSSFRAYR
AALAD--VEP-------PCIPY-LGLILQDLTFV--------------------------
-----HLGNP--DYI-------D---SKVNFS-K--------------------------

>Rapgef1_[Phoenicopterus_ruber_ruber]_677495181
-SHEIAEQLTLLDAELFYKIE---IPEVLL--------WA-KEQ-----N--EE-KSP--
-------------------NLTQFTEHFNNMSYWVRSIIMLQEKAQDRERLLLKFIKIM-
KHLRK-LNNFNSYLAILSALDSAPIRRLEWQKQTS-----EGLAEYCTLIDSSSSFRAYR
AALAD--VEP-------PCIPY-LGLILQDLTFV--------------------------
-----HLGNP--DYI-------D---SKVNFS-K--------------------------

>Rapgef1_[Physeter_catodon]_593753470
-SHEIAEQLTLLDAELFYKIE---IPEVLL--------WA-KEQ-----N--EE-KSP--
-------------------NLTQFTEHFNNMSYWVRSIIMLQEKAQDRERLLLKFIKIM-
KHLRK-LNNFNSYLAILSALDSAPIRRLEWQKQTS-----EGLAEYCTLIDSSSSFRAYR
AALSE--VEP-------PCIPY-LGLILQDLTFV--------------------------
-----HLGNP--DYI-------D---GKVNFS-KRWQQFNIL---DSMRCFQQAH-----
------------------YDIRRNDDIINFFNDFSDHLAEEALWELSLK---IKPR
>Rapgef1_[Poecilia_latipinna]_961885811
---EIADQLTLLDAELFYKIE---IPEVLL--------WA-KEQ-----N--EE-KSP--
-------------------NLTQFTEHFNNMSYWVRSLIIQQEKAQDREKLLLKFIKIM-
KHLRK-LNNFNSYLAILSALDSAPIRRLEWQKQTS-----EGLEEYCTLIDSSSSFRAYR
AALAE--VEP-------PCIPY-LGLILQDLTFV--------------------------
-----HLGNP--DLI-------D---GKVNFS-K--------------------------

>Rapgef1_[Poecilia_mexicana]_961888169
---EIADQLTLLDAELFYKIE---IPEVLL--------WA-KEQ-----N--EE-KSP--
-------------------NLTQFTEHFNNMSYWVRSLIIQQEKAQDREKLLLKFIKIM-
KHLRK-LNNFNSYLAILSALDSAPIRRLEWQKQTS-----EGLEEYCTLIDSSSSFRAYR
AALAE--VEP-------PCIPY-LGLILQDLTFV--------------------------
-----HLGNP--DLI-------D---GKVNFS-K--------------------------

>Rapgef1_[Pongo_abelii]_686731902
-SHEIAEQLTLLDAELFYKIE---IPEVLL--------WA-KEQ-----N--EE-KSP--
-------------------NLTQFTEHFNNMSYWVRSIIMLQEKAQDRERLLLKFIKIM-
KHLRK-LNNFNSYLAILSALDSAPIRRLEWQKQTS-----EGLAEYCTLIDSSSSFRAYR
AALSE--VEP-------PCIPY-LGLILQDLTFV--------------------------
-----HLGNP--DYI-------D---GKVNFS-KRWQQFNIL---DSMRCFQQAH-----
------------------YD------------------------------------
>Rapgef1_[Pseudopodoces_humilis]_929474213
---EIAEQLTLLDAELFYKIE---IPEVLL--------WA-KEQ-----N--EE-KSP--
-------------------NLTQFTEHFNNMSYWVRSIIMLQEKAQDRERLLLKFIKIM-
KHLRK-LNNFNSYLAILSALDSAPIRRLEWQKQTS-----EGLAEYCTLIDSSSSFRAYR

AALAD--VEP-------PCIPY-LGLILQDLTFV--------------------------
-----HLGNP--DYI-------D---SKVNFS-K--------------------------

>Rapgef1_[Pterocles_gutturalis]_678138997
-SHEIAEQLTLLDAELFYKIE---IPEVLL--------WA-KEQ-----N--EE-KSP--
-------------------NLTQFTEHFNNMSYWVRSIIMLQEKAQDRERLLLKFIKIM-
KHLRK-LNNFNSYLAILSALDSAPIRRLEWQKQTS-----EGLAEYCTLIDSSSSFRAYR
AALAD--VEP-------PCIPY-LGLILQDLTFV--------------------------
-----HLGNP--DYI-------D---NKVNFS-K--------------------------

>Rapgef1_[Rattus_norvegicus]_672016556
---EIAEQLTLLDAELFYKIE---IPEVLL--------WA-KEQ-----N--EE-KSP--
-------------------NLTQFTEHFNNMSYWVRSVIMLQEKAQDRERLLLKFIKIM-
KHLRK-LNNFNSYLAILSALDSAPIRRLEWQRQTS-----EGLAEYCTLIDSSSSFRAYR
AALSE--VEP-------PCIPY-LGLILQDLTFV--------------------------
-----HLGNP--DYI-------D---GKVNFS-K--------------------------

>Rapgef1_[Rhinopithecus_roxellana]_724871904
-SHEIAEQLTLLDAELFYKIE---IPEVLL--------WA-KEQ-----N--EE-KSP--
-------------------NLTQFTEHFNNMSYWVRSIIMLQEKAQDRERLLLKFIKIM-
KHLRK-LNNFNSYLAILSALDSAPIRRLEWQKQTS-----EGLAEYCTLIDSSSSFRAYR
AALSE--VEP-------PCIPY-LGLILQDLTFV--------------------------
-----HLGNP--DYI-------D---GKVNFS-KRWQQFNIL---DSMRCFQQAH-----
------------------YDIRRNDDIINFFNDFSDHLAEEALWELSLK---IKPR
>Rapgef1_[Saimiri_boliviensis_boliviensis]_725551265
-SHEIAEQLTLLDAELFYKIE---IPEVLL--------WA-KEQ-----N--EE-KSP--
-------------------NLTQFTEHFNNMSYWVRSIIMLQEKAQDRERLLLKFIKIM-
KHLRK-LNNFNSYLAILSALDSAPIRRLEWQKQTS-----EGLAEYCTLIDSSSSFRAYR
AALSE--VEP-------PCIPY-LGLILQDLTFV--------------------------
-----HLGNP--DYI-------D---GKVNFS-KRWQQFNIL---DSMRRFQQAH-----
------------------YDIRRNDDIINFFNDFSDHLAEEALWELSLK---IKPR
>Rapgef1_[Scleropages_formosus]_938060232
SAEEVANQLVAFDRELVGCIHEV----EFV-----SYVFQ-WEQA--RWR------PL--
-------------------NLELVLQRCSEVQHWVATEILQCQSLPKRLQLLRKFIKIA-
ALCKQ-QQDLLSFLAVVLGLDNPAVSRLRLTWESLPGKFKKQFQQFEAVADPSRNHKSYR
DLTAS--LQP-------PIIPF-IPLLLKDLTFL--------------------------
-----NESCK--TFH-------G---ELVNFE-KMHKIAEMV---RGIRRYRSSQLAVDS
EPSP--S---------------YLQTKAYVRQ-LQVIDNQNLLFELSCK---LEPK
>Rapgef1_[Sinocyclocheilus_anshuiensis]_1025025898
---EIADQLTLLDAELFYKIE---IPEVLL--------WA-KEQ-----N--EE-KSP--
-------------------NLTQFTEHFNNMSYWVRSLIIQQEKAQDREKLLLKFIKIM-
KHLRK-LNNFNSYLAILSALDSAPIRRLEWQKQTS-----EGLEEYCTLIDSSSSFRAYR
AALAD--VEP-------PCIPY-LGLILQDLTFV--------------------------
-----HLGNP--DLI-------D---RKINFS-K--------------------------

>Rapgef1_[Sinocyclocheilus_grahami]_1020480960
---EIADQLTLLDAELFYKIE---IPEVLL--------WA-KEQ-----N--EE-KSP--
-------------------NLTQFTEHFNNMSYWVRSLIIQQEKPQDREKLLLKFIKIM-
KHLRK-LNNFNSYLAILSALDSAPIRRLEWQKQTS-----EGLEEYCTLIDSSSSFRAYR
AALAD--VEP-------PCIPY-LGLILQDLTFV--------------------------
-----HLGNP--DLI-------D---GKINFS-K--------------------------

>Rapgef1_[Sinocyclocheilus_rhinocerous]_1025363012
---EIADQLTLLDAELFYKIE---IPEVLL--------WA-KEQ-----N--EE-KSP--

-------------------NLTQFTEHFNNMSYWVRSIIIQQEKAQDREKLLLKFIKIM-
KHLRK-LNNFNSYLAILSALDSAPIRRLEWQKQTS-----EGLEEYCTLIDSSSSFRAYR
AALAE--VEP-------PCIPY-LGLILQDLTFV--------------------------
-----HLGNP--DFI-------D---GKVNFS-K--------------------------

>Rapgef1_[Sorex_araneus]_505839952
-SHEIAEQLTLLDAELFYKIE---IPEVLL--------WA-KEQ-----N--EE-KSP--
-------------------NLTQFTEHFNNMSYWVRSIIMLQEKAQDRERLLLKFIKIM-
KHLRK-LNNFNSYLAILSALDSAPIRRLEWQKQTS-----EGLAEYCTLIDSSSSFRAYR
AALSE--VEP-------PCIPY-LGLILQDLTFV--------------------------
-----HLGNP--DYI-------D---GKVNFS-KRWQQFNIL---DSMRCFQQAH-----
------------------YDIRRNDDVISFFNDFSDHLAEEALWELSLK---IKPR
>Rapgef1_[Tauraco_erythrolophus]_678150753
---EIAEQLTLLDAELFYKIE---IPEVLL--------WA-KEQ-----N--EE-KSP--
-------------------NLTQFTEHFNNMSYWVRSIIMLQEKAQDRERLLLKFIKIM-
KHLRK-LNNFNSYLAILSALDSAPIRRLEWQKQTS-----EGLAEYCTLIDSSSSFRAYR
AALAD--VEP-------PCIPY-LGLILQDLTFV--------------------------
-----HLGNP--DYI-------E---SKVNFS-K--------------------------

>Rapgef1_[Tinamus_guttatus]_719760629
---EIAEQLTLLDAELFYKIE---IPEVLL--------WA-KEQ-----N--EE-KSP--
-------------------NLTQFTEHFNNMSYWVRSIIMVQEKAQDRERLLLKFIKIM-
KHLRK-LNNFNSYLAILSALDSAPIRRLEWQKQTS-----EGLAEYCTLIDSSSSFRAYR
AALAD--VDP-------PCIPY-LGLILQDLTFV--------------------------
-----HLGNP--DYI-------D---SKVNFS-K--------------------------

>Rapgef1_[Toxocara_canis]_734558230
SSVDIAQQLSIFHTQLFEATDEI----ELI-----TQVFG-RDQF--PGR-----IPS--
-------------------NLDLLMRRFNEVQFWTTTEVLLAHGPSKRVTMLKKFIKIA-
SHAKE-NRDLMSLFAIILGLSNVAVSRITHIWDKLPSKMRRQYAEFEALLDPSRNHRAYR
MLVAK--MSS-------PTVPF-VPLLLKDLTFT--------------------------
-----HEGNK--TYF-------A---GLVNFE-KMHMIANVL---RSFKQCKS-KYPVTS
FDHK--K---------------VFESKNLIRN-FKVIDNQRRLMEISFQ---IEP-
>Rapgef1_[Tyto_alba]_701396907
-SHEIAEQLTLLDAELFYKIE---IPEVLL--------WA-KEQ-----N--EE-KSP--
-------------------NLTQFTEHFNNMSYWVRSIIMLQEKAQDRERLLLKFIKIM-
KHLRK-LNNFNSYLAILSALDSAPIRRLEWQKQTS-----EGLAEYCTLIDSSSSFRAYR
AALAD--VEP-------PCIPY-LGLILQDLTFV--------------------------
-----HLGNP--DYI-------D---NKVNFS-K--------------------------

>Rapgef1_[Vicugna_pacos]_970743100
-SHEIAEQLTLLDAELFYKIE---IPEVLL--------WA-KEQ-----N--EE-KSP--
-------------------NLTQFTEHFNNMSYWVRSIIMLQEKAQDRERLLLKFIKIM-
KHLRK-LNNFNSYLAILSALDSAPIRRLEWQKQTS-----EGLAEYCTLIDSSSSFRAYR
AALSE--VEP-------PCIPY-LGLILQDLTFV--------------------------
-----HLGNP--DYI-------D---GKVNFS-KRWQQFNIL---DSMRCFQQAH-----
------------------YDIRRNDDIINFFNDFSDHLAEEALWELSLK---IKPR
>Rapgef1_[Zonotrichia_albicollis]_542177208
-SHEIAEQLTLLDAELFYKIE---IPEVLL--------WA-KEQ-----N--EE-KSP--
-------------------NLTQFTEHFNNMSYWVRSIIMLQEKAQDRERLLLKFIKIM-
KHLRK-LNNFNSYLAILSALDSAPIRRLEWQKQTS-----EGLAEYCTLIDSSSSFRAYR
AALAD--VEP-------PCIPY-LGLILQDLTFV--------------------------
-----HLGNP--DYI-------D---SKVNFS-K--------------------------

>Rapgef2_[Acinonyx_jubatus]_961717932
STVEVATQLSMRNFELFRNIEPT----EYI-----DDLFK-L-KS----K-TSC-A----
-------------------NLKKFEEVINQETFWVASEILRETNQLKRMKIIKHFIKIA-
LHCRE-CKNFNSMFAIISGLNLAPVARLRTTWEKLPNKYEKLFQDLQDLFDPSRNMAKYR
NVLNSQNLQP-------PIIPL-FPVIKKDLTFL--------------------------
-----HEGND--SKV-------D---GLVNFE-KLRMIAKEI---RHVGRMASVNMDP--

>Rapgef2_[Acromyrmex_echinatior]_746838618
---EVAIQLTLQDFSIFRQIEST----EYV-----DDLFE-L-KS----R-YGV-PM---
--------------------LSQFAELVNREMFWVVTEVCSEHNLVRRSKIIKQFIKIA-
RQCKE-CKNFNSMFAIVSGLGHGSVSRLRASWEKLPTKYQRLFSDLQELMDPSRNMSKYR
QLVASEQTQP-------PIIPF-YPVVKKDLTFI--------------------------
-----HLGND--SRV-------E---SLVNFE-KLRMIAKEV---RTLTNMCSSPYD---

>Rapgef2_[Acropora_digitifera]_1005422629

--------------------------------FWVITEICSESNLVKRMKIMKTFIKIA-
RLCKE-CKNYNTLFAIVSGLGHSAVQRLKNTWDKLPTRHSKAFEDLQGLMDPSRNMSKYR
NLLNGEHGEP-------PLIPF-FPVITKDLTFI--------------------------
-----HLGND--SII-------D---GLVNFE-KLRLIAREI---RQISKFNSIPYDP--

>Rapgef2_[Ailuropoda_melanoleuca]_752411684
STVEVATQLSMRNFELFRNIEPT----EYI-----DDLFK-L-KS----K-TSC-A----
-------------------NLKKFEEVINQETFWVASEILRETNQLKRMKIIKHFIKIA-
LHCRE-CKNFNSMFAIISGLNLAPVARLRTTWEKLPNKYEKLFQDLQDLFDPSRNMAKYR
NVLNSQNLQP-------PIIPL-FPVIKKDLTFL--------------------------
-----HEGND--SKV-------D---GLVNFE-KLRMIAKEI---RHVGRMASVNMDP--

>Rapgef2_[Alligator_mississippiensis]_1011564443
STIEVATQLSMRDFELFRNIEPT----EYI-----DDLFK-L-DS----K-TGN-A----
-------------------HLKQFEDVINQETFWVATEILLEPNQLKRMKIIKHFIKIA-
LHCRE-CKNFNSMFAVISGLNLASVARLRGTWEKLPSKYEKHFRDLQDLFDPSRNMAKYR
NILSSQSMQP-------PIIPL-FPVVKKDITFL--------------------------
-----HEGND--SKV-------D---GLVNFE-KLRMIAKEI---RQVIRMTSANMDP--

>Rapgef2_[Alligator_sinensis]_557280478
STVEVATQLSMRNFELFRNIEPT----EYI-----DDLFK-L-KS----K-SGC-T----
-------------------NLKKFEEVINQETFWVASEILRETNQLKRMKIIKHFIKIA-
LHCRE-CKNFNSMFAIISGLNLAPVARLRTTWEKLPSKYEKLFQDLQDLFDPSRNMAKYR
NVLNSQNLQP-------PIIPL-FPVIKKDLTFL--------------------------
-----HEGND--SKV-------E---GLVNFE-KLRMIAKEI---RHVGRMASVNMDP--

>Rapgef2_[Amyelois_transitella]_913296464
---EVAVQLTLQDFCIFRQIEST----EYV-----DDLFE-L-KS----R-YGT-PM---
--------------------LSQFAELVNKEMFWVVTEVVNEQNIVRRSKIIKQFIKVA-
RHCKE-CKNFNSMFAIISGLGHGAVSRLRMTWDKLPTKYYKLFTDLQMLMDPSRNMSKYR
QLVTTEQGRS-------PVIPF-YPVVKKDLTFI--------------------------
-----HLGND--TKV-------E---GMVNFE-KLRMIAKEV---RTLTNMCSSPYD---

>Rapgef2_[Anser_cygnoides_domesticus]_902881430
STVEVATQLSMRNFELFRNIEPT----EYI-----DDLFK-L-KS----K-TGC-T----
-------------------NLKKFEEVINQETFWVASEILRETNQLKRMKIIKHFIKIA-
LHCRE-CKNFNSMFAIISGLNLAPVARLRTTWEKLPSKYEKLFQDLQDLFDPSRNMAKYR
NVLNSQNLQP-------PIIPL-FPVIKKDLTFL--------------------------

-----HEGND--SKV-------E---GLVNFE-KLRMIAKEI---RHVGRMASVNMDP--

>Rapgef2_[Apis_cerana]_1035602531
---EVAIQLTLQDFSIFRQIEST----EYV-----DDLFE-L-KS----R-YGV-PM---
--------------------LRQFAELVNREMFWVVTEVCSEHNLVRRSKIIKQFIKIA-
RQCKE-CKNFNSMFAIVSGLGHGAVSRLRASWEKLPSKYQRLFSDLQELMDPSRNMSKYR
QLVASEQTQP-------PIIPF-YPVVKKDLTFI-------------------------
-----HLGND--SRV-------E---GLVNFE-KLRMIAKEV---RTLTNMCSSPYD---

>Rapgef2_[Apis_florea]_820851409
---EVAIQLTLQDFSIFRQIEST----EYV-----DDLFE-L-KS----R-YGV-PM---
--------------------LRQFAELVNREMFWVVTEVCSEHNLVRRSKIIKQFIKIA-
RQCKE-CKNFNSMFAIVSGLGHGAVSRLRASWEKLPSKYQRLFSDLQELMDPSRNMSKYR
QLVASEQTQP-------PIIPF-YPVVKKDLTFI-------------------------
-----HLGND--SRV-------E---GLVNFE-KLRMIAKEV---RTLTNMCSSPYD---

>Rapgef2_[Apis_mellifera]_1032018809
---EVAIQLTLQDFSIFRQIEST----EYV-----DDLFE-L-KS----R-YGV-PM---
--------------------LRQFAELVNREMFWVVTEVCSEHNLVRRSKIIKQFIKIA-
RQCKE-CKNFNSMFAIVSGLGHGAVSRLRASWEKLPSKYQRLFSDLQELMDPSRNMSKYR
QLVASEQTQP-------PIIPF-YPVVKKDLTFI-------------------------
-----HLGND--SRV-------E---GLVNFE-KLRMIAKEV---RTLTNMCSSPYD---

>Rapgef2_[Aplysia_californica]_871253417
TAHEVAWQLTLNDYRVFREIEPT----EYV-----DDLYN-L-QS----K-FGC-A----
-------------------QLHKFAELVNKEMFWVVTEICSEANLVKRTKLLKHFIKIA-
MHCKD-SKNFNSMFAILSGLGHGSISRLKLTWERLPSKYSKLFQDLQSFMDPSRNMAKYR
NLINS--VQA-------PFIPF-FPIFKKDLTFI-------------------------
-----HLGND--SHV-------D---GLVNFE-KLRMIAKEV---RNI------------

>Rapgef2_[Apteryx_australis_mantelli]_926516120
STVEVATQLSMRNFELFRNIEPT----EYI-----DDLFK-L-KS----K-TGC-T----
-------------------NLKKFEEVINQETFWVASEILRETNQLKRMKIIKHFIKIA-
LHCRE-CKNFNSMFAIISGLNLAPVARLRTTWEKLPSKYEKLFQDLQDLFDPSRNMAKYR
NVLNSQSLQP-------PIIPL-FPVIKKDLTFL-------------------------
-----HEGND--SKV-------E---GLVNFE-KLRMIAKEI---RHVGRMASVNMDP--

>Rapgef2_[Aquila_chrysaetos_canadensis]_768351322
STVEVATQLSMRNFELFRNIEPT----EYI-----DDLFK-L-KS----K-TGC-T----
-------------------NLKKFEEVINQETFWVASEILRETNQLKRMKIIKHFIKIA-
LHCRE-CKNFNSMFAIISGLNLAPVARLRTTWEKLPSKYEKLFQDLQDLFDPSRNMAKYR
NVLNSQNLQP-------PIIPL-FPVIKKDLTFL-------------------------
-----HEGND--SKV-------E---GLVNFE-KLRMIAKEI---RHVGRMASVNMDP--

>Rapgef2_[Ascaris_suum]_541048950
----VAAQLTLQDFAVFSSIEPT----EYV-----DNLFN-L-ES----R-YGW-P----
-------------------KLSEFEALFNKEMWWVPSEVCRERSIQKRAKLVKKFIKVA-
RHCRD-FHNFNSMFAIMSGLDKPAVRRLHHTWERVPNKYVKMFEDLQQLVDPSRNMSKYR
QHLSEVSNEP-------PVVPM-YPVLKKDLTFS-------------------------
-----HEGNP--TYC-------G---KLVNFE-KLRMIARAI---RSVTKLCSVPY----

>Rapgef2_[Athalia_rosae]_817056263
---EVAIQLTLQDFSIFRQIEST----EYV-----DDLFK-L-KS----R-YGV-PM---
--------------------LSHFAELVNREMFWVVTEVCSEHNLVRRSKIIKQFLKIA-

RQCKE-CKNFNSMFAIVSGLGHGAVCRLRGSWEKLPSKYQRLFTDLEKLMDPSRNMCQYR
QLVASEQTQP-------PVIPF-YPVVRKDLTFI-------------------------
-----HLGND--SRV-------E---GLVNFE-KLRMIAKEV---RTLTKMCSSPYD---

>Rapgef2_[Atta_colombica]_1009357161
---EVAIQLTLQDFSIFRQIEST----EYV-----DDLFE-L-KS----R-YGV-PM---
--------------------LSQFAELVNREMFWVVTEVCSEHNLVRRS----------
-QCKE-CKNFNSMFAIVSGLGHGSVSRLRASWEKLPTKYQRLFSDLQELMDPSRNMSKYR
QLVASEQTQP-------PIIPF-YPVVKKDLTFI-------------------------
-----HLGND--SRV-------E---GLVNFE-KLRMIAKEV---RTLTNMCSSPYD---

>Rapgef2_[Bactrocera_cucurbitae]_751445210
---ELAIQLTLQDFAIFRQIEST----EYI-----DDLFN-L-KT----K-YGV-PM---
--------------------LTKFAELVNREMFWVVTEICSEHNIVRRMKIVKQFIKIA-
RHCKE-CRNFNSMFAIISGLGHAAVSRLRLTWEKLPSKYQRVFSDLQDLMDPSRNMSKYR
QLVSLELFNQH------PIIPF-YPIVKKDLTFI-------------------------
-----HLGND--TRI-------E---GLINFE-KLRMLAKEV---RLLTHMCSSPYD---

>Rapgef2_[Bactrocera_oleae]_929380230
---ELAIQLTLQDFAIFRQIEST----EYI-----DDLFN-L-KT----K-YGV-PM---
--------------------LTKFAELVNREMFWVVTEICGEHNIVRRMKIVKQFIKIA-
RHCKE-CRNFNSMFAIISGLGHAAVSRLRLTWEKLPSKYQRLFSDLQDLMDPSRNMSKYR
QLVSSELLAQH------PIIPF-YPIVKKDLTFI-------------------------
-----HLGND--TRI-------E---GLINFE-KLRMLAKEV---RLLTHMCSSPYD---

>Rapgef2_[Balaenoptera_acutorostrata_scammoni]_594672686
STVEAATQLSMRNFELFRNVEPT----EYI-----DDLFK-L-RS----K-TSC-A----
-------------------NLKKFEEVINQETFWVASEILRETNQLKRMKIIKHFIKIA-
LHCRE-CKNFNSMFAIISGLNLAPVARLRTTWEKLPNKYEKLFQDLQDLFDPSRNMAKYR
NVLNSQNLQP-------PIIPL-FPVIKKDLTFL-------------------------
-----HEGND--SKV-------D---GLVNFE-KLRMIAKEI---RHVGRMASVNMDP--

>Rapgef2_[Balearica_regulorum_gibbericeps]_723572377
STVEVATQLSMRNFELFRNIEPT----EYI-----DDLFK-L-KS----K-TGC-T----
-------------------NLKKFEEVINQETFWVASEILRETNQLKRMKIIKHFIKIA-
LHCRE-CKNFNSMFAIISGLNLAPVARLRTTWEKLPSKYEKLFQDLQDLFDPSRNMAKYR
NVLNSQNLQP-------PIIPL-FPVIKKDLTFL-------------------------
-----HEGND--SKV-------E---GLVNFE-KLRMIAKEI---RHVGRMASVNMDP--

>Rapgef2_[Bombus_impatiens]_815915167
---EVAIQLTLQDFSIFRQIEST----EYV-----DDLFE-L-KS----R-YGV-PM---
--------------------LRQFAELVNREMFWVVTEVCSEHNLVRRSKIIKQFIKIA-
RQCKE-CKNFNSMFAIVSGLGHGAVSRLRASWEKLPSKYQRLFSDLQELMDPSRNMSKYR
QLVASEQTQP-------PIIPF-YPVVKKDLTFI-------------------------
-----HLGND--SRV-------E---GLVNFE-KLRMIAKEV---RTLTNMCSSPYD---

>Rapgef2_[Bombus_terrestris]_808120538
---EVAIQLTLQDFSIFRQIEST----EYV-----DDLFE-L-KS----R-YGV-PM---
--------------------LRQFAELVNREMFWVVTEVCSEHNLVRRSKIIKQFIKIA-
RQCKE-CKNFNSMFAIVSGLGHGAVSRLRASWEKLPSKYQRLFSDLQELMDPSRNMSKYR
QLVASEQTQP-------PIIPF-YPVVKKDLTFI-------------------------
-----HLGND--SRV-------E---GLVNFE-KLRMIAKEV---RTLTNMCSSPYD---

>Rapgef2_[Bombyx_mori]_512898941

---EVAVQLTLQDFCIFRQIEST----EYV-----DDLFE-L-KS----R-YGT-PM---
--------------------LSQFAELVNKEMFWVVTEVVNEQNLVRRSKIIKQFIKVA-
RHCKE-CKNFNSMFAIISGLGHGAVSRLRMTWEKLPTKYYKLFTDLQMLMDPSRNMSKYR
QLVTSEQGRS-------PVIPF-YPVVKKDLTFI--------------------------
-----HLGND--TKV-------E---GMVNFE-KLRMIAKEV---RTLTNMCSSPYD---

>Rapgef2_[Bos_mutus]_440905105
STVEVATQLSMRNFELFRNIEPT----EYI-----DDLFK-L-KS----K-TSC-A----
-------------------NLKTFEEVINQETFWVASEILRETNQLKRMKIIKHFIKIA-
LHCRE-CKNFNSMFAIISGLNLAPVARLRTTWEKLPNKYEKLFQDLQDLFDPSRNMAKYR
NVLNSQNLQP-------PIIPL-FPVIKKDLTFL--------------------------
-----HEGND--SKV-------D---GLVNFE-KLRMIAKEI---RHVGRMASVNMDP--

>Rapgef2_[Bos_taurus]_982949939
STVEVATQLSMRNFELFRNIEPT----EYI-----DDLFK-L-KS----K-TSC-A----
-------------------NLKTFEEVINQETFWVASEILRETNQLKRMKIIKHFIKIA-
LHCRE-CKNFNSMFAIISGLNLAPVARLRTTWEKLPNKYEKLFQDLQDLFDPSRNMAKYR
NVLNSQNLQP-------PIIPL-FPVIKKDLTFL--------------------------
-----HEGND--SKV-------D---GLVNFE-KLRMIAKEI---RHVGRMASVNMDP--

>Rapgef2_[Bubalus_bubalis]_594101758
STVEVATQLSMRNFELFRNIEPT----EYI-----DDLFK-L-KS----K-TSC-A----
-------------------NLKTFEEVINQETFWVASEILRETNQLKRMKIIKHFIKIA-
LHCRE-CKNFNSMFAIISGLNLAPVARLRTTWEKLPNKYEKLFQDLQDLFDPSRNMAKYR
NVLNSQNLQP-------PIIPL-FPVIKKDLTFL--------------------------
-----HEGND--SKV-------D---GLVNFE-KLRMIAKEI---RHVGRMASVNMDP--

>Rapgef2_[Buceros_rhinoceros_silvestris]_704156104
STVEVATQLSMRNFELFRNIEPT----EYI-----DDLFK-L-KS----K-SGC-T----
-------------------NLKKFEEVINQETFWVASEILRETNQLKRMKIIKHFIKIA-
LHCRE-CKNFNSMFAIISGLNLAPVARLRTTWEKLPSKYEKLFQDLQDLFDPSRNMAKYR
NVLNSQNLQP-------PIIPL-FPVIKKDLTFL--------------------------
-----HEGND--SKV-------E---GLVNFE-KLRMIAKEI---RHVGRMASVNMDP--

>Rapgef2_[Calypte_anna]_663260986
STVEVATQLSMRNFELFRNIEPT----EYI-----DDLFK-L-KS----K-TGC-T----
-------------------NLKKFEEVINQETFWVASEILRETNQLKRMKIIKHFIKIA-
LHCRE-CKNFNSMFAIISGLNLAPVARLRTTWEKLPSKYEKLFQDLQDLFDPSRNMAKYR
NVLNSQNLQP-------PIIPL-FPVIKKDLTFL--------------------------
-----HEGND--SKV-------E---GLVNFE-KLRMIAKEI---RHVGRMASVNMDP--

>Rapgef2_[Capra_hircus]_926712293
STVEVATQLSMRNFELFRNIEPT----EYI-----DDLFK-L-KS----K-TSC-A----
-------------------NLKTFEEVINQETFWVASEILRETNQLKRMKIIKHFIKIA-
LHCRE-CKNFNSMFAIISGLNLAPVARLRTTWEKLPNKYEKLFQDLQDLFDPSRNMAKYR
NVLNSQNLQP-------PIIPL-FPVIKKDLTFL--------------------------
-----HEGND--SKV-------D---GLVNFE-KLRMIAKEI---RHVGRMASVNMDP--

>Rapgef2_[Caprimulgus_carolinensis]_704313429
STVEVATQLSMRNFELFRNIEPT----EYI-----DDLFK-L-KS----K-TGC-T----
-------------------NLKKFEEVINQETFWVASEILRETNQLKRMKIIKHFIKIA-
LHCRE-CKNFNSMFAIISGLNLAPVARLRTTWEKLPSKYEKLFQDLQDLFDPSRNMAKYR
NVLNSQNLQP-------PIIPL-FPVIKKDLTFL--------------------------
-----HEGND--SKV-------E---GLVNFE-KLRMIAKEI---RHVGRMASVNMDP--

>Rapgef2_[Cariama_cristata]_698402657
STVEVATQLSMRNFELFRNIEPT----EYI-----DDLFK-L-KS----K-TGC-T----
-------------------NLKKFEEVINQETFWVASEILRETNQLKRMKIIKHFIKIA-
LHCRE-CKNFNSMFAIISGLNLAPVARLRTTWEKLPSKYEKLFQDLQDLFDPSRNMAKYR
NVLNSQNLQP-------PIIPL-FPVIKKDLTFL--------------------------
-----HEGND--SKV-------E---GLVNFE-KLRMIAKEI---RHVGRMASVNMDP--

>Rapgef2_[Carlito_syrichta]_640783230
STVEVATQLSMRNFELFRNIEPT----EYI-----DDLFK-L-KS----K-TNC-A----
-------------------NLKKFEEVINQETFWVASEILRETNQLKRMKIIKHFIKIA-
LHCRE-CKNFNSMFAIISGLNLAPVARLRTTWEKLPNKYEKLFQDLQDLFDPSRNMAKYR
NVLNSQNLQP-------PIIPL-FPVIKKDLTFL--------------------------
-----HEGND--SKV-------D---GLVNFE-KLRMIAKEI---RHVGRMASVNMDP--

>Rapgef2_[Cavia_porcellus]_884936739
SPTEVSKQLSMRNFELFRNIEPT----EYI-----DDLFK-L-RS----K-TSC-A----
-------------------NLKMFEEVINQETFWVASEILRETNQLKRMKIIKHFIKIA-
LHCRE-CKNFNSMFTIISGLNLAPVARLRTTWEKLPNKYEKLFQDLQDLFDPSRNMAKYR
NVLNSQNLQP-------PIIPL-FPIIKKDLTFL--------------------------
-----HEGND--SKV-------D---GLVNFE-KLRMIA---------------------

>Rapgef2_[Cerapachys_biroi]_759046373
---EVAIQLTLQDFSIFRQIEST----EYV-----DDLFE-L-KS----R-YGV-PM---
--------------------LSQFAELVNREMFWVVTEVCSEHNLVRRSKIIKQFIKIA-
RQCKE-CKNFNSMFAIVSGLGHGAVSRLRASWEKLPTKYQRLFSDLQELMDPSRNMSKYR
QLVASEQTQP-------PIIPF-YPVVKKDLTFI--------------------------
-----HLGND--SRV-------E---GLVNFE-KLRMIAKEV---RTLTNMCSSPYD---

>Rapgef2_[Ceratosolen_solmsi_marchali]_766935029
---EVAIQLTLQDFSIFRQIEST----EYV-----DDLFE-L-KS----K-YGV-PM---
--------------------LSQFAELVNREMFWVVTEVCSEHNLVRRSKIIKQFIKIA-
RQCKE-CKNFNSMFAIVSGLGHGAVSRLRASWEKLPSKYQRLFSDLQELMDPSRNMSKYR
QLVASEQTQP-------PIIPF-YPVVKKDLTFI--------------------------
-----HLGND--SRV-------E---GLVNFE-KLRMIAKEV---RTL------------

>Rapgef2_[Ceratotherium_simum_simum]_955490007
STVEVATQLSMRNFELFRNIEPT----EYI-----DDLFK-L-KS----K-TSC-V----
-------------------NLKKFEEVINQETFWVASEILRETNQLKRMKIIKHFIKIA-
LHCRE-CKNFNSMFAIISGLNLAPVARLRTTWEKLPNKYEKLFQDLQDLFDPSRNMAKYR
NVLNSQNLQP-------PIIPL-FPVIKKDLTFL--------------------------
-----HEGND--SKV-------D---GLVNFE-KLRMIAKEI---RHVGRMASVNMDP--

>Rapgef2_[Chelonia_mydas]_591353971
STVEMAAQLSMRNFELFRNIEPT----EYI-----DDLFK-L-KS----K-ISC-T----
-------------------NLKKFEEVINQETFWVASEILRETNQLKRMKIIKHFIKIA-
LHCRE-CKNFNSMFAIISGLNLAPVARLRTTWEKLPSKYEKLFQDLQDLFDPSRNMAKYR
NVLNSQNLQP-------PIIPL-FPVIKKDLTFL--------------------------
-----HEGND--SKV-------E---GLVNFE-KLRMIAKEI---RHVGRMASVNMDP--

>Rapgef2_[Chlamydotis_macqueenii]_705683189
STVEVATQLSMRNFELFRNIEPT----EYI-----DDLFK-L-KS----K-TGC-T----
-------------------NLKKFEEVINQETFWVASEILRETNQLKRMKIIKHFIKIA-
LHCRE-CKNFNSMFAIISGLNLAPVARLRTTWEKLPSKYEKLFQDLQDLFDPSRNMAKYR

NVLNSQNLQP-------PIIPL-FPVIKKDLTFL-------------------------
-----HEGND--SKV-------E---GLVNFE-KLRMIAKEI---RHVGRMASVNMDP--

>Rapgef2_[Chlorocebus_sabaeus]_635045487
STVEVATQLSMRNFELFRNIEPT----EYI-----DDLFK-L-RS----K-TSC-A----
-------------------NLKRFEEVINQETFWVASEILRETNQLKRMKIIKHFIKIA-
LHCRE-CKNFNSMFAIISGLNLAPVARLRTTWEKLPNKYEKLFQDLQDLFDPSRNMAKYR
NVLNSQNLQP-------PIIPL-FPVIKKDLTFL-------------------------
-----HEGND--SKV-------D---GLVNFE-KLRMIAKEI---RHVGRMASVNMDP--

>Rapgef2_[Ciona_intestinalis]_699243364
---EVAVFLTRKDYKMFQATAST----DFI-----EDLFH-L-DA----E------LT--
-------------------QFKAFEDLVNRELFWVVTTLCNEPNLIQRSKIIKHFVKIA-
HHCKI-LKNLNSTFALISGLGYRAVSRMKQTWEKVPAKSNRLFEELQALMDPSRNMSKYR
TLLNEMVSQP-------PVIPY-IPVIKKDLTFL-------------------------
-----DLGND--TKV-------E---GLINFE-KMRMIGKEV---RNV------------

>Rapgef2_[Clonorchis_sinensis]_358336436
TPEDLAIRVTLDDYEVFRAVQST----EYI-----DEVFG-LSPALNCLSLSDA-AV---
SHPTPT------GYATGHGNLDRFTELVNREAYWAPTEICMESNLNRRVDLIKRFIKLA-
KLCRD-LRNFNTMFCILVGLHQTPVERLKQTWERLPNKYFKMYRDLSMVLDTSRNFFQYR
SMLTGTEAAA-------PMLPY-LPLVLKDLTFI-------------------------
-----HLGNP--SRS-------SD--GLINFV-KLRMLAKEI---RAICRMCNVEYDIDA
AH--
>Rapgef2_[Colius_striatus]_706134628
STVEVATQLSMRNFELFRNIEPT----EYI-----DDLFK-L-KS----K-TGC-T----
-------------------NLKKFEEVINQETFWVASEILRETNQLKRMKIIKHFIKIA-
LHCRE-CKNFNSMFAIISGLNLAPVARLRTTWEKLPSKYEKLFQDLQDLFDPSRNMAKYR
NVLNSQNLQP-------PIIPL-FPVIKKDLTFL-------------------------
-----HEGND--SKV-------E---GLVNFE-KLRMIAKEI---RHVGRMASVNMDP--

>Rapgef2_[Colobus_angolensis_palliatus]_795294397
STVEVATQLSMRNFELFRNIEPT----EYI-----DDLFK-L-RS----K-TSC-A----
-------------------NLKRFEEVINQETFWVASEILRETNQLKRMKIIKHFIKIA-
LHCRE-CKNFNSMFAIISGLNLAPVARLRTTWEKLPNKYEKLFQDLQDLFDPSRNMAKYR
NVLNSQNLQP-------PIIPL-FPVIKKDLTFL-------------------------
-----HEGND--SKV-------D---GLVNFE-KLRMIAKEI---RHVGRMASVNMDP--

>Rapgef2_[Corvus_cornix_cornix]_727064554
STVEVATQLSMRNFELFRNIEPT----EYI-----DDLFK-L-KS----K-TGC-T----
-------------------NLKRFEEVINQETFWVASEILRETNQLKRMKIIKHFIKIA-
LHCRE-CKNFNSMFAIISGLNLAPVARLRTTWEKLPSKYEKLFQDLQDLFDPSRNMAKYR
NVLNSQNLQP-------PIIPL-FPVIKKDLTFL-------------------------
-----HEGND--SKV-------E---GLVNFE-KLRMIAKEI---RHVGRMASVNMDP--

>Rapgef2_[Crassostrea_gigas]_762094094
---EVASQLTLDDFKVFASIQPT----EYI-----DKLFN-I-KS----K-YGI-P----
-------------------NLEKFVELVNKEMYWVITEVCSEPNVVKRMRIIKQFIKIA-
KHCKD-CKNFNSMFSILSGLDKVYVSRLRNTWEKLSKKDLKTYDDLKELIDPSKNMSKYR
SYVGSEHVQP-------PMLPL-FPVAMKDLTFL-------------------------
-----KDGND--TKV-------D---GLINFE-KFRMIAKEI---R--------------

>Rapgef2_[Cricetulus_griseus]_1032926035
STVEVATQLSMRNFELFRNIEPT----EYI-----DDLFK-L-KS----K-TSC-A----

-------------------NLKKFEEVINQETFWVASEILRETNQLKRMKIIKHFIKIA-
LHCRE-CKNFNSMFAIISGLNLAPVARLRTTWEKLPNKYEKLFQDLQDLFDPSRNMAKYR
NVLSGQNLQP-------PVIP---------------------------------------

>Rapgef2_[Culex_quinquefasciatus]_170039216
NANELAIQLTLQDFSIFRQIEST----EYI-----DDLFN-L-KS----R-YGK-PM---
--------------------LVKFAELVNREMFWVVTEVCSEHNMMRRCKIIKQFIKIA-
RHCKE-CKNFNSLFAIVSGLGHAAVSRLRQTWEKLPSKYQKLFNDLQELMDPSRNMSKYR
QLIQTELNAQH------PVIPF-YPVVKKDLTFI--------------------------
-----HLGND--SKI-------E---SLINFE-KLRMISKEV---RTLLQMCNS------

>Rapgef2_[Cynoglossus_semilaevis]_1035251282
STVEVATQLSMRAFELFCAIEPT----EYI-----DDLFK-L-RS----K-TGS-A----
-------------------SLKRFEEAINHETFWVATEVTREPNQLKRMKTVKHFIKIA-
LHCRE-CKNFSSMFAIISGLNLAPVSRLRGTWEKLPSKYEKLFGDLQDLFDPSRNMAKYR
NVLNNQNLQP-------PIIPL-FPVIKKDLTFL--------------------------
-----HEGND--SKV-------D---GLVNFE-KLRMIAKEI---RHVGRMASVNMDP--

>Rapgef2_[Cyphomyrmex_costatus]_1009369324
---EVAIQLTLQDFSIFRQIEST----EYV-----DDLFE-L-KS----R-YGV-PM---
--------------------LSQFAELVNREMFWVVTEVCSEHNLVRRSKIIKQFIKIA-
RQCKE-CKNFNSMFAIVSGLGHGSVSRLRASWEKLPTKYQRLFSDLQELMDPSRNMSKYR
QLVASEQTQP-------PIIPF-YPVVKKDLTFI--------------------------
-----HLGND--SRV-------E---GLVNFE-KLRMIAKEV---RTLTNMCSSPYD---

>Rapgef2_[Danio_rerio]_1040663345
STVEVATQLSMRAFELFCAIEPT----EYI-----DDLFK-L-KT----RLTGP-P----
-------------------SLKLFEEAINRETFWVATEVVREANQLKRMKIVKHFIKIA-
LHCRE-CKNFNSMFAIISGLNLAPVSRLRGTWEKLPSKYEKLFSDLQDLFDPSRNMAKYR
NLLNSQNLQP-------PIIPL-FPVIKKDLTFL--------------------------
-----HEGND--SKV-------D---GLVNFE-KLRMIAKEI---RHV------------

>Rapgef2_[Diachasma_alloeum]_970900035
---EVAIQLTLQDFSIFRQIEST----EYV-----DDLFE-L-KS----R-YGV-PM---
--------------------LSQFAELVNREMFWVVTEVCSEYNLVKRSKIIKQFIKIA-
RQCKE-CKNFNSMFAIVSGLGHGAVSRLRASWEKLPSKYQRLFSDLQELMDPSRNMSKYR
QLVASEQTQP-------PIIPF-YPVVKKDLTFI--------------------------
-----HLGND--SRV-------E---GLVNFE-KLRMIAKEV---RTLTNMCSSPYD---

>Rapgef2_[Diaphorina_citri]_662216327
---EVATQLTLQDYAIFRQIEAT----EYV-----DDLFE-L-KS----G-YGV-PM---
--------------------LSQFAELVNREMFWVVTEVCSESNLVRRSKVIKQFIKVA-
RQCKE-CRNFNSMFAIVSGLGHGAVSRLRSSWEKLPSKYQRLFIRIKS------------
-------------------IPF-YPVVKKDLTFI--------------------------
-----HLGND--SKV-------E---GLINFE-KLRMIAKEV---RTLTNMCSAPYD---

>Rapgef2_[Dinoponera_quadriceps]_951557922
---EVAIQLTLQDFSIFRQIEST----EYV-----DDLFE-L-KS----R-YGV-PM---
--------------------LSQFAELVNREMFWVVTEVCSEHNLVRRSKIIKQFIKIA-
RQCKE-CKNFNSMFAIVSGLGHGAVSRLRASWEKLPTKYQRLFSDLQELMDPSRNMSKYR
QLVASEQTQP-------PIIPF-YPVVKKDLTFI--------------------------
-----HLGND--SRV-------E---GLVNFE-KLRMIAKEV---RTLTNMCSSPYD---

>Rapgef2_[Diuraphis_noxia]_985398052
---EVAVQLTLQDFSIFRQIEAT----EYV-----DDLFQ-L-DS----R-YGT-PM---
--------------------LSKFAELVNTEMFWVVTEICSEHNLIRRSKMIKQFIKVA-
RQCKE-CKNFNSMFAIISGLGHGAVSRLRQSWEKLPTKYQRLFSDLQDLMDPSRNMSKYR
QLVSNEQSQP-------PIIPF-YPVVKKDLTFI--------------------------
-----HLGND--THV-------E---SLINFE-KLRMIAKEV---RNLTNMCSSPYD---

>Rapgef2_[Drosophila_biarmipes]_1036675528
---ELAIQLTIQDFANFRQIEST----EYV-----DELFE-L-KS----R-YGV-PM---
--------------------LSKFAELVNREMFWVVTEICAEHNIVRRMKIVKQFIKIA-
RHCKE-CRNFNSMFAIVSGLGHGAVSRLRQTWEKLPSKYQRLFNDLQDLMDPSRNMSKYR
QLVSAELLAQH------PIIPF-YPIVKKDLTFI--------------------------
-----HLGND--TTV-------D---GLINFE-KLRMLAKEV---RLLTHMCSSPYD---

>Rapgef2_[Drosophila_suzukii]_1036058972
---ELAIQLTLQDFANFRQIEST----EYV-----DELFE-L-RS----R-YGV-PM---
--------------------LSKFAELVNREMFWVVSEICAEHNIVRRMKIVKQFIKIA-
RHCKE-CRNFNSMFAIISGLGHGAVSRLRQTWEKLPSKYQRLFNDLQDLMDPSRNMSKYR
QLVSAELLAQH------PIIPF-YPIVKKDLTFI--------------------------
-----HLGNN--TTV-------D---GLINFE-KLRMLAKEV---RLLTHMCSSPYD---

>Rapgef2_[Dufourea_novaeangliae]_987912049
---EVAIQLTLQDFSIFRQIEST----EYV-----DDLFE-L-KS----R-YGV-PM---
--------------------LRQFAELVNREMFWVVTEVCSEHNLVRRSKIIKQFIKIA-
RQCKE-CKNFNSMFAIVSGLGHGAVSRLRASWEKLPNKYQRLFSDLQELMDPSRNMSKYR
QLVASEQTQP-------PIIPF-YPVVKKDLTFI--------------------------
-----HLGND--SRV-------E---SLVNFE-KLRMIAKEV---RTLTNMCSSPYD---

>Rapgef2_[Echinococcus_granulosus]_576698151
SPEEVAIQLTLDDFSIFRSIEAA----EFV-----DKVFG-LTPP-ASSALDGC-SNSSN
NENIDNDGGGGGGFATGCGNLDAFADVVNKEAYWVPSELCAETSLPKRVDMLKRFIKIA-
KLCRD-LRNFNTMFCILVGLHMSPVERLRQTWERLPNKYVKMSRDLALVLDPSRNFAHYR
NLLTSPPQSPQTGAQCLPLVPY-LPLVLKDLTFI--------------------------
-----HLGNP--SRSPATSASSSACPQLINFA-KLRMFAKEI---RSLR-----------

>Rapgef2_[Echinops_telfairi]_507671420
STVEVATQLSMRNFELFRNIEPT----EYI-----DDLFK-L-KS----K-NSC-A----
-------------------NLKKFEDVINQETFWVASEILRETNQLKRMKIVKHFIKIA-
LHCRE-CKNFNSMFAIISGLNLAPVARLRTTWEKLPNKYEKLFQDLQDLFDPSRNMAKYR
NVLNSQNLQP-------PIIPL-FPVIKKDLTFL--------------------------
-----HEGND--SKV-------D---GLVNFE-KLRMIAKEI---RHVGRMASVNMDP--

>Rapgef2_[Eptesicus_fuscus]_641691941
STVEVATQLSMRNFELFRNIEPT----EYI-----DDLFK-L-KS----K-TSC-A----
-------------------NLKKFEEVINQETFWVASEILRETNQLKRMKIIKHFIKIA-
LHCRE-CKNFNSMFAIISGLNLAPVARLRTTWEKLPNKYEKLFQDLQDLFDPSRNMAKYR
NVLNSQNLQP-------PIIPL-FPVIKKDLTFL--------------------------
-----HEGND--SKV-------D---GLVNFE-KLRMIAKEI---RHVGRMASVNMDP--

>Rapgef2_[Equus_asinus]_958803745
STVEVATQLSMRNFELFRNIEPT----EYI-----DDLFK-L-KS----K-TSC-V----
-------------------NLKKFEEVINQETFWVASEILRETNQLKRMKIIKHFIKIA-
LHCRE-CKNFNSMFAIISGLNLAPVARLRTTWEKLPNKYEKLFQDLQDLFDPSRNMAKYR
TVLNSQNLQP-------PIIPL-FPVIKKDLTFL--------------------------

-----HEGND--SKV-------D---GLVNFE-KLRMIAKEI---RHVGRMASVNMDP--

>Rapgef2_[Equus_caballus]_953847565
STVEVATQLSMRNFELFRNIEPT----EYI-----DDLFK-L-KS----K-TSC-V----
-------------------NLKKFEEVINQETFWVASEILRETNQLKRMKIIKHFIKIA-
LHCRE-CKNFNSMFAIISGLNLAPVARLRTTWEKLPNKYEKLFQDLQDLFDPSRNMAKYR
TVLNSQNLQP-------PIIPL-FPVIKKDLTFL--------------------------
-----HEGND--SKV-------D---GLVNFE-KLRMIAKEI---RHVGRMASVNMDP--

>Rapgef2_[Esox_lucius]_742092564
STVEVATQLSMRAFELFCAIEPT----EYI-----DDLFK-L-KS----K-TGS-F----
-------------------CLKRFEEAINQETFWVASEVTREPNQLKRMKTVKHFIKIA-
LHCRE-CKNFNSMFAIISGLNLAPVSRLRGTWEKLPSKYEKLFGDLQDLFDPSRNMAKYR
NVLNNQNLQP-------PIIPL-FPVIKKDLTFL--------------------------
-----HEGND--SKV-------D---GLVNFE-KLRMIAKEI---RHVGRMASVNMDP--

>Rapgef2_[Falco_cherrug]_541968958
STVEVATQLSMRNFELFRNIEPT----EYI-----DDLFK-L-KS----K-TGC-A----
-------------------NLKKFEEVINQETFWVASEILRETNQLKRMKIIKHFIKIA-
LHCRE-CKNFNSMFAIISGLNLAPVARLRTTWEKLPSKYEKLFQDLQDLFDPSRNMAKYR
NVLNSQNLQP-------PIIPL-FPVIKKDLTFL--------------------------
-----HEGND--SKV-------E---GLVNFE-KLRMIAKEI---RHVGRMASVNMDP--

>Rapgef2_[Felis_catus]_755716562
STVEVATQLSMRNFELFRNIEPT----EYI-----DDLFK-L-KS----K-TSC-A----
-------------------NLKKFEEVINQETFWVASEILRETNQLKRMKIIKHFIKIA-
LHCRE-CKNFNSMFAIISGLNLAPVARLRTTWEKLPNKYEKLFQDLQDLFDPSRNMAKYR
NVLNSQNLQP-------PIIPL-FPVIKKDLTFL--------------------------
-----HEGND--SKV-------D---GLVNFE-KLRMIAKEI---RHVGRMASVNMDP--

>Rapgef2_[Ficedula_albicollis]_1020979019
STVEVATQLSMRNFELFRNIEPT----EYI-----DDLFK-L-KS----K-TGC-T----
-------------------NLKRFEEVINQETFWVASEILRETNQLKRMKIIKHFIKIA-
LHCRE-CKNFNSMFAIISGLNLAPVARLRTTWEKLPSKYEKLFQDLQDLFDPSRNMAKYR
NVLNSQNLQP-------PIIPL-FPVIKKDLTFL--------------------------
-----HEGND--SKV-------E---GLVNFE-KLRMIAKEI---RHVGRMASVNMDP--

>Rapgef2_[Fundulus_heteroclitus]_831545559
STVEVATQLSMRAFELFCAIEPT----EYI-----DDLFK-L-RS----K-TGS-V----
-------------------SLKHFEESINQETFWVATEVVREPNQLKRMKIVKHFIKIA-
LHCRE-CKNFNSMFAIISGLNLAPVSRLRGTWEKLPSKYEKLFGDLQDLFDPSRNMAKYR
NVLNNQNLQP-------PIIPL-FPVIKKDLTFL--------------------------
-----HEGND--SKV-------D---GLVNFE-KLRMIAKEI---RHVGRMASVNMDP--

>Rapgef2_[Haliaeetus_albicilla]_700326780
STVEVATQLSMRNFELFRNIEPT----EYI-----DDLFK-L-KS----K-TGC-T----
-------------------NLKKFEEVINQETFWVASEILRETNQLKRMKIIKHFIKIA-
LHCRE-CKNFNSMFAIISGLNLAPVARLRTTWEKLPSKYEKLFQDLQDLFDPSRNMAKYR
NVLNSQNLQP-------PIIPL-FPVIKKDLTFL--------------------------
-----HEGND--SKV-------E---GLVNFE-KLRMIAKEI---RHVGRMASVNMDP--

>Rapgef2_[Haliaeetus_leucocephalus]_729726819
STVEVATQLSMRNFELFRNIEPT----EYI-----DDLFK-L-KS----K-TGC-T----
-------------------NLKKFEEVINQETFWVASEILRETNQLKRMKIIKHFIKIA-

LHCRE-CKNFNSMFAIISGLNLAPVARLRTTWEKLPSKYEKLFQDLQDLFDPSRNMAKYR
NVLNSQNLQP-------PIIPL-FPVIKKDLTFL--------------------------
-----HEGND--SKV-------E---GLVNFE-KLRMIAKEI---RHVGRMASVNMDP--

>Rapgef2_[Harpegnathos_saltator]_749732961
---EVAIQLTLQDFSIFRQIEST----EYV-----DDLFV-L-KS----R-YGV-PM---
--------------------LSQFAELVNREMFWVVTEVCSEHNLVRRSKIIKQFIKIA-
RQCKE-CKNFNSMFAIVSGLGHGAVSRLRASWEKLPTKYQRLFSDLQELMDPSRNMSKYR
QLVASEQTQP-------PIIPF-YPVVKKDLTFI--------------------------
-----NLGND--SRV-------E---GLVNFE-KLRMIAKEV---RTLTNMCSSPYD---

>Rapgef2_[Heterocephalus_glaber]_351696315
---K-TSC-A----
-------------------NLKKFEEVINQETFWVASEILRETNQLKRMKIIKHFIKIA-
LHCRE-CKNFNSMFAIISGLNLAPVARLRTTWEKLPNKYEKLFQDLQDLFDPSRNMAKYR
NVLNSQNLQP-------PIIPL-FPVIKKDLTFL--------------------------
-----HEGND--SKV-------D---GLVNFE-KLRMIAKEI---RHVGRMASVNMDP--

>Rapgef2_[Ictalurus_punctatus]_1042371529
STVEVATQLSMRAFELFCAIEPT----EYI-----DDLFK-L-RS----RSSGL-S----
-------------------SLKLFEESINRETFWVATEVLREPNQLKRMKIIKHFIKIA-
LHCRE-CKNFNSMFAIISGLNLAPVSRMRGTWEKLPSKYEKLFSDLQDLFDPSRNMAKYR
NVLNSQNLQP-------PIIPL-FPVIKKDLTFL--------------------------
-----HEGND--SKV-------D---GLVNFE-KLRMIAKEI---RHV------------

>Rapgef2_[Ictidomys_tridecemlineatus]_914915193
STVEVATQLSMRNFELFRNIEPT----EYI-----DDLFK-L-KS----K-TSC-H----
-------------------NLKKFEEVINQETFWVASEILRETNQLKRMKIIKHFIKIA-
LHCRE-CKNFNSMFAIISGLNLAPVARLRATWEKLPNKYEKLFQDLQDLFDPSRNMAKYR
NVLNSQNLQP-------PIIPL-FPVIKKDLTFL--------------------------
-----HEGND--SKV-------D---GLVNFE-KLRMIAKEI---RHVGRMASVNMDP--

>Rapgef2_[Kryptolebias_marmoratus]_1041100174
STVEVATQLSMRAFELFCAIEPT----EYI-----DDLFK-L-RS----K-VGS-V----
-------------------SLKRFEESINHETFWVATEVVREPNQLKRMKIVKHFIKIA-
LHCRE-CKNFNSMFAIISGLNLAPVSRLRGTWEKLPSKYEKLFGDLQDLFDPSRNMAKYR
NVLNNQNLQP-------PIIPL-FPVIKKDLTFL--------------------------
-----HEGND--SKV-------D---GLVNFE-KLRMIAKEI---RHVGRMASVNMDP--

>Rapgef2_[Latimeria_chalumnae]_556992865
STIEVATQLSMRNFELFRNIEAT----EYI-----DDLFK-L-KT----K-VGS-S----
-------------------NLKKFEEVINQETFWVASEILKELNQIKRMKIIKHFIKIA-
LHCRE-CKNFNSMFAIISGLNLAPVARLRSTWEKLNSKYEKLFQDLQDLFDPSRNMAKYR
NVLNSQNLQP-------PIIPL-FPVIKKDLTFL--------------------------
-----HEGND--SKV-------E---GLVNFE-KLRMIAKEI---RHVGRMASVNMDP--

>Rapgef2_[Lepisosteus_oculatus]_972965628
STVEVATQLSMRNFELFCNIEPT----EYI-----DDLFK-L-KS----R-TGS-A----
-------------------NLKKFEEVINQETFWVASEIVREQNQIKRMKIIKHFIKIA-
LHCRE-CKNFNSMFAIISGLNLAPVSRLRGTWEKLPSKYEKLFQDLQDLFDPSRNMAKYR
NVLNSQNLQP-------PIIPL-FPVIKKDLTFL--------------------------
-----HEGND--SKV-------D---GLVNFE-KLRMIAKEI---RHVGRMASVNMDP--

>Rapgef2_[Leptonychotes_weddellii]_585176488

STVEVATQLSMRNFELFRNIEPT----EYI-----DDLFK-L-KS----K-TSC-A----
-------------------NLKKFEEVINQETFWVASEILRETNQLKRMKIIKHFIKIA-
LHCRE-CKNFNSMFAIISGLNLAPVARLRTTWEKLPNKYEKLFQDLQDLFDPSRNMAKYR
NVLNSQNLQP-------PIIPL-FPVIKKDLTFL--------------------------
-----HEGND--SKV-------D---GLVNFE-KLRMIAKEI---RHVGRMASVNMDP--

>Rapgef2_[Limulus_polyphemus]_926643048

--------------------------------FWVVTEVCSEPNPLRRMKIIKQFIKIA-
RQCKE-CKNLNSMFAILSGLDHGAVSRLRSSWEKLPSKYSKMFKDLSDFMDPSRNMCKYR
TLISSEHTQP-------PRIPF-YPIVRKDLMFI--------------------------
-----HLGND--TKV-------E---GLINFE-KLRMIAKEI---RQL------------

>Rapgef2_[Lingula_anatina]_919048487
-SIEIATQLTLEDFHIFANIEAT----EYI-----DDLFD-L-PS----K-YGT-P----
-------------------NILQFQELVNREMFWVTTEVVRETNIIKRMKIIKHFIKIA-
RHCKE-CKNFNSMFAILSGLGHGSVQRLRTTWDKLPNKYVKIYEDLQTIMDPSRNMAKYR
NLINSELVQP-------PLIPL-FPIVKKDLTFI--------------------------
-----HLGND--SKV-------D---GLVNFE-KLRMIAKEV---RHLCLMASAPYDPNA

>Rapgef2_[Macaca_mulatta]_297293615
STVEVATQLSMRNFELFRNIEPT----EYI-----DDLFK-L-RS----K-TSC-A----
-------------------NLKRFEEVINQETFWVASEILRETNQLKRMKIIKHFIKIA-
LHCRE-CKNFNSMFAIISGLNLAPVARLRTTWEKLPNKYEKLFQDLQDLFDPSRNMAKYR
NVLNSQNLQP-------PIIPL-FPVIKKDLTFL--------------------------
-----HEGND--SKV-------D---GLVNFE-KLRMIAKEI---RHVGRMASVNMDP--

>Rapgef2_[Macaca_nemestrina]_795381724
STVEVATQLSMRNFELFRNIEPT----EYI-----DDLFK-L-RS----K-TSC-A----
-------------------NLKRFEEVINQETFWVASEILRETNQLKRMKIIKHFIKIA-
LHCRE-CKNFNSMFAIISGLNLAPVARLRTTWEKLPNKYEKLFQDLQDLFDPSRNMAKYR
NVLNSQNLQP-------PIIPL-FPVIKKDLTFL--------------------------
-----HEGND--SKV-------D---GLVNFE-KLRMIAKEI---RHVGRMASVNMDP--

>Rapgef2_[Mandrillus_leucophaeus]_795325386
STVEVATQLSMRNFELFRNIEPT----EYI-----DDLFK-L-RS----K-TSC-A----
-------------------NLKRFEEVINQETFWVASEILRETNQLKRMKIIKHFIKIA-
LHCRE-CKNFNSMFAIISGLNLAPVARLRTTWEKLPNKYEKLFQDLQDLFDPSRNMAKYR
NVLNSQNLQP-------PIIPL-FPVIKKDLTFL--------------------------
-----HEGND--SKV-------D---GLVNFE-KLRMIAKEI---RHVGRMASVNMDP--

>Rapgef2_[Maylandia_zebra]_498996321
STVEVATQLSMRAFELFCAIEPT----EYI-----DDLFK-L-RS----K-TGS-V----
-------------------SLKRFEEAINHETFWVATEVTREPNQLKRMKIIKHFIKIA-
LHCRE-CKNFSSMFAIISGLNLAPVSRLRGTWEKLPSKYEKLFGDLQDLFDPSRNMAKYR
NVLSNQNLQP-------PIIPL-FPVIKKDLTFL--------------------------
-----HEGND--SKV-------D---GLVNFE-KLRMIAKEI---RHVGRMASVNMDP--

>Rapgef2_[Melipona_quadrifasciata]_925684072
---EVAIQLTLQDFSIFRQIEST----EYV-----DDLFE-L-KS----R-YGV-PM---
--------------------LRQFAELVNREMFWVVTEVCSEHNLVRRSKIIKQFIKIA-
RQCKE-CKNFNSMFAIVSGLGHGAVSRLRASWEKLPSKYQRLFSDLQELMDPSRNMSKYR
QLVASEQTQP-------PIIPF-YPVVKKDLTFI--------------------------
-----HLGND--SRV-------E---GLVNFE-KLRMIAKEV---RTLTNMCSSPYD---

>Rapgef2_[Melopsittacus_undulatus]_884809301
STVEVATQLSMRNFELFRNIEPT----EYI-----DDLFK-L-KS----K-TGC-T----
-------------------NLKKFEEVINQETFWVASEILRETNQLKRMKIIKHFIKIA-
LHCRE-CKNFNSMFAIISGLNLAPVARLRTTWEKLPSKYEKLFQDLQDLFDPSRNMAKYR
NVLNSQNLQP-------PIIPL-FPVIKKDLTFL--------------------------
-----HEGND--SKV-------E---GLVNFE-KLRMIAKEI---RHVGRMASVNMDP--

>Rapgef2_[Merops_nubicus]_675617135
STVEVATQLSMRNFELFRNIEPT----EYI-----DDLFK-L-KS----K-TGC-T----
-------------------NLKKFEEVINQETFWVASEILRETNQLKRMKIIKHFIKIA-
LHCRE-CKNFNSMFAIISGLNLAPVARLRTTWEKLPSKYEKLFQDLQDLFDPSRNMAKYR
NVLNSQNLQP-------PIIPL-FPVIKKDLTFL--------------------------
-----HEGND--SKV-------E---GLVNFE-KLRMIAKEI---RHVGRMASVNMDP--

>Rapgef2_[Mesocricetus_auratus]_880915360
STVEVATQLSMRNFELFRNIEPT----EYI-----DDLFK-L-KS----K-TSC-A----
-------------------NLKKFEEVINQETFWVASEILRETNQLKRMKIIKHFIKIA-
LHCRE-CKNFNSMFAIISGLNLAPVARLRTTWEKLPNKYEKLFQDLQDLFDPSRNMAKYR
NVLSGQNLQP-------PVIPL-FPVIKKDLTFL--------------------------
-----HEGND--SKV-------D---GLVNFE-KLRMIAKEI---RHVGRMASVNMDP--

>Rapgef2_[Metaseiulus_occidentalis]_391327442
-SGEVAAQLTLKDFEIFQQIEPT----EYV-----DDLFR-VFQS----R-YGA-PM---
--------------------LSKFADLVNREMFWVVTQIVSEPNVVRRMRTVKQFIKLA-
RYFRE-FKNFNSMFAILSGLGHQAVSRLKNTWDKLPSKYHRIFQDLQELMDPSRNMCRYR
TLVT--RLHP-------PLIPF-YPVVHKDLSFI--------------------------
-----HFGND--TLV-------D---GLVNFE-KLRMVSKEV---RTLMNMCSQPYVFPS
LASSSSNN--
>Rapgef2_[Microplitis_demolitor]_665818790
---EVAIQLTLQDFSIFRQIEST----EYV-----DDLFE-L-KS----R-YGV-PM---
--------------------LSQFAELVNREMFWVVTEVCSEHNLVRRSKIIKQFIKIA-
RQCKE-CKNFNSMFAIVSGLGHGAVSRLRASWEKLPSKYQRLFSDLQELMDPSRNMSKYR
QLVASEQTQP-------PIIPF-YPVVKKDLTFI--------------------------
-----HLGND--SRV-------E---NLVNFE-KLRMIAKEV---RTLTNMCSSPYD---

>Rapgef2_[Monodelphis_domestica]_612030789
STVEVATQLSMRNFELFRNIEPT----EYI-----DDLFK-L-KS----K-TSC-A----
-------------------NLKKFEEVINQETFWVASEILRETNQLKRMKTIKHFIKIA-
LHCRE-CKNFNSMFAIISGLNLAPVARLRTTWEKLPSKYEKLFQDLQDLFDPSRNMAKYR
NVLNSQNLQP-------PIIPL-FPVIKKDLTFL--------------------------
-----HEGND--SKV-------D---GLVNFE-KLRMIAKEI---RHVGRMASVNMDP--

>Rapgef2_[Mus_musculus]_887221696
STVEVATQLSMRNFELFRNIEPT----EYI-----DDLFK-L-KS----K-TSC-A----
-------------------NLKKFEEVINQETFWVASEILRETNQLKRMKIIKHFIKIA-
LHCRE-CKNFNSMFAIISGLNLAPVARLRTTWEKLPNKYEKLFQDLQDLFDPSRNMAKYR
NVLSGQNLQP-------PVIPL-FPVIKKDLTFL--------------------------
-----HEGND--SKV-------D---GLVNFE-KLRMIAKEI---RHVGRMASVNMDP--

>Rapgef2_[Mustela_putorius_furo]_859933738
STVEVATQLSMRNFELFRNIEPT----EYI-----DDLFK-L-KS----K-TSC-A----
-------------------NLKKFEEVINQETFWVASEILRETNQLKRMKIIKHFIKIA-
LHCRE-CKNFNSMFAIISGLNLAPVARLRTTWEKLPNKYEKLFQDLQDLFDPSRNMAKYR

NVLNSQNLQP-------PIIPL-FPVIKKDLTFL--------------------------
-----HEGND--SKV-------D---GLVNFE-KLRMIAKEI---RHVGRMASVNMDP--

>Rapgef2_[Myotis_davidii]_584034002
STVEVATQLSMRNFELFRNIEPT----EYI-----DDLFK-L-KS----K-TSC-A----
-------------------NLKKFEEVINQETFWVASEILRETNQLKRMKIIKHFIKIA-
LHCRE-CKNFNSMFAIISGLNLAPVARLRTTWEKLPNKYEKLFQDLQDLFDPSRNMAKYR
NVLNSQNLQP-------PIIPL-FPVIKKDLTFL--------------------------
-----HEGND--SKV-------D---GLVNFE-KLRMIAKEI---RHVGRMASVNMDP--

>Rapgef2_[Myotis_lucifugus]_558098423
STVEVATQLSMRNFELFRNIEPT----EYI-----DDLFK-L-KS----K-TSC-A----
-------------------NLKKFEEVINQETFWVASEILRETNQLKRMKIIKHFIKIA-
LHCRE-CKNFNSMFAIISGLNLAPVARLRTTWEKLPNKYEKLFQDLQDLFDPSRNMAKYR
NVLNSQNLQP-------PIIPL-FPVIKKDLTFL--------------------------
-----HEGND--SKV-------D---GLVNFE-KLRMIAKEI---RHVGRMASVNMDP--

>Rapgef2_[Nannospalax_galili]_674089172
STVEVATQLSMRNFELFRNIEPT----EYI-----DDLFK-L-KS----K-TSC-A----
-------------------NLKKFEEVINQETFWVASEILRETNQLKRMKIIKHFIKIA-
LHCRE-CKNFNSMFAIISGLNLAPVARLRTTWEKLPNKYEKLFQDLQDLFDPSRNMAKYR
NVLSGQNLQP-------PVIPL-FPVIKKDLTFL--------------------------
-----HEGND--SKV-------D---GLVNFE-KLRMIAKEI---RHVGRMASVNMDP--

>Rapgef2_[Nasonia_vitripennis]_1032757806
---EVAAQLTFQDFNIFRQIEST----EYV-----DDLFE-L-KS----K-YGV-PM---
--------------------LSQFAELVNREMFWVVTEVCSEHNIVRRSKIIKQFIKIA-
RQCKE-CKNFNSMFAIVSGLGHGAVSRLRASWEKLPSKYQRLFSDLQELMDPSRNMSKYR
QLVASEQTQP-------PIIPF-YPVVKKDLTFI--------------------------
-----HLGND--SRV-------E---GLVNFE-KLRMIAKEV---RTL------------

>Rapgef2_[Nothobranchius_furzeri]_1007706563
STVEVATQLSMRAFELFCAIEPT----EYI-----DDLFK-L-HS----K-TGS-V----
-------------------SLKRFEESINHETFWVATEVVRESNQLKRMKIVKHFIKIA-
LHCRE-CKNFNSMFAIISGLNLAPVSRLRGTWEKLPSKYEKLFGDLQDLFDPSRNMAKYR
NVLNNQNLQP-------PIIPL-FPVIKKDLTFL--------------------------
-----HEGND--SKV-------D---GLVNFE-KLRMIAKEI---RHVGRMASVNMDP--

>Rapgef2_[Ochotona_princeps]_837819646
STVEVATQLSMRNFELFRNIEPT----EYI-----DDLFK-L-KS----K-TSC-A----
-------------------NLKKFEEVINQETFWVASEILRETNQLKRMKIIKHFIKIA-
LHCRE-CKNFNSMFAIISGLNLAPVARLRTTWEKLPNKYEKLFQDLQDLFDPSRNMAKYR
NVLNSQNLQP-------PVIPL-FPVIKKDLTFL--------------------------
-----HEGND--SRV-------D---GLVNFE-KLRMIAKEI---RHVGRMASVNMDP--

>Rapgef2_[Octodon_degus]_820993492
STVEVSAQLSVRNFELFRNIEPT----EYI-----DDLFR-L-RS----K-TSC-A----
-------------------NLKRFEDVINQETFWVASEILRETNQLKRMKIIKHFIKIA-
LHCRE-CKNFNSMFAIISGLNLAPVARLRTTWEKLPNKYEKLFQDLQDLFDPSRNMAKYR
NVLNSQNLQP-------PIIPL-FPVIKKDLTFL--------------------------
-----HEGND--SKV-------D---GLVNFE-KLRMIAKEI---RHVGRMASVNMDP--

>Rapgef2_[Odobenus_rosmarus_divergens]_472349156
STVEVATQLSMRNFELFRNIEPT----EYI-----DDLFK-L-KS----K-TSC-A----

-------------------NLKKFEEVINQETFWVASEILRETNQLKRMKIIKHFIKIA-
LHCRE-CKNFNSMFAIISGLNLAPVARLRTTWEKLPNKYEKLFQDLQDLFDPSRNMAKYR
NVLNSQNLQP-------PIIPL-FPVIKKDLTFL--------------------------
-----HEGND--SKV-------D---GLVNFE-KLRMIAKEI---RHVGRMASVNMDP--

>Rapgef2_[Opisthocomus_hoazin]_700389884
STVEVATQLSMRNFELFRNIEPT----EYI-----DDLFK-L-KS----K-TGC-T----
-------------------NLKKFEEVINQETFWVASEILRETNQLKRMKIIKHFIKIA-
LHCRE-CKNFNSMFAIISGLNLAPVARLRTTWEKLPSKYEKLFQDLQDLFDPSRNMAKYR
NVLNSQNLQP-------PIIPL-FPVIKKDLTFL--------------------------
-----HEGND--SKV-------E---GLVNFE-KLRMIAKEI---RHVGRMASVNMDP--

>Rapgef2_[Ornithorhynchus_anatinus]_345307480
STVEVATQLSMRNFELFRNIEPT----EYI-----DDLFK-L-KS----K-TSC-V----
-------------------NLKKFEEVINQETFWVASEILRETNQLKRMKIIKHFIKIA-
LHCRE-CKNFNSMFAIISGLNLAPVARLRTTWEKLPSKYEKLFQDLQDLFDPSRNMAKYR
NVLNSQNLQP-------PIIPL-FPVIKKDLTFL--------------------------
-----HEGND--SKV-------D---GLVNFE-KLRMIAKEI---RHVGRMASVNMDP--

>Rapgef2_[Orussus_abietinus]_817220788
---EVAIQLTLQDFSIFRQIEST----EYV-----DDLFE-L-KS----R-YGV-PM---
--------------------LSQFAELVNREMFWVVTEVCSEHNLVRRSKIIKQFIKIA-
RQCKE-CKNFNSMFAIVSGLGHGAVSRLRASWEKLPSKYQRLFSDLQELMDPSRNMSKYR
QLVASEQTQP-------PIIPF-YPVVKKDLTFI--------------------------
-----HLGND--SRV-------E---NLVNFE-KLRMIAKEI---RTL------------

>Rapgef2_[Orycteropus_afer_afer]_634878373
STVEVATQLSMRNFELFRNIEPT----EYI-----DDLFK-L-KS----K-TNC-A----
-------------------NLKKFEEVINQETFWVASEILRETNQLKRMKIIKHFIKIA-
LHCRE-CKNFNSMFAIISGLNLAPVARLRTTWEKLPNKYEKLFQDLQDLFDPSRNMAKYR
NVLNSQNLQP-------PIIPL-FPVIKKDLTFL--------------------------
-----HEGND--SKV-------D---GLVNFE-KLRMIAKEI---RHVGRMASVNMDP--

>Rapgef2_[Oryctolagus_cuniculus]_655879045
STVEVATQLSMRNFELFRNIEPT----EYI-----DDLFK-L-KS----K-TSC-A----
-------------------NLKQFEEVINQETFWVASEILRETNQLKRMKIIKHFIKIA-
LHCRE-CKNFNSMFAIISGLNLAPVARLRTTWEKLPNKYEKLFQDLQDLFDPSRNMAKYR
NVLNSQNLQP-------PIIPL-FPVIKKDLTFL--------------------------
-----HEGND--SKV-------D---GLVNFE-KLRMIAKEI---RHVGRMASVNMDP--

>Rapgef2_[Ovis_aries_musimon]_803287877
STVEVATQLSMRNFELFRNIEPT----EYI-----DDLFK-L-KS----K-TSC-A----
-------------------NLKTFEEVINQETFWVASEILRETNQLKRMKIIKHFIKIA-
LHCRE-CKNFNSMFAIISGLNLAPVARLRTTWEKLPNKYEKLFQDLQDLFDPSRNMAKYR
NVLNSQNLQP-------PIIPL-FPVIKKDLTFL--------------------------
-----HEGND--SKV-------D---GLVNFE-KLRMIAKEI---RHVGRMASVNMDP--

>Rapgef2_[Ovis_aries]_803171614
STVEVATQLSMRNFELFRNIEPT----EYI-----DDLFK-L-KS----K-TSC-A----
-------------------NLKTFEEVINQETFWVASEILRETNQLKRMKIIKHFIKIA-
LHCRE-CKNFNSMFAIISGLNLAPVARLRTTWEKLPNKYEKLFQDLQDLFDPSRNMAKYR
NVLNSQNLQP-------PIIPL-FPVIKKDLTFL--------------------------
-----HEGND--SKV-------D---GLVNFE-KLRMIAKEI---RHVGRMASVNMDP--

```
>Rapgef2_[Pan_paniscus]_931570497
STVEVATQLSMRNFELFRNIEPT----EYI-----DDLFK-L-RS----K-TSC-A----
-------------------NLKRFEEVINQETFWVASEILRETNQLKRMKIIKHFIKIA-
LHCRE-CKNFNSMFAIISGLNLAPVARLRTTWEKLPNKYEKLFQDLQDLFDPSRNMAKYR
NVLNSQNLQP-------PIIPL-FPVIKKDLTFL--------------------------
-----HEGND--SKV-------D---GLVNFE-KLRMIAKEI---RHVGRMASVNMDP--
---------------------------------------------------------
>Rapgef2_[Pan_troglodytes]_694909391
STVEVATQLSMRNFELFRNIEPT----EYI-----DDLFK-L-RS----K-TSC-A----
-------------------NLKRFEEVINQETFWVASEILRETNQLKRMKIIKHFIKIA-
LHCRE-CKNFNSMFAIISGLNLAPVARLRTTWEKLPNKYEKLFQDLQDLFDPSRNMAKYR
NVLNSQNLQP-------PIIPL-FPVIKKDLTFL--------------------------
-----HEGND--SKV-------D---GLVNFE-KLRMIAKEI---RHVGRMASVNMDP--
---------------------------------------------------------
>Rapgef2_[Panthera_tigris_altaica]_987408706
STVEVATQLSMRNFELFRNIEPT----EYI-----DDLFK-L-KS----K-TSC-A----
-------------------NLKKFEEVINQETFWVASEILRETNQLKRMKIIKHFIKIA-
LHCRE-CKNFNSMFAIISGLNLAPVARLRTTWEKLPNKYEKLFQDLQDLFDPSRNMAKYR
NVLNSQNLQP-------PIIPL-FPVIKKDLTFL--------------------------
-----HEGND--SKV-------D---GLVNFE-KLRMIAKEI---RHVGRMASVNMDP--
---------------------------------------------------------
>Rapgef2_[Papilio_polytes]_909561101
---EVAVQLTLQDFCIFRQIEST----EYV-----DDLFE-L-KS----R-YGT-PM---
--------------------LSQFAGLVNKEMFWVVTEVVNEQNLVRRSKIVKQFIKVA-
RHCKE-CKNFNSMFAIISGLGHGAVSRLRLTWEKLPTKYIKLFSDLQMLMDPSRNMSKYR
QLVTTEQGRS-------PVIPF-YPVVRKDLTFI--------------------------
-----HLGND--TKV-------E---GMVNFE-KLRMIAKEV---RTLTNMCSSPYD---
---------------------------------------------------------
>Rapgef2_[Papio_anubis]_685543722
STVEVATQLSMRNFELFRNIEPT----EYI-----DDLFK-L-RS----K-TSC-A----
-------------------NLKRFEEVINQETFWVASEILRETNQLKRMKIIKHFIKIA-
LHCRE-CKNFNSMFAIISGLNLAPVARLRTTWEKLPNKYEKLFQDLQDLFDPSRNMAKYR
NVLNSQNLQP-------PIIPL-FPVIKKDLTFL--------------------------
-----HEGND--SKV-------D---GLVNFE-KLRMIAKEI---RHVGRMASVNMDP--
---------------------------------------------------------
>Rapgef2_[Parus_major]_998685096
STVEVATQLSMRNFELFRNIEPT----EYI-----DDLFK-L-KS----K-TGC-T----
-------------------NLKRFEEVINQETFWVASEILRETNQLKRMKIIKHFIKIA-
LHCRE-CKNFNSMFAIISGLNLAPVARLRTTWEKLPSKYEKLFQDLQDLFDPSRNMAKYR
NVLNSQNLQP-------PIIPL-FPVIKKDLTFL--------------------------
-----HEGND--SKV-------E---GLVNFE-KLRMIAKEI---RHVGRMASVNMDP--
---------------------------------------------------------
>Rapgef2_[Pelodiscus_sinensis]_558156869
STIEMAAQLSMRNFELFRNIEPT----EYI-----DDLFK-L-KS----K-TSC-A----
-------------------NLKKFEEVINQETFWVASEILRETNQLKRMKIIKHFIKIA-
LHCRE-CKNFNSMFAIISGLNLAPVARLRTTWEKLPSKYEKLFQDLQDLFDPSRNMAKYR
NVLNSQNLQP-------PIIPL-FPVIKKDLTFL--------------------------
-----HEGND--SKV-------E---GLVNFE-KLRMIAKEI---RHVGRMASVNIDP--
---------------------------------------------------------
>Rapgef2_[Peromyscus_maniculatus_bairdii]_1008761806
STVEVATQLSMRNFELFRSIEPT----EYI-----DDLFK-L-KS----K-TSC-A----
-------------------NLKTFEEVINQETFWVASEILRETNQLKRMKIIKHFIKIA-
LHCRE-CKNFNSMFAIISGLNLAPVARLRTTWEKLPNKYEKLFQDLQDLFDPSRNMAKYR
NVLSGQNLQP-------PVIPL-FPVIKKDLTFL--------------------------
```

-----HEGND--SKV-------D---GLVNFE-KLRMIAKEI---RHVGRMASVNMDP--

>Rapgef2_[Phaethon_lepturus]_723143942
STVEVATQLSMRNFELFRNIEPT----EYI-----DDLFK-L-KS----K-TGC-T----
-------------------NLKKFEEVINQETFWVASEILRETNQLKRMKIIKHFIKIA-
LHCRE-CKNFNSMFAIISGLNLAPVARLRTTWEKLPSKYEKLFQDLQDLFDPSRNMAKYR
NVLNSQNLQP-------PIIPL-FPVIKKDLTFL--------------------------
-----HEGND--SKV-------E---GLVNFE-KLRMIAKEI---RHVGRMASVNMDP--

>Rapgef2_[Physeter_catodon]_593730225
STVEVATQLSMRNFELFRNIEPT----EYI-----DDLFK-L-KS----K-ASC-A----
-------------------NLKKFEEVINQETFWVASEILREANQLKRMKIIKHFIKIA-
LHCRE-CKNFNSMFAIISGLNLAPVARLRTTWEKLPNKYEKLFQDLQDLFDPSRNMAKYR
NVLNSQNLQP-------PIIPL-FPVIKKDLTFL--------------------------
-----HEGND--SKV-------D---GLVNFE-KLRMIAKEI---RHVGRMASVNMDP--

>Rapgef2_[Plutella_xylostella]_768419725
---EVAVQLTLQDFCIFRQIEST----EYV-----DDLFE-L-NS----R-YGT-PM---
--------------------LSQFAELVNKEMFWVVTEVVTEQNPVRRSKIIKQFIKVA-
RHCKE-CKNFNSMFAIISGLGHGAVSRLRMSWEKLPTKYLKLFSDLQTLMDPSRNMSKYR
QLLTTEQVRS-------PVVPF-YPVVRKDLTFI--------------------------
-----HLGND--TKV-------E---GMVNFE-KLRMIAKEV---RTLVNMCSSPYD---

>Rapgef2_[Poecilia_formosa]_1025435386
STVEVATQLSMRAFELFCAIEPT----EYI-----DDLFK-L-RS----K-TGS-V----
-------------------SLKRFEESINHETFWVATEVVREPNQLKRMKIIKHFIKIA-
LHCRE-CKNFNSMFAIISGLNLAPVSRLRGTWEKLPSKYEKLFGDLQDLFDPSRNMAKYR
NVLNNQNLQP-------PIIPL-FPVIKKDLTFL--------------------------
-----HEGND--SKV-------D---GLVNFE-KLRMIAKEI---RHVGRMASVNMDP--

>Rapgef2_[Poecilia_latipinna]_961993309
STVEVATQLSMRAFELFCAIEPT----EYI-----DDLFK-L-RS----K-TGS-V----
-------------------SLKRFEESINHETFWVATEVVREPNQLKRMKIIKHFIKIA-
LHCRE-CKNFNSMFAIISGLNLAPVSRLRGTWEKLPSKYEKLFGDLQDLFDPSRNMAKYR
NVLNNQNLQP-------PIIPL-FPVIKKDLTFL--------------------------
-----HEGND--SKV-------D---GLVNFE-KLRMIAKEI---RHVGRMASVNMDP--

>Rapgef2_[Poecilia_reticulata]_1039360321
STVEVATQLSMRAFELFCAIEPT----EYI-----DDLFK-L-RS----K-TGS-V----
-------------------SLKRFEESINHETFWVATEVVREPNQLKRMKIIKHFIKIA-
LHCRE-CKNFNSMFAIISGLNLAPVSRLRGTWEKLPSKYEKLFGDLQDLFDPSRNMAKYR
NVLNNQNLQP-------PIIPL-FPVIKKDLTFL--------------------------
-----HEGND--SKV-------D---GLVNFE-KLRMIAKEI---RHVGRMASVNMDP--

>Rapgef2_[Pogonomyrmex_barbatus]_769831300
---EVAIQLTLQDFSIFRQIEST----EYV-----DDLFE-L-KS----R-YGV-PM---
--------------------LSQFAELVNREMFWVVTEVCSEHNLVRRSKIIKQFIKIA-
RQCKE-CKNFNSMFAIVSGLGHGAVSRLRASWEKLPTKYQRLFSDLQELMDPSRNMSKYR
QLVASEQTQP-------PIIPF-YPVVKKDLTFI--------------------------
-----HLGND--SRV-------E---GLVNFE-KLRMIAKEV---RTLTNMCSSPYD---

>Rapgef2_[Pongo_abelii]_686717383
STVEVATQLSMRNFELFRNIEPT----EYI-----DDLFK-L-RS----K-TSC-A----
-------------------NLKRFEEVINQETFWVASEILRETNQLKRMKIIKHFIKIA-

LHCRE-CKNFNSMFAIISGLNLAPVARLRTTWEKLPNKYEKLFQDLQDLFDPSRNMAKYR
NVLNSQNLQP-------PIIPL-FPVIKKDLTFL--------------------------
-----HEGND--SKV-------D---GLVNFE-KLRMIAKEI---RHVGRMASVNMDP--

>Rapgef2_[Propithecus_coquereli]_826315266
STVEVATQLSMRNFELFRNIEPT----EYI-----DDLFK-L-KS----K-TGC-A----
-------------------NLKKFEEVINQETFWVASEILRETNQLKRMKIIKHFIKIA-
LHCRE-CKNFNSMFAIISGLNLAPVARLRTTWEKLPNKYEKLFQDLQDLFDPSRNMAKYR
NVLNSQNLQP-------PIIPL-FPVIKKDLTFL--------------------------
-----HEGND--SKV-------D---GLVNFE-KLRMIAKEI---RHVGRMASVNMDP--

>Rapgef2_[Protobothrops_mucrosquamatus]_1002581465
STIEVATQLSMRNFELFRNIEPT----EYI-----DDLFK-L-KS----K-FNC-A----
-------------------NLKKFEEVINQETFWVASEILRETNQLKRMKIIKHFIKIA-
LHCRE-CKNFNSMFAIISGLNLAPVARLRTTWEKLPSKYEKLFQDLQDLFDPSRNMAKYR
NVLNSQNLQP-------PVIPL-FPVIKKDLTFL--------------------------
-----HEGND--SKV-------E---GLVNFE-KLRMIAKEI---RHVGRMASVNMDP--

>Rapgef2_[Pseudopodoces_humilis]_929412532
STVEVATQLSMRNFELFRNIEPT----EYI-----DDLFK-L-KS----K-TGC-T----
-------------------NLKKFEEVINQETFWVASEILRETNQLKRMKIIKHFIKIA-
LHCRE-CKNFNSMFAIISGLNLAPVARLRTTWEKLPSKYEKLFQDLQDLFDPSRNMAKYR
NVLNSQNLQP-------PIIPL-FPVIKKDLTFL--------------------------
-----HEGND--SKV-------E---GLVNFE-KLRMIAKEI---RHVGRMASVNMDP--

>Rapgef2_[Pterocles_gutturalis]_704502780
STVEVATQLSMRNFELFRNIEPT----EYI-----DDLFK-L-KS----K-TGC-T----
-------------------NLKKFEEVINQETFWVASEILRETNQLKRMKIIKHFIKIA-
LHCRE-CKNFNSMFAIISGLNLAPVARLRTTWEKLPSKYEKLFQDLQDLFDPSRNMAKYR
NILNSQNLQP-------PIIPL-FPVIKKDLTFL--------------------------
-----HEGND--SKV-------E---GLVNFE-KLRMIAKEI---RHVGRMASVNMDP--

>Rapgef2_[Rhinopithecus_roxellana]_724803574
STVEVATQLSMRNFELFRNIEPT----EYI-----DDLFK-L-RS----K-TSC-A----
-------------------NLKRFEEVINQETFWVASEILRETNQLKRMKIIKHFIKIA-
LHCRE-CKNFNSMFAIISGLNLAPVARLRTTWEKLPNKYEKLFQDLQDLFDPSRNMAKYR
NVLNSQNLQP-------PIIPL-FPVIKKDLTFL--------------------------
-----HEGND--SKV-------D---GLVNFE-KLRMIAKEI---RHVGRMASVNMDP--

>Rapgef2_[Rousettus_aegyptiacus]_1012013897
STVEVATQLSMRNFELFRNIEPT----EYI-----DDLFK-L-KS----K-TSC-A----
-------------------NLKKFEEVINQETFWVASEILRETNQLKRMKIIKHFIKIA-
LHCRE-CKNFNSMFAIISGLNLAPVARLRTTWEKLPNKYEKLFQDLQDLFDPSRNMAKYR
NVLNSQNLQP-------PIIPL-FPVIKKDLTFL--------------------------
-----HEGND--SKV-------D---GLVNFE-KLRMIAKEI---RHVGRMASVNMDP--

>Rapgef2_[Saimiri_boliviensis_boliviensis]_403272230
STVEVATQLSMRNFELFRNIEPT----EYI-----DDLFK-L-RS----K-TSC-A----
-------------------NLKRFEEVINQETFWVASEILRETNQLKRMKIIKHFIKIA-
LHCRE-CKNFNSMFAIISGLNLAPVARLRTTWEKLPNKYEKLFQDLQDLFDPSRNMAKYR
NVLNSQNLQP-------PIIPL-FPVIKKDLTFL--------------------------
-----HEGND--SKV-------D---GLVNFE-KLRMIAKEI---RHVGRMASVNMDP--

>Rapgef2_[Sarcophilus_harrisii]_821490707

STVEVATQLSMRNFELFRNIEPT----EYI-----DDLFK-L-KS----K-TSC-V----
-------------------NLKKFEEVINQETFWVASEILRETNQLKRMKTIKHFIKIA-
LHCRE-CKNFNSMFAIISGLNLAPVARLRTTWEKLPSKYEKLFQDLQDLFDPSRNMAKYR
NVLNSQNLQP-------PIIPL-FPVIKKDLTFL--------------------------
-----HEGND--SKV-------D---GLVNFE-KLRMIAKEI---RHVGRMASVNMDP--

>Rapgef2_[Schistosoma_haematobium]_844865153
--NELAARLTIDDYEIFRSVQST----EYI-----DEIFG-LSNA-HVNNISSC-VVGNN
SNSSNT------GYATGHENLDRFTDLVNREAYWAPTEICNETNLNRRVDLLKRFIKLA-
KLCRE-LRNFNTMFCLLVGLHQTPVERLKQTWDRLPNKYNELASYLQMVLDTSRNFHHYR
TLLSANNVSV-------PVLPY-LPLVLKDLTFI--------------------------
-----HLGNP--SCT-------PD--GLINFV-KLRMLAKEV---RAICR----------

>Rapgef2_[Scleropages_formosus]_938084963
STVEVATQLSMRNFELFCNIEPT----EYI-----DDLFK-L-RS----R-TGS-A----
-------------------NLKKFEEVINQETFWVASEIVHEPNQLKRMKIVKHFIKIA-
LHCRE-CKNFNSMFAIISGLNLAPVARLRGTWEKLPSKYEKLFQDLQDLFDPSRNMAKYR
NVLSNQNLQP-------PIIPL-FPVIKKDLTFL--------------------------
-----HEGND--SKV-------D---GLVNFE-KLRMIAKEI---RHVGRMASVNMDP--

>Rapgef2_[Sinocyclocheilus_anshuiensis]_1024942047
SCMEIANQLSARNYLLFSGIEPT----DYI-----TDLFK-L-HP----Q-EPP-T----
-------------------NLRNFEDLVNQETFWVATEIVQETNLAKRVKIIKHFIKIA-
LHCRD-CKNFNSMFAIISGFNLAPVSRLRSTWERLPGKYEKLLAELQDVFDPSRNMAKYR
NLLNKHNLQP-------PVIPL-FPVIKKDLTFL--------------------------
-----HEGNK--SKV-------D---GLVNFE-KLRMIAREV---RHV------------

>Rapgef2_[Sinocyclocheilus_grahami]_1020462828

--ILKVFIKIA-
LHCRD-CKNFNSMFAIISGFNLAPVSRLRSTWERLPGKYEKLLAELQDVFDPSRNMAKYR
NLLNKHNLQP-------PVIPL-FPVIKKDLTFL--------------------------
-----HEGNK--SKV-------D---GLVNFE-KLRMIAREI---R--------------

>Rapgef2_[Sinocyclocheilus_rhinocerous]_1025229282
STVEVATQLSMRAFELFCAIEPT----EYI-----DDLFK-L-KT----RLTRP-P----
-------------------SLKLFEEAINRETFWVATEVVRETYQLKRMKIIKHFIKIA-
LHCRE-CKNFNSMFAIISGLNLAPVSRLRGTWEKLPSKYEKLFSDLQDLFDPSRNMAKYR
NLLNSQNLQP-------PIIPL-FPVIKKDLTFL--------------------------
-----HEGND--SKV-------D---GLVNFE-KLRMIAKEI---RHV------------

>Rapgef2_[Sorex_araneus]_505792953
STVEVATQLSMRNFELFRNIEPT----EYI-----DDLFK-L-KS----K-TSC-A----
-------------------NLKKFEEVINQETFWVASEILRETNQLKRMKIIKHFIKIA-
LHCRE-CKNFNSMFAIISGLNLAPVARLRTTWEKLPNKYEKLFQDLQDLFDPSRNMAKYR
NVLNSQNLQP-------PIIPL-FPVIKKDLTFL--------------------------
-----HEGND--SKV-------E---GLVNFE-KLRMIAKEI---RHVGRMASVNMDP--

>Rapgef2_[Strongylocentrotus_purpuratus]_780098595
-SVEVAVQFSLRDYAIFEAIEPT----DYI-----EDLFN-L-KP----Q-GKK-DID--
-------------------RLKAYEELVNLEMFWVISEVVSEPSIMKRAKILKNFIKIA-
RHFKD-CHNFNSLFAVISGLGHGSVSRLRLAWDKVPSKYIKMFEDLQALLDPSRNMSKYR
NLINAENVQP-------PLMPF-FPIVKKDLTFI--------------------------
-----HLGND--SYV-------E---GLINFE-KQRMVAREV---RYFHSMGNAPYDP--

```
--------------------------------------------------------
>Rapgef2_[Struthio_camelus_australis]_697489370
STVEVATQLSMRNFELFRNIEPT----EYI-----DDLFK-L-KS----K-TGC-T----
-------------------NLKKFEEVINQETFWVASEILRETNQLKRMKIIKHFIKIA-
LHCRE-CKNFNSMFAIISGLNLAPVARLRTTWEKLPSKYEKLFQDLQDLFDPSRNMAKYR
NVLNSQNLQP-------PIIPL-FPVIKKDLTFL--------------------------
-----HEGND--SKV-------E---GLVNFE-KLRMIAKEI---RHVGRMASVNMDP--
--------------------------------------------------------
>Rapgef2_[Sturnus_vulgaris]_959071486
STVEVATQLSMRNFELFRNIEPT----EYI-----DDLFK-L-KS----K-TGC-T----
-------------------NLKRFEEVINQETFWVASEILRETNQLKRMKIIKHFIKIA-
LHCRE-CKNFNSMFAIISGLNLAPVARLRTTWEKLPSKYEKLFQDLQDLFDPSRNMAKYR
NVLNSQNLQP-------PIIPL-FPVIKKDLTFL--------------------------
-----HEGND--SKV-------E---GLVNFE-KLRMIAKEI---RHVGRMASVNMDP--
--------------------------------------------------------
>Rapgef2_[Sus_scrofa]_927167431
STVEVATQLSMRNFELFRNIEPT----EYI-----DDLFK-L-KS----K-TSC-A----
-------------------NLKKFEEVINQETFWVASEILRETNQLKRMKIIKHFIKIA-
LHCRE-CKNFNSMFAIISGLNLAPVARLRTTWEKLPNKYEKLFQDLQDLFDPSRNMAKYR
NVLNSQNLQP-------PIIPL-FPVIKKDLTFL--------------------------
-----HEGND--SKV-------D---GLVNFE-KLRMIAKEI---RHVGRMASVNMDP--
--------------------------------------------------------
>Rapgef2_[Thamnophis_sirtalis]_927176022
STIEVATQLSMRNFELFRNIEPT----EYI-----DDLFK-L-KS----K-FNC-A----
-------------------NLKKFEEVINQETFWVASEILRETNQLKRMKIIKHFIKIA-
LHCRE-CKNFNSMFAIISGLNLAPVARLRTTWEKLPSKYEKLFQDLQDLFDPSRNMAKYR
NVLNSQNLQP-------PIIPL-FPVIKKDLTFL--------------------------
-----HEGND--SKV-------E---GLVNFE-KLRMIAKEI---RHVGRMASVNMDP--
--------------------------------------------------------
>Rapgef2_[Tinamus_guttatus]_719747879
STVEVATQLSMRNFELFRNIEPT----EYI-----DDLFK-L-KS----K-TGC-T----
-------------------NLKKFEEVINQETFWVASEILRETNQLKRMKIIKHFIKIA-
LHCRE-CKNFNSMFAIISGLNLAPVARLRTTWEKLPSKYEKLFQDLQDLFDPSRNMAKYR
NVLNSQNLQP-------PIIPL-FPVIKKDLTFL--------------------------
-----HEGND--SKV-------E---GLVNFE-KLRMIAKEI---RHVGRMASVNMDP--
--------------------------------------------------------
>Rapgef2_[Toxocara_canis]_734559244
--------------------NMS----KYR-----QHLSE-V-SD----E-----P----
-------------------PV---------EMWWVPNEVCRERSVQKRAKLVKKLIKVA-
RHCRD-FRNFNSMFAIMSGLEKPAVRRLHHTWDRVPNKYVKMFEDMQQLVDPSRNMSKYR
QHLSEVSDEP-------PVVPI-YPVLKKDLTFC--------------------------
-----HEGNP--TYC-------G---RLVNFE-KLRMIARAV---R--------------
--------------------------------------------------------
>Rapgef2_[Trachymyrmex_septentrionalis]_1009425222
---EVAIQLTLQDFSIFRQIEST----EYV-----DDLFE-L-KS----R-YGV-PM---
--------------------LSQFAELVNREMFWVVTEVCSEHNLVRRS-----------
-QCKE-CKNFNSMFAIVSGLGHGSVSRLRASWEKLPTKYQRLFSDLQELMDPSRNMSKYR
QLVASEQTQP-------PIIPF-YPVVKKDLTFI--------------------------
-----HLGND--SRV-------E---SLVNFE-KLRMIAKEV---RTLTNMCSSPYD---
--------------------------------------------------------
>Rapgef2_[Tribolium_castaneum]_1008434452
---EVAIQLTLQDFSIFRQIEPT----EYI-----DELFE-L-KS----K-YGT-PM---
--------------------LEQFAALVNKEMFWVVTEVCSEHNPVRRMKIIKQFIKVAS
KQCKE-CKNFNSMFAIISGXGHGAVSRLRQSWDKLPGKYQRVFSDLQQLMDPSRNMSKYR
```

QLVSAEQSQP-------PIIPF-YPVVKKDLTFI--------------------------
-----HLGND--SQV-------E---GLVNFE-KLRMIAKEV---RSLSNMCSSPYD---

>Rapgef2_[Trichechus_manatus_latirostris]_471397362
STVEVATQLSMRNFELFRNIEPT----EYI-----DDLFK-L-KS----K-TSC-A----
-------------------NLKKFEEVINQETFWVASEILREPNQLKRMKIIKHFIKIA-
LHCRE-CKNFSSMFAIISGLNLAPVARLRTTWEKLPNKYEKLFQDLQDLFDPSRNMAKYR
NVLNSQNLQP-------PIIPL-FPVIKKDLTFL--------------------------
-----HEGND--SKV-------D---GLVNFE-KLRMIAKEI---RHVGRMASVNMDP--

>Rapgef2_[Trichogramma_pretiosum]_936679530
-AGEVAIQLTLQDYSIFRQIEST----EYV-----DDLFE-L-KS----K-YGT-PM---
--------------------LRQFAELVNREMFWVVTEVCSEHNVVRRSKIIKQFIKIA-
RQCKE-CKNFNSMFAIISGLGHGAVSRLRSSWEKLPSKYQRLFSDLQELMDPSRNMSKYR
QLVASEQTQPA------PIIPF-YPVVKKDLTFI--------------------------
-----HLGND--SEV-------E---GLVNFE-KLRMIAKEV---RNL------------

>Rapgef2_[Tupaia_chinensis]_562872629
STVEVATQLSMRNFELFRNIEPT----EYI-----DDLFR-L-RS----K-GSG-A----
-------------------NLKRFEDVINQETFWVASEILRETNQLKRTKIVKHFIKIA-
LHCRE-CKNFNSMFAVISGLNLAPVARLRTTWEKLPNKYEKLFQDLQDLFDPSRNMAKYR
NVLSSQSLQP-------PIIPL-FPVIKKDLTFL--------------------------
-----HEGND--SKV-------D---GLVNFE-KLRMIAKEI---RHVGRMASVNMDP--

>Rapgef2_[Tursiops_truncatus]_470634065
STVEVATQLSMRNFELFRNIEPT----EYI-----DDLFK-L-RS----K-TSC-A----
-------------------NLKKFEEVINQETFWVASEILRETNQLKRMKTIKHFIKIA-
LHCRE-CKNFNSMFAIISGLNLAPVARLRTTWEKLPNKYEKLFQDLQDLFDPSRNMAKYR
NVLNSQNLQP-------PIIPL-FPVIKKDLTFL--------------------------
-----HEGND--SKV-------D---GLVNFE-KLRMIAKEI---RHVGRMASVNMDP--

>Rapgef2_[Tyto_alba]_701382803
STVEVATQLSMRNFELFRNIEPT----EYI-----DDLFK-L-KS----K-TGC-T----
-------------------NLKKFEEVINQETFWVASEILRETNQLKRMKIIKHFIKIA-
LHCRE-CKNFNSMFAIISGLNLAPVARLRTTWEKLPSKYEKLFQDLQDLFDPSRNMAKYR
NVLNSQNLQP-------PIIPL-FPVIKKDLTFL--------------------------
-----HEGND--SKV-------E---GLVNFE-KLRMIAKEI---RHVGRMASVNMDP--

>Rapgef2_[Ursus_maritimus]_670985378
STVEVATQLSMRNFELFRNIEPT----EYI-----DDLFK-L-KS----K-TSC-A----
-------------------NLKKFEEVINQETFWVASEILRETNQLKRMKIIKHFIKIA-
LHCRE-CKNFNSMFAIISGLNLAPVARLRTTWEKLPNKYEKLFQDLQDLFDPSRNMAKYR
NVLNSQNLQP-------PIIPL-FPVIKKDLTFL--------------------------
-----HEGND--SKV-------D---GLVNFE-KLRMIAKEI---RHVGRMASVNMDP--

>Rapgef2_[Vicugna_pacos]_970736793
STVEVATQLSMRNFELFRNIEPT----EYI-----DDLFK-L-RS----K-TSC-A----
-------------------NLKKFEEVINQETFWVASEILRETNQLKRMKIIKHFIKIA-
LHCRE-CKNFNSMFAIISGLNLAPVARLRTTWEKLPNKYEKLFQDLQDLFDPSRNMAKYR
NVLNSQNLQP-------PIIPL-FPVIKKDLTFL--------------------------
-----HEGND--SKV-------E---GLVNFE-KLRMIAKEI---RHVGRMASVNMDP--

>Rapgef2_[Vollenhovia_emeryi]_795102055
---EVAIQLTLQDFSIFRQIEST----EYV-----DDLFE-L-KS----R-YGV-PM---

--------------------LSQFAELVNREMFWVVTEVCSEHNLVRRSKIIKQFIKIA-
RQCKE-CKNFNSMFAIVSGLGHGSVSRLRASWEKLPTKYQRLFSDLQELMDPSRNMSKYR
QLVASEQTQP-------PIIPF-YPVVKKDLTFI--------------------------
-----HLGND--SRV-------E---SLVNFE-KLRMIAKEV---RTLTNMCSSPYD---

>Rapgef2_[Xenopus_laevis]_147907136
STIEVATQLSMRNFELFRNIEPT----EYI-----DDLFK-L-KM----K-SGC-A----
-------------------NLKNFEEVINQETFWVASEILRETNQLKRMKIIKHFIKIA-
LHCRE-CKNFNSMFAIISGLNLAPVSRLRATWEKLPSKYEKLFQDLQDLFDPSRNMAKYR
NVLNSQNLQP-------PIIPL-FPVIKKDLTFL--------------------------
-----HEGND--SKV-------D---GLVNFE-KLRMIAKEI---RHVGRMASVNMDP--

>Rapgef2_[Xenopus_tropicalis]_847085554
STIEVATQLSMRNFELFRNIEPT----EYI-----DDLFK-L-KM----K-TGC-T----
-------------------NLKNFEEVINQETFWVASEILRETNQLKRMKIIKHFIKIA-
LHCRE-CKNFNSMFAIISGLNLAPVSRLRTTWEKLPSKYEKLFQDLQDLFDPSRNMAKYR
NVLNSQNLQP-------PIIPL-FPVIKKDLTFL--------------------------
-----HEGND--SKV-------D---GLVNFE-KLRMIAKEI---RHVGRMASVNMDP--

>Rapgef2_[Zonotrichia_albicollis]_542155070
STVEVATQLSMRNFELFRNIEPT----EYI-----DDLFK-L-KS----K-TGC-T----
-------------------NLKRFEEVINQETFWVASEILRETNQLKRMKIIKHFIKIA-
LHCRE-CKNFNSMFAIISGLNLAPVARLRTTWEKLPSKYEKLFQDLQDLFDPSRNMAKYR
NVLNSQNLQP-------PIIPL-FPVIKKDLTFL--------------------------
-----HEGND--SKV-------E---GLVNFE-KLRMIAKEI---RHVGRMASVNMDP--

>Rapgef2_[Zootermopsis_nevadensis]_646721548
---EVAIQLTLQDFSIFRQIEST----EYV-----DDLFE-L-KS----R-YGV-PM---
--------------------LSQFAELVNREMFWVVTEVCSEHNLVRRSKIIKQFIKVA-
RQCKE-CKNFNSMFAIVSGLGHGAVSRLRLSWEKLPSKYQRLFSDLQDLMDPSRNMSKYR
QLVSSEQSQP-------PIIPF-YPVVKKDLTFI--------------------------
-----HLGND--SKI-------E---GLINFE-KLRMIAKEV---RALTNMCSSPYD---

>Rapgef3_[Acinonyx_jubatus]_961722103
SAKDLAGQLTDHDWNLFNSIHQV----ELI-----HYVLG-PQHL----R--DV-TTA--
-------------------NLERFMRRFNELQYWVATELCLCPVPGLRAQLLRKFIKLA-
AHLKE-QKNLNSFFAVMFGLSNSAISRLAHTWERLPHKVRKLYSALERLLDPSWNHRVYR
LALTK--LSP-------PIIPF-MPLLLKDMTFI--------------------------
-----HEGNH--TLV-------E---NLINFE-KMRMMARAV---RMLHHC---------

>Rapgef3_[Ailuropoda_melanoleuca]_752406281
SAKDLAGQLTDHDWNLFNSIHQV----ELI-----HYVLG-PQHL----R--DV-TTA--
-------------------NLERLMRRFNELQYWVATELCLCPVPGLRAQLLRKFIKLA-
AHLKE-QKNLNSFFAVMFGLSNSAVSRLAHTWERLPHKVRKLYAALERLLDPSWNHRVYR
LALTK--LSP-------PIIPF-MPLLLKDMTFI--------------------------
-----HEGNH--TLV-------E---NLINFE-KMPLPHSLVCGEDSLHYVTWQAEPFRV
SSALSPPRSLFFLPPPDAPPVPLSPLRSRVSHLHEDSQAAR----TST---CSEQS
>Rapgef3_[Alligator_mississippiensis]_950944900
SSKDLASQLTDHDWTLFSSVHQV----ELL-----YHVLG-PQKF----P--GA-TTA--
-------------------NLERIMRRFNELQYWVSTELCLCGDVARRAQLLRKFIKLA-
AHLKE-QKNLNSFFAVMFGLSNTAVSRLAKTWERLPHKVRKLHAVLERMLDPSWNHRVYR
LAVAK--LSP-------PIFPF-LPLLLKDLTFI--------------------------
-----HEGNR--TLS-------E---NLINFE-KMRMMAKAV---RILHRCRSHF-----
-------------------HTPVSPLRSRSPHRLEDPKALR----ASTSDFCSK--

```
>Rapgef3_[Alligator_sinensis]_944358354
SSKDLASQLTEHDWTLFSSVHQV----ELL-----YHVLG-PQKF----P--GA-TTA--
-------------------NLERIMRRFNELQYWVSTELCLCGDVARRAQLLRKFIKLA-
AHLKE-QKNLNSFFAVMFGLSNTAVSRLAKTWEKLPHKVRKLHAVLERMLDPSWNHRVYR
LAVAK--LSP-------PIFPF-LPLLLKDLTFI--------------------------
-----HEGNR--TMS-------E---NLINFE-KMRMMAKAV---RILHRCRSHV-----
-------------------HTPVSPLRSRSPHRLEDPKALR----AST---CSEQS
>Rapgef3_[Anolis_carolinensis]_327279590
SSKDLANQLTEHDWNLFKSIHQV----ELV-----YYIFG-PQKF----P--NA-TTA--
-------------------NLERFLRRFNELQFWVTTELCLCPDVIKRAQLLRKFIKMA-
AHLKD-QKNLNTFFAVMFGLSHSAVSRLSKTWEKLPHKTRKLFNTMERMLDPSWNHRIYR
LAMAK--LTP-------PMIPF-MPLLLKDMTFI--------------------------
-----HEGNR--TSV-------E---NLVNFE-KLHMMAKAV---HLVHHCRSLP-----
-------------------NLPLSPLRSRPQHLLEEARAMR----AST---CSEQS
>Rapgef3_[Anser_cygnoides_domesticus]_902933497
SSKDLASHLTDYDWNLFKSIHQV----EMI-----HYIVG-PQKF----H--EV-TTA--
-------------------NLERVMRRFNELQYWVATELCLCPEVGRRAQLLRKFIKLA-
AHLKE-QKNLNSFFAVMFGVSNTAVSRLAKTWERLPHKIRKLHAALERMLDPSWNHRVYR
LAVAK--LSP-------PIIPF-VPLLLK-------------------------------
------------------------------------------------------------
--------------------------------------------------------
>Rapgef3_[Aptenodytes_forsteri]_686593488
SSKDLASHLTDYDWNLFKSIHQV----EMI-----HYIVG-PQKF----H--DV-TTA--
-------------------NLERVMRRFNELQYWVATELCLCPEVGRRAQLLRKFIKLA-
AHLKE-QKNLNSFFAVMFGVSNTAVSRLAKTWERLPHKIRKLHSALERMLDPSWNHRVYR
LAVAK--LSP-------PIIPF-VPLLLKDMTFI--------------------------
-----HEGNR--TLA-------E---NLINFE-KMHMMAKTV---RVLQRCRGDA-----
-------------------HAPLSPLRNRSPHRPEDPKAVR----IST---CSEQS
>Rapgef3_[Apteryx_australis_mantelli]_926500193
SSKDLASHLTDYDWNLFKSIHQV----EMI-----HYIIR-PQKF----H--DV-TTA--
-------------------NLERVMRRFNELQYWVATELCLCSEVGRRAQLLRKFIKLA-
AHLKE-QKNLNSFFAVMFGVSNTAVSRLAKTWERLPHKIRKLHSALERMLDPSWNHRVYR
LAVAK--LSP-------PIIPF-VPLLLKDMTFI--------------------------
-----HEGNR--TLA-------E---NLINFE-KMHMMAKTV---RVLQRCRGHA-----
-------------------HAPVSPLRSRSPHRLEDPKAVR----IST---CSEQS
>Rapgef3_[Aquila_chrysaetos_canadensis]_768403048
SSKDLASHLTDYDWNLFKSIHQV----EMI-----HYIVG-PQKF----H--DV-TTA--
-------------------NLERVMRRFNELQYWVATELCLCPEVGRRAQLLRKFIKLA-
AHLKE-QKNLNSFFAVMFGVSNTAVSRLAKTWERLPHKIRKLHSALERMLDPSWNHRVYR
LAVAK--LSP-------PIIPF-VPLLLKDMTFI--------------------------
-----HEGNR--TLA-------E---NLINFE-KM-------------------------
--------------------------------------------------------
>Rapgef3_[Austrofundulus_limnaeus]_928045560
SSKDIGIELTNYDWELFTAMHEV----ELV-----YYIFG-RHKF----P--GA-ITA--
-------------------NLERFVRHFNEVQYWVVTELCLCEDLVKRAVLLKKFIKIA-
SVLKD-QKNLNLFFAVMFGLGNSAVQRLYKTWERIPSKTKRIYCTYERLMDPSRNHRAYR
LAVTK--LSP-------PYIPF-MPLLLKDMTFI--------------------------
-----HEGNP--NCL-------D---KLVNFE-KMRMLAKTV---KIVRGCRSQP-----
------------------Y-VPSSPQRGLADRIFLEGP-TR----LSTS---SE--
>Rapgef3_[Balaenoptera_acutorostrata_scammoni]_594662221
STKDLAGQLTDHDWSLFNSIHQV----ELI-----HYVLG-PQPL----R--DV-TTA--
-------------------NLERFMRRFNELQYWVATELCLCPVPGLRAQLLRKFIKLA-
AHLKE-QKNLNSFFAIMFGLSNSAISRLAQTWERLPHKVRKLYSALERLLDPSWNHRVYR
LALTK--LSP-------PLIPF-MPLLLKDMTFI--------------------------
```

-----HEGNH--TLV-------E---NLINFE-KMRMMARAA---RMLHHCRSHS-----
-------------------SVPLSPLRSRVSHLHEDSQATR----ISM---CSEQS
>Rapgef3_[Bison_bison_bison]_742108280
STKDLAGQLTDHDWSLFNSIHQV----ELI-----HYVLG-PQHL----R--DV-TTA--
-------------------NLERFMRRFNELQYWVATELCLCSVPGLRAQLLRKFIKLA-
AHLKE-QKNLNSFFAIMFGLSNSAISRLAHTWERLPHKVRKLYSALERLLDPSWNHRVYR
LALTK--LSP-------PLIPF-MPLLLKDMTFI-------------------------
-----HEGNH--TLV-------E---NLINFE-KMRMMARAA---RMLHHCRSHS-----
-------------------NVPLSPLRSRVSHLHEDSQAVR----VST---CSEQS
>Rapgef3_[Bos_mutus]_555987329
STKDLAGQLTDHDWSLFNSIHQV----ELI-----HYVLG-PQHL----R--DV-TTA--
-------------------NLERFMRRFNELQYWVATELCLCSVPGLRAQLLRKFIKLA-
AHLKE-QKNLNSFFAIMFGLSNSAISRLAHTWERLPHKVRKLYSALERLLDPSWNHRVYR
LALTK--LSP-------PLIPF-MPLLLKDMTFI-------------------------
-----HEGNH--TLV-------E---NLINFE-KMRMMARAA---RMLHHCRSHS-----
-------------------NVPLSPLRSRVSHLHEDSQAVR----VST---CSEQS
>Rapgef3_[Bos_taurus]_528949062
STKDLAGQLTDHDWSLFNSIHQV----ELI-----HYVLG-PQHL----R--DV-TTA--
-------------------NLERFMRRFNELQYWVATELCLCSVPGLRAQLLRKFIKLA-
AHLKE-QKNLNSFFAIMFGLSNSAISRLAHTWERLPHKVRKLYSALERLLDPSWNHRVYR
LALTK--LSP-------PLIPF-MPLLLKDMTFI-------------------------
-----HEGNH--TLV-------E---NLINFE-KMRMMARAA---RMLHHCRSHS-----
-------------------NVPLSPLRSRVSHLHEDSQAVR----VST---CSEQS
>Rapgef3_[Bubalus_bubalis]_594071307
STKDLAGQLTDHDWSLFNSIHQV----ELI-----HYVLG-PQHL----R--DV-TTA--
-------------------NLERFMRRFNELQYWVATELCLCSVPGLRAQLLRKFIKLA-
AHLKE-QKNLNSFFAVMFGLSNSAISRLAHTWERLPHKVRKLYSALERLLDPSWNHRVYR
LALTK--LSP-------PLIPF-MPLLLKDVTFI-------------------------
-----HEGNH--TLV-------E---NLINFE-KMRMMARAA---RMLHHCRSHS-----
-------------------NVPLSPLRSRVSHLHEDSQAVR----VST---CSEQS
>Rapgef3_[Caenorhabditis_briggsae]_268573060
---------------------------ELL-----YQVIG-RESF--PQS-----MPF--
-------------------NLDLLVRRFNEVQHWSTTEILLA-TEDNRVEILKKFIHIA-
TIARE-YRDLLTVFAITLGLSHTSVSRLTLTWNKLPPATFKTFSELENLLDPTRNHRMYR
LLVSK--IAT-------PYIPF-VPLILKDLMFI-------------------------
-----HQGNK--SFY-------N---GLVNFE-KMHMFAKIF---RNFRQCKS--HMNDT
TDHQ------------------YVEPQSLIRN-LRVIDNQKILIQLSYG---IEPK
>Rapgef3_[Caenorhabditis_remanei]_308501823
---------------------------ELL-----YQVIG-RESF--PLS-----MPF--
-------------------NLDLLVRRFNEVQHWSTTEILLA-SEENRVEILKKFIAIA-
TIARE-YRDLLTVFAITLGLSHTSVSRLTLTWSKLPPITLKTFSELEHLLDPTRNHRMYR
LMVSK--MAS-------PYIPF-VPLILKDLMFI-------------------------
-----HQGNK--SFY-------N---GLVNFE-KMHMFAKIF---RNFRQCKS--QMNDS
SDHE------------------YVEPQSLIRN-LRVIDNQKKLMQISYE---IEPK
>Rapgef3_[Callithrix_jacchus]_675676220
SAKDLAGQLTDQDWSLFNSVHQV----ELI-----HYVLG-PQHL----R--DV-TTA--
-------------------NLERFMRRFNQLQYWVATELCLCVAPGPRAQLLRKFIKLA-
AHLKE-QKNLNSFFAVMFGLSNSAISRLAHTWERLPHKVRKLYSALERLLDPSWNHRVYR
LALAK--LSP-------PVIPF-MPLLLKDMTFI-------------------------
-----HEGNH--TLV-------E---NLINFE-KMRMMARAS---RMLHHCRSHN-----
-------------------PVPLSPLRSRVSHLHEDSQVAR----IST---CSEQS
>Rapgef3_[Callorhinchus_milii]_632985555

---------MNSFFAVAFGLSNSAVSRLSKTWEGLTNKTRRVYQSFERLMDPSRNHRAYR
MVAGK--LSL-------PFLPF-MPLLIKDLTFI-------------------------
-----EEGNK--TFV-------N---NLVNFE-KMRMIARTV---NLFRRCRTHSQMIPQ
KMEPEISSAQISTTSDQS-------------------------------------
>Rapgef3_[Camelus_dromedarius]_744616231
STKDLAGQLTDHDWNLFNSIHQV----ELI-----HYVLG-PQHL----R--DV-TTA--
-------------------NLERFMRRFNELQYWVATELCLCPVPGLRAQLLRKFIKLA-
AHLKE-QKNLNSFFAIMFGLSNSAISRLAHTWERLPHKVRKLYSALERLLDPSWNHRVYR
LALTK--LSP-------PVIPF-MPLLLKDMTFI-------------------------
-----HEGNH--TLV-------E---NLINFE-KMRMMARAA---RMLHHCRSHS-----
-------------------NVPLSPLRSRVSHLHEDSQATR----IST---CSEQS
>Rapgef3_[Canis_lupus_familiaris]_153791885
SAKDLAGQLTDHDWNLFNSIHQV----ELI-----HYVLG-PQHL----R--DV-TTA--
-------------------NLERFMRRFNELQYWVATELCLCPVPGLRAQLLRKFIKLA-
AHLKE-QKNLNSFFAVMFGLSNSAISRLAHTWERLPHKVRKLYSALERLLDPSWNHRVYR
LALTK--LSP-------PIIPF-MPLLLKDMTFI-------------------------
-----HEGNH--TLV-------E---NLINFE-KMRMMARAV---RMLHHCRSHS-----
-------------------NVPLSPLRSRVSHLHEDSQAAR----TST---CSEQS
>Rapgef3_[Capra_hircus]_926691591
STKDLAGQLTDHDWSLFNSIHQV----ELI-----HYVLG-PQHL----R--DV-TTA--
-------------------NLERFMRRFNELQYWVATELCLCSVPGLRAQLLRKFIKLA-
AHLKE-QKNLNSFFAVMFGLSNSAISRLAHTWERLPHKVRKLYSALERLLDPSWNHRVYR
LALTK--LSP-------PLIPF-MPLLLKDMTFI-------------------------
-----HEGNH--TLV-------E---NLINFE-KMRMMARAA---RMLHHCRSHS-----
-------------------NVPLSPLRSRVSHLHEDSQAVR----VST---CSEQS
>Rapgef3_[Carlito_syrichta]_640792033
SAKDLAGQLTDHDWSLFNSIHQV----ELI-----HYVLG-PQHL----R--DV-TTA--
-------------------NLERFMRRFNELQYWVATELCLCPVPGPRVQLLRKFIKLA-
AHLKE-QKNLNSFFAIMFGLSNSAISRLAHTWERLPHKVRKLYSALERLLDPSWNHRVYR
LALAK--LSP-------PVIPF-MPLLLKDMTFI-------------------------
-----HEGNH--TLV-------E---NLINFE-KMRMMARAV---RMLHHCRGHS-----
-------------------TVPLSPLRSRVSHLHEDSQAAR----IST---CSEQS
>Rapgef3_[Cavia_porcellus]_348580717
SAKDLAGQLTDHDWSLFNSIHQV----ELI-----HYVLG-PQHL----R--DV-TTA--
-------------------NLERFMRRFNELQYWVATELCLCSVPGLRAQLLRKFIKLA-
AHLKE-QKNLNSFFAIMFGLSNSAISRLAHTWERLPHKVRKLYSALERLLDPSWNHRVYR
LALTK--LSP-------PIIPF-MPLLLKDMTFI-------------------------
-----HEGNH--TLV-------E---NLINFE-KMRMMARAA---RLLHHCRNHS-----
-------------------TVPLSPLRSRVSHLHEDSQASR----IST---CSEQS
>Rapgef3_[Cebus_capucinus_imitator]_1044333204
SAKDLAGQLTDHDWSLFNSIHQV----ELI-----HYVLG-PQHL----R--DI-TTA--
-------------------NLERFMRRFNELQYWVATELCLCAVPGPRAQLLRKFIKLA-
AHLKE-QKNLNSFFAIMFGLSNSAISRLAHTWERLPHKVRKLYSALERLLDPSWNHRVYR
LALAK--LSP-------PVIPF-MPLLLKDMTFI-------------------------
-----HEGNH--TLV-------E---NLINFE-KMRMMARAA---RMLHHCRSHN-----
-------------------PVPLSPLRSRVSHLHEDSQVAR----IST---CSEQS
>Rapgef3_[Ceratotherium_simum_simum]_478509423
SAKDLAGQLTDHDWSLFNSIHQV----ELI-----HYVLG-PQHL----R--DV-TTA--
-------------------NLERFMRRFNELQYWVATELCLCPVPGPRAQLLRKFIKLA-
AHLKE-QKNLNSFFAVMFGLSNSAISRLAHTWERLPHKVRKLHSALERLLDPSWNHRVYR
LALTK--LSP-------PIIPF-MPLLLKDMTFI-------------------------
-----HEGNH--TLV-------E---NLINFE-KMRMMARAV---RMLHHCRSHS-----
-------------------SVPLSPLRSRVSHLHEDGQAAR----IST---CSEQS
>Rapgef3_[Cercocebus_atys]_795538404

SAKDLAGQLTDHDWSLFNSIHQV----ELI-----YYALG-PQHL----R--DV-TTA--
-------------------NLERFMRRFNELQYWVATELCLCPVPGPRAQLLRKFIKLA-
AHLKE-QKNLNSFFAVMFGLSNSAISRLAHTWERLPHKVRKLYSALERLLDPSWNHRVYR
LALAK--LSP-------PVIPF-MPLLLKDMTFI-------------------------
-----HEGNH--TLV-------E---NLINFE-KMRMMARAA---RMLHHCRSHN-----
-------------------PVPLSPLRSRVSHLHEDSQVAR----IST---CSEQS
>Rapgef3_[Charadrius_vociferus]_699656507
SSKDLASHLTDYDWNLFKSIHQV----EMI-----HYIVG-PQKF----H--DV-TTA--
-------------------NLERVMRRFNELQYWVATELCLCPEVGRRAQLLRKFIKLA-
AHLKE-QKNLNSFFAVMFGVSNTAVSRLAKTWERLPHKIRKLHSALERMLDPSWNHRVYR
LAVAK--LSP-------PIIPF-VPLLLKDMTFI-------------------------
-----HEGNR--TLA-------E---NLINFE-KMHMMAKTV---RVLQRCRGHA-----
-------------------HAPLSPLRNRSPHRPEDPKAIR----IST---CSEQS
>Rapgef3_[Chelonia_mydas]_465956413
SSKDLASQLTDYDWNLFSSVHQV----ELI-----YYTFG-QQTF----P--SA-TTA--
-------------------NLERVMRRFNELQYWIATELCLCAEPGKRAQLLRKFIKLA-
AHLKE-QKNLNSFFAVMFGLSNTSVSRLSKTWERLPHKTRKLHSALERMLDPSWNHRVYR
LAIAK--LTP-------PIIPF-MPLLLKDMTFI-------------------------
-----HEGNR--TLA-------E---NLINFE-KMRMMAKAV---RIIHHSQSHT-----
-------------------YGPISPLRSRPPQLLEDPQALR----IST---CSEQS
>Rapgef3_[Chinchilla_lanigera]_533187398
SARDLAGQLTDLDWSLFNSIHQV----ELI-----HYVLG-PQRL----R--DG-TTA--
-------------------NLERFMRRFNELQFWVATELCLCPAPGLRAQLLRKFIKLA-
AHLKE-QKNLNSFFAIMFGLSNSAISRLAHTWERLPHKVRKLYSALERLLDPSWNHRVYR
LALSK--LSP-------PLIPF-MPLLLKDMTFI-------------------------
-----HEGNR--TLV-------E---NLINFE-KMRMMARAA---RLLHRCRSHG-----
-------------------AVPLSPLRSRVSHLHEDSQASR----VST---CSEQS
>Rapgef3_[Chlorocebus_sabaeus]_635064868
SAKDLAGQLTDHDWSLFNSIHQV----ELI-----YYALG-PQHL----R--DV-TTA--
-------------------NLERFMRRFNELQYWVATELCLCPVPGPRAQLLRKFIKLA-
AHLKE-QKNLNSFFAVMFGLSNSAISRLAHTWERLPHKVRKLYSALERLLDPSWNHRVYR
LALAK--LSP-------PIIPF-MPLLLKDMTFI-------------------------
-----HEGNH--TLV-------E---NLINFE-KMRMMARAA---RMLHHCRSHN-----
-------------------PVPLSPLRSRVSHLHEDSQVAR----IST---CSEQS
>Rapgef3_[Chrysochloris_asiatica]_586469584
SAKDLAGQLTDHDWNLFHSIHQV----ELI-----HYVLG-PQHL----R--TV-TTA--
-------------------NLERFLRRFNELQYWVATELCLCPVPSLRAQLLRKFIKLA-
AHLKE-QKNLNSFFAIMFGLSNSAISRLAHTWERLPHKVRKLYSALERLLDPSWNHRVYR
LALTK--LSP-------PIIPF-MPLLLKDMTFI-------------------------
-----HEGNH--TLV-------E---NLINFE-KMRMMARAV---RMLHQCRSHS-----
-------------------NVPLSPLRSRVSHLHEDSQAVR----IST---CSEQS
>Rapgef3_[Clupea_harengus]_831315562
GSKDIASQLTNYDWELFTAMHET----ELL-----YYVFG-REKF----P--HT-TTA--
-------------------NLERFMRRFNEVQYWVVTELCLCVDLAKRAALLKKFIKMA-
IVLKE-QKNLNSFYAVMFGLTNSAVTRLYKTWERVPNKTRRIFTTYERILDPARNHRAYR
LAVVK--LSP-------PYIPF-MPLILKDMTFI-------------------------
-----HEGNK--NYT-------N---TLVNFE-KMRMIARTL---KIVTGCRNQS-----
------------------YSVPSSPQKGMTERMFLETPAIR----LSTY---SEQS
>Rapgef3_[Colobus_angolensis_palliatus]_795136375
SAKDLAGQLTDHDWSLFNSIHQV----ELI-----YYALG-PQHL----R--DV-TTA--
-------------------NLERFMRRFNELQYWVATELCLCPVPGPRAQLLRKFIKLA-
AHLKE-QKNLNSFFAVMFGLSNSAISRLAHTWERLPHKVRKLYSALERLLDPSWNHRVYR
LALAK--LSP-------PVIPF-MPLLLKDMTFI-------------------------
-----HEGNH--TLV-------E---NLINFE-KMRMMARAA---RMLHHCRSHN-----

```
-------------------PVPLSPLRSRVSHLHEDSQVAR----IST---CSEQS
>Rapgef3_[Columba_livia]_915562087
SSKDLASHLTDYDWNLFKSIHQV----EMI-----HYIVG-PQKF----H--EV-TTA--
-------------------NLERVMRRX---QYWVATELCLCPEVGRRAQLLRKFIKLA-
AHLKE-QKNLNSFFAVMFGVSNTAVSRLARTWERLPHKIRKLHSALERMLDPSWNHRVYR
LAVAK--LSP-------PIIPF-MPLLLKDMTFI-------------------------
-----HEGNR--TMA-------E---NLINFE-KMHMMAKTL---RVLQRCRGQA-----
-------------------HAPLSPLRSRSPHRPEDPKSVR----IST---CSEQS
>Rapgef3_[Coturnix_japonica]_1003995665
SSKDLASHLTDYDWNLFKSIHQV----EMI-----HYIVG-PQKF----H--EV-TTA--
-------------------NLERMMRRFNELQYWVATELCLCNELGRRAQLLRKFIKLA-
AHLKE-QKNLNSFFAVMFGVSNTAVSRLAKTWERLPHKIRKLHSALERMLDPSWNHRVYR
LAVAK--LSP-------PIIPF-IPLLLKDMTFI-------------------------
-----HEGNR--TLA-------E---NLINFE-KMHMMAKTV---RILQRCRGQA-----
-------------------HAPLSPLRTRSPHRPEDARAVR----IST---CSEQS
>Rapgef3_[Cricetulus_griseus]_625224026
SAKDLAGQLTDHDWNLFNRIHQV----ELI-----HYVLG-PQHL----R--DV-TTA--
-------------------NLERFMRRFNELQYWVATELCLCSVPGPRAQLLRKFIKLA-
AHLKE-QKNLNSFFAVMFGLSNSAISRLAHTWERLPHKVRKLYSALERLLDPSWNHRVYR
LALTK--LSP-------PVIPF-MPLLLKDMTFI-------------------------
-----HEGNH--TLV-------E---NLINFE-KMRMMARAV---RMLHHCRSHN-----
-------------------TVPLSPLRSRVSHIHEDSQASR----IST---CSEQS
>Rapgef3_[Cynoglossus_semilaevis]_657766970
SSKDLAYELTNYDWELFTAVHEV----ELV-----YYIFG-RQKF----H--GA-TTA--
-------------------NLERFVRRFNEVQYWVLTEVCLCEDVVKRASLLKKFIKIA-
AVLKE-QKNLNSFFAVMFALSNSAVQRLYKTWERIPSKTKRIYCAYERLMDPSRNHRAYR
LTVAK--LSP-------PYIPL-MPLLLKDMTFI-------------------------
-----HEGNS--NYV-------E---KLVNFE-KMRMMAKTV---KIVRGCRSQP-----
------------------Y-VPSSPQRGLADRMFLDGPANR----LSTY---SD--
>Rapgef3_[Danio_rerio]_688612298
-SKDMASQHTSYDWELFMAMHEV----ELV-----YYVFG-REKF----P--GS-TTA--
-------------------NLERFVRRFNEVQYWVVTELCLCEDLGKRAILLKKFIKMA-
VVLKE-QKNLNSFFAVMFGLSNSAVQRLNKTWERLPNKTKRIYCAYERLMDPSRNHRAYR
LTIAK--LSP-------PYIPF-MPLLLKDMTFI-------------------------
-----HEGNK--NYT-------D---KLVNFE-KMRMIARTV---KTVRDCRSQ------
--------------------------------------------------------
>Rapgef3_[Dasypus_novemcinctus]_488561334
SAKDLAGQLTEHDWTLFNSIQQV----ELI-----HFVLG-PQHL----R--DV-TTA--
-------------------NLERFMRRFNELQYWVATELCLCPVPGPRAQLLRKFIKLA-
AHLKE-QKNLNSFFAVMFGLSNSAISRLAHTWERLPHKVRKLYSALERLLDPSWNHRVYR
LALAK--LSP-------PVIPF-MPLLLKDMTFI-------------------------
-----HEGNH--TLV-------E---NLINFE-KMRMMARAM---RMLHHCRNHS-----
--------------------------------------------------------
>Rapgef3_[Echinops_telfairi]_850308220
------------------------------------------------------------
------------------------------------------------------------
-------------------------------------------------LDPSWNHRVYR
LALTK--LSP-------PIIPF-MPLLLKDMTFI-------------------------
-----HEGNH--TLV-------E---NLVNFE-KMRMMARAV---RLLHHCRSHGNVPLS
PLRSRASQLHEDSQAMRVSTCSEQS-------------------------------
>Rapgef3_[Egretta_garzetta]_697847879
SSKDLASHLTDYDWNLFKSIHQV----EMI-----HYIVG-PQKF----H--DV-TTA--
-------------------NLERVMRRFNELQYWVATELCLCPEVGRRAQLLRKFIKLA-
AHLKE-QKNLNSFFAVMFGVSNTAVSRLAKTWE-----------------DPSWNHRVYR
```

LAVAK--LSP-------PIIPF-VPLLLKDMTFI-------------------------
-----HEGNR--TLA-------E---NLINFE-KMVSGAGRL---PVDTSP---------

>Rapgef3_[Eptesicus_fuscus]_641700539
SAKDLAVQLTDHDWNLFNSIHQV----ELI-----HYVLG-PQHL----R--DV-TTA--
-------------------NLERFMRRFNELQYWVATELCLCPVPGPRAQLLRKFIKLA-
AHLKE-QKNLNSFFAVMFGLSNSAISRLAHTWERLPHKVRKLYSALERLLDPSWNHRVYR
LALTK--LSP-------PVIPF-MPLLLKDMTFI-------------------------
-----HEGNH--TLV-------E---NLINFE-KMRMMARAV---RMLHHCRSHS-----
-------------------NVPLSPLRSRVSHLHEDSQAAR----VST---CSEQS
>Rapgef3_[Equus_asinus]_958727868
SAKDLAGQLTDHDWNLFNSIHQV----ELI-----HYVLG-PQHL----R--DV-TTA--
-------------------NLERFMRRFNELQYWVATELCLCPVPGPRAQLLRKFIKLA-
AHLKE-QKNLNSFFAVMFGLSNSAISRLAHTWERLPHKVRKLYSALERLLDPSWNHRVYR
LALTK--LSP-------PVIPF-MPLLLKDMTFI-------------------------
-----HEGNH--TLV-------E---NLINFE-KMRMMARAV---RMLHHCRSHS-----
-------------------SVPLSPLRSRVSHFHEDGQAAR----IST---CSEQS
>Rapgef3_[Equus_caballus]_545219817
SAKDLAGQLTDHDWNLFNSIHQV----ELI-----HYVLG-PQHL----R--DV-TTA--
-------------------NLERFMRRFNELQYWVATELCLCPVPGPRAQLLRKFIKLA-
AHLKE-QKNLNSFFAVMFGLSNSAISRLAHTWERLPHKVRKLYSALERLLDPSWNHRVYR
LALTK--LSP-------PVIPF-MPLLLKDMTFI-------------------------
-----HEGNH--TLV-------E---NLINFE-KMRMMARAV---RMLHHCRSHS-----
-------------------SVPLSPLRSRVSHFHEDGQAAR----IST---CSEQS
>Rapgef3_[Equus_przewalskii]_664709775
SAKDLAGQLTDHDWNLFNSIHQV----ELI-----HYVLG-PQHL----R--DV-TTA--
-------------------NLERFMRRFNELQYWVATELCLCPVPGPRAQLLRKFIKLA-
AHLKE-QKNLNSFFAVMFGLSNSAISRLAHTWERLPHKVRKLYSALERLLDPSWNHRVYR
LALTK--LSP-------PVIPF-MPLLLKDMTFI-------------------------
-----HEGNH--TLV-------E---NLINFE-KMRMMARAV---RMLHHCRSHS-----
-------------------SVPLSPLRSRVSHFHEDGQAAR----IST---CSEQS
>Rapgef3_[Erinaceus_europaeus]_1016600502
SAKDLAGQLTDHDWGLFNSIHQV----ELI-----HYVLG-PQHL----R--DV-TTA--
-------------------NLERFMRRFNELQYWVATELCLCPLPGSRAQLLRKFIKLA-
AHLKE-QKNLNSFFAIMFGLSNSAISRLAHTWERLPHKVRKLYSALERLLDPSWNHRVYR
LALSK--LSP-------PIIPF-MPLLLKDMTFI-------------------------
-----HEGNH--TLV-------E---NLINFE-KMRMMARAV---RMLHHCRSHS-----
-------------------SVPLSPLRSRVSHLHEDSQAAR----IST---CSEQS
>Rapgef3_[Esox_lucius]_742184366
-SKDIAGQLTNYDWELFTAMHEV----ELV-----YYVFG-QHNF----P--GT-TTA--
-------------------NLERFVHRFNQVQYWVVTEVCLCDDLVKRAMLLKKFIKIA-
AMLKE-QKNLNSFFAVMFGLSNSAVQRLYKTWERVPSKTKRVYCAYERLMDPSRNHRAYR
LAVAK--LTP-------PYIPF-MPLLLKDMTFM-------------------------
-----HEGNQ--NYT-------E---KLVNFE-KMRMIAKIV---KIVRGCRSTP-----
------------------Y-VPSSPQKGLADRMFLETPSIR----VSTY---SEQS
>Rapgef3_[Falco_cherrug]_929429028
SSKDLASHLTDYDWNLFKSIHQV----EMI-----HYIIG-PQKF----H--DV-TTA--
-------------------NLERVMRRFNELQYWVATELCLCPEVGRRAQLLRKFIKLA-
AHLKE-QKNLNSFFAVMFGVSNTAVSRLAKTWERLPHKIRKLHSVLERMLDPSWNHRVYR
LAVAK--LSP-------PIIPF-VPLLLKDMTFI-------------------------
-----HEGNR--TLA-------E---NLINFE-KMHMMAKTV---RVLQRCRGHA-----
-------------------HAPLSPLRNRSPHRTEDPRAVR----IST---CSEQS
>Rapgef3_[Felis_catus]_410964201
SAKDLAGQLTDHDWNLFNSIHQV----ELI-----HYVLG-PQHL----R--DV-TTA--

-------------------NLERFMRRFNELQYWVATELCLCPVPGLRAQLLRKFIKLA-
AHLKE-QKNLNSFFAVMFGLSNSAISRLAHTWERLPHKVRKLYSALERLLDPSWNHRVYR
LALTK--LSP-------PIIPF-MPLLLKDMTFI-------------------------
-----HEGNH--TLV-------E---NLINFE-KMRMMARAV---RMLHHCRSHS-----
-------------------NVPLSPLRSRVSHFHEDSQAAR----TST---CSEQS
>Rapgef3_[Ficedula_albicollis]_1020933356
SSKELASHLTDYDWSLFKSIHQV----EMI-----HYIVG-PQKF----H--EV-ATA--
-------------------NLARVMRRFNELQFWVATELCLCPELGRRAQLLRKFIKLA-
AHLKE-QKNLNSFFAVMFGVSNTAVTRLAKTWERLPHKIRKLHSALERMLDPSWNHRVYR
LAVAK--LSP-------PIIPF-VPLLLKGL----------------------------
--CPALG-----
-------------------TLWVCPTLSGGPSDPRE--AVR----IST---CSEQS
>Rapgef3_[Fukomys_damarensis]_731247212
SAKDLAGQLTDHDWNLFNSIHQV----ELI-----HYVLG-PQHL----R--DV-TTA--
-------------------NLERLMRRFNELQYWVATELCLCSVPGLRAQLLRKFIKLA-
AHLKE-QKNLNSFFAIMFGLSNSAISRLAHTWERLPHKVRKLYSALERLLDPSWNHRVYR
LALTK--LSP-------PIIPF-MPLLLKDMTFI-------------------------
-----HEGNH--TLV-------E---NLINFE-KMRMMARAV---RLLHHCRSHS-----
-------------------TVPLSPLRSRVSHLHEDSQASR----IST---CSEQS
>Rapgef3_[Fundulus_heteroclitus]_831559266
SSKDVAIELTNYDWELFTAMHEV----ELV-----YYIFG-RHKF----P--GA-TTA--
-------------------NLERFVRHFNVVQYWVVTELCLCEDLVKRAILLKKFIKIA-
SVLKE-QRNLNSFFAVMFGLGNSAVQRLYKTWDRIPSKTKRIYCAYERLMDPTRNHRAYR
LAVAK--LSP-------PYIPF-MPLLLKDMTFI-------------------------
-----HEGNP--NYV-------D---KLVNFE-KMRMLAKTV---KIVRECRSQP-----
------------------Y-VPSSPQRGLADRMFLDGPATR----LSTY---SD--
>Rapgef3_[Galeopterus_variegatus]_667328784
----------------------V----ELI-----HYVLG-PQHL----R--DV-TTA--
-------------------NLERFMHRFNELQYWVATELCLCPVPGPRAQLLRKFIKLA-
AHLKE-QKNLNSFFAVMFGLSNSAISRLAHTWERLPHKIRKLYSALERLLDPSWNHRVYR
LALTK--LSP-------PIIPF-MPLLLKDMTFI-------------------------
-----QEGNH--TLV-------E---NLINFE-KMRMMARAV---RMLHHCRSHS-----
-------------------SVPLSPLRSRASHLHEDSQVAR----IST---CSEQS
>Rapgef3_[Geospiza_fortis]_930243948
SSKDLASHLTDYDWNLFKSIHQV----EMI-----HYIVG-PHKF----H--EV-ATA--
-------------------NLARVLRRGCQ------------PRDPIPAQRLPHKIRKL-
--HSALERMLDPSWNHRVYR
LAVAK--LSP-------PIIPF-VPLLLKDMTFI-------------------------
-----HEGNR--TLA-------E---NLINFE-KMHMMAKTV---RVLQRCRGHA-----
-------------------HAPLSPLRSRSPHRPEDTKAVR----IST---CSEQS
>Rapgef3_[Gorilla_gorilla_gorilla]_426372291
SAKDLAGQLTDHDWSLFNSIHQV----ELI-----HYVLG-PQHL----R--DV-TTA--
-------------------NLERFMRRFNELQYWVATELCLCPVPGSRAQLLRKFIKLA-
AHLKE-QKNLNSFFAVMFGLSNSAISRLAHTWERLPHKVRKLYSALERLLDPSWNHRVYR
LALAK--LSP-------PVIPF-MPLLLKDMTFI-------------------------
-----HEGNH--TLV-------E---NLINFE-KMRMMARAA---RMLHHCRSHN-----
-------------------PVPLSPLRSRVSHLHEDSQVAR----IST---CSEQS
>Rapgef3_[Haliaeetus_leucocephalus]_729737483
SSKDLASHLTDYDWNLFKSIHQV----EMI-----HYIVG-PQKF----H--DV-TTA--
-------------------NLERVMRRFNELQYWVATELCLCPEVGRRAQLLRKFIKLA-
AHLKE-QKNLNSFFAVMFGVSNTAVSRLAKTWERLPHKIRKLHSALERMLDPSWNHRVYR
LAVAK--LSP-------PIIPF-VPLLLKDMTFI-------------------------
-----HEGNR--TLA-------E---NLINFE-KMHMMAKTV---RVLQRCRGHA-----
-------------------HAPLSPLRNRSPHRPEDPKAIR----IST---CSEQS

```
>Rapgef3_[Heterocephalus_glaber]_513003372
SAKDLAGQLTDQDWSLFNSIHQV----ELI-----HYVLG-PQHL----R--DV-TTA--
-------------------NLERFMRRFNQLQYWVATELCLCSVAGLRAQLLRKFIKLA-
AHLKE-QKNLNSFFAVMFGLSNSAISRLAHTWERLPHKVRKLYSALERLLDPSWNHRVYR
LALAK--LSP-------PIIPF-MPLLLKDMTFI--------------------------
-----HEGNH--TLV-------E---NLINFE-KMRMMARTA---RLLHHCRSHS-----
-------------------TVPLSPLRSRVSHLHEDGQASR----IST---CSEQS
>Rapgef3_[Homo_sapiens]_530399604
SAKDLAGQLTDHDWSLFNSIHQV----ELI-----HYVLG-PQHL----R--DV-TTA--
-------------------NLERFMRRFNELQYWVATELCLCPVPGPRAQLLRKFIKLA-
AHLKE-QKNLNSFFAVMFGLSNSAISRLAHTWERLPHKVRKLYSALERLLDPSWNHRVYR
LALAK--LSP-------PVIPF-MPLLLKDMTFI--------------------------
-----HEGNH--TLV-------E---NLINFE-KMRMMARAA---RMLHHCRSHN-----
-------------------PVPLSPLRSRVSHLHEDSQVAR----IST---CSEQS
>Rapgef3_[Ictidomys_tridecemlineatus]_914907256
SAKDLAGQLTDHDWNLFNSIHQV----ELI-----HYVLG-PQHL----R--DV-TTA--
-------------------NLERFMRRFNELQYWVATELCLCPVPGLRAQLLRKFIKLA-
AHLKE-QKNLNSFFAIMFGLSNSAISRLAHTWERLPHKVRKLYSALERLLDPSWNHRVYR
LALTK--LSP-------PVIPF-MPLLLKDMTFI--------------------------
-----HEGNH--TLV-------E---NLINFE-KMRMMARAV---RMLHHCRSHS-----
-------------------TVPLSPLRSRVSHFHEDSQASR----IST---CSEQS
>Rapgef3_[Jaculus_jaculus]_507569974
SAKDLAGQLTDHDWNLFNSIHQV----ELI-----HYVLG-PQHL----R--DV-TTA--
-------------------NLERFMRRFNELQYWVATELCLCPVPGPRAQLLRKFIKLA-
AHLKE-QKNLNSFFAVMFGLSNSAISRLAHTWERLPHKVRKLYSALERLLDPSWNHRVYR
LALTK--LSP-------PVIPF-MPLLLKDMTFI--------------------------
-----HEGNH--TLV-------E---NLINFE-KMRMMARAV---RTLHHCRSHS-----
-------------------SVPLSPLRSRVSHLHEDSQASR----IST---CSEQS
>Rapgef3_[Kryptolebias_marmoratus]_1041103289
SSKDVAIELTNYDWELFTAMHEV----ELV-----HYIFG-RHKF----P--GA-ITA--
-------------------NLERFVRHFNEVQYWVVTELCLCEDLVKRANLLKKFIKIA-
SVLKD-QKNLNLFFAVMFGLGNSAVQRLYKTWERIPSKTKRVYCTYERMMDPSRNHRAYR
LAVTK--LSP-------PYIPF-MPLLLKDMTFI--------------------------
-----HEGNP--DCL-------D---KLVNFE-KMRMLAKTV---KIVRECRSQP-----
------------------Y-VPSSPQRGLADRIFLEGP-TR----LSTS---SE--
>Rapgef3_[Latimeria_chalumnae]_556946159
SSKDIANQLTDPDWDLFNCIHEV----ELVYYILGRHVFG-NITT----A----------
-------------------NLERFVCRFNEIQFWVVTEIVLCSDLNKRVTLLKKFIKVA-
TILKE-QKNLNSFFAIMFGLSNSAVSRLSKTWERLPNKTRKLYSGFERLMDPSKNHRAYR
LAIT--KLSP-------PIIPF-IPLLLKDMTFV--------------------------
-----HDGNK--TYL-------E---NLVNFE-KMRMIATTV---RIIQHCRSQ------
--------------------------------------------------------
>Rapgef3_[Lepisosteus_oculatus]_972964891
SSKDIACQLSDYDWELFGAMHEV----ELV-----YYIFG-RHKF----R--GA-TTA--
-------------------NLERFVRRFNEVQHWVATELCLCPDLGKRALLLKKFIKVA-
TVLKE-QKNLNSFFAVMFGLSNSAVQRLSRTWERLPSKTRRIYCAYERLMDPSRNHRAYR
LAVAK--LSS-------PYIPF-MPLLLKDMTFI--------------------------
-----HEGNK--NHI-------E---GLVNFE-KMRMIAKTV---KIVRECRSQP-----
------------------Y-VPASPQKGLTDRVFLETTAVR----VSTC---SEQS
>Rapgef3_[Leptonychotes_weddellii]_585154006
SAKDLAGQLTDHDWSLFNSIHQV----ELI-----HYVLG-PQHL----R--DV-TTA--
-------------------NLERFMRRFNELQYWVATELCLCPVPSLRAQLLRKFIKLA-
AHLKE-QKNLNSFFAIMFGLSNSAISRLAHTWERLPHKVRKLYSALERLLDPSWNHRVYR
LLLTK--LSP-------PIIPF-MPLLLK-------------------------------
```


>Rapgef3_[Loxodonta_africana]_731457673
SAKDLAGQLTDHDWSLFNSIHQV----ELI-----HYVLG-PQHL----R--TV-TTA--
-------------------NLERFMRRFNELQYWVATELCLCPVPGLRAQLLRKFIKLA-
AHLKE-QKNLNSFFAVMFGLSNSAVSRLAHTWERLPHKVRKLYSALERLLDPSWNHRVYR
LALTK--LSP-------PVIPF-MPLLLKDMTFI--------------------------
-----HEGNH--TLV-------E---NLINFE-KMRMMARAV---RMLHHCRGHS-----
-------------------NVPLSPLRSRVSHLHEDSQASR----IST---CSEQS
>Rapgef3_[Macaca_fascicularis]_982281158
SAKDLAGQLTDHDWSLFNSIHQV----ELI-----YYVLG-PQHL----R--DV-TTA--
-------------------NLERFMRRFNELQYWVATELCLCPVPGPRAQLLRKFIKLA-
AHLKE-QKNLNSFFAVMFGLSNSAISRLAHTWERLPHKVRKLYSALERLLDPSWNHRVYR
LALAK--LSP-------PVIPF-MPLLLKDMTFI--------------------------
-----HEGNH--TLV-------E---NLINFE-KMRMMARAA---RMLHHCRSHN-----
-------------------PVPLSPLRSRVSHLHEDSQVAR----IST---CSEQS
>Rapgef3_[Macaca_mulatta]_966971990
SAKDLAGQLTDHDWSLFNSIHQV----ELI-----YYVLG-PQHL----R--DV-TTA--
-------------------NLERFMRRFNELQYWVATELCLCPVPGPRAQLLRKFIKLA-
AHLKE-QKNLNSFFAVMFGLSNSAISRLAHTWERLPHKVRKLYSALERLLDPSWNHRVYR
LALAK--LSP-------PVIPF-MPLLLKDMTFI--------------------------
-----HEGNH--TLV-------E---NLINFE-KMRMMARAA---RMLHHCRSHN-----
-------------------PVPLSPLRSRVSHLHEDSQVAR----IST---CSEQS
>Rapgef3_[Macaca_nemestrina]_795561749
SAKDLAGQLTDHDWSLFNGIHQV----ELI-----YYVLG-PQHL----R--DV-TTA--
-------------------NLERFMRRFNELQYWVATELCLCPVPGPRAQLLRKFIKLA-
AHLKE-QKNLNSFFAVMFGLSNSAISRLAHTWERLPHKVRKLYSALERLLDPSWNHRVYR
LALAK--LSP-------PVIPF-MPLLLKDMTFI--------------------------
-----HEGNH--TLV-------E---NLINFE-KMRMMARAA---RMLHHCRSHN-----
-------------------PVPLSPLRSRVSHLHEDSQVAR----IST---CSEQS
>Rapgef3_[Mandrillus_leucophaeus]_795173402
SAKDLAGQLTDHDWSLFNSIHQV----ELI-----YYALG-PQHL----R--DV-TTA--
-------------------NLERFMRRFNELQYWVATELCLCPVPGPRAQLLRKFIKLA-
AHLKE-QKNLNSFFAVMFGLSNSAISRLAHTWERLPHKVRKLYSALERLLDPSWNHRVYR
LALAK--LSP-------PVIPF-MPLLLKDMTFI--------------------------
-----HEGNH--TLV-------E---NLINFE-KMRMMARAA---RMLHHCRSHN-----
-------------------PVPLSPLRSRVSHLHEDSQVAR----IST---CSEQS
>Rapgef3_[Marmota_marmota_marmota]_984114365
SAKDLAGQLTDHDWNLFNSIHQV----ELI-----HYVLG-PQHL----R--DV-TTA--
-------------------NLERFMRRFNELQYWVATELCLCPVPGLRAQLLRKFIKLA-
AHLKE-QKNLNSFFAIMFGLSNSAISRLAHTWERLPHKVRKLYSALERLLDPSWNHRVYR
LALTK--LSP-------PVIPF-MPLLLKDMTFI--------------------------
-----HEGNH--TLV-------E---NLINFE-KMRMMARAV---RMLHHCRSHS-----
-------------------TVPLSPLRSRVSHFHEDSQASR----IST---CSEQS
>Rapgef3_[Maylandia_zebra]_499019354
SSKDIASELTNYDWELFTAMHEV----ELV-----YYIFG-RHKF----P--GA-ITA--
-------------------NLERFVRRFNEVQHWVLTELCLCEDLVKRAMLLKKFIKIA-
SVLKE-QKNLNSFFAVMFGLSNSAIQRLYKTWERIPSKTKRIYCSLERLMDPSRNHRAYR
LAIAK--LSP-------PYIPF-MPLLLKDMTFI--------------------------
-----HEGNA--NYV-------D---KLVNFE-KMRMLAKTV---KIVRGCRSQP-----
------------------Y-VPSSPQRGLADRMFLEGAATR----LSTY---SD--
>Rapgef3_[Mesocricetus_auratus]_524925946
SAKDLAGQLTDHDWNLFNRIHQV----ELI-----HYVLG-PQHL----R--DV-TTA--
-------------------NLERFMRRFNELQYWVATELCLCSVPGPRAQLLRKFIKLA-

AHLKE-QKNLNSFFAVMFGLSNSAISRLAHTWERLPHKVRKLYSALERLLDPSWNHRVYR
LALTK--LSP-------PVIPF-MPLLLKDMTFI-------------------------
-----HEGNH--TLV-------E---NLINFE-KMRMMARAV---RMLHHCRSHS-----
-------------------TVPLSPLRSRVSHIHEDSQASR----IST---CSEQS
>Rapgef3_[Microtus_ochrogaster]_532021349
SAKDLAGQLTELDWSLFNRIHQV----ELI-----HYVLG-PQHL----R--DV-TTA--
-------------------NLERFMRRFNELQYWVATELCLCSVPGPRAQLLRKFIKLA-
AHLKE-QKNLNSFFAVMFGLSNSAISRLAHTWERLPHKVRKLYSALERLLDPSWNHRVYR
LALTK--LSP-------PVIPF-MPLLLKDMTFI-------------------------
-----HEGNH--TLV-------E---NLINFE-KMRMMARAM---RMLHHCRSHS-----
-------------------TVPLSPLRSRVSHIHEDSQASR----IST---CSEQS
>Rapgef3_[Miniopterus_natalensis]_1016658187
SAKDLAGQLTDHDWNLFNSIHQV----ELI-----HYVLG-PQHL----R--DV-TTA--
-------------------NLERFMRRFNELQYWVATELCLCPVPGPRAQLLRKFIKLA-
AHLKE-QKNLNSFFAVMFGLSNSAISRLAHTWERLPHKVRKLYSALERLLDPSWNHRVYR
LALTK--LSP-------PVIPF-MPLLLKDMTFI-------------------------
-----HEGNH--TLV-------E---NLINFE-KMRMMARAV---RMLHHCRSHS-----
-------------------NVPLSPLRSRVSHLHEDGQAAR----IST---CSEQS
>Rapgef3_[Mus_musculus]_1039748761
SAKDLAGQLTDHDWNLFNRIHQV----ELI-----HYVLG-PQHL----R--DV-TTA--
-------------------NLERFMRRFNELQYWVATELCLCPVPGSRAQLLRKFIKLA-
AHLKE-QKNLNSFFAVMFGLSNSAISRLAHTWERLPHKVRKLYSALERLLDPSWNHRVYR
LALTK--LSP-------PVIPF-MPLLLKDVTFI-------------------------
-----HEGNH--TLV-------E---NLINFE-KMRMMARAV---RMLHHCRSHS-----
-------------------TAPLSPLRSRVSHIHEDSQGSR----IST---CSEQS
>Rapgef3_[Mustela_putorius_furo]_859851834
SAKDLAGQLTDHDWSLFNSIHQV----ELI-----HYVLG-PQHL----R--DV-TTA--
-------------------NLERFMRRFNELQYWVATELCLCPVPGLRAQLLRKFIKLA-
AHLKE-QKNLNSFFAVMFGLSNSAISRLAHTWERLPHKVRKLYSALERLLDPSWNHRVYR
LALTK--LSP-------PIIPF-MPLLLKDMTFI-------------------------
-----HEGNH--TLV-------E---NLINFE-KMRMMARAV---RMLQHCRSHS-----
-------------------NVPLSPLRSRVSHLHEDSQVAR----MST---CSEQS
>Rapgef3_[Myotis_brandtii]_521032880
SAKDLAVQLTDHDWNLFNSIHQV----ELI-----HYVLG-PQHL----R--DV-TTA--
-------------------NLERFMRRFNELQYWVATELCLCPVPGPRAQLLRKFIKLA-
AHLKE-QKNLNSFFAVMFGLSNSAISRLAHTWERLPHKVRKLYSALERLL----------

>Rapgef3_[Myotis_davidii]_432114532
SAKDLAVQLTDHDWNLFNSIHQV----ELI-----HYVLG-PQHL----R--DV-TTA--
-------------------NLERFMRRFNELQYWVATELCLCPVPGPRAQLLRKFIKLA-
AHLKE-QKNLNSFFAVMFGLSNSAISRLAHTWERLPHKVRKLYSALERLLDPSWNHRVYR
LALTK--LSP-------PVIPF-MPLLLKGSQLP-------------------------
-----HRPGRGSALR-------P---GLMSLLCPQRMMARAV---RMLHHCRSHS-----
-------------------NVPLSPLRSRVSHLHEDSQAAR----VST---CSEQS
>Rapgef3_[Myotis_lucifugus]_940740350
SAKDLAVQLTDHDWNLFNSIHQV----ELI-----HYVLG-PQHL----R--EV-TTA--
-------------------NLERFMRRFNELQYWVATELCLCPVPGPRAQLLRKFIKLA-
AHLKE-QKNLNSFFAVMFGLSNSAISRLAHTWERLLHKVRKLYSALERLLDPSWNHRVYR
LALTK--LSP-------PVIPF-MPLLLKDMTFI-------------------------
-----HEGNH--TLV-------E---NLINFE-KMRMMARAV---RMLHHCRSHS-----
-------------------NVPLSPLRSRVSHLHEDIQAAR----VST---CSEQS
>Rapgef3_[Neolamprologus_brichardi]_583998066

SSKDIASELTNYDWELFTAMHEV----ELV-----YYIFG-RHKF----P--GA-ITA--
-------------------NLERFVRRFNEVQHWVLTELCLCEDLMKRAMLLKKFIKIA-
SVLKE-QKNLNSFFAVMFGLSNSAIQRLYKTWERIPSKTKRIYCSLERLMDPSRNHRAYR
LAIAK--LSP-------PYIPF-MPLLLKDMTFI-------------------------
-----HEGNA--NYV-------D---KLVNFE-KMRMLAKTV---KIVRGCRSQP-----
------------------Y-VPSSPQRGLADRMFLEGAATR----LSTY---SD--
>Rapgef3_[Nomascus_leucogenys]_820994222
SAKDLAGQLTDRDWSLFNSIHQV----ELI-----HYVLG-PQHL----R--DV-TTA--
-------------------NLERFMRRFNELQYWVATELCLCPVPGPRAQLLRKFIKLA-
AHLKE-QKNLNSFFAVMFGLSNSAISRLAHTWERLPHKVRKLYSALERLLDPSWNHRVYR
LAXRQ--ALP-------PVIPF-MLLLLKDMTFI-------------------------
-----HEGNH--TLV-------E---NLINFE-KMRMMARAA---RMLHHCRSHN-----
-------------------PVPLSPLRSRVSHLHEDSQVVR----IST---CSEQS
>Rapgef3_[Nothobranchius_furzeri]_1007768327
SSKDLAIELTNYDWELFNAMHEV----ELI-----YYIFG-RHKF----P--GA-ITA--
-------------------NLERFVRHFNEVQHWVVTELCLCEDLVKRAVLLKKFIKIA-
SVLKE-QKNLNLFFAVMFGLSNSAVQRLYKTWERIPSKTKRIYCTYERLMDPSRNHRAYR
LAVAK--LSP-------PYTPF-MPLLLKDMTFI-------------------------
-----HEGNP--NYT-------D---KLVNFE-KMRMLAKTV---KIVRGCRSQP-----
------------------Y-VPSSPQRGLADRIFLEGP-TR----LSTN---SEHA
>Rapgef3_[Ochotona_princeps]_504177543
SAKDLAGQLTEQDRDLFHSIHQV----ELI-----HYVLG-PQHL----R--NV-TTA--
-------------------NLERFMRRFNQLQYWVATELCLCPLPGPRAQLLRKFIKLA-
AHLKE-QKNLNSFFAIMFGLSNSAITRLARTWERLPQKVRKLHSALERLLDPSWNHRAYR
VALAK--LSP-------PVIPF-MPLLLKDMTFI-------------------------
-----HEGNR--TLV-------E---NLINFE-KMRMLARAV---RVLHHCQGHG-----
-------------------HAPLSPLRTRAAHIHEDSQASR----IST---CSEQS
>Rapgef3_[Octodon_degus]_820979431
SAKDLAGQLTERDWGLFKCIHQV----ELI-----HYVLD-PQHL----R--DG-TTA--
-------------------NLERVLRRFNELQYWVATELCLCSAPGLRAQLLRKFIKLA-
AHLKE-QKNLNSFFAVMFGLSNSAISRLARTWERLPQKVRKLYSALERLLDPSWNHRVYR
LALRK--LSP-------PLIPF-MPLLLKDMTFI-------------------------
-----HEGNR--TLV-------D---SLINFE-KMRMMARAA---RLLHYCRSHS-----
-------------------TAPLSPLRSRVSHLHEDSQALR----MST---CSEQS
>Rapgef3_[Odobenus_rosmarus_divergens]_823398553
SAKDLAGQLTDHDWSLFNSVHQV----ELI-----HYVLG-PQHL----R--DV-TTA--
-------------------NLERFMRRFNELQYWVATELCLCPVPGLRAQLLRKFIKLA-
AHLKE-QKNLNSFFAVMFGLSNSAISRLAHTWERLPHKVRKLYSALERLLDPSWNHRVYR
LLLTK--LSP-------PIIPF-MPLLLKDMTFI-------------------------
-----HEGNH--TLV-------E---NLINFE-KMRMLARAV---RMLHHCRSHS-----
-------------------NVPLSPLRSRVSHLHEDSQAAR----TST---CSEQS
>Rapgef3_[Opisthocomus_hoazin]_700398843
SSKDLASHLTDYDWNLFKRIHQV----EMI-----HYIVG-PQKF----H--DV-TTA--
-------------------NLERVMRRFNELQYWVATELCLCPEVGRRAQLLRKFIKLA-
AHLKE-QKNLNSFFAVMFGVSNTAVSRLAKTWE---HMMAKTVRVLQR-----------

>Rapgef3_[Orcinus_orca]_466031722
STKDLAGQLTDHDWSLFNSIHQV----ELI-----HYVLG-PQPL----R--DV-TTA--
-------------------NLERFMRRFNELQYWVATELCLCPVPGLRAQLLRKFIKLA-
AHLKE-QKNLNSFFAIMFGLSNSAISRLAQTWERLPHKVRKLYSALERLLDPSWNHRVYR
LALTK--LSP-------PLIPF-MPLLLKDMTFI-------------------------
-----HEGNH--TLV-------E---NLINFE-KMRMMARAA---RMLHHCRSHS-----

-------------------NVPLSPLRSRVSHLHEDSQAAR----IST---CSEQS
>Rapgef3_[Oreochromis_niloticus]_542216386
SSKDIASELTNYDWELFTAMHEV----ELV-----YYIFG-RHKF----P--GA-ITA--
-------------------NLERFVRRFNEVQHWVLTELCLCEDLVKRAMLLKKFIKIA-
SVLKE-QKNLNSFFAVMFGLSNSAIQRLYKTWERIPSKTKRIYCSLERLMDPSRNHRAYR
LAIAK--LSP-------PYIPF-MPLLLKDMTFI-------------------------
-----HEGNA--NYV-------D---KLVNFE-KMRMLAKTV---KIVRGCRSQP-----
------------------Y-VASSPQRGLADRMFLEGAATR----LSTY---SD--
>Rapgef3_[Ornithorhynchus_anatinus]_620972164
SSRDLASLLSEYDWGLFHCVHQV---EPIL-----YVLGP--QRL----R--DI-TTA--
-------------------NLERLMRRFNELQYWVVTELCLCPAPAARARLLSRFIKLA-
AQ--

>Rapgef3_[Orycteropus_afer_afer]_634839967
STKDLAGQLTDHDWSLFNSIHQV----ELI-----HYVLG-PQHL----R--TV-TTA--
-------------------NLERFMRRFNELQYWVATELCLCPVPSLRAQLLRKFIKLA-
AHLKE-QKNLNSFFAVMFGLSNSAISRLAHTWERLPHKVRKLYSALERLLDPSWNHRVYR
LALTK--LSP-------PVIPF-MPLLLKDMTFI-------------------------
-----HEGNH--TLV-------E---NLINFE-KMRMMARAV---RMLHQCRSHS-----
-------------------NVPLSPLRSRVSHLHEDSQAAR----IST---CSEQS
>Rapgef3_[Oryctolagus_cuniculus]_1040227932
SAKDLASQLTEQDRDLFYSVHQV----ELI-----HYVLG-PQHL----R--DV-TTA--
-------------------NLERFMRRFNELQYWVATELCLCLLPGPRAQLLRKFIKLA-
AHLKE-QKNLNSFFAVMFGLSNSAISRLAHTWERLPQKVRKLHSALERLLDPSWNHRAYR
VALAK--LSP-------PIIPF-MPLLLKDMTFI-------------------------
-----HEGNR--TLV-------E---NLINFE-KMRMMARAV---RVLQHCRGHG-----
-------------------HAPLSPLRTRASHVHEDSQASR----IST---CSEQS
>Rapgef3_[Oryzias_latipes]_765126149
GSKDMAAELTNFDWELFTAVHEV----ELV-----YYIFG-RNKF----P--GA-ITA--
-------------------NLERFVRHFNKLQYWVVTELCLCEDLMKRALLLKKFIKIA-
SVLKE-QKNLNSFFAVMFGLSNSAVQRLYKTWERIPSKIKRIYCTYERLMDPSRNHRAYR
LAVAK--LNP-------PYIPF-MPLLLKDMTFI-------------------------
-----HEGNP--SYV-------D---KLVNFE-KMRMLAKTV---NIVRGCRSQP-----
------------------Y-VPSSPQRGLADRMFLEGPATR----LSTY---SDHS
>Rapgef3_[Otolemur_garnettii]_831232292
SAKDLAGQLTDHDWNLFNSIHQV----ELI-----HYALG-PQHL----R--DV-TTA--
-------------------NLERFMRRFNELQYWVATELCLCPVPGPRAQLLRKFIKLA-
AHLKE-QKNLNSFFAVMFGLSNSAISRLAHTWERLPHKVRKLYSALERLLDPSWNHRVYR
LALAK--LSP-------PAIPF-MPLLLKDMTFI-------------------------
-----HEGNH--TIV-------E---NLINFE-KMRMMARAV---RMLHHCRGHS-----
-------------------SVPLSPLRTRVSHFHEDNQVAR----IST---CSEQS
>Rapgef3_[Ovis_aries_musimon]_803304007
STKDLAGQLTDHDWSLFNSIHQV----ELI-----HYVLG-PQHL----R--DV-TTA--
-------------------NLERFMRRFNELQYWVATELCLCSVPGLRAQLLRKFIKLA-
AHLKE-QKNLNSFFAVMFGLSNSAISRLAHTWERLPHKVRKLYSALERLLDPSWNHRVYR
LALTK--LSP-------PLIPF-MPLLLKDMTFI-------------------------
-----HEGNH--TLV-------E---NLINFE-KMRMMARAA---RMLHHCRSHS-----
-------------------NVPLSPLRSRVSHLHEDSQAVR----VST---CSEQS
>Rapgef3_[Ovis_aries]_803044458
STKDLAGQLTDHDWSLFNSIHQV----ELI-----HYVLG-PQHL----R--DV-TTA--
-------------------NLERFMRRFNELQYWVATELCLCSVPGLRAQLLRKFIKLA-
AHLKE-QKNLNSFFAVMFGLSNSAISRLAHTWERLPHKVRKLYSALERLLDPSWNHRVYR

LALTK--LSP-------PLIPF-MPLLLKDMTFI-------------------------
-----HEGNH--TLV-------E---NLINFE-KMRMMARAA---RMLHHCRSHS-----
-------------------NVPLSPLRSRVSHLHEDSQAVR----VST---CSEQS
>Rapgef3_[Pan_paniscus]_675783064
SAKDLAGQLTDHDWSLFNSIHQV----ELI-----HYVLG-PQHL----R--DV-TTA--
-------------------NLERFMRRFNELQYWVATELCLCPVPGPRAQLLRKFIKLA-
AHLKE-QKNLNSFFAVMFGLSNSAISRLAHTWERLPHKVRKLYSALERLLDPSWNHRVYR
LALAK--LSP-------PVIPF-MPLLLKDMTFI-------------------------
-----HEGNH--TLV-------E---NLINFE-KMRMMARAA---RMLHHCRSHN-----
-------------------PVPLSPLRSRVSHLHEDSQVAR----IST---CSEQS
>Rapgef3_[Pan_troglodytes]_694950439
SAKDLAGQLTDHDWSLFNSIHQV----ELI-----HYVLG-PQHL----R--DV-TTA--
-------------------NLERFMCRFNELQYWVATELCLCPVPGPRAQLLRKFIKLA-
AHLKE-QKNLNSFFAVMFGLSNSAISRLAHTWERLPHKVRKLYSALERLLDPSWNHRVYR
LALAK--LSP-------PVIPF-MPLLLKDMTFI-------------------------
-----HEGNH--TLV-------E---NLINFE-KMRMMARAA---RMLHHCRSHN-----
-------------------PVPLSPLRSRVSHLHEDSQVAR----IST---CSEQS
>Rapgef3_[Panthera_tigris_altaica]_591291251
SAKDLAGQLTDHDWNLFNSIHQV----ELI-----HYVLG-PQHL----R--DV-TTA--
-------------------NLERFMRRFNELQYWVATELCLCPVPGLRAQLLRKFIKLA-
AHLKE-QKNLNSFFAVMFGLSNSAISRLAHTWERLPHKVRKLYSALERLLDPSWNHRVYR
LALTK--LSP-------PIIPF-MPLLLKDMTFI-------------------------
-----HEGNH--TLV-------E---NLINFE-KMRMMARAV---RMLHHCRSHS-----
-------------------NVPLSPLRSRVSHLHEDSQTAR----TST---CSEQS
>Rapgef3_[Pantholops_hodgsonii]_556771829
STKDLAGQLTDHDWSLFNSIHQV----ELI-----HYVLG-PQHL----R--DV-TAA--
-------------------NLERFMRRFNELQYWVATELCLCSVPGLRAQLLRKFIKLA-
AHLKE-QKNLNSFFAVMFGLSNSAISRLAHTWERLPHKVRKLYSALERLLDPSWNHRVYR
LALTK--LSP-------PLIPF-MPLLLKDMTFI-------------------------
-----HEGNH--TLV-------E---NLINFE-KMRMMARAA---RMLHHCRSHS-----
-------------------NVPLSPLRSRVSHLHEDSQAVR----VST---CSEQS
>Rapgef3_[Papio_anubis]_402885758
SAKDLAGQLTDHDWSLFNSIHQV----ELI-----YYALG-PQHL----R--DV-TTA--
-------------------NLERFMRRFNELQYWVATELCLCPVPGPRAQLLRKFIKLA-
AHLKE-QKNLNSFFAVMFGLSNSAISRLAHTWERLPHKVRKLYSALERLLDPSWNHRVYR
LALAK--LSP-------PVIPF-MPLLLKDMTFI-------------------------
-----HEGNH--TLV-------E---NLINFE-KMRMMARAA---RMLHHCRSHN-----
-------------------PVPLSPLRSRVSHLHEDSQVAR----IST---CSEQS
>Rapgef3_[Parus_major]_998744296
--KDLASHLTDYDWNLFKSIHQV----EMI-----HYIVG-PQKF----H--EV-TTA--
-------------------NLARVMRRFNELQFWVATELCLCPELGRRAQLLRKFIKLA-
AQCP------------------------------PH-----------AQDPSWNHRVYR
LAVAK--MSP-------PIIPF-MPLLLKDMTFI-------------------------
-----HEGNR--TLA-------E---NLINFE-KMHMMAKTV---RVLQRCRGHA-----
-------------------HAPLSPLRNRSPHRPEDPKAVR----IST---CSEQS
>Rapgef3_[Peromyscus_maniculatus_bairdii]_589923021
SAKDLAGQLTDHDWNLFNKIHQV----ELI-----HYVLG-PQHL----R--DV-TTA--
-------------------NLERFMRRFNELQYWVATELCLCPVPGPRAQLLRKFIKLA-
AHLKE-QKNLNSFFAVMFGLSNSAISRLAHTWERLPHKVRKLYSALERLLDPSWNHRVYR
LALTK--LSP-------PVIPF-MPLLLKDMTFI-------------------------
-----HEGNH--TLV-------E---NLINFE-KMRMMARAV---RMLHQCRSHS-----
-------------------TVPLSPLRSRVSHIHEDSQASR----IST---CSEQS
>Rapgef3_[Physeter_catodon]_593715976
STKDLAGQLTDHDWSLFNSIHQV----ELI-----HYVLG-PQPL----R--DV-TTA--

-------------------NLERFMRRFNELQYWVATELCLCPVPGLRAQLLRKFIKLA-
AHLKE-QKNLNSFFAIMFGLSNSAISRLAQTWERLPHKVRKLYSALERQLDPSWNHRVYR
LALTK--LSP-------PLIPF-MPLLLKDMTFI-------------------------
-----HEGNH--TLV-------E---NLINFE-KMRMMARAA---RILHHCRSHS-----
-------------------NVPLSPLRSRVSHLHEDSQVAR----IST---CSEQS
>Rapgef3_[Poecilia_formosa]_617405404
SSKDIATELTNYDWELFTAMHEV----ELV-----YYIFG-RHKF----P--GA-TTA--
-------------------NLERFVRHFNVVQHWVVTELCLCEDLVKRAILLKKFIKIA-
SVLKE-QRNLNSFFAVMFGLSNSSVQRLYKTWERIPSKTKRIYCAYERLMDPSRNHRAYR
LAVAK--LSP-------PYIPF-MPLLLKDMTFI-------------------------
-----HEGNP--NYV-------D---KLVNFE-KMRMLAKTV---KIVRGCRSQP-----
------------------Y-VPSSPQRGLADRMFLDGPATR----LSTY---SD--
>Rapgef3_[Poecilia_reticulata]_658859927
SSKDIATELTNYDWELFTAMHEV----ELV-----YYIFG-RHKF----P--GA-TTA--
-------------------NLERFVRHFNVVQYWVVTELCLCEDLVKRAILLKKFIKIA-
SVLKE-QRNLNSFFAVMFGLSNSAVQRLYKTWERIPSKTKRIYCAYERLMDPSRNHRAYR
LAVAK--LSP-------PYIPF-MPLLLKDMTFI-------------------------
-----HEGNP--NYV-------D---KLVNFE-KMRMLAKTV---KIVRGCRSQP-----
------------------Y-VPSSPQRGLADRMFLDGPATR----LSTY---SD--
>Rapgef3_[Pristionchus_pacificus]_802732026
GSAELATQLGFFHMELFHATNEL----ETI-----AQVFG-RDAF--PGH-----IPS--
-------------------NLDLLLRRFNEVQYWATTEVLVSPAP-KRVQSLRKLIKIA-
HYSKQ-QGDLLSLFAIVLGLSNVAVSRLSMTWEKLPSRVKRMFSELESLLDPTRNHRAYR
SLIAK--MQP-------PFVPF-IPLLLKDLTFI-------------------------
-----HEGNK--TFF-------N---GLVNFE-KMHMIANVI---RTFKECKASASGVTG
ILHR-DE---------------KPDSQLHIRN-LRVIDDQRRLLEMSYL---IEPK
>Rapgef3_[Propithecus_coquereli]_826277148
SAKDLAGQLTDHDWNLFNSIHQV----ELI-----HYVLG-PQHL----R--DV-TTA--
-------------------NLERFMRRFNELQYWVATELCLCSVPGLRAQLLRKFIKLA-
AHLKE-QKNLNSFFAVMFGLSNSAISRLAHTWQRLPHKVRKLYSALERLLDPSWNHRVYR
LALAK--LSP-------PVIPF-MPLLLKDMTFI-------------------------
-----HEGNH--TLV-------E---NLINFE-KMRMMARAV---RMLHHCRGHS-----
-------------------SVPLSPLRSRVSHLHEDGQALR----IST---CSEQS
>Rapgef3_[Protobothrops_mucrosquamatus]_1002588985
SSKDLANQITEHDWNLFKSLHQV----ELI-----YYVVG-SQKF----P--SA-TTV--
-------------------NVERFLRRFNELQFWITTELCLCPDVIKRAQLLRKFIKLA-
AHLKD-QRNLNSFFAVMFGLSHSAISRLSRTWEKLPYKTRKLYGTMERMLDPSWNHRVYR
LAVAK--LSP-------PMIPF-MPLLLKDMTFI-------------------------
-----HEGNR--TLV-------E---NLVNFE-KLRMMAKAV---HLVHHCRSHP-----
-------------------NLPLSPLRSRPQHLLEEARAMR----TST---CSVQS
>Rapgef3_[Pseudopodoces_humilis]_929508805
--KDLASHLTDYDWNLFKSIHQV----EMI-----HYIVG-PQKF----H--EV-TTA--
-------------------NLARVMRRFNELQFWVATELCLCPELGRRAQLLRKFIKLA-
AHLKE-QKNLNSFFAVMFGVSNTAVTRLAKTWERLPHKIRKLHSALERMLDPSWNHRVYR
LAVAK--MSP-------PIIPF-MPLLLKDMTFI-------------------------
-----HEGNR--TLA-------E---NLINFE-KMHMMAKTV---RVLQRCRGHA-----
-------------------HAPLSPLRNRSPHRPEDAKAVR----IST---CSEQS
>Rapgef3_[Pteropus_vampyrus]_759143390
SAKDLAGQLTDHDWNLFNSIHQV----ELI-----HYVLG-PQHL----R--DV-TTA--
-------------------NLERFMRRFNELQYWVATELCLCAVPGPRAQLLRKFIKLA-
AHLKE-QKNLNSFFAVMFGLSNSAISRLARTWERLPHKVRKLYSALERLLDPSWNHRVYR
LALTK--LSP-------PVIPF-MPLLLKDMTFI-------------------------
-----HEGNH--TLV-------E---NLINFE-KMRMMARAV---RMLHHCRSHS-----
-------------------NVPLSPLRSRVSHLHEDSQAVR----IST---CSEQS

>Rapgef3_[Pundamilia_nyererei]_548483158
SSKDIASELTNYDWELFTAMHEV----ELV-----YYIFG-RHKF----P--GA-ITA--
-------------------NLERFVRRFNEVQHWVLTELCLCEDLVKRAMLLKKFIKIA-
SVLKE-QKNLNSFFAVMFGLSNSAIQRLYKTWERIPSKTKRIYCSLERLMDPSRNHRAYR
LAIAK--LSP-------PYIPF-MPLLLKDMTFI--------------------------
-----HEGNA--NYV-------D---KLVNFE-KMRMLAKTV---KIVRGCRSQP-----
------------------Y-VPSSPQRGLADRMFLEGAATR----LSTY---SD--
>Rapgef3_[Python_bivittatus]_602664072
SSKDLANQFTEHDWNLFKSIHQV----ELI-----YYVVG-PQKF----P--SA-TTA--
-------------------NVERFLRRFNELQFWVTTELCLCPDVIKRAQLLRKFIKLA-
AHLKD-QKNLNSFFAVMFGLSHSAISRLSRTWEKLPHKTRKLYGTMERMLDPSWNHRVYR
LAVVK--LSP-------PMIPF-MPLLLKDMTFI--------------------------
-----HEGNR--TLV-------E---NLVNFE-KLRMMAKAV---RLVHHCRSHP-----
-------------------NLPLSPLRSRPQHLMEEARAMR----TST---CSEQS
>Rapgef3_[Rhinopithecus_roxellana]_724952752
SAKDLAGQLTDHDWSLFNSIHQV----ELI-----YYVLG-PQHL----R--DV-TTA--
-------------------NLERFMRRFNELQYWVATELCLCPVPGPRAQLLRKFIKLA-
AHLKE-QKNLNSFFAVMFGLSNSAISRLAHTWERLPHKVRKLYSALERLLDPSWNHRVYR
LALAK--LSP-------PVIPF-MPLLLKDMTFI--------------------------
-----HEGNH--TLV-------E---NLINFE-KMRMMARAA---RMLHHCRSHN-----
-------------------PVPLSPLRSRVSHLHEDSQVAR----IST---CSEQS
>Rapgef3_[Rousettus_aegyptiacus]_1012262855
SAKDLAGQLTDHDWNLFNSIHQV----ELI-----HYVLG-PQHL----R--DV-TTA--
-------------------NLERFMRRFNELQYWVATELCLCAVPGPRAQLLRKFIKLA-
AHLKE-QKNLNSFFAVMFGLSNSAISRLARTWERLPHKVRKLYSALERLLDPSWNHRVYR
LALTK--LSP-------PIIPF-MPLLLKDMTFI--------------------------
-----HEGNH--TLV-------E---NLINFE-KMRMMARAV---RMLHHCRSHS-----
-------------------NVPLSPLRSRVSHLHEDSQAVR----IST---CSEQS
>Rapgef3_[Saimiri_boliviensis_boliviensis]_403301658
SAKDLAGQLTDHDWSLFNSIHQV----ELI-----HYVLG-PQHL----R--DV-TTA--
-------------------NLERFMRRFNELQYWVATELCLCAVPGPRAQLLRKFIKLA-
AHLKE-QKNLNSFFAVMFGLSNSAISRLAHTWERLPHKVRKLYSALERLLDPSWNHRVYR
LALAK--LSP-------PVIPF-MPLLLKDMTFI--------------------------
-----HEGNH--TLV-------E---NLINFE-KMRMMARAA---RMLHHCRSHN-----
-------------------PVPLSPLRSRVSHLHEDSQVAR----IST---CSEQS
>Rapgef3_[Salmo_salar]_929230181
-SKDIASQLTNYDWELFTAMHEV----ELV-----YYIFG-RHKF----P--GA-TTA--
-------------------NLERFVHRFNEVQYWVVTEVCLCADLMKRAMLLKKFIKMA-
AMLKE-QKNLNSFFAVMFGLSNSAVQRLYKTWERVPSKTKRVYCAYERLMDPSRNHRAYR
LAVAK--LSP-------PYIPF-MPLLLKDMTFI--------------------------
-----HEGNK--NYT-------E---KLVNFE-KMRMIAKTV---KIVRGCRSTP-----
------------------Y-VPSSPQKGLADRMFLEAPSIR----VSTY---SEQS
>Rapgef3_[Sarcophilus_harrisii]_821489511
SAKDLANQLTDHDWNLFKSIHQV----ELI-----HYVLG-PQQL----R--NA-TTA--
-------------------NLERFMRRFNELQYWVATELCLCPVPGLRTQLLRKFIKLA-
SHLKE-QKNLNSFFAIMFGLSNSAISRLALTWERLPHKVRKLYSALEQLLDPSWNHRVYR
LALTK--LSP-------PIIPF-MPLLLK-------------------------------

>Rapgef3_[Scleropages_formosus]_938064599
SSKDIASQLSDYDWELFGTMPEV----ELV-----YYVFG-RHKF----P--GA-TTA--
-------------------NLERFVRRFNEVQYWVVTEMCLCADL-KRALLLKKFIKMA-
TILKE-QKNLNSFFAVMFGLSNCAVQRLSKTWE-----------------DPSRNHRAYR
LAVAK--LNP-------PYIPF-MPLLLKDMTFI--------------------------

-----HEGNN--NYT-------E---NLVNFE-KMRMIAKTV---KTVRECRSQP-----
------------------Y-IPSSPQRSLADRMFLDAPPVR----ISTY---SEQS
>Rapgef3_[Sinocyclocheilus_anshuiensis]_1025146268
-SKDMASQHTSYDWELFMAMHEV----ELV-----YYVLG-REKF----L--GA-TTA--
-------------------NLERFVRRFNEIQYWVVTELCLCEDLVKRAILLKKFIKMA-
VVFKE-QKNLNSFFAVMFGLSNSAVQRLNKTWERLPNKTKRIYCAYERLMDPSRNHRAYR
LAVAK--LSP-------PYIPF-MPLLLKDMTFI-------------------------
-----HEGNK--NYT-------D---KLVNFE-KMRMIARTV---KTVRQCRSQP-----
------------------Y-VPSSPQKGLTERMFLDAQAIR----LSTY---SDQS
>Rapgef3_[Sinocyclocheilus_grahami]_1020398505
-SKDMASQHTSYDWELFMAMHEV----ELV-----YYVLG-REKF----L--GA-TTA--
-------------------NLERFVRRFNEIQYWVVTELCLCEDLVKRAILLKKFIKMA-
VVFKE-QKNLNSFFAVMFGLSNSAVQRLNKTWERLPNKTKRIYCAYERLMDPSRNHRAYR
LAVAK--LSP-------PYIPF-MPLLLKDMTFI-------------------------
-----HEGNK--NYT-------D---KLVNFE-KMRMIARTV---KTVRQCRSQP-----
------------------Y-VPSSPQKGLTERMFLDAQAIR----LSTY---SDQS
>Rapgef3_[Sinocyclocheilus_rhinocerous]_1025211968
-SKDMASQHTSYDWELFMAMHEV----ELV-----YYVLG-REKF----L--GA-TTA--
-------------------NLERFVRRFNEIQYWVVTELCLCEDLVKRAILLKKFIKMA-
VVFKE-QKNLNSFFAVMFGLSNSAVQRLNKTWERLPNKTKRIYCAYERLMDPSRNHRAYR
LAVAK--LSP-------PYIPF-MPLLLKDMTFI-------------------------
-----HEGNK--NYT-------D---KLVNFE-KMHMIARTV---KTVRQCRSQP-----
------------------Y-VPSSPQKGLTERMFLDAQAIR----LSTY---SDQS
>Rapgef3_[Sorex_araneus]_836708039
SAKDLAGQLTEHDWSLFNSIHQV----ELI-----HYVLG-PQHL----R--DV-TTA--
-------------------NLERFLRRFNELQYWVATELCLCPVPGPRAQLLRKFIKLA-
AHLKE-QKNLNSFFAVMFGLSNSAISRLAHTWERLPQKVRKLYSALERLLDPSWNHRVYR
LALTK--LSP-------PVIPF-MPLLLKDMTFI-------------------------
-----HEGNH--TLV-------E---NLINFE-KMRMMARAV---RMLHHCRSHS-----
-------------------NVPLSPLRSRVSHLHEDSQAAR----IST---CSEQS
>Rapgef3_[Struthio_camelus_australis]_697484424
SSKDLASHLTDYDWNLFKSIHQV----EMI-----HYIIR-PQKF----H--DV-TTA--
-------------------NLERVMRRFNELQYWVATELCLCPEVGRRAQLLRKFIKLA-
AHLKE-QKNLNSFFAVMFGVSNTAVSRLAKTWEKLPHKIRKLHAALERMLDPSWNHRVYR
LAVAK--LSP-------PIIPF-VPLLLKDMTFI-------------------------
-----HEGNR--TLA-------E---NLINFE-KMHMMAKTV---RVLQRCRGHT-----
-------------------HAPVSPLRSRSPHRLEDPKAMR----IST---CSEQS
>Rapgef3_[Sturnus_vulgaris]_959091038
--------------ALTCRLSQV----EMI-----HYIVG-PHKS---HE---V-ATA--
-------------------NLARVLRRFNELQFWVATELCLCPELGRRARLLRKFIKLA-
AHLKE-QKNLNSFFAVMFGVSNTAVTRLAKTWEMLPHKIRKLHSALERMLLWSWWPWLLS
MAPTVPLVAPTVPLVA-PTVPL-V-----------------------------------

>Rapgef3_[Sus_scrofa]_545893683

-------------------------RRFNELQYWVATELCLCSVPSLRAQLLRKFIKLA-
AHLKE-QKNLNSFFAVMFGLSNSAISRLAHTWERLPHKVRKLYSALERLLDPSWNHRVYR
VALTK--LSP-------PVIPF-MPLLLK------------------------------

>Rapgef3_[Takifugu_rubripes]_768914882
GSKDIATELTNYDWELFAAMHEA----ELI-----YYVFG-RHKF----P--GA-TTA--
-------------------NLERFVRRFNEVQHWVVTELCLCEDLVKRAVLLKKFIKIA-

SVLKE-QKNLNSFFAVMFGLSNSAVRRLYKTWERIPSKTKRIYCSYERLMDPSRNHRAYR
LTVAK--LGP-------PYIPF-MPLLLKDMTFI-------------------------
-----NEGNP--NYL-------E---KLVNFE-KMRMIAKTV---KVVRGCRSHP-----
------------------Y-TPSSPQRSLADRMFLDGPTTR----MSTC---SE--
>Rapgef3_[Tinamus_guttatus]_719772822
SSKDLASHLTDYDWNLFKSIHQV----EMI-----HYIVR-PQKF----H--DV-TTA--
-------------------NLERVMRRFNELQYWVATELCLCPEVGRRAQLLRKFIKLA-
AHLKE-QKNLNSFFAVMFGVSNTAVSRLAKTWERLPHKIRKLHSALERMLDPSWNHRVYR
LAVAK--LSP-------PIIPF-VPLLLKDMTFI-------------------------
-----HEGNR--TLA-------E---NLINFE-KMHMMAKTV---RVLQRCQGHA-----
-------------------HAPLSPLRSRSPHRLEDPKAMR----IST---CSEQS
>Rapgef3_[Trichechus_manatus_latirostris]_823404739
SAKDLAGQLTDHDWSLFNSIHQV----ELI-----HYVLG-PQHL----R--TV-TTA--
-------------------NLERFMRRFNELQYWVATELCLCPVPGLRAQLLRKFIKLA-
AHLKE-QKNLNSFFAVMFGLSNSAISRLAHTWERLPHKVRKLYSALERLLDPSWNHRVYR
LALTK--LSP-------PVIPF-MPLLLKDITFI-------------------------
-----HEGNH--TLV-------E---NLINFE-KMRMMARAV---RMLHHCRSHS-----
-------------------NVPLSPLRSRVSHLHEDSQPSR----ISM---CSEQS
>Rapgef3_[Tupaia_chinensis]_444514991
SAKDLAGQLTDHDWNLFNSIHQV----ELI-----HYVLG-PQHL----R--DV-TTA--
-------------------NLERFMRRFNELQYWVATELCLCPVPGPRAQLLRKFIKLA-
AHLKE-QKNLNSFFAVMFGLSNSAISRLAHTWQRLPHKVRKLYSALERLLDPSWNHRVYR
LALTK--LSP-------PIIPF-MPLLLKDMTFI-------------------------
-----HEGNH--TLV-------E---NLINFE-KMRMMARAV---RMLHHCRSHS-----

>Rapgef3_[Tursiops_truncatus]_470596594
STKDLAGQLTDHDWSLFNSIHQV----ELI-----HYVLG-PQPL----R--DV-TTA--
-------------------NLERFMRRFNELQYWVATELCLCPVPGLRAQLLRKFIKLA-
AHLKE-QKNLNSFFAIMFGLSNSAISRLAQTWERLPHKVRKLYSALERLLDPSWNHRVYR
LALTK--LSP-------PLIPF-MPLLLKDMTFI-------------------------
-----HEGNH--TLV-------E---NLINFE-KMRMMARAA---RMLHHCRSHS-----
-------------------NVPLSPLRSRVSHLHEDSQAAR----IST---CSEQS
>Rapgef3_[Vicugna_pacos]_970713237
STKDLAGQLTDHDWNLFNSIHQV----ELI-----HYVLG-PQHL----R--DV-TTA--
-------------------NLERFMRRFNELQYWVATELCLCPVPGLRAQLLRKFIKLA-
AHLKE-QKNLNSFFAIMFGLSNSAISRLAHTWERLPHKVRKLYSALERLLDPSWNHRVYR
LALTK--LSP-------PVIPF-MPLLLKDMTFI-------------------------
-----HEGNH--TLV-------E---NLINFE-KM-------------------------

>Rapgef3_[Xenopus_laevis]_148227834
SSKDLAHQMTDNDWNLFKSIHQV----ELI-----YHTFG-KQRF----R--NA-TTA--
-------------------NLERFMRRFNEVQFWVATEVCLCQEEERRAQLLKKFIKLA-
AYLKE-QKNLNSFFAVMFGLSNTAVSRLSRTWQKLPNKIRKLYSIFERLMDPSWNHRSYR
LAVAK--LSA-------PLIPF-LPLILKDLTFL-------------------------
-----HEGNR--STL-------D---NLVNFE-KMRMIAKTV---QMFHRCRSQA-----
-------------------YVPLSPLRTRPVYILEDPSAAR----ISA---CSEHS
>Rapgef3_[Xenopus_tropicalis]_847104409
SSKDLAHQMTDNDWNLFKSIHQV----ELI-----YHTFG-KQRF----R--NA-TTA--
-------------------NLERFMRRFNEVQFWVATEVCLCQEEERRALLLKKFIKLA-
AYLKE-QKNLNSFFAVMFGLSNTAVSRLSRTWQKLPNKFRKLYSIFERLMDPSWNHRSYR
LAVAK--LSA-------PLIPF-LPLILKDLTFL-------------------------
-----HEGNR--STL-------D---SLVNFE-KMRMIAKTV---QMFHSCRSQA-----
-------------------YIPLSPLRPRPVNILEDPSAAR----ISA---CSEHS
>Rapgef4_[Acanthisitta_chloris]_677814020

SSKDLAHQMTIYDWELFNCVHEL----ELI-----YHTFG-RHNF--K-K-----TTA--
-------------------NLDLFLRRFNEIQFWVVTEICLCSQLSKRVQLLKKYIKIA-
AHCKE-YKNLNSFFAIIMGLSNVAVSRLSLTWEKLPSKFKKIYAEFESLMDPSRNHRAYR
LTVAK--LDP-------PIIPF-MPLLIKDMTFT-------------------------
-----HEGNK--TFT-------D---NLVNFE-KMRMIANTV---RTVKFCRSQSFNPDA
ALAN-KN---------------HQDVRSYVRQ-LNVIDNQRTLSQMSHR---LEPR
>Rapgef4_[Acinonyx_jubatus]_961712463
SSKDLAYQMTIYDWELFNCVHEL----ELI-----YHTFG-RHNF--K-K-----TTA--
-------------------NLDLFLRRFNEIQFWVVTEICLCSQPSKRVQLLKKFIKIA-
AHCKE-YKNLNSFFAIVMGLSNVAVSRLALTWEKLPSKFKKFYAEFESLMDPSRNHRAYR
LTVAK--LEP-------PLIPF-MPLLIKDMTFT-------------------------
-----HEGNK--TFI-------D---NLVNFE-KMRMIANTA---RTVRYCRSQPFNPDA
AQAN-KN---------------HQDVRSYVRQ-LNVIDNQRTLSQMSHR---LEPR
>Rapgef4_[Acropora_digitifera]_1005470793
GAKELAYFISQYDFALFNAVNVY----EFI-----YHVFG-KEKY---NQ---I--TA--
-------------------NLDMLLRRLNEVRFWIETQVLLTQQLSKRVYLLKTFIKLA-
AHCKE-LNNWNSFFAILMALNSVAVSRLTQTWEKVPRKFRNILEQFDSILEPTRNHRVYR
LLVGKQ--KP-------PILPF-VPLLIKDMTFA-------------------------
-----NEGNK--TYL-------D---DLVNFE-KMRMIASSV---RFFHYCRSEPFTADC
PSLG---R-------------PSQEIAAFVRD-WKVIDNERTLTQLSHG---LQPK
>Rapgef4_[Acyrthosiphon_pisum]_641671152
STKELAYHMTLFDWELFWNVHEY----ELI-----YLTFG-RHRF--Q-Q-----ITA--
-------------------NLDIFLRRFNEIQYWVITEICTSQNISKRTNILKKMIKLA-
TYCKE-YYNFNAFFAILMGLSDVAVSRLSTTWDKLPSKSRKQFTEYETLIDPSRNHRAYR
ITVGK--LPS-------PMIPF-MPLLIKDMKFT-------------------------
-----HDGNK--THV-------N---GLVNFE-KMHMLAQTM---RTLRYCRARHLVL-D
PPVS-KN---------------EADVKSYVSC-LRCVNNERTLMSMSQK---LEPR
>Rapgef4_[Ailuropoda_melanoleuca]_752414095
SSKDLAYQMTIYDWELFNCVHEL----ELI-----YHTFG-RHNF--K-K-----TTA--
-------------------NLDLFLRRFNEIQFWVVTEICLCSQPSKRVQLLKKFIKIA-
AHCKE-YKNLNSFFAIVMGLSNVAVSRLALTWEKLPSKFKKFYAEFESLMDPSRNHRAYR
LTVAK--LEP-------PLIPF-MPLLIKDMTFT-------------------------
-----HEGNK--TFI-------D---NLVNFE-KMRMIANTA---RTVRYCRSQAFNADV
AQAN-KN---------------HQDVRSYVRQ-LNVIDNQRTLSQMSHR---LEPR
>Rapgef4_[Alligator_mississippiensis]_564252547
SSKDLAYQMTIYDWELFNCVHEL----ELI-----YHTFG-RHHF--K-K-----TTA--
-------------------NLDLFLRRFNEIQFWVVTEISLCSQLSKRVQLLKKYIKIA-
AHCKE-YKNLNSFFAIIMGLSNVAVSRLTLTWEKLPSKFKKIYAEFESLMDPSRNHRAYR
LTVAK--LDP-------PIIPF-MPLLIKDMTFT-------------------------
-----HEGNK--TFI-------D---SLVNFE-KMRMIANTI---RTVKFCRSQSFNPDA
ALTN-KN---------------HQDVRSYVRQ-LNVIDNQRTLSQMSHR---LEPR
>Rapgef4_[Alligator_sinensis]_944357962
SSKDLAYQMTIYDWELFNCVHEL----ELI-----YHTFG-RHHF--K-K-----ITA--
-------------------NLDLFLRRFNEIQFWVVTEISLCSQLSKRVQLLKKYIKIA-
AHCKE-YKNLNSFFAIIMGLSNVAVSRLTLTWEKLPSKFKKIYAEFESLMDPSRNHRAYR
LTVAK--LDP-------PIIPF-MPLLIKDMTFT-------------------------
-----HEGNK--TFI-------D---SLVNFE-KMRMIANTI---RTVKFCRSQSFNPDA
ALTN-KN---------------HQDVRSYVRQ-LNVIDNQRTLSQMSHR---LEPR
>Rapgef4_[Amphimedon_queenslandica]_761901406
GSREIAAHLTSYDWNLFSNIQQM----ELI-----YQVFG-RHRF---SR---I--TS--
-------------------NLDVMIRRFNEVQYWTVTEICKESNLQKRVKIIQKFIKIA-
SHCKS-FNNLNCFFAIVVGLMNGAITRLKQTWEKVSVKLRRRYEQFEALMDPSRNHRVLR
AYQQK--LQP-------PIIPF-MPLIVKDAFFL-------------------------
-----QEGNE--TFV-------D---GLVNFE-KMRMVASKV---NDFSYYRKGSLANEI

KMLSNKN----------------SELQRYIRD-FKVIDSQQVLMQMSHA---IE--
>Rapgef4_[Anas_platyrhynchos]_874471726
SSKDLAHQMTIYDWELFNCVHEL----ELI-----YHTFG-RHNF--K-K-----TTA--
-------------------NLDLFLRRFNEIQFWVVTEICLCSQLSKRVQLLKKYIKIA-
AHCKE-YKNLNSFFAIIMGLSNVAVSRLSLTWEKLPSKFKKIYAEFESLMDPSRNHRAYR
LTVAK--LDP-------PIIPF-MPLLIKDMTFT--------------------------
-----HEGNK--TLT-------D---NLVNFE-KMRMIANTV---RTVKFCRSQSFNPDA
ALTN-KN---------------HQDVRSYVRQ-LNVIDNQRTLSQMSHR---LEPR
>Rapgef4_[Anolis_carolinensis]_637340171
SSKDLAYQMTIYEWELFNCVHEL----ELL-----YHTFG-RHSF--K-K-----TTA--
-------------------NLDLFLRRFNEIQFWVVTEICLCSQLSKRVQLLKKFIKIA-
AHCKE-YKNLNSFFALIMGLSNVAVSRLTMTWEKLPSKFKKIYAEFESLMDPSRNHRPYR
LAVAK--LEP-------PVIPF-MPLLIKDMTFT--------------------------
-----HEGNK--TFI-------D---SLVNFE-KMRMISNTV---RTVKFCRSQPFTPDA
SLAN-KN---------------HQDVRNYVRQ-LSVIDNQRTLSQMSHR---LEPR
>Rapgef4_[Anser_cygnoides_domesticus]_902873590
SSKDLAHQMTIYDWELFNCVHEL----ELI-----YHTFG-RHNF--K-K-----TTA--
-------------------NLDLFLRRFNEIQFWVVTEICLCSQLSKRVQLLKKYIKIA-
AHCKE-YKNLNSFFAIIMGLSNVAVSRLSLTWEKLPSKFKKIYAEFESLMDPSRNHRAYR
LTVAK--LDP-------PIIPF-MPLLIKDMTFT--------------------------
-----HEGNK--TLT-------D---NLVNFE-KMRMIANTV---RTVKFCRSQSFNPDA
ALTN-KN---------------HQDVRSYVRQ-LNVIDNQRTLSQMSHR---LEPR
>Rapgef4_[Aotus_nancymaae]_817294754
SSKDLAYQMTIYDWELFNCVHEL----ELI-----YHTFG-RHNF--K-K-----TTA--
-------------------NLDLFLRRFNEIQFWVVTEICLCSQLSKRVQLLKKFIKIA-
AHCKE-YKNLNSFFAIVMGLSNVAVSRLALTWEKLPSKFKKFYAEFESLMDPSRNHRAYR
LTVAK--LEP-------PLIPF-MPLLIKDMTFT--------------------------
-----HEGNK--TFI-------D---NLVNFE-KMHMIANTA---RTVRYYRSQPFNPDA
AQAN-KN---------------HQDVRSYVRQ-LNVIDNQRTLSQMSHR---LEPR
>Rapgef4_[Apaloderma_vittatum]_699612544
SSKDLAHQMTIYDWELFNCVHEL----ELI-----YHTFG-RHNF--K-K-----TTA--
-------------------NLDLFLRRFNEIQFWVVTEICLCSQLSKRVQLLKKYIKIA-
AHCKE-YKNLNSFFAIIMGLSNVAVSRLSLTWEKLPSKFKKIYAEFESLMDPSRNHRAYR
LTVAK--LDP-------PIIPF-MPLLIKDMTFT--------------------------
-----HEGNK--TFT-------D---NLVNFE-KMRMIANTV---RTVKFCRSQSFNPDA
ALTN-KN---------------HQDVRSYVRQ-LNVIDNQRTLSQMSHR---LEPR
>Rapgef4_[Aptenodytes_forsteri]_686601170
-SKDLAHQMTIYDWELFNCVHEL----ELI-----YHTFG-RHNF--K-K-----TTA--
-------------------NLDLFLRRFNEIQFWVVTEICLCSQLSKRVQLLKKYIKIA-
AHCKE-YKNLNSFFAIIMGLSNVAVSRLSLTWEKLPSKFKKIYAEFESLMDPSRNHRAYR
LTVAK--LDP-------PIIPF-MPLLIKDMTFT--------------------------
-----HEGNK--TFT-------D---NLVNFE-KMRMIANTV---RTVKFCRSQSFNPDA
ALTN-KN---------------HQDVRSYVRQ-LNVIDNQRTLSHMSHR---LELR
>Rapgef4_[Apteryx_australis_mantelli]_926533754
SSKDLAYQMTIYDWEFFNCVHEL----ELI-----YHTFG-RHNF--K-K-----TTA--
-------------------NLDLFLRRFNEIQFWVVTEICLCSQLSKRVQLLKKYIKIA-
AHCKE-YKNLNSFFAIIMGLSNVAVSRLSLTWEKLPSKFKKIYAEFESLMDPSRNHRAYR
LTVTK--LDP-------PIIPF-MPLLIKDMTFT--------------------------
-----HEGNK--TLT-------D---NLVNFE-KMRMIANTV---RTVKFCRSQSFNPDA
ALAN-KN---------------HQDVRSYVRQ-LNVIDNQRTLSQMSHR---LEPR
>Rapgef4_[Aquila_chrysaetos_canadensis]_768339852
SSKDLAHQMTIYDWELFNCVHEL----ELI-----YHTFG-RHNF--K-K-----TTA--
-------------------NLDLFLRRFNEIQFWVVTEICLCSQLSKRVQLLKKYIKIA-
AHCKE-YKNLNSFFAIIMGLSNVAVSRLSLTWEKLPSKFKKIYAEFESLMDPSRNHRAYR

LTVAK--LDP-------PIIPF-MPLLIKDMTFT-------------------------
-----HEGNK--TFT-------D---NLVNFE-KMRMIANTV---RTVKFCRSQSFNPDA
ALTN-KN---------------HQDVRSYVRQ-LNVIDNQRTLSQMSHR---LEPR
>Rapgef4_[Astyanax_mexicanus]_597745805
SSKDLAYQLTMYDWELFNCVHEH----ELI-----YHTFG-RQHF--K-K-----TTA--
-------------------NLDLFLRRFNEIQLWVVTEVCLCSALSKRVQLLKKFIKIA-
AHCKE-FRNLNSFFSIIMGLGNPAVSRLSQTWEKLPSKFKKFYGEFENLMDPSRNHRAYR
LTVAK--LDP-------PIIPF-MPLLIKDMTFT-------------------------
-----HEGNK--TFI-------D---SLVNFE-KMRMIANTV---RIMRYCRSQPFTHEA
PQAT-KN---------------HQDVRSYIRQ-ISVIDNQRTLSQLSHK---LEPR
>Rapgef4_[Bactrocera_cucurbitae]_751444016
STKEIAYQMTLFDWDLFWAVHEY----ELL-----YQTFG-RHHF--G-K-----ITA--
-------------------NLDVFLRRFNELQFWIVTEIVSTSSMCKRVGLLRKFVKLA-
AYCKE-YQNLNAFFAIIMGLSNMAVSRLTQTWEKVPSKFRKLFQEFEALIDPSRNHRAYR
VYVGK--LQP-------PVIPF-MPLLLKDMTFA-------------------------
-----HEGNK--TSL-------E---GLVNFE-KMHMMAQTM---RTIRFCRSRSLGL-E
PPSP-KS---------------EGEVRSYISC-LRVIDNQRVLTAMSQK---IEP-
>Rapgef4_[Bactrocera_dorsalis]_751799864
STKEIAYQMTLFDWDLFWAVHEY----ELL-----YQTFG-RHHF--G-K-----ITA--
-------------------NLDVFLRRFNELQFWIATEIVTTSSMCKRVGLLRKFIKLA-
AYCKE-YQNLNAFFAITMGLSNMAVLRLTQTWEKIPSKFRKLFQEFEALIDPSRNHRAYR
VYVGK--LQP-------PVIPF-IPLLLKDMTFA-------------------------
-----HEGNK--TSL-------E---GLVNFE-KMHMMAQTM---RTIRFCRSRSLGL-E
PPSP-KS---------------EGEVRSYISC-LRVIDNQRVLTAMSQK---IEP-
>Rapgef4_[Bactrocera_oleae]_929345093
STKEIAYQMTLFDWDLFWAVHEY----ELL-----YQTFG-RHHF--G-K-----ITA--
-------------------NLDVFLRRFNELQFWIATEIVSTSSMCKRVGLLRKFIKLA-
AYCKE-YQNLNAFFAITMGLSNMAVSRLTQTWEKIPSKFRKLFQEFEALIDPSRNHRAYR
VYVGK--LQP-------PVIPF-MPLLLKDMTFA-------------------------
-----HEGNK--TSL-------E---GLVNFE-KMHLMAQTM---RTIRFCRSRSLGL-E
PPSP-KS---------------EGEVRSYISC-LRVIDNQRVLTAMSQK---IEP-
>Rapgef4_[Balaenoptera_acutorostrata_scammoni]_594670782
SSKDLAYQMTVYDWDLFSCVHEL----ELI-----YHTFG-RHHF--K-K-----TTA--
-------------------NLDLFLRRFNEIQFWVVTEICLCSQPSKRVQLLKKFIKIA-
AHCKE-YKNLNSFFAIVMGLSNVAVSRLALTWEKLPSKFKKFYAEFESLMDPSRNHRAYR
LTVAK--LDP-------PLIPF-MPLLIKDMTFT-------------------------
-----HEGNK--TFI-------D---NLVNFE-KMRMIANTA---RTVRYCRSQPFNPDA
AQAN-KN---------------HQDVRSYVRQ-LNVIDNQRTLSQMSHR---LEPR
>Rapgef4_[Balearica_regulorum_gibbericeps]_676248813
SSKDLAHQMTIYDWELFNCVHEL----ELI-----YHIFG-RHNF--K-K-----TTA--
-------------------NLDLFLRRFNEIQFWVVTEICLCSQLSKRVQLLKKYIKIA-
AHCKE-YKNLNSFFAIIMGLSNVAVSRLSLTWEKLPSKFKKIYAEFESLMDPSRNHRAYR
LTVAK--LDP-------PIIPF-MPLLIKDMTFT-------------------------
-----HEGNK--TFT-------D---NLVNFE-KM------------------------

>Rapgef4_[Bison_bison_bison]_742139618
SSKDLAYQMTIYDWELFNCVHEL----ELI-----YHTFG-RHHF--K-K-----TTA--
-------------------NLDLFLRRFNEIQFWVVTEICLCSQPSKRVQLLKKFIKIA-
AHCKE-YKNLNSFFAIVMGLSNVAVSRLALTWEKLPSKFKKFYAEFESLMDPSRNHRAYR
LTVAK--LDP-------PLIPF-MPLLIKDMTFT-------------------------
-----HEGNK--TFI-------D---NLVNFE-KMRMIANTA---RTVRYCRSQPFNPDA
AQAN-KN---------------HQDVRSYVRQ-LNVIDNQRTLSQMSHR---LEPR
>Rapgef4_[Bos_mutus]_555950853
SSKDLAYQMTIYDWELFNCVHEL----ELI-----YHTFG-RHHF--K-K-----TTA--

```
-------------------NLDLFLRRFNEIQFWVVTEICLCSQPSKRVQLLKKFIKIA-
AHCKE-YKNLNSFFAIVMGLSNVAVSRLALTWEKLPSKFKKFYAEFESLMDPSRNHRAYR
LTVAK--LDP-------PLIPF-MPLLIKDMTFT--------------------------
-----HEGNK--TFI-------D---NLVNFE-KMRMIANTA---RTVRYCRSQPFNPDA
AQAN-KN---------------HQDVRSYVRQ-LNVIDNQRTLSQMSHR---LEPR
>Rapgef4_[Bos_taurus]_982913240
SSKDLAYQMTIYDWELFNCVHEL----ELI-----YHTFG-RHHF--K-K-----TTA--
-------------------NLDLFLRRFNEIQFWVVTEICLCSQPSKRVQLLKKFIKIA-
AHCKE-YKNLNSFFAIVMGLSNVAVSRLALTWEKLPSKFKKFYAEFESLMDPSRNHRAYR
LTVAK--LDP-------PLIPF-MPLLIKDMTFT--------------------------
-----HEGNK--TFI-------D---NLVNFE-KMRMIANTA---RTVRYCRSQPFNPDA
AQAN-KN---------------HQDVRSYVRQ-LNVIDNQRTLSQMSHR---LEPR
>Rapgef4_[Bubalus_bubalis]_594078797
SSKDLAYQMTIYDWELFNCVHEL----ELI-----YHTFG-RHHF--K-K-----TTA--
-------------------NLDLFLRRFNEIQFWVVTEICLCSQPSKRVQLLKKFIKIA-
AHCKE-YKNLNSFFAIVMGLSNVAVSRLALTWEKLPSKFKKFYAEFESLMDPSRNHRAYR
LTVAK--LDP-------PLIPF-MPLLIKDMTFT--------------------------
-----HEGNK--TFI-------D---NLVNFE-KMRMIANTA---RTVRYCRSQPFNPDA
AQAN-KN---------------HQDVRSYVRQ-LNVIDNQRTLSQMSHR---LEPR
>Rapgef4_[Buceros_rhinoceros_silvestris]_704181635
SSKDLAHQMTIYDWELFNCVHEL----ELI-----YHTFG-RHNF--K-K-----TTA--
-------------------NLDLFLRRFNEIQFWVVTEICLCSQLSKRVQLLKKYIKIA-
AHCKE-YKNLNSFFAIIMGLSNVAVSRLSLTWEKLPSKFKKIYAEFESLMDPSRNHRAYR
LTVAK--LDP-------PIIPF-MPLLIKDMTFT--------------------------
-----HEGNK--TFT-------D---NLVNFE-KMRMIANTV---RTVKFCRSQSFNPDA
ALTS-KN---------------HQDVRSYVRQ-LNVIDNQRTLSQMSHR---LEPR
>Rapgef4_[Callithrix_jacchus]_296204512
SSKDLAYQMTIYDWELFNCVHEL----ELI-----YHTFG-RHNF--K-K-----TTA--
-------------------NLDLFLRRFNEIQFWVVTEICLCSQLSKRVQLLKKFIKIA-
AHCKE-YKNLNSFFAIVMGLSNVAVSRLALTWEKLPSKFKKFYAEFESLMDPSRNHRAYR
LTIAK--LEP-------PLIPF-MPLLIKDMTFT--------------------------
-----HEGNK--TFI-------D---NLVNFE-KMRMIANTA---RTVRYYRSQPFNPDA
AQAN-KN---------------HQDVRSYVRQ-LNVIDNQRTLSQMSHR---LEPR
>Rapgef4_[Callorhinchus_milii]_632945698
SSKDLAHQITIYDWELFNCVHEL----ELV-----YHTFG-RQNF--K-K-----TTA--
-------------------NLDLFLRRFNEIQFWVVTETCLCPQLSKRVQLLKKFIKVA-
AHCKE-YRNLNSFFAIVMGLSNVAVSRLSQTWEKLPSKFKKIYSEFESLMDPSRNHRAYR
LTVAK--LES-------PIIPF-MPLLIKDMTFT--------------------------
-----HEGNK--TFI-------D---NLVNFE-KMHMIGNTI---RTVRYCRSQSFNTD-
SIAN-KN---------------HQDVRVYVRQ-LNVIDNQRTLSQLSHR---LEPR
>Rapgef4_[Calypte_anna]_663246135
SSKDLAHQMTIYDWELFNCVHEL----ELI-----YHTFG-RHNF--K-K-----TTA--
-------------------NLDLFLRRFNEIQFWVVTEICLCSQLSKRVQLLKKYIKIA-
AHCKE-YKNLNSFFAIIMGLSNVAVSRLSLTWEKLPSKFKKIYAEFESLMDPSRNHRAYR
LTVAK--LDP-------PVIPF-MPLLIKDMTFT--------------------------
-----HEGNK--TFT-------D---NLVNFE-KMRMIANTV---RTVKFCRSQSFNPDA
ALTN-KN---------------HQDVRSYVRQ-LNVIDNQRTLSQMSHR---LEPR
>Rapgef4_[Camelus_bactrianus]_743729785
SSKDLAYQMTIYDWELFNCVHEL----ELI-----YHTFG-RHHF--K-K-----TTA--
-------------------NLDLFLRRFNEIQFWVVTEICLCSQPSKRVQLLKKFIKIA-
AHCKE-YKNLNSFFAIVMGLSNVAVSRLALTWEKLPSKFKKFYAEFESLMDPSRNHRAYR
LTVAK--LEP-------PLIPF-MPLLIKDMTFT--------------------------
-----HEGNK--TFI-------D---NLVNFE-KMRMIANTA---RMVRYCRSQPFNPDA
AQAN-KN---------------HQDVRSYVRQ-LNVIDNQRTLSQMSHR---LEPR
```

```
>Rapgef4_[Camelus_dromedarius]_744571846
SSKDLAYQMTIYDWELFNCVHEL----ELI-----YHTFG-RHHF--K-K-----TTA--
-------------------NLDLFLRRFNEIQFWVVTEICLCSQPSKRVQLLKKFIKIA-
AHCKE-YKNLNSFFAIVMGLSNVAVSRLALTWEKLPSKFKKFYAEFESLMDPSRNHRAYR
LTVAK--LEP-------PLIPF-MPLLIKDMTFT--------------------------
-----HEGNK--TFI-------D---NLVNFE-KMRMIANTA---RMVRYCRSQPFNPDA
AQAN-KN---------------HQDVRSYVRQ-LNVIDNQRTLSQMSHR---LEPR
>Rapgef4_[Camelus_ferus]_560926404
SSKDLAYQMTIYDWELFNCVHEL----ELI-----YHTFG-RHHF--K-K-----TTA--
-------------------NLDLFLRRFNEIQFWVVTEICLCSQPSKRVQLLKKFIKIA-
AHCKE-YKNLNSFFAIVMGLSNVAVSRLALTWEKLPSKFKKFYAEFESLMDPSRNHRAYR
LTVAK--LEP-------PLIPF-MPLLIKDMTFT--------------------------
-----HEGNK--TFI-------D---NLVNFE-KMRMIANTA---RMVRYCRSQPFNPDA
AQAN-KN---------------HQDVRSYVRQ-LNVIDNQRTLSQMSHR---LEPR
>Rapgef4_[Canis_lupus_familiaris]_928181558
SSKDLAYQMTIYDWELFNCVHEL----ELI-----YHTFG-RHNF--K-K-----TTA--
-------------------NLDLFLRRFNEIQFWVVTEICLCSQPSKRVQLLKKFIKIA-
AHCKE-YKNLNSFFAIVMGLSNVAVSRLALTWEKLPSKFKKFYAEFESLMDPSRNHRAYR
LTVAK--LEP-------PLIPF-MPLLIKDMTFT--------------------------
-----HEGNK--TFI-------D---NLVNFE-KMRMIANTA---RTVRFCRSQPFNPDA
AQAN-KN---------------HQDVRSYVRQ-LNVIDNQRTLSQMSHR---LEPR
>Rapgef4_[Capra_hircus]_926684811
SSKDLAYQMTIYDWELFNCVHEL----ELI-----YHTFG-RHHF--K-K-----TTA--
-------------------NLDLFLRRFNEIQFWVVTEICLCSQPSKRVQLLKKFIKIA-
AHCKE-YKNLNSFFAIVMGLSNVAVSRLALTWEKLPSKFKKFYAEFESLMDPSRNHRAYR
LTVAK--LDP-------PLIPF-MPLLIKDMTFT--------------------------
-----HEGNK--TFI-------D---NLVNFE-KMRMIANTA---RTVRYCRSQPFNPDA
AQAN-KN---------------HQDVRSYVRQ-LNVIDNQRTLSQMSHR---LEPR
>Rapgef4_[Caprimulgus_carolinensis]_704296106
SSKDLAHQMTVYDWELFNCVHEL----ELI-----YHTFG-RHNF--K-K-----TTA--
-------------------NLDLFLRRFNEIQFWVVTEICLCSQLSKRVQLLKKYIKIA-
AHCKE-YKNLNSFFAIIMGLSNVAVSRLSLTWEKLPSKFKKIYAEFESLMDPSRNHRAYR
LTVAK--LDP-------PIIPF-MPLLIKDMTFT--------------------------
-----HEGNK--TFT-------D---NLVNFE-KMRMIANTV---RTVKFCRSQSFNPDA
ALTN-KN---------------HQDVRSYVRQ-LNVIDNQRTLSQMSHR---LEPR
>Rapgef4_[Capsaspora_owczarzaki_ATCC_30864]_470294198
---DVARELTILDSQLFRDIQVQ----EYV-----EHLFK-LGIA----Q--TD-A----
--------------------LNKFVDRFNMINFWVVNEIVMTRDVKRRVDVIKAFVAIG-
KHLRT-FKNYNSLFAIVSGLSNTASTRLKETWEKVPKRVSESLKELEALMNPSRNMGIYR
ALLADSSA---------PMTPF-FPLLIKDLVFI--------------------------
-----HEGNP--SKV-------D---GMINFE-KLRMIARTL---RTI------------
--------------------------------------------------------
>Rapgef4_[Cariama_cristata]_698410374
SSKDLAHQMTIYDWELFNCVHEL----ELI-----YHTFG-RHNF--K-K-----TTA--
-------------------NLDLFLRRFNEIQFWVVTEICLCSQLSKRVQLLKKYIKIA-
AHCKE-YKNLNSFFAIIMGLSNVAVSRLSLTWEKLPSKFKKIYAEFESLMDPSRNHRAYR
LTVAK--LDP-------PIIPF-MPLLIKDMTFT--------------------------
-----HEGNK--TIT-------D---NLVNFE-KMRMIANTV---RTVKFCRSQSFNPDA
ALTN-KN---------------HQDVRSYVRQ-LNVIDNQRTLSQMSHR---LEPR
>Rapgef4_[Carlito_syrichta]_640812551
SSKDLAFQMTIYDWELFNCVHEL----ELI-----YHTFG-RHNF--K-K-----TTA--
-------------------NLDLFLRRFNEIQFWVVTEICLCSQLSKRVQLLKKFIKIA-
AHCKE-YKNLNSFFAIVMGLSNIAVSRLALTWEKLPSKFKKFYAEFESLMDPSRNHRAYR
LTVAK--LDP-------PLIPF-TPLLIKDMTFT--------------------------
```

-----HEGNK--TFV-------D---NLVNFE-KMRMIANTA---RTVRYYRSQPFNPDA
AQAN-KN---------------HQDVRSYVRQ-LNVIDNQRTLSQMSHR---LEPR
>Rapgef4_[Cathartes_aura]_677231281
SSKDLAHQMTIYDWELFNCVHEL----ELI-----YHTFG-RHNF--K-K-----TTA--
-------------------NLDLFLRRFNEIQFWVVTEICLCSQLSKRVQLLKKYIKIA-
AHCKE-YKNLNSFFAIIMGLSNVAVSRLSLTWEKLPSKFKKIYAEFESLMDPSRNHRAYR
LTVAK--LDP-------PIIPF-MPLLIKDMTFT--------------------------
-----HEGNK--TFT-------D---NLVNFE-KMRMIANTV---RTVKFCRSQSFNPDA
ALTN-KN---------------HQDVRSYVRQ-LNVIDNQRTLSQMSHR---LEPR
>Rapgef4_[Cavia_porcellus]_884941748
SSKDLAYQMTIYDWELFNCVHEL----ELI-----YHTFG-RHNF--K-K-----TTA--
-------------------NLDLFLRRFNEIQFWVVTEICLCSQLSKRVQLLKKFIKIA-
AHCKE-YKNLNSFFAIVMGLSNVAVSRLALTWEKLPSKFKKFYAEFESLMDPSRNHRAYR
LTAAK--LEP-------PLIPF-MPLLIKDMTFT--------------------------
-----HEGNK--TFI-------D---NLVNFE-KMRMIANTA---RTVRYYRSQPFSPDA
AQAN-KN---------------HQDVRSYVRQ-LNVIDNQRTLSQMSHR---LEPR
>Rapgef4_[Cebus_capucinus_imitator]_1044377132
SSKDLAYQMTVYDWELFNCVHEL----ELI-----YHTFG-RHNF--K-K-----TTA--
-------------------NLDLFLRRFNEIQFWVVTEICLCSQLSKRVQLLKKFIKIA-
AHCKE-YKNLNSFFAIVMGLSNVAVSRLALTWEKLPSKFKKFYAEFESLMDPSRNHRAYR
LTAAK--LEP-------PLIPF-MPLLIKDMTFT--------------------------
-----HEGNK--TFI-------D---NLVNFE-KMRMIANTA---RTVRYYRSQPFNPDA
AQAN-KN---------------HQDVRSYVRQ-LNVIDNQRTLSQMSHR---LEPR
>Rapgef4_[Ceratitis_capitata]_499006314
STKDLAFYITIYDWDLFWVVHEY----ELL-----YRTFG-RHHF--G-K-----ITA--
-------------------NLDVFLRRFNELQYWIVTDIVSASSMSKRVGLLRKFIKLA-
AYCKE-YNNLNAFFAIVMGLSNMAVSRLTQTWDKIPSKFRKLFQEFEALIDPSRNHRAYR
VYVGK--LQP-------PLIPF-MPLLLKDMTFA--------------------------
-----HEGNK--TSL-------D---GLVNFE-KMHMMAQTM---RTMRYCRSRTISL-D
PPSP-KS---------------EGDARSYICC-LRAIDNQRVLTAMSQK---LEP-
>Rapgef4_[Ceratotherium_simum_simum]_478504778
SSKDLAYQMTIYDWELFNCVHEL----ELI-----YHTFG-RHNF--K-K-----TTA--
-------------------NLDLFLRRFNEIQFWVVTEICLCPQPSKRVQLLKKFIKIA-
AHCKE-YKNLNSFFAIVMGLSNVAVSRLALTWEKLPSKFKKFYAEFESLMDPSRNHRAYR
LTVAK--LEP-------PLIPF-MPLLIKDMTFT--------------------------
-----HEGNK--TFI-------D---NLVNFE-KMRMIANTA---RTVRYCRSQPFNPDA
AQAN-KN---------------HQDVRSYVRQ-LNVIDNQRTLSQMSHR---LEPR
>Rapgef4_[Chaetura_pelagica]_701389410
SSKDLAHQMTIYDWELFNCVHEL----ELI-----YHTFG-RHNF--K-K-----TTA--
-------------------NLDLFLRRFNEIQFWVVTEICLCSQLSKRVQLLKKYIKIA-
AHCKE-YKNLNSFFAIIMGLSNVAVSRLSLTWEKLPSKFKKIYAEFESLMDPSRNHRAYR
LTVAK--LDP-------PIIPF-MPLLIKDMTFT--------------------------
-----HEGNK--TFT-------D---NLVNFE-KMRMIASTV---RTVKFCRSQSFNPDA
ALTN-KN---------------HQDVRSYVRQ-LNVIDNQRTLSQMSHR---LEPR
>Rapgef4_[Charadrius_vociferus]_699632744
SSKDLAHQMTIYDWELFNCVHEL----ELI-----YHTFG-RHNF--K-K-----TTA--
-------------------NLDLFLRRFNEIQFWVVTEICLCSQLSKRVQLLKKYIKIA-
AHCKE-YKNLNSFFAIIMGLSNVAVSRLSLTWEKLPSKFKKIYAEFESLMDPSRNHRAYR
LTVAK--LDP-------PIIPF-MPLLIKDMTFT--------------------------
-----HEGNK--TFT-------D---NLVNFE-KMRMIANTV---RTVKFCRSQSFNPDA
ALTN-KN---------------HQDVRSYVRQ-LNVIDNQRTLSQMSHR---LEPR
>Rapgef4_[Chelonia_mydas]_465978877
SSKDLAYQMTIYDWELFNCVHEL----ELI-----YHTFG-THNF--K-K-----TTA--
-------------------NLDLFLRRFNEIQFWVVTEICLCSQLSKRVQLLKKFIKIA-

AHCKE-YKNLNSFFAIIMGLSNVAVSRLSLTWEKLPSKFKKIYAEFESLMDPSRNHRAYR
LTVAK--LDP-------PIIPF-MPLLIKDMTFT--------------------------
-----HEGNK--TFI-------D---NLVNFE-KMRMIANTA---RTVKFCRSQSFNPDA
ALTN-KN---------------HQDVRTYVRQ-LNVIDNQRTLSQMSHR---LEPR
>Rapgef4_[Chinchilla_lanigera]_918612490
SSKDLAYQMTLYDWELFNCVHEL----ELI-----YHTFG-RHNF--K-K-----TTA--
-------------------NLDLFLRRFNEIQFWVVTEICLCSQLSKRVQLLKKFIKIA-
AHCKE-YKNLNSFFAIVMGLSNVAVSRLALTWEKLPSKFKKFYAEFESLMDPSRNHRAYR
LTAAK--LEP-------PLIPF-MPLLIKDMTFT--------------------------
-----HEGNK--TFI-------D---NLVNFE-KMRMIANTA---RTVRYYRSQPFSPDA
AQAN-KN---------------HQDVRSYVRQ-LNVIDNQRTLSQMSHR---LEPR
>Rapgef4_[Chlamydotis_macqueenii]_705667844
SSKDLAHQMTIYDWELFNCVHEL----ELI-----YHTFG-RHNF--K-K-----TTA--
-------------------NLDLFLRRFNEIQFWVVTEICLCSQLSKRVQLLKKYIKIA-
AHCKE-YKNLNSFFAIIMGLSNVAVSRLSLTWEKLPSKFKKIYAEFESLMDPSRNHRAYR
LTVAK--LEP-------PIIPF-MPLLIKDMTFT--------------------------
-----HEGNK--TFA-------D---NLVNFE-KMRMIANTV---RTVKFCRSQSFNPDA
ALTN-KN---------------HQDVRSYVRQ-LNVIDNQRTLSQMSHR---LEPR
>Rapgef4_[Chlorocebus_sabaeus]_635058803
SSKDLAYQMTIYDWELFNCVHEL----ELI-----YHTFG-RHNF--K-K-----TTA--
-------------------NLDLFLRRFNEIQFWVVTEICLCSQLSKRVQLLKKFIKIA-
AHCKE-YKNLNSFFAIVMGLSNVAVSRLALTWEKLPSKFKKFYAEFESLMDPSRNHRAYR
LTVAK--LEP-------PLIPF-MPLLIKDMTFT--------------------------
-----HEGNK--TFI-------D---NLVNFE-KMRMIANTA---RTVRYYRSQPFNLDA
AQAN-KN---------------HQDVRSYVRQ-LNVIDNQRTLSQMSHR---LEPR
>Rapgef4_[Chrysochloris_asiatica]_586472254
SSKDLAYQMTIYDWELFNCVHEL----ELI-----YHTFG-RHHF--K-K-----TTA--
-------------------NLDLFLRRFNEIQFWVVTEICLCSQLSKRVQLLKKFIKIA-
AHCKE-YKNLNSFFAIVMGLSNVAVSRLALTWEKLPSKFKKFYAEFESSMDPSRNHRAYR
LTVAK--LEP-------PLIPF-MPLLIKDMTFT--------------------------
-----HEGNK--TFI-------D---NLVNFE-KMRMIANTA---RTVRYCRSQPFNPDA
AQAN-KN---------------HQDVRSYVRQ-LNVIDNQRTLSQMSHR---VEPR
>Rapgef4_[Cimex_lectularius]_939265223
SAKEMAYHLTMFDWELFWNIHEY----ELL-----YHTFG-RHRF--G-Q-----NTA--
-------------------NLDVFLRRFNELQYWVVTEICLTQSLSKRVHVLRKIIKIA-
SYCKE-FHNINGMFALVLGLSNVAVSRLSATWDKLPSKSRKLYTQLEATIDPSRNHRAYR
AVVTS--MSS-------PLIPF-MPLLLKDMTFI--------------------------
-----HEGNK--TMV-------D---GLVNFE-KMHMLAQTL---RTLRYCRNRHMVI-D
PPTP-KN---------------ESEVRAYVRC-LRTIDNQRTLNSHSQK---LEPR
>Rapgef4_[Ciona_intestinalis]_699251985

---KM-YGNMFGFMAVMIGIGNQAISRLHGTWDKLPTKYRKLVDEFETILDPSRKHRNYR
VFVSS--TNS-------ARIPY-FPLLMKDLTFQ--------------------------
-----HEGND--TFNK------N---GLVNFE-KMHMLSNSI---RLVVHGCSRSLDNQF
FEST-KTA---------------EESRNYTRE-VSVITNQILLNEMSNK---LEPK
>Rapgef4_[Clupea_harengus]_831318321
SSKDLAYQMTLCDWELFNCVHEL----EVV-----YHTFG-RQHF--K-K-----TTA--
-------------------NLDLFLRRFNEIQLWVITEVCLCPQLSKRVQLLKKFIKIA-
AHCKE-YRNLNSFFAIIMGMSNPAVSRLSQTWEKLPSKFKKFYGEFENLMDPSRNHRAYR
LTMAK--LHP-------PIIPF-MPLLIKDMTFT--------------------------
-----HEGNK--TYI-------D---SLVNFE-KMRMIANTV---RILRYCRSQPFSVDA
SQVN-KN---------------HQDVRSYMHQ-LNVIDNQRTLSQLSHK---LEPR
>Rapgef4_[Colius_striatus]_706115660

SSKDLAHQMTIYDWELFNCVHEL----ELI-----YHTFG-RHNF--K-K-----TTA--
-------------------NLDLFLRRFNEIQFWVVTEICLCSQLSKRVQLLKKYIKIA-
AHCKE-YKNLNSFFAIIMGLSNVAVSRLSLTWEKLPSKFKKIYAEFESLMDPSRNHRAYR
LTVAK--LDP-------PIIPF-MPLLIKDMTFT--------------------------
-----HEGNK--TFT-------D---NLVNFE-KMRMIANTV---RTVKFCRSQSFNPDA
ALTN-KN---------------HQDVRSYVRQ-LNVIDNQRTLSQMSHR---LEPR
>Rapgef4_[Colobus_angolensis_palliatus]_795183856
SSKDLAYQMTIYDWELFNCMHEL----ELI-----YHTFG-RHNF--K-K-----TTA--
-------------------NLDLFLRRFNEIQFWVVTEICLCSQLSKRVQLLKKFIKIA-
AHCKE-YKNLNSFFAIVMGLSNVAVSRLALTWEKLPSKFKKFYAEFESLMDPSRNHRAYR
LTVAK--LEP-------PLIPF-MPLLIKDMTFT--------------------------
-----HEGNK--TFI-------D---NLVNFE-KMRMIANTA---RTVRYYRSQPFNPDA
AQAN-KN---------------HQDVRSYVRQ-LNVIDNQRTLSQMSHR---LEPR
>Rapgef4_[Columba_livia]_449270176
SSKDLAHQMTIYDWELFNCVHEL----ELI-----YHTFG-RHNF--K-K-----TTA--
-------------------NLDLFLRRFNEIQFWVVTEICLCSQLSKRVQLLKKYIKIA-
AHCKE-YKNLNSFFAIIMGLSNVAVSRLSLTWEKLPSKFKKIYAEFESLMDPSRNHRAYR
LTVAK--LDP-------PIIPF-MPLLIKDMTFT--------------------------
-----HEGNK--TFT-------D---NLVNFE-KMRMIANTV---RTVKFCRSQSFNPDA
ALTN-KN---------------HQDVRSYVRQ-LNVIDNQRTLSQMSHR---LEPR
>Rapgef4_[Condylura_cristata]_829983202
SSKDLAYQMTIYDWELFNCVHEL----ELI-----YHTFG-RHNF--K-R-----TTA--
-------------------NLDLFLRRFNEIQFWVVTEICLCSQLSKRVQLLKKFIKIA-
AHCKE-YKNLNSFFAIVMGLSNVAVSRLALTWEKLPSKFKKFYAEFESLMDPSRNHRTYR
LTVAK--LEP-------PLIPF-MPLLIKDMTFT--------------------------
-----HEGNK--TFI-------D---NLVNFE-KMRMIANTA---RTVRYYRSQPFNPDA
AQAN-KN---------------HQDVRSYVRQ-LNVIDNQRTLSQMSHR---LEPR
>Rapgef4_[Corvus_brachyrhynchos]_669280989
SSKDLAHQMTVYDWELFNCVHEL----ELI-----YHTFG-RHNF--K-K-----TTA--
-------------------NLDLFLRRFNEIQFWVVTEICLCSQLSKRVQLLKKYIKIA-
AHCKE-YKNLNSFFAIIMGLSNVAVSRLSLTWEKLPSKFKKIYAEFESLMDPSRNHRAYR
LTVAK--LDP-------PIIPF-MPLLIKDMTFT--------------------------
-----HEGNK--TFT-------D---NLVNFE-KMRMIANTV---RTVKFCRSQSFNPDA
ALTN-KN---------------HQDVRSYVRQ-LNVIDNQRTLSQMSHR---LEPR
>Rapgef4_[Corvus_cornix_cornix]_727051287
SSKDLAHQMTVYDWELFNCVHEL----ELI-----YHTFG-RHNF--K-K-----TTA--
-------------------NLDLFLRRFNEIQFWVVTEICLCSQLSKRVQLLKKYIKIA-
AHCKE-YKNLNSFFAIIMGLSNVAVSRLSLTWEKLPSKFKKIYAEFESLMDPSRNHRAYR
LTVAK--LDP-------PIIPF-MPLLIKDMTFT--------------------------
-----HEGNK--TFT-------D---NLVNFE-KMRMIANTV---RTVKFCRSQSFNPDA
ALTN-KN---------------HQDVRSYVRQ-LNVIDNQRTLSQMSHR---LEPR
>Rapgef4_[Coturnix_japonica]_1003923803
SSKDLAHQMTIYDWELFNCVHEL----ELI-----YHTFG-RHNF--K-K-----TTA--
-------------------NLDLFLRRFNEIQFWVVTEICLCSQLSKRVQILKKYIKIA-
AHCKE-YKNLNSFFAIIMGLSNVAVSRLSLTWEKLPSKFKKIYAEFESLMDPSRNHRAYR
LTVAK--LDP-------PIIPF-MPLLIKDMTFT--------------------------
-----HEGNK--TLT-------D---NLVNFE-KMRMIANTV---RTVKFCRSQSFNPDA
ALAN-KN---------------HQDVRSYVRQ-LNVIDNQRTLSQMSHR---LEPR
>Rapgef4_[Crassostrea_gigas]_405960606
---------------------------ELI-----YQVLG-RSNF--N-K-----ITA--
-------------------NLDLFLRRFNEVQYWVVTEMVLAQNVGKRVQLLRKFIKVA-
AHCKE-FQNLHSFFAIVMGLSNIAVSRLSQTWEKLPGKFKKMFADFETLMDPSRNHRVYR
LSVSK--LTP-------PIIPF-MPLLMKDLTFT--------------------------
-----HDGNK--TYF-------D---GLVNFE-KMHMIAQTI---RNVRICRSRRLDLE-

PPNTAKS---------------STEVQDYIRN-LQVIDNQRVLTQLSYK---LEPR
>Rapgef4_[Cricetulus_griseus]_344239574
SSKDLAYQMTTYDWELFNCVHEL----ELI-----YHTFG-RHNF----K---K-TTA--
-------------------NLDLFLRRFNEIQFWVVTEICLCSQLSKRVQLLKKFIKIA-
AHCKE-YKNLNSFFAIIMGLSNVAVSRLALTWEQ-------------------------

>Rapgef4_[Cynoglossus_semilaevis]_1035265328
SSKDLAYQMTMFDWELFSCVHEH----ELL-----YHTFG-RQSF--R-R-----TTA--
-------------------NLDLFLRRFNQVQLWVVTEVCLCGQLSKRVQLLKKFIKIA-
AHCRE-FKNLNSFFAIIMGMSNPAVSRLSQTWEKLPTKFKKFYAEFESMMDPSRNHRSYR
LTVTK--LEP-------PIIPF-TPLLLKDMTFT-------------------------
-----HEGNK--TFI-------D---NMVNFE-KMRMIANTI---RQVRHCRGQPFNPDI
CQPN-KN---------------QAEVRGYVRK-LCVIDNQRALTQLSYR---LEPR
>Rapgef4_[Cyprinodon_variegatus]_974086889
SSKDLAYQMTMFDWELFSCVHEH----ELL-----YHTFG-RHSF--R-R-----TTA--
-------------------NLDLFLRRFNQVQLWVVTEVCLCTQLSKRVQLLKKFIKIA-
AHCRE-FKNLNSFFAIIMGMSNPAVSRLSQTWEKLPTKFKKFYAEFESMM----------

>Rapgef4_[Danio_rerio]_688569967
SSKDLAHQMTLYDWELFHCVHEH----ELI-----YHTFG-RHHF--K-K-----TTA--
-------------------NLDLFLRRFNEVQLWVVTEVCLCPALSKRVQLLKKFIKIA-
AHCKE-FKNLNSFFAIIMGLGNPAVCRLSQTWEKLPSKFKKFYGEFENLMDPSRNHRAYR
LTMAK--LEP-------PIIPF-MPLLIKDMTFT-------------------------
-----HEGNK--TFT-------D---RLVNFE-KMRMIANTV---RIIRYCRSQPFDQEA
PQATGKS---------------HQDVRTYVRH-ISVIDNQRSLSQLSHR---LEPR
>Rapgef4_[Dasypus_novemcinctus]_488582892
SSKDLAYQMTIYDWELFNCVHEM----ELI-----YHTFG-RHNF--K-K-----TTA--
-------------------NLDLFLRRFNEIQFWVVTEICLCSQLSKRVQLLKKFIKIA-
AHCKE-YKNLNSFFAIVMGLSNVAVSRLALTWEKLPSKFKKFYAEFESLMDPSRNHRAYR
LTAAK--LEP-------PLIPF-MPLLIKDMTFT-------------------------
-----HEGNK--TFT-------D---NLVNFE-KMRMIANTA---RTVRYCRSQPFNLDA
AQGN-KN---------------HQDVRSYVRQ-LNVIDNQRTLSQLSHR---LEPR
>Rapgef4_[Diaphorina_citri]_1041549174
SAKELAFHMTLFDWDLFWSIHEY----ELV-----YHTVG-RHRF--Q-Q-----ITA--
-------------------NLDVFLRRFNEIQYWVITEILLVTSLNKRVQILRKMIKLA-
AYCKE-YRNINALFAVLMGLSNVAVSRLSLTWDKLPSKSKKTYTELEALIDPSKNHRAYR
QAVSK--LQS-------PVIPF-MPLLLKDLAFT-------------------------
-----HDGNK--TVV-------D---GLVNFE-KMHMFAQTL---RTLRYCRTR------

>Rapgef4_[Dipodomys_ordii]_852730826
SSKDLAYQMTIYDWELFNCVHEL----ELI-----YHTFG-RHNF--K-K-----TTA--
-------------------NLDLFLRRFNEIQFWVVTEICLCSQLSKRVQLLKKFIKIA-
AHCKE-YKNLNSFFAIIMGLSNVAVSRLALTWEKLPSKFKKFYAEFESLMDPSRNHRAYR
LTAAK--LDP-------PLIPF-MPLLIKDMTFT-------------------------
-----HEGNK--TFI-------D---NLVNFE-KMRMIANSA---RTVRYYRSQPFNLDA
AQAN-KN---------------HQDVRSYVRQ-LSVIDNQRTLSQMSHR---LEPR
>Rapgef4_[Diuraphis_noxia]_985397275
STKELAYHMTLFDWELFWNVHEY----ELI-----YLTFG-RHRF--Q-Q-----ITA--
-------------------NLDIFLRRFNEIQYWVITEICTSQNISKRTNILKKMIKLA-
TYCKE-YYNFNAFFAILMGLSDVAVSRLSTTWDKLPSKSRKQFTEYETLIDPSRNHRAYR

ITVGK--LPS-------PMIPF-MPLLIKDMKFT-------------------------
-----HDGNK--THV-------D---GLVNFE-KMHMLAQTM---RTLRYCRARHLVL-D
PPVS-KN---------------EADVKSYVSC-LRCVNNERTLMSMSQK---LEPR
>Rapgef4_[Drosophila_bipectinata]_1037099499
STKELAYHITLFEWDLFWAVHEY----ELL-----YHTFG-RHHF--G-K-----ITA--
-------------------NLDVFLRRFNEVQYWIVTELVSTPSLSKRVGLVRKFIKLA-
AYCKE-YQNLNAFFAVVMGLSNMAVSRLQQTWEKIPSKFRKIFQEFEALIDPSRNHRAYR
VFVGK--LQP-------PLIPF-MPLLLKDMTFA-------------------------
-----HEGNK--TSL-------D---GLVNFE-KMHMMAQTM---RTIRFCRSRSLGL-E
PPSP-KS---------------EGEVRSYISS-FRVIDNQRVLTAMSQK---VEP-
>Rapgef4_[Drosophila_eugracilis]_1036854417
STKELAYHITLFEWDLFWAVHEY----ELL-----YHTFG-RHHF--G-K-----ITA--
-------------------NLDVFLRRFNEVQYWIVTELVSTPSLSKRVGLVRKFIKLA-
AYCKE-YQNLNAFFAVVMGLSNMAVSRLQQTWEKIPSKFRKIFQEFEALIDPSRNHRAYR
VFVGK--LQP-------PLIPF-MPLLLKDMTFA-------------------------
-----HEGNK--TSL-------D---GLVNFE-KMHMMAQTM---RTIRFCRSRSLGL-E
PPSP-KS---------------EGEVRSYISS-FRVIDNQRVLTAMSQK---VEP-
>Rapgef4_[Drosophila_kikkawai]_1036730969
STKELAYHITLFEWDLFWAVHEY----ELL-----YHTFG-RHHF--G-K-----ITA--
-------------------NLDVFLRRFNEVQYWIVTELVSTPSLSKRVGLVRKFIKLA-
AYCKE-YQNLNAFFAVVMGLSNMAVTRLQQTWEKIPSKFKKIFQEFEALIDPSRNHRAYR
VFVGK--LQP-------PLIPF-MPLLLKDMTFA-------------------------
-----HEGNK--TSL-------D---GLVNFE-KMHMMAQTM---RTIRFCRSRSLGL-E
PPSP-KS---------------EGEVRSYISS-FRVIDNQRVLTAMSQK---VEP-
>Rapgef4_[Drosophila_miranda]_1037006478
STKELAYHITLFEWDLFYAVHEY----ELL-----YHTFG-RHHF--G-K-----ITA--
-------------------NLDVFLRRFNEVQYWIVTELVSTPSLSKRVGLVRKFIKLA-
AYCKE-YQNLNAFFAVVMGLSNMAVSRLHQTWEKIPSKFRKIFQEFEALIDPSRNHRAYR
VFVGK--LQP-------PLIPF-MPLLLKDMTFA-------------------------
-----HEGNK--TSL-------D---GLVNFE-KMHMMAQTM---RTIRFCRSRSLGL-E
PPSP-KS---------------EGEVRSYISS-FRVIDNQRVLTAMSQK---VEP-
>Rapgef4_[Drosophila_rhopaloa]_1036871102
STKELAYHITLFEWDLFWAVHEY----ELL-----YHTFG-RHHF--G-K-----ITA--
-------------------NLDVFLRRFNEVQYWIVTELVSTPSLSKRVGLVRKFIKLA-
AYCKE-YQNLNAFFAVVMGXSNMAVSRLQQTWEKIPSKFRKIFQEFEALIDPSRNHRAYR
VFVGK--LQP-------PLIPF-MPLLLKDMTFA-------------------------
-----HEGNK--TSL-------D---GLVNFX-KMHMMAQTM---RTIRFCRSRSLGL-E
PPSP-KS---------------EGEVRSYISS-FRVIDNQRVLTAMSQK---VEP-
>Rapgef4_[Drosophila_suzukii]_1036073995
STKELAYHITLFEWDLFWAVHEY----ELL-----YHTFG-RHHF--G-K-----ITA--
-------------------NLDVFLRRFNEVQYWIVTELVSTPSLSKRVGLVRKFIKLA-
AYCKE-YQNLNAFFAVVMGLSNMAVSRLQQTWEKIPSKFRKIFQEFEALIDPSRNHRAYR
VFVGK--LQP-------PLIPF-MPLLLKDMTFA-------------------------
-----HEGNK--TSL-------D---GLVNFE-KMHMMAQTM---RTIRFCRSRSLGL-E
PPSP-KS---------------EGEVRSYISC-FRVIDNQRVLTAMSQK---VEP-
>Rapgef4_[Drosophila_takahashii]_1036922079
STKELAYHITLFEWDLFWAVHEY----ELL-----YHTFG-RHHF--G-K-----ITA--
-------------------NLDVFLRRFNEVQYWIVTELVSTPSLSKRVGLIRKFIKLA-
AYCKE-YQNLNAFFAVVMGLSNMAVSRLQQTWEKIPSKFRKIFQEFEALIDPSRNHRAYR
VFVGK--LQP-------PLIPF-MPLLLKDMTFA-------------------------
-----HEGNK--TSL-------D---GLVNFE-KMHMMAQTM---RTIRFCRSRSLGL-E
PPSP-KS---------------EGEVRTYISS-FRVIDNQRVLTAMSQK---VEP-
>Rapgef4_[Echinops_telfairi]_507650143
SSKDLAYQMTIYDWELFHCVHEL----ELI-----YHTFG-RHNF--K-K-----TTA--

-------------------NLDLFLRRFNEIQFWVVTEVCLCSQLSKRVQLLKKFIKIA-
AHCKE-YKNLNAFFAIVMGLSNVAVSRLALTWEKLPSKFKKFYAEFESLMDPSRNHRAYR
LTVAK--LEA-------PLIPF-MPLLIKDMTFI--------------------------
-----HEGNK--TFI-------D---NLVNFE-KMRMIANTA---RTVRYCRSQPFNPDA
AQAN-KN---------------HQDVRSYVRQ-LNVIDNQRTLSQMSHR---LEPR
>Rapgef4_[Egretta_garzetta]_697837445
SSKDLAHQMTIYDWELFNCVHEL----ELI-----YHTFG-RHNF--K-K-----TTA--
-------------------NLDLFLRRFNEIQFWVVTEICLCSQLSKRVQLLKKYIKIA-
AHCKE-YKNLNSFFAIIMGLSNVAVSRLSLTWEKLPSKFKKIYAEFESLMDPSRNHRAYR
LTVAK--LDP-------PIIPF-MPLLIKDMTFT--------------------------
-----HEGNK--TFT-------D---NLVNFE-KMRMIANTV---RTVKFCRSQSFNPDA
ALTN-KN---------------HQDVRSYVRQ-LNVIDNQRTLSHMSHR---LEPR
>Rapgef4_[Elephantulus_edwardii]_585637321
SSKDLAYQMTIYDWELFNCVHEL----ELI-----YHTFG-RHNF--K-K-----TTA--
-------------------NLDLFLRRFNEIQFWVVTEICLCSQLSKRVQLLKKFIKIA-
AHCKE-YKNLNSFFAIVMGLSNVAVSRLALTWEKLPSKFKKFYAEFESLMDPSRNHRAYR
LTVAK--LEP-------PLIPF-MPLLIKDMTFT--------------------------
-----HEGNK--TFI-------D---NLVNFE-KMRMIANTA---RTVRYCRSQPFNPDA
AQAN-KN---------------HQDVRSYVRQ-LNVIDNQRTLSQMSHR---LEPR
>Rapgef4_[Eptesicus_fuscus]_641696747
SSKDLAYQMTIYDWELFNCVHEL----ELI-----YHTFG-RHNF--K-K-----TTA--
-------------------NLDLFLRRFNEIQFWVVTEICLCSQPSKRVQLLKKFIKIA-
AHCKE-YKNLNSFFAIVMGLSNVAVSRLALTWEKLPSKFKKFYAEFESLMDPSRNHRAYR
LTVAK--LEP-------PLIPF-MPLLIKDMTFT--------------------------
-----HEGNK--TFI-------D---NLVNFE-KMRMIANTA---RTVRYCRSQPFNLDA
AQAN-KN---------------HQDVRSYVRQ-LNVIDNQRTLSQMSHR---LEPR
>Rapgef4_[Equus_asinus]_958716129
SSKDLAYQMTIYDWELFNCVHEL----ELI-----YHTFG-RHNF--K-K-----TTA--
-------------------NLDLFLRRFNEIQFWVVTEICLCPQPSKRVQLLKKFIKIA-
AHCKE-YKNLNSFFAIVMGLSNVAVSRLALTWEKLPSKFKKFYAEFESLMDPSRNHRAYR
LTVAK--LDP-------PLIPF-MPLLIKDMTFT--------------------------
-----HEGNK--TFI-------D---NLVNFE-KMRMIANTA---RTVRYCRSQPFNPDA
AQAN-KN---------------HQDVRSYVRQ-LNVIDNQRTLSQMSHR---LEPR
>Rapgef4_[Equus_caballus]_953879137
SSKDLAYQMTIYDWELFNCVHEL----ELI-----YHTFG-RHNF--K-K-----TTA--
-------------------NLDLFLRRFNEIQFWVVTEICLCPQPSKRVQLLKKFIKIA-
AHCKE-YKNLNSFFAIVMGLSNVAVSRLALTWEKLPSKFKKFYAEFESLMDPSRNHRAYR
LTIAK--LDP-------PLIPF-MPLLIKDMTFT--------------------------
-----HEGNK--TFI-------D---NLVNFE-KMRMIANTA---RTVRYCRSQPFNPDA
AQAN-KN---------------HQDVRSYVRQ-LNVIDNQRTLSQMSHR---LEPR
>Rapgef4_[Erinaceus_europaeus]_1016663854
SSKDLAYQMTIYDWELFNCVHEL----ELI-----YHTFG-RHNF--K-K-----TTA--
-------------------NLDLFLRRFNEIQFWVVTEICLCSQLSKRVQLLKKFIKIA-
AHCKE-YKNLNSFFAIVMGLSNVAVSRLALTWEKLPSKFKKFYAEFESLMDPSRNHRAYR
LTVAK--LEP-------PLIPF-MPLLIKDMTFT--------------------------
-----HEGNK--TFI-------D---NLVNFE-KMRMIANTA---RTVRYCRSQPFNQLP
IEQS-LS---------------EKSKRSVILV-SNYAYNLKKIS------------
>Rapgef4_[Esox_lucius]_742138478
SSKDLAYQMTVYDWELFHCVHEH----ELI-----YHTFG-RQNF--Q-K-----TTA--
-------------------NMDLFLRRFNEIQLWVITEICLCAQLSKRVQLVKKFIKIA-
AHCKE-YKNLNSFFAIIMGMSNPAVSRLSQTWEKLPSKFRKFYSEFESLMDPSRNHRVYR
LTVAK--LDP-------PIIPF-MPLLIKDMTFT--------------------------
-----HDGNK--TFT-------D---SLVNFE-KMRMIANTV---RIVRYCRSLPFSAEP
SQTS-KN---------------HPDVRSYVRQ-LAVIDNQRTLSQLSQR---LEPR

>Rapgef4_[Eurypyga_helias]_704267608
SSKDLAHQMTVYDWELFNCVHEL----ELI-----YHTFG-RHNF--K-K-----TTA--
-------------------NLDLFLRRFNEIQFWVVTEICLCSQLSKRVQLLKKYIKIA-
AHCKE-YKNLNSFFAIIMGLSNVAVSRLLLTWEKLPSKFKKIYAEFESLMDPSRNHRAYR
LTVAK--LDP-------PIIPF-MPLLIKDMTFT--------------------------
-----HEGNK--TFT-------D---NLVNFE-KMRMIANTV---RTVKFCRSQSFNPDA
ALTS-KN---------------HQDVRSYVRQ-LNVIDNQRTLSQMSHR---LEPR
>Rapgef4_[Falco_peregrinus]_909796784
SSKDLAHQMTIYDWELFNCVHEL----ELI-----YHTFG-RHNF--K-K-----TTA--
-------------------NLDLFLRRFNEIQFWVVTEICLCSQLSKRVQLLKKYIKIA-
AHCKE-YKNLNSFFAIIMGLSNVAVSRLSLTWEKLPSKFKKIYAEFESLMDPSRNHRAYR
LTVAK--LDP-------PIIPF-MPLLIKDMTFT--------------------------
-----HEGNK--TFT-------D---NLVNFE-KMRMIANTV---RTVKFCRSQSFNPDV
ALTN-KN---------------HQDVRSYVRQ-LNVIDNQRTLSQMSHR---LEPR
>Rapgef4_[Felis_catus]_755759060
SSKDLAYQMTIYDWELFNCVHEL----ELI-----YHTFG-RHNF--K-K-----TTA--
-------------------NLDLFLRRFNEIQFWVVTEICLCSQPSKRVQLLKKFIKIA-
AHCKE-YKNLNSFFAIVMGLSNVAVSRLALTWEKLPSKFKKFYAEFESLMDPSRNHRAYR
LTVAK--LEP-------PLIPF-MPLLIKDMTFT--------------------------
-----HEGNK--TFI-------D---NLVNFE-KMRMIANTA---RTVRYCRSQPFNPDA
AQAN-KN---------------HQDVRSYVRQ-LNVIDNQRTLSQMSHR---LEPR
>Rapgef4_[Ficedula_albicollis]_525003360
SSKDLAHQMTIYDWELFNCVHEL----ELI-----YHTFG-RHNF--K-K-----TTA--
-------------------NLDLFLRRFNEIQFWVVTEICLCTQLSKRVQLLKKYIKIA-
AHCKE-YKNLNSFFAIIMGLSNVAVSRLSLTWEKLPSKFKKIYAEFESLMDPSRNHRAYR
LTVAK--LDP-------PIIPF-MPLLIKDMTFT--------------------------
-----HEGNK--TFT-------D---NLVNFE-KMRMIANTV---RTVKFCRSQSFNPDA
ALTN-KN---------------HQDVRSYVRQ-LNVIDNQRTLSQMSHR---LEPR
>Rapgef4_[Fukomys_damarensis]_731272285
SSKDLAYQMTIYDWELFNCVHEL----ELI-----YHTFG-RHNF--K-K-----TTA--
-------------------NLDLFLRRFNEIQFWVVTEICLCSQLSKRVQLLKKFIKIA-
AHCKE-YKNLNSFFAIVMGLSNVAVSRLALTWEKLPSKFKKFYAEFESLMDPSRNHRAYR
LTAAK--LEP-------PLIPF-MPLLIKDMTFT--------------------------
-----HEGNK--TFI-------D---NLVNFE-KMRMIANTA---RTVRYYRSQPFNPDA
AQAN-KN---------------HQDVRSYVRQ-LNVIDNQRTLSQMSHR---LEPR
>Rapgef4_[Fundulus_heteroclitus]_831555062
SSKDLAYQMTMFDWELFSCVHEH----ELL-----YHTFG-RHSF--R-R-----TTA--
-------------------NMDLFLRRFNQVQLWVVTEVCLCTQLSKRVQLLKKFIKIA-
AHCRE-FKNLNSFFAIIMGMSNPAVSRLSQTWEKLPTKFKKFYAEFESMMDPSRNHRSYR
LTVTK--LEP-------PIIPF-MPLLLKDMTFT--------------------------
-----HEGNK--TFI-------D---NMVNFE-KMRIIANTI---RQVRHCRSQPFNPDI
CQPN-KN---------------QADVRGYVRK-LCVIDNQRALTQLSYR---LEPR
>Rapgef4_[Galeopterus_variegatus]_667341846

----------------------------------KLPSKFKKFYAEFESLMDPSRNHRAYR
LTVAK--LEP-------PLIPF-MPLLIKDMTFT--------------------------
-----HEGNK--TFT-------D---NLVNFE-KMRMIANTA---RTVRYYRSQPFNPDA
AQAN-KN---------------HQDVRSYVRQ-LNVIDNQRTLSQMSHR---LEPR
>Rapgef4_[Gallus_gallus]_971405305
SSKDLAHQMTIYDWELFNCVHEL----ELI-----YHTFG-RHNF--K-K-----TTA--
-------------------NLDLFLRRFNEIQFWVVTEICLCSQLSKRVQLLKKYIKIA-
AHCKE-YKNLNSFFAIIMGLSNVAVSRLSLTWEKLPSKFKKIYAEFESLMDPSRNHRAYR
LTVAK--LDP-------PIIPF-MPLLIKDMTFT--------------------------

-----HEGNK--TLT-------D---NLVNFE-KMRMIANTV---RTVKFCRSQSFNPDA
ALAN-KN---------------HQDVRSYVRQ-LNVIDNQRTLSQMSHR---LEPR
>Rapgef4_[Geospiza_fortis]_930230268
SSKDLAHQMTIYDWELFNCVHEL----ELI-----YHTFG-RHNF--K-K-----TTA--
-------------------NLDLFLRRFNEIQFWVVTEICLCSQLSKRVQLLKKYIKIA-
AHCKE-YKNLNSFFAIIMGLSNVAVSRLSLTWEKLPSKFKKIYAEFESLMDPSRNHRAYR
LTVAK--LDP-------PIIPF-MPLLIKDMTFT--------------------------
-----HEGNK--TFT-------D---NLVNFE-KMRMIANTV---RTVKFCRSQSFNPDA
ALTN-KN---------------HQDVRSYVRQ-LNVIDNQRTLSQMSHR---LEPR
>Rapgef4_[Gorilla_gorilla_gorilla]_426337733
SSKDLAYQMTIYDWELFNCVHEL----ELI-----YHTFG-RHNF--K-K-----TTA--
-------------------NLDLFLRRFNEIQFWVVTEICLCSQLSKRVQLLKKFIKIA-
AHCKE-YKNLNSFFAIVMGLSNVAVSRLALTWEKLPSKFKKFYAEFESLMDPSRNHRAYR
LTVAK--LEP-------PLIPF-MPLLIKDMTFT--------------------------
-----HEGNK--TFI-------D---NLVNFE-KMRMIANTA---RTVRYYRSQPFNPDA
AQAN-KN---------------HQDVRSYVRQ-LNVIDNQRTLSQMSHR---LEPR
>Rapgef4_[Haliaeetus_albicilla]_700359613
SSKDLAHQMTIYDWELFNCVHEL----ELI-----YHTFG-RHNF--K-K-----TTA--
-------------------NLDLFLRRFNEIQFWVVTEICLCSQLSKRVQLLKKYIKIA-
AHCKE-YKNLNSFFAIIMGLSNVAVSRLSLTWEKLPSKFKKIYAEFESLMDPSRNHRAYR
LTVAK--LDP-------PIIPF-MPLLIKDMTFT--------------------------
-----HEGNK--TFT-------D---NLVNFE-KMRMIANTV---RTVKFCRSQSFNPDA
ALTN-KN---------------HQDVRSYVRQ-LNVIDNQRTLSQMSHR---LEPR
>Rapgef4_[Haliaeetus_leucocephalus]_729721426
SSKDLAHQMTIYDWELFNCVHEL----ELI-----YHTFG-RHNF--K-K-----TTA--
-------------------NLDLFLRRFNEIQFWVVTEICLCSQLSKRVQLLKKYIKIA-
AHCKE-YKNLNSFFAIIMGLSNVAVSRLSLTWEKLPSKFKKIYAEFESLMDPSRNHRAYR
LTVAK--LDP-------PIIPF-MPLLIKDMTFT--------------------------
-----HEGNK--TFT-------D---NLVNFE-KMRMIANTV---RTVKFCRSQSFNPDA
ALTN-KN---------------HQDVRSYVRQ-LNVIDNQRTLSQMSHR---LEPR
>Rapgef4_[Halyomorpha_halys]_939661167
SAKEMAYHMTMFDWELFWNIHEY----ELL-----YHTFG-RHRF--G-Q-----STA--
-------------------NLDAFLRRFNELQYWVVTEICLTQSLSKRVQVLRKIIKLA-
AYCKQ-YHNINGMFALVLGLSNMAVSRLSLTWDKLPSKSRKLYMQLEATIDPSKNHRAYR
GVVSS--MDS-------PLIPF-MPLLLKDMTFI--------------------------
-----HEGNK--TMV-------D---GLVNFE-KMHMLAQTL---RTLRYCRSRQLIIED
PPTP-KN---------------ESEVRAYVRC-LRTIDNQRTLTSLSQK---LEPR
>Rapgef4_[Heterocephalus_glaber]_861469824
SSKDLAYQMTIYDWELFNCVHEL----ELI-----YHTFG-RHNF--K-K-----TTA--
-------------------NLDLFLKRFNEIQFWVVTEICLCSQLSKRVQLLKKFIKIA-
AHCKE-YKNLNSFFAIVMGLSNVAVSRLALTWEKLPSKFKKFYAEFESLMDPSRNHRAYR
LTAAK--LEP-------PLIPF-MPLLIKDMTFT--------------------------
-----HEGNK--TFI-------D---NLVNFE-KMRMIANTA---RTVRYYRSQPFNPDA
AQAN-KN---------------HQDVRSYVRQ-LNVIDNQRTLSQMSHR---LEPR
>Rapgef4_[Homo_sapiens]_62088942
SSKDLAYQMTIYDWELFNCVHEL----ELI-----YHTFG-RHNF--K-K-----TTA--
-------------------NLDLFLRRFNEIQFWVVTEICLCSQLSKRVQLLKKFIKIA-
AHCKE-YKNLNSFFAIVMGLSNVAVSRLALTWEKLPSKFKKFYAEFESLMDPSRNHRAYR
LTVAK--LEP-------PLIPF-MPLLIKDMTFT--------------------------
-----HEGNK--TFI-------D---NLVNFE-KMRMIANTA---RTVRYYRSQPFNPDA
AQAN-KN---------------HQDVRSYVRQ-LNVIDNQRTLSQMSHR---LEPR
>Rapgef4_[Ictalurus_punctatus]_1042363418
SSKEVAYQMTLYEWELFNCVHEH----ELI-----YHTFG-RQHF--K-K-----ATA--
-------------------NLDLFLRRFNEIQLWVVTEVCLCSTLSKRVQLLKKFIKIA-

AHCKE-FRNLNSFFAIIMGLSNPAVSRLSQTWEKLPSKFKKVYVEYENLMDPSRNHRAYR
LTVAK--LDP-------PIIPF-MPLLIKDMTFT--------------------------
-----HEGNK--TFV-------D---SLVNFE-KMRMIANTV---RIIRYCRSQPFNHEA
PQAT-KN---------------HQDVRLYVRQ-ISVIDNQRTLSQLSHK---LEPR
>Rapgef4_[Ictidomys_tridecemlineatus]_532077758
SSKDLAYQMTIYDWELFNCVHEL----ELI-----YHTFG-RHNF--K-K-----TTA--
-------------------NLDLFLRRFNEIQFWVVTEICLCSQLSKRVQLLKKFIKIA-
AHCKE-YRNLNSFFAIVMGLSNVAVSRLALTWEKLPSKFKKFYAEFESLMDPSRNHRAYR
LTAAK--LEP-------PLIPF-MPLLIKDMTFT--------------------------
-----HEGNK--TFI-------D---NLVNFE-KMRMIANTA---RTVRYYRSQPFNPDA
AQAN-KN---------------HQDVRSYVRQ-LNVIDNQRTLSQMSHR---LEPR
>Rapgef4_[Jaculus_jaculus]_507553209
SSKDLAYQMTIYDWELFNCVHEL----ELI-----YHTFG-RHNF--K-K-----TTA--
-------------------NLDLFLRRFNEIQFWVVTEICLCSQLSKRVQLLKKFIKIA-
AHCKE-YKNLNSFLAIVMGLSNVAVSRLALTWEKLPSKFKKFYAEFESLMDPSRNHRAYR
LTAAK--LEP-------PLIPF-MPLLIKDMTFT--------------------------
-----HEGNK--TFI-------D---NLVNFE-KMRMIANTA---RTVRYYRSEPFNPDA
AQAN-KN---------------HQDVRSYVRQ-LNVIDNQRTLSQMSHR---LEPR
>Rapgef4_[Kryptolebias_marmoratus]_1041109010
SSKDLAYQMTMFDWELFSCVHEH----ELL-----YHTFG-RQSF--K-R-----TTA--
-------------------NLDLFLRRFNQVQLWVVTEVCLCTQLSKRVQLLKKFIKIA-
AHCRE-FKNLNSFFAIIMGMSNPAVSRLSQTWEKLPTKFKKFYAEFESMMDPSRNHRSYR
LTVTK--LEP-------PIIPF-MPLLLKDMTFT--------------------------
-----HEGNK--TFI-------D---NMVNFE-KMRIIANTI---RQVRHCRSQPFNPDI
CQPN-KN---------------QAEVRGYVRK-LCVIDNQRALTQLSYR---LEPR
>Rapgef4_[Larimichthys_crocea]_808857433
SSKDLAYQMTLFDWELFSCVHEH----ELL-----YHTFG-RQSF--R-R-----TTA--
-------------------NLDLFLRRFNQVQLWVVTEVCLCGQLSKRVQLLKKFIKIA-
AHCRE-FKNLNSFFAIIMGMSNPAVSRLSQTWEKLPTKFKKFYAEFESMMDPSRNHRSYR
LTVTK--LEP-------PIIPF-MPLLLKDMTFT--------------------------
-----HEGNK--TFI-------D---SMVNFE-KM-------------------------

>Rapgef4_[Lepisosteus_oculatus]_973161213
SSKDLAYQMTVYDWELFNCVHEH----ELI-----YHTFG-RQHF--K-K-----TTA--
-------------------NLDLFLRRFNELQLWVVTEICLCSQLSKRVQLLKKFIKIA-
AHCKE-YRNLNSFFAIIMGLSNPAASRLSQTWEKLPSKFKKFYSEFESLMDPSRNHRAYR
LTVAK--LDP-------PIIPF-MPLLMKDMTFT--------------------------
-----HEGNK--TFI-------D---SLVNFE-KMRMIANTV---RIVRYCRSQPFNPDA
SLAN-KN---------------HQEVRSYVRQ-LNVVDNQRTLSQLSHR---LEPR
>Rapgef4_[Leptonychotes_weddellii]_585180434
SSKDLAYQMTIYDWELFNCVHEL----ELI-----YHTFG-RHNF--K-K-----TTA--
-------------------NLDLFLRRFNEIQFWVVTEICLCSQPSKRVQLLKKFIKIA-
AHCKE-YKNLNSFFAIVMGLSNVAVSRLALTWEKLPSKFKKFYAEFESLMDPSRNHRAYR
LTVAK--LEP-------PLIPF-MPLLIKDMTFT--------------------------
-----HEGNK--TFI-------D---NLVNFE-KMRMIANTA---RTVRYCRSQAFNPDA
AQAN-KN---------------HQDVRSYVRQ-LNVIDNQRTLSQMSHR---LEPR
>Rapgef4_[Leptosomus_discolor]_700415867
SSKDLAYQMTIYDWELFNCVHEL----ELV-----YHTFG-RHNF--K-K-----TTA--
-------------------NLDLFLRRFNEIQFWVVTEICLCSQLSKRVQLLKKYIKIA-
AHCKE-YKNLNSFFAIIMGLSNVAVSRLSLTWEKLPSKFKKIYAEFESLMDPSRNHRAYR
LTVAK--LDP-------PIIPF-MPLLIKDMTFT--------------------------
-----HEGNK--TFT-------D---NLVNFE-KMRMIANTV---RTVKFCRSQSFNPDA
ALTN-KN---------------HQDVRSYVRQ-LNVIDNQRTLSQMSHR---LEPR
>Rapgef4_[Limulus_polyphemus]_926607017

SSHDLAHQMSLYDWELFNAIHEY----ELI-----YQVFG-RHQF--R-K-----SMS--
-------------------NLDMFLRRFNVLQFWVVTELCLASSLSRRVQLLRKFIKVA-
AYCRG-YQNLNAFYAIVMGLSNVAVNRMSLTWERLPGKLRKMYADFEALIDPSRNHRRYR
IFVAK--MAP-------PIIPF-MPLLLKDMTFS-------------------------
-----HEGNK--TYL-------E---GLVNFE-KMHMIAQTL---RTLRYCRSQALVLEA
PPMS-KV---------------QQEVREYIRN-IKVIDNQRRLTQLSSS---LEPR
>Rapgef4_[Lingula_anatina]_919011590
GSKDIAYHLTMYDWELLNTMHEY----EMI-----YLIFG-RSKF--Q-R-----ISA--
-------------------NFDRFLQRFNVVQYWVLTEMCLTPNLGKRVQMLKKFIKIA-
EYCLE-YQNLNSFFAIVMGLSNLAVSRLSQTWEKLPSKMKKLFSDFESMMDPSRNHRVYR
LTVAK--MDA-------PFIPF-MPLLMKDLTFT-------------------------
-----HDGNK--SYF-------D---GLVNFE-KMHMVALTM---RSIKFCRSKPLEVEP
PTVG-KN---------------AQDVKNYVRN-IRVIDNQRILTRLSQR---LEPR
>Rapgef4_[Lipotes_vexillifer]_602693655
SSKDLAYQMTVYDWDLFSCVHEL----ELI-----YHTFG-RHHF--K-K-----TTA--
-------------------NLDLFLRRFNEIQFWVVTEICLCSQPSKRVQLLKKFIKIA-
AHCKE-YKNLNSFFAIVMGLSNVAVSRLALTWEKLPSKFKKFYAEFESLMDPSRNHRAYR
LTVAK--LDP-------PLIPF-MPLLIKDMTFT-------------------------
-----HEGNK--TFI-------D---NLVNFE-KMRMIANTA---RTVRYCRSQPFNPDA
AQAN-KN---------------HQDVRSYVRQ-LNVIDNQRTLSQMSHR---LEPR
>Rapgef4_[Loxodonta_africana]_731458749
SSKDLAYQMTIYDWELFNCVHEL----ELI-----YHTFG-RHNF--K-K-----TTA--
-------------------NLDLFLRRFNEIQFWVVTEICLCSQLSKRVQLLKKFIKIA-
AHCKE-YKNLNSFFAIVMGLSNVAVSRLALTWEKLPSKFKKFYAEFESLMDPSRNHRAYR
LTVAK--LEP-------PLIPF-MPLLIKDMTFT-------------------------
-----HEGNK--TFT-------D---NLVNFE-KMRMIANTA---RTVRYCRSQPFNPDA
AQAN-KN---------------HQDVRSYVRQ-LNVIDNQRTLSQMSHR---LEPR
>Rapgef4_[Macaca_fascicularis]_544475839
SSKDLAYQMTIYDWELFNCVHEL----ELI-----YHTFG-RHNF--K-K-----TTA--
-------------------NLDLFLRRFNEIQFWVVTEICLCSQLSKRVQLLKKFIKIA-
AHCKE-YKNLNSFFAIVMGLSNVAVSRLALTWEKLPSKFKKFYAEFESLMDPSRNHRAYR
LTVAK--LEP-------PLIPF-MPLLIKDMTFT-------------------------
-----HEGNK--TFI-------D---NLVNFE-KMRMIANTA---RTVRYYRSQPFNPDA
AQAN-KN---------------HQDVRSYVRQ-LNVIDNQRTLSQMSHR---LEPR
>Rapgef4_[Macaca_nemestrina]_795659619
SSKDLAYQMTIYDWELFNCVHEL----ELI-----YHTFG-RHNF--K-K-----TTA--
-------------------NLDLFLRRFNEIQFWVVTEICLCSQLSKRVQLLKKFIKIA-
AHCKE-YKNLNSFFAIVMGLSNVAVSRLALTWEKLPSKFKKFYAEFESLMDPSRNHRAYR
LTVAK--LEP-------PLIPF-MPLLIKDMTFT-------------------------
-----HEGNK--TFI-------D---NLVNFE-KMRMIANTA---RTVRYYRSQPFNPDA
AQAN-KN---------------HQDVRSYVRQ-LNVIDNQRTLSQMSHR---LEPR
>Rapgef4_[Manacus_vitellinus]_675430506
SSKDLAHQMTIYDWELFNCVHEL----ELI-----YHTFG-RHNF--K-K-----TTA--
-------------------NLDLFLRRFNEIQFWVVTEICLCSQLSKRVQLLKKYIKIA-
AHCKE-YKNLNSFFAIIMGLSNVAVSRLSLTWEKLPSKFKKIYAEFESLMDPSRNHRAYR
LTVAK--LDP-------PIIPF-MPLLIKDMTFT-------------------------
-----HEGNK--TFT-------D---NLVNFE-KMRMIANTV---RTVKFCRSQSFNPDA
ALAN-KN---------------HQDVRSYVRQ-LNVIDNQRTLSQMSHR---LEPR
>Rapgef4_[Mandrillus_leucophaeus]_795195949
SSKDLAYQMTIYDWELFNCVHEL----ELI-----YHTFG-RHNF--K-K-----TTA--
-------------------NLDLFLRRFNEIQFWVVTEICLCSQLSKRVQLLKKFIKIA-
AHCKE-YKNLNSFFAIVMGLSNVAVSRLALTWEKLPSKFKKFYAEFESLMDPSRNHRAYR
LTVAK--LEP-------PLIPF-MPLLIKDMTFT-------------------------
-----HEGNK--TFI-------D---NLVNFE-KMRMIANTA---RTVRYYRSQPFNPDA

AQAN-KN---------------HQDVRSYVRQ-LNVIDNQRTLSQMSHR---LEPR
>Rapgef4_[Marmota_marmota_marmota]_984087885
SSKDLAYQMTIYDWELFNCVHEL----ELI-----YHTFG-RHNF--K-K-----TTA--
-------------------NLDLFLRRFNEIQFWVVTEICLCSQLSKRVQLLKKFIKIA-
AHCKE-YRNLNSFFAIVMGLSNVAVSRLALTWEKLPSKFKKFYAEFESLMDPSRNHRAYR
LTAAK--LEP-------PLIPF-MPLLIKDMTFT-------------------------
-----HEGNK--TFI-------D---NLVNFE-KMRMIANTA---RTVRYYRSQPFNPDA
AQAN-KN---------------HQDVRSYVRQ-LNVIDNQRTLSQMSHR---LEPR
>Rapgef4_[Maylandia_zebra]_835878367
SSKDLAYQMTMFDWELFSCVHEH----ELL-----YHTFG-CQSF--K-R-----TTA--
-------------------NLDLFLRRFNQVQLWVVTEVCLCGQLSKRVQLLKKFIKIA-
AHCRE-FKNLNSFFAIIMGMSNPAVSRLSQTWEKLPTKFKKFYAEFENMMDPSRNHRSYR
LTVTK--LEP-------PIIPF-VPLLLKDMTFT-------------------------
-----HEGNK--TFI-------D---NMVNFE-KMRIIANTI---RQVRHCRSQPFNPDI
CQPN-KN---------------QAEVRAYVRK-LCVIDNQRALTQLSYR---LEPR
>Rapgef4_[Melopsittacus_undulatus]_884829617
SSKDLAHQMTIYDWELFNCVHEL----ELI-----YHTFG-RHNF--K-K-----TTA--
-------------------NLDLFLRRFNEIQFWVVTEICLCAQLSKRVQLLKKYIKIA-
AHCKE-YKNLNSFFAIIMGLSNVAVSRLSLTWEKLPSKFKKIYAEFESLMDPSRNHRAYR
LTVAK--LDP-------PIIPF-MPLLIKDMTFT-------------------------
-----HEGNK--TFT-------D---NLVNFE-KMRMIANTV---RTVKFCRSQSFNPDA
ALTN-KN---------------HQDVRSYVRQ-LNVIDNQRTLSQMSHR---LEPR
>Rapgef4_[Merops_nubicus]_675626707
SSKDLAHQMTIYDWELFNCVHEL----ELI-----YHTFG-RHNF--K-K-----TTA--
-------------------NLDLFLRRFNEIQFWVVTEICLCSQLSKRVQLLKKYIKIA-
AHCKE-YKNLNSFFAIIMGLSNVAVSRLSLTWEKLPSKFKKIYAEFESLMDPSRNHRAYR
LTVAK--LDP-------PIIPF-MPLLIKDMTFT-------------------------
-----HEGNK--TFT-------D---NLVNFE-KMRMIANTV---RTVKFCRSQSFNPDT
ALAN-KN---------------HQDVRSYVRQ-LNVIDNQRTLSQMSHR---LEPR
>Rapgef4_[Mesitornis_unicolor]_704578686
SSKDLAHQMTIYDWELFNCVHEL----ELI-----YHTFG-RHNF--K-K-----TTA--
-------------------NLDLFLRRFNEIQFWVVTEICLCSQLSKRVQLLKKYIKIA-
AHCKE-YKNLNSFFAIIMGLSNVAVSRLSLTWEKLPSKFKKIYAEFESLMDPSRNHRAYR
LTVAK--LDP-------PIIPF-MPLLIKDMTFT-------------------------
-----HEGNK--TFT-------D---NLVNFE-KMRMIANTV---RTVKFCRSQSFNPDA
ALTN-KN---------------HQDVRSYVRQ-LNVIDNQRTLSQMSHR---LEPR
>Rapgef4_[Mesocricetus_auratus]_880859282
SSKDLAYQMTTYDWELFNCVHEL----ELI-----YHTFG-RHNF--K-K-----TTA--
-------------------NLDLFLRRFNEIQFWVVTEICLCSQLSKRVQLLKKFIKIA-
AHCKE-YKNLNSFFAIVMGLSNVAVSRLALTWEKLPSKFKKFYAEFESLMDPSRNHRAYR
LTAAK--LDP-------PLIPF-MPLLIKDMTFT-------------------------
-----HEGNK--TFI-------D---NLVNFE-KMRMIANTA---RTVRYCRSQPFNPDA
AQAN-KN---------------HQDVRSYVRQ-LNVIDNQRTLSQMSHR---LEPR
>Rapgef4_[Metaseiulus_occidentalis]_391334871
SSQDIAYHMTVYEWQLFTTVHEY----ELI-----YQVFG-RNQF--R-R-----IMS--
-------------------NLDVFQRRFNQVQFWVVTEMCLANSLSRRVTLLRKFIKIA-
QHCRE-YQNLNALFAIVMGLGNAAVSRLGQTWERLPSKLRRSFEELENLIEPSRNHKRYR
NAVAK--LQP-------PLIPF-IPLLLKDMTFC-------------------------
-----HVGNK--SYI-------D---GLVNFE-KMHMIGQTL---RHLRHSRSQRLAMEP
PQGSSKS---------------HHNVADYIRN-LKVIDNQRTLTKLSHQ---LEPR
>Rapgef4_[Microcebus_murinus]_829851297
SSKDLAYQMTLYDWELFNCVHEL----ELI-----YHTFG-RHNF--K-K-----TTA--
-------------------NLDLFLRRFNEIQFWVVTEICLCSQLSKRVQLLKKFIKIA-
AHCKE-YKNLNSFFAIVMGLSNVAVSRLALTWEKLPSKFKKFYAEFESLMDPSRNHRAYR

LTVAK--LEP-------PLIPF-MPLLIKDMTFT--------------------------
-----HEGNK--TFI-------D---NLVNFE-KMRMITNTA---RTVRYYRSQPFNPDA
AQAN-KN---------------HQDVRSYVRQ-LNVIDNQRTLSQMSHR---LEPR
>Rapgef4_[Microtus_ochrogaster]_913490049
SSKDLAYQMTTYDWELFNCVHEL----ELI-----YHTFG-RHNF--K-K-----TTA--
-------------------NLDLFLRRFNEIQFWVVTEICLCSQLSKRVQLLKKFIKIA-
AHCKE-YKNLNSFFAIVMGLSNVAVSRLALTWEKLPSKFKKFYAEFESLMDPSRNHRAYR
LTAAK--LDP-------PIIPF-MPLLIKDMTFT--------------------------
-----HEGNK--TFI-------D---NLVNFE-KMRMIANTA---RTVRYYRSQPFNPDA
AQAN-KN---------------HQDVRSYVRQ-LNVIDNQRTLSQMSHR---LEPR
>Rapgef4_[Miniopterus_natalensis]_1016626283
SSKDLAYQMTIYDWELFNCVHEL----ELI-----YHTFG-RHNF--K-K-----TTA--
-------------------NLDLFLRRFNEIQFWVITEICLCSQPSKRVQLLKKFIKIA-
AHCKE-YKNLNSFFAIVMGLSNVAVSRLALTWEKLPSKFKKFYAEFESLMDPSRNHRAYR
LTVAK--LEP-------PLIPF-MPLLIKDMTFT--------------------------
-----HEGNK--TFI-------D---NLVNFE-KMRMIANTA---RMVRYCRSQPFNLDA
AQAN-KN---------------HQDVRSYVRQ-LNVIDNQRTLSQMSHR---LEPR
>Rapgef4_[Monodelphis_domestica]_612019668
SSKDLAYQMTIYDWELFNCVHEL----ELI-----YHTFG-RHNF--K-K-----TTA--
-------------------NLDLFLRRFNEIQFWVVTEICLCAQLSKRVQLLKKFIKIA-
AHCKE-YKNLNSFFAIVMGLSNVAVSRLALTWEKLPSKFKKIYAEFESLMDPSRNHRAYR
LTVTK--LDP-------PLIPF-MPLLIKDMTFT--------------------------
-----HEGNK--TFI-------D---NLVNFE-KMRMIANTV---RTVRYCRSQTFNPDA
ALAN-KN---------------HQDVRGYVRQ-LNVIDNQRTLSQMSHR---LEPR
>Rapgef4_[Mus_musculus]_568917895
SSKDLAYQMTTYDWELFNCVHEL----ELI-----YHTFG-RHNF--K-K-----TTA--
-------------------NLDLFLRRFNEIQFWVVTEVCLCSQLSKRVQLLKKFIKIA-
AHCKE-YKNLNSFFAIVMGLSNVAVSRLALTWEKLPSKFKKFYAEFESLMDPSRNHRAYR
LTAAK--LEP-------PLIPF-MPLLIKDMTFT--------------------------
-----HEGNK--TFI-------D---NLVNFE-KMRMIANTA---RTVRYYRSQPFNPDA
AQAN-KN---------------HQDVRSYVRQ-LNVIDNQRTLSQMSHR---LEPR
>Rapgef4_[Musca_domestica]_557781733
STKELAYHITIFDWDLFWAVHEY----ELL-----YHTFG-RHHF--G-K-----ITA--
-------------------NLDVFLRRFNELQYWIVTEMVSTSSMSKRVGLLRKFIKLA-
AYCKE-YQNLNAFFAIVMGLSNMAVSRLQQTWEKIPSKFRKLYQEFEALIDPSRNHRAYR
VFVGK--LQP-------PLIPF-MPLLLKDMTFA--------------------------
-----HEGNK--TSI-------D---GLVNFE-KMHMMAQTM---RTLRFCRSRSLGL-D
PPSP-KS---------------EGEVRSFISC-LRVIDNQRVLTAMSQK---IEP-
>Rapgef4_[Mustela_putorius_furo]_511839232
SSKDLAYQMTIYDWELFNCVHEL----ELI-----YHTFG-RHNF--K-K-----TTA--
-------------------NLDLFLRRFNEIQFWVVTEICLCSQPSKRVQLLKKFIKIA-
AHCKE-YKNLNSFFAIVMGLSNVAVSRLALTWEKLPSKFKKFYAEFESLMDPSRNHRAYR
LTVAK--LEP-------PLIPF-MPLLIKDMTFT--------------------------
-----HEGNK--TFI-------D---NLVNFE-KMRMIANTA---RTVRYCRSQAFNPDA
AQAS-KN---------------HQDVRSYVRQ-LNVIDNQRTLSQMSHR---LEPR
>Rapgef4_[Myotis_brandtii]_554525590
SSKDLAYQMTIYDWELFNCVHEL----ELI-----YHTFG-RHNF--K-K-----TTA--
-------------------NLDLFLRRFNEIQFWVVTEICLCSQPSKRVQLLKKFIKIA-
AHCKE-YKNLNSFFAIVMGLSNVAVSRLALTWEKLPSKFKKFYAEFESLMDPSRNHRAYR
LTVAK--LEP-------PLIPF-MPLLIKDMTFT--------------------------
-----HEGNK--TFI-------D---NLVNFE-KMRMIANTA---RTVRYCRSQAFNLDA
AQAN-KN---------------HQDVRSYVRQ-LNVIDNQRTLSQMSHR---LEPR
>Rapgef4_[Myotis_davidii]_584081689
SSKDLAYQMTIYDWELFNCVHEL----ELI-----YHTFG-RHNF--K-K-----TTA--

-------------------NLDLFLRRFNEIQFWVVTEICLCSQPSKRVQLLKKFIKIA-
AHCKE-YKNLNSFFAIVMGLSNVAVSRLALTWEKLPSKFKKFYAEFESLMDPSRNHRAYR
LTVAK--LEP-------PLIPF-MPLLIKDMTFT--------------------------
-----HEGNK--TFI-------D---NLVNFE-KMRMIANTA---RTVRYCRSQPFNLDA
AQAN-KN---------------HQDVRSYVRQ-LNVIDNQRTLSQMSHR---LEPR
>Rapgef4_[Myotis_lucifugus]_558101259
SSKDLAYQMTIYDWELFNCVHEL----ELI-----YHTFG-RHNF--K-K-----TTA--
-------------------NLDLFLRRFNEIQFWVVTEICLCSQPSKRVQLLKKFIKIA-
AHCKE-YKNLNSFFAIVMGLSNVAVSRLALTWEKLPSKFKKFYAEFESLMDPSRNHRAYR
LTVAK--LEP-------PLIPF-MPLLIKDMTFT--------------------------
-----HEGNK--TFI-------D---NLVNFE-KMRMIANTA---RTVRYCRSQPFNLDA
AQAN-KN---------------HQDVRSYVRQ-LNVIDNQRTLSQMSHR---LEPR
>Rapgef4_[Neolamprologus_brichardi]_583982938
SSKDLAYQMTMFDWELFSCVHEH----ELL-----YHTFG-CQSF--K-R-----TTA--
-------------------NLDLFLRRFNQVQLWVVTEVCLCGQLSKRVQLLKKFIKIA-
AHCRE-FKNLNSFFAIIMGMSNPAVSRLSQTWEKLPTKFKKFYAEFENMMDPSRNHRSYR
LTVTK--LEP-------PIIPF-VPLLLKDMTFT--------------------------
-----HEGNK--TFI-------D---NMVNFE-KMRIIANTI---RQVRHCRSQPFNPDI
CQPN-KN---------------QAEVRGYVRK-LCVIDNQRALTQLSYR---LEPR
>Rapgef4_[Nestor_notabilis]_701300283
SSKDLAHQMTIYDWELFNCVHEL----ELI-----YHTFG-RHNF--K-K-----TTA--
-------------------NLDLFLRRFNEIQFWVVTEICLCSQLSKRVQLLKKYIKIA-
AHCKE-YKNLNSFFAIIMGLSNVAVSRLSLTWEKLPSKFKKIYAEFESLMDPSRNHRAYR
LTVAK--LDP-------PIIPF-MPLLIKDMTFT--------------------------
-----HEGNK--TFT-------D---NLVNFE-KMRMIANTV---RTVKFCRSQSFNPDA
ALTN-KN---------------HQDVRSYVRQ-LNVIDNQRTLSQMSHR---LEPR
>Rapgef4_[Nomascus_leucogenys]_821032839
SSKDLAYQMTIYDWELFNCVHEL----ELI-----YHTFG-RHNF--K-K-----TTA--
-------------------NLDLFLRRFNEIQFWVVTEICLCSQLSKRVQLLKKFIKIA-
AHCKE-YKNLNSFFAIVMGLSNVAVSRLALTWEKLPSKFKKFYAEFESLMVSDRNHRAYR
LTVAK--LEP-------PLIPF-MPLLIKDMTFT--------------------------
-----HEGNK--TFI-------D---NLVNFE-KMRMIANTA---RTVRYYRSQPFNPDA
AQAN-KN---------------HQDVRSYVRQ-LNVIDNQRTLSQMSHR---LEPR
>Rapgef4_[Nothobranchius_furzeri]_1007745272
SSKDLAYQMTMYDWELFSCVHEH----ELL-----YHTFG-RQSF--K-R-----TTA--
-------------------NLDLFLRRFNQVQLWVVTEVCLCTQLSKRVQLLKKFIKIA-
AHCRE-FKNLNSFFAIIMGMSNPAVSRLSQTWEKLPTKFKKFYAEFESMMDPSRNHWSYR
LTVTK--LEA-------PIIPF-MPLLLKDMTFT--------------------------
-----HEGNK--TFI-------D---NMVNFE-KMRIIANTI---RQVRNCRSQPFNPDI
CQPN-KN---------------QAEVRGYVRK-LCVIDNQRALTQLSYR---LEPR
>Rapgef4_[Notothenia_coriiceps]_736226861
SSKDLAYQMTMFDWELFSCVHEH----ELL-----YHTFG-RQSF--R-R-----TTA--
-------------------NLDLFLRRFNQVQLWVVTEVCLCSQLSKRVQLLKKFIKIA-
AHCRE-FKNLNSFFAIIMGMSNPAVSRLSQTWEKLPTKFKKFYAEFESMMDPSRNHRSYR
LTVTK--LEP-------PIIPF-MPLLLKDMTFT--------------------------
-----HEGNK--TFI-------D---NMVNFE-KMRIIANAI---RQVRHCRSQPFNPDI
CQPN-KN---------------QAEVRGYVRK-LCVIDNQRALTQLSYR---LEPR
>Rapgef4_[Ochotona_princeps]_504128086
SSKDLAYQMTIYDWELFNCVHEL----ELI-----YHTFG-RHHF--K-K-----TTA--
-------------------NLDLFLRRFNEIQFWVVTEICLCSQLSKRVQLLKKFIKIA-
AHCKE-YKNLNSFFAIVMGLSNVAVSRLALTWEKLPSKFKKFYAEFESLMDPSRNHRAYR
LTVAK--LEA-------PLIPF-MPLLIKDMTFT--------------------------
-----HEGNK--TFT-------D---NLVNFE-KMRMIANTA---RTVRYYRSQPFNPDT
AQAN-KN---------------HQDVRSYVRQ-LNVIDNQRTLSQMSHR---LEPR

>Rapgef4_[Octodon_degus]_507658538
SSKDLAYQMTIYDWELFNCVHEL----ELI-----YHTFG-RHNF--K-K-----TTA--
-------------------NLDLFLRRFNEIQFWVVTEICLCSQLSKRVQLLKKFIKIA-
AHCKE-YKNLNSFFAIVMGLSNVAVSRLALTWEKLPSKFKKFYAEFESLMDPSRNHRAYR
LTAAK--LEP-------PLIPF-MPLLIKDMTFT--------------------------
-----HEGNK--TFI-------D---NLVNFE-KMRMIANTA---RTVRYYRSQPFSPDA
AQAN-KN---------------HQDVRSYVRQ-LNVIDNQRTLSQMSHR---LEPR
>Rapgef4_[Octopus_bimaculoides]_961078509
GSQELAYHMTIYDWELFTSVPEY----ELI-----YQVFG-RYKF--A-K-----MTA--
-------------------NLDLFLRRFNEVQYWVVTEILLTQNLNKRVHLLRKFIKLA-
SYCKE-YQNMSSFFAIVLGLSNIAVSRLSQTWEKLPGKLKKMFSEFETIMDPSRNHRVYR
LSISK--MRP-------PIIPF-MPLMMKDMTFT--------------------------
-----HEGNK--TYY-------D---GLVNFE-KMHMIAQTL---RTVRCCRSMPLNLDI
PSSSVKP---------------PFEVKAYVRN-LCVMDNQRTLTQLSHK---LEPR
>Rapgef4_[Odobenus_rosmarus_divergens]_823421495
SSKDLAYQMTIYDWELFNCVHEL----ELI-----YHTFG-RHNF--K-K-----TTA--
-------------------NLDLFLRRFNEIQFWVVTEICLCSQPSKRVQLLKKFIKIA-
AHCKE-YKNLNSFFAIVMGLSNVAVSRLALTWEKLPSKFKKFYAEFESLMDPSRNHRAYR
LTVAK--LEP-------PLIPF-MPLLIKDMTFT--------------------------
-----HEGNK--TFI-------D---NLVNFE-KMRMIANTA---RTVRYCRSQAFNPDA
AQAN-KN---------------HQDVRSYVRQ-LNVIDNQRTLSQMSHR---LEPR
>Rapgef4_[Opisthocomus_hoazin]_700384686
SSKDLAHQMTVYDWELFNCVHEL----ELI-----YHTFG-RHNF--K-K-----TTA--
-------------------NLDLFLRRFNEIQFWVATEICLCSQLSKRVQLLKKYIKIA-
AHCKE-YKNLNSFFAIIMGLSNVAVSRLSLTWEKLPSKFKKIYAEFESSMDPSRNHRAYR
LTVAK--LDP-------PIIPF-MPLLIKDMTFT--------------------------
-----HEGNK--TFT-------D---NLVNFE-KMRMIANTV---RTVKFCRSQSFNPDA
ALTN-KN---------------HQDVRSYVRQ-LNVIDNQRTLSQMSHR---LEPR
>Rapgef4_[Orcinus_orca]_465994873
SSKDLAYQMTVYDWDLFSCVHEL----ELI-----YHTFG-RHHF--K-K-----TTA--
-------------------NLDLFLRRFNEIQFWVVTEICLCSQPSKRVQLLKKFIKIA-
AHCKE-YKNLNSFFAIVMGLSNVAVSRLALTWEKLPSKFKKFYAEFESLMDPSRNHRAYR
LTVAK--LDP-------PLIPF-MPLLIKDMTFT--------------------------
-----HEGNK--TFI-------D---NLVNFE-KMRMIANTA---RTVRYCRSQPFNPDA
AQAN-KN---------------HQDVRSYVRQ-LNVIDNQRTLSQMSHR---LEPR
>Rapgef4_[Oreochromis_niloticus]_542205128
SSKDLAYQMTMFDWELFSCVHEH----ELL-----YHTFG-RQSF--K-R-----TTA--
-------------------NLDLFLRRFNQVQLWVVTEVCLCGQLSKRVQLLKKFIKIA-
AHCRE-FKNLNSFFAIIMGMSNPAVSRLSQTWEKLPTKFKKFYAEFENMMDPSRNHRSYR
LTVTK--LEP-------PIIPF-VPLLLKDMTFT--------------------------
-----HEGNK--TFI-------D---NMVNFE-KMRIIANTI---RQVRHCRSQPFNPDI
CQPN-KN---------------QAEVRGYVRK-LCVIDNQRALTQLSYR---LEPR
>Rapgef4_[Ornithorhynchus_anatinus]_620978658
SSKDLAYQMTIYDWELFNCVHEL----ELI-----YNTFG-RHNF--K-K-----TTA--
-------------------NLDLFLRRFNEIQFWVVTEICLCSQLSKRVQLLKKFTKIA-
AHCKE-YKNLNSFFAIVMGLSNVAVSRLTLTWEKLPSKFKKIYAEFENLMDPSRNHRAYR
LTIAK--LDP-------PLIPF-MPLLIKDMTFT--------------------------
-----HEGNK--TFI-------D---NLVNFE-KMRMIANTA---RTVRYCRSQPFNPDA
ALAN-KN---------------HQDVRSYVRQ-LNVIDNQRTLSQMSHR---LEPR
>Rapgef4_[Orycteropus_afer_afer]_634820951
SSKDLAYQMTIYDWELFHCVHEL----ELI-----YHTFG-RHNF--K-K-----TTA--
-------------------NLDLFLRRFNEIQFWVVTEICLCSQLSKRVQLLKKFIKIA-
AHCKE-YKNLNSFLAIVMGLSNVAVSRLALTWEKLPSKFKKFYAEFESLMDPSRNHRAYR
LTVAK--LDP-------PLIPF-MPLLIKDMTFT--------------------------

-----HEGNK--TFI-------D---NLVNFE-KMRMIANTA---RTVRYCRSQPFNPDA
TQAN-KN---------------HQDVRSYVRQ-LNVIDNQRTLSQMSHR---LEPR
>Rapgef4_[Oryctolagus_cuniculus]_655828001
SSKDLAYQMTIYDWELFNCVHEL----ELI-----YHTFG-RHNF--K-K-----TTA--
-------------------NLDLFLRRFNEIQFWVITEICLCSQLSKRVQLLKKFIKIA-
AHCKE-YRNLNSFFAIVMGLSNVAVSRLALTWEKLPSKFKKFYAEFESLMDPSRNHRAYR
LTVAK--LEA-------PLIPF-MPLLIKDMTFT--------------------------
-----HEGNK--TFT-------D---NLVNFE-KMRMIANTA---RTVRYYRSQPFNLDA
AQAN-KN---------------HQDVRSYVRQ-LNVIDNQRTLSQMSHR---LEPR
>Rapgef4_[Oryzias_latipes]_765148741
SSKDLAYQMTMFDWELFSCVHEH----ELL-----YHTFG-RQSF--R-R-----TTA--
-------------------NLDLFLRRFNQVMLWVVTEVCLCNQLSKRVQLLKKFIKIA-
AHCRE-FKNLNSFFAIIMGMSNPAVSRLSQTWEKLPTKFKKFYAEFESMMDPSRNHRTYR
ITVTK--LEP-------PIIPF-MPLLLKDMTFT--------------------------
-----HEGNK--TFI-------D---NMVNFE-KMRIIANTI---RQVRHCRSQPFNPDI
CQPN-KN---------------QAEVRGYVRK-LCVIDNQRALTQLSYR---LEPR
>Rapgef4_[Otolemur_garnettii]_831228731
SSKDLAYQMTIYDWELFNCVHEL----ELI-----YHTFG-RHNF--K-K-----TTA--
-------------------NLDLFLKRFNEIQFWVVTEICLCSQLSKRVQLLKKFIKIA-
AHCKE-YKNLNSFFAIVMGLSNVAVSRLTLTWEKLPSKFKKFYAEFESLMDPSRNHRAYR
LTVAK--LEP-------PLIPF-MPLLIKDMTFT--------------------------
-----HEGNK--TFI-------D---NLVNFE-KMRMITNTA---RTVRYYRSQPFNPDA
AQAN-KN---------------HQDVRSYVRQ-LNVIDNQRTLSQMSHR---LEPR
>Rapgef4_[Ovis_aries_musimon]_965808735
SSKDLAYQMTIYDWELFNCVHEL----ELI-----YHTFG-RHHF--K-K-----TTA--
-------------------NLDLFLRRFNEIQFWVVTEICLCSQPSKRVQLLKKFIKIA-
AHCKE-YKNLNSFFAIVMGLSNVAVSRLALTWEKLPSKFKKFYAEFESLMDPSRNHRAYR
LTVAK--LDP-------PLIPF-MPLLIKDMTFT--------------------------
-----HEGNK--TFI-------D---NLVNFE-KMRMIANTA---RTVRYCRSQPFNPDA
AQAN-KN---------------HQDVRSYVRQ-LNVIDNQRTLSQMSHR---LEPR
>Rapgef4_[Ovis_aries]_426220871
SSKDLAYQMTIYDWELFNCVHEL----ELI-----YHTFG-RHHF--K-K-----TTA--
-------------------NLDLFLRRFNEIQFWVVTEICLCSQPSKRVQLLKKFIKIA-
AHCKE-YKNLNSFFAIVMGLSNVAVSRLALTWEKLPSKFKKFYAEFESLMDPSRNHRAYR
LTVAK--LDP-------PLIPF-MPLLIKDMTFT--------------------------
-----HEGNK--TFI-------D---NLVNFE-KMRMIANTA---RTVRYCRSQPFNPDA
AQAN-KN---------------HQDVRSYVRQ-LNVIDNQRTLSQMSHR---LEPR
>Rapgef4_[Pan_troglodytes]_1034150401
SSKDLAYQMTIYDWELFNCVHEL----ELI-----YHTFG-RHNF--K-K-----TTA--
-------------------NLDLFLRRFNEIQFWVITEICLCSQLSKRVQLLKKFIKIA-
AHCKE-YKNLNSFFAIVMGLSNVAVSRLALTWEKLPSKFKKFYAEFESLMDPSRNHRAYR
LTVAK--LEP-------PLIPF-MPLLIKDMTFT--------------------------
-----HEGNK--TFI-------D---NLVNFE-KMRMIANTA---RTVRYYRSQPFNPDA
AQAN-KN---------------HQDVRSYVRQ-LNVIDNQRTLSQMSHR---LEPR
>Rapgef4_[Panthera_tigris_altaica]_987393874
SSKDLAYQMTIYDWELFNCVHEL----ELI-----YHTFG-RHNF--K-K-----TTA--
-------------------NLDLFLRRFNEIQFWVVTEICLCSQPSKRVQLLKKFIKIA-
AHCKE-YKNLNSFFAIVMGLSNVAVSRLALTWEKLPSKFKKFYAEFESLMDPSRNHRAYR
LTVAK--LEP-------PLIPF-MPLLIKDMTFT--------------------------
-----HEGNK--TFI-------D---NLVNFE-KMRMIANTA---RTVRYCRSQPFNPDA
AQAN-KN---------------HQDVRSYVRQ-LNVIDNQRTLSQMSHR---LEPR
>Rapgef4_[Pantholops_hodgsonii]_556763448
SSKDLAYQMTIYDWELFNCVHEL----ELI-----YHTFG-RHHF--K-K-----TTA--
-------------------NLDLFLRRFNEIQFWVVTEICLCSQPSKRVQLLKKFIKIA-

AHCKE-YKNLNSFFAIVMGLSNVAVSRLALTWEKLPSKFKKFYAEFESLMDPSRNHRAYR
LTVAK--LDP-------PLIPF-MPLLIKDMTFT--------------------------
-----HEGNK--TFI-------D---NLVNFE-KMRMIANTA---RTVRYCRSQPFNPDA
AQAN-KN---------------HQDVRSYVRQ-LSVIDNQRTLSQMSHR---LEPR
>Rapgef4_[Papio_anubis]_402888644
SSKDLAYQMTIYDWELFNCVHEL----ELI-----YHTFG-RHNF--K-K-----TTA--
-------------------NLDLFLRRFNEIQFWVVTEICLCSQLSKRVQLLKKFIKIA-
AHCKE-YKNLNSFFAIVMGLSNVAVSRLALTWEKLPSKFKKFYAEFESLMDPSRNHRAYR
LTVAK--LEP-------PLIPF-MPLLIKDMTFT--------------------------
-----HEGNK--TFI-------D---NLVNFE-KMRMIANTA---RTVRYYRSQPFNPDA
AQAN-KN---------------HQDVRSYVRQ-LNVIDNQRTLSQMSHR---LEPR
>Rapgef4_[Parasteatoda_tepidariorum]_1009531414
NSLDIAYGMTIYEWDLFSCVHEY----ELI-----YQVFG-RHQF--R-K-----IMS--
-------------------NLDVFLRRFNEIQHWVVTEMVLCNNLSRRVALLRKFIKIA-
AHCRE-HQNMNSFFAIVMGLSNVAVSRMTQTWEKLPSKLKRTFSEFEALIDPSRNHRRYR
LTVSK--MVP-------PIIPF-MPLLLKDMTFS--------------------------
-----HEGNK--TYM-------E---GLLNFE-KMHMIAQSL---RTIRHCRSQPLELDP
PPGN-KP---------------QQDVRNYIRN-LRIIDNQKRLTQLSDV---LEPR
>Rapgef4_[Parus_major]_998705928
SSKDLAHQMTIYDWELFNCVHEL----ELI-----YHTFG-RHNF--K-K-----TTA--
-------------------NLDLFLRRFNEIQFWVVTEICLCSQLSKRVQLLKKYIKIA-
AHCKE-YKNLNSFFAIIMGLSNVAVSRLSLTWEKLPSKFKKIYAEFESLMDPSRNHRAYR
LTVAK--LDP-------PIIPF-MPLLIKDMTFT--------------------------
-----HEGNK--TFT-------D---NLVNFE-KMRMIANTV---RTVKFCRSQSFNPDA
ALTN-KN---------------HQDVRSYVRQ-LNVIDNQRTLSQMSHR---LEPR
>Rapgef4_[Pelecanus_crispus]_694636029
SSKDLAHQMTVYDWELFNCVHEL----ELI-----YHTFG-RHNF--K-K-----TTA--
-------------------NLDLFLRRFNEIQFWVVTEICLCSQLSKRVQLLKKYIKIA-
AHCKE-YKNLNSFFAIIMGLSNVAVSRLSLTWEKLPSKFKKIYAEFESLMDPSRNHRAYR
LTVAK--LDP-------PIIPF-MPLLIKDMTFT--------------------------
-----HEGNK--TFT-------D---NLVNFE-KMRMIANTV---RTVKFCRSQSFNPDA
ALTN-KN---------------HQDVRSYVRQ-LNVIDNQRTLSHMSHR---LEPR
>Rapgef4_[Peromyscus_maniculatus_bairdii]_1008739312
SSKDLAYQMTTYDWELFNCVHEL----ELI-----YHTFG-RHNF--K-K-----TTA--
-------------------NLDLFLRRFNEIQFWVVTEICLCSQLSKRVQLLKKFIKIA-
AHCKE-YKNLNSFFAIVMGLSNVAVSRLALTWEKLPSKFKKFYAEFESLMDPSRNHRAYR
LTAAK--LDP-------PLIPF-MPLLIKDMTFT--------------------------
-----HEGNK--TFI-------D---NLVNFE-KMRMIANTA---RTVRYYRSQPFNPDA
AQAN-KN---------------HQDVRSYVRQ-LNVIDNQRTLSQMSHR---LEPR
>Rapgef4_[Phaethon_lepturus]_723137806
SSKDLAHQMTIYDWELFNCVHEL----ELI-----YHTFG-RHNF--R-K-----TTA--
-------------------NLDLFLRRFNEIQFWVVTEICLCSQLSKRVQLLKKFIKIA-
AHCKE-YKNLNSFFAIIMGLSNVAVSRLLLTWEKLPSKFKKIYAEFESLMDPSRNHRAYR
LTVAK--LDP-------PIIPF-MPLLIKDMTFT--------------------------
-----HEGNK--TFT-------D---NLVNFE-KMRMIANTV---RTVKFCRSQSFNPDA
AITS-KN---------------HQDVRSYVRQ-LNVIDNQRTLSQMSHR---LEPR
>Rapgef4_[Phalacrocorax_carbo]_695135744
SSKDLAHQMTIYDWELFNCVHEL----ELI-----YHTFG-RHNF--K-K-----TTA--
-------------------NLDLFLRRFNEIQFWVVTEICLCSQLSKRVQLLKKYIKIA-
AHCKE-YKNLNSFFAIIMGLSNVAVSRLSLTWEKLPSKFKKIYAEFESLMDPSRNHRAYR
LTVAK--LDP-------PIIPF-MPLLIKDMTFT--------------------------
-----HEGNK--TFT-------D---NLVNFE-KM-------------------------

>Rapgef4_[Physeter_catodon]_593753582

SSKDLAYQMTVYDWDLFSCVHEL----ELI-----YHTFG-RHHF--K-K-----TTA--
-------------------NLDLFLRRFNEIQFWVVTEICLCSQPSKRVQLLKKFIKIA-
AHCKE-YKNLNSFFAIVMGLSNVAVSRLALTWEKLPSKFKKFYAEFESLMDPSRNHRAYR
LTVAK--LDP-------PLIPF-MPLLIKDMTFT-------------------------
-----HEGNK--TFI-------D---NLVNFE-KMRMIANTA---RTVRYCRSQPFNPDA
AQAN-KN---------------HQDVRSYVRQ-LNVIDNQRTLSQMSHR---LEPR
>Rapgef4_[Picoides_pubescens]_699690688
SSKDLAYQMTIYDWELFSCVHEL----ELI-----YHTFG-RHNF--K-K-----TTA--
-------------------NLDLFLRRFNEIQFWVVTEICLCSQLSKRVQLLKKYIKIA-
AHCKE-YKNLNSFFAIIMGLSNVAVSRLLLTWEKLPSKFKKIYAEFESLMDPSRNHRAYR
LTVAK--LDP-------PLIPF-MPLLIKDMTFT-------------------------
-----HEGNK--TFT-------D---KLVNFE-KMRMIANTV---RTVKFCRSQPFNPDA
ALAN-KN---------------HQDVRSYVRQ-LNVIDNQRTLSQMSHR---LEPR
>Rapgef4_[Podiceps_cristatus]_683474669
SSKDLAHQMTIYDWELFNCVHEL----ELI-----YHTFG-RHNF--K-K-----TTA--
-------------------NLDLFLRRFNEIQFWVVTEICLCAQLSKRVQLLKKYIKIA-
AHCKE-YKNLNSFFAIIMGLSNVAVSRLSLTWEKLPSKFKKIYAEFESLMDPSRNHRAYR
LTVAK--LDP-------PIIPF-MPLLIKDMTFT-------------------------
-----HEGNK--TFT-------D---NLVNFE-KMRMIANTV---RTVKFCRSQSFNPDA
ALTN-KN---------------HQDVRSYVRQ-LNVIDNQRTLSQMSHR---LEPR
>Rapgef4_[Poecilia_formosa]_617381053
SSKDVAYHMTFYDWELFHCVHEL----ELI-----YHTFG-RQHV--K-K-----TTV--
-------------------NLDLFLQRFNEIQFWVITEVCLCSQLNKRVQLLKKFIKIA-
AHCKE-YKNLNAFFAVILGLSHPAVSRLSQTWEKLPSKFKKFYGEFENLMDPSRNHRAYR
LTVAK--LAP-------PVIPF-MPLMIKDMTFT-------------------------
-----HEGNK--TFI-------D---NLVNFE-KMRMIAKTV---KTVRYYRSQTFSPDS
PQAG-KH---------------HPDVWTYVRQ-LSVIDNQRTLTQLSHG---LEPR
>Rapgef4_[Poecilia_latipinna]_961931717
SSKDVAYHMTFYDWELFHCVHEL----ELI-----HHTFG-RQHV--K-K-----TTV--
-------------------NLDLFLQRFNEIQFWVITEVCLCSQLNKRVQLLKKFIKIA-
AHCKE-YKNLNAFFAVIMGLSHPAVSRLSQTWEKLPSKFKKFYGEFENLMDPSRNHRAYR
LTVAK--LAP-------PVIPF-MPLMIKDMTFT-------------------------
-----HEGNK--TFI-------D---NLVNFE-KMRMIAKTV---KTVRYYRSQTFSWEA
S---
>Rapgef4_[Poecilia_reticulata]_658898199
SSKDLAYQMTMFDWELFSCVHEH----ELL-----YHTFG-RHSF--R-R-----TTA--
-------------------NLDLFLRRFNQVQLWVVTEVCLCTQLSKRVQLLKKFIKIA-
AHCRE-FKNLNSFFAIIMGMSNPAVSRLSQTWEKLPTKFKKFYAEFESMMDPSRNHRSYR
LTVTK--LEP-------PIIPF-MPLLLKDMTFT-------------------------
-----HEGNK--TFI-------D---NMVNFE-KMRIIANTI---RQVRHCRSQPFNPEI
CQPN-KN---------------QAEVRGYVRK-LCVIDNQRALTQLSYR---LEPR
>Rapgef4_[Pongo_abelii]_207080022
SSKDLAYQMTIYDWELFNCVHEL----ELI-----YHTFG-RHNF--K-K-----TTA--
-------------------NLDLFLRRFNEIQFWVVTEICLCSQLSKRVQLLKKFIKIA-
AHCKE-YKNLNSFFAIAMGLSNVAVSRLALTWEKLPSKFKKFYAEFESLMDPSRNHRAYR
LTVAK--LEP-------PVIPF-MPLLIK------------------------------

>Rapgef4_[Propithecus_coquereli]_826312084
SSKDLAYQMTIYDWELFNCVHEL----ELI-----YHTFG-RHNF--K-K-----TTA--
-------------------NLDLFLRRFNEIQFWVVTEICLCSQLSKRVQLLKKFIKIA-
AHCKE-YKNLNSFFAIVMGLSNVAVSRLALTWEKLPSKFKKFYAEFESLMDPSRNHRAYR
LTVAK--LEP-------PLIPF-MPLLIKDMTFT-------------------------
-----HEGNK--TFI-------D---NLVNFE-KMRMITNTA---RTVRYYRSQPFNPDA

AQAN-KN---------------HQDVRSYVRQ-LNVIDNQRTLSQMSHR---LEPR
>Rapgef4_[Protobothrops_mucrosquamatus]_1002568461
SSKDLAYQMTIFEWELFNCVHEL----ELV-----YHTFG-RHHF--K-K-----TTA--
-------------------NLELFLRRFNEIQFWVVTEICLCSQLSKRVQLLKKFIKIA-
AHCKE-YKNLNSFFALIMGLSNVAVSRLTMTWEKLPSKFKKIYAEFESLMDPSRNHRAYR
LTVAK--LEP-------PIIPF-MPLLIKDMTFT--------------------------
-----YEGNK--TFI-------D---NLVNFE-KMRMISNTI---KTVRFCRSLPFNPDA
SQAN-KN---------------HQDLRGYVRQ-LNVIDNQRTLSQMSHR---LEPR
>Rapgef4_[Pseudopodoces_humilis]_929420255
SSKDLAHQMTIYDWELFNCVHEL----ELI-----YHTFG-RHNF--K-K-----TTA--
-------------------NLDLFLRRFNEIQFWVVTEICLCSQLSKRVQLLKKYIKIA-
AHCKE-YKNLNSFFAIIMGLSNVAVSRLSLTWEKLPSKFKKIYAEFESLMDPSRNHRAYR
LTVAK--LDP-------PIIPF-MPLLIKDMTFT--------------------------
-----HEGNK--TFT-------D---NLVNFE-KMRMIANTV---RTVKFCRSQSFNPDA
ALTN-KN---------------HQDVRSYVRQ-LNVIDNQRTLSQMSHR---LEPR
>Rapgef4_[Pterocles_gutturalis]_704489718
SSKDLAYQMTIYDWELFNCVHEM----ELI-----YHTFG-RHNF--K-K-----TTA--
-------------------NLDLFLRRFNEIQFWVVTEICLCSQLSKRVQLLKKYIKIA-
AHCKE-YKNLNSFFAIIMGLSNVAVSRLSLTWEKLPSKFKKIYAEFESLMDPSRNHRAYR
LTVTK--LDP-------PIIPF-MPLLIKDMTFT--------------------------
-----HEGNK--TFT-------D---NLVNFE-KM--------------------------
----
>Rapgef4_[Pteropus_alecto]_586532746
SSKDLAYQMTIYDWELFNCVHEL----ELI-----YHTFG-RHNF--K-K-----TTA--
-------------------NLDLFLRRFNEIQFWVVTEICLCSQPSKRVQLLKKFIKIA-
AHCKE-YKNLNSFFAIVMGLSNVAVSRLALTWEKLPSKFKKFYAEFESLMDPSRNHRAYR
LTVAK--LEP-------PLIPF-MPLLIKDMTFT--------------------------
-----HEGNK--TFI-------D---NLVNFE-KMRMIANTA---RTVRYCRSQPFNLDA
AQAN-KN---------------HQDVRSYVRQ-LNVIDNQRTLSQMSHR---LEPR
>Rapgef4_[Pundamilia_nyererei]_548336931
SSKDLAYQMTMFDWELFSCVHEH----ELL-----YHTFG-CQSF--K-R-----TTA--
-------------------NLDLFLRRFNQVQLWVVTEVCLCGQLSKRVQLLKKFIKIA-
AHCRE-FKNLNSFFAIIMGMSNPAVSRLSQTWEKLPTKFKKFYAEFENMMDPSRNHRSYR
LTVTK--LEP-------PIIPF-VPLLLKDMTFT--------------------------
-----HEGNK--TFI-------D---NMINFE-KMRIIANTI---RQVRHCRSQPFNPDI
CQPN-KN---------------QAEVRAYVRK-LCVIDNQRALTQLSYR---LEPR
>Rapgef4_[Pygoscelis_adeliae]_690425254
SSKDLAHQMTIYDWELFNCVHEL----ELI-----YHTFG-RHNF--K-K-----TTA--
-------------------NLDLFLRRFNEIQFWVVTEICLCSQLSKRVQLLKKYIKIA-
AHCKE-YKNLNSFFAIIMGLSNVAVSRLSLTWEKLPSKFKKIYAEFESLMDPSRNHRAYR
LTVAK--LDP-------PIIPF-MPLLIKDMTFT--------------------------
-----HEGNK--TFT-------D---NLVNFE-KMRMIANTV---RTVKFCRSQSFNPDA
ALTN-KN---------------HQDVRSYVRQ-LNVIDNQRTLSHMSHR---LELR
>Rapgef4_[Python_bivittatus]_1004668302
SSKDLAYQMTIFEWELFNCVHEL----ELV-----YHTFG-RHNF--K-K-----TTA--
-------------------NLDLFLRRFNEIQFWVVTEICLCSQLSKRVQLLKKFIKIA-
AHCKE-YKNLNSFFALIMGLSNVAVSRLTMTWEKLPSKFKKIYAEFESLMDPSRNHRAYR
LTVAK--LEP-------PIIPF-MPLLIKDMTFT--------------------------
-----HEGNK--TFI-------D---NLVNFE-KMRMISNTI---RTVKFCRSLPFSLDA
SQAN-KN---------------HQDVRSYVRQ-LSVIDNQRTLSQMSHR---LEPR
>Rapgef4_[Rattus_norvegicus]_293346052
SSKDLAYQMTTYDWELFNCVHEL----ELI-----YHTFG-RHNF--K-K-----TTA--
-------------------NLDLFLRRFNEIQFWVVTEICLCSQLSKRVQLLKKFIKIA-
AHCKE-YKNLNSFFAIVMGLSNVAVSRLALTWEKLPSKFKKFYAEFESLMDPSRNHRAYR

LTAAK--LEP-------PLIPF-MPLLIKDMTFT--------------------------
-----HEGNK--TFI-------D---NLVNFE-KMRMIANTA---RTVRYYRSQPFNPDA
AQAN-KN---------------HQDVRSYVRQ-LNVIDNQRTLSQMSHR---LEPR
>Rapgef4_[Rhinopithecus_roxellana]_724894127
SSKDLAYQMTIYDWELFNCVHEL----ELI-----YHTFG-RHNF--K-K-----TTA--
-------------------NLDLFLRRFNEIQFWVVTEICLCSQLSKRVQLLKKFIKIA-
AHCKE-YKNLNSFFAIVMGLSNVAVSRLALTWEKLPSKFKKFYAEFESLMDPSRNHRAYR
LTVAK--LEP-------PLIPF-MPLLIKDMTFT--------------------------
-----HEGNK--TFI-------D---NLVNFE-KMRMIANTA---RTVRYYRSQPFNPDA
AQAN-KN---------------HQDVRSYVRQ-LNVIDNQRTLSQMSHR---LEPR
>Rapgef4_[Rousettus_aegyptiacus]_1012008377
SSKDLAYQMTIYDWELFNCVHEL----ELI-----YHTFG-RHNF--K-K-----TTA--
-------------------NLDLFLRRFNEIQFWVVTEICLCSQPSKRVQLLKKFIKIA-
AHCKE-YKNLNSFFAIVMGLSNVAVSRLALTWEKLPSKFKKFYAEFESLMDPSRNHRAYR
LTVAK--LEP-------PLIPF-MPLLIKDMTFT--------------------------
-----HEGNK--TFI-------D---NLVNFE-KMRMIANTA---RTVRYCRSQPFNLDA
AQAN-KN---------------HQDVRSYVRQ-LNVIDNQRTLSQMSHR---LEPR
>Rapgef4_[Saccoglossus_kowalevskii]_585695958
STKEVAYQMTLYDWELLNCLHEY----ELI-----YHTFG-RHKF--G-K-----ITC--
-------------------NLDMLLRRFNEVQFWVVTEMCLTNTLGKRVQLLRKFIKIA-
AFCKE-YKNMNSFFSIVMGLSNIAVSRLSQTWEKLPSKFKRMFAEFETTLDPSRNHRVYR
LAVAK--MQP-------PIIPF-MPLLMKDMTFT--------------------------
-----HEGNK--TYF-------D---GLVNFE-KMHMIATTI---RTIKYCRSEPFKVE-
PPPAVKN---------------VQEVRAYVRN-LSVIDNQRTLTQLSHK---LEPR
>Rapgef4_[Saimiri_boliviensis_boliviensis]_403258758
SSKDLAYQMTIYDWELFHCVHEL----ELI-----YHTFG-RHNF--K-K-----TTA--
-------------------NLDLFLRRFNEIQFWVVTEICLCSQLSKRVQLLKKFIKIA-
AHCKE-YKNLNSFFAIVMGLSNVAVSRLALTWEKLPSKFKKFYAEFESLMDPSRNHRAYR
LTVAK--LEP-------PLIPF-MPLLIKDMTFT--------------------------
-----HEGNK--TFI-------D---NLVNFE-KMRMIANTA---RTVRYYRSQPFNPDA
AQAN-KN---------------HQDVRSYVRQ-LNVIDNQRTLSQMSHR---LEPR
>Rapgef4_[Salmo_salar]_929285053
SSKDLAYQMTVYDWELFHCVHEL----ELI-----YHTFG-RQNF--K-K-----TTA--
-------------------NMDLFLRRFNEIQLWVITEICLCTQLSKRVQMLKKFIKIA-
AHCKE-YKNLNSFFAIVMGMSNPAVSRLSQTWEKLPSKFKKFYSEFESLMDPSRNHRAYR
LTVAK--LDP-------PIIPF-MPLLIKDMTFT--------------------------
-----HDGNK--TFI-------D---SLVNFE-KMRMIANTV---RIVRYCRSLPFSAEP
SQTS-KN---------------HLDVRSYVRQ-LAVIDNQRTLSQLSHR---LEPR
>Rapgef4_[Sarcophilus_harrisii]_395519780
SSKDLAYQMTIYDWELFNCVHEL----ELI-----YHTFG-RHNF--K-K-----TTA--
-------------------NLDLFLRRFNEIQFWVVTEICLCAQLSKRVQLLKKFIKIA-
AHCKE-YKNLNSFFAIVMGLSNVAVSRLALTWEKLPSKFKKIYAEFESLMDPSRNHRAYR
LTVAK--LDP-------PLIPF-MPLLIKDMTFT--------------------------
-----HEGNK--TFI-------D---NLVNFE-KMRMIANTV---RTVRYCRSQPFNPDA
ALAN-KN---------------HQDIRGYVRQ-LNVIDNQRTLSQMSHR---LEPR
>Rapgef4_[Serinus_canaria]_683906367
SSKDLAHQMTIYDWELFNCVHEL----ELI-----YHTFG-RHNF--K-K-----TTA--
-------------------NLDLFLRRFNEIQFWVVTEICLCSQLSKRVQLLKKYIKIA-
AHCKE-YKNLNSFFAIIMGLSNVAVSRLSLTWEKLPSKFKKIYAEFESLMDPSRNHRAYR
LTVAK--LDP-------PIIPF-MPLLIKDMTFT--------------------------
-----HEGNK--TFT-------D---NLVNFE-KMRMIGNTV---RTVKFCRSQSFNPDA
ALTN-KN---------------HQDVRSYVRQ-LNVIDNQRTLSQMSHR---LEPR
>Rapgef4_[Sinocyclocheilus_anshuiensis]_1025001496
SSKDLAHQMTLYDWELFDCVHEH----ELI-----YHTFG-RQHF--K-K-----TTA--

-------------------NLDLFLRRFNEVQLWVVTEVCLCPTLSKRVQLLKKFIKIS-
AHCKE-FKNLNSFFAIIMGLGNPAVCRLSQTWEKLPSKFKKFYGEFENLMDPSRNHRAYR
LTVAK--LEP-------PIIPF-MPLLIKDMTFT-------------------------
-----HEGNK--TFT-------D---RLVNFE-KMRMIANTV---RIMRYCRSQPFNQEA
PQATGKS---------------HQDVRSYVRH-ISVIDNQRTLSQLSHR---LEPR
>Rapgef4_[Sinocyclocheilus_grahami]_1020489045
SSKDLAHQMTLYDWELFDCVHEH----ELI-----YHTFG-RQHF--K-K-----TTA--
-------------------NLDLFLRRFNEVQLWVVTEVCLCPTLSKRVQLLKKFIKIA-
AHCKE-FKNLNSFFAIIMGLGNPAVCRLSHTWEKLPSKFKKFYVEFENLMDPSRNHRAYR
LTVAK--LEP-------PIIPF-MPLLIKDMTFT-------------------------
-----HEGNK--TFT-------D---RLVNFE-KMRMIANTV---RIMRYCRSQPFNQEA
PQATGKS---------------HQDVRSYMRH-ISVIDNQRTLSQLSHR---LEPR
>Rapgef4_[Sinocyclocheilus_rhinocerous]_1025414289
SSKDLAHQMTLYDWELFDCVHEH----ELI-----YHTFG-RQHF--K-K-----TTA--
-------------------NLDLFLRRFNEVQLWVVTEVCLCLTLSKRVQLLKKFIKIA-
AHCKE-FKNLNSFFAIIMGLGNPAVCRLSHTWEKLPSKFKKFYGEFENLMDPSRNHRAYR
LTVAK--LEP-------PIIPF-MPLLIKDMTFT-------------------------
-----HEGNK--TFT-------D---RLVNFE-KMRMIANTV---RIMRYCRSQPFNQEA
PQATGKS---------------HQDVRSYMRH-ISVIDNQRTLSQLSHR---LEPR
>Rapgef4_[Sorex_araneus]_505771648
SSKDLAYQMTIYDWELFNCVHEL----ELI-----YHTFG-RHNF--K-K-----TTA--
-------------------NLDLFLRRFNEIQFWVVTEICLCSQLSKRVQLLKKFIKIA-
AHCKE-YKNLNSFFAIVMGLSNVAVSRLALTWEKLPSKFKKFFAEFESLMDPSRNHRAYR
LTVAK--LDP-------PLTPF-MPLLIKDMTFT-------------------------
-----HEGNK--TFV-------D---NLVNFE-KMRMIANTA---RTVRYCRSQPFNPDA
AQAN-KN---------------HQDVRSYVRQ-LNVIDNQRTLSQMSHR---LEPR
>Rapgef4_[Stegodyphus_mimosarum]_675363725
NSLDIAYNMTIYEWDLFSCVHEY----ELI-----YQVFG-RHQF--R-K-----IMS--
-------------------NLDVFLRRFNEVQFWVVTEMTLATTLSRRVALLRKFIKIA-
AHCRE-YQNMNSFFAIVMGLSNVAVSRMSQTWEKLPSKLKRTFADFEALIDPSRNHRRYR
LTVAK--MVP-------PIIPF-MPLLLKDMTFS-------------------------
-----HEGNK--TFM-------E---GLLNFE-KMHMIAQTL---RTIRHCRSQPLVFDP
PPGN-KS---------------QQDVRNYIRN-LRIIDNQKRLTQLSDA---LEPR
>Rapgef4_[Strongylocentrotus_purpuratus]_390363651
SSRELAYQMTMYDWQLFLCTHEC----EFI-----YHTFG-RHKY--R-R-----ITA--
-------------------NLDVFLRRFNEVQSWIVTELCLTSHISKRVHLLKKFIKIA-
AHCKE-YQNMNSFYAIIMGLSHMSVSRLAQTWDKLPNKLKRVFSDFEALMDPSRNHRVYR
LALSK--MRP-------PIIPF-MPLLIKDMLFT-------------------------
-----HEGNK--TYF-------E---GLVNFE-KMHLVASIM---RVVKYCRSENFKLDS
PP-AVKN---------------VKEIVSYVRN-LQVIDNTKRLMQLSYT---LEP-
>Rapgef4_[Struthio_camelus_australis]_697502536
SSKDLAYQMTIYDWELFNCVHEL----ELI-----YHTFG-RHNF----K---K-TTA--
-------------------NLDLFLRRFNEIQFWVVTEICLCSQLSKRVQLLKKYIKIA-
AQ--

>Rapgef4_[Sturnus_vulgaris]_959065034
SSKDLAHQMTIYDWELFNCVHEL----ELI-----YHTFG-RHNF--K-K-----TTA--
-------------------NLDLFLRRFNEIQFWVVTEICLCSQLSKRVQLLKKYIKIA-
AHCKE-YKNLNSFFAIIMGLSNVAVSRLSLTWEKLPSKFKKIYAEFESLMDPSRNHRAYR
LTVAK--LDP-------PIIPF-MPLLIKDMTFT-------------------------
-----HEGNK--TFT-------D---NLVNFE-KMRMIANTV---RTVKFCRSQSFNPDA
ALTN-KN---------------HQDVRSYVRQ-LNVIDNQRTLSQMSHR---LEPR

>Rapgef4_[Sus_scrofa]_927210423
SSKDLAYQMTIYDWELFNCVHEL----ELI-----YHTFG-RHHF--K-K-----TTA--
-------------------NLDLFLRRFNEIQFWVVTEICLCSQPSKRVQLLKKFIKIA-
AHCKE-YKNLNSFFAIVMGLSNVAVSRLALTWEKLPSKFKKFYAEFESLMDPSRNHRAYR
LTVAK--LDP-------PLIPF-MPLLIKDMTFT--------------------------
-----HEGNK--TFI-------D---SLVNFE-KMRMIANTA---RTVRYCRSQPFNPDA
AQAN-KN---------------HQDVRSYVRQ-LNVIDNQRTLSQMSHR---LEPR
>Rapgef4_[Takifugu_rubripes]_768951741
SSKDLAYQMTMFDWELFSCMHEH----ELL-----YHTFG-RQNF--R-R-----TTA--
-------------------NLDLFLRRFNQVQLWVVTEVCLCSQLSKRVQLLKKFIKIA-
AHCRE-FKNLNSFFAIIMGMSNPAVSRLSQTWEKLPTKFKKFYAEFENMMDPSRNHRSYR
LTVTK--LEP-------PIIPF-MPLLLKDMTFT--------------------------
-----HEGNK--TFI-------D---TMVNFE-KMRIIANAI---RQVRHCRSQPFNPDI
CQPN-KN---------------QAEVRGYVRK-LCVIDNQRALTQLSYR---LEPR
>Rapgef4_[Tinamus_guttatus]_719773627
SSKDLAYQMTIYDWDLFNCVHEL----ELI-----YHTFG-RHNF--K-K-----TTA--
-------------------NLDLFLRRFNEIQFWVVTEICLCSQLSKRVQLLKKYIKIA-
AHCKE-YKNLNSFFAIIMGLSNVAVSRLSLTWEKLPSKFKKIYAEFESLMDPSRNHRAYR
LTVAK--LDP-------PIIPF-MPLLIKDMTFT--------------------------
-----HEGNK--TLT-------D---NLVNFE-KMRMIANTV---RTVKFCRSQSFNPDA
ALAN-KN---------------HQDVRSYVRQ-LNVIDNQRTLSQMSHR---LEPR
>Rapgef4_[Trichechus_manatus_latirostris]_823389193
SSKDLAYQMTIYDWELFNCVHEL----ELI-----YHTFG-RHNF--K-K-----TTA--
-------------------NLDLFLRRFNEIQFWVVTEICLCSQLSKRVQLLKKFIKIA-
AHCKE-CKNLNSFFAIVMGLSNVAVSRLALTWEKLPSKFKKFYAEFESLMDPSRNHRAYR
LTVAK--LEP-------PLIPF-MPLLIKDMTFT--------------------------
-----HEGNK--TFI-------D---NLVNFE-KMRMIANTA---RTVRYCRSQPFNPDA
AQAN-KN---------------HQDVRSYVRQ-LNVIDNQRTLSQMSHR---LEPR
>Rapgef4_[Trichinella_britovi]_954388358
TSTELAQHLLIYHWQLFINTHEY----ELL-----YHVVG-RNLF--PGK-----VPV--
-------------------NLDLLIRRFNELQFWVITEILLCGSVSKRAHVVKKFIKIA-
LHCKA-NQDLFSFFAITLGLSNVAISRLSQTWEKVNAKFRKLFFEFESLLDPSRNHRAYR
LLVAK--MKP-------PTIPF-IPLLLKDLLFA--------------------------
-----HEGNK--TYF-------D---GMVNFE-KMHMMAQTL---RNHRMYKSQMLQID-
---SSKI---------------PTDAQHLVRN-FRIIDDQRKFLRLSHL---LEPK
>Rapgef4_[Trichinella_nativa]_954534911
TSTELAQHLLIYHWQLFINTHEY----ELL-----YHVVG-RNLF--PGK-----VPV--
-------------------NLDLLIRRFNELQFWVITEILLCGSVSKRAHVVKKFIKIA-
LHCKA-NQDLFSFFAITLGLSNVAISRLSQTWEKVNAKFRKLFFEFESLLDPSRNHRAYR
LLVAK--MKP-------PTIPF-IPLLLKDLLFA--------------------------
-----HEGNK--TYF-------D---GM-------HMMAQTL---RNHRMYKSQMLQID-
---SSKI---------------PTDAQHLVRN-FRIIDDQRKFLRLSHL---LEPK
>Rapgef4_[Trichinella_papuae]_954590902
TSTELAQHLLIYHWQLFINTHEY----ELL-----YHVVS-RNLF--PGK-----VPV--
-------------------NLDLLIRRFNELQFWVITEILLCGSVSKRAHVVKKFIKIA-
LHCKT-NQDLFSFFAITLGLSNVAISRLSQTWEKVNAKFRKLFFEFESLLDPSRNHRAYR
LLVAK--MKP-------PTIPF-IPLLLKDLLFA--------------------------
-----HEGNK--TYF-------D---GMVNFE-KMHMMAQTL---RNHRMYKSQMPQID-
---SSKI---------------PMDAQHLVRN-FRIIDDQRKFLRLSQL---LEPK
>Rapgef4_[Trichinella_patagoniensis]_954341166
TSTELAQHLLIYHWQLFINTHEY----ELL-----YHVVG-RNLF--PGK-----VPV--
-------------------NLDLLIRRFNELQFWVITEILLCGSVSKRAHVVKKFIKIA-
LHCKA-NQDLFSFFAITLGLSNVAISRLSQTWEKVNAKFRKLFFEFESLLDPSRNHRAYR
LLVAK--MKP-------PTIPF-IPLLLKDLLFA--------------------------

-----HEGNK--TYF-------D---GMVNFE-KMHMMAQTL---RNHRMYKSQMLQID-
---SSKI---------------PTDAQHLVRN-FRIIDDQRKFLRLSQL---LEPK
>Rapgef4_[Trichinella_sp._T8]_954617710
TSTELAQHLLIYHWQLFINTHEY----ELL-----YHVVG-RNLF--PGK-----VPV--
-------------------NLDLLIRRFNELQFWVITEILLCGSVSKRAHVVKKFIKIA-
LHCKA-NQDLFSFFAITLGLSNVAISRLSQTWEKVNAKFRKLFFEFESLLDPSRNHRAYR
LLVAK--MKP-------PTIPF-IPLLLKDLLFA--------------------------
-----HEGNK--TYF-------D---GM-------HMMAQTL---RNHRMYKSQMLQID-
---SSKI---------------PTDAQHLVRN-FRIIDDQRKFLRLSQL---LEPK
>Rapgef4_[Trichinella_spiralis]_339242857
TSTELAQHLLIYHWQLFINTHEY----ELL-----YHVVG-RNLF--PGK-----VPV--
-------------------NLDLLIRRFNELQFWVITEILLCGSVSKRAHVVKKFIKIA-
LHCKA-NQDLFSFFAITLGLSNVAISRLSQTWEKVNAKFRKLFFEFESLLDPSRNHRAYR
LLVAK--MKP-------PTIPF-IPLLLKDLLFA--------------------------
-----HEGNK--TYF-------D---GMVNFE-KMHMMAQTL---RNHRMYKSQMLQID-
---SSKI---------------PTDAQHLVRN-FRIIDDQRKFLRLSQL---LEPK
>Rapgef4_[Trichinella_zimbabwensis]_954489457
TSTELAQHLLIYHWQLFINTHEY----ELL-----YHVVG-RNLF--PGK-----VPV--
-------------------NLDLLIRRFNELQFWVITEILLCGSVSKRAHVVKKFIKIA-
LHCKT-NQDLFSFFAITLGLSNVAISRLSQTWEKVNAKFRKLFFEFESLLDPSRNHRAYR
LLVAK--MKP-------PTIPF-IPLLLKDLLFA--------------------------
-----HEGNK--TYF-------D---GM-------HMMAQTL---RNHRMYKSQMPQID-
---SSKI---------------PMDAQHLVRN-FRIIDDQRKFLRLSQL---LEPK
>Rapgef4_[Tupaia_chinensis]_562844419
SSKDLAYQMTIYDWELFNCVHEL----ELI-----YHTFG-RHNF--K-K-----TTA--
-------------------NLDLFLRRFNEIQFWVVTEICLCSQLSKRVQLLKKFIKIA-
AHCKE-YKNLNSFFAIVMGLSNVAVSRLALTWEKLPSKFKKFYAEFESLMDPSRNHRAYR
LTVAK--LEP-------PLIPF-MPLLIKDMTFT--------------------------
-----HEGNK--TFI-------D---NLVNFE-KMRMIANTA---RTVRYYRSQPFNPDA
AQAN-KN---------------HQDVRSYVRQ-LNVIDNQRTLSQMSHR---LEPR
>Rapgef4_[Tyto_alba]_701375865
SSKDLAHQMTIYDWELFNCVHEL----ELI-----YHTFG-RHNF--K-K-----TTA--
-------------------NLDLFLRRFNEIQFWVVTEICLCSQLSKRVQLLKKYIKIA-
AHCKE-YKNLNSFFAIIMGLSNVAVSRLSLTWEKLPSKFKKIYAEFESLMDPSRNHRAYR
LTVAK--LDP-------PIIPF-MPLLIKDMTFT--------------------------
-----HEGNK--TFT-------D---NLVNFE-KMRMIANTV---RTVKFCRSQSFNPDA
ALTN-KN---------------HQDVRSYVRQ-LNVIDNQRTLSQMSHR---LEPR
>Rapgef4_[Ursus_maritimus]_670988645
SSKDLAYQMTIYDWELFNCVHEL----ELI-----YHTFG-RHNF----K---K-TTA--
-------------------NLDLFLRRFNEIQFWVVTEICLCSQPSKRVQLLKKFIKIA-
AHCKE-YKNLNSFFAIVMGLSNVAVSRLALTWERM-------------------------

>Rapgef4_[Vicugna_pacos]_560983560
SSKDLAYQMTTYDWELFNCVHEL----ELI-----YHTFG-RHHF--K-K-----TTA--
-------------------NLDLFLRRFNEIQFWVVTEICLCSQPSKRVQLLKKFIKIA-
AHCKE-YKNLNSFFAIVMGLSNVAVSRLALTWEKLPSKFKKFYAEFESLMDPSRNHRAYR
LTVAK--LEP-------PLIPF-MPLLIKDMTFT--------------------------
-----HEGNK--TFI-------D---NLVNFE-KMRMIANTA---RMVRYCRSQPFNPDA
AQAN-KN---------------HQDVRSYVRQ-LNVIDNQRTLSQMSHR---LEPR
>Rapgef4_[Xenopus_tropicalis]_512866136
SSKDLAYQMTIHDWDLFSCVHEL----ELI-----YHTFG-RQNF--K-K-----TTA--
-------------------NLDLFLRRFNEIQFWVVTEICLCPQLSKRVQLLKKFIKIA-

AHCKE-YKNLNSFFAIVMGLSNVAVSRLSLTWEKLPSKFKKIYAEFENLMDPSRNHRAYR
LTVAK--LDP-------PIIPF-TPLLIKDMTFT--------------------------
-----HEGNK--TFI-------D---NLVNFE-KMRMIANTL---RTIRYCRSVPFNPDA
SLAG-KN---------------HQDVRNYVRQ-FNVIDNQRTLSQMSHR---LEPR
>Rapgef4_[Xiphophorus_maculatus]_941773030
SSKDLAYQMTMFDWELFSCVHEH----ELL-----YHTFG-RHSF--R-R-----TTA--
-------------------NLDLFLRRFNQVQLWVVTEVCLCTQLSKRVQLLKKFIKIA-
AHCRE-FKNLNSFFAIIMGMSNPAVSRLSQTWEKLPTKFKKFYAEFESMMDPSRNHRSYR
LTVTK--LEP-------PIIPF-MPLLLKDMTFT--------------------------
-----HEGNK--TFI-------D---NMVNFE-KMRIIANTI---RQVRHCRSQPFNPDI
CQPN-KN---------------QAEVRGYVRK-LCVIDNQRALTQLSYR---LEPR
>Rapgef4_[Zonotrichia_albicollis]_929451867
SSKDLAHQMTIYDWELFNCVHEL----ELI-----YHTFG-RHNF--K-K-----TTA--
-------------------NLDLFLRRFNEIQFWVVTEICLCSQLSKRVQLLKKYIKIA-
AHCKE-YKNLNSFFAIIMGLSNVAVSRLSLTWEKLPSKFKKIYAEFESLMDPSRNHRAYR
LTVAK--LDP-------PIIPF-MPLLIKDMTFT--------------------------
-----HEGNK--TFT-------D---NLVNFE-KMRMIANTV---RTVKFCRSQSFNPEA
ALTN-KN---------------HQDVRSYVRQ-LNVIDNQRTLSQMSHR---LEPR
>Rapgef4_[Zootermopsis_nevadensis]_646703516
STKEVAYHMTQFDWDLFWSVHEY----ELI-----YHTFG-RHHF--G-Q-----ITA--
-------------------NLDVFLRRFNEIQFWVVTELCLTPSLSKRVQILRKFIKLA-
AYCKE-YQNLNAFFAIVMGLSNVAVSRLSLTWEKLPSKFRKLYTEFESLIDPSRNHRAYR
ISVGK--LQP-------PVVPF-MPLLLKDMTFT--------------------------
-----HEGNK--TCL-------D---GLVNFE-KMHMLAQTM---RTIRYCRSRHLLL-E
PPSP-KS---------------EAEVRSYISC-LRIIDNQRVLTSLSQK---LEPR
>Rapgef5_[Acanthisitta_chloris]_677780878
---DLALELTNFDWSLFNAIHEQ----ELI-----YFTFS-RQGS--A-E-----NTE--
-------------------NLSLLLQRCNEVQLWVATEILLCSQLCKRVQLVKKFIKIA-
AHCKA-QRNLNSFFAIVMGLNTASVSRLSQTWEKIPGKFKKLFTELESLTDPSLNHKAYR
DAFKK--MKS-------PKIPF-MPLLLKDVTFI--------------------------
-----HEGNK--TFL-------D---NLVNFE-KLHMIADTV---RSLRHCRNNQFGNEV
PSKE------------------HHELKPYVHH-LHVIDNQQALFELSHR---IEPR
>Rapgef5_[Acinonyx_jubatus]_961711358
---DLALELMNFDWSLFNSIHEQ----ELI-----YFTFS-RQGS--G-E-----HTV--
-------------------NLSLLLQRCNEVQLWVATEILLCGQLGKRVQLVKKFIKIA-
AHCKA-QRNLNSFFAIVMGLNTASVSRLSQTWEKIPGKFKKLFSELESLTDPSLNHKAYR
DAFKK--MKP-------PKIPF-MPLLLKDVTFI--------------------------
-----HEGNK--TFL-------D---NLVNFE-KLHMIADTV---RTLRHCRTNQFGGDM
SPKE------------------HHELKSYVNH-LYVIDSQQALFELSHR---IEPR
>Rapgef5_[Ailuropoda_melanoleuca]_752425000
---DLALELMNFDWSLFNSIHEQ----ELI-----YFTFS-RQGS--G-E-----HTA--
-------------------NLSLLLQRCNEIQLWVATEILLCAQLGKRVQLVKKFIKIA-
AHCKA-QRNLNSFFAIVMGLNTASVSRLSQTWEKIPGKFKKLFSELESLTDPSLNHKAYR
DAFKK--MKP-------PKIPF-MPLLLKDVTFI--------------------------
-----HEGNK--TFL-------D---NLVNFE-KLHMIADTV---RTLRHCRANQFGGDV
SPKE------------------HQELKAYVTH-LYVIDSQQALFELSHR---IEPR
>Rapgef5_[Alligator_mississippiensis]_564231390
---DLALELTNFDWSLFNSIHEQ----ELI-----YFTFS-RQGS--G-E-----NTV--
-------------------NLSLLLQRCNEVQLWVATEILLCSQLCKRVQLMKKFIKIA-
AHCKA-QRNLNSFFAIVMGLNTASVSRLSQTWEKIPGKFKKLFTELESLTDPSLNHKAYR
DAFKK--MKS-------PKIPF-MPLLLKDVTFI--------------------------
-----HEGNK--TFL-------D---NLVNFE-KLHMIADTV---RSLRHCRNNQFGNEV
PPKE------------------HQELKPYVHH-LRVIDNQQTLFELSHR---IEPR
>Rapgef5_[Amazona_aestiva]_944296329

```
------------------------------------------------------------
------------------------------------------------------------
--------------------------------------------------DPSLNHKAYR
DAFKK--MKS-------PKIPF-MPLLLKDVTFI-------------------------
-----HEGNK--TFL-------D---NLVNFE-KLHMIADTV---RSLRHCRNNQLGNEV
-PS--KE---------------HHELKPYVHH-LHVIDNQQALFELSQR---IEPR
>Rapgef5_[Anas_platyrhynchos]_874476610
---DLALELTNFDWSLFNAIHEQ----ELI-----YFTFS-RQGS--A-E-----NTE--
-------------------NLSLLLQRCNEVQLWVATEILLCSQLCKRVQLVKKFIKIA-
AHCKA-QRNLNSFFAIVMGLNTASVSRLSQTWEKIPGKFKKLFTELESLTDPSLNHKAYR
DAFKK--MKS-------PKIPF-MPLLLKDVTFI-------------------------
-----HEGNK--TFL-------D---NLVNFE-KLHMIADTV---RSLRHCRNNQFGNEV
PSKE------------------HHELKPYVHH-LHVIDNQQALFELSHR---IEPR
>Rapgef5_[Anolis_carolinensis]_327274863
---DLALELTNFDWNLFNLVHEQ----ELI-----YFTFS-RQGS--G-E-----NTV--
-------------------NLSLLLQRCNEVQLWVATEILLCNQLCKRVQLVKKFIKIA-
AHCKA-QRNLNSFFAIVMGLNTASVSRLSQTWERIPGKFKKLFTELESLTDPSLNHKAYR
DTFKK--MKS-------PKIPF-MPLLLKDVTFI-------------------------
-----HEGNK--TFL-------D---NLVNFE-KLHMIADTV---RSLRHCRCNQFGNEV
PAKE------------------HQELKSYVHH-LHIIDNQQMLFELSHR---IEPR
>Rapgef5_[Anser_cygnoides_domesticus]_902874207
---DLALELTNFDWSLFNAIHEQ----ELI-----YFTFS-RQGS--A-E-----NTE--
-------------------NLSLLLQRCNEVQLWVATEILLCSQLCKRVQLVKKFIKIA-
AHCKA-QRNLNSFFAIVMGLNTASVSRLSQTWEKIPGKFKKLFSELESLTDPSLNHKAYR
DAFKK--MKS-------PKIPF-MPLLLKDVTFI-------------------------
-----HEGNK--TFL-------D---NLVNFE-KLHMIADTV---RSLRHCRNNQFGNEV
PSKE------------------HHELKPYVHH-LHVIDNQQALFELSHR---IEPR
>Rapgef5_[Aotus_nancymaae]_817357660
---DLALELMNFDWSLFNSIHEQ----ELI-----YFTFS-RQGS--G-E-----HTA--
-------------------NLSLLLQRCNEVQLWVATEILLCSQLGKRVQLVKKFIKIA-
AHCKA-QRNLNSFFAIVMGLNTASVSRLSQTWEKIPGKFKKLFSELESLTDPSLNHKAYR
DAFKK--MKP-------PKIPF-MPLLLKDVTFI-------------------------
-----HEGNK--TFL-------D---NLVNFE-KLHMIADTV---RTLRHCRTNQFG-DL
SPKE------------------HQELKSYVNH-LYVIDSQQALFELSHR---IEPR
>Rapgef5_[Aptenodytes_forsteri]_686607637
---DLALELTNFDWSLFNAIHEQ----ELI-----YFTFS-RQGS--A-E-----NTE--
-------------------NLSLLLQRCNEVQLWVATEILLCSQLCKRVQLVKKFIKIA-
AHCKA-QRNLNSFFAIVMGLNTASVSRLSQTWEKIPGKFKKLFTELESLTDPSLNHKAYR
DAFKK--MKS-------PKIPF-MPLLLKDVTFI-------------------------
-----HEGNK--TFL-------D---NLVNFE-KLHMIADTV---RSLRHCRNNQLGNEV
PSKE------------------HHELKPYVHH-LHVIDNQQALFELSHR---IEPR
>Rapgef5_[Aquila_chrysaetos_canadensis]_768372916
---DLALELTNFDWSLFNAIHEQ----ELI-----YFTFS-RQGS--A-E-----NTE--
-------------------NLSLLLQRCNEVQLWVATEILLCSQLCKRVQLVKKFIKIA-
AHCKA-QRNLNSFFAIVMGLNTASVSRLSQTWEKIPGKFKKLFTELESLTDPSLNHKAYR
DAFKK--MKS-------PKIPF-MPLLLKDVTFI-------------------------
-----HEGNK--TFL-------D---NLVNFE-KLHMIADTV---RSLRHCRNNQFGNEV
PSKE------------------HHELKPYVHH-LHVIDNQQALFELSHR---IEPR
>Rapgef5_[Balaenoptera_acutorostrata_scammoni]_594672128
---DIALELMNFDWSLFNSIHEQ----ELI-----YFTFS-RQGS--G-E-----HTV--
-------------------NLSLLLQRCNEVQLWVATEILLCSQLGKRVQLVKKFIKIA-
AHCKA-QRNLNSFFAIVMGLNTASVSRLSQTWEKIPGKFKKLFSELESLTDPSLNHKAYR
DAFKK--MKP-------PKIPF-MPLLLKDVTFI-------------------------
-----HEGNK--TFL-------D---NLVNFE-KLHMIADTV---RTLRHCRTNQFGGDI
```

SPKE------------------HPELKSYVHH-LCVIDSQQALFALSHR---IEPR
>Rapgef5_[Bison_bison_bison]_742114515
---DLALELMNFDWSLFSSIHEQ----ELI-----YFTFS-RQGS--G-E-----HTA--
-------------------NLSLLLQRCNEVQLWVATEILLCSPLGKRVQLVKKFIKIA-
AHCKA-QRNLNSFFAIVMGLNTASVSRLSQTWEKIPGKFKKLFSELESLTDPSLNHKAYR
DAFKK--MKP-------PKIPF-MPLLLKDVTFI--------------------------
-----HEGNK--TFL-------D---NLVNFE-KLHMIADTV---RTLRHCRTNQFGGDI
SPKE------------------HPELKSYVHH-LYVIDSQQALFELSHR---IEPR
>Rapgef5_[Bos_mutus]_440901494
---DLALELMNFDWSLFSSIHEQ----ELI-----YFTFS-RQGS--G-E-----HTA--
-------------------NLSLLLQRCNEVQLWVATEILLCSPLGKRVQLVKKFIKIA-
AHCKA-QRNLNSFFAIVMGLNTASVSRLSQTWEKIPGKFKKLFSELESLTDPSLNHKAYR
DAFKK--MKP-------PKIPF-MPLLLKDVTFI--------------------------
-----HEGNK--TFL-------D---NLVNFE-KLHMIADTV---RTLRHCRTNQFGGDI
CPKE------------------HPELKSYVHH-LYVIDSQQALFELSHR---IEPR
>Rapgef5_[Bos_taurus]_741875903
---DLALELMNFDWSLFSSIHEQ----ELI-----YFTFS-RQGS--G-E-----HTA--
-------------------NLSLLLQRCNEVQLWVATEILLCSPLGKRVQLVKKFIKIA-
AHCKA-QRNLNSFFAIVMGLNTASVSRLSQTWEKIPGKFKKLFSELESLTDPSLNHKAYR
DAFKK--MKP-------PKIPF-MPLLLKDVTFI--------------------------
-----HEGNK--TFL-------D---NLVNFE-KLHMIADTV---RTLRHCRTNQFGGDI
SPKE------------------HPELKSYVHH-LYVIDSQQALFELSHR---IEPR
>Rapgef5_[Bubalus_bubalis]_594103843
---DLALELMNFDWSLFNSIHEQ----ELI-----YFTFS-RQGS--G-E-----HTV--
-------------------NLSLLLQRCNEVQLWVATEILLCSPLGKRVQLVKKFIKIA-
AHCKA-QRNLNSFFAIVMGLNTASVSRLSQTWEKIPGKFKKLFSELESLTDPSLNHKAYR
DAFKK--MKP-------PKIPF-MPLLLKDVTFI--------------------------
-----HEGNK--TFL-------D---NLVNFE-KLHMIADTV---RTLRHCRTNQFGGDV
SPKE------------------HPELKSYVHH-LYIIDSQQALFELSHR---IEPR
>Rapgef5_[Buceros_rhinoceros_silvestris]_676709688
----LAVELTNFDWSLFNAIHEQ----ELI-----YFTFS-RQGS--A-E-----NTE--
-------------------NLSLLLQRCNEVQLWVATEILLCSQLCKRVQLVKKFIKIA-
AHCKA-QRNLNSFFAIVMGLNTASVSRLSQTWEKIPGKFKKLFTELESL-----------

>Rapgef5_[Calidris_pugnax]_960957042
---DLALELTNFDWSLFNAIHEQ----ELI-----YFTFS-RQGS--A-E-----NTE--
-------------------NLSLLLQRCNEVQLWVATEILLCSQLCKRVQLVKKFIKIA-
AHCKA-QRNLNSFFAIVMGLNTASVSRLSQTWEKIPGKFKKLFTELESLTDPSLNHKAYR
DAFKK--MKS-------PKIPF-MPLLLKDVTFI--------------------------
-----HEGNK--TFL-------D---NLVNFE-KLHMIADTV---RSLRHCRNNQFGNEV
PSKE------------------HHELKPYVHH-LHVIDNQQALFELSHR---IEPR
>Rapgef5_[Callorhinchus_milii]_632937910
----MANELTSFDWDLFNCICEQ----ELI-----CYIFR-RQDS--GRS------TV--
-------------------NLNLLLQRCNEVQFWVATEILLCSQLSKRVQLVKKFIKIA-
AHCRA-LRNLNSFFAIIMGLNSAAVCRLGQTWEKVPGKFKRLFSELENLTDPSLNHKAYR
DAFKK--MQA-------PKIPF-MPLLLKDVTFI--------------------------
-----HEGNK--TFL-------D---NLVNFQ-KLHMIADTV---RLLRHCRSNQSSTEG
TYKE------------------HQDVKTYIQY-LHIIDNQQMLFQLSHR---LEPR
>Rapgef5_[Calypte_anna]_663262517
---DLALELTNFDWSLFNAIHEQ----ELI-----YFTFS-RQGS--A-E-----NTE--
-------------------NLSLLLQRCNEVQLWVATEILLCSQLCKRVQLVKKFIKIA-
AHCKA-QRNLNSFFAIVMGLNTASVSRLSQTWEKIPGKFKKLFTELESLTDPSLNHKAYR

DAFKK--MKS-------PKIPF-MPLLLKDVTFI-------------------------
-----HEGNK--TFL-------D---NLVNFE-KLHMIADTV---RSLRHCRNNQFGNEV
PSKD------------------HHELKPYVHH-LHVIDNQQALFELSHR---IEPR
>Rapgef5_[Camelus_bactrianus]_743744586
---DLALELMNFDWSLFNSIHEQ----ELI-----YFTFS-RQGG--G-E-----HTM--
-------------------NLSFLLQRCNEVQLWVATEILLCSQLGKRVQLVKKFIKIA-
AHCKA-QRNLNSFFAIVMGLNTASVSRLSQTWEKIPGKFKKLFSELESLTDPSLNHKAYR
DAFKK--MKP-------PKIPF-MPLLLKDVTFI-------------------------
-----HEGNK--TFL-------D---NLVNFE-KLHMIADTV---RTLRHCRTNQFGGDM
SPKE------------------HQELKSYVNH-LYVIDSQQSLFELSHR---IEPR
>Rapgef5_[Camelus_dromedarius]_744578310
---DLALELMNFDWSLFNSIHEQ----ELI-----YFTFS-RQGG--G-E-----HTM--
-------------------NLSFLLQRCNEVQLWVATEILLCSQLGKRVQLVKKFIKIA-
AHCKA-QRNLNSFFAIVMGLNTASVSRLSQTWEKIPGKFKKLFSELESLTDPSLNHKAYR
DAFKK--MKP-------PKIPF-MPLLLKDVTFI-------------------------
-----HEGNK--TFL-------D---NLVNFE-KLHMIADTV---RTLRHCRTNQFGGDM
SPKE------------------HQELKSYVNH-LYVIDSQQSLFELSHR---IEPR
>Rapgef5_[Capra_hircus]_926689784
---DLALELMNFDWSLFNSIHEQ----ELI-----YFTFS-RQGS--G-E-----HTV--
-------------------NLSLLLQRCNEVQLWVATEILLCSPLGKRVQLVKKFIKIA-
AHCKA-QRNLNSFFAIVMGLNTASVSRLSQTWEKIPGKFKKLFSELESLTDPSLNHKAYR
DAFKK--MKP-------PKIPF-MPLLLKDVTFI-------------------------
-----HEGNK--TFL-------D---NLVNFE-KLHMIADTV---RTLRHCRTNQFGGDM
SPKE------------------HPELKSYVHH-LYVIDSQQALFELSHR---IEPR
>Rapgef5_[Caprimulgus_carolinensis]_704331329
---DLALELTNFDWSLFNAIHER----ELI-----YFTFS-RQGS--A-E-----NTE--
-------------------NLSLLLQRCNEVQLWVATEILLCSPLCKRVQLVKKFIKIA-
AHCKA-QRNLNSFFAIVMGLNTASVSRLSQTWEKIPGKFKKLFTELESLTDPSLNHKAYR
DAFKK--MKS-------PKIPF-MPLLLKDVTFI-------------------------
-----HEGNK--TFL-------D---NLVNFE-KLHMIADTV---RSLRHCRNNQFGNEV
PSKE------------------HHELKPYIHH-LHVIDNQQALFELSHR---IEPR
>Rapgef5_[Carlito_syrichta]_640775670
---DLALELMNFDWSLFNSIHEQ----ELI-----YFTFS-RQGS--G-E-----HTA--
-------------------NLSLLLQRCNEVQLWVATEILLCSQLGKRVQLVKKFIKIA-
AHCKA-QRNLNSFFAIVMGLNTASVSRLSQTWEKIPGKFKKLFSELESLTDPSLNHKAYR
DAFKK--MKS-------PKIPF-MPLLLKDVTFI-------------------------
-----HEGNK--TFL-------D---NLVNFE-KLHMIADTV---RTLRHCRTSQFGGDL
SPKE------------------HQELKSYVNH-LYIIDNQQALFELSHR---IEPR
>Rapgef5_[Cebus_capucinus_imitator]_1044353746
---DLALELMNFDWSLFNSIHEQ----ELI-----YFTFS-RQGS--G-E-----HTA--
-------------------NLSLLLQRCNEVQLWVATEILLCSQLGKRVQLVKKFIKIA-
AHCKA-QRNLNSFFAIVMGLNTASVSRLSQTWEKIPGKFKKLFSELESLTDPSLNHKAYR
DAFKK--MKP-------PKIPF-MPLLLKDVTFI-------------------------
-----HEGNK--TFL-------D---NLVNFE-KLHMIADTV---RTLRHCRTNQFG-DL
SPKE------------------HQELKSYVNH-LYVIDSQQALFELSHR---IEPR
>Rapgef5_[Ceratotherium_simum_simum]_478489217
---DLALELMNFDWSLFNSIHEQ----ELI-----YFTFS-RQGS--G-E-----HTV--
-------------------NLSLLLQRCNEVQLWVATEILLCSQLGKRVQLVKKFIKIA-
AHCKA-QRNLNSFFAIVMGLNTASVSRLSQTWEKIPGKFKKLFSELESLTDPSLNHKAYR
DAFKK--MKP-------PKIPF-MPLLLKDVTFI-------------------------
-----HEGNK--TFL-------D---NLVNFE-KLHMIADTV---RTLRHCRTNQFGSDM
SPKE------------------HQELKSYVNH-LYVIDSQQALFELSHR---IEPR
>Rapgef5_[Chaetura_pelagica]_701358577
---DLALELTNFDWSLFNAIHEQ----ELI-----YFTFS-RQGS--A-E-----NTE--

-------------------NLSLLLQRCNEVQLWVATEILLCSQLCKRVQLVKKFIKIA-
AHCKA-QRNLNSFFAIVMGLNTASVSRLSQTWEKIPGKFKKLFTELESLTDPSLNHKAYR
DAFKK--MKS-------PKIPF-MPLLLKDVTFI-------------------------
-----HEGNK--TFL-------D---NLVNFE-KLHMIADTV---RSLRHCRNNQFGNEV
PSKE------------------HHELKPYVHH-LHVIDNQQALFELSHR---IEPR
>Rapgef5_[Charadrius_vociferus]_699639499
---DLALELTNFDWSLFNAIHEQ----ELI-----YFTFS-RQGS--A-E-----NTE--
-------------------NLSLLLQRCNEVQLWVATEILLCSQLCKRVQLVKKFIKIA-
AHCKA-QRNLNSFFAIVMGLNTASVSRLSQTWEKIPGKFKKLFTELESLTDPSLNHKAYR
DAFKK--MKS-------PKIPF-MPLLLKDVTFI-------------------------
-----HEGNK--TFL-------D---NLVNFE-KLHMIADTV---RSLRHCRNNQFGNEV
PSKE------------------HHELKPYVHH-LHVIDNQQALFELSHR---IEPR
>Rapgef5_[Chelonia_mydas]_465962692
---------------------------ELI-----YFTFS-RQGS--G-E-----NTV--
-------------------NLSLLLQRCNEVQLWVATEILLCSQLCKRVQLVKKFIKIA-
AHCKA-QRNLNSFFAIVMGLNTASVSRLSQTWEKIPGKFKKLFTELESLTDPSLNHKAYR
DTFKK--MKS-------PKIPF-MPLLLKDVTFI-------------------------
-----HEGNK--TFL-------D---NLVNFE-KLHMIADTV---RSLRHCRNNQCGNEV
PPKE------------------NQELKPHVYH-LHVIDDQQTLFELSHR---IEPR
>Rapgef5_[Chinchilla_lanigera]_533124335
---DLALELMNFDWSLFNSIHEQ----ELI-----YFTFS-RQGS--G-E-----HTV--
-------------------NLSLLLQRCNEVQLWVATEILLCSQLGKRVQLVKKFIKIA-
AHCKA-QRNLNSFFAIVMGLNTASVSRLSQTWEKIPGKFKKLFSELESLTDPSLNHKAYR
DAFKK--MKP-------PKIPF-MPLLLKDVTFI-------------------------
-----HEGNK--TFL-------D---NLVNFE-KLHMIADTV---RTLRHCRTNQFGSDM
SPKE------------------HHELKSYVTH-LYVIDSQQALFELSHR---LEPR
>Rapgef5_[Chlamydotis_macqueenii]_705662260
---DLALELTNFDWSLFNAIHEQ----ELI-----YFTFS-RQGS--A-E-----NTE--
-------------------NLSLLLQRCNEVQLWVATEVLLCSQLCKRVQLVKKFIKIA-
AHCKA-QRNLNSFFAIVMGLNTASVSRLSQTWEKIPGKFKKLFTELESLTDPSLNHKAYR
DAFKK--MKS-------PKIPF-MPLLLKDVTFI-------------------------
-----HEGNK--TFL-------D---NLVNFE-KLHMIADTV---RSLRHCRNNQFGNEV
PSKE------------------HHELKPYVHH-LHVIDNQQALFELSHR---IEPR
>Rapgef5_[Chrysochloris_asiatica]_586448209
---DLALELMNFDWSLFNSIHEQ----ELI-----YFTFS-RQGN--G-E-----HTV--
-------------------NLSLLLQRCNEVQLWVATEILLCSQLGKRVQLVKKFIKIA-
AHCKA-QKNLNSFFAIVMGLNTASVSRLSQTWEKIPGKFKKLFTELENLTDPSLNHKAYR
DAFKK--MKP-------PKIPF-MPLLLKDVTFI-------------------------
-----HEGNK--TFL-------D---NLVNFE-KLHMIADTV---RTLRHCRTNQFG-DM
SPKE------------------HQELKSYVNH-LYVIDSQQALFELSHR---IEPR
>Rapgef5_[Colius_striatus]_706151331
---DLALELTNFDWSLFNAIHEQ----ELI-----YFTFS-RQGS--A-E-----NTE--
-------------------NLSLLLQRCNEVQLWVATEILLCSQLCKRVQLVKKFIKIA-
AHCKA-QRNLNSFFAIVMGLNTASVSRLSQTWEKIPGKFKKLFTELESLTDPSLNHKAYR
DAFKK--MKS-------PKIPF-MPLLLKDVTFI-------------------------
-----HEGNK--TFL-------D---NLVNFE-KLHMIADTV---RSLRHCRNNQFGNEV
PSKE------------------HHELKPYIHH-LHVIDNQQALFELSHR---IEPR
>Rapgef5_[Colobus_angolensis_palliatus]_795175182
---DLALELMNFDWSLFNSIHEQ----ELI-----YFTFS-RQGS--G-E-----HTA--
-------------------NLSLLLQRCNEVQLWVATEILLCSQLGKRVQLVKKFIKIA-
AHCKA-QRNLNSFFAIVMGLNTASVSRLSQTWEKIPGKFKKLFSELESLTDPSLNHKAYR
DAFKK--MKP-------PKIPF-MPLLLKDVTFI-------------------------
-----HEGNK--TFL-------D---NLVNFE-KLHMIADTV---RTLRHCRTNQFG-DL
CPKE------------------HQELKSYVNH-LYVIDSQQALFELSHR---IEPR

>Rapgef5_[Columba_livia]_543740235
---DLALELTNFDWSLFNAIHEQ----ELI-----YFTFS-RQGS--A-E-----NTE--
-------------------NLSLLLQRCNEVQLWVATEILLCSQLCKRVQLVKKFIKIA-
AHCKA-QRNLNSFFAIVMGLNTASVSRLSQTWEKIPGKFKKLFTELESLTDPSLNHKAYR
DAFKK--MKS-------PKIPF-MPLLLKDVTFI--------------------------
-----HEGNK--TFL-------D---NLVNFE-KLHMIADTV---RSLRHCRNNQFGNEV
PSKE------------------HHELKPYVHH-LHVIDNQQALFELSHR---IEPR
>Rapgef5_[Condylura_cristata]_829998867
---DLALELMNFDWNLFNSIHEQ----ELI-----YFTFS-RQGS--G-E-----HTA--
-------------------NLSLLLQRCNEVQLWVATEILLCSQLGKRVQLVKKFIKIA-
AHCKA-QRNLNSFFAIVMGLNTASVSRLSQTWEKIPGKFKKLFSELESLTDPSLNHKAYR
DAFKK--MKP-------PKIPF-MPLLLKDVTFI--------------------------
-----HEGNK--TFL-------D---NLVNFE-KLHMIADTV---RTLRHCRTNQLGGDM
SSKE------------------HQELKSYVNH-LYVIDNQQALFELSHR---IEPR
>Rapgef5_[Corvus_brachyrhynchos]_669274841
---DLALELTNFDWSLFNAIHEQ----ELI-----YFTFS-RQGS--A-E-----NTE--
-------------------NLSLLLQRCNEVQLWVATEILLCSQLCKRVQLVKKFIKIA-
AHCKA-QRNLNSFFAIVMGLNTASVSRLSQTWEKIPGKFKKLFTELESLTDPSLNHKAYR
DAFKK--MKS-------PKIPF-MPLLLKDVTFI--------------------------
-----HEGNK--TFL-------D---NLVNFE-KLHMIADTV---RSLRHCRNNQFGNEV
PSKE------------------HHELKPYVHH-LHVIDNQQALFELSHR---IEPR
>Rapgef5_[Corvus_cornix_cornix]_727017011
---DLALELTNFDWSLFNAIHEQ----ELI-----YFTFS-RQGS--A-E-----NTE--
-------------------NLSLLLQRCNEVQLWVATEILLCSQLCKRVQLVKKFIKIA-
AHCKA-QRNLNSFFAIVMGLNTASVSRLSQTWEKIPGKFKKLFTELESLTDPSLNHKAYR
DAFKK--MKS-------PKIPF-MPLLLKDVTFI--------------------------
-----HEGNK--TFL-------D---NLVNFE-KLHMIADTV---RSLRHCRNNQFGNEV
PSKE------------------HHELKPYVHH-LHVIDNQQALFELSHR---IEPR
>Rapgef5_[Coturnix_japonica]_1003750304
---DLALELTNFDWSLFNAIHEQ----ELI-----YFTFS-RQGS--A-E-----NTE--
-------------------NLSLLLQRCNEVQLWVATEILLCSQLCKRVQLVKKFIKIA-
AHCKA-QRNLNSFFAIVMGLNTASVSRLSQTWEKIPGKFKKLFTELESLTDPSLNHKAYR
DAFKK--MKS-------PKIPF-MPLLLKDVTFI--------------------------
-----HEGNK--TFL-------D---NLVNFE-KLHMIADTV---RSLRHCRNNQFGNEV
PSKE------------------HHELKPYIHH-LHVIDNQQALFELSHR---IEPR
>Rapgef5_[Cricetulus_griseus]_537162100
---DLALELMSFDWSLFNSIHEQ----ELI-----YFTFS-RQGS--G-E-----HTV--
-------------------NLSLLLQRCNEVQLWVATEILLCSQLGKRVQLVKKFIKIA-
AHCKA-QQNLNSFFAIVMGLNTASVSRLSQTWEKIPGKFKKLFSELESLTDPSLNHKAYR
DAFKK--MKP-------PKIPF-MPLLLKDVTFI--------------------------
-----HEGNK--TFL-------D---NLVNFE-KLHMIADTV---RTLRHCRTNQFGSDM
SPKE------------------QQELKSYVNH-LYVIDSQQALFELSHR---LEPR
>Rapgef5_[Cynoglossus_semilaevis]_1035246651
---DVAVALTNFDWTIFNSIHEQ----QLV-----YFTFS-RHAG--SH------HTV--
-------------------ALELLLQRCNEVQLWVMTEVLLCPTLCRRVQLIKKFIKIA-
AHCKA-QRNLNCFFAVIMGLNAAAVGRLTQTWEKIPGKFKKLFSELETMTDPSLNHKAYR
DSFKK--MKA-------PKIPF-LPLLLKDITFI--------------------------
-----HEGNK--TFH-------D---NLVNFE-KLHMIADTV---RLIRQCQTDPMGNSI
TQKS------------------SPDVRAYVDY-LHIIDNQQTLFELSHR---LEPR
>Rapgef5_[Cyprinodon_variegatus]_974049548
---DVAVALTNFDWTIFTRMHEQ----ELV-----YFTFN-RNVY--SS------HTM--
-------------------QLELLLQRCNEVQLWVMTEVLLCPTPCKRVQLIKKFIKIA-
AHCKA-QRNLNSFFAIILGLNAAAVSRLSQTWEKIPGKFKKLFSELEAVTDPSLNHKVYR
ESFKK--MKS-------PKIPF-LPLLLKDITFI--------------------------

-----HEGNK--TFH-------D---NLVNFE-KLHMIADMA---RFIRDCQQDHLGNGI
AQKS------------------SSEVQAYTDY-LHIIDNQQTLFELSHR---LEPR
>Rapgef5_[Danio_rerio]_326676397
---DVAVALTNFDWNLFNSIHEQ----ELI-----FYTFS-RQAS--SG------HTV--
-------------------ALEFLLQRCNEVQQWVMSEVLLCPSLSKRVQLLKKFIKIA-
AHCKA-QRNLNSSFAIIMGLNTAAVSRLNQTWEKVPGKFKKLFSELELLTDPSMNHKAYR
DAFKK--MKP-------PKIPF-MPLLLKDITFI--------------------------
-----HEGNK--TFH-------D---NLVNFE-KLHMIADTV---RLIRHCQMDQTGNEL
SAVD------------------SAEVRSSVHY-LHIIDNQQTLFELSHR---LEPR
>Rapgef5_[Dasypus_novemcinctus]_488539542
---DLALELMNFDWSLFNSIHEQ----ELI-----YFTFS-RQGS--G-E-----HTV--
-------------------NLSLLLQRCNEVQLWVATEILLCSQLGKRVQLVKKFIKIA-
AHCKA-QRNLNSFFAIVMGLNAASVSRLAQTWEKIPGKFKKLFSELESLTDPSLNHKAYR
DAFKK--MKP-------PKIPF-MPLLLKDVTFI--------------------------
-----HEGNK--TFL-------D---NLVNFE-KLHMIADTV---RTLRHCRTNQFGGDM
SPKE------------------HQELKAYVNH-LYVIDSQQALFELSHR---IEPR
>Rapgef5_[Dipodomys_ordii]_852771935
---DLALELMNFDWSLFNSIHEQ----ELI-----YFTFS-RQGN--G-E-----HTV--
-------------------NLSLLLQRCNEVQLWVATEILLCSQLGKRVQLVKKFIKIA-
AHCKA-QRNLNSFFAIVMGLNTASVSRLSQTWEKIPGKFKKLFSELESLTDPSLNHKAYR
DAFKK--MKP-------PKIPF-MPLLLKDVTFI--------------------------
-----HEGNK--TFL-------D---NLVNFE-KLHMIADTV---RTLRHCRANQFGGDV
SPKE------------------HQELKSYVTH-LYVIDSQQALFELSHR---LEPR
>Rapgef5_[Echinops_telfairi]_850284753
---DLALELMHFDWSLFNAIHEQ----ELI-----YFTFS-RQGS--N-E-----HTA--
-------------------NLSLLLQRCNELQLWVATEILLCSQLGKRVQLVKKLIKIA-
AHCKA-QRNLNSFFAIVMGLNTASVSRLSQTWEKIPGKFKKLFSELESLTDPSLNHKAYR
DAFKK--MKP-------PKIPF-MPLLLKDVTFI--------------------------
-----HEGNK--TFL-------D---NLVNFE-KLHMIADTV---RTLRHCRTNLFDEE-

>Rapgef5_[Egretta_garzetta]_697833102
---DLALELTNFDWSLFNAIHEQ----ELI-----YFTFS-RQGS--A-E-----NTE--
-------------------NLSLLLQRCNEVQLWVATEILLCSQLCKRVQLVKKFIKIA-
AHCKA-QRNLNSFFAIVMGLNTASVSRLSQTWEKIPGKFKKLFTELESLTDPSLNHKAYR
DAFKK--MKS-------PKIPF-MPLLLKDVTFI--------------------------
-----HEGNK--TFL-------D---NLVNFE-KLHMIADTV---RSLRHCRNNQFGNEV
PSKE------------------HHELKPYVHH-LHVIDNQQALFELSHR---IEPR
>Rapgef5_[Elephantulus_edwardii]_585640271
---DLALELMNFDWSLFNAIHEQ----ELI-----YFTFS-RQGS--G-E-----HTV--
-------------------NLSLLLQRCNEVQLWVATEILLCSQLGKRVQLVKKFIKIA-
AHCKA-QRNLNSFFAIVMGLNTASVSRLAQTWEKIPGKFKKLFSELESLTDPSLNHKAYR
DAFKK--MKP-------PKIPF-MPLLLKDVTFI--------------------------
-----HEGNK--TFL-------D---NLVNFE-KLHMIADTV---RTLRHCRTNQFG-DV
SPKE------------------HQELKSYVNH-LYVIDSQQALFELSHR---IEPR
>Rapgef5_[Eptesicus_fuscus]_641693066
---DLALELMNFDWSLFNSIHEQ----ELI-----YFTFS-RQGS--G-E-----HTA--
-------------------NLSLLLQRCNEVQLWVATEILLCSQLGKRVQLVKKFIKIA-
AHCKA-QRNLNSFFAIVMGLNTASVSRLSQTWEKIPGKFKKLFSELESLTDPSLNHKAYR
DAFKK--MKP-------PKIPF-MPLLLKDVTFI--------------------------
-----HEGNK--TFL-------D---NLVNFE-KLHMIADTV---RTLRHCRANQCGGDV
SPKE------------------HHELKSYVTH-LFVIDSQQALFELSHR---IEPR
>Rapgef5_[Equus_asinus]_958806827
---DLALELMNFDWSLFNSIHEQ----ELI-----YFTFS-RQGS--G-E-----HTV--
-------------------NLSLLLQRCNEVQLWVATEILLCSQLGKRVQLVKKFIKIA-

AHCKA-QRNLNSFFAIVMGLNTASVSRLSQTWEKIPGKFKKLFSELESLTDPSLNHKAYR
DAFKK--MKP-------PKIPF-MPLLLKDVTFI--------------------------
-----HEGNK--TFL-------D---NLVNFE-KLHMIADTV---RTLRHCRTNQFGSDV
SPKE------------------HQELKSHVNH-LYVIDSQQALFELSHR---IEPR
>Rapgef5_[Equus_caballus]_194209713
---DLALELMNLDWSLSTSIHEQ----ELI-----YFTFS-RQGS--G-E-----HTV--
-------------------NLSLLLQRCNEVQLWVATEILLCSQLGKRVQLVKKFIKIA-
AHCKA-QRNLNSFFAIVMGLNTASVSRLSQTWEKIPGKFKKLFSELESLTDPSLNHKAYR
DAFKK--MKP-------PKIPF-MPLLLKDVTFI--------------------------
-----HEGNK--TFL-------D---NLVNFE-KLHMIADTV---RTLRHCRTNQFGSDV
SPKE------------------HQELKSHVNH-LYVIDSQQALFELSHR---IEPR
>Rapgef5_[Equus_przewalskii]_664704334
---DLALELMNFDWSLFNSIHEQ----ELI-----YFTFS-RQGS--G-E-----HTV--
-------------------NLSLLLQRCNEVQLWVATEILLCSQLGKRVQLVKKFIKIA-
AHCKA-QRNLNSFFAIVMGLNTASVSRLSQTWEKIPGKFKKLFSELESLTDPSLNHKAYR
DAFKK--MKP-------PKIPF-MPLLLKDVTFI--------------------------
-----HEGNK--TFL-------D---NLVNFE-KLHMIADTV---RTLRHCRTNQFGSDV
SPKE------------------HQELKSHVNH-LYVIDSQQALFELSHR---IEPR
>Rapgef5_[Erinaceus_europaeus]_1016649020
---DLALELMNFDWNLFNSIHEQ----ELI-----YFTFS-RQGN--G-E-----HTV--
-------------------NLSLLLQRCNEVQLWVATEILLCSQLGKRVQLVKKFIKIA-
AHCKA-QRNLNSFFAIVMGLNTASVSRLSQTWEKIPGKFKKLFSELESLTDPSLNHKAYR
DAFKK--MKP-------PKIPF-MPLLLKDVTFI--------------------------
-----HEGNK--TFL-------D---NLVNFE-KLHMIADTV---RTLRHCRTSQFG-DV
SPKE------------------HQELKSYVNN-LYVIDSQQALFELSHR---IEPR
>Rapgef5_[Esox_lucius]_884972983
---DIAVSLTNFDWSIFNSIHEQ----ELV-----YFTFS-RHAS--GG------HTA--
-------------------ALELLLQRCNEVQLWVMTEVLLCSTLSKRVQLIKKFIKIA-
AHCKV-QRNLHSFFAIVMGLNTAAVSRLSQTWEKVPGKFKKLFSELEMLTDPSLNHKAYR
DALKK--MKT-------PKIPF-LPLLLKDITFI--------------------------
-----HEGNK--TFL-------D---NLVNFE-KLHMIADTA---RLIRHCQEDHTGYEM
PQKS------------------GPEVRAYVDY-LHVIDNQQTLFELSHR---LEPR
>Rapgef5_[Falco_cherrug]_929428232
---DLALELTNFDWSLFNAIHEQ----ELI-----YFTFS-RQGS--A-E-----NTE--
-------------------NLSLLLQRCNEVQLWVATEILLCSQLCKRVQLVKKFIKIA-
AHCKA-QRNLNSFFAIVMGLNTASVSRLSQTWEKIPGKFKKLFTELESLTDPSLNHKAYR
DAFKK--MKS-------PKIPF-MPLLLKDVTFI--------------------------
-----HEGNK--TFL-------D---NLVNFE-KLHMIADTV---RSLRHCRNNQFGNEV
PSKE------------------HHELKPYIHH-LHVIDNQQALFELSHR---IEPR
>Rapgef5_[Falco_peregrinus]_529435002
---DLALELTNFDWSLFNAIHEQ----ELI-----YFTFS-RQGS--A-E-----NTE--
-------------------NLSLLLQRCNEVQLWVATEILLCSQLCKRVQLVKKFIKIA-
AHCKA-QRNLNSFFAIVMGLNTASVSRLSQTWEKIPGKFKKLFTELESLTDPSLNHKAYR
DAFKK--MKS-------PKIPF-MPLLLKDVTFI--------------------------
-----HEGNK--TFL-------D---NLVNFE-KLHMIADTV---RSLRHCRNNQFGNEV
PSKE------------------HHELKPYIHH-LHVIDNQQALFELSHR---IEPR
>Rapgef5_[Felis_catus]_755702256
---DLALELMNFDWSLFNSIHEQ----ELI-----YFTFS-RQGS--G-E-----HTV--
-------------------NLSLLLQRCNEVQLWVATEILLCGQLGKRVQLVKKFIKIA-
AHCKA-QRNLNSFFAIVMGLNTASVSRLSQTWEKIPGKFKKLFSELESLTDPSLNHKAYR
DAFKK--MKP-------PKIPF-MPLLLKDVTFI--------------------------
-----HEGNK--TFL-------D---NLVNFE-KLHMIADTV---RTLRHCRTNQFGGDM
SPKE------------------HQELKSYVNH-LYVIDSQQALFELSHR---IEPR
>Rapgef5_[Ficedula_albicollis]_524986500

---DLALELTNFDWSLFNAIHEQ----ELI-----YFTFS-RQGS--A-E-----NTE--
-------------------NLSLLLQRCNEVQLWVATEILLCSQLCKRVQLVKKFIKIA-
AHCKA-QRNLNSFFAIVMGLNTASVSRLSQTWEKIPGKFKKLFTELESLTDPSLNHKAYR
DAFKK--MKS-------PKIPF-MPLLLKDVTFI--------------------------
-----HEGNK--TFL-------D---NLVNFE-KLHMIADTV---RSLRHCRNNQFGNEV
PSKE------------------HHELKPYVHH-LHVIDNQQALFELSHR---IEPR
>Rapgef5_[Fulmarus_glacialis]_697038811
---DLALELTNFDWSLFNAIHEQ----ELI-----YFTFS-RQGS--A-E-----NTE--
-------------------NLSLLLQRCNEVQLWVATEILLCSQLCKRVQLVKKFIKIA-
AHCKA-QRNLNSFFAIVMGLNTASVSRLSQTWEKIPGKFKKLFTELESLTDPSLNHKAYR
DAFKK--MKS-------PKIPF-MPLLLKDVTFI--------------------------
-----HEGNK--TFL-------D---NLVNFE-KLHMIADTV---RSLRHCRNNQF----

>Rapgef5_[Fundulus_heteroclitus]_831522848
---DVAVALTNFDWTIFNSMHEQ----ELV-----YFTFN-RNVC--SS------HTK--
-------------------SLELLLQRCNEVQLWVMTEVLLCPTLCKRVQLIKKFIKIA-
AHCKA-QRNLNSFFSIIMGLNAAAVSRLSQTWEKIPGKFKKHFSELETVTDPSLNHKAYR
DSFKK--MKS-------PKIPF-LPLVLKDITFI--------------------------
-----HEGNK--TFH-------D---NLVNFE-KLHMIADMA---RFIRECQQDPVGNGI
AQKS------------------SSEVRAYIDY-LHVIDNQQTLFELSHR---LEPR
>Rapgef5_[Galeopterus_variegatus]_667318434
---DLALELMNFDWSLFNSIHEQ----ELI-----YFTFS-RQGS--G-E-----HTA--
-------------------NLSLLLQRCNEVQLWVVTEILLCSQLGKRVQLVKKFIKIA-
AHCKA-QRNLNSFFAIVMGLNTASVSRLSQTWEKIPGKFKKLFSELESLTDPSLNHKAYR
DAFKK--MKP-------PKIPF-MPLLLKDVTFI--------------------------
-----HEGNK--TFL-------D---NLVNFE-KLHMIADTV---RTLRHCRTNQFGGDV
SPKE------------------HQELRSYVNH-LYVIDSQQALFELSHR---IEPR
>Rapgef5_[Gallus_gallus]_971381104
---DLALELTTFDWSLFNAIHEQ----ELI-----YFTFS-RQGS--A-E-----NTE--
-------------------NLSLLLQRCNEVQLWVATEILLCSQLCKRVQLVKKFIKIA-
AHCKA-QRNLNSFFAIVMGLNTASVSRLSQTWEKIPGKFKKLFTELESLTDPSLNHKAYR
DAFKK--MKS-------PKIPF-MPLLLKDVTFI--------------------------
-----HEGNK--TFL-------D---NLVNFE-KLHMIADTV---RSLRHCRNNQFGNEV
PSKE------------------HHELKPYIHH-LHVIDNQQALFELSHR---IEPR
>Rapgef5_[Gavia_stellata]_678189231
---DLAVELTNFDWSLFNAIHEQ----ELI-----YFTFS-RQGS--A-E-----NTE--
-------------------NLSLLLQRCNEVQLWVATEILLCSPLCKRVQLVKKFIKIA-
AHCKA-QRNLNSFFAIVMGLNTASVSRLSQTWEKIPGKFKKLFTELESLTDPSLNHKAYR
DAFKK--MKS-------PKIPF-MPLLLKDVTFI--------------------------
-----HEGNK--TFL-------D---NLVNFE-KLHMIADTV---RSLRHCRNNQFGNEV
PSKE------------------HHELKPYVHH-LHVIDNQQALFELSHR---IEPR
>Rapgef5_[Gorilla_gorilla_gorilla]_426355608
---DLALELMNFDWSLFNSIHEQ----ELI-----YFTFS-RQGS--G-E-----HTA--
-------------------NLSLLLQRCNEVQLWVATEILLCSQLGKRVQLVKKFIKIA-
AHCKA-QRNLNSFFAIVMGLNTASVSRLSQTWEKIPGKFKKLFSELESLTDPSLNHKAYR
DAFKK--MKP-------PKIPF-MPLLLKDVTFI--------------------------
-----HEGNK--TFL-------D---NLVNFE-KLHMIADTV---RTLRHCRTNQFG-DL
SPKE------------------HQELKSYVNH-LYVIDSQQALFELSHR---IEPR
>Rapgef5_[Haliaeetus_leucocephalus]_729758116
---DLALELTNFDWSLFNAIHEQ----ELI-----YFTFS-RQGS--A-E-----NTE--
-------------------NLSLLLQRCNEVQLWVATEILLCSQLCKRVQLVKKFIKIA-
AHCKA-QRNLNSFFAIVMGLNTASVSRLSQTWEKIPGKFKKLFTELESLTDPSLNHKAYR
DAFKK--MKS-------PKIPF-MPLLLKDVTFI--------------------------
-----HEGNK--TFL-------D---NLVNFE-KLHMIADTV---RSLRHCRNNQFGNEV

PSKE------------------HHELKPYVHH-LHVIDNQQALFELSHR---IEPR
>Rapgef5_[Heterocephalus_glaber]_351695327
---DLALELMSFDWSLFNSIHEQ----ELI-----YFTFS-RQGS--G-E-----HTV--
-------------------NLSLLLQRCNEVQLWVATEILLCTQLGKRVQLVKKFIKIA-
AHCKA-QRNLNSFFAIVMGLNTASVSRLSQTWEKIPGKFKKLFSELESLTDPSLNHKAYR
DAFKK--MKP-------PKIPF-MPLLLKDVTFI--------------------------
-----HEGNK--TFL-------D---NLVNFE-KLHMIADTV---RTLRHCRTNQFGGDM
SPKE------------------HQELKSYVTH-LYVIDSQQALFELSHR---LEPR
>Rapgef5_[Ictalurus_punctatus]_1042325123
---DVSVALTNLDWSLFNSVHEQ----ELV-----YYTFS-RQAS--SG------HTV--
-------------------ALEQLLQRCNEVQQWVMSEVLLCPSLGKRVQLLKKFIKIA-
AHCKA-QRNLNSSFAIIMGLNTAAVSRLNQTWEKVPGKFKKLFSELELLTDPSLNHKAYR
DAFRK--MKP-------PKIPF-MPLLLKDITFI--------------------------
-----HEGNK--TFH-------D---NLVNFE-KLHMIADTV---RHIRHCQTEQTGSEV
APSD------------------SVDMRSSVHY-LHIIDNQQTLFELSHR---LEPR
>Rapgef5_[Ictidomys_tridecemlineatus]_914912372
---DLALELMNFDWNLFNSIHEQ----ELI-----YFTFS-RQGS--G-E-----HTV--
-------------------NLSLLLQRCNEVQLWVATEILLCSQLCKRVQLVKKFIKIA-
AHCKA-QRNLNSFFAIVMGLNTASVSRLSQTWEKIPGKFKKLFSELESLTDPSLNHKAYR
DAFKK--MKP-------PKIPF-MPLLLKVSSLLINLFLASPGPEFSLRGNPQEERRFKA
GGSEEENGSK--VGE-------A---ASVPEI-QQHMIADTV---RTLRHCRTNQFGGDV
SPKE------------------HQELKSYVNH-LYVIDSQQALFELSHR---LEPR
>Rapgef5_[Lepisosteus_oculatus]_973139205
--KDLSDIMVRAVFLLF--VFKQ----ELI-----HYTFG-QQGG--SRA------AV--
-------------------GLELLLQRCDEVQMWVMTEILLCTQLCKRVQLVKKFVKIA-
AHCRA-QRNLNSFFAIIMGLNTAAVSRLSHTWEKVPGKFRKLFSELELLTDPSLNHKAYR
DAFKK--MKP-------PKIPF-MPLLLKDITFI--------------------------
-----HEGNK--TFL-------D---NLVNFE-KLHMIADTV---RLVRHCQTDQLGSEG
GQKD------------------SQEVRSYVHH-LHIIDNQQALFELSHR---LEPR
>Rapgef5_[Leptonychotes_weddellii]_585182469
---DLALELMNFDWSLFNSIHEQ----ELI-----YFTFS-RQGS--G-E-----HTA--
-------------------NLSLLLQRCNEVQLWVATEILLCSQLGKRVQLVKKFIKIA-
AHCKA-QRNLNSFFAIVMGLNTASVSRLSQTWEKIPGKFKKLFSELESLTDPSLNHKAYR
DAFKK--MKP-------PKIPF-MPLLLKDVTFI--------------------------
-----HEGNK--TFL-------D---NLVNFE-KLHMIADTV---RTLRHCRTNQFG-DV
SPKE------------------HQELKSYVNH-LYVIDSQQALFELSHR---IEPR
>Rapgef5_[Leptosomus_discolor]_700421164
---DLALELTNFDWSLFNAIHEQ----ELI-----YFTFS-RQGS--A-E-----NTE--
-------------------NLSLLLQRCNEVQLWVATEILLCSQLCKRVQLVKKFIKIA-
AHCKA-QRNLNSFFAIVMGLNTASVSRLSQTWEKIPGKFKKLFTELESLTDPSLNHKAYR
DAFKK--MKS-------PKIPF-MPLLLKDVTFI--------------------------
-----HEGNK--TFL-------D---NLVNFE-KLHMIADTV---RSLRHCRNNQFGNEV
PSKE------------------HHELKPYIHH-LHVIDNQQALFELSHR---IEPR
>Rapgef5_[Loxodonta_africana]_731461513
---DLALELMNFDWSLFNSIHEQ----ELI-----YFTFS-RQGS--G-E-----HTV--
-------------------NLSLLLQRCNEVQLWVATEILLCSQLGKRVQLVKKFIKIA-
AHCKA-QRNLNSFFAIVMGLNTASVSRLSQTWEKIPGKFKKLFSELESLTDPSLNHKAYR
DAFKK--MKP-------PKIPF-MPLLLKDVTFI--------------------------
-----HEGNK--TFL-------D---NLVNFE-KLHMIADTV---RTLRHCRTNQFGGDV
SPKE------------------HQELKSYVNH-LYVIDSQQALFELSHR---IEPR
>Rapgef5_[Macaca_mulatta]_302564833
---DLALELMNFDWSLFNSIHEQ----ELI-----YFTFS-RQGS--G-E-----HTA--
-------------------NLSLLLQRCNEVQLWVATEILLCSQLGKRVQLVKKFIKIA-
AHCKA-QRNLNSFFAIVMGLNTASVSRLSQTWEKIPGKFKKLFSELESLTDPSLNHKAYR

DAFKK--MKP-------PKIPF-MPLLLKDVTFI-------------------------
-----HEGNK--TFL-------D---NLVNFE-KLHMIADTV---RTLRHCRTNQFG-DL
CPKE------------------HQELKSYVNH-LYVIDSQQALFELSHR---IEPR
>Rapgef5_[Manacus_vitellinus]_679187018
---DLALELTNFDWSLFNAIHEQ----ELI-----YFTFS-RQGN--A-E-----NTE--
-------------------NLSLLLQRCNEVQLWVATEILLCNQLCKRVQLVKKFIKIA-
AHCKA-QRNLNSFFAIVMGLNTASVSRLSQTWEKIPGKFKKLFTELESLTDPSLNHKAYR
DAFKK--MKS-------PKIPF-MPLLLKDVTFI-------------------------
-----HEGNK--TFL-------D---NLVNFE-KLHMIADTV---RSLRHCRNNQFGNEV
PSKE------------------HHELKPYVHH-LHVIDNQQALFELSHR---IEPR
>Rapgef5_[Mandrillus_leucophaeus]_795101220
---DLALELMNFDWSLFNSIHEQ----ELI-----YFTFS-RQGS--G-E-----HTA--
-------------------NLSLLLQRCNEVQLWVATEILLCSQLGKRVQLVKKFIKIA-
AHCKA-QRNLNSFFAIVMGLNTASVSRLSQTWEKIPGKFKKLFSELESLTDPSLNHKAYR
DAFKK--MKP-------PKIPF-MPLLLKDVTFI-------------------------
-----HEGNK--TFL-------D---NLVNFE-KLHMIADTV---RTLRHCRTNQFG-DL
CPKE------------------HQELKSYVNH-LYVIDSQQALFELSHR---IEPR
>Rapgef5_[Marmota_marmota_marmota]_984123699
---DLALELMNFDWSLFNSIHEQ----ELI-----YFTFS-RQGS--G-E-----HTV--
-------------------NLSLLLQRCNEVQLWVATEILLCSQLCKRVQLVKKFIKIA-
AHCKA-QRNLNSFFAIVMGLNTASVSRLSQTWEKIPGKFKKLFSELESLTDPSLNHKAYR
DAFKK--MKP-------PKIPF-MPLLLKDVTFI-------------------------
-----HEGNK--TFL-------D---NLVNFE-KLHMIADTV---RTLRHCRTNQFGGDV
SPKE------------------HQELKSYVNH-LYVIDSQQALFELSHR---LEPR
>Rapgef5_[Maylandia_zebra]_939298992
---DVAVALTDFDWAIFDAVHEQ----ELV-----YFTFN-RHAG--SN------HTT--
-------------------ALEFLLQRCNDIQLWVMTEVLLCPTLCKRVQLIKKFIKIA-
AHCKA-QRNLNCFFAIIMGLNTAAVSRLSQTWEKVPGKFKRLFSELETVTDPSLNHKAYR
DSFKK--MKA-------PKIPF-LPLLLKDITFI-------------------------
-----HEGNK--TFH-------D---NLVNFE-KLHMIADMV---RLIRQCQKDHMGNGI
TQKS------------------SSEVRAYIDY-LHIIDNQQTLFELSQR---LEPR
>Rapgef5_[Meleagris_gallopavo]_733892768
---DLALELTNFDWSLFNAIHEQ----ELI-----YFTFS-RQGS--A-E-----NTE--
-------------------NLSLLLQRCNEVQLWVATEILLCSQLCKRVQLVKKFIKIA-
AHCKA-QRNLNSFFAIVMGLNTASVSRLSQTWEKIPGKFKKLFTELESLTDPSLNHKAYR
DAFKK--MKS-------PKIPF-MPLLLKDVTFI-------------------------
-----HEGNK--TFL-------D---NLVNFE-KLHMIADTV---RSLRHCRNNQFGNEV
PSKE------------------HHELKPYIHH-LHVIDNQQALFELSHR---IEPR
>Rapgef5_[Melopsittacus_undulatus]_527270294
---DLALELTNFDWSLFNAIHEQ----ELI-----CFTFG-RQGS--T-E-----NTE--
-------------------NLSLLLQRCNEVQLWVATEILLCSQLCKRVQLVKKFIKIA-
AHCKA-QRNLNSFFAIVMGLNTASVSRLSQTWEKIPGKFKKLFTELESLTDPSLNHKAYR
DAFKK--MKS-------PKIPF-MPLLLKDVTFI-------------------------
-----HEGNK--TFL-------D---NLVNFE-KLHMIADTV---RSLRHCRNNQLGNEV
PSKE------------------HHELKPYVHY-LHVIDNQQALFELSHR---IEPR
>Rapgef5_[Merops_nubicus]_675634486
---DLALELTNFDWSLFNAIHEQ----ELI-----YFTFS-RQGS--A-E-----NTE--
-------------------NLSLLLQRCNEVQLWVATEILLCSQLCKRVQLVKKFIKIA-
AHCKA-QRNLNSFFAIVMGLNTASVSRLSQTWEKIPGKFKKLFTELESLTDPSLNHKAYR
DAFKK--MKS-------PKIPF-MPLLLKDVTFI-------------------------
-----HEGNK--TFL-------D---NLVNFE-KLHMIADTV---RSLRHCRNNQF----

>Rapgef5_[Mesocricetus_auratus]_880957321

-------MKP-------PKIPF-MPLLLKDVTFI--------------------------
-----HEGNK--TFL-------D---NLVNFE-KLHMIADTV---RTLRHCRTNQFGSDM
-SP--KE---------------QQELRSYVSH-LYVIDSQQALFELSHR---LEPR
>Rapgef5_[Microcebus_murinus]_829808826
---DLALELMNFDWSLFNSIHEQ----ELI-----YFTFS-RQGS--G-E-----HTA--
-------------------NLSLLLQRCNEVQLWVATEILLCSQLGKRVQLVKKFIKIA-
AHCKA-QRNLNSFFAIVMGLNTASVSRLSQTWEKIPGKFKKLFSELESLTDPSLNHKAYR
DAFKK--MKP-------PKIPF-MPLLLKDVTFI--------------------------
-----HEGNK--TFL-------D---NLVNFE-KLHMIADTV---RTLRHCRTNQFGGDL
SPKE------------------HQELKSYVNH-LYVIESQQALFELSHR---IEPR
>Rapgef5_[Microtus_ochrogaster]_531998388
---DLALELMSFDWSLFNSIHEQ----ELI-----YFTFS-RQGS--G-E-----HTV--
-------------------NLSLLLQRCNEVQLWVATEILLCSQLGKRVQLLKKFIKIA-
AHCKA-QQNLNSFFAIVMGLNTASVSRLSQTWEKIPGKFKKLFSELESLTDPSLNHKAYR
DAFKK--MKP-------PKIPF-MPLLLKDVTFI--------------------------
-----HEGNK--TFL-------D---NLVNFE-KLHMIADTV---RTLRHCRSNQFGGDM
SPKE------------------QHELKSYVNH-LYVIDSQQALFELSHR---LEPR
>Rapgef5_[Monodelphis_domestica]_1023030370
---DLAMELMNFDWSLFNSIHEQ----ELI-----YFTFS-RPGS--S-E-----NTV--
-------------------NLSLLLQRCNEVQLWVATEILLCSQLCKRVQLVKKFIKIA-
AHCKA-QRNLNSFFAIVMGLNTASVSRLSQTWEKIPGKFKKLFSELESLTDPSLNHKAYR
DAFKK--MKP-------PKIPF-MPLLLKDVTFI--------------------------
-----HEGNK--TFL-------D---NLVNFE-KLHMIADTV---RSLRHCRNSHFGGDV
SPKE------------------HQELRSYVHH-LHVIDNQQVLFELSHR---IEPR
>Rapgef5_[Mus_musculus]_238637293
---DLALELMSFDWSLFNSIHEQ----ELI-----YFTFS-RQGN--G-E-----NTV--
-------------------NLSLLLQRCNEVQLWVATEILLCSQLGKRVQLVKKFIKIA-
AHCKA-QQNLNSFFAIVMGLNTASVSRLSQTWEKIPGKFKKLFSELESLTDPSLNHKAYR
DAFKK--MKP-------PKIPF-MPLLLKDVTFI--------------------------
-----HEGNK--TFL-------D---NLVNFE-KLHMIADTV---RTLRHCRTNQFGSDV
SPKE------------------QQELKSYVNH-LYVIDSQQALFELSHR---LEPR
>Rapgef5_[Myotis_brandtii]_946777341
---DLALELMNFDWSLFNSIHEQ----ELI-----YFTFS-RQGS--G-E-----HTA--
-------------------NLSLLLQRCNEVQLWVATEILLCSQLGKRVQLVKKFIKIA-
AHCKA-QRNLNSFFAIVMGLNTASVSRLSQTWEKIPGKFKKLFSELESLTDPSLNHKAYR
DAFKK--MKP-------PKIPF-MPLLLKDVTFI--------------------------
-----HEGNK--TFL-------D---NLVNFE-KLHMIADTV---RTLRHCRANQFGGDM
SPKE------------------HQELKSYVTH-LYVIDSQQALFELSHR---IEPR
>Rapgef5_[Myotis_davidii]_987976186
---DLALELMNFDWSLFNSIHEQ----ELI-----YFTFS-RQGS--G-E-----HTA--
-------------------NLSLLLQRCNEVQLWVATEILLCSQLGKRVQLVKKFIKIA-
AHCKA-QRNLNSFFAIVMGLNTASVSRLSQTWEKIPGKFKKLFSELESLTDPSLNHKAYR
DAFKK--MKP-------PKIPF-MPLLLKDVTFI--------------------------
-----HEGNK--TFL-------D---NLVNFE-KLHMIADTV---RTLRHCRANQFGGDM
SPKE------------------HQELKSYVTH-LYVIDSQQALFELSHR---IEPR
>Rapgef5_[Myotis_lucifugus]_940733857
---------------------------ELI-----YFTFS-RQGS--G-E-----HTA--
-------------------NLSLLLQRCNEVQLWVATEILLCSQLGKRVQLVKKFIKIA-
AHCKA-QRNLNSFFAIVMGLNTASVSRLSQTWEKIPGKFKKLFSELESLTDPSLNHKAYR
DAFKK--MKP-------PKIPF-MPLLLKDVTFI--------------------------
-----HEGNK--TFL-------D---NLVNFE-KLHMIADTV---RTLRHCRANQFGGDM
SPKE------------------HQELKSYVTH-LYVIDSQQALFELSHR---IEPR

>Rapgef5_[Neolamprologus_brichardi]_584013381
---DVAVALTDFDWAIFDAVHEQ----ELV-----YFTFN-RHAG--SN------HTT--
-------------------ALEFLLQRCNDIQLWVMTEVLLCPTLCKRVQLIKKFIKIA-
AHCKA-QRNLNCFFAIIMGLNTAAVSRLSQTWEKVPGKFKRLFSELETVTDPSLNHKAYR
DSFKK--MKA-------PKIPF-LPLLLKDITFI--------------------------
-----HEGNK--TFH-------D---NLVNFE-KLHMIADMV---RLIRQCQKDHMGNGI
TQKS------------------SSEVRAYIDY-LHIIDNQQTLFELSQR---LEPR
>Rapgef5_[Nestor_notabilis]_701296279
---DLALELTNFDWSLFNAIHEQ----ELI-----YFTFS-RQGS--A-E-----NTE--
-------------------NLSLLLQRCNEVQLWVATEILLCSQLFKRVQLVKKFIKIA-
AHCKA-QRNLNSFFAIVMGLNTASVSRLSQTWEKIPGKFKKLFTELESLTDPSLNHKAYR
DAFKK--MKS-------PKIPF-MPLLLK-------------------------------

>Rapgef5_[Nomascus_leucogenys]_332207210
---DLALELMNFDWSLFNSIHEQ----ELI-----YFTFS-RQGS--G-E-----HTA--
-------------------NLSLLLQRCNEVQLWVATEILLCSQLGKRVQLVKKFIKIA-
AHCKA-QRNLNSFFAIVMGLNTASVSRLSQTWEKIPGKFKKLFSELESLTDPSLNHKAYR
DAFKK--MKP-------PKIPF-MPLLLKDVTFI--------------------------
-----HEGNK--TFL-------D---NLVNFE-KLHMIADTV---RTLRHCRTNQFG-DL
SPKE------------------HQELKSYVNH-LYVIDSQQALFELSHR---IEPR
>Rapgef5_[Nothobranchius_furzeri]_1007726540
---DVAVALTSFDWTIFTSMHEQ----ELV-----YFTFT-RHVC--SN------QNT--
-------------------ALELLLQRCNEVQLWVMTEVLLCSTLCKRVQLIKKFIKIA-
AHCKA-QKNLNSFFAIIMGLNAAAVSRLSQTWEKIPGKFKRLFSELETVTDPSLNHKAYR
DSFKK--MKA-------PKIPF-LPLLLKDITFI--------------------------
-----HEGNR--TFH-------D---NLVNFE-KLHMIADMA---RLIRQCQADPVGNGI
TQKS------------------SSEVRAYVDN-LHVIDNQQTLFELSHR---TEPR
>Rapgef5_[Odobenus_rosmarus_divergens]_472355446
---DLALELMNFDWSLFNSIHEQ----ELI-----YFTFS-RQGS--G-E-----HTA--
-------------------NLSLLLQRCNEVQLWVATEILLCSQLGKRVQLVKKFIKIA-
AHCKA-QRNLNSFFAIVMGLNTASVSRLSQTWEKIPGKFKKLFSELESLTDPSLNHKAYR
DAFKK--MKP-------PKIPF-MPLLLKDVTFI--------------------------
-----HEGNK--TFL-------D---NLVNFE-KLHMIADTV---RTLRHCRTNQFG-DV
SPKE------------------HQELKSYVNH-LYVIDSQQALFELSHR---IEPR
>Rapgef5_[Opisthocomus_hoazin]_700379874
---DLALELTNFDWSLFNAIHEQ----ELI-----YFTFS-RQGS--A-E-----NTE--
-------------------NLSLLLQRCNEVQLWVATEILLCSQLCKRVQLVKKFIKIA-
AHCKA-QRNLNSFFAIVMGLNTASVSRLSQTWEKIPGKFKKLFTELESLTDPSLNHKAYR
DAFKK--MKS-------PKIPF-MPLLLKDVTFI--------------------------
-----HEGNK--TFL-------D---NLVNFE-KLHMIADTV---RSLRHCRNNQFGNEV
SSKE------------------HHELKPYVHH-LYVIDNQQALFELSHR---IEPR
>Rapgef5_[Orcinus_orca]_465971427
---DIALELMNFDWSLFNSIHEQ----ELI-----YFTFS-RQGS--G-D-----HTV--
-------------------NLSLLLQRCNEVQLWVATEILLCSQLGKRVQLVKKFIKIA-
AHCKA-QRNLNSFFAIVMGLNTASVSRLSQTWEKIPGKFKKLFSELESLTDPSLNHKAYR
DAFKK--MKP-------PKIPF-MPLLLKDVTFI--------------------------
-----HEGNK--TFL-------D---NLVNFE-KLHMIADTV---RTLRHCRTHQFG-DI
SPKE------------------HPELKSYVHH-LCVIDSQQALFALSHR---IEPR
>Rapgef5_[Oreochromis_niloticus]_542195430
---DVAVALTDFDWAIFDEVHEQ----ELV-----YFTFN-RNAG--SN------HTT--
-------------------ALEFLLQRCNDIQLWVMTEVLLCPTLCKRVQLIKKFIKIA-
AHCKA-QRNLNCFFAIIMGLNTAAVSRLSQTWEKVPGKFKRLFSELETVTDPSLNHKAYR
DSFKK--MKA-------PKIPF-LPLLLKDITFI--------------------------

```
-----HEGNK--TFH-------D---NLVNFE-KLHMIADTV---RLIRQCQKDHMGNGI
TEKS------------------SSEVRAYIDY-LHIIDNQQTLFELSQR---LEPR
>Rapgef5_[Ornithorhynchus_anatinus]_620951764
---DLALELMNFDWSLFNSIHEQ----ELI-----YFTFS-RQGS--S-E-----NTV--
-------------------NLSLLLQRCNEVQLWVSTEILLCSQLCKRVQLVKKFIKIA-
AHCKA-QRNLNSFFAIVMGLNTASISRLSQTWEKIPGKFKKLFSELESLTDPSLNHKAYR
DAFKK--MKP-------PKIPF-MPLLLKDVTFI--------------------------
-----HEGNK--TFL-------D---NLVNFE-KLHMIADTV---RSLRHCRNNQFGGEV
SSKD------------------HQELKSYVHH-LHIIDNQQALFELSHR---IEPR
>Rapgef5_[Orycteropus_afer_afer]_634824811
---DLALELMNFDWSLFNSIHEQ----ELI-----YFTFS-RQGS--G-E-----HTV--
-------------------NLSLLLQRCNEVQLWVATEILLCSQLGKRVQLVKKFIKIA-
THCKA-QRNLNSFFAIVMGLNTASVSRLSQTWEKIPGKFKKLFSELESLTDPSLNHKAYR
DAFKK--MKP-------PKIPF-MPLLLKDVTFI--------------------------
-----HEGNK--TFL-------D---NLVNFE-KLHMIADTV---RTLRHCRANQFG-DV
SPKE------------------HQELKSYVNH-LYVIDSQQALFELSHR---IEPR
>Rapgef5_[Oryzias_latipes]_432909226
---DVAVALTDLDWTIFDSMHEQ----EVI-----YFTFN-RHVC--ST------YTM--
-------------------ALELLLQRCNEVQLWVMTEVLLCPTLCKRVQLIKKFIKIA-
SHCKA-QRNLNCFFAIFMGLNAAAVSRLSHTWEKVPGKFKKLFSELEAITDPSLNHKAYR
ESLKK--MKV-------PKIPF-LPLLLKDITFI--------------------------
-----HEGNR--TFH-------N---NMVNFE-KLHMIADMV---RLIRQCQKDRMGNGI
TQKS------------------SSEVRAYIDC-LHVIDNQQTLFELSHR---LEPR
>Rapgef5_[Ovis_aries_musimon]_803284450
---DLALELMNFDWSLFNSIHEQ----ELI-----YFTFS-RQGS--G-E-----HTV--
-------------------NLSLLLQRCNEVQLWVATEILLCSPLGKRVQLVKKFIKIA-
AHCKA-QRNLNSFFAIVMGLNTASVSRLSQTWEKIPGKFKKLFSELESLTDPSLNHKAYR
DAFKK--MKP-------PKIPF-MPLLLKDVTFI--------------------------
-----HEGNK--TFL-------D---NLVNFE-KLHMIADTV---RTLRHCRTNQFGGDM
SPKE------------------HPELKSYVHH-LYVIDSQQALFELSHR---IEPR
>Rapgef5_[Ovis_aries]_803062190
---DLALELMNFDWSLFNSIHEQ----ELI-----YFTFS-RQGS--G-E-----HTV--
-------------------NLSLLLQRCNEVQLWVATEILLCSPLGKRVQLVKKFIKIA-
AHCKA-QRNLNSFFAIVMGLNTASVSRLSQTWEKIPGKFKKLFSELESLTDPSLNHKAYR
DAFKK--MKP-------PKIPF-MPLLLKDVTFI--------------------------
-----HEGNK--TFL-------D---NLVNFE-KLHMIADTV---RTLRHCRTNQFGGDM
SPKE------------------HPELKSYVHH-LYVIDSQQALFELSHR---IEPR
>Rapgef5_[Pan_troglodytes]_1034060316
---------------------------ELI-----YFTFS-RQGS--G-E-----HTA--
-------------------NLSLLLQRCNEVQLWVATEILLCSQLGKRVQLVKKFIKIA-
AHCKA-QRNLNSFFAIVMGLNTASVSRLSQTWEKIPGKFKKLFSELESLTDPSLNHKAYR
DAFKK--MKP-------PKIPF-MPLLLKDVTFI--------------------------
-----HEGNK--TFL-------D---NLVNFE-KLHMIADTV---RTLRHCRTNQFEED-
--------------------------------------------------------
>Rapgef5_[Panthera_tigris_altaica]_987416040
---DLALELMNFDWSLFNSIHEQ----ELI-----YFTFS-RQGS--G-E-----HTV--
-------------------NLSLLLQRCNEVQLWVATEILLCGQLGKRVQLVKKFIKIA-
AHCKA-QRNLNSFFAIVMGLNTASVSRLSQTWEKIPGKFKKLFSELESLTDPSLNHKAYR
DAFKK--MKP-------PKIPF-MPLLLKDVTFI--------------------------
-----HEGNK--TFL-------D---NLVNFE-KLHMIADTV---RTLRHCRTNQFGGDM
SPKE------------------HQELKSYVNH-LYVIDSQQALFELSHR---IEPR
>Rapgef5_[Pantholops_hodgsonii]_556767097
---DLALELMNFDWSLFNSIHEQ----ELI-----YFTFS-RQGS--G-E-----HTV--
-------------------NLSLLLQRCNEVQLWVATEILLCSPLGKRVQLVKKFIKIA-
```

AHCKA-QRNLNSFFAIVMGLNTASVSRLSQTWEKIPGKFKKLFSELESLTDPSLNHKAYR
DAFKK--MKP-------PKIPF-MPLLLKDVTFI-------------------------
-----HEGNK--TFL-------D---NLVNFE-KLHMIADTV---RTLRHCRTNQFG-DM
SPKE------------------HPEVKSYVHH-LHVIDSQQALFELSHR---IEPR
>Rapgef5_[Parus_major]_998665175
---DLALELTNFDWSLFNAIHEQ----ELI-----YFTFS-RQGS--A-E-----NTE--
-------------------NLSLLLQRCNEVQLWVATEILLCSQLCKRVQLVKKFIKIA-
AHCKA-QRNLNSFFAIVMGLNTASVSRLSQTWEKIPGKFKKLFTELENLTDPSLNHKAYR
DAFKK--MKS-------PKIPF-MPLLLKDVTFI-------------------------
-----HEGNK--TFL-------D---NLVNFE-KLHMIADTV---RSLRHCRNNQFGNEV
PSKE------------------HHELKPYVHH-LHVIDNQQALFELSHR---IEPR
>Rapgef5_[Pelecanus_crispus]_694641940
---DLALELTNFDWSLFNAIHEQ----ELI-----YFTFS-RQGS--A-E-----NTE--
-------------------NLSLLLQRCNEVQLWVATEILLCSQLCKRVQLVKKFIKIA-
AHCKA-QRNLNSFFAIVMGLNTASVSRLSQTWEKIPGKFKKLFTELESLTDPSLNHKAYR
DAFKK--MKS-------PKIPF-MPLLLKDVTFI-------------------------
-----HEGNK--TFL-------D---NLVNFE-KLHMIADTV---RSLRHCRNNQFGNEV
PSKE------------------HHELKPYVHH-LHVIDNQQALFELSHR---IEPR
>Rapgef5_[Peromyscus_maniculatus_bairdii]_1008780728
---DLALELMSFDWSLFNSIHEQ----ELI-----YFTFS-RQGS--G-E-----HTV--
-------------------NLSLLLQRCNEVQLWVATEILLCSQLGKRVQLVKKFIKIA-
AHCRA-QQNLNSFFAIVMGLNTASVSRLSQTWEKIPGKFKKLFSELESLTDPSLNHKAYR
DAFKK--MKP-------PKIPF-MPLLLKDVTFI-------------------------
-----HEGNK--TFL-------D---NLVNFE-KLHMIADTV---RTLRHCRSNQFGSDM
SPKE------------------QQELKSYVNH-LYVIDSQQALFELSHR---LEPR
>Rapgef5_[Phaethon_lepturus]_677491453
---DLALELTNFDWSLFNAIHEQ----ELI-----YFTFS-RQGS--A-E-----NTE--
-------------------NLSLLLQRCNEVQLWVATEILLCSQLCKRVQLVKKFIKIA-
AHCKA-QRNLNSFFAIVMGLNTASVSRLSQTWEKIPGKFKKLFTELESLTDPSLNHKAYR
DAFKK--MKS-------PKIPF-MPLLLKDVTFI-------------------------
-----HEGNK--TFL-------D---NLVNFE-KLHMIADTV---RSLRHCRNNQFGNEV
PSKE------------------HHELKPYVHH-LHVIDSQQALFELSHR---IEPR
>Rapgef5_[Physeter_catodon]_593712772
---DIALELMNFDWSLFSSIHEQ----ELI-----YFTFS-RQGS--G-E-----HTV--
-------------------NLSLLLQRCNEVQLWVATEILLCSQLGKRVQLVKKFIKIA-
AHCKA-QRNLNSFFAIVMGLNTASVSRLSQTWEKIPGKFKKLFSELESLTDPSLNHKAYR
DAFKK--MKP-------PKIPF-MPLLLKDVTFI-------------------------
-----HEGNK--TFL-------D---NLVNFE-KLHMIADTV---RTLRHCRTNQFGGDI
SPKE------------------HPELKSYVHH-LCVIDSQQALFALSHR---IEPR
>Rapgef5_[Poecilia_formosa]_617455312
---DVAVALTNFDWTIFNSMHEQ----ELI-----YFTFN-RHVC--SS------YTM--
-------------------PLEMLLQQCNEVQLWVMTEVLLCPTLCKRVQLIKKFIKIA-
AHCKA-QRNLNSFFAIIMGLNAGAVSRLSQTWEKIPGKFKKLFSELETVTDPSLNHKAYR
DSFKK--MKS-------PKIPF-LPLLLKDITFI-------------------------
-----HEGNK--TFH-------D---NLVNFE-KLHMIADMA---RLIRECQQDHVGNGI
AQKS------------------SSEVRAYTDY-LHVIDNQQTLFELSHR---LEPR
>Rapgef5_[Poecilia_mexicana]_961877551
---DVAVALTNFDWTIFNSMHEQ----ELI-----YFTFN-RHVC--SS------YTM--
-------------------PLEMLLQQCNEVQLWVMTEVLLCPTLCKRVQLIKKFIKIA-
AHCKA-QRNLNSFFAIIMGLNAGAVSRLSQTWEKIPGKFKKLFSELETVTDPSLNHKAYR
DSFKK--MKS-------PKIPF-LPLLLKDITFI-------------------------
-----HEGNK--TFH-------D---NLVNFE-KLHMIADMA---RLIRECQQDHVGNGI
AQKS------------------SSEVRAYTDY-LHVIDNQQTLFELSHR---LEPR
>Rapgef5_[Poecilia_reticulata]_658895691

---DVAVALTNFDWTIFNSMHEQ----ELI-----YFTFN-RHVC--SS------YTM--
-------------------PLEMLLQQCNEVQLWVMTEVLLCPTLCKRVQLIKKFIKIA-
AHCKA-QRNLNSFFAIIMGLNAGAVSRLSQTWEKIPGKFKKLFSELEMVTDPSLNHKAYR
DSFKK--MKS-------PKIPF-LPLLLKDITFI-------------------------
-----HEGNK--TFH-------D---NLVNFE-KLHMIADMA---RLIRECQQDHVGNGI
AQKS------------------SSEVRAYTDY-LHVIDNQQTLFELSHR---LEPR
>Rapgef5_[Pongo_abelii]_686725649
---DLALELMNFDWSLFNSIHEQ----ELI-----YFTFS-RQGS--G-E-----HTA--
-------------------NLSLLLQRCNEVQLWVATEILLCSQLGKRVQLVKKFIKIA-
AHCKA-QRNLNSFFAIVMGLNTASVSRLSQTWEKIPGKFKKLFSELESLTDPSLNHKAYR
DAFKK--MKP-------PKIPF-MPLLLKDVTFI-------------------------
-----HEGNK--TFL-------D---NLVNFE-KLHMIADTV---RTLRHCRTNQFG-DL
SPKE------------------HQELKSYVNH-LYVIDSQQALFELSHR---IEPR
>Rapgef5_[Protobothrops_mucrosquamatus]_1002574502
---NLALELTVFDWNLFNLVHEQ----ELI-----YFTFS-RQGS--G-K-----NTV--
-------------------NLSLLLQRCNEVQLWVATEILLCSQLCKRVQLVKKFIKIA-
AHCKA-QRNLNSFFAIVMGLNTASISRLSQTWERMPGKFKKLFAELESLTDPSLNHKAYR
DAFKK--MKS-------PKIPF-MPLLLKDVTFI-------------------------
-----HEGNK--TFL-------D---NLVNFE-KLHMIADTV---RSLRHCRRNQFGNDI
PSKE------------------HEELKSYVHH-LRIIDNQQMLFELSHR---IEPR
>Rapgef5_[Pseudopodoces_humilis]_929416305
---DLALELTNFDWSLFNAIHEQ----ELI-----YFTFS-RQGS--A-E-----NTE--
-------------------NLSLLLQRCNEVQLWVATEILLCSQLCKRVQLVKKFIKIA-
AHCKA-QRNLNSFFAIVMGLNTASVSRLSQTWEKIPGKFKKLFTELESLTDPSLNHKAYR
DAFKK--MKS-------PKIPF-MPLLLKDVTFI-------------------------
-----HEGNK--TFL-------D---NLVNFE-KLHMIADTV---RSLRHCRNNQFGNEV
PSKE------------------HHELKPYVHH-LHVIDNQQALFELSHR---IEPR
>Rapgef5_[Pteropus_alecto]_989943388
---DLALELMNFDWCLFNSIHEQ----ELI-----YFTFS-RQGS--G-E-----HTV--
-------------------NLSLLLQRCNEVQLWVATEILLCSQLGKRVQLVKKFIKIA-
AHCKA-QRNLNSFFAIVMGLNTASVSRLSQTWEKIPGKFKKLFSELESLTDPSLNHKAYR
DAFKK--MKP-------PKIPF-MPLLLKDVTFI-------------------------
-----HEGNK--TFL-------D---NLVNFE-KLHMIADTV---RTLRHCRTNQFGGEM
SPKE------------------HQELKSYVNH-LYVIDSQQALFELSHR---IEPR
>Rapgef5_[Pteropus_vampyrus]_759124282
---DLALELMNFDWCLFNSIHEQ----ELI-----YFTFS-RQGS--G-E-----HTV--
-------------------NLSLLLQRCNEVQLWVATEILLCSQLGKRVQLVKKFIKIA-
AHCKA-QRNLNSFFAIVMGLNTASVSRLSQTWEKIPGKFKKLFSELESLTDPSLNHKAYR
DAFKK--MKP-------PKIPF-MPLLLKDVTFI-------------------------
-----HEGNK--TFL-------D---NLVNFE-KLHMIADTV---RTLRHCRTNQFGGEM
SPKE------------------HQELKSYVNH-LYVIDSQQALFELSHR---IEPR
>Rapgef5_[Python_bivittatus]_602660999
---DLALELTIFDWNLFNLVHEQ----ELI-----YFTFS-RQGS--G-K-----NTV--
-------------------NLSLLLQRCNEVQLWVATEILLCNQLCKRVQLVKKFIKIA-
AHCKA-QRNLNSFFAIVMGLNTASVSRLSQTWERMPGKFKKLFAELESLTDPSLNHKAYR
DAFKK--MKS-------PKIPF-MPLLLKDVTFI-------------------------
-----HEGNK--TFL-------D---NLVNFE-KLHMIADTV---RSLRHCRRNQFGNEI
PSKE------------------HEELKSYVHH-LHIIDNQQMLFELSHR---IEPR
>Rapgef5_[Rhinopithecus_roxellana]_724802520
---DLALELMNFDWSLFNSIHEQ----ELI-----YFTFS-RQGS--G-E-----HTA--
-------------------NLSLLLQRCNEVQLWVATEILLCSQLGKRVQLVKKFIKIA-
AHCKA-QRNLNSFFAIVMGLNTASVSRLSQTWEKIPGKFKKLFSELESLTDPSLNHKAYR
DAFKK--MKP-------PKIPF-MPLLLKDVTFI-------------------------
-----HEGNK--TFL-------D---NLVNFE-KLHMIADTV---RTLRHCRTNQFG-DL

CPKE------------------HQELKSYVNH-LYVIDSQQALFELSHR---IEPR
>Rapgef5_[Rousettus_aegyptiacus]_1012209897
---DLALELMNFDWCLFNSIHEQ----ELI-----YFTFS-RQGN--G-E-----HTV--
-------------------NLSLLLQRCNEVQLWVATEILLCSQLGKRVQLVKKFIKIA-
AHCKA-QRNLNSFFAIVMGLNTASVSRLSQTWEKIPGKFKKLFSELESLTDPSLNHKAYR
DAFKK--MKP-------PKIPF-MPLLLKDVTFI--------------------------
-----HEGNK--TFL-------D---NLVNFE-KLHMIADTV---RTLRHCRTNQFGGEM
SPKE------------------HQELKSYVNH-LYVIDSQQALFELSHR---IEPR
>Rapgef5_[Saimiri_boliviensis_boliviensis]_403287902
---DLALELMNFDWSLFNSIHEQ----ELI-----YFTFS-RQGS--G-E-----HTA--
-------------------NLSLLLQRCNEVQLWVATEILLCSQLGKRVQLVKKFIKIA-
AHCKA-QRNLNSFFAIVMGLNTASVSRLSQTWEKIPGKFKKLFSELESLTDPSLNHKAYR
DAFKK--MKP-------PKIPF-MPLLLKDVTFI--------------------------
-----HEGNK--TFL-------D---NLVNFE-KLHMIADTV---RTLRHCRTNQFG-DL
SPKE------------------HQELKSYVNH-LYVIDSQQALFELSHR---IEPR
>Rapgef5_[Salmo_salar]_929093147
---DVAVTLTNFDWSVFNSIHEQ----ELV-----YFTFS-RHAS--G-G-----HTV--
-------------------ALELLLQRCNEVQLWVMTEVLMCPTLCNRVQLIKKFIKIA-
AHCKA-QRNLNSFFAIVMGLNTAAVSRLSQTWE---------------------------

>Rapgef5_[Sarcophilus_harrisii]_821488958
---DLAMELMNFDWSLFNSIHEQ----ELI-----YFTFS-RQGS--S-E-----NTV--
-------------------NLSLLLQRCNEVQLWVATEILLCSQLCKRVQLVKKFIKIA-
AHCKA-QRNLNSFFAIVMGLNTASVSRLSQTWEKIPGKFKKLFSELESLTDPSLNHKAYR
DAFKK--MKP-------PKIPF-MPLLLKDVTFI--------------------------
-----HEGNK--TFL-------D---NLVNFE-KLHMIADTV---RSLRHCRNNQFGGEV
SPKE------------------HQELRSYVHH-LHVIDNQQVLFELSHR---IEPR
>Rapgef5_[Sinocyclocheilus_anshuiensis]_1024985992
---DVSVALTNFDWNLFNSIHEQ----ELI-----FYTFS-RQAS--SG------HTV--
-------------------ALEFLLQRCNEVQQWVMSEVLLCPSLSKRVQLLKKFIKIA-
AHCKA-QRNLNSSFAIIMGLNTAAVSRLNQTWEKVPGKFKKLFSELELLTDPSMNHKAYR
DAFKK--TKP-------PKIPF-MPLLLKDITFI--------------------------
-----HEGNK--TFH-------D---SLVNFE-KLHMIADTV---RLIRHCQTDQTGNEL
SPCD------------------SPEVRSSVHY-LHIIDNQQTLFELSHR---LEPR
>Rapgef5_[Sinocyclocheilus_grahami]_1020566004
---DVSVALTNFDWNLFNSIHEQ----ELI-----FYTFS-RQAS--SG------HTV--
-------------------ALEFLLQRCNEVQQWVMSEVLLCPSLSKRVQLLKKFIKIA-
AHCKA-QRNLNSSFAIIMGLNTAAVSRLNQTWEKVPGKFKKLFSELELLTDPSMNHKAYR
DAFKK--TKP-------PKIPF-MPLLLKDITFI--------------------------
-----HEGNK--TFH-------D---NLVNFE-KLHMIADTV---RLIRHCQTDQTGNEL
SPCD------------------SPEVRSSVHY-LHIIDNQQTLFELSHR---LEPR
>Rapgef5_[Sinocyclocheilus_rhinocerous]_1025274731
---DVSVALTNFDWNLFNSIHEQ----ELI-----FYTFS-RQAS--SG------HTV--
-------------------ALEFLLQRCNEVQQWVMSEVLLCPSLSKRVQLLKKFIKIA-
AHCKA-QRNLNSSFAIIMGLNTAAVSRLNQTWEKVPGKFKKLFSELELLTDPSMNHKAYR
DAFKK--TKP-------PKIPF-MPLLLKDITFI--------------------------
-----HEGNK--TFH-------D---NLVNFE-KLHMIADTV---RLIRHCQTDQTGNEL
SPCD------------------SPEVRSSVHY-LHIIDNQQTLFELSHR---LEPR
>Rapgef5_[Sorex_araneus]_836703300
---DLALELMNFDWSLFNSIHEQ----ELI-----YFTFS-RQGS--G-E-----HTV--
-------------------NLSLLLQRCNEVQLWVATEILLCSQLGKRVQLVKKFIKIA-
AHCKA-QRNLNSFFAIVMGLNTASVSRLSQTWEKIPGKFKKLFSELESLTDPSLNHKAYR

DAFKK--MKP-------PKIPF-MPLLLKDVTFI--------------------------
-----HEGNK--TFL-------D---NLVNFE-KLSII----------------------

>Rapgef5_[Stegastes_partitus]_657578353
---DVAVALTDFDLTIFDSMHEQ----ELV-----YFTFN-RHVT--SN------HTT--
-------------------ALELLLQRCNEVQLWVMTEVLLCPTLCKRVQLIKKFIKIA-
AHCKA-QRNLNCFFAIIMGLNAAAVSRLAQTWEKIPGKFKKLFSELETVTDPSLNHKAYR
DSFKK--MKA-------PKIPF-LPLLLKDITFI--------------------------
-----HEGNK--TFH-------D---NLVNFE-KLHMIAEMA---RLIRQCQKDPMGNGI
TQKS------------------SSEVRAYVDY-LHIIDNQQTLFELSHR---LEPR
>Rapgef5_[Struthio_camelus_australis]_697491700
---DLALELTNFDWSLFNAIHEQ----ELI-----YFTFS-RQGS--A-E-----NTE--
-------------------NLSLLLQRCNEVQLWVATEILLCSQLCKRVQLVKKFIKIA-
AHCKA-QRNLNSFFAIVMGLNTASVSRLSQTWEKIPGKFKKLFTELESLTDPSLNHKAYR
DAFKK--MKS-------PKIPF-MPLLLKDVTFI--------------------------
-----HEGNK--TFL-------D---NLVNFE-KLHMIADTV---RSLRHCRNNQFGNEV
PSKE------------------HHELKPYVHH-LHVIDNQQALFELSHR---IEPR
>Rapgef5_[Sturnus_vulgaris]_959041358
---DLALELTNFDWSLFNAIHEQ----ELI-----YFTFS-RQGS--A-E-----NTE--
-------------------NLSLLLQRCNEVQLWVATEILLCGQLCKRVQLVKKFIKIA-
AHCKA-QRNLNSFFAIVMGLNTASVSRLSQTWEKIPGKFKKLFTELESLTDPSLNHKAYR
DAFKK--MKS-------PKIPF-MPLLLKDVTFI--------------------------
-----HEGNK--TFL-------D---NLVNFE-KLHMIADTV---RSLRHCRNNQFGNEV
PSKE------------------HHELKPYVHH-LHVIDNQQALFELSHR---IEPR
>Rapgef5_[Sus_scrofa]_927180342
---DLALELMNFDWSLFNSIHEQ----ELI-----YFTFS-RQGS--G-E-----HTV--
-------------------NLSLLLQRCNEVQLWVATEILLCSQLGKRVQLVKKFIKIA-
AHCKA-QRNLNSFFAIVMGLNTASVSRLSQTWEKIPGKFKKLFSELESLTDPSLNHKAYR
DAFKK--MKP-------PKIPF-MPLLLKDVTFI--------------------------
-----HEGNK--TFL-------D---NLVNFE-KLHMIADTV---RTLRHCRTNQFGGDM
SPKE------------------HQELKSYVHH-LYIIDSQQALFELSHR---IEPR
>Rapgef5_[Taeniopygia_guttata]_823455916
---DLALELTNFDWSLFNAIHEQ----ELI-----YFTFS-RQGS--A-E-----NTE--
-------------------NLSLLLQRCNEVQLWVATEILLCSQLCKRVQLVKKFIKIA-
AHCKA-QRNLNSFFAIVMGLNTASVSRLSQTWEKIPGKFKKLFAELESLTDPSLNHKAYR
DAFKK--MKS-------PKIPF-MPLLLKDVTFI--------------------------
-----HEGNK--TFL-------D---NLVNFE-KLHMIADTV---RSLRHCRNNQFGNEV
PSKE------------------HHELKPYVHH-LHVIDNQQALFELSHR---IEPR
>Rapgef5_[Takifugu_rubripes]_768922189
---DVAVALTQLDWAIFHSVHEQ----DLV-----YFTFS-RHSS--SSSSSSSSRTV--
-------------------GLELLLQRCNEVQLWVMTEVLLCPLLCKRVRLIKKFIKIA-
AHCKA-QKNLNCFFAIIMGLNTAAISRLSQTWEKIPGKFKKLFSELEAITDPSLNHKAYR
DSFKK--MKA-------PKIPF-LPLLLKDITFI--------------------------
-----HEGNK--TFH-------D---NLVNFE-KLHMIADAV---RFLRQCQRDHMGNGI
IQKS------------------SSEARAHIDY-LHVIDNQQTLFELSHR---LEPR
>Rapgef5_[Trichechus_manatus_latirostris]_471379407
---DLALELMNFDWSLFNSIHEQ----ELI-----YFTFS-RQGS--G-E-----HTV--
-------------------NLSLLLQRCNEVQLWVATEILLCSQLGKRVQLVKKFIKIA-
AHCKA-QKNLNSFFAIVMGLNTASVSRLSQTWEKIPGKFKKLFSELESLTDPSLNHKAYR
DAFKK--MKP-------PKIPF-MPLLLKDVTFI--------------------------
-----HEGNK--TFL-------D---NLVNFE-KLHMIADTV---RTLRHCRTNQFG-DV
SPKE------------------HQELKSYVNH-LYVIDSQQALFELSHR---IEPR
>Rapgef5_[Tyto_alba]_701352674
---DLALELTNFDWSLFNAIHEQ----ELI-----YFTFS-RQGN--A-E-----NTE--

-------------------NLSLLLQRCNEVQLWVATEVLLCSQLCKRVQLVKKFIKIA-
AHCKA-QRNLNSFFAIVMGLNTASVSRLSQTWEKIPGKFKKLFTELESLTDPSLNHKAYR
DAFKK--MKS-------PKIPF-MPLLLKDVTFI-------------------------
-----HEGNK--TFL-------D---NLVNFE-KLHMIADTV---RSLRHCRNNQFGNEV
PSKE------------------HHELKPYVHH-LHVIDNQQALFELSHR---IEPR
>Rapgef5_[Ursus_maritimus]_670987468
---DLALELMNFDWSLFNSIHEQ----ELI-----YFTFS-RQGS--G-E-----HTA--
-------------------NLSLLLQRCNEIQLWVATEILLCAQLGKRVQLVKKFIKIA-
AHCKA-QRNLNSFFAIVMGLNTASVSRLSQTWEKIPGKFKKLFSELESLTDPSLNHKAYR
DAFKK--MKP-------PKIPF-MPLLLKDVTFI-------------------------
-----HEGNK--TFL-------D---NLVNFE-KLHMIADTV---RTLRHCRANQFGGDV
SPKE------------------HQELKAYVTH-LYVIDSQQALFELSHR---IEPR
>Rapgef5_[Vicugna_pacos]_970711077
---DLALELMNFDWSLFNSIHEQ----ELI-----YFTFS-RQGG--G-E-----HTM--
-------------------NLSFLLQRCNEVQLWVATEILLCSQLGKRVQLVKKFIKIA-
AHCKA-QRNLNSFFAIVMGLNIASVSRLSQTWEKIPGKFKKLFSELESLTDPSLNHKAYR
DAFKK--MKP-------PKIPF-MPLLLKDVTFI-------------------------
-----HEGNK--TFL-------D---NLVNFE-KLHMIADTV---RTLRHCRTNQFGGDM
SPKE------------------HQELKSYVNH-LYVIDSQQALFELSHR---IEPR
>Rapgef5_[Xenopus_tropicalis]_847139992
---DIAFELTSFDWSLFNSIHEQ----ELI-----YFTFS-RQGK--G-E-----NTL--
-------------------NLSLLLQRCNEVQLWVATEILLCNQPGKRVQLLKKFIKIA-
AHCKA-QRNLNSFFAIIMGLNTASVSRLSQTWEKIPGKFKKLFSELESLTDPSLNHKAYR
DAFKK--MKP-------PKIPF-MPLLLKDVTFI-------------------------
-----HEGNK--TFL-------D---NLVNFE-KLHMIADAV---RSVRYCRNNQFGNDL
PQKE------------------RQEAKFYSNH-LHVIDNQQTLFELSHR---IEPR
>Rapgef5_[Zonotrichia_albicollis]_929433197
---DLALELTNFDWSLFNAIHEQ----ELI-----YFTFS-RQGS--A-E-----NTE--
-------------------NLSLLLQRCNEVQLWVATEILLCSQLCKRVQLVKKFIKIA-
AHCKA-QRNLNSFFAIVMGLNTASVSRLSQTWEKIPGKFKKLFTELESLTDPSLNHKAYR
DAFKK--MKS-------PKIPF-MPLLLKDVTFI-------------------------
-----HEGNK--TFL-------D---NLVNFE-KLHMIADTV---RSLRHCRNNQFGNEV
PSKE------------------HHELKPYVHH-LHVIDNQQALFELSHR---IEPR
>Rapgef6_[Acanthisitta_chloris]_678007447
STIEVATQLSMRDFELFRNIEPT----EYI-----DDLYK-L-DS----K-TGN-A----
-------------------HLKQFEDVINQETFWVAMEILAEPNQLKRMKIIKHFIKIA-
LHCRE-CKNFNSMFAVISGLNLAPVARLRGTWEKLPSKYEKHLRDLQDLFDPSRNMAKYR
NILSSQSMQP-------PIIPL-FPVVKKDITFL-------------------------
-----HEGND--SKV-------D---GLVNFE-KLRMIAKEI---RQVVRMTSANMDP--

>Rapgef6_[Acinonyx_jubatus]_961725173
STIEVATQLSMRDFDLFRNIEPT----EYI-----DDLFK-L-DS----K-TGN-T----
-------------------HLKEFEDIVNQETFWVASEILTEPNQLKRMKIIKHFIKIA-
LHCRE-CKNFNSMFAIISGLNLASVARLRGTWEKLPSKYEKHLQDLQDLFDPSRNMAKYR
NILSSQSMQP-------PIIPL-FPVVKKDMTFL-------------------------
-----HEGND--SKV-------D---GLVNFE-KLRMIAKEI---RQVVRMTSANMDP--

>Rapgef6_[Acromyrmex_echinatior]_746838642
---EVAIQLTLQDFSIFRQIEST----EYV-----DDLFE-L-KS----R-YGV-PM---
--------------------LSQFAELVNREMFWVVTEVCSEHNLVRRSKIIKQFIKIA-
RQCKE-CKNFNSMFAIVSGLGHGSVSRLRASWEKLPTKYQRLFSDLQELMDPSRNMSKYR
QLVASEQTQP-------PIIPF-YPVVKKDLTFI-------------------------
-----HLGND--SRV-------E---SLVNFE-KLRMIAKEV---RTLTNMCSSPYD---

>Rapgef6_[Ailuropoda_melanoleuca]_752428017
STIEVATQLSMRDFDLFRNIEPT----EYI-----DDLFK-L-DS----K-TGN-T----
-------------------HLKEFEDIVNQETFWVASEILTESNQLKRMKIIKHFIKIA-
LHCRE-CKNFNSMFAIISGLNLASVARLRGTWEKLPSKYEKHLQDLQDLFDPSRNMAKYR
NILSSQSMQP-------PIIPL-FPVVKKDMTFL--------------------------
-----HEGND--SKV-------D---GLVNFE-KLRMIAKEI---RQVVRMTSANMDP--

>Rapgef6_[Alligator_mississippiensis]_950961550
STIEVATQLSMRDFELFRNIEPT----EYI-----DDLFK-L-DS----K-TGN-A----
-------------------HLKQFEDVINQETFWVATEILLEPNQLKRMKIIKHFIKIA-
LHCRE-CKNFNSMFAVISGLNLASVARLRGTWEKLPSKYEKHFRDLQDLFDPSRNMAKYR
NILSSQSMQP-------PIIPL-FPVVKKDITFL--------------------------
-----HEGND--SKV-------D---GLVNFE-KLRMIAKEI---RQVIRMTSANMDP--

>Rapgef6_[Amphimedon_queenslandica]_761908046
-AMQVAEELTRRDSSVFSSIFPS----EFI-----SDLWK-SPDK----A-----ARR--
-------------------HLIEFEEIPNDEMYWVITTIVSEPSATNRSKIIKHFIKIA-
KCCKT-LKNYNTMFHLLSGLNHGLVQRLRSSWEKVPHRYKKTMEDLGSFMNPFHNMVKYR
ELQRQ--TQP-------PLIPF-FPIIKKDLTFL--------------------------
-----FDGND--TIV-------S---GLINFE-KLRMLSRQI---RIV------------

>Rapgef6_[Anolis_carolinensis]_637269194
STIEVATQLSMRDFELFRNIEPT----EYI-----DDLFK-L-ES----K-TGN-A----
-------------------NLKQFEDVINQETFWVATEILSESNQLKRMKIVKHFIKIA-
LHCRE-CKNFNSMFAVISGLNLAPVARLRSTWERLPNKYEKHLRDLQDLFDPSRNMAKYR
NILSSQSMQP-------PIIPL-FPVVKKDITFL--------------------------
-----HEGND--SKV-------D---GLVNFE-KLRMIAKEI---RQVIRMTSANMDP--

>Rapgef6_[Anser_cygnoides_domesticus]_902883087
STIEVATQLSMRDFELFRNIEPT----EYI-----DDLYK-L-DS----K-TGN-A----
-------------------HLKQFEDVINQETFWVAMEILAEPNQLKRMKIIKHFIKIA-
LHCRE-CKNFNSMFAVISGLNLAPVARLRGTWEKLPSKYEKHLRDLQDLFDPSRNMAKYR
NILSSQSMQP-------PIIPL-LHPFYRNCVFN--------------------------
-----YAGND--SKV-------D---GLVNFE-KLRMIAKEI---RQVVRMTSANMDP--

>Rapgef6_[Apis_cerana]_1035602523
---EVAIQLTLQDFSIFRQIEST----EYV-----DDLFE-L-KS----R-YGV-PM---
--------------------LRQFAELVNREMFWVVTEVCSEHNLVRRSKIIKQFIKIA-
RQCKE-CKNFNSMFAIVSGLGHGAVSRLRASWEKLPSKYQRLFSDLQELMDPSRNMSKYR
QLVASEQTQP-------PIIPF-YPVVKKDLTFI--------------------------
-----HLGND--SRV-------E---GLVNFE-KLRMIAKEV---RTLTNMCSSPYD---

>Rapgef6_[Apis_dorsata]_572301839
---EVAIQLTLQDFSIFRQIEST----EYV-----DDLFE-L-KS----R-YGV-PM---
--------------------LRQFAELVNREMFWVVTEVCSEHNLVRRSKIIKQFIKIA-
RQCKE-CKNFNSMFAIVSGLGHGAVSRLRASWEKLPSKYQRLFSDLQELMDPSRNMSKYR
QLVASEQTQP-------PIIPF-YPVVKKDLTFI--------------------------
-----HLGND--SRV-------E---GLVNFE-KLRMIAKEV---RTLTNMCSSPYD---

>Rapgef6_[Apis_florea]_820851405
---EVAIQLTLQDFSIFRQIEST----EYV-----DDLFE-L-KS----R-YGV-PM---
--------------------LRQFAELVNREMFWVVTEVCSEHNLVRRSKIIKQFIKIA-
RQCKE-CKNFNSMFAIVSGLGHGAVSRLRASWEKLPSKYQRLFSDLQELMDPSRNMSKYR
QLVASEQTQP-------PIIPF-YPVVKKDLTFI--------------------------

```
-----HLGND--SRV-------E---GLVNFE-KLRMIAKEV---RTLTNMCSSPYD---
--------------------------------------------------------
>Rapgef6_[Aptenodytes_forsteri]_686614740
STIEVATQLSMRDFELFRNIEPT----EYI-----DDLYK-L-DS----K-TGN-A----
-------------------HLKQFEDVINQETFWVAMEILAEPNQLKRMKIIKHFIKIA-
LHCRE-CKNFNSMFAVISGLNLAPVARLRGTWEKLPSKYEKHLRDLQDLFDPSRNMAKYR
NILSSQSMQP-------PIIPL-FPVVKKDITFL--------------------------
-----HEGND--SKV-------D---GLVNFE-KLRMIAKEI---RQVVRMTSANMDP--
--------------------------------------------------------
>Rapgef6_[Apteryx_australis_mantelli]_926505088
STIEVATQLSMRDFELFRNIEPT----EYI-----DDLYK-L-DS----K-TGN-A----
-------------------RLKQFEDVINQETFWVAMEILAEPNQLKRMKIIKHFIKIA-
LHCRE-CKNFNSMFAVISGLNLAPVARLRGTWEKLPSKYEKHLRDLQDLFDPSRNMAKYR
NILSSQSMQP-------PIIPL-FPVVKKDITFL--------------------------
-----HEGND--SKV-------D---GLVNFE-KLRMIAKEI---RQVVRMTSANMDP--
--------------------------------------------------------
>Rapgef6_[Aquila_chrysaetos_canadensis]_768345498
STIEVATQLSMRDFELFRNIEPT----EYI-----DDLYK-L-DS----K-TGN-A----
-------------------HLKQFEDVINQETFWVAMEILAEPNQLKRMKIIKHFIKIA-
LHCRE-CKNFNSMFAVISGLNLAPVARLRGTWEKLPSKYEKHLRDLQDLFDPSRNMAKYR
NILSSQSMQP-------PIIPL-FPVVKKDITFL--------------------------
-----HEGND--SKV-------D---GLVNFE-KLRMIAKEI---RQVVRMTSANMDP--
--------------------------------------------------------
>Rapgef6_[Athalia_rosae]_817056269
---EVAIQLTLQDFSIFRQIEST----EYV-----DDLFK-L-KS----R-YGV-PM---
--------------------LSHFAELVNREMFWVVTEVCSEHNLVRRSKIIKQFLKIA-
RQCKE-CKNFNSMFAIVSGLGHGAVCRLRGSWEKLPSKYQRLFTDLEKLMDPSRNMCQYR
QLVASEQTQP-------PVIPF-YPVVRKDLTFI--------------------------
-----HLGND--SRV-------E---GLVNFE-KLRMIAKEV---RTLTKMCSSPYD---
--------------------------------------------------------
>Rapgef6_[Balaenoptera_acutorostrata_scammoni]_594681842
STIEVATQLSMRDFDLFRNIEPT----EYI-----DDLFK-L-DS----K-TGN-T----
-------------------HLKEFEDIVNQETFWVASEILTESNQLKRMKIIKHFIKIA-
LHCRE-CKNFNSMFAIISGLNLASVARLRGTWEKLPSKYEKHLQDLQDLFDPSRNMAKYR
NILSSQSMQP-------PIIPL-FPVVKKDMTFL--------------------------
-----HEGND--SKV-------D---GLVNFE-KLRMIAKEI---RQVVRMTSANMDP--
--------------------------------------------------------
>Rapgef6_[Bison_bison_bison]_742173397
STIEVATQLSMRDFDLFRNIEPT----EYI-----DDLFK-L-ES----K-TGN-T----
-------------------HLKEFEDIVNQETFWVASEILTESNQLKRMKIIKHFIKIA-
LHCRE-CKNFNSMFAIISGLNLASVARLRGTWEKLPSKYEKHFQDLQDLFDPSRNMAKYR
NILSSQSMQP-------PIIPL-FPVVKKDMTFL--------------------------
-----HEGND--SKV-------D---GLVNFE-KLRMIAKEI---RQVVRMTSANMDP--
--------------------------------------------------------
>Rapgef6_[Bombus_terrestris]_808120520
---EVAIQLTLQDFSIFRQIEST----EYV-----DDLFE-L-KS----R-YGV-PM---
--------------------LRQFAELVNREMFWVVTEVCSEHNLVRRSKIIKQFIKIA-
RQCKE-CKNFNSMFAIVSGLGHGAVSRLRASWEKLPSKYQRLFSDLQELMDPSRNMSKYR
QLVASEQTQP-------PIIPF-YPVVKKDLTFI--------------------------
-----HLGND--SRV-------E---GLVNFE-KLRMIAKEV---RTLTNMCSSPYD---
--------------------------------------------------------
>Rapgef6_[Bos_mutus]_440912203
STIEVATQLSMRDFDLFRNIEPT----EYI-----DDLFK-L-ES----K-TGN-T----
-------------------HLKEFEDIVNQETFWVASEILTESNQLKRMKIIKHFIKIA-
```

```
LHCRE-CKNFNSMFAIISGLNLASVARLRGTWEKLPSKYEKHFQDLQDLFDPSRNMAKYR
NILSSQSMQP-------PIIPLFFPVVKKDMTFL-------------------------
-----HEGND--SKV-------D---GLVNFE-KLRMIAKEI---RQVVRMTSANMDP--
------------------------------------------------------------
>Rapgef6_[Bos_taurus]_528955952
STIEVATQLSMRDFDLFRNIEPT----EYI-----DDLFK-L-ES----K-TGN-T----
-------------------HLKEFEDIVNQETFWVASEILTESNQLKRMKIIKHFIKIA-
LHCRE-CKNFNSMFAIISGLNLASVARLRGTWEKLPSKYDKHFQDLQDLFDPSRNMAKYR
NILSSQSMQP-------PIIPL-FPVVKKDMTFL-------------------------
-----HEGND--SKV-------D---GLVNFE-KLRMIAKEI---RQVVRMTSANMDP--
------------------------------------------------------------
>Rapgef6_[Buceros_rhinoceros_silvestris]_704183445
STIEVATQLSMRDFELFRNIEPT----EYI-----DDLYK-L-DS----K-TGN-A----
-------------------HLKQFEDVINQETFWVAMEILAEPNQLKRMKIIKHFIKIA-
LHCRE-CKNFNSMFAVISGLNLAPVARLRGTWEKLPSKYEKHLRDLQDLFDPSRNMAKYR
NILSSQSMQP-------PIIPL-FPVVKKDITFL-------------------------
-----HEGND--SKV-------D---GLVNFE-KLRMIAKEI---RQVVRMTSANMDP--
------------------------------------------------------------
>Rapgef6_[Calidris_pugnax]_960977010
STIEVATQLSMRDFELFRNIEPT----EYI-----DDLYK-L-DS----K-TGN-A----
-------------------HLKQFEDVINQETFWVAMEILAEPNQLKRMKIIKHFIKIA-
LHCRE-CKNFNSMFAVISGLNLAPVARLRGTWEKLPSKYEKHLRDLQDLFDPSRNMAKYR
NILSSQSMQP-------PIIPL-FPVVKKDITFL-------------------------
-----HEGND--SKV-------D---GLVNFE-KLRMIAKEI---RQVVRMTSANMDP--
------------------------------------------------------------
>Rapgef6_[Callorhinchus_milii]_632966534
STIEVATQLSMRDFELFRNIEPT----EYI-----DDLFK-R-ES----K-SGN-A----
-------------------HLKQFEDIINQETFWVATEILKEVIQLKRMKIIKHFVKVA-
LHCRE-CKNFNSMFAIISGLNLAPVARLRGTWEKLPNKYEKLLRDLQDLFDPSRNMAKYR
NVLSSQSLQP-------PIIPL-FPVVKKDLTFL-------------------------
-----HEGND--SKV-------E---GLANFE-KLRMIAKEI---RHVVRMTSVNMDP--
------------------------------------------------------------
>Rapgef6_[Calypte_anna]_663267201
STIEVATQLSMRDFELFRNIEPT----EYI-----DDLYK-L-DS----K-TGN-A----
-------------------HLKQFEDVINQETFWVAMEILAEPNQLKRMKIIKHFIKIA-
LHCRE-CKNFNSMFAVISGLNLAPVARLRGTWEKLPSKYEKHLRDLQDLFDPSRNMAKYR
NILSSQSMQP-------PIIPL-FPVVKKDITFL-------------------------
-----HEGND--SKV-------D---GLVNFE-KLRMIAKEI---RQVIRMTSANMDP--
------------------------------------------------------------
>Rapgef6_[Camponotus_floridanus]_752870378
---EVAIQLTLQDFSIFRQIEST----EYV-----DDLFE-L-KS----R-YGV-PM---
--------------------LSQFAELVNREMFWVVTEVCSEHNLVRRSKIIKQFIKIA-
RQCKE-CKNFNSMFAIVSGLGHGAVSRLRASWEKLPSKYQRLFSDLQELMDPSRNMSKYR
QLVASEQTQP-------PIIPF-YPVVKKDLTFI-------------------------
-----HLGND--SRV-------E---GLVNFE-KLRMIAKEV---RTLTNMCSSPYD---
------------------------------------------------------------
>Rapgef6_[Capra_hircus]_548473189
STIEVATQLSMRDFDLFRNIEPT----EYI-----DDLFK-L-ES----K-TGN-T----
-------------------HLKEFEDIVNQETFWVASEILTESNQLKRMKIIKHFIKIA-
LHCRE-CKNFNSMFAIISGLNLASVARLRGTWEKLPSKYEKHFQDLQDLFDPSRNMAKYR
NILSSQSMQP-------PIIPL-FPVVKKDMTFL-------------------------
-----HEGND--SKV-------D---GLVNFE-KLRMIAKEI---RQVVRMTSANMDP--
------------------------------------------------------------
>Rapgef6_[Caprimulgus_carolinensis]_704293339
```

STIEVATQLSMRDFELFRNIEPT----EYI-----DDLYK-L-DS----K-TGN-A----
-------------------HLKQFEDVINQETFWVAMEILAEPNQLKRMKIIKHFIKIA-
LHCRE-CKNFNSMFAVISGLNLAPVARLRGTWEKLPSKYEKHLRDLQDLFDPSRNMAKYR
NILSSQSMQP-------PIIPL-FPVVKKDITFL--------------------------
-----HEGND--SKV-------D---GLVNFE-KLRMIAKEI---RQVVRMTSANMDP--

>Rapgef6_[Cariama_cristata]_677240024
STIEVATQLSMRDFELFRNIEPT----EYI-----DDLYK-L-DS----K-TGN-A----
-------------------HLKQFEDVINQETFWVAMEILAEPNQLKRMKIIKHFIKIA-
LHCRE-CKNFNSMFAVISGLNLAPVARLRGTWEKLPSKYEKHLRDLQDLFDPSRNMAKYR
NILSSQSMQP-------PIIPL-FPVVKKDITFL--------------------------
-----HEGND--SKV-------D---GLVNFE-KLRMIAKEI---RQVVRMTSANMDP--

>Rapgef6_[Carlito_syrichta]_640806933
STIEVATQLSMRDFDLFRNIEPT----EYI-----DDLFK-I-DS----K-TGN-T----
-------------------HLKEFADVVNQETFWVASEILTEPNQLKRMKIIKHFIKIA-
LHCRE-CKNFNSMFAIISGLNLAPVARLRGTWEKLPSKYEKHLQDLQDLFDPSRNMAKYR
NILSSQSMQP-------PIIPL-FPVVKKDMTFL--------------------------
-----HEGND--SKV-------D---GLVNFE-KLRMISKEI---RQVVRMTSANMDP--

>Rapgef6_[Cebus_capucinus_imitator]_1044364608
STIEVATQLSMRDFDLFRNIEPT----EYI-----DDLFK-L-NS----K-TGN-S----
-------------------HLKRFEDIVNQETFWVASEILTEANQLKRMKIIKHFIKIA-
LHCRE-CKNFNSMFAIISGLNLASVARLRGTWEKLPSKYEKHLQDLQDIFDPSRNMAKYR
NILSSQSMQP-------PIIPL-FPVVKKDMTFL--------------------------
-----HEGND--SKV-------D---GLVNFE-KLRMISKEI---RQVVRMTSANMDP--

>Rapgef6_[Cephus_cinctus]_1000734957
---EVAIQLTLQDFSIFRQIEST----EYV-----DDLFE-L-KS----R-YGV-PM---
--------------------LSQFAELVNREMFWVVTEVCSEHNLVRRSKIIKQFIKVA-
RQCKE-CKNFNSMFAIVSGLGHGAVSRLRASWEKLPSKYQRLFSDLQELMDPSRNMSKYR
QLVASEQTQP-------PIIPF-YPVVKKDLTFI--------------------------
-----HLGND--SRV-------E---GLVNFE-KLRMIAKEV---RTLTNMCSSPYD---

>Rapgef6_[Ceratotherium_simum_simum]_955486624
STIEVATQLSMRDFDLFRNIEPT----EYI-----DDLFK-L-DS----K-TGN-T----
-------------------HLKEFEDVVNQETFWVASEILTESNQLKRMKLIKHFIKIA-
LHCRE-CKNFNSMFAIISGLNLSSVARLRGTWEKLPSKYEKHLQDLQDLFDPSRNMAKYR
NILSSQSMQP-------PIIPL-YPVVKKDMTFL--------------------------
-----HEGND--SKV-------D---GLVNFE-KLRMIAREI---RQVVRMTSANMDP--

>Rapgef6_[Cercocebus_atys]_795413614
STIEVATQLSMRDFDLFRNIEPT----EYI-----DDLFK-L-NS----K-AGN-T----
-------------------HLKRFEDIVNQETFWVASEILTEANQLKRMKIIKHFIKIA-
LHCRE-CKNFNSMFAIISGLNLASVARLRGTWEKLPSKYEKHLQDLQDIFDPSRNMAKYR
NILSSQSMQP-------PIIPL-FPVVKKDMTFL--------------------------
-----HEGND--SKV-------D---GLVNFE-KLRMISKEI---RQVVRMTSANMDP--

>Rapgef6_[Chaetura_pelagica]_701407210
STIEVATQLSMRDFELFRNIEPT----EYI-----DDLYK-L-DS----K-TGN-A----
-------------------HLKQFEDVINQETFWVAMEILAEPNQLKRMKIIKHFIKIA-
LHCRE-CKNFNSMFAVISGLNLAPVARLRGTWEKLPSKYEKHLRDLQDLFDPSRNMAKYR
NILSSQSMQP-------PIIPL-FPVVKKDITFL--------------------------
-----HEGND--SKV-------D---GLVNFE-KLRMIAKEI---RQVIRMTSANMDP--

>Rapgef6_[Chinchilla_lanigera]_533121507
STIELAAQLSMRDFDLFRNIEPT----EYI-----DDIFK-L-DS----K-TGN-T----
-------------------HLKEFEDIVNQETFWVASEILTESNQLKRMKIIKHFIKIA-
LHCRE-CKNFNSMFAIISGLNLAPVARLRGTWEKLPSKYEKHLQDLQDLFDPSRNMAKYR
NILSSQSMQP-------PIIPL-FPVVKKDMTFL--------------------------
-----HEGND--SKV-------D---GLVNFE-KLRMISKEI---RQVVRMTSANMDP--

>Rapgef6_[Chlamydotis_macqueenii]_705691314
STIEVATQLSMRDFELFRNIEPT----EYI-----DDLYK-L-DS----K-TGN-A----
-------------------HLKQFEDVINQETFWVAMEILAEPNQLKRMKIIKHFIKIA-
LHCRE-CKNFNSMFAVISGLNLAPVARLRGTWEKLPSKYEKHLRDLQDLFDPSRNMAKYR
NILSSQSMQP-------PIIPL-FPVVKKDITFL--------------------------
-----HEGND--SKV-------D---GLVNFE-KLRMIAKEI---RQVVRMTSANMDP--

>Rapgef6_[Chrysemys_picta_bellii]_530612721
STIEVATQLSMRDFELFRNIEPT----EYI-----DDLYK-M-DS----K-TGN-A----
-------------------HLKQFEDVINQETFWVATEILTEPNQLKRMKIVKHFIKIA-
LHCRE-CKNFNSMFAVISGLNLAPVARLRGTWEKLPSKYEKHLRDLQDLFDPSRNMAKYR
NILSSQSMQP-------PIIPL-FPVVKKDITFL--------------------------
-----HEGND--SKV-------D---GLVNFE-KLRMIAKEI---RQVVRMTSANMDP--

>Rapgef6_[Chrysochloris_asiatica]_586485014
STIEVATQLSMRDFDLFRNIEPT----EYI-----DDLYK-L-DS----K-TGS-T----
-------------------HLKEFEDIVNQETFWVASEILTESNQLKRMKIIKHFIKIA-
LHCRE-CKNFNSMFAIISGLNLASVARLRGTWEKLPNKYEKHLQDLQDLFDPSRNMAKYR
NILSSQSMQP-------PIIPL-FPVVKKDMTFL--------------------------
-----HEGND--SKV-------D---GLVNFE-KLRMIAKEI---RQVIRMTSANMDP--

>Rapgef6_[Colius_striatus]_706103685
STIEVATQLSMRDFELFRNIEPT----EYI-----DDLYK-L-DS----K-TGN-A----
-------------------HLKQFEDVINQETFWVAMEILAEPNQLKRMKIIKHFIKIA-
LHCRE-CKNFNSMFAVISGLNLAPVARLRGTWEKLPSKYEKHLRDLQDLFDPSRNMAKYR
NILSSQSMQP-------PIIPL-FPVVKKDITFL--------------------------
-----HEGND--SKV-------D---GLVNFE-KLRMIAKEI---RQVVRMTSANMDP--

>Rapgef6_[Colobus_angolensis_palliatus]_795224891
STIEVATQLSMRDFDLFRNIEPT----EYI-----DDLFK-L-NS----K-TGN-T----
-------------------HLKRFEDIVNQETFWVASEILTEANQLKRMKIIKHFIKIA-
LHCRE-CKNFNSMFAIISGLNLASVARLRGTWEKLPSKYEKHLQDLQDIFDPSRNMAKYR
NILSSQSMQP-------PIIPL-FPVVKKDMTFL--------------------------
-----HEGND--SKV-------D---GLVNFE-KLRMISKEI---RQVVRMTSANMDP--

>Rapgef6_[Columba_livia]_449267215
STIEVATQLSMRDFELFRNIEPT----EYI-----DDLYK-L-DS----K-TGN-A----
-------------------HLKQFEDVINQETFWVAMEILAEPNQLKRMKIIKHFIKIA-
LHCRE-CKNFNSMFAVISGLNLAPVARLRGTWEKLPSKYEKHLRDLQDLFDPSRNMAKYR
NILSSQSMQP-------PIIPL-FPVVKKDITFL--------------------------
-----HEGND--SKV-------D---GLVNFE-KLRMIAKEI---RQVVRMTSANMDP--

>Rapgef6_[Condylura_cristata]_507959748
STIEVATQLSMRDFDLFRNIEPT----EYI-----DDLFK-L-DS----K-TGN-T----
-------------------HLKEFEDIVNQETFWVASEILTESNQLKRMKIIKHFIKIA-
LHCRE-CKNFNSMFAIISGLNLASVARLRGTWEKLPSKYEKHLQDLQDLFDPSRNMAKYR

NILSSQSMQP-------PIIPL-FPVVKKDMTFL-------------------------
-----HEGND--SKV-------D---GLVNFE-KLRMIAKEI---RQVVRMTSANMDP--

>Rapgef6_[Corvus_cornix_cornix]_726993269
STIEVATQLSMRDFELFRNIEPT----EYI-----DDLYK-L-DS----K-TGN-A----
-------------------HLKQFEDVINQETFWVAMEILAEPNQLKRMKIIKHFIKIA-
LHCRE-CKNFNSMFAVISGLNLAPVARLRGTWEKLPSKYEKHLRDLQDLFDPSRNMAKYR
NILSSQSMQP-------PIIPL-FPVVKKDITFL-------------------------
-----HEGND--SKV-------D---GLVNFE-KLRMIAKEI---RQVVRMTSANMDP--

>Rapgef6_[Crassostrea_gigas]_762094130
-TTEVASQLTLDDFKVFASIQPT----EYI-----DKLFN-I-KS----K-YGI-P----
-------------------NLEKFVELVNKEMYWVITEVCSEPNVVKRMRIIKQFIKIA-
KHCKD-CKNFNSMFSILSGLDKVYVSRLRNTWEKLSKKDLKTYDDLKELIDPSKNMSKYR
SYVGSEHVQP-------PMLPL-FPVAMKDLTFL-------------------------
-----KDGND--TKV-------D---GLINFE-KFRMIAKEI---R--------------

>Rapgef6_[Cynoglossus_semilaevis]_1035260107
STMEVAAQLSMRDFELFRNIEST----EYV-----DDLFK-L-DS----S-VGS---G--
-------------------NLKQFEEVINQETFWVATEILKEPNTLKRMKTIKHFIKIA-
LHCRE-CKNFNSMFAIISGLNLAPVARLRSSWEKLPSKYEKLFGDLQDVFDPSRNMAKYR
NILSSQSMQP-------PIIPL-FPVVKKDLTFL-------------------------
-----HEGND--SSV-------D---GLVNFE-KLRMIAKEI---RHVVRMTSANMDP--

>Rapgef6_[Daphnia_magna]_1022775171
---EVAVQLTLQDFAIFTQIEST----EYV-----DDLFQ-L-RS----K-YGT-PH---
--------------------LSQFSELVNREMFWVVTEICAEHNLMRRMRAIKQFIKVA-
RQCKE-CKNFNSMFNIISGLGHGAVSRLKQTWEKLPGKYQRLFRDMQDLMDPSRNMSKYR
NLINSEHVQP-------PMIPF-FPVVKKDLTFI-------------------------
-----HLGND--TRV-------D---GLINFE-KLRMIAKEV---RSLSHMCSAPYD---

>Rapgef6_[Diuraphis_noxia]_985398056
---EVAVQLTLQDFSIFRQIEAT----EYV-----DDLFQ-L-DS----R-YGT-PM---
--------------------LSKFAELVNTEMFWVVTEICSEHNLIRRSKMIKQFIKVA-
RQCKE-CKNFNSMFAIISGLGHGAVSRLRQSWEKLPTKYQRLFSDLQDLMDPSRNMSKYR
QLVSNEQSQP-------PIIPF-YPVVKKDLTFI-------------------------
-----HLGND--THV-------E---SLINFE-KLRMIAKEV---RNLTNMCSSPYD---

>Rapgef6_[Echinococcus_multilocularis]_674571093
SPEEVAIQLTLDDFSIFRSIEAA----EFV-----DKVFG-LTPP-TSSALDGC-SNSSN
NGNIDNDGGGGGGFATGCANLDAFADVVNKEAYWVPSELCAETSLPKRVDMLKRFIKIA-
KLCRD-LRNFNTMFCILVGLHMSPVERLRQTWERLPNKYVKMSRDLALVLDPSRNFAHYR
NLLTSPPQSPQTGAQCLPLPVPY-LPLVLKDLTFI-------------------------
-----HLGNP--SRSPATSASSSACPQLINFA-KLRMFAKEI---RSLR-----------

>Rapgef6_[Echinops_telfairi]_507622381
STIEVATQLSMRDFDLFRNIEPT----EYV-----DDLFK-L-DS----K-TGN-T----
-------------------HLKAFEDIVNQETFWVASEILTESNQLKRMKIIKHFIKIA-
LHCRE-CKNFNSMFAIISGLNLAPVARLRGTWEKLPNKYEKHLQDLQDIFDPSRNMAKYR
NILSSQSMQP-------PIIPL-FPVVKKDMTFL-------------------------
-----HEGND--SKV-------D---GLVNFE-KLRMIAKEI---RQVVRMTSANMDP--

>Rapgef6_[Elephantulus_edwardii]_585707699
STIELAAQLSMRDFDLFRNIEPT----EYI-----DDLFK-L-DS----K-TGN-T----

-------------------HLKEFEDIVNQETFWVASEILTEANQLKRMKIIKHFIKIA-
LHCRE-CKNFNSMFAIISGLNLASVARLRGTWEKLPNKYEKHLQDLQDLFDPSRNMAKYR
NILSSQSMQP-------PIIPL-FPVVKKDMTFL--------------------------
-----HEGND--SKV-------D---GLVNFE-KLRMIAKEI---RQVVRMTSANMDP--

>Rapgef6_[Equus_asinus]_958795657
STIEVATQLSMRDFDLFRNIEPT----EYI-----DDLFK-L-DS----K-TGN-T----
-------------------HLKEFEDIVNQETFWVATEILTESNQLKRMKLIKHFIKIA-
LHCRE-CKNFNSMFAIISGLNLSSVARLRGTWEKLPSKYEKHLQDLQDLFDPSRNMAKYR
NVLSSQSMQP-------PIIPL-YPVVKKDMTFL--------------------------
-----HEGND--SKV-------D---GLVNFE-KLRMIAREI---RQVVRMTSANMDP--

>Rapgef6_[Equus_caballus]_545185041
STIEVATQLSMRDFDLFRNIEPT----EYI-----DDLFK-L-DS----K-TGN-T----
-------------------HLKEFEDIVNQETFWVATEILTESNQLKRMKLIKHFIKIA-
LHCRE-CKNFNSMFAVISGLNLSSVARLRGTWEKLPSKYEKHLQDLQDLFDPSRNMAKYR
NVLSSQSMQP-------PIIPL-YPVVKKDMTFL--------------------------
-----HEGND--SKV-------D---GLVNFE-KLRMIAREI---RQVVRMTSANMDP--

>Rapgef6_[Erinaceus_europaeus]_617645429
STIEVATQLSMRDFDLFRNIEPT----EYI-----DDLFK-L-DS----K-TGN-T----
-------------------HLKEFEDIINQETFWVASEILSESNQLKRMKIIKHFIKIA-
LHCRE-CKNFNSMFAIISGLNLASVARLRGTWEKLPSKYEKHLQDLQDLFDPSRNMAKYR
NILSSQSMQP-------PIIPL-FPVVKKDMTFL--------------------------
-----HEGND--SKV-------D---GLVNFE-KLRMIAKEI---RQVIRMTSANMDP--

>Rapgef6_[Esox_lucius]_884954377
STVEVAAQLSMRDFELFRNIEST----EYV-----DDLFK-LLDS----S-GGA-QQT--
-------------------RLKQFEEVINQETFWVATEILREANAVKRMKTIKHFIKIA-
LHCRE-CKNFNSMFAIISGLNLAPVARLRSSWEKLPGKYDKLFRDLQDIFDPSRNMAKYR
NVLSSQSMQP-------PIIPL-FPVVKKDLTFL--------------------------
-----HEGND--SSV-------D---GLVNFE-KLRMIAKEI---RHVVRMTSANMDP--

>Rapgef6_[Falco_peregrinus]_909808310
STIEVATQLSMRDFELFRNIEPT----EYI-----DDLYK-L-DS----K-TGN-A----
-------------------HLKQFEDVINQETFWVAMEILAEPNQLKRMKIIKHFIKIA-
LHCRE-CKNFNSMFAVISGLNLAPVARLRGTWEKLPSKYEKHLRDLQDLFDPSRNMAKYR
NILSSQSMQP-------PIIPL-FPVVKKDITFL--------------------------
-----HEGND--SKV-------D---GLVNFE-KLRMIAKEI---RQVVRMTSANMDP--

>Rapgef6_[Felis_catus]_755689987
STIEVATQLSMRDFDLFRNIEPT----EYI-----DDLFK-L-DS----K-TGN-T----
-------------------HLKEFEDIVNQETFWVASEILTEPNQLKRMKIIKHFIKIA-
LHCRE-CKNFNSMFAIISGLNLASVARLRGTWEKLPSKYEKHLQDLQDLFDPSRNMAKYR
NILSSQSMQP-------PIIPL-FPVVKKDMTFL--------------------------
-----HEGND--SKV-------D---GLVNFE-KLRMIAKEI---RQVVRMTSANMDP--

>Rapgef6_[Ficedula_albicollis]_1020955556
STIEVATQLSMRDFELFRNIEPT----EYI-----DDLYK-L-DS----K-TGN-A----
-------------------HLKQFEDVINQETFWVAMEILAEPNQLKRMKIIKHFIKIA-
LHCRE-CKNFNSMFAVISGLNLAPVARLRGTWEKLPSKYEKHLRDLQDLFDPSRNMAKYR
NILSSQSMQP-------PIIPL-FPVVKKDITFL--------------------------
-----HEGND--SKV-------D---GLVNFE-KLRMIAKEI---RQVVRMTSANMDP--

>Rapgef6_[Fundulus_heteroclitus]_831570941
STMEVAAQLSMRDFELFRNIEST----EYV-----EDLFK-L-DS----S-AGC---T--
-------------------NLKQFEEVINQETFWVATEILKEPNTMKRMKTIKHFVKIA-
LHCRE-CKNFNSMFAIISGLNLAPVNRLRSSWEKLPSKYEKLFEDLQDVFDPSRNMAKYR
NVLSSQSMQP-------PIIPL-FPVIKKDLTFL--------------------------
-----HEGND--SSV-------D---GLVNFE-KLRMIAKEI---RHVVRMTSANMDP--

>Rapgef6_[Gallus_gallus]_971418567
STIEVATQLSMRDFELFRNIEPT----EYI-----DDLYK-L-DS----K-TGN-A----
-------------------HLKQFEDVINQETFWVAMEILAEPNQLKRMKIIKHFIKIA-
LHCRE-CKNFNSMFAVISGLNLAPVARLRGTWEKLPGKYEKHLRDLQDLFDPSRNMAKYR
NILSSQSMQP-------PIIPL-FPVVKKDITFL--------------------------
-----HEGND--SKV-------D---GLVNFE-KLRMIAKEI---RQVVRMTSANMDP--

>Rapgef6_[Gavia_stellata]_698442235
STIEVATQLSMRDFELFRNIEPT----EYI-----DDLYK-L-DS----K-TGN-A----
-------------------HLKQFEDVINQETFWVAMEILAEPNQLKRMKIIKHFIKIA-
LHCRE-CKNFNSMFAVISGLNLAPVARLRGTWEKLPSKYEKHLRDLQDLFDPSRNMAKYR
NILSSQSMQP-------PIIPL-FPVVKKDITFL--------------------------
-----HEGND--SKV-------D---GLVNFE-KLRMIAKEI---RQVVRMTSANMDP--

>Rapgef6_[Haliaeetus_leucocephalus]_729760461
STIEVATQLSMRDFELFRNIEPT----EYI-----DDLYK-L-DS----K-TGN-A----
-------------------HLKQFEDVINQETFWVAMEILAEPNQLKRMKIIKHFIKIA-
LHCRE-CKNFNSMFAVISGLNLAPVARLRGTWEKLPSKYEKHLRDLQDLFDPSRNMAKYR
NILSSQSMQP-------PIIPL-FPVVKKDITFL--------------------------
-----HEGND--SKV-------D---GLVNFE-KLRMIAKEI---RQVVRMTSANMDP--

>Rapgef6_[Halyomorpha_halys]_939647710
---EVAVQLTLQDFSIFRQIEST----EYV-----DDLFG-I-KS----R-YGT-PM---
--------------------LTQFAELVNREMFWVVTEVCSEHNLVRRSKIIKQFIKVA-
RQCKE-CKNFNSMFAIISGLGHGAVSRLRTTWDKLPSKYQRLFQDLQQIMDPSRNMSKYR
QLVNS--GTP-------PIIPF-YPVVKKDLTFI--------------------------
-----HLGND--TKV-------E---GLINFE-KLRMIAKEV---RTLTNMCSSPYD---

>Rapgef6_[Heterocephalus_glaber]_512948458
STIELATQLSMRDFDLFRNIEPT----EYI-----DDIFK-L-DS----K-TGN-T----
-------------------HLKEFEDIVNQETFWVASEILTESNPLKRMKVIKHFIKIA-
LHCRE-CKNFNSMFAIISGLNLAPVARLRGTWEKLPNKYEKHLQDLQDLFDPSRNMAKYR
NILSSQSMQP-------PIIPL-FPVVKKDMTFL--------------------------
-----HEGND--SKV-------D---GLVNFE-KLRMISKEI---RQVVRMTSANMDP--

>Rapgef6_[Hydra_vulgaris]_828198711
---DVAKELTLQDFDLFRNVDSR----EYI-----YNLFE-SNNE----R---K-SK---
-------------------NLRCVEQTTNTEMFWVISEICLESNIAKRVKLLKYYIKVA-
KYCKD-FKNYNSMYAVISGLANTAISRLKHTWEKLPQKHEDMFQDLLDLMDPSRNMSKYR
NMFTGEQCYP-------PIIPW-FPIVKKDMTFL--------------------------
-----HLGND--THV-------D---GLVNFE-KLRMIAREV---RRVCKFCAIGYDP--

>Rapgef6_[Ictalurus_punctatus]_1042302961
STLEVSAQLSMRDFSIFRNIEST----EYV-----DSLFK-L-NS----S-SG-------
-------------------HLKQFEDLINQETFWVATEILHEANALKRMKTIKHFIKIA-
LQCRE-CKNFNSMFAIISGLNLAPVARLRSTWEKLPSKYEKLFRDLQDIFDPSRNMAKYR
NILNSQSMQI-------PIIPL-FPVVKKDLTFL--------------------------

-----HEGND--TNV-------D---GLVNFE-KLRMIAKEV---R--------------

>Rapgef6_[Ictidomys_tridecemlineatus]_532100447
STIEMATQLSMRDFDLFRNIEPT----EYI-----DDLFK-L-DS----K-TGN-T----
-------------------HLKEFEDIVNQETFWVASEILTESNQLKRMKIIKHFIKIA-
LHCRE-CKNFNSMFAIISGLNLAPVARLRGTWEKLPSKYEKHLQDLQDLFDPSRNMAKYR
NILSSQSMQP-------PIIPL-FPVVKKDMTFL--------------------------
-----HEGND--SKV-------D---GLVNFE-KLRMIAKEI---RQVVRMTSANMDP--

>Rapgef6_[Larimichthys_crocea]_808885705
STMEVAAQLSMRDFELFRNIEST----EYV-----DDLFK-L-ES----S-RGS---G--
-------------------NLKQFEEVINQETFWVATEILKEPNALKRMKTIKHFIKIA-
LHCRE-CKNFNSMFAIISGLNLAPVARLRSSWEKLPSKYEKLFGDLQDVFDPSRNMAKYR
NILSSQSMQP-------PIIPL-FPVVKKDLTFL--------------------------
-----HEGND--SSV-------D---GLVNFE-KLRMIAKEI---RHVVRLTSANMDP--

>Rapgef6_[Lepisosteus_oculatus]_972976864
STVEVATQLSMRDFELFRNLEPT----EYI-----DDLFK-L-DS----A-ASN-T----
-------------------HLKQFEDVINQETFWVATEILKEPNHFKRMKTIKHFIKIS-
LHCRE-CKNFNSMFAIISGLNLAPVARLRGTWEKLPSKYEKLFRDLQDIFDPSRNMAKYR
NLLSSQSMQP-------PIIPL-FPVVKKDLTFL--------------------------
-----HEGND--SSV-------D---GLVNFE-KLRMVAKEI---RHVVRMTSANMDP--

>Rapgef6_[Leptonychotes_weddellii]_585151000
STIEVATQLSMRDFDLFRNIEPT----EYI-----DDLFK-L-DS----K-TGN-T----
-------------------HLKEFEDIVNQETFWVASEILTESNQLKRMKIIKHFIKIA-
LHCRE-CKNFNSMFAIISGLNLASVARLRGTWEKLPSKYEKHLQDLQDLFDPSRNMAKYR
NILSSQSMQP-------PIIPL-FPVVKKDMTFL--------------------------
-----HEGND--SKV-------D---GLVNFE-KLRMIAKEI---RQVVRMTSANMDP--

>Rapgef6_[Limulus_polyphemus]_926627213
---EIATQLTLEDFSIFCQIEPT----EYI-----DDLFD-I-KS----R-YGT-P----
-------------------KLSKFGELVNREMFWVVTEVCSEPNHLRRMKIIKQFIKVA-
RQCKE-CKNFNSMFAILSGLDHGAVSRLRSAWEKLPSKYSKMFKDLSDLMDPSRNMCKYR
TLISSEHTHP-------PMIPF-YPVVKKDLTFI--------------------------
-----HLGND--TKV-------E---GLVNFE-KLRMVAKEV---RQL------------

>Rapgef6_[Lingula_anatina]_919032373
-SIEIATQLTLEDFHIFANIEAT----EYI-----DDLFD-L-PS----K-YGT-P----
-------------------NILQFQELVNREMFWVTTEVVRETNIIKRMKIIKHFIKIA-
RHCKE-CKNFNSMFAILSGLGHGSVQRLRTTWDKLPNKYVKIYEDLQTIMDPSRNMAKYR
NLINSELVQP-------PLIPL-FPIVKKDLTFI--------------------------
-----HLGND--SKV-------D---GLVNFE-KLRMIAKEV---RHLCLMASAPYDPNA

>Rapgef6_[Macaca_mulatta]_109078488
STIEVATQLSMRDFDLFRNIEPT----EYI-----DDLFK-L-NS----K-AGN-T----
-------------------HLKRFEDIVNQETFWVASEILTEANQLKRMKIIKHFIKIA-
LHCRE-CKNFNSMFAIISGLNLASVARLRGTWEKLPSKYEKHLQDLQDIFDPSRNMAKYR
NILSSQSMQP-------PIIPL-FPVVKKDMTFL--------------------------
-----HEGND--SEV-------D---GLVNFE-KLRMISKEI---RQVVRMTSANMDP--

>Rapgef6_[Macaca_nemestrina]_795530958
STIEVATQLSMRDFDLFRNIEPT----EYI-----DDLFK-L-NS----K-AGN-T----
-------------------HLKRFEDIVNQETFWVASEILTEANQLKRMKIIKHFIKIA-

LHCRE-CKNFNSMFAIISGLNLASVARLRGTWEKLPSKYEKHLQDLQDIFDPSRNMAKYR
NILSSQSMQP-------PIIPL-FPVVKKDMTFL-------------------------
-----HEGND--SEV-------D---GLVNFE-KLRMISKEI---RQVVRMTSANMDP--

>Rapgef6_[Mandrillus_leucophaeus]_795275466
STIEVATQLSMRDFDLFRNIEPT----EYI-----DDLFK-L-NS----K-AGN-T----
-------------------HLKRFEDIVNQETFWVASEILTEANQLKRMKIIKHFIKIA-
LHCRE-CKNFNSMFAIISGLNLASVARLRGTWEKLPSKYEKHLQDLQDIFDPSRNMAKYR
NILSSQSMQP-------PIIPL-FPVVKKDMTFL-------------------------
-----HEGND--SKV-------D---GLVNFE-KLRMISKEI---RQVVRMTSANMDP--

>Rapgef6_[Marmota_marmota_marmota]_984113345
STIEMATQLSMRDFDLFRNIEPT----EYI-----DDLFK-L-DS----K-TGN-T----
-------------------HLKEFEDIVNQETFWVASEILTESNQVKRMKIIKHFIKIA-
LHCRE-CKNFNSMFAIISGLNLAPVARLRGTWEKLPSKYEKHLQDLQDLFDPSRNMAKYR
NILSSQSMQP-------PIIPL-FPVVKKDMTFL-------------------------
-----HEGND--SKV-------D---GLVNFE-KLRMIAKEI---RQVVRMTSANMDP--

>Rapgef6_[Melopsittacus_undulatus]_527258131
STIEVATQLSMRDFELFRNIEPT----EYI-----DDLYK-L-DS----K-TGN-A----
-------------------HLKQFEDVINQETFWVAMEILAEPNQLKRMKIIKHFIKIA-
LHCRE-CKNFNSMFAVISGLNLAPVARLRGTWEKLPSKYEKHLRDLQDLFDPSRNMAKYR
NILSSQSMQP-------PIIPL-FPVVKKDITFL-------------------------
-----HEGND--SKV-------D---GLVNFE-KLRMIAKEI---RQVVRMTSANMDP--

>Rapgef6_[Merops_nubicus]_675611298
STIEVATQLSMRDFELFRNIEPT----EYI-----DDLYK-L-DS----K-TGN-A----
-------------------HLKQFEDVINQETFWVAMEILAEPNQLKRMKIIKHFIKIA-
LHCRE-CKNFNSMFAVISGLNLAPVARLRGTWEKLPSKYEKHLRDLQDLFDPSRNMAKYR
NILSSQSMQP-------PIIPL-FPVVKKDITFL-------------------------
-----HEGND--SKV-------D---GLVNFE-KLRMIAKEI---RQVVRMTSANMDP--

>Rapgef6_[Microcebus_murinus]_829753489
STIELATQLSMRDFDLFRNIEPT----EYI-----DDLFK-L-DC----Q-TGN-T----
-------------------HLKEFEDIVNQETFWVASEILTESNQLKRMKIIKHFIKIA-
LHCRE-CKNFNSMFAIISGLNLAPVARLRGTWEKLPSKYEKHLQDLQDLFDPSRNMAKYR
NILSSQSMQP-------PIIPL-FPVVKKDMTFL-------------------------
-----HEGND--SKV-------D---GLVNFE-KLRMIAKEI---RQVVRMTSANMDP--

>Rapgef6_[Mus_musculus]_568972368
STIEVATQLSMRDFDLFRNIEPT----EYI-----DDLFK-L-DS----K-TGN-T----
-------------------HLKQFEDIVNQETFWVASEILSESNQLKRMKIIKHFIKIA-
LHCRE-CKNFNSMFAIISGLNLAPVARLRGTWEKLPSKYEKHLQDLQDLFDPSRNMAKYR
NILSSQSMQP-------PIIPL-FPVVKKDMTFL-------------------------
-----HEGND--SKV-------D---GLVNFE-KLRMIAKEI---RHIIRMTSANMDP--

>Rapgef6_[Myotis_brandtii]_521025561
STVELATQLSMRDFDLFRNIEPT----EYI-----DDLFK-L-DS----K-TGN-T----
-------------------HLKEFEDIVNQETFWVASEVLTESNQLKRMKIIKHFIKIA-
LHCRE-CKNFNSMFAIISGLNLASVARLKGTWEKLPSKYEKHLQDLQDLFDPSRNMAKYR
NILSSQSMQP-------PIIPL-FPVVKKDMTFL-------------------------
-----HEGND--SKV-------D---GLVNFE-KLRMIAKEI---RQVVRMTSANMDP--

>Rapgef6_[Myotis_davidii]_432109543

STVELATQLSMRDFDLFRNIEPT----EYI-----DDLFK-L-NS----K-TGN-T----
-------------------HLKEFEDIVNQETFWVASEVLTESNQLKRMKIIKHFIKIA-
LHCRE-CKNFNSMFAIISGLNLASVARLKGTWEKLPSKYEKHLQDLQDLFDPSRNMAKYR
NILSSQSMQP-------PIIPL-FPVVKKDMTFL--------------------------
-----HEG--

>Rapgef6_[Myotis_lucifugus]_558176198
STVELATQLSMRDFDLFRNIEPT----EYI-----DDLFK-L-DS----K-TGN-T----
-------------------HLKEFEDIVNQETFWVASEVLTESNQLKRMKIIKHFIKIA-
LHCRE-CKNFNSMFAIISGLNLASVARLKGTWEKLPSKYEKHLQDLQDLFDPSRNMAKYR
NILSSQSMQP-------PIIPL-FPVVKKDMTFL--------------------------
-----HEGND--SKV-------D---GLVNFE-KLRMIAKEI---RQVVRMTSANMDP--

>Rapgef6_[Nomascus_leucogenys]_332221642
STIEVATQLSMRDFDLFRNIEPT----EYI-----DDLFK-L-NS----K-TGN-T----
-------------------HLKRFEDIVNQETFWVASEILTEANQLKRMKIIKHFIKIA-
LHCRE-CKNFNSMFAIISGLNLASVARLRGTWEKLPSKYEKHLQDLQDIFDPSRNMAKYR
NILSSQSMQP-------PIIPL-FPVVKKDMTFL--------------------------
-----HEGND--SKV-------D---GLVNFE-KLRMISKEI---RQVVRMTSANMDP--

>Rapgef6_[Odobenus_rosmarus_divergens]_472377738
STIEVATQLSMRDFDLFRNIEPT----EYI-----DDLFK-L-DS----K-TGN-T----
-------------------HLKEFEDIVNQETFWVASEILTESNQLKRMKIIKHFIKIA-
LHCRE-CKNFNSMFAIISGLNLASVARLRGTWEKLPSKYEKHLQDLQDLFDPSRNMAKYR
NILSSQSMQP-------PIIPL-FPVVKKDMTFL--------------------------
-----HEGND--SKV-------D---GLVNFE-KLRMIAKEI---RQVVRMTSANMDP--

>Rapgef6_[Orcinus_orca]_466058795
STIEVATQLSMRDFDLFRNIEPT----EYI-----DDLFK-L-DS----K-TGN-I----
-------------------HLKEFEDIVNQETFWVASEILTESNQLKRMKIIKHFIKIA-
LHCRE-CKNFNSMFAIISGLNLASVARLRGTWEKLPSKYEKHLQDLQDLFDPSRNMAKYR
NILSSQSMQP-------PIIPL-FPVVKKDMTFL--------------------------
-----HEGND--SKV-------D---GLVNFE-KLRMIAKEI---RQVVRMTSANMDP--

>Rapgef6_[Ornithorhynchus_anatinus]_620947523
STIEVATQLSMRDFDLFRNIEPT----EYI-----DDLYK-L-DS----K-TGN-A----
-------------------HLKEFEDIINQETFWVATEILAEPNQLKRMKIVKHFIKIA-
LHCRE-CKNFNSMFAVISGLNLAPVARLRGTWEKLPSKYEKHFRDLQDLFDPSRNMAKYR
NILSSQSMQP-------PIIPL-FPVVKKDITFL--------------------------
-----HEGND--SKV-------D---GLVNFE-KLRMIAKEI---RQVIRMTSANMDP--

>Rapgef6_[Orussus_abietinus]_817220783
---EVAIQLTLQDFSIFRQIEST----EYV-----DDLFE-L-KS----R-YGV-PM---
--------------------LSQFAELVNREMFWVVTEVCSEHNLVRRSKIIKQFIKIA-
RQCKE-CKNFNSMFAIVSGLGHGAVSRLRASWEKLPSKYQRLFSDLQELMDPSRNMSKYR
QLVASEQTQP-------PIIPF-YPVVKKDLTFI--------------------------
-----HLGND--SRV-------E---NLVNFE-KLRMIAKEI---RTL------------

>Rapgef6_[Orycteropus_afer_afer]_634856153
STIEVATQLSMRDFDLFRNIEPT----EYI-----DDLFK-L-DS----K-TGN-T----
-------------------HLKEFEDIVNQETFWVASEILTESNQLKRMKIIKHFIKIA-
LHCRE-CKNFNSMFAIISGLNLAPVARLRGTWEKLPNKYEKHLQDLQDLFDPSRNMAKYR
NILSSQSMQP-------PIIPL-FPLVKKDMTFL--------------------------
-----HEGND--SKV-------D---GLVNFE-KLRMIAKEI---RQVIRMTSANMDP--

```
---------------------------------------------------------
>Rapgef6_[Oryzias_latipes]_765140209
STMEVAAQLSMRDFELFRNIEPT----EYV-----DDLFK-L-DS----P-LSR-G----
-------------------NLKRFEEVMNQETFWVATEILREPNSVKRMRTIKHFIKIA-
LHCKE-CKNFNSMFAIISGLDLPPVARLRSSWEKLPSKYAKLFGDLQDVFDPSRNMAKYR
NMLSSQSMQP-------PIIPL-FPVIKKDLTFF--------------------------
-----HEGND--SSV-------D---GLVNFE-KLRMIAKEI---RHVVRMTSANMDP--
---------------------------------------------------------
>Rapgef6_[Ovis_aries_musimon]_803320919
STIEVATQLSMRDFDLFRNIEPT----EYI-----DDLFK-L-ES----K-TGN-T----
-------------------HLKEFEDIINQETFWVASEILTESNQLKRMKIIKHFIKIA-
LHCRE-CKNFNSMFAIISGLNLASVARLRGTWEKLPSKYEKHFQDLQDLFDPSRNMAKYR
NILSSQSMQP-------PIIPL-FPVVKKDMTFL--------------------------
-----HEGND--SKV-------D---GLVNFE-KLRMIAKEI---RQVVRMTSANMDP--
---------------------------------------------------------
>Rapgef6_[Pan_troglodytes]_114601518
STIEVATQLSMRDFDLFRNIEPT----EYI-----DDLFK-L-NS----K-TGN-T----
-------------------HLKRFEDIVNQETFWVASEILTEANQLKRMKIIKHFIKIA-
LHCRE-CKNFNSMFAIISGLNLASVARLRGTWEKLPSKYEKHLQDLQDIFDPSRNMAKYR
NILSSQSMQP-------PIIPL-FPVVKKDMTFL--------------------------
-----HEGND--SKV-------D---GLVNFE-KLRMISKEI---RQVVRMTSANMDP--
---------------------------------------------------------
>Rapgef6_[Panthera_tigris_altaica]_591323552
STIEVATQLSMRDFDLFRNIEPT----EYI-----DDLFK-L-DS----K-TGN-T----
-------------------HLKEFEDIVNQETFWVASEILTEPNQLKRMKIIKHFIKIA-
LHCRE-CKNFNSMFAIISGLNLASVARLRGTWEKLPSKYEKHLQDLQDLFDPSRNMAKYR
NILSSQSMQP-------PIIPL-FPVVKKDMTFL--------------------------
-----HEGND--SKV-------D---GLVNFE-KLRMIAKEI---RQVVRMTSANMDP--
---------------------------------------------------------
>Rapgef6_[Pantholops_hodgsonii]_556768283
STIEVATQLSMRDFDLFRNIEPT----EYI-----DDLFK-L-ES----K-TGN-T----
-------------------HLKEFEDIVNQETFWVASEILTESNQLKRMKIIKHFIKIA-
LHCRE-CKNFNSMFAIISGLNLASVARLRGTWEKLPSKYEKHFQDLQDLFDPSRNMAKYR
NILSSQSMQP-------PIIPL-FPVVKKDMTFL--------------------------
-----HEGND--SKV-------D---GLVNFE-KLRMIAKEI---RQVVRMTSANMDP--
---------------------------------------------------------
>Rapgef6_[Parasteatoda_tepidariorum]_1009601738
-SVEVATQLTLEDFSTFRQIEPT----EYI-----DDLFE-V-TS----K-YGI-P----
-------------------QLSKFAELVNHEMFWVVTEVCAEHQLIRRMRIVKQFIKVA-
RQCKN-CKNFNSMFAILSGLGHGAVSRLRTTWEKLPTKYQKMFSDLQDLMDPSRNMCKYR
SLLNSEHTQP-------PIIPF-YPVVKKDLTFI--------------------------
-----HLGND--TFV-------D---NLVSIK-K--------------------------
---------------------------------------------------------
>Rapgef6_[Peromyscus_maniculatus_bairdii]_1008772024
STIEVATQLSMRDFDLFRNIEPT----EYI-----DDLFK-L-DS----K-TGN-T----
-------------------HLKQFEDIVNQETFWVASEILSESNQLKRMKIIKHFIKIA-
LHCRE-CKNFNSMFAIISGLNLAPVARLRGTWEKLPSKYEKHLQDLQDLFDPSRNMAKYR
NILSSQSMQP-------PIIPL-FPVVKKDMTFL--------------------------
-----HEGND--SKV-------D---GLVNFE-KLRMIAKEI---RHIVRMTSANMDP--
---------------------------------------------------------
>Rapgef6_[Physeter_catodon]_593723311
STIEVATQLSMRDFDLFRNIEPT----EYI-----DDLFK-L-DS----K-TGN-T----
-------------------HLKEFEDIVNQETFWVASEILTESNQLKRMKIIKHFIKIA-
LHCRE-CKNFNSMFAIISGLNLASVARLRGTWEKLPSKYEKHLQDLQDLFDPSRNMAKYR
```

NILSSQSMQP-------PIIPL-FPVVKKDMTFL-------------------------
-----HEGND--SKV-------D---GLVNFE-KLRMIAKEI---RQVVRMTSANMDP--

>Rapgef6_[Picoides_pubescens]_699645489
STIEVATQLSMRDFELFRNIEPT----EYI-----DDLYK-L-DS----K-TGN-A----
-------------------HLKQFEDVINQETFWVAMEILAEPNQLKRMKIIKHFIKIA-
LHCRE-CKNFNSMFAVISGLNLAPVARLRGTWEKLPSKYEKHLRDLQDLFDPSRNMAKYR
NILSSQSMQP-------PIIPL-FPVVKKDITFL-------------------------
-----HEGND--SKV-------D---GLVNFE-KLRMIAKEI---RQVVRMTSANMDP--

>Rapgef6_[Poecilia_formosa]_1025473374
STMEVAAQLSMRDFELFRNIEST----EYV-----EDLFK-L-DS----S-AGC---T--
-------------------NLKQFEEVINQETFWVATEILKEPNTLKRMKTIKHFIKIA-
LHCRE-CKNFNSMFAIISGLNLAPVARLRSSWEKLPSKYEKLFEDLQDVFDPSRNMAKYR
NVLSSQSMQP-------PIIPL-FPVVKKDLTFL-------------------------
-----HEGND--SNV-------D---GLVNFE-KLRMIAKEI---RHVVRMTSANMDP--

>Rapgef6_[Poecilia_reticulata]_658884510
STMEVAAQLSMRDFELFRNIEST----EYV-----EDLFK-L-DL----S-AGC---T--
-------------------NLKQFEEVINQETFWVATEILKEPNTLKRMKTIKHFIKIA-
LHCRE-CKNFNSMFAIISGLNLAPVARLRSSWEKLPSKYEKLFEDLQDVFDPSRNMAKYR
NVLSSQSMQP-------PIIPL-FPVVKKDLTFL-------------------------
-----HEGND--SNV-------D---GLVNFE-KLRMIAKEI---RHVVRMTSANMDP--

>Rapgef6_[Polistes_dominula]_972179004
---EVAIQLTLQDFSIFRQIEST----EYV-----DDLFE-L-KS----R-YGI-PM---
--------------------LSQFAELVNREMFWVVTEVCSEHNLVRRSKIIKQFIKIA-
RQCKE-CKNFNSMFAIVSGLGHGAVSRLRASWEKLPSKYQRLFSDLQELMDPSRNMSKYR
QLVASEQTQP-------PIIPF-YPVVKKDLTFI-------------------------
-----HLGND--SRV-------E---SLVNFE-KLRMIAKEV---RTLTNMCSSPYD---

>Rapgef6_[Pongo_abelii]_686719316
STIEVATQLSMRDFDLFRNIEPT----EYI-----DDLFK-L-NS----K-TGN-T----
-------------------HLKRFEDIVNQETFWVASEILTEANQLKRMKIIKHFIKIA-
LHCRE-CKNFNSMFAIISGLNLASVARLRGTWEKLPSKYEKHLQDLQDIFDPSRNMAKYR
NILSSQSMQP-------PIIPL-FPVVKKDMTFL-------------------------
-----HEGND--SKV-------D---GLVNFE-KLRMISKEI---RQVVRMTSANMDP--

>Rapgef6_[Protobothrops_mucrosquamatus]_1002581315
STIEVATQLSMRDFELFRNIEPT----EYI-----DELYK-L-DS----K-TGN-T----
-------------------NLKQFEDVINQETFWVATEILSETNQLKRMKIVKHFIKIA-
LHCRE-CKNFNSMFAVISGLNLAPVARLRSTWEKLPSKYEKHLRDLQDLFDPSRNMAKYR
NILSSQSMQP-------PIIPL-FPVVKKDITFL-------------------------
-----HEGND--SKV-------D---GLVNFE-KLRMIAKEI---RQVVRMTSANMDP--

>Rapgef6_[Pseudopodoces_humilis]_929446448
STIEVATQLSMRDFELFRNIEPT----EYI-----DDLYK-L-DS----K-TGN-A----
-------------------HLKQFEDVINQETFWVAMEILAEPNQLKRMKIIKHFIKIA-
LHCRE-CKNFNSMFAVISGLNLAPVARLRGTWEKLPSKYEKHLRDLQDLFDPSRNMAKYR
NILSSQSMQP-------PIIPL-FPVVKKDITFL-------------------------
-----HEGND--SKV-------D---GLVNFE-KLRMIAKEI---RQVVRMTSANMDP--

>Rapgef6_[Pygoscelis_adeliae]_690439934
STIEVATQLSMRDFELFRNIEPT----EYI-----DDLYK-L-DS----K-TGN-A----

-------------------HLKQFEDVINQETFWVAMEILAEPNQLKRMKIIKHFIKIA-
LHCRE-CKNFNSMFAVISGLNLAPVARLRGTWEKLPSKYEKHLRDLQDLFDPSRNMAKYR
NILSSQSMQP-------PIIPL-FPVVKKDITFL--------------------------
-----HEGND--SKV-------D---GLVNFE-KLRMIAKEI---RQVVRMTSANMDP--

>Rapgef6_[Python_bivittatus]_602628149
STIEVATQLSMRDFELFRNIEPT----EYI-----DDLYK-L-ES----K-TGN-T----
-------------------NLKQFEDVINQETFWVATEILSEANQLKRMKIVKHFIKIA-
LHCRE-CKNFNSMFAVISGLNLAPVARLRGTWEKLPSKYEKHLRDLQDLFDPSRNMAKYR
NILSSQSMQP-------PIIPL-FPVVKKDITFL--------------------------
-----HEGND--SKV-------D---GLVNFE-KLRMIAKEI---RQVVRMTSANMDP--

>Rapgef6_[Rhinopithecus_roxellana]_724913891
STIEVATQLSMRDFDLFRNIEPT----EYI-----DDLFK-L-NS----K-TGN-T----
-------------------HLKRFEDIVNQETFWVASEILTEANQLKRMKIIKHFIKIA-
LHCRE-CKNFNSMFAIISGLNLASVARLRGTWEKLPSKYEKHLQDLQDIFDPSRNMAKYR
NILSSQSMQP-------PIIPL-FPVVKKDMTFL--------------------------
-----HEGND--SKV-------D---GLVNFE-KLRMISKEI---RQVVRMTSANMDP--

>Rapgef6_[Rousettus_aegyptiacus]_1012239520
STIELATQLSMRDFDLFRNIEPT----EYI-----GDLFK-L-ES----K-TGN-T----
-------------------HLKEFENIVNQETFWVASEILTESNQLKRMKIIKHFIKIA-
LHCRE-CKNFNSMFAIISGLNLASVARLRGTWEKLPSKYEKHLQDLQDLFDPSRNMAKYR
NILSSQSMQP-------PIIPL-FPVVKKDMTFL--------------------------
-----HEGND--SKV-------D---GLVNFE-KLRMIAKEI---RQVVRMTSANMDP--

>Rapgef6_[Saimiri_boliviensis_boliviensis]_725550658
STIEVATQLSMRDFDLFRNIEPT----EYI-----DDLFK-L-NS----K-TGN-S----
-------------------HLKRFEDIVNQETFWVASEILTEANQLKRMKIIKHFIKIA-
LHCRE-CKNFNSMFAIISGLNLASVARLRGTWEKLPSKYEKHLQDLQDIFDPSRNMAKYR
NILSSQSMQP-------PIIPL-FPVVKKDMTFL--------------------------
-----HEGND--SKV-------D---GLVNFE-KLRMISKEI---RQVVRMTSANMDP--

>Rapgef6_[Sarcophilus_harrisii]_821455239
STIEVATQLSMRDFELFRNIEPT----EYI-----DDLFK-L-NS----K-TGN-T----
-------------------HLKEFEAIINQETFWVATEILTESNQLKRMKMIKHFIKIA-
LHCRE-CKNFNSMFAVISGLNLAPVARLRGTWEKLPSKYEKHFQDLQDLFDPSRNMAKYR
NILSNQSMQP-------PIIPL-FPIVKKDITFL--------------------------
-----HEGND--SKV-------D---GLVNFE-KLRMIAKEI---RQVVRMTSANMDP--

>Rapgef6_[Sinocyclocheilus_anshuiensis]_1024970981
STVEVAAQLSMRDFGLFRNIEST----EYV-----DDLFK-R-DP----G-GGG-S-S--
-------------------HLKQFEEVINQETFWVATEILREPNALKRMKTIKHFIKIA-
LHCRE-CKNFNSMFAIISGLNLAPVARLRSTWEKLPSKYEKLFRDLQDIFDPSRNMAKYR
NILSSQSVQP-------PIIPL-FPVVKKDLTFL--------------------------
-----HEGND--SSV-------D---GLVNFE-KLRMIAKEI---RNVVHMTSANMDP--

>Rapgef6_[Sinocyclocheilus_grahami]_1020491327
STVEVAAQLSMRDFDLFRNIEST----EYV-----DDLFK-R-DP----G-GGG-N-S--
-------------------HLKQFEEVINQETFWVATEILREPNALKRMKTIKHFIKIA-
LHCRE-CKNFNSMFAIISGLNLAPVARLRSTWEKLPSKYEKLFRDLQDIFDPSRNMAKYR
NILSSQSVQP-------PIIPL-FPVVKKDLTFL--------------------------
-----HEGND--SSV-------D---GLVNFE-KLRMIAKEI---RNVVRMTSANMDP--

```
>Rapgef6_[Sinocyclocheilus_rhinocerous]_1025396494
STVEVAAQLSMRDFGLFRNIEST----EYV-----DDLFK-L-DP----G-GGG-S-S--
-------------------HLKQFEEVINQETFWVATEILREPNALKRMKNIKHFIKIA-
LHCRE-CKNFNSMFAIISGLNLAPVARLRSTWEKLPSKYEKLFRDLQDIFDPSRNMAKYR
NILSSQSVQP-------PIIPL-FPVVKKDLTFL--------------------------
-----HEGND--SSV-------D---GLVNFE-KLRMIAKEI---RHVVRMTSANMDP--
---------------------------------------------------------
>Rapgef6_[Sorex_araneus]_505829185
STIEVATQLSMRDFDLFRNIEPT----EYI-----DDLFK-L-DS----K-TGN-T----
-------------------HLKEFEDIVNQETFWVASEVLTESNQLKRMKIIKHFIKIA-
LHCRE-CKNFNSMFAIISGLNLACVARLRGTWEKLPSKYEKHLQDLQDLFDPSRNMAKYR
NILSSQSMQP-------PIIPL-FPVVKKDMTFL--------------------------
-----HEGND--SKV-------D---GLVNFE-KLRMIAKEI---RQVVRMTSANMDP--
---------------------------------------------------------
>Rapgef6_[Struthio_camelus_australis]_697433805
STIEVATQLSMRDFELFRNIEPT----EYI-----DDLYK-L-DS----K-TGN-A----
-------------------RLKQFEDVINQETFWVAMEILAEPNQLKRMKIIKHFIKIA-
LHCRE-CKNFNSMFAVISGLNLAPVARLRGTWEKLPSKYEKHLRDLQDLFDPSRNMAKYR
NILSSQSX------------PL-FPVVKKDITFL--------------------------
-----HEGND--SKV-------D---GLVNFE-KLRMIAKEI---RQVVRMTSANMDP--
---------------------------------------------------------
>Rapgef6_[Sturnus_vulgaris]_959086183
STIEVATQLSMRDFELFRNIEPT----EYI-----DDLYK-L-DS----K-TGN-A----
-------------------HLKQFEDVINQETFWVAMEILAEPNQLKRMKIIKHFIKIA-
LHCRE-CKNFNSMFAVISGLNLAPVARLRGTWEKLPSKYEKHLRDLQDLFDPSRNMAKYR
NILSSQSMQP-------PIIPL-FPVVKKDITFL--------------------------
-----HEGND--SKV-------D---GLVNFE-KLRMIAKEI---RQVIRMTSANMDP--
---------------------------------------------------------
>Rapgef6_[Sus_scrofa]_545812182
STIEVATQLSMRDFDLFRNIEPT----EYI-----DDLFK-L-DS----K-TGN-T----
-------------------HLKEFEDIVNQETFWVASEILTESNQLKRMKIIKHFIKIA-
LHCRE-CKNFNSMFAIISGLNLASVARLRGTWEKLPSKYEKHLQDLQDLFDPSRNMAKYR
NILSSQSMQP-------PIIPL-FPVVKKDMTFL--------------------------
-----HEGND--SKV-------D---GLVNFE-KLRMIAKEI---RQVVRMTSANMDP--
---------------------------------------------------------
>Rapgef6_[Trichechus_manatus_latirostris]_471406089
STIEMATQLSMRDFDLFRNIEPT----EYI-----DDLFK-L-DS----K-TGN-T----
-------------------HLKEFEDIVNQETFWVASEILTESNQLKRMKIIKLFIKIA-
LHCRE-CKNFNSMFAIISGLNLAPVARLRGTWEKLPNKYEKHLQDLQDLFDPSRNMAKYR
NILSSQSMQP-------PIIPL-FPVVKKDMTFL--------------------------
-----HEGND--SKV-------D---GLVNFE-KLRMIAKEI---RQVVRMTSANMDP--
---------------------------------------------------------
>Rapgef6_[Tyto_alba]_701405792
STIEVATQLSMRDFELFRNIEPT----EYI-----DDLYK-L-DS----K-TGN-A----
-------------------HLKQFEDVINQETFWVAMEILAEANQLKRMKIIKHFIKIA-
LHCRE-CKNFNSMFAVISGLNLAPVARLRGTWEKLPSKYEKHLRDLQDLFDPSRNMAKYR
NILSSQSMQP-------PIIPL-FPVVKKDITFL--------------------------
-----HEGND--SKV-------D---GLVNFE-KLRMIAKEI---RQVVRMTSANMDP--
---------------------------------------------------------
>Rapgef6_[Ursus_maritimus]_671012702
STIEVATQLSMRDFDLFRNIEPT----EYI-----DDLFK-L-DS----K-TGN-T----
-------------------HLKEFEDIVNQETFWVASEILTESNQLKRMKIIKHFIKIA-
LHCRE-CKNFNSMFAIISGLNLASVARLRGTWEKLPSKYEKHLQDLQDLFDPSRNMAKYR
NILSSQSMQP-------PIIPL-FPVVKKDMTFL--------------------------
```

-----HEGND--SKV-------D---GLVNFE-KLRMIAKEI---RQVVRMTSANMDP--

>Rapgef6_[Vicugna_pacos]_970734891
STIEVATQLSMRDFDLFRNIEPT----EYI-----DDLFK-L-DS----K-TGN-T----
-------------------HLKEFEDIVNQETFWVASEILTESNQLKRMKIIKHFIKIA-
LHCRE-CKNFNSMFAIISGLNLASVARLRGTWEKLPSKYEKHLHDLQDLFDPSRNMAKYR
NILSSQSMQP-------PIIPL-FPVVKKDMTFL--------------------------
-----HEGND--SKV-------D---GLVNFE-KLRMIAKEI---RQVVRMTSANMDP--

>Rapgef6_[Xiphophorus_maculatus]_941798194
STMEVAAQLSMRDFELFRNIEST----EYV-----EDLFK-L-DS----S-SGC---T--
-------------------NLKQFEEVINQETFWVATEILKEPNTLKRMKTIKHFIKIA-
LHCRE-CKNFNSMFAIISGLNLAPVARLRSSWEKLPSKYEKLFEDLQDVFDPSRNMAKYR
NVLSSQSMQP-------PIIPL-FPVVKKDLTFL--------------------------
-----HEGND--SNV-------D---GLVNFE-KLRMIAKEI---RHVVRMTSANMDP--
